# Supplementary material for: Acceptability of Digital Adherence Technologies to support people with drug-susceptible TB in South Africa
Source: PLoS One. 2025 Sep 24;20(9):e0332103. doi: 10.1371/journal.pone.0332103 (PMC12459780; doi:10.1371/journal.pone.0332103)
Supplement: S1 File — (DOCX) [file pone.0332103.s001.docx]

**Annexure 1 Interview Guide for PWTB**

QUESTIONS

1. **Experience, attitude, feeling and level of satisfaction of the differentiated model of care and use of the <insert DAT> (motivators and barriers)**

- *Could you describe your experience of being part of the differentiated model of care?*
- *Who explained to you on how to use the <insert DAT> and how did you feel about this? Is there anything that you would change about how you were informed about using the <insert DAT>?*
- *How easy was it for you to use the <insert DAT>?*
- *Please describe the difficulties you experienced when using the <insert DAT>.*
- *Please describe the reasons for accidentally opening the medication device [for pill box users only].*
- *What were your concerns about using or having the <insert DAT>?*
- *How did you feel about telling people about the <insert DAT>? Who did you tell and why?*
- *What was helpful about using the <insert DAT> for TB treatment?*
- *What was difficult about using the <insert DAT> for TB treatment?*
- *Please describe any cultural or traditional barriers to the differentiated model of care and use of the <insert DAT>.*
- *Please describe your level of satisfaction with use of the <insert DAT>.*
- *What would make it easier for you to use the <insert DAT>?*

1. **Perceptions, experiences and feelings toward to the retention activities [only ask if relevant]**

- *How did you feel about receiving reminder SMSs? What did you like or dislike about this?*
- *How did you feel about staff visiting your home? What did you like or dislike about this?*
- *Please describe your experience of the counselling received. What did you like or dislike about this?*
- *Of all the activities that you were part of during this study, which was most influential in helping you take your TB treatment and, which, did you find was not useful? Please elaborate.*
- *Could you describe any gaps which exist in the way the intervention was delivered currently?*

**ANY OTHER COMMENTS**

- *Are there any final thoughts you have about the differentiated model of care and use of the <insert DAT>?*

End of session

Now we have come to the end of our discussion. Thank you for your participation. If you have any questions about your study participation, please contact us. Thank you.
