# Supplementary material for: Acceptability of Digital Adherence Technologies to support people with drug-susceptible TB in South Africa
Source: PLoS One. 2025 Sep 24;20(9):e0332103. doi: 10.1371/journal.pone.0332103 (PMC12459780; doi:10.1371/journal.pone.0332103)
Supplement: S2 File — (DOCX) [file pone.0332103.s002.docx]

**Annexure 2 Interview Guide for HCWs and stakeholders**

Questions

1. **General**

- *What is the title of your current position?*
- *How long have you held this position?*
- *How are TB services delivered at your level with regard to the intervention? (i.e. District, Provincial, National).*
- *Please describe your role with the differentiated model of care intervention.*

1. **Feasibility of implementing the differentiated model of care (motivators and barriers)**

- *Please describe the cadre of staff that were involved with delivering the differentiated model of care?*
- *Could you describe your experience of delivering the differentiated model of care?*
- *Could you describe the training and resources that staff received prior to or during delivery of the differentiated model of care? What was your opinion of the training and resources received?*
- *Could you describe the benefits of the differentiated model of care and use of the medication device technology?*
- *How could the differentiated model of care be sustained?*
- *Could you describe challenges of the differentiated model of care and use of the medication device technology?*
- *How could TB treatment be improved using this differentiated model of care and the medication device technology?*

1. **System level challenges of delivering the intervention.**

- *Please elaborate on the positive changes of the differentiated model of care and use of the medication device technology. How do you think these positive changes could be sustained?*
- *Please elaborate on the negative changes of the differentiated model of care and use of the medication device technology. How do you think the negative changes could be addressed?*
- *Could you describe to us what systems are in place that could monitor the differentiated model of care and use of the medication device technology?*
- *Please describe to us what system level structures need to be improved in order to integrate the differentiated model of care and medication device technology into the existing TB programme system.*

1. **Factors that influence the sustainability of the intervention**

- *Please describe to us the expectations that you may have had about the differentiated model of care and use of the medication device technology.*
- *Please describe your positive and negative experience of delivering or implementing the differentiated model of care.*
- *How different is the current intervention model compared to your initial expectations and how do you think this model could be more effective?*
- *Could you describe any gaps which, exist in the way the intervention is being delivered currently?*

**ANY OTHER COMMENTS**

Are there any final thoughts you have about the differentiated model of care and use of the medication device technology?
