# Supplementary material for: Acceptability of Digital Adherence Technologies to support people with drug-susceptible TB in South Africa
Source: PLoS One. 2025 Sep 24;20(9):e0332103. doi: 10.1371/journal.pone.0332103 (PMC12459780; doi:10.1371/journal.pone.0332103)
Supplement: S4 File — (ZIP) [file pone.0332103.s004.zip › S4 Transcripts/HCWs and Stakeholders/IDI 10_ HCW.docx]

**TRANSCRIPTION NOTATIONS**

| **Label Key** | **Meaning** |
| --- | --- |
| **I** | Start of each new utterance by the Interviewer |
| **P** | Start of each new utterance by the Participant |
| **N** | Note taker |
| **{ }** | Indicates that details were changed or pseudonyms were used to anonymise data |
| **( )** | Indicates the description provided to anonymise data |
| **XXX** | Words were omitted to anonymise data |
| **-** | Breaking into a sentence by the next speaker |
| **…** | Pause or drawn out words |
| **[ ]** | Indicates noise made, e.g. [laugh], [sigh], [pause] |
| ? | Beginning of utterance by unidentified speaker or questionable text |
| **[inaudible segment]** | Unclear section of the recording |

I: Do we have a permission to audio record you?

P:Yes, you have a permission

I: Okay thank you, uhh date of ID is xxxxxx (interview date) ,location xxx [clinic name], language used English, PID of participant is xxxx .The time the session start is 12:34pm, okay, so what is the title of your current position?

P: My title of my position is an internship.

I: Okay, what is it that you do under this position?

P: I am a data capture and I’m helping people to use the smart pill box

I:Okay and how long have you been in this position?

P:It’s 4months now.

I: Four months? Okay when it comes to patient care and counselling *neh* (right), patient care and counselling, what are your role and responsibilities? What role do you play when it comes to counselling patient, you know?

P:Yes, okay the role that I play when it comes to counselling patient is to make a patient feel comfortable and feel safe to talk, so I can counsel him or her and then I always have to use a private space where there’s no disturbance.

I:Okay, uh have you counselled any patient so far since you said you have four months since you started working, have you done any counselling so far?

P:Yes, I’ve done 1,only 1 patient .

I: How was the experience?

P: Mmm, the experience was good [laughing], although it was tough because it was first the time, but it was good.

I: Okay, did you realise or did you see any change or improvements after the counselling session because I assumed that before or the reason to counsel this individual or a patient it because he or she was struggling somehow and so now I wish to know if the was an improvement after the counselling session. Did you see any improvement or positive change following the counselling session?

P: Yes, I saw the positive change because the patient was refusing to take his medication because he found himself struggling to accept that he has TB, so like I gave him a counselling. After the counselling session and everything was well, and she was ready to take her medication.

I: Okay, do you have any clue why she was refusing treatment?

P:Uhh, I don’t have any clue like maybe she was scared, I think, maybe she thought that it will kill her or maybe like she thought she will have to drink the medication for the rest of her life. No, after counselling and had made things clear that, TB, you drink medication for just a period of time and you are good.

I: Well, I would like to know what you know about ASCENT right, if you were to explain to someone who knows nothing about ASCENT, what would you tell the person?

P: ASCENT? If a person asked me like what is ASCENT okay, I would simply tell him that ASCENT it where we help people to make their life easier. Okay, for example, here we are carrying a smart pill box that will remind a patient when to drink the medication and what time should drink medication. Okay and then I would also tell them if they ask about ASCENT that through ASCENT, you have no doubt but for sure like I can say 99% sure that you would be cured, when drinking TB medication.

I: What type of DAT (Digital Adherence Technology) are you using in this facility?

P: It is the box.

I: Okay uh and are you familiar with xxxx ( adherence platform)?

P: Yes, I’m familiar.

I: Please tell me little bit about xxxx (adherence platform), yes, how does it work.

P: Alright xxxx (adherence platform) , I think the person who made xxxx (adherence platform) App is a genius it makes your life easier. We merge the pill box on xxxx (adherence platform) App to see or to know whether a patient is drinking his medication on time or if a patient is defaulting his medication and then if the patient is not drinking the medication, I will easily call to remind them that you are not drinking your medication and you are supposed to call a patient after two defaulter doses, yes, that’s the xxxx (adherence platform). It helps a lot, yes.

I: Okay since you are mentioning the follow up phone calls with patient who seem to have missed their doses uh, what has been the success in terms of calling the patients who have missed their dose. Did you see any changes after you phoned them or talk to them?

P:Yes, I’ve saw a lot of change because like when I’m calling my patients maybe after missing two doses the patient says she will make sure that she drinks the medication every day and on time. Some forget to open, some maybe put the medication aside ,they take it out, I remind them with a call that you didn’t drink the medication then they drink them, so my success is that I can see my patients who are drinking the medication.

I: After the phone call?

P:After the phone call, doing everything.

I: Okay are you familiar with task list uh, I know that there is something called task list on (adherence platform).

P: *Yah* (yes), I’m familiar.

I: Are you using it?

P:[laughing] no, I don’t want to lie, I’m not using it.

I: Okay are you the one who is doing the follow up phone calls, are you the one who personally phones patients?

P:Yes.

I: Did you have challenges maybe uh in trying to call some of the patients?

P: *Yah* (yes) I have a challenge . Some of their numbers go on voicemails when I try to call them several times I have found a solution. So, if I try to call maybe four days without any success, I’m sending the WBOT (ward-based primary health care team) and then after I’ve sent the WBOT and they tell the patients to come to the facility. When they came, I asked what’s the problem with his phone not working, then he simply told me that okay “my phone was stolen” or he gave me wrong a number. Then, I write it down and every time when I call, everything is working.

I: So, do the WBOT usually find these patients when they looking for them?

P:Yes,yes, they find them.

I:They never had issues of wrong address?

P:No, they never had issues of wrong address or maybe the patient has moved away. *Yah* (yes) that’s where I find the problem.

I: Okay have you had any case of a patient who relocated?

P: Who relocated?

I: *Yah* (yes) and changed the address?

P: No, no ,no.

I: Not so far?

P: Not so far.

I: Okay and then you said you have conducted counselling session?

P:Yes.

I: With one patient and the experience was great?

P:Yes, experience was great

I: Okay uh, who else does the counselling?

P: Who does the counselling uh, Sister that I’m working with in the TB room.

I: [background noise] okay, you have already told me that you phone patients and then you also communicate with the WOBOT (ward-based primary community health team)?

P:Yes

I: Uhh, and [ background noise] you also mentioned that you are involved in counselling session sometimes?

P:Yes, sometimes I’m involved

I:Alright, so now I wish to know what is it that you do frequently between the follow up phone calls and the home visit?

P:Yes.

I: Which one do you do frequently?

P: Frequently?

I:*Yah* (yes) between the two, in calling patients and sending the WOBOT to go and look for patients?

P:Uhh, come again

I: What is it that you do frequently or more between the two, phoning patients or sending WOBOT?

P: Okay, frequently uh, I’m calling patients and then when the calls are unsuccessful, that’s when I am sending the WOBOT, but frequently I’m using the phone call.

I: Okay and then uh, you said there’s been a success, you said the WBOT are successful in most of the cases when they are searching for patients?

P:Yes, they are successful.

I:The are no any other issues or challenges that they encounter?

P:No, up to so far, the are no challenges.

I: And how are the responsibilities and the duties shared between you and other health care workers in the facility or in the TB room, yes. How are the duties and responsibilities shared amongst you?

P: Okay in the TB room I’m working with Sister, just the two of us. So, like the things that I do, I collect the sputum and then write lab form sometimes.

I: Okay, alright, uh so when you first heard about the DAT or the box uh, what were your expectations before you came in this facility and started implementing it. What was your ,or what were your expectations?

P: My- sorry, can you please come again?

I: Like how do you think it was going to be before you started implementing or heard that there is this DAT uh, which helps patients in this way, what came to your mind or your expectation. What did expect from it?

P: From it?

I: Yes.

P: Okay I expected the DAT to make people’s lives easy because when I heard about it and looked at it I saw maybe this will work, maybe this will make people not to default. It will reduce the defaulting of people because some patients when they drink medication and feel better, they just stop the medication. So, when I saw that the pill box will always be there, and it reminds you that you must drink your medication for that period of time. So, that’s where I saw that DAT will work for patients .

I: Okay then, did your opinion change after implementing. You said you expected this box to help patient and reduce the defaulters and so on.

P:Yes.

I: Now, uh do you think those expectations that you had uh, were met by this DAT?

P: Yah(yes) most of them were met, I saw the defaulters going down a lot and then since the DAT many people are finishing their treatment well.

I:Did you have a training before you started implementing, did you receive the training?

P:Yes, we had a training

I:Do you still remember what you were being trained on, what was done, what were activities that were done on that training. What were you trained on to be specific?

P: Okay we were trained on how to implement, how to implement the smart pill boxes, yes. Uh at the training, they told us that like when you give a patient a smart pill box, the patient must sign a consent form and then on xxx (adherence platform) when you register a patient you register with the serial number and then before giving patient a smart pill box. You must just give a small counselling and then explain to him or her how it works and how those three light work; the green light reminds you to take your medication okay and the yellow light reminds you oh, no, sorry the green light it will tell that you have you take your medication okay and then the yellow light will remind you when to come and collect your medication. Then the red light will explain to a patient that the battery is low and the battery needs to be recharged which means the patient must come and I’m going charge the smart pill box for him or her.

I:Besides the light that you mention, the one’s that reminds the patient, How else does this box reminds patients that now it’s time to take the medication?

P: Okay uh, it has an alarm, let’s say the patients drink the medication at 8:00 and then it its 08:00 the box will ring the alarm then remind the patient that no, now it’s time to take your medication.

I:Okay and where did you attend this training, were you trained in the facility or somewhere else?

P:No, we were trained at xxx (area), yes.

I: Who trained you? Do you still remember?

P:[laughing]Yes, I was trained by xxx [ ASCENT staff member’s name] and xxx [ASCENT staff member’s name].

I: And what was your first impression after the training, what did you think about the training?

P: Uh about the training, I was so excited about the training, okay, now here’s the solution to make like people drink the medication. Yes and then *yah* (yes) I can say that.

I: Huh, did you find the training helpful? Did you find the information at the training useful to you guys?

P:Yes, I have found the training useful, yes, I found the training very useful because if they just said okay do this without the training I would be clueless with the patient. I wouldn't know how to register a patient so I found the training more useful.

I: Okay has your opinion changed after the training, after you were being trained, your opinion with the DAT. Did it change following the training session?

P: Following the training session? No, my opinion didn’t change.

I: Okay, do you have any suggestion on how the training can be improved in future.

P: In future? Uh at the moment, I think the training is- because like- what can I say, they make you understand a lot better how to do about this smart pill box ,yes.

I:Okay and then how long do you think the training should take uh, in terms of the duration, should it be one day or a week?

P:No, I think because somethings you won’t get it in one day you know, I think it should take a week and then *yah*(yes) after a week I’m sure you will be ready to give a patient to start using the smart pill box in xxx (adherence platform)

I: And how long did the training you attended last?

P:The training lasted three days.

I: And did you find those three days enough, were you able to comprehend and understand everything?

P:Yah (yes) on my side I found that the training was enough because like they explained everything and it more important when they training to write down in order to remember when you get home or when you have a time. You will rehearse for sure those things that they told you, it will be useful to you and so.

I: Who should attend these trainings, who do you think should attend these trainings?

P: These trainings? Us interns should attend these trainings.

I: In the facilities, who should attend the training?

P: Okay in the facility, I think the Sister (TB Nurse) that we work with should attend this training and make her understand what is being done by us, yes.

I: Okay, for now, do you think she understands?

P:For now, yes, I think she understands after I explained everything to her. I think she understands how it works because she asked and I sat down with her and told her what to do.

I:From your perspective *neh (*right*).*

P:Yes.

I: Can you describe the benefit of the DAT to patients?

P: To patients?

I: How does it benefit patients?

P: It benefits a lot because patients will know what time to drink his or her medication. One thing I love about a smart pill box is that you take it anywhere with you.

I: Okay and how does it benefit you as the research staff together with other health care workers who are working in this facility?

P: Okay, it benefits me because I can always like look together with the Sister (TB Nurse) and see and whether a particular patient is really drinking his or her medication because if she doesn’t drink her medication and skip her doses it tells us that this person is not drinking her medication. If they miss medication, we call them to find out the reason why she is not drinking medication. If she says she is drinking but it does not reflect we kindly ask her to bring the box back.

I: Okay can you describe the challenge of the differentiated model of care uh, the follow up calls, the home visit. Are there any challenges so far?

P: So, far our patients didn’t give us any trouble, so like up to so far everything is going well.

I:You don’t have cases of patients who don’t like being called if they missed their dose and say, oh maybe why are you people calling me.

P:Every patient when we call , is friendly, she will just do what is being told.

I: Okay and no patient who have complained about home visit?

P:No, there’s no patient.

I: Okay and then any challenges with the box, do you have patients perhaps who refuse the box?

P:Yes, we have patients who refuse the box especially the older people, yes. Some patients refuse to take the box.

I: Why do you think they refuse to take the box?

P: I once experienced that one lady, she told me that she’s not taking TB medication only. she is taking diabetes medication, she said 'no, I don’t think that it will be useful to me because I’m taking my diabetes medication so I don’t see it useful since its for TB only

I: Uhh, any network issues with the platform, adherence platform and the box?

P: And the box? No, I don’t have any network issues.

I:No patient has reported the issues with network?

P:No, patient has reported the issue.

I: Okay uh, stigma. Do you have patients maybe who experienced stigma because of DAT or the box?

P: No, I don’t have any patients.

I: Okay, do you have homeless people who are using the box?

P:Yes, I have homeless people who are using the box.

I: Okay and then did some of the homeless people maybe lost their box before?

P: Who have lost their box? No, I don’t have people who have lost their box, I don’t have that case.

I: And then with homeless people uh, are you able to reach them when you do home visit?

P: Home visit? Yes, I reached them.

I: Do they have cell phones?

P:Yes, they have cell phone. Those homeless people I’m talking about like they have a home just that they are always on street, but like the good thing they fetched their medication. They drink their medication.

I: From your perspective, do you think TB treatment can be improved using the DAT intervention?

P: My perspective yes, I think it can be improved.

I: How so?

P: Uhh, using the DAT makes life easier so I think when you are using the DAT everything is good. It will go well, and a patient will be able to drink his medication and he will know he does not have a chance to skip his medication because if he skips his medication the DAT will automatically tell the TB nurses or the TB practice, so I think like it works.

I: Do you think DAT (Digital Adherence Technology) impact on patient’s adherence?

P: Sorry

I: Do you think the DAT impact on patient adherence?

P:Yes, I think so.

I: How, have you seen any changes?

P: Yes, we saw less defaulters since the DAT was started. There are less defaulters, people are drinking their medication almost every day.

I: Okay and then do you think the DAT improves the relationship between patients and their health care workers?

P:Yes, it’s improving the relationship because the patient will call when they are seeing problems with the smart pill box. So, maybe when a box has a network problem as I mentioned. Maybe when you open a smart pill box, and it doesn’t clock (alerts) to you that the patient opens this box.

I: Okay do you think the box, or the DAT has helped reducing the workload in the TB room?

P: Yes because patients will not just come to the facility, the patient will come to the facility when the smart pill box is lighting a yellow light to remind them that today your medication collection day.

I: Okay, so in the absence of the ASCENT staff, you guys you are implementing the DAT uh, when now ASCENT is handing over the DAT or the project to the department of health to take over now. What do you think or what structures do you think should to be put in place to sustain the intervention of the DAT? What is needed for the intervention to continue working the way it works?

P:The way it works? I think with the absence of the DAT (Digital Adherence Technology) like they will be starting to face challenges you know so and challenges that will be facing the patients will start again. Defaulting, they will be more defaulters sometimes, so like that will make the facility workers to have more home visits, but in the presence of the DAT, there are less like, everything is going well. I think the government will find it difficult in the absence of the DAT because like DAT has more impact on the TB patients.

I: Okay uh, when now the ASCENT staff who are helping with the box -when you are no longer in the facilities helping, but the box is still in the facilities. They are still giving the box to patients, but you guys are no longer here. So I’m asking what structures do you think the department of health needs to put in place in order to ensure the DAT or implementation of the DAT continues without any disruption or any challenges?

P: Mmm

I: What are the resources that are needed?

P: I think like the government needs to make an App okay, where any patient that is defaulting will be automatically called to remind them and say if you have missed your doses please drink your medication, yes. I think they will need that because in our absence, they have to be more technological advanced.

I: Okay, please elaborate on the negative changes of different model of care and the use of the medication device technology uh, do you have any negative changes that you have saw when it comes to the different model of care like the follow up phone calls uh, the home visit, the SMS’s. Is there a case of patients who don’t like the SMS’s or patient who are not answering their phone’s when you trying to reach them to communicate upon their missed doses or patients who don’t like home visit, yes?

P: Uhh, up to so far, i don’t see any negativity.

I: Did you have patients who opened their box without taking their medication?

P:Uh, no.

I: Okay and then uh can you please describe the structures that need to be improved in order to interreact the different model of care and medication device in the TB programme system like who should prepare the boxes and who should help in case they are any technical issues with the platform. Who should help when it comes to those issues solving them and who should prepare the boxes and what are resources needed?

P: Needed?

I: Yes, for the intervention to be successful, to be a success?

P: Uhh, resources that are needed up to so far well while I’m giving patients box and explained to them, I think I don’t see any resources that are needed because like I think as ASCENT interns like to and like make people see how this smart pill box are so useful to them

I: Who should prepare the box?

P: Who should prepare the box? Yes, uh like who to prepare it for them?

I: When you guys are no longer here, yes, as the ASCENT staff when the Department of Health has taken over the project, who should help now in preparation of the boxes helping with the technical issues should there be any issues with the App, the xxxx (adherence platform) App or with the boxes?

P: With the boxes? I think uh, the Sisters who are working in the TB room will prepare them. If they go to the training and be trained about it I think the Sisters will prepare for the patients.

I: And then when it comes to solving now the technical issues, who should help with that maybe there is a problem with the App and it is not working.

P:Yes,okay yah (yes) when the App is not working or as I mentioned they have to be more technological advanced because in order for them to not to have those challenges, they have to make sure that the App is never going to be a problem for them, yes. I think they have to be more technological advanced to make sure that the App is running well, while there’s a network or no network.

I: Uhh, so do you capture the challenges or the success uh or the problems that you encounter with the DAT. Is there way you capture-let me say if as you spoke about patients who were not taking medication, then you phoned and you saw that they started taking their medication after what, counselling session as you said that you’ve done one and then you saw that they were positive changes. Is there a way you capture that?

P:Yes, I capture them on my care logs.

I: Okay.

P:Yes, they give care logs where we capture all where we record all our progresses, yes. If for example, every time I call a patient maybe a patient missed his dose. There’s somewhere where I write on differentiated logs, I write it down and then I write when I called the patient; the date, the patient call was successful or not, I write it down.

I: Okay, okay we are almost done uh, do you see any gaps uh, which exist in a way the intervention is currently delivered- do you see anything that needs to be improved with the way intervention is being delivered currently?

P:No, I don’t see any gaps, I think everything is smooth because like patients are doing things. So, I don’t see any gaps.

I:So, everything is fine with the App, the box uh, you don’t see anything that needs to be improved. So, you think everything is fine the way it is?

P: Yah (yes) everything is fine the way it is, everything is smooth.

I: Okay uh, do you have any other comments or anything you want to comment on maybe something that we did not touch on.

P: No.

I: About DAT (Digital Adherence Technology)?

P:No, I don’t have any comments, everything is going well.

I: Okay now we have come to an end of our discussion *neh* (right), so thank you for your time, thank you for meeting with us uh, the session ends is 1:06pm uh.
