# Supplementary material for: Acceptability of Digital Adherence Technologies to support people with drug-susceptible TB in South Africa
Source: PLoS One. 2025 Sep 24;20(9):e0332103. doi: 10.1371/journal.pone.0332103 (PMC12459780; doi:10.1371/journal.pone.0332103)
Supplement: S4 File — (ZIP) [file pone.0332103.s004.zip › S4 Transcripts/HCWs and Stakeholders/IDI 11-HCW.docx]

**TRANSCRIPTION NOTATIONS**

| **Label Key** | **Meaning** |
| --- | --- |
| **I** | Start of each new utterance by the Interviewer |
| **P** | Start of each new utterance by the Participant |
| **N** | Note taker |
| **{ }** | Indicates that details were changed or pseudonyms were used to anonymise data |
| **( )** | Indicates the description provided to anonymise data |
| **XXX** | Words were omitted to anonymise data |
| **-** | Breaking into a sentence by the next speaker |
| **…** | Pause or drawn out words |
| **[ ]** | Indicates noise made, e.g. [laugh], [sigh], [pause] |
| ? | Beginning of utterance by unidentified speaker or questionable text |
| **[inaudible segment]** | Unclear section of the recording |

[Car hooting]

I: [Inaudible segment] So do we have permission to record you?

P: Yes

I: Thank you. Date of the interview xxxxx (interview date). Clinic location it’s xxx (clinic name). language used for the interview it’s English. The PID of the participant it’s xxx. And the interview is starting at hhhhh 12: 55. (……) So, can you please tell me what’s the tittle of your current position?

P: Professional nurse.

I: Okay. How long have you been in this position?

P: Hhhhh xxxx (number of years).

I: xxxx years. So, what are your roles and responsibilities in this position currently?

P: I’m working in a TB room hhhhh screening and investigation and management of TB clients.

I: Okay. So, what are your other roles?

P: I’m also a data champion and the HTS group champion.

I: Okay. What do you mean by HHTS group champion?

P: The HIV testing and hhhhh counselling and testing and treatment.

I: Okay. Alright. So, in your everyday life hhhhh what do you call digital adherence technology, what do you understand by digital adherence technology?

P: The box one? Okay hhhhh I don’t know much about the registration but I know about the box that hhhhh the client is registered on the system

I: Hhhhh

P: They select a specific time when they going to take their treatment and then it has an alarm, it set on the system which by at that time that they selected the alarm is gonna go off then it’s gonna be quite the moment they open and take treatment from the box.

I: Okay. So, what do you call that platform where you register the patient?

P: On the system?

I: Yes

P: I’m not sure, really [laugh]

I: Okay. Alright. So, how does it work, the platform and, and the box?

P: As in?

I: Hhhhh the relationship between the two.

P: Okay hhhhh there is good communication, I can say that because if, it indicates on the system how the client is taking their treatment hhhhh if they are taking, I saw the green light and if they are not taking, the red light will be up on the system, it shows.

I: Okay. So hhhhh what do you understand by differentiated model of care? Hhhhh

P: Differentiated model of care?

I: Yes. How you support the patients that are on the box?

P: Oh, okay hhhhh if we seeing the red colours, we normally call them and try to find out what the problem is. Because now the system is saying you are not taking your treatment.

I: Hhhhh

P: And then try to intervene that one, is to how we can go back to the green light.

I: Okay

P: Hhhhh

I: You mention-

P: We-

I: Yes

P: We recall the clients to the clinic hhhhh some they say they did take or the box will still alarm even if they did take treatment.

I: Hhhhh

P: So, they come back with the box, we see if the box is, there’s something wrong with or if the client is missing the doses.

I: Hhhhh

P: Then we take it from there.

I: Okay. So, you mentioned phone calls as differentiated care, what else do you do?

P: Hhhhh I think it’s mainly the phone calls and when they come to the clinic, we see as to what we do to back-

I: And-

P: To go back on track.

I: With the patients who don’t have phones, what do you do if you see red?

P: Sho. Only the tracing [laugh] sisters or the Wbots can help us there, when we give them the addresses. *Ya* [yes].

I: How do they help?

P: They go to the households. *Ya* [yes] cause hhhhh we usually have Wbot teams in Asande areas. So, we give to a certain team if we have a client in that area, we give them the addresses, they go and recall hhhhh recall a client to the clinic.

I: Okay

P: Hhhhh

I: So, once the, the client comes to the clinic, what do you say to the patient, what do you do with the patient?

P: We ask if they’ve been adhering to the treatment, and see if they having any problems that will make them be diffic-, that will make a difficult for them to take their treatment in time.

I: And if there’s problems, what happens?

P: If it needs social worker then we refer accordingly to the need, yes.

I: (……) So, if you were to explain what the digital adherence technology intervention is to another health care worker who doesn’t know anything about this hhhhh what would you tell them?

P: Okay, I’ll tell them that hhhhh the moment we start you on TB treatment, we give you a box where you going to be taking your treatment from, and this box will be set to a time you hhhhh the client chose and then that it will alarm at that specific time and then also it being monitored on the system, it will show if the client is taking their treatment or not.

I: (……) So, please tell me your role with the differentiated model of care we spoken about earlier, what your role in that process?

P: [Laugh] okay, my main one is the health education about the box.

I: Okay.

P: *Ya* [yes] I educate them that okay hhhhh when we give you this med- treatment we gonna put your medication in the box hhhhh you going to give us a time that you gonna take your treatment on. We will set that time on the system and the, it will alarm at that certain time that you chose

I: Hhhhh

P: And then you’ll open it and take your medication and that it will be quite only when you open and take the medication.

I: Okay. So, the differentiated model of care is those hhhhh follow up we talked about earlier, right?

P: Hhhhh

I: So, what’s your role in that? You mentioned phone calls, you mentioned hhhhh home visits. So, what’s your role? In following up with the patients and how do you identify patients who need to be followed up, starting from there?

P: Well, obviously, they’ll be identified on system or their files, it will say okay this patient has not been to the clinic and obviously because I give treatment that I count at the return date this patient will be taking maybe their last tablets then they need to come and take more. Then if they don’t show up, we also get hhhhh lost to follow up list from the data team hhhhh and the tracing Sisters from TDH when they come we give them that list with the address and the phone numbers for those who have phone numbers and they recall the client and they give us feedback when the clients come we get back to new track on adherence and compliance.

I: Okay. You mentioned the system, that you check on the system. Can you tell me more about checking non adherence patients on the system?

P: Well, I don’t know much about the logging in but I was shown that if it’s this green light it means the person is compliant, if it’s this red light it means that the, the patient is not taking the treatment.

I: Hhhhh

P: Yes.

I: Is there a way you know which can highlight to you the patients easily on the system?

P: Hhhhh I think the only those red lights, those are the ones I know of.

I: Okay.

P: *Ya* [yes]

I: And then with the phone calls and home visits and counselling, what, what’s your role in those three?

P: I don’t do the house visits hhhhh I can only make a phone call.

I: Okay.

P: Yes. And need to make the client aware that we need to see them at the clinic.

I: Okay.

P: *Ya* [yes]

I: So, which method follow up method do you do all the time frequently?

P: Most frequently is the phone call.

I: Hhhhh

P: Yes

I: And which one do you rarely do?

P: Hhhhh I think they are all used the same [phone rings] because if you were hhhhh if hhhhh for those who have phone calls, we call but sometimes you find that the phone number is not working, you try the next of kin then you cannot find then you left with one option with is, which is tracing. The, the, the TB sisters from xxxx (district name) or the Wbot team that they work together. Because at some areas, tho- there are people who don’t want other people going in there, they can only go there with the Wbot team.

I: Okay.

P: *Ya* [yes]

I: So hhhhh can you, you’ve already started this conversation, can you tell me the type of staff that are involved in this differentiated care, who exactly is making the phone calls, who exactly is doing the home visits?

P: Okay. For the phone call hhhhh [***] and I but mostly she’s the one who’s hhhhh showing us that, oh this person has missed treatment for sometime now, and then she makes the call and then some, I do make the calls and then we also have hhhhh the Wbot team which has a professional nurse and the community health care workers, and then we have the TB tracers from xxxx (district name) that’s two sisters that come here for the list of the lost to follow ups.

I: Okay

P: Or the defaulters.

I: So, the professional nurse you’ve mentioned and community health care workers, what exactly do they do?

P: It a household visit

I: Okay

P: They go there, they do the household registrations and also follow up on the ones they did maybe they had a referral

I: Hhhhh

P: To see if the client really did go to the clinic and was seen. *Ya* [yes].

I: Hhhhh

P: And also tracing.

I: Okay. So, when you were first told about this technology, what were your expectations?

P: Hhhhh I was expecting to see the, like the monitoring basically, if the client is complying or not.

I: So, did you have any expectation about how it would assist you in, in your work as a TB nurse? What did you, how did you think it was going to assist you?

P: In monitoring, *ya* [yes] because that’s the most important one because the clients come and take treatment, we are not there with them, so, we wouldn’t know if the client is on treatment or not. So, it’s helping a lot in that, in monitoring.

I: Okay hhhhh so, did your opinion change after the, the intervention, after you saw it being implemented, what your initial thoughts about this, did they change?

P: Hhhhh no.

I: (……) So, can you tell me the training and resources that you received before you started using the box with the patients and also the differentiated care, like the phone calls and the, the home visits?

P: I haven’t received any.

I: So, how do you know about it?

P: xxx (Intern name) showed me out [laugh] cause she’s the one who’s like okay I’m forever helping you out with your things, so, you should also tell them about the box, what does it do, setting it up. *Ya* [yes] so, I haven’t had any formal training.

I: Okay. So hhhhh the intern trained you on site?

P: Yes.

I: Okay. So, what was your opinion on the training you received?

P: Hhhhh it was fine, just that I didn’t get training on the system, on how to enrol a client, setting up the alarm, that I didn’t get.

I: So, what exactly hhhhh were you trained on, what-

P: On how the box works and show me how to see if the client is complying or not on the colour codes on system, *ya* [yes].

I: Okay. So, do you think it was hhhhh useful or sufficient?

P: The training that I got from her?

I: Yes.

P: Useful, yes hhhhh sufficient, no since I didn’t get to know cause if it was sufficient I will, I’ll be able to know also how to enrol a client on the system.

I: Hhhhh

P: Yes.

I: Okay. So, do you have any suggestions on how to improve the training?

P: Maybe if I can get a formal training.

I: Okay

P: *Ya* [yes]

I: So, what would be your suggestion at that formal training?

P: Hhhhh training-

I: How long do you think it should be hhhhh who should attend?

P: I think people working at a, in a TB room should attend.

I: Okay

P: *Ya* [yes] be it hhhhh professional nurse or enrolled nurse or assistant as long as they are placed in there. They need to know because we there to treat monitor, yes.

I: Okay. And how long do you think the training should be?

P: Hhhhh maybe a day, that’s 8 hours.

I: 8 hours?

P: Yes.

I: Okay. So, before we started implementing this xxx (name of city) , we trained TB nurses.

P: Isn’t?

I: Yes. So, when did you start hhhhh working a xxxxx clinic?

P: Hhhhh 2013 but I was away for 2 years, 2016,17, came back 18. *Ya* [yes].

I: Okay. In the-

P: So 2018

I: TB room?

P: No. In the TB room sometime last year I think October.

I: October?

P: *Ya* [yes] late last year.

I: So, the training was conducted at the beginning of, of the year.

P: But no one went from us.

I: In February?

P: I don’t remember.

I: Okay. Alright. So hhhhh we understand, so if you were not in the TB room it means someone else was working there-

P: Yes.

I: And-

P: Cause we always a pair.

I: Might have attended the training. And how do you suggest hhhhh on site trainings can be done? Because we understand, my point is that, a lot of staff rotation that goes on.

P: Hhhhh

I: Right? So, how do you suggest hhhhh we deal with staff rotation in terms of training because the trained nurse might not always be in that room, in the TB room?

P: I think if we all attend, like the professional nurses because we rotate. Like if we all get training then all of us will be knowledgeable. If I’m not there, someone goes in and then it’s easy to work. *Ya* [yes].

I: All professional nurses? Whether they are in TB room or not?

P: Yes. Cause somewhere somehow, they going to relieve in a TB room.

I: Okay. Thanks for the suggestion. So hhhhh in your opinion as a, as a health care worker, can you describe the benefits of hhhhh the differentiated model of care those follow ups and also hhhhh the use of the med- medication device technology, what are the benefits?

P: Okay. The benefits are for the clients, you get to take your treatment on time, you get to comply because there’s always a reminder beside you

I: Hhhhh

P: And also, for us the health care workers it helps with the monitoring, we get to, to do the follow ups based on the information we get from the system. Yes.

I: Okay. So, in terms monitoring, how exactly does it help you, how different is this monitoring from the way you’ve been doing things?

P: Hhhhh it’s easy with the system because if we didn’t have that we wouldn’t have hhhhh something that is saying this client is not taking the da- doses then we, with the system I think *ya* [yes] it, it’s effective.

I: Hhhhh

I: *Ya* [yes] because otherwise we would only know when we do hhhhh follow up smear that will give us a positive and we think okay maybe a client missed some doses, it will be hhhhh assumptions.

I: Hhhhh

P: *Ya* [yes] or when you see a client that, clinically they not improving

I: Hhhhh

P: Then you think maybe something is wrong, maybe some doses were missed.

I: Hhhhh

P: *Ya* [yes]

I: Okay. And hhhhh your relationship with the patients, you can think of a patient you’ve been supporting using the box, the technology hhhhh how has that impacted your relationship with the clients?

P: Well, the clients that are using boxes hhhhh the relationship is okay, some do complain that it makes a lot of noise we laugh about it and then it ends there but they still continue using the box.

I: Okay.

P: *Ya* [yes]

I: What about the issue of stigma?

P: *Ya* [yes] they also have a problem with that some hhhhh because some are working then if it’s time that is between their working hours then it’s going to be a problem because then a box will be alarming and there will be working there with colleagues but hhhhh we always suggest that they take, they use a time where they not gonna take the box to people.

I: Hhhhh

P: *Ya* [yes].

I: Okay. So, can you let me know hhhhh the challenges of the differentiated model of care and the use of medication?

P: The challenges would be now the boxes alarming even after it was opened, when I do not know what the problem is hhhhh but I think hhhhh the intern did something on the system to fix that, I don’t know what but some of the boxes were fixed that way and as for treatment the challenge can be now the tricky one, maybe a client just wants the box to be quite you just open and then close it again without actually taking the meds.

I: Hhhhh

P: Hhhhh

I: What other challenges?

P: With the box?

I: Yes. With hhhhh the differentiated care, the phone calls, the home visits and the box.

P: Okay hhhhh with the calls can be that the number is no longer in use, and the other ones is when the client gives us the wrong address cause we do get those. You find the tracers they go there and the person is nowhere to be found. Yes, those are the challenges.

I: Hhhhh do you have a group of people who hhhhh you finds difficult support with the digital adherence technology? The certain group of people.

P: Hhhhh not quite. Just that I think we once had a client staying at hhhhh at this Mahala flat, we call it mahala flat because a lot of people who are staying there are or drug users and it happened that he was putting his staff in a waste hhhhh container, this huge one, the bin then everything was there in the box so they stole that thing with everything of his so *ya* [yes] that about it.

I: Okay.

P: But otherwise, they are taking care of those boxes and when they- their treatment are- they reach the treatment completion period, they do bring us the box back.

I: Okay.

P: Hhhhh

I: So, do you have hhhhh a lot of patients in that situation, drug users, homeless?

P: Yo [laugh] a lot. It like TB is just roaming in that building.

I: So-

P: We try doing hhhhh outreach there because the- we, our doctor has a clinic there every Tuesday they go there with the Ward Bases Outreach (WBOT) team. I also went there for two days for an outreach, believe you me, those people will never come to attend.

I: Hhhhh

P: So, it’s just difficult and TB is all over that place. We also go with the health promoter to the back because it’s a flat then there’s squatters at the back, we also go there, listen guys we have such services at the tent please come through, they will never come through. Only a few.

I: Hhhhh

P: And they are like drinking, smoking, you drink you pass, staff like that.

I: So hhhhh have you put any of those on the digital adherence technology?

P: Yes we have

I: What has been your experience with those using the DAT whilst smoking and drinking?

P: Hhhhh some will do miss a few doses but they do come to the clinic hhhhh those who don’t have phones, the Wbot team go there for tracing and then they tell them you are needed at the clinic, they do come.

I: Okay.

P: *Ya* [yes]

I: So hhhhh according to the differentiated hhhhh model of care we supposed to do a home visit after four days, you know, of seeing red, how feasible is that? Is it possible for you?

P: It’s possible via WBOTS.

I: Okay.

P: Yes.

I: So, you’re able to send hhhhh WBOTS after four days of mi- a missed dose?

P: *Ya* [yes] every week they are there. Hhhhh

I: Okay.

P: Every day actually.

I: Okay.

P: *Ya* [yes]

I: So, what other challenges can you think of in implementation of this box? You’ve mentioned the technical glitches hhhhh homeless people and drug users.

P: Hhhhh

I: Hhhhh cell phones wrong numbers, what else can be the challenge for you as, as health care workers providing the service, what can be the challenge?

P: It think that just about it

I: Hhhhh

P: And we’ve also had a client who refused treatment as in they don't want to take treatment because the wife was on TB treatment then as a contact he came, we tested him, results came back positive then he came, he was started on treatment then I think two days later he came through saying that "I was okay, now that you started me on TB treatment, I’m feeling more sick" and then few days later we got a call from the lab saying he’s resistant and hhhhh we told him he need to come back to the clinic. He was resistant and then the wife passed on, then that was the last of him. We never saw him again, he refused, refused, refused.I: Hhhhh

P: Hhhhh

I: So, the patient totally refused-

P: *Ya* [yes] he totally refused and he’s there with the MDR so we couldn’t get him to come to the clinic.

I: Okay.

P: Hhhhh

I: And then hhhhh the issue of staff rotation

P: Hhhhh well it’s normally you stay in a room for a year. *Ya* [yes].

I: Hhhhh

P: A full year you in one place then you move to other services.

I: Hhhhh

P: *Ya* [yes] but back in the days, you’d find that the sister is in TB room for years or the pap smear room for years but recently we said we agreed on one year. So *ya* [yes].

I: So hhhhh do you think it’s a challenge to have a TB nurse for one year in a room?

P: I think so because I believe sometime next year in March, we will be having one who is going to take over in April for training maybe at least two weeks then we show her what is what on the TB room before we leave the room. Because if it’s the new person and who has not been exposed then it’s going to be a problem.

I: Okay. So, you suggesting that you can train hhhhh the new person coming in?

P: Yes

I: To make sure they understand. So, from your perspective as a TB nurse hhhhh can TB treatment be improved using this differentiated model of care and the digital adherence technology?

P: Yes, it can be.

I: How so?

P: Because for one if I’m taking treatment from the box I wouldn’t want it to be making a lot of noise for a very long time. So, once it alarms, I’ll go and take my treatment.

I: How else does it improve treatment?

P: Technology helps with early tracing of the ones who are missing their doses and then terms of relationship with patients. There is a good relationship between us and the clients, and the box.

I: How so? Can you think of an example of a patient?

P: Okay, we once had a client transferred in, so, we give him a box, so, the client was like what if this box rings when I’m at the drinking place? We were like, how do you take this box to a drinking place? He was like, isn’t it that I’ll be taking it in the morning? Then we were like will you be drinking alcohol from early hours? No, you must do a treatment then you can leave your box-. So, I think there is a good relationship there.

I: Hhhhh

P: They don’t really despite the box thing. *Ya* [yes] some do have a problem, what am I going to do with this box when it’s making noise when there are people around. So, that’s when we advise, no you choose a time that is convenient, when there’s lesser people around. Maybe you’ll take it before you go to work, then it’s fine.

I: So, what’s the main issue with the box ringing when there are people around?

P: Like the one that likes drinking too I told her that wena [you] everybody will be knowing that you are taking TB treatment because you’ll be travelling with the box to the drinking place. So, some have that stigma that oh ayi [no] no she’s taking TB treatment because some of them they do know the, the tablets. They have a stigma that they are taking TB treatment and they are using a box. Maybe to people it feels like this client is not complying that why, that why they give her a box, so that it reminds her all the time.

I: So, is this issue of, of stigma?

P: Yes

I: Okay and also in terms of you as the TB nurse hhhhh can you comment on your workload when using the box, in relation to the digital adherence technology.

P: Aah with the box it’s fine. The only overwhelming thing is the recording, that’s it. But with the box it’s fine.

I: Okay

P: *Ya* [yes]

I: Do you think it will have hhhhh an impact on the workload you already have?

P: As long as we have someone to do it I don’t think it will be a high workload but if I am to do it myself then obviously there will be some workload because from the records to, to enrolling a client on a system I think it will be too much, ya [yes].

I: Okay, you think it will be too much? Okay, so can you please hhhhh elaborate on the positive changes of the differentiated model of care and the use of the technology hhhhh how do you think these positive changes can be sustained?

P: Hhhhh if we have someone who is constantly there, there like now we don’t have anyone and we having new clients. So, we not giving the boxes because we don’t have the device to enrol, so meaning, we only going to rely on the client for monitoring. Nothing else.

I: Hhhhh

P: *Ya* [yes]

I: So, to suc- sustain this, what can be improved from your facility level, from your level?

P: Okay hhhhh if we can have someone who will be doing the phone calls cause sometimes I’ll be working alone and I can’t, I won’t be able to do the phone calls, plus the consultation.

I: Hhhhh

P: *Ya* [yes] cause if we are two at least one will be seeing clients, I’ll be reviewing the files and checking if they missed their dose then we recall the client to the facility or send people for tracing.

I: Okay.

P: *Ya* [yes]

I: Hhhhh you mentioned that if there’s someone to make the phone call. Who do you think hhhhh that someone should be, should hhhhh we finish the project and then we hand over to the Department of Health, who could be that someone to make the phone calls?

P: Well, the intern was helping in the Department because she’s the one who sees, she’s the first one to see who’s missing the doses then we take the file then she makes the phone call, *ya* [yes].

I: So, you saying the Department of Health can also provide interns?

P: *Ya* [yes] if we can get someone who can focus maybe on the phone calls cause now they say with TB clients cause you find that hhhhh we need to call to remind them about the appointments and also to check about them. I personally feel we need to call and check about them.

I: Hhhhh

P: Because recently we’ve had about three deaths and it’s those kinds of deaths where you like no this person missed the date hhhhh cause I was checking the files and I said to sister [***] I was going on lunch and said please call this client, the last time I saw him I sent him to casualties, he missed his two days. Then she called, when I came back from lunch she said the client passed on. So, we need now maybe on week basis that one day hhhhh we make calls on a client to check up from them. Cause the other one it, it a mother and son, they both having boxes so this sone was on the red like hhhhh the intern called hhhhh to check what’s wrong, why is he missing his doses, so the son didn’t pick up the call. When she called the mother, someone has answered and said so and so’s phone hallow then she was saying I wanted to talk to so and so then they were like no so and so it’s very critical, we were even thinking of coming to the clinic but then now we taking her to the hospital. Only to find out later that it because the mother was critical, the boy now was not taking the medication and then the mother passed away.

I: Hhhhh

P: So

I: Is, is hhhhh patient the boy taking medication now?

P: *Ya* [yes] they both because he, he got TB from he was a [inaudible segment] *Ya* [yes]

I: So, has his hhhhh adherence changed?

P: Yes, it has, it has.

I: So, after talking to, to [***] what happened, to the intern what happened?

P: Hhhhh we recorded on the file hhhhh that she said they were taking the client to the hospital then we record on the file then she called again later after a few days they said no this person passed away. Then we also record on this one taking to the data room to update on the system.

I: So, the mother passed away but the boy is still alive?

P: The boy is still alive.

I: How was the boy supported?

P: Hhhhh

I: By you-

P: Yoh

I: And is he now taking-

P: He is taking but I-

I: Medication?

P: But I haven’t seen him in a while but he is taking now.

I: How did you support him, besides the phone call?

P: I think that was the only thing he got, phone call cause I didn’t see him when he came.

I: Okay

P: *Ya* [yes]

I: And what was discussed, you know-

P: Hhhhh

I: What type of support was given even through the phone call?

P: Shooo I don’t wanna lie there.

I: Okay

P: *Ya* [yes], [laugh]

I: Alright then. And hhhhh what else do you think can be improved? You mentioned that if there’s someone who can make the phone calls, so imagine that hhhhh like the way the intern has left, xxx (organisation name) has left-

P: Hhhhh

I: You know, the organisation has left and it’s now DOH implementing this on its own?

P: Isn’t?

I: How, who would you suggest will be making those phone calls?

P: Yoh, if they can send us someone, I don’t know who but if they can send us someone-

I: Okay

P: Who will be making phone calls.

I: Okay

P: *Ya* [yes]

I: So, in addition to the phone calls, what else do you think should be improved at facility level?

P: Hhhhh

I: To sustain the, this positive changes that the DAT has brought.

P: Hhhhh I think also because the intern was really of much help because if we are busy, the intern will be checking also the results for us on the lab track then it’s quicker to diagnose, quicker to treat and also quicker to do the follow ups if we have a, someone who’s detected but the person is not coming through to the clinic. It’s normally if we see you today and we say hhhhh Wednesday you come back for the results. Some they do come, some they do not come and you find that we have a detected who doesn’t have a phone call then if we having someone who’s getting that then we sending tracers. *Ya* [yes]

I: Okay. And in terms of resources

P: *Ya* [yes]

I: Can you think of what can be required in order to sustain this?

P: Hhhhh

I: You mentioned human resource, someone to assist with the phone calls or checking the lab tracker to quickly diagnose.

P: *Ya* [yes] and also I think to also get a phone because we use one phone the whole clinic so it’s somehow difficult because I’ll be using my phone for lab track, sometimes most of the time I call them from my phone.

I: Hhhhh

P: So

I: Okay hhhhh okay. So, it’s human resources and also phones?

P: Hhhhh

I: Okay. And can you elaborate on the negative changes of the differentiated model of care and use of the DAT, the Digital Adherence Technology and hhhhh you mentioned that, patients can open the box and not take medication.

P: *Ya* [yes]

I: That one of its. Can you elaborate more on that and also maybe think of how it can be address?

P: I don’t know if there can be an upgrade that will be like hhhhh it doesn’t take only to open and close for it to swap like where you can actually monitor the taking of the medication. I don’t know how that is going to happen.

I: Hhhhh

P: But because you’ll find out that the boxes are making noise then I call the intern, hey the box is alarming then she says ai I’m coming, then she doesn’t come at that moment and it doesn’t stop and say hey the box is alarming then she says just open the box and close. That’s when I found out that oh you just open and close then it keeps quiet. So, that one ah it’s not okay. Cause clients can simply open and close.

I: Hhhhh

P: Hhhhh like the one now, the one that I was saying I wanted to confirm.

I: So, you said hhhhh to address it. How can that be done?

P: To address? *Ai* [no] I’m saying I don’t know how it can be improved.

I: Okay

P: *Ya* [yes]

I: But it a challenge?

P: *Ya* [yes]

I: And then in terms of following up with the patients hhhhh what are the challenges and how can they be addressed? Like when you are making phone calls and home visits, what are the challenges with those?

P: Well, with phone calls we do get them ninety percent of the clients, it only a few is like the number is no longer in use or they gave us the wrong number or the one who doesn’t have a phone at all hhhhh with the home visits it’s when we get a wrong address or people who are living in the streets then it’s a huge challenge cause the guy would say *ai* [no] kerobala [I sleep] in front of the Sunnyside police station, when you get there, they are not there. Like hhhhh most of the people sleeping in the parks, by the time you do the walks to go search for them, they are not there because at night yes, they do sleep there but in the morning they pack up because now people are coming in. *Ya* [yes] It’s a challenge.

I: And how can these challenges be address starting with phone calls hhhhh getting wrong numbers or the number changes?

P: I think for the wrong numbers we must just have a phone whereby when the client says 072 you actually punch it in and call right there and there.

I: Hhhhh

P: *Ya* [yes]

I: And for those who change the phone numbers later on?

P: Well, for change, always ask the, the client’s phone number when he come for a visit, for a follow up.

I: Okay

P: *Ya* [yes] that’s the best we can do there.

I: Okay. And in terms of home visits, wrong addresses and not finding-

P: Whoo-

I: The clients

P: That one, we’ve had two resistants’ one. One was Covid positive and TB positive and resistant hhhhh gave us wrong address, we couldn’t find that one.

I: Hhhhh

P: So, I don’t know what happened to the, to the client and people around. So, it’s, I don’t know how we going to verify the address.

I: Address

P: *Ya* [yes]

I: Okay hhhhh so, please tell us hhhhh the system level of structures that need to be improved in order for us to, to integrate the differentiated model of care and the medication device technology on to the existing TB programme. So, what needs to be done?

P: I think that formal hhhhh training

I: Hhhhh

P: On the system

I: Yes

P: So that even though, even when the intern is not there I can do it on mine

I: Okay

P: And we can have a device logged in or maybe, I don’t know, if I can have if it’s an app or I can have it on my phone, I don’t know. *Ya* [yes].

I: So, what exactly would you want to be trained on?

P: From the enrolling to the setting up.

I: Hhhhh setting up of?

P: Of the time.

I: Of the time?

P: Of taking medication and also to view the, the progress, *ya* [yes].

I: And in, in terms of the box?

P: The charging, because hhhhh sometimes they do come with boxes saying that it doesn’t alarm anymore. *Ya* [yes] the charging, *ya* [yes].

I: So, who should be doing the charging?

P: [Laugh] the intern was charging the boxes, yes. *Ya* [yes]

I: So, going forward when we scale up, who do you suggest should be doing the syst- the charging?

P: If we get someone to help us.

I: Okay

P: The one who’ll be calling, we do have an extension even though the plugs are not working now this side

I: Okay

P: Like hhhhh we’d plug things with the extension ten she plugs maybe four boxes at a time.

I: Hhhhh

P: *Ya* [yes] four batteries, *ya* [yes]

I: And in, in terms of technical issues, you mentioned that there can be technical issues, like, the alarm the’ll- the alarm does not stop-

P: *Ya* [yes]

I: Ringing. So, how do you, what structures do you need as Department of Health to deal with those issues hhhhh in scaling up?

P: I think it also goes back to the training cause she managed to silence them, how, I don’t know but she did something then they were on silent.

I: Hhhhh

P: *Ya* [yes]

I: Hhhhh can you tell us, what systems that it placed that could monitor hhhhh the differentiated model of care and use of the device technology? (…...) so, do you, *ya* [yes] do you monitor or capture the challenges and the successes or problems using the, the technology?

P: She used to.

I: How?

P: She writes I called, like used to show me, I called, this is what I got. Like-

I: Hhhhh

P: We do on the file.

I: Yes

P: Yes

I: Okay. So, what needs to be in place for you to continue doing that?

P: We need a person one and that device for enrolling. *Ya* [yes]

I: What device exactly?

P: She was using a tablet so I don’t know.

I: Do you think hhhhh a tablet will also be useful for you?

P: *Ya* [yes]

I: Or another-

P: Cause a phone-

I: Phone device?

P: Cause a phone is small, I know I use it when I do this Clicks registration for clients then in have to rotate it because it’s too small, unlike the computer.

I: Hhhhh

P: Ya [yes]

I: Okay. So, you said the intern was capturing the challenges and the successes hhhhh was there any point where you did it personally, capturing?

P: No. Only wrote on the file.

I: (…...) So, is there some common tool or approach to do in this clinic shared by other health care workers?

P: *Ya* [yes] we do have hhhhh like the Clicks one, but just that the Clicks you don’t monitor, you just sending clients to collect medication at Clicks. And the NMC, is it NMC or what, *ya* [yes] it’s the one where we enrolled, we notify TB clients.

I: Hhhhh

P: When we get a new case then we enrol them there.

I: Okay

P: Just for recording, I think. Not monitoring.

I: Okay

P: *Ya* [yes]

I: Alright. Hhhhh (……) so can you tell me the gaps which exist in the way the intervention is being delivered? And, *ya* [yes] what are the gaps?

P: Hhhhh well, the gaps with device can only be that one, where you get your greens but when the client comes, clinically it doesn’t show that, the client was on the greens.

I: Hhhhh

P: So, *ya* [yes]

I: That’s the main gap you have?

P: *Ya* [yes]

I: And suggestions for improvement?

P: Whoa that one, which I don’t know how we gonna do it.

I: Which is?

P: The opening and closing.

I: Hhhhh

P: Hhhhh

I: Okay. So hhhhh before we wrap up hhhhh can you tell me your positive and negative experience with hhhhh this whole programme?

P: Well, the positive is that you able to monitor.

I: Hhhhh

P: And you can get it quicker if the client is not complying. *Ya* [yes]. And the negative is that tricky one where you find that cause I think Friday I had a client hhhhh due for discharge but clinically he wasn’t okay, so I couldn’t discharge him but then he completed his TB treatment and I can’t give him more. So, I’m not sure as to if he was really taking his treatment or if he was just opening the box and closing it because one he stays alone and he doesn’t have a treatment buddy or anyone who’s monitoring or supervising with his treatment.

I: What’s a treatment buddy?

P: Hhhhh treatment buddy is the one who, like, how do I put it, a supporter anyway. Like it can be your friend, family. *Ya* [yes] it’s a treatment buddy.

I: Hhhhh

P: *Ya* [yes]

I: So how do think this can be integrated? Treatment buddy and the digital adherence technology?

P: Well, I think it’s easier when one has a treatment buddy cause we wouldn’t worry much and we wouldn’t expect more red on the system since you have someone who’s motivating you and supporting you. *Ya* [yes]

I: So hhhhh in terms of this opening and closing, you said you not sure of what can be done but hhhhh in the meantime, what would be your suggestion hhhhh to over and above the box, what do you suggest can be done to ensure that the patient is actually taking treatment?

P: I think close monitoring of the ones who you see they are not clinically, they are not posing well, then instead of giving two months, you can give that one month. So that hhhhh you see them more often.

I: Okay. So, you are currently giving patients treatment for two months?

P: It depends. If I’m seeing you for the first time, it’s gonna be for that two weeks either you are new here or a transfer in, I’m still gonna give you that two weeks. Then when you come I give you that one month, then when you come again, say maybe we hhhhh we gonna be reaching a point where we switching you from the intensive phase to the continuation phase then I’ll give you maybe a week. We do the test today for the smear then when you come in your next time hhhhh if the results are fine then we switch then I can give you two months.

I: Hhhhh

P: Depending on how I see you.

I: Hhhhh

P: Yes

I: Okay. So, you suggest that in addition to the box, close monitoring, close clinically monitoring patients?

P: *Ya* [yes]

I: Okay. I think we’ve reached the end of our discussion. Thank you very much for hhhhh your inputs. The time is 13:59.

P: You’re welcome.
