# Supplementary material for: Acceptability of Digital Adherence Technologies to support people with drug-susceptible TB in South Africa
Source: PLoS One. 2025 Sep 24;20(9):e0332103. doi: 10.1371/journal.pone.0332103 (PMC12459780; doi:10.1371/journal.pone.0332103)
Supplement: S4 File — (ZIP) [file pone.0332103.s004.zip › S4 Transcripts/HCWs and Stakeholders/IDI 12- HCW.docx]

**TRANSCRIPTION NOTATIONS**

| **Label Key** | **Meaning** |
| --- | --- |
| **I** | Start of each new utterance by the Interviewer |
| **P** | Start of each new utterance by the Participant |
| **N** | Note taker |
| **{ }** | Indicates that details were changed or pseudonyms were used to anonymise data |
| **( )** | Indicates the description provided to anonymize data |
| **XXX** | Words were omitted to anonymise data |
| **-** | Breaking into a sentence by the next speaker |
| **…** | Pause or drawn-out words |
| **[ ]** | Indicates noise made, e.g. [laugh], [sigh], [pause] |
| ? | Beginning of utterance by unidentified speaker or questionable text |
| **[inaudible segment]** | Unclear section of the recording |

I: Okay. Hello again, uh do you allow us to audio record this interview?

P: Yes ma’am.

I: Okay uh date of the IDI: xxxx (interview date). Location: xxx [clinic name] clinic. Language used for the IDI session: English. xxxx .Huh the time which the interview starts 12:07. My name is xxx [name of the interviewer]. Alright (……) so I’m going to start with our questions. What is your role as a treatment supporter, like how long have you held this, like done this?

P: Two years now, okay and my role is to make sure that the, the box is fill with pills and to make sure that uh I check the lights, if it shows uh green or orange. Okay, orange shows that the patient should come for treatment.

I: How long have you been supporting the patient with the box?

P: I think it was since last year, *yah* [yes] last year.

I: So, you only have one patient that you’ve been uh assisting with taking the medication?

P: *Yah* [yes] only one patient.

I: Only one patient?

P: Mmm.

P: So, uh when the patient was diagnosed with the TB, were you available?

P: Yes

I: Like how do you support the patient in taking care, in taking their medication- their TB medication?

P: How do I support them like how, like?

I: Do you, do you just go to open the box or you just assist patient in taking the medication?

P: I’m uh I assist in taking all the medication, like taking the medication out of the box and give her. I assist her with all the medication because there’s a lot of medication that she’s taking you see.

I: Okay.

P: It’s not only for TB.

I: Okay.

P: So I take all the medication out of the pack and then I give it to her to drink, *yah* [yes].

I: Okay, and who are you supporting?

P: Who am I supporting, it’s my mother.

I: Oh okay, okay, alright-

I: And then uh -okay besides making sure that the pill box is filled with medication. What else do you do to support the patient?

P: I entertain her, I make her forget that she’s sick.

I: Okay.

P: *Yah* [yes]

I: Forget her that she’s sick how?

P: Like make her forget that she’s sick, like take her out, take her out for walks, like make her gym, things like that, *yah* [yes].

I: Okay.

P: Nice breakfast in bed, things like that, you know [laugh]

I: Okay, besides uh, besides your mother that you’ve been supporting, have you ever supported any other patient?

P: Ah, no

I: No, it’s the first time?

P: My dad but then he was not uh he didn’t have TB, it was something different.

I: Okay.

P: So, it’s not involved *neh*? [right]

I: Okay

P: *Yah* [yes] it’s only my mom.

I: Okay. So, like uh your experience in your, your mother-

P: Mmm.

I: Tell me about it, like what is your experience actually with supporting your mother?

P: At first it was bad. *Yah* [yes] because she’s my mother, you know, seeing her like that but then as time went by with the pills and everything, there was a lot of change. Plus, the box, it also helps with time to take medication, you know, so, everything like, the pills are also helping her, like she’s recovering. At times she was not fine but then, I’m not saying it’s fine now but then now [laugh] it just, *yah* [yes].

I: Okay, how was she when she was diagnosed with uh TB at first?

P: Yoh she was critical.

I: Mmm.

P: She couldn’t speak she was like a zombie I could say, *yah* [yes].

I: And then how, how does, how did that make you feel?

P: Very bad.

I: Mmm.

P: As I said, like at first it was, it was hectic but then as time went by with the pills and everything, things started changing. *Ankere* [isn’t] I get used to everything. So, *yah* [yes] everything changed and then uh she, she, she’s getting better, I think, *yah* [yes] from my perspective, I don’t know from the inner side but then from my perspective.

I: You find, you see her getting better?

P: *Yah* [yes]

I: And then when she was diagnosed uh were you there on the day of diagnose when she was told about this, and then how, how did she receive it, the results and how did you feel?

P: Huh, I was not around like at the hospital.

I: Mmm.

P: But then, me I felt bad but then they comforted us telling us that “no it’s not something, she cannot die over such thing, it’s curable, pills are there” so *yah* [yes] they comforted, like *bare* *comfortile* [they comforted us] *yah* [yes]

I: As a family.

P: As a family, *yah* [yes].

I: Okay, so how many people are you staying with at home as a family?

P: It’s me, my mom, my dad, my two siblings.

I: Okay.

P: And my step bother, five, *yah* [yes].

I: Okay, so did you all get tested for TB since she was?

P: [Laugh] no.

I: Okay, and then uh so does the facility like *mo* *cliniking* [here at the clinic] do they know that you are the one who is uh supporting your mother, like?

P: *Yah* [yes]

I: Okay.

P: Because I also bring her for check-ups and everything.

I: Oh okay.

P: Even where I collect the pills also, ko Mediclinic that side, they also know it’s me.

I: So, at Mediclinic which, which pills are you collecting?

P: It’s the *bare* *keing* [how do they call it] yoh the *tse* *tsadi* [the one for pain block] pain block or, yoh I should have come with the list, it a lot of [laugh] it a lot of, it’s just a lot of pills, like twenty pills and so.

I: Okay

P: *Yah* [yes]

I: So, she’s taking all those pills?

P: *Yah* [yes]

I: So, she doesn’t have TB only, she’s also have other-

P: *Yah* [yes]

I: Oh okay, other chronic medication. Is it chronic medication or?

P: *Yah* [yes] it also chronic.

I: Oh okay

P: Mmm.

I: Okay, so uh I would like to know about the, this smart pill box, the box.

P: Okay.

I: *Yah* [yes] what do you know about it? Can you, can you tell me about it *nje* [like]?

P: [Laugh]

I: Like [laugh] if someone like who, like someone who doesn’t know about the box, how can, what can you tell them about the box?

P: I can tell them that the box is should be their friend [laugh], if you have a lot of things on your mind, the box reminds you to make sure the patient takes pills on time, like it makes things sometimes easy for you. I think the box is also uh stated hore [that] if iyaloa tswantswe unwe de pilisi [if the box beeps, you have to take your pills] If it shows orange then the patient should come for check-ups, if it shows red then the box should come, should come this side. You should bring the box here for battery.

I: Mmm.

P: *Yah* [yes].

I: Okay, so whenever the box like uh the patient, the box ring, how does it remind the patient *hore* [that] this is the time for-

P: No, it reminds me.

I: You?

P: *Yah* [yes] it reminds me.

I: Oh, so you, you are the one who’s a bit reminded about the box, then *wena* [you] what you, what do you do?

P: I take all the pills that I give the patient.

I: So, the patient doesn’t have, doesn’t like sit with the box and know about the box?

P: *Hha* [no].

I: So, they don’t know about the box?

P: The patient doesn’t know about the box, they explained to me. She has a clue but then she doesn’t know how to use it or when to use it, she doesn’t a right she can take the pills. So, she just knows hore [that] there’s a box that has my pills and at time it will make just a bit of a noise.

I: Mmm.

P: Then, then *mina* [me] because it’s my responsibility, I take the pills then I give it to her. She doesn’t touch, she doesn’t do anything about the box.

I: Oh, so where do they keep the box is it with you all the time?

P:Not always with me but then in her room maybe upstairs.

I: Okay.

P: Not always with me but then in her room maybe upstairs where she cannot reach. The thing I like about this box is that it alerts you, can never miss day without medication because it irritates.

I: It irri- how does it irritate?

P: *Twi,* *twi* [imitating the beeping] [laugh].

I: Okay, and then how do you, how do you, did you get to know about this box?

P: At the clinic, they came and explain it to us.

I: They came to you or?

P: No, we came here.

I: *Ehhe* [yes] *yah* [yes] when she came for check-up.

I: *Ehhe* [yes]

P: They explained that there is a box that they have to issue us-

I: Was it there when they came for check-up or when they came for consultation for maybe starting of the medication?

P: Yah [yes] it was when she was starting the medication here because it was after she was discharged ko [at] xxx [hospital name] .

I: Okay

P: And they referred us here *hore* *aye* *ko* *check*-up [to do the check-ups]

I: Mmm.

P: *Aye* *ko* *checkiwa* [to do the check-ups] should come this side.

I: Mmm.

P: So, *yah* [yes]

I: Mmm.

P: The time she came here.

I: Okay

P: She came, started the treatment, like taking the dose here-

I: Here?

P: And everything.

I: Okay, and who explain it to you?

P: Yoh, nurse, this nurse, I forgot her name, this light skinned one, the- because I think there’s only three TB uh nurses here.

I: Okay. So, were you there with the patient or you were there like alone and they, they explained to you?

P: We were together, we were together.

I: Okay, you were together?

P: *Yah* [yes]

I: Okay. What was your first impression when they were explaining to you about this box, like how did you feel about the box when they were telling you about it ?

P: I thought it was not necessary, but why use the box? why does it ring? why open? why alert? but then as time went on I could see oh yah [yes] at least it does something, yah [yes] it helps two minutes nyana [just two minutes] yah [yes] yah [yes].

I: Oh, it helps two minutes nyana? [just two minutes]

P: I didn’t see the necessity for us to be given the box. I thought maybe they can just give us the pills, yabo [you see] but then as time proceeds, I saw the necessity, yah [yes].

I: Okay. Was the information you were given enough to know about the box?

P: Yah [yes] I think so because it doesn’t contain a lot of things in it because there’s only three lights and then there’s only a sound and there’s batteries there so there’s nothing complicated.

I: When they were explaining to you what did you like most about that box that explanation?

P: The alert, *yah* [yes]

I: The alert?

P: Yes that it shows time for treatment and time for check-up. So it’s good, it’s actually good.

I: Okay. So, since it was the nurse who explained to you, do you think she was the right person to explain to you as a treatment supporter about the box?

P: *Yah* [yes]

I: Did you receive sufficient information?

P: Yes, it was, *yah* [yes] it was also useful, *yah* [yes].

I: For you?

P: *Yah* [yes]

I: Did they explain to you about adherence, that this person is taking medication, and then this is the adherence, when you come back to the clinic like showing you if the patient is taking the medication, this is how it works on the system like did they show you?

P: Huh, I’m not sure if they showed my dad *ko* Mediclinic (at Mediclinic) but then-

I: No, I mean here.

P: No

I: Here, with the technology, with the box when they explained to you about the box did they show you anything?

P: She just explained hore [that] we should be aware of the lights and the sound and take care of it. It shouldn’t stay in a hot place or things like that, yah [yes].

I: Okay, okay

P: Mmm.

I: So, *wena* [you] do you think it was useful the way they explained it to you or what?

P: *Yah* [yes] it was useful. It is still useful even now.

I: Okay (……) from your perspective as a treatment supporter *neh* [right] can you describe like the benefits and then like the SMSs, did you receive any SMSs uh phone calls home visits?

P: Phone calls, we received when- *yah* [yes] phone calls we received. Home visits, we never received.

I: Okay

P: What else?

I: Oh, there was no, like in phone calls, what were the phone calls all about?

P: Oh, like the nurse, I don’t know who called but then maybe let’s just say the nurse

I: Okay

P: She, she just wanted to confirm the reason why did the patient miss medication but it was the time that she was admitted.at xxxx [hospital name]. The nurse called wanting to know if she had the box with her at the hospital, she was just concerned. She was just showing concern about the box asking where its is and why she [supported patient] missed medication. Then I explained to her *that*  “no, she has been admitted” then she told me *that*  okay we should take the box to the hospital so that *they can see because the medication is always inside.*

I:. How, how, how does the nurse know *that* the patient missed the medication, did they explain to you on that part *?*

P: *Yah* [yes] *bare* [they said] through the box.

I: Yes, but the nurse doesn’t have the box, the box is with you.

P: *Yah* [yes] if you open it-

I: Yes

P: *Ankere* [isn’t] it should make *twi* *twi* *twi* [beeps]

I: Yes

P: So, it clicks that side that “today the box was opened.”

I: Where, which side?

P: *Hhe* [pardon?]

I: Which side?

P: I don’t know which side, but somewhere around the TB uh facility, it gives the information that the box today it was not opened.

I: Oh, okay

P: [Laugh]

I: Okay [laugh] so, did you receive any couns- [counselling] did the patient and the family receive counselling?

P: Counselling?

I: Mmm.

P: No, no counselling.

I: So-

P: *Nna* [me] I didn’t receive any counselling, I’m not sure about my mother.

I: Mmm.

P: And my dad *ayi* [no] that one doesn’t need any counselling, never received counselling.

I: How do you mean they do; they don’t need counselling?

P: Like, what, what counselling should we get?

I: About the box, about the treatment, about everything.

P: Oh, *ayi* [no] we never got everything, counselling, never.

I: Okay. And then the sister called about that time, about the missed visits *neh* [right]-

P: Mmm.

I: Huh ,did, how did uh you feel about receiving that call?

P: Ah very nice, very cool that these people care about my mother even though they are not around do you understand me?

I: Mmm.

P: *Yah* [yes] but I felt happy. I even told her *hore* [that] “keep, you’re doing a very good job.”

I: Okay. So, did you, have you ever received SMS, I don’t know which phone, which contact uh are they using here, are they using your phone or your mother’s phone or your father’s phone uh to, to, to get SMSs, I don’t know. Did you guys receive SMSs about the reminder-

P: *Yah* [yes]

I: Or maybe how often?

P: Mmm if she misses a day, say nine.

I: Mmm.

P: Like saying before it jumps to the next day.

I: *Yah* [yes]

P: It says, “don’t miss your medication before twelve, twelve middays.”

I: Okay.

P: I receive those SMSs.

I: You do receive SMSs, and-

P: It’s my phone and my phone.

P: It’s my phone and my phone.

I: How do you feel about receiving those SMSs also?

P: Even if I’m far and I’m drunk when I see that SMS then I quickly run home.

I: So, *kunale* *de* challenges *tse* [is there any challenges] that you received uh challenges in, in, in the box?

P: No.

I: Any challenges, like the network issues?

P: No.

I: Huh maybe let’s say they phone, like you missed, let’s say like you receive SMSs maybe even though the patient has, even though maybe you opened the box-

P: Ehh [no]

I: Did you ever receive SMSs while you’ve opened the box?

P: No

I: You’ve never received that?

P: No

I: The benefits of the box, are there any benefits of using the box?

P: Ash [eish] benefits, me?

I: Benefits you as a treatment supporter and then with your mother also, and then, *yah* [yes] what can you say that you have benefited in using this box so far?

P: *Yah* [yes] maybe the time because usually I’ve released that it rings at nine, so I’ve benefited uh like my changing of, like routine. Nine o’clock I know breakfast should be done, pills should be taken because, *yah* [yes] like I’ve benefited just the routine.

I: The routine of taking the medication?

P: Yah [yes] maybe used, I used to give her pills like around eleven

I: Mmm.

P: *Ankere* [isn’t it] it’s three times

I: Mmm.

P: Some pills, *tsa* TB *ke* *de* one time [for TB it’s one time].

I: Mmm.

P: Maybe I could give her maybe *vrou* *kaboma* eleven [in the morning around eleven]

I: Okay

P: Then *hape* *kaboma* two, then *hape* *kaboma* eight [then again around two, then again around eight].

I: Okay. So-

P: La TB *nou* [for the TB now]

I: Mmm.

P: It changed the routine.

I: *Yah* [yes]

P: It’s morning nine o’clock

I: *Ehhe* [yes]

P: So, it changed it benefitted me because it makes me give her pills early in the morning pn time.

I: Nine o’clock

P: Very early.

I: Okay. So, besides TB medication, is there any other medication that has been kept inside uh the box?

P: No, only a TB

I: So

P: Only a TB medication, but then they also give us this pack in a while, they also said it’s for TB and what.

I: Okay

P: And what, what.

I: *Yah* [yes]

P: *Yah* [yes]

I: Oh, so you only keep medication TB in the box?

P: Yah

I: You said the box rings at nine o’clock. Have you ever oped the box at nine o’clock then again you open the box after an hour or two.

P: Yah [yes] it happens because you know why? It can be nine and the weather is not nice and I am still sleeping then it rings at nine and the patient has not taken breakfast yet. So, I can just open it just to keep it quiet but then I know hore [that] I should-

I: You should open it for medication.

P: *Yah* [yes] then after breakfast, after everything, that’s where I reopen it again.

I: Okay. So, was ever a time where you forgot maybe like, like you’re cold, you didn’t wake up early for that time to give the patient medication ka nine o’clock. Then you forgot that “I haven’t given the patient medication”. Has that ever happened?

P: Ayi [no] no ayi, ayi [no, no] I will never forget to give my mom, I’ll never.

I: Oh, okay. So, if maybe you are not available as a treatment supporter does the patient know how to open the box?

P: *Yah* [yes] she knows how to open but then she doesn’t know which pills to take.

I: Okay.

P: Or how many doses to take. I strictly told her that I made myself available and I even told her that she must not dare touch the pills. So, she doesn’t touch the pills, even I if I’m not around I make sure that, you understand me?

I: Mmm.

P: So, *yes* I’m the only one that’s touching her pills. She doesn’t have access to open those pills. She knows, she has an information that there’s a box, there’s pills that I have to take but she never opens it.

I: Okay. So, does this reminder of the box remind her to take other medication besides for TB

P: Not really because she takes other medication different times of the day.

I: So, has your mother told anybody except family about the box, like there are any other people that knows about the box?

P: No

I: So, it’s only the family that knows about the box?

P: *Yah* [yes]

I: Okay. Besides you, if you are not there, is there anybody that can be able to assist her in taking medication?

P: No.

I: So, it only you who knows about the box?

P: *Yah* [yes]

I: Don’t you feel that maybe you want to explain to anybody to take the box?

P: Ah

I: Like to take the medication, like maybe, your, your siblings or your dad?

P: I feel like it’s too much for them, it’s fine with me. It’s too much pressure, like-

I: How, in what way? [Laugh]

P: Explaining that take care of the box, listen to the box, medication *ayi*, *ayi*, *ayi* [no, no, no].

I: So, you don’t want to explain that [laugh]

P: *Ayi* [no]

I: So, you can’t, so you’re telling me that you can’t explain this to another person?

P: No, I can explain it but then I won’t give the person the duty to do it, to open the box and to give my mother medication, no.

I: You want that to be your baby?

P: My baby [laugh].

I: Okay. And then have you travelled with your mother, with the box?

P: Uh recently, no. Ever since she has been given a box we have never travelled.

I: You have never travelled?

P: No we haven’t. We had to in December but then she got admitted at the hospital.

I: Okay. So as a treatment supporter neh [right]? What can be improved using this uh this medication box?

P: Maybe the, maybe the beeping of the sound  *s*hould be connected on the phone also.

I: So, meaning you want that when the box beeps it should also beep with the, together with the alarm, with the phone so that it can, like an alarm also? Link them together with the phone?

P: Only if it’s possible.

I: Okay. In terms of the sound uh is the sound, okay?

P: *Yah* [yes] no the sound is-

I: It, it, it’s fine?

P: Yes.

I: And the size of the box, is it big, small-

P: I think its fine. The pills fit.

I: And the colour of the box, since it’s white?

P: *Yah* [yes] it’s right, it’s fine.

I: It’s fine? [laugh]

P: [Laugh] You want to make it orange?

I: I don’t know. I am asking you what you would suggest.

P: Oh.

I: Maybe do you have any, maybe, do you think maybe do you think maybe it can be something with any colour?

P: No.

I: Okay. So, do you think this box maybe can be used for other pills besides TB medication?

P: *Yah* [yes] it can also, yah it can also be used for other pills, it can help beause if it doesn’t allow you to miss a dosage. It’s good, it actually good. I really like it for that.

I: Can you explain, like elaborate on the positive changes that happen after using the box? Any positive changes in terms of adherence ?

P: Huh ever since, positive, the positivity that I found after I using the box?

I: Yes

P: She [supported patient]  has never missed the dosage after she received te box but then the time before we got the box she used to miss one or two because you know these thing can be stressing but then after the box, she never missed the medication.

I: Okay.

P: Mmm.

I: Okay. Alright, any negative changes caused by the box?

P: No, negative one.

I: And then like in terms of phone calls, do you find it useful for people to call you, do you easily answer when they call you?

P:  Yes I answer and its good. It shows that they [health care workers] care.

I: Okay, okay

P: And the SMSs also they’re good.

I: Do you think uh you’ll able to, to explain to the people about the box, those people like “what is this box all about and what does this box do”, or you guys are not ready to, to go there?

P: I can explain, *yah* [yes] I can explain.

I: Okay, any comments about the box, SMS, phone calls? You can share any comments that you’ve got, anything that you want to share with us, any interesting.

P: The comment is that you are doing a good job, nothing negative ever since I’ve started giving my mom pills with this box. The SMSs are a good supporting structure, no stress. It shows you care.

I: *Yah* [yes]

P: *Mina* [me] I’m on the positive side of this.

I: Mmm okay. Any suggestions that you’ve got, maybe any improvement?

P: Mmm improvement, let’s connect this box with the phone.

I: Like the time of taking medication or what?

P: Yes if the box beeps maybe it can buzz the phone also or send a message at the same time the box is ringing.

I: Oh, the box should ring the same time with the alarm on the phone?

P: Yes because you will find that the box is in the other room and I’m in another other room

I: Yes

P: It can ring and I don’t hear it maybe I have my own stress

I: Yes, yes.

P: Then other patients who don’t have TB will be helped if the box is implemented. It will also help them because they won’t have a lot their our mind about remembering pills*.*

I: Alright, okay. Do you think other patients besides the TB patients will benefit from this box?

P: Yes they would. It’s also going to help them because we won’t have a lot in our mind, pilisi, pilisi [pills, pills] you know.

I: *Yah* [yes] any other comments? Anything that you want to say about the use of this box?

P: Mmm the use of this box is user friendly, it’s good.

I: Okay

P: Mmm *yah* [yes] it’s strictly consistent with time I am loving it.

I: [Laugh] thank you so much for this mmm.

P: [Laugh]

I: Thank you for, for agreeing to take part in the interview and then thank you so much for answering the questions uh end session is

12:42.

P: 12:42
