# Supplementary material for: Acceptability of Digital Adherence Technologies to support people with drug-susceptible TB in South Africa
Source: PLoS One. 2025 Sep 24;20(9):e0332103. doi: 10.1371/journal.pone.0332103 (PMC12459780; doi:10.1371/journal.pone.0332103)
Supplement: S4 File — (ZIP) [file pone.0332103.s004.zip › S4 Transcripts/HCWs and Stakeholders/IDI 13-HCW.docx]

**TRANSCRIPTION NOTATIONS**

| **Label Key** | **Meaning** |
| --- | --- |
| **I** | Start of each new utterance by the Interviewer |
| **P** | Start of each new utterance by the Participant |
| **N** | Note taker |
| **{ }** | Indicates that details were changed or pseudonyms were used to anonymise data |
| **( )** | Indicates the description provided to anonymise data |
| **XXX** | Words were omitted to anonymise data |
| **-** | Breaking into a sentence by the next speaker |
| **…** | Pause or drawn out words |
| **[ ]** | Indicates noise made, e.g. [laugh], [sigh], [pause] |
| ? | Beginning of utterance by unidentified speaker or questionable text |
| **[inaudible segment]** | Unclear section of the recording |

I: Do you give us permission to be audio recorded?

P:Yeah, I do.

I:Okay, thank you. Date of the I D I xxxx (interview date), location, xxx [ Clinic name] Clinic language that will be used. English. P I D of a participant is huh xxxx ,the time at which the session start, it's 11:14 AM.

I:Okay. Um, what was the title of your current, um, sorry, previous position?

P:Uh, okay. I was a research assistant at xxx [Clinic name] Clinic. Mm-hmm. And then I was working hand in hand with, uh, xxx [ nurse's name] , who is the TB nurse at xxx [ Clinic name].

I:Okay.

P:Yeah.

I: And then how long did you work, um, as a research assistant in this facility?

P: It was only a xxx (number of months) opportunity.

I: Okay. xxx months. Okay. And then when it comes to patient care and counselling. What were your roles and responsibilities?

P: Okay. Okay. Firstly, uh, patients are the people that we deal with on daily basis and then, we were there to make sure they do adhere to their medication. They do take their medication as prescribed, so we were there to push them to finish that treatment as they were requested to see the results. Yeah.

I: Okay. Did you have any role when it comes to counselling, any patients who needed counselling? Were you involved in counselling?

P: Yeah, I did. Unless if there was a situation where I see it's bigger than I, so I will invite the TB nurse to come and assist. If not, I will a ask the... nurse for mental health to come and do their psychiatry professional work to that person because some people come at the clinic with challenges. Uh, you find people deal with backgrounds that are not well equipped or anything. So, they need somebody to talk to, whereas at home they do not have someone to talk to. Yeah. So, I believe at the clinic is where they're supposed to come and talk and get all those counselling.

I: Okay. Um, were any of those sessions successful? Did you see any changes or improvement after the counselling sessions, did you see any patient improving who maybe was struggling before and then undergo the, the counselling process and then afterwards there was improvement?

P:Yeah, I will say they were a big success because at first you, you get a patient who's not willing to take their medication and then after you do talk to them and show them that they really need to take the medication for them, not for other person, they do continue to take their medication and end the treatment session. They then come to clinic to thank whoever that got involved with them, that they truly showed them the way, whereas they were, they weren't seeing the right procedure or way or the purpose to take the medication because as they see themselves, they thought maybe it's over with them or it's the end when they find out they have TB, yeah.

I: And what were some of the reasons that made them not to take medication?

P:Uh, in most cases, one, you can say unemployment, and at a town, most the patients are homeless. Uh, you find a person who's not, who does not know where to put the box, they're always on the street. Like they, they complain. They cannot roam around with the box. They do not sleep at the same place. You find they put the box there and then tomorrow they wake up at the other place. So yeah, I can see unemployment and being homeless.

I: Okay, I'm speaking of homeless people, so in your facility, you had a population of homeless people who were using the DAT (digital adherence technology).

P:Yes.

I:Okay. And then speaking of the DAT , did you perhaps, um, have cases of patients who reported maybe losing the box or the box being broken since you mentioned that sometimes they don't have a, um, a safer space to put their box. You know, things like that. Did you have any cases of patient who came reporting that, ah, maybe I've lost my box, or My box was broken where I stay, this and that?

P: Uh, it was only one incident, whereby a client came and then they said they put the box inside a bin where they stay then the municipality came and took those bins and the box was taken with the bin and all the medication. So, they came to the clinic to ask more of medication and to be replaced with the box.

I: Okay. And then did that patient get another box?

P: Yeah.

I: Okay. Okay. Um, so you mentioned unemployment and staying in the shelter. Um, as the cause for some of these patients, to skip treatment, right?

P:Yeah.

I: Um, can you elaborate a bit on that? What do you mean it's unemployment? How does being unemployed, you know, um, affect their medication intake or for them to adhere to treatment?

P: Okay. I can say firstly, I know with TB medication; it's recommended that its best if you take them before eating, but then after taking your medication you have to take something to eat. But then with this situation, they know they have to take medication, whereas they do not know where the next meal will come from, or they do not know where they're going to sleep . Yeah, you cannot take medication on an empty stomach.

I: Mm-hmm.

I: And then with this, um, population of homeless people who were using the DAT do you know, perhaps what was the, the percentage of them in your facility, how many would you say.

P: I can say we have maybe 10 plus.

I:Yeah.

P: Yeah. 10 plus patients

I:Okay. Mm-hmm. And how were they doing? Were they adhering? Or we had some challenges.

P: Okay. Hence, you said, do we do counselling, or do we get involved in such? Uh, firstly, when we come across those patients, we make sure that before we hand over the box, we examine our situation, we talk to them and find out whether they're going to use their box or whether they're going to see any challenges at their treatment. Mm. What's, what's this treatment timeframe given? So, we made sure that we gave them, uh, supplements to make sure that they do take their medication. So, supplements were, were really helping with these patients because they knew when they come to clinic, they're going to get supplements. If we give you supplements, we have to make sure that you gain weight and your TB is getting better than before you came. Yeah. Then we can continue giving you those supplements. Yeah

I: Okay. So, amongst these, um, homeless people who were on, who were on DAT (digital adherence technology) or using DAT , did you perhaps, um, have some who refused the box when you were offering them the box?

P: Yeah. Uh, with my situation, no. No one because I made sure I talk to them, and I showed them the, the role of the box and the importance. Because you can see now in South Africa as a whole, I can say we are experiencing loadshedding and most people rely on their cell phone but if you find your cell phone dies because of loadshedding , where are you going to see the time or something to remind you. Yeah. So, the box was the best option for them.

I:Okay. Um, now I, I would like to find out, um, what do you know or what do you understand, um, about ASCENT. Um, if you were to explain to someone who doesn't know anything at all about ASCENT.

P: Mm-hmm.

I: Um, what would you tell that person?

P: Oh, okay. So, I would say ASCENT is simply there to push adherence using technology, uh, because they have, I believe they were studies that were there before ASCENT came, and then they were not equipped in technology. In today's century, we believe in technology, and we live in technology. So, without technology it's more like we are more behind. So, with ASCENT it's more, I can say it's, it's, mm, what can I say?. I can say I put it first before the other studies because with the other studies, you can do them face to face and stuff, people get tired coming to the clinic and sometimes they do not have money to come to the clinic. So, with technology, we know we get in contact with you anytime in the comfort of your own home and it's promoting adherence .

I: Okay. Um, you mentioned, um, the technology, right? Um, if I may ask which technology or um, DAT were you implementing in your facilities? Between the box and the stickers?

P: I was using the box.

I:Okay.

P:Yeah.

I:And you never implemented the stickers before?

P: No.

I: Okay. And you said something about technology being able to monitor patients remotely and unlike them having to come to the clinic every day.

P: Yeah.

I: So how exactly where you monitor monitoring patient using this? Um, this technology or DAT.

P: I was using the xxxx (adherence platform name). So, with the App I will monitor clients that I have. It depends whether six or a hundred. Uh, my daily duty was to make sure I use the App every day to check if the people are still using the box. If not, I get in touch with them to find out the problem. If they have any challenges they are experiencing we will have to fix it at the moment, you find others, other boxes are flat and then we have to exchange the box. Uh, I used to call them, if I find the patient box is flat, I'll ask the patient to come with the box if they're closer. If not, I'll ask in the next appointment to bring the box for exchange. Yeah.

I: Okay. Um, Are you familiar or with, or were you familiar with, um, something called task list?

P: Task List? Yes.

I: Okay.

P: Mm-hmm

I:And what was your experience, um, with task list? I mean, how does it work? And what can you say about it?

P: Uh, okay. Task list was there to remind us as, as staff, I can say it was there to bring more information by listing patients who needed to be followed up on.

I: Mm-hmm. Okay. Um, there are these differentiated model of care, right, which involves phone calls, SMSs, and home visit, right?

P:Yeah.

I: So, I want to know that what was your role on that, when it comes to follow up phone calls, um, home visits and SMSs.

P: Okay. Uh, okay-

I: let's start with phone calls. Yeah.

P: Yeah. With phone calls I'll call a patient and then we talk about the challenges or anything that is there at the moment. Uh, If I do not find the patient, that's when I sent SMSs and then if they do not respond to those SMSs, that's when I do home visits. Yeah, because every Friday there were people who went to site to look for the patients who are not adhering correctly or not coming to clinic.

I: Okay. So, you were also involved in doing home visits?

P:Yeah. Yeah, I did.

I: Okay. And then what has been your experience with home visits? What can you share about your experience?

P: Okay. Uh, very great. Uh, I never experienced any challenges until I came across this patient who was called xxx [patient's name] so we went there and found him at his flat. I remember it was his last month to come and collect the medication as the sixth month. He told us he knows that he has to take medication and we didn't have to come to his place then we were chased away. He said he will come to clinic whenever he feels. Yeah.

I: And that was the only time you had such an experience.

P: Yes.

I: But the rest were success, you say?

P: Yeah. Yeah.

I: Okay, you didn't have any challenges with wrong addresses, wrong phone numbers, going straight to voicemail when you're trying to reach patients.

P: Those one I did experience it mostly with, uh, homeless patients, they give you the address that firstly come in their minds. Yeah. And the cell phone number, they give you the number that belongs to their mothers you find their mothers are still home in xxxx(city name), or in xxxx (province name) and when you call them, they do not know where the person is. Yeah.

I: Okay. So, in that case then what did you normally do in order to make sure that you reached them at the end of the day? Because as you said that you want, um, patients to be cured. Oh. You know, help them if they, if they need to be counselling, counsel them. So, what, um, helped you to overcome those challenges of wrong addresses and, um, wrong, cell phone numbers.

P: Okay. So, uh, before I noted that there were motorists who went out every Friday. So, in xxx(city name) xxx [clinic name] we know these homeless people, if they're not at xxxx(name of a flat), you find they are at xxxx (location) , so we do more visits at those places, and then they know each other. Those homeless people you tell them, I'm looking for this person. They'll tell them, no, "we know where he is " and then somebody comes in to assist and you show where the person is. Then you will find them. Definitely. You'll find them. Yeah.

I: So, at the end you'll find them regardless of the wrong addresses-

P: Yeah.

I: You know knew places with homeless people.

P: Yeah.

I: Okay. Okay. So, speaking of homeless people, right?

P: Yeah.

I: While we are still talking about them. I mean, um, how many of them had cell phones and how many did not have cell phones.

P: Few had cell phones, yeah, few. But then they had cell phone, but then most cases they weren't working because they're homeless. It's a challenge for them to charge a cell phone. Yeah.

I: So, every time you need to get in touch with them you struggled.

P: Yeah. You had to struggle. I remember there was this old white guy, he was called something xxx [patient's name]. He did have a phone, but then it was always off but then whenever he gets those notifications, missed calls, he will come back to you. Yeah. That's when you'll find the person.

I: Okay. Um, with the platform, you mentioned xxxx (adherence platform name), right?

P: Yeah.

I: Um, did your patients report um, getting the SMS reminders after they've missed some dose or after they've taken their dose did, they report anything? Um, about SMS reminders that they were receiving automatically from the platform, xxxx (adherence platform name).

P: Okay. Yeah. I did have patients who were getting SMS I don't know if you are saying if they, they did get SMSs after they have missed or they get SMSs whereas they have taken the medication.

I: Uh, okay, let me put it this way. Did they report getting any SMSs at all?

P: Yeah.

I: From the App?

P:Yeah.

I: Not from you, but -

P: Okay. Yes. Yeah. The xxxx (adherence platform name) did send the messages to patients.

I: Okay. And, um, what do you know, what would those SMS normally say when they're being sent to patients?

P: I was, I was shown when SMSs it, it goes like something. "We have seen you have missed, uh, today's dose please take your medication and then if there's an error, you can report to us. Yeah.

I: Okay. Yeah. Okay. Um, please describe your role with the differentiated model of care. Um, okay. We spoke about the use of task list already. Follow up communication with patients. Um, okay. You mentioned already that you normally you would call them, those who have cell phones, you'll be able to reach them, but then for those who did not have, or who provided the wrong cell phone numbers, you would definitely have to track them down. Go to places where you can find them.

P: Yeah.

I: Okay. Um, so what is it that you did all the time between phoning them (referring to patients) and doing home visit? What did you do frequently between these two?

P: Uh, mostly I was calling. .

I: You would call them?

P: Yeah.

I: Okay-

P: remember, I would only visit if a patient does not show up, uh, let me say for two missed, uh, appointments. Yeah. I'll give them at least two months. If they have missed two months, then I know for sure I'll have to look for them.

I: Yes.

P: But then with the phone call, when you miss two doses, I know I have to call you.

I: Yeah.

P: Yeah.

I: Okay. Did you perhaps have patient who missed doses maybe for five days in a week.

P: Yeah, yeah, yeah. They do. They do, they did, uh, Sometimes when you call them some, they try to lie to you.

I:Mm-hmm.

P: "I have taken my medication and stuff, "but others will just come clean. "I have, forgotten to take the medication because I left them at home. I went to a funeral. Then I have to assist them. Yeah, I did have them.

I: Okay. If patients had to go somewhere, do you know if they took their box with them, or they were leaving it behind?

P: That's why I said some will tell you "I have taken my medication, but then I have left the box at home". Or sometimes they said " the box was at the boot of the car so whenever it rang, I didn't hear it but then definitely I did, took my medication".

I: Okay. Um, since you are working in a TB room with other healthcare workers, how were the responsibilities and duties shared, um, amongst you as the research assistant and together with the nurses?

P: Okay. Uh, with me at xxx [clinic name] it was, it was more fun because it felt as if we were doing the same thing whenever I was not available. They would call, "xxx [Intern name] we have a client, and then can I assist the patient?" And I'll say Yes. And then whenever they were not there if a person has come and need to be checked the sputum, I never denied myself opportunities. I did everything that, it gave me more experience because I would do my ASCENT work, and also, I will assist nurses to take the sputum every day. And then also I was doing blood tests to take blood pressures. Yeah, so it was nice. I never felt as if I was working for another company and then they were working for other company. We were working the same job. Remember the, the plan or the strategies is to win to be patients. Yeah.

I: Okay. And then when you first heard about the DAT or the box in your case, since you were implementing the box in your facility, um, what were your expectations before it was being implemented? When you first heard about this box, DAT what came to your mind?

P: Okay. Firstly, I was a bit, a bit shaken. I was scared because I thought I was going to recruit people outside, saying to them, come, we have the box that we need to test and stuff. But then, uh, knowing the box, uh, getting in touch with it, uh, knowing, working with it, uh, I experienced that it is more easier than I thought because at the end of the day, uh, even HIV clients were there asking for the box, and I said to them, no, I, I'm not allowed to give you the box, because it rings once. So, it's only for TB patients because they take the medications once a day. Yeah.

I: So, you had a number of patients who were asking for a box who were taking both TB and ART?

P: Even those who were not taking TB medication but only ARVs were asking for the box.

I:That's good. That's great. Okay. Um, did your opinion change then afterwards, after you started implementing now?

P: Mm-hmm. Yes. A lot. Hence, I said, uh, I was more educated and then when you see clients coming back to you and say, " xxx [Intern's name] thank you, the box was very good and we are cured from TB because of the box." Sometimes, uh, remember we as people we do forget, uh, you find we have missed more than 20 doses but this box is there to remind you every day to take a medication. You can miss if only you go for unplanned sleepovers but then when you have sleepovers that you do plan, you pick your box and go with it. Yeah.

Yeah.

I: Okay. Um, speaking of the box, um, I wish to understand, um, how did this box actually, help patients? What is it about this box that was so useful to patient, or beneficial to patient? Um, let’s say there's someone who doesn't know about this box or is their first time seeing it?

P: Mm-hmm.

I:Um, what would you tell them about test box? What it, what, what does it do actually?

P: Okay. Firstly, uh, remember with medication, we have to store them at the right place, uh, far away from sunlight, far away from temperature stuff like that. So, with the box, it's safe to put your medication inside, whether you're homeless or not. Uh, because of the heat, uh, more especially with people that are homeless medication’s  effectiveness is  affected by the sunlight mostly. So, with the box it's safe for the medication. Secondly, it's there for alarm reasons to remind you, this is the time to take medication and then it is portable, easy to carry, to carry everywhere you go, yeah.

I: Okay. Did you have complaints of patients who said maybe the box is not alarming at the time it's supposed to alarm.

P: Mm-hmm [Yes]. I did have, uh, it rang even after they have taken the medication but we had to fix those boxes because I had to get back to the xxxx (adherence platform name) to rectify the mistakes and then mostly I'll have to turn off the clock and turn it on again. You find it's an error from the box or the xxxx (adherence platform name). Yeah. .

I: So, what you're saying is that you were successful in solving these issues?

P: Yeah only if you are using your xxxx (adherence platform name), there's no way that you are not going to succeed because you'll know your work and then you'll know the xxxx (adherence platform name), more like WhatsApp. We do know. Yeah. So, it is easy to use. Only if you get into it every day frequently you'll know how it works.

I: Okay. Um, did you receive any training before you started implementing the, the box in your facility?

P: Yeah, I did. It was a five day training. They taught us about the box and the patient and importance of the box and everything that we need to know about the patients and the box. Also that the patient's attitude is going to be fluctuated it's not going to be the same every day. Yeah. And how to deal with the patient every. Yeah, we were taught that.

I: And where were you trained? Were you trained in the facility that you were working in or somewhere? Somewhere else?

P: No, I was trained in xxxx (hospital name), is it? Yeah. Xxxx (hospital name)

I: And who trained you?

P: There were study researchers.

I: Okay.

P: Yeah. Uh, study researchers, study coordinators were there.

I: Mm-hmm.

P: And the IT lady was there. Yeah. Everyone was there to make sure that we are well equipped before we go to the facilities. Yeah.

I: Okay. And what was your first impression when you started with this training? As you said it was a five day, um, training, right?

P: Yeah.

I: On, on your first day. What was your first impression? After the training?

P:Mm-hmm.

I:Yeah. What did you take-

P: first day at work?

I:After everything? Yes.

P: Yeah. First day at work was a good experience because the same day that I came to clinic, I got a patient that I needed to counsel and give the box . Yeah. That was the time I needed to show everything that I was taught about implementing on patients here. I believe I was taught well because the patient did not deny or anything of some sort, even though I did doubt my cell phone the first day but then it, it was a successful day because the patient agreed to have the box.

I: Okay. And then did you think the training was useful or sufficient, the information that was provided during the training?

P: Yes, it was because even today I do know how to talk to patients how to deal with the patient. Uh, even though they do have mood swings, I know how to react around them. So yeah, it was a lot.

I: Okay. And do you have any suggestions maybe on how, um, this training can be improved? I mean the duration of this training-

P: Mm-hmm [No]

I: Do you have any suggestion on you know, um people who should attend this training and the content that should be delivered during this training.

P: With the training, I can say  I was really satisfied I cannot say I do have anything that can be added, uh, because everything that I was taught worked very well. Uh, and then it was fruitful because not even one patient denied to take the box. .

What I can suggest is that maybe if they call us, uh, the staff members, they should request, uh, a TB nurse so that they must also have the information with them because you find when you go to the clinic, you go back with the new information and some nurses give our colleague challenges, but then with me it was very easy because we were getting along with, uh, TB nurses. So, whenever I have information, I'll call them. Even when I get home, not when I go to work, I'll call them. "Today, we have this new information, and we were asked to implement it to patients." Then whenever we go to work, it was that thing, okay, we have to implement it. "xxx [Intern's name] come and show us how do we do it." So, I believe it will work well they called our TB nurses. To train us sometime.

I: Okay. So, you are saying that, um, it'll be better next time to also, um, involve TB in this training.

I: Yeah.

P: So that they know when you go back to the facility,

I: So, it'll be easy for, for, for you guys to work?

P: Yeah, because with them, when they do go for TB training, I don't go with them all, or it was only with my facility. Whenever they go to their training with what government has planned for them, they will come and sit us down, like whoever who's working in a TB room, this is a new rule, this is what we are given, this is what we need to, to implement to patients. And then if it has to do with cleanness, we have to make sure it's clean. If we have to make sure the TB clients are made happy, then we do it all. Yeah.

I: Okay. And then do you think this five day training is enough or it should be, should take a month or one day is enough?

P: Five days was enough for that time because we came not knowing anything about the adherence, about the TB clients about the, them denying to take medication but I knew after training. It is enough.

I: Okay. Um, so besides the TB nurses. Um, who else do you think should attend these training?

P: I suggest it's best with the TB nurses because with clients, clients, I mean the patients they will disappoint you at the end of the day. You'll make sure you have called, uh, let me say, xxx [intern's name] come to where and where, or maybe let's say we have organized a transportation for you. Come wait there. Like you'll wait until forever. Some they do not show up if they heard about training and stuff. Uh, in most cases, you'll know working with patients, you know, they do not have time. They don't have time. Their time is only to come take medication and they run wherever they they have the plan to.

I: Okay. And from your perspective, right?

P: Mm-hmm.

I: Um, what can you say are the benefits of the DAT or the box.

P:Mm-hmm.

I: To patients?

P: Okay. The box works very well when it comes to adherence, medication or doses are never missed, or when missed, they do record, then the, those doses missed, they can be replaced by the TB nurses. Yeah, it's best this way. And then whenever the clients came to clinic, you'll show them your xxxx (adherence platform name) that on Monday or Tuesday, Wednesday, Thursday, Friday, you missed your, your doses and then they come to you, they say," No, I only missed three, I remember very well." But then when you show them, they'll say, "ah, yes, I remember I was so and so, so I didn't take," so it's working. It's working because with them, uh, remember it's, you don't only rely on them, you also rely on your technology to show you that this person has missed their doses unless if there's a technical error where you need to fix. But then if they have really missed and then you work together and show each other this I have missed.

I: Yeah.

P: Then it can be solved out.

I: Okay. So, do you think it does, um, improve adherence?

P: Yeah, a lot because the alarm is a privilege to them, uh, because it's a reminder. I talked about loadshedding I can say everyone must rely on the box now because even us who do not have TB have cell phones and the battery gets flat anytime.

I: Yeah. Okay. And then, um, did the DAT or the implementation of it, um, improve the relationship between you? And together with the, the other healthcare workers in the TB room between you, them and, and the patients. Yeah. It's improving your relationship between you guys in the TB room, um, um, between you guys and the patients.

P: Yeah I can say a lot because, uh, remember there are situations whereby you tell a client that they need to sign for the box and stuff. They'll tell you, yes, I do agree with what you said but then at the moment I have this and this and this challenges which they are going to, uh, disturb me from using the box or taking medication. Okay. Then I'll go back to the facility worker, the TB nurse and I'll share the story. Then we'll come together and see what's going to work for the patient, we'll need to make sure that we come together and make that person take medication as successfully.

I: Okay. Um, can you think of a patient who was using the box and was not doing so well before, before they had the box.

P: Yeah.

I: And then after they got the box, they improved the adherence.

P: Yeah.

I: Started shining. Can you think of one patient?

P: Mm. Who was this? Who was this xxx [patient's name]? I have this one patient. So, uh, he is homeless. I think he came for the third time with TB and the reason is that he did not finish taking the medication previously. We have to sit with him down to check what was  causing him not to take his whole course of medication. So, he told us that he does take the medication, but then he doesn't see a need because whenever takes his medication, he gets hungry, and where is he going to get the next meal. So, we had to sit with him down and then told him, okay, here's a solution before we give you supplements. Please make sure you take your medication for these two weeks. Then we'll have to check, uh, how well have you been? And then that person went back home and took his medication as promised and came back to clinic. Whenever he came, he came with, uh, maybe something. He has gained a lot of weight, so it was suggested that we gave him Ensure supplements, so we gave him three, ensures whenever he came for appointments. So that pushed him to, to take his medication because he knew whenever he is not taking his medication, he's not going to get those supplements. And then he was not only given ensure, but he was also given, uh, food parcel whenever there were opportunities for food parcel. Like you made sure those people are the first priorities. Yeah. Whenever there's something that they need to get, you call them, then that will obviously push them to finish taking the course of medication.

I: Okay. And then for you, um, as healthcare workers, um, how did the box help you in terms of maybe, um, dealing with the overload of work.

P:Mm-hmm.

I: And the success of contacting patients and all that? How did it help you-

P: the box the box?

I: Yes.

P: Okay. I believe when you talk about the box, we talk about the box and the xxxx (adherence platform name).

I: Yes. Together, it goes together. Yes.

P: Okay. So firstly, uh, I believe whenever the, the patient has the box at home, I can work hand in hand with them using my xxxx (adherence platform name), uh, as I have stated that I made sure that I use my xxxx (adherence platform name) every day, even on weekends when I'm at home. I need to make sure I use, uh, my cell phone, uh, when whenever a patient has missed, I call whenever a patient battery is low, I call, whenever I see maybe something that is not right at the moment, I did call the patient . When working with TB clients and you find there are a lot and let's say you have, uh, six people who are missing with different addresses, you find somebody is in xxx (city name), somebody's in xxx (area name), how you going to deal with them. So, with the xxxx (adherence platform name), it's very easy. You only contact them and then check whether is there anything wrong with their box or whether is there any challenge they're facing at the moment that is, uh, disturbing them from taking that medication. They will tell you the reason and then after that they'll will make sure and then, uh, promise you, "no, I'll use the box even now." and then after speaking to that person, you'll see a green bar .Then you see you have worked for that day and then it's up to you if, uh, on those red doted you have to make them green if the person has told them, no, I have been using, maybe the box had not been reporting to you back. Yeah.

I: Okay. Can you describe any challenges now, um, that you had with the DAT or the xxxx (adherence platform) platform? Did you have any challenges?

P: Okay. Uh, the xxxx (adherence platform name) it did not give me any challenges. Uh, it was more like any App someday it had that thing that it's not working. Yeah. A patient is here and then you need to do so and so, but then the app doesn't want to open, or the patient is here, you need to give the box and the app is not opening but then other than that, no.

I: Okay. And then speaking of that issue of the App not working when you needed to do something, how often did it happen?

P: Mm, not really much. Not really much, but then I had a technique, how to deal with that. I'll give a patient a box and then write the. It's IME number, I'll write the number down and then the time that the client suggested if eight or what, and then when the xxx (adherence platform ) starts working I need to register the monitor phone even though they're back at home.

I: Yeah. Okay. So, you were capturing this information separately or-

P: Yeah.

I: On something, or-

P: Yes.

I: Were you using-

P: But I had diary with me to record it. Yeah.

I: Okay.

P: Because if a client goes back and then you haven't given them the box they were going to take treatment for a month without the box.

I: Yeah. Okay. Um, did patients report any, um, network issues? Uh, maybe technical glitches, the box not, um, alarming at a certain time?

P: Mm-hmm.

I: Yes. Did you have, um, those cases?

P: Some told me the box has rang, even if they have taken the medication but then we did not have issues  with the network, no.

I: Yeah.

P: Yeah.

I: And did you have patients who reported that they have been experiencing some sort of a stigma because of the box that they're using?

P: No. No. Not because unless at home, but then they have not been reported them to me. They were used to the box at  the clinic because even the other departments were wishing that they had  box with them. So, everyone was friendly with the box because even when they have to collect the medication, they will come with the box on their hand, they will know that i will have to cut them and place their medication. Yeah.

I: Um, from your perspective.

P: Mm-hmm.

I: Um, do you think, um, TB treatment can be improved using the DAT (digital adherence technology) intervention.

P: Mm-hmm.

I: Whether it's the box or the labels.

P: Ah, no. Uh, myself, I have to go with the box.

I: Mm-hmm.

P: The labels, I'm not sure how do they work because I  have never worked with them before.  I believe the box is  more well equipped because as I stated, it is safe to store because with the stickers, it'll have to depend on you, where you put your medication, you find people putting their medication in plastic all day, running all along, so it is not safe for them or their medication. So, at the end of the day, it'll cause them food poisoning. You find the sun has interrupted with the pills, so with the box it's safe. Even if you have to put your box  anywhere and the pills are inside, you know, that medication is safe. And then with the alarm again, you know you are safe. There's no, there's no moment that you say, oh no, I have taken my medication five minutes before or an hour later. No.

I: Yes. Okay. Okay. And then in the absence of xxx (organisation name) or the xxx (organisation name) staff, which was supporting this intervention, right?

P: Yeah.

I: Um, What, um, what do you think, um, should be put in place to ensure the sustainability of the DAT intervention in the absence of xxx (organisation name)? What is needed, who should be, um, involved with this intervention? Yes.

P: Okay. Uh, I will say xxxx (organisation name) has, has put their foot down and showed the government that we are here to help. We are here to show you the way, uh, the government has just to come in and continue implementing this whole thing without xxx (organisation name) or anything.

I: Okay. And then in the presence of the government now taking over or, handing over to the government.

P: Yeah.

I: What needs to be in place in terms of the, the resources, the staff, and the duties the role who needs to do this and that? Yeah.

P: I believe the Department of Health are firstly starting at the facilities where we do it because that's where it started and that's where they knew the success and the good fruits that we have come with at the clinics. Uh, so I believe whenever they go to their trainings, they do talk about the staff that are there in the clinic. They do talk about the boxes. They do talk about the success. Uh, they do talk about the success percentage that it's rising up because of the boxes that came into their facilities. Yeah. They need to just report to the government because I know the government really relies on the facility workers because them, they do not know anything unless they come to the ground and the ground shows them. Yeah. Everything starts here. Okay?

I: Mm-hmm. Um, can you please tell me of any negative changes that you've seen that were associated with the differentiated model of care? The, the home visits, follow up phone calls. Were there any negative, uh, things about the differentiate model of care? Did you perhaps have patients who were complaining about getting phone calls or not wanting home visits?

P: I will say, uh, it did not happen frequently. Only few times for example, I told you, xxx [patient name] told me that, "no, I know I have to come to clinic. Uh, you didn't have to come all this way to check up on me." So those are the patient that , yeah, they do offramp.

I: Okay. Okay. You said you; you didn't have any cases reported on stigma?

P: No.

I: Okay. Okay. Um, um, yeah, we are almost done and then, um, what, um, structures do you think needs to be put in place, um, going forward to ensure the improvement and the smooth running of this intervention in, in future? I mean, who should, uh, be put in place to prepare the boxes, you know, to help with those issues? You remember you mentioned something about the xxx (adherence platform) l not opening and you were not able to do whatever that you are supposed to do at that moment. And you had to capture this on your diary. So, you had to do that on your own to try to solve those technical issues on your own. Right.

P:Yeah.

I: So, in future, who do you think should be responsible now for solving those issues of technicalities uh, and also preparing the box and stuff, because, you know, we have to prepare the box, like charging them and all that before handing them over to patients.

P: Yeah.

I: Yes.

P: Okay. Firstly, I will say, uh, with the xxxx (adherence platform) l, I do believe there are people who are taking care of the App whenever we have a technical error. Uh, because whenever we did experience technical challenges I reported to  xxx [study coordinator's name] and then it will be fixed. So it is good  because you don't have to spend days with the app not working. Yeah. Eventually it'll be fixed.

I: Okay. Alright. Oh, you said, uh, you would normally capture the details of the page. If you were not successful in registering them exactly at the moment.

P: Yeah.

I: Okay. You use your diary, right?

P: Yeah.

I: And then with these technical issues, did you normally, um, capture them somewhere also that okay. Um, this morning I was trying to, um, register patient and the App was not working, blah, blah, blah. Did you capture those, um, somewhere?

P: No. No. Unless I reported to my senior

I: Okay. And then would she come through and help?

P: Yeah. Obviously, she will come with the feedback

I: Okay. Okay. Um, last question. Um, any gaps? Do you see any gaps? Um, anything to be improved?

P: Okay.

I: Um, with the Intervention-

P: The whole thing?

I: Yeah, the whole thing-

P: ah, okay-

I: The box, the, the, the platform people who are working, you know.

P: Okay. With me, the only gap eh is  I experienced shortage of boxes.  I would run out of boxes, and I'll have to wait for a week, and then if maybe a patient had to bring a box, then I'll have to maybe make a plan to close that box under a client  who has completed the medication to give to the next person. You would  find that  the battery is sitting at 20%, but you are trying to close the gap so that this patient does not go home without the box. You even give that person a box whereas it is at 20% battery, uh, hoping that next month, whenever they come, they'll have, they'll get a hundred percent box. Whenever the patient had to go, you ask, uh, so and so can you bring the box next month when you come? So, I have to change the battery.  So yeah, this is the only challenge that I have experienced. Shortage of boxes.

I: Okay.

P: Yeah.

I: Um, but did you have a patient who had to go back home without the box? Who wanted the box, but you couldn't give?

P: Yeah. How many of boxes-

I: how many would you say?

P: Yeah, because maybe- in a day I will have to see three new clients. I can say maybe, uh, you find six new clients during that week while waiting for new stock to be delivered to me.

I: Would you give them after you received the stock.

P: Yeah.

I: When they come back for their next appointment, yes.

P: Whenever they, they're there. I do tell them. I do talk to them. Uh, Uh, I do, what's this? The questionnaire? I do talk to them and then they sign the-

I: the ICF.

P: The ICF form, yes. So, and then I explained to them, uh, unfortunately this week I'm in short of boxes, but then I will give you whenever you come, if staying far but then if they are close I would call them when the boxes arrive.

I: Yes.

P: Yeah.

I:Okay. Um, and then any other comments, um, something we didn't talk about that you wish to share, maybe-

P: Mm-hmm. [No]

I: Something I didn't touch on. Do you have anything you want to comment on?

P: Okay. Uh, patients who successfully complete treatment using the boxes come back thanking us for making efforts for them to finish treatment. So, I believe even us, the ASCENT staff should have something that we have to give for to thank them. Like, uh, even if it's not big, but then maybe a pen or something. Something that you have been working with us well and then you have been faithful to us to return the box to us, so here is something to say thank you to you. You have worked well with us. Yeah.

I: Oh, okay. Something to give to patients.

P: Yeah.

I: Just to encourage them.

P: Yes.

I: Okay. Okay. That would be nice actually.

P: Yeah.

I: Okay. Um, no, we have come to an end of our session. Thank you so much.

P: Okay.

I: Your time and for agreeing to meet with us. Thank you. Thank you. Um, the time is now 12:00 PM.
