# Supplementary material for: Acceptability of Digital Adherence Technologies to support people with drug-susceptible TB in South Africa
Source: PLoS One. 2025 Sep 24;20(9):e0332103. doi: 10.1371/journal.pone.0332103 (PMC12459780; doi:10.1371/journal.pone.0332103)
Supplement: S4 File — (ZIP) [file pone.0332103.s004.zip › S4 Transcripts/HCWs and Stakeholders/IDI 14-HCW.docx]

**TRANSCRIPTION NOTATIONS**

| **Label Key** | **Meaning** |
| --- | --- |
| **I** | Start of each new utterance by the Interviewer |
| **P** | Start of each new utterance by the Participant |
| **N** | Note taker |
| **{ }** | Indicates that details were changed or pseudonyms were used to anonymise data |
| **( )** | Indicates the description provided to anonymise data |
| **XXX** | Words were omitted to anonymise data |
| **-** | Breaking into a sentence by the next speaker |
| **…** | Pause or drawn out words |
| **[ ]** | Indicates noise made, e.g. [laugh], [sigh], [pause] |
| ? | Beginning of utterance by unidentified speaker or questionable text |
| **[inaudible segment]** | Unclear section of the recording |

I: So, do we have permission to record you?

P: Yes, you do.

I: Date of the in-depth interview: xxxxx (interview date). Location XXX [name of clinic], language used for the session is: English. The PID of the participant is xxxx. The time at which the session started is: 13:04. The interviewer is: XXX [Name of interviewer]. Thank you very much for agreeing to participate in the study.

I: So, can you tell me what’s the title of your current position?

P: I’m a clinician, working at the TB focal.

I: You’re a clinician, working at the TB?

P: Focal.

I: Okay.

I: So, how long have you held this position?

P: Which position? As a clinician? Wow

I: Yes.

P: Since xxxx (year).

I: Okay. So, how long have you been working in the TB room?

P: Since xxx (year)

I: Since xxxx (year). So, what are your roles and responsibilities, when it comes to TB care?

P: Diagnosing patients and giving them treatment.

I: Okay. What else do you do besides diagnosing and giving treatment? How else do you support these patients?

P: Adherence counselling. That’s the major part [inaudible segment].

I: Okay, huh. So, how are TB services delivered at your level? Are you working at a facility, district, or provincial level?

P: Facility level.

I: Okay. So, now I want to know what you know about ASCENT right. If you were to explain to another health care worker, what digital adherence technology is? What would you tell them?

P: Wow [Laugh] Okay I’d say ASCENT is supplying us with these beautiful digital boxes which help our patient’s with adherence. And this box is very nice, it reminds our patients when to take their medication.

I: How does it remind patients how to take medication?

P: It has an alarm which is set according to the patient’s desired time, so when the time comes, it will beep and then the patient will know that it is medication time.

I: Okay.

P: Mmm

I: So, you have told us what digital adherence technology is from a patient’s perspective. So, what are you using as the TB nurse?

P: Okay, I’m using it to monitor if patients are taking their medication.

I: How are you monitoring them? …… How are you monitoring these TB patients?

P: Okay, this smart box is linked to the device and then when the patient didn't take their medication, I'm able to see that they didn't take their medication.

I: So, how do you see on this platform that they've taken the medication, or they've not taken their medication?

P: If they didn't take their medication, the colour coding will reflect as reddish and if they. If they took medication at the exact time, it will be dark green.

I: So, besides the alarm, how else are the patient's reminded to take medication with the use of this technology?

P: Okay, because this system shows if the patient didn't take, so they'll be reminded via the SMSs and through a call.

I: Okay, what does the SMS say? Do you know?

P: I’ve never received that SMS [Laugh]

I: Okay, alright, and then huh, you said that there’s also a call.

P: Mmm.

I: Huh, who makes those phone calls?

P: I think the ASCENT people because I'm not the one who does that. and even our supporters do call the patients.

I: So, besides phone calls how else do you follow up on a patient who has missed medication?

P: They do home visits.

I: Can you tell me more. Who is responsible for the home visits?

P: Our WBOT (Ward Based Community Health Worker Outreach team) team and the ASCENT huh, support team members

I: Okay, so what is your role in these home visits?

P: Okay, my role in the home visit is to let them know where they can find the patient and tell them when we are missing a patient. Then, I let them know and then they go and visit the patient.

I: Okay, do they give you feedback?

P: Yes, definitely.

I: Alright. So, what are some of the reasons that they've given? Why patients missed doses?

P: No, you know what if patient misses, they don't even have valid reasons. Some will be saying, “I went away and forgot my pill box,” and then sometimes the patient comes to report and say they didn't miss their medication. I think maybe it was due to some technical errors from the box but otherwise it's just few patients of course not all maybe 1 out of 50 and only 1 reported that he went away, forgot, and didn’t take the box with him.

I: Okay, so what are the reasons why he left the box behind when he travelled?

P: [Laugh] He was going to see his girlfriend. Then, they are saying the box is a little bit bigger, and then sometimes I'll say it raises eyebrows to the other person who’ll be asking what that is. You know people are so curious. Ya (yes)

I: And what's wrong when people asking?

P: Because the one who said that, maybe he didn't want his girlfriend to know that his taking the TB treatment.

I: Okay, alright. So, he had not disclosed to the girlfriend? Do you know possible reasons for non-disclosure?

P: I think huh, maybe the fear of being discriminated or being rejected.

I: Okay, alright, and how do you handle this huh, non- disclosure issue when you initiate patient's on treatment?

P: When we initiate patients on treatment, we-we encourage them to disclose, because all the family members who are staying with the patient need to be screened and tested.

I: Okay, alright. So, now huh, back to the phone call. So, what's your role when it comes to making phone calls? You mentioned that they are supporters who make those phone calls who are these supporters?

P: The ASCENT supporters.

I: Okay. So, how often are these phone calls made?

P: Regularly depending on what can I say, depending on the results they are getting from the gadget.

I: Okay.

P: If there are no defaulters but every day we check the system, if there are defaulters, we remind them.

I: And you mentioned that regularly depending on what the platform is reporting-

P: Let me just say daily.

I: Daily so?

P: Yes.

I: Okay, and once the patient is brought to the facility after either a phone call or a home visit, what do you do with this patient?

P: Adherence counselling, adherence counselling and adherence counselling.

I: Okay. So, which support method or follow up method do you use mostly? Is it the phone calls, the home visits or huh, counselling? What do you do more frequently?

P: We do the counselling but then the other ones depend on the response of the patient remember, every time they come to the facility, we do adherence counselling and if they don't respond, then we'll do a phone call. If they don't respond to the phone call, it means a home visit. So, every time they’re here, they'll be adherence counselling.

I: Okay. So, when you first heard about the digital adherence technology, right, the box and the system you're using to monitor, what were your expectations before it was implemented at the clinic?

P: Before it was implemented, I was curious. I wanted to find out about this box.

I: Did you think it was going to make your work easier?

P: [Laugh] Yeah

I: How so?

P: Because you know it saves a lot of time for us going out there every day checking patients, yeah. So, now I'm monitoring patients here at work, While I'm busy doing other duties.

I: Okay.

P: Mmm.

I: Alright. Did you think it was going to be complex to use or easy to use the first time you got to know about it?

P: Obvious first thing you ask is “how am I going to use it"? Ya (yes) but eventually we found that it is easy and simple as 123.

I: Okay, so it means your opinion is changed after you started using it?

P: *Yebo (Yes)* [laugh]

I: [Laugh]

I: Okay, so let's talk about the training and resources you received on huh, this digital adherence technology.

P: Mmm

I: How-how were you trained? Who trained you?

P: The ASCENT people.

I: Okay. Where were you trained?

P: XXX [Name of hotel] somewhere in town.

I: Okay. So, you attended the mass training?

P: Yes- yes

I: Okay. So, do you remember the training activities? That happened there.

P: Yeah.

I: What was covered?

P: [Laugh] the whole thing. how does the box work? How do we monitor the box? that’s it.

I: Okay. So do you think the training was comprehensive?

P: Yes, I think the training was comprehensive. Although I will emphasize that even though we had the mass training, at least the on-site training should also be offered.

I: Okay. How often should this on-site training be offered?

P: It depends on-on how fast someone can grab the whole thing. Remember when you're there, it’s a once off thing and then when you come here, some of the things you forgot, and you’ll keep on phoning and asking how we go about this but I think the on-site training should be done as a follow up of the mass training.

I: Okay. So, who do you think should be doing these follow up trainings, the on -site trainings

P: The people who offered the mass training [laugh]

I: Alright. So, what are your other suggestions to improve training? You have mentioned that continuous on-site training what will be the suggested duration for this trainings?

P: The training will only depend on how quick do I or someone grabs the whole thing, how does someone tend to adjust to the system?

I: Okay. So, going forward, who do you think should attend these trainings?

P: I think this one will be for all nurses, all healthcare practitioners. The nurses, doctors and the WBOT (Ward-Based Primary Health Care Outreach Team). All healthcare practitioners or those who are dealing with patients.

I: Okay. Why do you think it all healthcare practitioners?

P: Because from where I’m sitting. I think this is a very useful huh, equipment to use.

I: Mmm

P: So, I think if all of us can be skilled about this, not only the TB nurses, remember I’m not working there alone, and I'm not going to be there forever. So, whoever comes in, should be knowledgeable about the whole system.

I: So, why wouldn't you be there for-for forever? What are the reasons why?

P: Sometimes I’ll retire, sometimes I'm not here, I'm sick. A person who will step in must be able to know how to monitor the whole system.

I: Okay, can you tell me about staff rotations going on?

P: Yes, also the staff rotation. we are doing that. So, if I know this then the next person must also know how to use this.

I: Okay, so how often does this staff rotation happen in the TB room?

P: Huh, yearly.

I: Yearly?

P: Yeah

I: So, how do you suggest the next nurse must be trained? Let's say you are here for a year and then you're exiting. Who should be training huh this nurse taking over?

P: The next the nurse who is handing over should do the training.

I: So, you should be equipped enough to be able to train the next one?

P: *Ya (Yes)* to do a proper handover.

I: Okay. Interesting. So, from your perspective as a TB nurse? Can you tell me the benefits of the differentiated care? Which are the follow up actions we do when a patient misses the dose, like your SMS, your phone calls, and your home visits, what are the benefits of those?

P: The challenges are there. remember we are not safe out there. You don't know what's going to happen and then they don't want to open for you. When you get there. They ask, “nurse why you are here”? You understand such things? As I have indicated some didn't even disclose, some are tenants. Then you are now raising eyebrows to people who will ask “why are you here”? So, I think it's not it's not easy for someone to go and do home visit. The transport challenges, and safety issues

I: Are you the one who does the home visits?

P: Sometimes, not always.

I: Ok. So, what's been your experience when you do the home visits?

P: The challenges are there. remember we are not safe out there. You don't know what's going to happen and then they don't want to open for you. When you get there they ask, “nurse why you are here”? You understand such things? As I have indicated some didn't even disclose, some are tenants. Then you are now raising eyebrows to people who will ask “why are you here”? So, I think it's not it's not easy for someone to go and do home visit. The transport challenges, and safety issues

I: Can you elaborate more on the transport challenges?

P: I'm using my own transport. If I don’t have a car, how am I going to get there? I need to walk around and it's not safe out there.

I: So, what are the other challenges with home visits? Besides the transport issues, the issue of it not being accepted because of non- disclosure? Do you always find the patients on the addresses they give you?

P: No, [Laugh].

I: [Laugh] what are the reasons?

P: People are moving around you get into a household, and they say he was staying around and now he is no longer staying here. He stays at XXX [patient’s location] He visits here and like I said its difficult people are moving around.

I: Okay.

P: Mmm

I: And huh, what are the challenges with phone calls?

P: Giving the wrong phone numbers, network system is now common, you find that there is load shedding. We are having the network challenges *ya (yes)*. But in case you don't have the electricity challenge, or the patient gives you the correct numbers. I think the phone calls would do best.

I: Okay.

P: Yeah.

I: Why do you say the phone call would do best?

P: Because you can reach the patient quickly and easy, unlike the SMS you're not even sure whether the patient read the SMS or saw the SMS, you just sending an SMS you're not sure whether the patient saw it, but with a phone call you are sure because you're talking to the person on the other side.

I: So, you mentioned that huh, some patients provide wrong numbers. What are some of the reasons why they do that?

P: They don't want to be called. Sometimes you find that they gave the correct numbers and then they change them along the way. You see their phones maybe got lost and then sometimes their phones get broken. So, when you call the patient, the number is no longer working.

I: So, in terms of cell phones. Do all patients have cell phones?

P: Yeah, so far. The ones that I know do have. Those who don't have, will provide they’re next of kin’s numbers.

I: Okay, so what's the experience of you contacting the next of kin? Is it- is it easy?

P: Easy, not easy. The easiest part of it is that you can find the patient, but the difficult part of it is that you find sometime saying I'm not at home, I’m on duty or the patient is no longer staying with us understand. So, that's the challenging part of it.

I: So, how do you think this problem of patients providing the wrong numbers can be resolved?

P: Mmm. I don’t know [laugh] clueless.

I: Clueless okay. So, in terms of home visits, do you always have the WBOT or support staff to go to the houses to do a home visit after four days?

P: It depends, not always because remember, they're also having other things to do. We are not having enough WBOT members to visit. So, if the WBOT team member who is working at the facility is sick or is at training then I'm alone mmm.

I: Alright. So, can you let me know the benefits now of the technology? We've touched on the benefits of following up. What are the benefits of the technology itself, the box, and the platform?

P: In what way?

I: How does the box benefit the patient and how does the system benefit you as the health care worker?

P: Ok. I'm- I’m not going to talk on behalf of the other patient. I don't know how the box benefit the patient [laugh]

I: Have they reported to you?

P: But from what I've heard from the report they gave me. It really helps them to keep time. Yeah, it really helps them not to miss their pills. Yeah, but then from my side with the good adherence from the help of the box. I see a lot of improvement in TB being cured.

I: Okay.

P: Yes.

I: Alright Interesting. So, how were you monitoring patients before the system came up?

P: Before the system came up, every week we pull the file, check the register, appointment register, whether they’ve came or not and then we do the follow-up.

I: Okay. So, can you compare the two systems?

P: [Laugh] they're not comparable. The digital one is good because the register is taking a lot of time [laugh]. There is a lot of writing but this one is smart. It makes our life easier and our work to be enjoyable.

I: So, has this technology improved the relationship you have with patients?

P: Yeah.

I: How so?

P: *Yoh* [Laugh] in a sense that it’s easy to monitor them, it’s easy for them to report to us. Even patients who are not TB patients, are interested in the box. they want to have the box for themselves.

I: That’s good.

P: Mmm

I: So, do you have patients who are taking other medication besides the TB medication?

P: Yes. Those who are taking the ARV’s. We are having other chronic patients like hypertension, diabetes. Yeah.

I: So, what are your perceptions on them using this box?

P: They are feeling good about the box and unfortunately, after the TB treatment, they are returning the boxes back and then you feel like they don't want to give the box. They really enjoying having the box with them because its helpful for other medication.

I: Okay. So, what do you think should happen going forward?

P: I would really love to see other chronic patients getting the box.  I don't know how, but maybe if possible just get some portable boxes so that they can carry the box along wherever they are going. but I think it will be really much appreciated if all the chronic patients can get this box it’s a really a good tool.

I: That's good to hear. So, can you tell me the challenges of the technology now the box and the system you have experienced so far?

P: The box doesn't have much challenges. The only challenge is that it’s big. Some patients cannot move around with the box. Yeah, but the system power failure [laugh]

I: Okay.

P: Yeah, but other than that no, you don't have much challenges.

I: So, how is power failure a challenge ?

P: Like I have indicated that if you are having a power failure we cannot reach our patients. but then the box is doing well.

I: Okay.

P: And you know what I like about this box, it doesn't even require the intellectual capability of a patient. Even if you are illiterate, you can use the box.

I: Okay, interesting. So how else does the power failure affect the patients? you mentioned not being able to reach the patients. But do you think it affects the system where you monitor the patients. Have you experienced challenges with the system caused by power failure or network issues? [Baby crying]

P: The only challenge is that you cannot reach them, but other than that no

I: So-

P: if our things are fully charged. We are ok.

I: Okay, okay and then do you have issues with acceptability. Are there patients who refuse to take the box?

P: No, [laugh].

I: They’ve all accepted?

P: All accepted, and all interested

I: Okay. Do you have huh, a certain group of people who are rather maybe difficult to support using the technology for example, homeless or drug users?

P: No, because we are having some who are homeless, they carry the box and bring the box to the clinic every time.

I: Wow. Can you tell me more about your homeless patients?

P: Ya (yes) the homeless patients. You know you’ll be surprised. They lose their things when they are out there but they always bring the box to the clinic. I don't know where do they hide it? [Laugh] but yoh they're bringing the boxes to the clinic.

I: Okay.

P: Although it’s difficult for us, you know, to treat them because they don't have phone numbers. Remember, they don't have cell phones. So, when they are missing their doses it’s difficult for us to trace them but when they decide to come to the clinic, they are bringing the box.

I: Nice and interesting, are there issues of stigma that have been reported in relation to the box as a challenge?

P: No stigma has been reported because everybody is interested in the box, all of them, they want to have the box, so they didn't label it that bad. So, even those who have the box are bragging about it [laugh]

I: That's nice. They are bragging.

P: [Laugh] *ya (yes) they* have the box; they are bragging about it.

I: [Laugh] that’s nice. So, from your perspective as a TB nurse do you think TB treatment can be improved using this technology?

P: It has improved,

I: How so?

P: it can be improved. It has improved.

I: Yes.

P: We don't have a lot of defaulters anymore.

I: Okay, and huh, in terms of your workload, has it changed?

P: Not really.

I: When it comes to the technology, the use of the technology? You mentioned how you used to do things how you used to monitor patients before has that changed your workload if you're using the digital technology?

P: Yeah, in that way, yes it has changed, because we are doing away with a lot of registers and now everything is at the fingertip you just press, and the information is there.

I: Okay, alright. And then in terms of patients adherence. How were you monitoring it before? How did you know if a patient is adherent or not, before you were using the system?

P: Yoh by their cards and you remember some they even forget to bring their cards. So, they come you are not even sure if they’ve taken, they’re pills maybe they just took them out today because they are coming to the clinic [inaudible segment]. So, those are the challenges, but huh, we were using the cards to monitor if they’ve taken their pills at home.

I: Okay. So, were you recording the information on the card somewhere?

P: Yes, in they’re files.

I: In they’re file. So, if a patient comes with a ticked card you report/ document that it’s you who ticked?

P: Mmm.

I: Okay. So, besides the file. Were you doing it electronically by any chance?

P: No.

I: Okay.

P: We are doing it on the files and their cards.

I: Only?

P: Mmm

I: Okay, alright.

I: Can you tell me about the positive changes that have been brought by this technology and how they can be sustained in the absence of xxxx (organisation name)? Let’s say we handed over to the Department of Health, you as TB nurses are working on it on your own, or the Department of Health Staff are doing it on their own.

P: *Yoh* huh, I don’t know how that can be maintained, because I don’t know the financial status of the state but from my side I will love the box to continue I really wish the Department can adapt that and move on with it.

I: Okay, and then are there negative changes you can think of that have been brought by this follow-ups we do and huh, the box itself. Are you concerned that a patient could open and close the box without taking medication?

P: Cheaters will forever be there at the end of the day, it’s still up to an individual, you understand but I think this box is really making it work you know, patients know that they are being watched on the other side, they’re taking their treatment. So maybe if you can install the camera [laugh] in the box so that I am able to see the patient open take medication and close. I don’t know how.

I: Okay.

P: But remember as long as I’m not with the patient on the other side. I’ll never know if the patient opened and closed. Do you understand?

I: Mmm.

P: So yeah, but then I think they’re taking, because as I’ve indicated there is a very good progress. So, if they are cheating, they are cheating themselves but I don’t think their cheating [laugh]I: So, how do you think these cheaters can be addressed? How can this be addressed?

P: I don’t know. But as I’ve indicated maybe if you can install a camera.

I: A camera

P: Yeah, on the box, or maybe if- if maybe the box can be timed somehow so that you are able to see the duration of the box being opened.

I: Okay.

P: *Ya (Yes)* to avoid the opening and closing.

I: Mmm.

P: *Ya (Yes)* maybe if they can be somehow timed

I: And in terms of counselling?

P: The counselling?

I: Part, yes

P: The counselling continues,

I: Yes

P: Every time.

I: Okay. And huh, in terms of patients not answering their phones or giving wrong numbers. How can these be addressed?

P: That is difficult for me, because if the patient is not answering their phones, you don't know what is happening on the other side, maybe they're not taking their phones with them. Maybe there's a network problem on the other side, you don’t know.

I: Okay. So if this intervention, the box, and the system intervention is to be integrated to the existing TB program we've always had. what is needed for you to continue?

P: The boxes and I think more support members because remember when you enrol a patient on the system and giving the whole information it’s a bit time consuming.

I: So, who can the support members be?

P: Anybody who can support the program [laugh]

I: Who do you think the department can provide to support. Do you think it can be someone already within that facility or it’s someone new?

P: I’m not going to say someone already in the facility or someone new, it will depend upon the environment, you understand. When you say someone already in the facility who’s going to be there because all those who are here have their own duties to take care of you understand. I think they should be a designated person. to say work with this or to help us with the whole thing.

I: Okay.

P: I don’t know whether from inside or outside, because immediately that person comes in, he/she will be inside.

I: Is it not possible to identify someone already inside to assist?

P: [Laugh] Who because as I’ve indicated, all those who are here have their own duties and responsibility, we cannot take a clerk to come and do this, you cannot take a general worker to come and do this they are busy cleaning. You understand what I'm saying? All those who are here in the facility have duties delegated to. So, there should also be a delegated somebody to come and do the whole thing.

I: Okay.

P: Yes, so that you know when you're doing the daily delegation today, you’ll be doing the registrations.

I: So, who do you think should be responsible for preparing the boxes before they are issued to the patient things like charging the box?

P: *Yoh,* I don’t know.

I: Okay.

P: I think if there is a person who's delegated to do the registrations and staff will do the charging to.

I: Okay, and if you encounter challenges, let's say there's a problem with the box or the problem with system, the platform you use to monitor patients. How do you think that should be handled when it's now with you as the Department of Health and xxxx (organisation name) is out?

P: I think when the department has taken over, they'll be some channels in place to say if the boxes are giving you the challenges you must report to this person like now if we are having challenges with the computers we know where to go. Yeah, we know who to call. So, even with the boxes I think there’ll be some challenges and channels to follow when we are experiencing some problems.

I: Okay. So, you mentioned that you need boxes, right? What are other resources that you think can be needed to continue with this intervention?

P: If the boxes are available. I think that all the gadgets that accompany the box must also be available. The phones should be operational at all times.

I: Yes.

P: *Ya* (yes) the boxes should be available and let me just say it must have enough stock so that I don’t find myself admitting a patient today huh, and not having a box and others do have. Along the way we don’t have, and the project will collapse.

I: Okay.

P: Yes

I: That’s true. And huh, in terms of the gadgets to monitor, what would be needed?

P: The gadget itself [laugh] how am I going to monitor the box if I don't have the monitoring gadget?

I: Yes. Do you mean a tablet, or you’d prefer a desktop or? What would be the best?

P: I think the desktop or both of them. Because with tablet I can monitor the patient wherever I am and then the desktop is there in my office, you understand, then I can use it when I'm in the office. And the tablets huh, I can say they are very much better than the desktop in the sense that I can charge them and then I can use them when there is no power. But unfortunately, the desktop I can’t use when there is no power.

I: Okay.

P: But the tablets can be used all the way.

I: That’s true, that’s interesting. So, huh, when you have a challenge with the box or the technology, how are you currently recording those challenges? Or those successes you're encountering? As you are using the box? Do you have anywhere where you are recording?

P: No, not really

I: Okay.

P: We are writing in the patient’s files

I: In the patient’s files

P: We are just recording that they are taking their pills and the adherence is good.

I: Yeah, so do you have any gaps you think are the in the way that intervention is currently being delivered?

P: Not really.

I: There are no gaps, or do you have any suggestions to improve how things are currently being done? So, you’ve talked about the size of the box.

P: Mmm. Currently with how we have implemented this. I think we are doing well.

P: Yeah. Like I have indicated maybe the size of the box, but it’s not a big challenge per say. because people can still accept it the way it is, maybe when they're travelling but those who were taking the box from the clinic to their households are fine with it.

I: And in terms of patients with multiple morbidities. How can they also be supported with this?

P: Yoh. I don’t know because we are not going to give a bigger box [laugh] to fill in all their medication, but I think if you are having a big box and smaller nyana (little bit) one they can do the refill.

I: Okay. So, maybe patients can receive more than one box?

P: Yes so they can be supported.

P: Mmm

I: So, they can be supported. What are your final comments about the whole intervention the box?

P: [Clearing throat]

I: The system you are using to monitor, the phone calls, the home visit’s. What are your final comments about everything?

P: Huh, my final comment is that can we please to have the box going on and huh, I think some of the obstacles/challenges we will overcome as we move on, but huh may the project please proceed.

I: Okay. Thank you very much Sr. huh we have reached the end of our interview. The time is: 13:48.

GLOSSARY:

Ya (Yes)

Yebo (Yes)

Nyana (Little bit).
