# Supplementary material for: Acceptability of Digital Adherence Technologies to support people with drug-susceptible TB in South Africa
Source: PLoS One. 2025 Sep 24;20(9):e0332103. doi: 10.1371/journal.pone.0332103 (PMC12459780; doi:10.1371/journal.pone.0332103)
Supplement: S4 File — (ZIP) [file pone.0332103.s004.zip › S4 Transcripts/HCWs and Stakeholders/IDI 15-HCW.docx]

**TRANSCRIPTION NOTATIONS**

| **Label Key** | **Meaning** |
| --- | --- |
| **I** | Start of each new utterance by the Interviewer |
| **P** | Start of each new utterance by the Participant |
| **N** | Note taker |
| **{ }** | Indicates that details were changed or pseudonyms were used to anonymise data |
| **( )** | Indicates the description provided to anonymise data |
| **XXX** | Words were omitted to anonymise data |
| **-** | Breaking into a sentence by the next speaker |
| **…** | Pause or drawn out words |
| **[ ]** | Indicates noise made, e.g. [laugh], [sigh], [pause] |
| ? | Beginning of utterance by unidentified speaker or questionable text |
| **[inaudible segment]** | Unclear section of the recording |

I: Huh, thank you so much for agreeing to participate in our interview today. Can you please allow us to record the interview?

P: Yes, you can audio record.

I: Okay. PID number is: xxxx. Date of the interview: xxxx (interview date). Location: XXX [clinic name] clinic xxx (district name) in xxxx (province name). Type of the interview: healthcare worker. Facilitator: [interviewer’s name]. Time of the interview is: 12:16. Alright thank you so much sister (nurse). Huh, can you please tell me what your current position at this clinic is?

P: Huh, my position at xxx [clinic name] clinic, I'm a professional nurse and I'm also a- I’m a TB focal person, also monitoring the intern who was doing ASCENT. Yeah.

I: Huh, okay. So, in relation to TB, what exactly are you doing?

P: In relation to TB? We check patients. We initiate patients on TB. And then also we monitor those patients for six months, after monitoring, we also check that they do not default on TB.

I: Okay.

P: Yes.

I: Okay, how is the defaulter rate in the facility as you're saying you're also checking patients that they mustn’t default. How is the defaulter rate in the clinic?

P: Now?

I: Mmm (yes).

P: Huh, after- I think let me start- first we had a large number of patients defaulting but after we had training and ASCENT was introduced to us to give patients the boxes. I think our defaulters have now reduced now.

I: Okay. What is contributing to the defaulter rate going down?

P: I think now- it's only because we can monitor patients now. We can see which ones are not drinking their medications and which ones are not coming to the clinic because we also have an intern again, it's not like we are working alone as professional nurses.

I: Mmm.

P: You have a health worker, and intern and a professional worker, there are people who are helping you to monitor that patient. So, now as compared to before- at first, we couldn’t see anything. A patient will just come and take tablets, after taking tablets, they won't drink them or come back to the hospital. You will only see maybe a data capture telling you that this patient hasn't been coming.

I: Okay.

P: So, now even though you don't know where the person stays, whether the current address she or he gave you is the one right or not you put the patient on treatment, then you can monitor the patient with a phone, seeing whether they will drink the medication or not. After that you will know whether the patient is defaulting and you won't even leave the patient for long because if the patient doesn't open the box for one day we see it and we can follow up on them

I: Okay, and before, how were you monitoring adherence before the smart pill box came? How?

P: Before?

I: Mmm (yes).

P: It was only with the help of data captures, they will take you to this system, after this system will tell you if only that patient was coming for monthly medication or not. Besides that, there was nothing.

I: Okay, alright. That's good to hear that. So, when it comes to patient care and counselling, what are your responsibilities?

P: Mmm coming to patient care and counselling, we do counsel patients who are on TB a lot. You start by telling them that TB is not a lifelong- a lifelong thing, it will go away, it only take six months.

I: Mmm.

P: So, you counsel them to take their medication every day. Not to- to default on their treatment.

I: Okay, all right. How are TB services delivered at your level with regards to digital adherence intervention? In this facility, what exactly are you doing with regards to DAT?

P: With a new system. I can say the new system.

I: Yes, it’s DAT.

P: I as a professional nurse, I only monitor what the intern will do. You monitor if the patient defaults for a day, then the intern has to come and say sister so and so patient is defaulting or maybe the patient has a difficulty of opening the thing. So, after that, you have to phone the patient. Or I'll go and tell the health worker to go and check that patient if he or she has a hassle on opening or whether she has forgotten that you took those tablets, she has to open.

I: Okay. What else are you doing in the ASCENT program as a nurse?

P: Huh, we have to initiate that the patient must take the box. You have to counsel them on that the box is better. It will remind them to take their medication, they will drink it or timely every day. They won't forget it. When stopping, they have to take that thing back to me as a professional nurse, I have to initiate what the intern has told the patient so that if the patient doesn't believe the person or you refuses this, so I have to initiate a lot better so that the patient will not refuse the box.

I: Okay. So, tell me about your experience. You've done that, you’ve explained to the patient about the box, how was the acceptance of the box from the patient after you have explained to them?

P: Okay. I have many patients that we have initiated on, on- on- on the box *ne (right)*-

I: Mmm.

P: Maybe let me start firstly- when it was new, the intern just explained to them that this box will help you, it will remind you of the time that you take your medication and mostly they thought that this thing maybe it's a monitor or an alarm or something which will tell where you stay, maybe it's a scam or something but then as a sister, you have to come in and tell them “you know this box will help you for ,especially those that I don’t see often.” I like telling them you know, because this thing is a project. It will help other patients- let's say we have a patient that has TB, but then he's blind. It will help that patient a lot because it will remind you that when it beeps then you have to take your medication. So, if the patient can’t see, he will only open and then take the medication, you see, it does help the other person who is vulnerable to something. So, they will just have to accept it because other patients take time to- to accept something from a new person than a person who's always been there and they know the person who's been on that field every day, like I’m a sister, we see you every day. So, if you tell them I'm introducing something new, then they just accept it, and say yes sister I’m going to take it.

I: Okay, it’s good to hear all of that and then what else are you doing in the- in the project? You told me interestingly so that you will make sure that you initiate the box that you register the patient and also explain further to the patient. How is the box going to help them- what else are you doing just in the program? Anything else that you're doing in the program?

P: Huh, the other thing we are doing on the program-we did huh… we also conduct campaigns.

I: Okay.

P: Yes, we have to conduct campaigns so that many of our communities learn about this thing that we are initiating, if we are introducing something new. I think for us if we conduct campaigns, then campaigns make it easier for patients to know of something that is new because a with a campaign there is going to be a lot of people trying to come in hear what is happening. You will be introducing something new to them. So, they'll go and tell neighbours and neighbours will tell others that there's something new, so that when the patient comes in and you initiate that new thing, he won’t be surprised.

I: Alright good to hear that [Phone ringing] [pause]. Can you please describe your role with a differentiated model of care in the program? What is your role in the differentiated model of care? So, differentiated care is the follow up of patients. The SMS, the calling, the home visit, what are you doing on those activities? Which one are you mostly assisting with?

P: Huh on my side, I would say that the one that I'm assisting with is- my intern will make calls, because he has a phone and home visits I’ll just conduct or maybe tell the community health care worker to go and do, but there are other home visits that they won’t do because it it will be difficult for them to get in the yard. So, sometimes I go with them. Sometimes if a patient sees that it a sister (nurse), and she will open, but they don't open for other community health workers because they say that health workers come every day into our yards.

I: So, it's different if it's a nurse going there?

P: Yeah, because even other weekends we can go you know,-even other weekends you get patients there and they will listen to you but if I say a Community Health Worker should go and see if there's a patient in XXX (Location) somewhere, that patient will just tell you that the gate is locked and will not open.

I: Okay, so when you sometimes do these home visits and check on the patient on why are they not taking medication? What is it that you identify as a challenge leading to non-adherence?

P: But then it's not as if she was drinking only TB. Yeah, but also other medication. That's why I'm saying other patients are not well informed actually. They're coming out somewhere else and there they not doing the same as us, until you go to the patient and tell the patient to take out everything that we gave them. They would take out from the wardrobe and you tell them to stop this and this, drink what our intern gave you, drink only that. So we do find them, but it's not patients that are from our XXX (Location) but its some of those coming in from other clinics who are not well educated of other things, or we don't do things the same. We don't do things the same at all

I: So, what is the other major problem that you are noticing is leading to non-adherence?

P: With regards to the boxes, or the old?

I: Both? Even if they've got the box, what are you identifying as a major problem?

P: I think some other patients, they're still stigmatized over TB, because now akere (isn’t it) we have boxes. There are other patients that will tell you that sister you know this box will show that I have TB maybe the battery's low, you ask them to bring it for recharging and others will say people will notice if I come up with the box. People will notice that I'm here for what, you see.

I: Okay.

P: But it's better than previously, because previously none of this would be encountered, like they will come no matter what's happened, but the patient has to come to the clinic. *Akere (isn’t it)* the battery will be low or the intern will tell the patient that it will be better this time. It will light like this, It will do what and so if the patient doesn't notice all of that, he has to come in ask you how you said this, and this is- from the previously - on the previous one. The patient wouldn’t come, you only give a date and they come at this date and this date. So, even if he misses that date, there's nothing that will tell you if the patient did come or didn’t come.

I: Alright, okay, so when you first heard about these digital adherence technologies, what were your expectations? What are you thinking about before they came and implement? What were you thinking about these boxes?

P: Huh, For me,

I: Mmm (yes).

P: firstly, when I heard before seeing the box- before seeing that box-

I: Mmm (yes).

P: You know when you're told you're going to train for something, then you will just memorize. I just thought it’s going to be something difficult, patients in XXX (location) will find it difficult to use. What about the old one and mostly here I don't think that they are well educated, maybe I thought its digital technology. So, if there are other patients who can’t use phones, so how is this going to be for them to use the box?

I: So, were your expectations changed?

P: Yes, a lot- a lot (Laugh) because when we met and trained on the box, it was a lot easier. You could see you were told, even on whether which colour to see, it would light green, and if the battery is low it will light like this. So, there are colours that tell you how this one is differentiated from this. They're not the same. So, a patient has to ask themselves even if they're not well informed, but regarding the colours, there are different colours, oh, this one is green, but now it's yellow. So even with us when you put the patient on -on the phone, yes on the register [platform] if the patient defaults for a day, there's a colour. If the patient defaults for two days, there's some colour, but if a patient takes medication for the whole- does not default at all, then there’s a colour to see that patient does well on her medication.

I: Okay, so it was easy?

P: Very easy (Breathing heavily).

I: For you. So, you mentioned earlier on, that you know you were thinking about older people. What was your worry about older people in relation to the box?

P: There are other older people that can't or what would I say, they're not well, even if you could train them on something, even if you can train them on something, after two hours they have forgotten, yeah. But now regarding this, it’s something that- what can I say, its objective goal. Is more like objective, because even if you could tell them there’s beep that's going to be at nine o’clock? Nine o'clock it will be beep, yeah. So, it’s not like nine o'clock a grandmother can go to the box and open it, sometimes it will remind you that you said nine o'clock. So, come in open.

I: Alright. It’s good to hear all of that. Can you please describe the training and the resources that staff received on delivery of the digital adherence technologies, including differentiated care? How was the training that you received about this digital adherence technologies?

P: The training that we received on ASCENT?

I: Yeah, how was it?

P: The training was well; how could I say? I won't say the word nice (laugh). It was well for me and everyone. I went with the health worker and the counsellor. We well understood everything. Everything was understandable. There was nothing that I would say was difficult for us.

I: Okay.

P: Yes, and the resources that were used, the ones that we received.

I: Mmm.

P: Yeah, when we got there. We were trained on the box. The battery recharges, the USB that's going to be used and also the tablet. How to register a patient, how to register the names address, which TB the patient has, and the medication, you put in the date that you start the patient on. Until the day that the patient finishes the treatment, huh we also received T- shirts and caps.

I: (Laugh) okay.

P: Yes (laugh).

I: Alright, so the training was -was fine?

P: Very fine.

I: Okay, how do you feel about the duration of the training? Is it fine for you? The duration of the training?

P: We only had the training for a day.

I: Mmm

P: We only had the training for a day. It was very short.

I: It was short?

P: Yeah

I: Okay, what would you suggest?

P: Maybe a two weeks training because for me, I knew other things, but for others, the juniors that I was with, maybe the health worker, especially the community health worker and the counsellor, maybe there are other things that were supposed to be taught on regarding TB.

I: Okay. So, do you still remember how you were trained? Was the training done at the clinic or you went somewhere else?

P: We went to A new something, I forgot.

I: Okay.

P Is it a guesthouse or a lodge?

I: It’s a hotel.

I: Okay. So, if you are to suggest something for us to improve on the training, I hear that you're mentioning that healthcare workers maybe community health care workers are supposed to be trained longer. What else can -can you suggest for us to improve the training?

P: On training?

I: Mmm

P: On training, there is none.

I: There is nothing, okay. From your perspective as a healthcare worker, can you please describe the benefits of implementing digital adherence technologies in the facility? What are the benefits of this program?

P: The program will help us a lot ne (right), firstly as a professional nurse it will help me- because for me, it will help me because firstly, monitoring a patient will be easier for me and also getting defaulters will be easier. If you have a patient who's struggling with something, it will be easier for you to know whether the patient is defaulting or whether there's a problem with regards to the box that you gave, so you will know quickly, because if the patient doesn't open the box for a day then you will know something is up, and you have to go and phone the patient. They will tell you, “sister, I'm struggling with this thing. It's not that I'm not taking my medication."

I: Okay.

P: Yes and also, for us professional nurses, because we do a lot of work, it minimizes work for us because you have a community health worker, and you have an intern, so if you initiate the patient, your intern will be here also, counselling the patient on ASCENT, not only counselling the patient on ASCENT, also on TB as a whole. So, you will be doing something, and the other one is helping you with this. So, the work becomes easier for you.

I: Alright.

P: Even if you get a patient on TB initiation. You won't just get a headache that I have to do a lot of work. I have to write there and there; you know an intern will come with this and the other one will come it becomes easier.

I: So, let's think now in the future without the ASCENT or without the xxxx (organisation name) support which is the intern. How is it going to be like for health care workers to implement this smart pill box?

P: If there's no?

I: Support from the xxx (organisation name)?

P: But you have the box?

I: Yeah, you have the box only, you have to just give the patient the box and explain how the box works. How is it going to be like, do you see it possible, is it doable to deliver. I just want you to have a picture without the support of xxxx (organisation name) it’s just the box only. You're just offering the box to the patient. What will be the challenges?

P: No, it won't be easier for us.

I: Why do you feel it won't be easy?

P: Mmm you’ll have to monitor the patient, but that's when it's easier you can monitor because you will be using a phone and then huh, regarding other staff and counselling. I think now you will be alone as a nurse,

I: Mmm.

P: But it won’t be easy.

I: What will be difficult because all other things are not going to be there, it's only the box that is going to be there?

P: So, you're going to give out the box.

I: Yes, that's the only thing that you're going to be doing is giving the box and registering the patient on the app and monitoring them from the following day.

P: But then, if it's like that, then if the box is not the same as the other one, then you will just have to give the patient the box, then after you register the patient, you monitor the patient after six months the patient gives back your box. Then you give out the box, it can do be done.

I: It can be doable?

P: Yeah.

I: Okay, All right. And then from- can you please describe challenges now? I've heard about the benefits. How is it benefiting you, It's making your life easy. Minimizing the work, you know, how do you think it's benefiting the patient? I've heard you talking about the healthcare worker in terms of the patient, how are the patient's benefiting from using the smart pill box?

P: They're benefiting a lot, because it’s alarming the patient on a time, you have to take your medication.

I: Mmm

P: They're benefiting a lot, because it’s alarming the patient on a time, you have to take your medication. There are other patients who forget, you will give them today, but tomorrow they even forget that they came to clinic, and you gave them or others who don’t take their conditions seriously. If a patient has TB, we'll just say you have TB, then you say I've been hearing from others that they have TB, so this one is better because it alarms the patients that you said nine o'clock. Then nine o'clock every day drink your medication.

I: Okay, all right. So, let's think about challenges now. What are the challenges of implementing the smart pill box? This technology together with the differentiated care, the calling, home visit, the SMS thing? What are the challenges of implementing the whole program?

P: Huh, the only challenge that I encounter sometimes maybe because we have a problem with load shedding, load shedding problems here in XXX [clinic name]. Electricity will go 3 times or 4 times. So, SMSing, other times, maybe the patient don't get those SMS’s because they haven't charged their phones.

I: Mmm.

P: And also, I think others it’s the battery. I don't know if it's possible if the battery can be for a six months lifelong battery.

I: Yeah, it is for six months.

P: Is it?

I: Mmm (yes).

P: So, they don't have to come back, because I have had the other ones who came back.

I: Is it what they are complaining about? What are they all about?

P: Isn't the device.

I: I want to hear from you. What are the complaints, are they complaining about the device itself? Or is it the battery? Do you have an idea?

P: I think it's the battery, the other one was the battery.

I: Okay.

P: Yeah.

I: All right. Okay, no, there is a plan for charging the battery, you can even give them cables-

P: To do at home?

I: Yes, to charge. Okay, and then from the- from your perspective as a healthcare worker, do you think TB treatment can be improved by implementing digital adherence technologies at XXX [clinic name]? Do you think TB treatment TB -TB treatment can be improved by implementing this digital technology technologies in the- in the facility?

P: Yes. It does a lot.

I: Huh, okay. So, we're still talking about the benefits do, you think from the healthcare workers perspective, can TB treatment be improved by using these digital adherence technologies?

P: Yes, like I said, like I said that it's alarming. It reminds them, it will remind them every time and also registering them on the tablets helps us to know of patients who are defaulting and stuff. So, it's improving that because you get a defaulter before the patient defaults for long.

I: Mmm

P: Yeah. So, if it's a day then you go and call the patient to remind them. He’ll know that tomorrow I have to drink my medication.

I: Does giving the patient a smart pill box as a health care worker improving your relationship you have with the patient?

P: Huh, it does, because every time you will have to check and ask the patient whether the box is working fine, are they good with the box, is it working. So, you get a relationship with the patient, because you always talking to the patient rather than giving the medication and the patient goes.

I: Mmm, how do you feel about that, that it strengthens the relationship you have with the patients?

P: (Laughs) it feels good, it feels very- very good because its helping us and the patient, akere (isn’t it) mostly the health care worker and patient relationship doesn’t strengthen that much at other times, but now because on daily basis you ask the patient if that thing is working, are you good, so it strengthens the relationship, and it feels so nice.

I: Alright, please elaborate on the positive changes that the implementation of the box and the differentiated care brought in the facility? What are the positive changes that you can highlight to me? Ever since we have started to implement?

P: The default rate has gone down.

I: Okay. What else that went gone down?

P: Huh, I would say maybe that thing that patients had before to be afraid to take out sputum, so now they cough, when you tell the patient go and cough they do.

I: Okay. So, before patients were scared of taking out sputum? Were they scared?

P: It’s not like they were scared as such, but when you tell patients to go and cough, they associate coughing with other staff that are not there, and that were told by others.

I: Things like what? So, we were still talking about the positive changes, and you highlighted that the defaulter rate went down. So, I just have an interest to know what else improved, because of the implementation of the smart pill boxes that you've noticed.

P: Maybe for our- for our facility.

I: Mmm (yes).

P: Yes. So, we have improved because all our TB patients are taking medication and they're finishing on time.

I: Okay, that's good to hear and do you think it’s because of this box?

P: I would say it’s because of the box, because the box forms part of monitoring, yeah. So, you monitor them digitally and you see everything. So, the box forms part of it.

I: Okay, so what are the negative changes that the implementation of the ASCENT or the digital adherence technologies brought in the facility that you have noticed?

P: The negative?

I: Yeah.

P: When you are carrying a white box, then they know what are you here for, but then it's not like you're coming with that box every day. Maybe if you're initiating ,then tell a patient you will be given this box and it will come in? And then the patient will say when I come out of here, they will know I came for something different from them.

I; Okay, alright .Can you please tell me about people that are difficult to- to- to give this small pill box? Who are those people like, do you have a group of people that you find it difficult to offer them the smart pillbox.

P: Regarding what, race or gender?

I: Both, anything that you can think of that make you struggle to give this patient a box?

P: It’s the males.

I: The males, why?

P: I don't know, they take time to understand what you're initiating to them. If we give them a new thing, it will take time for them to understand that thing.

I: Okay. Well, what about the homeless people and maybe people that are on drugs? Do you ever have a problem to –?

P: Do we have a patient that we initiated that is homeless?

I: Mmm (yes), you never had something like that?

P: I’ve never had.

I: Okay .So you mentioned earlier on that, you know, patients will start to be worried in terms of the negative changes. They start to think that people will associate the box with TB and that's a negative experience. How can this be changed from your perspective? How can you improve this negative?

P: And there is no other way that the box could be smaller, there's no way because it's going to contain pills

I: And that is the smallest box?

P: Yes, I think only health education for the patients because we can’t change the box to be in a smaller so that they'll put it in a pocket afterwards. Maybe going out. No one will see what are you here for, but huh it's only by winning a patient if you can health educate them, that it’s not as if patients or anyone notices when you go out if you have a white box and they’ll know what you are here for.

I: Okay, can you please describe to us what the systems in place are? That could monitor- that could monitor the digital adherence technology program. What can we do to monitor if this program is working or not? What are the systems in place to monitor these technologies? In the clinic?

P: Besides the one that you have?

I: What do you have?

P: We have the tablet, and the data capture has the thing on the system.

I: Besides that, one

P: Besides that, one?

I: Mmm, how can we monitor the ASCENT program if it's working or not?

P: Because now you said ASCENT is digital?

I: Yeah.

P: I can say the digital. So, I think if the person or people who are working with that will have something or maybe a software can be put on my phone as a- as a- as a professional. So that, maybe when I'm home I ,I could see if so and so rather than specifically being given, is it doable?

I: Being given what?

P: Not specifically being given a tablet so that this one tablet specifically belongs, is it acceptable that everyone can have who's in the facility can have that software to see on?

I: Mmm, yes

P: So, a data capture and all of us nurses. If you could see when you are home ,then you could say oh, so and so is doing this.

I: Okay.

P: Other than waiting for whoever or the data capture to say come and see.

I: So, everybody must have the APP to be able monitor-

P: The App, yes.

I: To be able to monitor if TB patients are doing well or not?

P: Yes.

I: So, how can we capture that, anything that you capture to indicate that this program itself is doing well? Any idea, we capture maybe have a document where we are capturing maybe write every week, just to monitor the program itself. Now we're not monitoring the patient. But we're monitoring the problem itself. How can we look monitor if this program is doing well or not?

P: So maybe you'll have stats.

I: Stats for?

P: For patients and whether they (people) who are working with it are agreeing with that? Is it doable? Does it work? Maybe monthly, we'll get- we'll get some sort of a report from patients-

I: And also from the staff will be giving us a report to say?

P: Maybe statistically 98% for this week, we have work to 98% or is it 88% of patients-

I: That are registered.

P: We have registered, there are no defaulters, the ones that are coming for treatment.

I: So, we need to capture that down?

P: Yes.

I: And have something like a report.

P: Yes.

I: it’s making sense.

I: Okay, so if we are to collaborate, please, elaborate. Please describe to us what system level structures need to be improved in order to integrate the differentiated care and the technology, together with a TB existing program system. What can we do to collaborate the two, the ASCENT program and the one that you have at the moment at the clinic? What is it that we can collaborate?

P: The existing one besides the box?

I: Yes.

P: At our facility we are using the cards.

I: Yes.

P: Those ones can just be collaborated, because with the cards *akere (isn’t it)* you have to take out the file, the patient has to take out the file, irrespective whether that patient has a box or not. So, we were using green cards, so they can be used together with this one.

I: How can we use those together? The green card and the box?

P: The box?

I: Mmm

P: When patients come for their tablets, sometimes they don’t come with the box-

I: Mmm

P: Others don’t come with the box, those that know how to open the box and refill-

I: Mmm

P: When they get here at the facility, they have to provide us with a green card, so that we know that these ones are our TB patients.

I: Mmm. So, who do you think can work with you guys to sort of assist you in the- in the program in the implementation of this digital adherence technologies? What kind of staff can work closely with you? Besides interns, in the team that you have at the clinic,

P: The team that we have?

I: Mmm the team that is already working? The team that is already working with TB, TB management or TB support? Who do you think can work closely with you when you have to implement the digital technologies?

P: I think the counsellor.

I: The counsellor?

P: Yeah, the counsellor will help us to counsel the patients and also the counsellors, the health workers will come in when the patient has defaulted.

I: Mmm

P: Yeah,

I: So, counsellors are the clinic?

P: Yes.

I: Okay. So, those are the people that can work with you closely to implement the program?

P: Yes.

I: Huh, okay, so what do you think- you are mentioning that the counsellors will counsel the patient? What else do you think they will do? Just think about the whole program *ye* (of) ASCENT?

P: Yes. They will counsel the patient and then they will introduce the box to the patient, yes.

I: Okay.

P: Refilling of those boxes of patient-

I: Okay.

P: Yes.

I: All right. So those are the things that you think they can support you with.

P: Yes.

I: Huh, okay. Anything else that you think is important that you would like to mention to me that we did not touch on thinking about the ASCENT program, in terms of the implementation anything that you think you'd like to talk about?

P: No, no, I have nothing.

I: Okay, thank you. Thank you so much for the information that you have provided.

I: We really appreciate, it means a lot to us. And we are at the end of the interview and now time is: 13:01.

P: Thank you.

**GLOSSARY**

*Akere (Isn’t it)*

*Ne (Right)*

*Mang mang (Whoever)*
