# Supplementary material for: Acceptability of Digital Adherence Technologies to support people with drug-susceptible TB in South Africa
Source: PLoS One. 2025 Sep 24;20(9):e0332103. doi: 10.1371/journal.pone.0332103 (PMC12459780; doi:10.1371/journal.pone.0332103)
Supplement: S4 File — (ZIP) [file pone.0332103.s004.zip › S4 Transcripts/HCWs and Stakeholders/IDI 16_ HCW.docx]

**TRANSCRIPTION NOTATIONS**

| **Label Key** | **Meaning** |
| --- | --- |
| **I** | Start of each new utterance by the Interviewer |
| **P** | Start of each new utterance by the Participant |
| **N** | Note taker |
| **{ }** | Indicates that details were changed or pseudonyms were used to anonymise data |
| **( )** | Indicates the description provided to anonymise data |
| **XXX** | Words were omitted to anonymise data |
| **-** | Breaking into a sentence by the next speaker |
| **…** | Pause or drawn out words |
| **[ ]** | Indicates noise made, e.g. [laugh], [sigh], [pause] |
| ? | Beginning of utterance by unidentified speaker or questionable text |
| **[inaudible segment]** | Unclear section of the recording |

I: Uh-mm thank you for agreeing to talk to us today. Uhh I would you to allow us to audio record this interview.

P: Yes.

I: We can record the interview?

P: You can record the interview.

I: Oh okay, uh PID number is 35… it’s xxx. Date of the interview xxxx (interview date), location xxx [clinic name], xxxx (district name) in the xxxx (province name). Facilitator: xxx [interviewer’s name]. Time of the interview, it’s 14: 53. Uhh tell me sister, what is the title of your current position at the moment?

P: I’m a Professional nurse.

I: Okay.

P: Yes, at xxx [clinic name] Clinic.

I: Mmm.

P: And based in…wellness, that is TB and HIV.

I: TB and HIV?

P: Yes. Including under chronic conditions.

I: Okay.

P: Yes.

I: How long have you held this position?

P: ….Yoh, I graduated long time ago, it’s-but in this facility, it’s xxx years now.

I: It’s been three years now?

P: Yes.

I: Okay. So, when it comes to patient care and counselling, what are your roles and responsibilities?

P: Uhh counselling is a continuous thing, remember we- with TB clients, let me be specific- with TB clients, it’s continuous. We need to counsel almost every day, so that they comply and adhere to their treatment. If you don’t keep on counselling, *yah* [yes] somewhere- because they, they come to the- but there are changes. Remember we have the social issues, health issues and what. So, if you don’t counsel, you’ll never see even when the patient need to be referred or when the patient has a social problem that needs your attention more especially when we talk about uh referring to dieticians. They don’t have food to eat, they have to- so, if you don’t engage with them, you’ll never get them right. So, that’s what I… I can say about counselling. So, my role is to deal with counselling on daily basis as they come to collect their treatment and I have to refer not only to a dietitian but to some othe- we have Batho Pele, it’s one of the organisations that is based here. So, it's just near Sunrise, so they ( patients) are able to go there to collect food. They are able to send them- and like now that uhh xxxx mine was looking for TB patients, including those with HIV, and those that are not working. Those that don’t have anything to put on the table, so what they do is, they go there and xxx would give them vouchers (patients). So, they benefit by from that. Other than that, my role again is to see *ukuthi* [that] they are connected with that…lady so that, *lebona* [and them], they need to be happy too, honestly speaking. Remember… you cannot drink medication on an empty stomach, right?

I: Mmm.

P: And even there by the gardens- when we have some spinach and other things, we do give them.

I: Mmm.

P: Uh its sorts of a… a support.

I: Okay. So how is it to refer a patient to Batho Pele and the other NGO that you’ve mentioned?

P: Yes.

I: Is it- how is the process of referring a patient? Is it easy for you to do that?

P: Very, very easy. When I talk of DOH (department of health) and the partners good relationship makes it much better. The manager there, we communicate almost every day. If we have a challenge here, I just send them WhatsApp, then he responds and sometimes we call, and he does visit here.

I: The clinic?

P: Yes. And sometimes if there’s like- he the one who said, “can I have a group of HIV and TB clients who are not able to provide for themselves and those that do get the R350 (social relief grant) but it’s not enough.

I: Mmm.

P: Yes. It’s a relief but it’s not enough because you find that there are too many people in the household, like that other house that we visited. They were- I think plus minus 11 people in the very same house with a TB client. So, it becomes a problem, how do they fit? How do they sleep? How is the ventilation? How is- are the children at school?

I: Mmm.

P: So, it, *yah* [yes] but we have a good relationship. Even in my absence, she (referring to a patient) does come.

I: Okay.

P: Yes.

I: So, you are mentioning that you’re doing counselling-

P: Mmm.

I: And also refer a patient when needs help-

P: Mmm.

I: And make sure that they are assisted-

P: Definitely.

I: What else- what else are you doing within the TB care, except counselling. What else are you doing?

P: Support

I: Mmm.

P: Uhh support, we are- I think from the counselling part of it, that’s when we give support, so that’s my role.

I: Mmm.

P: Unless if I don’t get the question right. It supports, the most. Remember we support them even in hard times, for them to be able to finish their treatment *ne* [right]?

I: Mmm

P: And even the support - there are those who are not supported from home, not anywhere and being there, empathising with them, being an approachable person, people are able to open up.

I: Mmm.

P: Even in hard times, support is really needed by them (patients).

I: Mmm.

P: Yes.

I: Alright. That’s good to hear and I can feel that you are doing this with a vey strong passion. And how are the TB services delivered at your level regarding the intervention, which is the DAT intervention?

P: Can you repeat that?

I: How are TB services delivered at your level?

P: Uhh.

I: What exactly are you doing in the facility when it comes to intervention?

P: Okay. Uhh remember, from day one when they come it’s a process. It’s TB screening first before we can get the TB client.

I: Mmm.

P: So, my job is to see- to find TB patient through TB investigations, and it’s only through screening.

I: Mmm.

P: One who coughs, from the, the que, it- she or he need to come forward. There is a fast que, so that if she or he is TB positive, she doesn’t infect other people. So, the long que that I’m doing, starts there. TB invest- screening first, then we investigate with our Gene Expert, and when positive, you start treatment. So, when we start- that my role and I initiate TB patients and conduct counselling after. And from there, we are looking for the contacts of those client to say because you are living with these people, so they must come also and give us their sputum. And then from there, we enrol that patient on your- *mina* [me] I call it a pillbox, anyway

I: Mmm.

P: Yes. We enrol them on the pillbox. I know how to enrol patient on pillbox.

I: Mmm.

P: And it goes with passion and it’s so simple, and it’s very, very beneficial to me and to people in the health facility. I think it beneficial to the Department of health as well. If they were using it. I’m not sure if all the clinic are using it , but I really love that.

I: Mmm.

Yes.

I: Uhh so you are mentioning that you really love it, what-

P: Uhh.

I: What is it that you love the most about DAT?

P: What I love is that, let me tell you- remember I have uhhh 95 clients that are on TB. So, which means I have 90 files. On TB, now it says every now and then, for me to get the defaulters and to see how they are doing. I must pick up files one by one.

I: Mmm.

P: But with the model, it’s very, very simple. You just go there, and you search your patient, you get your patient. If the patient is not on treatment, you are told, it informs you. So, it has got too much information and it makes my life easy, truly, honestly speaking.

I: Mmm.

P: There’s an SMS, you can say *abuti* [brother] you didn’t come to collect your treatment, very, very simple. Unlike going to all these files. Remember I have patients to take care of.

I: Mmm.

P: I have patients to initiate, I have patients to support, and I have patient to educate. So, it takes time for me to do all that, it’s time consuming. To look for the files one by one, for me to get the defaulters. So, with this phone, it’s very simple, truly, and honestly speaking. I love this and the other thing, it reminds our clients. when we talk about electricity now, *ne* [right]? there’s no electricity and I cannot charge my phone, but the pillbox goes wow. It knows it job and what to do, and that a reminder. I never charge my phone, this (pillbox) reminds me as it supposed to and that promotes adherence, and it also promotes the compliance. So, you can say, it something like a pillbox but a box that does wonders.

I: Mmm.

P: Truly and honestly speaking, and on top of that, I don’t have too many defaulters because of that, *ne* [right]?

I: Mmm.

P:On the other hand, most of my people that have completed treatment. They completed treatment without any problem. So, its something to manage very easily.

I: Mmm.

P: Mmm.

I: Uhh.

I: Okay.

P: Even the children, when you are watching, when it says twi twi twi [beeping] they just like, “*papa* [dad] your time is here.” You, see? So, it something else- unlike waiting for Generations (TV drama) , if there’s no electricity, the Generation is not playing on the TV and if your phone doesn’t have battery, you won’t remember to take your medication. And now it doesn’t give a very good adherence.

I: Mmm.

P: *Ya* [yes] so it does wonders xxx [interviewer’s name], it does.

I: Mmm.

P: It does.

I: Good to hear that. So, how do you call this uhh ,how do you call the platform for monitoring adherence, a platform that is on the… how do you call it?

P: Yoh you know what I do?

I: Mmm.

I: How do you call the platform?

P: What is it, Everwell, *ya* [yes] it Ever, ya [yes]

I: Everwell?

P: *Ya* [yes] when I go here, I just remember the ever, part of it.

I: Oh

P: *Ya* [yes] so when the phone, the, when, if I see the Ever then I go.

I: [Laugh]

P: *Ya* [yes] it’s only the Ever that is there.

I: Oh

P: So, it’s Ever, okay.

I: So, once you see Ever, you go for it?

I: And register a patient?

P: Yes

I: Okay huh if you have to explain, what is DAT intervention, including, medication device and monitoring platform to another health care, who knows nothing about it, what would you tell her? If you have to explain.

P: If I have to explain?

I: To someone-

P: That, *ya* [yes]

I: Who does not know about DAT.

P: *Ya* [yes] okay

I: Including medication device, monitoring platform. What can you say to that health care?

P: Uhh the, the knowledge that I’m giving to the client, it like- look there’s a new model of TB that we are using in our facility. It’s a very simple, manageable, available, you know, model and what it does, it works in this way uh you- it works on newly diagnosed TB clients. When you get your client, when you want the client to comply and adhere to treatment, then you register them on this model, and after registering, it takes everything. It, almost the blue file that the client it is using. So, that information- you put that information on the phone. And uhh after that, it will help you to see if your patient has taken the treatment for the day. So, it just a simple thing, it’s a pillbox, it has got batteries, so every time when the patient comes or when the patient before he comes for the second time. On the first time you give the patient the pillbox and then you explain them, then he or she will sign the consent that I am receiving this model as a reminder for my treatment, and then from there, you just chill, you put the tablets that the patient is going to collect inside the pill, and you close it. You inform the patient about the batteries, and about the yellow light and the red light and then from there, when, when, when you open the pill-

I: mmm.

P: It will reflect green and when it’s time for you to take the treatment, it will keep on ringing, ringing like a siren that is saying it’s time for medication. Then the patient will just take their medication and it is less time consuming for us when we are going look for defaulters. You going to do your uhh what is it? Uhh what do we call that, or did, so you do it there, you confirm it here and it will direct you straight if your patient is taking treatment or not. So, it is an easy model available and after, lasts for something like 6 months, so you know very well that we’ve got six months TB and there are those that can, that exte- that is extra pulmonary TB. But within these six months, when we talk about TB that is very infectious to, to, to, to other people, then the six months is okay, but if the battery gives you a problem, it a matter of just a phone call and then they come, they fix there or you change another battery, so it as simple as ABCD.

I: Okay. So, in the programme, we also have what we call, differentiated care. So differentiated care is when the patient when they are missing medication.

P: Mmm.

I: They get an SMS, we call them, sometimes we even do a home visit.

P: Mmm.

I: And you know, in the morning, you check your tablet and check all your patients if they are missing medication. People who missed yesterday and follow up on them. So, I want to know, in terms of all the activities that I’ve mentioned, which one are you really doing most of the times, are you doing all of them or some of them, and which ones are those that you are doing?

P: I think we do all of them.

I: Mmm.

P: Remember, the client- we send an SMS, you call, and then the client doesn’t come.

I: Mmm.

P: *Akere* [right]? You call again, the client doesn’t come, so, remember there’s an address for the client when we, where they are staying.

I: Mmm.

P: And we just (…...) refer, it’s not, *ya* [yes] it’s a referral.

I: Mmm.

P: We just take a (…) it to the O.T, what is it, OTL (outreach team leader) *neh* [right]?

I: Mmm.

P: They’ll send the nurses that works outside, *akere* [right]?

I: Mmm.

P: Then we give them- they are also at the facility *akere* [right]? Remember, we are working, working as a multi- disciplinary, it’s not only me, but there’s also somebody who’s doing it outside then we say we’ve got patient number one, house number this uh she missed treatment for these days then we give the address, and they go.

I: Mmm.

P: Yes. So, because xxx [clinic name] is very big, we engage with the, the clinic committee, they go there.

I: Mmm.

P: Yes. our chairperson, the clinic chairperson.

I: Mmm.

P: Uhh there are very, very familiar with the place because they’ve been there for a long time. So, they, they do go and check for the address if the address is correct.

I: Okay So, in this differentiated care, what exactly is your role, what, what exactly do you do? You first check in the-

P: Mmm.

I: Platform and then, can you just give me in steps *ukuthi* [that] what exactly are you doing when you are offering differentiated care to the patients.

P: Okay. We check the inside the phone and then after checking there-

I: What are you checking on the phone?

P: We are checking the missed doses, the number of doses and then after that, we call the patient first before we can, and then sometimes we find that, when we call, the patient is not there, remember, some of them are working underground, so underground you can’t reach them at all. So that’s when now we can send an sms. Remember they don’t don’t know their phone; other people just leave the phone when it rings because have got other issues. So, they just say, more especially if they don’t know the number. So, if you send an SMS and then after SMS you see that they are not coming. *Akere* [right]?

I: Mmm.

P: So, the next step is- you, you go to uhh what is it uhh we, we send the OTL to go to the address of the patient. So, we send the OTLs to go there, and that is home visit now.

I: Okay

P: Yes

I: Thank you for that. And then please describe the type of staff that were involved in delivering differentiated care, so you mentioned earlier on that you are sending these who, how do call them? the type of staff that you send.

P: *Ya* [yes]

I: To do the home visit. How do you call them?

P: Its OTL

I: Mmm.

P: OTL it’s also, what do we call this? We’ve got community health care workers, *akere* [right]? CHW

I: Yes.

P: So, the OTLs are their managers, they are nurses.

I: Oh.

P: They are nurses

I: Okay.

P: *Ya* [yes] so those are nurses, they do everything outside, immunisations and what, what. Home visits. So they work outside. They are not working for the facility per say, in the facility, they are road wise.

I: Mmm.

P: So, *ya* [yes] so that’s the OTL, that-

I: It’s the nurses?

P: They are nurses

I: Okay

P: And that is CHW (Community Health Care Workers) and through all the sections we’ve got, like, we have so many sections, but you find that they are two, they are employed by the uhh Department of Health, they are trained, they are paid for it.

I: Mmm.

P: Yes. And because most of the community health care’s stay in xxx, they know most of the patients there, even when we say a patient stays in xxx place- we have two CHW that are based at xxx . So, they know the people there.

I: Mmm.

P: Yes, they know the place. So those are people who will go there. I would give them the address, they are the ones who say, “no I’ll go there.”

I: Mmm.

Yes. And the OTL will only go in if the person didn’t come after receiving a message from the CHW uhh message.

I: Okay

P: Mmm.

I: Oh, so can you please tell me how are you sharing the responsibilities? Let’s start by you here at the clinic, and you mentioned what uhh the nurses that are placed in the community doing.

P: Mmm.

I: And what are, the CHWs are doing?

P: Yes, uhh.

I: So, tell me how you are sharing the responsibilities amongst the three of you. Starting from you, so you see the patient here at the clinic.

P: I see the patient at the clinic, I go through my, my digital, *akere* [right]?

I: Mmm.

P: And then if I found that there’s patient that missed that dose, I call the patient and then if the patient didn’t pick up for some reasons, I don’t know. We send SMS and if they don’t respond after the SMS , we give grace, my grace it’s only three days, if you don’t turn up in three days then, remember we trying to, to say, we don’t want to build resistance towards the TB. So, within seven days the client needs to be here, but three days, if the client is not coming, I’ll only take it as maybe there’s a problem but then it’s then that I call the CHWs in.

I: Mmm.

P: And say, CHW I have this person who’s not coming in here. This is Mr so and so and then then the CHW will go first.

I: Okay

P: Yes. And as the CHW goes there and the person is not there so I’ll call the OTL to, to go in there, but the thing is, both are engaged at the same time. Remember, the OTLs are the supervisors of this one, so I don’t really have to send them without their knowledge.

I: Okay

P: So, they need to give me the time for them to go. So, it something else.

I: Okay

P: *Ya* [yes] but that is how it works.

I: Okay

P: Mmm.

I: Uhh do you remember your expectations about DAT interventions before it was implemented?

P: *Ya* [yes] one of them-

I: What were your expectations?

P: Getting to zero

I:Uhh?

P: Getting 100% cure rate, truly and honestly speaking

I: Mmm.

P: To see TB infections declining and higher cure rate, my cure rate was my first priority, to see the people getting cured, to see the people completing their treatment, to see the people beating MDR. So, I was like, that was my expectations.

I: Mmm.

P: Yes.

I: Okay

P: And it makes it very simple for the people to be reminded, not to be difficult for them to remember to take their treatment.

I: Okay

P: Ya [yes]

I: So, you expected it to be, something that is easy to-

P: Easy to manage.

I: Mmm.

P: Yes. And that was my last ninety. Remember, there’s ninety, ninety, ninety, *neh* [right]? 90 percent of the people need to be screened for TB, 90 percent need to start on TB treatment immediately when they are positive-

I: Mmm.

P: Or Gene Expert detected, and 90 percent of them need to be cured.

I: Mmm.

P: Yes

I: Did your expectations change, like all these things that you’ve just mentioned, did they change? You were expecting it to be easy like you said you were expecting this DAT to be easy, to manage patients, to decrease-

P: I expected that-

I: TB

P: But for me it did make a change, truly speaking, it did make a change. We had a very, very serious challenge when we came in here.

I: Mmm.

P: *Ya* [yes] when we came in here it was chaos (…) if you can look at 199 (…) 2019 when they opened here.

I: Yes

P: It was so difficult to reach the people with TB, and the other thing, including the address, they were not the same (…...) . So [ door opened] even the addresses that they give, it’s not easy for you to get them, but with this one [laugh] there’s a communication.

I: Mmm.

P: Has improved a lot. So, there’s a lot man.

I: Mmm.

P: *Ya* [yes]

I: So, are your expectation met?

P: Met?

I: For this one?

P: Yes they were met although I cannot say, it, it was met 100 percent. Okay? Remember, this is a new model. Right?

I: Mmm.

P: And *ya* [yes] so, but I can say we are getting there.

I: Mmm.

P: We are getting there. And if the DOH can really adopt this model, I think we can go. Let’s say in South Africa-

I: You think we can? If we can, if we can, if they can adopt?

P: *Ya* [yes] adopt.

I: Mmm.

P: It all about the adoption. Yes, if they can adopt this, I don’t want to lie, remember, even the phones that are here on this file are wrong, but they give you the right phone numbers when you go there. The right address, but you find that, this and that don’t match, you go in here you get them right.

I: Mmm.

P: *Eh* [yes] so I love this. Fez

I: So, what will happen if DOH can adopt this strategy?

P: Ah we going to go to my 90, my other 90 cure rates will go high.

I: *Ya* [yes]

P: I think so. And the defaulter rate will definitely drop. And the MDR part of it *liyona* [even it] will drop.

I: Mmm.

P: Mmm.

I: Why do you have this strong feeling that-

P: I’ve used the device and I know what it does. I know what it did to me at the clinic. I know how people love it.

I: Mmm.

P: Yes.

I: Okay. Can you tell me about your experience using the DAT in terms of, what is it that it has done to you as you are saying?

P: It’s a very, very good model and very simple to use, accessible and there’s nothing difficult with it.unlike paper work.

I: Mmm.

P: *Ya* [yes]. Remember, files got missed, man. Sometimes you can’t even find the file, but with this one. I love this, it makes my work very easy. For the patients, is it making their lives easy.

I: Mmm.

P: Because you can see even when they come here, man, they are, they’ve got their pillboxes with them. It’s something you don’t-if you’ve got something that you don’t love, you can’t really bring it to the clinic like this, no. You’ll put it there, you won’t even use it. But for the mere fact that they are using it xxx [interviewer’s name], I think its really working for them. The reminding part of it, I think it’s doing wonders. “Yes, Sister, can you see, I am only left with one tablet.” Sometimes you find that I give the wrong date, but with this, *neh* [right]? It shows and the pills when they take it almost every day, it can say now you are left with three-

I: Mmm.

P: Tablets. It’s Friday, you’re left with three tablets. What are you going to drink on Sunday and Saturday and Sunday? They come on Friday.

I: Mmm.

P: Yes. They come. For me, it’s doing better for the patients and if you just put your treatment there-

I: Mmm.

P: And go there, nothing, nothing encourages you, nothing puts you to go there. But here you see the light, you hear the ring, you’ll go. So, it’s doing very good for them.

I: Mmm.

P: Uhh it’s doing very good. They might have told me long time ago that this is not doing well.

I: *Ya* [yes]

P: *Ya* [yes]

I: If it wasn’t

P: *Ya* [yes] if it wasn’t working for them, they were supposed to be open. I’m an open book way, very, very approachable. So, they were supposed to have told me in a long time ago and I would have informed you long time ago that ah xxx [interviewer’s name] your thing is not doing its thing.

I: Mmm.

P: Right?

I: Okay. That’s good to hear. Can you please describe the training and the resources that staff received prior to or during delivery of differentiated care?

P: Ai it was good

I: Mmm.

P: There’s nothing that I didn’t understand. The only thing is that I was so lazy to do it here [laugh], I thought it’s a huge thing and I thought-

I: What?

P: The training part of it, even the, the model, I thought it was going to be very difficult but as soon as the model comes in I saw it very, very simple. So, the training was uhh ok even though we didn’t practice it that much, as soon as you send the lady who’s working with us to help us, *neh* [right]?

I: Okay.

P: *Yeah* [yes] because there was an (…) we were (…) people were interactive. So, it was good.

I: Mmm.

P: *Yeah* [yes]

I: Okay. What was your opinion of that training and resources you received during the training?

P: What was my?

I: What is your opinion about the training and even the resources that you received during training?

P: The trai- the training was good xxx [interviewer’s name].

I: Mmm.

P: And even the [inaudible segment] that was used, I still-

I: The?

P: The T- shirt.

I: Okay.

P: *Yeah* [yes] I still wear that. It talks about ASCENT and uhh you know, the training was good. It just that, the implementation part, you think that it won’t- this thing won’t be working. The material was good mmm. For me I can rate it 100 percent. Even the trainers were good.

I: Mmm.

P: *Yeah* [yes] *yea*h[yes] truly and honestly speaking, it was good.

I: Okay. So, you are mentioning something like you thought that in terms of implementing, it was going be difficult.

P: It was that uhh reaction towards change. You know, here we are people who resist change, but uhh I didn’t resist anyway. I only think. *Yeah* [yes] a new model, wow.

I: Mmm.

P: Let’s see if it can work. But for you to push it to work, it works uhh harmoniously it is good.

I:Mmm.

P: I don’t want to lie and it did wonders.

I: Mmm.

P: *Yeah* [yes] it just that where there is change, there’s always resistance, what would you be praying, what would you do, will we (…) *yeah* [yes].

I: Mmm.

[Somebody talking].

P: Mmm.

I: Mmm.

P: *Yea* [yes].

I: Yeah [yes].Okay. So, do you have suggestions to improve the training that you have received? How can we improve the training if (…) who should be trained, duration of the training, who should attend, anything that you can improve in the training?

P: I don’t think there are some deep holes-

I: Mmm.

P: From the training, part of it. All the people there, they were prepared, they know their story, the only advice I can say uhh I am able to use the device, it okay.

I: Mmm.

P: And I was trained as a nurse from this facility. And I think other nurses from other facilities should come in and be trained as well. Reason being, remember, you, sometimes you take xxx [intern name] -

I: Mmm.

P: So that I don’t have to wait for xxx [intern name] , I’ve always got hands-

I: Mmm

P: So, I need to use the device.

I: Okay

P: So that she can leave the device unlike saying, the patient will be registered on this model when xxx [intern name] comes back.

I: Okay.

P: No. I don’t want to wait for somebody to come and do it while I know how to enrol a patients . You can help me to do it but, in her absentia, we need to continue working.

I: Okay. So, you need the device?

P: I do

I: To make sure that there is a device.

P: Yes.

I: Okay.

P: Or in her absentia, she can leave the device to work on.

I: Okay.

P: *Yea*h [yes] in her absentia.

I: Okay.

P: *Yeah* [yes] and I’ll take care of it.

I: Okay. It’s making sense. So, can you please describe challenges uhh you have with differentiated care. Right?

P: Wow [laugh].

I: Calling patients, home visits, you know, SMSs. What challenges do you have when you offer differentiated care to patients?

[Background noise]

P: The first challenge it’s uhh is the battery, when the battery is not working. [background noise] Challenge?

I: Mmm.

P: When the batteries are not working [laugh] the patients cannot come now, he or she will wait for the appointment that we gave.

I: Mmm.

P: So, that’s the challenge. And you find that uhh from the device, it shows red that he or she is not taking medication but only to find that if the device is not working, he was just supposed to come uhh three days ago when the device was not working. *Ne* [right]?

I: Mmm.

P: Or within a week, he can come and report that.

I: Mmm.

P: *Yea* [yes] I think it one of uhh something that we need to do on our, our daily education or when we engage them and register them on this. So, you only going to find out that this person is not a defaulter when he comes because the other one is like, “Sister I’m taking my treatment, why are you calling me.” Okay, and others, they didn’t disclose that they are on TB treatment. So, when uhh you call, *ne* [right]? it’s a problem if the wife can be the one who’s taking this. And then there are these ones *ne* [right]?

I: Mmm.

P: They leave their medication and the pillbox at work, they have lockers.

I: Mmm.

P: They know when it says, “ting, ting, ting, ting” [beeping], they just go and take and come back.

I: Mmm.

P: And then they don’t take the medication at home. But we are still encouraging disclosure because myself, I need contacts. So, with this TB and what, what. It, it, it’s a challenge, electricity, I mean the battery. That number one challenge.

I: Mmm.

P: And not reporting at the same time, and that one, patient will say, “I’m taking treatment, why are you keeping on calling me.”

I: Mmm.

P: That’s the other one.

I: Mmm so what are other challenges to implement this technology that you are experiencing? You mentioned the battery.

P: *Yeah* [yes] it’s the battery. Only the battery. .

I: Mmm.

P: The device is good. Only the battery.

I: Mmm.

P: *Yea* [yes] only the battery, *yeah* [yes]

I: Okay. Uhh is it something easy to give a patient the box, do you have some patients who feel somehow taking this box?

P: *Yea* [yes].

I: Can you talk about that?

P: But it’s one out of ten, right?

I: Okay.

P: Its one out of ten. Others they like to extend that uhh.

I: That one out of ten, what is she saying?

P: I have my phone; I don’t want any reminder.

I: Okay.

P: *Yeah* [yes] and, so as long as my signature is there, I don’t want that, even though you can say, others are-remember that somewhere when we talk about confidentiality, *akere* [right]?

I: Mmm.

P: So, when you start explaining the usage, the simplicity, the reminding part of it, that’s where they start to understand, most of them. But this one just said, “no Sister I don’t want that.” Even after explaining, “no, no, no I really don’t want that. I don’t think that will work for me.”

I: Mmm.

P: *Yea* [yes].

I: Okay.

P: I don’t. *Yeah* [yes].

I: So those-

P: So, it- remember have rights. You really, really not have to impose them.

I: Mmm.

P: For something that they don’t want. *Yeah* [yes]

I: Mmm.

I: Okay. So, from your perspectives as health care worker, can TB treatment be improved using this differentiated care model and the medication device, this box, this smart pillbox? In your opinion as a health care worker, can TB treatment be improved using differentiated care model and a smart pillbox?

P: Be improved?

I: Mmm.

P: Model? TB treatment adherence has improved because of this box.

I: Mmm.

P: *Yeah* [yes] it’s improved. We have so many people who are taking medication and that have improved and the improvement part of it *ne* [right]? You can see it uhh within 14 days when they are on treatment. You can see them uhh improving physically and uhh the other thing that I forgot, *ne* [right]? Uhh the people can say we are taking our treatment but not improving, right? And with the pillbox, it shows you that they opened, they drank. They opened, and they drank. And with others, it was very, very difficult for us to see, but its something that you see that is there. And it doesn’t take your time , you just open there, you take out in the morning. You don’t go inside the file and look for what, So you can do it uhh in the morning, I think so. I’m not sure if I did answer your question well.

I: Yes. You did. And uhh is the DAT and the use of this technology is, is it improving the way you work, the way you support your patients?

P: Yes

I: How?

[background noise]

P: Uhh if I didn’t check at it, I will never know that the patients did not take the medication.

I: Mmm.

P: And if she didn’t, I’m able to call the person. *Akere* [right]? And if you call that person, you can send an SMS. If there’s no reply, you can go straight there by sending the OTL to come, and that is a of lot of improvement. So other people, they don’t even want uhh us to go that road. You only do it once. And that you will never, never, uhh default again.

I: Mmm.

P: And remember, through the very same thing *ya* [of] *ya* [of] *ya* [of] defaulting, from there we have to go, we also talk about the adherence *akere* [right]? I told you this is a continuous education.

I: Mmm.

P: So, if you, you continue talking of adherence, talking of compliance, talking of uhh being open for them to say we have social problems, just tell us, we can help those struggling with food. They know very well that you going to refer them. So, it is working, it’s working.

I: Mmm.

P: It’s working, the box, even from the weight part of it, when they come you assess them it shows you that there’s a lot of improvement on this patient Right?

I: Mmm.

P: Yes. You can see.

I: Okay. So, can you please elaborate positive changes that the differentiated care and this smart pillbox, that is a technology uhh that it brought into the system?

P: Mmm.

I: And how can we sustain these uhh positive changes that you noticed ever since implemented the technology?

P: Number one.

I: Mmm.

P: Reduced defaulter rate. Increased uhh cured or treatment completion, and uhh lower uhh numbers of MDR for this year.

I: Mmm.

P: Reduce death rate, it can be one out of ten. Uhh and we can sustain it, it just that uhh this is not uhh my decision to make. But uhh for it to stay here and be longer, I don’t know how we can do it with the Department of Health to adopt the model.

I: Mmm.

P: To adopt the model. These are squatter camps, these are overcrowded uhh-

I: Areas.

P: Areas. So, we are also at risk - the health of TB as health professionals. But adopting this can, really, really make a lot of improvement.

I: Mmm.

P: *Yeah* [yes] it can, it can, a lot.

I: Mmm so-

P: *Yea*h [yes] because it, like you are looking at a camera. You are looking at somebody, some far away from you.

I: Mmm.

P: It seems as if you’re here with your patients. So, I wish they can adopt it, *yeah* death rate can reduce, the mortality, no.

I: Mmm.

P: But at least this year, I think few, few, few people.

I: Mmm.

P: *Ya* [yes] two or three since we used this. I think I only have one two deaths.

I: Mmm.

P: *Yeah* [yes].

I: Okay. So, at your level as a, as a TB nurse here at the clinic-

P: Mmm.

I: What can we impro-what can you improve for this technology to happen in the facility, what can be done to ensure that uhh it’s sustained, it’s continuing at your level as a nurse?

P: Mmm let me say, for now, for now it’s me and my partner who are working in here sometimes there’s another nurse who’s coming in.

I: Mmm,

P: I teach her.

I: You teach?

P: Yes, we teach. You, remember we need sustainability? In my absentia and if my partner uhh or subordinate uhh is absent, this need to continue, right? So, there’s uhh our manager, there’s another TB nurse who comes in here and they also need to know how to use the device and how to register people on the device and how to check your defaulter rate.

I: Mmm.

P: So, it’s educating other nurses.

I: That’s what they’ll do.

P: Yes, because uhh we rotate and when we rotate it’s not like they need to go and call me there, no, they need to know how to use the device.

I: Please elaborate on the negative changes, on the negative changes of the differentiated care, on the differentiated model of care and the use of the medication device, how do you think the negative changes could be addressed, what are the negative experiences that the DAT and the differentiated care is bringing and how can we address those negative changes?

P: Bringing where? To the facility?

I: Yes. To the patients, to you as a health care worker. Anything that is negative that you are experiencing because of this. And how can be changed?

P: I don’t have any negative, negative things that uhh I did. It’s only that challenge when someone says I don’t want to do this. But uhh like I said, we don’t really have to force the person but uhh on the other hand, it -what can I say, it favours others and not others. So, what I can say it’s uhh is that this uhh ASCENT part of it, remember they are not given the model, for me it’s not fair. Because they do love them, do love this pillbox and now [laugh] the first day I didn’t know, that was something that it, I don’t really understand. So, I take it as being negative.

I: Okay.

P: Because *yeah* [yes] and when they like it uhh, I think you can adopt the ages of being an adolescent. It can be given to somebody who can make thoroughly decision who understand, who can give a go ahead on herself. They’ve got choices as well.

I: Mmm.

P: So, they can say, “no I don’t want this, no I want that, no I don’t want this.” And the other one was really *mama* [mother] is taking this back and I just want to use this tablet for something else, I mean this pillbox for something else. And they are not engaged in doing that.

I: Okay

P: So, its something. So, if we can take it uhh- treat them according to AYF (Youth and Adolescent user Friendly), Adolescent User-Friendly services.

I: Mmm.

P: *Yeah* [yes] they take uhh youth as from the age of 12.

I: Mmm.

P: *Yeah* [yes] so, remember, I have these uhh 16 years, she’s on treatment and is not given the box but the mother received it and not her.

I: Mmm.

P: *Yeah* [yes].

I: Okay.

P: So, she says, “no, this one is drinking the tablets when I drink them, when I come from school.”

I: Mmm so-

P: Yeah. [yes]

I: So, earlier on you said, another thing that is negative is this thing of patients not wanting to take the pill- the box.

P: Mmm.

I: What can we do to address that? To make people receive it, accept it?

P: I think it continuously uhh educating them about the benefits, about the benefits

I: Okay.

P: So, the other one she was like no I don’t want it, I don’t want the box. So, he she didn’t mention why doesn’t she didn’t want it and some they are not open but it, I think the continuous education is needed remember initial phase it’s two months, so the first week we give two weeks treatment.

I: Okay

P: *Ya* [yes]

I: Alright. Can you please describe to us, what system- level or structures need to be improved to integrate the differentiated model of care and medication device technology into the existing TB programme systems.

P: Can you read the question again? Integrated?

I: *Yeah* [yes] please describe what system level structures need to be improved to integrate this technology.

P: Mmm.

I: With TB, with the existing TB programme.

P: So, the integration, you know if it just the pillbox. It caters only for TB drugs, right?

I: Mmm.

P: If we can have a small compartment for ART in the box, you know, a compartment that is very- too small for the ART medication. It will be nice.

I: Mmm.

P: *Yeah* [yes] so that it reminds in the morning, it reminds in the night. So that’s the integration part of it. *Neh* [right]? And then in case there is diabetic and TB, same thing that uhh space that we used to put ARV we put the HPT drugs or any other chronic drugs or the diabetic drugs in there. So, if it has got two compartments like this because for now, they put TB only.

I: Mmm.

P: What I noticed. They put TB and then they put pyridoxin, remember, for peripheral neuropathy they use it. They put in there, and they also put in the cotrimoxazole.

I: Oh okay.

P: But then the ART, no space.

I: No, space for ART?

P: Mmm [yes].

I: Okay. Okay, so in terms of uhh preparing the smart pillbox like, in your opinion, how can we integrate what uhh the DAT programme is bringing together with what you guys are doing, like who can prepare the boxes for the patients in terms of a staff now? Who can do what?

P: Mmm.

I: If you are to integrate-

P: *Yeah* [yes] If we have to do that uhh because I don’t know how long. But for me as the person who’s working in TB, I can do that or we ask one of the assistant nurses, they can also go there.I think the assistant nurse can also do that. Remember?

I: Can also do what?

P: Can also be taught how to use the device. That’s the integration part of it, not just me and sister that I’m working with. There should be somebody to come and do the programme. Uhh remember we cannot put anyone - it should be a health professional. We cannot put somebody like, we have plenty of workers- uhh the cleaners, the clerks and what, what so in case of confidentiality, I think nurses are the ones we can train on this.

I: Nurses

P: Nurses and, and *yeah* [yes] assistant nurse, enrolled nurses they are also nurses.

I: Mmm.

P: They can do this, yes.

I: Okay.

P: When coming to confidentiality part of it, *ya* [yes].

I: Mmm.

P: They can do this. *Ya* [yes].

I: Alright. So, can you please describe to us, what systems are in place that could monitor the differentiated care model and the use of medication device?

P: System?

I: What systems are in place that could monitor this whole programme?

[LOUD]

P: Uhh-not, I’m not clear, but we have a model.

I: Is there any system that you have placed that maybe uhh documenting what the programme is doing if maybe there’s a, there’s anything to document?

P: Mmm no, the document-

I: To monitor the programme if the programme is doing well.

P: Okay. Where’s that, up to far I (…)

I: Do you capture-

P: Capture-

I: Or write challenges that you have with the programme here in the facility?

P: In the-I have uhh what is this uhh , I do write if there’s a problem.

I: Mmm.

P: Uhhh or a challenge. Where is the diary. There’s a diary.

I: You use a diary?

P: *Ya* [yes] we, I use a diary to write and there’s this other book, I don’t know where it is, it’s a notebook anyway.

I: Mmm.

P: Yes, I use that.

I: What do you usually document in that notebook?

P: Names of the patients

I: Mmm.

P: And then their phone numbers, and then what happened after - there’s a book xxx [interviewer name] I (…) what happened after I called them right? Because you call, you need to say I called- you record xxx [interviewer’s name].

I: Mmm.

P: Otherwise, something that is not recorded is not done.

I: Okay

P: And then you, you send the CHWs there, you record. There’s a book, even when, *ya* [yes] because there’s an address there. It’s used by the, by the OTLs we do record.

I: Mmm.

P: Yeah [yes] there’s a recording part of it, and then even these things, remember there are in here but they are still going to the records of the tier.net.

I: Mmm.

P: So, me, I’m using my own thing but the tier.net even the, the auditors when they come, they want to see those uhh *yeah* [yes]- what did you do about your defaulter.

I: So, you-

P: What did you do, yes.

I: So, you give them the book-

P: There’s a book *ya* [yes]

I: That you are writing on?

P: There is also a book, I remember I record even in this blue file that I did call this person, yes. So, we do.

I: Okay.

P: Mmm.

I: So, what are other issues that you are documenting in that book? Is defaulting, and what else do you usually document that is problematic? Issues or challenges.

P: I can’t remember. It’s all, but uhh most of the time it, it uhh even the patient, it’s not only that, even the patients who are supposed to come and collect sputum. *Akere* [right]?

I: Mmm.

P: When they didn’t come, you know very well. I don’t know [inaudible segment] you know very well that these dates, this person was supposed to come and bring sputum.

I: Mmm.

P: And then didn’t bring it.

I: Can you do follow up?

P: Yes. We do follow up. Remember here *akere* [right] there’s no sputum in the device that says, today is your sputum day.

I: *Ya* [yes]

P: So, you record that in that-

I: In that book?

P: Yes

I: Okay

P: But it’s a diary

I: Oh

P: It’s a diary.

I: Alright. Alright, how can the differentiated model of care be sustained, you know the calling, the SMSs, the home visit?

P: Mmm.

I: How can that, those activities be sustained?

P: I think uhh how can I-

I: How can you make sure that they are continuing, they are not stopping?

P: Good communication.

I: Mmm.

P: *Ya* [yes] with each staff. Akere [right]?

I: Mmm.

P: Remember, it a nurse, a patient, uhh CHW, communication sister.

I: Mmm.

P: It should be done uhh amongst us.

I: Mmm.

P: And the relationship amongst nurses and patients. including the CHWs and uhh respect them. It one, not to say that they are community health workers, you just talk to them the way you want. You really, really need to respect them and, and, and make follow up on what they are doing but in a polite manner, you understand?

I: *Yeah* [yes].

P: Saying, *yeah* [yes] wed need to do this uhh , you can’t really, really, really manage uhh TB alone, never. Not even TB only, but you can’t manage alone. But along that, the communication, it’s one of the main thing and respect. Remember other ones, they are not nurses. So, if you really, really don’t care about them, you just treat them like (…) no, no, no.

I: Mmm.

P: It won’t. no, it won’t work.

I: Okay

P: So, you really have to put yourself in their shoes and just come down to mother earth.

I: Mmm.

P: And talk to them like any other human being.

I: Mmm.

P: *Yeah* [yes] then they will do you wonders. Even patients, it’s on the (…) the nurse and patients’ relationship, it’s very important.

I: Mmm okay. So, uhh what are the challenges- can you please describe positive and negative experiences of delivering differentiated care model that the health care workers and those nurses that you work with-what are the challenges-

P: To the nurses?

I: Yes. The nurses that are outside together with the health care (…) together with the community health care workers-

P: Mmm,

I: What challenges do you have when you follow up patient, call them, do home visit, what challenges are you experiencing in doing that, that care, that support?

P: Okay. The only challenge is when I can’t reach them sister.

I: When you can’t reach them?

P: When I can’t reach them.

I: Mmm.

P: Yes, when I can’t reach them and when they gave wrong addresses, when they gave wrong phone numbers of next of kin, when they, when you call and they don’t pick up, it’s a challenge.

I: Mmm.

[LOUD]

P: *Yeah* [yes]. So, yeah [yes] those are the challenges, sister.

I: Mmm.

P: *Yeah* [yes] you don’t really become frustrated; you’ll only go frustrated when they visit. When you call, you send SMS, it okay, you see, no my client will come but at the end of the day when you go there, you find that it’s a wrong address.

I: Mmm.

P: That’s a problem.

I: So, how do you think this differentiated care model that is the calling of the patient, SMSs, and home visit, how do you think can be improved so that it’s more effective?

P: Uhh so, what, we are doing now we (…) when we register our patients-

I: Mmm

P: Remember, these are old files, *ne* [right]?

I: Mmm.

P: And then when you take the new TB file, you compare the addresses, the address that he is giving you with the address on the file, and you compare the phone number that is giving you with the phone number on the file-

I: Which is you compare the information on the file and the information on the gadget on the device?

P: Yes (……) on those three things, this is where you put the address on the blue book.

I: Mmm.

P: The first time the client comes for a visit. So, this is the address that you can see and then this is the address here.

I: Mmm.

P: And then the address that you going to put here.

I: On the phone?

P: On the device now.

I: Mmm.

P: Yes, with the phone numbers here, the phone numbers, and the phone numbers that are here.

I: *Yeah* [yes].

P: These are three registrations. So, you need to compare. Yeah [yes], so that you get the right information in here. So, immediately after getting this, you need to change this, and change this.

I: Mmm.

P: Yes, but sometimes when it gives you this phone, with the phone, with the phone number it’s so simple, you just dial the phone.

I: Mmm.

P: Remember, you just dial the phone if the client has got the phone. So, it’s very simple, but with the address, and if you don’t understand this address, you just give it to the clinic chairperson.

I: Mmm.

P: Then he goes and identify the address.

I: Okay

P: Because some of the addresses, we don’t really know, so now there’s new squatter camps.

I: Mmm.

P: So, it becomes again difficult because they are just put themselves there and there, there are no numbers.

I: Mmm.

P: *Yeah* [yes].

I: Okay.

P: *Yeah* [yes]. So, it still a challenge there. Even though I haven’t had a TB client that is staying on those, *yeah* [yes] but I can see I have a challenge when coming to-

I: To?

P: ART, HIV.

I: What, what is the challenge there?

P: The challenge is, is the addresses.

I: Mmm

P: Yes, so, still the same. It still the same addresses, the phone numbers, these are old files so, with HIV it not the same as 1,2,3.

I: Mmm.

P: It only 1 and 2.

I: Okay.

P: Yes. The device is not there.

I: For HIV?

P: Yes.

I: Okay. Can you describe any gaps which exist in the way the intervention is being delivered currently?

P: I don’t know.

I: What are the gaps, things that you feel like, you know, for the whole smart pillbox, DAT programme-

P: Mmm.

I: What are the gaps that you see in the programme in terms of the way we are implementing.

P: Mmm.

I: This intervention.

P: There are no gaps.

I: Mmm.

P: If you can just fix my, my batteries that would be great, remember we are saying to the client six months.

I: Mmm.

P: *Akere* [right]? Then your battery will be okay for six months, this pillbox will last for three months, the battery currently will go for six months and then it doesn’t go for six months, let say it goes for three weeks and then the battery starts to do its own challenges.

I: Okay

P: *Ya* [yes] so that can be-

I: Are you talking about the battery or the device itself?

P: No. akere [right] it the pillbox?

I: Mmm.

P: *Ne* [right]? So, I think it the device.

I: The device?

P: So, it just that I’m calling it the batteries alone

I: Okay. You talking about the device itself?

P: *Ya* [yes]

I: Alright

P: Mmm.

I: Okay. No, it’s noted. It’s noted

P: Mmm.

I: So-

P: Only the device.

I: The device?

P: *Ya* [yes]

I: Okay.

P: And the (…...) you see, the ARV portion.

I: Okay

P: Mmm.

I: Alright. that’s noted. So, we are at the end of the interview. Is there anything that you think we did not mention that is important or closing remarks?

P: No. I think you touched everything.

I: Mmm.

P: I think you touched everything. Electricity, we cannot change it [laugh] *ya* [yes] but otherwise, our pillboxes are not using electricity, remember.

I: Mmm.

P: *Ya* [yes] only the phones.

I: *Ya* [yes]

P: *Ya* [yes] so, that (……) we cannot charge, but it doesn’t go for long. Remember, we are not using it like-so it doesn’t go off, charging again.

I: Thank you so much for giving us all the information. We really appreciate your time, and we are right at the end of the interview and time for ending the interview it’s (…) it’s 16:03. Thank you.

P: Okay. Thank you.[
