# Supplementary material for: Acceptability of Digital Adherence Technologies to support people with drug-susceptible TB in South Africa
Source: PLoS One. 2025 Sep 24;20(9):e0332103. doi: 10.1371/journal.pone.0332103 (PMC12459780; doi:10.1371/journal.pone.0332103)
Supplement: S4 File — (ZIP) [file pone.0332103.s004.zip › S4 Transcripts/HCWs and Stakeholders/IDI 17-HCW.docx]

**TRANSCRIPTION NOTATIONS**

| **Label Key** | **Meaning** |
| --- | --- |
| **I** | Start of each new utterance by the Interviewer |
| **P** | Start of each new utterance by the Participant |
| **N** | Note taker |
| **{ }** | Indicates that details were changed or pseudonyms were used to anonymise data |
| **( )** | Indicates the description provided to anonymise data |
| **XXX** | Words were omitted to anonymise data |
| **-** | Breaking into a sentence by the next speaker |
| **…** | Pause or drawn out words |
| **[ ]** | Indicates noise made, e.g. [laugh], [sigh], [pause] |
| ? | Beginning of utterance by unidentified speaker or questionable text |
| **[inaudible segment]** | Unclear section of the recording |

I: Huh, thank you very much for agreeing to talk to us today. Can you please allow us to audio record this interview?

P: Yes, you may record me. I allow you to make a record and audio record me.

I: Okay. PID number is: xxxx. Date of the interview: xxxx (interview date). Location: xxx [clinic name]. Type of the interview: healthcare workers interview: Facilitator: xxx [facilitator’s name]. Time of the interview is: 11:42.

I: Okay, sister tell me what is your current position here at the clinic?

P: My current position, I am a staff nurse, a focal person in TB since xxx (year) to date. I am diagnosing patients through the results by Gene expert, and then the other ones that have been diagnosed, are diagnosed by the doctor through the X-rays, the blood, like the ESR and when they check the results, they find the ESR. Then they can diagnose the patient that he or she has TB and then they transfer them to the TB room. Then, I take over, I initiate the patient. Then, I start giving them treatment and collecting sputum, the AFB, the Gene expert like that. So, when the gene expert is positive, we start immediately, then we collect AFB.

I: Mmm.

P: When the X-ray says the patient has TB, we collect Gene expert, so that we can have the resistant or is sensitive to rifampicin, because here in XXX [clinic name] we don't admit patients who are resistant. The patients who are resistant to TB, go to XXX [clinic name].

I: All right, for MDR?

P: For MDR, yes, resistant is MDR, we are not allowed to give them treatment because they will be resistant to maybe Rifampicin… Rifampicin or Isoniazid, either Isoniazid resistant or Rifampicin. We don't -we cannot give them treatment because the treatment we are giving is this one of rifafour, rifafour has rifampicin and Isoniazid. So, most are resistant to Rifampicin or Isoniazid.

I: Mmm.

P: So, hence if it says resistant, they must go to XXX [clinic name].

I: Okay.

P: So that they can give them the correct medication.

I: That’s side?

P: Yes.

I: All right. Okay, thank you so much for that. When it comes to patient care and counselling, what are your roles and responsibilities?

P: Care and counselling, normally here they screen them at the front there *ne (right).*

I: Mmm.

P: There are nursing assistants there, they do the TB screening, and then when they realize the patient is having signs and symptoms of TB. They refer them to my room to collect the sputum, so that we can exclude the TB from the patient, hence now its winter and most of the patients are coughing.

I: Yeah

P: Yes. So, even the-the- the registered nurse at the consulting room usually refer the patient to me to exclude huh, TB on them, and then the first bookings in our facility who are newly diagnosed with HIV. We exclude TB before we can initiate ART, so we don't want them to have the- the IRIS-

I: Mmm.

P: Because if we start giving them HIV treatment before we could exclude TB and then they're just going to have… to be having that IRIS because we started with the ART treatment before excluding the TB. So, hence we… they normally refer them to me to exclude, to test the sputum to know if they are not having TB or not. If they're not having TB. After two days, three days then we can initiate and give ART and continue properly with the patient with the correct medication.

I: Okay.

P: Yes

I: In terms of counselling. Who does counselling?

P: The counselling is done, we have counsellors here,

I: Mmm.

P: Yes, if a patient is newly diagnosed, tested TB *ne* *(right*) tested TB must also be tested on HIV. We must know the HIV status. We must know that the… if a patient is screened, saying he is HIV positive and has signs and symptoms of TB. We must test HIV first, we cannot just say he is having signs and symptoms of TB without knowing the HIV status. If the patient is already on ART, already, maybe two years, three years. We do the test of TB.

I: Mmm.

P: Then if it's positive, we continue we don't stop the ART, but if it's a newly diagnosed, we give treatment after receiving the TB result.

I: Mmm

P: Before, because if you give HIV treatment before excluding the TB. Then it’s a problem.

I: Mmm.

P: The patient will deteriorate and will be very sick.

I: Mmm, okay. I hear that and then how do you monitor treatment adherence *ye (of)* TB, after you initiate. How do you monitor treatment adherence on these TB patients?

P: Huh, no, we were having this file *ne (right)*. The admission file. Then we are having the admission file of TB, even if the patient is HIV negative or positive. Now there were people from xxx (organisation name) they gave us the device, the ASCENT device.

I: Mmm.

P: So it helps us *ne (right)* concurrently with the file. So, we check, we check on the file that the date, the return date must honour the return that date. We give them the first week, the first 2 weeks if the patient is newly diagnosed with ART, we give them the first 2 weeks. We give TB treatment for 2 weeks. Then from there we… we… we… give ART after 2 weeks, it’s 2 weeks to 8 weeks. We usually check the patient; we assess if the patient is not very sick. Then we give after 2 weeks if the patient is very sick, you give after 8 weeks ART. Then we check-we give health education to every patient who gets here, we give health education. We give the side effects of our medication. If he's having this problem. We normally even give that the number of our clinic, if you have experienced a problem like this call the TB team or TB room then they’ll just take the phone to us and then we explain to the patient come to the clinic. Maybe if he has constipation, maybe vomiting like that. And then if the patient doesn't come because we have the books, we open the books for our admission, we normally check the files and co-current with the book. So that this one was supposed to come on this date, this one was supposed to come on this day. And then with the device, it helped us very easily and then we no more going back to the books, going back to the file. We just check on the tablet. You just open that… that tablet, then you can see oh this one was supposed to drink. No, he didn't drink yesterday, the colour of the coding colour or is red, its yellow is like that. So, if he didn't take the treatment it’s going to show the orange, then we call immediately, because we are having the tablet, it has airtime. We call the patient. “Why didn't you take that treatment on this time?” Then patient will , “no, I forgot,” then immediately take the treatment on the spot. With the device, it’s very easy. It's very quickly and then like going to the file- to that book of mine checking it’s a long process.

I: Mmm.

P: Hence, the file stays at the filing room. Imagine when I go to the filing room taking all the files coming here like auditing them, opening it one by one and in XXX [Clinic name] we are having about 100 and something TB patients, it’s a lot, imagine I'm alone, checking the files, helping the patient, you know, otherwise I cannot reach them all but with the device and the tablet, it’s very easy. You just open, and just scroll check- check this one took, this one took. Then it’s fine

I: Okay.

P: You just open, and just scroll check- check this one took, this one took. Then it’s fine

I: Okay, so if you're comparing before, we came with a device and now, is there a difference in terms of monitoring your patients?

P: Yes. It is a big difference.

I: Mmm.

P: And it helped me, you don't have to stress too much. Hence, I was saying I must go to the filing room to retrieve the files, come with the file, sit here with hundred and something files, opening, checking my-my- my- my books that I wrote so that it can be easy. Now the patient is here, I leave to attend the patient. Huh, its trauma, its trauma and it doesn't make my work very easy, but with the tablet, I just scroll open very quickly, check Oh, call, “why didn't you take your tablet? Where are you,” and wait for the patient to explain, “no, I’m in town. I was going to, then I went wherever and then say I will take them immediately when I get home.” We check tomorrow and find that patient took the medication.

I: Mmm that’s nice to hear. So, how are TB services delivered at you’re … at your level with regards to the digital attendance technologies. So, with regards to adherence, I mean to the technology. What exactly are you doing? Can you list the things that you are doing in the Ascent project?

P: With the device?

I: What are you doing? What are you specifically focusing on? The things that you do.

P: On the devices, when the patient comes first time *ne (right)*, we open… we open the file. When we open the file. There's the lady who's helping me here. Then she registers the patient on the tablet, she gives the patient the consent form to sign, to tell him or her that we are given this device and then we explain how it works, how easily it works and how easily it is going to help her or him not to forget to take the treatment.

I: Mmm.

P: And we are also able to monitor and help her to remind her when taking the medication.

I: Okay.

P: So, it's very, very easy. We can monitor the patient, we can… we can register the patient very quickly; it doesn't take even 10 minutes.

I: Mmm.

P: We do it very quickly. And then when the patient is happy and go home, because they don't like to stay here for the whole day. You'll find the patient is staying here for four hours. So, it’s stresses, you know. The patient maybe is coughing and is still here and also myself. I don't want the patient to stay in one room for more than four hours coughing here and having TB-

I: Mmm.

P: And struggling, so the device is helping us really-

I: Yeah.

P: And then when we explain to the patient, we teach the patient that there are lights. The orange, the red, the red is showing that the battery is getting… it must be charged. So, if it's red, you bring the device, so that we can change the battery for you. And then we educate them that the battery lasts for six- six months. And then when it's orange you didn't take your medication, when its red, when it rings you take your medication, it’s a user-friendly device.

I: Mmm

P: it’s very- very easy like now we are using the technology, and everything is easier that way.

I: Okay.

P: We tell them we monitor them, checking them through it. Everyone now is adhering as I speak. Yesterday, I went to tier and check how many are lost to follow, how many missed, early, and late missed. You know, you can’t believe I'm at 00, they are complying. They are definitely complying. So, it's very nice. This device is working.

I: That’s good to hear. So, how long do you take to register a patient? I heard you earlier on you said it's not even taking a lot, for you as a nurse. How long do you take to register a patient on the platform?

P: On the platform?

I: Mmm.

P: On the file *ne (right)* it can be 30 minutes.

I: If you are using the device?

P: If I’m using the device, it’s 10 minutes.

I: Okay.

P: To to- to- to I'm finish

I: You’re done, is it easy to register a patient or it's-

P: It's very easy. We just ask the patient, where do you stay, and you put the name, you put everything the address and everything, it’s very quick even to check again, you go back and you check, it’s very easy.

I: When you’re checking what?

P: When I'm checking if maybe they didn't take the medication.

I: Okay.

P: Yes, when I check why didn't you take the medication? Maybe they're like this yellow when I open it again. I go and I can retrieve all the patients that I have on the device. One by one, I check, I open.

I: Mmm.

P: So ,it is easy-

I: It’s easy, it’s not difficult?

P: It’s not difficult. Not difficult. You can understand it, like when you're having your cell phone. You can understand, it is even easier.

I: Okay, thank you so much for all the information that you have given me in relation to the…the activities that you do in the project. So, if you have to explain huh, what is digital adherence technology intervention to another healthcare worker who doesn't know about it? What would you tell them?

P: Huh, I will tell them this device *ne (right)*,

I: Mmm.

P: it’s a tracking device of a patient who's taking TB treatment. We know that TB- TB is a -is a communicable disease.

I: Mmm.

P: It’s a tracking device of a patient who's taking TB treatment. We know that TB is a communicable disease. If there was one patient who doesn't take treatment. It can affect other patients. From one, it can be 1000s and 1000s. So, with this device [box], we can track the patient that is taking the medication at the right time, he’s taking it often. He doesn't miss the doses and then we can even see the progress of the patients, when the patient comes to us, we see that the patient is really taking medication, that is the most thing… most important thing, because if the patient doesn't take the medication, he’s going to infect other people in the community. And then we'll end up having 1000s and 1000s-

I: Mmm,

P: Hence, here I'm having about 100 patients in-in-in my facility. So, you'll find 100 when you can multiply those 10 that didn't take medication, it will be how many 1000s. So, with the device it’s very, very easy.

I: Mmm,

P: And then we monitor them and then we make sure they take treatment. If you see that the patient didn't take medication today. Now we call, he takes the medication, he’s not going to infect the household people so we are now saving the community.

I: Mmm.

P: Yes

I: Okay.

P: And then it's easy, the most important thing is that its easy to use. Unlike writing - on those files, going to retrieve the files in the filing room, coming checking one by one by one. It’s stressing, it’s stressing.

I: Mmm.

P: It’s stressing, if we could even maybe not use those files and use the device just enter the information of the patient. The address, the name, the surname, everything, the phone number, and then check it on the devices it was going to be easy. Just take that information to tier. So, it was going to help us and save time, because the patient is staying… is sitting here with me- is with me and then they just want help and go home.

I: Mmm.

P: Then we'll minimize the time. We'll be minimizing the time the patient is in the facility. Hence, we saying TB patients must not stay for a long time in the facility, we must give treatment and go, and the queue must be fast for the patient. So, it was going to help us.

I: Okay.

P: Maybe we no more use these files writing. We could maybe write, if maybe this device could have that. And maybe where we could write and say admission on the patient date is this and then how is the patient, maybe we could add that portion on the tablet.

I: Mmm.

P: It was going to be user friendly.

I: We have that part of the notes.

P: We have yes, we have and then we write, no more using this writing thing.

I: On the files. All right, that's good to hear. So, you're mentioning that you know you have a way of checking, you open your device and check the people that are adhering and those who are not adhering, how do you call that part where you are checking?

P: Huh, that part is… Is I forgot, what… I forgot that part mmm, let me open this ……

I: How do you call it?

P: the- the- the- when we open when we check the patient?

I: Mmm

P: On to the tablet *ne (right)?*

I: Mmm

P: It’s xxxx (adherence platform), it’s the app, you just go to the app. xxxx (adherence platform), and then you get the patient inside.

I: Okay.

P: Yes.

I: All right. So, those are the things that you will tell the other… The other nurse, somebody who doesn't know about it. That this is an app that we're using. This is where we register the patient.

P: Mmm

I: Okay, please describe your role with differentiated model of care Intervention, what exactly are you doing when it comes to differentiated model of care?

P: When I- I- I differentiate, the call with the device and the file or-

I: Yes, with the device, differentiated care are those-

P: Care of the patient?

I: Follow up.

P: The follow up?

I: Yes, what exactly are you doing when it comes to follow up?

P: Yes, every- every, every morning neh(right).

I: Yes

P: Every morning we open our tablets, our device, our tablet, so that we check how many today, how many maybe missed, but normally we check every morning, every morning, we open it, then we go to the current patient, then we check. Then, it gives us their names and everything and then we check. So, this one did take their medication, this one didn't take, then we then call them immediately. We don't waste time because if we waste time, it means if the patient was supposed to drink, isn't it we get to work at seven o'clock, from 7 o'clock, we check if others are taking at 6, then we check which one at 6 didn’t take medication, then we remind them to take medication and then avoid miss taking their table

I: Okay, so you're telling me that every day in the morning you check and see those who are adhering and those who are not adhering, you call the ones that are not adhering. After then, sometimes you do home visits right?

P: Yes, we do. The home visit we do it, we don't do it like if maybe the patient they didn't take today. Then tomorrow we go no. We give the patient a chance. We're saying okay, we monitor, we check we monitor that adherence of the patient. If it’s now exceeding from 3 days then we go, we send the community healthcare worker. We have community healthcare workers, then we send them. We call the community healthcare worker ,”we say go and check this patient.” Their number, the address is this, this, this.

I: Okay.

P: We call the patient first. We call, then we call, then we call and then if it’s 3 days, this patient doesn't respond, doesn't adhere to medication. Then we send the community health care worker-

I: Okay.

P:To go to the household to check what is a problem, why is he not taking his medication? Because the device is ringing, its alerting the alarm is- is alerting him or her to take the medication.

I: Okay.

P: Yes.

I: Alright and then huh, I understand that you wake up every day in the morning to check the adherence, if you see non- adherence, you call, if it's more than 3 days you do a home visit. Give me an idea of how are you sharing these responsibilities, the calling, the checking of the adherence and home visit, who does what?

P: Yes, me I'm checking here with the tablet *ne (right)*

I: Mmm.

P: I'm checking all the patients, then I call for 3 days, I call, I call and then when the patient is not responding, then I send the community health care worker for the different area and we having the addresses, who's checking maybe xxx (area name), who’s checking maybe xxxx (area name) like that.

I: Mmm.

P: Then, I call. I call the healthcare worker to go there and check and we also have the sisters who are working with the healthcare workers.

I: Mmm.

P: I'm checking all the patients, then I call for 3 days, I call, I call and then when the patient is not responding, then I send the community health care worker for the different area and we having the addresses, who's checking maybe xxx (area name), who’s checking maybe xxx (area name) like that. I call the healthcare worker to go there and check and we also have the sisters [TB Nurses] who are working with the healthcare workers.

I: Okay.

P: Yes.

I: So, what are their experiences these community healthcare workers and nurses, when they are coming to give you feedback, what exactly are the challenges of the patient? why patients are not taking treatment using the boxes, what do they tell you?

P: Uhh ,it’s a lot of challenges. A lot of challenges XXX [facilitator’s name] when you get into the household, maybe they will tell you they found that household. Maybe three days, they went without food, there was no food. The patient is complaining, “I don't have food and I couldn't take treatment, because I was hungry. I couldn't drink them on an empty stomach.” And they say even the environment you would see they are not having anything to eat. And that’s a challenge, the most challenging thing is that one.

I: Of patients not having food?

P: Yes, the nutrition, they don't have anything to eat, they’re not working, there's no one who’s working in the household.

I: Mmm.

P: And then the patient says, they couldn't take treatment, because they were hungry and when they take the treatment without anything to eat they will complain that they start having the abdominal cramps and what- what.

I: Mmm.

P: You know. So, the challenge is that, then someone saying, “no I'm staying at xxx (area name), from xxx (area name) to here. They must take their public transport.

P: Maybe it's R15, the public to come here. R15 and R15, It’s R30 within XXX [Clinic name] yes. but they must catch a taxi to come. Then, others say, “I didn't have money to come, it’s R30,I didn't have R30.” And now the treatment will be finished and you see the patient didn't comply. That's a problem.

I: Mmm.

P: Otherwise we try. We try to ask the food from the social welfare. We try to give those letters to go to SASSA to ask for the grant. But not everyone is given that grant, it is granted by SASSA temporarily that she's now taking TB treatment, then they say most people don't take treatment because they know they are going to be granted TB temporary grant.

I: Mmm.

P: Before they could just give, but now they need… they go through the file and before they can give that grant, it's not everyone who receives it.

I: Mmm.

P: Now it’s a challenge for them to take treatment without food.

I: Yeah.

P: Yes.

I: What about the food parcel?

P: The food parcels we asked the Social Welfare, we send them to XXX [Location] we take them… we have social workers this side. We take the patient to the social workers and explain the situation, the patient has no food at home, and now has TB, maybe even has HIV. You say now the pill burden, taking TB treatment in the morning, in the evening they taking ART treatment without food. Then, they organize for them to get the social welfare to come and give them the food parcel, I’m having the other lady with a child. They could stay maybe a week without food and everything, but now we assisted them. They are getting SASSA grant. At first, they were getting those parcels-

I: Food parcels?

P: Yes, food parcels, because they were not taking treatment at all, saying that treatment makes them very sick, because they take treatment, but they don't have anything to eat. So, the mother was very, very skinny. You'd see their skin, you see malnutrition on the child, the mother. Mmm when you look at them, you'll see really, they needed an intervention, they needed someone to help. But now we are happy, they are complying now, they are taking treatment correctly. And even the weight, the weight gain, you will see the weight gain, even their skin, you can see they are eating properly.

I: All right. That’s good to hear and then so when you first heard about digital adherence technologies, what were your expectations before it was implemented. What -what were you thinking about this strategy?

P: No, I just told myself let us see if it's going to work or not. Because you see with TB it’s a challenge, because if someone doesn't take treatment within six months, complete the course of six months, it’s a problem. Those patients will come back now with MDR, now the resistance of treatment because it went back and then we come we re-initiate the patient, one patient has four files, defaulting coming back, defaulting coming back. I just told myself let us try this one, because when they explained to us, when they taught us, then we went for workshop and then they showed us then I just said will this device will make a difference?

I: Mmm.

P: But when we implemented, it’s then when we see this thing is working and it makes it better you know, if we just tell him this device works like this and when you don't take medication we could we could see you, then is now saying, “oh let me take my medication because they see me,” (laugh) just like that and they do that, they take, you see even when they receive the messages saying, “you didn't take the tablet yesterday, you didn't take the medication the day before.” They even come to me and say, “sister, those people up there they are telling me that… they send me messages, that I didn't take medicine.” And I say yes, you see, they see you. I normally tell them and even say XXX (President’s name) can see you know, they'll come with the police and take you because you infect other people, now they start complying.

I: Okay (laugh).

P: They start complying (laugh), so it needs attachment, they even form an attachment to the sister?

I: Mmm.

P: When you are polite to them you explain to them they will cooperate they are cooperating very nice. And indeed, they think you-you see them, because if they just testing in the morning, he or she was supposed to take treatment at 7 now it’s 8 o'clock you are calling, why you didn't take treatment. “Huh, sister I forgot. I was busy.” And I say, “huh, huh, take treatment,” he or she is never going to miss the treatment the following day because they know that you see them, but you are just checking on the device, huh, these patients, but now really, I am happy. I am more than happy.

I: Ne *(Right*)

P: If we could have these devices, if we could run with these devices forever. We will never- never have the lost to follow. You'll never have the lost to follow, imagine I didn't take treatment 8 o'clock, 30 minutes sister is calling, you didn’t take treatment.

I: Mmm.

P: So, you'll never have-

I: Why are you feeling like we will never have lost to follow?

P: It’s because we call *akere (right)*. We are checking, we open the App and check the patient oh this one didn't take, we call him and then even 3 days, we send the health care workers with their sisters to the household. So, how can we have the lost to follow?

I: Okay.

P: Definitely, we won't have lost to follow, definitely.

I: Mmm.

P: And it's easy unlike going to the file retrieving. You will find that I was supposed to work from 7 to 4, it’s 16 hours,

I: Mmm.

P: Then we forgot- we forgot to check the other files. We checked, maybe 10 files, I'm having 100 files. You see. I didn't reach that 90. But with the device, I could just check quickly, helping the patient, calling, sending the messages to the sisters, go fetch this patient. The patient comes after 3 days before defaulting-

I: Mmm.

P: Because when the patient defaults, it’s 2 months for the patient saying his defaulted treatment.

I: 2 months?

P: Yes, is 2 months? But now if the patient complies 3 days, we can catch up with that 3 days add the treatment for 3 days again, it's easier.

I: Okay.

P: It's very easy but with checking from the files, like manual like one file to another file to another file to another file it’s stressing and here you find and I'm not just sitting like with the files I'm having patients every day, Monday to Friday I'm having patients that I see. Sometimes you have a patient that needs to be attended to the casualty.

I: Mmm.

P: Yes, a critical patient, very Ill-patient, but others are waiting for you. Then, you miss that date to check the file, when you check that this patient didn't take treatment, you find it’s now 2 months. You missed it.

I: Mmm. So, you are telling *ukuthi* (*that*) this device can improve lost to follow. What is causing lost to follow from your perspective? What is causing lost to follow in the facility?

P: The way I was telling you (door knock) the lost to follow in our facility ne (right) are caused by the way I was explaining to you, that others are having problem with money, the others are having problems with food, and they don't have food at home and then you'll find that the patients say I couldn’t walk in that area I'm staying, because I didn't have energy. I didn't eat. I didn’t have anything to eat for three days- four days. So, I couldn't even walk and come to the clinic, you know.

I: Mmm.

P: And here I have the other challenge with these boys who are smoking the Nyaope substance (type of illicit drug). Huh, who are under nyaope substance are a problem because those ones you give treatment and then you say take treatment, we give them the boxes. Huh, you see today he’s taking, tomorrow he doesn't take, two days he doesn't take, mmm. It’s a problem and they are a lot here in XXX [clinic name].

I: Mmm

P: Then, I think those nyaope’s now. Most of them here in XXX [clinic name] are lost to follow, if you go to the household the client is not there. When you ask the parents or the people, the siblings who are staying there, they say this one doesn't stay here. He just goes out smoking nyaope and doing whatever that he’s doing and that the drugs are a problem. And even the boxes, you find today they open three days he doesn't open, even when now you tried to call, you don't find him, you find maybe the mother or the father, but you can’t reach the client and even the community health care workers and other sisters [nurses] who go and check them. They don't find him. Mmm, it’s a struggle.

I: Mmm

P: I think this nyaope (drug substance) is also contributing to the lost to follow.

I: Okay, alright.

P: If the patient is not on nyaope, most it’s the food, the transport for coming to the clinic.

I: Okay, but in terms of monitoring the nyaope patient, how do you feel? *Ukuthi* (that) you are able to check even though they are not reachable, they're not at home. But how do you feel that at least when you open up the platform you can see *ukuthi* (*that*) today he has taken you know, they only missed 2 days but 4 days he has been taking treatment even though it’s difficult to get to know where are they, how do you feel about that?

P: Huh, it’s a problem *akere* (*right)* TB doesn't end up here with me only, or in our facility only, you know, This is a problem because it's contagious. It can pass from one patient to another. Let me give you the example, If this patient is a nyaope, they smoke, when he smokes and doesn't take the treatment. He gives his friend whatever they are smoking. Then immediately from his mouth to the other one, he’s passing TB from that one, maybe they are group of 6, one client is passing TB to another 5, now they are going to be 6.

I: Mmm.

P: So, with nyaope it’s a struggle.

I: Mmm.

P: And even now, we are trying to send the social worker, so that they can try and find them… Take them to rehab so that they can help them because eish with nyaope, it’s a problem.

I: Mmm what else is the problem with the nyaope group?

P: The nyaope group, sometimes maybe they forget to return the boxes. They even forget to take treatment because of those nyaope drug after taking it, they say they sleep. Hence, you find the person sleeping like this, like sleeping, sleeping. And then when he wakes, up it's late. The clinic is closed, you see now, huh, tomorrow again because they say they smoke three times in the morning during the day and when they go to sleep. They say it must be three times.

I: *Yoh.*

P: If they don't take it three times, they become sick and now we give them boxes like now to monitor them, to see the progress to see that they are taking medication. Huh, when you go to them, when you try to find them maybe it's now lost to follow, you want the box, and they don’t even know where the box is. When you ask the household, they say no, the box was here, he took it, and we don't know where he took the box, and then you don't find him or her. So, I don't know now if these boxes we give to all patients or… us only we give to all patients ,yes. But if we would… I don't know how we will do it. I don't know but if we can take this box and give to another one another one after 6 months we give to the other one these devices would help us.

I: Mmm.

P: Yeah, now if we have 10 Boxes I'm giving an example. We have 10 boxes. We gave four boxes to the Nyaope users. Now we are running short of four, because they didn't bring them back, now we could only give to 6,

I: Mmm

P: We are going to struggle again.

I: So, all of the nyaope people did not bring them back?

P: Not all of them, .

I: Okay. Some they bring them back.

P: Some they bring, only those you will find are not taking medication. We check the adherence is poor, poor adherence, will go to the household and we don’t even find that person.

I: Mmm. So, you mentioned earlier on that your expectations about the DAT were that it’s going to make things better. And were your expectations met?

P: Exactly huh. Mmm, I am happy. I am more than happy.

I: Mmm.

P: I would wish those boxes to stay with me, because as I told you earlier. I tell you now, I don't have lost to follow.

I: Mmm.

P: I don't, even those nyoape’s. I don't have lost to follow even those on the nyaope right now in this month. I don't have any missed. I don't have late missed. I went to tier and check my things yesterday. I am on 100%. I can rate myself 101%. Huh, it’s very nice.

I: Mmm.

P: You know all of my patients adhere. They are adhering

I: Mmm.

P: When they come to collect medication; they bring the boxes. They open, they put the medication. I ask them every time when they come, they come every month. I ask them how many tablets you take, How do you take it, how often… how often, they just tell me in the morning. “I take my medicine, the alarm rings. I open, I take medication, it’s easy sister, I don't forget, because this alarm is ringing, it’s alarming, it’s an alarm, it’s telling me to take treatment on time all the time.” So, I say, “oh, it's easy, yes.” “Even if maybe I'm busy, the alarm rings and say its time. I don't forget. I take my medicine.”

I: Very good to hear that. So, can you please describe the training and the resources that staff received on delivery of the DAT including differentiated care? How was the training that you received?

P: About the device?

I: Yeah. How to implement the-

P: The training was very good, because when we came, we could implement it [door knock] [pause].

I: Yeah, so you were still telling me that the training was very easy, because you managed to-

P: Yes, the training… The way they explained to us at the training *ne (right)*

I: Mmm.

P: it was easy to come back and implement, because they told us what to do and everything and then they started teaching us with the boxes, how to put the patient on the tablet. How to know if the box is having a problem. Those lights, the yellow, the green, the red, everything and then how to retrieve, how to check if the patient is taking medication or not.

I: Mmm.

P: Yes.

I: Okay, how were you trained? When you trained.

P: We were trained at the…We were called *ne (right),*

I: Mmm.

P: And then we were trained. I think it's XXX [training location]. Yes. Then we were different clinics. At the time,

I: Mmm.

P: We were different clinics. Then we went there with the auxiliary nurse, the staff nurse, the PN, the health care worker,

I: Mmm

P: It’s a group. We cannot run TB program alone if you are a PN, if you are professional nurse alone, you need to have a community healthcare worker. You need to have an assistant nurse, you need to have the- the –the- people who are helping you with the program.

I: Mmm

P: Because if we have patients who are not adhering, you need to send someone like the healthcare worker.

I: Okay.

P: Yes. And then the assistant nurse can help you to retrieve the patient, to see if he’s taking treatment or it’s a loss to follow like that, but now with the tablet, the app of DAT device it’s very easy, it's very easy.

I: Okay. All right. And then what suggestions do you have for the training for us to improve these trainings. What can we do to improve the training?

P: Improve the training for now?

I: What else can we do to improve the training?

P: No, nothing for now with me-

I: Mmm.

P: The way they explained to us, the way us implementing it, the way we are running it for now, it’s fine. Its fine,

I: Mmm.

P: I don't see any loopholes. I don't see anything to add up, for now its running perfect.

I: Okay.

P: Yes. I don't think we can add something, because the way we are running it here, it’s working for us.

I: Okay.

P: Yes, we don't have any challenges so far.

I: Mmm.

P: Yes, it’s running perfectly.

I: Okay. In terms of the rotation of the nurses?

P: The rotation of the nurses, like I am from the training-

I: Mmm.

P: The rotation of the nurses, like I am from the training I came back and trained the others, so that they must do exactly what I'm doing. Because we cannot … they cannot we … we cannot be trained every time and every time calling different maybe the DAT team, calling everyone every month no, it’s not going to work. But when I came back, I -I explained to the facility, the whole facility, where every morning, we go at the maternity that side. Then we give the challenges when you’re from training. They will ask you what you learnt. What did you do, then you teach and give a lesson to others.

P: But specifically, I have the ones that I taught. I have two professional nurses that I taught about the devices about

I: Okay, we can continue from your perspective as a healthcare worker. Can you please describe the… Can you please describe the benefits of implementing of using this DAT? What are the benefits?

P: Huh, the benefits *ne (right)?*

I: From your side?

P: The benefit it’s saving me time. Number one. I don’t have lost to follows. I don’t have people who are not adhering right now. All my clients are adhering. I don't have lost to follow and I maintain time. I help people. I totally give them nursing care,

I: Mmm.

P: Because I would stay with a patient complaining of maybe huh, huh, pain or what I could even do totally nursing care to the patient, because of the limited time that I'm having using the device. So, it benefited me perfectly, especially lost to follows, because they'll go out not taking medication and infecting the community.

I: Mmm.

P: Mmm. So, I am happy, and I just need these devices to stay just to help us not to have those loss to follow, you know, lost to follow is a problem. The patient will come back very sick, very ill. Now you start them from zero, you start giving them, you start initiating, and it’s a lot of money again because you collect again sputum. It’s like you starting a new patient. You collect sputum from scratch.

I: Mmm.

P: You continue with it like that, and opening again the file, writing those files and it’s a lot of paperwork, huh, it’s a lot of paperwork.

I: Mmm, But there's the digital adherence technology-

P: Huh, the DAT machine huh. The device is very, very easy. It's very easy and then the way I like it, you can see now, not tomorrow when you open it when you check the current patient, and you check them you see this patient was supposed to take treatment at 7, they didn't take, and you call the patient unlike the files. The device is working for us.

I: Mmm.

P: It’s working for us.

I: Okay.

P: It’s a user-friendly device. Since I worked in TB since 2013. When I received this device mmm, I was so happy. When I see now, when I check now. I can even say it worked for me.

I: Mmm-mmm.

P: The improvement in our facility. Success rate went from maybe 70 something percent, now I think that we on 100%. Because of this DAT device.

I: DAT.

P: I mean DAT device, yes.

I: All right. Okay, so in terms of differentiated care, which is the support action that we give the patient, how is it beneficial to you?

P: To me?

I: Yes, from your perspective, what are the benefits of doing differentiated care? that is extra support that we give patients?

P: Huh, with the patient and the device,

I: Mmm.

P: They won’t miss the- the- the- the treatment, it improves the patient, the progression of the patient, and they will be cured, because of this DAT device,

I: DAT.

P: DAT *ne (right).*

I: Mmm

P: ASCENT device is going to improve them. They are going to complete their treatment because of the help of the device. The alarm is reminding them,

I: Mmm

P: Yes, and then the improvement is going to be a great success.

I: Okay.

P: Yes

I: Is it easy to implement the differentiated care model, that is calling, home visit, reminding the patients-

P: It’s very easy. The device… the tablet has airtime akere (right) we can call immediately, like here in our facility, we have one cell phone, working on the area, we having a baby clinic we having a maternity, we having PHC, we having TB. We having repro. That phone is like a cell phone. One is taking it that side, when you want to call. You see the patient didn't come for a week. When you want to call the patient, then a patient comes in you have to attend the patient then you forgot about calling. You open the file again. Maybe I'm not having this device, I'm not having the tablet. Then when you say… when you remember oh the phone is in casualty and when you check the time its past 4, you are going home, but with the device it’s very easy, you call and then you monitor. You call them immediately, when you check on the tablet. You check then you see oh two missed treatment. Immediately in the morning, you call them and then you remind them and then you talk to them and say "don't do that because TB, you take treatment every day on the same time".

I: Mmm.

P: Yes.

I: Alright, so that's nice to hear that it makes life easy for you. It's easy. The tablet is always here with you, rather than using that other phone that is used by other nurses and you always have the tablet with you to prioritize TB patients. And then mmm I want to hear now about challenges of using this technology. I heard about benefits. Can I… can you tell me more about challenges that you came across within during the implementation?

P: Huh, challenges I would say the flat battery. We had some few clients bringing back the device, saying huh, the battery is finished. But it's not that strong of a challenge, because we just change the other battery and we put the other battery, then they go home.

I: Okay.

P: My worry is that some complain about that the fair, the transport because maybe he was supposed to come on… on the 30th of July. Now today is the 20^th^, but he came earlier because the battery is flat. He came and changed the battery, that’s the only challenge, I think.

I: Okay, is it the battery or device they are complaining about?

P: The battery from the device is flat,

I: Okay.

P: Yes.

I: Is it the device or the battery they are complaining about?

P: The battery from the device.

I: Okay.

P: *Akere (right)* some devices, the ASCENT is having the battery inside. We have those three lights when the battery is flat, it’s showing red.

I: Okay.

P: Now we taught them when the battery is flat, you'll see the red light, saying the battery is flat, immediately bring back the device to us so that we can change the battery, so that you can take your treatment properly and then it’s on the green light so that you can take it.

I: Okay, earlier on when we were talking about the training. You mentioned that you can be able to train other nurses?

P: Yes.

I: Is it possible that a nurses can train other nurses about the ASCENT?

P: Yes

I: Yes. Is it something that you can do as a nurse?

P: It’s very easy, As long as I went to the training and they have properly trained me. It was easy because I knew what to do. And then they trained me properly they showed me how the device works, how to take out the battery, how to put on the battery in the device, how to close it, how to open it, how those three lights work. How the tablet works with the device, how to enter the patient in the device, in the tablet, so that you can check if the patient is medication, how he's taking medication, how he's complying the compliance.

I: So you can train?

P: I can train- I can train them

I: Alright. So, in relation … as I was still asking you about the challenges or negative experience of having these boxes, these smart pillboxes [door knock] [pause] I still want to know the challenges in relation to stigma, any challenge that is related to stigma that you've experienced?

P: Mmm, no.

I: Patients are fine just to have the box?

P: They are happy

I: Okay.

P: You even see them when they come with those boxes, we didn't have any stigma, and they enjoy the boxes. Huh, they enjoy them. You see if someone is happy with something saying this is working for me. They even see when the battery is flat. When they see the light is now red. They come and say sister I came here I need help with the battery. They even understand. They even know the coding of colours. They know red is saying the battery now is getting finished, the orange is saying I didn't take medicine. They even explain to you. You see the patient understood and understand the devices.

I: Alright.

P: So, we are happy we are happy. We didn't have any negativity saying oh these boxes of yours is doing what- what, maybe it's making noise. They even saying huh you cannot even miss because this alarm helps you. You just go and take your medicine and close it when you fine. They're not even complaining of the alarm, or anything, no.

I: Okay.

P: They’re happy.

I: All right from your perspective as a healthcare worker, can TB treatment be improved by using this technology?

P: Huh, it improved our facility, here it improved. It's improved very- very much from the percent that we were before, now it escalated.

I: Adherence?

P: Adherence, as I told you yes. We are now not having lost to follows, early missed, late missed. We are on track, I think it’s this device.

I: Okay. Please elaborate on the positive changes that the differentiated care and the smart pill box brought into the facility. When it comes to TB management. What are the strong positive changes that you can say this technology brought to my patients?

P: Huh, it brought smiles (laugh). We don't have lost to follows. That is my strong case.

I: Mmm

P: You know lost to follows will lead you to having a lot of patients out there. Other one infecting the other. We don't have lost to follow,

I: Mmm.

P: Cure rate, our cure rate is up.

I: Mmm, okay.

P: Imagine if you’re having 20 patients, the adherence is on the dot. They come on the date given, they take treatment. There’s an improvement, even the patients are now totally improved, they’re cured, they completed treatment, and you don't have lost to follow.

I: Mmm.

P: So, this is what we need in our facility.

I: Okay.

P: We need to have patients who are cured, who completed treatment.

I: Mmm.

P: Yes.

I: In terms of your relationship with the patient, how has it improved your relationship with the patients?

P: it’s very, very nice. They like it when they come, you’ll see someone having their device putting it on your table, opening it. Saying, “sister” … you see others even come with the empty containers inside (laugh) saying, “you see, I finished all of them inside the box.” And I say it's fine. You can even throw the empty containers; you don’t have to come in show me

I: The box?

P: Mmm the box, they just come in with it. “Come sister, you see, I finished all of it.”

I: Mmm

P: And the box reminds me the following day. The others say the box reminds them today so that tomorrow they can come to the clinic. They're saying this box is working nice. It even reminds me of the date for collection.

I: The follow up date

P: Yes, the follow up date, then they come.

I: Mmm how can all these changes… positive changes that you see can be sustained? How can we sustain these changes?

P: Of the patient? If we keep the boxes, yes it’s going to sustain us and then it’s going to help the patient, you know sometimes you forget you having a card ne

I: Mmm

P: We wrote the date on the card and then put the card in the drawer or wherever in the house. But with the device shows yellow light to say tomorrow you go for collection.

I: Mmm

P: They know, so with the box they won’t make mistakes. They are reminded easily unlike the card, that we are giving, that green card, yes we wrote the date to return to care, but if they don't come, they forgot because the card is put maybe in the drawer wherever, but with the box it reminds them to collect. They always tell me huh you know if it wasn't this box. Huh, I was having a problem, I was having stress, but the box reminded me. So, tomorrow is for the follow up.

I: Okay, all right that's good to hear all of that. So, please describe the negative, the negative experiences or negative changes that the DAT program brought in your patients, anything negative?

P: The negative?

I: Mmm, we spoke about the positive changes. What about the negative changes?

P: The negative huh, huh I don’t have a negative (laugh),

I: What are the negative experiences that patients or maybe you even you as a healthcare worker, anything negative?

P: No. I can't remember anything negative *aus*i *(sister)* XXX (facilitator’s name)

I: Alright.

P: I don't remember anything. It's only the positive.

I: Mmm.

P: It's only the positive. You know when the clients come, they smile. They’re happy. They didn't miss the date. They didn't miss the treatment. The adherence they are on point. They took all the medicine at the right time at the right date. And even they are on the right date. \

I: Mmm.

P: They come with a smile every time. So, I cannot say anything with the device that is negative now.

I: So, huh sometimes according to differentiated care, we have to call patients, you know do that follow up if we see non- adherence, home visits. What are the challenges on that on that side?

P: Huh, on that one of the home visit-

I: The calling-

P: The others are naughty, there are those naughty patients. Maybe staying at … I’m giving an example here in XXX [clinic name] we must only admit the patients who are staying in XXX [clinic name] and initiate only the patients who are staying in XXX [clinic name ]so that if the patient, missed the treatment, or didn't take treatment, we can go to the household and check the patient. But the others are staying in XXX [far location] is our neighbouring area in XXX [far location]they come here and say they stay in XXX [clinic name] they walked and see the address and the house. They just say I’m staying in xxxx (area name) , this number what- what and when you go to xxx (area name), they know that they don't know that patient, they’ve never seen the patient. They gave us the wrong address and wrong telephone. That is where we encounter a problem but not with a device, but with a client giving us the wrong telephone number, the wrong address.

I: Mmm, mmm okay. So, tell me what system is in place that could- that could monitor the differentiated care and the use of these technologies in the facility. How do you monitor this technology? Is there anything that you are doing to monitor?

P: Yes, we give the patient the device. Then we teach the patient how to use the device. We even educate to take care of the device and to put it on a safe place. Not where the children can reach, they must put it very safe where the patient cannot reach,

I: Mmm.

P: Because the patient can take medicine inside the device and drink it. So, we even educate of the risk of putting nearby, because if the alarm rings, the child will want to check what is ringing and then you find the device is ringing and then you just open huh, there’s medicine this tablet, the patient will just take the medicine and drink. We educate them to put it in a safe place and then where they could reach, but not the children, away from the children. So, the device, we teach them after 6 months, they must bring the device so that we could help other… others, so that they can benefit, have the benefit that the other one had to take the treatment properly.

I: Okay. Do you write somewhere? Is there a document where you documenting all these?

P: No, we don't write them.

I: Okay.

P: We just teach them and then we just write the- the- the consent, we give them the consent.

I: Okay All right. So, just think about … about it like this without xxx (organisation name) support. That is the intern support and the support that we're giving you to implement the strategy. Will you be able to still implement the ASCENT project even without the xxxx (organisation name) support? Can you do it all alone as a facility?

P: We can do it, but it’s nicer with an xxx (organisation name) person because they are the ones who came with this device. I think it would be nicer if I had the help of the… like now I'm having that lady who is helping me with the devices and the client, if I can be with her. Working concurrent with her. It will be nice like that because I cannot run the program alone but if I'm with one of the xxxx (organisation name) … like this lady who is helping me, it’s easier

I: Okay.

P: Because sometimes I am alone as I tell you, I have a patient who's critically ill. I send him to the casualty, because this is a healthcare facility, it’s a big facility.

I: Mmm.

P: It's not a small clinic, it’s a big clinic. So, hence I'm having a lot of patients, you know.

I: Yes.

P: When I'm busy with this patient, I have a critical patient. I have a patient who's coming to take treatment. I have to enrol them on the devices huh, it’s a lot. I need someone who can help me on that one

I: Okay, so what is it that this someone will do and what is it that you are going to do in the project?

P: So that someone would enrol on the device, so if the person who’s coming to help me is from xxx (organisation name) and trained on the device, able to put the patient on the device it’s easier. When I give medicine, I write because when I initiate, I open the file, we have the files.

I: Mmm.

P: For DOH, we open the file. So, when I write on the file, she or he can continue this side with the device *ne (right)*

I: Mmm.

P: So, to enrol the patient on the device, it limits time, my time.

I: Okay. Then in terms of monitoring patients, if the patient is adhering well, is it okay for that to be done by a nurse to monitor?

P: Yeah, there was also an assistant nurse when I taught, he can- he can monitor, check the tablet

I: Okay.

P: Mmm.

I: Thank you so much for all the information, so we are done now in closing remarks. Is there anything else that you want to mention to me? That is important that you did not talk about?

P: Mmm I… I… I wanted to talk about this box

I: Mmm.

P: You know when they bring, they bring many, let me say they bring 20 boxers and then we lose sometimes, we lose 2 boxes. Then we are running short of 2,

I: Mmm.

P: Now is 18 and we have to give 18 patients, as time goes on again, we lose maybe 2 now we're left with 16. So, my worry is I don't know how to… how to…how can we… we do about with these boxes so that we cannot lose them, especially to patients like those using this nyaope substance?

I: Mmm.

P: That is the only challenge that I'm facing with this lost to follow, especially those who are using nyaope (drug substance). That is only my worry,

I: Okay.

P: Because the other 4 will suffer waiting, because now we will be waiting for this 16, we’ll be working with 16 instead of 20.

I: No, it’s noted, we are going to see what we can do about those, but are they a lot those who are not returning?

P: No. I only have 2 now.

I: Who did not return?

P: Yes

I: Okay.

P: Most of them returned them. I only have 2, XXX [Patient’s name] and the other guy is XXX [Patient’s name].

I: Are they both on nyaope?

P: XXX (patient 1) is not on nyaope, he had cancer and saying this TB medication is making me very sick. This one XXX (patient 2) was on nyaope.

I: So, XXX (patients 1) did not return?

P: Patient 1 doesn’t fight with me, XXX [patient name] says, “I'm coming. I'm coming. I’m coming tomorrow.” He’s always coming and then when they go and check him at the house all the time the house is closed. And the health care worker cannot go late hours maybe at night.

I: Okay.

P: I don't know if it’s working, what’s going on with him, all the time they see him, even neighbours say XXX (patient 1) stays here, but they don’t find him during the day. And when I call him he answers. And say, no I’m coming don’t worry sister tomorrow -tomorrow I'm coming, he promises but doesn’t come. But with XXX (patient 2) No, phone, no tracking. We cannot track him at his home they say they don't know where XXX (patient 1) is.

I: And on the platform. It looks like he's not adhering.

P: Yeah. [Inaudible segment].

I: The boxes are red?

P: Mmm.

I: Okay, sister, no, I hear you and all the information that you are giving us is taken into consideration. Thank you so much for your time,

P: Thank you sister [facilitator’s name]

I: Thank you and the end time of the interview is 13:05.

**GLOSSARY**

*Akere (Right) Sotho*

*Ne (Right) Zulu*

*Ukuthi (That)*

*Ausi (Sister)*
