# Supplementary material for: Acceptability of Digital Adherence Technologies to support people with drug-susceptible TB in South Africa
Source: PLoS One. 2025 Sep 24;20(9):e0332103. doi: 10.1371/journal.pone.0332103 (PMC12459780; doi:10.1371/journal.pone.0332103)
Supplement: S4 File — (ZIP) [file pone.0332103.s004.zip › S4 Transcripts/HCWs and Stakeholders/IDI 18-HCW.docx]

**TRANSCRIPTION NOTATIONS**

| **Label Key** | **Meaning** |
| --- | --- |
| **I** | Start of each new utterance by the Interviewer |
| **P** | Start of each new utterance by the Participant |
| **N** | Note taker |
| **{ }** | Indicates that details were changed or pseudonyms were used to anonymise data |
| **( )** | Indicates the description provided to anonymise data |
| **XXX** | Words were omitted to anonymise data |
| **-** | Breaking into a sentence by the next speaker |
| **…** | Pause or drawn out words |
| **[ ]** | Indicates noise made, e.g. [laugh], [sigh], [pause] |
| ? | Beginning of utterance by unidentified speaker or questionable text |
| **[inaudible segment]** | Unclear section of the recording |

I: Huh, thank you so much for agreeing to participate in the study today, huh, can you please allow us to audio record this interview?

P: Yes, you can start recording-

I: Okay.

P: The interview.

I: Alright. PID number is: xxxx Date of the interview its: xxxx (interview date). Location its: xxx [clinic name]. Type of the interview is: Community health care worker. Facilitator: xxx [facilitator’s name]. Time of the interview is: 13:22.

I: Alright *sisi (sister)* can you please tell me about your current position?

P: I’m a CHW (Community Health Worker).

I: Mmm.

P: *Mo (Here) xxx* [clinic name].

I: What are you doing in relation to TB?

P: Huh, we are working with patients who have TB.

I: Mmm.

P: Community health work, we are helping who have TB at home.

I: Mmm, what exactly do you do with them?

P: Huh, we doing a DOT.

I: Mmm.

P: We check patients who have TB every day, we diagnose those patients with TB. So we go every morning to check if patients have medication, and if the patient is taking their medication. There is a box which was given to them at the clinic, so we make sure that they are taking medication because the box shows a green light when the patient is taking their medication.

I: Mmm.

P: And then there’s huh, a red light on the box which shows us that the battery is going to die.

I: Mmm.

P: And then we have a yellow one and an orange one.

I: Mmm.

P: The orange one shows the patients has medication, so if the box the patient has taken medication it shows a green light.

I: Where do you see the green light on the box?

P: On the box.

I: Where do you see?

P: We see the green light on the box, where the patient has put in their medication.

I: Mmm.

P: It shows that the patient has taken their medication, and also the green card is ticked everyday if the patient has taking medication.

I: Huh, they are also ticking on the green card?

P: Yes, everyday

I: Huh, so you are telling me that you go to their households every day and check if they’re taking they’re medication?

P: Yes.

I: Do you have challenges with patients that are not taking medication at home?

P: Yes, we do have challenges.

I: What are those?

P: Some patients are not taking medication, especially those that are using drugs.

I: Mmm.

P: Yes, they are the ones who are giving us problems, but other patients are not giving us problems with taking medication because, we are giving them counselling and talk to them about TB treatment.

I: Mmm

P: Yes, every day, so that they continue adhering to taking medication.

I: Mmm. So, you are telling me that you have a problem with patients who are using drugs?

P: Yes, they take medication on the first few months, but after maybe three months of seeing that they are getting better, they stop taking medication.

I: Mmm

P: But we don’t give up on them-

I: Mmm.

P: Yes. We do follow-ups and tell them to come and continue with treatment.

I: Mmm.

P: Yes.

I: So when you are doing counselling as well, what is the main problem? Why are they not taking medication?

P: Huh, the challenge is food, and others just wake up and forget taking their medication.

I: Mmm okay, so when it comes to patient care and counselling. What are your responsibilities?

P: Huh, we make sure *huri (that)* patients are taking TB treatment every morning.

I: Mmm.

P: And make sure that the treating is well on them, we refer patients back to the clinic and sisters check for symptoms that make patients sick.

I: So, you were telling me that you go and check patients-

P: Every morning

I: Every morning, and you are also telling me that there also boxes, smart pill boxes. So, I am trying to understand *ukhuti (that)* how does the box help the patient and how do you help the patient?

P: The boxes help the patients because if they put the box on table like this?

I: On the?

P: Table.

I: Mmm.

P: In the morning when they see this box and the green light, it reminds them that they

must take medication.

I: Mmm.

P: Yes, and we also check to see the green light and it shows us that the patient has taken medication-

I: Mmm.

P: And then we count on the green card again to see if the patient has taken medication.

I: Okay, is there a case whereby *ipatient (the patient)* *ibox (box)* is showing green and hear the alarm and the patient did not take medication, even though the alarm reminded them to take their medication?

P: Yes, some of the patients forget to take the medication on time-

I: Even though the box is there with the alarm?

P: Because the patient hasn’t gotten used to the box, but that’s why we make sure that we visit them every day.

I: Mmm.

P: Yes, and make sure *huri (that)* take their medication, and we do help them take medication, and remind them to put the box on a place where it will remind you, especially if you see the green light which shows them that it’s time to take medication, and it makes they’re life easy and reminds them.

I: Okay, because it’s the green light and the alarm, are there patients who say they don’t hear the alarm?

P: Huh, They hear it, it’s only sometimes when the patient says he’s busy.

I: Mmm.

P: Yes, or maybe he is far from the box, but when the alarm rings they take medication.

I: Okay.

P: It’s easy for them especially with the help of the box. It’s very easy for them with the box helping them to take medication on time.

I: What is easy with this box? How is it helping them?

P: Huh, especially the family,

I: Mmm

P: Even if the patient is sleeping, the family is reminding when they see the green light as a reminder that the patient must take medication, that’s when the family reminds the patient that it’s time for medication.

I: Okay. So, it also helps the family to support the patient-

P: To support the patient.

I: Okay, alright, that’s good to hear that. So ,when it comes to the-the- the digital adherence platform or digital adherence technology which is the ASCENT intervention, what are you doing in the project? How are you supporting the implementation of the smart pill boxes?

P: How are we supporting the patients?

I: Yeah, how are you supporting the patients according to the program itself?

P: Okay. We visit the households and make sure the patients have medication in the boxes, and remind them to refill their medication when it is finished-

I: Mmm.

P: If only the patient is left with pills for one day, we remind them to go and collect pills tomorrow morning.

I: Okay, alright. What else are you doing in working with the ASCENT team to support TB patients?

P: Huh, we give them counselling.

I: Mmm.

P: Because most of the time huh, the nurses are busy because of the queue-

I: Mmm.

P: So, we help TB patients by giving them counselling.

I: Mmm.

P: Yes. We tell the patients and the family to open windows every day and we teach them how to live with TB patients.

I: Okay, alright, so when you have to go and do home visits, how do you know who to go to?

P: We take the patient from the clinic.

I: You take the patient from the clinic?

P: Yes.

I: Where? Who is giving you those patients that you need to visit?

P: Sister XXX (nurses name)-

I: The sister who is-

P: Who is working with TB patients.

I: Okay.

P: Yes, she is the one who gives us patients on TB treatment, she gives us names and addresses, that’s when we go to their households and visit them.

I: Okay, alright. So, are those people having boxes?

P: Yes, and they put medication in those boxes, that’s why we can see if the green light shows, then we know that the patient is taking medication.

I: Oh, all right. If you- if you are asked to explain to another healthcare worker about digital adherence technologies, which is the ASCENT program, what can you tell them about this program?

P: I have to explain to another CHW?

I: Mmm who doesn't know about the technology? What can you tell them about these technologies?

P: I'll tell them if she goes to a patient that has TB. She must look at the boxes, the digital box. If it shows a green light, it shows that the patient has taken medication,

I: Okay.

P: Yes, if it shows the red light, it means that the battery is going to die. So you must check the box, if it shows the orange one, the orange one is the alarm. So, I will teach the CHW, that they must look at the -the patient's treatment in the boxes. You must make sure that the patient is taking the medication, he can open the box, but not take the medication. So, we must make sure you count the pills to see that the patient is taking the medication every day.

I: Okay.

P: And the green card also shows if the patient has taken medication, he must tick the card every day. Make a pill count every day.

I: Okay. What can you tell the other community healthcare worker about the alarm about the box? What does the box mean? If it's alarming? What does that mean to the patient?

P: If the box is alarming-

I: Mmm.

P: It reminds the patient that you must come and check medication.

I: Okay, alright. Huh, please describe huh, please describe your role when it comes to the support action *andithi* (*isn’t it*) when the patient is not taking medication we can see on the platform. So now we need to- we need to call we need to do a home visit, what is your role when it comes to this Support action?

P: If the patient doesn't take the medication, I’ll refer the patient back to the clinic. And first, I must talk to the patient. Why don’t they take medication? If you have a problem, maybe the medication doesn't sit well on him. So we must refer the patient to the clinic.

I: Mmm.

P: Yes, to see a sister or a doctor.

I: Okay, what else do you do when trying to help the patient?

P: Huh, we are supporting the patient. Sometimes the patient doesn't- doesn't take medication well because of huh- because of the diagnoses. Sometimes the patient is in denial. Then we support them through counselling, talking to them and how to take treatment every day.

I: Huh Alright. So when you first heard about this technology, digital adherence technology, what were your expectation like what were you thinking?

P: I was like huh, is the patient going to take this box serious? Or they're just going to leave the boxes. And I was like, hey, the patient will not use them but we have seen patients using the boxes very wisely. So, I say it's fine. It's working for them.

I: Okay. Why are you saying it's working for them?

P: Because of the green light and the alarm-

I: Mmm.

P: Yes, it's reminding them that it’s time to take medication.

I: So, did your expectation change?

P: Yes. It did change a lot

I: Okay, how?

P: Huh…because the patients are taking the medication on time.

I: They are taking the box seriously. And you were thinking that the patients won’t take the box serious.

P: They are taking the box serious, even when they come to the clinic. They come with the box and that’s how we see that the patient has been taking the medication, especially after two weeks, when you see the patient is doing well.

I: Mmm. Where do you see that the patient is taking the medication? You’re saying that you can even see that the patient is taking medication. Where do you see that?

P: We are doing home visits every morning, and I know the patient will take medication. Maybe by 8 o'clock. So, I make sure that by 8 o’clock I am at the patient’s household and helping the patient to take medication. And we- we teach them how to take medication.

I: Okay.

P: Yes.

I: So, have you ever checked treatment adherence on the tablet on the platform that is on the tablet?

P: Yes.

I: Have you ever checked the patients there on the platform?

P: Which?

I: The tablet?

P: No, we don't see it. We check the green card.

I: Alright.

P: Yes, at the clinic they give patients a green card that shows us that the patient is taking medication, because everyday that the patient takes the medication we tick.

I: Okay, so you don't use the tablet that is used by the nurse and the intern? You’ve never checked adherence of the patient there using this tablet?

P: The tablet?

I: Yeah.

P: It’s used by the sister.

I: So, the sister is using it?

P: Yes,

I: So you've never checked *wena (you)* have they ever showed you how to see if the patient is adhering or not?

P: On the tablet?

I: Yeah, like to see the adherence?

P: Yes, the sister shows us.

I: What do they show you?

P: That the patient is doing- is taking the medication.

I: So you're able to see if the patient is taking medication or not on the tablet?

P: On the tablet?

I: Yeah.

P: No, we don’t check on the tablet.

I: You've never checked?

P: We don't check on the tablet. We count the pills every day.

I: Okay.

P: It shows that the patient is taking the medication and we make sure that the patient that we are there to see the patient taking medication.

I: Okay, alright, so were you trained? Can you please describe the training that you received about Ascent, were you trained about the ASCENT?

P: [inaudible segment]

I: No, about the smart pill box.

P: Sister XXX (nurse’s name) trained us here at the clinic.

I: Oh, she trained you?

P: Yes, she trained us here at the facility

I: Huh, okay, do you still remember a few important things that she told you when she was training you about this smart pill box. What did she say to you? When she was training you, something that stood out that is important for you that you still remember?

P: Huh, the important thing that she taught us, was about the box and the green light.

I: Mmm

P: Yes, if the green light shows green, which means the patient is taking medication well,

I: Mmm.

P: And then the red light, the red light shows the battery.

I: Mmm.

P: Yes-

I: So, if there's a red light. What does it show?

P: The red light shows that the battery is going to die.

I: Okay. What are the patients supposed to do if the battery's dying?

P: Huh, if the battery is dying, they must…I forgot

I: They're supposed to come to the clinic-

P: To show the sister and the sister a gives them a new battery.

I: A new battery?

P: Yes.

I: Alright. And then how was the training? How was how do you feel about the training that the sister gave you?

P: Training was well-

I: Mmm.

P: We've learned much about the box.

I: What much did you learn?

P: We learned that the treatment pills mustn't be all the over the place. All the medication must be put in the box. So that it's safe there in the box

I: Alright, so you're also teaching patients to keep the medication-

P: Yes, in the box.

I: Why?

P: Because if at home there are children, they can take the medication pills and lose them.

I: Okay. But now if they have the box?

P: If the medication is in the box, it's safe.

I: Alright. So from your- from your own, from your perspective as a community health care worker. What do you think are the benefits of- of using the smart pill box? How does this whole ASCENT program help TB patients and also how is it helping you as a healthcare worker?

P: Huh, as a community -as community health workers, it helps us see that the patient is on treatment. And then their pills as safe on the box. Especially with the family. The pills are going to be safe in the box. And then it will help them to remind the patient to take medication especially with alarm it helps the family a lot, because other families don't know how to remind the patient to take the medication. But if the box is still there, it shows the green light and alarms. It helps the family to give the patient the pill.

I: Okay.

P: Yes.

I: Alright. So, I hear about the benefits. Thank you so much about the benefits that you told me about. So and now I want to know about the challenges. What are the challenges of using this box in supporting TB patients?

P: The challenge is especially more with patients living alone, maybe the patient is bedridden.

I: Is?

P: Bedridden and starting TB treatment. Maybe if the patient is living alone, and then the box is there, but it’s far from him or her, but with our help, we come to the household and help the patient get treatment, especially on the people that are taking drugs

I: Mmm.

P: Yes, that's where we get challenges. Because if the patient doesn't take medication. So, the box is going to be on yellow, it shows that the patient doesn't take medication.

I: Okay. So, if the box is there, and it’s reminding them, what makes them not to take medication, because they have the box and yet *iyalarma* (*it alarms*) why are they not taking medication and yet the box is there?

P: Huh, some patients say the TB treatment is not easy to take. But with our help. We are treating them to make it easy for them to take the medication.

I: Okay.

P: Yes, with the box that shows the green light, if the patient missed the time of taking medication. It helps them if the green light on the box still shows, it reminds them that you didn’t take your medication, go take your medication.

I: Okay, alright. So what are other challenges of using this box that the patients are telling you about? Besides somebody who's sick, they can’t go and maybe take the box, because they are bedridden like you’re saying, what are other challenges?

P: Some of them say can’t take the box with if they going to a funeral or town, but we teach them *huri (that)* if you are going somewhere take the pills and rap them with something safe.

I: Mmm.

I: So, they don't travel with a box. What is their challenge?

P: Some of them are maybe ashamed of taking the box to public places.

I: Okay.

P: But we are trying to talk to them.

I: Mmm

P: Yes. You can take the box and put it in the bag and go where ever you are going

I: Okay.

P: Yes.

I: Huh, so what exactly is their challenge with taking the box? Any experience that they told you about, by being seen by people with their box? Or having the box

P: Yes, because if you are taking the box and then it alarms, people will feel ashamed you know, people will see me taking the pills and ask me what is this in the box, but it's a smart box, we are trying to talk to them. If you're taking this box, put it in the bag its safe and then even if you are far from home, the box is safe, and it will remind you when it's time for medication.

I: Okay, how do they take that? Do they take your advice if you're telling them *ukhuti* (*that)* they must take the box with?

P: Because we are telling them that you must not be ashamed of taking medication.

I: Mmm.

P: Yes, because it's helps a lot to take the box. If the patient is going to visit, then take the box with them because the alarm helps.

I: Mmm.

P: Yes.

I: Okay, alright. So, in your thinking, do you think that huh, these technologies can improve TB treatment adherence?

P: Mmm.

I: The smart pill box, the use of this technology, can it make the adherence better?

P: Yes, it makes adherence better. It makes the adherence better, this one is good for the patients.

I: Mmm why are you saying it's good for patients?

P: Huh because of how it reminds them,

I: Mmm.

P: Yes, and how it shows the orange and green lights.

I: Mmm can you please - can you please talk about the- the negative experiences of using the box?

P: Huh, the negative is the patient who is not opening at home.

I: Who is not opening at home?

P: Yes, and then we tell them about the TB treatment, huh, TB is a serious illness. So, people were afraid - were afraid of TB.

I: Mmm.

P: But with the help coming from us. At least we teach them how to live with a patient who has TB, the boxes makes their life easy.

I: How?

P: Huh, everything the box has with reminding them,

I: Okay.

P: Yes.

I: Alright how can we maintain these benefits of using this box? How can we make sure *ukuthi (that)* it’s continuing?

P: Huh, the box?

I: Mmm how can we make sure *ukhuti (that)* the benefit that people are getting from the technology is not stopping it continuous? How can we make sure *ukhuti (that)* it's sustainable like it's something that is doable?

P: Okay, huh… for it to be sustained it helps the people to take medication and then at the end the patients who are taking TB treatment are getting well with the help of those boxes.

I: Alright mmm…so can you please describe- can you please tell me how were you monitoring? How were you monitoring treatment adherence before using these boxes? How were you making sure that patients are reminded about taking medication before we came with the technology?

P: Huh, every day we are doting the TB patient. Every morning. There's a green card, given to patients at the clinic and we monitor the patient by helping them take the medication. There's a green card where we tick every day, but it was difficult because of maybe let's say I'm doting 5 patients-

I: Mmm.

P: if I get at your place in the morning by 8o'clock, you are taking medication by 8 o’clock and the time is clocking when I get there, or if you're taking medication by 8:30.if the first patient is taking medication at 8 and then I enter into another patient that is taking medication also at 8. I wouldn’t make it to both households on time, time won’t be on my side, but with the help of the boxes. It reminds the patient that it’s time for medication, at least when I get to the second house, to the second patient then it will show me that this patient has taken medication-

I: Okay.

P: It makes their life easy because the box is clicking and shows the green light-

I: Even if you are late-

P: Yes, the box will remind the patient that you must take the medication.

I: Okay, alright. So, with the use of the box, it makes it easy for you even if you're running late, because now if maybe five patients are taking treatment at eight. It's not easy for you to be-

P: to reach them at the same time. But now with the help of the boxes, it helps the patient to take medication.

I: Even if you're not here-

P: Yes, even if I’m not there.

I: Alright, that's a very good point that you're making there sisi and thank you so much for all the information that you have given us. And then before we can close, we are actually closing the interview, now that we can close. Is there anything that you want to tell me about huh that is important about using these boxes? How are they benefiting patients any last important thing that you can talk to me about that we didn’t talk about?

P: Huh, we are so grateful of the boxes that you have come with, because it makes our work easy. Especially with the time the patient takes medication. It helps us.

I: How? Can you explain how is it helping with the time?

P: With the time, if three patients are taking medication by the same time. Maybe they're taking medication at eight o'clock in the morning. If I'm running late, maybe I'll be at the first household and this other patient will not suffer, because they will no longer wait for me to help them to take medication-

I: Mmm

P: At least the box will remind them and show them that it's time for taking medication. It helps a lot.

I: Alright. Anything else interesting that you want to mention again about using these boxes and how are they helping the healthcare workers and the patient's on treatment adherence?

P: Okay. The boxes help the patients and also CHW’s, to see if the patient had taken the medication and it’s easy because if we get to the patient, we're doing a pill count. The patients with a green light it shows us that the patient has taken medication (facility worker walked in)

I: Alright anything else that you want to mention?

P: Huh, keep up the good work with the boxes and then don't stop them, because it helps the patients and us a lot, so don't stop with this program of the boxes.

I: You are saying it helps you a lot. How is it helping you?

P: It helps us with a patient especially when keeping the medication.

I: With keeping medications?

P: Yes, It keeps their medication safe.

I: Okay, alright. Thank you so much sisi for all the information that you have given us. It’s really the end of the interview now, and the time of ending the interview is: 1:58

I: Thank you for your time.

**GLOSSARY**

*Mo (Here)*

*Sisi (Sister)*

*Huri (That) Sesotho*

*Ukhuthi (That) IsiZulu*

*Ipatient (The patient)*

*Ibox (The box)*

*Andithi (Isn’t it)*

*Wena (You)*

*Iya-alarma (Is alarming)*
