# Supplementary material for: Acceptability of Digital Adherence Technologies to support people with drug-susceptible TB in South Africa
Source: PLoS One. 2025 Sep 24;20(9):e0332103. doi: 10.1371/journal.pone.0332103 (PMC12459780; doi:10.1371/journal.pone.0332103)
Supplement: S4 File — (ZIP) [file pone.0332103.s004.zip › S4 Transcripts/HCWs and Stakeholders/IDI 19-HCW.docx]

**TRANSCRIPTION NOTATIONS**

| **Label Key** | **Meaning** |
| --- | --- |
| **I** | Start of each new utterance by the Interviewer |
| **P** | Start of each new utterance by the Participant |
| **N** | Note taker |
| **{ }** | Indicates that details were changed or pseudonyms were used to anonymise data |
| **( )** | Indicates the description provided to anonymise data |
| **XXX** | Words were omitted to anonymise data |
| **-** | Breaking into a sentence by the next speaker |
| **…** | Pause or drawn out words |
| **[ ]** | Indicates noise made, e.g. [laugh], [sigh], [pause] |
| ? | Beginning of utterance by unidentified speaker or questionable text |
| **[inaudible segment]** | Unclear section of the recording |

I: Do you agree to be audio recorded?

P: [Laugh] Yes, I agree.

I: Thank you, thank you for blessing us with your time. Thank you for coming.

I: Mmm ok. Date of IDI: xxxx (interview date) , Location XXX [name of the facility] language: English and PID of the participant is: xxxx and the time is now: 2:28 huh 14:28pm.

I: Mmm. What was the title of your previous position?

P: It was Research Assistant Intern.

I: Mmm, and what were your duties? What were you doing as a Research Assistant?

P: Ok, on a daily basis, I would come to work, identify new patients, go collect sputum’s to make sure that we get the target that is needed, because at the training they said we must at least get 5 patients on a weekly basis so, I was working on getting that target as much as possible. So, I would do Urine, sputum, then sometimes I'll go take blood to identify those suspects. And if we get a new patient, what I would do personally. I would withdraw them from the TB nurse and then take them to my room, explain the box to them and then ask them if they are interested in having it and I told them the disadvantages and advantages of having a box. However, I was not forcing them, I was giving them the space to think about it and they can come in because when we initiate them we give them two weeks prior so that they can figure it out whether it's something that they want or they don't want it that's how I kept it going from my side.

I: And how long did you work as a Research Assistant Intern?

P: A year, from xxxx (month, year) to (month, year)

I: Ok, huh if you were to explain to someone who doesn't know about the DAT intervention, what would you tell this person about this DAT intervention, the box? What is it?

P: Ok, I would just get them a sample of the box so that they can have an idea of what I'm talking about and then as I explain what it does and how- it can assist them to better take their medication and everything. I would just give them the paper because they gave us the paper that shows all the things that I was saying regarding the box, so meanwhile as I’m explaining the box you will be reading for example. Some people couldn't understand that why when you open it, it makes a sound, and it even makes the same sound when you close it. So I had to explain that to see that your box is working and functioning fully you need to make sure that when you open it, it flashes the green light and makes the beeping sound and even when it closes, so that it can report correctly to the platform. Another thing when the battery is red, they kept on asking about the battery, do we have to charge it whatsoever, so I always make sure and emphasize that whenever you see the red flash on the box, You know it's time to come to the clinic or on your next visit you come with the box and give it to us and explain that you know my box has reached that point of flashing the red lights, then what’s the go ahead and then we change the battery and those kinds of things and always encourage them to take their TB medication from the box so that it can be easy to engage with the TB nurse and see if the person is following whether they will be any complications, because we find that some patients will be taking the medication, TB medication correctly on time. But then they are deteriorating instead of getting better. So the DAT helped us to see okay, this patient was taking their medication correctly at all times. What went wrong? Sometimes it can be the medication rejecting their body completely. What needs to be done? So it was working as a backup for the TB nurse to say oh now I know my patient. This one has been this kind of a patient for me. I need to make further investigation with their diseases.

I: Ok. And then with this box *ne (right)?* What processes mmm that mmm had to be done before handing over this box to the patient as you that you were giving this box to patients? Before you could hand it over to a patient?

P: Ok, first things first, you explain who you are to the patient, you give the person the consent form. They need to consent before taking it away. Then there are some SOPs that you personally as a research assistant need to fill while you're with the patient because they will be asking at some point they're asking you about the patient's age, telephone numbers all those kind of things. So you put all your documents that you're going to need in front of you along with the box. If the patient is interested in that you give them the consent form first so that they fill it in and by doing so they are informing and allowing you to get them to participate in the research study. So that's the first thing first.

I: Ok, and when you were offering patients boxes, I mean in that process did you encounter a situation where there is one patient who refused the box, and say no I don’t want it?

P: Yes, there is one patient who refused the box, but later on realized that they need it back, maybe at first it’s because she mentioned that everything was too much at first, she just discovered that she had three things in her body going on. I can’t disclose that. However, it was a shock of a moment then I told her about the box at that moment so she refused. And the following week I went to follow up with the patient how are you? Are you still interested? I’m still giving those boxes. Are you still interested in that? Then she later gave me the permission to say hey, yes I can do that. What is it all about, do I need to pay, and some people refuse because they thought we are selling it. So we need to emphasize that no this is a support box from the clinic so it’s for free. And then you’re going to enjoy having it, make it like a smarties type of thing, so yea.

I: Ok, and then you are mentioning that this box reminds patients to take medication. It’s like support to them mmm. What I would like to know is how or what else does this box come with. I mean how did you support patients using this box, what else did you use together with this box? You know to support patients?

P: Ok, if the patient is not taking their medication first thing when you enter you check your platform, who took medication yesterday, who missed doses, what's going on with who. Then from that platform it gives you options whether you do home visit. Maybe the person is not taking medication for the third time, you send the CHW’s (Community Health Worker) to their home. And then they reach out to the patient and see what's going on you make a phone call and you check what's going on. Sometimes you find that the patient took the medication but the system didn't capture that, I don't know what happened I found a lot of those. Another thing on the visit you show the person their platform to encourage them like hey, look what how you’ve been doing, this has been your platform and how it looks like then compare to maybe someone who’s not taking their medication. You compare it with someone who's really consistent and you see where the danger is for yourself. Then with the box what you will do is they give you time then you set the alarm at home. If the time passes, they will get reminders like “hey you haven’t taken your medication. Please don't forget to do that.” Yeah.

I: Ok, You mentioned the platform where you see if a patient is missing a dose and you make those follow up phone calls. Do you still remember the name of this platform [laugh] what is it called?

P: Oh, the name is xxxx (adherence platform name)

I: Ok, And where did you- after how many missed doses would you call a patient?

P: Immediately when I get to the clinic, I would want to you know why didn't you take yesterday's doses? What happened? Then If I can't get a hold of her then I'll just leave it for a day and then tomorrow if still fail try to get hold of the patient I will just arrange with the CHW that tomorrow if you’re not coming or going to this place please check on this person because today is the second day they’re not taking medication which alerts that tomorrow they might not take it or maybe they're not at home or something, please confirm for me because I can’t get hold of her.

I: Ok, and the CHC you said are the what?

P: CHW (community health workers).

I: CHW *askies (sorry)* are the ones that track down the patients?

P: Yes.

I: Ok, mmm can you think of one patient who was struggling and CHW had to be sent to look for this person? Can you think of one situation and just briefly describe to us what happened? What was the problem with this patient? And were the CHW’s successful in tracking this patient? and also trying to get them back on treatment. What happened?

P: Ok, mmm can I just disclose the patient’s name?

I: Mmm, no you may refer to them as Sir?

P: Ok, there’s this patient who was on Nyaope substance. At first, for the first two weeks he was fine coming to clinic doing follow ups and everything. After 2 months of taking medication he felt so better. Like he didn't come to the clinic he just went MIA (missing in action) I asked the CHW to identify the patient and go to the house. Only to find out that this patient is not staying there, they staying on the streets and everything. Because of the dedication of this woman, she went and found where he is located exactly, then every time I will give the lady the CHW the date of the patient and she will make sure that every day when she comes to work, she will get the patient and come with the patient to the clinic prepare everything for the patient and take them home. She will make sure that this patient was taking his medication on a daily basis because the box was staying with the CHW. So every time she would go to the patient and open the box and make sure that the patient is taking the medication. So that was more of their intervention and working together with her up until the end of their TB treatment.

I: And do you If that patient has finished they’re treatment or they’re still on treatment?

P: No, they completed their treatment.

I: Yes, and the adherence was good?

P: Yes, the adherence was perfect.

I: Yes, ok mmm and you mentioned mmm something about wrong addresses where the patient was not found. He reported they were staying there. How often you did you have such cases or people who give out wrong addresses or people who move from where they were staying and when the CHW goes there they don’t find them? Are you aware of any cases where CHW had to track down this patient?

P: Yes. I had such kinds of moments with patients, however they had a way of communicating because I would make them engage. I would find the CHW and the patient to and say you are going to work together with the CHW throughout the process. Where are you staying? Because there are certain people who stay in areas where there is no address there is nothing so we need to find something that will identify when you need to, and then you need to know someone who is famous staying around there you can ask. Do you know so and so I can know how far you staying from that person or how close you staying to the person then that's how they used to identify each other. Unfortunately, we couldn't find them up until they come for the visit in the clinic, and we ask where are you really staying? They will be like I stay in farms in XXX [location] and whatsoever unfortunately CHW’s can’t reach there and we don't have the right to take away the medication from them.

I: Ok, according to your knowledge was this the only patient who was on the substance of drugs?

P: No.

I: There are others?

P: Yes, there are others.

I: And what has been the experience with this group of people [substance abusers] because of their habits I would say they present other challenges when it comes to the box, or the adherence?

P: Sometimes it goes to who are you and how do you present yourself to them because when you're mean and rude to them they don't want anything that's connected with you or come to you. Some of them- we create good relationship with them until we make sure the treatment is complete.

I: Yes.

P: And unfortunately some of them pass away throughout the journey because they relapse they’re taking ARV’s and they're taking TB treatment then they relapse, the re- infections of all those kinds of things because they injecting themselves and you can’t stop them from doing that during that time.

I: Yes.

P: But then you can guide them. I had a focus group on Tuesdays. Where I met all of them. And then we talk about the experience. we talk about what went wrong in life. So that's how I personally manage to get through them and encourage them to finish the course and give them hope of life. And you get to see that some of them they fall into whatever they are doing because they needed someone to listen to. Nobody was there for them. So only friends and advisers that were there and led them to that route. So yeah, they are my friends. [Laugh]

I: [Laugh] Ok and how were the responsibilities and duties shared amongst you and other health care worker’s as you mentioned that there are the people that you're working with, in particular, how were the duties shared? What did you do and what did the other’s do?

P: [Laugh] Ok. It will depend on daily basis. Because for me it was more of learning and enjoying what I was doing. I didn't really have strict responsibility. XXX [referring to interviewer] this is what you do on a daily basis. Daily, I knew that every day I needed to issue a patient a box and I need to work with them with love and make sure that we take care of each other, because remember these people are the ones who get patients. So I get patients from them, so I need to respect them, so whatever they would say hey, girl do this I would do it with respect and with all my heart. So it ended up not knowing who is who who's doing what because we are doing it repeatedly. I would know how to prepare the medication for the new patients what is needed what is not needed. The only thing she would just fill in the file sometimes they would get under pressure and forget certain things. So when you’re there you're supporting them as well, to make sure that everything is going smoothly on daily basis the facility can go crazy at times. So responsibility. You were responsible for everything as long as you were reporting for work. As long as you’re in the TB room you are responsible for everything that’s happening in the TB room. So, yeah.

I: And mmm, have you been involved in counselling a patient?

P: Yes.

I: What was the problem with the patient?

P: As I mentioned, those Nyaope boys.

I: Yes.

P: The focus group was more of counselling and getting to know what's going on. I had elders. Sometimes it's history at home. So, yes, I had moments with those it’s not like they were being affected in medication but then they needed someone to talk to they needed support in a different aspect of their lives. So yes I've been involved in several counselling’s.

I: Ok, And when you first heard about the DAT intervention *ne (right) mmm.* What were your expectations before it was implemented or before you were involved in implementing this DAT? What were your expectations? I mean, did you think it was going to be – [inaudible segment]

P: Before I knew about this, it was that thing of like it is what it is for me, it was a thing that comes, then I’m going to do it with the belief that I’ll be the best in everything that I do. So whatever it is, I need to make sure that I find the best way of doing it and enjoying it. So my expectations was to be the best [laugh] in whatever I’m going to do. So, yes.

I: [Laugh] Mmm, did you receive training before you started implementing the DAT?

P: Yes, we did, it was in XXX [training location] the training.

I: And mmm, do you think the training was successful? I mean was the information that was provided to you guys, was it sufficient?

P: Yes, the training was successful and it was sufficient. However, there are some certain things I don't think people who were training us were aware of, because they mentioned that it's a new thing for them. So whatever we're going to expect and whatever we experiencing in our facilities, we are reporting back to them so it was more of a learning process for everyone. So yeah.

I: Do you think there is a room for improvement in this training? How can the training be improved? How can it be sufficient and how can the training be improved?

P: Huh how the training can be improved? It was sufficient. I think that if they can improve all the mistakes that we have reported the errors that have been happening. Then, it will be sufficient, like they won’t encounter much problems as we did. So, area of improvements are those errors that we reported.

I: Ok, and how frequent do you think the health workers should be trained? I mean on how to implement or use the DAT?

P: At least if they were given enough training as the research assistant and the research assistant should work openly to them with everything throughout the process. I think that will be fun because that's what I did. I involved them in each and everything I did. So which made it easier for them to engage with me as well.

I: And going forward do you think they should undergo trainings in between? Or everything is fine. There's no need to have more trainings?

P: Mmm. I think they can have feedback meetings where each and every one would express their feelings, their learning process because we are not the same. Some people need more training as they encounter, some need encouragement, and some need different things. So feedback meeting will give them hope and a moment of engaging with other people to share experiences, ideas on how they are improving their facilities and how they do that. So, yeah.

I: Mmm from your perspective *ne (right)* Mmm can you describe some of the benefits of the differentiated model of care? Do you still remember [inaudible segment]

P: Yes, home visit, phone call.

I: From your perspective. What do you think are some of the benefits of this intervention? The phone calls, the home visits? [Inaudible segment]

P: The facility visits, you get to see the person in their condition and whatever they are saying you will be able to determine whether something is fishy, something is however. As for home visits, I hadn't been to home visits, but I believe that those who have done it can see the area that the person is living in, and they can encourage what to do and what not to do. All those kinds of things. As for phone calls, phone calls, they [patients] lie. They [patiens] lie about situations they lie about it, they will tell you what you want to hear. For me, it was not working because I didn't want the patient to tell me what I want to hear. I wanted to know the truth, so, yeah.

I: Ok and then the DAT, What are some of the benefits that comes with the DAT from the patient's perspective? How does this DAT- how do you think this DAT benefits patients?

P: For patients: those who are using it and have given me feedback, they were emphasising that sometimes it's more of an alarm because they use it to go to work set it as an alarm to go to work, it encouraged them both ways. You need to take your medication and prepare yourself to go to work. Then some of them are lazy to take their medication but there will be that thing making noise inside of the house. It's either you stand up and switch it off and the moment you switch it off, you switch it off in a correct way by taking medication. That's how they benefited. Some of them it was more of needing something to know that there will be people supporting me as they are staying alone and staying alone they complained about no one's taking care of them. Some things are hard. So it was more of like they have a friend who's reminding them that hey we are here. If they are not taking their medication, there will be a message like “hi friend don’t forget to do this and this.” So those were the benefits for them from my side and their side.

I: And from your side, how did this DAT benefit you? I mean you and other health care workers?

P: From our team, it made us have effective communication, everything used to flow. Our engagements used to be more in standard, I would say we would make sure that we filled in gaps together as a team. So, it made us have more relationships than having to say I'm at work then I do me. So we needed to come up with strategies and come up with what works for us and what needs to be done to fill in these gaps. So it was more of like team building type of thing.

I: Ok and did the DAT improve the relationship that you had with patients? Do you think it improved?

P: Yes it did a lot. Because when they get to the clinic they’ll be like where is that sister who uses the box? Like every time they would search for that person before they go and see who they are here for. So, yeah.

I: I mean can you think of just one patient that will say *ey* using this DAT really worked for me and this patient we became close because of the DAT? Just one patient?

P: Mmm that’s all my patients that I've worked with. Ok there is this patient who was transferred from hospital, he had machines all over him, so everything with this patient was like we need to encourage, emphasize, encourage, emphasize in each and every visit. So we had to have a relationship together in a team and know that hey, we have this critical person and every week won’t pass by without checking on him and each and every week, we will make sure that we meet him because he was losing hope of life. Every week we made sure that we remind him that you know if you need anything you have a family in TB room, that’s where you can come and laugh about it. We make sure that even if our patients, who don't do things correctly we have a nice way to make them come back and do things the right way. We don’t fight with patients but we laugh with them about the matter and make them realize that, that’s not how it’s going to work.

I: Ok. Now can you describe the challenges of the differentiated model of care? What are some of the challenges that you had with phone calls, home visits and SMS’s- Automated SMS’s?

P: Automated SMS’s, people used to complain about it that they get SMS’s even though they took their medication. So it was frustrating because I didn't know why because I was told that they were only going to get the messages only if they didn't take their medication. But these people are taking their medication but still they're getting the SMS’s. With home visits, not everyone accepts people at their home, especially when they're sick. So to encourage or to insist in going to someone’s house while they're sick. It was the most difficult thing ever because you would get nasty words, you would get crushed by the door. So, the CHW’s work is very difficult because they need to deal with those challenges on a daily basis and come back and report. Mmm facility visits challenges is that some people don’t really honour their dates and you need to drag them to come to the facility to do all the things that need to be done. So those were the challenges l can think off thus far.

I: Ok and the Issue of the Automated SMS-

P: Mmm

I: I was asking if the issue of those automated SMS coming in when the patient took their doses. Was it resolved? And I would like to know how often did it happen; how often did you have such cases being reported people saying I’m getting these SMS’s even after I've taken my medication?

P: Almost all of my patients were complaining about that thing happening on a daily basis, up until they come to the facility and report it saying I should switch it off on the xxx (adherence platform) so I did that with all my patients. However, It was not really effective because remember when we give them box we said it’s going to remind them if they’re not taking their medication, but then now that opportunity is taken away from them because we switched it off due to the errors.

I: Ok and switching on and off the App mmm did it have any impact on the adherence?

P: No. I haven’t experienced any of that.

I: Ok, and were there any cases or issues of network being reported? Or you yourself, did you experience any network issues with the platform when you're trying to register a patient or you look for the missed doses on your platform?

P: Yes. I encountered a lot, up until my supervisor said some of those you can do them at home because here at the facility the network was not really perfect. So, I ended up doing some of the things at home because of the network.

I: Yes, ok any cases of stigma that were reported? A patient who reported stigma because they are on the DAT?

P: No, no one has reported stigma.

I: Ok, you said you had patients who are using drugs. Now I wish to know if you also had a group of patients who were homeless.

P: No, I didn't have anyone who was homeless. They had homes but they choose not to go to streets.

I: Mmm. We talked about home visits earlier on. So now I wish to know if the staff was always available when mmm home visits needed to be done.

P: The CHW’S?

I: Yes.

P: They were there, it’s their job, so they always provided their services when needed.

I: Ok, and from your perspective, do you think TB treatment can be improved using DAT or the box?

P: It can be improved.

I: Specifically, the adherence?

P: Yes the adherence can be improved using the box because I've realized that the moment you show them that you see them they have that fear. It puts fear of like what if I don't take it and then when I go to the facility they are going to tell me about it. So they always try to make the right thing despite you seeing them or not seeing them. So it puts a little bit of fear and that makes it worthwhile.

I: And how was the adherence monitored before the introduction of the DAT? Do you know how patients were being monitored if they are adhering to their treatment or not. What system was in place before the DAT was introduced?

P: Huh the only thing I know is that they were working with the CHW’s. So they would wait up until two months then the people will be considered as lost to follow, then that’s when the CHW’s goes to visit the patient and see if the patient is still alive. What happened whatsoever? So unless the family members report something to the clinic, so yes.

I: Ok and did the DAT have any impact on the workload, was there a change after the implementation of the DAT? when it comes to the workload that you have in the TB room?

I: Ok and did the DAT have any impact on the workload, was there a change after the implementation of the DAT? when it comes to the workload that you have in the TB room?

P: Yes, it reduced a little bit, because it made it easy for the TB nurse to use the platform the xxx (adherence platform) platform to see who is who is she dealing with, who does she need to focus on for example, seeing that the person hasn't taken doses for like 7 days. She was able to identify the patient and take out the file and write the history on the file so that when they are auditing or monitoring the patients file, they would know that this person this and this has been done but then still no improvement or this and this has been done hence the patient passed away because they come back questioning why did the patient pass away so as a nurse you need to be responsible for that. So now the DAT helps them to know which patients are taking medication fully and who's not. So, it's more like identifying the monster inside the group.

I: Ok in the absence of xxxx (organisation name) *ne (right)*, let’s say xxx (organisation name) is exiting now and a new team will take over the project. What is needed in order to sustain these positive changes you have realised? Since you mentioned the improvement on the adherence, it’s easy to monitor patients you know. What is needed in order to sustain this project to make sure that the intervention continues to run without any complications?

P: I would say they need to have a person specifically working with that, because the TB nurses have a lot of paperwork happening with them and then even the DAT on itself has work that needs to be done. You need to explain it, you need to make sure that everything is correct. The battery, the patient's taking the medication correctly, you need to follow up on the patient. However they still need to write the medication given on the next date of the treatment, all those kinds of things. It's exhausting. It's draining for them. And then you can only imagine how many patients that they see on the daily basis and how much of work that needs to be done. Especially when they have newly diagnosed patients. It's way worse because the process is longer.

I: Ok mmm can you elaborate on the negative changes of the differentiated model of care? Mmm the phone calls, you mentioned earlier people who don’t like home visits, you said something about some patients maybe being bed ridden and you go there and it’s a lot to them, mmm you mentioned something about- I can’t remember

P: The call.

I: Yes, yes. What are some of the negative things or changes? That relate to the differentiated model of care that you can think of?

P: Ok, mmm I will go to home visits because there was a patient who was very angry that we went to her house without letting her know. However we couldn't get hold of the patient. Some people perform certain rituals and they don’t want people to interrupt and interfere. Unfortunately we wouldn't know who does what at their home and who doesn't do it and it puts us in a more uncomfortable space because some people come to us furious about something that happened long time ago. We need to be accountable or responsible for it forgetting that we are also responsible for whatever medication they are taking or whoever in your family is taking and no one's taking responsibility to come and inform us about what’s happening about the patient or the whereabouts of the patient. And we need to report again back to the people who are holding the program. What happened to the patient? So it was more of an uncomfortable situation that we were being in.

I: Ok mmm are you aware of patients who were opening the box without taking their medication and just open?

P: Yes. I had a patient who like was diagnosed with multiple diseases. And he chose that he's not going to take TB treatment and he was opening the box every day on daily basis, and he came this other day I was checking up on him and said,” hey sister, you know what, I'm not taking TB medication because it has been the third time drinking this meditation but nothing. So, I’m giving other treatments time in my body .So, this one, I'm not going to drink just know that I’m just opening the box.” That's all and there is nothing I was going to do because it was his choice.

I: Ok, huh going back to the phone calls and follow up calls. I mean did you have a challenge of patients who provided wrong cell phone numbers and when you call it goes to voicemail?

P: Yes. I had multiple of those, some will give you Ex’s numbers when you call. They're like *yho mina* *(me and this person*) and so- so broke up long time ago and I would be like oh I didn’t know I’m calling from the clinic. Some just go straight to voicemail, some it's their siblings who were not even close to one another anymore. So yeah.

I: And what would you do in that situation?

P: I would wait for the patient to come to the clinic and ask what went wrong. What transpired? Why did do you give me so and so number’s? And then they will explain some of them don't have phones at all. So you need to take what they are offering you to get hold of them. Unfortunately you don't get hold of them through that number again, so it was quite difficult.

I: And what resources- What do you think are the resources needed to improve mmm the implementation of DAT? What are the resources that are needed in order improve the implementation of the DAT? What is needed?

P: What is needed to improve the DAT is that they should emphasize during training, they should consider that there are some areas that don't have good connections and in some areas they are working under pressure. So that can lead to their work being sabotaged in some way somehow or not being performed accordingly. For example, here in XXX [facility] some things needed to be provided before and prior in regard with what? Regarding me when I started my internship here. I needed to be introduced to the TB nurse because the TB is always occupied she needed to be explained everything and all the procedures that are going to be done. I needed to do it myself. And it was so hard because I’m new to this person and she's also new to me. So the communication was more like an interview of her work. So they need to make sure that they give the interns an open platform to work in so that it can be easy to engage not everyone can do that?

I: Ok and going back now to this question. In the absence of the xxx (organisation name) staff *ne (right)* who should help with preparing the box because I assume before you hand them over to patients, you spoke about charging and all those things even if they’re bringing it back saying now the battery is low someone has to charge it. Who should do that? Who should be responsible for that?

P: As I said they need to give the TB nurse a third hand. Whoever they will be identifying as the third hand in that team- the TB team. That person should be the one who’s responsible for all those kinds of things so that they don't have give all the responsibility to the TB nurse it’s not going to be fair.

I: Mmm you think that person should come from inside the facility?

P: It can be from the facility; it can be from outside. But they need to have the third person. Not giving all the things to one person.

I: And you mentioned the issues with the box mmm the technical glitches you mentioned where you ended up switching on and off the box in that situation who should help now in solving those technical issues? Should it be the same person who is preparing the box or you think someone else should focus on that technical side of it?

P: This third person needs to deal with each and every single thing that happens with the box. He must be responsible for the boxes in all the areas that are coming with the boxes. So, he or she will be the box master for that moment.

I: Ok, and the challenges that we had with follow up phone calls, the home visits and the boxes mmm. Did you record those somewhere?

P: Yes, they gave you us the SOP’s. So one of the log had that, where you fill in all those things that you did, with whom, when, the date, time and those kind of things.

I: Ok. So you could only document that on the logs as you saying? Was there anywhere on the platform where you could also-?

P: Yes. We had a box that says action, you could action it actually.

I: Ok mmm, ok mmm any gaps that you have identified which exist now in the way the intervention is being delivered? Are there any gaps you can think off that you found?

P: Not really, not really. I haven’t found gaps.

I: Ok mmm. Did you have any special group of people that huh were using the DAT? Mmm maybe… a certain age group or population who you think the DAT was working better for them compared to a certain age group. Was there some sort of difference between the age groups when it comes to using the DAT amongst your patients?

P: Yes, people who were only allowed to participate- remember they said to us this is a research so, you can’t research on a 2 year old. So our participants were like from 18 and above. So those were my target, my target was like18 and above. So yes there were age restrictions

I: Now was there a difference between those who were 18 and maybe late 20’s and those who were maybe 40 – 60?

P: Regarding the adherence?

I: Yes.

P: Yes. There was a gap in between. Because those who are younger, the 18 and 21 you will find that on weekends they’re not taking their medication. When you ask they’ll be like, I was not home. I was attending whatsoever, so it was more of like a lifestyle implicating with their medication.

I: Ok. Mmm were almost done. Were done actually. But mmm before we close. Do you have any other comments, anything you want to comment on maybe something that I didn’t talk about? It could be anything that relates to DAT maybe good or bad, Opinion anything?

P: No, I don't have any opinion [Laugh].

I: Mmm.

P: I don’t have. I have said it all, I think.

I: Yes. Ok huh it looks like we have come to the end of our session. And I thank you, we thank you for your time mmm for participating in this interview. Thank you so much.

I: Mmm the time is now 15:21pm

GLOSSARY:

*Ne* (*Right*)

*Askies*  (*Sorry*)

*Mina* (*Me*)
