# Supplementary material for: Acceptability of Digital Adherence Technologies to support people with drug-susceptible TB in South Africa
Source: PLoS One. 2025 Sep 24;20(9):e0332103. doi: 10.1371/journal.pone.0332103 (PMC12459780; doi:10.1371/journal.pone.0332103)
Supplement: S4 File — (ZIP) [file pone.0332103.s004.zip › S4 Transcripts/HCWs and Stakeholders/IDI 1_HCW.docx]

**TRANSCRIPTION NOTATIONS**

| **Label Key** | **Meaning** |
| --- | --- |
| **I** | Start of each new utterance by the Interviewer |
| **P** | Start of each new utterance by the Participant |
| **N** | Note taker |
| **{ }** | Indicates that details were changed or pseudonyms were used to anonymise data |
| **( )** | Indicates the description provided to anonymise data |
| **XXX** | Words were omitted to anonymise data |
| **-** | Breaking into a sentence by the next speaker |
| **…** | Pause or drawn out words |
| **[ ]** | Indicates noise made, e.g. [laugh], [sigh], [pause] |
| ? | Beginning of utterance by unidentified speaker or questionable text |
| **[inaudible segment]** | Unclear section of the recording |

I: Uh sister, do you agree for us to record this interview?

P: Yes, agree.

I: Todays date is the xxxx (interview date), the language that will be used for this interview is English. Uh the name of the facility is xxxx (clinic name), the PID of a participant is xxx and the time is 13: 35 PM.

I: Uh sister what is the title of your uh current position?

P: I’m a professional nurse.

I: Professional nurse. How long have you held this position?

P: xxxxx (date) till to date.

I: Ok. How long have you been working in the TB room?

P: … xxxx (number of months) now.

I: And what are your roles and duties in the TB room?

P: Ok, my main role here is to identify patients who have symptoms of TB, screen them and collect sputum and if they are coughing and having a productive cough then I do other examination to screen for TB such as x-rays. To give treatment, -to start treatment and to monitor patients on treatment and make sure they complete treatment…yah and more.

I: [laugh] Ok. And how are TB services delivered at this facility uh is it at facility level, district level or provincial level?

P: I don’t think I understand, can you maybe rephrase the question?

I: Uh what I’m trying understand is how are TB services delivered at your facility?

P: Ok. We see our patients on daily basis, I don’t know if I’m answering relevantly. We see our client as outpatients, they come, and we see them and go home. Then we give them a return date; they come and follow-up until they finish their treatment but if they have other problems or if they need admission, we refer them to xxxx [district hospital name] for further management and that’s where they are managed furthermore and discharged back to the clinic when they are stable.

I: Umm

P: And sometimes we get admissions like uh-some patients are admitted with TB from xxxx [district hospital name] and transferred to our clinic when they are stable, and we continue to manage them until they finish their treatment.

I: Mmm

P: I don’t if I answered you relevantly?

I: Mmm do you work at the facility, provincial or district level?

P: At the district level.

I: Ok. Umm what can you tell me about ASCENT? What do you know about ASCENT?

P: ASCENT?

I: Mmm

P: I don’t know much about ASCENT, but I am aware of xxxx (organisation name).

I: Mmm

P: I don’t know if I can- I don’t know the difference between ASCENT and xxx (organisation name) but what I know is xxxx (organisation name) is like an NGO and together working with the Department of Health in support of TB patients; they help to monitor patients on TB treatment.

I: Ok. Mmm, you mentioning the monitoring of patients-TB patients. Can you tell me more about how do they monitor patients?

P: What they do is when a patient is initially admitted, I will admit a patient on my side and handover the patient to the research assistant from the xxxx (organisation name) and that’s where a patient gives consent to be monitored on treatment. So, the monitoring happens like this; they give a pillbox, and they will tell them how to use it and reset the pillbox and patient put their medication on a pillbox, and it assist them with a reminder on daily basis on when to take their treatment so that they don’t forget to take their treatment. And also, there is a reminder to come back to the facility for review and in other cases it helps where a patient is not opening the box and then you know that a patient is not taking medication for that day and can easily phone the client and find out why they didn’t take medication.

I: Ok. You are mentioning the pillbox reminding patients to take their treatment and when they should come back for their appointments.

P: Yes

I: But do you know exactly how this box reminds them?

P: Yes. Ok I’m sure about the colour of the lights when the alarm goes off to remind them.

I: Mmm

P: But what my clients tells me is that there is a certain colour that reminds them-like you must open the box and take medication and on appointments dates it rings differently compared to like when you must open the box to take treatment.

I: Mmm

P: Mmm, so I think there is two different alarms; one is for opening and drinking medication and the other one is for coming back to the clinic to get treatment.

I: Ok. Besides the box, do you know any method or DAT (Digital Adherence Technology) that is being used to help or monitor patients?

P: Yes, I do.

I: Mmm-

P: Ok. Currently the system that we use to monitor or track patients neh, mmm we have the booking system.

I: Booking system yes.

P: We book our clients and then on daily basis, everyday in the morning we go through our booking system, and we retrieve the files of the patients we are expecting for that day and we see-if the patient missed the appointments, we are able to take the file and call the clients and find out why they missed the appointment. And also, even on Tier.net system, if a client missed uh appointment, it appears as early missed or late missed.

I: Ok but do you know any technology that is used besides the box? Are you familiar with any other forms of DAT that are being used? The one called labels or stickers. Have you ever heard about the labels or stickers?

P: No, I haven’t.

I: Oh ok…Umm…and then you were telling me about patients you were using the box and you explained that the box reminds them to take their medication and it helps them to remember their appointments date. Do you know another way they are being reminded by the box?

P: Another way they are reminded?

P: Yes, another way they are reminded by the box? You mentioned uh the lights and the alarm. Do you know how else they are reminded by the box?

P: I don’t think I understand. What do you mean do I know any other way the box works except for the alarm?

I: Except being reminded to take their medication and coming for their appointment’s dates?

P: Like any other duties of that box?

I: Yes.

P: No, I don’t know.

I: Mmm, you never heard of uh the SMS?

P: Uh I haven’t heard.

I: Ok…mmm you said patients are being monitored. How exactly are they monitored, beside the box? What else is being used to monitor them?

P: Ok. I don’t exactly how the system works neh, but I will explain what I think is happening neh.

I: Yes.

P: She’s got uh-the research assistant will have a phone with them and I’m not sure if it connected to the pillbox neh, so that if the patient is not opening the pillbox, it can then notify the research assistant to say this client did not open the pillbox on this day.

I: Ok. And do you know exactly how do they see when they have opened the box and when they have not opened it? On the phone as you said they see on the phone.

P: I haven’t seen.

I: You haven’t seen it?

P: Mmm

I: Ok, ok. Please describe your role in the differentiated model of care like the follow-up phone calls and home visits. What is your role on that?

P: Ok. Like in cases where maybe a client missed an appointment?

I: Mmm

P: Ok. First thing when my client misses an appointment neh. For that day I will be aware that a patient didn’t come for their follow-up visit neh-

I: -Mmm

P: I will try to call either that afternoon or tomorrow morning neh. I will make a phone call-I have a challenge, sometimes I call, and I don’t get them on their cell phones, but we also take next of kins contacts neh and if I don’t get the next of kin which happens most often. Then I make a referral to WBOT team members neh. They go door to door deliver CCMDD (Central Chronic Medicine Dispensing and Distribution) medication and other things, they go and give support to clients. So, I make a referral and write the patients address and they go trace the patient physically at their home. Also, we do sometimes have a challenge where a patient provides wrong address, then I also escalate the referral to… {nurse’s name} he is a TB nurse working for the Department of Health. They also assist we tracing clients.

I: Ok. And then what happens to those patients who have been found- you have been traced and found?

P: Who have been traced and found?

I: Yes, what happens next?

P: Ok. They are being uh linked back to the facility and then we check for how long did the patient missed the treatment. If it more than two months, obviously a patient will restart treatment-we going to restart TB treatment-we going to restart examinations and restart TB treatment but if it less than two months then we are going to extend TB treatment with the days the client missed. Then we emphasised uh the importance of adhering to treatment and the completion of TB treatment.

I: Ok. And mmm…what happens to a patient who has missed a dose? What did the intern normally do if they see that uh a patient missed a dose? Do you know what action is taken if say uh a patient missed the dose as you said the intern will see on the phone that a certain patient missed the dose?

P: Ok. Mmm I’m so sorry for this negative report neh but this is what has been happening neh. Sometimes she would call the clients neh, then put the file there neh, like here. And then I’m thinking she did it just to follow up the client the next day. Then maybe forget to notify me so that I can also try to trace the client cause I can also try something to trace the client and link them back to the facility. So, she did call neh [knock on the door] and she puts the file aside and not keep it in the filing room. She sometimes forgot to notify me so that I can also try other means of tracing clients.

I: Mmm

P: That’s the experience we had. [pause] I want to ask something neh. I saw that the research assistant would trace the patients but I’m not sure if they are allowed to write on the patient’s file that they traced the clients because it shows that you did something trying to link the client back to the facility. I never saw them write it down that at least they did something to trace the client, so I don’t know if they are allowed to write on the patient’s file. [pause]

I: Ok. So, when you first heard about the digital adherence technology what were your expectations about it?

P: When I first heard about?

I: The digital adherence technology before it was being implemented in this facility. What were your expectations about it?

P: Eish

I: What did you think about it? What were your thoughts about it?

P: Ok. My answer to that question is I already found it here, so I never had that thing in mind of thinking about like it like how it going to be and how it going to help because I found it already in place when I came in here.

I: Ok.

P: So, it not something which I knew about it before it was brought to the facility. I found it here, I don’t know if I answer your question?

I: Yes. Ok since you came here and found this DAT, what are your thoughts about it [door slams] [pause] do you find it useful or?

I: Ok. [phone rings] ok I find it useful neh if the clients are not abusing it or using it the wrong ways because some of the clients come here and be like “no this box is making a lot of noise and I just open and close it, then it won’t ring again”. So, if the client is responsible for their own health and they want to take the treatment and finish it then, it useful for them but some clients find it annoying, so they would just open the box and close it, so it doesn't continue to ring. So, I don’t find it not beneficial for that kind of client with that kind of attitude but otherwise it a very useful device to use and to monitor clients on treatment. So, it depends on the attitude of the client, how they use it at home because we don’t see them and when they open the box you just assume that they take the medication. Only to find out that they just open and close it just to keep it quite.

I: Ok. Mmm how often do have cases of patients who come forward and report that the box is ringing nonstop?

P: Sorry [ pause].

I: Mmm I was asking how often do have cases of patients who come forward and report the ringing of the box nonstop and annoying them?

P: Out of 100% I can say about 10% of clients complain that the box is malfunctioning. I’ve had few cases of patients who come and say “the box, alarm is ringing every now and then and not even when I’m supposed to open it” like they set it for six o’clock then it continue ringing past six o’clock. I don’t know what happens in cases like that, but I would ask them to bring it back and then ask the research assistant to reset it and they take it back home. I think they managed to reset two and the other one ended up saying “I don’t want this box anymore, it making a lot of noise” but it few cases not a lot.

I: Mmm do you know if the malfunctioning of the box still happens or if it was fixed somehow?

P: … It been a while now, maybe it been three months now without having the same complaints. So, I’m hoping the boxes which the clients currently have are working in the right way otherwise they would have reported.

I: Mmm ok. So, you said you started working here last year?

P: Yes.

I: If I may ask, did you receive training on DAT?

P: Sorry did I receive what?

I: Training on DAT or the box?

P: No, I didn’t receive; I was not trained on the box.

I: Ok. So, how do you know about the DAT? Who trained you- who told you about it?

P: Ok. So, when I came to this TB room neh, I just found the research assistant sitting there and I greeted her and introduced myself and asked what is her role in the TB room and she told me that she is a research assistant, and she gives pillbox after signing consent forms. It reminds them when they are supposed to take medication and when they must visit for follow-up, and it also help her to check if they take medication. What I know about the box is what they told when I asked them, but I never received training.

I: So, when the research assistant was telling you about the box, did you find that information enough?

P: It was not enough because I still don’t know several things about the box.

I: Mmm ok…Mmm do you have any suggestions on how the training can be improved?

P: You mean the training for research assistant or for us as the staff members?

I: Training for you as staff members.

P: Ok before suggestion, can I ask a question. Do you also train us on how to use the box?

I: Yes, staff members who works in the TB room as well as research assistant receive training on the DAT intervention before being implemented to any facility that is selected for the study.

P: Ok. I think sister {nurse’s name} got trained before she resigned in August the 31^st^ neh

I: Mmm

P: Then I started on the 31^st^ of September here but since I got here in September, I never got any training.

I: Ok if you were to receive training, what do you think you should be trained on?

P: Mmm on how to reset it for the client neh, like reset reminders for follow-up visits and also for taking medication on their daily basis and I also have a consent about the battery life of the box. Does it last for six months? I also need a clarity on that and how do you get notified and how are you notified if a client missed an appointment. Mmm.. I can’t think of anything else.

I: Mmm and how long do you think this training should be?

P: Mmm I think it can be done in one day.

I: One day. And then uh who should attend-who do you think should attend this training?

P: The TB nurse, there is a need for TB nurse to know and attend the training and the counsellor working with the TB nurse…and also the research assistant. And also, other professionals nurse assisting in the TB room in the absence of the TB nurse.

I: Ok. Can you tell me of the benefits of the box that you see in this facility?

P: Ok. The benefits of the box is that patients don’t miss time to take medication and it also reminds them of the appointment date if they happen to forget.

I: Mmm ok.

P: Those are the benefits of the clients. The benefits for us as health care workers is that we know when a client took their medication or opened the pillbox, and we are also able to trace patients who did not open their pillbox meaning they did not take their medication.

I: Ok. Mmm, do you know of a patient using the box who might have experienced stigma?

P: Stigma with box?

I: Yes.

P: No.

I: [ knock on the door] Mmm can you describe any challenges you might have so far with the box?

P: The only challenge we had is of those few patients who would come and say “the box is ringing even when it not time for me to take medication neh [ knock on the door] or the other one came and said “your box is not ringing, I waited, and I even set an alarm on my phone, but it didn’t ring. I even waited for 15 minutes hoping that it will ring but the alarm didn’t go off, so I just took my medication 15 minutes later”. So, I’ve had that incident and I also had an incident when the box was ringing even when the patient has opened the box and took medication.

I: …So, do you know of any challenges with follow-up phone calls? You said an intern would call if she saw that a client has missed their dose. Are there any challenges with follow-up phone calls or reaching them?

P: She was able to reach them but unfortunately, we are not able to get hold of others because you will find out that their phones are on voicemail, then we have to physically trace them via WBOT (Ward Based Outreach Team). But she would trace and find most of them and they promise to come-they rearrange the visits date.

I: Ok. Uh do you have patients who maybe refused to take the box.

P: …No I’ve never had patients who refused the box but what I have observed is that patients who cant read and write don’t qualify to receive the box.

I: Ok. Do you a certain group of people who are struggling with the box like homeless people and drug users?

P: You mean do I have clients who are struggling to use the box?

I: Yes.

P: No, I don’t have clients who are struggling with the box. I think she explains the instructions so clear that that they don’t have a problem using it.

I: Mmm

P: When they leave here, they understand what is expected from the box that they were given.

I: Mmm earlier on you mentioned physical tracing which is done by WBOT (Ward Based Outreach Team) -which is normally done by WBOT.

P: Yes.

I: Uh do you know if maybe there are some challenges with tracing patients uh do they encounter any challenges?

P: They never mentioned any challenges besides the issue of wrong addresses but if the address that was given by the clients is correct then they are able to trace them and deliver the message to relatives sometimes and the client is linked back to the facility,

I: Mmm why do you think patients give wrong address? Why would they give wrong address?

P: I don’t want to lie, I emphasized to patients all the time that they must give correct address and contact details so that we are able to trace them, but they continue to provide wrong details. I think maybe they don’t want to be traced or get home visits. Maybe it is the stigma around TB; maybe they are trying to hide their TB status to their loved ones. I’m thinking that could be the reason.

I: Mmm what do you think can be done to avoid this issue of wrong address?

P: Ok. I spoke with the supervisor of the WBOT team recently and she advised me to try the strategy of getting contact details and address of a client on the first visit and refer immediately to them so they can start regular visits right away in order to avoid wrong address from the onset. So, that if a client gave the wrong details on the first visit, then we are able to correct it on the next visit. We tell the client that we are aware that you gave wrong details, please give us the correct ones so we can have WBOT team members come and give you support at home and remind you of your appointment dates.

I: Ok.

P: Mmm

I: Mmm you mentioned something about stigma neh, you said maybe the reason for patients to give wrong address is because they are afraid that certain people might know of their TB status hence, they don’t feel comfortable with home visits. Uh what do you think can be done to help or deal with the issue of stigma because maybe it not only this facility but there may be other facilities that are having the same issue of stigma?

P: Ok what can be done to try alleviate the issue of stigma is to give clients education neh, teach them about TB and how it transmitted, and also inform them that TB is curable if you take and complete treatment. I think another thing we can do is to…encourage TB clients to have TB buddies at home so that they can give them support. So, that they know how to take care of themselves at home to avoid transmission, but the best thing is to give them health education so they know more about TB.

I: Ok. So, from your perspective do you think DAT can be used to improve TB treatment?

P: You mean the box can be used to improve adherence to treatment?

I: Yes.

P: Yes.

I: Ok. Could you please explain a bit further on that?

P: When you give the box to the clients and they open it, you get the impression that they are taking medication but only if the client is responsible enough to open the box and drink medication then it very much helpful and we know that our client is taking medication in a right way.

I: Mmm ok. Before the box was being implemented. What other method were you using to see if patients are adhering to treatment?

P: Mmm I have no idea; this is my first time working in the public primary health care setting. I used to work in the hospital setting, so I’m not sure what was used before the pillbox to ensure that clients are adhering to treatment.

I: Mmm ok…mmm so uh in the absence of the research assistant how do you think the existence of digital adherence technology can be sustained in the facility? Uh what is needed to ensure that it operates the way it is operated at the moment?

P: Ok. I think then, the staff will need proper training on how to use the box so that when we admit clients with TB, we know how to issue the boxes and teach clients how to use them-so yah training is needed.

I: Ok. Please describe the level of structures that need to be improved in order to integrate the differentiated model of care with the use of the box.

P: I’m sorry you said the level of structures that must be improved to integrate-

I: - integrate the differentiated model of care with the use of the box?

P: I don’t think I understand.

I: Ok mmm I mean [cough] what structures can be put in place to improve the follow-up phone calls and home visits in integration with the box or DAT?

P: Mmm I’m thinking…we will need training, enough boxes to give to clients because we once encountered a problem where we didn’t have enough stock of boxes and at that time, we had clients who were willing to receive pillboxes but because we didn’t have pillboxes patients had to go home without them. So, we need training and enough boxes.

I: So, in case there are technical issues with the box who do you think should be put in place to help with that situation?

P: The sponsor of the box.

I: Ok. Who do you think should prepare the boxes because they must be prepared before being issued? Things like charging and everything.

P: Oh, I’m thinking we will need someone to assist with the preparation of the boxes.

I: Ok. Can you think of any other resources which are needed to ensure the smooth running of the DAT? What else is needed? You already mentioned training and more boxes. Can you think of anything else?

P: …Mmm…I have no idea.

I: Ok. You mentioned patients who were having issues with the box. Did you perhaps capture the complaints somewhere?

P: I think I wrote a few on the patients file when the research assistant was absent, and I would give it to the research assistant when she comes back to work.

I: Ok.

P: But if the research assistant is present, then I would refer them to her, and she would assist client immediately before they go home.

I: Mmm you said there should be someone put in place to assist with preparation of the boxes and everything. Who do you think should be assisting and should it be someone from the outside or someone here at the facility?

P: Yoh someone from outside like the research assistant who was here because she was very much helpful. It would make it easier for us because I can imagine if I have to admit a client, I can take about 45 minutes with admission alone. Then having to also handover the box to the client after consenting and explaining the procedure; it would be a very late admission for me. So, it will make things easier if I have someone assisting me. I can’t think of someone who’s already working here because they have their duties already, so it will mean extra job for that person to handover the pillbox to clients and having to deal with technical issues. Having a research assistant would make things easy and better.

I: Ok. So, are you saying the research assistant is the right person?

P: Yes, definitely.

I: Ok. Can you think of any existing gaps in the implementation of the box, and do you have any suggestions on how they can be improved?

P: Uh I would suggest that if a client missed any appointments and the research assistant knows, immediately inform us so we can trace the patient immediately because sometimes I would have clients who missed appointments and not able to see that on time. Then you find that the research assistant was aware that the client missed the appointment but didn’t inform me. So, it better if she let me know early so that I can also try trace the client or document on the file as well because it very important to know when client has missed an appointment. It helps us in rescheduling another appointment immediately and try to get hold of the client so they can get medication as soon as possible or increase the dosage on the next visit since they missed days.

P: Ok. Do you have any suggestions on how the box itself can be improved? Things like the shape and size of it?

I: Ok, I want to talk about the size neh because for patients who are taking five tablets of Rifafour it becomes difficult for them to fit all medication inside the box. So, they end up keeping other treatments outside the box because it doesn’t fit. If maybe the size can be a bit bigger than this one, then everything will be fine. The alarm system must also be improved as I mentioned earlier on that we had complaints from clients about the alarm ringing when it is not expected to ring and sometimes it doesn’t go off at all. So, improvement on the size and the alarm.

I: Ok. Do you have any final comments on the differentiated model of care and the box?

I: Yes.

P: Ok. Uh the box neh is very helpful for clients because most clients do come here and inform us that I very helpful for them and it reminds them to take their medication on time and also… Its a good way of tracing and knowing that our clients are taking medication or not. So, we are assured that our clients are taking medication correctly when we get the notifications that clients opened their boxes.

I: Ok. Thank you for time. The time is now 14:32 PM.

Glossary

WBOT -Ward-Based Outreach Team

CCMDD - Central Chronic Medicine Dispensing and Distribution

DAT- Digital Adherence Technology

Neh- Right
