# Supplementary material for: Acceptability of Digital Adherence Technologies to support people with drug-susceptible TB in South Africa
Source: PLoS One. 2025 Sep 24;20(9):e0332103. doi: 10.1371/journal.pone.0332103 (PMC12459780; doi:10.1371/journal.pone.0332103)
Supplement: S4 File — (ZIP) [file pone.0332103.s004.zip › S4 Transcripts/HCWs and Stakeholders/IDI 2-HCW.docx]

| **Label Key** | **Meaning** |
| --- | --- |
| **I** | Start of each new utterance by the Interviewer |
| **P** | Start of each new utterance by the Participant |
| **N** | Note taker |
| **{ }** | Indicates that details were changed or pseudonyms were used to anonymise data |
| **( )** | Indicates the description provided to anonymise data |
| **XXX** | Words were omitted to anonymise data |
| **-** | Breaking into a sentence by the next speaker |
| **…** | Pause or drawn out words |
| **[ ]** | Indicates noise made, e.g. [laugh], [sigh], [pause] |
| ? | Beginning of utterance by unidentified speaker or questionable text |
| **[inaudible segment]** | Unclear section of the recording |

I: So, do we have permission to record you?

P: Yes.

I: Date of the interview is: xxx (interview date). Location : xxx [clinic name]. The language used : English. The PID is xxx. The time we are starting the session is 11: 00. Thank you very much for agreeing to participate in the study. So, can you tell me what your current position here is?

P: Okay, I’m a Professional Nurse.

I: Okay.

P: Yes. Working as a quality assurance nurse.

I: Okay. So, how long have you had this position?

P: Huh, I started January.

I: January.

P: Yes.

I: Okay. So, what-

P: As a xxx (position) but being a professional nurse, I’ve started in xxxx (year).

I: Okay.

P: Yes.

I: So ,what are your roles and responsibility in TB care?

P: Okay, in TB care I do see patients who come in complaining of coughing. We start by screening them. Then we test- either we take patient’s sputum or send patients to do the X-ray. Then when they come back and the x ray or the sputum come back positive, that’s when we start the treatment.

I: Okay.

P: And the X-ray are reviewed by the doctor, he’s the one who tells us to start the treatment.

I: Okay. So, when you initiate a patient on treatment, what happens during that session?

P: Okay, firstly, we have to take the vital signs, when she comes in to evaluate the vital signs. Then after that we sit with the patient; tell her or him that the results came positive, or the X-ray came positive, so we have to start TB treatment. So, we explain to the patient about TB treatment. How long is she going to take the treatment? How many tablets is she going to take per day? And what time is she going to take the medication and we also add the diet there. What kind of foods that she can eat and to bring the other family members who are staying with that person, so that we can also screen them.

I: Okay.

P: Yes, and what else. Okay, we explain also about the box since we were using the box.

I: Mmm.

P: Yes, we explain to the patient that this box is going to help you with the time to take medication because some of the patients are from disadvantaged backgrounds. Maybe she doesn't have a phone or a watch with an alarm so she won't know what time to take medication as you know, TB treatment must be taken early in the morning.

I: Okay.

P: So, we tell her that we're going to set the alarm on the box. So, when the alarm rings, you must start taking your medication.

I: Okay, interesting.

P: Mmm.

I: So, how are TB services delivered at your level. Are you at facility level or district?

P: Yeah, we are at facility level.

I: Okay.

P: Yes.

I: Now that you started talking about the box. I would like you to tell me what you know about the ASCENT study. So, if you were to explain what digital adherence technology is to another healthcare worker, what would you tell them?

P: Wow the box *ey*. I will tell her that the box is so helpful with the TB treatment. What I can say is that before we use the box, we had the high defaulter rate. So, since we use the box, when I'm comparing the data from previous and now, we have much improvement in TB.

I: That’s interesting.

P: Yes, there is much improvement in our patients, because TB before, I don't know- it was like what can I say? It was discriminated. When you're having TB, it was like I don't know you're having something infectious that people are not supposed to be with you. So, since I started in TB treatment, we were fast tracking the patients when they come in and having that box, they feel so honoured, you know, it makes them you know, no, ?. So, it gives them that feeling that they're so important also in the clinic. So, it makes me… it made me happy anyway seeing that, you know, people now are taking TB like a chronic condition. They are not discriminating it from the other conditions. So, what I can say it really helped with huh, compliance and also with taking out the discrimination part from the chronic conditions, even other patients, not the TB patients only, they feel like maybe if we can continue with other conditions. Every patient we give that box, maybe it's going to be something better for the patients. Because with other conditions, people are forgetting to take medication. So, if you have something that is going to remind you that now you have to take your medication, I think it's going to be a good thing. Yeah.

I: So, what exactly has contributed to the lower defaulter rate and compliance? When you think about the box?

I: Yes, because it has an alarm. I think that's it. We also explain to the patient that if you don't take treatment, this thing is going to report to us, they know that if they don't take treatment, I'm going to know the minute that he didn't take it. So that makes them aware that no, I have to take the treatment. So that's why we don't have defaulter rates there. The compliance is very good because of the alarm ringing. So, if you have to take the medication, we tell them that even if you can open the box and close it and don't take the medication, I am going to know about that. So, they know that no, we have to take the treatment. You don't have to play so that's why we have a good compliance rate.

I: Okay.

P: Mmm.

I: So, you mentioned that huh, you know, when they take medication or when they don't take medication-

P: Mmm.

I: How do you know?

P: Okay the… we use the box the tablet, which is connected to the tower where the box and the tablet records when the patient opens the box, we connect them to the- to the database. So, it reports that patient so and so has opened the box, and it gives the colours- the different colours. We have different colours to know that okay, this one has taken the medication that's why the colour is green, and this one didn't take the medication the colour is red and maybe if this one took the medication but later. The colour is different. It's orange also. So, we can see that no, there are different colours that tell us different stories about patients.

I: Okay.

P: Mmm.

I: So ,what's the name of this system on the tablet you are using?

P: Mmm by the way-what's the name? (Laugh) I forgot the name, let me check the information that I have written here.

I: Okay. So ,can you tell me about the differentiated model of care?

P: Mmm. What do you mean by that?

I: Okay, so differentiated model of care is the follow up actions taken when you see that the patient did not take medication, a patient has a red calendar. What do you do?

P: Huh okay, so let's say this patient XXX [Patient’s name] didn't take her medication. Today's the first time that she didn't take her medication. We call her to inform her that we see that here in our tablet it reported that you didn't take the medication, what is wrong because maybe something is wrong where she is. So, she will explain to us why she didn’t take medication. Then if it happens, it continues for maybe continuous days, maybe three to four days. Then we send our outreach team to go and investigate what's wrong, because some patients don't want to talk. So, we have to send our team out there to check what's happening with that patient. Then they will give us the report when they found one, two and three, then we'll attend to that and we do also have a social worker in our team that is helping us with uh, social problems. So, if it happens that the patient needs more care than just uh, clinical care, maybe it's a social problem. Then we sent the social worker to go and attend to that problem.

I: So ,what are some of the common reasons why patients don't take medication?

P: Uhh, most people here are disadvantaged, they are poor. They will tell you that I don't have food and you can’t take treatment continuous days without eating. It's not going to treat you good. So, some of the problem is that they don’t work, so they don't have food, before this COVID thing, we had uh, what you call social relief where we give food parcels. So, after that things changed, but we also help them, if she comes and complain to me that I don't have food. I have to provide something to eat so that she can take treatment.

I: Okay. So ,what are other reasons?

P: So, most of them were taking treatment, the other ones would tell you that I'm staying in XXX [location], so I was visiting, maybe XXX [location], so I didn't take the box, but I did take the medication. So, that’s why the box didn't report that the patient has taken their medication ,but they will give us the assurance that no, the medication I did take, but didn't take the box with me to XXX (location). It’s where we see that okay, maybe it's true because we don't know, but most of them were loyal, I can say they were telling the truth.

I: So, what are some of the reasons why they leave the box behind, and not travel with it?

P: They say they are afraid maybe they can get mugged on the way, so they won’t take the box with that medication. So, they won’t risk the medication, or maybe it's a funeral thing, maybe there are a lot of people there, and maybe the box is going to get lost. They are afraid of that.

I: So, besides fear of getting lost, are there any other reasons why do you think patients leave the box?

P: Yeah, I think maybe they don't want people to see that they're taking treatment and they can't explain that I’m just taking medication with me not the box. The thing is that- I think they will-maybe they will treat them different because they know that they are on TB treatment. Because I once had a patient- a guy from XXX (location). So, he's visiting here- so he was afraid to take the box saying no, I know those people that I'm staying with. If they see this, then they know that I have TB. They can even throw me away.

I: So, how can this issue of stigma be addressed?

P: So, now with all these conditions, we try to normalize it like, like every condition there is-chronic. We treat it as the same. There is no difference between the conditions, because now we are having treatment for all these conditions as long as the patient is taking treatment and stay away for certain time. So, you don't have to worry that I'm going to get this condition from this patient.

I: Okay.

P: Mmm. So, it’s how we try to stop the stigma because you can see since the boxes, there is not that much stigma about TB, it’s being treated like a normal chronic condition.

I: So, uh, back to the follow up actions you take, you said you make a phone call. How is the patient reminded before you make the phone call?

P: Okay, even the- the boxes we tell them they're going to change the colour towards the next appointment. So, we tell them that you must know when the colour changes that the medication is going to get finished. So ,you must come to the clinic and we also call them before like maybe a week before to inform him that, are you aware that next week it’s your appointment date, so that they mustn’t forget.

I: And then once the patient misses the dose before you call them, what happens?

P: Before we make the phone call?

I: Yes.

P: Uh okay, let's say-

I: How are they reminded at first?

P: At first?

I: Yes.

P: Before they come to the clinic for follow up?

I: Before you made the phone call.

P: Let's say maybe this patient didn't take their medication today.

I: Yes.

P: And we didn't call-

I: Yes-

P: to inform him. What's your question there?

I: How are they reminded? Is there any other way they are reminded? Before you call, you make a phone call?

P: Uh, most of them are advised to bring family members with them when they come for treatment, so that that person also can help us with the treatment, because we know that when you're sick, sometimes you forget. So, we ask them to check the medication if they stay together because some will say no, I’m staying alone. So, if they stay alone, so it's going to be difficult, but if they have somebody in the family, we ask them to please help the patient with the medication or remind him because sometimes they forget.

I: So, do you know about the reminder SMS?

P: A reminder SMS?

I: Yes.

P: Yes. I was forgetting about. I know that they send them the SMS, and most of them they complain that you know I've taken medication, but I’m seeing this medication saying, no, I didn't take the medication. So, we reassure them that no, don't worry about that. They just wanted to remind you that you mustn’t forget to take your medication.

I: So, what are some of the reasons why they get the reminder SMS? Yet, they got… they took the medication.

P: Some say they took the medication, but I don't think they can send them the remainder if they didn't. So, we just reassure them.

I: Okay.

P: That you know if they send you an SMS, if you have took your medication, don't worry, just ignore it. But *wena* (you) make sure that you take the medication.

I: Okay. So, uh, on the tablet, how do you know which patients to follow up on?

P: Okay they- they do have different colours as I say, the ones that are complaint are going to be all green most of the month. So, we check the colours. If this one is having most of yellow, most of orange or most of red, we prioritize, because the red ones are the ones that are going to have a problem because they're not complying. So, we start with them and attend them first, before we can go to the orange ones because the orange ones are taking it even though they don't take it the same time. But we all inform them that you know when you take your medication because it causes a chain. The minute you start changing your time, you open a gap and it's where you're going to have a problem because then the condition is going to multiply, then you're not going to get healed sooner than we are expecting you to get healed, but they do understand.

I: Okay.

P: They do comply after talking to them, you can see there are going to be some changes.

I: Okay .So do you know about the task list?

P: The Task list?

I: Yes.

P: The task list, but most of the time they were being done by the person who was having the tablet most of the time, she would come in the morning and explain to me that today we are expecting so and so, and tomorrow maybe it’s how many patients, then it's when I attend to that.

I: Alright, so can you tell me what your role in these follow up actions when it comes to phone calls, and also home visits. What's your responsibility?

P: We do have a committee- we formed a committee, so that we help each other because it is not only Aurum that is supporting us in TB. We also have IRD also. So, we sit down and discuss the patient, because we have the outreach team, and we have a social worker as I said. So, if we see that this kind of patient need to be followed up from their home. So, we sit down and negotiate with them, because we serve different places. We have this site, this site, and this site. So, we then share as outreach team members depending on how many patients we have. We share equally most of the time.

I: Okay.

I: So, when it comes to home visits, your role is to allocate who is going where?

P: Mmm and then after they come back and give me the feedback, that from this patient we get 123 from this one, we get 123, then from there we then sit down and discuss what's the way forward depending on the outcome and situation of the patient.

I: And then when it comes to phone calls, what's your role there?

P: Yes, I do make the phone calls. I do make the phone calls, because they telling me this and this and this is the one that needs to be followed up. So, I'm the one who is making the phone call.

I: Okay. So, which follow up action do you do most of the time? Is it the phone call or the home visits?

P: We do them both-

I: Mmm

P: Depending *huri (that)* on how is the patient responding, because some of them you know, since we are next to the hospital and next to the mall, they give us the wrong address because they wanted us to help them. They are not from our area. So, when you go for a home visits from the address that they have given you , you find out that people don't know this patient who gave us this address, and sometimes when we call and that’s when she's going to explain the story. “No, I didn't give you the right address because I was afraid that you're going to turn me away not helping me.”

I: Mmm so how can that be resolved? Of patients giving wrong addresses.

P: *Eish,* It's a big problem because with this demarcation, we use demarcation to help the patients. Some they say no, *nna (I)* I don't like the clinic from where I come from.

I: Mmm.

P: And I don't want them to know that I’m sick with this condition. So, it's a problem and I don't think it’s going to be sorted.

I: What are the reasons why they don't want to use their nearby clinics and they don't want to be seen?

P: Because of the stigma.

I: Stigma, can you tell me more about that?

P: Because- let me say maybe I'm staying here in XXX [location] and my neighbour is a nurse. She's working there in XXX [clinic name] clinic. When I go there, she'll see me, and as a patient, I will think she's going to tell people that I'm sick.

I: Okay.

P: Mmm.

P: That’s their problem, they think I'm going to tell the community about your condition-

I: Mmm-

P: And which is not what we're doing.

I: Okay.

P: Mmm.

I: So, when you first heard about digital adherence technology, what were your expectations?

P: Firstly, I didn't understand how it's going to work. I had my doubts about it and I wondered if it’s going to work, is it going to improve our situation. But after seeing that this is really working.

I: Mmm.

P: Yeah, I've seen that. No, this is a way to go.

I: Okay.

P: It's a way to go. It's really helping.

I: So, has your opinion changed after we started implementing it?

P: Yes.

I: How so?

P: Because we were using it; I can see that this is helping

I: Mmm-

P: Because it's monitoring the patient while I'm here, and the patient is at home. But I can see the- the activities if the patient is taking treatment or not because if it wasn’t for this device, I wouldn’t know if she doesn't take it. And when he comes back, he tells me, no I've taken treatment, but he didn't do that and then we will… we will be surprised to see there is more MDR’s- we'll see more deaths. You see.

I: Mmm.

P: Mmm.

I: Okay. So, can you tell me about the training and the resources you received before the digital adherence technology was implemented?

P: The training about TB?

I: About that digital adherence technology?

P: Okay-

I: Did you attend?

I: Yes, we did attend the training in XXX [location]-

I: How was it?

P: It was good. It was good and it was understandable.

I: Okay. Did you think it was comprehensive?

P: Yeah, it was comprehensive.

I: Do you think it was useful?

P: Yes, very useful.

I: Okay.

P: Because it was the first time seeing the device.

I: Okay.

P: Mmm.

I: So, besides the box, which other digital adherence technology do you know?

P: They say it was a pill box and uh, the envelope thing *ya (yes)*, they were talking about two things, the envelope thing (labels), and this pillbox.

I: So, do you remember how the envelope works?

P: No. They didn’t go deeper into envelopes because they grouped the facilities, my facility had the box one. So, they didn’t explain much about the envelope one.

I: Okay. So, do you have any suggestions on how training can be improved going forward?

P: Yes, maybe if they can start by choosing nurses that are working in TB, initially we just went, we didn’t know who is working at TB. So, they must choose the nurses that are working at TB.

I: Mmm.

P: Because they are the ones who understand how it works in TB treatment.

I: So, who do you think should do the trainings going forward?

P: It must be nurses, because they are the ones who are dealing with the patient.

I: Mmm.

P: Mmm. It must be nurses.

I: Okay. So how long do you think the training should be?

P: Uh, 2- 3 days.

I: Okay.

P: Mmm 2-3 days, because some people don’t understand the same way, some are quick some are slow. If it’s one day, I will say I understand, but when I get to the facility. And sometimes I’ll be stuck and feeling like *yoh,* I can’t ask them. What they were saying, but if it’s 2 days, if I’m slow, I will pick up along the way.

I: Okay.

P: Mmm

I: What do you think you should be trained on? The content?

P: The content must be about the-the tablet and the box and also about the treatment.

I: Okay.

P: Mmm, they must also include the treatment.

I: So, from your perspective as a TB nurse, can you tell me the benefits of uh, using the digital adherence technology?

P: It really benefitted us in improving our stats in TB, because it reduced the default rate, it increased the success rate of the TB treatment, and it also reduced the deaths that we had in TB.

I: Okay.

P: Yes.

I: And when it comes to monitoring, how has it impacted how you monitor patients?

P: Even the monitoring of patients, as I have said earlier [inaudible segment]- as I was saying [pause].

I: You were talking about monitoring-

P: I can monitor the patient while I'm here at the clinic and the patient is at home, before we didn't know the activities that were happening with a TB treatment and the patient while we are here. So, with these new devices, we can see that the patient is taking treatment on the right time, and if it's not taken at the right time, I will know that no, patients so and so didn't take treatment at the right time. And even when he didn't take it at all on that day, I will see that no, this one didn’t take it totally for that day. So, before it was hard.

I: Okay. So, has this improved the relationship you have with patients?

P: *Ya (yes)* it did a lot.

I: How so?

P: The-the relationship. It was like you know, different and I think it's because of the box because they know that sister is monitoring me, even if I'm not at the clinic, she knows what's happening with me and my medication. So they have to be compliant. And even if he's having a problem, he can call, “no sister don't be surprised if you see anything funny on the tablet because I'm having a problem with 123.”

I: Okay.

P: Mmm.

I: So, what are some of the problems they report when they call?

P: Sometimes somebody has to go somewhere. They didn't plan that trip, maybe it’s an urgent thing. He had to go somewhere and didn't have the medication with him. So, they will explain to you know, I'm going somewhere. They called me, so I have to be there today. Maybe I'll come back tomorrow, so I can’t take medication. Don't be surprised if that thing reports that I didn't take medication.

I: Okay.

P: Mmm.

I: So, how were you monitoring adherence before the technology?

P: It was difficult. It was difficult because you have to believe whatever they tell you and you can see that no man this one is not taking medication, because the condition is not improving. But he tells you no, I'm taking my medication every day and they end up having MDR.

I: Mmm.

P: Mmm and before they were giving them a grant for TB. So, these people (patients) didn't want to lose that grant because they're not working. So, they wouldn’t take medication so that he doesn't get healed and prolong to be on TB treatment so that they can get something.

I: So, how has digital adherence technology changed all that?

P: The?

I: The issues you were having, difficulty with monitoring?

P: Like with this grant thing, the government also stopped it, because it was a bad impact, because we were having more death rates than the success rate. So, they stopped the grant, so that people should not get the grant. They were giving them food parcels instead. So, it was better, but it was not like completely helping because some just don't take medication, so that they can also get that food parcels.

I: Okay.

P: Mmm.

I: Then what are the benefits of the differentiated model of care? This follow-ups you're able to make-

P: Mmm

I: The phone call within a day, home visits after three to four days. What are the benefits?

P: They do benefit us. As I say, our success rate has improved. Even our…the patients who complete their treatment on time. So, this really helped us a lot.

I: Okay. So, can you tell me the challenges you've had with the differentiated model of care? The follow-ups?

P: There were not much challenges. I can't remember having a challenge because you know, we had the phone to make a phone call when we need to, and the outreach team to support us to go out if we can't reach this person. So, we didn't have challenges as such.

I: Are you able to get hold of every patient you try to call, or you try to visit?

P: So, on the list that we had, we didn't have anyone who was giving us a challenge, of not getting hold of that patient, all of them that we’re registered on our system, we could get contact with them.

I: Okay.

P: Yes.

I: Okay, you once mentioned people giving wrong addresses?

P: Yes.

I: Has that happened?

P: We did have one that was not from our area, but we got hold of him on the phone.

I: Okay.

P: We did find him, and he was attending the clinic, yes because he didn't want to be at his place attending his local clinic. So, he didn't absent himself until he finished treatment.

I: Okay.

P: Mmm.

I: So, do you have issues with acceptability, are there patients who declined to take the box when you introduce it to them?

P: Yeah, some said no. They don't want that thing. And most of them were old people because they don't understand technology. They just say, “just give me medication. I'm here for medication. I don't want that thing of yours.” So, we do explain to them that it’s going to help you, they say, “no, I'm fine with medication only.” So, we do take it as a good thing because we can’t force patients to do something that they don't want to, and we understand that it’s age.

I: Mmm besides age, what are some of the reasons why they've declined?

P: Uh, here most of them are young people. They didn't have problems.

I: Okay.

P: They didn't have a problem.

I: So, has there been network issues in relation to the use of the digital adherence technology?

P: Mmm-mmm (yes).

I: Yes, can you tell me more?

P: Sometimes we do have a problem with the signal because of -of network and this electricity problem of black outs sometimes. So, we do have problems sometimes the- the lights are going to be different colours, whereas the patient tells you no *nna (I)* take the medication. Because where I'm saying there is no network, maybe that's why because most of them they live in informal settlements and there is no electricity there. So, you know the network is going to be a problem.

I: So, how does lack of electricity and network affect the way the digital adherence technology works?

P: It affects it because of the signal. Sometimes if the patient took the medication and the signal didn't report that this patient has taken medication, it’s going to give me the wrong colour. And I will assume that no, this one didn't take medication whereas the patient took it. That is why they say even the SMS, they send us SMS’s whereas we took the medication. So, it gives us the wrong idea. Whereas the patient has taken the medication.

I: So, how do the patients react when you follow up on them?

P: Sometimes they are angry and say, “no, you make me a bad person. I’m telling you that I took medication and you’re saying that thing and says I didn't take medication.” They get angry sometimes, but we do talk to them. We say no it's not us because we check the tablet, and it is the one that is talking to us. So, we do apologize because as you say maybe it can be network problems. So ,we do agree with you that you know it's a network problem. So, we do apologize.

I: Okay. So, have you had issues with patients who have no cell phones?

P: Yes, we do. But luckily other family members do have cell phones where we can call the patient, because some people aren't working and can’t afford to have a cell phone. But if a family member has a cell phone, we use for family members, so that you can get hold of that patient.

I: Is it easy to get hold of them through the family member?

P: If they stay in the same household, it's easy, but if they say no, *nna (i)* I’m staying very far from her it’s going to be a problem, because they have to take a trip to give them the phone so that we can talk to that person.

I: So, have you had any challenges with that?

P: Yeah, there was the other one. He was not staying with that person who gave us her number, yes, they were staying far from each other, when we call, he said, “no I'm not at home. I'm at work and she's at home, but I will give her the message when I get home.”

I: So, do they end up giving them the message?

P: Yeah, they do give them the message, because maybe we call to remind her that no, next week is your appointment. She will come and say, “no, she gave me a message that you called.”

I: Okay and then the issue of stigma. Have you had any reports about stigma related to the box?

P: No, not at all

I: Alright and is it only people leaving the box for the fear of stigma?

P: They leaving the box?

I: Behind?

P: No, I can’t say they feel the stigma, sometimes I can see that these boxes were making them feel more respected because we give them the first preference to be seen in the clinic. They feel like when they have this thing (pillbox), they know it’s in and out. So, I'm not going to stay long in the clinic. It's like their passport for in and out.

I: Okay. So, do you have a certain group of people who you find difficult to support using the boxes?

P: With the group that we had, no, we didn't have any problems.

I: For example, a homeless person or someone that is abusing substances?

P: Yeah, we had one who was using drugs. Uh, that person ended up dying. He was staying on the street, but his mother would find him and bring him for his appointment date, but he will tell us that you know what it's difficult for- for him to take medication because sometimes he doesn't come back home, his in the street.

I: So, did you give this patient a box?

P: Yeah, we did because when he came back- when he came for the first time, we didn't know that he's using drugs. We knew after some few visits that he's on drugs.

I: So, how was his calendar like?

P: It was having more red than green. It was having more red than green. That's why he ended up being demised.

I: Okay.

P: Mmm, because the treatment was not working the way it should. Because most of the time his on the street and doesn't take treatment and even with food, you wouldn’t know if this person is eating okay, or what.

I:So, how do you think these patients can be supported going forward?

P: I think we can have uh, I don't know. We must find something like… we find the spot where they stay, because I know they have spots where they stay, they are not scattered here in XXX (location) I know they have a spot where they live. So, if we can take medication and maybe food parcels and go there to support them. And I don't know if we encourage them that you must go back home because living in the streets is not good. Then give the ones who are sick medication and food. I think maybe it can help. It can open their minds that they should try to go back home to live a normal life than to be on the street.

I: So, do you think the box can still help them in addition to these other supports you’ve mentioned?

P: like yeah, like you know what with the box, I can maybe think of a way to award them. We tell them that if you take medication, this box is going to tell us that you're taking medication and we want you to do 123 maybe that thing can work.

I: Okay, alright. So, do you always have staff to do home visits after three? Four days?

P: We have an outreach team. We don’t have a problem with that.

I: Alright that’s good then. So, from your perspective as a TB nurse, do you think TB treatment can be improved using the digital adherence technology?

P: Yes, I do.

I: Okay, how so?

P: Because with technology, like with this box and the tablet, it showed that we can monitor them being away from us. It's really making a good impact on our stats.

I: Mmm

P: Really, we have better result from the previous years that we didn't use the pill box. So, I think if we improve even the technology, our lives can be much better.

I: Okay.

P: Mmm.

I: So, how has it impacted your workload? As a nurse, as a TB nurse this digital technology?

P: Uh, the work- I can't say it's a given me more work, because I'm not working alone. I'm working with the intern who is the one- who's enrolling them on the system. After enrolling them, then he brings them to me so that I can start with the clinical assessment and the treatment. So, it was not that much. Actually, it was not that much. The only thing is that you have to explain how it works, even if the intern did that part, but you also- we have to emphasize, so that the patient can be more aware of that because when the patient are told by some other person other than the nurse, they don't take it seriously, they wanted to hear from you as a nurse.

I: As a nurse?

P: Yes, so when you emphasize, then they will see that it's from the nurse meaning I have to do that.

I: Okay.

I: So, you mentioned that there are patients with other conditions. Do you know if they only keep TB medication there or if the digital adherence technology is assisting with other conditions they have?

P: No, some were putting other medication and with our patients here most of them are HIV positive. So, they put their ARV’s there because there is this combination. If they have HIV and TB at the same time, they have to take uh, double the dosage of their ARV’s, so they were putting the TB medication there, and saying, “no I'm taking it at the same time as the TB treatment.” So, I think it's better if I put them together.

I: So, do they find the box useful for both conditions?

P: Mmm because it's having an alarm, so you won't miss the time for taking medication. It was the helping for both conditions.

I: So, what's your suggestion on that going forward?

P: My suggestion is that if they can also take the box to other conditions not only for TB, but for other conditions. I think it's going to be better, because you find a person coming in- you've given that person medication for a month, meaning its 28 tablets. They come in for a check –up and say, “no my medication is finished.” How is it possible? “No, but I took as you say, one per day” whereas maybe she forgot, and she took twice. So, if the alarm rings, they will know that I have to take medication. But if they don't have something that reminds them, you see, they're going to take medication twice, whereas they have to take it once. So, I think if they can take it to other conditions, it can help.

I: Okay.

P: Mmm.

I: So, before the box with the alarm. How were patients reminded to take medications?

P: Most of the TB treatment, I advise them to take medication at six.

I: Okay.

P: All of them, I don't want a different time. So, I was thinking 6 because some they'll tell you no, “I'm working, but at 6 I’m at work or maybe I’m 6am waking up.” So, it was a convenient time for both patients.

I: 6pm or 6am?

P: Am, *akere (*you know*)* you take TB treatment on an empty stomach. You're not supposed to take it after eating. So, you take it in an empty stomach, and some at 7 they started to eat. So, if they take it at 6 so they have to stay 30 to 1 hour before they eat. So, it was a convenient time for them.

I: Alright.

P: Mmm.

I: So, can you tell me about the positive changes that have been brought by the box?

P: The positive is the cure rate. Most of my patients have been cured and the defaulter rate has dropped. We don't have that alarming number that we had before the pillbox.

I: Okay.

P: Mmm.

I: So, what can be improved at the facility level for us to… for this to be sustained?

P: *Shoo (wow)*, with our facility, if you can just continue not stopping the program, the program- the program I think it must just continue. I think it's doing more good. Our patients are free to take TB treatment compared to previous times. So, I think it's a good thing so that we can sustain this thing. Just continue with it, and maybe even extending it to the other two conditions. I think it's a good thing.

I: And can you tell me some of the negative changes that have been brought by the digital adherence technology and the follow ups?

P: Uhh, the negative thing I can say is that because we give our patients first preference. Some other patients feel like no, I've been here before this person and this person just gets in. [Phone beeping] [pause] whereas I've been here maybe hour before. So, I think that one is giving us a negative impact on other patients with other conditions, because I'm not seeing TB patients only, I’m helping other patients. So, if I've chosen some patients and say no, I will help you, but if a TB patient comes. I tell them that I'm going to stop and attend this one. So, it's, I think it gives a negative thing because they think we are avoiding to help them. We prefer the TB patients.

I: Okay, when it comes to the follow ups, you mentioned that some SMS’s are wrongly sent to the patients and sometimes you call a patient who already took medication-

P: Mmm

I: How has that negatively impacted them?

P: They feel that we don't believe them, but we try to counsel them that it’s not that I don't believe you. It’s because we are using technology, technology can sometimes make some mistakes, so you don't have to be mad at me that nah, I don't believe you. It's just that we had a record that says that you didn't take medication. And I think it’s because of the signal. Don't be upset with me so that we can have a good relationship because if we don't trust each other, the relationship won’t be good. So, we have to have that good relationship so that we can finish in a good sense. [Phone ringing] [pause].

I: Okay, so how can these have a negative outcome?

I: So, how can these negative changes be addressed?

P: Mmm, I think if uh, like a TB nurse, if we can only allocate a TB patient for her, not putting other patients, because when we add another patients on his queue it’s going to be a problem, because this patient will feel like they are left out. They prefer only TB patient from that room. So, if we can just allocate only TB patients for her that will be better because there won't be confusion to come in on that TB room, it’s going to be TB patients, so they won't fight the queue with other patients.

I: Okay.

P: Because if it's not follow up, obviously she must admit the patient. So, I think she will always be busy. She can’t stand there doing nothing. I think if she is not busy, she can attend stationery, it will give peace for that room so that we can’t have any conflict with other patients. I think they can just allocate TB patients, no other patients.

I: And then in terms of this miss-communication using SMSs and phone calls, how can that be addressed?

P: Uh, since we working with network for the SMSs ,I don't think we can… we will like win the signal thing, because we're going to have that problem, but with the phone calls. It can be improved by only calling those who are defaulting, and we see that they're missing medication and for the remainder also for the appointments. We can use the phone calls, the SMS one is going to give us problems because if there is no signal and the patient took medication, and the SMS came in saying you didn't take your medication that makes them mad.

I: Mmm

I: So, how can this situation be handled?

P: Uh, I think we can stop the SMS thing. Just continue with phone calls.

I: Okay.

P: Because I think even if he misses a dose, we can call rather than giving him the automated SMS. Yeah, I think only phone calls can be used so that they can also feel like they are empowered because they're doing the right thing. So, let's just award them by making them feel that no, it's a good thing. Just continue doing that good thing.

I: Okay. So, have you had any concerns about patients opening the box without taking medication?

P: Yeah, sometimes it happens, she will tell you, “no, I was busy doing whatever I was doing. So, I opened the box by mistake, but it was not the time to take medication.” Because every time when you open it -it reports that patient so and so opened the box. Yeah, I think it is for those who have other medication. If she's taking medication, the TB medication is taken in the morning *ne (right),* so the evening one are the ARV which are taken at night, there are those that are taken at night so she will open it again at night. And it will report that the box has been opened. But with those ones, I also advise them to take out the night medication from the box so that it doesn't disturb the -the beeping of the box.

I: Okay.

P: Mmm so just put your medication next to the box, but not inside the box. And for the night-time meaning you have to find another alternative reminder.

I: Okay, so- so you've explained patients will take TB medications, but they open it again, to take other medication.

P: Mmm.

I: So, have you been concerned about patients who just open the box, but they don't take TB medication at all?

P: Yes, it can happen sometimes that they open it, and they didn't take it, because when you open the box and take medication, you take time, isn't it? It will report that now. Now she's taking her medication, but if you just open it to avoid that it will report that you didn't take the medication, it also report, and we do tell them that we see that if you just open it and close it. It will tell us that you didn't take your medication because you can’t take medication one second, you open it and close it. You didn't take your medication; it reports that you didn't take your medication.

I: Alright.

P: They will tell you no sorry sister yes, I did that, because I didn't want it to report that I didn't take medication at all.

I: Alright.

P: Mmm.

I: So ,what was the reason why that person didn’t-?

P: She will tell you I was busy with something.

I: Okay.

P: Yes. I was busy with something that's why I did that.

I: Okay, so can you tell us the system level structures that are needed in order to integrate this program with the existing TB programme?

P: The?

I: The digital adherence technology-

P: Mmm

I: Programme and the differentiated model of care we're using to follow up. What is needed for it to fit in or to be integrated to what you're already doing permanently?

P: I think what is happening now, with the boxes, I think you just need sustainability, because it's working very well. I don't think there is something that needs to be changed because whatever is happening now already is good. So, the only thing that's needed is sustainability.

I: Okay.

P: To continue.

I: So, in the absence of Aurum, when Aurum is no longer there. Who do you think should be preparing the boxes before they are issued to patients you know they need to be charged?

P: They need to be charged…mmm but, okay, we're have enough staff here. We must allocate somebody to work with the TB nurse. But the TB nurse must be the one who's responsible for the boxes to make sure that they have enough batteries and then they are charged, they are cleaned so when they go to the patient, they don't go there with a low battery and not being clean. So, the TB nurse should have another person to help her.

I: So, what will be the title of that other person?

P: I think we can call… (Laugh) my God maybe navigator, I think it’s a good name,

I: A navigator?

P: (Laugh) yes, navigator, because they are navigating the treatment there.

I: Okay.

P: Yes, I think it can be called that, a treatment navigator.

I: Okay, so is this someone new or existing staff, who would it be, who you're thinking of?

P: Mmm we have to take somebody from the clinic, we can’t hire the other person.

I: So, who can you take from the clinic?

P: We can use… there are different programs that the government has started. We have Expanded Public Works Programme (EPWP) and we have uh, community health care workers. So, from these two groups, we can choose one.

I: Okay.

P: Yeah.

I: And then should you have any issues with the technology? How do you think they should be handled once the department has taken over, let's say if you're having issues with the tablet, or the box?

P: Uh, as long as we have some where to register, so they can register that I’ve used so much tablets and so much boxes and have a monthly check on them, that they're still in a good condition. They're also available because sometimes they can get lost. So, I think that can work.

I: Okay, and then once- let's say you have a technical glitch, you can't log on to the tablet.

P: Mmm

I: What do you think should be done?

P: So, they have to give us the number for the person who can help us with technology because with computers we know that the IT guys are always there. So, they can give us somebody like an IT person who’s going to help us with the tablets and the boxes if they're having any problems.

I: Okay.

P: Yeah, we must have somebody because our only role here is to make medication and send the boxes and give it to the patient, so we don't know about technology.

I: (Laugh) Okay.

P: (Laugh)

I: So, what resources are needed for scale up to happen and needed to continue as Department of Health?

P: Mmm what can I say? Well actually what do you mean by that?

I: What is needed in terms of resources for Department of Health to continue or for you to continue in the absence of Aurum?

P: like maybe the availability of the boxes and their batteries. I think if they can just continue to order more.

I: Okay.

P: Because we are having little, like it was only just for that study. So, if they can bring more boxes and more batteries for them and chargers, then I think we can continue smoothly without any problems.

I: And then In terms of monitoring, do you need any support with the devices to monitor patients?

P: So far, I don't think so.

I: Okay.

P: The- the way they did teach us it was a straightforward thing.

I: Mmm

P: It's not like something that is going to give us problems, yes, it's a straightforward thing. So, so far it's good.

I: Okay, so you have tablets or computers in TB rooms?

P: Yes, we do have a computer there. We use it and we also use cell phones. We don't use landlines in the clinic. So, we do have a cell phone also.

I: Okay, that's good.

P: (Clearing throat) so, I don't think there's going to be a problem to continue with the project.

I: So, can you tell me if you have systems in place to report any successes or challenges, you're having currently with the implementation? Where do you record if you have a challenge now-?

P: Yeah, with uh, clinic as a whole, we do have something called indicators. So, they choose the programs that are very important like TB, pregnant women, children uh, immunizations. So, those indicators, they monitor them every month and yearly, so it is the one that tells us that now-now in TB, they're doing good-

I: Mmm

P: Or now they're doing badly, yes.

I: That's good. So, let's say there's a problem with the tablet or the box. Do you record it somewhere?

P Yes, we do have a book. We call them MDS. Every patient that I'm seeing, I write here, and this book has a comprehensive register, PHC register, and it has definitions of these elements. So, if I see a patient with TB like maybe today you come in new and there is a part for TB, there's a part for HIV, every indicator we are dealing with is here. So, I'm going to tick here so that I've seen a TB patient who started TB treatment. And if it's a follow up, there is a part where I say I screened for TB. Even if the tablet is not there. I will have a record for the day for TB, and for whatever program that the patient came for, I will know because every day we take it to the admin where they will capture the stats.

I: Okay. So, if a patient comes with an issue of the box or issues with a tablet, you record it in this book?

P: I write the name- this one is a PHC comprehensive check register [pause].

I: You were saying you right the name?

P: The name and the surname. And then I tick that you’ve been seen by a nurse, if it’s a doctor, the doctor will tick from which service point have you seen her.

I: Okay.

P: Yes

I: And then uh, can you tell me any gaps which are existing in the way the digital adherence technology is being delivered, and how they can be improved?

P: The gaps- the gaps, I don’t think we have gaps for that. So, far this initiation is something that has improved a lot in TB.

I: Mmm.

P: And it’s something that government has to see the need for it to go on

I: Mmm.

P: Because it’s really helped.

I: Mmm

P: Especially in our facility, it does wonders.

I: Nice.

P: Yes, really.

I: Do you have any suggestions to improve the way the box looks? Or the way the platform works. The tablet.

P: No, no -no everything is fine, it’s standardised

I: Okay, that’s good.

P: Even the size is normal. The patient can carry it without any problem. I don't think it's something that needs to be changed. No, it's fine that way.

I: Okay.

P: Even the tablet is working okay.

I: Mmm.

P: Mmm.

I: So, now we are about to wrap up our discussion. So, what are your final thoughts about the box, the digital adherence technology, and this follow ups; the differentiated model of care, the whole package of intervention, what are your final comments on that?

P: You know, I think with me, it makes my life better in TB because most of the nurses here in our facility don’t want to work in TB. And before- before this thing, I had that thing *linna (me too)*  that no, I don't want to work in TB. But with this, it's really helped because it was difficult really with TB because you have to monitor them. And you must make sure that you keep the success rate high, so without any intervention like this, it was difficult. So, you were fighting a losing battle. But with this box uh, coming in, it really helped me a lot it made my life easy in being in TB as a TB nurse and I was happy seeing my patients finishing treatment term and being healed with no complications. So, it really makes wonders for TB. So according to me, this thing can just go on.

I: Okay.

P: Yes. It will help more patients rather than losing people, whereas we see this helping we just say no there is no money for it really. It's really helped a lot. According to me, we just have to continue with it.

I: Okay. That's good to hear.

P: Yes.

I: So, I have one more point of discussion. So how were you monitoring uh, adherence before you were just looking at the platform? How were you knowing that the patient is taking medication?

P: As I say with… without technology, it was tough because this person will come and take medication, put you on hold and doesn't want to drink it. And just stay there when he come back, he comes, back being weak. So, you know, you can see that no, no, I'm working for something that I don’t know, because I'm trying to help this person, but doesn't want to take medication. So, with this sophisticated things they feel… I don't know, different.

I: Mmm.

P: Yes, they have a different perspective for TB treatment, because before they think maybe I don't know it's killing them or what I don't know. But now with these boxes, I think I have to take it as long as they make this Box it means this is a very important thing to do. And seeing other patients being healed. I think it also gives them that no, I can also be healed.

I want to be like that one. Because when they come in you show them, the state they are in that this person was worse than you. But see now she's healthy, you see, so they get that courage to say no. I'm also going to take treatment and I'm going to finish my term.

I: And then in terms of adherence, were you recording? Was there a way of recording adherence stats? Before-

P: Before the tablet?

I:Yes.

P: No, we were just seeing them like that, there was no way you could record that, it was only this form that was helping to record that I have seen patient so and so.

I: Alright thank you so much for your information it was fruitful for our discussion-

P: Okay.

**GLOSSARY**

*Nna (I)*

*Ya (Yes)*

*Linna (Me too)*

*Ey Slang expressive term*

*Eish Slang expressive term*
