# Supplementary material for: Acceptability of Digital Adherence Technologies to support people with drug-susceptible TB in South Africa
Source: PLoS One. 2025 Sep 24;20(9):e0332103. doi: 10.1371/journal.pone.0332103 (PMC12459780; doi:10.1371/journal.pone.0332103)
Supplement: S4 File — (ZIP) [file pone.0332103.s004.zip › S4 Transcripts/HCWs and Stakeholders/IDI 20-HCW.docx]

**TRANSCRIPTION NOTATIONS**

| **Label Key** | **Meaning** |
| --- | --- |
| **I** | Start of each new utterance by the Interviewer |
| **P** | Start of each new utterance by the Participant |
| **N** | Note taker |
| **{ }** | Indicates that details were changed or pseudonyms were used to anonymise data |
| **( )** | Indicates the description provided to anonymise data |
| **XXX** | Words were omitted to anonymise data |
| **-** | Breaking into a sentence by the next speaker |
| **…** | Pause or drawn out words |
| **[ ]** | Indicates noise made, e.g. [laugh], [sigh], [pause] |
| ? | Beginning of utterance by unidentified speaker or questionable text |
| **[inaudible segment]** | Unclear section of the recording |

Thank you so much for agreeing to talk to us today, can you please allow us to audio record this interview.

P: Yes.

I: We can record?

P: Yes, you can record.

I: Okay, PID number is xxxx, date of the interview xxxx (interview date), location xxx [clinic name], type of the interview health care worker, facilitator xxx [interview’s name] time, it 09H42 am. Alright, so tell me what is your current position at the clinic?

P: Huh, currently at the clinic, I’m a professional nurse…

I: Mmm.

P: Huh, also I’m the professional nurse, focal person for TB patients. Recently in the ASCENT study, I’ve been working hand in hand with the intern. My job specifically, I was monitoring patients and also registering patients together with the intern.

I: So, huh as you are telling me you have been registering and monitoring patients on the ASCENT project, tell me how has it been to do that?

P: Huh, registering patients on the huh, ASCENT project, it was not difficult since we were trained on how to register them. Huh, the only job was to roughly explain to the patients what it is all about and our patients were being positive towards our explanations.

I: Mmm.

P: So, it was basically simple to register them.

I: Okay.

P: Yes.

I: How does it feel like as a nurse to be able to monitor the adherence as you are saying that you are monitoring the adherence, how does it feel like to do that?

P: Huh, it feels very, very great because before the ASCENT project, one wouldn’t be sure if the patient is really, really adhering to the medication. So, every day when you are opening the App and you see the chart of the particular patient is green, not red. It brings the good impression to me as a nurse that I have done my job of adherence. So, seeing the patient adhering huh, it very, very, very good.

I: Mmm.

P: It very good- great.

I: Okay.

P: Then it comes to the point where one could be sure that the outcome of the patient will be good.

I: Okay, what were you using prior [ringing phone] [pause] the implementation of ASCENT project? Yes, you can continue.

P: Yes, huh monitoring it was very, very, very great meaning that huh, once I can see that the graph is going well. It gives me the good impression that at the end of the six month [door opening] [pause] it will be…

I: So, you were still mentioning that at the end of the six months you will know that…

P: The six months.

I: Mmm.

P: I will know that huh, the outcomes is going to be a good outcome.

I: Huh-

P: Because once the graph of the adherence goes well, it gives the right impression that the results at the end of 23 weeks for treatment will bring a negative AFB sputum.

I: Okay.

P: Which means it’s a good success rate for completion of treatment.

I: Mmm, so can we talk about what is happening when it comes to the progress in treatment huh, in managing TB treatment in the facility ever since we’ve started with the program in this facility. How is it like now since we’ve started the program?

P: The progress since the ASCENT study, it went very, very well because the number of our loss to follow patient has decreased. The reason it decreases, it because when using the box, it makes sure that the patient is always aware that now it my turn to go back to the facility and I as a healthcare, I know that today that my patient is having the last medication. So, for sure tomorrow the patient will be coming to the facility. If the patient doesn’t come, when I check the app, I respond to the patient immediately, I call the patient, I remind the patient and the patient come immediately to the facility. Which was a very, very big success because before we had a lot of loss to follow patients and we couldn’t turn them down. Most of them, we track them after many days, but now with the ASCENT study, tracing patients was very, very, very good because mostly the intern was also working hand in hand with us making sure that all our patients are adhering and coming back to the facility.

I: Okay, I just want to have an idea, how is the technology or the implementation of this technology, how is it improving loss to follow?

P: Huh, it is improving loss to follow because huh, the way the, the box has been designed. Huh, that alarm it gives the patient more consistency in his or her treatment. The more the patient is consistent in checking the box, that gives the impression that I mustn’t miss a date to the facility. Meaning that once the patient can see that now I’m left with one tablet. He or she knows that tomorrow I’m going back to the facility and that reduces the amount of loss to follow.

I: Mmm.

P: And also, in the graph when I’m monitoring, when I see that is red. I have to wonder why it is red, immediately when I call the patient. The patient will say, “eish my tablets are finished. I’m coming”, that means we have minus one loss to follow.

I: Mmm.

P: Meaning that the number of loss to follow have been reducing, reducing.

I: Okay.

P: Yes.

I: Alright, so what were you using before huh, ASCENT in terms of monitoring adherence on patients?

P: Huh, before ASCENT huh, we were only using the idea of emphasizing taking treatment during diagnosis and also in each and every visit of the patient of which huh some patients- yes, we could emphasize, but because they had nothing to remind them about the treatment. They tend to default their treatment, but since ASCENT, it has been good because huh, something to remind about treatment has been there, that box huh, it is emphasizing adherence by itself. When the patient sees the box knows that my treatment is inside there, I’m going to take it unlike when there is nothing. The patient can put the tablet inside her bag and find that the bag is not open for almost two days.

I: Mmm.

P: But when there is a box which is ringing, we knew that adherence is going to be good, yes.

I: Alright that’s good to hear all of that and if you have to explain huh, what is digital adherence huh, intervention to another healthcare worker who knows nothing about this program what, what can you tell them?

P: Huh, well I will tell them that huh, the digital program is designed to store treatment for pat- TB patients.

I: Mmm.

P: And also, the device when using, it huh, in correspondence with the App. In the App we register the patient, after registering the patient, we are registering all the necessary information about the patient, especially the address, the number phones and also the tablets the patient is going to take. So, the digital huh platform is very, very, very, very user friendly huh, device for the patient and also the nurse. This huh, reduce the nurse, the nurse workload and the nurse do not stress about TB defaulters.

I: Mmm.

P: Because through the device patients are going to adhere to the medication, so to the next healthcare provider I will say, “huh there is no need to worry about this device. The only thing is to accept the change and allow to use the device because huh it’s going to bring more, more change in the world of TB.”

I: How is it going to bring change in the world of TB?

P: It going to bring change in instance that huh, we are no longer going to be crying about huh relapse of TB because of huh non-adherence of patients.

I: Mmm.

P: Remember if the TB patient is not adhering, that means there are high possibilities of relapse and also treatment failure, so through the digital platform, you are going to be 100 % sure that huh the graph of my patient is going well my patient is adhering to medication. That gives me confidence that at the end of treatment, I’m going to be having the positive outcome.

I: Mmm.

P: My treatment- patient is not going to fail, but it’s going to be successful.

I: Mmm.

P: So, as the healthcare provider you know that you are relieved from stressing now and then about the outcomes of the patients.

I:Ehh.

P: Yes.

I: Okay, we spoke earlier on about the things that you do in the ASCENT pro- program that you know you register patient also monitoring the adherence, it’s good and if it’s not good, you are calling the patient.

P: Mmm (yes).

I: Who is calling the patient, is it you or someone else?

P: Huh, doing the calls it me and also the intern, you will find that I’m busy, but the intern is free, I will request the intern to call.

I: Okay.

P: Or the community healthcare workers.

I: Alright, so they…

P: They are really assisting us especially in the physical tracing.

I: Home visits?

P: Yes

I: Okay, that’s nice to hear.

P: Yes.

I: So, when you have to call the patient, I just want to have an idea, is it something that is doable for you as nurse to call patients?

P: Yes, it’s doable

I: Mmm.

P: It’s very doable

I: After monitoring after seeing…

P: After seeing the, the App.

I: Mmm.

P: It’s doable, the moment you see that my patient now is in red, you have to eager to find out why, why.

I: Okay.

P: So, you immediately call the patient.

I: Okay.

P: Hence, I mentioned that you have to, when registering, you have to make sure you enter the number phones that makes it easy to reach the patient.

I: Alright.

P: Yes.

I: So, when you really quickly call the patient because you’ve seen on the App that they are not taking medication and you quickly call them, what do they usually tell, tell you? Do they confirm that they take medication? Do they or they are saying they took? I just want to hear those huh, comments that they tell you when you actually call them.

P: Yes, the patient huh, normally they confirm that they didn’t take the medication. Some will come with this- someone will come with the story huh, “I forgot the box at the house ,I went out very, very early. So, when I when I arrive home, I will make sure I take my medication.”

I:Mmm.

P: Those were one of the excuses that the patient normally give us or some patient will say huh, eish I visited my relative and forgot the box, but I came with the medication. Meaning the patient took out the medication from the box and left the box at home. And that one is going to be a signal to me that my patient is not taking treatment. So, usually, we were emphasizing that wherever they go, they go with the box.

I: Mmm.

P: Because when the box is left behind, it will be a sign that they are not taking treatment.

I: Mmm.

P: So, they normally tell us that I left the box behind and took the treatment.

I: Okay.

P: Yes.

I: What makes patient to take treatment and leave the box behind from your perspective?

P: From my perspective, huh some patient, they are only using huh, taxis. Some are using cars, so normally, they want to reduce the burden in their bags.

I: Mmm.

P: And some they are afraid that the alarm will be ringing while they are on their way. So ,they are afraid that people will be curiouss about them opening the box and taking out the tablets.

I: Mmm.

P: Yes.

I: Okay, so can we talk about the stigma in relation to the use of this box, what are your thoughts around that?

P: Huh, my thoughts around the stigma of using the boxes, is that the box it’s in two ways. The box can bring stigma and the box can prevent stigma. It can bring stigma in a way that huh, I am a patient and I’m carrying the box, I am in a public transport and the box rings. I am going to open the box, when I take out treatment it going to bring stigma through the community. They will want to know what the treatment is for, and I am no longer going to be free. Then, it prevents stigma because no one after I visited the facility or where I am, no one will be able to know what is inside the box.

I: Mmm.

P: Because the box won’t be ringing, and I won’t be opening the box. No, one will be able to know what is inside the box and when no, one knows what is inside the box. There is no one who will give me stigma and discrimination.

I: Okay.

P: Yes.

I: Alright can you please describe your role with differentiated model of care that is Huh what, what, what is your role in the study when It comes to differentiated of care?

P: Huh, my role is to make sure that huh, the patient (.)-

I: So, the differentiated of care is the follow up actions that you do.

P: Follow up actions?

I:Mmm.

P: Mmm.

I: So, what exactly do you do when it comes to follow up patients on adherence?

P: Okay, my role is to follow up the patient, sit down the patient huh, reemphasize adherence counselling.

I:Mmm.

P: And show the importance of taking treatment to the patient. Remember huh, there are many, many challenges that makes one to, to not adhere to the treatment.

I: Highlight few of those challenges.

P: Huh, one of the challenges is the side effects, the side effects of the medication. They make it difficult for the patient to adhere huh, for instance, Rifafour, Rifafour have got different huh, side effects such as uhm general body itchiness uhm and also weight gain.

I: Mmm.

P: Rifafour can also make the urine to be orange throughout the treatment. So, some patients and also their peripheral neuropathy. So, some patients- most patients are unable to bear the side effect of Rifafour. So, my duty is to do continuous counselling, continuous huh, emphasizing of the side effects, so that patient will not give up on treatment. Huh, the other challenge is what will people say, so I always huh counsel the patient that they mustn’t focus on what people are saying. They must focus on their treatment because if they think people will say this, no one can say something about them, if they are adhering to their medication and no one will see that they are not well if they are adhering to their medication.

I: So, huh earlier on you mentioned that you know you would sit huh- monitor adherence on the platform and then do some callings and sometimes do home visits. I want to know, and you mention that you are doing this work with the intern and sometimes with the community healthcare worker. I just want to know how are you sharing this huh this activity that I just mentioned calling, monitoring adherence on the platform, calling if you see that there’s non-adherence and requesting for home visits, I want to know who is doing what between you, the intern the community healthcare worker? Just give me an idea of how are you, how are you sharing these responsibilities?

P: Huh, since uhm I was the one having access to the App and also the intern.

I: Mmm.

P: We would communicate every day that huh, so and so patient huh, haven’t taken the treatment today. If I am able to, I do the follow up, if I’m not, I communicate with the intern. Please huh follow up with this patient who is not taking treatment, then if we are failing together, me and the intern. Then we escalate to the community healthcare worker, then they assist us with the home visits.

I: Mmm.

P: Yes, so we were doing it hand in hand together with the intern. If I’m not able to open the app, the intern can open, if I’m not able to communi- to call, I request the intern to call.

I: Okay.

P: Yes.

I: How has it been doing that, is it easy?

P: Huh, it has been simple.

I: Mmm.

P: Huh, it was very, very easy because huh, communication was making it simple.

I: Okay.

P: Yes.

I: Alright, so when, when you first had about digital technologies what were your expectations before it was implemented?

P: Huh, the first time I heard about it uhm I never thought it is something which will run smooth knowing how are our patient are. As huh, people are resistant to change.

I: Okay.

P: So, after starting to implement it, I saw that it is huh, it is doable because patients were loving it, patients were enjoying it because it always reminded them.

I: Mmm.

P: It always reminded them of taking their treatment.

I: Mmm.

P: Knowing one will be sure of what the box is carrying, positive respond of the patients is the one that made me to be sure that indeed something that we can use for future.

I: Mmm.

P: Yes.

I: So, you are mentioning that you know patients, so you were worried about patients?

P: Yes

I: What exactly were, you worried about?

P: Huh, I was worried that huh, patients huh, will refuse to take the boxes thinking that the boxes are going to bring some suspicion from the community. But that never delayed the patients to take the boxes.

I: Okay.

P: They took the boxes, used them regardless of what the community would think.

I: Mmm.

P: And I also thought maybe the patients will say it will be heavy or they won’t have space to put it, but know the patient huh, took them and enjoyed them.

I: Okay, so were your expectations changed about?

P: My expectation, my expectations were indeed changed.

I: Mmm.

P: Were indeed changed and then I realized that this is something that we can use for the future because huh, it was really, really making things to be simple. The patient comes, the patient comes back with the box for the next visit. We load the treatment; the patient goes back happy with the box, and you could see that they are enjoying to be having their boxes.

I: Okay.

P: And some, some other patients because as the month for TB treatment huh, reduce huh- they were also putting their other medication inside although we told them that they must not put other medication. But because they were used seeing the user friendly of the box. They put their other medication inside and when they take their TB treatment, they are also taking their other medication simultaneously and enjoying.

I: Mmm.

P: So, we couldn’t tell them to remove their medication because they were enjoying.

I: Alright.

P: Yes.

I: Okay, that’s good to hear all if that, uhm can you please describe the uhm, training and uh resources that staff received on the delivery of the digital adherence technologies including the differentiated of care, how was the training that you received?

P: Well, uh we received the training of the devices.

I: Mmm.

P: Then it was good, although some staff uh, they were resistant to change. They didn’t uh understand it, uh once.

I:Mmm.

P: I had to reemphasize, although some were still resisting to change.

I: Mmm.

P: So basically, the training was really, really good, it did help.

I: Do you still remember the activities that you did in training?

P: Huh (…).

I: What, what is it that you did on that day if you still remember the activity?

P: Huh, it was the manual for the devices.

I: Mmm.

P: Then also the usage of the battery, the insulation and also when and when not to, to open the box.

I: Mmm.

P: That was what, it is was done during the training.

I: Okay.

P: And also [pause] [door opening].

I: Okay, you can continue.

P: Ehh.

I: And also?

P: Mmm (…).

I: Anything else?

P: And also, the training on the registering and of monitoring on the App.

I: Okay.

P: Yes.

I: So, huh how, how were you trained? Where were you trained do you still remember?

P: Huh, we at certain lodge in Rustenburg.

I: Okay.

P: Kashan

I: Alright.

P: If I’m not mistaken, uh it around Kashan waterfall.

I: Okay.

P: What is your opinion about training and resources that you received?

I: Huh, my opinion is that huh the resources were good, and the training was also good because it was practical, the boxes were there and the App was there which made it to be simple for one to understand. So, basically uh, I was uh, impressed with the training of the ASCENT program.

I: Mmm.

P: Yes.

I: Do you have any suggestions in terms of how can we, how can we improve the training, the training of this whole program?

P: Huh, my suggestion is that the program should not only be introduced to the TB focal people only, but also other healthcare providers should also be trained continuously because uh, not only one person will be remaining to be the TB focal person. All the healthcare providers, the professional nurses and doctors should be trained on the program because huh [pause].

I: Mmm.

P: They should be trained on the program so that there is continuous implementation of the program.

I: Mmm.

P: Yes.

I: Huh, any idea on how can we do this training of the other healthcare workers, like how can we do it, we doing it on?

P: It can be done huh by visiting uh, the facilities.

I: Okay.

P: Visiting the facilities conduct the training, in-service training. although as a healthcare worker who was trained, I do implement the in-service training. It is not the same as you guys come and train them, it is not the same. They don’t receive it the same way, you will come and uh, train them.

I: Okay.

P: You see.

I: Mmm.

P: Because uh, at some point, they see me as the, as one of them.

I: Mmm.

P: So, as I’m passing time so, but when somebody from the office come and train, it became simple.

I: Alright, okay so tell me uh, how, how will be the ASCENT- this program be without the asc- the xxx (organization name) support as department of health, can you implement and run with it all alone without the uh, ASCENT…

P: Honestly.

I: Without xxxx (organization name) support?

P: Honestly without the xxxx (organization name) support, I doubt the DOH can make it.

I: For now,

P: For now, they can’t make it without the xxxx (organization name) support.

I: Why do you feel like that?

P: Huh, there is a lot going on around the department of health. Many programs are not running, many programs are not running, but with the support of xxx (organization name) this program was running without any delays. So, in the department of health many things are in need of implementation.

I:Ehh.

P: But they are just hanging on.

I: Okay.

P: So, meaning there is a very, very big need of assistance to the department of health as you know from the national, there is a lack of many things so that’s why I’m saying the support of xxxx (organization name) brought a very, very big change to this uh, program.

I: Okay.

P: Yes.

I: So, was the duration of the training that you received- are you happy with the duration of the training or?

P: Huh, the duration of the training was fine, it was a day, so a day almost eight to nine hours it was fine.

I:Mmm.

P: Mmm, it was fine.

I: It was enough?

P: Mmm, it’s enough.

I: Okay, from the healthcare worker perspective, can you please [door opening] [pause] can you please describe the benefits uhm, of implementing uh, the digital adherence technologies together with the differentiated care of model?

P: The benefits of implementing uh, is that the number of loss to follow have decreased.

I: Okay.

P: That is the major benefits, another benefit is that the monitoring of adherence was simple.

I: Mmm.

P: Because every day I could see how the patient’s graph is going on, when the patient sputum is new, the app will reflect.

I: Mmm.

P: Those were the benefits, and the other benefit is that the patients were coming back honoring their appointments.

I: Mmm.

P: When the patient opened the box and see that now I’m left with one day treatment, it means my appointment has arrived, the patient came to the facility.

I: Mmm.

P: Yes.

I: Okay, in terms of the healthcare worker, how is it going to benefit you as a healthcare worker?

P: Huh, it benefited me in a sense that, it relieved my job, it relieved me from stressing about the patients not adhering neh (right).

I: Mmm.

P: And also, it really improved me from emphasizing the importance of taking TB treatment.

I: Mmm.

P: And also, it also assisted me in knowing that uh, my patients indeed are going to have a positive outcome.

I: Okay.

P: Yes.

I: In terms of uh, challenges of implementing these technologies and the differentiated care what are challenges that you came across. Anything that was sort of negatively in the implementation, what challenges?

P: The challenge was uhm of a patient coming from Lesotho.

I: Mmm.

P: I had a patient from Lesotho, so the patient took the box. He was honoring his appointments.

I: Mmm.

P: All of the sudden, now his becoming red. I’m trying to trace down, to trace down. The phone is not ringing.

I: Mmm.

P: Meaning that person have crossed the border.

I: Mmm

P: Fortunately, I came across the child.

I: The?

P: The child of the patient.

I: Okay, here?

P: Huh, the child came to request treatment for the father. I was trying to ask no, did he go to Lesotho? He left the box behind. That’s the challenge he crossed the border and left the box behind, meaning that he is taking treatment while the box is left behind. So, that was the challenge in implementing. Then, uhm, another challenge was about patients who were waiting in line some they were saying that the time, the morning time is difficult for them.

I: Mmm.

P: But I did advise them that they can find a consistent time that they will be taken before going to work, then we agreed. Then, they said, no, immediately this alarm rings.

I: Mmm.

P: I will be preparing myself to go to work, I will take the treatment, so they were no, basically they were no challenges.

I: Mmm.

P: The program was running well, and we were not facing any challenges, our patients were happy about the boxes.

I: Okay.

P: They were happy about the boxes, just that some brought the boxes back when they were not ringing.

I: While they were not alarming?

P: Huh when they were not alarming.

I: Okay.

P: Then we changed- we gave them the other ones and they were alarming.

I: They were fine?

P: They were fine.

I: Okay.

P: Yes.

I: So, why was uh, the child of the parent came to collect the medication, yet the parent went to Lesotho?

P: The father always leaves the card behind and tell the child that on this day go and fetch my medication. If I’m not yet back from Lesotho, you will send somebody by the taxi with the medication.

I: Huh, okay.

P: Yes.

I: So, they are not giving them medication in Lesotho?

P: They do give them, but some patients are, are afraid of going and queuing in Lesotho to be asked many questions.

I: Mmm.

P: Yes, that how they do.

I: Okay.

P: Yes.

I: Then up until they come back?

P: Up until they come back.

I: Huh, so do you usually give the child that medication even though the parent is in Lesotho?

P: Well, huh, in that instance we had to, to give her to give him.

I: Mmm.

P: Because huh, she emphasizes that she is going to Lesotho, then she will come back with him.

I:Mmm.

P: And indeed, in a week they were back, and the father came, we checked him he was still fine. We emphasize that wherever you go you must take the box with you and when you want to go to somewhere you must come, we end the treatment.

I:Mmm.

P: Yes.

I: What was his reason of leaving the box behind?

P: He thought the box won’t work after crossing the border.

I: Mmm.

P: He thought the box won’t work after crossing the borders.

I: Huh, okay.

P: Yes, that was the reason.

I: Then, then you thought him that it will work?

P: It will work, yes.

I: So, do you think the patient now is crossing with the box now?

P: The patient is now done with the medication.

I: Huh, and he is cured?

P: He is cured.

I: How do you feel about the patient who is cured, who was using the box?

P: I’m very, very excited.

I:Mmm.

P: It very, very exciting.

I: Did the box contribute on that, huh?

P: The box indeed contributed to the patient to be cured.

I: Mmm.

P: It contributed because without the box huh, adherence was not going to be simple.

I: Okay.

P: Yes.

I: Alright that’s good to hear all of that, from your perspective as a healthcare worker, can you uhm- can TB treatment be improved by using digital technologies and together with the differentiated of care?

P: Yes, it can be improved because huh, using the box makes adherence to TB treatment to be easy.

I: Mmm.

P: Yes.

I: Different in terms of treatment uh before the intervention and after the intervention?

P: Before the intervention when it comes to treatment many patients were defaulting, were missing their appointments, but after the intervention patients were con- consistently honoring the appointments. We were not having number of patients who were relapsing, patients who were loss to follow.

I: Mmm.

P: All our patients were being managed very, very well without uh, uh stressing about the that this patient will relapse, this patient will be treatment failure.

I: Mmm.

P: Our patients were successful.

I: Okay.

P: Yes.

I: After doing…

P: After the intervention, yes.

I: Okay, please, please elaborate on positive changes uhm that the program has brought in the facility?

P: In the facility like I have indicated in the beginning the positive change is about the reduction, the inducement of TB loss to follow, TB relapse and also the good adherence of the patient using the box in the very same time consistently every day. It made our patient not to be having AFB monitoring sputum which were positive in week seven of treatment and week twenty-three of treatment because they were taking treatment uh consistently with time.

I: Mmm.

P: It was working very well for them that why there are sputum for monitoring where always negative yes.

I: That’s good to hear, so what can we do to sustain these positive changes?

P: To sustain these positive changes is to continue using the box so that our patient is consistent with time.

I:Mmm.

P: Remember when you are not consistent with time, taking treatment whenever you like, it increases the chances of the sputum to be positive the one for monitoring.

I: Huh, just like a person who is on Anti retrovirus if they are taking treatment not consistent with time the treatment won’t work for them, their viral load will remain going up.

I: Mmm.

P: Yes.

I: Okay, but now the box is helping to…

P: The box helped.

I: To, to…

P: Improve.

I: On that?

P: Yes.

I: Okay, that’s good to hear, can you reflect on the negative changes now that the technology has brought to our patient and also to the way that you are doing things?

P: Huh, I can’t think of the negative changes [laugh].

I: Mmm.

P: Because this box brought positive changes to be honest.

I:Mmm.

P: It brought positive changes.

I: Mmm.

P: Yes.

I: But what are the negative experience because of using the box [door opening] [ringing phone] [pause]. Huh, I was still asking you to reflect on the positive I mean…

P: Negative.

I: Negative experiences because of using technology both on the patient side and also on your side as a healthcare worker? What are the negative experiences that you have encountered because of using the technology?

P: Huh, the negative uh experience in my side was that of the patient who left the boxes behind.

I: Mmm.

P: Because the moment I see that the patient is being red I start to stress a lot and wonder what is happening, what is happening and when I found out the patient left the box behind. It really, really gives me stress that did I fail to explain to the patient that wherever the patient goes must carry the box.

I: Mmm.

P: So, it brings that challenge, so to the patient uh well it was what can I say about the patient, this patient was very great to them uh just that only few were forgetting to carry the box.

I: Mmm, okay.

P: Huh, that was the negative part.

I: Okay.

P: Yes.

I: So how can we improve on the negative part what is that we can, like this one that you have just told me about, that you know the patient when they are leaving, they leave the box behind, you see non-adherence only find out they are taking treatment, and this is frustrating you. What, what can we do to improve this?

P: What can we do is to really, really emphasize on the patient when we are giving them boxes that whenever they go they must carry the box. They are going to be working wherever they go.

I:Mmm.

P: That will really, really make the patient.

I:Mmm.

P: Not to let them behind.

I:Mmm.

P: Because if we emphasize, they won’t leave them behind.

I:Ehh.

P: Yes.

I: Okay, please describe what system structures need to be improved in order to integrate these tools that the technology and the existing here in the clinic what can we do to integrate the two?

P: Huh, what can be done is to do training to the healthcare providers, that’s the only thing to integrate.

I: Mmm.

P: Is to train the healthcare workers so that we can be able to adjust to change.

I: Mmm.

P: Yes, to change to use the devices.

I: Okay.

P: Yes.

I: Then when we are doing the integration, what is it that we need to emphasize so that it easy for both this new thing and what is happening now to come together and be one thing?

P: Huh to emphasize the main objectives of the devices.

I: Okay.

P: And also, the previous positive thing that the device has done.

I:Mmm.

P: So that everybody can be aware of what the devices does. That will make one to be able to see that indeed this something that we can hands on and use.

I: Okay.

P: For instance, if we are telling them that this device makes patients to adhere, this device makes it for you to simple monitor the adherence, no one will refuse that.

I: Mmm.

P: That will make it possible for integration.

I: Okay.

P: Yes.

I: Alright, so in terms of -can you please describe what systems are in place to monitor if this technology is working, for what can we do to assess if the technology is working?

P: Huh, is to check monthly on how the response from the patent was or to also check in months if the patient’s sputum was negative, it also what can be done is to monitor the success of patients. If we are saying, we have ten patients in the uh study and all of them they have responded well.

I:Mmm.

P: That means that the study is working. If one is dragging, we need to find out why is that one dragging.

I: Okay.

P: So that will help us to improve.

I: Huh, to evaluate in that?

P: To evaluate uh…

I: Technologies is well?

P: Is working well.

I: Okay, how can we do this, are we going to check. I hear that part that we are going to check if their doing better.

P: Mmm.

I: Doing better, how can we do that, like do we need to write it somewhere?

P: Yes, we need to conduct a report for that.

I: Mmm.

P: We have to document it; we have to write it.

I: Mmm.

P: And sit down and see what quality improvement can be done.

I: Okay.

P: That will help us.

I: We need to capture it down?

P: Yes, we need to capture it down.

I: Okay.

P: Yes.

I: Okay, so at the moment what are we checking the effectiveness of the program here?

P: Huh checking the effectiveness of the program is to check the patient we are having if the patient we started them uh February treatment we have to check after that this is the six months they complete.

I: Mmm.

P: If they completed that means that the program really works.

I: Okay

P: And also, to check the sputum taken if the sputum were taken that means it was working.

I: Okay.

P: Yes.

I: Okay, thank you for all the information that you have given us, Huh, we are really, we are towards the end of the interview ,but before we can close, can you please share any few remarks reflects back on the implementation of the project anything that you would to mention now that we did not touch during the interview that you want us to hear about the, the program.

P: Huh, the program really, really good I enjoyed being part of the program I, I learned a lot from the program uh because the way the App was designed for monitoring. I was really, really enjoying when I opened and see my patients in green, it always gives me motivation.

I:Mmm.

P: So, all in all uh I was impressed with the, with the program.

I: Mmm.

P: Yes.

I: Where do you see this program in the future?

P: In future, I see this program running well not only for TB patients. It going to integrate also for other patients on other chronic medications for instance the ones on antiretroviral therapy. It will assist them a lot.

I: Mmm.

I: In terms of?

P: In terms of being consistence with time, adherence that will reduce the number of viral loads that are escalating because patients have not been consistent with time.

I: Alright.

P: Yes.

I: Thank you very much uh for the time and this interview, we really appreciate.

P: Thank you.
