# Supplementary material for: Acceptability of Digital Adherence Technologies to support people with drug-susceptible TB in South Africa
Source: PLoS One. 2025 Sep 24;20(9):e0332103. doi: 10.1371/journal.pone.0332103 (PMC12459780; doi:10.1371/journal.pone.0332103)
Supplement: S4 File — (ZIP) [file pone.0332103.s004.zip › S4 Transcripts/HCWs and Stakeholders/IDI 21-HCW.docx]

**TRANSCRIPTION NOTATIONS**

| **Label Key** | **Meaning** |
| --- | --- |
| **I** | Start of each new utterance by the Interviewer |
| **P** | Start of each new utterance by the Participant |
| **N** | Note taker |
| **{ }** | Indicates that details were changed or pseudonyms were used to anonymise data |
| **( )** | Indicates the description provided to anonymise data |
| **XXX** | Words were omitted to anonymise data |
| **-** | Breaking into a sentence by the next speaker |
| **…** | Pause or drawn out words |
| **[ ]** | Indicates noise made, e.g. [laugh], [sigh], [pause] |
| ? | Beginning of utterance by unidentified speaker or questionable text |
| **[inaudible segment]** | Unclear section of the recording |

I: Thank you very much for agreeing to participate in the study. Can you please allow us to audio record the interview?

P: Yes, you can record.

I: Can you tell me sister what is your current position here in the clinic? What are you doing in the clinic?

P: Okay, my work is I am a CHW (Community Health Worker), I am working in the community.

I: Mmm.

P: Yes, and I am tracing patients in the community, those defaulting the treatment [pause].

I: Yes, you were still telling me- what are you doing in the clinic?

P: I am a CHW, I’m working in the field, and my job is to trace patients and work with the patient. If the patient is defaulting uh, treatment, I go in the field and trace them and come back to the clinic.

I: Mmm.

P: Yes.

I: Okay, so how is like to trace TB patients?

P: Because there is someone who is working with TB patients, if there is difficulty that they can’t find them, they put them in the M phone so that I can trace them in the field.

I: Okay.

P: I go there and look for the numbers and where they stay and trace the patient. When the patient is there because there is- they are using the ASCENT boxes.

I: Mmm.

P: Yes, when I go there, I check if the patient is complying with the treatment.

I: Mmm.

P: So, I talk with the patient so that he must comply with the treatment. If there is difficulty with the treatment, they must come back to the clinic and ask again for help so that she can go well with the treatment.

I: Okay.

P: Yes.

I: So, do patients struggle to take treatment when you go to their houses, do you find them having challenges to drink treatment at home?

P: Sometimes because sometimes they don’t comply with the treatment.

I: Mmm.

P: Others are smoking and drinking.

I: Mmm.

P: So, they are smoking and drinking alcohol whereas they are on treatment. They have been put on the treatment, so we talk to them that they must not drink alcohol whereas they are on treatment. They must finish the treatment, then after they can go with their duties of new enjoyment activities whatever they want to do.

I: Mmm.

P: After they comply with the treatment, but it depends on them if he wants drink the alcohol or want to smoke again.

I: Mmm.

P: Because there is a danger in that, yes, we talk to them like that. And if the patient- others face difficulty with these ASCENT boxes and they call me, they call. When they call me, “I ask XXX [intern] so that she can help with that box because others complain of not getting the messages or alarm, if there is information there, they watch.

I: Mmm.

P: That box must ring right because if the patient takes the pill the box must ring so that the information can be, can get the information in that box.

I: Mmm

P: So that she can open the box and drink the treatment.

I: Okay.

P: Even if there is a complain sometimes, they phone me so that I can tell XXX [intern] that no, this box doesn’t reflect the light so that it can show her (intern) that she (patient) has taken the treatment.

I:Mmm.

P: Yes.

I: So, when they are calling you and telling you about the box and complaining about the box, what exactly are they complaining about? What is their complain about the box?

P: She ( a patient), she fears that maybe the, the person responsible for the boxes might think that his or she is not taking the treatment well.

I: Mmm

P: XXX [intern] will say, “no, you didn’t take the treatment because I didn’t see any sign here.”

I:Mmm.

P: Yes.

I: But they are taking the treatment?

P: They take the treatment.

I: Okay.

P: Huh, but sometimes, it doesn’t click (alarm or flash a light).

I:Mmm.

P: So that it can show that she (a patient)-XXX [intern] can see that he or she takes the treatment.

I: Okay.

P: That the problem, but there is fear. He (a patient) calls me, “phone XXX [intern] and tell her that the box doesn’t reflect.

I:Mmm.

P: So, I would call XXX [intern].

I: Mmm.

P: Yes, I say, “XXX [intern] there is a patient- whom, whom, whom- so and so-can you help him or her. I give the phone numbers to XXX [intern], XXX [intern] phones that patient and they talk.

I: Okay.

P: Yes.

I:What is the other challenge of patients about the box is that thing of uh, they are worried that they will be called by the intern saying the box, I mean they did not take medication. What is the other challenge that patient is complaining of about the box?

P: No, I think there is- that is the only complain because sometimes it doesn’t click (alerts) to inform the patient to inform XXX [intern] that the patient has opened the box now.

I: Okay.

P: Yes.

I: It only that problem?

P: It only that problem, but they comply in the treatment, that box helped a lot.

I:Mmm.

P: Because there are no difficulties in that- because they get more information so that they can take that treatment.

I: Mmm.

P: Yes.

I: You are saying that there is no other complain, but the patients are getting help from the box?

P: Yes.

I: What is helping the patient about that box? What exactly is helping them using the box?

P: Because patients- if that box wasn’t there, patients wouldn’t comply. They default the treatment and go where they want to go without treatment.

I: Mmm.

P: Taking the treatment because that box is there now, they fear that uh, they see that I am not taking the treatment.

I: Mmm.

P: You know yes, they take that treatment and drink the treatment.

I: Mmm.

P: And if you don’t take the treatment at the clinic, they will see that you did not take the treatment because-

I: Mmm.

P: Because they can take bloods so that they can check you.

I: Mmm.

P: That you are taking the treatment if you don’t take the sputum’s. They will take the sputum so that they can see that you don’t take the treatment well.

I: So, as a community health care worker, how is the use of this box helping you to support your patients?

P: It is helping me because I don’t get any difficulty that patients because they are complying with the treatment.

I: Mmm.

P: Yes, it helps me a lot.

I: Okay.

P: Yes, because they comply- there is no- sometimes you could not get- so that they say go and look for the patient for TB treatment to see if they are taking treatment well. They are complying these days, there are no problems but compared to when they didn’t have these boxes. They default and they go away sometimes.

I: Mmm.

P: Huh, and others go and come and take and take their treatment and go away maybe xxx (neighbouring country) or (another province) and default the treatment sometimes that is why there is no tracing.

I:Mmm.

P: The tracing does not go well because when we go there, she is not there; the phone numbers are not working as well.

I: Mmm.

P: Is the problem that one.

I: Is it the people who have a box that are doing that? Is it people who have a box?

P: No, most of them don’t have the boxes.

I: Mmm.

P: Mmm.

I: Okay.

P: Maybe those ASCENT boxes were not there when they defaulted, I don’t know because I am not the one who’s using these boxes giving them, but the ones that I know now they don’t default treatment, and they ask me when we finish with the boxes, what must we do with them? “No, take the boxes and return them at the clinic.”

I: Do you think they want to keep their boxes and not return them?

P: Others do ask- “want to know what must I do with it, or should I keep it.” Huh, you should return it back to the clinic.

I: What else do they put in the box except TB treatment?

P: They put the TB treatment only.

I: Mmm.

P: But others you find that they say because I have another medication that I take, can’t I put it together in the box.Huh, you find that others want to put other medication and now I say I will ask at the clinic what are they saying.

I: Okay.

P: Huh, I will ask the person who is working with the boxes that if you should return the box, or you can keep it at your house so you can put your tablets.

I: Alright.

P:Mmm.

I: What other tablets do they want to put?

P: The long treatment ART.

I: ART treatment and which ones?

P: The high blood sometimes.

I: Mmm, okay.

P: Huh, and other treatment, they are different medications.

I: Mmm.

P: Mmm.

I: So, they want to use it for other medication?

P: Huh, others want to use it for other medication, it is important to them.

I: What?

P: They say the boxes are important because you don’t forget to take treatment.

I:Mmm.

P: “These boxes are very important to us.”

I: They say that?

P: Huh, “they are important.”

I: Okay.

P: Huh, “because you don’t forget that you must drink your medication, you know that you must take treatment now. When I open the box, it reminds me it rings.”

I:Mmm.

P: “That I must take my medication and drink them now, it is the best.”

I: Mmm.

P: “Because I don’t forget to take my medication.”

I: Alright.

P: “All this time when I look for it where I keep it, I can hear it rings. Sometimes even if it does not ring if the time arrives, I know that I have to take medication.”

I: Okay.

P:Mmm.

I: Which other way does this box helps patients? It rings, it is a reminder to take treatment. Which other way does this box help patients?

P: Mostly I help them keep it in a place where they won’t forget and keep it in a safe place.

I: Mmm.

P: They must not forget their medication.

I: Mmm.

P: If she goes around, she can see it, so that she won’t forget because it happens that it doesn’t remind, it doesn’t ring maybe it damaged sometimes.

I: Alright, How does these boxes help you as a community healthcare worker to help your patients?

P: They help because I do not go every day to say take your medication. I go maybe after four to five days.

I: Mmm.

P: Huh, just to check if she did take medication. If I get there I can see, it shows that uh, she’s adhering.

I: Mmm.

P: Huh, then if all is well, “carry on comply with your treatment and eat healthy food so that you can be better.”

I: Okay.

P: Huh, “do exercises, uh be better.”

I: Okay, when you compare the time before we brought these boxes here in clinic and now, they are here in the clinic. Which is better to support patients without boxes or with boxes?

P: It now, it much better now with the boxes Because at the clinic, they don’t ask me to go look for so and so, it look like they are not taking their medication or every time when he comes to the clinic his weight is not right or his not fine, go look for him.

I: Mmm.

P: Now when they come here uh, they have light and fine.

I: Mmm.

P: We don’t trace a lot because the boxes help a lot.

I: Mmm you are not tracing a lot?

I: Okay.

P: They say the boxes help them [door opening] they say, “uh, they help us a lot.”

I: And please explain if [door opening] there is another community healthcare worker right-

P: Mmm.

I: Who does not know digital adherence technologies, smart pillbox, what can you say to them when you explain about the smart pillbox? They know nothing, you must explain to them. What can you explain about the program?

P: Okay, I can tell them that you can see when you go around the houses as a CHW.

I: Mmm.

P: You have to look at the patient-

I: Mmm.

P: When you arrive at that house, you tell her that you are asking for her box-

I: Mmm.

P: Huh, “can I please see your box as a CHW,” when you look at the box there are letters supposed to be ticked-

I: Mmm she opens when the box ring she does not tick.

P: Okay, uh I tell her to open her box ,but sometimes we also suggest that in order to see that she complies she must have a notebook-

I:Mmm.

P: Huh, and do the lines so-

I:Mmm.

P: So, she can tick, tick, tick, tick-

I: Mmm.

P: Then we compare with the box-

I: Okay.

P: Huh, we compare the tablets and check if the tablets are right.

I:Mmm.

P: They go with the dates.

I: Okay.

P:Mmm.

I: We check that okay, it goes well with the treatment and the box, he even takes treatment well.

I: Mmm.

P: Huh, [noise in the background] now you must look at the box if she takes medication correctly and ask her questions and ask how she is feeling.

I: Mmm.

P: When using the box.

I: Mmm.

P: Huh, so that she can have full information.

I: Mmm.

P: About the patient if she’s complying.

I: Okay.

P:Mmm.

I: Alright which other things you can explain to the healthcare worker who knows nothing about digital adherence technology. What can you say is important about what the box to the patient?

P: (…) [Heavy sigh].

I: You are explaining to someone who doesn’t know, they are asking what is this box for? How does it helps the patient? What will you say?

P: Huh, I will tell them that the box puts in medication.

I: Mmm.

P: You put in medication inside the box, it will remind you, it will ring. If the box ring, immediately if it rings, it means it’s time to take medication.

I: Mmm.

P: Huh, you have to take the medication and drink and close the box again.

I: Mmm.

P: Put it in a safe place.

I: Mmm.

P: Ehh.

I: Mmm, okay.

P: Mmm.

I: You did not tell me which follow ups are you doing with TB patients, what are you doing in terms of follow ups with TB patients?

P: Huh, I do follow ups, I look at the patient’s treatment if they comply well.

I:Mmm.

P:Ehh.

I: Mmm.

P: The right route to take medication and the right dose, if they take treatment correctly the way they explained and the correct time.

I: Okay.

P:Mmm.

I: Alright.

P:Mmm.

I: Okay, then when you say the sister- intern and the nurse talk to you and tell you about the patient who does not take treatment and you go and check, do you come back and give them feedback?

P: Huh, if I go to the field, trace and come back with the feedback that uh, I did not find that person or I did find the person.

I: Mmm.

P: If I did find the person, I do tell them to come to the clinic if they supposed to come.

I: Okay.

P:Mmm.

I: Ehh.

P: Mmm.

I: You do not find patients if you go to the field sometimes?

P: Mmm (yes).

I: What causes that?

P: Some give wrong numbers. Huh, you find that they gave the wrong house number.

I: Mmm.

P: If they come to the clinic, its difficult to find them and others move. You find that they were staying at number, maybe 1226.Or moved to another place.it very difficult that we can’t find them.

I: Mmm.

P: Even the phone number, you find that they have been changed.

I: Mmm.

P: The number is wrong; house number is wrong and cell phone number is wrong.

I: Mmm.

P:Mmm.

I: So, they give wrong address and wrong cell phone?

P: Mmm some of them.

I: Okay, how do you fix that challenge?

P: That challenge?

I: Huh, how do you fix that problem?

P: Huh, that challenge you find that uhm, we come to the clinic and tell them, if we come, we tell them to ask for the correct number when the patient to the clinic.

I: Mmm.

P: And the phone numbers.

I: Okay.

P: Huh, the correct one.

I: Mmm.

P: The patient must change the address and give the correct one where they stay.

I: Mmm.

P:Mmm.

I: Okay.

P: And others move, you find out that the patient was staying at Nkanini or Phutsane they have moved now they are at Mfiligwe, but they stay in Mfiligwe and come take treatment here. and the patient don’t update the facility that they changed address.

I: Mmm.

P: Mmm (yes).

P: Huh they don’t.

I: Mmm.

P: Other one stays at Mfiligwe but takes treatment here whereas they registered with the Phutsane or Nkanini address. now it becomes difficult to trace that person. Now we ask them that if that person come, they must tell them the right address or the right phone number.

I: Okay, please explain mother uhm, you who told you about the program? How did you hear about the program?

P: XXX [intern] was talking about it, its XXX [intern].

I: Explaining to you?

P: Mmm (yes).

I: Mmm, okay.

P: She wanted to know how it’s going.

I: Okay.

P:Mmm.

I: So, after she explained to you uh, about this technology how it works, please may I ask what did you think when she explained to you? Explaining that we give patients boxes, it reminds them, it has an alarm. What were you thinking about when she told you about the program?

P: Huh, I thought that it will help us a lot. Help us a lot because people, our patients say forget to take their medication.

I: Mmm.

P: So, now if there is this box, it will help to remind because it rings.

I: Okay.

P: Like a watch, it is set.

I: What does it do?

P: Its ring isn’t they set it to remind the patient.

I: They set it?

P: They set it, uh.

I: Okay.

P: So that it can remind you now, it helps them a lot because you find that others uh,” I forgot to take medication and I went to town.”

I: Mmm.

P: Now you tell him okay, before you go to town, first weight for your time and drink your medication and go.Take your medication or maybe if you are going to another place fetching wood. Start by taking your medication, mmm.

I: Is it the things you were thinking?

P: Huh, because you find that they forget to take treatment and go do another thing maybe they are fetching woods, or they went to town, the box do remind them.

I: Mmm.

P: Its like this system -this program, what they are doing and wish it can be done to others too who take other treatment,so that they can use these boxes to remind them.

I: Okay.

P: Mmm.

I: Alright.

P: Mmm [cough].

I: Okay, uhm, uh how did the intern train you about this program? Did she train you well? Did you understand everything about this technology?

P: She trained me even though I still don’t know other things like if I stay with her some other time so that we can do it.

I: Mmm.

P: To know it thoroughly.

I: Mmm.

P: Huh, learn more about this program.

I: Okay.

P: Huh, maybe if we can sit down and she teach me about the program, I will be perfect.

I: It can be better?

P: Mmm.

I: What do you think we must do to make sure that you know thoroughly about the program?

P: If.

I: What must happen?

P: Training uhm.

I: Mmm.

P: Training uhm.

I: Where must you be trained here or how should we train you?

P: Maybe- I don’t know if I say maybe that I don’t know how you do your trainings and where you do them? Where you do trainings it fine.

I: Okay.

P:Mmm.

I: Alright.

P: Mmm.

I: Who else do you think they need to be trained for this program, who can benefit, who help TB patients?

P: The community healthcare workers.

I: And who else?

P: [Heavy sigh] Huh, I don’t know.

I: Okay.

P:Mmm.

I: So, tell me about the benefits of using this program, what are the benefit that you see about using this box for the patients and as for you as a community healthcare worker?

P: I also benefitted a lot.

I: Mmm.

P: I benefited a lot.

I: Mmm.

P: That in the future I will be able to work for other people and know how to help them.

I: Mmm.

P: Huh, in this program, this program which is done by CHW can help a lot because we will gain knowledge, we will gain knowledge and have full information now.

I: Mmm.

P: Huh that our patients how are we going to train them or how do we reach their houses and see that uh, this one it’s not going well in this program which we have.

I: Mmm.

P: Huh, be able to sit down with them, sit them down and teach them, and also show them.

I: Mmm.

P: Huh, that uh, you do like this.

I: Mmm.

P: Huh, more, more, more information more than the one they knew.

I: Mmm.

P: Because they get short explanation at the clinic about the box, how they must use it.

I: Mmm.

P: But they don’t know thoroughly.

I: Mmm.

P: No, we know that we teach them that you are supposed to do what is right about the box, keep it in the correct place.

I: Mmm.

P: Mmm.

I: (.) Mmm, okay.

P: Mmm.

I: Huh, what are the challenges about the box? You explained the benefits, which are benefits of using this box.

P: Okay, maybe when they take the box home maybe they should not be careless.

I: Mmm.

P: Huh, patients, the children take it, go play with it thinking it’s something to play with.

I: Mmm.

P: They play with it.

I: Mmm.

P: Huh, that is the challenge, the children.

I: Mmm.

P: We suppose to teach them that they should keep it where children cannot reach, not where they can reach.

I: Mmm.

P: Keep it on top because children play with it, maybe one will keep it in place where they won’t remember, maybe the person is at work.

I: Mmm.

P: They don’t know where they kept it.

I: Mmm.

P: The person keeps it careless.

I: Mmm.

P: And don’t take treatment that day.

I: Mmm.

P: You, see?

I: Okay.

P: Huh, that is the challenge, they are supposed to teach them carefully that they should keep it where they will remember where they kept it. The children will not touch it or shift it and keep it somewhere where they will find it easy.

I: Mmm.

P: Then just say they cannot find the box, it’s lost, they don’t know where they kept it. Where my partner or husband kept it, or my sibling kept it or I don’t know where the children placed it and they are at school today.

I: Mmm.

P: Those are the challenges.

I: Okay.

P: Mmm.

I: Okay.

P:Mmm.

I: How can we address those challenges; how can we end them?

P: We can end them by that the person who takes the treatment supposed to keep the box in a safe place.

I: Mmm.

P: The right storage, where they will store it and keep it there.

I: Mmm.

P: Huh, that there won’t be a problem when they want the box, they will find it easy. Whey they want their treatment they will find it where they kept it. They should be the only one who knows that place or tell their children that you must remind me, I kept it here.

I: Okay, I heard you talking about the benefit a lot of using the box. What can we do to carry on uhm using the box? To carry on with the benefits.

P: Huh, the person when they come to the clinic, they should be given the box.

I: Okay.

P: Go home with it and take treatment.

I: Okay.

P: Because uh the box helps shame.

I: Mmm.

P: Mmm they help, and you find that the patient is alone, the patient is sick in house. Huh, when you go there, they don’t know where they kept treatment. The box helps sister.

I: Okay.

P: Mmm they help us a lot of shame, only if we can get the right training all the CHW.

I: Mmm.

P: Find the training and know everyone who’s on the treatment.

I: Mmm.

P: Huh, work together with them with the right program.

I: Which is the right programme which you can use well with the box when there is a box?

P: When we arrive at the house be able to see that the patient complies with the treatment and check the box.

I: Mmm.

P: We should ask questions, ask if you use-if they are fine to use the box and give information correctly.

I: Mmm.

P: How we should use it with the tablets, how you should take treatment on the right time, the right dose at the right time.

I: Okay, now if you think how should we do it to work together here in the clinic with the help of the box? There are community healthcare workers and nurses. What can we do to work together using this box?

P: If everyone can be the trained and know about the boxes how they work. , it can be better because we work together here in the clinic, we are one thing. We should all know about the box, how they work.

I: Mmm.

P: Huh, if they come with a problem, they should not sit in a line longer, they should know that the box works like this, this, this. Get help with a speed and go home.

I:Mmm.

P: Isn’t they came here with the box and set for a long time.

I: Mmm.

P: Huh, we all know the work we should do.

I: Mmm.

P: How we should help them.

I: Okay.

P: Mmm.

I: You have to know how to help them?

P: Huh, because others will complain that uhm if we brought our boxes here because they are not working, we stay in the line longer. You only here for the box not for the treatment, the treatment is inside here.

I: Mmm.

P: If we all know how it works, we all have that information we know uh, how to help them.

I: Mmm.

P: So, they don’t give up.

I: Okay.

P: Mmm.

I: Alright, so now this box what can you say, let us talk about the positive changes that were brought by this box. Which positive changes did this box bring in terms of treatment adherence and TB management?

P: Huh, it brought a big different.

I: Mmm.

P: Mmm.

I: What is that difference?

P: The patients adhere to treatment.

I: They adhere?

P: Huh, they adhere to treatment, even XXX [nurse] can see that. Others do adhere, they are doing well because they know that if they do not take treatment, it will report here.

I: Mmm.

P: Huh, she shows you find that I am here right and I see people from where I stay that they are doing well they are complying and she shows me.

I: Where does she show you.

P: On her phone.

I: On her phone?

P:Mmm.

I: Okay.

P: Huh, if she completes her treatment, she shows me the completed ones and the problem ones who do not comply who stay at other places.

I: Alright.

I: So, now the negative changes which are brought by the box, like we brought the boxes to the clinic. What are the negative changes you experience because of this box, because of using this box? Are there any things that patients complain about?

P: Huh, no they only complain if it did not ring others call me, “and I went to them (clinic) and told them that uh, they must call XXX [intern]. They must call XXX [intern].”

I:Mmm.

P: Or they can come to the clinic maybe there is a problem, they can fix it for them.

I: Okay.

P: Mmm.

I: How can we improve these changes or the negatives changes that they call them and say they did not take the medication. What can we do to improve those issues to make it better?

P: I can say maybe even us.

I: What do you do?

P: We can go to them.

I: Okay.

P: Everytime we go check that there is no problem with the box.

I: In the box?

P: If she takes her treatment, there is no problem in the box.

I: Okay.

P:Mmm.

I: Huh, you have to look in the box?

P: Huh, that she takes medication well, her box did not give her the problem and see if she takes medication well.

I: Okay.

P:Mmm.

I: Okay, the community healthcare workers go there?

P: Networking, yes, they should work together.

P: Mmm.

I: Okay.

P: Mmm.

I: Alright, is there anything we didn’t touch on about the program of ad- ad- technologies, adherence technology that we brought here in the clinic. Do you wish to tell me anything that we did not touch we did not talk about that is important?

P: [door opening] [pause] I was saying maybe-

I: Mmm.

P: Others you find that they complain, they say uh, they don’t have food, they want porridge Huh, you find that the person has not eaten anything, and they default treatment

I: Do they get the food here at the clinic?

P: Mmm.

I: Huh, do you think they should be given?

P: Huh, you find that others do not have porridge or food and struggle to take treatment

I: Mmm-

P: Huh, if they can get those porridges, it can be better. Others you find that uh, they say I have not eaten. I am hungry. You will find someone I haven’t easten now I have to take medication and am hungry, the porridge was better the one we used to get.”

I: Mmm.

P: “Huh, it was helping us.”

I: Okay, is there anything you want to tell us about the smart pill box we brought here in the clinic, that you want to tell me about that is important you are thinking about?

P: Huh, I can say I have said enough that the boxes are good for the program that you brought here, it has helped many people.

I: Mmm.

P: I wish it can reach other people, like those who are taking ART to remind them. They will not forget to take their treatment.

I: Okay.

P: Mmm.

I: Huh, because the patients- it helps them with not forgetting to take treatment?

P:They will not forget they have to take treatment, it reminds them right, once it rings ok or wherever you are sitting it will remind you to take treatment even if you are at your house, it will remind you that Mmm you have to take treatment.

I: Mmm.

P: Mmm.

I: Huh, alright sister uh, please, thank you so much PID xxx of date of the interview it’s xxxx (interview date), location of the interview it’ xxx [clinic name], type of the interview, uhm community healthcare worker, facilitator XXX [interviewer’s name] Huh, this is at the end of the interview and time of ending the interview it’s 11H25. Am - huh thank you so much sister for the information you have given us. Thank you very much.

P: Thank you miss XXX [interviewer’s name].

I: We really appreciate, thanks.

P: Thank you.
