# Supplementary material for: Acceptability of Digital Adherence Technologies to support people with drug-susceptible TB in South Africa
Source: PLoS One. 2025 Sep 24;20(9):e0332103. doi: 10.1371/journal.pone.0332103 (PMC12459780; doi:10.1371/journal.pone.0332103)
Supplement: S4 File — (ZIP) [file pone.0332103.s004.zip › S4 Transcripts/HCWs and Stakeholders/IDI 22-HCW.docx]

**TRANSCRIPTION NOTATIONS**

| **Label Key** | **Meaning** |
| --- | --- |
| **I** | Start of each new utterance by the Interviewer |
| **P** | Start of each new utterance by the Participant |
| **N** | Note taker |
| **{ }** | Indicates that details were changed or pseudonyms were used to anonymise data |
| **( )** | Indicates the description provided to anonymise data |
| **XXX** | Words were omitted to anonymise data |
| **-** | Breaking into a sentence by the next speaker |
| **…** | Pause or drawn out words |
| **[ ]** | Indicates noise made, e.g. [laugh], [sigh], [pause] |
| ? | Beginning of utterance by unidentified speaker or questionable text |
| **[inaudible segment]** | Unclear section of the recording |

I: So do we have permission for us to record you?

P: Mmm, yes.

I: Today's date is: xxxxx (interview date): PID: xxxx. Facility name XXX [name of the facility]. [Turning pages] The language used is: English. The time is: 10:54.

I: So can you tell me what your current position is? Or what was your position in the project?

P: Mmm, my position was to give out the boxes. I was an intern so I would give out the boxes.

I: How long did you have this position for?

P: One year?

I: Ok. So, in addition to giving out the boxes, what else were you doing?

P: Basically everything at the clinic that has to do with TB. I would help out the nurses with whatever the nurse would do, we would help out with basically everything that has to do with TB. I would help the nurses, I would even help out with the files, everything that we can do to help at the clinic I would do.

I: Ok, so when you say everything, Can you unpack that a bit?

P: Mmm ok, at the clinic besides giving out the boxes, we would take out the files, help at the reception, we would huh –maybe- how can I put it we would help out where they check the temperature I would help out with patients that come.

I: And then in terms of the project, what exactly where you doing? There ASCENT project.

P: For me basically, for ASCENT project, I would give out the boxes but the nurse didn't allow me to like, go in like take out the saliva when the patient has TB like he would do everything because at first we were told that they should do everything because after the project they're going to do everything on their own we won’t be there. So my on my part I would focus on the patients and the tablet and see if the patient is taking medication or not. So he would do everything.

I: Ok, so you were just giving the patient the box?

P: Yeah, and sometimes you would talk to the patient if the patient doesn't want to take their pills and like I would counsel them and tell them that it's important to talk to somebody and so on.

I: Ok, so you were also involved in counselling?

P: Yeah.

I: And before you issued the box, what were you doing?

P: At the clinic?

I: Yes.

P: I was just volunteering, volunteering, just helping at the clinic not being paid just a volunteer.

I: Ok. So can you tell me the process of giving the box to the patient? So if a patient, a TB patient arrives, what was the process of issuing the box?

P: Ok, when I first got at the clinic, we obviously, we didn't understand and it was just a mess. So for me, every time on Monday its TB we used to call it TB Monday because on Monday, the nurse checks with only people with TB they come on Monday. You can come on Tuesday or whatever you can come if the nurse gave that date but Monday, they would get in a line and stay there and wait for their nurse to come. So I would not make them stand in their line. I would take their cards they would be there at the back. They even knew my name. They would stand there at the back I would take their cards, take out their files pack them and give it to the nurse so that the nurse can check them and help them because if I didn't do that, they would stay there long. And one would come I would miss out one and like it was just a mess. So if I took out their files, then I know these are the TB people and they would stand in line and I would know when they come out. I would come and tell them that I'm XXX [intern’s names] from ASCENT and I would explain the whole process to them. That's what I did when I got to the clinic.

I: Ok, so can you tell me that part of explaining the whole process? What exactly we're you doing?

P: I would sit down and tell them I'm XXX [intern’s name] from ASCENT project. Most of them even knew me because I was willing to tell them that we giving you this box, because it will help you because sometimes there is no electricity you would set on your alarm and there's no electricity then the alarm doesn't ring because you have to drink your medication early in the morning. So I would tell them that it's important to take your medication so that you can get well, and this box would help you to be reminded so that you don't forget. It's not for storing lipstick or money box or whatever. We would tell them that it's important. We did this project and we making this project so that we can see if it's going to help people so you can refuse if you don't want to use this box. You can use this box. Then I'll explain going through the huh ASCENT paper explaining everything to them. It's important to actually sign and so on so,

I: Ok, so they were signing?

P: Yeah, they were signing. That’s the first thing you should do even like before, after they agree that's the first thing you should do make them sign before even giving out the box.

I: So if you were to explain what huh DAT (Digital Adherence Technology) is, the DAT intervention is to another healthcare worker, what would you tell them?

P: Saying it to another health care worker?

I: Yes.

P: I would start to explain and tell them that. The box is important, including this project. It's important because it helps people, especially people from XXX [facility], they live in places where there's no electricity. This lady I helped even hugged me and cried. It was like I'm giving her money or something, she was like “this box is helping me because where I live, there's no water, there's no electricity, there's nothing. So this box is basically reminding me because sometimes I would have stress and I would forget to take my medication. So this box is really helping me”. So I would tell the CHW that this project is very helpful and it really helped a lot of people from my understanding.

I: Ok, besides the box, what else is included in this digital adherence technology besides the box.

P: besides the box?

I: Yes. The technology, the DAT is about the box and what else?

P: Helping people.

I: Yes. So ok, when the patient takes medication like you're using the box, how do you know that the patient is taking medication?

P: They gave us tablets so when they open it you can see that the patient has drank their medication when the patient hasn't taken their medication an SMS will be sent. Then after 2 days or 3 days we take the phone and call the patient and ask them what's wrong. Are you taking your medication or is it the box not showing us on the system?

I: Ok, so what's the name of the system on the tablet you were using?

P: The name?

I: Yes.

P: DAT.

I: Ok, so when you get to the tablet, where would you go for you to see the patient?

P: The patient information?

I: Yes,

P: Yeah, you would click on the names and there are numbers. So you know your patient and you click on the on the information so that you see everything, the patient's name everything, the number everything.

I: What’s the name of that application you were using?

P: I forget [laugh]

I: Ok, so you mentioned something about making phone calls to patients who do not take their medication, yes, and what was your role in this differentiated model of care? Do you remember what differentiated model of care is?

P: Yeah, my role was to make sure the patient take their medication, I call and after calling I add information and write on the on the task list or something we used to write information and write about the patient that I called this patient today, this patient said they took their medication or this patient said they didn't take their medication. Like you write information what you heard from the patient, you just write everything.

I: Ok. So how were you identifying the patients who have not taken medication?

P: You would see when they come to take their medication that this one is not getting well because those who really took their medication, you would see when they come that this person is really taking their medication because they would get well even though when you call them you ask them did you take your medication then they say yes yet they just opened the box and close it and when they come you would see that this one is not getting well. We did our best we called and asked if they did take their medication and the will be like yeah I take my medication. Yes, I opened the box but it did this. Then I will tell the patient here it's it shows me that you didn't take your medication and I'm sure you got the message and the patients says it's this box of yours bla bla bla bla, but you would see that there is something wrong with the box or this patient is really not taking their medication. So you would tell if it's the box or this person is really not taking the medication.

I: Ok. You mentioned that some patients would just open and close the box. What could be some of the reasons why they were doing that?

P: There was this lady who was saying, “I can't take this medication because my stomach gets sore- I can't eat it’s so painful. I'm getting rashes, its itchy” *eng eng* (*what what*) some patients didn't want to take their medication because of the effects.

I: The side effects.

P: Yeah side effects.

I: Ok. What other the reasons that were mentioned?

P: There was this other patient. He left to go to XXX [country] so I called, I called, I called the phone didn't go through. I called and I called the phone but when he came back, I called him I didn't even know that he came back. I tried calling because the sister was saying XXX [interns name] what’s wrong with this patient. So when he came back I called him to ask what's wrong  your box is just red and your phone calls didn't go through I even thought you changed the numbers or maybe because this person didn't take a transfer. So he said to me, he left the box. He left the box at home, took out the medication and left. So he didn't want to go the box because it's too much baggage. Like he had too much bags and the box is adding so I felt like he was scared that people at home will know that he has TB because the box rings so he had to tell them or explain that this box is for 1-2.Then my other patient told me that I must turn off the alarm. When I offered her the whole thing I explained and she was like "okay, I want the box" but then after 2 months she told me that I should turn off the alarm.

I: Why did she want the alarm off?

P: She didn't say she just said the box is fine because today it would be red tomorrow it would be green. 2 days, red 2 days green, like not consistent. So I was like what's wrong? Can we meet and talk? I think she didn't want people to know but at home I think they knew.

I: And then in terms of home visits, how you're managing that?

P: CHWs. I would send a CHW (Community health care workers).

I: Ok, and did the CHW give you feedback when they come back?

P: Yeah. They would then I would write it on the tablet.

I: Ok, so what were some of the reasons you got from the CHW for none adherence?

P: Mmm the one patient didn't take their medication. She said she's taking her medication but she didn’t take her medication so when the CHW got there she’s like me, I opened this box I drink every day. I don't know maybe its this box of yours and stuff but you could tell gore (that) this patient is not well. So she just made an excuse that it’s the box. It's not reporting right things to me.

I: So how were the responsibilities shared between you and other health care workers? Like you've mentioned that you would tell the community health care workers the list to do home visits, how else were you sharing this follow ups task responsibilities?

P: Mostly I would do it on my own because the CHW's today would come this one tomorrow it’s this one tomorrow it's that one tomorrow but when the project started, our manager called all of them to explain that there's a new project in the clinic 123123 but it only happened once that I sent a CHW to go do something for me. The rest of the things I did alone, they only knew what was happening. They were not involved.

I: Ok, so were you also doing the home visits alone?

P: We only did it once the rest I would get them when I was at the clinic or I would get them through a call, but the home visit just happened once because this patient was really sick and it was maybe 10 days, 11 days without opening the box. She was really sick.

I: Ok, and when you first heard about the Digital Adherence technology, what were your expectations before you started implementing it?

P: It was exciting, because obviously even though I was at the clinic but we were not involved in such things as TB, HIV, we didn't know much. We were just volunteering basically there in admin fixing files, but when I first heard it was interesting because it was something new. And now everything is about technology and improving everything so it really made a difference, especially at the clinic. They even made me a little party celebration because at the clinic, TB people were not adhering but when we came with boxes at the TB room. There were less files, like it was just- So yeah.

I: That's wonderful. When you first heard about the box, did you think it was going to be easy?

P: Not really, easy, but I just thought that there are people who are stubborn and there are people who don't like other people to know that they have this kind of sickness. So I was just worried the box was going to ring and they would have to explain that so and so will know that I'm drinking this medication. Every time they leave the clinic, they would come with a big bag and put the box inside because people would ask what this box is for I also want it. People would ask boxes from me and I’m like you can't get the box because I didn’t want to tell them it’s for TB. So yeah, the box helped them but they didn't want people to know what it is for and why they were taking it but they appreciated it. It really helped them, but some were stubborn and be like it rings the whole night it's ringing more than once it’s irritating. They would be complaining some, but most were adhering nicely.

I: So you mentioned something interesting about possible stigma, whereby you were concerned that patients wouldn't want the box because it rings. Did you have any patients who told you that?

P: Yeah. The school child didn’t want the alarm it irritated her or whatever.

I: Ok. So were there any reports of stigma from the time you were implementing?

P: Mmm, mmm.

I: Or a patient fearing stigma or telling you that?

P: No. I don’t want to lie.

I: Ok, so did your opinion change when you started implementing? Because remember you said when you first heard about the box you were concerned that other patients might be worried?

P: Yeah it changed, because I saw a patient would come there was this mother with her 3 kids, they had TB and its small babies not like babies but one is 12 or 11. So they were really sick. After I gave them the box. It was maybe 2/3 months they were so different. They were telling me that- I was asking is the box giving you problems when they come to the clinic? No, it's not giving me any problems. And the thing is that with my patient every time they come on Monday I will check on them and not waste their time but have a little conversation with them and find out how they are doing because that's how the nurses like having this relationship with patients. My manager XXX [managers name] would work with him at the clinic and you go to his room like you would stay there 30 minutes or 1 hour like him finding about everything about you, making sure you’re ok, so in that process, I would share a room with him then I would find out hey, this patient is really happy the box is helping this patient. So the mother was really excited even the kids looked better. Like it changed people's life for me. Yeah, I think it had- it had problems like other patients would complain but most majority 90% they were so happy and I saw changes in them.

I: Ok. So can you tell me about the training and resources that you received on the delivery of the implementation including the differentiated model of care? Do you remember the training that happened before you started?

P: At the training we basically learned a lot even counselling people, I had no idea how you start talking to somebody who's sick. Where do you start? How do you console them or how do you do whatever? So at training, we really learned a lot.

I: So what was your first impression about the training?

P: [Laugh] I was excited because it was literally my first job, so I was excited.

I: Ok, do you think the training was comprehensive?

P: Yeah, it was. Yeah, I think it was.

I: Do you think it was useful?

P: Yeah, it was, because without the training, we would have no idea.

I: Ok. Do you think it was sufficient?

P: Yeah, it was for us. It was.

I: Ok. So how often do you think healthcare workers maybe if they are to start implementing this should be trained?

P: How often?

I: Yes, how long should be that first training?

P: Even like we took a week.

I: A week?

P: Yeah, I think it was a week. Yeah, a week is enough. I don't know. Maybe I'm not hard headed but for me a week is enough like yeah, because it's not it's not difficult you just add names. You just check. It's not. It's not yeah.

I: Ok, all right,

P: Maybe when it comes to the Redcap, but not the first training.

I: Ok. So did you have patients who do not disclose their TB status to families?

P: Mmm. They would not say but you could tell, like the man I told you about who went to XXX [country] you could tell that he was hiding the box. That's why he didn't go with the box because he made an excuse that his baggage was a lot because he had a lot of bags and stuff but so I think he didn't want his family to know. He didn't want to to explain why this alarm, why this box, this device.

I: Mmm. So from your perspective, can you describe the benefits of differentiated model of care and the use of the box?

P: The benefits of the box? It helps, first thing it helps you and it reminds you to take your medication. Mmm it helps you to put your pills in one place Because most of the time we put our pills where ever maybe you have high blood sugar and stuff and you mix them you don't know which one you took and so it's safe for you, you put them in one place so that's the benefit of the box. It helps you to be reminded the alarm reminds you to take a medication like if you drink at 8 o'clock every day it will be 8 o'clock because you don't have the alarm today at 8, tomorrow at 5 o'clock, you'll forget tomorrow this time and this time and this time.

I: Ok, Can you think of a patient and reflect on this issues you're raising?

P: A patient that got helped from?

I: Yes.

P: There was a pregnant lady. There was a pregnant lady she was like huh "I'm taking high blood medication now I have TB, huh this is a lot for me". She's like "I'm taking even for pregnancy this this medication they gave us in the clinic it's a lot. Now where am I going to put this box?" And I'm like oh do you have TB? Okay, come on. Let's talk. I took her to the room and told her about the box and the whole thing. So she was like, okay, that's nice. Ok my TB pills I will put them in the box because it's just 6 months. I'm like, yeah, if you continue taking your medication right it will be just 6 months. So it helped her because she put the medication in one place. She didn't have to mix with the high blood medication and the pregnancy medication.

I: Ok. And then in terms of huh differentiated care. what are the benefits of differentiated care? The phone calls and the home visits?

P: The benefits. It was. [Car hooting] I think it was because here there is information where you write after you called your patient and you can reflect when you look at the patient if the patient is drinking medication or not. We can reflect back like for example, you call the patient today and wrote the information then you called maybe next month, then you can reflect back and check gore (that) this patient said the same thing the last time is this patient lying to me or not? So differentiated care helped us a lot. It really helped us because everything even information was in one place and we didn't disclose the information to anyone it was with us.

I: Ok, what about the relationship you had with patients?

P: Yeah, it was good some even offered me money I'm like huh I can’t, [laugh] some came with sweets and stuff ok food I would and be like thank you XXX [Aunty] for lunch, but money. I didn't take because it will be like I'm giving them the box then I want money. Only few were stubborn and  I had 2 patients who are not mentally well, so with them, because sometimes they would not come to the clinic sometimes they would come but my relationship - they even knew me even now when I am not at the clinic XXX [manager] would call and be like XXX [intern] just come one day and come check come check us because people are asking about you. They still remember they ask where is XXX [intern]. Where is nurse XXX [intern] [laugh]?

I: That’s nice.

P: [Laugh] I was nurse XXX [intern] because I have a friendly face so even if they didn't come for their TB thing they would like please help me sister XXX [intern]. I'm late for work. I'm like *wena wasokodisa (you are trouble)* and I would take out their file and they would get the help and stuff. So even if it's not for TB, they would come to me.

I: Ok, that's good. You have narrated the benefits of the box from the patient's perspective. What about from the healthcare workers perspective? What were the benefits of this digital adherence platform, the box and the adherence platform you were checking.

P: Ok let me say the nurse, not the CHW’s because the CHW’s they didn't have much input or whatever help, but the nurse yeah.

I: Yes

P: I was working with XXX [nurse] he was the one excited about this whole project. He even wanted to learn more, because he was like, yoh, you're leaving, and when you are leaving. I'm going to struggle. So he also had relationships with the patient because if XXX [manager] is not there, he's the one giving patient’s medication.

I: And then in terms of monitoring adherence for this TB patients, how did this technology assist the nurse?

P: For XXX [nurse], he tried to participate in monitoring and actually I would force him to  just take two minutes to show him. I would go to him and show him that this one patient goes 123 and 123. Then when they come he would know because  he would see on the tablet. So he would know that okay this one is the one that XXX [intern] spoke about that sometimes he drinks medication or not. So he would be like we see you. Yes we are tracking you. That box is a tracking device. If you don't take medication, we can tell so I know you're not taking, they will be like XXX [nurse] I’m sorry last week Tuesday and Monday I didn't take . Yeah, serious is the box tracking us?

I: Ok. So did it assist in monitoring patients?

P: Yeah, it did because it would tell you everything it did. It did.

I: Ok. And can you describe the challenges of using the differentiated model of care? Let's start with a differentiated model of care, phone calls and home visits. What were the challenges?

P: For me I didn't have challenges because we always had data, airtime. The only challenges was when a patient is not answering or when they blocked you. So there is nothing you can do. You would send a CHW and you find out they went to work. When the CHW gets there, they're not there then there's nothing you can do. You have to wait for them to come to the clinic where you can confront them.

I: So what were some of the reasons for them to block you or not answer?

P: Because I would call XXX [patient] why aren’t you taking your medication, they would say I am taking my medication that box is lying or makeup a story but it's not always. Sometimes they forget so maybe I would annoy them by calling and asking it's been 3 days what happened because everybody needs their space and maybe they have problems so I'm adding to their problems and calling and asking.

I: And were there issues in terms of network?

P: For me mmm, mmm, because other people complained about Cell C, Vodacom, but for me MTN was ok.

I: Did you always have the correct number from patients?

P: Yes, because we would make sure at reception, we would tell them if you don't bring your number, the correct numbers we are not making a new file for you because they always do that. They would say yeah, we're not going to help you without an ID, correct number, correct address and say call your husband we need ID, we need right addresses. So I took that information from the file. I didn't take it from them. I took it from the file, they would give me but I would double check on the file.

I: Ok. So how were they verified? That is the correct number.

P: Mmm. Mostly, I think mostly they would give correct numbers because XXX [manager] would call them, our manager calls them to come take their results. With results, like when they took your saliva for testing, and they call, you know, come next weekend and so they had to give right numbers because they knew that they were going to be called to come take their things.

I: Ok. And then what were the challenges with home visits? Were there any challenges?

P: Mmm, mmm. No the only thing you would find out that they went to work and the patient maybe knocks off at 5 and the CHW 1:30.

I: Ok. So were there issues of stigma when trying to call or sending a community healthcare worker for home visits. Did you pick up any stigma?

P: Mmm, mmm.

I: And were the community healthcare workers always available to do the home visits after 4 days?

P: Mmm.

I: And then are there any patients who declined to take the box when you offered the box?

P: Not necessarily declined but were given- like when we came to the clinic they will tell us that take people that maybe like 2months, 3months new patients, but huh patients that mostly I didn’t put on the DAT were people that were left with 1month or 2months. So I didn't even ask them because it was 1month or 2months, so yeah.

I: Ok, so there were no patients you tried to offer and they said no I don't want the box everyone you offered the box agreed to take the box?

P: Yeah.

I: Ok. And huh in terms of the box itself, what were the challenges with the box?

P: Patient’s would say the alarm rings more than 2 times if it was supposed to ring only 1 time, it rings 2 or 3 times a day. Or yeah, basically it was mostly about the alarm.

I: Ok, so what were the other issues they reported besides the alarm?

P: Maybe when they were putting the box on top then it fell then it's now messed up so I have to change and give them the new one. And yeah, I would call XXX [Research Assistant] he would help me with number of battery. He would change it and give the patient a new one.

I: Ok, so how often did that happen? That a box can fall gets damaged and-

P: it happened once.

I: Ok, so can you reflect on that patient who had a time they were not adhering on the platform because it was damaged?

P: No, it happened gore (that) it was It was damaged on yeah, I think it was on a Friday so she called me and she was like yo ausi (sister) XXX [Interns name] this box just fell. So I don't know should I bring it or not? But the alarm kept on ringing fine. So she was not comfortable gore (that) there's a hole you could see inside the box and stuff but she was like, Can I please have a new box? Of course it's fair that this patient is requesting a new box because that one fell XXX [intern] Then XXX [study coordinator] was like is it giving her problems? I'm like mmm- mmm, it's not so we basically changed the battery and put it in a new box and gave her a new box but the alarm was ringing fine. It didn’t have problems.

I: Did it have any effect on the adherence calendar?

P: No it didn’t

I: Then huh from your perspective, can TB treatment be improved using this technology, the medication box?

P: Yeah, it can. It can be improved, because basically as soon as this project came at XXX [facility] clinic let's say-. Let me talk about the clinic I worked in. It really improved the stats of TB. Even when I went last week, XXX [manager] was like yo I'm struggling. There are a lot of patient’s, it's December time and I'm struggling I wish you were here to help us because now patients with TB are more patient than when you left and we just struggling there’s lost to follows even though they send CHW’s there but yeah.

I: Ok. So how has the box assisted? How do you think the box will assist with TB treatment?

P: It assists because it helps them, it reminds them to take their medication. Basically you take medication at the same for you not to confuse your body system by taking it today at 1 o'clock tomorrow, 8 o'clock at night or mixing or whatever. So it helps them to take it on time and it's important to take it in the morning.

I: And then in terms of the patient provider relationship, how does the box and the adherence platform improve that?

P: Sorry?

I: In terms of the patient provider, the relationship between the patient and the TB nurse or the patient and you, how does the technology ensure that the relationship is improved and patients can take treatment?

P: Mmm because like I said they gave us airtime to call patients so even when I was with the nurse, we both would call the patient and ask them *gore (that)* are you taking your medication. So the technology makes it easier, yeah, it really makes it easier because even the CHW’s didn't do much because we had cell phones and we made sure that even when you come next time we ask you did you change your numbers. Can we have your wife's numbers or your brother if you don't mind then they say *hai* (*no*) not my brother take my wife’s. Take my wife’s my brother talks too much. Whatever so like, its fine so your wife knows? Yeah, my wife knows my wife knows Okay, take my wife's numbers. Yeah. When I call the person and the person is not there, I call the wife and she’s like, huh, is he not taking medication? I'm like no *mama (Mom)* I'm asking if there is something wrong with the box? Or is your husband not taking medication I just called to confirm and know, she will them say she will ask him when he comes out of work. Like that relationship.

I: So you also now developed a relationship with family members through this differentiated care?

P: Yeah, even when they sometimes would come and fetch the pills for their husbands they will be like *sifhuna uNurse (we want)* XXX [interns name] or they would call nurse XXX [interns name] is my husband coming to take medication tomorrow please check for me. Then I'll go to reception and make sure it was the right date, yes tomorrow your husband is coming.

I: Ok. All right. And then in terms of monitoring the patients. Do you think this technology will improve monitoring of patients?

P: Yeah, because it was simple. Green, the patient took the medication, red means the person didn't open their box. It was simple and easy.

I: Ok, so how were patients monitored before this technology?

P: Through CHW's and for the nurse that I was working with thought on the tablet it's better than when you go to their house because sometimes people want their privacy and they would say ole batho bakho clinic (people from the clinic) are always there in their houses. Why eng-eng (what what) like stigma and stuff. So it was better checking through the phone than always sending nurses and CHW’S at their house.I: And then in terms of the workload, did the technology help with the workload in terms of monitoring?

P: Yeah, it did.

I: How so?

P: It did because everything was on the tablet and the information. If there's something missing, you go on your tablet or you re- check on your consent form if there's anything missing or you check on your file.

I: Ok. So can you tell me the positive changes that have been brought by this technology in terms of supporting TB patients?

P: This technology made a difference because patients were excited. Before I came patients were lost to follow because I had to start and check those files and whatever but before I left when I did Redcap it was treatment complete, nobody was lost to follow or nowhere to be found or not taking their medication because I would talk to them and counsel them and say if you're not taking your medication, it's really bad for you and you're not going to heal and it's just 6 months and I would try and talk to them.

I: Ok. So in the absence of xxx (organization name) or of interns like you were, how do you think these positive changes can be sustained in the facilities? So what can be improved at the facility for these changes to be consistent?

P: I think maybe if they could involve the CHW (Community health care workers) to do most of the work that we did, because now the nurses will be doing it but half of it because the nurses are always busy. At our clinic if the nurse is not taking calls or not giving the patient pills, he's busy at emergency and the line would still be there and the patient will be annoyed and say "hey, I'll get the medication tomorrow". So if they would involve the CHW because we are not there anymore and we did almost everything like we helped them a lot. So if they could involve at least the CHW’s to help the nurses, that would be better because the workload would be too much alone for for the nurse alone. It'll be too much.

I: Ok in terms of even giving the box and monitoring?

P: Mmm. I don't think the nurse would do it alone. I don't think the nurse would do it alone

I: Why?

P: It's because at the clinic for us at the XXX [facility] clinic we are near the mine and its always packed. There are a lot of people who are renting that side and there’s rural areas where people stay. So it's always packed, sometimes the nurses would start working from 7:30 until 4 without even going to lunch. So when will the nurse have time to check and monitor? At home and call them when at home? What time yeah, so I think the CHW should help. Not do everything but just help, just put a hand.

I: Ok thanks for that information. Can you tell me about the negative changes that have been brought by this differentiated model of care or the use of the box?

P: For me? There's nothing negative.

I: Ok, you once mentioned that there were patients who were opening and closing the box but not taking medication?

P: Mmm, mmm.

I: How do you think that can be addressed?

P: I think huh like that patient should get help. They should be told like I did. She ended up taking the medication because we told her that the reason why you are like this, you are getting more sick is because you are not drinking your medication because we can tell when you come again for your check up that people are gaining weight and getting better but you just losing and losing and losing. So you need to, you have kids you need to take care of yourself and take this medication. TB can be cured. So there should be more counselling and people should understand what TB is it can be cured.

I: Ok.

P: They should understand.

I:You also mentioned that some patients were not answering the phones when you're following up? How can these also be addressed?

P: That’s where the CHW’s get in and check the patient at home.

I: Ok, and mmm was there any stigma reported by a patient because of their participation in in the project? Maybe because a phone call was made or they were seen with the box or the box rang?

P: Mmm, mmm only a patient when I said can I have your brother's numbers or your wife’s? That’s the only time he said, hey, not my brothers. He talks too much not my brother's but besides that, mmm mmm.

I: Ok, and then that issue of the patient who left the box behind going outside the country, how can that also be addressed?

P: Huh on that I have no idea how can that be addressed but I think it was a matter of the the box ringing. He didn't want to explain to his family why is he having this box ringing and explain what's the box is for. Yeah, He didn't want his family to know. So we have to respect people's spaces and decisions gore (that) they don’t want people to know or to disclose their status.

I: Ok- ok. So what resources are needed in facilities for them to continue with this program on their own without the support of xxxx (organization name) or anything, what needs to be in place?

P: Yeah, if they have their boxes, tablets, airtime, data [laugh]. Yeah. Then the nurses will take it from there.

I: And who should be preparing the boxes, the charging and so forth?

P: The CHW’s, they should train them or they should hire us back [Laugh]

I: Ok. So what else do you think is needed? So if huh there's a technical glitch or something who should be assisting?

P: Yeah, that one is tricky because we used to be assisted by XXX [study coordinator] or XXX [intern] so I think they should be maybe data captures because they know a lot about IT and those things or maybe if they teach one of the data capturers something about recharging and fixing the glitch on the box. Yeah.

I: Ok. So, can you describe to us how you were monitoring the differentiated care?

P: Mmm monitoring it through the tablet

I: Yes, we were you capturing the issues and the challenges?

P: Yeah, immediately when you find an issue about the box, before even calling I would write it down and say this patient 123123 and write it down, then call XXX [study coordinator] after fixing it. Then write again the information. Because you will forget if you don't write it immediately. So we taught to write the information immediately. But sometimes you would forget and write it at night. Because I would leave with my tablet I didn't leave it at the clinic I called this patient and I didn’t write. I write it quickly.

I: Ok. And then in terms of this thing of recording any problems besides the tablet. Was there any other place you were documenting these issues?

P: Yeah on the ICF’S yeah, we did record. We used to have papers to record and we write *gore (that)* this is what happened.

I: And then the clinics? Do they have systems for that? Where they document challenges and successes?

P: Mmm. I think at the reception data captures they would write, but not like we did. Us we would write every information. Every little information was important.

I: Where do they write?

P: Under the person's name on the computer. There’s where they would check for me where they check the date when the person is coming back or any information. They will have information but they didn't have much.

I: Ok. So can you tell us the gaps that were in the way the intervention was delivered? And if yeah, were there any gaps?

P: Gaps on?

I: How the project was run?

P: Mmm, mmm for me, no.

I: Ok. Do you have any suggestions for improvement?

P: On?

I: On the project, like the box, the differentiated model of care and the way it was implemented?

P: On the box maybe if they could work on the glitch of the box, but obviously it's not going to be perfect but people were complaining especially other clinics. Maybe I was lucky yeah, I got good boxes but other clinics when we met or when we went to training they would complain that my box has this network and complain about network because of the box. some complaints I didn’t even understand because I didn't have any problems but they would complain about network problem and they would complain about things not showing on the tablet. Yeah, maybe if they could work on those things but I didn't have any problems with it myself.

I: Ok and then with patients who have other diseases besides TB, what will be your suggestion in the use of the Digital Adherence Technology because this was only meant for TB medication?

P: Yeah, I think I think even if maybe they could use this box, even for HIV, maybe mix the medication. I don't know if it'll be a good idea or not. But because most of the patient who had TB, most they had HIV, and they- they their medication. I don't know if they drink it on the same time or not. But I think it would help if they used it for HIV also, because most most patients 90 who had HIV they had TB.

I: Ok. So was there a special group of huh people who benefited more from the use of the box or you think maybe people of a certain age group or?

P: Yeah, older people. Older people were mostly the ones who I think benefited because the the box helped them because they would come to me and tell me that yho I would forget to take my medication I would remember this one and forget this one. So hey, this box yago (of yours), older people, younger people, I think they remember but older people. It helped them because they would forget.

I: Ok. Did you have any homeless patients in your facility?

P: Not homeless. I think there was this one, but I don't think he's homeless. He just complained *gore (that)* where he stays is not a nice place, there's no water and he doesn't have food where he stays . He didn't want to explain *gore* (*that*) is he homeless or what, but the address he gave us was a home address it was fine. In terms of numbers and stuff he had a phone, a small phone but he looked like a person who was struggling so much. Also, the woman who had three kids with TB didn't have clothes for the kids. She was complaining that this medication is making her kids eat and she doesn’t have money to buy food. *Yho* and I didn't know what to do. I didn't even know how to answer. So I took her to XXX [manager] so that he can talk to her because I didn’t know how to address the food situation.

I: Ok. So you're saying in addition to the box supporting?

P: Mmm.

I: There are other challenges. So what are those challenges?

P: People not having food, places to stay and because they would complain how can I drink medication when I don’t have food? I'm not drinking this medication because I don't have food akena dijo so konnwa jwang (I don’t have food so how will take) medication because this medication makes my stomach itch and and and so when it came to those things, people asking me for food. I didn't know how to address that and I took them to XXX [manager].

I: Alright. Thank you very much for your time. XXX [intern], do you have any comments on the project, the box, the xxxx (adherence platform) platform?

P: My comment is xxx (organization name) should just keep doing the good work that they did because this box helped a lot of people they may not see it, but it really did a lot of change especially to my clinic because people there really struggled with TB because it's a mining area. So it's so easy to get TB so it really helped people take treatment.

I: Ok. Thank you very much XXX [Interns name]. We have reached the end of our interview the time is: 11:40.

GLOSSARY:

*Eng eng (What what)*

*Ke box eyalona (It’s your box)*

*Ke box ya eng linna ke batla box (What is this box for I also want it)*

*Iyakhala ubusuku bonke (It rings the whole night)*

*Wena wasokodisa (You are trouble)*

*Gore (That)*

*Ausi (Sister)*

*Hai (No)*

*Sifhuna (We want)*

*Wena (You)*

*Batho (People)*

*Akena dijo so konnwa jwang (I don’t have food so how will I drink)*

*DAT Digital Adherence Technology*

*CHW Community Health Workers*
