# Supplementary material for: Acceptability of Digital Adherence Technologies to support people with drug-susceptible TB in South Africa
Source: PLoS One. 2025 Sep 24;20(9):e0332103. doi: 10.1371/journal.pone.0332103 (PMC12459780; doi:10.1371/journal.pone.0332103)
Supplement: S4 File — (ZIP) [file pone.0332103.s004.zip › S4 Transcripts/HCWs and Stakeholders/IDI 23-HCW.docx]

**TRANSCRIPTION NOTATIONS**

| **Label Key** | **Meaning** |
| --- | --- |
| **I** | Start of each new utterance by the Interviewer |
| **P** | Start of each new utterance by the Participant |
| **N** | Note taker |
| **{ }** | Indicates that details were changed or pseudonyms were used to anonymise data |
| **( )** | Indicates the description provided to anonymise data |
| **XXX** | Words were omitted to anonymise data |
| **-** | Breaking into a sentence by the next speaker |
| **…** | Pause or drawn out words |
| **[ ]** | Indicates noise made, e.g. [laugh], [sigh], [pause] |
| ? | Beginning of utterance by unidentified speaker or questionable text |
| **[inaudible segment]** | Unclear section of the recording |

I: Thank you so much for agreeing to talk to us today, can you please allow us to audio record this interview?

P: Yes, you can record it.

I: Okay, PID number is xxx, date of the interview is xxxx (interview date), location is xxx [clinic name] , type of the interview is healthcare worker, facilitator is xxx [interviewer’s name], time of the interview is 10:31. Alright sister, can you please tell us what is your current position at the clinic?

P: I am an enrolled nurse, mostly I am working with TB patients.

I: Oh okay, what exactly are you doing regarding the TB patients?

P: I consult on children’s site, Expanded Programme on Immunization (EPI), family planning, chronic, that’s most of the job that I am doing.

I: Oh, okay when it comes to TB management, what exactly are you doing with TB patients?

P: For TB management, I am working with TB patients; I am the one who is initiating them on TB treatment.

I: Mhm.

P: When the sputum is collected- when the results came back positive. So, I am the one who is dealing with them mostly until they finish their treatment.

I: Okay, how is it to handle TB patients in the facility?

P: It was very difficult because most of the patients they don’t comply, they don’t come on their next appointment date but since they brought this program of ASCENT that is introduced by (organisation name), we have improved a lot.

I: Mhm.

P: Because most of our patients when we initiate them on TB, we are telling them about this ASCENT program when we are giving them the white box, that this box has a battery and an alarm. They put their medication inside the box. We also write down what time they are going to take their medication, so we agreed on the time. Then, we set the box to remind them on the time to take the medication and even on the next visit it’s helping us a lot because the box is set, and it will remind the patient a day before that tomorrow you are going to go to the clinic for your medication.

I: Mhm.

P: Yes.

I: So, how is the turnaround of patients, do they come nicely now?

P: Yes, we have improved a lot, we have improved a lot because before this pill box some patients wouldn’t come. They wouldn’t… we were having many of those lost to follow on TB program.

I: What has improved lost to follow?

P: It’s this pill box because when we are getting them (patients), we are telling them that if you didn’t take the medication the box will be telling us that you didn’t open the box, so when you didn’t open the box it shows on our tablet, it shows red. When you open the box, it shows us green, after a day if you didn’t take your medication an automatic message on your phone will come reminding you about taking your medication. On the second day if you didn’t also take your medication, it will-

I: Just continue.

P: Okay, the message will go automatically on the first day, if you didn’t respond again on the second day- you still didn’t open the box- your pill box, we call you. If we didn’t get you when we are calling you, we will send our CHW, community health worker to your home address because we already have your address to come- to do home visit.

I: Okay.

P: Yes.

I: Okay, so uhm you are telling me that you check patients as in you call and sometimes you do home visits. Uh… how is this whole work that you are doing how is it? Is it something that is doable or?

P: Yes, it is working for us uhh a lot because if the patient has got the home visit, you will find that maybe the patient was not at home that’s why he left the box, but he took the medication out of the box. Some of them have gone to work, some they got transfers, so we need all that information so that we can update.

I: Oh, okay so earlier on you were telling me that these boxes are improving the lost to follow.

P: Yes.

I: Can you please elaborate and tell me more about that, how do the smart pill boxes improve the lost to follow?

P: The smart box has improved a lot on us because the patient will tell us that I didn’t open the box because I was going to work, so we explain to him that if you are going to work you must take it, open the box, and take your medication with you so that the box can reflect that you’ve opened it. Before we were having the, we were having- we were had boxes, we were not aware if the patient is taking the medication or not taking the medication, but now we can see that they are complying. They are taking the medication.

I: Mhm.

P: Yes.

I: Oh okay, how are TB services delivered at your level with regards to digital adherence intervention. What exactly are you doing in the ASCENT program?

P: We monitor them on our boxes, and we also do home visit to those who are not complying. Most of them are complying, they are complying. It’s few who are not complying.

I: Yes.

P: And with this ASCENT, we have discharged many patients who have finished their medication, before we had this ASCENT program most of the patients were not finishing their medication. They just left without finishing their medication telling us that they are lazy to come to the clinic, sometimes they forgot to come to the clinic.

I: Mhm.

P: Yes.

I: What are their reasons for not coming to the clinic except lazy and forgetting, what other reasons?

P: Some of them knock off at work late, so now we have told them that if you are, you have knocked off at work late you must make a plan maybe the following day you ask your management, you tell them you are coming to collect your medication so they can release you early or else you start at the clinic in the morning when you are done at the clinic, you can go back to work.

I: Mhm.

P: Yes.

I: Okay. So, you told me that you call- you monitor patients, you call patients and then you also arrange uh for your home visit for those who are taking treatment. What else are you doing as a healthcare worker, as a nurse when it comes to the ASCENT program. What else are you responsible for?

P: I am responsible for managing my patients because if they are not managed, we are going to lose them.

I: Mhm.

P: Because before the program of ASCENT, we didn’t care about TB because of COVID-19. ASCENT made us to know that we are still having TB patients, we must also test the patients who came to our facility for TB because we have focused too much on COVID-19.

I: Mhm.

P: Forgetting that the TB still exist.

I: Mhm.

P: Yes.

I: Okay, alright. So, you told me earlier on that you monitor patients, where do you monitor patients?

P: The patients are monitored on the, on our tablet. I am having my colleague who is xxx [intern name] , he is also helping me. We are help each other every day when we came in. If I am busy with something, he will, he will tell me that this patient has not yet taken their medication and the automatic telephone message has been sent to the patient, but the patient didn’t respond, so it’s where we start calling them.

I: Okay, do you also call the patient?

P: Yes.

I: Okay, is it easy for you to take the tablet and call the patients?

P: Sometimes we don’t use the tablet to call them, we use our facility phone.

I: Landline?

P: Yes.

I: Okay, alright. Uhm if you are to explain what is digital adherence technology or digital adherence intervention to another healthcare worker who knows nothing about this technology, what is it that you would tell them, you would tell them?

P: This is the technology that has brought to us an easy way to work with TB patients because this system when we are having TB patients it decreases a lot of paperwork. We do everything automatically on the tablet. You register, we’ve got the phone number of the patient, what time the patient will be taking the medication and it’s also easy to monitor if the patient has taken the medication or not because before this digital, we were having the files on the file it’s where you write only you cannot see if the patient does take the medication or not.

I: Okay, what else can you tell another new person who doesn’t know about ASCENT ? You are going to tell them that you know we monitor patient on the tablet, it helps us to see on time, what else are you going to tell somebody new you are telling somebody new about this program?

P: This program also helps the patient; it reminds them about taking the medication because the box is having an alarm when the patient has said I am going to take the medication at 8 o’clock. So, we set 8 o’clock even if the patient is busy at the time, the alarm will remind him or her to take the medication.

I: Alright. Okay please describe your role in the differentiated model of care intervention, what exactly are you doing in that differentiated of care?

P: In that differentiated care, it helps us a lot to have less lost to follow patients because we are doing telephonically to check the patient why they didn’t come to the facility, is it he is sick or what’s the problem. If the patient doesn’t answer our calls, we are also having our OTL (Outreach Team Leader) and CHW (Community Health Workers) who are doing the home visits to the patients.

I: Mhm.

P: But we are also having a challenge on that one because some patients don’t give us the correct home addresses.

I: Mhm.

P: Yes.

I: What are other challenges that you have, it’s the, it’s the address?-

P: It’s the address and the phone numbers, they change the numbers. Sometimes they give you the wrong numbers that why we do not get them when we are calling.

I: Mhm.

P: Yes.

I: Okay, so you are mentioning that there are community healthcare workers that you work with and the OTL’s?

P: Yes.

I: What are the OTL’s again?

P: It’s the sister who is managing the community service program.

I: Oh okay, so I want to know how do you share, how do you share the work together in this program, you are the one who is checking and then you liaise with the OTL’s, and the community healthcare worker just want to know how do you share the work together?

P: Okay, the OTL and the community healthcare workers are helping us a lot because they are the ones who goes to the community.

I: Mhm.

P: When we are explaining to them that I am having this patient he or she is not answering our calls and this is his address. Most of them, you will find that knowing their address saying, “no, this place, we used to check some of the patient in this place.” So, it’s easy for them to fast track those patients to come back to the facility.

I: Mhm, okay. Alright so they are doing home visit and then you are informing them of the patients that need to be checked?

P: Yes.

I: That’s how you share?

P: Yes.

I: The differentiated care activities?

P: Yes.

I: You are the one who sees, who monitors and call?

P: Yes.

I: And then inform them if you are not getting the patient?

P: Yes, and they are helping us a lot because most of the patients if they do, they’ve done home visit, a patient will come same day or the following day.

I: Oh okay. So, what is their reason for not coming to the clinic the patients when they are coming now?

P: Some of them would say they knock off late at work. Some of them would say they were busy taking care of the children waiting for someone to come and help them with their children before they came to the clinic.

I: Mhm.

P: The OTL helps us with those ones who are having small kids, the ones who are having no one to look after their kids because she’s using her own transport to go and fetch the person and come with the person and we assist the patient, and she takes the patient back home.

I: Okay, alright so it’s helpful?

P: Yes, very.

I: Okay so when you first heard about digital adherence technologies what were your expectations before the implementation of these boxes?

P: When I first heard about it, I was not sure if it was going to work for us or if people are going to comply with it. I just… I was just waiting for it to come so that we can do it and see if it’s working for us.

I: Mhm.

P: Yes, but now I have seen it, it’s working, and it is reducing paperwork because on paperwork we have to write everything, and you cannot see if the patient has come or has not yet came. So, it’s easy with this one.

I: Okay, so why were you worried if people are going to comply or if it going to work. What made you to think those things?

P: It’s because we were having many lost to follow TB patients.

I: Mhm.

P: Yes.

I: So, what were you thinking around the lost to follow and the box?

P: I was thinking maybe they are going to be lazy to take the box, but most of them- the box helped us a lot.

I: Okay so your expectations changed?

P: Yes, they have changed a lot because the box is working. Even some of the them when we are discharging them, they will say, “can I keep the box for my other medication because it has helped me a lot with taking all my medication.”

I: Mhm, okay that’s nice. Which medication are they talking about?

P: Some are talking about their ARV’s and some about their high blood medication.

I: Okay, so they are keeping the other medication?

P: Yes. When they are taking the- all the medication at once. If they are taking TB at eight, they know that I took a TB pill at eight and then after 30 minutes or an hour later, I must take the other one.

I: Okay, so what else do they tell you about the box that is interesting?

P: It’s the box reminding them of taking their medication even their next appointments, it’s reminding them. So, now they know that I am going to go to the facility to collect my medication, it’s my time to collect my medication.

I: Okay. Alright it’s good to hear all of those things. Uh can you please describe the training and the resources that staff received on the, on the deliverance of the digital adherence technologies including differentiated care, how was the training that you received?

P: The training was very good because some of the things they were telling us it’s the things that we had forgotten about and focusing mostly on COVID-19, forgetting that there are still patients who are infected by TB. So, the training has made our minds to focus and knowing that there are still patients who are still infected by TB, and they need to be taken care of also because the COVID-19 came, and we forgot about the other disease.

I: Okay, do you still remember activities that were done at training? When we were training you, what did we do?

P: Okay some of them were introduced to the box, we were shown how to put the batteries, how to register the patient on the tablet and also when the patient has taken the medication the tablet will show you a green bar. If the patient has not yet taken the medication it will show you red and we were also told that an automatic message will go to the patient if the patient didn’t take the message because when the patient were registered on the tablet all their information were taken, all their numbers were taken.

I: Mhm.

P: Yes.

I: Mhm. Okay what else do you remember, uh the activities that we did?

P: There were many some of them I forgot.

I: Mhm [Laughs]. So how were you trained uh who trained you, was it here at the clinic or you were trained somewhere else?

P: We were trained somewhere else at hunters rest.

I: A hotel?

P: Yes. When we got there, we were welcomed by the ladies, those ladies were lovely, and they were showing us love. They didn’t want us to feel lonely because we didn’t know them it was the first time, we met so even during the course of the day we didn’t starve they were giving us refreshments; the training was the best.

I: Okay, that’s good to hear. So, what was your opinion about the training and the resources? What is your opinion about the training and resources received during training?

P: Everything was done nice and well and they made us to know that there are other companies who still support as DOH. (organisation name) has done a great job on us.

I: Okay, alright. What suggestions do you have uh in terms of training in terms of improving training?

P: I think for the training they can maybe in 3 months or in 6 months we must also go to training so that we can refresh if there are new things to be implemented, they can give us the information.

I: Who must do these uh refresher training?

P: The TB management including (organisation name) and DOH (Department of Health).

I: Mhm.

P: Yes.

I: Oh, how do you feel about the duration of the training?

P: It was not that much long but we’ve learned a lot on it.

I: Okay, are you happy with the duration or do you suggest something about the duration of the training?

P: Yes, I am happy because it was almost 8 hours.

I: Mhm.

P: Yes, but if there are many things it cannot be, they cannot finish by 8 hours maybe they can add 2 days or 3 days.

I: Okay, alright. From your perspective as a healthcare worker, can you please describe the benefits of differentiated care model and using this digital adherence technologies in the facility? What are the benefits of implementing these technologies?

P: It has benefited our facilities a lot because most of the TB patients with this pill box are explaining to the others and giving hope to those who are newly diagnosed. This box has helped them by reminding them to take medication and their visits to clinic. Some forgot to take the medication so the support they got from the facility like the automatic message they receive and home visits helped them a lot until they completed their treatment.

I: How is the implementation of this technology in this facility benefited you as healthcare workers?

P: It benefits us a lot because we are having many challenges in our facility there’s shortage of staff sometimes you would forget that the person didn’t come for collection of medication but since implementation of these tablets usually we log on the tablet in the morning and check the green bars. If we see that some have not taken medication for more than a day and they didn’t respond to the automatic message we call them if we don't find them telephonically we ask our OTL (Outreach Team Leader) and CHW (Community Health Worker) to do home visit. So now TB has improved a lot.

I: Okay, alright. What are the, how has it improved your relationship with the patient?

P: It has improved my relationship with the patient a lot because most of the patients are complying, yes. Even when they came to our facility, they came with a smile knowing that we didn’t just give them treatment and forgot about them. We check them on our tablet, we care about them, home visits are done for those who are not complying.

I: Okay, alright. Can you please describe now the challenges of implementing the differentiated care and smart pill box technologies, what are the challenges?

P: Some of the pill boxes were having a problem with the battery and some had their alarm ringing now and again but we sorted that one. The challenge with home visit is that some of the patients were giving us a wrong address even their phone numbers are wrong, so when we are trying to call the patient, the person would answer saying it’s a wrong number.

P: Yes. Also most of the people here came for work, some are working in the mines, some are working on  contracts and are always are relocating.

I: Oh, so people would just go?

P: Yes, they go without telling us where are they going when home visit is done, they (Community Health Workers) were told that this person was working on this contract company they moved to Mpumalanga or moved to Limpopo.

I: Oh, so would they, would they go with the box or when return the box?

P: Some of them we were having those.

I: Are there a lot that would go with the box to those that return the box?

P: No, most of them when they are done with the treatment, they bring the box back.

I: Oh okay. Oh, let’s talk about stigma and the use of the smart pill box. What is your opinion and your experiences about providing the box and the stigma?

P: Those TB patients are free because they say the box reminds them to take medication and remember the visit dates. Even those who are not having the boxes when they saw them (TB patients) having the boxes and asked them questions they would explain to them why they are having these boxes. Others wish the boxes can be implemented to all chronic medication.

I: Okay, alright. So, you were telling me other people that are making it difficult for you are people that are leaving because they were here because of their jobs.

P: Yes.

I: What is the other group that is difficult to work with using the smart pill box to give the smart pill box?

P: It’s the language, their real language because some of them are Shona or Ndao speakers. It’s difficult to explain them how to use the box and when he can’t understand maybe for first time, he’ll come with somebody who is going to translate.

I: Mhm.

P: But when he came alone, it’s difficult for them to understand what you are saying.

I: Okay.

P: Yes.

I: So, in terms of the groups now, I want to have an idea of what are other people that are difficult to give the box and monitor. You told me that it’s those who just go because uh they were here because of the job now they are going because of the job-

P: Yes-

I: Now we don’t know what is happening with them, now I want to know what other groups that are difficult to work with in terms of using the box?

P: The other ones, they are the ones that were saying I am working at night, I am working 6 to 6, so I don’t have time for this box, I even took my medication late at night when I came. I am tired, I eat and then take my medication. I don’t have time for the box. Some are choosing the box.

I: Mhm.

P: Yes, but they are few who do this and the other one who said he is working as a car guard, he said he can’t take the box because he is moving a lot because of his job.

I: So, have you, have you given homeless and people that are on drugs the pill box?

P: No we haven’t yet had that type of TB patients

I: Oh okay. From your perspective as a healthcare worker how can TB treatment be improved by using differentiated care and and this uh medical device technologies?

P: Yes, it can be improved because in this short time we have initiated them on this ASCENT box and this home visit TB treatment improved a lot, I think this can carry on because most of the people are complying and we are not having many patients lost to follow it will be one or two but before this implementation we were having more than 10 to 15.

I: Lost to follow?

P: Lost to follow.

I: Okay. Any difference in terms of treatment before the intervention uh and after the intervention in this facility?

P: Yes, now we have managed our patients and we have discharged them before we had this implementation, some of the patients were not finishing the treatment. They would take treatment one month or two months and came maybe after 8 months or 9 months having TB again.

I: Mhm, but now it’s better?

P: Now it’s much, much better because we have managed to discharge more than 6 patients and now, even this month July we have discharged about 5. Next month we are still going to discharge patients who are complying.

I: Okay, alright. Please elaborate on the positive changes that this smart pill box brought in your facility. What are the positive changes that you can think of?

P: It has made uh positive change because most of our patients know when to return even their dates are set on their boxes, they don’t forget their dates. They are complying.

I: Mhm.

P: Yes.

I: How can we sustain these positive changes?

P: I think we can give also give other chronic medications the box not only TB patient so that we the same results as TB.

I: Alright, what are the negative experiences that the use of smart pill boxes and differentiated care brought in the facility?

P: So far, we are having uh less, few.

I: Mhm. What are those few?

P: Because there was another guy who took a box and then we visited him at home- visit was done. The address was the wrong address, but he came on the other day saying I am bringing your box because I no longer need it but by the time he was given information and the pill box was explained to him he said he understood everything.

I: Mhm, what was the reason of the patient to refuse the box?

P: The patient is having an alcoholic problem; he is drinking too much.

I: Mhm.

P: Yes.

I: So, what was the problem of using the box?

P: He said the box is making noise.

I: And did he give the box back?

P: Yes, he brought the box back.

I: Oh okay, did he… did he finish the treatment?

P: Yes, he did because of the help that we were getting from OTL because the OTL do home visit and took his medication.

I: Okay.

P: Yes.

I: So how can we improve this negative uh experiences?

P: There are few people who are having this negative, most of the people are happy with the box.

I: Mhm.

P: I think when we are telling the patient about the box, we must also tell them if you don’t want it, you must not take it.

I: Oh okay.

P: Yes.

I: Oh alright. And how… and uh… how can we, please describe to us what system level structure needs to be improved in order for us to integrate differentiated care and this technologies into the TB system programs?

P: Another thing that can be improved in this differentiated care, most of our patients are complaining about poverty. I think if we can give them the box and have some, like sort of feeding scheme. So that maybe during the week they can come maybe 3 times a day to fetch some food from this feeding scheme program because some would say, “I didn’t drink it yesterday because I was having no food.”

I: Mhm.

P: Yes, we also having those challenges because of the COVID-19, most of their family members have lost their jobs.

I: Oh okay.

P: Yes.

I: Because of COVID-19?

P: Yes.

I: Alright. What can we do to integrate the ASCENT program with the existing program that we have here at the clinic? How can integrate the two into one?

P: I think it will take time to make them one because we are… as here squatter camp we will always have challenges because it’s a squatter camp. People come and go.

I: Mhm.

P: Yes. They come and go; they are not permanent here.

I: Mhm.

P: Yes.

I: Okay, so it’s difficult to-

P: Yes, it’s difficult.

I: Okay. Uhm can you please describe to us what systems are in place that could monitor the program, the ASCENT program? How would you monitor the ASCENT program?

P: We monitor our own tablets.

I: Mhm.

P: We are given tablets, we are using tablets that way to register the patient.

I: Mhm.

P: Yes.

I: Like I want to know how do you monitor the program of itself now, the smart pill box program if it’s working or not how do you check?

P: Okay, I am having my colleague xxx [intern name], he is the one mostly doing it.

I: Okay.

P: Yes.

I: Alright, and without the (organisation name) support in the future, if we were to escalate to other facilities and uhm there’s no Arum support how, how… can you continue to work to implement these technologies in the facilities without the support from (organisation name).

P: We can continue to work without (organisation name) but we will be having challenges because those (organisation name) people are helping us. (organisation name) colleagues are helping us a lot because in our facility there is always shortage of staff. So, if there is a shortage of staff, I don’t focus on one program. I work there, go here, go here, go here. So, when are having them, they are assisting us a lot. I: Okay.

P: Yes.

I: Alright what else will, that will happen when (organisation name) support is not here, and you are implementing the smart pill box alone?

P: I think it will decrease management. It will go back to that part where TB patients weren’t taken care of as I have said that there is a shortage of staff at our facility, we don’t focus on one program. If someone is sick, maybe someone is on leave there is still a shortage of staff like now on our vital station we are only having one EA, so there is a huge gap. The other one went on maternity leave, the other one is on leave, and the other one is off. We are only having one, shortage of staff is our biggest challenge.

I: Okay.

P: Yes.

I: So, there is support from (organisation name) and we are also here at the clinic we are implementing this smart pill box who is going to do what in this ASCENT program.

P: We are helping each other. Yes, because when we came in the morning my other colleague from (organisation name), he goes first to the tablet and he would tell me this patient isn’t taking medication and then he didn’t respond on the message, so we call him. Okay, we call him, if he didn’t respond also on telephonically call we ask our OTL to do home visit when we are two. Having the support from our colleagues from (organisation name) is better because when I'm busy with something, he can see that the patients are not attended well so there is this problem we have to attend. When you are alone, I'm alone. I cannot do it alone.

I: Okay.

P:Yes.

I: Okay, so how are you going to divide the work now that there is a registration and monitoring patients. I just want to have an idea how, how are we going to make sure that we collaborate nicely, and we share, we share the responsibility. Who's going to do what?

P: Mostly when we are having a new initiation- a patient. He's registering the patient and I am doing the paperwork. Sometimes he can come with the tablet and then we are assisting each other so that we can finish fast.

I: Oh, okay.

P: Yes.

I: All right. That's very good to hear sister, and we are almost at the end of the interview. Before we can end the interview, I, I want you to tell me about like, in your last remarks, what is it that you can tell me that we did not talk about? About the whole program, like the smart pill box, the differentiated care, and anything that you wanted to tell me that is important that we did not cover.

P: I think we have covered everything about our pill box, our differentiate care of a patient. We've covered all of them.

I: Okay. All right. And then this is the end of this interview and thank you so much for your time. End time is 11:09. Thank you.
