# Supplementary material for: Acceptability of Digital Adherence Technologies to support people with drug-susceptible TB in South Africa
Source: PLoS One. 2025 Sep 24;20(9):e0332103. doi: 10.1371/journal.pone.0332103 (PMC12459780; doi:10.1371/journal.pone.0332103)
Supplement: S4 File — (ZIP) [file pone.0332103.s004.zip › S4 Transcripts/HCWs and Stakeholders/IDI 24-HCW.docx]

**TRANSCRIPTION NOTATIONS**

| **Label Key** | **Meaning** |
| --- | --- |
| **I** | Start of each new utterance by the Interviewer |
| **P** | Start of each new utterance by the Participant |
| **N** | Note taker |
| **{ }** | Indicates that details were changed or pseudonyms were used to anonymise data |
| **( )** | Indicates the description provided to anonymise data |
| **XXX** | Words were omitted to anonymise data |
| **-** | Breaking into a sentence by the next speaker |
| **…** | Pause or drawn out words |
| **[ ]** | Indicates noise made, e.g. [laugh], [sigh], [pause] |
| ? | Beginning of utterance by unidentified speaker or questionable text |
| **[inaudible segment]** | Unclear section of the recording |

I: Thank you for agreeing to talk to us today, uhh can you please allow us to audio record this interview?
P: Yes, I do allow. For… the recording of the interview.

I: Okay, thank you so much. The PID number is xxxx, date of the interview is xxxx (interview date), location xxx [clinic name]. Type of the interview: healthcare workers interview, time of the interview: it’s 11:04. Facilitator: xxx [interviewer’s name]. Uhmm, tell me sister what is your title of for your current, of your current position?

P: I am working as a nurse.

I: Mhm.

P: But uh… specifically, I am helping the TB patients, that’s the program that I have been holding at xxx [clinic name].

I: Okay, what are you... what exactly are you doing at the program as you say you are helping the TB program, what are your focus?

P: More focusing on uh, making sure that patient attends their appointment correctly and then giving off medication. Making sure that they adhere, and they take their treatment well, then… yeah.

I: Okay, when it comes to patient care and counselling, what are your roles and responsibilities?

P: Uhh… my role when we start the patient with the TB treatment, I would always emphasize the… the importance adherence, taking of the treatment and then, uh explaining also to the patient the importance of taking the treatment… yeah.

I: Okay, so in relation to uhm… treatment adherence, emphasizing uh, on treatment adherence with your patients, how has it been your experience with doing that with your patients?

P: So, far it’s uhh… it’s well because I have- I can say I have a strategy of making them adhere to their treatment well. Normally, I say they must come straight to me, I keep their file and then they normally don’t stay on the line. So, that’s how I make them to make sure that they come to the clinic for the collection of treatment.

I: Mmm. What challenges do you have with patients and treatment adherence like what are the challenges that you experience sometimes with adherence?

P: Uhmm… the challenge that I always experience it’s that one most of the time patient will say uhm, I forgot the return date and then some of them will not come, and some- I will struggle to get them, but it normally doesn’t take a long time before I get them to come to the clinic.

I: Mmm. Well what strategies do you do to get them as you are saying you don’t take time?
P: I would ehh, call them.

I: Mmm.

P: And then if maybe let’s say I don’t get over the phone, because normally I don’t take one cell phone number because I know that sometimes you will never get the patient, so I always make sure that patient leaves another phone number of the next of kin and if maybe let’s say I don’t get the patient over the phone. Then we have a team of uh, people who will go to those patients addresses, at home and then to find out why did the patient not come to the clinic.

I: Mmm. What are the reasons of them not coming to the clinic when you go there?
P: They would normally say they forgot the date, that’s uh… most of the time that’s the reason that I would find. Some of them will be ill and unable to come to the clinic, but I normally advise them to send a family member to come and collect on behalf of them.

I: Okay, is it working… the sending, to ask them to send family members?

P: Yeah, it is, it is working.

I: Mmm.

P: It is working.

I: Then, how do you follow-up to check if your patient is getting better, do they come later or after?

P: They do, they do come later if the patient is feeling better.

I: Okay, okay what are other strategies that are helping you to build towards adherence amongst the patients, besides calling or you going there, what else are you putting in place to make sure that you are improving adherence in the clinic?

P: I have uh… I have seen before that I have worked with uh, the TB treatment patients, before they were struggling to get the files. A patient would stay for a long time maybe searching for the file, so I came up with a strategy whereby I keep their files, so normally the patient will know that my file is at the TB room, yes so that’s how I came up with a strategy of them not struggling to be assisted.

I: Oh, so you keep the files?

P: Yes, I am keeping the files, and then they normally don’t queue.

I: Okay, alright. So, in relation to the digital adherence uh, intervention uh, the ASCENT project that is implanted here at the clinic, what exactly are you doing within the project in relation to TB management?

P: Okay, I am… helping to enrol and then beside enrolling the patient, I would explain to the patient how does uh, the system work, how does this technology work and yes.

I: Mmm, what do you tell the patient when you are telling them how does it work?

P: Uhm… normally I say to that patient that you do know it’s not a must to take this thing, but I would advise the patient that uh, this is how this is going to help you and then would sign the consent form depending on if the patient do need this uh, technology or not.

I: Mmm.

P: Yes.

I: So, when you tell them how it works, what exactly do you tell them what are those things that you tell them?

P: Uhm, number 1 the system is working like this you choose the time whereby you are going to take your treatment and then when that time comes for you to take the treatment the box will alarm you in order to remind you that this is the time for you to take the treatment and then we do set an appointment date a day before you come for the refill. Then, the box will also remind you of tomorrow as the day to refill. Uhh… yeah and then basically I will tell them that the battery, if the battery is flat and then this is how it’s going to be, the light will go red. Then, yeah.

I: Okay, and what do you say to them if the light goes red, what are they supposed to do?

P: They must come back to the clinic so that uh, we charge the box.

I: Okay, in relation to differentiated care, what do you tell them about it, anything that you mention to them in relation to differentiated care?

P: Uhm… okay, on that note normally that one there will be a team that goes to the patient and then making sure that the patient takes the treatment and then by the time the… the… the box maybe the battery is flat and then that team will come with the battery- the box so that we can charge it in the clinic.

I: Okay, alright. So, if you have to explain- okay, so you have been telling me that you are able to register the patient, teach them how the box works uh, can you please tell me in terms of your experience how do you find that- is it easy for the healthcare worker to do that, to register the patient and explain how it’s supposed to work and basically monitor the patient and the device. Is it easy for the healthcare worker to do all of that?

P: Yeah, it is, I would say. This uhm… this, I don’t see anything difficult in that uhh, procedure.

I: Mmm.

P: Because it also helps the… the… the care worker.

I: How is it helping the healthcare worker?

P: On the basic of…of adherence on the patient because at least we can know if the patient is not taking the treatment, then we will make the necessary follow-ups to check why did the patient not take the treatment.

I: Have you done that? Like to see on the platform and… have you done that?

P: Yeah, I did-

I: How did you do it?

P: We uhm… I normally call them and then tell the patient that I did see that you didn’t take your treatment this morning. So, the patient will say, “no I did take the treatment, it’s just that I took the treatment before the time because I was going somewhere.” So, I do make those uh, follow-ups.

I: Mhm, okay. So ,how do you see this whole process on the side of the patient now? Is it benefiting patients or what is your opinion?

P: Uhm… this one won’t be my opinion but, on my opinion, I would say they, it benefits the patient but normally I would… normally I talk with my patient and then ask them this thing- how does it help you and then is it helping you or do you see it unnecessary. So, most of them would tell me uh, this device is helping them a lot because it can remind them that this is the time for you to take the medication, it can remind them that tomorrow you must go for the refill at the clinic. Some of them do forget to take their treatment, so the alarm- it’s difficult for them to ignore the alarm.

I: Mmm.

P: Yes.

I: Okay, so that in return it helps to improve the-

P: The adherence, the treatment taking of the patient.

I: Okay.

P: Yes.

I: Okay, that is good to hear that, and if you have to explain what is digital adherence to another healthcare worker who knows nothing about that, what would you tell them?

P: Are you asking if… maybe if I say to them this is how we use it or finding out if it’s important to use or not?

I: Anything that you would tell the uh, the healthcare worker who knows nothing, if you were to explain that okay this is, how are you going to tell them if you are to teach someone new who doesn’t know about it?

P: Okay, I would start uhh… by showing that healthcare worker how does the device work and then also how do we enrol the patients and then also the importance of using the device and tell the… tell him/ her also that we need uh, the consent from the patient before you enrol the patient on the device.

I: Mmm.

P: Yes.

I: Okay, what else that is important that you can tell the healthcare worker about the smart pill box? Something that the healthcare worker needs to know especially when they tell the patient about the box.

P: Uhm, it’s not a must.

I: Mmm.

P: For the patient to use the box, it comes from the patient. If the patient don’t want to use the box, then you are not supposed to force the patient to use the box.

I: Mmm.

P: As there is a consent form.

I: What else can you tell the other healthcare worker about the box now, the way the box works what are the important things to tell the healthcare worker?

P: Okay, if uhm… the patient did not open the box uhm, to take the medication. Then because we enrolled the patient and the… the… the system will tell us that the patient did not take the medication and the SMS will be sent to that patient automatically if the patient didn’t take the medication as their reminder and still if there is no action that the patient is taking. Then, we call the patient to make sure that the patient takes the treatment. If we don’t get the patient still, then we make the follow-up of home visit.

I: Thank you very much, then please describe your role with differentiated model of care which is follow-up’s taken when we see that the patient did not take the medication on the platform what do you do?

P: Uhm, on that point my role is to take the information of the patient, the cell phone number, the address, and everything because I am not the one who is doing the home visit, so we have uhm, a group of people who specifically do visit, to go and visit the patient and then they will come with the report back.

I: Okay, do they update you?

P: Yes, they do.

I: Okay, so what do you usually do after they have updated you?

P: Uhm… after the report and then normally sometimes they would say they didn’t get the patient from the address that was given maybe the address was wrong and then on that part we normally wait for the patient to come because there is no other way that we can find the patient.

I: Mmm.

P: But uh… since I have [inaudible segment] with the TB, normally I don’t have uhm the cases whereby the patient do not come to the clinic, they do come to the clinic.

I: Okay, so you are saying you work with the community healthcare workers?-

P: Yes.

I: To do the home visit?

P: Yes.

I: How do you share this work like between you guys like what do you do, what do they do?

P: I treat the patient if the patient is uhh, here in the clinic and then their work is to make sure that they find the patient and then bring the patient to the clinic.

I: Okay, do they win sometimes?

P: Yes. They do win, they do win.

I: They get them?

P: Yes.

I: Mmm, so when you talk to the patient?-

P: Apparently, I have this patient. So, this patient receives medication at home.

I: Mhm.

P: Yes, every morning.

I: The community healthcare workers?

P: Yes.

I: Everyday, they go to give the patient medication?

P: Yes, because the patient was not adhering well.

I: The patient was?

P: The device showed that the patient was adhering well because she was opening the box every morning as if she’s taking the treatment, but we heard from the family that actually she’s not taking the treatment. Uhm… they said it as it is that they are staying with the patient and then the patient will open the box as the box alarms, but she doesn’t take the treatment.

I: How did you find out from the family that the patient is not taking the treatment?

P: They came to the clinic to report.

I: Okay.

P: Yes.

I: Did they tell you that the patient is just opening the box?

P: Yeah.

I: Is not taking medication?

P: They did say.

I: How did they tell you, what did they say about that?

P: Uhm… they said it as it is that they are staying with the patient and then the patient will open the box as the box alarms, but she doesn’t take the treatment.

I: Mmm.

P: So ,the treatment will just stay like that.

I: Okay, so the family came to report?

P: Yes.

I: Were they here with the patient when they came to report?

P: No, apparently the patient was already weak, so they were unable to come to the clinic.

I: Who came, who came from the families? The mother, the father?

P: No, the sister, the sister.

I: The sister?

P: Mmm.

I: So, is it a guy or-

P: Lady, the sister-

I: The person that was taking the treatment-

P: No, it’s a lady.

I: Mhm. So, when you were asking the sister, what was the reason of this patient not to take the medication but just to open the box only and not actually take the medication when you were investigating from the family? What is the main reason for the patient not to take medication?

P: Umm-

I: What did you pick up-

P: Actually, I didn’t ask many questions from the lady, we just took an action to make sure that the patient takes medication.

I: Okay, so when you got to the patient like the action you are telling me about. You got to the patient and tried to investigate, what was her reason of just opening the box without taking medication? What is it that you picked up?

P: He didn’t say much. She didn’t say the reasons why she didn’t take medication even when we asked her.

I: Mmm, how is the situation now after you intervened.

P: Umm the situation is better because even the patient is able to come to the clinic now to take treatment.

I: Mmm-

P: mmm

I: Does she still have the box-

P: Yeah, she is still using the box.

I: Okay, is it helping from the-

P: Umm hearing from the family it is helpful-

I: Uh-mm what are they saying?

P: Umm they say it helpful because the patient will know the time of taking the treatment. So, they won’t just say take the treatment- at least it help them take the treatment same time every day as it supposed to be.

I: Mmm okay, so when you went-like I am trying to understand what made the patient not to take medication because she was just opening it without taking medication-

P: Mmm-

I: then you did this intervention. I just want to understand what is it that you did to change this person’s mind?

P: Uh-mm I did speak to the patient and told the patient the disadvantages of not taking medication because it obvious that it can lead to death if you don’t take medication. So, I think that’s the reason for her to change her mind and started taking treatment well.

I: So, you basically conducted adherence counselling?

P: Yes.

I: Okay and then you saw the change after?

P: Yes.

I: Okay, that’s good to hear that the patient is doing well now. Is the counselling ongoing now or you only did it once?

P: Now because she able to come to clinic-so everytime she comes to the clinic, we are able to continue with adherence counselling.

I: Uh-mm

P: Okay, is it helpful?

I: Yes, it helpful.

P: Mmm

I: It good to hear that. So, when you first heard about the digital adherence technology, what was your expectation before it was implemented? First time hearing about this technology, what is it that you were thinking about?

P: Umm to be honest, knowing the kind of people that we are helping in this community.

What were your expectations?

P: Um, to be honest, knowing the kind of people that we are helping in this community, I thought it would be difficult for them to use the pill box.

I: Mm-hmm.

P: But to my surprise, it was not that bad because most of them were happy to use the box.

I: Mm. Okay. So, you were thinking that it was going to be difficult?

P: Yeah. I was thinking they will refuse to use the box.

I:Mm.

P: Maybe thinking that they will be exposed, that they are taking the TB treatment and so on. But I saw a different thing. Like it was not difficult for them to agree to use the box.

I: Okay, so what- why, why were you thinking that it might be difficult for them to use the box?

P: Um, I'm working with those people every day.

I: Mm-hmm.

P: And then if you work with people every day, you can see how difficult they are in some of the things, because some can still say no, people are going to see me, that obviously I'm taking the TB treatment.

I: Mm-hmm ,okay.

P: Because of the box. Yes.

I: Okay. How is the stigma here in relation to that box? Are you seeing people concerned now? What is this box?

P:No, I never hear anyone complaining about it.

I: Oh, okay.

P:Yes.

I: Alright. So, are your expectations changed now?

P: Yeah, they did change how, um, I can say most of them, they agreed to use the pill box.

I: Mm-hmm

P: Yes.

I: Okay.

P: And, uh, there was not the thing that I expected.

I: Mm-hmm. You were thinking that they will refused to take the box.

P: Mm-hmm.

I: How do you feel that most of them took the box?

P: Um, I feel so happy because it's really helping them, and I am relieved that at least they can relate to the technology.

I: Mm-hmm.

P: Yeah.

I: Okay. That's very good to hear. Can you please describe the training and the resources that staff received on delivery of the digital adherence technology, including differentiated care? What kind of training did you receive? Can you please describe it?

P: Okay. When the devices were delivered- the training-

I: Were you trained for the ASCENT project? How was the training? What kind of training did you receive?

P: Okay. The kind of training that I received, uh, they showed us how to use the pill box, and then they also showed us, um, how do you explain to the patient on how to use the box and then the consent form about the box and then that we enrol only the people over the age of 18.

I: Mm-hmm.

P: Yes.

I: Okay.

P: That was the training that I received.

I: Okay. So how were you trained and who trained you when you trained? How were you trained? If you were to give us feedback about the training that you received, about the ASCENT and those technologies, how was it for you?

P: Okay. No, the training was fine. Nothing was difficult to understand. Um on the note of who trained us. I only remember you; I don't remember others who were there.

I: Okay. Do you think it was comprehensive, the training that you received, was it comprehensive?

P: Yeah, it was.

I: Why are you saying that?

P: Um, It was perfect. They were giving the information in a way that we understand how to use this.

I: Mm-hmm.

P: Yes.

I: Okay. Was it easy for you to understand what are you going to do when you're at the clinic, when you have to implement it?

P: Yes, it was, mm, it was.

I: What challenges did you experience when you were implementing the technology for the first time?

P: I would say there was no challenges. Everything was working well. The only challenge that we had was that patients were complaining that they would receive the SMSs even though they took the medication. Yeah. That was the only thing that was a challenge.

I: Mm-hmm. Okay. Do you have suggestions to improve training? How can we improve the training that you received?

P: Um, no, because I think the training went well.

I: Mm-hmm.

P: So, I have no complaint about the training.

I: Okay.

P: For me.

I: From your perspective as a healthcare worker, can you describe the benefits of the differentiated model of care and the use of a medication device technology? Can you describe the benefits?

P: Okay.

I: Of the differentiated care in the smart pill box itself.

P: Okay. On the side of the benefits, I would say since we started using the pillbox , um, the patient have been adhering very well, and then they've been taking their treatment very well. And then, yeah, because they do say that it helps them a lot because it reminds them when to take the treatment, when to come for the follow up at the clinic.

I: Mm-hmm. In terms of the relationship with the patient, has it improved your relationship with your patients?

P: Um, yeah, the relationship with the patient. Uh, yeah, it's fine.

I: How has it improved your relationship with the patients?

P: Um, what can I say?

I: Think of a patient who has been- whom you have been supporting using the DAT and think about stigma related issues, like how has it improved your relationship with your patients?

P: Okay. The relationship, it's been well, and then it's fine. No patient ever complained about, um, the device and then they are all happy and then they are all thankful to use this technology device.

I: Mm-hmm. Okay. Why are you saying patients are happy? Anything that they've said to you that makes you think that they're happy to use the device?

P: I mean, I think if they were not happy to use the device, they would've returned the device. So, they always say what this device is helping them.

I: Okay.

P: And remember from the beginning we told them that it's not a must to take the device. So, I would say if they were not happy about the device and then I think they would return the device and say, we don't want this device.

I: Mm-hmm.

P: Mm-hmm.

I: How is the level of people returning the device because they don't like it.

P: It's very less. I only remember two patients who returned the box.

I: Okay. Do you remember the reasons of returning the bo the box?

P: Um, the reasons was stigma that, uh, at home, uh, people can see that I'm taking the treatment, uh, so forth. There was their reason.

I: Okay

P: Mm-hmm. So at least if there's no box, then nobody will see that I'm taking the treatment.

I: Mm. Oh, any different reason of returning the box?

P: The other one, who, what is it that they said? Um, there's no other reason. It always, the problem of them not wanting people to know that they are taking the treatment because this box will show people that they are taking the treatment.

I: Okay. So, what would you suggest in that case of somebody who is returning the box because of the stigma? In terms of supporting the need, what can we do?

P: I think on that part is just to tell the patient that there's um, no problem in the family knowing that you are taking the TB treatment since the treatment, the TB is transmissible. So, I think it's wise for the family to know that you are taking the TB treatment. So, it's just maybe patient, I don't know how do they take it, but we normally tell them that the family, they also have to know that you are taking the TB treatment so that they can come for screening to make sure that they do not have TB.

I: Okay. Did you try that with this two patient to tell them that there's actually no big problem for the family to know. They need to know so that they can even give them support. Right.

P: I did, I did tell them.

I: What did they see? How did they see it? After talking to them in those lines, I um, they still insist to return the boxes for all.

P: Yes.

I: All right. Okay. Uh, can you please describe challenges of differentiated model of care and the use of, uh, medication device technology? What are the challenges? I hear the benefits, but now I want to know about the challenges and what are the challenges of using the box and as well as the differentiated of care.

P: Um, the only challenge of patient using this box, like I said, it's um, the family knowing that they are on TB treatment. And then, uh, the other challenge was that one of receiving the SMS, even though they are taking the treatment mm-hmm. Because, uh, um, they were complaining that it's more like we are not taking the treatment because you guys always remind us to take the treatment even though we have taken the treatment.

I: Mm-hmm.

P:Yes. So that was the only challenge? Yes.

I:Okay. On the side of a healthcare worker, what is the challenge of implementing the strategy? What is the challenge of using the technologies on the side of a healthcare worker now?

P: Um, there was no challenge.

I:There's no challenge at all in your side?

P:Yes.

I:Okay. What are the benefits for you at the clinic, how are you benefiting from supporting patients through technologies?

P: Um, I benefit because the patient come to the clinic in time because at least the device can remind them that tomorrow is your appointment date, even though. Yeah, because there is not enough time for them, they normally forgot. So, at least the technology- this technology is there to remind them that tomorrow you must go for the refill.

I:Yeah.

P: Yes.

I:Mm-hmm. How else has it benefited you as a healthcare worker?

P: I can see if the patient didn't take the medication and then at least there's a way of making a follow up that the patient didn't take the medication as before we were just issuing the medication and then the patient- whether the patient did take the medication or did not take the medication, we didn't know but today, at least we know if the patient has missed the dose, that a patient has missed the dose.

I: Mm-hmm. How does that make you feel? Were you're able to monitor immediately?

P: I can say it makes me feel happy because it benefits the patient and then at least I can see that the patient is taking the treatment.

I:Mm-hmm. Okay. From your perspective as a healthcare worker, can TB treatment be improved using differentiated model of care and the medication device technology?

P: Yes, it can.

I: How?

P: Um, because already now, by using this, uh, technology, we've shown that it helped the patients.

I: Mm-hmm.

P: Yes.

I: Okay. In terms of the workload, is it improving your workload as a healthcare worker?

P: Yes, it does.

I: How?

P: Because, um, normally we will go through the- it makes, uh, the work easy because of, um, we can see if the patient didn't take the treatment, then we can make the follow up for the patient. And then we can also see when there is, if it is the time for the patient to come to the clinic, then if you missed the date and then we can call the patient to come to the clinic.

I: Mm-hmm. So how is it decreasing your workload?

P: Um, I don't have to go through file by file to see if this patient did come or if this patient didn't come for the appointment.

I: How do you check?

P: You don't have to go to the file, right?

I:Yes.

P: When we enrol the patient, there's um -we make, uh, a reminder that the patient must come for the refill?

I: Mm-hmm.

P:Yes.

I: Okay.

P: So, if it's time for the refill, then we can see that the patient must come back for the refill. I:Okay. Please elaborate on the positive changes of the differentiated model of care in the use of medication device. What are the positive changes that the differentiator of care in the device itself brought to the patient?

P:It.Okay. Um, like I said, it helps the patient to take the treatment on time. It also helps the patient to know when to come to the clinic. Yes.

I: How can these positive changes be sustained?

P: Um, I think by continuing to use the digital technology.

I:Okay. All right. Please elaborate on the negative changes that the differentiate of care and the Smart pill box brought to the lives of the patients.

P: Um, so far, I don't see any negative changes about, um, the digital technology?

I: Mm-hmm.

P: Yes.

I: Okay. How do you think the negative changes could be addressed, or you said you don't use, see any negative challenges. Please describe to us what system level structures need to be improved in order to integrate differentiated model of care and medication device technology into the existing TB program.

P: Can you please repeat?

I: Please describe to us what system level of structures need to be improved in order for us to integrate the DAT program into the existing TB program? What is it that we need to do to integrate the tool?

P: Okay. I think the working system. That is working now is fine and helping a lot.

I: So, what can be done to integrate the DAT and the current system, do you feel like the two can be integrated?

P: Yeah.

I: Um, if we had to take the system, the DAT system that we're using now and integrate it with the current system, what is it that we need to do?

P: The current system? The DAT one?

I: Yes. And together with the one that you are using now, what system are you using now for TB management? What have you been using before the DAT. What were you using?

I: Um, no, we were just telling the patients to come and then give the treatment on the treatment day and then write on the appointment book when will the patient come for the follow up.

I : Okay.

P: So, it was like that. The manual one. The manual one is without the DAT.

I: Oh okay. So, how can the DAT improve the manual one? What are the things that you can see already that with this DAT can be improved. The manual one can be improved this way.

P: Um, already DAT is , uh, it is helping us a lot.

I: Mm-hmm.

P:Um, it's helping us a lot. Um, before it was not like now. Yes.

I: Mm-hmm. What was happening before. What is happening now?

P: Now at least patient do come in time for their appointment.

I: Mm-hmm.

P: So, before we were struggling, the patients were not coming. Uh, the appointment date is due.

I: Mm-hmm.

P:Yes.

I: Okay. So, now they're coming.

P: Yes. They're coming.

I: Okay. So, with uh Tier.net system, how can we, uh, how can we integrate the tool, DAT with the tier.net system?

P: Now, I do know the Tier.net system. Tier net system.

I: Oh, okay. So, uh, how do you capture your patient?

P: Do what?

I: What are you using to capture your patient?

P: Um, after seeing the patient? Yeah. I take the files to data room and then data captures are the ones who captures the data.

I: Okay. So, uh, do you capture, do you have a document where you capture, uh, the number of devices that you gave to the patient? That I gave this patient this device. The challenges that maybe the community healthcare workers are telling you after they did a home visit. Do you somewhere where you capture all those things?

P: No, I don't have where I record, uh the part of the health care workers.

I: Mm-hmm. The report and everything of the care workers you don't have, where do you capture challenges and issues like the report back from the home visit?

P: I normally write on the files of the patient or on the patient file.

I: Mm-hmm. Okay. Do you capture somehow the challenges, and other indicators of how you use DAT and differentiated care? Do you have system where you capture?

P: Yes.

I: Are you the one who's doing that?

P: Yeah, I'm the one writing the reports here.

I: Okay. So, you only write on the patients file.

P: Yes, oh, maybe I don't understand the question because I capture on the file.

I: Mm-hmm.

P: Yes, we do, I do take the patient's file to data captures uh for capturing. Yes.

I: You take the file to data captures.

P: Mm-hmm.

I: What is it that they're capturing?

P: Uh, on data. The visits of the patient.

I: Okay.

P: Yes, we capture the visits and when will be the next appointment date.

I: Okay.

P: So, that's, that's how we do it this clinic, we take the file to data capture.

I: What system are they using there?

P: It's Tier. Yeah, I think it's Tier. Yeah.

I: Okay.

P: And then I also capture, um, what the outcomes on the file, I mean, I only write.

I: What do you write?

P: The visit of the patient.

I: Okay.

P:Yes.

I:Okay. So, you write the visit of the patient in the file.

P: Mm-hmm.

I: You take the file to data capture?

P: Yes.

I: What else do you capture? What else do you write on the patient file besides the visits? The next visit?

P:The issuing of treatment.

I: Mm-hmm.

P: Yes.

I: Okay. Mm-hmm. All right. Thank you very much xxx [participant’s name], for all the information that you have given us in this interview. Uh, we are at the end of the interview, but before we can close, uh, please, can you give me few closing remarks? Anything that you want to say about the ASCENT project? Eh, this technology, differentiated of care, whatever that you want to reflect on before we close.

Okay. What I can say is that, um, the, this technology of DAT, has helped us a lot on reminding the patients about taking the treatment, and then I've talked with the patients, and most of them, they are happy to use the device because they say it makes their life easy. As it always remind them that they must take the treatment, that they must go to the clinic for the refill.

I: Okay. Thank you so much for making that last comment. We appreciate your time and thank you very much. And time of ending the interview, it's 11: 55. Thank you so much.
