# Supplementary material for: Acceptability of Digital Adherence Technologies to support people with drug-susceptible TB in South Africa
Source: PLoS One. 2025 Sep 24;20(9):e0332103. doi: 10.1371/journal.pone.0332103 (PMC12459780; doi:10.1371/journal.pone.0332103)
Supplement: S4 File — (ZIP) [file pone.0332103.s004.zip › S4 Transcripts/HCWs and Stakeholders/IDI 25- HCW.docx]

**TRANSCRIPTION NOTATIONS**

| **Label Key** | **Meaning** |
| --- | --- |
| **I** | Start of each new utterance by the Interviewer |
| **P** | Start of each new utterance by the Participant |
| **N** | Note taker |
| **{ }** | Indicates that details were changed or pseudonyms were used to anonymise data |
| **( )** | Indicates the description provided to anonymise data |
| **XXX** | Words were omitted to anonymise data |
| **-** | Breaking into a sentence by the next speaker |
| **…** | Pause or drawn out words |
| **[ ]** | Indicates noise made, e.g. [laugh], [sigh], [pause] |
| ? | Beginning of utterance by unidentified speaker or questionable text |
| **[inaudible segment]** | Unclear section of the recording |

I: Thank you for agreeing to participate in the interview today. Can you please allow us to audio record this interview?

P: Yes, you can record it.

I: Okay, PID number its xxxxx, date: xxxx (interview date). Location: xxx [clinic name] in xxx (name of town) , xxx (name of district), type of the interview: community healthcare worker, facilitator: xxx [interviewer’s name], time of the interview: it’s 9:38. Okay, tell us what is your current profession at the clinic?

P: Oh, my current position is community healthcare worker at xxx [clinic name].

I: Okay, so what are your responsibilities in relation to patient care and counselling?

P: Oh, my responsibilities?

I: Yes, your roles?

P: My roles? Okay, my roles or my responsibilities is to trace, trace… our chronic people.

I: Mmm.

P: All the chronic diseases that we have here at our facilities we are actually tracing all of them and to do campaigns concerning our immunisations and to deal with where the vitamin stays and again to register all the households that our clinic serves… our entire ward that the clinic is serving.

I: Okay, you are registering them.

P: Yes, registering all households that has been served by our clinic.

I: Okay. You are registering them on what?

P: Uhm, we register the household because we have to know the population.

I: Mmm.

P: Yes, that has been set by our clinic.

I: Okay.

P: Yes.

I: Then in relation to TB?

P: In relation to TB?

I: What are you doing?
P: Okay. We normally, we make sure… we… we actually some of the people- the TB patients we deliver some medication sometimes to those who are not able to come to the facilities due some reasons, yes.

I: Okay. So, what are the reasons of patients not to come to the clinic especially TB patients?

P: Mostly, it can be someone-some of them are living alone at their place and most of them will be very sick and not be able to come to the facility and some of them will complain about the transport money to come to the facility.

I: Mmm.

P: Yes.

I: How do you know about all this information, who told you? Who is telling you that this patient has got this problem?

P: Uhh, we are getting the information from our data captures or the facilitators, then the TB nurses will be saying these people are no longer coming to the clinic, here’s the file he/ she was supposed to come on this date, but she missed the date that they will be telling us and giving us the address and name of that patient so that we can go physically to the person and see what’s the problem for him or her not to come to collect the medication to the facility.

I: Mmm.

P: Then there is this uhh, box that has been introduced.

I: Mmm.

P: For those kinds of things, for us not to wait for them not to come to the facilities we should see same on day that we should see- on the exact date that if the patient has not taken his or her medication.

I: Mmm.

P: Then we will be able- then those boxes help us to attend to some kind of patients as soon as possible because we will be seeing the problem as it happened… on the exact date.

I: Mmm.

P: Yes, and those box I think they are very important because now, because now….[Inaudible segment].

I: Mmm, because… you are saying that boxes are important, how are they important?

P: They are important because if the patient is not taking the medication, we will actually… see.

I: Mhm.

P: Then we will have to attend and check why the patient is not taking his or her medication.

I: Okay.

P: And [Music]. I think those boxes are important because they are actually-I can say they helped us as us community healthcare workers because we used to go house to house to check if people- if our patients are taking medication in time and if they are taking them every day.

I: Mmm.

P: Like they have to.

I: Mmm.

P: So now we can see every day if they are taking, or they are not taking, but some of them got this tradition of opening the box when the box is indicating that it’s the time for them to take their medication, they are just opening the box. I am saying that because I had this other patient here at xxx [clinic name] who used to do that and then we made it our responsibility to go and check every day. We actually took the medication.

I: Mmm.

P: And made sure that she drank medication every day while we were there and monitoring her while she was doing that because she used to take the medication and put them under the mattress.

I: Mmm.

P: She wasn’t taking the medication and what made us realise that she wasn’t taking the medication is because whenever she came to the facility uhh, they will be checking that if the medication is responding well to her body, but it seemed like it wasn’t responding so they realised that maybe she wasn’t taking her medication. So, us as community healthcare workers we think it will be better if this uhm… those boxes will actually respond- reflect to our phone as community health workers so that we can- so that we will… so that we should know how many patients, how many patients do we have and that so that we can go maybe each and every Friday to see if they are really drinking their medication not to just rely on the box because some are just opening the box to have the good uhm… to have the good… I could say report good they should be on their good books here are our facilities knowing exactly that they don’t take their medication. So, I think it will be better if we will be able to… those patients should be allocated to our phone and we should also see whenever they are opening their box it should also reflect to our phone and it should, they should uhh, make it our responsibility to know how many patient do we have each facilities so that each and one of us should be allocated maybe two or five patients that you know that each and every Friday you have to attend those patient.

I: Mmm.

P: It should be every week.

I: Okay.

P: For us to make sure that the boxes are… really, really working that one will know 100 % that they are very helpful. I can say they are they are very helpful.

I: Mmm.

P: But… people that we are leaving-people that- our patient. It’s not all of us who will respond and cooperate with the system that has been introduced like those boxes.

I: Mmm.

P: For an example.

I: Mmm.

P: It’s not all of them who will cooperate well, but I think it’s also our responsibility to go and check them regularly to make sure that we are 100% sure that the boxes are very helpful.

I: Okay.

P: Yes.

I: So, you are mentioning that uh, the other patient here at the clinic was just opening and not drinking the medication.

P: Yes.

I: How did you find out that this person is not drinking he is just opening?

P: Our TB nurse told us, “okay, xxx [ interviewer’s name] I think you should go and check xxx [patient name], to check who is she staying with,” then I went to the house and when I got there… there was someone who was taking care her, but it was a friend and the friend told me that whenever she is cleaning, she got the pills under the mattress and then my TB nurse realized that it may be the reason she wasn’t responding to the medication.

I: Mmm.

P: There wasn’t big changes.

I: Mmm.

P: Even if she was drinking her medication , but that’s how she realized that maybe this person is not taking the medication right.

I: Even though the box was there?

P: Even though the box was there to remind her. The reason was not… the reason for her not to take the medication was not that she forgot, or she actually got reminded by the box, but she didn’t want to take the medication, yes.

I: Okay. So, you went to the patient?

P: Yes, I went to the patient and asked her why don’t you take your medication and she was saying okay these pills are making my foot sore, yes so it went itchy like I can’t. My foot are painful, I think it’s because of this medication because I got sick after I took this TB medication. So, she thought that the reason why she was sick is because of the medication to show she decided to stop the medication.

I: Yoh, and just open the box?

P: Yes.

I: Mmm. Did you see any difference after you… so what else what did you tell the patient? I hear that you wanted to know why they are not taking medication and they told you that they think the medication makes them sick and now they are just taking it out of the box and put it under the pillow, why did you- how did you resolve this? What did you say to the patient?

P: Okay, I… I came back to the facility and I asked the operational manager if it will be fine if I take the box and make it my responsibility to give the patient medication everyday while I am there with her physically so that I can see if she drinking the medication.

I: Mmm.

P: And then I did that for a month.

I: What-how did she do?

P: She was responding very well and the weight, she even picked up the weight.

I: Oh, what did you do? Did you take the box?

P: I took the box, then I went to her place every day.

I: So, the box was with you?

P: Yes. The box was with me and whenever I go to my place, I’ll open the box.

I: Okay.

P: And giving her the medication, then I would go back to my place with the box. Then tomorrow morning, I will go again.

I: Okay.

P: Yes.

I: So, what time was the box alarm and what time would you be able to go to her place?

P: We had to change the time because I couldn’t go to her place at night, it was far from my place.

I: Oh.

P: The only time I could make it was for me to go there in the morning.

I: Okay.

P: Yes.

I: How did you change the time?… you changed it where? With the intel here at the clinic?

P: No, I… yes, I asked uhm… Sister [ nurse’s name]if I could give her the medication in the morning instead in the evening.

I: Okay.

P: Because it seems like she wasn’t drinking the medication in the evening, and I won’t be there in the evening to be able to make sure that she is drinking her medication, then we just swapped it to morning. She then asked me what time can you be there in the morning every day.

I: Mmm.

P: Then I said I will be there at 7 o’clock in the morning every day because I knock uhm, I go to work at 8 0’oclock, then I decided to be there at 7 o’clock.

I: With the box?

P: With the box every day.

I: Okay.

P: Which I did for a month then after a month eh, the brother came to our facility to tell us that he is taking her with him, she is no longer staying here at xxx [ patient’s resident]. She is now far, but he made a promise that he will make sure that she is drinking the medication.

I: Okay.

P: And then the nurse asked her not to take the transfer because they wanted to monitor if she is responding well as she was responding while I was giving her the medication.

I: Okay.

P: Yes.

I: For how long the nurses asked not to go for how long?

P: Until she completed the-

I: Treatment?

P: Treatment.

I: That is the nurses asked her to stay here for 6 months?

P: Not here but he, they… they didn’t they asked him not to take her.

I: That-

P: Other facilities.

I: Oh.

P: Yes.

I : For a few he would still come here with you?

P: Yes.

I: Even though she’s-

P: Even now, yes.

I: She is no longer staying with?-

P: It’s not that far, but for me as a community healthcare worker because I don’t have transport, it was going to be hard for me to go and give her the medication every day.

I: Okay.

P: Yes.

I: So how is the patient now?

P: The patient, I have never seen her because it happened last month so now I… I was, I am from leave.

I: Okay.

P: I had a two week leave, so I am not sure because it was my first day after a leave, after that leave.

I: Okay.

P: Yes.

I: So, did the brother huh, promise to continue to support the patient?

P: He did, he did make the promise that he will.

I: Did you give them the box?

P: Yes.

I: So, the brother was continuing with the same thing?

P: Yes, but he was continuing. I have never went to [ intern name] to ask her to check for me if she is doing the right thing on the…

I: Platform?

P: Platform, yes.

I: Oh, okay. What did you talk-what did you say to the brother in how to support the patient with the box?

P: Uhm… I… I actually told them that… I think that person need to adhere every day.

I: Mmm.

P: Because I think she was actually… she was losing hope.

I: Okay.

P: She was very ,very sick.

I: Mm.

P: Which she also didn’t have any hope.

I: Mmm.

P: And then I was- I think for me going to her place every day made a difference because I would stay for 30 minutes and talk to her and reminding her. Asking her to show me her pictures, old pictures. She would be showing me the pictures and reminding her where she- who she was before she became like this and telling her that you can go back to this body, you can go back to wherever you were before you get this illness.

I: Mmm.

P: And I think she was responding well because she had someone who was encouraging her, motivating her to… what, I am not sure if the brother is doing the same thing because he is working, and he will only be with her in the afternoon. During the day she told me that the patient will be left with the brothers girlfriend.

I: Mm.

P: Yes.

I: Okay, so what did you say to him? To the brother about the box? Did you explain to the brother?

P: I explained how the box is working and I told him why the box ended up being in my hands.

I: Mmm.

P: Not with the sister’s hands (patient).

I: Mmm.

P: I told him that the sister (patient) was just opening the box and didn’t take the medication. I told him that you should make sure that the patient is drinking the medication and check if she did really drink the medication.

I: Mmm. So, when you were telling the brother how the box works, what did you say to the brother about how the box works?

P: I told him that the box is set, it set the time that the time that the patient will drink the medication and the box will ring.

I: Mmm.

P: Beep, and when the box is beeping it will…

I: Flash?

P: It will flash, the lights will flash as the reminder to our patient that it’s time for you to take your medication you should also listen to that box and give the medi… open the box and give the patient the medication and then whenever you open the box, that box reports back to our facility that you did open the box and took your medication and close it again. Make sure that it’s very closed and will be, whenever the box is red you should take it back to our facilities so that they can charge it and give it back to you or give you the charged one and set it again to the time that the patient is drinking the medication same time that the patient is taking the medication, yes.

I: Okay, so did the brother understand how the box works.

P: He did understand, yes.

I: Okay. And he continued to support the patient?

P: Yes.

I: Alright that’s very good to hear. So, I want to know what are you doing in supporting the ASCENT program?

P: Uhm…

I: What are your roles? What are your responsibilities in the ascend project? How you work with the interns and nurse?

P: Uhm, my responsibility is whenever we see that there are patients who are not drinking their meds properly, it’s my responsibility to go back to them and check why, what is the problem that they aren’t taking their medication right and after getting what’s the problem. Then I will see if I can solve that problem like that other one I think I had solved, but I didn’t actually solved it, I asked permission to my seniors and to those people that have more knowledge, than I have too. So, my responsibility… go back to those people who aren’t taking those medication right and show them how it, and… encourage them to take their medication and make sure that they return to our care to get the proper care they need to be to get.

I: Okay.

P: Yes.

I: Okay, so you mentioned that uhm, your responsibility is to go and go follow up the patient that are not taking the medication-

P: Yes.

I: How do you get to know that this patient and this patient and this patient are not adhering to treatment? How do you get to know all of that?

P: After they have missed their appointment or after they have come for their appointment and then our TB nurse will be telling me that the patient is not responding, I will just have to wait for the feedback from my TB nurse.

I: Mmm.

P: Which I think it’s… it can be… uhm, how to put it… it can make it easier for us to know even earlier than after coming to their appointment.

I: Okay.

P: That’s why I said that it will be easy if those boxes can be linked, can be linked to our phone.

I: Mmm.

P: For us to get, to even know them faster than we should wait to hear from ward TB nurse.

I: Okay, alright that is noted. And uh if you were to explain to a new community healthcare worker about what is this digital adherence technology is all about, what would you tell them?

P: I would tell them that… we’ve got an essential… an important resource that… need to… it’s a good, I think it’s a good important resource it just needs a little improvement.

I: Mm.

P: That will benefit our patient and us as community healthcare workers and it will make our work easier.

I: Okay. What is that what is your idea on that needed improvement?

P: For it not to link with only the facilities but also to link with all the community healthcare workers phones.

I: Okay.

P: Yes. Apart from that-it is very good. It has helped out most of our patient, it has helped… our workers, our nurses yes to see that is progress to whatever they are doing as nurses. Yes, it has made a good impact.

I: Okay.

P: Yes.

I: Okay, what else can you say to a new community healthcare worker that you tell them about the boxes, what else can you tell them how, if you are to explain the boxes work? What would you say to the new community healthcare worker that you have to teach them this box?

P: I would say… okay the box is-it’s for our TB patient that uh… contain their medication and then it helps… the nurses to see if their patients are taking the medication well.

I: Mhm.

P: And it helped us as community health workers… to… to see if our patient are taking their medications-

I: Mmm.

P: Daily, we don’t have to wait for longer to see if the patient are taking their medication like before because before we had to wait for them to come to their next appointment so that we can check on the card to see if they are taking their medication but now it’s more easier because we can see if they are taking them daily like they have to do.

I: Mmm.

P: It has made everything simple; it has made everything possible.

I: Okay.

P: So, yeah.

I: What else is important that you can tell the new community health care worker about the box, the importance of the box? I can hear that you are saying it contains the medication for the patient, it helps you to see and monitor early that this patient is taking their medication and what else can you tell somebody new about this box? Another important component of the box for the patient?

I: Okay, most of our patient- some of them are old some and forgets easily. So, it helps them not to forget, it reminds them by beeping and flashing so there is no way that our patient can say I forgot and then it can take it everywhere you go with it.

I: Mmm.

P: That’s the advantages. So, there is no way that you can’t say I forgot my medication. It’s portable, you can take it in your bag and yes, I think those are the most of them.

I: Thank you so much for mentioning all of the, that important information. Can you please describe your role with uh follow-up’s? Your role with differentiated care? Meaning that with these follow-up’s that we do what is it exactly that you when you are following up on patients?

P: Oh, I normally phone them.

I: Mmm.

P: If I don’t get them on their phone, I will be having their address, then I will physically. I will be physically going to their place to check them physically.

I: Okay. What challenges are you experiencing when you have to do this follow- up? You have to call, uh visits, how is it to do that?

P: Uh, the only challenge that I have is sometimes transport because we don’t have transport. I don’t think there are many of the challenges, there aren’t many challenges. It’s only that transport thing, but apart from that… no, no, no, no, again we will be meeting people with different attitude with different personalities so… you won’t even know if this person will, is going to cooperate, is going to work with me on what I am actually here for. So, those are the only challenges that I think we normally face, but apart from that… I don’t think there is anything else.

I: Okay, alright so tell me how do you share the responsibilities between you and the healthcare worker and the TB nurse? How do you share the responsibility of following up patients? What does the nurse do and what are you supposed to do?

P: I think it’s…

I: How do you share the work of following up on patients?

P: How do we share the work?

I: What exactly the nurse does, what do you do? And maybe the other person does, I want to see how do you share the work of following up on patient as TB team?

P: Okay, because all the information about our patient, we got that from our nurses, our TB nurses.

I: Mhm.

P: So ,we will just have to wait as community health workers for them to give us those patients that we have to follow.

I: Mhm.

P: But if they are not giving us any patient, we won’t know that we do have the patients, the TB patients. We will just have to wait for them to give us the patients that we have. That’s why I said I think all the TB patient need to be the nurses responsibilities and the community healthcare workers responsibilities, we should know all of them and all of them should be allocated to us individually maybe each should get 3 that you know that you actually making the follow ups each and every week.

I: Okay.

P: Even if they do have those boxes, I think it will be easy if we check them each and every week that will be easy, we won’t have to wait for the nurses and tell us that this patient has defaulted, this person missed his/ her appointment. We will be able to see that because we will be visiting them every week.

I: Mmm.

P: Yes.

I: And you also suggested that you also need to help them?

P: Yes.

I: Help them, why?

P: To have them on to be allocated to our phones.

I: Mmm.

P: Yes.

I: Okay, alright. So, are you working well together?

P: Yes-

I: Are you sharing the job?

P: We are working well together…

I: With the nurses?

P: With the nurses, it’s just that little improvement that need to be made.

I: Like what? What improvement?

P: The one that I just said that we need to know all the TB patient not wait for them not to wait for them to miss the appointment.

I: Okay.

P: Yes.

I: Alright, so how is that going to help?

P: It is going to help because we won’t get to have to have those people who are not drinking their meds, who just open their box and not… if we do have those patients, we will know that okay, we will check, we will see that in time and try to solve the problem as early as possible before it gets late.

I: Okay.

P: Yes.

I: Alright. And then so when, when you first heard about this digital adherence technologies what were your expectation about this technology?

P: My expectation was… we are going to… fight this TB thing, this is going to be a great help to our patients we won’t have more patient that we do have. More TB patient that we do have because people will be taking their medication and it won’t be that it won’t be that contagious because there will be taking their medication well in time and they won’t have any excuses that I forgot. I thought it was going to help to reduce the changes the chances of people getting infected.

I: Mmm.

P: Yes.

I: So, did you?-

P: Which I think it still can because it has erased most of the things that… that we… the negative things that we actually experienced about the, at first with our TB patients, like those who will be like saying I forgot, eish I am staying alone, I have got so many things in my head those pills sometimes they are the last thing in my mind like they won’t have, we don’t have those excuses anymore.

I: Because of the box?

P: Because of the box.

I: What will the box do to make sure that those experiences are no longer there, like I am trying to understand how is the box going to make uh, this improve?

P: I don’t say they forgot because they are got a lot of things on their mind. The box will beep while flashing even if that persons is not hearing, but he will see the flashing of that thing trying to assist while box is ringing.

I: Mmm.

P: Mmm, even those who can’t hear, they can see that the box is flashing which means it reminding me to go and drink my medication.

I: Okay.

P: Yes.

I: So, did your expectation change?

P: Yes.

I: How?

P: We don’t have most- we don’t have uh, the fault. The huge percentage of the default that we used to have before.

I: Mmm.

P: Yes. It dropped.

I: Okay.

P: Yes.

I: What made it to drop?

P: I think this box have, had a great impact on that because just like I said, they had many excuses before that box can be… before that box is there.

I: Mhm.

P: Yes.

I: Okay. So now the box is supporting them?

P: The box is supporting and it’s helping.

I: Mmm. What else do you hear from patients that make you feel like the box is helping? What are the patients telling you about the box?

P: Oh ,most of them feel that they are very special, and they are very, very valuable because… all their complains and their… have been solved. Just like I says they will be saying I forgot, I am staying alone, I am old; I can’t remember all those things. So ,now they don’t have those excuses. They feel very special.

I: Mm.

P: And they are now taking their lives seriously more seriously because they have seen that the government itself took… responsibilities.

I: Mm.

P: Yes.

I: Okay that’s good to hear all of that can you please describe the training and the resources uh, that you received on how to deliver digital adherence technologies? How was the training?

P: Uh it was very good, excellent I’d say.

I: Mm.

P: Because even there at the training I have learnt some of things that I didn’t know just like… there could be that- for an example that box. I felt it made things- it actually has- I thought it was going to make things more easier and which I believe it is making things easier.

I: Mmm.

P: It turned out my thoughts were correct; it became exactly as I anticipated. So, that training was very helpful.

I: Mm. Do you still remember where you attended raining?

P: Yes, I do.

I: Where was it?

P: Hunters rest.

I: Okay. Do you still remember that activities that you did on the training? What exactly did you do in terms of the activities there?

P: The activities we…

I: What did you learn?

P: Uhm, I was trained on how the box works and then… I was told it has the battery that lasts for 6 months.

I: Mhm.

P: Yes, and then after that 6 months. I was also told that the battery can only be charged by the facility user.

I: Mmm.

P: Yes, the patient cannot charge the box himself/ herself… and I have learnt, I learnt more strategies, more new strategies of how to adhere that I didn’t know before.

I: Okay, you’ve learnt strategies of what?

P: How to encourage or how to adhere.

I: Mmm.

P: Yes.

I: On how to encourage to?-

P: Encourage people to-

I: To adhere?

P: Yes.

I: Okay, so why did you learn about that?

P: Uhm…

I: Those strategies that you didn’t know about?

P: Not that I didn’t know, but the things that I didn’t realize that can, that can help.

I: Mhm.

P: Our patients to understand better.

I: Things like what?

P: Things like you have to empathize, to put yourself in the patient’s like… yes, different natural skills actually from different people, yes.

I: Okay. It’s good to hear that, okay. So uhm anything that you can suggest how can we improve the trainings? What can we do to make the training better? What gap you didn’t notice on our trainings?

P: Uhm, the training was excellent, the only thing that I can do to… to, I think to improve, I would put this suggestion box for each and every one who attends the training to put their… to put their different inputs or their…

I: Comments?

P: Comments yes.

I: Okay, about the training?

P: About the training yes.

I: Why do you suggest that? How is it going to help?

P: Uh, it is going to help because most of us- it’s not each and every one who will be able to stand up in front of many people and express their feelings or express their knowledge. Most of… or some of us aren’t comfortable to stand up in front of each and every one, but they do have valuable things that they can… I believe, I believe they do have valuable things that they can say that can help us to improve. I think it would better if you put the suggestion box for us those who are… aren’t that brave to stand up in front of everyone to put our comments there.

I: Okay.

P: Yes.

I: That’s good to hear that and from your perspective as a healthcare worker uh can you please uhm describe the benefits of implementing these technologies in supporting TB patient? How is it beneficial as a community healthcare worker?

P: It benefits us community healthcare workers because uhm, we no longer have many people who needs to be traced, we no longer have many defaulters yes. So, it has worked on our favour… yes.

I: Mmm. Okay, what are the challenges of implementing the smart pill box and the differentiated care like supporting patients, calling and home visit of the smart pill box, what are the challenges that you experienced?

P: The challenges are that whenever our patients come to our facility the, the main thing that we all want as community healthcare workers, nurses and professional nurses is to see their patients getting better to whatever that bothering them, whatever that’s bothering them. So, this box have helped us a lot because we have seen a lot of improvement to our patients, they are drinking their medication well in time, so I would say this box have helped a lot.

I: Okay. Any challenges that you can think of that you have experienced presenting the boxes?

P: Oh, any challenges, the challenges are we will be expecting them to get better, we will be expecting to see the difference because each and every employee whenever she or he is doing something he expects, or she expects to receive the… to receive the…

I: The change?

P: The change. So, if we are working and we are not seeing the changes we will just say there isn’t anything that we are doing. So, whenever-so with those people who are just opening up the box and giving us hope that we are doing the right thing not knowing that they are just doing their own thing we didn’t tell them to do, it hurts us, so I see that as a… as a challenge.

I: Okay.

P: Yes, which needs to be attended as I said it would be better if we could improve by linking… the information from those boxes to the community health workers phones.

I: Okay, Alright. So, what are the, what are the positive changes that this box brought into the facility?

P: The positive-

I: Changes-

P: Changes is that… we no longer have defaulters, many defaulters like before.

I: Mm.

P: Uhm most of our TB patients respond very well, they not- as we know that our TB patients have uh, have to take their medication for 6 months, but if you are okay after that 6 months you don’t have to go for another medication for another month and most of our patients takes treatment and complete within this six months. So, this means these boxes have helped a lot.

I: Okay.

P: Yes, like before.

I: Mhm.

P: Yes.

I: That’s very good to hear that. So, what are the negative changes that these boxes brought in the facility with TB patients?

P: I don’t see anything negative, it’s just that it needs to be improved, but apart from that we don’t have anything negative.

I: Okay.

P: About the box.

I: Okay, when you say-

P: But it needs to be improved, what I could say might be a negative thing is that we are not sure whether patients are drinking their medication.

I: Mmm.

P: Knowing that it’s possible for them to open up and not drink the medication.

I: Okay.

P: Yes, that’s the only thing that I can say, it gives us the false hope.

I: Okay.

P: Yes, as community health workers or as TB nurses.

I: Okay.

P: Yes.

I: Alright what can we do to improve this negative uh, what do you think can improve this negative?

P: By physically monitoring them regularly to see if they are really taking their meds, yes.

I: Okay, that’s very good to hear. So, so how do you think uhm, do you think you will be able to run with this program and support nurses, support patients with these boxes and make sure that everything is going smoothly without the support from xxx (organisation name)?… just think about it when-

P: Will, I think?

I: When we are no longer here just have the boxes, the nurses giving the boxes to the patients, you having the platform in the phone and the nurses having the platform in the phone you know going home and supporting them monitoring them on your phone as to the patient is taking cared or not, do you think you will be able to implement this uh smart pill box on your own without the ascent, without the xxx (organisation name) without ASCENT or xxx (organisation name)?

P: I don’t think we would.

I: What will be the problem? Because you’ve been trained and everything you will know, you will know how to register, you will know how to monitor because we are going to install, we are going to teach you everything then at some point we will have to go where do you run with the program. Do you see it possible for you to do that to get the lessons for all other people without the xxxx (organisation name) team?

P: Uhh, it won’t be possible, we won’t do it without xxx (organisation name) because each and every innovation will need to be improved because we not- the world is changing, and life is changing, each and everything is changing. So, everything need to be improved as… the changes, the life change will actually be there like I said now we thought that the box is, we thought that, that box is complete.

I: You thought the box is complete?

P: We actually thought when- when those people who invented that box, they thought it’s complete, it will help 100%, not knowing that there will be people who think ,okay ,let me just open the box and do this. Those people will think I drank my meds. Like now, like as I said now, we need to improve it, so I think most of the improvements we get they are from xxx (organisation name).

I: Mmm.

P: So, I don’t think we will do well without them, even if they have trained us and have gifted us those boxes and showed us how it works, but the improvement is needed. So, I think and trust that they can be the good people to do so.

I: To?

P: To… like ASCENT you have trained.

I: Mhm.

P: We have been trained and then we’ve been… but there are changes, there are changes every day.

I: Mmm.

P: So, the improvement needs to made every day.

I: Mmm.

P: So, I can’t say we will cope without xxxx (organisation name). We will also need them for that, their improvement.

I: Okay.

P: Yes.

I: Alright. Okay uhm… okay so we are almost at the end of this interview and then before we can close this interview is there anything else that is important that you wish to mention in interview before we can close it, anything that you would like to tell me like closing marks about implementing this strategy you know more things you would like me to about that you can tell me before we can close?

P: Mm… I don’t think there is anything that I have left out.

I: Mhm. Except what?

P: Uhm… I think that’s enough.

I: Mhm.

P: Yes.

I: Okay, alright. Thank you so much for your time and really agreeing to participate in the study and the strong information that you were giving us, we really appreciate it, your time and this is the end of the interview and time ending the interview it’s 10:00 am, thank you.
