# Supplementary material for: Acceptability of Digital Adherence Technologies to support people with drug-susceptible TB in South Africa
Source: PLoS One. 2025 Sep 24;20(9):e0332103. doi: 10.1371/journal.pone.0332103 (PMC12459780; doi:10.1371/journal.pone.0332103)
Supplement: S4 File — (ZIP) [file pone.0332103.s004.zip › S4 Transcripts/HCWs and Stakeholders/IDI 26_ HCW.docx]

**TRANSCRIPTION NOTATIONS**

| **Label Key** | **Meaning** |
| --- | --- |
| **I** | Start of each new utterance by the Interviewer |
| **P** | Start of each new utterance by the Participant |
| **N** | Note taker |
| **{ }** | Indicates that details were changed or pseudonyms were used to anonymise data |
| **( )** | Indicates the description provided to anonymise data |
| **XXX** | Words were omitted to anonymise data |
| **-** | Breaking into a sentence by the next speaker |
| **…** | Pause or drawn out words |
| **[ ]** | Indicates noise made, e.g. [laugh], [sigh], [pause] |
| ? | Beginning of utterance by unidentified speaker or questionable text |
| **[inaudible segment]** | Unclear section of the recording |

I: So, do we have permission to record you?

P: Yes, you have.

I: Thank you. Date of the interview it’s xxxx (interview date). The location is xxx [clinic name]. The language used for the in-depth session is English. And the participant ID is xxxx. The time we are starting the session is 1 minutes past 12. So, can you tell me what’s the title of your current position?

P: Professional nurse.

I: How long have you held this position for?

P: xxxx (number of years).

I: Okay. So, what are your responsibilities when it comes to TB patient care?

P: It uhh, the, the uhm, the total management of TB patients starting from uhh, screening, testing, and treating the initiating patients on treatment and treating the patients throughout the uhm, the duration of treatment course.

I: Okay.

P: Even also referral if where- when when it is necessary.

I: Okay. And uh at your level, how do you deliver services- at which level are you delivering services, is it at a district level or provincial or facility level?

P: We are- it’s facility at- at local level.

I: Okay, at local level?

P: *Yah* [yes] at, at a facility.

I: Okay. So, now I would like to know what do you know about the ASCENT study. So, if you were asked to explain what Digital Adherence Technology is to another health care worker who knows nothing about ASCENT , who knows nothing about DAT, what would you tell them?

P: I would say that uh, Digital Adherence Technology is whereby we monitor the adherence, patient’s adherence to treatment remotely.

I: Mmm.

P: We, we, we don’t have to be in physical contact with a patient, we monitor the adherence of a patient remotely.

I: Yes.

P: Yes, so, that what, that what, to be in short, that what it is.

I: Okay. So, can you tell me what is used to monitor uhh, patients remotely. How does this remote monitoring work exactly?

P: Huh, Digital Adherence Technology uh, we, we, we, we able to, to be in contact with a patient using a, a, a smart box.

I: Okay.

P: And which enables the patient- enable us the, the clinicians to monitor the patient’s adherence taking the treatment on a daily basis as well as it reminds on the other, and it reminds a patient to take the treatment by you, by -what do we call it uhm, an alarm, that will, that will moni- that will help the patient remind a patient to- when it’s time to take the treatment. Also, will remind a patient when it’s time, a day before to refill to come and collect the patient at the facili- uhh, the treatment at the facility.

I: Okay, and how do you monitor these patients remotely, what do you use?

P: On a daily basis we use uhh, platform whereby we enter it to the platform and then on daily basis we check if the patient did uhm, take the treatment because when the patient opens a box to take the treatment, it’s register on a platform whereby we on the, on the computer on the PC we are able to, to, to see that, to monitor that on a daily basis.

I: So, how, how do you identify patients who have taken medication and not taken medication on the platform?

P: When you open the platform, the platform will show red when the patient didn’t take the treatment. Remember the patients will not take the treatment at the same time, so when we open for, like today, when I open it, the, the, the platform I will, red will be indicated for the patient who didn’t take the previous day.

I: Mmm.

P: That will be an early lost to follow.

I: Mmm.

P: Early uhh, missed doses and then uhm, if the patient did take the treatment, it will register, it will show green. That will be, that will tell us that the patient did the treatment on that day. If it’s red, the patient didn’t take on that day and we can see it. At least earlier the previous day and then it will be an early uhh, an early lost to follow that we can quickly get to the patient and telephone- by telephone.

I: Mmm.

P: To remind or find out what happened, why the patient did not take the treatment on that day.

I: Okay.

P: And then we have, *yah* [yes].

I: Okay. So, what do the patients receive when they don’t open the box before you even contact them telephonically, what happens to the patients?

P: An alarm reminder-

I: Mmm.

P: Will always be beeping to remind a patient to take the treatment.

I: Yes.

P: Then unless a patient is not with a, with the box nearby-

I: Mmm.

P: Then the patient will not be reminded but as long as the box is next to the patient, the alarm will keep on beeping until the patient takes the treatment. Or unless maybe the patient has moved to another place and left the box behind and could not hear the beep.

I: Mmm.

P: Then that’s when the patient could miss the treatment.

I: Okay. So, what other forms of communication is used to check on the patient before you make the phone call (……) uh do you know about the SMS?

P: *Yah* [yes] the SMS *yah* [yes]

I: Yes, yes, can you tell me more about that one?

P: Not too much, not much but we are simple, the SMS will uhh (……) I’m not too familiar with the SMS.

I: SMS?

P: No, not really.

I: Okay.

P: But I know that the SMS will automatically be generated- the message to the patient automatically, we don’t have to send a message. It will automatically be sent to also remind the patient.

I: Okay, that, that’s right. So, can you tell me your role within the differentiated model of care of intervention? Do you understand what that means, the differentiated care?

P: Can you please elaborate a bit?

I: So, it- these are the follow up actions taken when a patient misses a dose, and you see red. So, the phone calls, the home visits, so, what’s your role in this follow up action?

P: Uhh, firstly, it will be to, to, to, after the patient missed a dose for, for a first day, we will remind the patient, find out what is happening by telephone.

I: Mmm.

P: After the message, SMS message and then uhh, if we don’t get uhm positive response from the patient, on the second day we will send a message again, but then on the third day. Then we will initiate uhh, track and trace, physical track and trace meaning we will send somebody to the address of- to, to, to go and find out what is happening with the patient.

I: Okay. So, what do you do uhh, all the time, which action do you do all the time more frequently?

P: More frequently when patient is visiting the clinic-

I: Mmm.

P: We continue with uhh adherence counselling.

I: Okay.

P: It has to be, it has to be, it has to be continued that normally, a normal practice for patients to, whenever we refill or hand out treatment or prescribe or uh what we mean, we review the patient on a review day.

I: Mmm.

P: Continuous counselling, it includes continuous counselling which is very important.

I: Mmm.

P: It’s continuous, it has to go on for until the patient completes the treatment, unfortunately.

I: Okay, okay. And which follow up, follow up action you do least, which one do you do less frequently?

P: Less frequently it will be uh trying to find out if the patient is taking the treatment when the patient is not, in physical contact because now then the, the, the smart box, it will do it.

I: Okay, alright.

P: Mmm.

I: So, how do you work with other health care workers in terms of the differentiated model of care because you mentioned that you send people to trace- to do physical tracing, who do you send and how are you working with these other staff members?

P: It’s staff members that is helping with tracing lost to follow up patients.

I: Mmm.

P: Are from WOBTS (Ward-Base Primary Health Care Outreach Team), Ward-base, uhh *yah* [yes] Ward-base.

I: Yes.

P: *Yah* [yes] Ward-based staff that goes to physical addresses of the patients on daily basis. Their main function is to, is track and trace.

I: Mmm.

P: And then all the lost to follow up patients are traced by them.

I: Okay

P: And also, they also do visit a patient to uhh physical- help the patients- those who could not, who are like uhh, [inaudible segment]

I: Okay.

P: That’s what their most function, we the clinic at a facility level, we mostly uh work hand in hand with them for track and tracing uhm-lost to follow up patients.

I: Okay. And, so, when you first heard about digital adherence technology before it was implemented, what you, what were your expectations about maybe it use and what it going do to your job, to your role, what were your expectations?

P: My expectation with the digital adherence technology is to help out with adherence because now adherence to treatment is the main problem with TB.

I: Mmm.

P: That an- it like a backbone of uhh, TB success.

I: Yes.

P: TB treatment success.

I: Mmm.

P: Without adherence uh compliance, then you are not doing- you are not going anywhere.

I: Mmm.

P: So, I was expe-, I am expecting, and I was also expecting it to ease the burden of, you know following up the patient up and down trying to make sure that the patients are taking their treatment.

I: Mmm.

P: As also before the, the, the digital technology, our, our- the programme that we followed, we followed a DOT that is the Directly Observe Treatment-

I: Mmm.

P: Short course-

I: Mmm.

P: The DOT meaning that the patient has to come to the clinic on daily basis to come and collect their treatment and you have to see if the patient swallowing the, the, the treatment.

I: Mmm.

P: Of- was also a burden to the, to us clinicians because now we have to see the patient taking the treatment on the other hand and other patients are waiting, the time we could use to- utilise- to be saving other patients so to shortened the waiting time at the clinic. We are also busy seeing the patient applying the short course uhh DOT, DOT programme.

I: Mmm.

P: And also, the patient- it’s difficult for the patient. It makes the patient’s life difficult when they have to instead of going to find something for themselves since there is an unemployment problem.

I: Mmm.

P: Patients go out there to look for jobs.

I: Yes

P: Something to- that will help them out to get something to put something on the table. The time they waste to come to the clinic in the- every morning to come and collect the treatment is the waste of time.

I: Mmm.

P: Of which now it’s a, it’s a disadvantage the DOT programme because now that- why now because it causes, it causes a lot of patients to, to know, to leave the treatment to, to, to just forget about the treatment and not want to continue with the treatment because they take their time to come to the clinic everyday to take their, instead of going to find some, something to eat out there.

I: Mmm.

P: So, we, we, we are expecting uh the digital technology to, to help out there, of, of which is already doing that, it is doing that.

I: Okay.

P: So, we are happy to, to be having the adherence technology because now that burden has, has fallen off.

I: Okay.

P: Patients are happy with it, I’m happy with it because we don’t- we no longer have a problem like that.

I: Okay. So, you’ve answered my next question, which was uhm, if your opinion about the Digital Adherence Technology changed after implementation, do you have any more comments on that?

P: *Yah* [yes] what are, what uhh in fact, it has done a lot with the helping out- stop uh for relieving the patients from coming to the clinic on daily basis and for the clinicians to be looking after the patients to take their treatment.

I: Mmm.

P: That part is done but we are also- if possible, help out with the trach and tracing because now there are patients who do not have addresses, to trace those patients physically it, it, it’s so difficult.

I: Okay.

P: So, if maybe it could be improved a little bit, I don’t know how-

I: Mmm.

P: But like if you can, what you call it, the, it’s something like uhh that could give the address, give the position of someone, where someone is.

I: Mmm.

P: What we call it, *yah* [yes] then, it will be able to, to help out with the track and tracers now, those people like WOBOT, those who are going out to track the patient.

I: Mmm.

P: It will give them the, the point where the people, the patients are and then it would be easy for them to, to, to get to the, that, to the patient, especially those patients who don’t even have addresses.

I: Okay.

P: Who are living in the streets.

I: Yes. Okay, that, that’s noted. So, can you tell me about the training and the resources you received before we started delivering the Digital Adherence Technology?

P: The training, I think we had a sufficient training.

I: Okay.

P: That was given by Aurum.

I: Yes

P: On how to, what it is and how, how to operate it, how, how- what is the, the, the aim and how to, to, to operate the platform.

I: Mmm.

P: And what is the expectation and how to, to, to manage patients on, who’s on, on, on, on, on using the smart box.

I: Where were you trained?

P: Mmm one of the-

I: Was it here at the clinic or-

P: No, it was-

I: A mass training?

P: It was a mass training, *yah* [yes]

I: Okay.

P: *Yah* [yes] we were called out to the, to the mass training.

I: Okay, so-

P: But then it was also a continuous at a clinic level.

I: Mmm.

P: There was somebody who’s, who’s visiting and who would be able to answer some questions that are still maybe a little bit vague.

I: Mmm.

P: And then give us even more information on, on, ongoing.

I: Who is that somebody, from where?

P: From Aurum, from Aurum *yah* [yes]

I: Alright. so, do you think the uhh, training was sufficient uhh, both maybe the mass and also the on-going, do you think it’s sufficient?

P: From my side, *yah* [yes] because I uh- from my side *yah* [yes], it was sufficient because I don’t have any-

I: Okay

P: *Yah* [yes]

I: Going forward, do you have any suggestions on how the trainings can be improved uh, maybe the duration or the frequency or uhh, who do you think should attend these trainings?

P: Huh, I think uhm what it, what would be most important is to, all the clinicians need to attend training because uh once someone is off, absent from work-

I: Mmm.

P: There must be somebody always- to be able, to not to wait for that person who’s absent or who’s on leave. There must be always somebody who’ll be, who will be able to manage-

I: Mmm.

P: Huh pa- TB patients especially those who are- because everybody I believe would be on-

I: Mmm.

P: On, on, on the same platform, so, everybody must be able to, to, to operate the platform that is now- everybody must, must be able, every clinician I mean must be able to, to operate that is must be computer literate-

I: Mmm.

P: To be able to enter to the platform and operate with the platform.

I: Mmm.

P: And understand the overall of the platform and the smart box.

I: Okay.

P: And the continuous counselling of the patient. That it should be to every, should be extended to all the clinicians who, who are interested in managing TB.

I: Okay. And uh how long do you think the training should be?

P: To me, it there was sufficient but others- to others maybe 2 days *yah* [yes]

I: Okay.

P: There is sufficient.

I: Okay. And the content?

P: The content was great, was enough.

I: Mmm.

P: I don’t think it-

I: Okay

P: Need more on the content.

I: Alright.

P: Because some, some are already, you know DOT when we, when we are, when we started enrolling out the DOT programme it’s not too different from the DOT program. It’s most of the things are, the only difference is that this one is digital, it’s not like physical.

I: Okay.

P: But once you understand, once you are trained in TB management and then it’s so easy to adapt to, to the, the digital.

I: Okay.

P: *Yah* [yes]

I: (……) So, from your perspective as a health care worker, can you tell me the benefits of uh, the box first and also the differentiated model of care which is follow up visits, what are the benefits of those two?

P: (……) Fo- let me start with the follow up visit, I think with follow up visits it’s so easy for- we don’t have to worry more about appointments of the patient because now box itself, it would remind a patient before the date-

I: Mmm.

P: That on this date you have to go and refill, so that part is done.

I: Mmm.

P: And it’s a relief to, to, to, to be struggling with appointments

I: Mmm.

P: Even if the patient is illiterate, it’s just a good thing to, to, to be there.

I: Okay.

P: And then uhh, when the patient comes to the clinic, the app- you know, the application for me it’s easy to operate, to change the appointment, refill the box, can even extend, give extra two months on them as long as the treatment you are issuing fit the box.

I: Mmm.

P: And we also have the-on follow ups even the, the, the pharmacy assistant can also do that, it, it’s user friendly.

I: Mmm.

P: Because we also have pharmacy assistant at this facility who went to the training.

I: Okay

P: Who knows how, when, when, how to refill and what to do when refilling.

I: Oh, interesting.

P: *Yah* [yes]

I: So uhh, can you tell me about the tasks list, tasks list?

P: Tasks list-

I: It’s on the everwell platform. Do you know about it?

P: *Yah* [yes] *yah* [yes] task *yah* [yes] I know about it uh, it’s very important that we, we, we start looking at, at a tasks list. Tasks list is where we normally check the, the missed, missed, missed appointments-

I: Okay.

P: Especially missed doses.

I: Mmm.

P: When you open it, it tells immediately whether the patient has missed dose or not missed any doses.

I: Mmm.

P: And then we do our follow ups on that.

I: Okay. And uh, can you comment on the relationship with patients, how it has been impacted by the digital adherence technology, has it changed the way you relate to your, your patients?

P: Mmm most patients would like to keep the smart box forever.

I: Mmm.

P: What happens is, it also advantage the patient by safe keeping the medication, especially those who do not have a nice place to stay. So, their medication are safe in the box-

I: Yes.

P: And then uhh, *yah* [yes] I think mostly all the patients that I’ve, I’ve been in contact with, are happy about it. I, up to so far there was no complaint from the patients to me about using the smart box.

I: And then the way you follow up with them, how has it impacted your relationship with them when you follow up with the phone call or a home visit?

P: Home visit uhh, we did not have to do the home visit because we didn’t have patients that we have to, you know-

I: Mmm.

P: We have to uh do a physical follow up.

I: Okay

P: But those who come to the clinic, time saving, it’s easy-

I: Mmm.

P: To oper-, they, they, they do not have to wait a long time to, to be, to be, to refill their treatment or-

I: Mmm.

P: For their review.

I: Mmm.

P: *Yah* [yes] it’s very easy, it’s fast mmm.

I: Okay.

P: *Yah* [yes]

I: Can you tell me uh any challenges you have had with this digital adherence technology like the box itself and also the follow ups, what are the challenges with them?

P: I might- I cannot say it’s a challenge, but it’s only when the battery dies and the patients has to come to, to, to, to recharge the battery but not really a problem because we don’t have enough chargers to give to the patients to charge at their own space but we do recharge the battery while the patient is waiting and then uh, if possible just change the battery and give the full battery while we remain with the old battery to recharging it.

I: Okay

P: So, it’s not really a challenge.

I: Okay

P: Mmm.

I: And in terms of follow ups, what are the challenges with making phone calls and also home visits?

P: We, we do not have to make phone calls and home visits because patients will be here-

I: Mmm.

P: Huh, will be reminded by the smart box to, to come to the clinic, will be reminded day before-

I: Mmm.

P: To come to the clinic, so up to so far, we do not have problems with following up the patients, phoning them-

I: Mmm.

P: “You have to come to the clinic,” we do not do that, it saves a lot.

I: Mmm.

P: It saves a lot of time to, to be doing that, to be following up on patients because patients will be reminded, and the patient will come to the clinic.

I: Okay. And let’s say you see red blocks on the calendar- on the platform and you have to make a phone call, have you had any challenges with, with that?

P: No, since we have assistants that will be doing that-

I: Mmm.

P: No, no challenges at all because they will just call-

I: Mmm.

P: And then the patient will be, will explain-

I: Mmm.

P: Like the ones that we have “no I was admitted in hospital” just for a, for example, we had the one patient that was admitted in hospital-

I: Mmm.

P: And then the, the, the box were red-

I: Mmm (yes).

P: But to find out that the patient left the box at home because was in a- it was an emergency-

I: Mmm.

P: Patient was- you know, confused and all that and then did not take a box-

I: Mmm.

P: When ambulance took the patient to the hospital but did tell them at the hospital that he is taking treatment and all that. And when we phoned after I think two days-

I: Mmm.

P: Then the patient told us that he was admitted in hospital xxx [hospital name].

I: Mmm.

P: And then left the box at home-

I: Mmm (yes).

P: And then we recorded in the, where we should record that no the patient is now admitted and continuing with the treatment, but the box is left at home.

I: Okay.

P: So, really that’s not, that’s not a challenge.

I: *Yah* [yes] that’s a success story of, of a follow up.

P: *Yah* [yes]

I: Yes. So, are you able to get through all the patients you call?

P: Some do not have phones if we have to call.

I: Mmm.

P: Some do not have phones at all. That’s where now it, it, it’s would be easy if at least we had a donning some not even just a donning but just GPS like.

I: Mmm.

P: That will tell us the physical, physical uhm pa- physical place where the patient is, physical address because some of them do not have address *yah* [yes], but physical address.

I: Mmm.

P: Then when we send- when we initiate track and trace it will help make it easy for the track and tracers to reach the place, the patient’s position. But we do not have that problem.

I: Mmm.

P: I’m just saying in future if we have, may have that problem, it will be helpful if we have something like a GPS to help us out there because most of our patients especially in our area-

I: Mmm.

P: Do not have addresses, do not have- homeless some of them.

I: Mmm.

P: And some of them do stay somewhere, they do not even know their addresses where they are staying. And they would just tell you just for the sake of opening a file, addresses that they do not even know it belongs to me, they give me my address-

I: Mmm.

P: Not knowing.

I: [Laugh]

P: [Laugh] *Yah* [yes] but then now, that’s when something like a GPS that will be able to tell us where the patient is. It would be very, very, very advantageous.

I: Okay.

P: If it’s added to the, to the smart box.

I: Okay. So, have you had patients who refused to take the box?

P: Only one patient, yes but uh, my understanding- what the patient did not understand exactly and did not give us enough time to, to, to, to explain.

I: Okay.

P: He was right out just like being negative or somebody who was just negative and did not want to hear what, what we explaining-

I: Mmm.

P: To, to, to her.

I: Do you probably know why he was like that, why he had that attitude?

P: Oh, possible it’s just personal things.

I: Personal things?

P: *Yah* [yes] it’s not like something picking up from the facility over there-

I: Mmm.

P: It was just-

I: And then have you had any report about stigma related to the box?

P: No, because this, this box doesn’t have any (……) any look what is inside.

I: Okay.

P: Huh, so *yah* [yes] not, not, up to so far there is no, no problem about stigma

I: Okay.

P: With the box.

I: And from your perspective as a professional nurse, do you think the box and the differentiated model of care which are these follow ups uh are going to improve TB treatment?

P: *Yah* [yes] I think with this technology-

I: Mmm.

P: [Clicking tongue] once the, the adherence is good-

I: Mmm.

P: That’s because now adherence is backbone-

I: Mmm.

P: Of TB treatment success

I: Yes.

P: Once we get that right, then TB will be gone.

I: Mmm.

P: Before we know it.

I: Okay, that’s true. So, you mentioned that the, you used to do DOT, can you elaborate more on the differences now between the way you were monitoring patients with DOT and how you are doing it now with digital adherence technologies?

P: Huh, DOT patients has to come to the clinic on daily basis-

I: Mmm.

P: And then those who could not come because of physical disability uh home visit has to be done, one person has to go there and ensure that the person is taking treatment.

I: Mmm.

P: Those who can come to the clinic, they would come to the clinic, but then now it’s a lot of time and manpower, waste of manpower there-

I: Mmm.

P: To someone to- everybody on daily basis to go and visit the patient just to give the treatment

I: Mmm.

P: That- and then those who have to come to the clinic instead of going out there to, to, to look for work-

I: Mmm.

P: And then those who are doing what we call uh, recycling, they wake up very early in the morning starting to recycle around 5 o’clock in the morning and then how is the person going to the clinic half past seven when the clinic opens-

I: Mmm.

P: Again, to come and collect treatment, that was a burden.

I: Mmm.

P: So, the, on the other hand, the digital technology has changed all that.

I: Mmm.

P: Now we monitor the patient, we, remotely.

I: Mmm.

P: The patients doesn’t have to come to the clinic for the treatment.

I: Mmm.

P: And we also updated on daily basis about the movement of the patient taking the treatment or not. And then we can act quickly when the patient missed, start missing the doses. We can arrange, swiftly arrange for, for, for the, to return the patient back to the treatment, very early before it’s too late.

I: Okay. So, you have mentioned uh a lot of positive changes that have been brought by the digital adherence technology, how do you think these positive changes can be sustained, let’s say uhh in the absence of Aurum, there’s no intern, how can we maintain these positive changes at a facility level?

P: I think uhm that would be the National Government from there, the National Government adapting to the, to, to, to the programme.

I: Mmm.

P: And then ensure that procurement is in place by the National Government, I think. If that could be done that, the only problem would be the, the procurement.

I: Mmm.

P: Because it does need, it’s digitally needs to be, everything have to be updated all the time and then it needs a platform to work on

I: Mmm.

P: You need a box which uses a battery as well.

I: Yes

P: So those, those things will be needed uh, it could be only be sustained by the National Government, I think.

I: Okay

P: Because- otherwise maybe with the help of, you know, private-

I: Mmm.

P: Who are interested in seeing uhh, TB eradicated.

I: Okay, uhm do you have any negative changes you can think of, that have been brought by the box and how can they be addressed, for example, do you have any concerns about maybe patients opening the box without taking medication?

P: No, that could be, if that happens, that could be sorted out by counselling, proper counselling only, because now the patient has to be made understand through counselling, that it, it, it’s for, for, for the, the patient’s own good.

I: Mmm.

P: If you cheat then, you know, it’s not going to work.

I: Mmm.

P: It’s not for the, for the clinician.

I: Mmm.

P: It, but it, it, it’s for the, it’s good for the patient. So, the patient has to be made to understand even if the box is not there, if the person is going to cheat, he’s going to cheat.

I: Mmm.

P: It’s only good to use the box because we can monitor them.

I: Mmm.

P: We don’t have to call the patient to come to the clinic on daily basis.

I: Okay.

P: That’s very helpful and the patient will be reminded and then if they don’t want to be, to be bothered by an alarm-

I: Mmm.

P: It’s easy to-for those who will know when to take or use alternative alarm but still using the box, the alarm could be switched off, but the box will continue monitoring. I mean uhh, [clicking tongue] indicating that the patient is taking the treatment.

I: Okay.

P: And we can monitor that from, from the facility.

I: Okay.

P: Wherever the patient is.

I: Alright. and in terms of stigma, I know that you have not experienced any, but should there be any patient who uhh is concerned about stigma that’s why they leave the box behind, that’s why they don’t want to take uhh the box or they don’t want to accept the box. Huh, how do you think this can be addressed, this negative thing of now stigma attached to the box can be addressed?

P: Huh, I uhm, the box can also be uhm what you call it now, remodelled-

I: Mmm.

P: To make it something different.

I: Okay

P: *Yah* [yes] and then again, we can also use uh pocket, paper pocket because we have been using paper pocket, we have them, we have been using uhh pocket but-

I: Mmm.

P: Pocket to put in the box.

I: Okay.

P: So that when they walk out, they don’t have to, to attract- because we know that it’s a stigma. So, people be looking what is he having, why I’m not having it.

I: Mmm.

P: They will also wish to know, you understand, what is happening with that, the, the box, you understand.

I: Okay. So, you suggest uh giving patients something to, to, to maybe hide the box.

P: That will put inside because you cannot walk out, it’s like walking out from the shop with the loaf of bread-

I: Okay.

P: Everybody knows it’s bread-

I: Mmm.

P: But you know if you walk around, it’s something else, just to, you know, the, so *yah* [yes]

I: Okay. So, can you let us know what system level structures need to be improved in order to integrate this digital adherence technology and the differentiated model of care in the existing TB programme, right, so, what needs to be in place for, for this project which is currently being run by Aurum to be integrated into the Department of Health activities, so it becomes part of the daily routine ?

P: What needs to be in place?

I: Yes, yes.

P: I think uh, my understanding is that it is already, we are, we are already moving towards paperless uhh, consultations now like E-health.

I: Mmm.

P: We are not going to use paper files, we are going to use, we are going digital, it’s already been started and enrolled out at other clinics.

I: Mmm.

P: So, the digital technology came at a right time because it just has to be integrated-

I: Mmm.

P: With everything there-

I: Mmm.

P: And then as it is, it’s like it has everything, it has a file of the patient just like it be structured with the paper file it’s structured. It is structured like it has almost everything there.

I: Mmm.

P: It has where we have, where we need to put information that we-

I: Mmm.

P: It just have to be connected to, to, to the E-health that is being enrolled out.

I: Okay

P: Then once it’s collected, we, we, we are going to, to need it anywhere.

I: Mmm.

P: With the E, E-file, not E-filing but the E-health-

I: Mmm.

P: Where we use paperless, no more uh paper files and all that stuff where we going to consult on, on the PC digitally, everything is going to be digitally.

I: Mmm.

P: So, it’s at the right time, we cannot go back now-

I: Mmm.

P: It has to continue.

I: Mmm.

P: Because going back now we cannot go back to DOT, you understand [laugh] when, when we have moved so far, when we have ad-, when we have advanced as much-

I: Yes.

P: Already. So, we are only waiting for the PC to be upgraded to, to, to, to consult on it-

I:Mmm.

P: And then uh digital adherence to be included there.

I: Mmm.

P: Then we have a complete set-

I: Okay.

P: Of consulting a patient, everything is there.

I: That, that’s interesting. And *yah* [yes] currently there is a lot of preparation that goes.

P: *Yah* [yes].

I: Huh, that happens before the box is actually issued, so going forward, who do you think should be responsible for preparing the boxes?

P: (……) huh, [background noise] like uh can you please rephrase or repeat the question.

I: Okay, so the uhm, currently Aurum is preparing boxes, right? Before they are issued to patients. So, once the Department takes over, what needs to be in place, who do you think should be responsible for preparation of boxes like charging and so on and so forth?

P: I think that could be done at, at any, every facility- facility could be, could be meant to do that.

I: Mmm.

P: Because it’s not a lot of, lot of work to do that.

I: Mmm.

P: To prepare that. As long as we have counselling, counsellors then, we can also use counsellors-

I: Counsellors

P: We have already HT-, HTS counsellors who could also be integrated

I: Mmm.

P: In helping out.

I: Okay.

P: When the, when the workload is too much.

I: Too much?

P: Too much.

I: Alright.

P: Because manpower, I don’t think manpower will be, will be a problem.

I: Okay.

P: In going forward with the, with the programme.

I: And then should there be let’s say a technical glitch or a technical issue with the box or Everwell, how do you think that can be resolved when it’s now within the Department?

P: Uhh I don’t, I think uh Aurum introduced the box and when it comes to technology of the, whether any, any problem uh about the box and the platform-

I: Mmm.

P: They are the ones who know how to operate it and how to go about it.

I: Okay.

P: I think they would be there in standby all the time.

I: Okay, but if they are not there, they handed over now to the Department, how do you think should, it should work, let’s say you have a challenge, you can’t log on to Everwell but Aurum is no longer in the picture?

P: I think there would be someone that we can contact-

I: Mmm.

P: And ask her, especially the tech- technician from, there would be someone, there would be someone in place.

I: Mmm.

P: Like any other platforms that are used like Tier.net there are always people there that we contact when we have uh glitches.

I: Okay.

P: Mmm.

I: That, that’s interesting. So, there’s an existing help desk you have for the existing programmes you are using?

P: The existing ones, *yah* [yes] there is.

I: Yes

P: This one also there would, there must be somebody who uhm *yah* [yes] it only depends on how we go about it and who that person.

I: Mmm.

P: From which side should the person be from, but there must be somebody there.

I: Okay.

P: Always, like we using uhm, SAP, there are always SAPs specialists, especially people who are just standby what, they are waiting for problems-

I: Mmm.

P: People to complain about SAPs that they do not attend to those problems they are called, SAPs specialists-

I: Yes

P: So that applications and platforms-

I: Mmm.

P: Always have people who knows how to, who were there to help manufacture them to programme, they are always there to, to help out.

I: Okay. So, do you have systems in place uh to monitor this uhm, digital adherence technology, the box and also the differentiated care like where you record any successes, where you record any challenges, you are having, do you have a system in place to the document, all those things?

P: Right now, we are using Tier.net. Tier.net is, is also doing almost structured almost like the platform where we are using for, for, for digital.

I: Mmm.

P: It’s only- it’s not digital, it’s not, the only thing it’s differs it’s not digital.

I: Mmm.

P: Yes, the Tier, the, the, the, the digital technology it’s, it’s digital.

I: Mmm.

P: Tier.net you have to do all the things manually.

I: Mmm.

P: Uh, it does monitor, it does also- can draw out report from it.

I: Mmm.

P: I think the platform can also do that because I’ve browsed to the platform, and I can see that it can also do the same.

I: Mmm.

P: It can also give us report, we can draw report from it, if it can just be moderated a bit.

I: Yes

P: Mmm.

I: Okay. So, should you have, let’s say you are having issue with Everwell platform, where do you reco-, document that today I have an issue? Are you currently doing that yourself?

P: No, not currently.

I: Okay

P: But that could be, it should be.

I: Mmm.

P: So that we monitor what if, what the glitches that we are having.

I: Mmm.

P: We, we- there should be monitored.

I: Mmm.

P: And then uhh attended to.

I: Yes, how do you think they can be best documented going forward (…...) these challenges?

P: If we have challenges?

I: Yes, yes.

P: It has to be Aurum, since Aurum is still on the, on the field. It has to be reported to them.

I: Okay.

P: We have to write down-

I: Mmm.

P: All the challenges that we are having.

I: Yes.

P: And then hand them over to, to Aurum-

I: Okay.

P: For now.

I: Okay. Alright. So, can you tell me any gaps which are currently existing in the way the digital adherence is currently being delivered and how they can be addressed? Are there any gaps you can think of and how they can be addressed to improve, let’s say maybe the way the box looks or the way the box operates or the way the platform works?

P: Huh, up to so far, the platform uh when I browse to the platform, I see there it has a lot of things there, things that we really need, except that it doesn’t, we can’t draw report from it, of which it could be easy to adjust there.

I: Yes.

P: Then on the box- I think what I’ve been doing. I I’ve been putting a sticker on medicines, medicine issue sticker, I think that could be some adjustment that we could make, to save that the medicine inside that box even if it a little bit inside the box.

I: Mmm.

P: Where you can put it and save the medication that is inside the box, it is this medication with the barcode on and the expiry date because that is very important.

I: Mmm.

P: And how the patients is taking, should take the treatment as a reminder.

I: Mmm.

P: To say I’m taking 5 tabs daily or 3 tabs daily.

I: Mmm.

P: And what’s more important, it’s type of medication that is inside the box there, where you can just put it in there.

I: Mmm.

P: Barcode which is important if anything happens about the medication and also the expiry date.

I: Mmm.

P: Just a small thing that’s not big, just something nice.

I: Mmm.

P: It could be inside the box, not even outside the box. It could be on the, on the lid of the box.

I: Huh, so you suggest additional information?

P: That information, *yah* [yes] because right now it doesn’t have any information what is inside the box. When you open it, it should say this is what is inside.

I: Mmm.

P: Mmm.

I: Thank you for the information. We have, we are reaching to the conclusion of our session, do you have- what are your final comments digital adherence technology as a whole where you have experienced them here at xxx [clinic name] clinic, what are your final words?

P: Huh, I think it, we are excited about it, *yah* [yes] and then we need it.

I: Mmm.

P: Because it, it, it, it’s advantageous, *yah* [yes] but we also need some, a little bit of improvement there, especially that, the GPS part even if I don’t know how but there could be something that is easy

I: Mmm.

P: That will just say “here is a patient” at this time, if I physical want to go to the patient and then I could reach the patient there.

I: Yes.

P: That will help the track and tracers when, whenever the patient doesn’t, for those patients who doesn’t have tele uhh, cell phone numbers. They only have the box and the box could just, the box could lead us to the patient.

I: Yes, yes. Okay, we have reached the end of our session uhh, the time is 12:48. Thank you very much for the information.

P: You’re welcome.
