# Supplementary material for: Acceptability of Digital Adherence Technologies to support people with drug-susceptible TB in South Africa
Source: PLoS One. 2025 Sep 24;20(9):e0332103. doi: 10.1371/journal.pone.0332103 (PMC12459780; doi:10.1371/journal.pone.0332103)
Supplement: S4 File — (ZIP) [file pone.0332103.s004.zip › S4 Transcripts/HCWs and Stakeholders/IDI 27_STK.docx]

**TRANSCRIPTION NOTATIONS**

| **Label Key** | **Meaning** |
| --- | --- |
| **I** | Start of each new utterance by the Interviewer |
| **P** | Start of each new utterance by the Participant |
| **N** | Note taker |
| **{ }** | Indicates that details were changed or pseudonyms were used to anonymise data |
| **( )** | Indicates the description provided to anonymise data |
| **XXX** | Words were omitted to anonymise data |
| **-** | Breaking into a sentence by the next speaker |
| **…** | Pause or drawn out words |
| **[ ]** | Indicates noise made, e.g. [laugh], [sigh], [pause] |
| ? | Beginning of utterance by unidentified speaker or questionable text |
| **[inaudible segment]** | Unclear section of the recording |

I: So, do we have huh permission to record you?

P: Yes.

I: Thank you. Date of the interview: xxxx (interview date). Location it's: XXX [name of the facility]. PID: It’s stakeholder 01. The time is: 9:50. The interview is being conducted by: XXX [name of the interviewer].

I: … [Paper sound] So, May you please tell me what your role and responsibility is in this office?

P: I’m responsible for control and management of Communicable Diseases in the city. That includes TB and many other communicable diseases, except the HIV.

I: Ok. How long have you been in this position?

P: Since xxxx (start date).

I: Ok, alright. So now I'd like to know what you know about the ASCENT project or tell us what you know about that?

P: It’s a project that is aimed at implementing new technologies to monitor the compliance of patients who are initiated on TB medications. The project assists to monitor if the patient is taking treatment as required.

I: Ok. So can you tell me more about the types of technologies that you know?

P: Ok, the one that was introduced and mainly used in XXX [name of facility] was the one called a pillbox. A pillbox has got a machinery and a software inside that uses a SIM card and there is a battery that operates it, and this box, communicate with a tablet that is kept at the facility by somebody who's monitoring to check if the patient is literally opening the box to take the medication.

I: Ok.

P: The time the patient opens up the box, the signal is sent to the tablet at the facility indicating that the patient has opened the box, so we take it that by that time the patient is taking the medication. Another one that was also discussed with the- the coordinators of this adherence support was the one with the numbers in the pill. The sheets, the pills- the sheet. There are numbers there. When the patient opens up and take the pill. There's a number that reflects there. So, expectation is for the patient to send that SMS to the facility. And then we'll look at the number to say yes, indeed, the patient opened the sheet, the tablet sheet and then indeed took out the medication, because the number corresponds with what we have to indicate that yes the patient opened the medication, the medication sheet to take out the medication.

I: Ok, alright interesting. So you mentioned that huh if the patients opens the box or sends the SMS, huh information is sent to the tablet at the clinic for the nurse to see, what would happen if they don't see that the patient has taking medication?

P: Sot, from the facility side or from the clinic side, if it doesn't reflect that the patient opened the pill box. Let’s talk about the pill box

I: Yes.

P: So, the TB nurse or the tracer from the facility will contact the patient because remember, the patient's clinical folder is with a clinic at that time. So, they will go into the clinical folder get the contacts of the patient, and then phone the patient to remind the patient to say please remember to take your medication, because from our side there's no record that you really took the medication. So, it's- it's serves to- to- to increase compliance by reminding the patient to take their medication.

I: Ok, and whose responsibility is it to make the phone call?

P: Number 1, because the clinics work in a team format. So I think it depends on the setup at the clinic. There is a TB nurse who's working in that unit, who's ensuring that the clinical information goes into the clinical record, even if the nurse did not phone the patient, maybe the support staff working with the sister phone the patient, but the information and the feedback will have to go to the sister who will document in the clinical record to say the patient was reminded to take their medication, patient opened the pill box, so the patient indeed took the medication. So it's a team effort it’s not like one person. They work as a team. But the nurse who's responsible in the TB section is the one who will allocate or will literally call the patient to get that information.

I: Ok. So you mentioned something interesting about support staff working with the Nurse, what are the titles of that kind of staff?

P: Well, working with the xxx (organisation name) for those facilities that were participating in this study, so they appointed people who were placed within those facilities to work with the facility to support the facility.

I: Ok.

P: I don't have their titles, but they are trained by the xxxx (organisation name) to say, this is what you supposed to do. And then when they get into the facility, they are orientated into their facilities to say, this is how we do things here in this facility, so that information from the xxxx (organisation name) and from the facility assist these people who are placed within the facility. To work with their facility.

I: Ok. So besides these interns from xxxx(organisation name), who else is supporting the nurses?

P: Mmm. We have also district supporting partners. One of them is xxxx (organisation name), they also have a supporting team placed within the facilities. Some of them they do the - we call it the tracing, It’s a tracking trace via the phone. Some of them are responsible even for capturing this information. On the system that is available in the clinics, like a Tier.Net system that assists also to get the report to get the feedback about the patient’s performance in the facility.

I: Alright, Interesting. Mmm so huh if the TB nurse or the support staff are not able to get hold of the patient via phone call. What happens?

P: In that case, then the clinics have outreach teams. So they will draw the report for this patient that they could not get hold of. That will include the contact details, addresses and so on, and give it to the outreach team. The coordinator for the outreach team will give it to a team that will literally visit the site, looking at the address to go and trace the patient at home. They will go there literally and knock on the door to say we're looking for this person, where is this person? So those are the outreach teams that the district is working with.

I: Ok. So are they the Department of Health outreach teams?

P: They are both, others belong to the Department of Health. Others belong to - to the district supporting partners within the district. For an example, we have the -from supporting district partners, the people who are doing literally visiting the patients at homes, from the department, we have those that we call ward based outreach teams. Those are based in the wards. That's why they're called award based. So it depends on where the patient is and then they will allocate the case to that specific coordinator for that ward and then they will do the visit

I: Ok.

P: We also have for local authority, we have a multi sectorial unit for AIDS. Those are the Peer educators who are doing the home visit for HIV related, STI related at their homes. We also have a good relationship with them because they already know these people because they are working in the community. We also utilize them to assist us to locate these patients that perhaps did not come back for follow up or are not picking up the phone and then they are missing the medication.

I: Ok- Ok, that's interesting. So does this differ per facility that some facilities have Department of Health based outreach teams, some have from district partners, so how does this work?

P: It does differ, because the situation in for example XXX [facility] is not the same. Let me give you an example. There are areas that we call elite areas, where we don't have the outreach teams in there, because the clinic is situated in a suburb with a high walls, big dogs and so on. For those areas. We don't have ward based outreach teams, but we utilize other mechanisms, like the phone calls and so on to get access there because even if you send a person it's going to be difficult for that person to go in number one. Number two, even the owners of the place might either be suspicious to say but who are you and so on. You see something like that. And we have those areas where we have those outreach teams in those areas. Most of the wards are covered by these outreach teams, either from the partners or from the department, but they are covered.

I: Ok. That's interesting. So what have been the challenges let's start with phone calls, following up with phone calls. What have been the reported challenges with that one?

P: For now, the problem that the facilities mainly reported, was that they receive these numbers they document, but when they make a call, either the response will be this number does not exist. Number one, or somebody will pick it up and say no, no, no, no, it's not me you're looking for, you phoned a wrong number. So meaning that obviously the number that was given is not the correct number. So those were the challenges that we are facing now with the calling and so on.

I: Ok. And then how have you tried to resolve that challenge?

P: From the program side, we have encouraged the facilities to have at least more than one number, more than one contact number: number one, and also to have contact details of a family or a friend, so that if we can’t get hold of this person, then we know that there is somebody that we can go to, then we try to go through that person to get assistance to say we're looking for this person, we can't get hold of this person. The number is off, or his number is not working.

I: Ok, alright. Thank you for that information. And what have been the challenges with home visits?

P: Most of the challenges that we received from the facilities, are that sometimes the address that the clinics has got in their record in the report is the wrong number or wrong address. The address is correct but there's no such a person staying at that address. I think this is one of the big big problems that we having. So the question is, why our patients are given us wrong addresses, because obviously it’s wrong. And number two, sometimes you get there, there’s nobody there, and you send the people for the second time. There's nobody. Either the people who are staying there are working and going early to work coming back late. There's nobody maybe children during the day, there is no one, or sometimes when we get there, and this is what was also reported that there is no body, but they have big dogs and so they can’t even go and try to knock. because if there are no dogs, and then the gate is not locked, they are able to go in and knock, maybe somebody's sleeping, maybe needs urgent attention in bed, but with all those dogs and so on and there's nobody who's responded, and it becomes difficult.

I: Ok. So now I'm just asking questions regarding to the DAT itself. So, when you first heard about the Digital Adherence Technology which I will refer to as DAT. What did you think about it, both the label and the box?

P: Well, we had an orientation. I think in December, we were called and they were displayed and explained to say how they work. To me, I was Impressed, because at the first go I thought this is good, because it’s going to assist us to monitor the patient easier than to call patient every day to come to the clinic or maybe the patient is not coming to the clinic, the patient says I will take medication at home on my own, and we’ll never know if the patient took the medication or not, but with this DAT, I think it’s excellent. This is what we wanted.

I: Ok. So back to the training you had in December 2020. Do you feel that the information was enough?

P: I think it was enough, remember we did not know anything about it, so whatever information that was shared with us. To me when I looked at it, I was happy because we we’re even given an opportunity to ask questions as they go along presenting. We were asking questions for clarity and so on. So to me the information was enough,

I: Ok.

P: Based on it was- it was the first contact with DAT system, so I was happy with it [laugh] I am talking for myself.

I: That’s good to hear.

P: Ok.

I: And huh who trained you?

P: Mmm, they were many, XXX [Trainer] I think is one of them [laugh] but XXX [Interviewer] were you not there?

I: [Laugh]

P: This face and the name. I think I know you my sister, but I think I know you. It was XXX [trainer] It was XXX [interviewer] I can’t remember the others, also a chair person, I think it was somebody also from province who was there to support from central office ne, I don’t know who, It’s a long time ago, remember I am getting old,

I: Yes.

P: But I think there were quiet a number even from the xxxx (organisation name), it was not only the 2 or 3 of you. There were also I think senior managers as well,

I: Yes

P: Either 1 or 2 sitting in the corner there.

I: Yes, yes. So do you have suggestions to improve the training going forward? Like maybe the duration of the training, the frequency, who should attend?

P: I think because that was basically an orientation to all, including programme managers. I think it was okay. I’m happy with it. And then again what they indicated from there after identifying the facilities, there was another training that was done at a local level,

I: Mmm.

P: At the implementation site, remember we are not in the clinic all of us. So there was another training there. I was not part of, it was specific for nurses and whoever that is managing the TB at that site, to me that training was okay, sufficient based on the fact that there was another one that was done at the facility, and then even the first training, I remember the facility managers were invited. For those clinics were invited in that training, and then there was another opportunity for them to be trained again on site with the clinicians, so to me I think it was okay.

I: Ok- ok. So going forward if we were to train for rollout or implementation. Who should be trained, who should be targeted?

P: I think the approach that the xxxx (organisation name) did was ok, number 1, The programme managers, the area managers, that means the big clinic managers need to be part of that and then from there going down now to the clinic site,

I: Yes.

P: My thinking is, if you’re going to separate the two to have the one that looks at the overall information session, it’s ok, it must include the managers of the clinic and then now going down to the implementation site, it’s ok, but suggestion is- I’m not sure how it was done because I’m not Part of the clinic, but suggestion is, train everybody- everybody in the clinic form the admin up to TB nurse, up to even those who are not doing TB, the reason is, because in the clinics they rotate and then if the project like this one ended now, if the department adopt it for- for use forever, obviously it’s going to be in facility for many many years and then when the nurses rotate and if this one was not part of the initial training that means this nurse will be forced to be trained by a nurse who was trained by you. So it’s better if you train everybody.

I: Yes

P: So that when they rotate this person will also remember some of the things, and then they can just get additional information from the person who was implementing, because after training when you implement everything dwells in your mind and you go back to your chronic medical dispensing whatever, then by the time you come back 3 months- 6months you can’t remember everything, but if you’ve been trained and somebody just take you through a crush course like, then things will be superb.

I: Ok. Thank you for that. So, from your perspective as a xxxx (position). Can you tell us the benefits of the Digital Adherence Technology? You can start with the benefits of the labels and the box?

P: Ok the … I’m not sure how to respond to the benefits of the box, except that it’s portable, it has got latest technology of communicating with the- the mother tablet or whatever gadget that the information goes to, and then again also with the- the pill containers, I did not see this one being used in XXX [facility] we were using the pill box in most of the clinics I’ve been to, the benefits are, it’s easy for us to monitor the patient at a distance, you don’t have to have a patient coming here, because the patient doesn’t have to travel all the way to come to the clinic

I: Mmm.

P: And the DAT also assists the clinic in terms of the crowding, because now if everybody will have to come here for this patient to be monitored and take treatment and we have many other sick patients, you see that exposure and the crowd. So if they are monitored at home and so on, at the comfort of their homes. So, to me it’s beneficial and also for the facility side they will be able to manage whatever headcount that is there, whereas they just give a call somebody to monitor on the tablet to see if this person is doing well, so in essence you doing more with less, you have the tablet, you do all this, monitor the patient at home and you can even draw a report from there.

I: Mmm.

P: So the benefit is that you can draw a report from there to say according to this XXX [patient] performance, get a report and then you communicate with the client or maybe when you report to the next person.

I: Ok. So do you feel that it had any impact on the relationship the TB staff has with patients?

P: Definitely it has, because remember during the counselling when patients are being diagnosed for the first time and are being taught to use the Digital Adherence Technology, for the patient is that they are being appreciative because they are being considered to be a value to be given something like that to use at home, number 1 ; to me it increase trusting and a good relationship between a patient and the nurse or the patient and the facility, number 2; patients now know that I don’t’ have to come here to say medication is finished yesterday, the day before and so on, because from the gadget you can monitor to say, the patient has already finished the medication and then you can prepare the medication and call the patient can you come for follow up we want to see you because already we are monitoring there, we’ve got a record to say the patient is now on day 10 on day 28 the patient must come back now for maybe sputum or collection and so on and so no. So, to me I think its ok.

I: Ok. So, can you tell me what have been the challenges with implementing the project? What have been the reported challenges?

P: Ok. The reported challenges, most we got from the clinics, but what we received when we were engaging with the coordinators is that sometimes from the clinic side. I think we were slow to try and recruit patients or some of the patients were reluctant to participate in the technology, the new technology, but to me that shouldn’t be something we can say we not going to go ahead with it because of patients who are refusing or maybe from our side as clinicians we are not really pushing patients or encouraging patients to participate, that will be a bit of a low down, but to me a lot of challenges- I don’t think we received from the programme side, maybe the clinic might have something they did not report, but from our side not really.

I: Ok. So you mentioned the issue of acceptability from patients, do you know what some of the reasons were for patients not to accept the technology?

I: Ok. So you mentioned the issue of acceptability from patients, do you know what some of the reasons were for patients not to accept the technology?

P: Huh ok, this is what was reported also for those few that didn’t participate, they felt that no-no I can’t be seen taking this container with me. I can’t be seen going around with this container and so on, I just wanted those small boxes with the tablet, but it’s just a small number, it’s just a small number, it’s not really huh-huh a high number of patients that will make us worry to say but will this ever work or not. because especially when more people appreciate and take it as an option then I think it’s ok. So, the only thing that we had was that other’s felt that they were not comfortable with carrying all those but it’s not really a big issue.

P: I think huh - this is now me talking. I think maybe others felt that they are being closely monitored, you know. Their privacy is being infringed by people knowing that you have taken medication or you have not taken medication, and it’s not about that, it’s about the outcome of you as a patient taking medication with the support from the clinic, that is why I’m saying everything goes back to the issue of the counselling and counselling and counselling, if the patient refuses to take it first, doesn’t mean that the patient is going to be discarded from further trying to issue DAT because it assists us to monitor them.

I: Ok, and then were there any reports of stigma?

P: I think the-the issue that I mentioned just now to say some patients don’t want to be seen carrying that is an issue of stigma. To say if you are carrying this you are being monitored, you have TB or something you see it’s an issue of stigma, but the issue of stigma also is addressed at counselling. So we trust that our clinicians are doing their utmost best to address the issue of stigma.

I: So, were there any homeless people or drug users who were given the technology from your knowledge?

P: No. That one, I don't know about that. We haven't received that report.

I: Ok- ok, and did you always have staff ready to go and do home visits after 4 days? Of not opening the box or not sending the SMS?

P: Yes, we have. We always have. Remember we mentioned the issue of ward based outreach team. We mentioned the issue of support from the district partners and also the team from AIDS unit, that are already on the ground in the communities, it’s quite a bit number, we've got more than 800 of them in the wards, going house to house. So we have all those.

I: Ok. You have the support.

P: We have the support.

I: Ok, and in terms of staff turn- over or staff rotation, can you comment on that in relation to the use of technology in the TB area?

P: Well, what I can say about staff rotation is that it’s good but what we need to be careful of is that whoever we place in that unit should be a person who's well orientated on how the system works. The initial orientation you remember I said that everybody in the facility should go through it, and then when there is a rotation, the person who's coming in should have a crush session with whoever was rendering a service in there not that somebody must come from outside to come and do. No, it can be done locally. And then from there, we'll take it from there. So staff rotation is ok, it’s Okay, it's good. But we need those people to be thoroughly orientated into the system.

I: Ok. Yes, as they hand over to the department?

P: Yes, that’s correct.

I. So, can you elaborate on the positive changes that have been brought by the Digital Adherence Technology?

P: What we have seen from the reports is that the compliance to medication has improved. Up to 90% of our clients are now complying with medication because of the Digital Technology.

I: What have been the positive changes brought by diffrentiated care?

P: Ok. The follow ups In terms of the phone calls, and so on,

I: Yes,

P: Ok. That one we haven’t received any problems from the facility, except where the phone numbers are wrong or addresses are wrong, but in relation to calling and so on. There was no problem from the facility. The problem would be maybe at the end, were we supposed to get the patient.

I: Mmm.

P: But in terms of the service from the facility, no it’s always available, it’s there.

I: Ok. You mentioned that huh adherence, compliance has improved. It's now over 90%. How were you measuring it before? How do you see the improvement? How can you compare this?

P: We did not physically monitor the compliance, but we were monitoring the outcome-

I: Mmm.

P: Of the whole medication treatment and so on.

I: Ok

P: So, for now for me to say it has improved it’s because it's been monitored closely by our partner xxxx (organisation name), the one who were engineering this and then they are sharing with us the reports on regular basis to say this is how far we are, these are the challenges. This is the clinic that’s still lacking behind in terms of the numbers. This is the clinic that is doing very well in the district and so on. So for now, in terms of monitoring the compliance, we were relying on our partner xxxx(organisation name) to give us the information.

I: Ok.

P: What are we doing now, we monitor only the outcome now, because outcome is the one that is documented in records, in reports to say this is the outcome of the that cohort that started medication on this date up to now.

I: Ok. So what could be improved at the level of the facility and the district or the TB program in general in order to maintain these positive changes like the good compliance?

P: I think what needs to be maintained is the regular feedback, but now your project has ended.

I: Yes, in the absence of xxxx (organisation name)?

P: In the absence of xxxx (organisation name). I think it's our responsibility as xxxx (position) to take the baiting and to continue to monitor and to support the patient, but now that needs approval from national. Remember, there is a data issue when the two systems are communicating. So the department expectation is department needs to support us with a provision of the data to continue with the system to continue with communicating with our clients because as a program, TB program, we don't have budget for data for the facilities to communicate with their client. But because xxxx (organisation name) assisted us with the data, the department needs to come in now to provide the data for the gadget to communicate to each other, all the clinics do have telephones to communicate with our clients, but the one that came also with this system also improved a lot, because at a glimpse you can see that the patient did not take medication and that needs the data to monitor.

I: Ok. And can you tell us about the negative changes that were brought by the technologies, if any? For example, were there- What are your perception or your experience or reports about patients who just let’s say open the box without having taken the medication?

P: Well, we haven’t received that report, to say sometimes the patient opens for an example, like you are saying the patient opens the box maybe just testing the system, because some patients sometimes they can just do it just to test the system to see if it's working. We haven’t received that report. So and I'm not saying there are no negative outcomes or something. But they haven't -they haven't come to our attention. If they are maybe the facilities might be having them but from my side, no, they haven’t reached our office.

I: Ok. and can you tell us system level structures that are needed to be improved in order for this to be implemented in the absence of xxxx (organisation name), in terms of implementing the technology and also the follow ups in terms of follow up SMS’s, phone calls and visits?

P: I think the issue is the facilities needs to look at their organizational structure. Remember when xxx (organisation name) came in, they came in with the support staff and the support staff made things easy for the clinic not to feel like this is extra work, because there is a person who was supporting and also the coordinators from the xxxx (organisation name) were supporting. So, maybe the system that needs to change is to look at the organizational structure and to have that extra pair of hands in the TB section and then I hope it will assist. So that can happen, it’s just that we need to submit the motivation. To say this is what we want. This is what we have seen, this is what we think we can adopt. And it will work magic for the department, and then we need to look at those structures and appoint those people,

I: Ok.

P: At the moment we have, it is not difficult to appoint people because we have but now it’s on the contract basis like EPWP Staff, they can be trained and so on. The unfortunate part is that after a year, then the contract ends, but we are able to get another batch, but now it's back to square one now with the training, orientations and so on.

I: What are EPWP?

P: So this is the unemployed people in our community, who are employed on a temporary basis. EPWP Is: Employee program- I can't remember everything, but because we used to EPWP. It’s a program for temporary employment for the community by the not only municipality or the department even xxxxx province and so on. They have those guys that are appointed for a temporary basis for a year just to relief you know poverty at home and so on. So, especially the youth and women are appointed through the program.

I: So, you're suggesting that those can also be supporting?

P: They can assist, because that one is easy to get. We have already the register of how many let's say for XXX [facility] How many, for which category, for which gender are not employed. So, on annual basis what the municipality does they use what they call filtering system, they run the names through the computer, them it randomly selects a certain number. Let's say Department of Health says we want 300 then will run and get 300. And then they will bring the list to us and other departments. So, in our case, in our case, it's easy to get them because we have them. The only challenge is regular training because after a year they are gone. Then you get another one, then you need somebody again, to train them to work with them until they are on par with what the system requires.

I: Ok- ok. So, can we discuss a bit more about the responsibility of each maybe staff member or the support staff who will be assisting with this part of program, because boxes needs to be charged and prepared. Someone needs to add the patient to the platform, someone needs to be monitoring and if there are technical errors, someone has to report and resolve them. So let's start with charging, who can be responsible for charging of boxes, preparation of the devices?

P: I think everyone because that is done at the clinic site, and the clinic is in a better position to respond to that.

I: Mmm

P: But to me you don't need technician or a professional nurse to do it, the support staff can be orientated and trained on how to do it,

I: Alright.

P: As much as the support staff are also the people who are assisting with capturing the patient information on our computer system, like a Tier system and so on. Those are the support -support staff. So we just need the support staff to do that.

I: Ok. And who should be monitoring the patient on the platform now?

I: Ok. And who should be monitoring the patient on the platform now?

P: That one is a responsibility of the clinician because it's the person who will ensure that the patient has got enough medication and the outcome of the patient is what we expected. The follow up date for maybe to come and collect the sputum and so on is the responsibility of the clinician or a nurse who's supposed to do it, but in the support of this support staff, because her alone she cannot do all these stuff.

I: Ok. So during the program huh, xxx (organisation name) was responsible for resolving any technical glitches that were happening. So should the department take over and there are technical glitches? How do you suggest those can be resolved?

P: We have the IT department that is responsible for all the IT equipment, Computers, Printers and so on, so to me if the department takes over even those gadgets. The running and so on. Those IT guys will have to come in and assist if there are those technical glitches, they can assist you to unblock all those things, because they are trained, they are they're already on the system. It's not that we need to appoint people for IT.

I: Mmm.

P: Unless if it’s something that does not need a speciality like IT. Then for an example. In our district, we have Health Information Management. They are working with the computers and so on, loading ensuring that the patient information is correct on the system, data is correct on the system and so on, so if maybe DAT system is giving those problems like the Tier. They are able to identify the problem, before even going to IT. They are able to identify the problem and then solve it. So we can utilize those guys because the department has got them, you can utilize the IT guys because the department has got them. We don't have to go and appoints other people for that duty.

I: So huh besides the support staff, what else is required for scale up?

P: Well it’s the equipment themselves. For now in XXX [facility] we have 18 facilities, and then if we want to scale it up we want to add far more, then we are going to need more of those boxes, more of those tablets or gadgets and so on, those are the other things we are going to require to reach far more clinics than the number that we have now.

I: And Is there may be a plan of how to require them. Where do you think they'll come from?

P: Definitely, as a primary health care we’ll have to write a the motivation to our management to say we need this and why do we need it, because we have the report to say this is effective there are improvements and so on. And then it’s that they would have to buy everything, once procured for the clinic, let’s say one tablet for the clinic can run several pill box messages. So you need just want one tablet and few of the boxes and then some of the boxes we'll have to procure and then those boxes at the end of the treatment phase for a patient, they can be kept back into the clinic and be utilized for the next patient. That’s why I’m saying that we can do it. Motivation, procure, the tablets we’ll procure,

I: Mmm

P: The pill box we’ll have to procure more, because one clinic might have maybe 10- 20 per clinic, but it's not that every year we'll need 20 because that 20 can be used next year and so on and so on. As long as it's regularly charged. Everything is re-ensuring that it’s working perfectly well.

I: Ok. That's good. Can you tell me do you have systems in place that you use to- to capture the progress for patients, where you're monitor or capture the challenges and successes with the use of the Digital Adherence Technologies?

P: We- we don't have as a program, but it's not difficult to edit because we have our assessment tools that we use regularly. When we are assessing how the facilities are doing. We can slot in some of the issues in that assessment tool. So that whenever we go to a facility to do the quality checks and so on, then we can add some of the questions in that assessment tool that becomes standard for everybody to use, and then that tool also assist us to draw the report in terms of what are the challenges, what are the prospects of failure here and what- what, because from that assessment tool, then we are able to draw that report. So we can- we can add that in our checklist,

I: Mmm

P: For now what we have in that same checklist that I'm talking about. We have a question to a facility, to say are you participating in a Digital Adherence Technology and who's supporting you. But now we did not unpack it further, because it was still under monitoring by the xxxx (organisation name) to offer assistance. But now because the term has ended, and then we can expand on that too to say are you participating, because I don't think all the clinics at a go will be or at that level.

I: Mmm,

P: So are you participating, they will follow up with the questions and, you know, requiring more to identify if there are gaps, maybe what is it that we can do as a program to support for success of the implementation of the program, because the success of the program leads to success of the treatment outcome of the patient.

I: Ok. Interesting. So, can you tell us any gaps that are existing in the way the intervention was done? Which can be improved.

P: Well, I'm not sure if there are any gaps, but what I think, this is what I only looked at late, is that when we implement something like this to monitor the adherence, it’s good. But the question is, what was the adherence level before the implementation? So that we go back and say adherence level, we were at 80 now at 90. And then another thing, you remember we had –the- the control facilities-

I: Mm-

P: They were 18,

I: Mmm.

P: Something that I think we should have done was to look at the performance of this control versus this one that had intervention and do the comparison. I don't remember seeing that, maybe it was done. I missed the meeting, but I don't remember seeing that because it was going to be very interesting to say where the facilities did not have a support of the DAT. This was their performance, where facilities had the support. These are the performance. Therefore, the implementation of DAT yields more positive results as compared to the control facilities that did not have the support staff, the DAT and so on. So I'm not sure if it was done, but I think if it wasn’t, we need to look at that in future maybe.

I: Ok, just to respond to you on that one. That was the whole point of randomizing and that's what we are looking at-

P: Ok-

I: Now with the results. So we are going to compare and provide you the results.

P: Ok.

I: And in terms of multi morbidities we understand most patients are taking more than TB treatment. What do you think about the use of DAT’s?

P: Most of the huh chronic medications are taken daily. TB treatment is taken daily. So it only depends if the daily we're talking about the evening or the morning but to me, it can work well, even for those patients who had other comorbidities for an example, if the patient is taking, let's say, hypertension, diabetes, whatever medication and then using the same system that we're using now, for TB, for compliance, it will effectively work even for those patients.

I: Mmm.

P: And then if all those patients are put under one umbrella, and we use the DAT, so that means it will also render positive results for these patients to monitor that this patient who's hypertensive is taking treatment regularly, and so on and so on. So that when they come back to the clinic for follow up when we measure their BP, their BP is not controlled, then it's going to be easy to check and say but you did not take treatment for 3-4 days. You didn't take treatment for 7 days, this is why maybe your BP is not be coming down [laugh].

I: Mmm

P: And then from there then the patient will say you know what, if I don't take my pills up and my BP goes up and at the clinic, they are able to pick it up, then that will also improve further compliance. So, to me it will work across the board, except only in the acute infections, acute treatment because it's a few days and so on. But for all the chronic diabetes, hypertension HIV, everybody, it will effectively work.

I: Ok .so do you have any suggestions for improving the box itself, the device, the box?

P: *Eish* I’m not sure, if we make it smaller, will it be able to keep those pill blister packs and so on. Because I was looking at it. It's okay. I don’t have a problem with it. But for others who say oh, I can't walk around with this.

I: Mmm

P: I thought maybe we can make it smaller, but now if you make it smaller, that means the packs for the medication must be smaller. So to me, I think it’s okay I love it. Maybe what we can consider is to give it nice colours. You know.

I: Mmm. What colours?

P: The unfortunate part is that you know with the gender, there are those who like the pinkish, there are those who like the bluish [laugh]. And if you make it bluish and you have more female, they’ll say now you give me the male one, maybe a colour that is neutral for both genders, make it colourful and so on. Even if they put it somewhere at home, so it's beautiful it matches with whatever they have at home.

I: Mmm- mmm

P: Let's just give it a nice multicolour something.

I: Mmm

P: Otherwise it’s perfect. I love it.

I: Ok, so how do you think we can address those afraid of stigma, who declined the box because of that? How can that be addressed?

P: I think that can be addressed from the facility side. When after doing the diagnosis, during the counselling-

I: Mmm

P: I think that's where it's supposed to be started. The emphasis should be on the compliance, than on the container by the nurse or whoever is doing the counselling.

I: Mmm

P: They must just support the patient, not the patient to be like what if they are looking at the box, but they must look at the ultimate goal.

I: Mmm

P: They must encourage the patient to say the ultimate goal of this box is 1234 and that is what the patient wants. Everybody who’s sick wants to be well again. So I think it goes back to the issue of the counselling and counselling and counselling from the facility side not to focus on the box, but to focus on the outcome,

I: Mmm

P: After taking the medication using the box, what is it that you want to achieve.

I: Ok, so you mentioned that we- we were having two technologies, the box and the labels for SMS. So can you compare the two technologies? Which one works better?

P: The- to me the one that works better is the pillbox.

I: Yes. Why do you say that?

P: Because it immediately gives a message to say a box is opened or not opened. The pill the client will have to not repeat the blister with the number. The client have to enter their number, so that one you don't have to enter anything, immediately when you open the message is gone. So it's less activity for a client to inform us that he or she opened the box and is taking medication as compared to that one that they open and they have to send the-the-the pin code or the number from the blister pack.

I: So, what were the other challenges with that one-off sending the SMS?

P: Huh, you remember I said I was not aware that there are facilities who are using that?

I: Mmm

P: So, I'm not sure if they have challenges, maybe the facilities will be better to respond.

I: Ok, that's fine.

P: I don't want to assume and yet maybe I'm wrong.

I: Ok. So what do you think could be the challenges, your perception now with the SMS method?

P: It could be number one, maybe the client might even forget to send the message, to send the code. They may send the wrong code. I think those are the things that I'm thinking about now. Because sometimes if we put ourselves in the boots of the patient, sometimes they just want to take their medication now and the bus is coming and so on, and I will send the SMS later. So, those are the things that I'm thinking about. With the box you just open and close then it’s done

I: And in terms of phone ownership. What are your thoughts on that as well?

P: The- the challenge with that is, that the department I don't think we'll be able to offer each and every one a phone, because people will end up using those phones for whatever reason. So when comparing with the box. The box is used for one thing only,

I: Mmm.

P: Even if they may put some of the medications that they did not get from us there, just to serve as a reminder when they take the TB medication it will also work for them.

I: Mmm-mmm

P: Because you cannot send the SMS to the friend or something, no, but that other one, I think the client might end up even replacing the SIM card-

I: Mmm.

P: With the SIM and do whatever they do.

I: And then the issue of load shedding with using that type of technology as well?

P: Remember they've got batteries for that-

I: Mmm.

P: I think it's only important that we emphasize the issue of charging all the time,

I: Yes.

P: because when charged load shedding is now 2 hours, maximum is 4 with this high level 6 whatever. So for –for- for those during the counselling, we have to indicate to them that please keep it charged all the time, so that when the load shedding come it will operate with a battery. You can still send a message, we can still see the notification that you opened the box.

I: Yes- yes. Ok. So we are about to wrap up. Can you comment if you're seeing any better outcomes ever since the implementation of the Digital Adherence started?

P: I checked the outcomes of the facilities that were- were using. I checked XXX [name of facilities who were using the [DAT] and so on. What I realized is that there is a slight improvement but now another thing that I looked at was the overall for the district. The overall for the district. There is also slight improvement, because I think the last time when we reported I think we were at 79 and so on, and the province was not happy that how can we be at 79. So I think I saw 89 or 80, something so there is a slight improvement. It's a good start, because we we’re not implementing this across all the facilities. It was only few facilities out of so many-

I: Mmm

P: And those few facilities even if you see an increase of 1- 2% it’s a good thing. Just imagine now if you roll it [DAT] across then you might have an improvement of 3 or 4% and 3 or 4% is a lot, because if you were at 79 and then you get 3 more percent, you are at 83. Next year, you push and push to get another 3%, you are at 86%. That will reach the 90% that we supposed to be. So there is a slight improvement. And I'm happy because it's only from few facilities. Not all the facilities.

.I: Yes. That's great to hear. And then ok before we wrap up just back on the training issue.

P: Mmm

I: If we hand over to the department, who should be the one- who do you think should be the one to train the support staff or the TB nurses everyone who will be working with the technology. whose responsibility should it be to train?

P: Ok. So for local authority, we have a training section, specifically for that. Not only that but for many other trainings, those are the people who will be responsible for training, but we work hand in hand with them as a program. Because quite often as a program we have specialist of a certain program and they are generalist, so when they are [inaudible segment]. We be part of them. So that we support, we support them.

I: Ok.

P: But in terms of organization, arranging for venues, getting people it’s their responsibility then we come in to support if there are issues and so on, program side we respond. So from local authority, we have that unit, specific unit responsible for training,

I: Ok.

P: Training nurses, doctors, everybody.

I: Ok. That’s interesting. So what are your final comments with regards to the Digital Adherence Technology implementation? What are your final comments?

P: [Laugh] I’m happy with it, I love it, because we seeing the results now and remember it’s only one year, with TB, you start now and you only measure the outcome after a year. So since 20 January, the 1st quarter, January, February, March the 2021, the outcome now is better than before, that’s why I’m saying we seeing a good result. I love it. The only thing is, we need to support and also from the program side we need to continue to support because, quite often the facilities when you leave you give us the report and you go to another district or province to do something they tend to relax, because now the people who were here it’s xxxx (organisation name) they are gone, no- no. So we have to continue to support as a program from our side, now I’m talking from our offices. We have to take that baiting and carry on with it.

I: Alright. Thank you very much for all the information, we have reached the end of our interview. The time is: 10:51

P: 10:51 from what time was it? [Laugh] Yes, my tea is cold.

GLOSSARY

*Eish*  (*Vernacular slang word expressing someone who speechless)*
