# Supplementary material for: Acceptability of Digital Adherence Technologies to support people with drug-susceptible TB in South Africa
Source: PLoS One. 2025 Sep 24;20(9):e0332103. doi: 10.1371/journal.pone.0332103 (PMC12459780; doi:10.1371/journal.pone.0332103)
Supplement: S4 File — (ZIP) [file pone.0332103.s004.zip › S4 Transcripts/HCWs and Stakeholders/IDI 28_ STK.docx]

**TRANSCRIPTION NOTATIONS**

| **Label Key** | **Meaning** |
| --- | --- |
| **I** | Start of each new utterance by the Interviewer |
| **P** | Start of each new utterance by the Participant |
| **N** | Note taker |
| **{ }** | Indicates that details were changed, or pseudonyms were used to anonymise data |
| **( )** | Indicates the description provided to anonymise data |
| **XXX** | Words were omitted to anonymise data |
| **-** | Breaking into a sentence by the next speaker |
| **…** | Pause or drawn-out words |
| **[ ]** | Indicates noise made, e.g. [laugh], [sigh], [pause] |
| ? | Beginning of utterance by unidentified speaker or questionable text |
| **[inaudible segment]** | Unclear section of the recording |

I: So, mam do you agree for us to interview you?

P: Yes.

I: [Noise] Date of the interview xxxxx (interview date), location xxx [location of interview]. The PID is stakeholder number 3, the time is 09H56 am. So, can you tell me what is the current title of your position?

P: I am a xxxx (position).

I: Okay, how long have you held this position for?

P: (.) Mmm, I started xxxx (start date).

I: xxx (year) okay, so what are your roles and responsibilities within the programme?

P: Mmm (.) is to supervise the-or maybe to ensure that the guidelines of the program are being followed in the facilities together with the management of patients-of TB patients- of HIV patients.

I: Okay, alright, so what do you know about the uh ASCENT study?

P: Mmm ASCENT study, as I said, I was not there in the training but when I was attending the feedback that they were giving…

I: Mmm.

P: Uh it was the study that was there to, Mmm my understand is to ensure that the patient comply with their treatment, taking treatment. So, they were given this pill box that they are supposed to use when they open, then the message was being send to the… I don’t know who was controlling it and then they will see that all patients have opened a box. They will be assured that the patient has taken the treatment uh.

I: And uh you mentioned the box- which other technology is being implemented by ASCENT besides the box?

P: Mmm other technology- am not sure what it was, but they were talking about a patient uh sending an SMS when they take their medication, to send an SMS to the… I don’t know maybe they were given the number…

I: Mmm.

P: that they were supposed to SMS, and then they will know that the patient has taken the treatment.

I: Okay, so how were the nurses knowing that take, treatment have been taken?

P: Uh there was a device that was kept in the facility. I think you had one of your… I don’t know who the lady, the one that were entering patients in the uh. So, I think they were checking on the device to know that the patient has taken medication.

I: Okay, okay, so if a patient who have not taken treatment, what is the way forward?

P: Mmm.

I: If they saw on the device that treatment was not taken?

P: Was not taken.

I: Yes

P: I think they would call the patient…

I: Yes

P: to find out what is happening, why the treatment was not taken. I think they were calling twice or three times and then from there maybe they will make a follow up, yes.

I: Yes, so, what was your role in this?

P: Mmm.

I: As the xxxx (position).

P: As a xxx (position) my role was to maybe encourage the TB nurses in the TB room to have as many patients as possible to enroll in the program.

I: Okay, alright and you mentioned that uh patients were called if they missed doses. Uh can you let me know who was calling those patients and who was also conducting. Let start with the calls, who was making the phone calls?

P: I think the people who are doing the- I don’t know who was controlling the gadget.

I: Mmm

P: Uh I think they are the ones or maybe there were other people who were doing it under them to call the patients and say you didn’t take the treatment.

I: Okay, alright who was responsible for home visits?

P: Uh with us, we have tracers, tracers that do home visits and the WBOT (ward-based primary health care outreach team), community healthcare nurses, community health care workers who we also send to do the physical trace of a patient if we can’t get hold of them on the phone.

I: So how do they work together with the facility-based staff like the nurses?

P: Usually what happens, the nurses uhm they will have the- let say whether they retrieve the files of patients that were supposed to come or maybe the patient that did not take the treatment maybe some days and then they will retrieve the file and then checking the file maybe they will start by calling, calling the patient. If there is no phone number or the phone is not answered and then they refer the patient to the WBOT. So, they invite those WBOT, the community healthcare workers, to give them the name of the patient and the address so that they should go to the address of the patient and find out what is happening, why the patient is not taking the treatment. And then we would ask them to ask the patient to come back to the facility so that they can continue with their treatment.

I: Okay, so these WBOT are from the department of health?

P: Yes, they are from the department of health.

I: Okay, only? Do you have support from other partners in that regard for tracers?

P: Mmm during that time there was support from (organization name) , we had also tracers that were tracing and they also providing the psychosocial support to the patients that got any challenges. Now we don’t have (organization name), so it only the WBOT and the nurse tracers sometimes. We do have- I think few in the district that are working in the sub-district for tracing.

I: So, when you first had about the pill boxes, the digital technology, the pill boxes, and the labels and SMSing…

P: Mmm.

I: What was your expectations?

P: Uhm my expectation was that uh maybe we will have maybe less patients that are defaulting the treatment, maybe it going to improve the adherence to the patients and improve our treatment success. Having more patients that completed their treatment.

I: So, has your opinion changed about that ever since the implementation has gone on?

P: It did help but even though it not so much like, uh the changes were not so much but in a certain way it did help to adhere to their treatment.

I: Okay, in what way did it help the patients?

P: [Laugh] no, some patients were adhering even now they get the call, and they will say I must take my treatment and maybe they will, I don’t know if they were setting reminders for them uh to take the treatment. So, the reminder- it was also reminding…

I: Huh.

P: not to forget their treatment, to take the treatment.

I: Okay, so you think that it helped the patients.

P: [Laugh] I should think so.

I: Okay, so did you attend any training?

P: No.

I: Uhh, alright, so when did you hear about the pill box?

P: Uhm, uh I say I started in 2021, March 2021. Uh that was the time I heard about it but not immediately because in the office I didn’t hear anything about that. I think the only time I heard about it was when XXX was having meetings with XXX.

I: Okay.

P: Uhh that when I heard there is this thing in the facility, because sometimes when you go to the facility they won’t tell you, maybe they assume that you know what is happening, but you are just coming in you don’t know what is happening uh.

I: Did you see this box at the facility?

P: Yes, I did.

I: Okay.

P: Mmm.

I: So, what was the feedback you got from the staff about it?

P: Uhm they were just saying no the patients are being monitored when they take their treatment. This is the box, once they open, then it sends a message to say the patient has opened the box, and then they assume that the patient has taken medication.

I: Yes, so from your perspective first as, as the xxxx (position), can you tell us maybe the benefits of uh the box and the uh the labels (.) to the patients and also maybe to the TB nurse as well? What was the benefit of this project?

P: I think the benefit might be that the patients are being reminded by the alarm on the box. There was a reminder for them to take the treatment and if they don’t take, they will be called. They will be maybe traced out, to know what is wrong with them, why did they not take treatment. If they have any problems they were being helped also maybe psychosocial support or whatever challenges that they will be having.

I: Okay, and then from the healthcare worker perspective, what have been the benefits of this technology?

P: Uh for them also, to see that there are patients that are taking their treatment, yes. Though I said that they- it was difficult to know exactly if the patient did take the treatment because it just the opening of the box that will send the message to say the treatment was taken.

I: But they were able to see or monitor?

P: Yes.

I: So, how were you monitoring patients before?

P: If they take their treatment?

I: Yes.

P: Uhm usually what was happening was that the patient would come to initiate them on treatment, they give them treatment, they explain, they educate them about the treatment every day. Uhh and then they were given the patients carry card, a green card every time they take treatment, and they will be told every time you take treatment you tick on this card. So, when they come the sister would check the card if all the days have been ticked…

I: Mmm.

P: That the treatment was taken and sometimes maybe they will ask them to bring it, if there is other remaining treatment so that they could do the pill count to see that okay, we gave you so much and you took many days, and this is left. And so, by that okay the patient has been taking treatment uh.

I: How else were patients monitored to make sure pill count and also the card check?

P: The patient carry card?

I: Yes, uh, was there DOT implementation?

P: Uhh DOT, uh because mostly the health care workers if we ask them, they will say they find that this patient maybe is having adherence problem. They will attach that patient to you. Community healthcare workers who are making sure that the patient is taking treatment visit the patient and then they will see whether the patient is swallowing depending on the time they go or maybe they will go and check the tablets and then they will take the green card of the patient but unfortunately, most of the patients are working, so even if they go there during the day or during the week, they won’t find them…

I: Mmm.

P: They are working uh, I think.

I: So how would you compare this technology to this uh, home based DOT?

P: Uhm another thing of DOT maybe they will bring a buddy or maybe a family member or a co-worker or whoever who supposed to monitor the patients that are taking treatment and support them, reminding them that this time for medication and take medication. So, almost the patients- it better those who are staying in town, they will tell you, no I stay alone or whatever they won’t give you…

I: Mmm.

P: Others don’t even tell their family member that they are on treatment, so that is the challenge also.

I: Yes, yes.

P: The other challenge of the community healthcare worker is that they go there to see the- some of them (patients) don’t want the healthcare worker to come to the house because the neighbors will see them. The healthcare workers put on the uniform, they see that somebody has been coming in out every day. The neighbors will be asking questions, what is happening in that house. So, others would not like to be visited.

I: Mmm, thank you for, for highlighting those challenges, so reflecting to those challenges you have just mentioned on this home-based DOT…

P: Mmm.

I: By community healthcare workers or family members?

P: Mmm, others DOT, the sister would tell the patient to come to the facility every day…

I: Mmm.

P: Depending on if they have got transport or maybe where they are staying is not far from the clinic, they will ask them to come to the facility every day to take treatment. So, the sister will make sure the patient comes, they will give them the treatment. They will give them water to drink the pills and then they will be the one ticking the green card…

I: Okay.

P: ….and that is another way of making sure the patients are taking treatment.

I: Okay and what are the challenges with that method of the patient going to the facilities?

P: The challenge is that if the patient is staying a bit far and they will say I don’t have transport to come here uh, I think that is the major challenge.

I: Okay, so having mentioned…

P: or patients that are working…

I: Yes, yes.

P: Uhm those wont able to come every day in the facility.

I: Yes, yes you have mentioned patients that are working who are not able to be found by community healthcare worker, should they go there, and they are not able to come to the facility. You have mentioned that some may not have transport money…

P: Yes.

I: to come to the facility and some patients don’t want to be visited due to stigma…

P: Yes.

I: Uhh so what do you think the benefit of this technology is looking at this challenge you have mentioned?

P: Uhh looking at the challenges, I think the pill box would be helpful to patients instead of DOT because they won’t be exposed to that kind of stigma and somebody coming to their place every time or maybe for them to come to the facility every time or maybe to take their medication so they just doing it in the comfort of their home as long as they open it somebody is monitoring that treatment has been taken.

I: Alright thank you, thank you for that information. Can you let us know the benefits of the differentiated care, so the differentiated care model are the actions that are taken should the patient not take medication for example the phone calls and the home visits, so what have been the benefits of the phone calls after looking at the platform and we see non-adherence what are the benefits of the platform of the phone calls?

P: The phone calls uh though some of the patients don’t want to take the medication and they don't answer their calls or maybe they give the call -the phone numbers that is not their own. They give maybe the family members, and you find that maybe when you call, they are not with the patient at that time you see, but for those who have their phone with them all the time I think it was helpful because looking at the reminder also to make sure they are taking their treatment, if they don’t take somebody else is monitoring them will call them what is happening and ask them to come to the facility and seem like that…

I: Okay.

P: I think uh.

I: Okay and uh what is the benefits of home visits?

P: Home visits uhh you are able to see the patients there and we are able to see their home environment. Do they have enough food? Do they have maybe the accommodation also in the house? How many are staying in the house. Is there enough ventilation in the house? They are able to assist those situations.

I: Okay.

P: Yes, and maybe if you find that no, maybe if let say there is nobody working in the house. Maybe refer them to the social development so that they should do something to help the patient.

I: Okay, so before the implementation of ASCENT when was phone call or a home visit given to a patient?

P: (.) Mmm a phone call would be when they don’t turn up for their appointment…

I: Yes.

P: Uh and then you call them, let say the patient was supposed to come today, today he didn’t come, tomorrow you supposed to call that patient and find out why they didn’t come…

I: Mmm.

P: you see others would say no yesterday I had this commitment, but I will come tomorrow or maybe, or I will come. They will tell you when they are supposed to come. Others would say I couldn’t come because I don’t have transport money things like that but usually was after they missed their appointment or maybe when the results come let say the patient was uhm a sputum for conversion and then maybe the result come they are still positive, we still need that patient to come back.

I: Mmm.

P: Then we call the patient, but it was not the calling of every day.

I: And with the use of technology you know, do you know where the call is made?

P: Uhm this technology?

I: Yes.

P: When the patient misses the dose on that day uh maybe they will call.

I: So, can you comment the different between waiting for the month to call the patient versus calling when you see they missed the dose?

P: Uh because the one waiting for a month is waste because we don’t know if they been taking the treatment when they go home. So, we don’t know whether they are taking or not, so the only time we would want to call them is when they have missed their appointment then you say ok there is something going on here let call this patient. So, if they are not taking the treatment all along or maybe for some few days a week wouldn’t know that…

I: Mmm.

P: Uh all you know is that the patient comes they miss their appointment and they come and then we check that ok the patient was not taking treatment by then it will be bit late because they missed for some days uh but then for this one that you call patient misses the dose today. You call and the patient answer and then, I think this one is better because the calling is done before the patient misses a lot of doses.

I: Okay.

P: Yes [phone ringing].

I: And uh what have been the challenges with the phone calls? You have mentioned that sometimes they don’t give correct numbers, or they don’t answer…

P: Yes.

I: What are some of the challenges you have with the phone calls?

P: Mmm for the facility sometimes if it the public facility you find that maybe most of the facility, they don’t have any landline anymore. Previously they used the landline to call the patient but now they are using cellphones. You find that maybe there are times or maybe the cellphone is used by so many people from mother to child- they want the cellphone, this one casualty, they want to call the ambulance whatever so sometimes you find that the time you want the phone to call maybe the phone is not available and then if you are busy you forgot to call that day maybe you will be reminded after a day or two or sometimes you allow maybe two days you are not on duty and the other one those who do not know the patient has to be called but from your side calling the patient. I don’t know if there will be any challenges I am assuming, I am assuming that you have got all the airtime and whatever and the phone is there any time you can call.

I: Okay, yes, uh thanks for highlighting the challenges within the program because the project came with the tablet and the phone but it also important for us to know that if that removed the situation like…

P: Yes.

I: And uh what are other challenges from the patient side with the phone calls?

P: Uhm from the patient side as I said maybe they don’t answer their phones and sometimes they gave the wrong numbers uh.

I: And uh in terms of the uh the loadshedding we are having, uh are there any issues with that, how does that affect making of phone calls?

P: Uhh by the time the study was done, I think there was no loadshedding to say okay it is affecting now I am not so sure how that is affecting the patient with the phone calls because the cellphone I think they need to be charged and then uh because some facilities are twenty hours maybe they charge when the electricity come back…

I: Mmm.

P: But then the one that maybe are eight hours I assume the sisters take their work phones home maybe they are charging them at home uh, so I don’t think there is…

I: Okay.

P: Much impact uh.

I: And then network?

P: Uhm network is also a challenge like the, the cellphones for the facility they are issued by the government so sometimes you find that maybe even the, the airtime is finished because they give limited airtime uh…

I: Mmm.

P: So, if it used for a long time maybe but before the end, they put airtime every month end maybe beginning of the month…

I: Mmm.

P: It supposed to last for the whole month even if it happens that it finishes before the end of the month then by that time waiting for the month to end there is no call that you can make from there. So, sometimes they tend to use their own phones, but it depends on individuals…

I: Mmm.

P: Others like their work that they can use their phones to make calls, like I am willing to call this patient, others will say, “no there is no airtime, there is no phone, I can’t call” and they will wait until the the airtime is loaded.

I: Okay and what has been the challenges with the home visits?

P: Home visits challenges are the wrong address uh they give the wrong address and then others also like uhm people that are staying in the CBD maybe clinics like [noise] Skinner, you find that also patients who are homeless don’t know where they stay, where to find them is a challenge. Some of them don’t even have uh even the phone themselves.

I: Mmm.

P: Uh I think that is the main challenge.

I: So, how does this box assist the homeless people?

P: Uh I am not even sure homeless people were part of the study; I am not so sure because there is difficultly uh especially if they don’t have a phone. How are you going [inaudible segment] the address also, there is no address and the address where you going to send this person.

I:Huh.

P:Mmm.

I: So, do all patients have a cellphone?

P: Not at all, others don’t have…

I: Okay.

P: It means those who don’t have a cellphone were excluded from the study…

I: Uh-huh.

P: Were they not excluded?

I: No.

P: Okay, how were they monitored if they don’t have a cellphone?

I: The box was communicating…

P: Okay.

I: To the platform.

P: Okay but when they miss, what has to be done, physical tracing?

I: Yes.

P: Okay.

I: And uh in terms of staff, staff turnover or rotation, what are your comments on that in terms of implementation because we trained some TB nurses…

P: Mmm.

I: And we understand they rotate, so what are [inaudible segment].

P: Uhh they do rotate, that one also is a challenge unless if the one is leaving the TB room trains the one who is coming in the TB room so that they know what is going on.

I: Okay.

P:Mmm.

I: So that is your recommendation?

P: I think so.

I: Alright.

P: There are also other facilities where they change every month or every three months. So usually, I ask the managers to say at least at the TB room can you please leave this person to stay at least for the whole year so that she can see how patients are progressing uh not three months, it would be a challenge to train every now and then.

I: Mmm.

P: Okay.

I: Yes, and uh were there any issues of acceptability that were reported to you of the technologies that the pill box or the labels were there any patient maybe who declined to participate?

P: Uhh that one I am not sure. I am not sure if they were any.

I: Okay.

P: Uhh of who declined.

I: And then acceptability from the healthcare worker side, were there any maybe TB nurses who felt it was not good uh?

P: Mmm as I say that when I arrived, I found already using it, so I don’t know when it started if they were others who said there were not happy about it.

I: Okay.

P: But also, you had your people in the facilities, so who were also helping to enroll these patients.

I: Yes.

P: Uhh so I don’t think for them they could have seen it as a more work for them because there was somebody who was helping.

I: Okay, alright so uh what were the challenges with implementing the labels this method of SMSs.

P: You know this one I am not so (.) so familiar with that I can explain what was happening, I just know that they were labels, so I don’t know exactly how it went.

I: Okay.

P: Uhh I am not so sure.

I: Okay, what do you think would be the challenges in implementing this method?

P: By the way, how was it working, what was it?

I: The patients were expected to send an SMS…

P: Uh everyday with their phones?

I: Yes.

P: Ok. (.) Uhh their airtime, were they agreeing to use their airtime to send SMSs or it was…

I: The SMSs were free.

P: Free SMSs okay.

I: But they were supposed to have positive airtime balance.

P: Mmm, its also a challenge uh for them to have a positive airtime balance, most of them you know don’t have airtime…

I: Mmm.

P: So they would say, “uh I didn’t send the message because I don’t have airtime” uh.

I: And then what are the challenges you can think of with the labels beside the positive airtime balance when you think of patient sending an SMSs with their phone every day after taking treatment

P: Some of them might forget to send the SMS sometimes.

I: Okay, and what were the challenges with implementation of the boxes (.) that you know of?

P: I can’t think of any.

I: Okay.

P: [laugh].

I: Were there any reports of technical glitches?

P: Like the, the thing of not working?

I: Mmm.

P: Not having the signal that the patient has opened?

I: Yes.

P: I am not sure of any.

I: You are not sure that’s fine, and uh do you always have staff available to do home visits four days after the patient have missed the dose?

P: Uhh in the facilities where we have the community healthcare workers, they are always there, they are always there unless if there are other… if the community healthcare worker is doing all the programs, they do the immunization, they do what, they do what. So, if there are not any other major events on the head calendar that is happening…

I: Mmm.

P: They are always available.

I: Okay, okay.

P:Mmm.

I: So, from your own perspective uh do you think the digital adherence technology can impact patient’s adherence?

P: Yes, in a way I think…

I: How so?

P: [laugh].

I: How so?

P: Uh, uh the fact that they get reminders for them to take treatment, I think that helps them adhere to treatment though we are not sure if they really take the treatment because they could just open and close the box [laugh].

I: So, you have that concern?

P: Uhh that is a major concern because they could just open and close if they don’t want to take medication even if they get the reminder. If I tell myself I don’t want to take this treatment. I will just open and close so that you shouldn’t call me uh [laugh].

I: Uh, yes, do you think this technology improved the relationship between the patient and the healthcare worker at the facility?

P: Uh it did because of this contact maybe communication with the patient uh the healthcare workers if they will be checking this patient everyday so unlike when you just give them medication for a month you go. Uh you will always see them when they come back but with this one [DAT] I think there was that contact communication with the patient.

I: And in terms of workload, what would be your comment regarding the healthcare workers workload using the technology reflecting on the challenges you mentioned maybe with DOT?

P: With?

I: With DOT.

P: With DOT, uh, they uhm… the challenges with the DOT most of the time it was for the WBOT to do- who are asked to go and supervise the patient. Isn’t the WBOT (ward-based primary health care outreach team) work with the area, they’ve got the area that they work…

I: Yes.

P: And it should be close to where they stay, and they are able to. Its their walking distance for them to go there. So, they should be able to do most of the patient taking treatment at that time…

I: Okay.

P: But then that one of the patients coming to the facility. It not so much of the workload for the nurses, the patient comes, they give the medication they drink, usually they would advise them to come in the morning before there is clinic visit, having a lot of patients so they attend them in the morning they leave the facility.

I: Okay, so was the technology uh a bit of workload for the patients- which can reduce for, for the healthcare workers -which reduces the working load or it increased their workload?

P: I am not sure how they saw it uh [laugh]

I: Okay, its fine and uh in terms of monitoring uh, did the technology improve the monitoring of patients?

P: Yes.

I: How so?

P: The reminders.

I: Uh okay, how?

P: And the phone calls.

I: Okay and can you let us know the positive changes that were brought by this technology?

P: Mmm (…) I can’t think of any [laugh]

I: Any positive changes?

P: Can’t think of any except the one communication with the patient.

I: Yes, yes.

P:Mmm.

I: So how can this be sustained in the absence of xxxx (implementing organization name), if xxxx (implementing organization name) is no longer there with the tablet uh with the intern and supporting, how can the positive that have been brought by the technology be sustained by department of health?

P: Mmm, uh, you mean the department buying the boxes or leaving the boxes behind [laugh] and then there will be somebody who would be monitoring?

I: Yes, yes, yes.

P: Uh because for the monitoring also you need the extra staff for doing that isn’t. TB nurse won’t be able to do that well…

I: Yes, Okay.

P: And that thing even the reminders for just the ordinary automatic from the box for the patient to take the medication, how does the reminder work?

I: Uh the box has an alarm.

P: Mmm it has an alarm okay, alright that one is fine uh.

I: So, what will be needed by the facilities, district or by national department for this program to continue. What are the requirements if xxxx (implementing organization) is no longer there?

P: Mmm.

I: You mentioned staff, what kind of staff would be needed?

P: Our thinking of the monitoring of the boxes when somebody will see that you didn’t take medication.

I: Yes.

P: I don’t know who is doing that now with xxxx (implementing organization) and do we need that extra person to be sitting somewhere to be observing this checking if the patient is taking treatment or what. Uhh I think that would come with extra staff to be monitoring for the whole let say for the whole district uh, I think but in the facilities I think the nurses already being used to it but then the staff rotation that you are talking about maybe that would be also another thing that would not help it to do it uh because if this one goes and the other one comes uh.

I: You mentioned something interesting about someone from the district. How would you imagine it to work if someone is monitoring at the district level?

P: [laugh] uh I don’t know maybe they need somebody that would be sitting in the office and having that technology, I don’t know what is it that you are using uh that would be checking that okay even knowing maybe the boxes would be telling you this box is open, this box has not been opened and then maybe that somebody also been informing the facility that this patient didn’t open the box can you please find out and somebody to phone, it means the facility has to phone. Those things need to be sorted out before.

I: Okay, so you are saying the monitoring can be done in the district level and then you inform the facilities to do the follow ups with the patients?

P: Uh that is what I am…

I: Okay.

P: I am thinking, I don’t know if it can work…

I: Yes.

P: Because maybe at some district levels we have got sub-district so like me I am responsible for a sub-district so maybe at sub-district level have someone who will be checking for the facility of that sub-district then will be able to admin all the sub-district have got somebody or maybe to have one in the district who will be doing that I am not sure.

I: That’s, that’s interesting and uh you mentioned staff, what else is needed in addition to staff for the program to continue?

P: The gadget uh the tablet uh and then the airtime also to be used all the time.

I: Mmm and then if there are technical glitches how would you think they would be resolved if the department has taken over?

P: No, it means xxxx (implementing organization) before they leave, they need to empower us this is what we do when they are taking treatment, this is what we do it, how we manage uh I think [laugh].

I: So do you monitor or capture challenges and successes of using the technology do you know if it captured?

P: Mmm captured where?

I: At a facility level, district level is there a way you are capturing the successes…

P: Not, not…

I: and challenges.

P: I am not aware of any.

I: Okay…

P: I don’t think they are capturing anywhere maybe the TB nurse would just say these are the challenges I am having, and I don’t think there is anyone who capture them to keep record uh.

I: So how can it be captured going forward with the department of health? Or is there an existing system that can be used that is being used for other things that can be modified to capture those challenges is there anything you can think of?

P: Mmm I am not sure

P: Because the system that we use for TB, and HIV. We got Tier.net that’s where we capture the patient uh but then I am not sure if this can be included in that I am not sure or maybe we need a different system.

I: Okay, so if you are having challenges, the facilities have challenges with the TB program how do they report those challenges? Do they document them anywhere?

P: Mmm challenges like what?

I: Any challenges that they can face with the patients or?

P: Uh usually if it the challenge from the patient they document those things in the patient file…

I: File okay.

P: Maybe they can inform us say okay this patient whatever they done.

I: Okay.

P: Mmm.

I: Alright uh can you tell us maybe any gaps in a way the intervention was done by the ASCENT project?

P: I think the main gap that I see when it comes to taking the treatment uh because you find that they are opening the box and the (nurses) are not sure if a patient has taken the treatment. So, that is the gap and I don’t know what can be done about it.

I: From your perspective do you think that happened that patients open the box without taking medication?

P: It might happen because there are those patients who stop taking TB treatment and they feel its not TB, it something else. You see if they believe that its not TB it something else probably, they will stop taking TB treatment…

I: You mentioned something interesting about beliefs, so can you elaborate more about beliefs that stop people taking treatment.

P: Uh if others maybe find that they are sick, they are taken by a family member lets go to the clinic they test they find TB or maybe they go there by themselves but then maybe the way they feel I don’t know at home somebody says no let go to a traditional healer so that they can see what is wrong with you. Then they go there, they find out something different from what they came for and they tell them you should stop treatment. “I am giving you this, go and use this one” and then they stop taking the TB treatment. They believe what the traditional healer has told them because that is what they believe more than what they were told in the clinic, uh that the situation.

I: Okay and you mentioned the traditional healers just someone believe, or they just believe, what else can stop patient to take treatment when using DAT?

P: Mmm with TB maybe sometimes when they come the first two weeks, they are very sick when they take treatment the first two weeks a month after that they feel better and the decide to stop treatment. I think I am better now I am not feeling what I was feeling before then they will stop, they will not understand that they are up for treatment for six months once the continue feeling better they stop.

I: And how do you think that can be resolved?

P: That one is through dedication telling the patient about TB on their initiation when they start the treatment, they need to explain the TB so that the patient can understand how does the medication work, how long does it take to finish treatment and what can happen if they don’t finish treatment even if they feel better, they have to continue with the treatment.

I: Okay and uh what other reasons… what are the other reasons that patient stops taking medication when using DAT?

P: (.) maybe side effects uh, they are experiencing side effects and they don’t come to the clinic to say no since I started taking the treatment, I am experiencing one, two, three as they decide to stop. You know these tablets they are making me feel this way and they decide to stop taking treatment uh.

I: So how do you think that problem can be resolved?

P: The same education in the beginning, you educate the patient about the side effect of the medication. You tell them you are going to experience one, two, three. And then if you do, please come to the facility and tell us and then we will find the way managing side effects but don’t stop the treatment.

I: Okay, and in terms of comorbidities, what do you think about the use of the box, patients who have TB and other diseases? Let say they have HIV and TB.

P: Mmm, I think the use of the box is good because of the reminder, it will help them to take other medication when they are taking the TB treatment. I think they will also remember the other treatment to take.

I: Yes.

P:Mmm.

I: Yes, okay, so do you have any suggestions to improve how the box looks?

P: How the box looks?

I: Yes.

P: Or how it works?

I: Yes, how it works or how it looks?

P: I don’t remember how it looks, I just know that it a box when you open it, there is some technology inside…

I: Yes.

P: Uhh so that one I don’t know.

I: And it white in colour…

P: Yes.

I: Do you have any comment on the colour?

P: The colour, I don’t think the colour has any…

I: Effect.

P: Uh I don’t know with the patient, but to me any colour will be fine.

I: Okay.

P:Mmm.

I: And then how it works, what are the suggestions to improve how it works?

P: No, the working is fine the way it is working

I:Mmm.

P: But uh the fact that we are not seeing the patient taking medication also is a concern. I don’t know how it can be uh solved or maybe put a bit of a camera in the box [laugh] I don’t know if that can happen that can also work, I don’t know.

I: Okay.

P: Mmm.

I: How do you think that will play out the camera with the box?

P: I don’t know when they open then maybe we are going to advise them. when you open don’t close it until you take your medication then the camera will be taking the photo how they are drinking the medication, I don’t know.

I: And who will see that?

P: The controller who is controlling maybe we will be able to see.

I: Okay [laugh].

P: [laugh].

I: That’s an interesting…

P: [laugh]

I: Uh, okay and what are your suggestions for improving the differentiated model of care. Meaning how are we calling patients after missing two doses, sending home visits after missing four or more visits how can that be improved?

P: Mmm I think that one it can be sustained the way it is, the calling uh. I think after missing two days and then the home visits uh.

I: It can be sustained?

P: Mmm.

I: Okay, so patients were also receiving automated SMSs if they miss the dose on the same day…

P: Mmm.

I: So, what are your comments on those uh reminder messages going to the patients phone every day, if they miss, if they don’t open the box on the time?

P: That one will depend on the individual, others will read the message or just ignore it. Others won’t even read, they will say, “uh it that message” even if you know the message an then maybe it won’t make them to take medication. It will depend on why they are not taking their treatment that day uh and that is also a good idea technology to use, it will depend maybe it might not work for everybody but it is also a good thing.

I: So, do you have any suggestion to improve the SMS method?

P: Mmm in what way [laugh] in what way I don’t know?

I: Okay, it is fine what are your overall comments about digital adherence technology?

P: No, the technology I think it is a good initiative to be done as I say that it, it is… it is helping in a way uh depending on a patient saying that maybe they will just take it or whatever depending on a resolution, but I think it a good technology. Uh it will be a nice if we continue with it beyond xxxx (implementing organization).

I: Okay.

P: Mmm.

I: Alright, thank you very much for your input for discussion. The time is 10H44 am.

P: Okay, no it was my plessure.
