# Supplementary material for: Acceptability of Digital Adherence Technologies to support people with drug-susceptible TB in South Africa
Source: PLoS One. 2025 Sep 24;20(9):e0332103. doi: 10.1371/journal.pone.0332103 (PMC12459780; doi:10.1371/journal.pone.0332103)
Supplement: S4 File — (ZIP) [file pone.0332103.s004.zip › S4 Transcripts/HCWs and Stakeholders/IDI 29_STK.docx]

**TRANSCRIPTION NOTATIONS**

| **Label Key** | **Meaning** |
| --- | --- |
| **I** | Start of each new utterance by the Interviewer |
| **P** | Start of each new utterance by the Participant |
| **N** | Note taker |
| **{ }** | Indicates that details were changed or pseudonyms were used to anonymise data |
| **( )** | Indicates the description provided to anonymise data |
| **XXX** | Words were omitted to anonymise data |
| **-** | Breaking into a sentence by the next speaker |
| **…** | Pause or drawn out words |
| **[ ]** | Indicates noise made, e.g. [laugh], [sigh], [pause] |
| ? | Beginning of utterance by unidentified speaker or questionable text |
| **[inaudible segment]** | Unclear section of the recording |

I: So, do we have permission to audio record you, mam?

P: Yes, you have permission.

I: Thank you, date of the interview xxxxx (interview date), the PID is stakeholder number 4, the location is xxx [location of the interview]. Uhm the language used is English, the time is 12H26. [shuffling] the interview conducted by XXX [interviewer’s name]. So, mam can you tell me what is the title of your current position?

P: Uhh I am an acting xxx [position] for xxxx [sub-district name]

I: How long have you been in this position?

P: For a xxxx (duration).

I: Okay.

P: xxxxx (months)

I: xxxx (months)?

P: Yes.

I: So, what are your roles and responsibilities?

P: Uhm I coordinate uh TB around all the xxxx (number) facilities in xxx [sub district name] including the mines and hospitals.

I: Okay, so I just want to know what do you know about ASCENT?

P: Uhm what do I know about ASCENT. ASCENT I know that is uh adherence uh- I don’t want to say club, but it is form of uh adherence support system that has been used in different clinics as a trial. We use a pill box to uh encourage patients to take their treatment on daily basis and at a certain time. Do you need me to explain how the box works?

I: Yes, please.

P: Uhh, okay, so uh the patient will come with the box to the facility. Okay, that when the patient already has the box but before then the facility will issue the patient with the box and then they will educate a patient on how the box works and then the patient will… and then the nurse will put the medication inside the box and close it and then the patient will go home with the box, at a certain time which was agreed upon by the patient and the nurse. The box will make a sound, let’s say for instance, they agreed on 08H00 and then at 08H00 the box will make a sound reminding the patient to go and open the box and take the treatment. Once the patient opens the box and take the treatment, then it clicks on the tablet that the nurse has at the clinic to show that the patient has taken treatment, yes.

I: Yes, thank you very much for the information, uh is there any other digital adherence technology besides the box?

P: Uhh there was, I think there was an SMS before I think you had three trials. It was the SMS- it was the box- I forgot the other one but then the box is the one that implemented right? Because the SMS couldn’t be implemented due to people not having data and etcetera and etcetera. Network loadshedding.

I: Yes, yes uh so can you tell us about your role within the differentiated model of care which means uh the home visits, phone calls, the follow ups that happens…

P: Okay, okay.

I: With patients?

P: Uhm I was not on patient care when it came to the box, but I was responsible for making sure that uh the data that is recorded on the tablet is also recorded on the Teir.net because we use Teir.net to monitor patient success again. So, I will make sure that the data that is on the tablet is also the same data that is on uh Teir.net and also it assisted us with our success rate.

I: Okay.

P: Yes.

I: So, you were mentioning about uh how the smart pill box works that uh TB nurses are informed that patients have taken medication…

P: Mmm.

I: What happens if the patient does not take medication?

P: If the patient does not take medication. I think on the nurse’s tablet, it makes a red indicator. I think to show that the patient did not take medication so the nurse would either call the patient to find out why the patient did not take the medication. There were times when the patient took the medication but left the box at home and went to visit a friend uh avoiding to go with the box to wherever the patient was going or just maybe the patient forgetting maybe the boxes inside the house the patient is sitting outside or uh, just as a reminder.

I: Mmm, okay, are there any challenges with the phone calls?

P: Uhm yes because sometimes when you know that this number is a nurse calling, you don’t answer it.

I: Mmm.

P: Uhh [laugh] I think that one of the challenges yes, but I say even if you don’t answer its still a reminder. The fact that you saw it but did not answer it as a reminder that you need to go and take medication.

I: Mmm.

P: Even if you missed the call the minute you see that I missed this call you remember that no I think they were calling me because they wanted me to take my medication.

I: Okay.

P: Mmm.

I: So, before the uh TB nurse makes the phone calls what are other reminder is there for the patient if they don’t open the box?

P: Mmm I have no idea.

I: Okay, do you know about the reminder SMS?

P:Uhh, uh the reminder SMS yes, the one they get on their phone. Yes, the reminder yes.

I: Okay.

P: Mmm I forgot about that one.

I: Alright so you mentioned that sometimes patients might leave the box behind when visiting a friend. Can you explain more why you think they would do that?

P: Because its big [laugh] its big and I think when you are holding it people will ask you what that is for because it uh it is a different box you can’t we are not familiar with this kind of a box so, uh, it grabs attention.

I: Oh okay

P: Yes.

I: And what wrong with being asked?

P: Okay, uh to me there is nothing wrong but then remember I am not the patient.

I: Mmm.

P: Yes, so maybe to the patient she doesn’t want or he doesn’t want to answer questions regarding their treatment and stuff because once you say its treatment, they will ask you for what and then uh.

I: Okay.

P: Mmm.

I: Uhm so you mentioned that one of the challenges with phone calls that they might not answer…

P: Mmm.

I: Because you see the… the healthcare workers number, what are the other challenges with phone calls?

P: Okay, I think that the other challenges with phone calls sometimes due to network remember now we have loadshedding. When you have loadshedding you don’t have network. So, it might not go through and then just sometimes the patient doesn’t have electricity the patient did not charge their phones so you might not be able to get the patient through telephonically yes, Mmm.

I: Okay, thanks…

P: Because sometimes they might have lost their phones and sometimes patients share phones remember uh then we are in a household we are five, but we only have one cellphone, yes.

I: Okay, uh, do they always give correct numbers?

P: No, absolutely not but most of TB patients I think they do give correct numbers uh.

I: Alright.

P: The only thing they don’t do, they don’t update…

I: Mmm.

P: They don’t update their numbers once they change them.

I: Mmm and then if the healthcare workers are not able to get hold of the patient through the phone calls what is the next step?

P: A home visit.

I: So, who conducts the home visits?

P: CHW (community health workers) with the OTL (Outreach team leader).

I: Okay.

P: Mmm.

I: So, uh, who are these CHW working for? Are they working for government or?

P: Uhm from department of health from the government. Every sub-district has OTL and CHW and every facility has OTL and CHW.

I: Okay, so what are the challenges of uh home visits?

P: Wrong address, they give us, they give them wrong address and sometimes they move without changing the address at the clinic and sometimes they don’t want to open the gate and.

I: Alright and then … you have mentioned challenges from the patient’s perspective, are there any challenges from the service provider, the healthcare worker’s side with conducting home visits. Are there available resources to conduct those home visits?

P: I think they are it is just that the problem is transport but then the transport is part of it. I don’t think it should be a problem per say because when they hire OTL they said uh one of the requirements was for you to have your own car uh so uh.

I: Can you tell me what OTL?

P: Uhh Jehovah I forgot out based team leader uh. Out based team leader uh.

I: Okay.

P: Mmm.

I: So how are the responsibilities shared between the TB nurse and the community of healthcare workers?

P: Okay the TB nurse uh would use Tier.net and also using this the… the pill box because now remember they are inter tasked if I may put it that way because once the patient miss taking treatment also they will be a miss in the Tier.net so they pull out any missed list uh and then they call the patient and then once they call the patient three times and there still, they have not reach uh the patient they will send they will take the list and give it to the OTL. The OTL is responsible for the CHW, so the OTL will give the list to the CHW to say because they are responsible for this site maybe let say for example xxxx [area name]. So, they are going to go look for this patient because according to the address this patient stays in xxx [area name] and then as a CHW that maybe uh works for xxxx [area name] and this patient stays in xxx [area name] uh please go and trace this patient for us and then some times the patient who will struggle with and then we might send the CHW with the OTL. Remember now there is one OTL but there is a lot of CHWs. So, if the CHW struggles with the patient then the OTL might accompany the CH- will actually accompany the CHW because now the OTL is the professional nurse remember to go and attend that certain patient Huh.

I: Alright, so uh where did you first hear about the digital adherence technology?

P:Uhh, Mmm training I think in where was it? Xxxxx [venue name] , yes.

I: Okay, so do you remember the training activities that were delivered?

P: Yes, yes, I remember they taught us about the pill box and then we had our own experience with it because we registered as dummies and then, uh we went through tablet and stuff to see how it works.

I: Okay, what was your first impression?

P: Uhm you know uh it is a good idea, it is. It is a good idea but is it feasible, is it? Like (.) is its long term?

I: What are your thoughts on that?

P:Uhh, uh, will it be long term. Like and when patients get exhausted from using it. The kids- I don’t know what they said but again even i they get used to it they outgrow it if I put it that way. But I think once they have outgrown it, they would have taken treatment by the time they have outgrown it because I do not think it will be right it just that trial and error for using TB treatment. Are we planning to implement it with all the other common treatments?

I: For now, its TB.

P: It because you know uh, I am just imagining let say for instance you are taking, because TB is six months after six months you are done uh but let say you are taking ART, ART is lifelong uh and then you use this pill box. I think you will use it for maximum of a year or six months and you get used to it I don’t think you can continue going forward but and then you will know that it will be in your system *kuthi* (that) I need that to take treatment at that certain time…

I: Mmm.

P: So, I don’t think it can be used as for long term, but I think it can be used as short term. Like to just uh to memorize patient taking treatment uh I think it good when it comes to that.

I: Okay, so you are anticipating that patients can have technology fatigue?

P: Yes.

I: Alright.

P: It is a long run yes, but I think once that fatigue is there they stop using but now they are used to taking treatment. So, I think it a start, it something to use to… you know how uh just to, to push them in the right direction of reminding them about treatment.

I: Okay, so can you explain where the… how will they get tired with just opening the box?

P: Uhh guys I don’t know with technology we can get tired. we use WhatsApp we get tired of it. We are deactivating our WhatsApp; we use Instagram we get tired of it anything that is technology you get tired. Like when buying a new air fryer…

I: Mmm.

P: You going to use it but at some point, you like uh I am tired let me just use the stove I don’t have the energy of opening this thing and you see, uh I think… but now you know where to run to when you need a certain thing.

I: Mmm.

P: Mmm.

I: Okay, that is interesting.

P:Mmm.

I: Uhm so back to the training, do you think it was comprehensive enough?

P: Uhh it was, it was. You mean the training from the pill box?

I: Yes, the training.

P: Mmm, uh.

I: And did you think it easy to implement?

P: The pill box? Very easy.

I: Yes, from the training.

P: It easy, it easy you put you pills in the box you open the box clicks there and [laugh].

I: Okay, can you comment on the efficiency that comes with the box?

P: Meaning?

I: Uhh the efficiency in monitoring TB patients.

P: Uhh okay, I think it good. It, it very good because you know why… you know with missing appointments you need to go to the system to Tier.net and pull out the list and give it to somebody but with this pill box you have it ready available within your tablet even if you are home you can still see *ukuth* (that) no XXX is not taking treatment.

I: Alright.

P: XXX is not taking treatment and you can attend to there and there.

I: So, do you have any suggestions to improve training for example we should train- uh how long should we do it?

P: I know, I think it good because as long as the TB nurse, the TB focal nurse and one extra person who goes into the TB room when the TB focal nurse is not on duty is trained. I think that’s ok.

I: Okay.

P: Uhh because I think even nurses the one that will tell you patient, right?

I: Mmm.

P:Mmm.

I: Okay, so in terms of who should attend you are saying the TB nurse?

P: Uhh the TB focal nurse.

I: Okay, alright.

P: Mmm.

I: So, are there staff rotations that happens with the facilities?

P: It depends on the facility, but we are encouraging every facility to have a TB focal nurse, but you know due to shortages and other staff it not possible.

I: Okay, alright so from uh your own perspective as the xxxx [position] Mmm can you describe the benefit uh a smart pill box has?

P: Okay, I think one of the things it promotes treatment compliance uh.

I: How so?

P: Especially since I have realized not all of them but most of our TB patients especially those who are not co-infected and also those who co-infected most of the time when you are diagnose with HIV and then you are tested for TB and then you start TB treatment. Meaning that you haven’t been at the clinic you only came to the clinic because you were sick and now all of a sudden you have to come to the clinic every month that is something new so you need somebody or anything to assist you in order for you to comply to treatment. The pill box it one of those things that remind you on a daily basis to do something that to you are not used to yes so that's why I was talking about treatment fatigue saying after six months even if you are tired of the box but you now familiar with taking treatment on daily basis so even if you don’t use the box but you will always remember.

I: Yes

P:Mmm.

I: Okay, and from the healthcare worker side what are the benefits?

P: Uhh they can monitor it only [laugh] and its every easy using the tablet Mmm, everything is already available…

I: Okay.

P: You don’t have to go and pick up the file from the file room and see *ukuthi* (that) uh *konje* (that) *ubani* *bani* (who and who) was supposed to come to the clinic on the 13^th^ and then now only to realize that the patient is not even taking treatment for the past ten days when you call them because they didn’t come on the written date that you gave them Mmm. You can monitor them on daily basis.

I: And uh in terms of the relationship with patients, can you please comment on how this uh technology has an impact on the relationship healthcare workers have with patients?

P: No, I can’t I have never asked them about it.

I: Okay.

P:Mmm.

I: Can you tell me uh the benefits of the differentiated model of care, you have touched on the benefits of the smart pill box and also of the platform the healthcare workers are using to monitor…

P: Mmm.

I: Now I want to understand from you the benefits of the follow ups which is the differentiated model of care for example the phone calls?

P: Uhh like the SMS?

I: Yes, and the phone calls and the home visits.

P: [Breath-in] uh you know- okay let me not use the blanket method but with the phone calls, I think for other patients it assists them because maybe really, they forgot to come to the clinic…

I: Mmm.

P: Uhh especially the young ones…

I: Okay.

P: Because something is new *vele* (obvious) you are bound to forget so it good but the ones who have been in the clinic for long time uh I don’t think so because I think now you set a reminder, like going to the clinic it part and parcel of your retrain so you know that on this day I am supposed to go to the clinic so uh…

I: Mmm.

P: *Akitsi* (I don’t know) others- it works for others it does not work; it depends, it depends on a patient honestly…

I: Okay.

P: And then we can not say we should stop it because it does not work for others while it works for others.

I: Mmm.

P: Uhh so it one thing that according to me you just continue with it moving forward.

I: Okay.

P: Mmm.

I: And then the reminder SMS, what your comment on that?

P: The reminder SMS, uh I think its good. The reminder SMS is good, I think you should continue with it because uh, it will remind you even with debt, it will remind you your debit order is due…

I: Okay.

P: On this certain date you should have enough money.

I: And the home visits what are the benefits of that?

P: Okay, I think the home visits uh … I think when you do home visits you should do home visits and give patient treatment at home…

I: Mmm.

P: Okay, I think the home visits uh … I think when you do home visits you should do home visits and give patient treatment at home and not just home visits to go and check out the patient at home, how they are doing and stuff because sometimes a patient is not able to come to the facility because they don’t have money  to come to the facility so when you do a home visit and then you go there with your treatment already if they are due for blood collection you collect blood already. If they are due for sputum collection you collect the sputum at home, then Mmm.

I: Okay, so can you describe uh the challenges of using the technology, were there any issues uh in terms of acceptability of this device that you may know of?

P: Mmm I am not really sure but what I know is that other patients don’t want it because its big and you can see it, they didn’t want to be asked…

I: Mmm

P: What this box for?

I: Okay, and uh were there any network issues?

P: Mmm not that I know of.

I: Okay and in terms of staff turnover and staff rotation. How did that uh affect implementation?

P: I have no idea.

I: Okay, uh and were there any concerns of stigma?

P: Yes, but on the patient not from the nurse to the patient but from the patient to going with the box at home uh.

I: Okay, and uh so and then was there also a group of people who struggled to implement it for example homeless patients or drug users?

P: Not that I know of.

I: Okay, and in terms of home visits, were there staff always available to conduct the home visits after four days of missed doses?

P: No.

I: Okay, what was the challenge of that…

P: The challenge is the transport and again the list of patients that we have to see we have to do home visits on daily basis so if maybe they might go after six days not four days.

I: Okay.

P:Mmm.

I: And all patients have cellphones?

P: No.

I: So how do you manage…

P: They will give you the next of kins number.

I: Okay.

P:Mmm.

I: So, how is that following up using the next of kins number?

P: Uhh you will call and then someone will say no she is here, or she is not here. He left for town and, uh.

I: Is that effective?

P: That's we have uh [laugh].

I: Okay.

P: Think the rule is always having uh… it better to have a number then to not have one.

I: Yes.

P: Mmm.

I:Uhh, so from your perspective as the stakeholder, can TB treatment be improved using this technology?

P: Yes, it can.

I: How so?

P: Like I was saying earlier on that where most patient who started on TB treatment they have never been in the facility before so it their first time ever being on treatment and being required to take treatment on daily basis so this will assist them as a reminder.

I: Okay.

P: Mmm as a daily reminder to say no you need to get used to the treatment.

I: Okay.

P: Uhm especially for those who don’t have what do we call them support partners uh.

I: So, you mentioned earlier that it helps with treatment success..

P: Success rate yes.

I: Can you elaborate more on that.

P: It helps with success rate uh meaning that uh all the patients, most of the patients who are using the pill box complete treatment, yes most of them complete treatment. I think, what where are we standing in Rustenburg in the facilities that new implemented. What was your success rate?

I: In terms of adherence?

P:Mmm.

I: We are above 80.

P:Mmm.

I: So, is there a different in terms of TB before intervention and after intervention?

P: Uh I will be lying but I think there is.

I: Okay.

P: Based on my success rate, based on how the sub-district doing…

I: Mmm.

P: Yes, I would say so.

I: Okay.

P: Yes.

I: In terms of…

P: But based on facility I think then the facility person will be the one who will be able to answer that one.

I: Okay and in terms of uh monitoring you think it has uh…

P: Uhh yes, like I was saying we monitor the patient on spot…

I: Mmm.

P: Using the tablet.

I: Uhh so how can this cause the positive changes that you have mentioned like maybe improve success rate, should success completion be maintained?

P: I think it will keep the box and then give it to more patients.

I: Okay.

P: Mmm, we don’t just give it to certain facilities, we implement across all, yes.

I: In the absence of xxxx (organization name) what is be needed for it to be sustained let’s say xxxx (organization name) is gone and now the project is with the department of health?

P: Mmm, uh I think all we need is the boxes. Proper working boxes and tablets.

I: Okay.

P: Mmm

I: Alright.

P: Because they have the skill *akere* (right) the skill was given to them already, yes.

I: Okay and in terms of uh technical issues who do you think should be resolving for all those technical glitches?

P: I think our support system. I think Mmm I have no idea [laugh] because we struggle with IT and stuff.

I: Okay.

P: Mmm.

I: And (.) are there any negative changes you can think of that have been brought by the digital adherence technology?

P: Absolutely not.

I: Okay.

P: Because remember now the box was not forced on to a patient, yes.

I: Ok and were there any concerns that patients were opening the box without taking-

taking medication, were you trusting that if the patient opens the box medication was

was taken?

P: But remember they said uh the patient opens the box, the tablet will be green if

the patient opens the box for a certain period of time, yes whether the patient took

the medication or not that one guys will never know uh and there is

nothing you can ever do to make sure that the patient takes treatment it only that

alone and uh what do you call this AFB monitoring Mmm.

I: Ok but, but as the staff do you trust the patient once you see the green box do you give patients benefits of doubt in trusting.

P: Uhh I think it go down to the relationship that you have with your patients. So, as a nurse you will be the one who will know *that* this one I trust that the patient took the medication.

I: Mmm.

P: Mmm and also when the patient comes back in for checkup uh you have to see if,

they improved or not.

I: Okay

P: What are the factors affecting the patient if the patient has not improved.

I: So, how can we address the challenge of patients not answering the phone calls

made by the healthcare workers?

P: (…) [laugh] I, I…

I: Or sharing of the phone numbers?

P: I don’t know but waht I usually encourage the focal nurses to do every time is when the patient comes in for a visit, just double check if the patient still using the same number and also I think every patient differs sometimes other patients prefer other methods of communication. They should ask which one the patient prefers. Do they prefer you to call them after four or early in the morning? I think it goes down to that. Because others prefer you to send an SMS or they prefer you to call them, I think uh every patient differs.

I: Uhm how do you think the issue of stigma can be addressed you once mentioned that some patients declined the box because it was too big and they fear maybe or are scared that other people will see them. How can that be addressed?

P: We educate, we continue by educating patients and educate the family members that is why we always advise patients who start TB treatment to come in with one family member so that they are not stigmatized at home, I think even if somebody can be stigmatized from outside you don’t care because you know that the people that you live with they know and have accepted so uh.

I: So, can you let us know the system level structures that are there in order to

integrate the smart pill box and differentiated model of care which is the phone calls

and home visits after a few days into the existing TB program system.

P: Boxes should be properly working.

I: So, you mentioned that uh boxes should be properly working, what do you mean by properly working boxes.

P: The, the… what do you call that thing that you charge when putting in the box?

I: The module.

P: The module yes, yes maybe it should work properly. Remember now you are dealing with a small number of people so now if you are scaling up that means you are you will have more. So, I think it should be working properly and if it not working properly there should be a uh how do I put it, like when the patient brings back the module. Then the patient is supposed to go back home with the module that is working same day. Unlike you say the patient wait for five working days because then she is going to relapse.

I: Yes.

P: She is going to relapse uh.

I: Mmm and uh who do you think should be responsible for preparing boxes

because now is xxxx (organisation's name) is preparing those boxes.

P: [laugh] I have no idea.

I: Who, which kind of staff can you think of that can support with that?

P: For preparing the boxes?

I: Yes, within the existing program. Who can you think of?

P: I see, I think the CHW (community health workers).

I: Ok?

P: Uhh because the boxes, the, the module is prepared from wherever it comes from *akere* (right).

I: Mmm, it needs to be charged.

P: It needs to be charged?

I: Yes.

P: So, somebody must charge it?

I: Mmm.

P: And then?

I: For it to last for…

P: Six months

I: Six months

P: So, it doesn’t come charged when it comes from the factory?

I: No, it doesn’t come charged because its a hazard.

P: Mmm.

I: They cannot transport it charged.

P: Mmm so somebody must charge it, make sure that it fully charged and then be inserted on all the boxes.

I: Yes.

P:Uhh , uh then I think we need to employ people for that guys because imagine the facilities with a thousand patients who are on treatment. Then let’s say someone has to go and check patients at home and stuff, it won’t be possible and how long do you charge this module?

I: Uhh depends on… every 3 hours, four hours

P: Mmm.

I: So, it will be fully charged.

P: From the top of my head, I can’t this of anyone because already in the system…

I: Who can assist with that?

P: Who can assist yes, I would say the TB focal nurse but then uh *akitsi* (I don’t know) let me not lie I don’t know.

I: Okay and uh is there an existing system that is being used to document feedback from the phone calls meaning successes or the challenges and also challenges with the box is there a system that currently being used?

P: Yes, there is a tracing book.

I: Okay.

P: Where every time you call a patient you record from the tracing book the outcome.

I: Ok.

P: Mmm.

I: So, who is responsible for that?

P: Whoever is calling the patient.

I: Okay.

P:Mmm.

I: In terms of the medication devices is there a where it being recorded, any challenges and successes?

P: Medication devices meaning?

I: At the box looks at your box. Any challenges of the box?

P: Not that I know of.

I: Okay so the differentiated care which is the phone calls and home visits that are being recorded?

P: Yes, yes I think the box they record who they gave too.

I: Ok.

P:Uhh , uh because they have to return it *akiri* (right).

I: Yes.

P: Yes so we need to know who gave it to and what date you gave it.

I: Yes so…

P: Mmm.

I: Going forward uh can you think of a system that can be used to also capture how the smart pill box is going the same way home visits and phone calls are captured?

P: But then they use the tablet can they not print out uh the outcome from the tablet, I think that will be easy uh, uh to take the report that you print from the tablet for that certain patient and then you put it inside the patients file.

I: Ok.

P: Mmm.

I: And when let's say the patient struggles to use the technology let's say there was a technical glitch. How do you think that can be recorded?

P: When the nurse or the patient struggles?

I: The patient. Let’s say the patient has the box and it fell at work and there was a glitch and they report to the facility…

P: Mmm, I think that should be recorded on the file.

I: File.

P: Patients file?

I: Patients file.

P: Or but then technology is very advance they can take it and record it on the tablet.

I: Yes.

P: Mmm.

I: Yes, okay, uh so can you tell us any gaps that exist in the way intervention is being delivered currently?

P: No, I can’t think of anything the only thing is that it for few patients it was implemented but anyway it was a trial so it’s understandable.

I: Okay so what are your thoughts with the patients who have multiple morbidities, lets say HIV and they are also taking TB treatment using the box?

P: I don’t think there is a problem they can put all the medication in the box the problem is will it fit? But then no wait because now remember we have a drug interaction so it might be a problem because then uh let say for instance you can’t take uh your Rifafour with your ARV at the same time maybe you take your Rifafour in the morning you take the ARV at night so then, are you guys able to make the reminder for all?

I: So far it only has one reminder.

P: I think that’s a gap uh to say we should have, multiple reminders for patients who are taking other treatment like ARV's

P: Mmm, uh.

I: Okay.

P:Mmm.

I: Thanks, interesting suggestion.

P: Mmm.

I: And do you have any suggestions, you have mentioned the issue of multi reminders for patients who have multi morbidities…

P: Mmm.

I: Do you have other suggestions for improving the device when you look at it?

P: Uhh it’s a box man uh it’s a box [laugh] maybe it can have compartments inside to say Monday, Tuesday, Wednesday, Thursday, Friday, Saturday, Sunday. Mmm, but besides that it a cool box

I: And what you think of the colour?

P: Change the colour make it black.

I: Why?

P: Its extra work to always wipe it and make sure that it clean before you go to the facility.

I: Okay are they clean when they bring them to the facility, do you know?

P: No, I, I don’t know. I am just thinking on the top of my head looking at it that if it was mine [laugh] I have to make sure every morning that before I go to the facility, I have to clean it first.

I: [laugh] okay and then do you have any suggestions to improve uh the platform the nurses look at check adherence platform monitoring happen.

P: I think the only thing is printing… can they print the report from that for each patient, or is it possible?

I: Uhh it is possible to, to print if you are using let say on the desktop you can send to the printer.

P: And print it okay.

I: Mmm.

P: Then if that the case its good, can you make comments?

I: Yes.

P: Okay that’s perfect if they just print and the put it in patient’s medical record.

I: Okay uhh do you have any suggestions for improving uh the differentiated model of care, which is phone calls, that will be made two to three days and home visits after four days of missed doses.

P: Mmm, nothing its just more patient related like they need to be encouraged to give us correct numbers and correct addresses and state when we can come for a home visit and explain. I think it should be written because some patients go to work like when you work at Shoprite then you are not allowed to answer your phone while at the till.

P: But then who is going to call you after seven, its the other thing Mmm.

I: So, what are your final thoughts about the smart pill box implementation the phone calls and this whole project…

P: I think this was a great project and I think we should continue with it yes. Especially for patients who come to the clinic for the first time and are put on chronic medication, yes even if its for the six months. For six months then the box… even if its for ART, just for six months or the patient will decide can I use this box for three months so that they can be assisted to make sure that they are on treatment on daily bases. Once they are used to it they can bring it back then we give it to someone else.

I: Okay.

P: Mmm.

I: Do you think it feasible for the department of health to carry on with it without the support of xxxxx (organisation’s name) to scale up.

P: If they do it right, yes.

I: What do you mean if they do it right?

P: If they do it right like you guys have been doing. People monitoring the project move around, there are people who are able to charge this like the way you guys are doing it and having people from DOH doing the same thing that you are doing then I think we will be fine unlike just giving it to the facility and the facility must see everything, no.

I: Uhh so how do you think it must happen?

P: Uhh no *akitsi* (I don’t know).

I: You are saying people where are these people going to come from? Who are going to charge?

P: Uhm honestly, I don’t know but for each facility I think the facility can work around it and to say you know what let take *bani* *nabani* (who and who) do this and then see but then it’s doable especially if you are not going to give the box to all the patients you can start with the small number and see how it works uh.

I: Alright thank you very much for your information, do have any more comments?

P: No, I am good thank you.

I: We have reached the end of the interview the time is 13H04.
