# Supplementary material for: Acceptability of Digital Adherence Technologies to support people with drug-susceptible TB in South Africa
Source: PLoS One. 2025 Sep 24;20(9):e0332103. doi: 10.1371/journal.pone.0332103 (PMC12459780; doi:10.1371/journal.pone.0332103)
Supplement: S4 File — (ZIP) [file pone.0332103.s004.zip › S4 Transcripts/HCWs and Stakeholders/IDI 30_STK.docx]

**TRANSCRIPTION NOTATIONS**

| **Label Key** | **Meaning** |
| --- | --- |
| **I** | Start of each new utterance by the Interviewer |
| **P** | Start of each new utterance by the Participant |
| **N** | Note taker |
| **{ }** | Indicates that details were changed, or pseudonyms were used to anonymise data |
| **( )** | Indicates the description provided to anonymise data |
| **XXX** | Words were omitted to anonymise data |
| **-** | Breaking into a sentence by the next speaker |
| **…** | Pause or drawn-out words |
| **[ ]** | Indicates noise made, e.g. [laugh], [sigh], [pause] |
| ? | Beginning of utterance by unidentified speaker or questionable text |
| **[inaudible segment]** | Unclear section of the recording |

I: So, do we have permission to audio record you ma’am?

P: Huh, you do.

I: Thank you, date of the interview xxxxx (interview date), location xxx [facility name] [pause] the location is xxx [facility name] in xxxx sub district.

P: Mmm.

I: The PID is stakeholder number 05, the time is 11H10. The interview is being done by XXX [name of interviewer]. So, ma’am can please let me know huh what’s your role? What’s your position?

P: Okay, xxxx (position) including huh TB- both the huh first line TB which is our normal TB and the MDR together. I am also responsible for any diseases that is communicable like your COVID-19, your measles and any other condition that is communicable. So, huh my role is to see that all xxx number facilities in xxxx (sub district district) are meeting the requirements that is needed by the organization as a sub-district to reach 90- 90 -90 huh strategy in terms of TB success rate and cure rate.

I: Okay, so how long have you been in this position?

P: Huh, xxxx (number of years).

I: xxxx (number of years)?

P: Mmm.

I: So, can you tell me what you know about the ASCENT project if you have to explain what digital adherence technology is to another stakeholder or healthcare worker who does not know anything about it, what would you tell them?

P: Huh I would say that’s it’s a strategy that has been implemented by, by huh-mmm our non-governmental organization huh partners or is xxxx (organisation’s name) huh with the aim of improving the, the… adherence in TB treatment. So, basically how it works it digital in such a way that huh they are using a prepacked device where there are tablets for TB. Huh it can be the Rifafour for intensive base and the Rifinah, for the continuation but how, how it works is that each time the patient opened the container i it's linked to, a device in a facility where the patient is going to be registered. So, as it is linked like that each time when the patient opened the advice to take tablets it also reports to huh the device that is linked at the facility. So, it shows us like on a daily basis huri (that) when a patient has taken a treatment. So, it's improved adherence in such a way a that if a patient did not open the, the device it also shows to us with the red calendar that XXX [patient’s name] or so and so did not open the container. So, it also works as a reminder to remind the patient that okay, today we didn't see the activity of you opening the box, pillbox. So, if maybe the patient had forgotten, then he will immediately take a pill and then if maybe it's, it's starting to be a problem- we see that the patient is doing it on purpose not to open the box. Then, it alerts us to start with the tracing and follow up so as to see what the problem is because sometimes you can find out that maybe the devices is off or maybe the patient is working far away from, from the device or even the patient is dead. So, we can even help us to get the outcome quickly and know what is happening with the patient. Then, we will follow up reason why the patient is not being opening the box. So, what actually happens is that when we actually enroll the requirements the patient should be new, it should be the first-time huh the patient is taking a treatment. If it's a transfer, it shouldn't be more than three to four weeks initiated on treatment .Therefore we will educate them on how- what is a device, how does it work if there is anything maybe that they don't understand before we could enroll them in a study and then we clear such concepts…

I: Okay.

P: Huh that's what I understand about the digital adherence technology.

I: Okay, so you talked about the container or the smart pillbox…

P: Yes.

I: Is there any other digital adherence technology you're aware of which is part of the study?

P: No, for now at xxxx [sub district name] I only know of this one.

I: Okay, so you mentioned about tracers who follow up on patients if they don't open the, the box…

P: Mmm.

I: So, how does this happen, how is the patient followed up if they see a red on…

P: Mmm.

I: The system at the facility?

P: So, on initiation, what we actually do we keep the their traceable physical addresses to say this device we are giving it to so and so he stays at house number so and so. Then, you know with-then initially DOT supporter or directly observe supporter will be allocated on the initiation day, we call them the CHW, the community healthcare workers so that they are aware huri (that) you are responsible for XXX [referring to a patient]. Huh throughout the period of six months when you will be taking the treatment, so if we see that there is huh a lack of this activity of opening then we just say go and check XXX [referring to a patient] today she didn't open the box or it's two days now we didn't open the box she is also not answering the phone. So, it's a list we retrieve huh on a daily basis of the people that we are waiting for that day then they go and check them.

I: Do you make a phone calls?

P: Huh we do it huh- basically our tracing is divided into two; there is telephonic where, where such immediately action is taken when we don't see you any activity, we don't wait for too long, we act immediately huh. Huh we make a list and then they (tracers/ community workers) will follow up.

I: Okay.

P: Mmm.

I: So, who's responsible for making phone calls, is it the same team?

P: We have a tracer; a person who is a hired- employed as a trace with the duty to see that all these lists of people who were supposed to be seen today did not come, then he calls them and then he even records the outcome if the phone huh- for instance maybe it went to voicemail. He will then say I've called XXX [referring to a person] two times today his phone is going straight to voicemail, or I found her, and she promised that she will open the box just that she was busy with something in town.

I: Okay, alright so how else is a patient reminded to take their medication? You mentioned that if they see on the system that the box was not open they call the patient to remind them…

P: Mmm.

I: So, before that happens, how is the patient reminded to take medication?

P: Huh from the facility?

I: From the device by the device.

P: I think it's it, it, it makes a sound or a peep, yes.

I: Okay, alright [shuffling]. So, you mentioned about huh assigning a DOT supporter to each patient in every patient upon initiation, can you maybe highlight the differences between using digital adherence technology versus the, the DOT supporter because you mentioned that huh in the, in the beginning huh- previously you used to (.) used to follow up, you follow up on a patient once they've missed-

P: Huh with the technology, the best thing about it is that it doesn't wait for a day for you to have missed the appointment. We can see here on the list and say, go and check XXX [referring to a patient]. This one reminds you immediately, it's sort of it traces, you immediately- once we see a day gone by and you missed medication- that you did not open. The box, immediately reminds you- you get a reminder- a message, a peep [alarm] and yes.

I: So, when you first heard about that digital adherence technology, what were your expectations before it was implemented here in xxxx [sub district name] ?

P: Huh I was expecting really huri (that) it will improve our adherence in a way. We were failing a lot when it comes to adherence really because people would just take medication and go home. Some don't have families or a support system to remind them or to give them that medication but then when, when these devices came and this project started, I was like sure at least you know because some will give you a funny reason to say, “no, I just forget nje (like) you understand” and it's even worse if there is nobody who will go and knock at the door to check up on them. But not with this one, this one will annoy him [patient] until he takes medication. So, I felt like this one at least will improve our adherence versus what we're doing.

I: So, did you think it was an easy technology to use first?

P: Huh for who? for us or for the patient?

I: Huh maybe we can start with the healthcare workers.

P: Huh with the healthcare workers, I thought *huri* (that) it going to be a bit challenging because you know most of the nurses when we, we, we, we, get out of school you know that technology thing, you know it needs a lot of teaching and I felt *huri* (that) well maybe they are going to experience a problem in their daily use of it but as the time went on I knew that they will get used to it eventually. So, I was worried about the first two months of implementation or might get you know the variances between you know what is expected and what they're actually doing Huh.

P: Did you think it was going to make work efficient for healthcare workers?

P: Mmm (yes).

I: How so?

P: Mmm (yes) in a way that you know it reduced this thing of sitting down with a phone when there is even no data or airtime to call the patient. The patient is going to be notified immediately, unlike you wanting to phone a patient and there is only one phone in the facility, and sometimes with no airtime or when you try to call the patient and the phone just go straight to voicemail. So, with this one I just felt huri (that) you know it's going to be very much helpful because once the patient is having a device at home, that means they will be reminded immediately, you know, unlike using a phone call.

I: Mmm.

P: As a first step because that is what we do.

I: Alright.

P: Mmm.

I: And then from the patient's perspective, what were your thoughts on that?

P: Huh I thought huri (that) okay before they get used to it, it's going to be a problem because looking at our area xxxx [sub district name] is very rural where network- before I even talk about loadshedding. Network is a problem, so if the device is depended on network, then it can just be a challenge because sometimes we experience network shortage- maybe the network can can fail and huh also looking at their literacy level, their low level of education might make it difficult for them to understand the usage, you know, how to use it and what not, yes.

I: So did you, did your opinion change about this after implementation started?

P: Huh, huh it did it, did after I see the, the first huh outcomes for the quarter-the, the, the, the study was being implemented, I have seen *huri* (that), you know, huh to the, to the healthcare professionals who are using it there is a lot of improvement; they understand the device better, you know, when I come they teach *mina* (me) I didn't know about this, this information, the information that I'm giving you, I got it from the, the, the, the, the, the personnel who are using the device. When I come, “okay, sister let me show you how this works, you know, you know, when you open it, you open the previous and it shows you the current. It shows you the success rate you know, the pie, you know, thing you know it shows you.” So, I, I have seen that okay they understand the device. I would say it so easy for them to use it now, same as the patient when I look at the total number. If they initiated like ten and you find that eight managed to complete successfully. So, both from the patient side and the care personnel side I felt *huri* (that) *mina* (me) to me it's working.

I: Okay.

P: Mmm.

I: So, you mentioned that you learned about the device from the personnel, which personnel exactly?

P: Huh the one that was trained to use the device, the one who’s a TB focal because in xxxx [sub district name] we got people that we call TB focal they're dealing specifically with the TB program. When I came here I went straight there and I asked them, “I heard about the project, how is the project going,” and they put me on board

I: Okay, so did you by any chance attend any training that was conducted by xxxx (organization name)?

P: No not at all.

I: Okay, so maybe going forward if the project is to be scaled up maybe at the programmatic level, how do you think the training should be conducted, who should be trained?

P: The TB focal,it is going to be even easier unlike if we just do it randomly people leave; people go to night duty, you know, people change their area of work and sometimes you find they are in TB, sometimes you find they are in maternity. So, it might be a problem but if we focus on those people who are appointed by letter, saying wena (you) specifically you are doing TB. I think it would be better if also the community healthcare workers maybe were to be trained, these ones I was telling you about. They are like DOT supporters who go and ensure that patients are taking their treatment so that they can even have just a little idea or the basic huri (that) of what is going on with this device- with this project, even if they are not going deep.

I: You mentioned about rotation, so do this TB focal not rotate at all?

P: Once they are appointed with the letter, no. That’s why if you found XXX [referring to a health care worker] at a TB room last year-even if you can go now, you still going to find her.

I: Okay, that's interesting and so from your perspective as a district coordinator can you let us know the benefits of the box?

P: The benefit of the box to me it’s TB focal are starting to work smarter, it's reduced the workload of having to see patients and having to make calls and having to make a lot of lists that are not even being followed. Huh having to deal with a lot of follow ups- to follow-up. Having to deal with a lot of people who are missing, their, their doses; interrupting and then the success rates and the outcome basically for this whole sub district has improved a lot.

I: Okay.

P: Mmm.

I: Do you think the use of the devices huh improved or has had an impact on the relationship with patients?

P: Improved in which sense?

I: Huh like the use of the box-

P: Mmm.

I: From the facility side, does it have any impact on the relationship with the patients. So, maybe with the way they interact because they're interacting more?

P: Huh *mina* (me) to me it's still the same I don't think it has even changed because they're not coming on a daily basis. I give medication and say, “go and drink,” we still talk. They still talk telephonically if there is something that they don't understand-

I: Mmm.

P: They're still allowed to come and say-

I: Mmm.

P: You know *itimile* (it off) or I don't know what to do with it or I'm unable to, to, to open the box. So, the interaction is still the same because we were not seeing them daily anyway. We're giving them two weeks and then if they're working or they're there they're there the engage with other activities will even give them a month-

I: Mmm.

P: So, it's not like we are seeing them on a daily basis. They engagement between the patient and the healthcare personnel, I think still remain the same.

I: The same, so what are the benefits of huh the differentiated care meaning the follow-ups that were being done if the patient does not open the phone- the box, for example, a phone call or a home visit. So, what are the benefits of those actions?

P: The, the, the which one? The home visit or the, the, the box?

I: You can start with the phone calls, now we are on the follow-ups-

P: Mmm.

I: The phone calls or the home visit, so what are the benefits of those actions?

P: Okay, the benefits of the phone call, the patient is huh meant to, to save the little money that he doesn't have to come by the facility to do the follow up or to ask. So, it will remind him there. Unlike *atsiye* *chelete* (take money) the money that he doesn't have to come to the, to the facility, it’s a benefit. Then, the home visit, yes, because now it's coming at the comfort of your home, we come and knock and say, “hey you know you didn't take your medication, so please take” but *ewu* (that one) the disadvantage is *huri* (that) we might even go there later after three to four days…

I: Mmm.

P: Mmm that the disadvantage of the [knock] physical home visits.

I: Okay, so huh what are the challenges with the home visits? let's start with home visits?

P: Huh wrong addresses, we are given wrong addresses where a person will just pick up the number on his way to the facility and say okay, I will go… they know we're going to need the house numbers, we just going to give them 1496 and say this is where I am staying and then when you go there you find out that there is no person. Remember with TB you only need to miss one day or two days to make it a mess. So, now if we're looking for you today and tomorrow we can’t find you it means that where you are you're not taking medication. So, it has serious impact on, on, on us and then the other thing is, is employees who are moving around like working in farms seasonal; workers, you know, today, yes, he gave you the correct address but tomorrow he decided to go and join people who are picking up oranges because they just know the contract on this side is terminated. So, wrong addresses it's, it's, it's the, the movement, the migration of the patient is giving us a problem with physical tracing.

I: Yes, so are the issues around stigma in terms of huh physical stigma- physical tracing?

P: Huh there is huh people feel *hore* (that) if I come… normally I would be wearing my T-shirt written xxx (district name) Department of Health maybe stop TB or xxx (organization’s name) whatever TB or HIV. So, they feel that once people see the printing on the uniform, people in the community start discriminating. So, it does have an impact on our patients to say huh, you know, people think *hore* (that) I'm very sick than anyone here. So, some even stop taking treatment.

I: And what are the challenges with the phone calls?

P: Huh challenges with the phone call one at our site, our phones does not always have airtime and sometimes it off due to loadshedding. The battery is not fully charged or even went missing around the facility because the facility is big maybe someone is currently using and you can’t find get it. Two it's, it's this wrong telephone number that you get and then it's being answered by XXX [referring to a random person] or the white man who is swearing at you to say, “don’t tell me I don't know the person, huh don't ask me about the person even,” or the phone cellphone got lost mmm.

I: So, you mentioned challenges with telephones from the facility side in terms of not having airtime or a phone itself…

P: Mmm.

I: What are some of the challenges in terms of home visits from the facilities side, are there always people to go and trace? Is there always transports?

P: No, huh what actually happened is people who are tracing are CHW (community health care workers) who are allocated per section. So, what we normally do when we initiate a patient on treatment we check the section where they are staying huh and decide then who we are going to allocate. The CHW staying at your section will be allocated so that he or she will be able to walk and come see you but now the challenge is that even if they [community health workers] go there they will find that the patient got a job in another area and is now staying there so that he can reduce the expenses on transport. Normally you don't get people there, it either you going to get the neighbor or grandmother or the parents of that patient.

I: Mmm okay.

P: Mmm.

I: So now can we talk about the challenges with the device itself, what have been some of the challenges with the use of the box?

P: Huh I will give my experience of xxx [ facility name], I think I was here last week Thursday, and it was huh off and I don't know whether it was the battery or the issue of the charging or the electricity. And the other thing I think you guys are using the MTN and MTN around here is a problem. If you're not using Vodacom sometimes it's very difficult for it to connect in this area. Sometimes its not connecting when there’s patient that you want to enroll on the devices. The device won't be connecting at all due to network issues.

I: Network huh so what are the other issues?

P: Huh besides the, the, the…

I: The network issue.

P: Huh maybe if, if the person who was trained to use the devices is on leave and there is another nurse who is leaving because she also has to go to rest or when she wakes up sick. So if at least we train a person in the facility, in facilities that are big like xxx [facility name] , xxx [facility name] the CHCs (Community Health Centre) at least if we can have two people per facility so that when the other one is not in, the project must not stop.

I: Okay and were… Are there issues of acceptability you are aware of, of the project or the box itself or the system?

P: By who us or the, the, the, the clients?

I: The clients.

P: You know you know with the clients honestly clients do what we tell them, it depends on how we market it and how we sell it. So, I didn't see any, any, any problem.

I: Okay and were there any issues of acceptability from the healthcare workers side now?

P: Huh let me say because now we are only having four facilities that are you know huh are participating, these four professional nurses who are using the device, I didn't see any challenge in terms of accepting it.

I: Okay.

P: Mmm.

I: And are you aware of any technical glitches that have happened?

P: Huh yes I think it was when the first group exited the first group that you trained when they were exiting huh for you to come and start again. That period it was like there was no one who knows what is going on, whether the study is continuing or it completed because when I went to the facilities I asked her XXX [intern name], “it's like they paused the study.”

I: And then with the box itself with the any glitches that were reported on the box?

P: Not that I know, not that I know.

I: Okay and huh are there any issues around stigma that have been reported, stigma with the box now?

P: Huh no, not that I know. I haven't had anything that of that sort.

I: So, what are your thoughts regarding stigma in the box?

P: Huh mmm not to me I don't see it will because any stigma since people are taking other medication like your hypertension medication. Huh, it's prepared it's like the ones that are collected at Clicks for CMDD, they are carrying their packs even though this one is even more you know nicer and sexier than these big packs that people are going to collect . So, I think it start with you the person using it, then other people are going to accept it eventually if you feel that it working for you.

I: So are they maybe homeless people or drug users you are aware of who have used the, the box?

P: Huh I think I’m having one or two here in xxx [facility name] and the other one is in xxxx [facility name] , I even called him myself when I saw that he is the only person with the red boxes (non- adherence calendar) you know, I feel like he didn’t like the idea of the devices , maybe he felt like he was being tracked, like he is being followed everywhere he goes, even in the toilet. Also, the idea of calling him when he missed medication. So when I followed up myself and called the parents. I found out that the patient recently moved to xxx [CBD are] there doing *Nyaope* ( doing drugs) this drug is problem in our villages and I think, I also have one here in xxx [facility name] who is who also ran away from home to stay in the streets but there's nothing that we can do about those it will always happen if you enroll ten there will always be one who’s problematic.

I: Mmm so when you followed up on the patient using drus and said this device is tracking him what was the outcome of that follow up?

P: Huh he failed completely, failed completely I think maybe the parents ended up bringing the box back at the facility huh.

I: Huh so do you think the box assist in any way to support this kind of groups of people?

P: Who are doing drugs?

I: Yes, the homeless people.

I: Huh so do you think the box assist in any way to support drug users staying in the streets?

P: Huh to me I will never say yes because if he is carrying the box and he's not going to open and take medication it's still the same as if he's having the pack that we give- a normal pack that she would just leave at home and not drink. So this kind of group of people that you are talking about I think maybe they need something else because nothing will ever work for them giving them normal boxes or the device. I think they will always refuse and leave it at home.

I: How do you think they can be assisted breath to continue and to complete the treatment?

P: Huh for me I think what maybe if we can deal with the problem that they're facing the drug addiction so that they can come back to their normal being and understand that they need to take treatment maybe we can start there let’s deal with the problem that the drug addiction because we cannot force them really, it's like they don't understand, and they don't care. They don't see it worth.

I: Okay, so you're suggesting that maybe psychosocial support-

P: Let’s let start with that yes.

I: Okay.

P: Even if it's if it means that taking them to rehab and then we introduce this program gradually while they are still there you know as they are recovering and then maybe they will have an understanding but out of the blue going out at the garage or wherever and give them a box honestly we wouldn’t be making justice to ourselves.

I: Okay, so from your perspective as a district coordinator can TB treatment be improved by using the box as huh- does it have an impact on patient adherence?

P: Huh it does, it is going to improve it-it has improved already looking at the quarters since the study was implemented around xxx [sub district name] between these four facilities isnt it that we check this before the study was implemented at this facility. We were running at 60-65% success rate, struggling to reach the set target but since this project started, they are running at 80-85% sucess rate if they are failing at least they are at 79% success rate you understand. So, I do see the difference. This program is working for me.

I: How has the project impacted monitoring of patients?

P: It has improved a lot because before you would do home a visits, when there is a possibility that you won’t get a patient right but with the device the box is there with the patient, so it's immediate and effective.

I: Okay, how has the project impacted on the healthcare worker’s workload?

P: Okay, it has reduced workload because maybe if you were allocated, lets say for instance, 30 people to visit and now maybe you have 10 that are on the study who are being monitored by this device and you wake up every morning you check okay, this one has taken his medication at least it will reduce the total number of visits which are not easy to do every day.

I: So can you tell us maybe the negative changes that have been brought by the digital adherence technology, are there any negative changes?

P: Huh not, not that I have noticed.

I: Okay, do you trust patients that when they open the box it means they've taken medication. Are there any concerns you may have that patients can open the box without taking medication?

P:  I trust that they open and take medication you know with TB  at end of the day, you have to be cured. The results are going to tell me because the device did not change anything in terms of the monitoring of the patient I'm still going to take your smear for conversion and take it for continuation phase when it's time for you to complete.  I'm still going to take the smear so if you come back negative it  means the TB is cured in your system because the system will never lie and say we don't see TB anymore when it's still there. Then those patients that will continue to be positive since they start maybe they're the ones who are just opening and not taking but it's not the picture that I have from all those patients who have been  enrolled in this study at the end of the study, at the end of the study they are all negative it means that they were taking. Isn’t it  you just sit and hope  they are drinking same as the ones taking  this other chronic medication high blood you have to give them six months and what not, we are not even there to monitor them just okay, when he comes to high blood is normal, the blood pressure is normal. So, even with this one the last smear will tell me.

I: Okay.

P: Mmm.

I: And huh can you tell us the system level structures that are needed to be improved in order to integrate this program the way the box works, the system linked to it at the differentiated model of care which is the phone calls and home visits that had been after you have seen thar that the box didn’t report the dose…

P: Mmm.

I: So, what needs to be improved for this to be integrated in the TB program. Let us say xxxx (organization name) is gone, the NGOs are gone. What needs to be in place?

P: Huh money because when you, when you go with your funds and everything I know that the department will be saying we don't have money for these three months or four months to buy these, you know, the batteries, you know, everything that is connecting to the device. The logistics can be a problem because we need to buy, we need to make sure that the device is always charged -the batteries and you guys are able to do that but with us it might be a problem because now remember maybe it will be the whole 23 facilities within the district which is going to be too much for them.

I: And then in terms of the, the staff to implement the study?

P: Huh I think training, training, training, training-

I: Mmm.

P: Lets train as much as we can.

I: And then who should be implementing once xxxx (organization name) is gone?

P: Definitely it going to be me and us as a sub district.

I: So, what will be your role as, as a coordinator?

P: It will be to ensure that every facility within all facilities is having enough boxes. For example whe they are running out of boxes in facility A and there is another facility that is having more than 60 boxes that are not even using all of them I will redistribute.

I: Mmm.

P: Huh so that I must make sure that everything to do with this huh, huh project it is- it's being you know catered for.

I: Mmm and then in terms of actually enrolling the patient and monitoring on the patient on the system who should be doing that?

P: Huh mmm enrolling in the system?

I: Yes.

P: I think we should also include the data capturer who will be then responsible for this project to know as xxx [distrct name] as a whole, we have registered maybe 355 because that is what the number that we normally have in a quarter. We have registered 355 from twelve is from xxxx [facility name] , 9 is from this facility, you know, just like Tier because when they are sitting on Tier they know which facility is having a problem, how many did we initiate, how many completed, how many loss to follow.

I: Okay and what you think should be the role of the TB nurse?

P: Continuous monitoring because they are the ones who understand that now when this patient is 8 weeks on treatment they are now due to go to continuation phase. They actually know that it's time now to collect sputum which must convert. They also know how to manage HIV and TB co-infection.

I: Okay and then if there are technical glitches with the box who should be responsible for fixing it if xxxx [organization’s name] is out of the picture?

P: I think the district manager and the sub district manager.

I: Okay, so do you currently monitor or capture the challenges successes or problems using the digital adherence technology?

P: Huh yes I do, I do when I visit these facilities enrolling people in this project. I do go and check beside checking the normal TB program that they're doing. I also check if there is any problem with the device, any patients you know giving you problem what are other problems and what you think we should do then, yes, they will explain to say in this facility is only one patient and so and so is away. Okay treatment is enough and then we are having three who maybe district did not change treatment, but we called them, and we got them today they have taken their medication.

I: Okay. Do you record those challenges anywhere?

P: I just take few notes to say okay the project at xxx [facility name] this is what is going on so that when the review- when they are absent it does not sound like it's new, I don't even know the facilities. I don't know what is going on, I don't know anything about the project-

I: Mmm.

P: So, despite that I am not trained like you know I've never been to a formal training, but I must have a picture of what is going on in these facilities.

I: Okay, so huh if let us say it's scaled up right to be conducted in all facilities, how do you think the successes or challenges should be captured is there a tool in existing tool that can be used?

P: No, for this TB program you know we only using Tier, so we are used to how Tier works when you open it, it shows us the challenges and we act on them.

I: Okay, what are your thoughts about linking the platform we use in the project to Tier?

P: Huh I think it can be linked to tier in such a way that we will see number of patients starting treatment without the device and those with the device. Maybe you can just add on device on Tier, then at the end when we do consolidate data and make a comparison. If they are all linked to tier to say, okay, total number initiated is 14 out of 14 ,7 were on the on the device and this other seven were not on the device. Out of seven that are on the device who completed the treatment, who were lost to follow .You know and their success rate, same as the other one who are just you know, initiated on treatment using the normal way.

I: Huh so do you think it is feasible for this project to be scaled up maybe across the whole district or the province and nationally to be taken over by the department without xxxx (organization name)?

P: I think there should be funds to sustain because this is very a beautiful project. For them to sustain it, it would mean that boxes or the containers must always be there, treatment must always be there, people who are trained to do that to continue with this project are always there, data capturers are always there. It can then be implemented and be linked to Tier and then there's a person who is always responsible for checking that. So I think the department should make sure of that because we can implement, then six months down the line we don't even have money to buy all those containers and, and, and, and, and then we end up going back where we started ,you know, our success rate drop, loss to follow up goes up.

I: And can you let us know any gaps which exist in the way the project was delivered?

P: Huh mmm uh, uh you know you mean going forward?

I: No, the, the way the project was done all this, were there any the gaps?

P: Huh mmm I think the information was not escalated evenly as there are facilities who don't even have a clue of what we are talking about. At least if we have a basic training just to highlight to say now that there are people in xxx [sub district name] who are doing this and the aim is you know to improve adherence. There are possibilities that it might be rolled out to all facilities so that they know and have a clue. That way it was even going to reduce the problems with understanding and you starting afresh you know with new people who don't even know what is going on mmm. So, I think information sharing.

I: That's, that is a good suggestion, so what are your thoughts on patients with multi morbidities and the use of the box because so far it was just for TB treatment?

P: I think because we are saying, we are trying to reduce the number of days and the money for them [TB patients] to come to clinic every time for example if someone is HIV positive maybe on diabetic medication and hypertension and also on TB. They will come and collect the box or refill and go home and then you will be given another date or to come for this other medication which is not fair it's like we didn't reduce any financial burden because the patients still come two or three times. Maybe we need to consolidate and pack everything in one just like what they're doing in the CMDD. If you go there you take your whole package and go home and then the visiting date is the same to collect blood, collect medication, you take your TB, you take your ARVs, you take your HPT medication. So, I think you should make a, a device that will accommodate all these other comorbidities.

I: That's a very good suggestion and do you have any suggestions for improving the devices itself how the box looks and also maybe the system?

P: I was concerned about this size because it's big but then looking at the number of tablets that the patient have to see there's nothing that you can do about the size, it has to remain that size or even bigger if we're going to add other medication inside yes, so what else?

I: The colour?

P: Of course it is white.

I: Yes.

P: Huh you know with the colour huh anyone can still say even if it's black it means white it's red means danger *na* (I) for me the white colour [laugh] it's still ok because others can still label and say huh the one with the red box it means danger and what not and what not but *na* (I) the colour I won't say anything about it.

I: Okay.

P: Mmm.

I: And the monitoring system?

P: The monitoring system I wish they can be a way that we know that he opened the box and drink the tablets, not just to know that he opened that because patients are funny sometimes they can open and do not take medication.

I: Huh so how do you think that can be achieved?

P: Huh I have got no idea; really I've got no idea, but I wish there could be a way *where* we can see *hore* (that) Mmm.

I: Okay, so what are your final thoughts about the digital adherence technology?

P: I think it is working for us, it's something that we've been waiting for a very long time even though we didn't have any idea how to do it. Finally at least a device as a start it will address all these issues with non adherence. We know that it will come with its weaknesses and in as much it has weaknesses it has its strengths you know. So I think it is working for me.

I: Okay so what are the main strengths of this device?

P: On the side of the personnel, it's reducing workload in such a way that you know that the number of telephone- I mean of calls that you have to do is going to be reduced because the device is going to do that for you and then huh the physical visits where we are going to get the wrong address and all those at least it's been reduced. And then it strengthened compliance because some have funny reasons for not taking medication like they say they just forgot but with this device at least it strengthened adherence because when it beeps and it annoys them it makes them take their medication.

I: And the weaknesses of the digital adherence technology?

P: Huh the weaknesses is that it does not include other comorbidities and remember with TB patients, TB as an opportunistic infection waits until we have all these other things that will drop you. So, most of the patients with TB are people with other comorbidities like HIV and, and the weakness is that it does not include them.

I: Okay.

P: Mmm.

I: Any other weakness you can think of?

P: Huh what uh nothing.

I: Nothing, alright thank you very much for the information, we've reached the end of the interview. The time is 11H59.
