# Supplementary material for: Acceptability of Digital Adherence Technologies to support people with drug-susceptible TB in South Africa
Source: PLoS One. 2025 Sep 24;20(9):e0332103. doi: 10.1371/journal.pone.0332103 (PMC12459780; doi:10.1371/journal.pone.0332103)
Supplement: S4 File — (ZIP) [file pone.0332103.s004.zip › S4 Transcripts/HCWs and Stakeholders/IDI 31-STK.docx]

**TRANSCRIPTION NOTATIONS**

| **Label Key** | **Meaning** |
| --- | --- |
| **I** | Start of each new utterance by the Interviewer |
| **P** | Start of each new utterance by the Participant |
| **N** | Note taker |
| **{ }** | Indicates that details were changed or pseudonyms were used to anonymise data |
| **( )** | Indicates the description provided to anonymise data |
| **XXX** | Words were omitted to anonymise data |
| **-** | Breaking into a sentence by the next speaker |
| **…** | Pause or drawn out words |
| **[ ]** | Indicates noise made, e.g. [laugh], [sigh], [pause] |
| ? | Beginning of utterance by unidentified speaker or questionable text |
| **[inaudible segment]** | Unclear section of the recording |

I: So, do we have permission to record you?

P: Yes [Laugh].

I: Date of the interview: xxxx (interview date). Location is: XXX [name of the location]. The PID is: Stakeholder 02. The time is: 11:20, yes.

I: So, Ma’am can you tell us your- your role?

P: I’m a xxxx position.

I: How long have you held the position?

P: Since xxxx (start date).

I: Ok, so what are your responsibilities?

P: My responsibility is to support the clinics with TB.

I: Ok. So now I want to know, what you know about the ASCENT project. Can you tell us what you know about the project?

P: The project I know is ASCENT that is doing, I think there are two types if I'm not wrong, the one of the box and the one of the pill. I don't know what you call it. That one, yes.

I: Is it the label?

P: I don’t know, it has pills and something like that. Yes

I: Yes, ok. Can you tell me how the box works?

P: The box, what I understand when I went to the clinic- but we also went for training, but we also went to the clinic and see how it works. The box works like- if the patient is given the box, to take it home, and when the patient opens the box, the box reports, it reports on a tablet that is held by one of your staff members to indicate that the medication has been taken on that day. Then if that day the box was not opened, it would indicate also and if the box is opened several times, it will also show that the patient has been opening it. It's like the patient is playing with the box or whatever. Or maybe the child is playing with the box. Yes. That's how I know it works.

I: Ok. And then the other method which is the labels. how does it work? The second technology.

P: I don't remember well [laugh], I don't remember well how it works.

I: Ok, that's fine.

P: Because the one that I saw happening in the clinic is the box one.

I: Ok, that's fine. So you mentioned that the box reports if the patient is taking medication?

P: Yes, yes.

I: So who is monitoring that tablet? Do you know who check if the patient is taking medication or not.

P: What do you mean?

I: The device, who is checking if the box was opened or not?

P: There's one of your employees that is doing that on the tablet.

I: Ok. So, who do they work with at the clinic?

P: I saw them, they were working with the TB nurse.

I: The TB nurse?

P: Yes, at the clinic.

I: Ok- ok, so if they see that the box was not opened, what happens?

P: They said they are tracing the patient, they call the patient to find out what could be the problem.

I: So who is responsible for calling?

P: The same guy that works with you.

I: Ok. And what's the role of the nurse in this?

P: The role of the nurse- because when the patient is not taking medication [inaudible segment] because the nurses have their TB files in the rooms. They know who's coming today, who’s not coming today. Then they will be able to know from the tablet, then the nurse will know that the patient did not take his medication? [Inaudible segment]

I: So when it comes to following up with patients, you have mentioned that the intern also calls the patients and what's the role of the nurse, the TB nurse in terms of following up with patients?

P: The role of the nurse is to make sure that the report is being sent to us.

I: Ok.

P: Of the tracking, we have a tracking tool that we use when the patient did not come. They did not take medication, yes.

I: Mmm. Ok, can you explain to us how the tracking tool works?

P: The tracking tool is whenever you call the patient, you have to report that you've called the patient. You are the one that does the phone tracing and the one that does physical tracing. If you have to go to the patient, you also write down on the form.

I: Ok. So who writes in that tracking tool?

P: Anyone, if the nurse called to track the patient, they write. If your intern calls, they supposed to write on the same form, because we're using a universal form for the whole city.

I: Ok, so how often do they send you this form?

P: We want it every month,

I: Every month.

P: When they send different reports.

I: Ok, that's interesting. And so huh in terms of home visits, who is responsible for that?

P: Unfortunately with the city we don't have, we don't have home visits as such, but the partners do help us. We used to work with xxxx (organisation name) which the contract ended in December. They used to do that. Going to homes and we also work with WBOT (Ward-based primary outreach team) from the- from the district, if they're available or attached to the clinic. They're the ones who go for home visits.

I: Ok, so what have been the challenges, maybe the reported challenges in terms of following up with phone calls. If the patient doesn't open the box?

P: Phone calls, if it goes on voicemail, then physical tracing needs to be done, but the challenge is, if the clinic doesn't help, it's not attached to WBOTS, they don't have partners who can assist them, and then we'll lose the patient.

I: Ok. So what has been the experience with the facilities in trying to get hold of the patients, let's say using phone calls, has it been easy or difficult?

P: It has been difficult especially now with load shedding. Network is not good.

I: Ok, network due to load shedding. What are the other challenges in terms of trying to get hold of a patient using a phone?

P: I don’t think there’s any other.

I: Ok. So it's only the load shedding and network.

P: Network problems, if you call the patient and the call can’t go through or maybe the patient lost the phone, you won't be able to get hold of them.

I: Yes- yes. So do they all give correct details?

P: They don't. That's the challenge that we've been having for years. I don't know when that will ever be corrected, patients never give right address, and they never give right phones. He/she will give you a phone now, call it now, it’s not working. But he gave it to you now. And I mean, if somebody gives you a phone number now, you expect the person to have the phone with him, but we call the same number, still it’s not working.

I: Mmm

P: They are giving false information. So that's the challenge we have as a district.

I: Mmm. So what have you done to try and resolve this challenge?

P: This challenge is not easy to resolve, because we were intending as a district if the partners can assist us, if the patient comes today, for medication, then they can go back with the patient at home to see where the patient is staying. Also the challenge with xxxx (province name) is the migrating communities patients are always moving. And we also have people that stay in the streets, they don't have addresses and even if they come to the clinic and go back with them you know he's been sleeping on the corner what- what -what but when you go there, he’s no longer there. Those are some of the challenges we have.

I: Ok. So, can you tell us the benefits of this huh Digital Adherence Technology, the pill box?

P: The advantages of this box is, I went to XXX [name of facility] that’s the first time I saw it. I asked the sister [TB Nurse] does it benefit you and she said it benefits a lot, because most of the patients using the box are completing their treatment. I think maybe the patients are feeling like they're supported. So that is why when they use the box, they take their medication until they finish the treatment.

I: Ok.

P: That's what I've seen. Most of them they finish [referring to treatment]

I: They finish [referring to treatment]. Ok, so do you know how this box is supporting them?

P: I mean, if you know somebody's looking up to you to get your medication to make you better that is a support.

I mean, you know somebody's looking up to you, because when you don't take medication, they are concerned to know why you did not take your medication on that day. This is support.

I: Ok. That's good to hear. So when you first heard about the technology, the box. What did you think about it?

P: I was having no idea what is it, and what does it do, first time when I saw it at the clinic. I was like what is this thing. And they said to me that is a box, and we ended up going to training where they explained about it. So I understood how it works.

I: Ok, did you think it was easy?

P: It’s easy, you just open *mos (though)* I don't need to charge. I understand the battery lasts for one year or something so you don't charge it. You have load shedding, you don't have to worry. Yes it’s always working, it’s not a hustle. You just open and drink your medication. That's it.

I: Ok. So when you attended the training or when you first saw the box did your opinion change? After you saw it.

P: Regarding?

I: Like okay, your first impression with a box right and you're like okay, what is this and then you were told how it works and you saw how it is? Did your impression change about the box?

P: I was impressed, that at least you are doing something for our community to get better regarding TB because TB has been a long term problem.

I: Mmm.

P: Hopefully it will assist TB patients to take the medication.

I: Ok- ok. So huh how did you get to know about the box?

P: I saw it at the clinic one day when I went there.

I: Mmm. And then did you attend any training?

P: We had a meeting last year with xxxx (organisation name), where they were explaining the box, how it works and up to so far what improvement has been done at the clinics, that's where I finally got the information.

I: Ok, so did you think the training was comprehensive and useful?

P: It was.

I: Mmm.

P: Because if I'm going to give you something for the first time and get to know how it works and it was informative.

I: Ok, so how did you feel when you first heard about it?

P: About?

I: The box?

P: I was impressed *gore (that)* at least the partners are trying to assist.

I: Ok. So do you have any suggestions to improve training in terms of you know, the duration of the training and who should attend?

P: No, I think the training was fine. Even the days that they scheduled, and because not all nurses like to do TB, those who are willing to do TB they should be trained on the box, and how it works because they rotate at the clinics, so they should be trained thoroughly to understand what they are doing

I: Ok. So can you tell me how does the rotation work, is it only people interested in TB who can sit in the TB room or anyone can find themselves there?

P: Anyone can find themselves there, but not specifically coerced or pushed to do TB, they rotate at the clinic, they know sister who is doing TB for a year, when the year starts sister who is going to be in charge they change every time because they prefer to stay for a year, because if you for 3 months, you’re still learning TB, others they prefer to stay there forever. So they never want to change, because they interested in TB.
I: Mmm.

P: So, if someone is willing to do something, then you know they will do a good job. You leave them there.

I: Ok. So besides TB- nurses interested in TB and TB nurses. Who else should be trained on the box?

P: And doctors, everybody that works at the clinic. General workers, cleaners, admin, they should understand what is TB, and the importance of treating TB for all the sick people to be cured.

I: Ok. And huh from your perspective as the coordinator, can you tell us the benefits of the differentiated care, which is the follow ups that were happening if a patient does not take the medication? For example, at first there was an automated SMS that would go to the patient, can you tell us what your thoughts are regarding that?

P: That was a good thing but the challenge is if the patient doesn't have a phone, it’s lost, and then they won't get the message. If there’s load shedding, network, it won’t work, so they don't get the message. So those are some of the challenges which are beyond us.and we cannot do more or nothing about them.

I: Ok.

P: But I think maybe if this- if the system allows, maybe they should allow maybe 2 numbers, so that the person that's supporting you should get the message at the same time maybe.

I: Ok.

P: Yes.

I: And in terms of the phone calls, what have been the challenges with trying to make phone calls?

P: It's the same challenges of network and load shedding.

I: Ok. And In terms of home visits?

P: Home visits, the challenge is that, if the clinic doesn’t have WOBOT and the physical training team, it’s a challenge.

I: Ok. And huh have there been any fear or reports of stigma in relation to the use of Digital Adherence Technology?

P: I don't think so. Because you're not going around carrying the box to show everyone that I’m using this box, it’s your personal use. And when you go out of the clinic, nobody can see, they don't even know what that is? So I don't see any stigma. Unless you advertise. Mmm I'm taking TB medication. You know.

I: Ok, do you know how the patient is reminded to take medication with the box?

P: I think it makes an alarm, something like that.

I: Yes.

P: I think it makes an alarm for the patient to remember.

I: Yes. So what are your thoughts about stigma in relation to the alarm?

P: I mean if I’m staying in my house. Nobody will have a problem because the whole family will be knowing that I am taking medication, unless maybe you stay in a group of people that you’re not used to maybe that could be, but now. I don't think that could be a problem. Because if you are positive, and staying with a group of people, all of them have to go for the test. I think they will be knowing that you have TB

I: Ok.

P: And they'll have to support you.

I: Yes, yes and then can you comment on the availability of staff to do home visits, you said you're supported by partners. So are they always available to send?

P: They’re not always available, like if the partner is not there, they’ll be no one to do home visits. They’re not always available.

I: So how often do you get that kind of support? So what's the waiting period? You can have? Let's say you have a patient that needs to be traced physically. How long do you sometimes Wait, what's the longest you can wait for you to get someone to send there?

P: I don’t have a specific time, because if you don't have anyone to go there, then we cannot gauge time. So the patient will only be telephonically traced not be physically traced.

I: And huh can you tell us the positive changes that have been brought by the use of the box?

P: Positive changes that I saw physically, as I asked the sister, she said its bringing positive change because most patients are finishing with their treatment when they're using the box.

I: Ok. So what could be improved? At the level of facility and also district for this to continue? In the absence of xxxx (organisation name)?

P: Without the box?

I: With the box, Yes but without the support of xxxxx (organisation name)?

P: No, I mean, when the partners are part of us, what they are giving us, what they're teaching us. We have to take it over and continue with it. Especially if something that’s bringing positive change in the clinic. Yes

I: So, what is needed for this to continue at the facility and district level?

P: I think supply of the box has to be available to continue.

I: So, who would be responsible for let's say preparing the boxes the charging?

P: If the department decides to use the box, they must take responsibility of payments because the partner won’t be there to support with money and the boxes.

I: Yes- yes. So who should be charging the box? I mean preparing it for use remember it has a battery-

P: It’s the nurse who’s supposed to use it.

I: The TB nurse?

P: Yes, together with the- who do you call them? Your employees that are working with the nurse. What do you call them?

I: The interns.

P: They interns must assist the nurse.

I: if the intern is not there?

P: Huh, then the nurse should know how to do it.

I: On their own?

P: Yes.

I: Do you think they'll have time to do that?

P: The challenge with the clinics now, there's a lot of shortage of staff. So, the shortage of staff is the one that will bring a challenge, but if it brings a positive outcome, we just have to squeeze it in.

I: Mmm. So what do you think can support the nurse to do this? The TB nurse from the clinic.

P: I mean the general worker can assist, the admin can assist, anyone at the clinic who's available

I: And if there are technical glitches with the use of the box or the platform where you see that adherence. How can they be resolved if the department has is taken over?

P: Huh. I don’t know. I'm not sure, because the partner won’t be there. For the box if it’s not working, but if there's a supplier, who’s supplying the box, they would be able to assist with that and service the boxes if they need to be serviced.

I: Ok. You think the supplier would be the person?

P: Yes.

I: Ok. … Has there been any reported challenges with the use of the box?

P: Never, I've never had any report. So there have never been in a challenge.

I: Ok. So we do have some groups of people let’s say who are homeless or who drug users are. Have you had any reports about them using the box?

P: No. I've never had any report.

I: Ok. So when the department takes over. Who do you think should be responsible for training The TB nurses and people interested in TB and all those general workers you suggested that should be trained?

P: The department has to continue to take from the partner and continue doing training.

I: So who exactly in that department?

P: In the department?

I: mmm.

P: It should be training department.

I: Ok.

P: Yes.

I: Is there an existing training department?

P: Yes, We do have.

I: Ok, alright. Huh so do you think this use of Digital Adherence Technology has improved the relationship between the patients and the healthcare workers?

P: Yes, it has, because remember if you see a patient today, you might not even call him after 2 months. You only see patient after 2 months, but with this it makes you to always be aware that XXX [referring to a patient] is taking her medication, XXX [referring to a patient] is taking her medication. So, the relationship improves and also when it’s late maybe they did not take medication and you call the patient reminding them that builds a relationship.

I: Ok.

P: Yes, because maybe the patient goes to work and takes the box with him, that day they forgot to take the box.

I: And huh, can you comment on the healthcare workers workload when using this Digital Adherence Technology?

P: *Aowa (No)* I think workload doesn't become a lot, because the patient is not specifically at the clinic. You only contact the patient through the phone.

I: Ok

P: You will only check on the tablet if they’re taking medication. I don’t think it increases workload.

I: Ok, And huh in terms of our patient’s adherence, can you comment more on that one? The use of the box and adherence.

P: The patients adhere more when they’re using the box, because they are aware that we have this box. I have medication in it that I have to take meaning it makes the patient take responsibility of using the box to take medication. I think helps them to remember to take medication.

I: So, you mentioned one clinic that you saw using the box, is there other clinics where you found them with the box and do you know the experiences with that box?
P: XXX [clinic name] and XXX [clinic name] told me they are using it but I didn't find them with the patient who's using the box, but they do have clients using the box.

I: So, in your role as a coordinator, have you by any chance encountered a patient using the box and you managed to talk to them about it?

P: No. I didn’t meet the patient.

I: And can you tell us maybe gaps you think are existing in the way the intervention is being delivered at the district?

P: By?

I: The way the intervention the implementation was being done? Are there any gaps? You think are existing that and can be resolved?

P: I did not identify any gaps.

I: Huh, do you have any suggestions for improving the device, the box itself?

P: No, to me I see it as a good box. So, I don't have any suggestions on what to remove, what to add, no.

I: Ok and also in terms of the platform that's used to show the adherence. Do you have any suggestions to improve it?

P: Mmm –mmm, no suggestion.

I: Ok, and in terms of differentiated care, those phone calls and follow up visits, what can be improved?

P: Nothing.

I: Ok, so is there a method you use that is being used by the facilities to capture any challenges or successes with Digital Adherence Technology with the project?

P: I'm not aware of any, where they record if they have challenges because we've never received anything regarding challenges, because the only tools that we have is for tracing, the phone. But with the technology, no we don't have.

I: So, is there an existing tool or system that's- that can be used to record the challenges and successes?

P: No, we don’t have a tool like that.

I: Ok. So if the department is to implement or to roll it out, how do you think they can be reporting challenges and successes?

P: I think they can create a tool for that. So that we track if this technology is helping us or not.

I: Mmm.

P: Yes.

I: Ok …… So do you think huh the use of the DAT’s, these boxes have improved outcomes? Have you seen any change?

P: With the clinic that I asked, they said yes, it has changed. It has improved treatment success it was lower, and it went up.

I: Ok. In terms of continuity, you mentioned that okay, the devices are needed for the project to continue and staff are also needed, what else is needed? Because tablets are currently being used to monitor the patient. So what can be used to monitor patients?

P: You can also use maybe your own phone.

I: Personal phones?

P: Maybe there must be an app that even the sister can use on her phone.

I: Ok, so what are your thoughts about patient- sisters, or nurses agreeing to use their own devices for that?

P: They are using their devices, because I also created a TB WhatsApp group, it’s their phones that they're using and we’re using WhatsApp for all the facilities, so I don't see them having a problem with that.

I: Ok.

P: Because it’s part of work. I mean, putting an App in your phone, it's not a hassle.

I: And in terms of data, are they provided with Wi-Fi or data?

P: They are not, it’s Just like when you open your WhatsApp *mos (though)*, maybe they will do an app that doesn't want data, you only open the data, but it doesn't use data. I think that one could help

I: Ok. So, you're saying an App which does not need data to be used, can be used by- by healthcare workers with their personal phones?

P: Yes.

I: Ok, alright. So what are your comments or your concluding comments? About this box and the Digital Adherence Technology and the follow ups that were happening? If the patient does not open the box?

P: I would say we are living in the years of technology, so our mind set have to change to improve. So this box is good. I think they should be continued. The department should consider using it, because it has improved the clinic that I’ve asked, it has improved adherence. My conclusion is, I would vote for it to continue being fully used.

I: Ok.

P: Thank you.

I: Thank you very much for the information.

P: Pleasure.

I: The time is: 11:59.

GLOSSARY

*Mos (Though)*

*Aowa (No)*

*Gore (That)*
