# Supplementary material for: Acceptability of Digital Adherence Technologies to support people with drug-susceptible TB in South Africa
Source: PLoS One. 2025 Sep 24;20(9):e0332103. doi: 10.1371/journal.pone.0332103 (PMC12459780; doi:10.1371/journal.pone.0332103)
Supplement: S4 File — (ZIP) [file pone.0332103.s004.zip › S4 Transcripts/HCWs and Stakeholders/IDI 32-STK.docx]

**TRANSCRIPTION NOTATIONS**

| **Label Key** | **Meaning** |
| --- | --- |
| **I** | Start of each new utterance by the Interviewer |
| **P** | Start of each new utterance by the Participant |
| **N** | Note taker |
| **{ }** | Indicates that details were changed or pseudonyms were used to anonymise data |
| **( )** | Indicates the description provided to anonymise data |
| **XXX** | Words were omitted to anonymise data |
| **-** | Breaking into a sentence by the next speaker |
| **…** | Pause or drawn out words |
| **[ ]** | Indicates noise made, e.g. [laugh], [sigh], [pause] |
| ? | Beginning of utterance by unidentified speaker or questionable text |
| **[inaudible segment]** | Unclear section of the recording |

I: Thank you so much for agreeing to participate in our interview. I would like you to allow us to audio record this interview?

P: Yes, you can audio record it.

I: Ok, thank you. Date of the interview is: xxxxx (interview date). Location: XXX (name of the facility). Language of the interview is: English. PID number is: Stakeholder Qualitative interview 006. Huh, the time of the interview is: 12:09. The interviewer is: xxxxx (interviewers name). I: Alright ma’am, thank you so much for agreeing to participate in the interview. So can you please tell- tell me about your current position?

P: My current position is; I’m the HAST (HIV, AIDS, STI, and TB (HAST) xxxx (position) of the district in XXX (name of the district).

I: Ok. What are your main responsibilities as a HAST manager in terms of TB?

P: In terms of TB, my main responsibilities are to monitor the sub district or at facility level, that they are implementing the policies accordingly. And then my main thing also is to make sure *gore (that)*, which facilities are using this digital adherence technology, so that we can be able to see whether it's working or not. And then also we need to make sure we follow up on the using of this digital boxes, whether is it efficient to patients at facility level or not. So, and then are they seeing any improvement in that, so that it can be able to- at least we can recommend it in future. So the most important thing also to make sure that patients that are on digital adherence technology boxes are using it, if they are using it, is it very efficient, in a sense that patients are adhering to treatment.

I: Ok, so with regards to what you just told me, that you are checking if the facilities that are using the box, is it efficient? So what is your experience about that? Is it -is it doing what you thought it's supposed to do?

P: Yes, according to myself, it is doing what it’s supposed to do, remember XXX (name of the district) has a lot of mines, it has a lot of rural settlement, informal settlements, also where we experienced a lot of TB in- in those settlements, informal settlement, so it is very efficient in a sense that we have realized, when we analyse our data on a quarterly basis, we can see that we have a reduction in- in lost follow up as an indicator that we are reporting for the MIP? So we have seen that at least there is adherence, follow up on patient’s are being done on time. And then also, we have a lot of improvement. So our lost to follow up indicator has decreased a lot.

I: Ok. So it's the lost follow up that has decreased, and what else did you notice?

P: Huh, lost to follow up has decreased and also we have huh- huh there’s an improvement in the outcomes *tsa (of)* huh our- our... We report APP. I have a mental block. We have the lost to follow up. We have the outcome of TB treatment success rate. Yes, I wanted to talk about treatment success rate, it has improved. A lot.

I: Mmm

P: That is why I'm saying this digital technology is assisting us. So I feel really patients are adhering to their treatment. This adherence is really assisting, due to these boxes.

I: Mmm

I: So what is the most important thing about this box? Like I want to understand as you’re saying that treatment success rate has improved. What is the most helpful thing about this technology?

P: The most helpful thing about this technology, is that patients are adhering to their treatment,

I: Mmm

P: and then the other point, it reminds patients about the time of which it has to be consistent.

I: Mmm

P: Patients have to take on regular times. It reminds patients and you can hear from what the interviews that I made with facilities that patients are very happy with it, even though you have gone out somebody can remind you. The one who is supporting you or a family member, or even if you can carry it along even if you go to town, even if you go anywhere, so, I think this box is really assisting patients from my view, patients are happy about this.

I: Ok.

P: Mmm.

I: Alright, and how do you feel about this box? As a HAST manager from XXX (name of the facility).

P: Huh, my feeling is- I think this box can as a support from you, ASCENT can just continue or can be -can be supported to continue using this digital adherence technology boxes. So that most of the patients can- can- can see the need of adhering to treatment. And then also my view is, if it can be used to each and every patient, we are going to see a healthy community in regard to TB huh-huh treatment or in regard to TB as a program. So it will assist a lot if we can be able to use it to all patients, reminding patients is the most important thing, because most of them lack adherence to time, as a consistent something. So if you can utilize this, the whole of XXX (name of the facility) it will really assist in our outcomes.

I: Ok. So you’re saying we need to use it to all patients?

P: Yea.

I: When you say all patients, what do you mean?

P: Patients who are on TB treatment,

I: Ok.

P: Not everyone, but all patients who are on TB treatment. It will really assist us, and also it will really assist us to make sure patients are being followed up on time, patients are being huh-huh-huh seen on time at facility level. So as much as the system is assisting, it’s going to assist us. I think that’s my view.

I: Ok.

P: Yea.

I: Alright, is there any difference that you have noticed in terms of treatment success rate and adherence before we came with the program in the district and after? Is there any difference between-?

P: Initially we were thinking *gore (that)* this box or digital technology boxes are going to waste our time. Because we've been having a challenge in lost to follow up here and there. But since it was used or the project was implemented. We have seen a lot of difference. XXX (name of the facility) has never had a treatment success of above 80, of which now we are above 80, with the treatment success. And then XXX (name of facility) at least has never had an outcome of to follow up of below 10. As we're speaking from the whole of 2022 up to date, we've been performing well. With above 80 and we’ve been performing well with below 10 with the lost to follow up. So it's very important for us. So implementation of this ASCENT really assisted a lot, on improving the outcomes,

I: That’s really nice to know.

P: And with the death rate also has decreased in XXX (name of the facility).

I: Mmm.

P: Death rate, so people are adhering due to this box. So the death rate has decreased even though it is not a huge decrease, but we have decreased a lot in death rate seeing that adherence is very important according to patients. also the health professionals that are- that are monitoring this, say this has really impacted much on their performance or on their monitoring for this program.

I: Ok, alright that's very nice to hear. That's very nice to hear. So you were saying you are a HAST manager? For how long have you been in the position?

P: I've been in the position of Hast manager for only a year, but, I came to the district in 2021 March, where I was called to come and assist the TB program. So initially I was a PI before Hast manager, and I got another post. So I was just considerate on TB in the district.

I: Ok

P: So as a Coordinator previously.

I: Ok. Alright. That’s nice to hear, huh I would like to know, what would you say to someone who doesn’t know about this ASCENT program? If you are to tell your colleague about this program. What can you highlight to them?

P: What I can highlight to them is that, an ASCENT project is about huh monitoring patients whether they are taking treatment at home or whether they are adhering to treatment according to time consistency, and I will them that there is a box, there are slips but we are not utilizing that method. There is a box that is being used, it is a system that is being used to patients to remember or at what time to take treatment.

I: Mmm.

P: So that box is a system that will be ringing at a certain period of time, so that the patient can be able to remember when it’s time for taking treatment. And also the system is linked to the facility, when it’s linked to the facility, there is someone, it might be a TB focal who is monitoring the system at facility level. So to see whether this patient is opening the box on daily basis, whilst opening the box, they will also find out that the patient is not just opening the box they are at least taking their medication. So on the system it will just reflect green if the patient is taking medication accordingly, but when it’s red, it will alert the person monitoring at the facility level that there is a challenge here, that is when they will be able to say can we call the patient and find out if there is a challenge or send someone to go and see if there is any challenge on the box or the system is functioning well or it’s not functioning well.

I: Ok. Very nice to hear all of that. So in terms of differentiated care. What do you understand?

P: Mmm, about differentiated care, we talk about adherence *akere (right)?*

I: Mmm.

P: So this huh- huh model, differentiated care is that we need to make sure that we monitor patients at the level of the community, where at least we check if patients are taking treatment at home, how well are they taking treatment. And improvement in that, and then the adherence also is the most important thing. What support are they getting at the level of huh- huh the community and also huh how best do they think they are being assisted.

I: Yes, so In terms of differentiated care model, if the patient is not adhering on treatment, they get an automated SMS, they also get a call,

P: yes,

I: And we also do a home visit. How do you feel about this kind of support that we give TB patients?

P: This kind of support is very good, in a sense that even for those patients who don’t have Cell phones, someone in the family, if he/ she has a cell phone can also assist on the SMS’s, that I got the SMS. did you manage to take your treatment. So I should really think that it assists, it really gives us the follow up for the patients, it makes it easy for us to make sure that as CHW’s are working with interns at the level of facility, TB focal are able to follow up on the patient on time.

I: Mmm

P: And also the action. Acting on time, whether the patient is not getting any medication.

I: Yes. So as we are reflecting a lot on the support that we give patients, like your calls. Home visit. What are the challenges that the team are experiencing in terms of these follow ups? like your calls and home visits? Are there any challenges that you can reflect on?

P: Yea. There are a lot of challenges in regard to patients being followed up,

I: Mmm

P: the first challenge is that, our patients as we know are not giving us the correct contacts or telephone contacts or sometimes they don't have phones.

I: Mmm.

P: For us to follow them up, and then also a challenge of following those patients up, where there are informal settlements that don’t have any streets that are not having addresses, such is very difficult if we don't get the patient on the phone for us to follow up with the Sister to follow up the patient, we will be having difficulties in locating the patients, and then also giving the addresses, addresses are difficult to follow up. So those are the challenges that we have. So, it's not a usual thing, but those are the challenges and also what I can say, some of the patients as we are on mines, it’s very difficult to track them, in a sense that some of them would be leaving the areas where they are leaving, because they are here only for work,

I: Mmm.

P: So those are the challenges that I can highlight.

I: Mmm, So as a Hast manager, what is your responsibility in terms of these follow ups? What are your responsibilities?

P: Huh as a Hast manager *akere (right)* we are analysing data, we need to find out if there is any collaboration at the level of the facility, between the CHW’s that are following up patients, the TB focal or the nurses, even the interns, Are they collaborating together for follow ups. And my responsibility also is to make sure that the patient doesn’t miss treatment, are they drawing the reports of the missed appointments from the system so that they can see which patients are being followed up on that day or on daily basis or on that week.

I: Mmm.

P: So if they follow up on these policies in regard to WBOT and the facility that is making sure we have implemented the policies accordingly.

I: Ok.

P: Yes.

I: So how does this ASCENT program assist the facilities in terms of follow up?

P: In terms of the follow up, the ASCENT assisting, when the patient is just opening the box we will see.

I: Mmm

P: Because of the duration of time,

I: Mmm

P: it will highlight us on the system. The person who is in charge will see that there's a challenge here, we need to follow up this patient. Also, if the patient is not opening the box, there will be a challenge, we need to see what the challenge is really. We need to follow up this patient. We need to send somebody who’s tracking telephonically, we need to send somebody to check on what is really the challenge.

P: So the ASCENT box is really assisting us to act on time.

I: Yes. Thank you so much to hear that. So when you - when you first heard about these digital adherence technologies, what were your expectation about this program?

P: Huh My expectation about this program was huh … we need to have outcomes that are beneficial to the patients into the health system. We need to have our- our program being monitored in- in a manner that would assist us to make sure that the patients are adhering. So the other expectation that I had, is that this system will improve our outcomes.

I: Mmm.

P: Mmm.

I: Ok. Were your expectations changed?

P: Huh, my expectation did not change, it did not change. But what we expected to see has really assisted us, because there's an improvement in the outcomes,

I: Ok. Alright. That's nice to hear that you expected that it would change outcomes and for real the program changed the outcome.

P: The program changed the outcome it really changed,

I: Ok. All right. So can you please describe the training and resources that staff received on delivery of DAT? Including these follow ups. What resources did you receive?

P: people were trained on the level of facility,

I: Mmm

P: The coordinators, even ourselves in the district we were trained, ASCENT project managers did give us training, and resources that we got, we had interns that were hired at facility level contractually, and that were assisting the DAT to at least be monitored and function well or to be implemented well, also we received the boxes that were assisting patients to take their treatment or to adhere to treatment in their homes. We received also the… I said there are interns and the boxes, we received the tabs, and I don’t know what they are called-

I: The tablets.

P: The tablets to at least assist the TB focals to at least to monitor whether the system is working at home or not.

I: Mmm, ok. So was the training that you received comprehensive enough for you to understand the whole program?

P: It was very comprehensive in a sense that it- it was simplified more simplified for us to understand on the how it is working. So, it was easier for us at least you can just be able to utilize it and then those who are not informed can be able to, the way it was simplified you can be able to tell a person that this is how this system is working. Very simple.

I: Mmm, Ok. Do you remember some of the activities that you did on a training?

P: Huh, some of activities that we did on training. What I remember is were shown the box. we were shown how its functioning and then we were shown at least an example of the system when the patient is not utilizing the box, what colour do you see on the system, when the patient is utilizing the box the colour that you see, then you can be able to monitor that. and then also when it will show you not show you specifically, but make you huh- huh act firstly so that you can be able to recognize whether the patient is adhering to treatment or not,

I: Ok.

P: the box mostly the functioning of it, that's what we saw, it was practically done

I: So in terms of those colours that you are telling me about. Can you tell me what you can still remember about those colours and what do they mean?

P: Huh If I can remember well, I know it was red and green *ne (right)*?

I: Mmm.

P: I don't remember any other colour.

I: So what does that red mean?

P: red means maybe the patient is not taking treatment accordingly. So you have to act and find out earlier what is the real challenge. And then also red if I am not mistaken, it will show you if the patient is just opening the box not taking treatment or not. Then the green will show you this patient is adhering.

I: Alright. Ok, that's very nice to hear that. I can see that you do have knowledge of the program.

I: and was the training sufficient and useful for you?

P: Very sufficient,

I: Mmm.

In a sense that implementation was easy at facility level at facilities that are implementing,

I: Mmm

P: and also it assisted the coordinators to at least be able to manage the program well.

I: Ok.

P: In a sense that sometimes coordinators are busy, they cannot go to each facility. So the system that is at the facility will just be able to get the progress,

I: Ok.

P: very sufficient.

I: So what suggestions would you give us in terms of improving the way we train- we train staff on ASCENT?

P: Huh the training on ASCENT, what can improve is- But before I say that what we can improve on *ke* (*is*) the how we monitor? I wish you could have a lot of staff just too at least monitor, like you're having maybe interns to monitor whether the system is functional or not functional and how fast can the system be turned If it's not functional, and then maybe improve, also I think training staff, also mentoring. Close mentoring is needed in all the facilities or maybe increasing how the mentoring is being done, like in a sense that close… monitoring in those facilities that are implementing.

I: Mmm.

P: Yeah to make sure everything's being done according to the policy or according to DAT.

I: According to DAT. Ok, so in terms of the training, so who should train? Or mentor as you’re saying?

P: Huh I'm not sure who should be training, the training being done by you as the project managers is good, but interns- interns must at least be able to understand the training especially at the level of CHW’s, at the level of facility, they need to know what is it that they are doing, what I realized most of the facilities said interns made it very easy for them to understand and implement.

I: Ok, alright. Who should- who else should be trained on the program in the facility level?

P: In the facility level, support staff, I should think this program must be known by each and every staff in that facility, professional nurses, CHW’s, even the admin clerks they need to know that there is this project or there is this program that they need to know. So I think in meetings during facility meetings they need to train each and every staff, as long as their operational managers are well conversant with this program, they need to tell people and train everyone in the facility, so that even if some of the people are not around, everyone should be able to say there's this project that is being used here it’s so nice they are using the boxes, the boxes are assisting the patients to remember to take treatment so you can see patients are happy about it.

I: Mmm

P: So staff members, all of them in the facility need to be trained, they need to be given information about DAT.

I: About this program, where can we train them? At the facility?

P: It’s good at the facility level.

I: Ok.

P: I think training of the ASCENT project cannot take more than 30 minutes because, it's simple- simple

I: Yea.

P: and it’s sufficient enough to give information that is needed, so everyone else should know, it’s just like the ideal, ideal needs everyone from the gate until at the facility level operational manager need to know. So with this project, everyone must know there’s this project and if everyone knows, CHW’s, other professionals, ENA’s at the facility, will be able to cascade the information at the level of the community, so it will be easy for the treatment adherence.

I: Alright. Nice to hear that…Ok, alright. So you said a lot about how the differentiated model of care benefits, and how- how this whole program improved the adherence and the outcomes. So I just want to know now from your knowledge, what are the benefits of the -of the box itself from the [inaudible segment] How is it benefiting patients?

P: Huh the box is benefiting patients in a sense that it assists in adherence. The box is benefiting patients in the sense that they get their treatment on time, on regular times, that is the benefit.

I: Mmm.

P: And also the box is assisting on the treatment success, whether the patient has- what do you call it, the patient has completed treatment or successfully being treated through sputum, if they were taking sputum, that’s the benefit that I see. So under G it assists the patient to just get- get healed,

I: Mmm.

P: *Ya* (*Yes*) from TB. And also because every time when he or she is reminded about treatment they won't be able to skip their medication.

I: Yeah.

P: that is the benefit. And then also the benefit of this box in regard to us as the health care providers, it assists us to monitor our, our- our program, or our patients, whether they're taking treatment on time or whether they're taking treatment whilst they are at home. That is the most important thing, also with the system it assists us to act on it on time –

I: Mmm

P: act on time before the patient can default treatment or not. And also with us it can assist us to improve our outcomes, lost to follow ups and the success rate. So at least we are going to have a healthy community that is free of TB, with the very same box that we’re utilising.

I: That's very good to hear. So, earlier on, we spoke about the challenges of differentiated care, which is the follow ups, calling patients that got wrong addresses. So are the challenges of this differentiated care whereby you have to go. So now I want to know. What are the benefits of supporting patients in terms of the follow ups?

P: That project?

I: Yea, the follow ups

P: Follow ups huh- huh benefits the patient, it makes sure that the patient is able to have the pills counted, that am I doing well or not on taking pills and also the following up benefit, if you don't get the patient on the phone, collaboration with the Sister and the interns are able to make sure that they follow up on the patient they go to address if they didn't get the patient at least the patient can be tracked especially from the neighbours if you didn’t get the patient, but something will come out of that. That’s the benefit at least.

I: Ok, the support?

P: The support

I: Ok, alright. So in terms of the challenges with this box. What are the challenges that you think are there when we are using this technology?

P: The technology is good, but what I’ve seen is that patients have tricks

I: Tricks?

P: Yes,

I: Ok, can you talk about tricks

P: The trick is, patients can just say I am taking medication, so as for the box challenge. I have noticed or I have heard from the facilities that some of the patients can just open the box as if they are taking medication and then if they understand well they can at least stay for a certain duration, so that you cannot recognise if they opened the box for a small duration. So it means the patient is not taking medication. So patients have a lot of tricks of not taking medication.

I: Mmm.

P: So that one they can do it, even though it’s important to keep on health educating the patients about the importance of taking medication, how the box will assist them not to forget.

I: Mmm.

P: So that will be very important for us to maybe emphasise on that.

I: Ok. I get that. So besides patients not opening the box or just opening so that they are not called. What are other challenges or problems that you think are related to this program?

P: Huh the other challenge is that even though we will be monitoring if the box is being returned, when they have completed treatment, some of them can just … loose them, others can go with them… or without even returning it, a person might be relocating, you can just travel with it, even though you have health educated them as much. So those are the things that we can at least say can be a challenge.

I: Ok

P: or misplacing or patients who are moving up and down. Maybe leave the box from where you are staying.

I: Mmm

P: Those are some of the challenges.

I: How can we improve those things? Starting from the one that the patient is just opening and not taking medication and this one of patients not returning the box?

P: Huh about this one where patient's opening and not taking medication, we can- we can make sure we emphasize on the importance of taking treatment, then we can make sure we tell patients of the complications of not taken treatment. If you are not taking treatment this can happen to you, so for your own health, make sure you are utilizing the box as it its assisting you to adhere.

I: Mmm

P: So it's very important, but for- for traveling or for losing the box or misplacing them. You need to make sure the patient is making sure the box is the first thing as is their life.

I: Mmm.

P: So they make sure where ever they go make sure they don't lose the box. And then they make sure that the box is returned on completion.

I: Alright. That's very nice to hear. So in relation to stigma, I just want to hear your thoughts about stigma and using this box. What are your thoughts around that?

P: About stigma, there is not much about stigma, because they said… huh… the box is just normal to carry around, because it’s not so heavy. But huh for the patient who is well informed. I have not heard much about stigmatization about the box.

I: Ok.

P: Because carrying it along is like carrying anything else that belongs to you everywhere. So most of them say, no it’s just like a box of pills, when somebody asks you, it’s a box of pills. I carry it where ever I go. So stigmatization because it’s a close something. I heard it’s not so much.

I: Ok. So from your personal experience or personal opinion, do you think boxes might have something to do with stigma or you don’t think they can?

P: Boxes don’t have stigma, boxes are assisting, as long as they are covered, they won’t have stigma. My view is; most of the people are now buying boxes for pills wherever they go, this now has a system, yes, it’s a little bit bigger. But I don’t think it will have stigmatization due to the box, but if a patient is well informed, they can say this is a system that is assisting me to take my medication, so I am not ashamed of it.

I: Mmm, alright. That’s nice to hear that. So from your perspective as a health care worker, can TB be improved by using these technologies?

P: It can- it can really especially these technologies that are coming. I know they can be improved much better and better, but for this DAT, I know TB program can be much improved, for what we have seen in XXX (name of the facility) we have mines,

I: Mmm.

P: There is a lot of TB and if at all we are succeeding in treating these patients with these methods or systems we going to improve.

I: Mmm.

P: In improving the program.

I: Yea, ok. Do you think personally that DAT has got an impact on patient’s treatment adherence?

P: Definitely, this huh… huh this system really has an impact on adherence,

I: Mmm

P: We know as individuals we have own views or our own things, but since this DAT method is used at facilities that use it, it has an impact on improving the program really. So I should think for a start this technology will be very huh… important to assist in our adherence with patients.

I: Does this technology also improve the relationship between the nurses and the patients?

P: That is the most important thing. This technology also improve the relationship between the nurse and the patient in sense that, they when they come back the health care provider can be able to ask that how do you see this box as assisting you. In most cases that I’ve had, the patients are happy with this box, this box is really assisting me, and sometimes I don’t have a watch. I don’t have a phone, those who don’t have phones can be reminded by the support system of their families or whoever, but also even if there is no support, they said still it’s very helpful. When it rings it clicks on your mind that this is the time for my medication, so they won’t be able to miss their doses.

I: Mmm- mmm. That’s very good to hear ma’am. Huh did it improve the monitoring of the patient?

P: Exactly, monitoring has improved a lot, especially to us because since we are unable to make sure that patients are taking treatment at home, before this DAT system. Now at least it assists, also with the system, because we were monitoring manually, whether the patients are taking treatment at home or whether they are adhering on their treatment, but seeing the system that we have linking to the patient at home, it really assists on monitoring and also assists the health care providers, at least they make sure their checking the patient on regular basis, and adherence of patients to their treatment.

I: Ok, that’s very good to hear. How do you think these positive things, these positive changes can be sustained?

P: Mmm my view is; I hope the ASCENT project can continue- can continue- can continue until we make sure that we have fought TB.

I: Mmm

P: Huh In our community, this digital adherence technology I think let it continue, we must not leave it. We must make sure that we are going to have a healthy community that is having huh… less TB.

I: Yea, so in the absence of xxxx (organisation name) team. How do you see this program going?

P: Huh the absence of xxxx (organisation name) team. We need to make sure that as you are assisting us and mentoring us that we have people who can be able to continue with this project at the facility level that will monitor, that will make sure that boxes are being returned, that will make sure that patients are being taken care of at the level of the community,

I: Mmm.

P: So without xxx (organisation name). We will have to choose or we’ll have to delegate people who will work on this project at the level of the facility.

I: Mmm, otherwise is it possible for you to run on your own?

P: We can run on our own in future as DOH, if we have people at the facilities that are well trained, like you have trained people.

I: Yea. So who are these people that will make sure that everything is in order? Who are those people that you can delegate, like you said *ukhuthi* (*that*) As long as you delegate people that are going to make sure you run things smoothly. Who are those people?

P: We have- we have TB focals at the level of facility, those are the people who will be monitoring, but they will work together with the ward based outreach teams. So that they make sure that this system is functional, following up will be done by the ward based outreach teams, and then making sure that the resources are being well maintained and systems are being well monitored, patients are coming back to the clinic TB focal, since each and every facility has a TB focal, they’ll be able to do this

I: Ok. So at a higher level who can make sure that the program runs smoothly?

P: At a higher level at the district, we have a person who is known as a coordinator of TB

I: TB Coordinator

P: At the level of the district, they are called Program implementers.

I: Ok.

P: At the level of the district. So these people have to monitor through reports, they have to monitor through checking at the facility level that telephonic through the Coordinators how the system is, as long as they send a report at the level of district and make sure that this system is functional and still maintained

I: Huh, ok, and those you call TB Coordinators-

P: TB Coordinators at the level of district.

I: District level?

P: They are program implementers.

I: Ok. So those are the people who need to be taught and be equipped to make sure the program runs smoothly?

P: Yes, Be equipped to monitor the district

I: Ok, that’s good to hear. Please elaborate on the negative changes of the program. What are the negative things that you think the program has in the district?

P: Huh… The negative things… What do you call the negative things the program has on the district, you know huh we don’t- even though we want to have focals *ne (right)*?

I: Mmm.

P: We need to inform everyone like I said previously,

I: Mmm

P: *Gore (For)* this program, because you know with TB initially, people were having this negativism that they will have TB if they assist patients at the level of facilities, so we are taking out this mentality as long as we have this system to take out this mentality, because we know that we have a problem that TB patients are very difficult to manage, so with the DAT system, people are seeing that systems are being brought to improve TB management at the level of facility, it will change their mind-set. So I should think systems and changes that are coming will make sure that people are able to assist the program so that it improves in future.

I: Alright. So earlier on we spoke about TB coordinators that you said can be mentored to ensure that all facilities have got the box and everything, are these TB coordinators per sub-district or they are per facility?

P: Yea, there are TB Coordinators per sub-district.

I: Ok.

P: And then we have TB Coordinator overall in the district, they call that person Program implementer who will make sure that TB coordinators are monitoring at the level of the sub-districts, those at the level of district will make sure that TB Coordinators at the level of facility are doing their part.

I: Ok, it’s not Hast Coordinators?

P: No, it’s not Hast, here in North West we don’t call them Hast managers at the level of huh- the TB implementer or TB Coordinator at the level of district is reporting to Hast. Also hast would need to know what is really happening as Hast manager per program.

I: Ok.

P: Inclusive of TB.

I: At the district?

P: Yea

I: Alright. Now I get you very well. So please describe to us what system level structures need to be improved in order to integrate the ASCENT program. What can we do to integrate the ASCENT program with what is happening in the facility now to make sure that it's efficient? P: it's efficient?

I: Yes. How can we integrate what is happening with this ASCENT?

P: And I don't know if I understand in integrating this, we need to make sure that all people at the facility level know the program of ASCENT for people to be well conversant with it.

I: Ok.

P: So then we need all the stakeholders to know about this ASCENT, including maybe NGO’s that are assisting *ka (with)* TB program, there needs to be inclusive collaboration, then we need to make sure that W-Board is well conversant with this program. So we would integrate this ASCENT project seeing how well it’s functioning will assist us when we have at least in cooperated everyone in the program.

I: Ok, so I terms of the systems now. I understand *ukhuti (that)* there is a system running; Tier *ne (right)*?

P: Yes, In terms of the system, huh it’s nice because the system didn’t change anything, but what will assist us is; if patients are being monitored well with this system that we have, the DAT system, it can be- the DAT system can assist us to update the file.

I: Mmm.

P: The file is being captured on the system of Tier, so every time when they monitor we won’t have a back lock on updating the system, so when the DAT system is monitored, the file is being updated, the file is being used to capture on the system of Tier. It will mostly assist, it’s very efficient

I: Huh, so they can work?

P: They can we work hand in hand with this system

I: With the systems?

P: Yes, with the systems,

I: So can we be able to integrate the DAT system to the Tier? To sort of join it

P: That one I'm not sure off, but if it's an efficient system, then the province will see to it that, because I don't think the activities that are in Tier and DAT are much different because Tier is used to manage patients, but DAT system is assisting us to efficiently manage the patient at the level of the facility. Because with Tier system if you are not having a file that is updated, then you are not having a system that is workable or a system that is sufficient.

I: Mmm.

P: So I should think in future if we-we can be able to-to speak about how best can these activities be collaborated or integrated due to the reason I have given. I think the province will recognize that.

I: To integrate the 2 systems?

P: Yes, to integrate the 2 systems and make it 1.

I: The DAT and the Tier systems?

I: Why do you think it will be beneficial to integrate the 2?

P: It'll be beneficial. Sometimes it's not good to have too many systems.

I: Yea

P: Sometimes having many systems can make a person to lose focus, but if the systems are integrated and see how best they can do to assist. The reason I'm saying – we are utilizing Tier for laptops and desktops and this DAT system is movable, you can carry it and everywhere you can check everything with the tablet, so I think you can come up with something that could make them integrate. So that even if you’re not in the facility but you are able to see what is happening with a patient, off which with Tier you won't see anything if you are home.

I: ok, you only have an access-

P: Only when you’re at the facility.

I: which is the difference with the DAT, because you can just see it on your phone.

P: Mmm

I: Alright. So it will be beneficial if we find a way-

P: Find a way of integrating it, it would be beneficial.

I: Ok. So what- what are the systems in place that could monitor differentiated care in the facility? How can we monitor the ASCENT program? If it’s doing well or not.

P: Through delegation of a human resource that is relevant to just give feedback on weekly or on monthly basis.

I: Ok.

P: And then also, if you can have huh- huh a lot of DAT systems that are being used in each facility to have that, it can be beneficial

I: if the facilities have got what?

P: If each facility who are not on the project have all these resources like boxes, the systems like the tablets. And have a Human resource that can monitor even at the level of facility that are delegated.

I: Ok, alright… (Turning page) So I also wanted to know, how can we- how can we document the challenges that we have with the DAT? If we want to monitor if the program is doing so well, how we can we document, how can we record the challenges, the success

P: I would think there’s a need for the tablet, the system has where you comment?

I: Mmm.

P: I think we need to have a monthly report where everyone can be able to report to the district that these are the challenges that we had for this month-

I: Mmm.

P: About the DAT system

I: Ok

P: So, then maybe we can be able to have interventions through assistance of xxxx (organisation name) or through us, especially if we are well conversant like I said, especially if it's on our hands, we can be able to make sure that it’s user friendly for us.

I: So it will be good for you to find a way of documenting and report the challenges.

P: So monitoring every month, if they tell us the challenge that they have with the DAT system, then there's always a way of how can we fix this challenge.

I: So it's something that needs to be reported?

P: documented and reported.

I: then even in terms of success as well? That how many patients have been given the box

P: Yes- yes, so that through DAT we have achieved that out of so many patients, these are the patients who have been using the box and then this is the achievement that they have successfully been treated, then on some days, we can be able to go present it to our principals at provincial.

I: Ok, alright. Thank you so much for those inputs. Can you please describe any gaps which exist in the way the intervention was being delivered? Any gaps that you identified in how we have implemented the program and how can we improve?

P: Huh, the gaps that can be identified, let me say it’s huh…huh… if the project is new, not all of us have trust in the project, we will need to see how best it's implemented so that we can- we can we- can be able to see its impact. I think we don't have a lot of boxes, but what I can say about a lot of gaps- what I can say about this is… Let it not be implemented in certain facilities. I think every facility would have tasted on how is it being implemented so that- huh so that even if it didn't work or it has worked everyone should be knowing what to do. I wish it could have… I know there are few facilities that are implementing, I wish it could have been each and every facility that tried this, not a certain portion. I know it was just a project to check whether it works or not. But that’s my gap.

I: You feel like we should have included-

P: Many more facilities, that When we see its efficiency we can be able to say we are not starting afresh,

I: ok

P: training people, because if you have used it in a certain number of facilities, so we have a challenge that we need to train more, distribute to every facility, we need to start re-monitoring, though it will be a process. That’s my view.

I: Ok, alright. So in terms of future advice, when we are implementing programs like this, we need to sort of –

P: Especially for programs like TB. We need to sort of take majority instead of taking a few.

I: Why do you feel like that?

P: Majority TB is a [inaudible segment]. We need to take it serious, in a sense that if we implement efficient projects, it works for the majority of people. So that we reduce huh-huh the spread, that is very important.

I: Ok, sure, thank you very much about those last important points. We are really at the end of our interview now, we are wrapping up, but before we can wrap up I'd like to hear your final remarks. Of course, touching on the program, you know, just tell me anything that you think we did not touch on that is very important for this interview. With regards to the technologies in XXX (name of the facility).

P: Huh. What I can say is that… this program for me will assist the district, if it can be equally distributed in each and every facility, it will assist us, and it will assist us to reduce the effect of TB at the level of community. The reason behind that; If TB is being reduced, people are taking their medication, they’re adhering, and the spread of infection would be minimal. We will have a TB free community, that is very important, and also in increasing our outcomes, lost to follow up, death and success rate, the box will be able to do wonders for us. We can be able to reach a national target,

I: Mmm.

P: as we have seen the improvement and impact lately, we are no more in the 70’s, we can reach 85 improvement, we can reach the 85%

I: Yea

P: With the introduction of this box in all facilities in XXX (name of the facility)

I: Mmm

I: Alright.

P: That’s my view.

I: Alright. Thank you so much for that wonderful closing remark, your information is very valuable. We really appreciate this time and information that you have given us, and thank you so much, and this is the end of the interview, and the time of the end of the interview is: 13:14

I: Thank you.

P: Thank you very much.

GLOSSARY

*Gore (That)*

*Tsa (Of)*

*Akere (Right) Sotho version*

*Ukhuti (That)*

*Ne (Right) isiZulu version*

*Ke (It’s)*

*Ya (Yes)*
