# Supplementary material for: Acceptability of Digital Adherence Technologies to support people with drug-susceptible TB in South Africa
Source: PLoS One. 2025 Sep 24;20(9):e0332103. doi: 10.1371/journal.pone.0332103 (PMC12459780; doi:10.1371/journal.pone.0332103)
Supplement: S4 File — (ZIP) [file pone.0332103.s004.zip › S4 Transcripts/HCWs and Stakeholders/IDI 33-STK.docx]

**TRANSCRIPTION NOTATIONS**

| **Label Key** | **Meaning** |
| --- | --- |
| **I** | Start of each new utterance by the Interviewer |
| **P** | Start of each new utterance by the Participant |
| **N** | Note taker |
| **{ }** | Indicates that details were changed or pseudonyms were used to anonymise data |
| **( )** | Indicates the description provided to anonymise data |
| **XXX** | Words were omitted to anonymise data |
| **-** | Breaking into a sentence by the next speaker |
| **…** | Pause or drawn out words |
| **[ ]** | Indicates noise made, e.g. [laugh], [sigh], [pause] |
| ? | Beginning of utterance by unidentified speaker or questionable text |
| **[inaudible segment]** | Unclear section of the recording |

I: Right, thank you so much for giving mhhm us a chance to talk to you today. My name is xxxx [interviewer’s name]. Do you give us a permission to continue recording? (LOUD noise)

P: Yes, I give permission to continue to record.

I: Thank you so much. uhm, Today is xxxx (interview date). Interview at the department of health in xxxx (province name). The language we are using is English. PID number: AUR-2-1-268-002 (STK 7) Time for starting is 10:54am. (…) So, maybe as we start, we would like to know briefly about your career. How long have you worked with the department and what is it that you’ve been doing mainly?

P: Ok, (LOUD noise)Thank you very much, uhm for the department of health I started in xxxx xxx. I’m responsible for the HIV/AIDS/STI and TB programs however, in the field I’ve got 20 years doing the same programs, different departments. I worked in xxxx. I worked for xxxxx (organisation name).

I: Mmm you’ve got quite a deep experience in the department. So, right now what exactly do you do in this office?

P: Ok, I’m xxxxx (position) and TB programs whereby I’m overseeing those programmes , the implementation of the programs to five districts which is xxxx, xxxx, xxxx, xxxx, and xxxx (district names)

I: Alright, so currently what tools are available in the department to support or manage TB programs?

P: Ok, TB program I can say it has been disadvantaged its not like HIV programs whereby there are lots of resources that are being implemented in the HIV program. But, when comes to TB, it’s little bit neglected, its still struggling. There are not much tools. As for now, we are having as xxx (Province name) the performance, the 90 90 90 strategy for xxxx (Province name) for the first, we are able to screen, and we are able to take sputum, we are in a process of buying the mobile Xray to go to the communities. We are having the district partners that are having x-rays, who are implementing x-rays in the communities so that we screen as much people a we can. And then, when it comes to the second 90 of initiating, we are able to initiate those who are diagnosed with TB. Aah, we are above 90%. But when it comes to keeping those clients on the treatment until they finish. That is where the problem starts. We are having the very high lost to follow up, our clients are just disappearing. Others are going back to their respective provinces. As we all know that xxxx (province name) is a highly mobile province whereby everyone, I mean most people are coming here for better job opportunities. So, those people when they go back to their countries, to their provinces, then in the system they are like nowhere to be found. They are lost to follow up. We are having a very high lost to follow up. Thirdly, we are having a very high death rate meaning we are not getting it right to make our patients to finish treatment and to trace them and to curb the mortality.

I: So, you mentioned a bit of neglect as compared to ART. What is it that you wish could be done as to not neglect TB programs?

P: Ok, Thank you. I think if we can just focus on especially on the cases that are diagnosed for TB, like the tracing part of it. Like getting the system, like I use to tell my colleagues I like the partner, because when the partner didn’t see you come in to take the treatment, they will send an SMS “we didn’t see you”. Just to focus on lost to follow up, to trace them, the resources to trace lost to follow up clients, so that they complete their treatment. I think if we can get that, we can get it right, it will also reduce our death rate and also increase our treatment success as I didn’t talk about it. We are unable to reach the 90% of the treatment success because of we are having high death rate and high lost to follow up. So, if our resources can focus mainly on those who are diagnosed, on treatment so that they are not disappearing in the air. They are being treated successfully. We reach 90% and we curb the issue of death rate, automatically it will be reduced.

I: You mentioned issues of lost to follow up, death rates and issue with TB outcomes, so currently in the country do we have a digital adherence technology or platform that assist with TB patients?

P: No, currently there is no effective digital technology in the department that is tracing the patients. What we are doing now, there was this partner that was assisting with the tracing. It was a pilot. So, it was not all the districts, it was only at xxxx (district name) like xxx (organisation’s name) was assisting with the tracing of the patients, but we saw also the improvement because at xxx (district name) performance got better. But, with the department we tried to hire nurses to trace those patients, there are also Ward Based Outreach Teams (WBOTS) but, you know a human being is a human being, Also resources like cars, if its far. cell phones and all those things and other technologies that might help us. We are still running in the same pace because there is not much that we can implement only the WBOT and those tracer nurses that we appointed to do tracing and also the pilot study which was done at xxxx (district name) for the lost to follow up and patients.

I: Taking you back to xxx (district name), and the study that was done. Uhm, can you tell me more about what is it that was advantageous or what is it that they were doing that you think was helping?

P: Ok, at xxx (district name) the partner came to introduce at the province that they are having this , it’s a small lunch box (referring to the smart pill box*)*, it’s a small container whereby they are using the system to trace those patients. So, it gave a positive yield because those patients who were traced using that device came back to care, and they finished their treatment. So, unfortunately it was a small scale but if it can be taken to a larger scale, other districts, I think that will assist because it gives the update of the patient who didn’t come to take treatment, I mean who was not taking treatment because they are assessing the device, the nurses , the coordinators are assisting to asses because they are monitoring the taking of treatment which we encourage the completion of the treatment. Then it will reduce the death rate. Hence, the treatment success will reach 90% and also most of the patients will be healed. We know that TB is curable. We don’t have to lose patients because of TB, because we have treatment, Its curable. It’s so unlike if we lose many patients because of TB.

I: So, do you know uhm, this platform used in xxx (district name), if you know if it was used for any other programs?

P: No, for now I know that it was just for TB, other programs I never heard that it was used for any other program. I just know they came to ask for permission to use for TB patients.

I: Is there any other knowledge or experience you had with this platform?

P: Not necessarily. I just know it was assisting us with lost to follow up. The device also to trace the patients and to give information to the patients which is very much important because the patients who are taking TB treatment or any other treatment that doesn’t have information, they are the one that makes aah… I mean they are the one who are defaulters and also the support system to the family and also to encourage the family members to be part of the treatment team.

I: So, you’ve been mentioning the support that comes with that technology and the ability to monitor, Is there anything that you wish that this technology can do?

P: Yahhh, on top of support and I think it was doing the things that we are expecting like support like making sure that adherence to treatment and then, patient making sure that they finish the treatment. I think it was comprehensive one, based on what it was doing. Other gaps that we identified is patients who are from families that cannot afford to buy food. We know that TB is associated with poverty, aaah, if maybe the technology can also assess the patient’s nutritional status. If they are they accessing basic food because if they don’t have food automatically they are not going to take treatment. I think if they can incorporate with the issue of nutritional status of the patient so that we know that we are giving patients a treatment and they have food. Because not having food will encourage the non-adherence to treatment. Yes, taking other factors also like your social factors, the food one I think is the basic one. Yah.

I: Alright. You also spoke earlier about the lost to follow up as the issue. Is there anything that this digital adherence technology can further do to assist in that part?

P: Yah, lost to follow up is the issue and also the death rate as I have already mentioned. The death rate is too high and some patients we know them very late that they die without being monitored. Maybe if it can trace also, I don’t know how, It can trace, It can connect to home affairs, I don’t know how it will trace. When you punch the ID of the client, It will show the client has died. Because we are having the issue of high death rate. I don’t know how. The researchers can maybe find the place where they can incorporate the death rate. But, I don’t know how for now.

I; Alright, so do you think that data that is collected in that platform could be shared or integrated with other platforms that are existing in the department?

P: Yah, I think integration is vey much important because remember if we are not integrating, we are working in.silos. I usually say we are washing one baby hundred times. They usually laugh at me in the meetings because lack of integration can make us name a patient several times but if we are integrating we will identify that this patient is the very patient that we reported. There will be no need for double dipping. So to avoid to double reporting, I think integration is the key, we don’t work in silos. We integrate with other programs to avoid the double counting.

I; Alright. So, double counting and other programs that you wish to have the DAT integrated to. Which specifically are those programs?

P: Ok, especially for those patients’ who are on Highly active antiretroviral therapy (HAART) because at the moment we don’t have a unique identifier. We are having challenges, perhaps if we can have a system like this that we can monitor our lost to follow in the HAART program. For instance, you call a patient that is lost to follow-up, when you call the patient, the patient will tell you that I’ve got treatment. Because of lack of unique identifiers and lack of good monitoring system, patients tend to take that opportunity to have treatment form several health facilities. And then, when she is collecting treatment from clinic A, she will be lost to follow up at clinic B where she went last month. So, the system can be integrated and avoid that. I used to say, its like South African Social Security Agency (SASSA ) a mother cannot go and get the grant twice. Why can’t we do the system like that? If someone got treatment, we know that you got treatment. There will be no chance to get the second.

I: So, when you talk about a unique identifier is there anything specific you have in mind that could be made as a unique identifier?

P: Yah, I think a system, digital system that will identify thumbs when you collect treatments so that it will avoid when you go to another, that will be universal at all facilities and also when I go and collect I use my thumb. I can’t use my thumb twice. If I collected at clinic A, it will show that I already collected to clinic A. I think that will assist us most because we are lacking that system and its possible to have it at SARS, SARS is having a system (cellphone vibrating), home affairs is having a system. So we are the only department without that system. That will protect, patient will only take treatment once.

I: Ok, so when it comes to digital adherence technology and the future, What do you think will be the biggest challenge for the department to implement this in a much bigger and a broader scale?

P: Ok, the biggest challenge, number one will be if it needs the network. We know that network is a problem in our country. And then the other second challenge, now loadshedding(power cuts) hheheh [(laughs)] which is currently on. And third one is will need the capacity building for the health care workers so that they are capacitated with the technology, they know about the technology because the technology could be very good but if there is no capacity building that is (mumbles) the health care workers are not workshopped, they won’t know how to use the technology. So, I think those are the main main issues that they need to be covered and put the knowledge to people who are supposed to use the technology and also the resource, like your network, your other resource that will be required are always available.(…) Yah.

I: Do you see this technology being implemented in the future in this country?

P: Yah it could be possible; hence it shows the positive outcome when doing the pilot. I think it could be possible to implement at a larger scale and it could be beneficial to the clients of xxx (province) and other places in South Africa.

I: At any point have you ever thought of creating your own platform in the department?

P: In the department we never thought of creating anything because we are relying mostly on partners that are assisting us and also the institution of higher learning like universities. They must come with technologies and assist us. Yah, we are relying muchly on them as we are working on integration and multidisciplinary.

I: Ok, So, for the future, what do you think about the budgets and the cost around the digital adherence platforms? What comes to your mind when you think about costs?

P: The cost will be, like the budget will be very high budget because the system, we talking about the system here. The system is expensive because it involves many things that’s why in the department, we are unable to do it because of the expensive system. So, Im thinking of the very high budget, I can’t estimate the amount, but it will be very high. Unless if ever they come with other methods of cutting the costs low, perhaps that can do it. I don’t know.

I: So when you say they come up with, Who are you referring to?

P: I mean the funders. Isn’t that there are funders. Those having dollars, From the American funders [laughs] If they come up with a system that is low cost we can accept that and we be appreciate that.

I: Do you think that the department will be able to fund this kind of technology [cellphone vibrating and ringing]

P: At the moment the department, aahh, that is why we are here where we are, because if it was able to fund it we were going to implement it. To see us not able to means the department is unable to fund. We are looking for the assistance from other platforms to assist us because if we were able to fund it, by now we would have funded it because this is a problem. I think it’s a priority.

I: So, if you can think generally, do you think there is a business model that could be existing in other countries like low middle income countries that you think are working well to fund the technologies?

P: Yah.. I think in other countries because they are forward but at the moment I never met with any business model that I saw, but I just think because other countries are forward. When we read from research you could hear other countries are trying other better technologies and I think there are, although I never met. I never come with another technology that is being introduced in other countries. But from my thinking, I just think there are. I Think from the research you could here, Other countries are managing well patients, maybe if we could just benchmark with the country which is doing well, I think we could get it right.

I: So, we are at the end of our discussion, before we close is there anything that you would like to say, comment or add on what we have been talking about?

P: Ok, what I can just say is thank you very much for giving me this opportunity to take part in the study. I do believe that our patients are going to benefit from the technology, especially on the gaps that I identified in our interview. If we can just manage the gaps, I think we can move forward to get 95-95-95 as we are gearing up to 95- 95-95.

I: So, uhm just one last question. in terms of covid-19, is there any link that you can make in terms of what covid presented us and the platforms? Is there anything that could, that we have learned, basically?

I: Yah, In terms of covid, I think resource, most resources they took them to covid, because remember covid you go and test, contact tracing was so easy. With covid it was aggressively responded to. So we have learned to say if we can do it with covid, why can’t we do it with other programs? Because with covid, there was, the results, starting with the testing, contact tracing and the identifying patient, controlling the disease, it was, so we did very well as a country. So, I think if we can just use those, like now with TB with the lab we are trying to copy that style of covid of like notifying the health facilities within 24 hours of the results of TB positive sputum. Tracing like where is the sputum now, lab or the results, time frame. Trying to integrate using the covid response to other programs. But, as you know government, we are slow, but we are trying to do that to see, with covid we did very well. Then why can’t we also integrate with other programs.

I: So, with covid, do you think the pandemic revealed a need for any DAT or virtual care that are not currently being used in the country?

P: Yah, yah. Definitely. I think we saw the gaps that we were having like for instance, not even focusing on the epidemic, Teams meeting we were not using it but, during covid we discovered that teams meeting, you can have your meetings using teams, using ZOOM. With covid we learned many things that we were not aware of. I think.. Yah.

I: Ok, Is there a need for a platform that can work in such a situation that was presented by the pandemic? To care for our TB patients.

P: Yah, I think if we can just sit down and come up with the ideas we can find something that can work. That is why I’m say we are trying to copy the covid responses to the TB and see how, can’t we get it right ,. because now we’re integrating covid and TB. Whatever that we do, we’re doing with covid, we’re trying to do we are saying if ever, in the hospital they will say screen for covid. When you go to the facility, or wherever, or a shop, they screen for covid. Why can’t I be screened for TB? Because screening is just to ask those questions they were asking. Did you go overseas..? Did you have symptoms..? But with TB it’s like it’s a big thing. It cannot be done but with covid, it was easily done.

I: So do you think that digital adherence platform was useful during the times of covid?

P: Yah It was very much useful because it was it was limiting clients to come to the facilities. We know that our facilities were hotspots for covid, so most of the clients were not aah feeling comfortable to come to clinics. So having the devices at home, taking treatment, monitoring its was like limiting them to come to the health facilities. So it was assisting.

I: Was it only assisting the patient in terms of not coming to the clinic or the health care workers had some benefits too?

P; No, No, No. Even the health care workers had benefits because the workload it was taken from the- it was reduced. It was not like before where they had to monitor the patient in the room. Now the patient is been monitored in remotely and then, it was , reduced to even for the health care workers. Yes.

I: Alright., Thank you so much for giving us time, we have come to the end of our interview and time now is 11:17.

P: Ok, thank you very much.
