# Supplementary material for: Acceptability of Digital Adherence Technologies to support people with drug-susceptible TB in South Africa
Source: PLoS One. 2025 Sep 24;20(9):e0332103. doi: 10.1371/journal.pone.0332103 (PMC12459780; doi:10.1371/journal.pone.0332103)
Supplement: S4 File — (ZIP) [file pone.0332103.s004.zip › S4 Transcripts/HCWs and Stakeholders/IDI 3_HCW.docx]

**TRANSCRIPTION NOTATIONS**

| **Label Key** | **Meaning** |
| --- | --- |
| **I** | Start of each new utterance by the Interviewer |
| **P** | Start of each new utterance by the Participant |
| **N** | Note taker |
| **{ }** | Indicates that details were changed or pseudonyms were used to anonymise data |
| **( )** | Indicates the description provided to anonymize data |
| **XXX** | Words were omitted to anonymise data |
| **-** | Breaking into a sentence by the next speaker |
| **…** | Pause or drawn-out words |
| **[ ]** | Indicates noise made, e.g. [laugh], [sigh], [pause] |
| ? | Beginning of utterance by unidentified speaker or questionable text |
| **[inaudible segment]** | Unclear section of the recording |

I: Sister, do you- do we have permission to record you during this interview?

P: Yes, I agree.

I: Thank you. So, date of the interview xxxx (interview date). The PID it’s xxx. The location is xxx (clinic name) . The language used for the session is English. And the time is 10:15. So, sister can you tell me what’s the tittle of your current position?

P: Okay. Me, I’m a registered nurse; I’m working in TB.

I: Okay. How long have you been in this position?

P: As Professional nurse?

I: Yes

P: It’s xxx (number of years) now, but in TB it’s xxxx (number of years) now.

I: Okay.

I: So, can you tell me your roles and responsibilities in TB care?

P: Okay where to start [laugh] okay, so we’ve got a TB identification book, *neh* [right]? Where we, we identify all the patients who we suspect that they’ve got TB, *ne* [right]? And then we do gene expert, *neh* [right]? And then we take sputum for gene expert, *neh* [right]? And then when the gene expert came back positive is where we start initiating patient on TB treatment, *neh* [right]?

I: Yes

P: And then some- we do X-rays because other don’t have a sputum, *neh* [right]? Then, Doctor will recommend X-rays, *neh* [right]? And *kobanneng* [on children] we do the Mantoux.

I: Okay

P: Yes

I: So, after testing, what do you do?

P: Okay, after?

I: Yes

P: If the patient has got TB?

I: Yes

P: Okay. Then we initiate-we start patient on, on TB treatment, *neh* [right]? And then we start TB treatment and then *ya* [yes] we put patient on TB treatment and then we start TB treatment.

I: Okay

P: Yes

I: So, can you tell me more about- you know, the initiation-

P: Ehe [yes]-

I: Process?

P: Okay

I: Yes, of the patients.

P: Oh okay, we will do a gene expert, then a gene expert will come positive, *neh* [right]? And then we start patient on TB treatment and then we collect another sputum for bacilli AFB, *neh* [right]?

I: Uh-huh.

P: To check the, the, the bacilli, *neh* [right]? Then we collect the baseline AFB and we put the patient on TB treatment.

I: Okay

P: Yes

I: Thank you. So, for- besides collecting another sputum and putting the patient on treatment, what else do you tell the patient?

P: Okay, okay, we give the patient health education and tell them also the side effects of medication, neh [right]? Because rifafour has got many side effects, neh [right]? And then we tell the patient about the side effects, and they must report the side effects immediately in the clinic.

I: Okay

P: They must come back

I: So, what are some of the side effects?

P: Uh nop [no], because are, some are minor side effects like vomiting then we give some treatment and then the symptoms disappear. Some are minor, then the major one we refer the patient to the hospital.

I: Okay

P: Yes

I: So, how are TB services delivered at your level in relation to the Digital Adherence Technology, are you at facility level or District, Provincial or National level?

P:Facility level

I: Okay. So, now I’d like to know what you know about ASCENT, so if you were to, to explain what Digital Adherence Technology is to another health care worker who knows nothing about these Digital Adherence Technologies, what would you tell them?

P: Okay Digital Adherence Technology helps a lot, neh [right]? It’s where we can see that the patient’s taking medication well because that digital will report to us that the patient is taking medication and then this programme is doing well in our facility, it’s doing well in our facility.

I: Can you tell me more about how this digital adherence works?

P: Okay uhhh [background noise]

I: So, I was saying how does this digital adherence technology work?

P: Okay. First if the patient has got TB, then we initiate the patient treatment. We register the patient to this digital adherence technology, neh [right]? And then we register the patient and then this will remind the patient to take medication because we set the alarm, neh [right]? And then we set the reminder date whereby the patient will be reminded that it is the day to go to the clinic. So it works very well in our facility.

I: Okay. So, what does it look like?

P: The box?

I: Yes

P: Okay-

I: The digital adherence technology

P: Okay, we’ve got a box, *neh* [right]? We use the box and then somewhere there, we were using the stickers, *neh* [right] but now we are using the box and the stickers, yes.

I: So, can you tell me more about the stickers first?

P: Oh, the stickers, *neh* [right]? We used stickers before, *neh* [right]? Where we register the patient and give the patient the stickers, *neh* [right]? And then on top of the stickers there is a number then we tell the patient that you must SMS the number, *neh* [right]? The patient will SMS the number. If that patient didn’t take his medication, then there will be the red flag, that the patient didn’t uhhh take her medication, *neh* [right]? Then the reminder, we will call the patient why you didn’t take your medication, *neh* [right]? *Ya* [yes] and the box one because it has alarm, it will remind the patient to take her medication, *neh* [right]? And then there is a, like the *mabone* [lights] what, there is green, and orange and red lights. And then the green light it shows that the patient uhhh is taking her medication well, the yellow light uhhh the orange light is a reminder that the patient should come back to the clinic, *neh* [right]? And then the red one is for the battery, *ya* [yes] is for battery.

I: Yes

P: Uhhh.

I: So, you mentioned that you register a patient. Where do you register this patient?

P: Oh, there is a hub, it is an App *neh* [right] I’m registering because I’m working together with [intern’s name].

I: I see.

P: There is a hub uhhh *ke* *tsena* *kamo* App [I use an App]

I: Yes.

P: Neh [right]? And then I register the patient and the battery has got numbers, neh [right]? I register the patient and how much rifafour they take and then everything of the patient, neh [right]? And then the uhhh the battery has got the numbers neh [right], we register the numbers so that the box will remind us, it will connect with us hore [that] patient is taking medication well.

I: Okay

P: Uh-huh.

I: So how does it tell you if the patient is taking well treatment or not?

P: Okay. There is a platform, *neh* [right]? *Ya* [yes] there is a platform then it will show, if the patient is taking well, it will show green, green, green but the, if the patient is not taking a treatment well it will show the red flag then we will uhhh phone the patient immediately.

I: Okay

P: Yes

I: So, you phone the patients immediately?

P: Yes, we phone the patient. Maybe patient he didn’t uhhh take his medication because of maybe he takes its at 11 o’clock, it will be red but if maybe its now eleven o’clock then we will call the patient asking why. Maybe patient will say I’m at the funeral, I’ll take my medication. Yes things like that.

I: Okay

P: But it works very well.

I: Okay that’s good to hear.

P: Mmm.

I: So, besides calling the patient, what else do you do with patients who reflects red on the platform?

P: Uhhh for how long maybe?

I: For any duration

P: Any duration, sometimes we do a home visit.

I: Okay

P: Yes, we do home visit.

I: After how long?

P: Uhhh *ya* [yes] maybe two to three days we do home visit.

I: Yes

P: Yes, we do home visit.

I: Alright

P: Mmm.

I: So, you mentioned that you, you do home visit. Can you tell me uhhh, what your responsibility is when it comes to differentiated care? Do you understand what we mean by differentiated care?

P: Uhhh Oooh okay, uhhh you can, pardon? I didn’t-

I: Differentiated care, do you understand, tell me what you know about differentiated care.

P: Oh okay, the differentiated care, *neh* [right]? uhhh, we call the patients *akere* [right]?

I: Yes

P: Then sometimes we do the home visits and then sometimes we-what can I say, there is a reminder which reminds them that they must take their treatment.

I: Where does this reminder come from?

P: Uhhh it’s us, yah [yes] it’s us, yah [yes] it’s us now because we have been how do they call it? in-service about the, the box.

I: Okay

P: We’ve been in-service about the box, it’s us, we call the patient.

I: You call the patient?

P: Yes

I: So, besides the phone call or the alarm you mentioned and the home visits, how else is the patient reminded to take medication?

P: Uhhh we’ve got ,uhhh again the green card that reminds them that they must take the, their medication.

I: Yes

P: Yes

I: Do you know anything about the SMS?

P: Yah [yes] the SMS yah [yes] the SMS yah [yes] because uhhh we were using how do they call it these stickers neh [right]? Where they will SMS , yah [yes] they will SMS and right to the platform, if they didn’t sms it will show again that no, the patient didn’t SMS , yes. I forgot it to tell.

I: And then about SMSs patients receive with they not take medication?

P: Yes it reflects.

I:Okay so can you tell me your role in differentiated care. What’s your role when it comes to the phone calls and the home visits?

P: Oh okay, my role *neh* [right]?

I: Yes

P: Okay, akere [isn’t] we will call the patient neh [right]? And then we will ask the patient to come to the clinic neh [right]? And then if they come to the clinic we then reinforce, we educate them about the box and then the importance of taking medication and then the importance of this box that it will remind them.

I: Okay, and when it comes to home visits, what’s your role?

P: We are working with the health care workers, the home based who are working outside neh [right]? We ask them, then we write down the name of the patient then they will go door to door neh [right]? And then, yah [yes] they will come with the patient.

I: Okay

P: Uhhh they will come with the patient.

I: So, when it comes to home visits, your role is to let these home-based health care workers know the name of the patient?

P: *Yah* [yes] the name of the patient because we are working together with them *neh* [right]?

I: Yes

P: Then they come with them.

I: Okay

P: Mmm.

I: They, they bring them back to the facility?

P: *Yah* [yes] they bring them back to the facility.

I: Okay

P: Mmm.

I: So, which uhhh follow up action do you do more often, is it the phone call or home visits, which one is more common?

P: Uhhh phone call because home visit is for the ones who do not come

I: Okay

P: Yah [yes] we call if the patient does not come then we do a physical tracing.

I: Okay

P: Yes

I: So, when you first heard about uhhh digital adherence technology, what were your expectations before it was implemented?

P: Uhhh what were my expectations? uhhh to have a good outcome uhh I was expecting that we must have a good outcome because it’s a reminder, it will remind a patient to take their medication.

I: Mmm

P: To have a good outcome.

I: Mmm

P: Uh-huh

I: And when it comes to using a technology itself, what did you expect?

P: Uhhh yes better results, my dear

I: Did you think it was going to be easy to use. What were your thoughts when you first heard about technology?

P: [Laugh] I thought maybe it will waste time; you know but as time went on I realised that no this is much easier.

I: Mmm

P: UhhhI thought maybe it will waste our time because we must initiate patients, after we must uhhh do the registration for the, yah [yes] I thought maybe it will waste time but as time went on I realised that no it’s much uhhh it’s faster.

I: It’s fast?

P: *Yah* [yes] it’s faster.

I: Okay

P: Mmm.

I: What makes you think it’s easier and fast?

P: Uhh because it doesn’t take long

I: Mmm

P: *Yah* [yes] it doesn’t take long, maybe 5 minutes

I: Mmm

P: Then we are finished

I: Okay

P: Mmm.

I: Good to hear. So, okay, did your opinion change after the implementation? You said you thought it was going to waste your time, is that changed?

P: Uhhh very, very, I see a change [laugh]

I: [Laugh]

P: I see a change.

I: Okay

P: *Yah* [yes] a lot.

I: How so?

P: Uhh.

I: You mentioned that you realised it’s faster-

P: *Yah* [yes] it’s faster.

I: What else changed about your perceptions-

P: Uhh.

I: About it?

P: Uhh because our patients love this box.

I: Okay

P: *Yah* [yes] when they come they say yoh this box, if the alarm rings I know that it’s time to take my medication, so it makes things mmm easier.

I: Okay

P: Mmm

I: That’s good. So, can you tell me about the training you received on digital adherence technology-

P: Mmm.

I: And also, the differentiated model of care? *Yah* [yes] how, who trained you?

P: Oh, we are working with uhh [intern’s name]

I: Okay

P: Yah [yes] [intern’s name] uhhh first day she said sister I want to show you how this uhhhdigital uhhh adherence technology works

I: Mmm.

P: And it became easier you see

I: Mmm. So, did you attend uhhh training?

P: No.

I: Formal training?

P: No, no

I: So, you were trained on site by the intern?

P: Yes

I: That’s good

P: Yes

I: So, what was your first impression when she told you about the digital adherence technology?

P: Uhhh it reduces the missed appointments and defaulters.

I: So, did you think her training was comprehensive?

P: A lot. *Yah* [yes] it was well done.

I: Okay [laugh]

P: [laugh]

I: Did you find it useful?

P: Yes, yes, I do, I do [laugh]

I: [Laugh] okay

P: Yes, I do

I: So, do you have suggestions on how the training can be improved for health care workers?

P: Uhh *yah* [yes] I think uhhh we need more training like other, other colleagues *neh* [right]?

I: Mmm.

P: *Yah* [yes] on this digital uhhh adherence technology. I think if maybe if you can come to, train other colleagues in-service

I: Okay, when you say other colleagues-

P: Mmm.

I: What positions are you talking about?

P: Uhhh professional nurses uhhh professional nurses.

I: Okay, and how long do you think the training should be?

P: How long?

I: Yes

P: Mmm only one day

I: One day?

P: *Yah* [yes] only one day

I: Okay

P: Mmm.

I: And what should they be trained on, what do you think should be the content of the training?

P: How to use this digital uhhh adherence technology.

I: Okay. What do you mean how to use it?

P: Isn’t it I’m not the only one who’s working at TB. P: *Neh* [right] We relieve each other if maybe somebody is in there, she must know how to use this, *neh* [right]?

I: Mmm.

P: Because it’s easier and faster and more.

I: Okay, so from your perspectives as a TB nurse, can you tell me the benefits of the differentiated care and the use of the digital adherence technology, what are the benefits?

P: Uhhh the benefits neh [right] this digital is more advanced you see.

I: Mmm.

P: Then calling patients, it’s more advanced.

I: Okay

P: Mmm (yes).

I: What else are the benefits?

P: Uhh-

I: You can think of a patient you’ve worked with using the box and just see and tell us-

P: Oh okay

I: What the benefits was-

P: Okay. Everyone loves the box. Every patient who was uhhh given the box, she can tell, or he can tell that you no I like this box because it reminds me every time to take my medication.

I: So, they like it because it reminds them?

P: Yah [yes] it reminds them, it’s easy because they don’t forget when to come to the clinic. you see?

I: Mmm.

P: Yes

I: Has the use of technology changed your relationship with uhhh patients as the TB nurse?

P: Not such because we are working together akere [isn’t].

I: Okay, that’s fine. So, uhhh you mentioned the benefits in relation to the patients-

P: Mmm.

I: That it reminds them, they love it-

P: Uhhh

I: So, what are the benefits uhhh of this technology from your side as, as a nurse?

P: Okay, my benefits *neh* [right]? Uhhh we can work using this digital technology and then *yah* [yes] it benefits a lot.

I: Mmm.

P: On our side we benefit a lot because it reduces the defaulter rates, you see. And then the missed appointments are very low especially in our facility.

I: Can you tell me more about the use of the platform and how it’s benefiting you?

P: Okay uhhh the use of the platform is to check if the patient is taking medication well and then there will be a calender that shows us that the patient is taking medication well right.

I: Okay. Do you know about the task list?

P: The task list?

I: Mmm (yes).

P: uhhh the task list, what do you mean my dear?

I: Okay it’s on the platform.I: Okay it’s on the platform.

P: Uhh.

I: And it shows patients who should be prioritised, patients who missed yesterday’s dose, patients who used, who missed the last two to three doses. Do you use it?

P: *Yah* [yes] we use it

I: Okay

P: Uhhh *yah* [yes] we use it.

I: Okay

P: Mmm we use it.

I: So, what do you think about that task list?

P: Uhh I don’t get the question

I: So, what your perceptions about the task list, how do you view it?

P: I think it makes uhh things easier.

I: Okay

P: Mmm.

I: And uhhh you mentioned that you were once using uhhh the labels or the stickers then you changed to the box-

P: Mmm.

I: Uhhh what was your experience with the stickers?

P: Oh, the stickers *neh* [right]?

I: Yes

P: The patients with stickers would forget to sms, others they don’t have cell phones some would say say no uhhh I lost my cell phone, you see.

I: Mmm

P: It was a challenge.

I: Besides having a cell phone or forgetting to sms, what were the other challenges with use of stickers?

P: Uhhh other challenges uhhh (……) Mmm

I: Where there network problems?

P: *Yah* [yes] the network, yes *yah* [yes] the network also, *neh* [right]?

I: Yes

P: *Yah* [yes]

I: Can you tell me more about that one?

P: Uhhh (……) uhhh on our side or on patient’s side?

I: On both sides

P: On both sides?

I: Yes

P: Because yah [yes] some would say "the network was not available, that is why I didn’t sms", yes.

I: Okay

P: *Yah* [yes]

I: Alright, and in terms of uhhh acceptability-

P: Mmm

I: Uhhh did you have patients who refused to take the digital adherence technology?

P: *Nop* [no] no

I: For both the label and the box?

P: No

I: Okay

P: No

I: Alright. And uhhh can you also tell me about the challenges with the use of the box? You reflected on the challenges with the labels. What are the challenges with the box?

P: Uhhh with the box uhh recently so *neh* [right]? It was one patient who said uhhh the box is always on red, *neh* [right]? Then we said to him that it’s a battery.

I: Mmm.

P: *Yah* [yes] it was only the one patient. I don’t know if maybe *the b*ox fell or what, but the box was red, it was only one patient that came and said no it always shows the red light.

I: Okay. So, do you have any challenges with the differentiated care? What are the challenges with the phone calls or home visits?

P: Uhhh with phone calls *akere* [isn’t] sometimes *iya ko de* [it goes to] voicemail, you can’t uhhh reach the patient. And home visit, patient uhhh relocate, *wabona* [you see]? *Yah* [yes] it was challenge.

I: So, why, what would be some of the reasons why it goes to voicemail, why the phone goes to voicemail-

P: Mmm.

P: When you try to make a phone call?

P: Others *akere* *batlobutsa* *gore* [they will tell you that] when, if maybe I’m at work, I don’t uhhh use phone, *wabona* [you see]?

I: Yes

P: *Yah* [yes] it was a challenge.

I: Okay, and then uhhh with the home visits, patients relocate?

P: *Yah* [yes] patients relocate, *wabona* [you see] patients give us wrong addresses, you know *yah* [yes] *yah* [yes]

I: So, how do you think these challenges can be resolved?

P: Uhhh it can be resolved *akere* [isn’t] if maybe the patient *bona* *kabobona* [them] they give us uhhh the correct addresses, if the patient relocate, they must come back to the clinic and say no I’m no longer staying in my area, I’m relocating, *wabona* [you see]? *Yah* [yes].

I: Do you inform them about the importance of informing you when they are moving?

P: *Yah* [yes] *yah* [yes] then, *yah* [yes] we tell them

I: And when do you tell them all that information?

P: Uhhh in the first ini- initiation, *yah* [yes] at the first initiation.

I: What exactly do you tell them, regarding?

P: Uhhh to say maybe if maybe uhhh they relocate, they must come back to the clinic and the, we will give them the transfer letters, but *renna* [us] in our facility, we don’t have uhhh problems with the patients, no.

I: Okay, alright. So, do you always have staff available to do home visits after the two to three days you mentioned?

P: Yes, yes, we do, we do.

I: So, they always go after two to three days?

P: They always go because every morning they take uhhh list of patients.

I: Okay

P: Mmm.

I: Can you comment on staff rotation like the rotation of TB nurses in the TB room?

P: Oh

I: In relation to the digital adherence technology-

P: Oh

I: And how it can be a challenge?

P: Oh, okay no we don’t rotate in TB, we don’t rotate. Maybe when I’m off or I’m sick, there will be a reliever, but we don’t I’m, I’m staying in, in TB.

I: Okay

P: I’m working in TB.

I: Okay

P: Yes

I: That’s interesting

P: Yes

I: So, from your perspective as a nurse uhhh do you think TB treatment can be improved using the digital adherence technology?

P: Uhhh exactly, exactly.

I: How so?

P: Uhhh patients uhhh *barata* [they like] this, they like, they love this

I: Mmm.

P: Mmm programme, they like this, they like this problem- uhhh programme

I: Okay

P: And then they used to it because uhhh Yoh no, *yah* [yes] *bayayithanda* [they like it].

I: What exactly did they say they like about it?

P: They say, maybe uhhh, can I speak my language? [laugh]

I: Yes

P: [Laugh] no sister *waisti* *bengkele* [I was] in the kitchen, *neh* [right]? So, *ngwana* *uthlwile* *de* box [the baby heard the box] uhhh alarm then he came *Koko* *Koko* [knock] it’s your time to take medication, *wabona* [you see]? *Yah* [yes] *yah* [yes] so they can’t miss their medication, unless maybe I don’t know but, no it works.

I: Okay, so there’s also family support?

P: Yes, family support *koko,* *koko* *ashi* box *yahao* [knock, knock here is your box] it’s how they told us, hey box *iyasebetsa* [box is working] [laugh]

I: [Laugh] that’s good to hear.

P: [Laugh]

I: So, is there a difference in terms of TB treatment uhhh before we, you started using the box?

P: Mmm.

I: And afterwards

P: Mmm.

I: In terms of how you monitor the patients and uhhh your relationship with the patients?

P: Mmm there is a much improvement

I: Uhhh can you tell me more?

P: There is a much improvement mam *neh* [right]? Because now uhhh as I, I, I mentioned that the, the, the, the, the defaulter rate has gone down, down, down, down because uhhh patients they comply now.

I: Okay

P: They comply

I: That’s interesting

P: Mmm they comply.

I: And in terms of your, your workload as well, can you comment on that?

P: uhhh the workload, like? I don’t get it.

I: Like the amount of work you, you have following up with the patients, with the-

P: Yes

I: Box, before the box?

P: *Yah* [yes] before the box because now the box reminds them to take medication and then, *yah* [yes] *yah* [yes]

I: Okay

P: Mmm.

I: So, do you think it, the workload has improved, or it has worsened?

P: No, it has improved a lot

I: Okay

P: Mmm (yes).

I: So, can you tell me more about the positive changes of the differentiated care, these follow ups we make, you make and the use of uhhh digital adherence technology and *yah* [yes] what are the positive changes?

P: Mmm.

I: Which has brought.

P: (……) uhhh, Yoh uhhh there is a lot of change, there is a lot of change especially uhhh since we start on this box, the App uhhh thing digital adherence technology, there is a lot of improvement and then it uhhh *bare* *keing* [how do they call it] e *fekuditse* *le* *nako* *ela* *enchi* *Wabona* *ya* *gore* we must call the patient and we must do physical tracing e *fokoditse* [it reduced a lot of time, you see that we must call the patient and we must do physical tracing].

I: Okay. So, were you doing the D- DOT before?

P: Uhhh like uhhh tracing the patients?

I: The D- the Direct Observed Therapy.

P: Uhhh no *akere* [isn’t] patients they, they, they take medication to our facility, no.

I: Okay. So, with the positive changes you mentioned-

P: Mmm.

I: That has been brought by the, the, the technology-

P: Uhhh.

I: Or the programme, what do you think can be improved at the facility level-

P: Mmm.

I: For us to, to sustain-

P: Okay

I: These changes?

P: Okay uhhh *tswantswe* *lekesoe* [we should go to] training *neh* [right]? I think all registered nurses should be trained on this, *neh* [right]? And then we need more boxes, *neh* [right]?

I: Okay

P: So that *akere* [isn’t it] uhhh TB is there and then *reokopa* [we will need] more boxes so that *le* *renna* [even us] we can uhhh register patient because-

I: Okay

P: *Yah* [yes] it helps a lot

I: Alright

P: And the training, I think maybe you can come and train our staff *neh* [right] about this *neh* [right]?

I: Alright

P: *Yah* [yes] it works

I: Can you think of the negative changes that have been brought by the box or the differentiated care?

P: Negative? No [laugh]

I: Have uhhh any patient reported stigma or are there any, any issues around stigma?

P: No, because *akere* [isn’t] we approach them very well, we approaching, no we don’t experience that.

I: Okay

P: No

I: And uhhh the issue of patients not answering their phone calls, do you have challenges?

P: *Yah* [yes] we’ve got challenges because some they don’t answer their phone calls, that is why we do tracing, physical tracing, *neh* [right]? *Yah* [yes]

I: Okay. So, have you had any concern about the box representing patients haven’t taken the medication, did you have any concern, concern about that? That if you see green-

P: Mmm.

I: It means the patient has taken the medication.

P: Mmm.

I: Have you ever been worried that it might not be the case?

P: Uhhh no, because *akere* [isn’t] we’ve got an outcome, we’ve got an outcome of uhhh cured, TB completed, because the, the AFB sputum will tell if the patient is, is, is not taking her medication, *neh* [right]?

I: Okay, so you are not worried about-

P: *Yah* [yes] we are not worried because the outcome it take, *yah* [yes] we’ve got uhhh the outcomes *tsedi* smart [the smart outcomes] mmm.

I: Okay, so can you tell me about system level structures, structures that needs to be improved in order to integrate this programme of digital adherence technology and differentiated model of care into existing TB programme formally-

P: Mmm.

I: That it’s brought out everyone is using it? What needs to be in place? Can you think about it, let’s say the absence of the xxxx (organisation name), in the absence of (xxxxx)?

P: (…) uhhh (…) that is why *kere* [I say] uhhh *hakakeriya* [if we can get] in-service training, *wabona* [you see]?

I: Yes

P: *Yah* [yes] that is why *kere* [I say] *hakakeriya* the in-service training

I: Mmm.

P: And then we service on this so that we can continue with this.

I: Okay

P: *Yah* [yes] programme.

I: What resources would be required for you to continue as Department of Health from your own?

P: Uhhh *kete* boxes *akere* [it’s the boxes, right?]

I: Mmm.

P: *Yah* [yes] we will need boxes and the (…)

I: Okay

P: And the, the, the concern forms

I: Okay

P: Mmm.

I: And uhhh who do you think should be preparing the boxes?

P: Uh-huh.

I: Like charging before it’s issued to the patients.

P: It’s us professional nurses.

I: Professional nurses?

P: Yes

I: Okay

P: Mmm.

I: Do you think someone should be assisting with that?

P: Yes, yes

I: Who exactly?

P: Uhh *akere* [right] [***] were assisting before?

I: Yes

P: Yes, I think maybe if maybe *hokaba* *le* someone [if there can be someone] to assist, *yah* [yes]

I: So, what would be the title of that *patie*- of that person assisting?

P: Mmm anyone [laugh]

I: Okay

P: [laugh]

I: Alright, and then let’s say you have technical issues with the technology, how, how do you think those should be handled?

P: Mmm.

I: When you are working alone as the Department of Health

P: Oh, alone, as the Department of Health, *neh* [right]? And then if maybe *loraba* *ledi* challenges [we get challenges] like maybe battery and everything uh *yah* [yes] it will be a problem [laugh]

I: [Laugh] who do you think should be assisting you with those issues? Let’s say there’s a problem with the adherence platform or the box, how can that be handled by the Department of Health?

P: Uhhh I cannot answer that [laugh].

I: Okay.

P: [Laugh].

I: [Laugh] okay. So, what are the systems in place that could moni- that could monitor differentiated model of care and uhhh the medication device technology? So, what do you use to monitor these home visits uhhh do you record the phone calls, feedback from the home visits-

P: Oh, feedback from, okay

I: Or if you have challenges with the technology?

P: Uhhh I, pardon, I, I didn’t get the answer- the, the question

I: So, do you monitor, or do you capture challenges or successes or pro-, or problems using-

P: Oh

I: The technology?

P: The technology?

I: Yes

P: Oh, as I said *hore* [that] it was only one patient who reported the battery, only one, *wabona* [you see]

I: Okay

P: Out of all patients, it was only one who reported that the battery is ever red, *neh* [right]?

I: Mmm.

P: And then uhhh when coming to tracing, *neh* [right]? *Rennale* [we have] the tracing uhhh tool where we trace our patients and we, we, we file them, we file the, the, the, the, the, I don’t know how to, to put it [laugh]

I: Okay. So, with this one patient who had an issue with the battery, did you document it?

P: Oh, *yah* [yes] *yah* [yes] *yah* [yes]

I: Where did you document it?

P: Uhhh I phone, I think I phone [***] yes, I phone [***] that the patient is having a problem *mara* [but] [***] will, will see if the patient is having the problem because the patient will not uhhh the platform *akere* [isn’t] Will, will reflects red.

I: Mmm.

P: Maybe he will call the patient, it was last week Thursday.

I: So, did you write it somewhere that the patient and the problem and this is what I-

P: Yes

I: Where did you write?

P: Uhhh we’ve got a file where we put everything in the file.

I: Okay

P: *Yah* [yes].

I: Alright, so you did it yourself?

P: Yes, I did it myself.

I: Okay.

P: Yes

I: So, you write it manually on the file, do you use any other system to capture it electronically?

P: No, no, no

I: Okay, so can you tell me any gaps which exist in the way the intervention has been delivered, so this whole programme of the, the box and everything at differentiated care, what are the gaps do you think they are there?

P: Mmm.

I: And how they can be improved?

P: *Yah* [yes] (……)

I: Okay, can you think of you know, the way the box looks, right, do you have any suggestions on how that can be changed?

P: Uh-mmm no.

I: No?

P: No

I: Okay, so do you have any patients who take other treatment other than TB treatment?

P: Yes

I: And are, are they using the box?

P: No

I: So, currently they are only using it for TB treatment?

P: *Yah* [yes] they are using it for TB treatment.

I: Okay

P: *Yah* [yes] for TB treatment but *yah* [yes] we’ve got other patients who are taking other treatment.

I: Mmm.

P: But those with uhhh because every time they come with their treatment, *neh* [right] but the, the, the TB treatment is there in the box.

I: Mmm.

P: Yes

I: Uhhh okay

P: Because *akere* [isn’t it] we open once

I: Yes

P: Yes, and sometimes they take uhhh medication in the morning and afternoon, *yabona* [you see] it will be a challenge.

I: A challenge?

P: Yes

I: Okay. So, do you think the box, or the digital adherence technology can also be useful for other diseases?

P: I think so, *yah* [yes] I think so, *yah* [yes] I think so

I: Okay, how so? Can you tell me more about that?

P: Because it uhhh easier to use *akere* [isn’t]?

I: Mmm.

P: It’s easy to use and then it, it reminds patie- uhhh people uhhh patients to take their medication *akere* [isn’t]? And it’s also going to help, *akere* [isn’t]? Because *iya* alarm *akere* [it alarms, right?] *tititititi* [beeping]

I: Mmm.

P: *Yah* [yes] *yah* [yes]

I: So uhhh I just wanted to find out, do you have a group of people you think uhhh are bit of a challenge, you know when it comes to the use of digital adherence technology who finds it difficult to use or you find it difficult to support using the digital adherence technology?

P: Uhhh with stickers before, yes.

I: Mmm.

P: But now with box, no.

I: So, with, with stickers who, which group was difficult to support with the stickers?

P: Uhhh *yah* [yes] old age like, *yah* [yes] old age people because it was difficult for them to, to, to, to sms and sometimes they forget, *wabona* [you see]? But with box it o-, they open once *akere* [isn’t]? The, the, they take their medication and close, no, box is.

I: Okay.

P: *Yah* [yes]

I: Alright

P: Uhhh.

I: Thank you very much uhhh we’ve reached the end of our interview. Thanks for your information sister.
