# Supplementary material for: Acceptability of Digital Adherence Technologies to support people with drug-susceptible TB in South Africa
Source: PLoS One. 2025 Sep 24;20(9):e0332103. doi: 10.1371/journal.pone.0332103 (PMC12459780; doi:10.1371/journal.pone.0332103)
Supplement: S4 File — (ZIP) [file pone.0332103.s004.zip › S4 Transcripts/HCWs and Stakeholders/IDI 4-HCW.docx]

Transcription English

| **Label Key** | **Meaning** |
| --- | --- |
| **I** | Start of each new utterance by the Interviewer |
| **P** | Start of each new utterance by the Participant |
| **N** | Note taker |
| **{ }** | Indicates that details were changed or pseudonyms were used to anonymise data |
| **( )** | Indicates the description provided to anonymise data |
| **XXX** | Words were omitted to anonymise data |
| **-** | Breaking into a sentence by the next speaker |
| **…** | Pause or drawn out words |
| **[ ]** | Indicates noise made, e.g. [laugh], [sigh], [pause] |
| ? | Beginning of utterance by unidentified speaker or questionable text |
| **[inaudible segment]** | Unclear section of the recording |

I: Date of IDI xxxx (interview date), location xxx [facility name], language English, PID xxx, interviewer: xxxx (interviewer’s name), time 12:41PM. Uhm…thank you so much for allowing us to interview you. So, now we are about to start. Uhm…do you agree for us to record this interview?

P: Yes, I agree

I: Okay, thank you so much. Uhm…so, tell me what was the title of your position where you were working?

P: I was working as a research assistant at xxxx (clinic name)

I: And how long did you work as a research assistant in xxxx (clinic name)?

P: I started in xxxx (date) , xxx (date) until xxxx (date).

I: And when it comes to patients’ care and counselling, what were your roles and responsibilities?

P: Eh…I was dealing with adherence. We were helping participants or patients to take their medication each and every day. So, we were giving our patients- TB patients, a smart pill box whereby this smart pill box was assisting them to take their medication. Eh…this box reminds them to take medication each and every day. If a patient didn’t take a medication it appears on the system. So, maybe after two days, if the system tells me that no, this Mr So and So, didn’t take the treatment, I call the patient. But maybe if he doesn’t take it, like for four days, I refer him to Ward-based primary health care outreach teams (WBOT) team. So, WBOT team is the team that do a home visit to our patients.

I: Okay. And how were TB services delivered at your level with regards to the DAT intervention?

P: Eh…district level.

I: Oh, district level.

P: Yes

I: Okay, thank you. Uhm…I would like to know what do you know about ASCENT? If you were to explain to another person or another health care worker about ASCENT, what would you tell this person about ASCENT?

P: ASCENT is dealing with the research specialising with the TB, and they are dealing with DAT which is a Digital Adherence Technology whereby there is the smart pill box, that I was talking about, the labels, the stickers. On the stickers the patients were supposed to send a code. Each and every day after taking a medication, a patient sends a code. It’s a proof that he /she took a medication.

I: Okay, thank you. Uhm…you said you were using a box, smart pill box to monitor patients’ adherence, so, I would like to know if you can explain to me how exactly were you monitoring this patient, how exactly was the process being done?

P: Okay, I was giving TB patients the smart pill box. I was recruiting patient to participate in the study and then we sign a consent form, after signing a consent form, I register a patient on a platform on our tablet or on our device, so that I can see if a patient did or didn’t take a medication.

I: Okay, I hear you talking about the platform, what is the name of this platform that you were using, do you still remember?

P: Yes,xxxx (adherence platform name)

I: Okay. Uhm…and you say you use xxx (adherence platform name) App to monitor patient who are using the box. So, earlier on you mentioned that you check if patient took their medication, if they missed for several days, then you could refer them to the WBOT. So, what I would like to understand is, how exactly on this xxxx (adherence platform name) platform that you were talking about, where you’re seeing that so and so patient has missed a dose, how exactly? When you log on – I believe that you log on into this App and then from there, how do you see that this patient did not take her medication?

P: I was checking on task list. *Yeeah* …like if a patient didn’t take for two days, it tells me that this patient didn’t take a medication for two days. For four days, there is a calendar on a task list. If a patient didn’t take medication it appears on red, then if he did take a medication it shows with green bar.

I: Okay. Uhm…this box *neh*(Right*)*?

P: Mm*.*

I: How exactly does it help patients or how does it work for patients that you are monitoring as you said you are monitoring their adherence? How this box – how does this it benefits patients?

P: It was reminding them to take their medication because some of our patients, doesn’t have cell phones. So, even if you have a cell phone, maybe if you forgot to set an alarm today, maybe to remind you to take a medication, this box reminds them every day. We set alarm on a device, then it reminds a patient each and every day to take a medication.

I: Okay [inaudible segment]

P: Oh! And if a patient didn’t take a medication, as I said, it appears on the system. So, I’m also assisting them, like to remind them or to ask what happened if I could see that no, this patient didn’t take a medication, I do a phone call and ask what happened.

I: Okay, thank you. And how else were these patients reminded? You mentioned the alarm, you mentioned the phone call where you make a phone call. Uhm…is there another way that they are being reminded by this smart pill box or the platform rather, just to say, they have to take their medication? How else were they also reminded besides the alarms and you, reminding them, was there another way where they have been reminded [inaudible segment] box, the alarm, the platform?

P: Eh…they were also getting messages to remind them, “Please don’t forget to take your medication.” *Yah(*yes*)*, they were also getting messages on their cell phones.

I: Okay. And you talked about home visits earlier on, that you refer patients who missed doses, you refer them to WBOT teams to go and look for these patients, right? So, what I would like to know is what was the success of those home visits? Were they successful or were there any challenges?

P: *Yeah* (Yes), it was successful and there was also a challenge. They were also experiencing some challenges. *Yeah* (Yes), but some home visits became successful because they were also coming with feedback to us, and they were also coming with the patients to us. But sometimes maybe you will find that a patient is no longer staying at home, maybe a patient is using drugs, he’s addicted. So, sometimes there were those challenges.

I: Okay. Uhm…if I hear you correctly, you say that sometimes they’ll go and have a challenge of not finding a patient where they said they reside. So, that means there was the problem with the addresses that had been provided by the patients.

P: No, maybe if they do home visits and then they never get that patient. Maybe they find a sister, patient’s sister, she will tell the WBOT that no, this person is no longer staying with us, she’s staying on the streets, *yeah*(yes)

I: Okay [inaudible segment] –

P: But they were trying by all means to get those patients even if they’re on the streets using drugs, they were trying by all means to get them, *yeah (*yes*)*.

I: Okay, you mentioned that drug users or patients who were using. How many patients in your facility were you aware of, who were using the substance, the drugs?

P: Maybe fifteen percent.

I: Fifteen percent?

P: *Yeah (*yes*).*

I: Okay. And what were some of the challenges you faced when it comes to these patients who were using – what was some of the challenges compared to others who were not using?

P: Most of them [drug users] don’t have cell phones. So, even if you can see on the system that this patient doesn’t take medication, there is no way to get a patient because even if you refer him to the WBOT, the WBOT team will do a home visit only to find out that the patient is no longer staying at home but is now living on the streets taking drugs. *Yeah (*yes*)*, those were the challenges that we were experiencing. Okay, maybe if you find a patient or you get a patient over the phone, the patient will tell you that I lost the box, the box is broken. *Yeah (*yes*)*, those were the challenges.

I: And how often had it happened where you get a patient who report that they lost the box?

P: Maybe twice.

I: Okay, were they- those ones that you said they were staying on the street, they’re using the drugs, or it also include just other patients that were not homeless?

P: No, it’s only those drug users.

I: And what were some of their reasons for them to lose the box?

P: No, those are only reason.

I: I mean, what would they normally say happened?

P: If they didn’t take medication?

I: No, I mean you said they would come and report that they lost the box, so, I’m asking what are some of their reason could be that they lost the box, what happened, who took the box, were they robbed, what happened when they loss the box?

P: Maybe the patient would tell you that maybe he has been robbed, they took the box. *Yeah*, those were the reasons.

I: And how was their adherence, those patients who were using the drugs? How was their adherence compared to the other patients? Were they taking treatment or?

P: *Yeah (*yes*)*, most of them were taking treatment. Most of them were taking treatment. *Yeah (*yes*)*, maybe only six percent of the drug users –

I: Who were not?

P: Who were not taking.

I: Uhm…You mentioned the issue of patients not having cell phones. I just want to understand that, during those times where you had to call those patients to find out why they missed their dose – how were you able to reach them, those who did not have cell phones?

P: Uhm…most of them were giving me cell phone of their family members. So, I was calling their family members to ask what happened to the patient, *yah (*yes*)*.

I: Okay, are there any other challenges with home visit that you know, besides the one that you have already mentioned? The issue of addresses – were there any other challenges that you can think of, when it comes to home visit?

P: Maybe, they will find the door closed, maybe they don’t find anyone. *Yeah (*yes*)*, those were the reasons that they were experiencing.

I: Okay. And was the staff always available, the WBOT who were tracing these patients? Were they always available to do home visits?

P: Yes, they were always available because each and every day in the morning, they were coming to TB room to get the list of those people who need to be traced.

I: Okay. And were you also involved in counselling of the patients? You mentioned patients who were struggling, drug users, who were not taking treatment correctly. So, I assume that after they were being traced and found, maybe one of the things that would be normally done is to do another counselling process. So, I just want to understand if you had played a role of counselling some of these patients.

P: Yes, I was also involved, but in most of the time in TB room, we have a social worker from xxx (organisation name), so, in most of the time she was the one who was doing counselling. If maybe patient is not adhering, she was the one.

I: Okay. Uhm…please describe your role within differentiated model of care intervention. What was your role when it comes to differentiated model of care. Already you mentioned that you were calling patients sometimes when they didn’t take their medication. Also, you mentioned that you were not involved when it comes to home visits, but you would refer to the WBOT team to trace those patients. So, right now what I want to understand from you is uhm…you mentioned that you were not involved on a home visit but the WBOT team were doing the home visits, but you were calling patients and you were also involved in counselling which falls under the differentiated mode of care. So, now I want to understand which one you were doing the most between the two? Which one were you doing frequently between phoning patients and counselling them? Which one did you do more often?

P: Calling patients because each and every day when coming to the clinic, I check on the system, on the task list, how many patients had missed their doses. So, I was calling them, like every day.

I: And how successful were those phone calls? Uhm…were you reaching patients every time when you call them?

P: In most of the time I was reaching them.. And then after that – after doing the phone call or even after getting feedback from our WBOT team, I was also recording the feedback about phone call, about home visit on the system on xxxx (adherence platform name), on the system and also on our logs. *Mm* (yes)

I: Okay. How were the responsibilities and duties being shared between you and other health care workers in the TB room?

P: Eh…

I: What did you do and what did the other health care workers who were working in a TB room, the sisters – what did they do and what did you and how were your duties shared amongst you?

P: Oh! I was the one who was like following up patients, doing phone calls and if I don’t get a patient maybe or if a patient doesn’t take treatment for like four days, that’s when I refer him/her to our WBOT team. And also, TB nurse was working with us, *yeah* (yes).

I: Okay. Were there any duties that you were performing in the TB room that were not relating to the DAT?

P: Yes.

I: And what were those duties that you performed?

P: I was also collecting sputum, checking the results, writing the results for TB, I was checking BP for patients, I was doing a vital sign yah.

I: Okay when you first heard about the DAT intervention, right? What were your expectations before it was implemented – before you were involved in implementing?

P: [Breath in and out] hey –

I: What did you think about it? What did you think.

P: I was concerned if maybe this technology will help our patients. But I was like more concerned about stickers because they have to send the messages each and every day after taking the medication, but I was concerned more about adults, adult people because maybe most of them don’t have cell phones. Maybe even if they have cell phones, when you call, he / she will tell you that I forgot to take a medication but *i-box* (the box) like, *yeah*. I was concerned about stickers, *yah*. It helps them a lot – it helped them because they were adhering, they were taking their medication very well even those who were using stickers. In terms of i-box, *yeah*, the patients were taking the medication very well because it was reminding them every day to take medication. The only thing that I was concerned about was the labels.

I: And what was the concern if you may remind me about the labels?

P: I was like asking myself if it will help them or what, the labels, because they had to send a message after taking the medication, but it helped them, because they were sending messages like *yeah (*yes*)*. They were doing very well even those who were using the labels.

I: Were there no challenges or patients who were struggling who were using the labels?

P: Some of them would tell you that I lost my phone, my phone has a problem, like. *Yea(*yes*)*, those were the only challenges that maybe the patient tells you that I lost my phone, my phone has a problem, maybe the screen is not working, I can’t send a message. But I was also recording on the system.

I: Okay. You talk about the box and the labels. So, I just want to understand which one – were you implementing both in your facility or one of them?

P: Yes, I was implementing both. I started by the stickers and then maybe after a month, we were using a box. So, I was implementing both, labels, and box.

I: Okay, that’s interesting. And how was the difference in terms of the adherence between those who were using the box and those who were using the labels? Was there anything different in terms of the adherence?

P: *Yeah (*yes*)*, there was a different because those who were using *i-box* (the box)*,* the box was reminding them to take medication, so they were not sending any message. If the patient didn’t take the medication, it appears on the system. But those who were using the labels, they had to send message every time after taking a medication. So, *yeah*, there was a little bit of difference.

I: Okay. If I hear correctly, are you saying that there was a challenge in sending the SMS after taking the medication? –

P: *Mm* –

I: Or what?

P: *Yeah*, sometimes in sending – after taking a medication. Sometimes they will tell you that *iyooh*, I forget but I took my medication. I forgot to send an SMS, but I did take my medication.

I: Okay. So, you will find out when you call them that they took their medication, but they forgot to send?

P: Yes

I: Okay. And what were some of the challenges with the stickers that you can think of, besides the issue of forgetting to send an SMS – what was some of the issues that had been reported by the patients who were using stickers?

P: Patient will tell you, like, I forget to take the medication, my phone has a problem, I lost my phone. It was cell phones most of the time.

I: Okay, you mentioned elders who were on stickers, right? That there were big challenges with using the cell phone. So, now I want to understand that did you continue to give elderly people stickers or what was the solution because I believe that they were also interested in the DAT, and you made the comparison between the box and the stickers that it's better with the box because you don’t have to send a message, like the box reminds you and with the stickers you have to send the message and now you said older people were challenged when it comes to sending an SMS. So, now I want to understand that where there any old people within your population or in your facilities, whom you offered stickers or who were on stickers, and how were they doing --were they able to send or were there any challenges with older people who were using stickers?

P: *Yeah* (yes), some of them were able to send messages. Like some of them were doing very good but if I see that this patient is struggling, I was changing them from labels to a box. *Mm*, if I see that a patient is struggling.

I: Okay, and after you switch them to the smart pill box, right, was there an improvement? –

P: Yes

I: In terms of adherence?

P: Yes, there was an improvement.

I: Okay, you said that you were bit concerned about the stickers before you started implementing, then you were concerned if they are going to help patients or not. So, I just want to understand that after you started implementing or giving patients stickers, did then your opinion change afterwards, where you are now? Are you sure whether is working or not?

P: Yes, it changed because, like, most of them were really enjoying this thing of sending messages. *Yeah* (yes), they were adhering. *Yeah* (yes), it’s changed a lot.

I: Uhm…you mentioned that they were excited about these stickers, can you think of any patient whom you know who was really excited with the labels, who never wanted maybe even when you introduced a box, because you said you start with the label first, then introduce the box later on. So, now even when you introduced a box, but that patient said no, I don’t want a box, I’m still fine with the stickers – is there any patient like that?

P: Yes

I: *Mm*

P: Yes

I: Tell me more about that patient.

P: Okay, there was this patient, who was using the labels and then he saw another patient who was using a box, and he asked me, “what is this box dealing with, what is this box about?” and I told him that no, this box is working the same with the labels but the difference between these two is *i*-(the) box is reminding them to take *i*(the)-medication but *i*(the*)*-stickers, you are the one who send us the message. And then I asked him that are you willing to use *i*-box, then he said no, I’m fine with the stickers. And most of them – some of them were even reminding me, maybe if I’m not at TB room, maybe I’m busy with another patient, maybe I’m doing vital signs , they were even reminding *u*(the*)*-sister, maybe sister, a TB nurse forgot to put the labels on their treatment, they were even reminding [laughing] *u*-sister *ukuthi* (that) sister, no, you didn’t even put the stickers on my treatment.

I: Oh! That’s interesting. Uhm…now you said those patients who were using the box were also getting an SMS reminder. So, now I want to understand if patients who were using stickers were also getting an SMS, some sort of an SMS or only those who were using the box were reminded with an SMS.

P: Even those who were using the stickers were also getting an SMS beause even if a patient did send an SMS, they were getting the SMS, *ukuthi* (that) please don’t forget to take your medication. So, they were worried about that because I took my medication, but I still get a reminder, SMS, what’s going on? But I explained to them u*kuthi* (that) no, this SMS, like it’s automatically, then they understood *ukuthi* (that) okay, I understand. And then I told them *ukuthi* (that) no, I can see even if you get an SMS, but I can see if you didn’t take your medication. So, if I don’t call you, it means you did send an SMS.

I: Oh! Okay, so, at first, they were bit concerned about this SMS reminder.

P: Yes

I: Because they doubt maybe they were getting it because they were not taking their medication.

P: Yes

I: Oh, and then you explained to them that it’s an automated SMS that just reminding them.

P: Yes

I: Whether they send SMS or not, but they will get this message, is that what you are saying?

P: Yes

I: Okay. And with those patients who were using stickers, right, uhm…including those older people who were using stickers, when you were introducing the box now – were there any patients who were interested in the box, who wanted to switch from the stickers to the box?

P: Yes, especially those elderly people, maybe if a patient is struggling with a cell phone but if a patient is fine with a cell phone, *hai* (no). *Yeah*.

I: Okay, I see. You’re emphasising that it was older people, so I want to understand that was it only the older population or also the young ones who were also interested in the box, or it was only old people who wanted to switch.

P: *Yeah*, even the young ones. *Yeah* (yes).

I: They were interested in the box.

P: They were interested in box.

I: Okay. Were you trained – did you receive training before you started implementing?

P: Yes, I did.

I: Okay. Uhm…do you still remember what you have been trained on, the activities that were done during the training, what exactly were you guys being trained on?

P: I was trained about GCP, which is good clinical practice. We were trained based on DAT, about a box, smart pill box, labels, even this one, a video, video one, *yeah*. But the training was very good. Eh…even counselling, we were trained about counselling on how to do *i*-counselling, *yeah* (yes).

I: Who trained you?

P: It was xxx [ASCENT staff member], xxx [ASCENT staff member] xxx [ASCENT staff members], xxx [ASCENT staff member]–

I: And where were you trained?

P: And xxx [ASCENT staff member].

I: Okay, so, when you first started with the training, right? What was your first impression, what did you think of the training, how did you find its overall?

P: The training was very good. It’s really help us, or it’s help me a lot. Especially like *i*-GCP. *Yah*, it’s really helps me a lot, like they are making the examples, like *i*-GCP. You can’t conduct research like in a clinic or in a facility, like if you approach a person or if you approach a patient, *i*-GCP, *be isiza ukuthi* (it was helping), okay, you approach a person in a respect way. Like, how to approach a person, if you recruit a person. *Yah*, we have to be careful about that, on how to approach a person, like in a respected way.

I: Okay, do you have any suggestions on how to improve the training?

P: No, the training was good, I don’t have any suggestion.

I: Okay. And who do you think should conduct those training?

P: The xxxx team (organisation name), ASCENT team.

I: Okay, and how long should it last, this training?

P: Even a week, one week.

I: Okay, and who do you think should attend these trainings, since you have now been involved in implementing the DAT and now you know people who are involved in implementing the intervention. So, who do you think should attend training?

P: I think everyone who is dealing with patients especially in TB room, because they are dealing with TB patients. The TB nurse, the interns, health care workers, WBOT team, *yeah*.

I: Thank you, okay? And from your perspective *neh*(right*)*?

P: *Mm*

I: Can you describe the benefits now of the differentiated model of care? What are some of the benefits, your phone call, home visits, the SMS? What do you think are the benefits of these supporting action?

P: It improve adherence, as I was saying like *i*-box reminds them to take a medication, if a patient didn’t take a medication, we do phone calls, home visits. So, it really improves *i*-adherence.

I: Okay, and then the box now. What are some of the benefits of the box, from the patient’s side – how do you think the box benefits patients?

P: It reminds them to take a medication, *yeah* (yes*)*.

I: Okay. So, does that also apply to the stickers?

P: *Yeah* (yes)

I: Because you were implementing both the box. and the stickers.

P: *Yeah (*yes*).*

I: Okay, and any other benefits of the labels or the stickers to patients?

P: Uhm…it was reminding them to take the medication. *Yah*, those were the benefits.

I: Okay, and then now, to you as the staff, the health care workers, everyone else – let me say the TB staff who were involved in implementing the DAT, so, how do you think this box, the smart pill box or the labels were benefiting the staff or the health care workers?

P: Eh…*i*-level of adherence, like it’s increased a lot. There’s a difference before the box was implemented and now. So, it’s like the difference is increased level of adherence.

I: So, are you saying that before there was box, there was low adherence and after the box was implemented, it improved?

P: Yeah (yes).

I: Okay, you mentioned that you were monitoring patients, using the xxxx (adherence platform name), right? So, I want to understand that how did this platform made it easy for you to monitor patients compare to before it was implemented. Was there a difference between before it was being implemented and after it was implemented? Was there a difference –did it make it easy for you to monitor patients?

P: Eh…*i*-device, *i*-platform, xxx (adherence platform name)?

I: Yes

P: Yea, it makes easier for me because I can see on the platform, if a patient didn’t take a medication.

I: And before the xxxxx (adherence platform name) platform and the smart pill box and the labels, how were patients’ adherence monitored before? How was it being monitored, do you know?

P: They were using green card. *Yea*, they were giving patients the green card. The sister told me that before this technology was implemented, they were using the green card. Every time, after taking a medication, a patient will tick on the green card. It’s a sign that I did take a medication but if a patient didn’t take a medication, he/ she will leave it blank. So that was the way.

I: Okay, how did they also see that a patient was not taking medication, as you say that with the platform you can easily see that okay, yesterday the patient didn’t take medication, and with the card I assume that the nurses would see the card maybe after a month when they are coming for a review or something that they ticked all the days of that month, do you understand? So that to say they were taking the treatment. And now when they come to the clinic, how did they confirm if patients were taking medication or not beside the clinic card, how else did they confirm that patients were really adhering?
P: If *u*(a) sister *a bona kuthi* (see that*)* no, there is like, *a kuna (*there is no) *i*-improvement, like *i*-patient is still the same. *Yea*, if like *u* (a)- sister *a bona ukuthi (*see that*)* no, *i*-patient maybe *a ka gaina-nga i-weight* (is not gaining weight*)*. *Yea*, TB nurse will notice *ukuthi* (that) maybe is it a treatment or maybe a patient is not taking treatment at all. Maybe the treatment is affecting a patient cause we had this patient, he was taking a TB treatment for normal TB, DS-TB, and then patient *siyabona* *ukuthi* (see that) no, *i*- TB *nje (*this kind of TB), *mase si checka futhi* (when checking) a sputum, the results came back, still positive with MDR, that’s when *bam- changa* *ke* (changed) to MDR treatment.

I: So, this patient was DS before and then they were not adhering until they were MDR?

P: *Mm*

I: Okay, and then how did the nurse see that they were not improving? You said they what? – check the weight and what? – the sputum test?

P: *Yea*, a sputum. *Angithi* (isn’t it) after two months, after a patient started medication, TB medication, we collect a sputum to see if there is any improvement or what. But if the results come back still positive, *yea*, then *u notisa lapho (*she notices*) u-*sister *ukuthi* (that) no, this patient is supposed to be discharged but still *a* *kuna* (there’s no) improvement and then sister asked me to collect another sputum, and then the results came back with the MDR results.

I: Okay, and you said you were implementing both the labels and the box, right? So, now with the box, I want to understand that cause you said the patients will take their treatment from the box, I want to understand that did you trust that the patients were really taking the medication when they open the box?

P: Eh…that’s the problem cause immediately after opening a box, on the system it will appears as if the patient did take a medication but there is no proof or evidence that patient did take a medication.

I: Okay, were there any patient who were using the labels or the stickers whose adherence was perfect or good but when they come for their visits, you could see that they are not improving?

P: Yes, the one that I was talking about, the one with MDR had the DAT and when we checked on their platform the calendar was green as if they were taking medication but now when they came to the facility we could see that no, they are not improving.

I: Oh! They were using the DAT.

P: Yes

I: Which one?

P: A box

I: They were using the box.

P: Yes. Yeah, he was using a box.

I: Okay. And any other patients besides this one that you know of, who were using either the labels or the box but not taking medication?

P: No

I: It’s only this one that you know?

P: *Yeah.*

I: Okay, and do you think the DAT, whether is a box or the labels improved the relationship between the health care workers and patients? Did it improve the relationship between the two, the health care workers and the patients?

P: Yes, it’s did.

I: *Mm*, can you think of the patient who was using either the DAT or the box, whose relationship was good between the patient and the health care workers, because of the DAT?

P: Eh…most of them – yes, there was a relationship between our patients and TB staff. *Yeah*, as I was saying even if sister forgot to put the stickers on the treatment the patient will remind this sister that no sister, you forgot to put the stickers on my treatment, how am I going to send message if you don’t put the stickers on my – then *yeah, mm*.

I: Okay. That’s interesting.

P: Mm

I: Uhm, anyway, are there any issues of a stigma that you were aware of with patients that were using the DAT?

P: No

I: There were no cases of stigma being reported?

P: No

I: Okay. Uhm…can you describe now the challenges with the differentiated model of care with the use of DAT? Were there any challenges with phone calls, home visits, the SMSs.

P: No, I had never experienced like challenges besides the one with a reminder, SMS reminder. *Yeah*, besides that.

I: Okay, and then did you have any patient who refused any of the DAT that you were implementing, whether is the box or labels? Did you have patients who said no, I don’t want, when recruiting them?

P: No

I: So, all the patients that you approached, they accepted?

P: Yes

I: Okay, were there any issues of network for those who were sending an SMS, all that? Did they report any issues when they were trying to send an SMS maybe it wasn’t going through?

P: Yeah, maybe a patient will tell you that I was trying to send an SMS but network issues on my side. Maybe if a patient visited the relatives, maybe he/she is struggling with network. Some of them were even calling me that “*iyooh*, sister, I’m struggling with network – I was struggling with network, so, I sent an SMS not exactly after taking a medication, but I did take my medication and then I sent an SMS, maybe after two hours cause I was struggling with the networks.” Some of them were even calling me to report that no. *Yeah*.

I: Okay, and with box were there any technically [inaudible segment] that being reported, maybe patient opened the box, took medication but it didn’t report on your platform?

P: Yes, there was. Uhm…maybe if you call a patient and ask “why didn’t you take your medication? I can see that you didn’t take your medication the day before yesterday and yesterday, what happened?” A patient will tell you that no, I did take my medication yesterday and before yesterday, I did take my medication. But on the system, it appears that patient didn’t take a medication.

I: Okay, and were you able to solve those technical issues?

P: Yes, we were ticking on the system as a patient took the medication, and write a comment that this patient did take a medication but on the system it appears as if a patient didn’t take a medication.

I: Alright, and from your perspective do you think TB treatment can be improved using the DAT, whether is the box or the stickers?

P: Yes, it can be, cause it motivate them to take their medication. We were also doing phone calls, home visits, that’s the easiest way to see if a patient didn’t take a medication and to improve adherence.

I: Okay, and when it comes to workload, did it help in relieving the workload that you had in the TB room?

P: Yes because before DAT was implemented, as I was saying that they were given this green card, so, some of them were supposed even to come every day, so, the sister will tick that okay, you did take a medication. So, now there is no need for a patient to come to the clinic every day to show u-sister ukuthi (that) no, I did take my medication since DAT has been implemented.

I: Uhm…can you please elaborate on those positive changes that you saw with the implementation of the DAT? You said it improved the adherence and also the relationship between the health care workers and the patients – so, now, how can those positive changes been sustained, you know? They let say in the absence of xxxx (organisation name), when xxxx (organisation name) is no longer in the facilities, or the xxxx (organisation name) staff – how do you think those positive changes, good adherence, the relationship between health care workers and patients can be sustained moving forward?

P: I think if these DAT program can continue even if ASCENT is no longer dealing with this technology, they can continue with DAT on their facilities.

I: Okay. Uhm…can you comment on the negative changes now that were brought by differentiated model of care, the phone calls, home visits, and the SMSs? The negative changes – is there any negative changes?

P: No. There were not.

I: Okay

P: The only thing that the patients were complaining about or some of them, were complaining about the size of the box. Yes, because some of them were getting lot of medication, so the box – they were complaining that maybe if the size of the box can like *ithi* -*ikhuphuka ka ncane (*can be increase a bit*)*.

I: Okay, thank you for that. Uhm…so you said that you are calling patients – you were the one who was calling patients. Did you experience difficulties in reaching patients or you were successful in reaching them? Did they always answer their phones when you were calling them?

P: Yes

I: Okay. And now – can you now describe to me what needs to be put in place – what needs to be there in the facilities, as I said in the absence of xxxx (organisation name), that can help or to make sure that the implementation of the intervention continues and those positive changes are sustained moving forward. What needs to be in place – what is needed for the department of health to take over, you know, to be able to achieve? You said you were seeing good results with the adherence and all that, what do they need now when xxxx (organisation name) is no longer there in order to continue to carry out these positive changes – what do they need to be in place?

P: Oh! To train the staff cause sometimes, like in TB room, sister…*ba ya shintshana (*they are rotating). Maybe *u*-sister, like he’ll be working at TB room for six months, maybe after six months *kuze omunye u*-sister (comes another sister), so, if *i*-training *izo* *qhubeka e be khona* (if there could be continuous training) and to train the new staff, maybe if they take the new interns [inaudible segment]

I: Okay, and what are the resources needed?

P: The devices, *yeah*. The devices cause *angithi iya siza nayo kuthi* (because it helps to)– to check on the system, and the boxes, smart pill box and the labels and even human resources.

I: Okay, And who should prepare the boxes now? Who should help in preparing the box? Who do you think should assist with that?

P: The interns, cause sometimes you will find that TB nurse is busy, so, I don’t think he can be able to deal with giving patients *ama* (the boxes*)*-box and doing vital signs and like giving *ama* (the)-patient *i*-treatment.

I: Okay, and that person, who you think should assist in preparing the box, should it be a person from the facility, or it can be anyone even outside the facility?

P: It can be anyone as long as they get a training, *yeah*.

I: Okay, and you mentioned technical issues with the box where patient would open the box, but it could not report to the platform that a patient has opened the box and take their medication. So, now, who should help to assist solving those technical issues? Who do you think should help?

P: Eh…*i*- DOH is the one who is supposed to deal with those issues.

I: Okay. Uhm…did you capture some of those challenges that you mentioned with patients who were not taking medication and also patients who reported that they were taking medication, but it seems like the box was malfunctioning or the issues with network hence they could not send SMS? Did you capture somewhere those things?

P: Yes I captured on differentated care log and Everwell system

I: Okay, and what are your thoughts with patients, with other chronic diseases – do you think the box, or the labels can work for them cause I believe this was only for TB patients. So, now, do you think the box, or the labels can also work for patients who have other chronic conditions or diseases?

P: Yeah, it can help. It can – especially those who are taking ARVs, cause HIV goes hands in hands with TB. So, most of our patients who were taking TB treatment were also taking ARVs. So, they were even asking us *ukuthi* (that) no, is it possible if we can get the boxes or if these boxes, as I was saying that if *i*-size *ye* (of ) box *ika khuphuka* (increase), so that they can be able to put even their ARVs, *yeah*.

I: Okay, now we spoke about capturing those [inaudible segment] or challenges being reported by patients or the ones that you encountered as you were implementing the DAT. And then you said you were capturing using the…you said is the…what’s the log, the name of the log?

P: Device accountability log.

I: Okay, and you were also capturing on the platform.

P: *Yeah.*

I: So, now I want to understand that is there any other way that those things were captured in the TB room – was there a system that these other health care workers including the sister, and whoever who is working in the TB room – was there also a system or a way that they were capturing some of the challenges of patients that were not taking medication, or not improving?

P: *Yeah*, there are even i-updating. Eh…they had even the forms where they are writing if they get a phone calls cause some of them were also doing phone calls, but I was the one who was doing in most of the time. We were even recording or writing on the blue files, on their files, that I tried to call a patient, but the phone was off. Maybe if a patient has missed a date for treatment, we were also calling those patients. If a patient was supposed to come maybe on Monday, then patient didn’t come, we were calling them and writing on their blue files.

I: Okay. And were there any gaps in the way that the intervention has been delivered?

P: No.

I: Okay, and do you have any suggestions on how to improve the device furthered, the box, the labels, the EVERWELL platform?

P: No.

I: Okay.

P: Besides increasing i-size of i-box.

I: Okay.

P: *Yeah* if the size of the box can be like increased.

I: Okay, then any improvement with the differentiated model of care, the phone call, SMS, home visits?

P: No, I don’t have any suggestion.

I: Okay. Thank you so much for giving us your time. We have come to an end of our discussion and the time is now 1:57PM
