# Supplementary material for: Acceptability of Digital Adherence Technologies to support people with drug-susceptible TB in South Africa
Source: PLoS One. 2025 Sep 24;20(9):e0332103. doi: 10.1371/journal.pone.0332103 (PMC12459780; doi:10.1371/journal.pone.0332103)
Supplement: S4 File — (ZIP) [file pone.0332103.s004.zip › S4 Transcripts/HCWs and Stakeholders/IDI 5- HCW.docx]

**TRANSCRIPTION NOTATIONS**

| **Label Key** | **Meaning** |
| --- | --- |
| **I** | Start of each new utterance by the Interviewer |
| **P** | Start of each new utterance by the Participant |
| **N** | Note taker |
| **{ }** | Indicates that details were changed or pseudonyms were used to anonymise data |
| **( )** | Indicates the description provided to anonymize data |
| **XXX** | Words were omitted to anonymise data |
| **-** | Breaking into a sentence by the next speaker |
| **…** | Pause or drawn-out words |
| **[ ]** | Indicates noise made, e.g. [laugh], [sigh], [pause] |
| ? | Beginning of utterance by unidentified speaker or questionable text |
| **[inaudible segment]** | Unclear section of the recording |

I: Okay. Thank you so much for agreeing to participate in our study. Can you please allow us to audio record this interview?

P: Yes

I: We can record?

P: Yes, you can.

I: Okay. PID number it’s xxx. Date of the interview: xxx (interview date). Place: xxx [clinic name]. Time of the interview it’s 10:01. Type of the interview: Health Care Worker. Facilitator: xxx [interviewer’s name]. So, when you speak try to be loud so that we can hear each other *neh* [right]? So, that it’s clear on the audio.

P: Okay.

I: *Yah* [yes] so, what is your tittle, your current position at the clinic?

P: Uhm, I’m a Professional nurse-

I: Mmm.

P: Currently working under the TB focal point, yes.

I: Okay. So, what are you doing in terms of TB management? What exactly are your responsibilities?

P: Okay, I’m a TB, Head TB Sister in the facility-

I: Mmm.

P: So, what happens is, patients come here for either assessment, screening or initiation on TB medication. And also, for further management after they have started our TB treatment. So, I’m a responsible person in making sure that when they start their treatment, they adhere to treatment, if they don’t, I have to send Health Care Workers to go and, and find them. And also try and notify those who have passed away.

I: Okay.

P: So, that is my responsibility.

I: Okay, that’s very interesting. So, in line with the ASCENT project, what exactly are you doing in terms of TB management?

P: Okay uh, at first, I didn’t understand the ASCENT project-

I: Mmm.

P: Mmm, completely because it was xxx [Intern name] was just, *wangilethelwa* [she was brought] she was just brought in without xxx organisations name uhm, giving me prior time to know what this ASCENT project is, how will it work and what the, the people they are sending are going to be doing in the facility.

I: Okay.

P: But as time went on, that’s when I understood that it is helping us in monitoring the patient’s treatment profile per say.

I: Okay.

P: *Yah* [yes]

I: Okay. So, after you’ve found out how is this project works, how did you feel?

P: Mhm, at first, I was a bit sceptic because most of our clients, they are not people who are reliable, by saying people who are not reliable, we have a lot of drug users.

I: Mhm.

P: So, these people, they turn to take- so they were taking boxes, and not using them. Some thought it was bombs, some sold them but we did try and recover those boxes. Some they will take them, but just leave it at home and then a person continues working like wherever he’s staying in the drug area.

I: Mmm.

P: And when we try and call, that person is not there.

I: Okay.

P: Again, what we expe-, I experienced was, people would come to complain that this box is making too much noise.

I: Mmm.

P: “It’s disturbing me, I’m not staying alone. When it says *twi,* *twi,* *twi* [imitating the box alarm]”-

I: Mmm.

P: “Now it disturbs other people. And again, it doesn’t create privacy for me because already now, the people, if I’m trying to hide that I’m taking the treatment, people now know that I have a box that goes *twi,* *twi* [Imitating the box alarm].”

I: Mmm.

P: “So, that for me is creating a problem.” The other problem they came across was, it was a little bit of a challenge when they have to go to work, let’s say maybe a patient uh, set the time that they’ll take the treatment maybe in the afternoon or in the evening, now they have to work night shifts, they complain that they can’t carry that box to work.

I: Okay.

P: “Now that creates a challenge for us.”

I: Alright. Thank you so much for reflecting all of, all of those patient’s experiences that you’ve heard about. So, from you as a Health Care Worker, how do you see the, the implementation of these DAT s in the clinic?

P: Okay, uhh it, it’s a good project, if I can say, but the way it was implemented, it was not actually, we were not given time to understand what the project is and how it is going to work.

I: Okay.

P: We were just given people who came with boxes and tablets, and they were telling us “This is what we are going to do and we are going to show you this and this” and they were orientating us along the way.

I: Okay.

P: So, if maybe we were given time to be trained also on this project and understand - It would have made a bit of a difference for us as Health Care Worker.

I: Okay.

P: For me as a health care worker because at times I found it challenging that xxx [intern’s name] is not here, who was previously stationed here, maybe she’s sick or on leave. Now I have to be the one issuing out the boxes, sometimes it’s the stickers, so it becomes a bit tricky because somewhere along the lines, I was not orientated to how it’s working. They would come back, patients with uhm, they say the battery is not working, I don’t know how to change the battery [laugh] or how it works, I’m not technological survey [laugh] uhh, introduced. I was not introduced to this box.

I: Mmm.

P: And how it works.

I: Okay. So, you basically did not attend the training?

P: *Yah* [yes]

I: Oh, okay. Do you know who went for the training in this facility?

P: No one went.

I: We invited nurses.

P: No, no one.

I: Okay

P: No one.

I: Okay, we, okay, okay, no, it’s fine, and we are taking note of the, of the importance of training nurses as you saying that it would make it easier for you.

P: Mmm.

I: So, so, now like you are telling me that the intern had to train you and to show you how it’s done, you know. So, what is it that you were involved in, when, when, when now you’ve got used to it and you’ve got to understand, what is exactly happening, what were you doing?

P: Okay, so at first uhh, when you would initiate the, the patient-

I: Mmm.

P: And have to issue out the medication-

I: Mmm.

P: Now I know what the box is for, and how it works.

I: Mmm.

P: Mmm, I would teach the clients to say “this is your box, you take it home, this is how it works, when it starts to have this green *tig*, *tig*, *tig*, *tig* [beeping] it means it’s time to take your medication. If it’s faulty, if you feel that it’s not doing the *tig*, *tig*, *tig*, *tig* [beeping] thingy, bring the box back so that we can, we can check. And also keep it far away from the children”-

I: Mmm.

P: Because they can manipulate how it works.

I: *Yah* [yes]

P: And also, the tablet, it was also helping us in tracing on how the patient is taking the medication.

I: *Yah* [yes]

P: Because it shows us when the patient took the medication, if maybe the following day or two days the patient is not taking the medication, it will show you in red. So, that for us was a bit of an easier way of tracing these patients who are not taking treatment on time.

I: *Yah* [yes]

P: And also, it, it gave me easy access to patient’s details because sometimes patients give us uh, false information in the files, but as to compare when they see that you are using a tablet now, you are putting their information and you call them instantly here when they are here to confirm their number, that’s when we got to re-correct now information now.

I: Mmm.

P: You see, so now it helped us in that instant.

I: Okay, this is very interesting. So, how else is the, is the box and, and you know, this capturing patients on tablets on the system and the tablets is helping you as a nurse? I can see that you reflected that it’s helping you, you know, uhh, it’s easy, it tells a patient, it’s easy for you to explain to the patient how the box works, and you are able to, to gather the correct information, how else is this programme helping you as a nurse in the facility?

P: I can say it make, it’s makes life easier for us because based uhm, on the old uhm, region where we would used to write all the information and it, it’s terrier to go back, like now I have to come and check all the patients TB files one by one to say “okay this one didn’t come for the appointment, this one didn’t come and also check with the data, data room, as to, which patient didn’t come, which one missed the appointment”. The tablet, you just go, you check, you put the patient’s ID number, their name, it just shows you everything that is needed. When to call or if the patient is now due to, to finish their medication, they are due for their sputum, it, it makes life easier for us.

I: Okay.

P: Mmm.

I: Okay, that’s good to hear all of that sister. So, I would like to know uhh, what you uhm, what you know about ASCENT like, you told me that okay there’s a system that you see how patients are adhering and you know, you give the patients the box and you explained to them how the box works. What else that you think you know about the ASCENT?

P: (……)uh, what else (……) okay, besides the tracing-

I: Mmm.

P: Uhm, the patient’s information fi- on the, on the file uhh, (……)

I: In relation to Differentiated Care, what do you, what do you know, what do you do exactly in differentiated care?

P: Okay uhm, I can say-

I: Are you involved in calling patients and when they are, when you see nonadherence, do you sometimes call the patient or there’s somebody responsible for that?

P: Okay, in, in that department, there was someone responsible for that, who was xxxxx (organisation name) trained but if she can’t call them, we use our own phones to call them. If we can’t get them through the phone, that’s when we do the physical tracing.

I: Okay

P: *Yah* [yes]

I: How does the platform helps you to see people who need call and uhm, home visit, do you use the platform?

P: It makes it faster to, to see who needs to be traced-

I: Oh, how-

P: That’s why I was, because when you enter the patient, you know we had an appointment system to say okay on Monday we are seeing so, so many patients, we retrieve those files prior. And also on the tablets, it tells you that this patient is, is coming for an appointment on such and such a date.

I: Mmm.

P: So, when that person didn’t come, it already gives you, it awakens you alarms to say why this patient missed their appointment.

I: Okay

P: You, see?

I: Mmm.

P: I can say it makes it easier for you to go back and trace these patients because there in the, in the system, it shows that this person has missed treatment for a certain number of days.

I: Mmm.

P: Now you must be worried, if the patient is still alive or they are dead-

I: Mmm.

P: Now you need to stand up and go check on that patient.

I: Okay, alright. So now according to the differentiated of care, we call patients, patient gets an SMS and if they are missing medication, we also call them and uhm, if they are missing medication two or for about third and forth time now which means they qualify for this home visit so that they can be fully supported *neh* [right]? How do you see that in uhh, in the TB, in the TB management, how do you see that process? Is it something that is uhh, how do you see it?

P: Okay, it is something that is good because if they get an SMS, it reminds them-

I: Mmm.

P: To say okay I’ve missed the box, but the SMS is there, but then another challenge is when we get senior citizens who are not technologically survey, those people can’t read SMSs [laugh]

I: Mmm.

P: So even if they get an SMS, they just turn to ignore.

I: Okay.

P: When we don’t get them on the phone, we have to do physical tracing.

I: Okay.

P: But the, that one for calling them, the SMS, it, it’s helping us a lot.

I: Okay.

P: Mmm.

I: Alright. That’s good to hear that. So, if you have to explain uhm, what is a Digital Adherence Technology intervention to another health care worker, what would you tell them about, what would you say to them?

P: Okay, I can say it’s technologically survey.

I: Mmm.

P: Uhm, programme which helps us in patient’s treatments adherence and compliance and also in tracing and seeing if patients are missing their treatment.

I: Mmm.

P: So, it’s a technologically service study that shows us how to follow up on our patients-

I: Mmm.

P: Rather then doing the physical paperwork, you can see it on the box or on the tablet.

I: Okay

P: Mmm.

I: So, I can see that you are reflecting in terms of your health care worker now, this is how it works for you. For a patient, what would you tell another health care worker, how is it going to help this patient, this technology?

P: Okay, for the patient, it’s going to remind them on how to take their treatment.

I: Mmm.

P: And also, it’s going to increase adherence.

I: Okay.

P: Mmm.

I: How does it increase adherence?

P: When you have a box in your house and it keeps on flickering-

I: Mmm.

P: Automatically, it will either remind you or it will annoy you to say let me just take the treatment and get it over here.

I: Okay. Alright, that’s good to hear that uhm, please describe your role in the differentiated of model of care like remember I told you about the differentiated care, what exactly are you doing with that uhm, with those activities, the calling, and the home visits, what exactly are you featuring?

P: Okay, okay, first uhh, on a weekly basis I diarise patients whom I have to see- a doctor Once those patients miss their appointment, my role is to call them uhm, to find out why they missed their appointment, so if two days pass and I can’t get hold of them on the phone, I have to send people for physical tracing.

I: Okay

P: Mmm.

I: Okay, so do you do that alone or you are sharing those responsibilities?

P: Uhm, currently, I’m sharing those responsibilities-

I: Mmm.

P: But not with the Professional nurse, but with other Uhm, Ward Based Health Care Workers.

So, for me, I fill the forms for physical tracing and give them to say please go to trace these patients for me if I can’t get them telephonically.

I: Okay.

P: And also, the information must tally with that from the xxxx (organisation name) team also, okay we also have the xxxx (organisation name) team who also help us in physical tracing, *yah* [yes] and also on the system for Tier. The, my information must tally that this patient has missed so many, so many days for treatment.

I: Oh, okay.

P: Mmm.

I: Okay. Do you know what uh, tasks list is this on the tablet, are you familiar with task list?

P: Ehh,[no]

I: Okay, you have never used the task list?

P: No.

I: Okay (……) so you told me that you are sharing the work with the other, with the community health care workers, the things that you can’t do here?

P: Yes

I: Okay, do you win when you go and look for the patient at home if you are struggling to get hold of them on the phone?

P: Most of the time they find the door locked or people open dogs for them.

I: They do what?

P: They [laugh] they unlock dogs, the dogs.

I: Oh! At home?

P: Mmm,[yes]

I: Okay.

P: So, it becomes difficult, especially if someone has not accepted that they, they have TB or the, because of the stigma maybe that is attached to TB.

I: Mmm.

P: Or if they are co-infected, the stigma attached to them, so sometimes they don’t want their families to find out what is happening, so if they see people physically coming, they become embarrassed.

I: Mmm.

P: So, it becomes a bit of a challenge.

I: Okay. Yoh, so, what are the acti-, what exactly do health community health care workers do when they finally get them, what do they do when they get to their home? Obviously, the concern is about the medication.

P: *Yah* [yes]

I: What else do they do when they are there?

P: They, because there’s a physical form that the person must sign-

I: Mmm.

P: That they have been seen.

I:

I: Mmm.

P: Or remind her that please tomorrow come to the facility as soon as possible.

I: Okay. Oh so, are there any changes after do, who offers adherence counselling, do community health care workers do counselling at home when they visit?

P: Yes, they do counselling and also, we have our HIV counsellors though they’re not uhm, TB based-

I: Mmm.

P: Uhm, orientated but when they come to do counselling, they do that, they do give counselling.

I: Any changes that you are noticing after the adherence counselling on those patients that are struggling?

P: I can say 80% out of 100

I: Mmm.

P: We do see changes, but the 20% is still, is still at struggle.

I: Okay.

P: So, what the facility has done was that people who come here for TB, they should not wait for long at the facility because we thought maybe it’s one of the reasons that makes people not want to come to the facility. So, our facility has a high headcount of uhh, high influx of people who are coming into the facility. So, find that sometimes they get stacked-

I: Mmm.

P:

I: Mmm.

P: Because of the waiting time and all that, so as a facility we decided that you know what, once there’s a TB patient coming in, the staff, the, the, the reception staff must retrieve the file and we come and see the patient and then we dispense, usually we don’t dispense, we let them go to the pharmacy-

I: Mmm

P: And their treatment is dispensed but now as a, as a way making sure that they get treatment fast and they don’t stay long in the facility, to encourage them to come back-

I: Mmm.

P: We dispense for ourselves.

I: Okay, yoh that a good strategy for them, really, it’s nice to fast tract them.

P: *Yah* [yes]

I: And make sure that they are at home.

I: *Yah* [yes] so earlier on, right at the beginning of our conversation, you mentioned something about the drug users, I just want to know the percentage of the drug users uhh, you just, you worried about the technology being given to drug users-

P: Mmm.

I: Or not using it and all those things, I just want to have an idea, how many, are the patients on drugs uh, that, that are also have TB?

P: *Yah* [yes]

I: Is it a lot of them?

P: I can say about 25%

I: Mmm.

P: Of our patients are drug users.

I: Okay.

P: Known drug users and heavy drug users.

I: 25%?

P: Yes.

I: Okay. So, how, how is the technology helping patients that are on drugs?

P: I can’t say it helps them because it’s very hard to, to work with the drug user. You- they come here when their health status is deteriorating.

I: *Yah* [yes]

P: So, we would refrain from giving them boxes but giving them the, the sticker, the sticker one for SMSing or we would ask a family member, you know, a trustworthy one to say can we give you this box for this person, but it would be in vain because this person wouldn’t even be at home most of the time, they stay wherever they staying the drug arears.

I: And then, and they don’t go with the box to where they are staying?

P: Either they want to sell that box [laugh]

I: [Laugh]

P: Or they want to sell that box, or they just leave that box at home because they think, they thought because of when I interviewed one of the patients to say “why do you leave the box at home” she said this thing, you guys follow me through this box, so-

I: Oh, they believe that you are going to follow them.

P: Mmm [yes]

I: Mmm.

P: So, they say “*letlo* *tracer* *kayona”* [you are going to trace me with it] this box, so no, I don’t wanna be found, if I want to come at the facility, I’ll come on my own.

I: Mmm.

P: So, that’s when we, we realise that drug users it-

I: So, even after explaining that it’s just for-

P: For treatment purposes.

I: *Yah* [yes]

P: *Yah* [yes]

I: To remind you. They still believe that it’s for tracing them?

P: Yes

I: Okay.

P: They think we’ve put a tracker on it.

I: Mmm.

P: Mmm.

I: And, and sometimes they are even attempting to sell it?

P: Mmm (yes).

I: Yoh

P: These people can sell anything [laugh]

I: [Laugh] *yah* [yes] I get that, and so what do you think can be done to improve uh, I’m really conc-, this is a group that I’m concerned about because they are also sick, and they need support as well-

P: *Yah* [yes]

I: So, in terms of *ama* technologies and all these systems that we are bringing into the facility, especially specifically to this adherence technology-

P: Mmm.

I: What can be done to support those people? What, what can we do to improve? What best can we do for them?

P: Mmm.

I: For this specific group?

P: Technologically wise uhm, I am really not sure as to how we can help them because already we’ve seen that it does not work for them.

I: The box?

P: Mmm (yes).

I: What works for them, what do you think can work for them?

P: Direct Observed Treatment, they come into the facility and then they drink, so we were helped by the xxxx (organisation name) team that they will go and fetch, physically fetch the patient, they drink their tablets while they are here and then they go home .

I: Every day?

P: Yes

I: Oh, so you mentioned earlier on, the stickers.

P: *Yah* [yes]

I: Are those working for this group?

P: They lie about the numbers, the cell phone number, okay sometimes they give you the right cell phone number to say okay this is my number, but when it comes to SMSing the code every time when they drink, they don’t.

I: They don’t send?

P: Ehh, [no]

I: Mmm.

P: When he come back after a few days they say, “no I lost my phone or I don’t have a phone, what must I do?” Give me another sticker. Mmm.

I: Okay. Alright. Okay. So, do you uhm, did your opinion- okay, so, so when you first heard about the DAT *neh* [right]? I know that you heard it from the intern-

P: Mmm.

I: That was working with you, so when you heard about this DAT, what were your expectation before, you know, you started to work with the intern uhh, supporting the intern on, on, you know, issuing a box and supporting the patient before *nje* [like] after you heard about this-

P: Mmm.

I: Programme, what were your expectation?

P: *Yah* [yes] that was my one and only expectation to say okay this is going to increase adherence in the facility and also the cure rate for TB, it’s going to increase.

I: Okay. Were your expectations changed?

P: [Laugh] not per say, they are still the same.

I: Okay.

P: *Yah* [yes]

I: Let’s talk about that.

P: Because-

I: Mmm.

P: Mmm, once we started using this technology-

I: Mmm.

P: We saw that, more and more patients who were being on the box were more adherent , there were a few of those who were not adhering.

I: Mmm.

P: And also, the cure rate was, was high.

I: It’s improving?

P: Yes.

I: Oh, okay, so your, your, your, your expectations are met?

P: *Yah* [yes], they were met.

I: Alright, that’s, that’s good to hear.

P: Mmm.

I: What else did you think about when you were hearing about this technology, when you first knew about it?

P: I thought it was going to be overwhelming- the load of the patients.

I: How so?

P: These patients has pills, doesn’t have boxes to carry, now we are giving this patient another box, so emotionally that person does not have a right state to, to really understand what we are saying about the box, but as time went on, I saw that they understood, and they used it well. So, my part was more on the psychological aspect.

I: Mmm.

P: To say, will they be able to understand even now they just told this person they have TB; they’re going to give so many drugs to them and now we are also giving a box, are they going to understand what is happening? Are they in a state of mind to understand what is really happening? But as time went on, I saw that I was wrong.

I: Mmm, okay, alright, what were your other concerns about this box except the psychological part of the patient when you look at the box and you giving the patient the box, what were your other concerns about the box?

P: I was not sure if the box was- you were able to lock it.

I: Literally lock it?

P: No, like it, it, it locks itself.

I: Oh!

P: Or sometimes it would have faulty, be faulty and open itself.

I: Mmm.

P: So, there are patients that stay with children, so what if it’s faulty and the child opens and takes those pills, *yah* [yes] that are my, my concerns.

I: Okay.

P: Mmm.

I: Alright, so-

P: Raising from those that I’ve seen, that they say even after they took the medication, this keeps beeping, that could be the problem. So, we’ve heard those patients who say I’ve taken my, my medication but this box keeps beeping. Or we would call the patient saying “why didn’t you take your medication” and the patient would say “no but I opened the box”. On the system it would say this person didn’t open the box but, on their side, they say no I’ve opened the box, so it would be “you sit, I sit.”

I: Mmm, did you experience a lot of that?

P: Mmm, not much, I can say maybe five out of ten.

I: Okay.

P: Patients. *Yah* [yes] they are complaining about that.

I: Oh, then how would you resolve that one when patient says they take their medication and they are saying, but on the system, it says they don’t take the medication, how would you conclude that, how would you handle that as a nurse?

P: Because we, we, we wouldn’t know how to fix the box or we wouldn’t know whether the box has an error, we would ask him to bring the box back and then we give her another one.

I: Okay, and you are saying it didn’t happen more often *neh* [right]?

P: *Yah* [yes]

I: It was not uhm, something common?

P: No.

I: Oh!

P: It happened once.

I: Okay

P: *Yah* [yes]

I: Alright, that’s good to hear that. So, can you please describe the training that you have received about the programme, how, how were you trained on the ASCENT programme?

P: Oh [laugh] I can’t say I was trained-

I: Okay.

P: It was spot on training and some of the things were self-taught

I: Self-taught ?

P: *Yah* [yes]

I: Oh, that’s very interesting.

P: Because I would want to know what, what exactly are you doing on the tablet, in case you are not here what, how would I use this tablet, you know?

I: *Yah* [yes] okay

P: So, I was not formally trained as to how we are using this new technology, the ASCENT, but I was informally trained.

I: Okay. Okay, I note that, so how, how did you feel about how the intern informed you about how it works? Did you get some knowledge on how it works?

P: I can say she was a very informative person on uh how it worked because most of the time when we struggle, I struggle she would show me-

I: Mmm.

P: What must be done.

I: Okay

P: So, I don’t have any complaints on that.

I: Okay. Alright, so in terms of improving on these trainings, what can you suggest to us around-

P: *hakakeriya* training [if we can get training] [Laugh] Eventually *hakakeriya* training [if we can get training] for health care workers to understand what this programme is about.

I: Mmm.

P: And not only for TB sisters. Most Sisters who are doing chronic because you find that most of our patients are co-infected-

I: Mmm.

P: Now this person is co-infected uhh we get another sister (nurse) who’s not TB trained, they still have to manage their patients, we cannot turn them back.

I: Mmm.

P: Now, if I have knowledge as to this ASCENT uh project that is happening, I won’t have a problem with seeing the patient. I would be able to decant boxes and also register them on the system.

I: Mmm, okay.

P: So, such thing should be broad not only specifically, be specifically for TB nurses but for all health care professionals to understand what is happening because if I can say if most people would understand what is happening about this technology even that Sister who’s working that side, they might have uh a person at home who has a box, but they don’t know what the box is about.

I: Mmm.

P: *Yah* [yes] so it goes on give health education to our fellow members to say now we understand it, make awareness to everyone. And this will tops the stigma of saying if I carry a box, they know that I have TB.

I: Mmm, okay, so while you’re mentioning something about stigma, have you experienced any stigma in the facility because of the boxes?

P: Through some patients.

I: Mmm.

P: *Yah* [yes] they would refuse to take it uhh they say now people would know that I have TB, but then we would have to explain that no this box is not written as to what it is for, it’s the same as the, the pill decanter you find in Clicks or Disc- hem, it just that this one we are giving it to you for free, but those ones they are buying their pill decanters.

I: Mmm.

P: So, we would have to, to go into details and re-assure the patient that we are really not trying to shame you or show the whole world that you have TB, it’s just our way of monitoring your progress and also helping you to remember-

I: Mmm.

P: To take your medication.

I: Mmm, so do you think it’s a bit of a problem or it’s not really a big issue?
P: I can say, with health education, it can be eradicated but for now a few people are still sceptic about it because of lack of knowledge.

I: *Yah* [yes]

P: Not understanding what is about.

I: Okay, so you are recommending a lot of education around this technology?

P: Yes

I: Okay. Who needs to be educated mostly about the technology? Is it the, the, the patients or nurses or everyone-

P: Everyone.

I: Like who needs this more?

P: Everyone, health care professionals as to what the technology is about and how to use it. And also, it should be extended to the patients, not only the TB patients but everyone because you’ll find that there’s a, there’s a lady who’s taking her hypotension medication but has a child who’s taking TB medication this side, so they are able to understand why this child has a box in the house.

I: Mmm.

P: And it can help also to say, this box is to help your child to take their medication.

I: *Yah* [yes]

P: *Yah* [yes] they would also be able to remind-

I: Mmm.

P: Their child to, it’s time for your medication.

I: Oh, so who should do these health talks around the technology on how it works on health care workers and on the patients, who must do this education?

P: Okay, on health care workers I would like XXX Institute to do that, since they have broader knowledge as to what this technology is all about. And then on the community and uhh patients or clients, it can be us health care workers because we, we would be able to explain what is this and how it works.

I: Okay

P: But on a superficial basis.

I: Mmm.

P: Mmm.

I: Okay. So, earlier on, you also mentioned that uhm training should be, training about the programme should be, should be everyone, like all the nurses are supposed to be trained so that they all know about this, who do you think can do the training?

P: For the nurses?

I: Yes, of the, of the programme.

P: xxxxxx (organisation name)

I: Axxxxx (organisation name)?

P: *Yah* [yes] or ASCENT or the ASCENT uhm project [laugh]

I: Project?

P: *Yah* [yes]

I: How long do you think uhm the training for the health care workers about the programme can take?
P: It can be a week to a few days.

I: Few days to a week?
P: *Yah* [yes]

I: Alright. why do you feel like few days to a week?

P: Mmm, I think most of us, we, we are technological survey, you know? So, we would be able to understand a few things when it comes to technology.

I: Mmm.

P: *Yah* [yes] so I don’t think it would-

I: Okay, so-

P: Take a lot of time. But for those who are the older generation, you might need to repeat the same thing repeatedly until they understand how it works. They also need time to re-emphasise something repeatedly.

I: Alright

P: Mmm.

I: Okay. So, you mentioned that we need to train all the nurses on the programme even if they are not really working on TB, Who else in the, in the, in the facility needs to, needs a strong training of this programme?

P: Okay, we have uhh, what we call them, *bababetsang*? [how do they call them?] EPWP (Expanded public work programme )

I: Okay, EPWP?

P: *Yah* [yes] EPWP uhh the, these are our part time employees, they are here on contract basis.

I: Okay

P: But they are from the community, surrounding community.

I: Okay

P: So, they can also impart their knowledge on the boxes to their fellow mates at home. And also, the health care workers, the Ward Based Health Care Workers. They should be trained also, because when we send them for physical tracing, they should be able to know what the box is for. *Yah* [yes] so when we send someone to say “go trace this person” and they find a box, they don’t know what the box is for. So, those are the main people.

I: Okay. Alright, thanks for that. Huh,(……) so, so, so from your perspective as a health care worker, can you please describe the benefits of the differentiated model of care and also this box, like what are the benefits that you can explain for you even this box and the differentiated care that I’ve explained, just strong benefits, that you can tell?

P: Number one, we had a poor uh cure rate in the facility-

I: Mmm.

P: But since this technology came, our cure rate is, is high. Our compliance rate was a bit lacking from our clients, but since through this technology, now compliance is at a higher rate. So, those are the two main things that I see that this technology is helping us. And, in speedy tracing of our patients-

I: Mmm.

P: When they missed their doses, then it makes it quicker for us to trace the patient and see if they’ve missed the doses, so those are the three main things. So, it will highlight in red. So, that for us it would be an alarm to say ah “this person we must, we must see as to why they not taking the treatment.”

I: Alright. Okay, from the patient’s side, what are the benefits of the technology and the differentiated model of care?

P: From their side-

I: Mmm what are your thoughts?

P: [Laugh] I can say it reminded them to take their treatment. *Yah* [yes] so far uh it would, the only thing that would be a challenge was to bring, for them to bring back the box. Because I don’t know whether they became attached to the box or maybe they thought the box is for them for ever.

I: Okay

P: *Yah* [yes] so, but either than that, I think it reminded them to take their medication, which is good. Sometimes as humans, we do forget. So, when you have something that constantly reminds you, then I don’t see any problem.

I: Mmm

P: And they, they completed their, their treatment course within a record time. Also, that for us was, was marvellous.

I: Okay. So, you mentioned earlier on that uh the technology has assisted you to, to, to increase the adherence-

P: Mmm.

I: And it also helps you to monitor the treatment adherence per patient. Before we came with this in programme in this clinic, how were you monitoring the treatment adherence?

P: Mmm before, we would have to retrieve files, physically retrieve files-And go to the data room, so the data would uh open the Tier, there’s what we call Tier.net-

I: Yes

P: Mmm so, we have to compare what is on the file and what is, what does Tier says. So, sometimes it, it takes time, it’s time consuming, so that what we would have to do. But since this thing came, you just request, it shows you that, this person has missed doses. So, and also, we had these big green cards-

I: Mmm.

P: That we would issue out to the patient-

I: Mmm.

P: To say “when you take your medication you must tick” so, sometimes they would uh, falsify the information and say we drank, they tick that they took their medication but in actual fact they didn’t take it. So, for us, it wouldn’t, it was not reliable, as to compare to the box, the box we are sure that this person has opened the box, and not sure if they took it or they just open the box and throw the, the tablets away-

I: Mmm.

P: That’s another way but at least it was a bit of a re-assurance to say at least they took their medication.So, with the green cards, sometimes they would come back with empty-

I: Without tick?

P: *Yah* [yes]

I: So, do you address that? What does that mean to you?

P: More health education because it means this person doesn’t understand, so we had to re-emphasise, emphasise to say, “if you are not taking your medication, it shows that you must take, if you take it, you, you must tick, if you didn’t take it, you must do a cross” so some of them they, they would understand, some would say “I don’t have time to do this.” They say “I don’t have time, I’m busy at work, what if I’m at work and I have to just take my medicine, I’m busy, sometimes I forget Sister, I was at work or I was busy, so I would forget.” So-

I: So, compared to the box, how can you compare the two?

P: The two are not comparable.

I: Mmm.

P: Because the box, it shows directly. With this one I would have to wait for the patient to come-

I: *Yah* [yes]

P: Physically and show me. So, with this one, I can monitor while the patient is not-

I: Here

P: Is not here.

I: Alright. Okay, so now I’ve heard all the benefits, you know, about the technology, you know, the differentiated care that you’re reflecting to us. Can you please tell us about the challenges now of implementing the differentiated care and actually giving patients this smart pill box? What are the challenges that you came across or that you think uh are coming with this programme, what are the challenges?

P: Number one: the storage of the box.

I: Storage?

P: Yes

I: Where?

P: Where we going to store those boxes.

I: In the facility?

P: Yes

I: Okay

P: [Laugh] So it means you guys must give us maybe a place or a box or something where we can store those boxes, maybe a cabinet or something, because at the pharm-, if you put them at the pharmacy, the pharmacy it’s pharmaceutical-

I: Mmm.

P: Things, here in the TB room, it’s not control, anyone can open the carboard, they are not locked. So, we need something where we can control, to say okay we have stock control of how many boxes and we can control the stock. So, for now, it’s something that we don’t have, we would, we just spoke to the pharmacy to say okay for the meantime, just put the boxes for us.

I: So, the boxes, you are keeping them in the pharmacy for now?

P: *Yah* [yes] for now. But it was just an agreement to say, “when you guys have your own space for these boxes you must remove.”

I: Okay

P: *Yah* [yes]

I: So, you suggesting this thing like a cabinet-

P: Yes

I: That is going to keep the boxes?

P: *Yah* [yes]

I: Oh! A locker

P: And have a register as to when the patient returns-

I: The box?

P: The box. And we make them sign.

I: Alright. Okay, what, what are other challenges that you experienced in implementing the technology?

P: Okay uhh the other challenge, it was these drug users that didn’t want to return the boxes.

I: *Yah* [yes]

P: *Yah* [yes] that was the other challenge. And another challenge was uhh the faultiness in the boxes.

I: Mmm.

P: *Yah* [yes] patient would say the battery is not working or the battery needs to be charged, so they would have to come back to the facility.

I: Mmm.

P: From this local region, so they would have to travel all the way. Sometimes they would make them to say, “I’m not coming; I’ll see you on the other appointment.” So, on the system it will say the patient didn’t take the treatment while they are taking, they are really taking but on, on the system it says-no

I: It’s not showing?

P: Yes

I: Okay, so the facility, I’m aware that this facility was using the stickers, stickers that you mentioned earlier on. What are the challenges of, of, what are the challenges and the benefits of using this, the, the sticker?

P: I can’t say we had challenges with the sticker per say, because most of the patients, they would SMS, some they would, if you forgot to give them the sticker after you’re done, they would remind you to say, “please give me the stickers so that I can SMS.”

I: Oh! The patients were reminding you about the stickers.

P: *Yah* [yes] [laugh] to say please give me the stickers so that I can-

I: SMS

P: SMS

I: Mmm.

P: Huh, the only challenge was the senior citizens because they would say “I can’t use the phone to SMS,” you know, so those are the ones we decided to give the box-

I: Okay

P: To. *Yah* [yes] so but those who are clever, who are, who can use the phone and SMS may- they would do it.

I: Okay

P: Mmm.

I: They were fine with the sticker compared to the box?

P: *Yah* [yes] they were fine because sticker is just small.

I: Okay

P: *Yah* [yes] compared to the box, the box is big.

I: Alright. So, were the older people happy with the box compared to the sticker?

P: The older ones because they are not, they can’t use the phones, so they were okay with it.

I: With using the box?

P: Mmm.

I: They didn’t mind the size of the box?

P: Well, they would question “why the box” [laugh] “why are you giving me the box.”

I: Mmm.

P: Then we have to go in details-

I: And explain.

P: As to why we are giving them the box.

I: Oh, were they happy with how the box functions, what is it that the box is going to help them with, when you explain to them why the box?

P: No, some even suggested that we should, maybe the box should vibrate.

I: Mmm.

P: Because the *twi* *twi* *twi* *twi* [beeping] is annoying [laugh]

I: So, they wanted the box to vibrate?

P: [Laugh] yes

I: Okay. Are they going to hear it if it’s vibrating?

P: [Laugh] I don’t know.

I: Okay, okay, okay. So, from your perspective as health care worker, can TB treatment be improved by using these technologies?

P: I can say it has improved.

I: Okay

P: *Yah* [yes]

I: Alright (……) okay uhh, please elaborate on the positive and the-on the positive and the negative experience of using- that you have experienced ever since we implemented the technologies.

P: I think I have already highted them. The positive is the high uh cure rate and it improved treatment adherence.

I: Mmm

P: The negatives uh is that less people were trained about it. So not many people were very informative about it-

I: Okay. I hear you.

I: Alright. Okay, so earlier on, you spoke about the sticker, using the sticker and the box in the facility. Were there patients who refused the box, and then say *hha-a* [no] I want the sticker, or other way around?

P: Mmm, I can’t say they refused-

I: Mmm.

P: Per say, because you, you, you as yourself you assess the patient-

I: Mmm.

P: To say can they, are they reliable enough to, that they would text you, if you give them the sticker or maybe we should just give them the box

I: Mmm.

P: Because the box will directly be giving you, you assess based on, you give them based on your assessment.

I: Okay. So, you spoke about the positives, and you said, you know, the, the programme has improved your adherence, cure rate and you getting patient in terms of reminding them to take their treatment. How are these uh positive changes that we brought in the facility because of the DAT can be sustained?

P: Mmm how can they be sustained?

I: *Yah* [yes]

P: Okay, continue bringing us uh people like xxx [intern name] and xxx [intern name].

I: Mmm.

P: To support us

I: The interns?

P: *Yah* [yes]

I: Okay

P: When the interns are here, I know when I’m not here, the intern is there.

I: *Yah* [yes]

P: To give feedback. So, support from xxx the implementing organisation.

I: Okay. So, without the, the support from uh xxx the implementing organisation, how can this programme be sustained? Do you see it moving smoothly without xxx(Implementing organisation) support?

P: It can, but through training. If they train all the health care workers, then it would be sustainable because it won’t be one person who understands the programme.

I: Mmm.

P: Everyone would be understanding the programme on how it works. So, if you remove one champion, the rest who don’t know will be stranded.

I: *Yah* [yes]

P: But if everyone knows what this programme is about and how it works, then it’s continuous and sustained.

I: Okay. So, as you talking about, as you’re mentioning that we need to do the training?

P: Mmm.

I: Okay, alright uh so, you mentioned these, these negative things about the programme, you know uh, how can we improve on those negatives uh negative things that, how can we improve that negative experiences on implementing this project?

P: Okay, based on the boxes, maybe if you make them colourful, lightly because it’s just a white box. we have children that come to collect TB treatment here. They need as flower stickers, you know, it would encourage them to, you know.

I: To use the box?

P: *Yah* [yes] and also, maybe if it had a place where we can place a name tag, to say okay you see, it’s yours, it, it would, it would be something, you know.

I: Okay

P: And also, when they complete their treatment, something to acknowledge, maybe a cup or a pen, to say you know, well done, you’ve finished your treatment. As an acknowledgement. To say, you’ve been keeping our box and thank them for bringing back our property back. So, somewhere somehow it-

I: It will improve?

P: It would encourage them because when you give them the box, you say, you know after you finish your treatment and you bring back the box, you’ll get a cup, you’ll get a T-shirt or you’ll get a pen, you know, it kind of gives a moral, a moral boost.

I: Okay.

P: Mmm.

I: Okay, no, your suggestions are noted. So, please uh describe what system level structures that needs to be improved in order to integrate the two, you know. For now, you are talking about Tier, you know, that’s the only way you can check you know, before the, the programme-

P: Mmm.

I: So, I’ve been trying to think *ukuthi* [that] what can we do to integrate the two, the ASCENT programme and what is happening at the clinic. What can we do to integrate the two?

P: Okay, I can say the two are already integrated because when xxx [intern name] or xxx [intern name] is here-

I: Mmm.

P: I take her to, to the data room to check what she has on the system, does it tally with what is on the Tier. And most of the time it, it tallies.

I: It tallies?

P: *Yah* [yes] so when sometimes at the Tier, they don’t have information, they do call xxx [intern name] and xxx [intern name] has all the information.

I: What kind of information do they check at the Tier, and they check they confirm it to xxx [intern name].

P: It can be physical address, telephone numbers hhhhh sometimes the type of treatment the patient is taking. So, xxx [intern name] also she can show them on her system.

I: On the system?

P: *Yah* [yes] if that side issues that this person has not been taking treatment, on xxx [intern name] it can show that this person has been adhering.

I: Okay. So, they have a way of updating?

P: Yes

I: Okay

P: So, that’s why we’ve linked the two rooms closer to each other so that they both work hand in hand.

I: Mmm.

P: So, it’s just down the passage.

I: Okay. So, any type of staff that you think can do that or the TB team can do the integration, can work together and make sure that, you know, whatever that is on the ASCENT is the same, or do we need somebody to, to make sure that the information is the same or team itself, the TB team can do that, there’s no need for someone new?

P: *Yah* [yes] we already have a team.

I: Okay

P: *Yah* [yes] we have xxx [Intern name] from you guys.

I: Mmm.

P: And we already have xxx [ clinic staff member] from Tier, they work together, *yah* [yes].

I: Okay

P: They are the ones to compile report for me or compile uh the list as to we’ve missed so many patients, we are missing this and this and that, so they work hand in hand.

I: Oh! Okay.

P: Mmm.

I: So, do you think this smart pill box can be used for other chronic or it can be used for TB only? What are your thoughts about expanding for other chronic using this technology?

P: It can, it can be used-

I: Mmm.

P: But bear in mind that it’s still de-atomisation based on chronic conditions.

I: Mmm.

P: Especially HIV.

I: Mmm.

P: Cause already now this small container of ARVs-

I: Mmm.

P: They are complaining that it makes noise, when you shake it, it makes a lot of noise, so people around you will know that you are taking ARVs. So, they turn to take it out from the container of ARVs. So, now imagine if they have a box.

I: Okay, okay. So, even, even after teaching the community and the health care workers about the technology, do you think it would still be a problem?

P: If we, we did health education, I think we would be empowering them, knowledge wise.

I: It would be much better?

P: *Yah* [yes] it would be much better. But if then we just decide to give them boxes, it’s going to create problems. And who is, will be reliable for decanting those boxes and monitoring those boxes because bear in mind, those chronic uh patients it’s lifelong.

I: Mmm.

I: Mmm.

P: Who will be responsible for charging those boxes, who will be responsible for making sure the batteries are working and who will be responsible for [laugh] actually ensuring that those boxes are maintained?

I: Mmm.

P: Maybe they need to be calibrated once a year. Who will do that?

I: *Yah* [yes]

P: Mmm, if we have a person in place for that then it’s okay. But here in TB you just know, only six months then you are done. But that side it’s a lifelong process.

I: *Yah* [yes] okay, I get your point, I get your point. So, what are, what are, what are other systems that you had in the clinic to make sure you’re monitor *ku* TB [at TB] management better, besides here TB dot- Tier-

P: Tier.com

I: *Yah* [yes] Tier.com, what else are you using to monitor and make sure that the information is captured, what are other systems that are you using?

P: Okay, on a weekly basis we have meetings.

I: Meeting?

P: *Yah* [yes]

I: Okay.

P

I: Mmm.

P: There also.

I: Okay

P: Mmm.

I: How do you capture all those activities?

P: Mmm, we do not capture them in physical but it’s just a meeting.

I: Okay

P: For us the xxx [ Intern name] tell us on, on your side, how many patients do you see they have defaulted.” “[Intern name] tell us, how many patients have defaulted.” and then we gather that information and then we send it to Ward, Ward Based Team-

I: Okay

P: To say, okay trace for us.

I: Okay, so it’s more like a report?

P: Yes

I: Okay, then you communicate with those teams to fast-track tracing if there’s a need for that?
P: Yes

I: Oh!

P: But also, before uh physical tracing, we call, WITS they call those who are co-infected. XXX [intern name] also calls those who are TB and also co-infected. I also call those, if they can’t call.

I: Okay. How do you find that as a team? Is it easy thing to do, there are challenges to do that?

P: I can’t say we have challenges doing that because if I can’t call, xxx [Intern name] can call. If xxx [Intern name] can’t call, the WITS team can call.

I: Okay. Are you winning with that, in terms of TB management, is it helpful?

P: Yes, we are.

I: Is it assisting you?

P: I can say, it is assisting us.

I: Okay, okay, so can you please uh tell us about the gaps that exist in our programme? What is it that you have noticed as a gap, something that you can say it something that as a gap?

P: Okay uhm when your interns go-

I: Mmm.

P: They go with their information, they go with those tablets, they go with those forms that we need to fill in, so it becomes a problem

I: Mmm.

P: For sometimes to say okay, what do we do now. Are they going to bring another intern, what do we do now, *yah* [yes] that is a gap we’ve noticed.

I: Okay, that is noted. And we are really at the end of the interview now Sister. And thank you so much for all the information that you have given us. We really appreciate.

P: You welcome

I: Mmm, while we, you mentioned shortly about the gaps, we are, I’m not saying that, you know, the resources that you, you know, if you’re not here, there’s that gap. What is the other gap that you are noticing, besides that, what else do you think it a, it’s a gap in how the project was implemented.

P: You guys implement?

I: Mmm, the project was implemented?

P: Huh, I can’t say the gap per say, but it’s also the facility’s responsibility to have uh what we call, uh campaigns.

I: Mmm.

P: *Yah* [yes] I’ve noticed that not many campaigns are being done to facilities I’m not sure if it’s our facility, but it will be helpful if with the xxxx (organisation name) team, we can make health care campaigns on TB.

I: And also teach about the DATs?

I: Yes, you, see?

I: Okay, alright, that is noted uh is there anything else that you think it’s important and we did not cover, that you would like us to talk about?

P: Mmm, based on the interns-

I: Mmm.

P: At least when you bring them, let us know for how long they’ll be here.

I: How long?

P: Yes. Their, their contracts. Their time of arriving at the facility and when they must leave the facility.

I: Mmm.

P: Because those are some of the things that we are not entirely sure of.

I: Mmm.

P: And there- I can say, their job description per say. As to, what exactly is their job description in the facility, besides this thing of decanting. And what exactly are there for in fact.

I: Oh, besides TB, what else can they do?

P: What else can they do.

I: Oh, okay.

P: Because you find sometimes, they don’t have anything to do.

I: They are doing what?

P: They are just bored when we don’t have TB patients. So, now the poor child doesn’t know what to do.

I: Okay

P: Mmm.

I: Alright. And there’s, there’s some work that you think they can do?

P: *Yah* [yes] maybe they can help here and there, but if it’s not dedicated to TB then

I: Alright, okay I hear that. And you mentioned uh earlier on that you know, you meeting with all the groups like xxxx (organisationa name), the xxxxx (organisation name) and the xxxxxx (organisation name) and discuss about challenges and, you know, who needs to, who needs to done home visits and all those things. Are there any challenges when it comes to home visits and, you know uh after those discussions and, you know, sending the report to the other guys that are working outside? Do you win after this, do you have any challenges around that?

P: The only challenge that we’ve had uh was with physical training, tracing. Most uh physical tracers we get turn in a very painful and shameful manner by our clients. So, that’s the only challenge we have.

I: What, what was happening there, sorry?

P: They chase them away.

I: They chase? Oh! This thing of chasing?

P: *Yah* [yes]

I: Using the dogs and what what.

P: Yes

I: Oh! So, those are the experiences?

P: So, even if we use the xxxxx (organisation name) team, they, they become used to being fetched by car.

I: Mmm.

P: So, when someone must come to the facility now, they, they feel like it’s their right to be fetched by car.

I: Okay

P: Huh, so even if when xxx [Intern name] is not [laugh] calling them, now they feel they are entitled to be called. It’s not their responsibility to come to the facility. They feel, they feel sub-entitled to be called.

I: Because of call?

P: *Yah* [yes] to all these resources we are offering them per say.

I: Oh! Okay so, instead of improving it makes them re, re-

P: Relaxed. To say, someone out there would remind me to take my pills. Someone out there will come and fetch me at home to bring me to the facility.

I: Mmm.

P: Huh, it’s like we, now we are going behind them, begging them to take their treatment, they don’t take responsibility.

I: Mmm.

P: Mmm.

I: Okay. Of which that’s not the phone call?

P: Huh, but it’s a few of them

I: It’s a few, not all of them?

P: Mmm.

I: Alright, so about the DATs earlier on we spoke about the challenges that you had in the batteries, sometimes you know, the patient is saying they take the medication but on the system it doesn’t, it shows red-

P: Mmm.

I: It shows that the person was not taking the medication and you end up calling the patient-

P: Mmm.

I: While the patient is saying something else and now you are saying this, did you have a way of documenting all of, all of those challenges that you had using the-

P: Prior-

I: *Yah* [yes]

P: Okay, because-

I: Do you have a way?

P: XXX [intern name] was the one who was documenting, she had the forms of which after she left, we had a problem of opening that carboard because we didn’t know what-

I: What to do.

P: What was inside, *yah* [yes] so then we stopped, but she was the one who was recording in the xxxx (organisation name) forms.

I: Okay

P: And she had a book also, that she left with, so-

I: Okay, alright. Okay Sister thank you so much for all this. Is there anything important that you want to touch on, before we can close?

P: Mmm, I can say no, no for now.

I: You fine?

P: Yes

I: Okay. Thank you so much Sister, we really appreciate your time and everything. And this is the end of the interview, then time is uh 11:10. Thank you so much

P: *Yey* *iyadonsa* *lento* [this thing is taking so long] [laugh].
