# Supplementary material for: Acceptability of Digital Adherence Technologies to support people with drug-susceptible TB in South Africa
Source: PLoS One. 2025 Sep 24;20(9):e0332103. doi: 10.1371/journal.pone.0332103 (PMC12459780; doi:10.1371/journal.pone.0332103)
Supplement: S4 File — (ZIP) [file pone.0332103.s004.zip › S4 Transcripts/HCWs and Stakeholders/IDI 6-HCW.docx]

**TRANSCRIPTION NOTATIONS**

| **Label Key** | **Meaning** |
| --- | --- |
| **I** | Start of each new utterance by the Interviewer |
| **P** | Start of each new utterance by the Participant |
| **N** | Note taker |
| **{ }** | Indicates that details were changed or pseudonyms were used to anonymise data |
| **( )** | Indicates the description provided to anonymise data |
| **XXX** | Words were omitted to anonymise data |
| **-** | Breaking into a sentence by the next speaker |
| **…** | Pause or drawn out words |
| **[ ]** | Indicates noise made, e.g. [laugh], [sigh], [pause] |
| ? | Beginning of utterance by unidentified speaker or questionable text |
| **[inaudible segment]** | Unclear section of the recording |

I: So, do we have permission to record you?

P: Yes.

I: Date of the interview xxxx (interview date), location is XXX [clinic name] clinic, the language used for the session is English and Zulu, the participants ID, it’s xxx, the time the interview start, it’s 12:07. So, can you tell me what is your title, your current position that you have?

P: At the clinic or at TB?

I: Uhh, both.

P: At TB, I assist Sr [TB nurse] with collecting sputum’s and checking return dates for defaulters. If there is a defaulter, we have to call him/her or we do physical tracing [BP machine sound].

I: So, what are your other roles in the TB?

P: Is to record the sputum’s and then I put results in the case identification and then check turn around time that is alright. I check all the patients at the end of the month and check all those who tested positive for GeneXpert and have started treatment.

I: So, what is the name of your position at the clinic?

P: I’m just a General Worker, I just do anything they want me to do.

I: Alright.

P: Mmm.

I: So, do you work at a clinic level or district level, or national level?

P: It’s clinic level.

I: Okay, now I just want to know what do you know about ASCENT, the ASCENT project. So, if you were to explain to another health care worker what ASCENT is, what digital adherence technology is, what would you tell them?

P: ASCENT works with digital technology they assist people to drink their treatment correctly and it monitors them through a gadget if they open the box that we gave them. If the person does not open the box after a day or two, we must call them and ask why they aren’t opening the box that we gave them. So, if we can’t get a hold of them through the phone after about two or three days, we conduct physical tracing to see what is wrong with that person, why aren’t they opening the box or when they are not sending an SMS.

I: So, how does the health care worker know that the patient did not take medication?

P: We see through a gadget; we link the box that we gave the patient, give them ID code or something we can see that it corresponds with the code and that we don’t give a person someone else's code.

I: Alright, so in this ASCENT we have what we call differentiated model of care which are follow up actions when you see that the patient missed the doses. So, what do you do when you see on the gadget that the patient missed a dose?

P: We phone the patient and ask if they have any problems, why they aren’t opening the box, maybe most of the time some do have food. A person says I didn’t drink the pills because I don’t have any food, others move from one place to another and not take the box thinking about where they are going if the box is going to make that beeping sound [imitating the box sound] what people are going to say. So, they leave that box and say let me remove the treatment inside the box and go with the treatment and leave the box at home because of the beep sound, maybe some are going to funerals and family, whatever those things.

I: So, why do think they leave the box behind?

P: I think they are afraid of that beep sound, they are going to say, “what is ringing ?” because of stigma maybe other people don’t take diseases they way we think, they still have that fear of what other people are going to say. Things like that.

I: Can you tell me more about the stigma?

P: Mmm it is still a difficult thing the issue of stigma because other people use wrong addresses, attend clinics that are very far because when I come to a clinic that is closer to where they know me, what are they going to say when I’m in this queue and they don’t know the services offered in that particular room and others have to cough up sputum’s. Others will think that you have TB when you have to produce those sputum’s, so they feel ashamed that my neighbour saw me on a queue not knowing what are you going to do there.

I: Okay, so you said you checked on the gadget. So, do you have a specific place you go to when you want to know uhm-

P: Can you please repeat yourself?

I: Do you have a specific spot where you go to on the gadget to see who [phone rings] [pause] missed their doses, how do you check on the gadget?

P: Mmm.

I: So, I was saying on the gadget how do you know that this patient is missed doses?

P: Mmm on the gadget there is a patients template, it has green colour it means patients have opened the box and drank treatment and there is also an orange colour it means that the patients has missed the time they are supposed to be drinking their treatment and there’s a red colour, it means that the patient hasn’t drink. It means that they didn’t open the box, so it means we have to take actions as to why that robot colour stream is on red. It simply means that the patient does not comply.

I: So, do you check the red-?

P: The red colour.

I: So, what’s your role in terms of following up with the patients, once you see that there is red, what’s your role?

P: Mmm usually I phone the patient and ask why they didn’t open the box or why didn’t you attend the clinic on the date they wrote on your follow up card.

I: You phone patients and what else do you do?

P: Physical tracing.

I: So, which one do you do all the time?

P: It’s phoning.

I: Which one do you rarely do?

P: Physical tracing.

I: So, how are you working with other health care workers like the TB nurse in or the intern in terms of this follow-up patients, the use of digital adherence technology?

P: Mmm how am I helping them?

I: So, what was their role? What is your role, how are you working together?

P: Ooh the Sr [TB nurse] checks a patient, she admits the patient. My roles is to collect sputum’s when necessary, like on the first visit I collect GeneXpert, ehh, when GeneXpert is positive, I collect sputum for AFB on HIV negative patients and those who are HIV positive I collect sputum for culture and then do follow ups by Lab Tracking results so that I can speed up the process of the patient so that he/she can get treatment ASAP.

I: So, in terms of using the digital adherence technology, the box and the gadget. What’s your role, what’s the intern’s role and what’s the nurse’s role?

P: Mmm my- (.) my role is to check the patient if they drink medication on time and then mmm do follow ups right, according to the gadget and the follow up cards, yes.

I: So, who was registering patients on the platform?

P: It’s XXX [intern name].

I: So, when you first heard about the digital adherence technology, what were your expectations the first time you heard about it, what were you expecting?

P: My expectations was that lost to follow up patients be reduced a bit and that this thing is going to help remind them about the treatment, and I thought the lost to follow up will be reduced. Only to find out those lost to follow-up already have boxes but because of stigma, they can’t move with those boxes from one place to another they remove the treatment inside and then they leave the boxes at home. Then, they leave with the medication.

I: So, did your expectations change after you start using it?

P: Ehh, little bit I can say

I: How?

P: Because uhm out of 50 we have ehh, 6 or 7 lost to follow out of 50. So, I thought it’s going to be 100% cure rate.

I: So, did you attend training on digital adherence technology?

P: Yes, I did

I: Where?

P: But mmm at the A New hotel, I don’t remember the date and ehh, but it was last year.

I: Okay, so do you remember the training activities that happened on that day?

P: Mmm eish it’s been a while.

I: So, what was your first impression, how did you see the training?

P: It was sublime I can say.

I: Did you think it was comprehensive like you were taught everything you need to know on the box?

P: Yes, yes.

I: Do you think it was useful?

P: Yes, it was useful [BP machine sound]. It was useful because back then we had 25 lost to follow-up out of 50, maybe ehh, but now mmm, it’s a plus or minus 6 I can say.

I: Okay and then the training itself, do you think it was sufficient? You were given enough information at that training at the Anew Hotel?

P: Yes, yes.

I: So, do you have any suggestion to improve our trainings? For example, who should do the training and how long it should be?

P: It can be ehh two weeks or and then maybe per clinic neh (right) uhm, three or four personnel can attend the training

I: Which personnel exactly would you like to attend?

P: Like uhm, people who work with TB patients I can say.

I: Which title?

P: Or maybe [bp machine sound] maybe some, what can I say. Maybe the person who relieve the Sr (TB nurse) can also have an idea what is happening with digital, if the Sr is on leave someone can take over or when XXX [intern name] is not here. At least I can continue because now I am left with the boxes, and they are over there ringing, and I don’t know how to disconnect them. So, I have a big task when I get here and the box is ringing in the morning, I have to open it and then close it at least it would have left- when XXX [intern name] left, she should have left that gadget at least I could discharge people, or I can disconnect those boxes.

I: Okay, so how often do you think these training should be done?

P: Maybe twice a year.

I: Okay and what do you think uhm, people should be trained on, or health care workers should be trained on in those trainings? The digital adherence technology, what should they be taught on, what should the trainers teach on?

P: Uhm, maybe they can tell us how to deal with people who still have stigma and how we can convince them to move from one place to another with those boxes, so that a person doesn’t leave the box behind. Explain to them that the box is used for certain pills, when they go to the funeral or go wherever they go, go with the box and don’t say what will the family say when they hear that beep sound. What I have realised is that some change the time they take medication, they themselves decides that they are going to drink at 8 and all of a sudden, they drink at 6 and they leave that box open, some do that. You can see that the box has been opened in a wrong time and they say “no I went to a funeral” such things.

I: So, can you tell me from your perspective as a health care worker what are the benefits of the box and also the follow-ups we do, the SMS, the phone call- [phone ringing] [pause] I was saying what do you think are the benefits of the box and also the follow ups we do on patients?

P: Mmm the cure rate is improving a little bit I can say. Ehh, we are on 70 something percent cure rate because of the digital.

I: What was it before?

P: Mmm about 60, 50 something, 58 thereabout.

I: So, what are the other benefits of this digital adherence technology?

P: Mmm eish at least people have something that reminds them to come to the clinic or the box reminds them to take the pills on time, same time.

I: And what have been the benefits from the health care workers side? You mentioned that it reminds patients, now you as a health care worker what are the benefits?

P: At least the patients come to the clinic on time, there is no patient that comes after 12. So, at least they are early because they have what reminds them on time, previous patients would start by the station to run their errands and when they come back, they see the card at home saying she should come to the clinic, there she is taking a bath around 1 and comes to the clinic around 2. At least these ones they are one time.

I: And then in terms of monitoring patients, how has it impacted on monitoring patients on the digital adherence technology?

P: Mmm-

I: From the way you were doing it before having the box and the gadget and now, how has it changed?

P: Mmm it has changed a lot; the performance now is extra- extra [repeated mispronunciation] what do they call it- extraordinary. Ehh, it’s improve a lot.

I: So, before the use of digital adherence technology, how were you monitoring if the patient is taking medication?

P: Yoh, we were just giving the patient medication and then tick the amount of ehh, the pills that we gave to the patient back then and then we tell them to tick on that- ehh ticking *ka* (on) blue card or green card.

I: How often were you doing this checking of the card?

P: Each and every visit.

I: How often is the visit?

P: Mmm maybe twice a month I can say.

I: And now with the box, how different is it?

P: Mmm we are just checking the empty packets from the box; we suggest that the patients can take the pills and then take the empty bag back to the box so that we can check if those packets are empty, all of them.

I: So, you mentioned that previously you were checking the ticks on the card?

P: Yes.

I: So, how are you checking now if they have been taking medications, instead of the ticks, in addition to the ticks?

P: We check- we advise patients that they drink treatment and then they return empty sachets inside their boxes, at least when the patients come back with 4 sachets, and we gave them 4 sachets and those sachets are empty. We monitor it by the emptiness of the sachets.

I: Okay, and then on a daily basis, how has monitoring changed, monitoring patients everyday changed?

P: Mmm it has changed a lot.

I: When you are using the digital adherence technology, the box, and the gadget. How has it changed the way you monitor the patient instead of seeing them after two weeks, how has it changed?

P: I can say it has changed, but I don’t know how but there’s an improvement.

I: Okay.

P: Yes.

I: Okay, so can you tell me the challenges you have with following up on patients using the differentiated care like ehh, the automated SMS or phone calls or home visits. So, what have been the challenges with those?

P: The challenges are the wrong addresses, that’s what I can say and what else? Even when a person has a child under the age of 5 they say the child just visits and doesn’t stay with me, only to find out that the child is exposed. She doesn’t want other people to see that the child’s takes the same path as the infected person does, other people hide contacts.

I: So, what else are the challenges with maybe calling patients?

P: It’s the wrong numbers, yes, wrong numbers.

I: And how do you think that can be addressed?

P: At least others can have a phone that has airtime on the patients first visits, when they give you that number, you call it immediately so that you can see if it’s working.

I: So, you mentioned that in terms of visits, the challenge can be wrong addresses. What else can be the challenges with home visits?

P: It’s the distance, people can leave xxxxx (province name) and they come to xxxx (another province) to take treatment, that person would even argue with you and say I stay at this particular place yet that person stays in xxxx (province name). You say please tomorrow bring sputum for conversion and then they say, “I don’t have money for transport”. How come you don’t you have money for transport because you said you stay in XXX [location], why don’t you walk at maybe around 8 and come to the clinic and now they tell the truth. You want them here today and tomorrow and they say “honestly, I stay very far, I have to take three taxis to come here, or I have to wait for a bus for a certain time to get here. If that bus leaves me, it means I will not attend.”

I: So, how do you support these patients far in xxxx (province name)?

P: We just go easy on them so at least they can finish treatment and we don’t- what can I say, we don’t desert them. We just be with them throughout their TB journey.

I: So, how is the box assisting this patient?

P: It assist because it reminded that person who completed yesterday, but he didn’t bring back the box, Mr XXX [patient’s name] started treatment in January. He was supposed to bring the sputum and then he had problems and told us he doesn’t have transport money, the bus left him, the bus did whatever such things. I don’t know when he will bring the box because he completed treatment.

I: Mmm and then in terms of giving out the box, what are the challenges with giving the box?

P: The problem is that patients put Panado’s inside and the ARV’s yet the box is for TB. They don’t understand why the box doesn’t remind them also on the ARV’s, so they want something that will also remind them with ART.

I: And uhm, what are your suggestions about using the box for people taking different medications?

P: Maybe we could introduce another box for whatever chronic treatment I can say or whoever.

I: And then do you have patient who refused to take the box when they were offered?

P: No, no. Everyone is happy with the box; some are excited and then others use it accordingly.

I: Okay, and then uhm, you mentioned the issue of stigma, has it been a challenge, can you think about a patient you have worked with and tell us if the issue of stigma has been a challenge when using the box?

P: Mmm who is it that patient who came back from a funeral and left the box here? I don’t want to tell you people’s name there is a patient we call Mr XXX [patient name] we once gave him the box and he was supposed to go to his grandmother’s funeral, and he left the box. So, another person found it at home and I don’t know he put something else inside the box because he once came back with it smelling, and when we asked him why the box is smelling, he said “I went to a funeral, and I left the box.” I don’t know what if they put food or what ”. He didn’t want that beep sound to ring around people at the funeral so he left it.

I: So, why do you think they left the box?

P: It’s stigma, that beep sound, that’s what I am thinking.

I: Okay and then do you have a group of people who you find they have challenges with using the box, like a certain group of people?

P: No, we gave everyone the box and they use it correctly and they are alright.

I: Okay and then you say you do tracing, physical tracing after 3 or 4 days of seeing red?

P: Mmm.

I: Do you always have uhm, staff or people ready to go there?

P: Yes, yes, we have the WBOTS (Ward-based primary care outreach team) from Department of Health, we have xxx (organisation name) ehh, and xxxx (organisation name) helps with physical tracing.

I: So, how do you work with these people, the WBOTS, xxxx(organisation name) and xxxx (organisation name) ?

P: So, I go through each and every file checking the return dates. I go through the file and then I compile a list of people who have missed their appointment dates and even at Tier, they help us with printing lost to follow ups maybe four or five times a month they print those lost to follow ups.

I: So, do you wait for the return date, or you look at the gadget and see three or four missed doses for you to send people?

P: No, I usually go through files every Friday those files I go through them.

I: So, you only check the files, do you by any chance use the gadget to see who needs to be followed up on?

P: No, we go through physical.

I: Physical files?

P: Mmm.

I: Okay, but you mentioned that you always have tracers to assist with the home visits.

P: Yes, yes.

I: Have you conducted a home visit yourself-?

P: Yes-.

I: For a patient who was using the box?

P: Yes.

I: And what was the experience, can you tell us what was the story with that patient?

P: Mmm the story with XXX [patient name] we diagnosed her with TB maybe around April, so we gave her the box and she was alright. She came after two weeks for follow up, after that two weeks we gave her a month treatment and when we gave her pills for a month .She started not complying and I had to conduct a home visit. So, when I got there, XXX [patient name] didn’t have food [people talking in the background]. So, XXX [patient name] is a bit paralyzed, the boyfriend has her social grant card and well as the kids. So, the boyfriend has XXX [patient name] card, she can’t buy food so that she can drink the pills. So, she can’t drink the pills on an empty stomach, sometimes she drink and then sometimes she doesn’t. She opens the box and then sometimes she doesn’t, so I used to go there to a point she got angry with me because I am always pursuing her, but she was supposed to go to continuation phase, I think last- around June she is supposed to go to continuation phase and XXX [patient name] hasn’t been to continuation phase. It is difficult for her to walk to the clinic and bring the sputum bottle for conversion, she can’t because she doesn’t have money and the boyfriend has the cards. So, the family tries to help there and there but XXX [patient name] stands for the boyfriend, she doesn’t have a phone that wake her to come to the clinic sometimes she comes around half past 12 to 1 “no I was walking, I didn’t have money” she can also send a 13-year-old child to attend her follow ups and can’t walk and say XXX [patient name] is paralyzed. The boyfriend has- I am even scared to go there, I am scared for my life when going there he could attack me because it’s like I irritate them or something. So, XXX [patient name] stands with her boyfriend.

I: So, you mentioning that the patient was not taking medication because of lack of food?

P: Yes.

I: And then were not coming to the clinic because they didn’t have transport money?

P: Yes.

I: How do you think these patients can be supported?

P: Maybe we can support them by taking them and go somewhere where they can drink the treatment there and when they are alright, they can go back to their families just like XXX [patient name] case. She will even infect the kids with TB.

I: And in terms of food, how can they be supported food and money?

P: I don’t think it’s right or maybe the food parcels could help there and there at least they don’t go themselves and buy those food because you will think they went to buy food and at the end of the day they buy clothes or whatever with that money. At least food parcels could make a difference.

I: And you mentioned that she was a bit paralyzed, do you know what was the cause? Was it side effects, what was it?

P: She was born paralyzed.

I: Okay, so do you think TB treatment can be improved using the digital adherence technology and the differentiated care which is the follow ups?

P: Aah this thing is alright, the digital thing.

I: Why do you say so?

P: It is alright because it reminds the patients that they should drink treatment and they be alright.

I: So, how do you think it has impacted the relationship between you as a health care worker and a patient, the digital adherence technology?

P: Aah they are really grateful; they are grateful that it reminds them most of the times that they should drink treatment on time.

I: And in terms of you monitoring a patient, what can you comment on that one?

P: I don’t remind them like I used to back then, I wait for a person to come back after two weeks or after a month to see if honestly, they are really drinking or not. Now I monitor each and every hour because they don’t use the same hours, if XXX [patient name] is drinking at 8, XXX [patient name] is drinking at 9, at least I am always checking that XXX [patient name[ opened they box around 9 or 10. It becomes simple.

I: So, can you think about a positive change that the digital adherence technology has brought?

P: Mmm at least per month we could detect around 10 people or so, but now it becomes 7 and those people are excited that they are going to start treatment, they are determined. Others are ART defaulters, so those ones came here already as defaulters and even with the box, there are defaulters, but we conduct follow ups ASAP.

I: So, what’s needs to be improved at the clinic for us to keep it up, let’s say we are thinking of xxxx (organisation name) is no longer here, the intern is no longer here, what is needed for us to keep that standard?

P: At least ASCENT could hire another intern and that intern should be based here each and every day. That intern should not work at three clinics because she can’t monitor maybe 70 patients at XXX [clinic name], 20 patients at the other clinic and then 100 at another clinic. She will miss some at some point that she must make follow ups, I can say, or other patients won’t have the opportunity to receive the box. Sometimes when we admit a patient and XXX [intern name] is not here, we miss that patient and they no longer get the box maybe it was going to benefit them more than the person we gave the box, maybe they would be responsible.

I: So, where should this intern come from, what do you think? So, because the ASCENT will finish the project, xxxx (organisation name) will no longer be here, so who should provide these?

P: Maybe it could be the government, to have something they can do.

I: And then can you talk about the negative changes that the box has brought?

P: No, there are no negative comments or ehh.

I: Do you have any concerns about the patient using the box?

P: No-

I: And you checking the platform and thinking that green means medication has been taken, do you have any concerns about that?

P: No, I don’t have.

I: Okay, you mentioned stigma right, how do you think it can be addressed?

P: Mmm it can be addressed, or you can act as if there is a treatment that you are taking even though you are not taking any treatment. So, you can say when you see me like this “there are pills that I am taking”, you do it so that he/she can be comfortable or if there is something she wants to talk about maybe she can talk. You say now we are living for our children we no longer live to impress our neighbours or anyone, TB kills if you are not drinking treatment correctly. If you are sick and you want to be assisted, you come to the clinic at least you can be like everyone and come to the clinic on the date and time they wrote for you then be able to drink correctly and not look who says what or when.

I: Okay, do you have any concerns about patients who opened the box without taking medication?

P: Mmm no, I don’t think there is a patient who has ever done that because on the first visit we can see that they are very sick and on the second visit you can see an improvement. The patient can come to the clinic on their own and can also stand on their own like they are not like the way they were when they first came to the facility. Usually on the second visit they come on their own and not with a family member, so we believe that they open the box. I: Okay.

P: Yes, there is an improvement.

I: So, can you tell us what system level structure, what is needed for you as the Department of Health to continue using the digital adherence technology? What do you need in place for you to continue without ASCENT, without xxxx (organization name), without the intern, what is needed by you?

P: Mmm maybe I could also have the gadget and a laptop, and they could also bring things that will support me so that I can monitor those patients or maybe they could take me to a training so that I can know more about whatever gadget they are going to introduce.

I: Okay and then in terms of preparing the boxes, can you tell me what was going on about that- before it’s given to a patient?

P: So, you have to make sure that the battery is fully charged, you must make sure that the box is in good condition that you can give the patient and then you explain to the patient how the box works and then link whatever gadget with that box at least that way you can monitor the patient.

I: So, who do you think should be responsible for charging and making sure that the box is in good condition?

P: Intern or I can do it myself.

I: If you have challenges, let’s say technical challenges with the box, have you experienced any?

P: No, no.

I: Okay, if you were to experience challenges, let’s say the patient already has the box and there are issues. Who do you think should be helping with those technical issues?

P: Maybe I can phone the helpline of whoever brought the gadget or the boxes and explain the problem about the box and then they will give me an idea on what to do and then fix the problem.

I: So, if it’s the Department of Health, the Department of Health should have the helpline?

P: Yes.

I: So, do you currently have a system where you are documenting any technical issues you might experience?

P: No, no.

I: So, do you- can you tell me any gaps that are existing in the whole intervention in terms of the box itself and also the differentiated care, which is the follow ups. What gap do you see there?

P: Mmm I see a gap because the intern does not come every day, so the day she is not here that means a gap is opened up at least they could have a back-up if whoever is in charge went on a leave or is sick, there can be a person who fill in and keep the workload flowing. Work should not be put on hold and at least all the patients could be on the system, and we monitor them as much as we can.

I: Okay and then in terms of the box itself how it looks, do you have any suggestions on how it can be-

P: *Ai* (no), it is alright the way it is, I think it’s alright. At least maybe you can make a bigger box, but at a training you once told us that you will bring a bigger box for MDR, so that they can also be monitored.

I: Okay and then in terms of the follow ups, do you have any suggestions for improving the follow ups?

P: Mmm the follow ups have improved at least we now can see immediately that a certain person did not take medication or no longer comes, and we must do a follow up quickly so that the patient don’t develop MDR because they skip medication. At least the digital has made an improvement and then we could see the patient’s improvement when they come here after two weeks.

I: So, earlier on you mentioned that you thought lost to follow-up was going to be solved by the box, but now it’s not 100% solved, you still have some issues with lost to follow-up? What do you think can be done to achieve 100% of no lost to follow-up?

P: At least some complain about transport money, only if they could fill in for social grant on the first visit so that they don’t blame someone else that they don’t have transport money, I don’t have money for food and I can’t come to the clinic or I can’t drink medication, but others deserve the food parcels and others money. As time goes on, we become a family with the patients and we can see what is happening with everyone and other things, so I think social grant will make a difference. It could make a huge difference because other patients due to stigma leave clinics close to them and they come to our clinic, when they must come for follow ups, they don’t have money for transport at least on a first visit they have something and could take treatment correctly.

I: How do you think the issue of stigma can be resolved with now patients leaving clinics that are closer to them and then go to far away clinics, how can they be- how can that be resolved?

P: I don’t know, we talk to them about the issue of stigma, we talk about ourselves and act as if we have the same status and you also are drinking a certain treatment and that people won’t help you with anything and why don’t you attend a clinic that is closer to where they can monitor you and conduct a physical tracing or they see the environment you stay in if it’s suitable for person and even the cleanliness.

I: So, you mentioned that in addition to the box, patients should be getting [people talking in the background] social grant and also maybe food parcels, do you think counselling can also assist in this?

P: Yes, even counselling could help and it doesn’t have to be something for a certain time, it should be an on-going counselling. If you see that the patient is down at that time, there should be someone specifically for counselling because the patient would have had problems. Sometimes even the Sister [ TB Nurse] is busy and concentrates on the queue that is outside and doesn’t see what’s the problem with this person. So, someone leaves and another one comes in and he is too is afraid to mention what’s wrong with him, so at least if there could be someone who is able to conduct counselling each and every day I can say.

I: Okay, so do you have final comments about the digital adherence technology, the box, the platform you use to monitor and the follow-ups? What are your final comments?

P: The digital did a good thing because we no longer lose patients like before, when they come for treatment, they are excited, and they want to know more about it. At least there should be more for adolescents so that they could also drink treatment or when they go to school, they could leave the box and then it reminds them, and it shouldn’t have age restrictions. Everyone has rights and could use the box.

I: Thank you very much for your information, we reached the end of our interview, the time is 1 o’clock- 13 hundred. 1 o’clock.
