# Supplementary material for: Acceptability of Digital Adherence Technologies to support people with drug-susceptible TB in South Africa
Source: PLoS One. 2025 Sep 24;20(9):e0332103. doi: 10.1371/journal.pone.0332103 (PMC12459780; doi:10.1371/journal.pone.0332103)
Supplement: S4 File — (ZIP) [file pone.0332103.s004.zip › S4 Transcripts/HCWs and Stakeholders/IDI 7_HCW.docx]

**TRANSCRIPTION NOTATIONS**

| **Label Key** | **Meaning** |
| --- | --- |
| **I** | Start of each new utterance by the Interviewer |
| **P** | Start of each new utterance by the Participant |
| **N** | Note taker |
| **{ }** | Indicates that details were changed or pseudonyms were used to anonymise data |
| **( )** | Indicates the description provided to anonymise data |
| **XXX** | Words were omitted to anonymise data |
| **-** | Breaking into a sentence by the next speaker |
| **…** | Pause or drawn out words |
| **[ ]** | Indicates noise made, e.g. [laugh], [sigh], [pause] |
| ? | Beginning of utterance by unidentified speaker or questionable text |
| **[inaudible segment]** | Unclear section of the recording |

I: Do you give concern to be audio recorded?

P: Yes, I do give concern.

I: Okay. Thank you, uhhh date of IDI it’s xxxx (interview date). Location: xxx [clinic name], language: English, PID xxx uhhh interviewer: xxx [interviewer’s name]. And the time is now 12 pm. Uhhh so, tell me, what was your uhhh position- uhhh where you were working?

P: Okay, I was uhhh I was still an intern for Research Assistance and uhhh I was working at [facility name].

I: Yes.

P: Yes.

I: Okay, and then how long did you work as an intern Research Assistant?

P: It was a year and (…) it was a year and few months, I can’t even remember because our contract was supposed to end in xxxx (month) then they extended until xxx (month and year), *yah* [yes] xxx (year)

I: Okay, so what were your duties and roles when you were acting uhhh as an uhhh Research Assistant?

P: Okay uhhh my duties were dealing with patients I was working at the TB room-

I: Mmm.

P: And we were testing uhhh patients, patients who had symptoms, we were also giving out results to patients uhhh I was also uhhh I was also initiating patients on our study at xxx (organization name) uhhh the study that was, the study that we convey was uhhh was using, no, letting, letting patients use the pillbox or the stickers. Yes, so basically uhhh I was supposed to recruit patients (TB patients) who are new or who are probably on the, on two months uhhh starting the medication to join the study that was convey by xxxx (organization name) whereby they had to use pillbox or the sticker labels. Yes, so the pillbox uhhh we gave patients to monitor the time to drink medication and with the label, we also did that but for older patients it was kind of difficult and they had issues only older patients, but younger patients had no issue with that.

I: Okay uhhh and how were TB services delivered at your uhhh facility, I mean were you working at the district level, Provincial level, or facility level?

P: It was the faci-, no, no, no, the Provincial actually, *yah* [yes] it was the Provincial level.

I: Okay uhhh now I would like to know what you know about ASCENT, uhhh let’s say if you were to explain to another Health Care Worker or anyone who is within the uhhh Department of Health, uhhh what would you tell this person uhhh- what would you say ASCENT is?

P: Okay uhhh for me it was more based on patients uhhh like they were taking care or they told us to take care of patients uhhh because the TB level was very high so when ASCENT stepped in uhhh ASCENT changed that because patients were more appealing to the, to the treatment, adhering very well also, doing follow ups, *yah* [yes] there were no complaints. So, ASCENT is more of a company that is putting patients first, the patient’s needs first.

I: Okay uhhh so you told me about the tool uhhh intervention, I mean the DATs uhhh the box uhhh also the labels. So, what I want to understand is which one were you implementing, were you implementing one of these or both?

P: Okay I started, oh sorry, I started implementing the, the sticker, the labels, and then after, I saw that my patients actually are more older people because xxx [facility name] is more of a rural place, semi-rural and the older people were struggling to use the sticker so uhhh I implemented two, which was the pillbox and sticker.

I: Okay uhhh if I hear you correctly uhhh you were saying that uhhh you realised that older people were struggling with the labels hence you switched them to the box, right?

P: Uhh, yes.

I: Okay uhhh what I want to understand first is what are some of the other challenges that you were aware of- that uhhh this group of, of, of patients were challenged by?

P: Okay, uhhh the first challenge was they would tell you that they cannot use a cell phone because with the sticker- label it didn’t need a smart phone because you just need to SMS, there’s a code that we gave you every time you drink your pill, *yah* [yes] and also it was tollfree, the SMS is tollfree so you didn’t really need uhhh a smart phone, just a phone, a regular phone is fine. I would give elderly patients who probably came with the children and they do say that they will try to help them, as soon as *umama* *athatha* *ama* pills [as soon as mother takes the pills] and they would send SMS but it would not happen. The elderly patients would tell us that they cannot use a phone, or the SMS does not go through, or they did take the pills, but they forgot to SMS.

So hence I uhhh I asked to, I asked for the pillbox so that maybe younger people would get the labels to SMS because they always on the phones and then the elderlies can get the pillbox. Also young people, some of them didn’t have phones, so we I would also give them the pillbox, who are interested in being in the ASCENT study uhhh yes. So, you would get a challenge with the pillbox whereby the patient is telling you that he or she opened the box but the platform did not alert us. You would find that a patient is busy opening the box for a week but it shows red there by a patient’s name, *yah* [yes]. We would also ask our manager to please help us with that because we were busy calling the patients yet the patient is opening the box, the patient is taking medication but the platform showed us the that the patient is not opening the pillbox and we need to call or we need to do home visit also. So, it was kind of frustrating for a patient who is taking the medication but on the system it would show us that the patient is not taking medication whereas the patient is, but some, some, some patients would lie also, like you would see that okay this one is lying or she’s lying, *yah* [yes].

I: Okay, so with the labels were, were there issues of uhhh the network, you said-

P: Yes

I: Uhhh where the SMS would not go through-

P: Mmm (yes).

I: Uhhh and also there was a challenge uhhh with patients who take their medication as they say but forget to send an SMS. And then you are saying older people sometimes uhhh I believe they; they were not, they didn’t know how to send an SMS-

P: Yes

I: Or using a smart phone. So, what you are saying is that there was also an element of illiteracy there, where older people who were not uhhh- were challenged when it comes to technology-

P: Yes

I: Then you switched them. So, now what I want to understand is how was their adherence when they were still using the labels and after you switched them? Was there a change?

P: Yes

I: Yes

P: Yes, uhhh with the elderly people there was a change because with them, its like, you know, they were pleased that you are here, they were really excited that you are here. And then when you switched them, they would tell you that “this thing helps me a lot, I wish I could keep it and also, like adhere on different medication” because probably you would get a patient who takes TB medication, high blood uhhh- sugar diabetes, a lot of medication. But only with the pillbox uhhh it was for TB medication only. *Yah* [yes] so for them uhhh the adherence changed a lot, it changed a lot, it improved on the TB side, also the other illnesses, *yah* [yes] because like they set one time for taking the medication for all treatments, *yah* [yes]. So, for them, it did improve actually, it did improve from SMSing, because you’ll find maybe the patient, or the nephew is just lazy to send an SMS. But with the pillbox I mean *umagogo* [granny] would just open the pillbox then it rings.

I: Yes

P: *Yah* [yes]

I: Okay. And with the box also, you mentioned that uhhh there were cases where you find that a patient has been opening the box or they claim rather that they were opening the box however on your platform as you say uhhh you’ll see that, or maybe it wouldn’t show whether they are opening or not uhhh and then you thought maybe it was the issue of uhhh what, the technical issues with the platform or the box. So, now what I want to understand is how often did it happen where you have patients who come forward and say uhhh “no I’ve been opening my box” even though maybe on your side it shows that I was not opening it.

P: Okay uhhh with me because my facility was the label clinic uhhh the time they brought me pillboxes, the first issue was I couldn’t, like uhhh I couldn’t set them to work for patients, so I thought probably they would just gonna fix it, yes they did fix it but also as time goes on uhhh the pill, the platform started showing like red for all patients *yah* [yes] then we had to fix it because we don’t know uhhh whether the patient is opening the box or not because it can also go for a month on one patient-

I: Mmm.

P: *Yah* [yes] like there was a patient that didn’t take any medication according to platform then we did a home visit and we found that the patient was opening. So they also told us uhhh if a patient says he or she’s opening the box and then it shows red for a week, there was a manual uhhh manual like to change colour because they get SMSs also or they got calls, they get irritated also, *yah* [yes] we could also change it manually. But the challenge was you need to know your patients, *yah* [yes] because you cannot just change it for everybody because I had this one difficult patient, that guy, yoh that guy, that guy was also taking drugs, so with him, not that I didn’t believe everything but also the mother actually, the mother was the one. We even exchanged numbers, the mother was the one who was like see if the patient is opening the box or not. So that when we call, he must not tell us that he opened the box because number one: the guy takes drugs, he is not at home forever, hence I say you must know your patients, we cannot just assume because a patient is telling you that “I did open my box” and then you just change it manually. So, you should not do that, I mean you should know your patients also. So, I had only this guy who was very, very, very difficult, so I got contacted the mother and a brother actually, *yah* [yes]. So, they helped me a lot to change his platform because his platform was forever red.

I: Mmm.

P: It could go for three months also, *yah* [yes] so they helped me to just like help him also, *yah* [yes].

I: Okay uhhh you just touched on the issue of uhhh not trusting if some of the patients were really taking their medication as they were opening the box uhhh which is quite interesting. So, now what I want to know is uhhh did you believe uhhh that uhhh some of your patients were opening the box, besides this one-

P: Mmm.

I: With others, did you trust that they were really opening the box, oh I mean they were really taking medication-

P: *Yah* [yes]

I: When they were opening the box or they were just opening it and then closing without taking the-

P: Yes

I: Medication

P: Yes, not all the patients I trusted

I: Mmm.

P: Hence I was saying you need to know your patients; I mean you need to know “this person is lying” yes you are not supposed to tell them “*Haaah* *wena* you are lying” [no, you are lying] but you can see when a person is lying. So, there were quite few patients whom it would also show when they go on the weight scale, you would see that “okay, if a patient is taking his medication correct at this certain time why is their weight dropping, number one, because what we know is with TB medication you gonna pickup weight, *neh* [right?]. So, we would see that okay no, this patient probably he’s lying to me, probably he’s just doing this like closing because I also had this one patient, he was a male also, that guy used to take TB medication if maybe his weight, *angithi* [isn’t it] when you pick up weight there’s more pills that you need to drink. So, this guy was supposed to drink five pills because he was over 70kg but he took one everyday like on those five pills and the calender would show green. Every time we open the box when he came for his follow up, you’ll find that he has so many pills, we were wondering *ukuthi* [that] why is this person, how is this person taking medication until he told us that he was taking one pill every day, like one pill, one pill, one pill. So, we would also see with the weight also that you are probably lying because what we know is TB medication makes you eat, makes you eat so that you can pick up weight, *yah* [yes] it was not all our patients we trust actually, *yah* [yes].

I: Okay, and with this patient who was taking, who was supposed to take five but took one, where do you think the problem was uhhh, was it maybe the issue of counselling maybe he was not properly counselled or told as to how many pills he should take per day, or it was just him deciding to do this?

P: No, with him actually uhh it was more of accepting because he knew when he tests for TB, he also knew you should know your status, *yah* [yes] so we test him for status also and he found that he’s also positive, so it in denial of all these uhhh illnesses, *yah* [yes] because they did, I’m sure they did like five counselling with him, and he would agree but do the opposite. So, I was thinking probably it’s in denial, like he has not accepted all these illnesses that he’s going through. And *yah* [yes] you would also get like nurses would also like you know, like being patient with him and I mean you must be patient with him, for him to understand, you need to be patient, because we also did uhhh visit at his place and we found out that okay no he, he is not also copying with household things, *yah* [yes] so *yah* [yes] so with him it was a bit challenging and hence he would like, he would, he would go correct for like two weeks then I had no idea what was distracting this guy, he would come again taking one pill, one pill, one pill, one pill, *yah* [yes].

So, with those patients, me and TB nurse had to come with our strategy whereby we would only give him a week medication because he was unemployed, there’s no like, and he left uhhh behind the clinic, so there was no way that he’s gonna complain that “no, I don’t have transport money or,” you see, so with him, we would give him like a week, a week uhhh no two weeks actually, two weeks medication then he must come back in two weeks again. Or maybe somebody like one of the WBOT (ward-based primary health care team) would take them because we saw that we can monitor him only if he takes like for two weeks, two weeks because we had other patients whereby you give them two months because they were adhering very well, but with him we couldn’t do that, *yah* [yes] on both sides on HIV and TB also. *Yah* [yes] we had, we, we, we also got assistance from the WBOT and xxxx (organization name) also *yah* [yes] because they did home visit also, *yah* [yes].

I: Okay, and with this issue uhhh of patients who open the box uhhh without taking medication uhhh what do you think can be the solution or how can this DAT be improved in order to make sure that when a patient has opened the box, I mean we are sure that okay the patient has opened the box and took medication and not just open it without taking medication?

P: Uhm I don’t think there’s much to be improved because with the pillbox it does anything for them, it an alarm, reminder, there’s a reminder also to come to the clinic. And also, when the pillbox is battery low uhhh with that issue I feel like it based on a patient, hence I say with that previous question, probably it’s in denial of your status and what or whatever that you gonna take, but also with other patients because I had this young girl uhhh this girl she was interested but she didn’t have a phone. She didn’t have a phone, she was very interested, she’s in her early twenties uhhh but the girl she was okay for a while until uhhh she had to maybe go to school and what not, so the pillbox had this alarm and it was very noisy for her or maybe it would go before her wake up time, *yah* [yes]. So, she started like now just living it open, *yah* [yes] not closing it for her to remind her then she will just take her pills just there, *yah* [yes] and she actually she did confess that this is what I’m doing because I feel like this thing is waking me up before uhhh before my bath time to go to school. *Yah* [yes] so with that issue, we did note it down and we also thought maybe if they reduce the alarm, *yah* [yes] so the alarm now was reduced and she was okay with it, *yah* [yes].

I: Oh, okay uhhh you kept on mentioning the platform [laughing] that you were using together with the DAT uhhh I just want to know what was the name of this uhhh platform you were using?

P: It was xxxx (adherence platform name)

I: Okay, so you would use this platform to check on patients?

P: Yes

I: If they did take medication or not?

P: Yes

I: Okay, and then so when you log onto this uhhh platform uhhh, then how did you see that okay this patient did or did not take medication-

P: Okay-

I: On this day

P: On this day. Okay, with the platform when you logon you put in your clinic name and the password, you enter xxx [facility name] and with xxx [facility name] clinic it’s patients that you initiated on the platform because with the, with the pillbox there was a code actually, the IME- the code at the, at the back of the battery whereby you need to enter it then it activates uhhh for that patient. And then with the sticker label, you just initiate them there’s no code but uhhh there’s a name and surname of that patient and also with the pillbox. So, with the platform, in order for you to see that the patient did not take, it becomes red, yes it becomes red and then if the patient did open the pillbox, it’s green, *yah* [yes].

I: Okay uhhh are you familiar uhhh with task list?

P: Yes.

I: Okay, and how does it work uhhh on the platform?

P: Okay, with task list, we used it also to call patients and then give feedback also, *yah* [yes] and uhhh give feedback and also if maybe we had an issue of patient not adhering in opening the box or sending SMS, we would go to the task list and note it that, so that they should not call, they should not bother the patient every day, *yah* [yes] we would note it down that okay “I did call the patient, he or she did respond or he or she did not respond”. And also, uhhh conveying household, house visit actually, a house visit for patients who did not uhhh respond because with the, with the xxx (adherence platform) platform, two days you call a patient if the platform is red, two days you call a patient, four days you do a home visit.

*Yah* [yes] two days you call or send an SMS either way he’s going to send or he’s going to get the SMS. The third day you see maybe it’s not opened, you try to call them maybe they might respond, if they don’t respond on the fourth day you do a home visit for that particular patient then you must find or get an understanding. Also, on the task list you must note it down that you went or you have done a home visit for this patient and this is the response that you got.

I: Okay uhhh were you involved in initiating the home visit or there were people that uhhh people that you were uhhh a group of people that you were working with?

P: Yes, I had a group of people, and the company is called xxxx (organization name) something like that, *yah* [yes] uhhh I would give them a list of patients that I need them to go do home visit and they would give me a response because they write it down also, I would write down what they wrote down. Because at my facility you couldn’t find WBOT , they would tell us they are at xxx (area name), I don’t know why xxx [facility name] WBOT are at xxxx (area name), so xxx (organization name) was there also to assist me even xxxx (organization name), I was working with this Uhhh guy who would do a home visit. So, I would also gave him a list maybe if xxxx (organization name) didn’t come, I would give the guy from xxxxx (organization name) a list because I explained to them how, how the ASCENT uhhh thing works. So, they told me “if you need help tell us, we just want to do this for you also” *yah* [yes].

I: Okay, and were they always available to do home visit or in some days you would find that they are not available or maybe they are short staffed?

P: *Yah* [yes] *yah* [yes] actually with Uhhh with xxxx (organization name) they were very, very short staffed, probably they went to different facilities because xxx (area name) has so many clinics and then with xxx (organization name) you would find that uhhh one took a car to different clinic also. So, you need to wait maybe for a day or two, *yah* [yes] for a home visit to be done for that particular patient.

I: Okay, and were you involved in counselling uhhh patients? I believe that you mentioned a patient who was struggling uhhh with treatment, I believe that one of the steps that are taken is to counsel the patient, *yah* [yes]. So, I want to understand if were you also involved in counselling patients?

P: Uhm yes, I was actually, and there were patients that did understand because we had this uhhh a very young woman who told us that she is afraid of telling her family her status, *yah* [yes] her TB status.

I: Mmm.

P: So, we told her that you can give uhhh, you can give family time, or you can give yourself time just to accept first. You need to accept yourself first then you can tell your family about your status. So, *yah* [yes] I was more involved also. And then, because you would find people whenever you tell them your status, your TB, and results, they panic, they think that TB is gonna kill them, and it’s not like that, it’s really not like that, only if you take your treatment correctly.

I: Okay uhhh you already spoke about the phone calls which you have made when you see that a patient is not uhhh taking their medication for two days, you said-

P: Mmm.

I: Or you sent an SMS and uhhh you also mentioned that uhhh you uhhh you would talk to the WBOT or the guys who were responsible for home visit and tell them that I have this patient so and so who must be visited and all that Uhhh. So, what I want to understand is uhhh which one of these action, support action did you do the most, uhhh were you always have to call on patients more or there were more patients who needed home visit?

P: Okay, with my, with my uhhh with my patients uhhh only a few who needed uhhh who needed the calling of, like reminding them every day, even though they had a reminder, they needed a reminder, you know “open your box” and not just a two-day reminder, like everyday reminder. But because we were not allowed to do that, I mean they had a pillbox to remind them to open the box, y*ah* [yes].

I:Mmm.

P: So, some of them would say, “oh no I closed it” when we give them these pill boxes we told them do not put it next to or close to the sun or close to kids because a kid would always play with it, opening, closing it, because it also showed on the platform at what time did you open this, on the 24th of January you opened this at 8 o’clock then next thing on the 24th of January you opened it at 10 o’clock so you see that okay probably there’s a kid playing with it or you are doing something wrong with the pillbox. So, you would tell them that “just put it very far from kids and also the sun”. Okay fine, probably it because the alarm was also reduced and they couldn’t maybe hear it from where ever they placed the pillbox but they know that at a certain time they need to take the medication but some of them would forget, *yah* [yes] maybe open it 30 minutes after the alarm, *yah* [yes].

I: Okay uhhh you are mentioning a quite interesting uhhh thing here where a patient- you would see- I meant you’ll see that uhhh on the platform that box maybe has been opened many times in a day-

P: Yes

I: Meaning it could be a case of maybe a kid playing with the box or the patient opening the box multiple times. So, I mean, how often did you see or had those cases of patients who- where you see that okay this patient is opening this box maybe five times a day or twice a day because I believe they just have to open it once?

P: Yes

I: So, *yah* [yes]

P: Okay, I had that, not much often but it was only on elderly people, I thought to myself probably they were putting uhhh not only the TB medication but more pills, probably the high bloods, the sugar diabetes pills so it also would clock okay “eight o’clock it’s the TB medication” because you need to drink your TB medication in the morning, maybe 08 o’clock it TB medication then around 12 you see the box is opened, *kanjani* [how come] probably that person entered uhhh inside ama more treatment that she was taking.

I: Mmm.

P: *Yah* [yes] then you would also tell that person when they come for follow up that “this box is only for TB medication, you do not put your high bloods and your sugar diabetes and *ntoni* *ntoni* [and whatsoever] just put your TB medication because it shows, it shows when you open it.” You cannot open uhhh you cannot open a pillbox and we know that you are taking your TB medication once a day, next thing you open it every day-

I: Mmm.

P: So during a day, you open it like three times, that means you’re putting something, then they will “say I thought to myself that uhhh this thing can also remind me” but with that also, you tell them that a reminder reminds you once because we do not do reminders, *yah* [yes]. So, do not uhhh put your other medication on there, just put your TB medication and maybe whenever uhhh you are done because they will say maybe some of them just hang the pillbox, probably they don’t know where to put the medication, not that it not gonna remind them it just a safety box for them with other treatment. But only if they completed the TB medication.

I: Mmm okay, uhhh so speaking of patients who were using the box for other medication uhm when they were expected to use it only for TB medication. So, now my question is do you think it would be flexible for uhhh patients to use this box for other treatment as well or other chronic uhh for- other chronic medication?

P: Yes, it would really assist so many patients.

I: Mmm.

P: Because you would also get uhhh a patient that is taking TB medication but because he or she is concentrating only on TB uhhh they forget also about the other medication, then you find the high bloods high, *yah* [yes] then they need to go see a nurse, a different nurse that side-

I: Mmm.

P: So, I think it would really, really, really assist them if they also do it for other types of uhhh medicines for patients because you can get maybe a patient who has uhhh maybe high blood or your sugar diabetes and needs that like a reminder. It would work perfectly for people with sugar diabetes

I: Mmm.

P: It would

I: Mmm, why, why, why you say so?

P: Because sugar diabetes people they, they do things at like time state I feel like the pill box can also, yoh it can also help them so much because with uhhh with the sugar diabetes people they need, they taking the injection at a different time maybe some of them they forgot, they take it very late, *yah* [yes] so it would actually help them actually, it would really assist them.

I: Okay, thank you for that uhhh and how were the responsibilities uhhh shared between you and other Health Care Workers, I mean your nurse, your TB nurse and everyone else who was working in the TB room?

P: Okay

I: Mmm.

P: Uhm with the nurse, the nurse, we know that she initiates *neh* [right?] she initiates and then there will be like these other girls who were working for EPWP they would assist, they would assist in maybe packing the pillboxes sometimes. Because the nurse would also initiate on the platform because we teach them, that you know what “you need to know how to insert a patient, how to, you need to know, you need to know” but the nurse taught this other lady so that maybe if she is busy with something, she can start, because we do not wanna keep patients for very long time inside the room, I mean there’s gonna be a que, there’s a que outside actually. So, the responsibilities were shared whereby we must share everything actually with us besides the initiation of patients, we check *ama* [the] results, she would also check results and uhhh we would test because we have this thing called a TB Day, we would go outside and we would like test whoever, whoever had symptoms but whoever also has a patient at home who has TB, *yah* [yes] because sometimes the symptoms would show later. *Yah* [yes] we would also do that, like we would share different responsibilities but we all knew what we were supposed to do, *yah* [yes].

I: Okay, so the TB nurse was also able to use the platform?

P: Yes.

I: See and check patients-

P: Initiating patients, yes.

I: *Yah* [yes] initiating patients.

P: *Yah* [yes] because for us to have a good uhhh good communication work uhhh we would show the nurse that “look at this patient, he or she is not adhering very well” then the patient, the nurse would also tell us okay when this patient comes for follow up, the nurse also was ask questions *ukuthi* [that] why aren’t you taking your medication properly and also we would see again, I told you with the weight gain or weight, because you would find a patient uhhh claiming that he or she is taking medication but his weight is dropping from 60 to 40 something, that means he or she is not taking the medication correctly, maybe it those type of people who just open it and leave the box then uhhh the nurse actually came with this idea whereby when you take your medication leave the empty containers inside the pillbox so that when you come back to the facility. We must count them if you did take your medication. *Yah* [yes] so that actually worked actually, it worked.

I: Okay uhhh, okay and then uhhh so when you first heard about uhhh this DAT intervention- the box and the labels since you were implementing both-

P: Mmm.

I: Uhm what were your expectations before you were involved in implementing and enrolling patients but let me say when you were trained, you know during the training what was your-

P: Mmm yoh *nna* [me] my expectations *wena* [you] yoh this was gonna be very difficult because uhh with me, I think it also depends on the person, *nna* [me] I’m a very shy person I need to get to know a person when I thought *ukuthi* [that] how am I gonna do this, how am I gonna approach a patient, what if there’s like tell me no, no I’m not interested, before you saying something. So, I thought that it gonna be difficult until they said “no, you are allowed to go work at TB room also” that’s how you gonna get your patients, *yah* [yes] that’s you gonna get your patients and uhhh when the nurse is done talking to the patient, you can approach them, you talk about pillboxes, you talk about labels, *yah* [yes].” So, at first, I felt like yoh I’m not going to get any patients.

I: Mmm.

P: I’m really not going to get anybody but uhhh it was very, very, yoh it was heart-warming working with those type of people, *yah* [yes].

I: So, your opinion changed after you-

P: After I went to the field actually, my opinion changed after I went to the field. During the training I was like yoh how am I going to do this but as soon as I entered at TB room, things changed, also the nurse was very welcoming and the other uhhh other girls they were working with her they are very welcoming and I thought *ukuthi* [that] no I’m gonna made it, *yah* [yes].

I: Okay uhhh and did you go other trainings before you start implementing?

P: Other trainings, yes, yes actually we, *yah* [yes] we did training, *yah* [yes] I think we did actually also, *yah* [yes] I think, *yah* [yes] we did actually at the Anew hotel I think there were, were ladies who already were doing this so they were showing us how it’s done and how we should approach patients also and *yah* [yes] we did go to training actually.

I: Okay, and do you still uhhh perhaps remember what are some of the things that you were trained on, the activities that were done the duration of the training?

P: Okay *yah* [Yes] with the training it was quite a lot actually, there was a training of counselling like how you should be sitting with a patient, there was a training on how to approach a patient using the label, there was a training on how to approach a patient using the pill box, there was a training on how to not read the form word for word because the patient is going to be very impatient. *Yah* [yes] so the training, and also you should know your form actually you should know and you should maybe summarize everything or the important facts and then tell a patient, because some patients are very impatient, *yah* [yes]. I mean that person is done with the nurse next thing you want to pull them aside and talk about this and is kind of you know, time taking for that person so *yah* [yes] actually there was a training on a lot of things. There was a training also on how to read the form, on how to sign, how to make the patient sign and how you should sign also and also a training on if a patient comes with a weakness there was quite a lot of trainings actually.

I: Uhm speaking of initiating or enrolling patients, *neh* [right?] uhhh did you uhm have patients who refused uhhh the DAT, either the box or the labels?

P: Yes [laughs]

I: Mmm.

P: Yes, yoh yes [laughing] uhh I had a patient he expected the stickers ones because he said the pillbox is so loud. *Yah* [Yes] so fine I told him there is another one whereby you take your medication and you send the SMS, it is a toll-free SMS and you have a code and for each packet of pill there is a code, for each packet of pill there is a code, okay the patient was interested. But as time goes by, a patient came back to the facility “no, I wanna be removed from this, I wanna be removed from this because I do send SMSs but it also tells me that I should send SMSs”, so I would like tell the patient okay if you feel like you want be removed, it’s fine you can leave it but also you would find *ukuthi* [that] they are busy initiating a new patient, you are there also, you want to enrol this patient and he/she are not interested, I would get rejected actually.

I: Mmm.

P: There is quite a lot of rejection actually, they like “no I don’t need this, I don’t need anybody to remind me to take my medication, I know that I need to take my medication” so *yah* [yes] there was quite a lot of rejection. And then people who also like already enrolled would come back and change their mind they like “no, please remove me from this” *Yah* [yes] with that with patients who got enrolled and then changing they mind I felt like it’s something of a stigma that people are going to know that you’re taking medication because there’s this thing is making noise and probably we have visitors and they gonna ask “what is that”? some of them were not ashamed because it also helped them to help others and then would be like “I wish there was more of this type of pill boxes for different illnesses because we only taking it for TB medication” *yah* [yes].

I: Uhh speaking of patients who opted out uhhh as you say that you suspected that maybe it was the issue of stigma. Uhm did you perhaps have one patient or more than one who actually told you that or even actually they didn’t say it exactly like there’s stigma or whatever, but they gave you an idea that the reason they are returning this thing because they don’t want other people to see them using and then find out actually what this thing is for?

P: *Yah* [yes] I had quite a few but with one of them uhhh he had only two months to complete TB treatment then after that he was not appearing because we had to do the last sputum to see if the patient TB has been cured, so *yah* [yes]. I thought because he was a very young boy also, so there was one actually but this one he was SMSing, so he was like “no, Aunty I’m tired I’m always SMSing, at work they busy asking me why am I SMSing and what am I SMSing for?” So, I thought to myself that probably at work they don’t know that he is taking TB treatment then I was like it’s fine I’ll remove you from the platform also, *yah* [yes].

I: So, this was a younger person?

P: *Yah* [yes] there was, it was a younger person and an older guy like in your early 30s or 40s

I: Okay, and now going back to the training that you received *neh* [right?] uhhh after two days or a day of being trained uhhh what was your first impression of the training session? How did you find it?

P: Uhh the training for me, they made it seem so easy because uhhh they had to also do a training whereby you get rejected when you approach a person, and that person rejects you *yah* [yes]. So, the training seemed so easy, but the field was more difficult but also enjoyful and it was quite easy for some patients because they were very glad that we are here and we are here for them because we also received uhhh a complaint from a patient while they were doing home visits, a patient was, that was after we closed for December when we came back and the patient was busy crying saying that I got hurt by a nurse and the nurse told the patient that you know what this is your health I do not care if you die you die. So, the patient actually *ungwalile* [stopped taking treatment]. So, when I come back, I see the patient the patient and this person was adhering very well, he’s a very old man, he was adhering very well. So, when I see on the platform, I’m like what happened to this one. So, I tried to call him, I tried to call him until he comes for his follow up visit but this was after I think a month and he did not even pitch up okay…then he was like eish I need to talk to you like specifically not you and the nurse but I want to talk to you alone. So, we found a room we talked and then he was very emotional and was like I really do not know why this happened and... but I would talk to a nurse because we had a very good relationship with her *yah* [yes]. I would talk to a nurse and just find out *ukuthi* [that] why is this happening or why did this happen and when you talk to the nurse and the nurse would tell you that “no I didn’t say directly but I told the patient that he must take care of his health and what not and what not” and it was like…it was stories of he said she said but at the end of the day they did apologize to each other before it get further, going the head nurses and yeah they did it like it ended in the TB room they did apologize to each other and then we took the sputum because he was supposed to also uhhh finish treatment yeah so *yah* [yes]. I think the guy got cured eventually uhhh it was a very successful case actually we had so many successful cases-

I: Mmm.

P: *Yah* [yes] we had so many successful cases on the labels and the pill boxes, but we also had a downfall like uhhh from your DS-TB to DR-TB to your TB- mode *yah* [yes] we had a case like that actually. And from there we were like okay we cannot give you the pillbox anymore we need to take it back because with the TB mode the pills are quite a lot they come from different hospitals *yah* [yes] they need to go check-up that specific hospital I think its xxxx (hospital name) it’s more of yoh [shocked] from a small TB up…so it was the very same patient who the mother gave us her phone number and the brother because this guy we really don’t know what kind of drugs he was taking but it was killing him because he started from the DS-TB then it went from the DR-TB with extended going to the TB- mode and it was very, very, very at this…what is it? It was more of like the nurse felt like she failed the patient because this guy he’s not healing instead the TB is growing actually and we thought *ukuthi* [that] the pill box was going to assist him actually but it didn’t actually so *yah* [yes]…we also had a guy who from DS-TB to a TB spine also, hence I’m telling you that we had good success and we also had very bad bad, bad, bad success yeah… and the bad success we took them very serious, we were like where did we go wrong with this patient? What or how did we treat this patient, is it our fault? What was happening basically with this patient? Yeah

I: Okay… so these patients that you are talking about uhhh were they all on DAT that time? When they went from DS to XDR?

P: Yeah they were, because their TB was just a normal TB you know it was DS-TB, it was fine but you know with TB whenever you approach two months of completing your treatment we need to take your last sputum to check if the TB has been cured. You will find that “no it has been positive, there’s three pluses, no how did this happen, we didn’t see it” because the patient is taking treatment very well, you see that he is picking up. And like what happened like how did we miss this? *Yah* [yes] then yoh it will go from that to worse.

I: Mmm.

P: *Yah* [yes] patients need to be uhhh patients -need to go to xxxx (hospital name) every Tuesday, and it’s a different story, *yah* [yes] it’s a different story. Then we had remove them on the DAT, on the platform because I add, yet I didn’t have the very big pillboxes and in my facility I had so many patients who needed the pillboxes for those Uhhh TB that, for TB patients that go to xxx (hospital name). I think I did uhhh tried to talk to my manager about it and she was like no, because many patients that come to xxx [facility name] clinic are patients that are going to xxxx (hospital name) on Tuesdays. I think we had ten patients that were going to xxxx (hospital name) at one facility. *Yah* [yes] it was not the normal TB anymore, it was more because with these patients we get them at home it’s that patients, it’s the daughter, it’s the father, it’s the mother then this patient infected all three. *Yah* [yes] all three maybe on all three, one can be DS -TB, two can be like TB they all need to go to Hospital, *yah* [yes].

I: Okay, because these patients were taking more pills?

P: Yes.

I: Hence you suggested that they get a bigger box.

P: *Yah* [yes] because they did tell us about a bigger box for the TB mode and the TB, so because the pillbox is very small, it’s really small and those people they do not take only Rifafour and Refina, *yah* [yes] they take more, they do not take those. *Yah* [yes] because I think with the normal TB it has Rifafour, Refina and Pyridoxine, *yah* [yes]. *Yah* [yes] I think it’s that only, but with that, yoh it just a lot of pills, it’s a lot of pills because it also comes in a pocket.

I: Mmm.

P: *Yah* [yes]

I: So, there’s issue with the box size?

P: Yes

I: For some patients?

P: For some patients.

I: Mmm.

P: *Yah* [yes]

I: Okay, and now going back to that patient uhhh patients rather where the uhhh an argument with the nurse, uhhh which led him (patient) uhhh discontinue his treatment I believe. Uhhh so what I want to understand is uhhh did he continue to use uhhh DAT since he was on one of the DATs?

P: No, actually.

I: He-

P: He stopped

I: With the DAT?

P: No, no, no he didn’t bring it back, he stopped opening the box, he stopped his treatment completely because this was during December, I think we closed uhhh. So, it was his follow up date. So, I think it happened that day then the patient had to come back uhhh January so that they can do the last sputum uhhh, I think the patient was supposed to finish his treatment on March. *Yah* [yes] because the January one was the patient coming to do the last sputum, *yah* [yes]. So, that patient didn’t even take his medication because on January he explained to me what happened, and he even showed me that he didn’t even open the box. And I saw on the platform he was not opening the box the whole of December even on January until he came to the facility. So, I was like okay, like he was angry to a point whereby because of the words that they told him, he wanted those words to happen because apparently, they told him that “you going die because you don’t take care of your health”. So, he didn’t even take this all his medication, the medication was just full inside the pillbox, we explained to him, talked to him, and then apologies were made on both nurse and the patient. And *yah* [yes] we did the sputum then we told him that “can you please take, continue taking your medication” then after that, he did start opening the box. He didn’t bring back the box-

I: Mmm.

P: He didn’t want to remove it until his treatment date was finished, *yah* [yes] he didn’t want us to take the box, he was just angry at the nurse and then he was like “now I’m going to take my medication, I’m not gonna even open my pillbox”.

I: Okay uhh do you know if he was cured though at the end?

P: Yes

I: He was?

P: Yes

I: Okay, that’s great, that’s great. Uhhh with the training, do you think it was enough uhhh the way you were trained, the information you were provided with, do you think it was sufficient uhhh for you guys to go out there and do what was suspected of you?

P: Yes, yes, the training was properly done, though they should have showed us that there are patients that going reject us when we approach them. But the training properly done, like after the training it was like “okay I’m ready to go on the field now because I know *ukuthi* *ebile* with the training [I know that actually with the training] because it was done before us going to the field so we must start afresh, you know. It was like the training was happening yesterday so we remembered everything quickly. I remember this one, this group did this, so you go, and you do this, you do the patients how the training was done. So, *yah* [yes] I think the training was successfully done and they showed us everything actually that you needed to know.

I: Okay, any suggestions on how, maybe, or where to improve next time, they do these kinds of trainings that they should improve?

P: Uhh *yah* [yes] the rejection.

I: *Yah* [yes], yes [Laughing].

P: [Laughing] because mina *nne* [I, right?] at my first day, I got rejected, I think yoh five or six patients and these were new patients, like newly initiated patients and because I was doing the stickers, I thought *ukuthi* [that] okay because uhhh maybe it the sticker thing.

I: Mmm.

P: It’s difficult for them or uhhh people get irritated by always SMSing because you took your medication, *yah* [yes] and then I was like “no but this also helps you and it also helps the nurse to show that yes you are taking your TB medication” and then the patient would say “no I’m not interested” actually one patient knew about us and then she was like “my friend has a pillbox” and then she was like “the day you will give me a pillbox then I will join you, like I will let you to enrol me.”

I: Mmm.

P: “Because with the SMS, I’m not gonna always SMS that I took my medication” so she was like “no, my friend has a pillbox, I want a pillbox” so that’s when we also introduced a pillbox Uhh at the facility because my facility was the label, stickers, was the label facility but it also implemented the box. *yah* [yes] with the stickers I would get uhhh our youth would be excited because with the pillbox it’s loud and everyone is gonna see that he or she is taking medication. So, with the stickers it’s fine, you drink your medication there in the room, you send an SMS, nobody is gonna see, so, *yah* [yes].

I: So, if I understand you well, you were saying that uhhh within the older generation, right?

P: Mmm.

I: Uhm you had patients who were challenged, technologically challenged I mean uhhh they could not operate the phones, send an SMS and all that, they needed someone to assist them.

P: Mmm (yes).

I: Mmm and you would switch them-

P: Mmm (yes).

I: And the younger ones they would prefer the SMS?

P: Yes.

I: Because they are afraid that maybe people might now start seeing them with the box-

P: Mmm.

I: And ask questions, right? So, and again you had uhhh younger people who were also not sending the SMS?

P: Mmm (yes).

I: Mmm.

P: Yes, so with that it was probably an issue of patients who come back and tell you when you ask them “why aren’t you sending an SMS” when you call, they would tell you that “my phone got stolen or my phone is off, like my phone does not want to switch on anymore. Uhhh I do not mind to have the pillbox as you said the alarm now is lower, it’s normal. It is fine I will come to the facility and then you can switch me to give me the pillbox” *yah* [yes] and then with the elderly people, they literally didn’t want the SMS, *yah* [yes] they didn’t want to be on the phones, *yah* [yes]. “This thing is a bit tiring for us because you would take your medication then you need to SMS, no.”

I: Mmm.

P: *Yah* [yes] but with the youth, you would get also young people maybe you enrol them and then a person tells you “I don’t have a phone at all but I wanna be on the study.”

I: Mmm.

P: Then you introduce a pillbox then they will be like “this thing is for older people, one of my grandma’s friend has this, it’s for older people-

I: Mmm.

P: Don’t you guys have another thing besides SMS and a pillbox?” then I will be like “no, this is the only two things that we have” then they will also like, they would say “okay no, it’s fine I live with my aunt uhhh I can SMS with my aunt’s phone” because youth found the pillbox for older people, I don’t know why.

I: Mmm.

P: But they found that this is for older people who don’t want to use this, we would rather do the SMS.

I: Okay, there was an issue of an alarm with the box-

P: Yes

I: Especially to young uhhh generation.

P: Mmm.

I: Uhh with the SMS uhhh you had older people who refused the SMS because they thought maybe they would end up experiencing a fatigue of SMSing each time they taking their medication, okay?

P: Mmm.

I: Okay, and also, there was an issue of lack of cell phones-

P: Mmm (yes).

I: Did you have people uhhh- if I may ask, did you have people who were interested in the labels but now the issue was lack of cell phones?

P: Yes

I: Mmm.

P: I have an example with that guy, he was interested in being on the ASCENT then but also, he was like “I do not have a phone but I don’t want a pillbox” so I’m going, because I live with my aunt, I’m going to ask my aunt to uhhh borrow me her phone as soon as I’m done with taking my medication then I can SMS with my aunt’s phone.” *yah* [yes] the pillbox for youth it was an issue of like “no, people are going to know.”

I: Mmm.

P: “This thing is a bit loud.” I don’t know why they thought it’s loud.

I: Mmm.

P: “Because as soon as you open the, the, the, the beep sound it just,” like it’s only you that you can hear it. It’s not like it’s going , the neighbours will hear that, no. But they didn’t, some of them didn’t mind it-

I: Mmm.

P: Because it’s some of them who had no phones but it was also some who do have phones and they feel like doing an alarm on the phone is quite, like there’s so many alarms on the phone, so they would take the pillbox because it had an alarm and you wouldn’t miss it, you’ll need to know that at a certain time you just open your box, you take your medication, you close it and then you put it very far, same day tomorrow. So, some youth didn’t mind it.

I: Mmm.

P: And some did mind it. Yes, we did have an issue whereby patient is interested to be on the study, but challenge was he or she did not have a phone, but it was a family member actually who had phone at home. But also, another challenge with us was when the patient did not SMS, maybe the aunt was out of the city and then patient is taking he or she uhhh medication, but she cannot send an SMS because the aunt is far and then you call because the phone number is the aunt’s number. *Yah* [yes] when you call the aunt will be kind rude *ukuthi* [that] “hey this person I’m sure he or she is taking medication why you busy calling me. *Yah* [yes] to do home visit and check if she or he is taking medication” because this person is far from a person who is taking medication. *Yah* [yes] so it was kind of a challenge there and then with that we would also try to offer that patient *ukuthi* [that] “no, just try a pillbox for a month then if this is irritating you or you cannot copy with it, just bring it back so I will remove you from the platform because we did that maybe your aunt is at work and we are busy calling, she cannot take the phone or she gets SMSs she gets irritated because the phone belongs to her it’s not yours, you asked to borrow the phone at your aunt.” So, *yah* [yes] some of them would say “okay no it’s fine, I understand let me just take the pillbox because I do want to be on the study” *yah* [yes].

I: Okay, so you had patients who were sharing the phones with their family members?

P: Yes.

I: Okay uhhh did it work having a patient who is using DAT, but they do not own their cell phones, you just highlighted the issue of maybe the aunt at work or something?

P: Uhhh no we would get maybe some of them are unemployed so they always at home, so it’s fine for them, you know, they say, “aunt I’m taking my pills please send an SMS” and then an aunt does that or maybe a mother does that, *yah* [yes]. So, some did work, some were like “yoh no guys, stop calling me.”

I: Mmm.

P: “So, please stop calling me because I’m not the one who is taking the medication, please go to this person blah blah blah blah, *yah* [yes]. So, *yah* [yes] we would, we would get successful results but also, we would get like “hey guys you are really irritating because you send SMS, you call, you send SMS, you call and I’m really fed up, why didn’t this person tell you that I’m leaving the city, I’m no more in Pretoria or I’m at work I’m working-

I: Mmm.

P: And you guys are busy calling me” so tell aunt when is the best time. And we also got patients who telling you that you can call around six but we knock off at four, just for patients to tell us uhhh what was the reason of them not opening the pillbox, so patients would tell us that maybe like one of the aunt or mother who that uhhh person is okay with, a patient using hhhhh her phone hhhhh maybe she would, she would respond at work then she would say “I’m knocking off at four or at six, call me around six then you can uhhh ask questions to that particular patient.” *Yah* [yes] so we would call at six to check if or just to understand why didn’t a patient send an SMS then we would find that okay “my mom was out of time for that weekend, mostly it happened during weekends. Monday to Friday it was okay.

I: Mmm.

P: During weekends it was… Saturday and Sunday it’s red. Monday it’s fine, Tuesday it’s fine. From Saturday and Sunday uh then we would feel *ukuthi* [that] okay maybe the mother travels, the mother goes somewhere on weekends, and we cannot be sending an SMS, so with those type of patients we would offer them uhhh a pillbox and they would agree because *ukuthi* [that] okay no it says like with red the nurse would like shout at them, so they don’t want to be shouted at. So, they would be like “no, nurse I do take my medication but also I do understand, I can, you guys can switch me from the SMS to the pillbox it’s fine so that you can see I do take my medication properly” because we would get patients who are trying to impress the nurse *ukuthi* [that] no I’m a very good patient, I do take my medication, I do want to heal, I want to be cured” *yah* [yes] “I don’t want to come back here.

I: Mmm.

P: *Yah* [yes].

I: Okay uhhh you mentioned a patient who was on substance-

P: Mmm.

I: Who was using drugs ,I mean hence his adherence was not so good, uhhh so I want to know if you had other patients uhhh in the facility who were struggling because they were on drugs.

P: Yes, actually we found out later though, when a patient was, I think a patient was like three months, *yah* [yes] only three months we found out that okay this patient actually is using drugs. Actually, the cousin came, the cousin came uhhh the reason why the cousin came it’s because this patient for a month didn’t open the box, *neh* [right?] and then, but before we know we do home visit and what not and then every time we went to his place it would be like empty, nobody would respond every time they go the (organization name) people would come back with the feedback and say “no, the patient is not home, even the neighbours would tell the (organization name) people that this person I think it’s been two weeks that he’s not home.” So, this other time, the cousin came and he told us that this person is in hospital, he’s hospitalised due to overdose of drugs uhhh then we would be like okay no wonder why he was not opening the box and this patient was supposed to be, like was supposed to complete treatment but because he was taking drugs, the TB grew from DS-TB to DR-TB. *Yah* [yes] I think we had only three patients that were taking drugs but the one actually passed away uhhh the one passed away uhhh these two uhhh one of them it was the one with the TB spine, *yah* [yes].

The drugs caused very, very bad things on his body. Then the other one was the other guy who was taking uhhh his TB medication at the xxxx (hospital name). So, the one with the TB spine uhhh the nurse was like “no, this patient he’s not going to learn” because I think it was his third time taking TB treatment, you know you get those type of patients, they, they, it’s not uhhh TB cured but it’s TB completed treatment, *yah* [yes]. He was one of those type of patients, basically TB would always come back to him, so it was like his third time taking TB medication but this time it created TB spine. The nurse was like “no, but just remove this person because next year he’s going to come again, the other year he’s going to come, other year he’s going to come” and with the TB spine. I think it can make you take your treatment for one year I’m not sure how many months, but it was a lot of years due to that uhhh with the platform, the platform also would remind you that this person was supposed to be cured and we still have this person is taking medication then you need to explain on the platform that this is extra pulmonary TB or something TB or, *yah* [yes].

I: Okay, thank you uhhh, did you have any other group of people uhhh in your facility beside the ones on drugs uhhh for example homeless people, did you have homeless people who were using uhhh one of the DATs?

P: Yes

I: Mmm.

P: Yes, because this guy even sold the pillbox actually.

I: [Laughing] Tell me more about it.

P: [Laughing] Yes, we had this other guy uhhh he did write an address but that not where he lives, probably it was like he was just passing by and he saw, he was like “okay, I’m going to tell them that I’m using this address” because what happened was with him *neh* [right?]. He came to test and then the result came out maybe Friday, let’s say he came on Tuesday and the results came out on Friday. So, Friday the nurse uhhh mostly Fridays TB room they do not operate but they help with other clinical things inside the clinic, maybe the nurse would do chronic but first we do our stats because stats we complete everything. Friday, we do not book patients, we do our stats, mostly Friday we test actually, that’s when we get our customers actually. Fridays we would test, do a lot of testing *yah* [yes] but until a certain time and then the nurse would maybe go help with other, with other services in the clinic, *yah* [yes]. And then *yah* [yes] Fridays mostly we would go home around twelve, so this guy we called him Friday, oh also on Friday we would call patients and give them the results.

We called this guy uhhh because he would, I don’t know whose phone numbers were those, I think it was maybe his friend’s phone numbers, so we called we tell this person that “can you please tell this certain person to come Monday at the facility around seven o’clock”, the person agreed then they did tell the patient but the patient came on Wednesday instead uhhh, yes we showed him the results and we told him that “you going to take your TB medication”, he was okay with everything then we told him that uhhh we going to enrol him on this pillbox, he was very excited because he told us that “you know what, I don’t have a phone, I don’t know, I don’t, I don’t know who’s going to give me time to take my medication and” so I told him “don’t worry I have this thing called the pillbox, it actually reminds you, so what time you wanna take your medication?” he said six o’clock because he has piece jobs, but we didn’t know that this person, there’s no piece jobs, there’s nothing, he was very homeless.

So, he even came with the friend that day, so the friend was like, like he was stirring at him maybe he didn’t want to tell us the truth but we wanted the patient to be open with us. So fine, it’s fine, they left, a patient for a month took treatment very well then second month, like when we approach a second month but around like two weeks, now it started being red, okay and then we were trying to call this number of a friend, a friend is not responding but it does ring, the phone, the person is not responding. Next thing you trying to call, the phone is off, is always off, *yah* [yes]. So, uhhh this other time, this patient, this particular TB patient he was also needed at the HIV room so that how we found out *ukuthi* [that] this person is homeless, this ID number, this address sorry, this address is not where he stays because we did the home visit and they really don’t know that patient, they never see him and like they said it the second time this happens, *yah* [yes]. So, they told us *ukuthi* [that] probably he’s homeless, actually he was homeless and then uhhh he came to the facility because with TB patients actually with also HIV patients you need to take your TB medication for a month and after they see your weight that it picked up then they can give you for two months. But we need to monitor you maybe for a month or three months, he would take it, like he would take a month, a month, a month then after that we give you two months. So, this guy he came, like I was thinking *ukuthi* [that] what is he drinking because he needs to, he needs to come back to the facility to get medication, okay uhhh one of the people who was working at the clinic told us that “guys I saw your patients, your patients” because xxx [facility name] has, like next to xxx [facility name] there’s this scrap yard, they told us that he’s there, and we were like no, we cannot go there, like everybody cannot be there, I’m sure he’s going to run away. But also, it’s not allowed for you to go there. So, uhhh I think one of the people told him that he is needed in the clinic. He came to the clinic and that’s when he told us that “*nna* [me] I’m homeless and the pillbox I sold it, I’m sorry my sister” I was like yoh you sold it, why, and for how much? And then he told us that he sold it with pills inside? And I’m like, okay where did you sell it, did you sell it to patient who takes TB medication or what he's like with the pills? Then I’m like yoh this guy.

I: Mmm.

P: Yes, we did have a homeless person and he would sell the pillbox, also I was afraid to give him the second one because it gonna happen again, so I would call my manager and then she would tell me that “okay, it’s a bit risk with him, at least maybe if he told you like it’s lost then you would note it, but he sold it, you have to write that, the patient sold the box and you have to write “the patient sold the pillbox with the pills inside” probably that guy was, or maybe the person that he sold he was busy opening it and it was like he’s taking medication. Because that’s when we found it, we found it later, he said the pillbox with the pills inside with everything, like he sold his TB medication, the pillbox, he sold his HIV uhhh medication. I’m like “dude how do you do that” but he did it.

I: [Laughing] Yoh it’s quite interesting.

P: *Yah* [yes]

I: Mmm, what do you -uhhh we talked about uhhh the training, right? You explained that you, you were, you think the information like uhhh was provided to you guys was enough and you were expected to go and do what you were trained at in the facility. So, now what I want to know is in future uhhh for these trainings neh, who should fit in to these trainings since you have been involved with the implementation of this uhhh DAT, so now going forward, who do you think should or must really attend these trainings?

P: Uhm I also think patients, like they should also try to involve patients…*Yah* [yes] they should also try to involve patients and also maybe at the facility also the nurse I’m not sure if the nurse do go for this type of trainings they do, okay *yah* [yes]. I think maybe patients also should be involved in this because I mean we are enrolling patients so we should involve them in such things *ukuthi* [that] they go to the facility they know okay we are ready for this because this is going to help us *yah* [yes]. I think patients should be involved also.

I: Okay and how long do you think they should last? These trainings?

P: Mmm, I think it has to be different for nurses and your research assistant should…okay no for nurses it should be three days neh for them to also understand the platform works how the platform works understanding *ukuthi* [that] we are trying to baby the patient because my nurse would always tell me “you are babying these people you must be strict you must be firm” which is not like that you are firm in your own way. So, with the nurse it should be three days, with the research assistant it should be five days like along with the…it should be like along with the moment you start actually the moment you start with your exams your tests because we did write our tests and the moment you get recruited to xxx (organization name) the training should be starting from there. And then with your patients it should be maybe a day, I think a day would be enough for them.

I: What type of content should be provided? what activities should be done uhhh what these people should be trained on basically?

P: Okay with the patients I think they should be trained on acknowledging that there will be a study or a there will be a system that will be used for their treatment, with the nurse it should be like that but also them acknowledging that they are going to come and also them understanding *ukuthi* [that] this is their role, these people, the duties and their roles are this way like do not when you are trying to talk to a patient sister *uyangena* [enter] *yah* [yes] “You must listen to this person” no they should understand that I’m giving you time with this patient also you also should respect that because we would get a patient who would also say “no its fine I do not want that” or we would get a patient who agrees on what the sister is saying “do this get enrolled because you’re a headache” no a patient should be willing to join so the nurses also should understand you do not force a patient to join because next thing it’s your problem because the patient is not adhering properly and the nurse also knows this person it’s the fourth time he/she is taking the TB medication why are you forcing this person to join do not force him. I used to tell my patients that the thing is that my facility is so small I didn’t have my own room I wish I had my own room so that patients can be free with me can be free with me and say “yes I do want to join and I want to join because I feel like this is going to assist me” not because the nurse said “*wena* [you] join” because you get a patient whose joining because the nurse is starring at that person in the eye and they be like “yes please enrol me” but you see that this person is not like probably he/she wanted to think about it first before joining but because the nurses should understand that we are here for patients but patients also should have the right to say “yes” willingly not because you said “*Yey* [you] join” because I would get patients I think I had two patients who joined because the nurse said they must join, which is wrong what if the patient didn’t want to join? But because the nurse would say “*Yey* *wena* [hey you] join” because you know that you don’t take your treatment and the patient would laugh saying no its fine, I do want to join but you’d see that she doesn’t want to join girl and you don’t really need to join because sister said you must join. So, I feel like they should know that this is how we operate and the patients also should know that with this study we are here for them, and we not here for them by forcing them, no we here for them by them willingly joining not by forcing them which is wrong.

I: Mmm did they tell you that uhhh I joined because I felt that the nurse persuaded me?

P: No, because in their minds they like no this person works with the nurse if I tell this person *ukuthi* [that] I joined because… no but you could see *ukuthi* [that] okay you are busy explaining okay and the patient does understand but maybe he/she wants to think about it because number one guys it’s the results, you are shocked that you have TB and then now it’s this. I mean you need time to get home and explain to your family that this is how things are going to operate I’m no more gonna use your glasses if I drink out of a bottle do not use my bottle because I have TB. These things need step-

I: Yes\.

P: You know especially with people who cannot accept their results but because the nurse is here “*Yey* *wena* [hey you] join this is going to help you” no, this person must do this willingly because with TB after two weeks the person is going to come back I’m sure the mind will be fresh the patient would have understood what is happening in his/her body, then she would come back and tell me that “sister I’m ready to join” or maybe “sister I’m not ready to join” but you do get those patients who are initiated first but they are ready to join immediately but others its shocking for them *ukuthi* [that] I have TB because people picture TB as a killing disease which is not and we need to explain to a patient that this is not a killing disease you going to be okay if you take your treatment properly you gonna be cured, it’s not going to come back only if you take your treatment correctly. So, you would get patients from sister *ekutshela* *ukuthi* [telling you that] telling them to join and the patient would say “okay nurse please I want to join” which is wrong I mean that person probably needs time and I feel like even if it’s like that I mean you’re a sister and you can see that this person is shocked by the results you need to be sincere with that person you need to think for that person. Okay with this study I pictured it as let me put myself in these peoples shoes because number one we don’t know what they go through at all and you are busy insisting and shouting and you know probably she/he when he gets home his gonna hide it back, he’s gonna open it whenever his mind is telling him go open, so I wanted all my patients actually because I had patients who were actually forced to join but I also wanted patients to be enrolled willingly you know with a mindset because I felt like with those type of patients that’s… those patients who would just open and close and because its opened its fine for them, number one sister bothered me to join so I’m going to bother her.

I: Okay, interesting uhhh so from your perspective what do you think are the benefits uhhh of the differentiated model of care uhhh I’m talking about your phone call, follow up phone call, SMS, and home visits?

P: The benefits of that.

I: Mmm (yes).

P: Okay, with the phone call uhh okay with the SMS actually it’s, the SMS, and the phone call I feel like the benefits are more likely the same and this patient sees *ukuthi* [that] we really do care about them, *neh* [right?] we really do care about them. With the home visit it, is like okay these people they really, really trying to help me get cured, not TB completed but get cured. But with the TB you might be TB complete, TB treatment completed and come back after years, so you would see *ukuthi* [that] patients are really glad when you come to a place “okay no guys, thank you for coming and checking” because you would, I would also tell the xxxx (organization name) team, right?] if I see *ukuthi* [that] this person opened the box for two weeks properly, like and maybe the date is very far. So maybe they don’t have too much work to do I would tell them “go please go to this person and tell them *ukuthi* [that] thank you, thank you so much for opening, just check up on them because I remember I had a patient who had Cancer, lung cancer and TB also, *yah* [yes] so with her hhhhh it was kind of difficult but with her I used to go *mina* [me] personally with the other colleagues from xxx (organization name) , yes I used to go personally just to check up on her, she was a very lovely uhhh lady and very young, so she would like tell us *ukuthi* [that] “yoh guys are kind in that clinic, ever since you two groups came in the facility so thank you” and we would also acknowledge *ukuthi* [that] “no we thank you also for taking the treatment, like you are really helping us help you, *yah* [yes].

Cured, get cured because I think the reason why she had cancer is because I think she discontinued treatment and cancer developed then she took treatment again. That was hurting her, I think it was lung cancer or something, *yah* [yes]. Because I think she stayed at the hospital while she was enrolled, she stayed at the hospital for three weeks because she didn’t open the box for three weeks until we went to her place because the husband was not, the husband was ignoring our calls, so we went there and the husband was like “yoh I’m sorry but I was kindly stressed because my wife has cancer now and she’s in hospital and at hospital they told her to discontinue and she was stopped on TB treatment until further notice, something like that, until she starts keno, *yah* [yes] until she starts keno. And then after, she will start take TB treatment only when they see that keno is working for the patient. So, and then we would also go to the nurse, to the TB nurse give her the feedback that the patient is at the hospital, that’s the reason why she couldn’t come, but also, starting from now the xxx (organization name) would be like, we would be the one who are taking the patient’s treatment at her place because she cannot come here daily or monthly. *Yah* [yes] so the xxxx (organization name) offered to take the patient’s medication whenever it’s the follow up date and then because one of the xxxx (organization team) team was a nurse also, she was like “no, I’ll also check the blood pressure, I’ll also check the weight, I’ll just check everything then I’ll come and give you everything so that you can write on the file, so it was.

I: Okay.

P: So, *yah* [yes] uhhh I think, I think that’s it.

I: [Laughing] Okay, and then now, the benefits of uhhh of the box or the labels, I don’t know if you can start with one or just use a blanket approach maybe you just combine them, the benefits of the DATs whether it’s the box or the labels to patients, on the patients’ side. How does it benefit, how do you think it benefits patients?

P: Okay uhhh I’m going to start with the label, *neh* [right?]

I: Mmm.

P: I felt like the benefits of the label is because you would find patients who want to show a nurse *ukuthi* [that] I can do this. So, with the label I feel like the benefits was like “nurse do you see I’m taking my medication” and also it was a proof to a nurse *ukuthi* [that] “okay, this person is taking medication” because you take your medication and SMS and then there’s a code, *yah* [yes]. So, you would use that code until one pocket if finished, another code, that’s the pin code but the number of SMS does not change. So, the benefit of the SMS for me was like the patients uhhh fighting this illness actually, *yah* [yes] trying to get cured. And then with the box, the benefits of the box is so much, patient being reminded, and it also benefited the people that uhhh patients that did not have a phone. Another one is a patient being reminded to come to the facility, it’s patient who do not maybe check the cards because the follow up date they would put it on the card, maybe the patient did not see a card or lost the card and then the pillbox is there, you know *ukuthi* [that] the yellow light is me going to the facility, the green light is me opening the box, the red light oh my battery is low I need to go back to the facility and change my battery, I mean I’m enjoying this, I wanna get cured. I feel like with uhhh with this study the aim was to cure a lot of patients, not for patients to be TB treatment to be completed but to get cured and we did get many cured uhhh outcomes for patients, *yah* [yes]. We did get cured and then we also get TB completed treatment completed but it was not a failure for us at least these patients completed, unlike TB failure

I: Mmm.

P: Or lost to follow, no we did get lost to follow also.

I: Yes

P: *Yah* [yes] so with this study I feel like it’s trying to cure the rate of TB, *yah* [yes].

I: Okay, now uhhh the benefits to you as Health Care Workers in the TB room, how did it benefit or assist you guys in terms of doing your job?

P: Also-

I: Mmm.

P: It, it, it, it’s the outcome that comes with each patient *ukuthi* [that] “okay, that one is cured” and also it less list of patients, list of TB rate is going down, like it’s going down, it’s going down. So, for a nurse it’s like “yoh I’ve done a very good job” even for us also the Research Assistant it’s like “yoh mom basically this is helping them” so, it’s a well done for everybody, it’s a wining situation actually. So, it you cured this patient, you helped this person uhhh get cured and for you it’s outstanding, it’s like yoh you did your job. Now nurses enjoying, now nurses enjoy their job *ukuthi* [that] yoh at least I can cure somebody, yes.

I: Okay uhhh and then how did then help you guys to monitor adherence compared to traditional ways of monitoring adherence?

P: Okay, *angithi* [isn’t] back then they used to check maybe inside the files *ukuthi* [that] okay when is this patient coming back or also, they would also like check with the pills *ukuthi* [that] how many pills do you bring back as a patient, *yah* [yes] but since we introduced this technology as more, now you see particularly that this person did take the pills today, this person took the pills yesterday, tomorrow, it’s a daily routine check-up, *yah* [yes]. So, for the nurses it was like “yoh okay now I see *ukuthi* [that] this one is fooling around, this one is busy taking, this one, weekend there is an issue. So, *yah* [yes]” it’s a daily check-up routine and for nurses it was very nice for them because they know *ukuthi* [that] when you lie they would see on the platform and when you come back for the follow up they would tell you *ukuthi* [that] “*wena* [you] on Thursday, what was your issue not opening the box?” then a patient “yoh nurse *ngikhohliwe* [I forgot] I’m so, so sorry about that but I did open my box, it just that later on” *yah* [yes] but because the platform uhhh knowledge *ukuthi* [that] eight o’clock you must open your pillbox uhhh later on it will become yellow when you open it maybe later on it becomes yellow and then maybe after tomorrow it’s gonna show *ukuthi* [that] this patient opened the pillbox, with the yellow maybe it’s still refreshing *ukuthi* [that] okay you did open your box but you opened it later on, *yah* [yes] so *unurse* [the nurse] would also see that “no, I see that you opened but also remember, you gave us a certain time so please when the alarm goes on, just take your medication at that certain time then you can be busy with whatever you were busy with” *yah* [yes].

I: Okay, and then do you think it’s impacted on the relationship between the nurses and patients in a good way?

P: *Yah* [yes] yes it did.

I: Mmm.

P: Yes, it did because now you would see *ukuthi* [that] okay the nurse is just uhhh happy when they see the patients and also the patients were just you know, they either to come for the follow up date, they would also come a day before or two days before that’s how they are eager to get cured and they enjoying this not that they enjoy taking the medication but they enjoying the-

I: The support

P: The services and support yes, the support and the service that’s being given to them and yeah.

I: Okay uhhh stigma? Were there any concerns? Besides the one case you mentioned?

P: Oh, *yah* *yah* *yah* [yes yes yes] I think it was just only that like the youth would say “this is for old people” or “no people are going to say I’m not only taking TB medication I’m taking something else I don’t want this. Its fine when I SMS” *yah* [yes].

I: Okay and then now challenges with the differentiated model of care uhhh on calls, SMS home visit you can start with SMS or phone call.

P: Okay challenge with the SMS uhhh, actually with SMSs there were not a lot of challenges just that you would get feedback from the patient *ukuthi* [that] “hey no, I see an SMS but I did open my box and but you guys did send me an SMS also” so I was like we need to talk about this SMS issue but we did address it with the manager and I think the patient came back and he was like “thank you for addressing that with your boss but the SMSs has stopped”. With phone calls my challenge was I wouldn’t get that person; I would maybe get uhhh I would like the phone call you would probably think that you must leave a message but next thing like maybe the patient hangs up quickly *yah* [yes]. And also I would get uhhh maybe the phone I don’t know if they blocked my phone number or what and also the phone is always off then maybe you would hear when they come for their next visit they like “no” … hence I said I felt like that place is a bit rural like the network issue was very very bad and you know patients you would think that maybe they blocked you but its not like that or maybe the patients phone is always off no its not like that it’s the issue of network because we had patients who lived in xxxx (area name) which is after xxxx (another area name), it’s way far, it’s after that so there its *emukhukhwini* [Informal settlement] so network is not really like the reception until maybe you stand at xxx (area name) then that’s when you pick up the network, but you can not like take your medication and run to xxx (area name) to send you and SMS or maybe whenever you get a call run to xxx (area name) so yeah the challenge also was that network issue. With home visits the challenge was they gave us wrong addresses guys I think I did address this with the sister in charge *ukuthi* [that] it’s very difficult for us to do home visits, number one your patients, you allow patients to open files without proof of res. I mean I’m a patient uhhh I walk down I see there its written xxx (area name), I’m going to take your location and when you go there you people don’t know who is this person and the challenge was just wrong addresses, wrong addresses and these people they live at your xxx (area name) and your xxx (area name) and that place is very far from xxx (area name) its outside xxxx (area name) its the entrance of xxx (are name). So, it’s a bit far to do home visits there because number one the place does not have street names its only like addresses its just house numbers and you find *ukuthi* [that] this is house number 214 xxx(street name) that one is 214 (same street name) then which house is right? You, see? The challenge was just addresses yoh their addresses because we had a patient who lied about her address apparently that person lives in xxxx (area name) so that person actually didn’t mind going to xxx [facility name] than going to facilities in xxx (area where he stays) , so we were like “yoh this lady” because I think one of the friend actually knew her *ukuthi* [that] “no this person lives in xxx (area name), this address is not hers I don’t know why she uses this address I don’t know. Maybe if you go check probably, she rented there” but when we go there, they don’t even know that person kanti this person lives xxxx (area name) I’m like how are we going get there and this person had a pill box also I’m like how are we going to do this, but at least she was drinking her medication properly more than the people who lived very close to the facility. She was taking her medication properly there were no issues I remember didn’t have to call her every time or do home visits actually just that xxxx (organization name) wanted to do speed time check for the family so that’s when we found out that this person lives in xxx (area name) so how are we going to do this? But because xxx (organization name) also traces patients in xxx (area name) I think that’s how they found her and yeah… so she ran away from the clinic in xxx (area name) then went to xxx [facility name] to get help there. So, the challenge with the SMS is network and with the phone call it’s also network with the address those people gave us the wrong address, wrong addresses. You’d find *ukuthi* [that] a person lives at (area name) and then that person *uya* [goes to] xxx (clinic name) and then they said its not allowed because there’s a clinic close by *yah* [yes] uhhh because it’s also difficult *angithi* [as I say] WBOT because they walk, they have to go from a certain area to until a certain area, but xxx (organization name) didn’t mind as along as its in xxx (province name) *yah* [yes]. So with the WBOT it was a challenge actually *ukuthi* [that] we cannot walk from here to xxx (area name) and we cannot also use our money because we not going to get our money back but if this patient comes just try to take that patient to the correct clinic but also you’ll find *ukuthi* [that] patients they are okay with this facility they don’t want to be moved maybe this person lives in xxxx (area name) and there is a clinic in (area where they live) that person chooses to come to xxx [facility name] you cannot chase the patient away, that’s what we learned ukuthi [that] you don’t chase patients away we sit with them, if the patient does come for his/her follow up then I don’t see the issue of you fussing as a nurse ukuthi[that] why are you coming to this facility if they find *ukuthi* [that] xxx [facility name] service is proper than xxx (area name) its fine leave them there. Then you would find the nurse saying “they creating no patients for us” I’m like okay with that I’m not concerned but I feel like if a patient feels ukuthi[that] you guys are bringing better services than a different clinic you guys should be happy not chasing people away *yah* [yes].

I: Okay you mentioned SMS one patient who was receiving an SMS uhhh I just want to understand what was happening, was he getting an SMS after opening the box?

P: Yes

I: Mmm.

P: After and before. So the patient was like okay I really do not know what’s happening but I’m getting SMSs reminders *ukuthi* [that] for the day please don’t forget to send an SMS or for a day please do not forget to open your box so the patient would call me actually telling me *ukuthi* [that] “hey sister xxx [intern’s name] I’m getting an SMS ukuthi I must open and I do open my box” and I see on the platform *ukuthi* [that] this person opened the box at 8 o’clock, 8 o’clock it’s the alarm time but also he opened the box at again at 10 because he got that SMS probably he thought that *ukuthi* [that] maybe the platform did not read it then he would open the box at 10 then I would tell him “no, whenever you get another SMS do not open the box just leave it neh” but I’m going to talk to my manager about this SMS uhhh thing because you do, you do take your medication you open and you drink then you close it but still you get SMSs then he would get an SMS again at 8 o’clock during the night *ukuthi* [that] for the day you must just open…I’m like yoh okay no we need to address this because the patient is going to get fed up next thing his going to tell us guys please remove me from this because I’m getting irritated but at least the patient was very understanding *ukuthi* [that] okay no I know its not you sending the SMS but just at least please talk to them, but the SMSs stopped actually…then he was like “ okay no, thank you so much.”

I: Okay, and with the issue of wrong addresses I mean uhhh in future or even now because there’s still an issue, I believe, how do you think it can be solved?

P: Okay, because with the wrong address it starts at the filing room when we open a file for a patient *neh* [right?] I fell like *ukuthi* [that] they must just ask for proof of res, if maybe you don’t have proof of res, guys our bank statements come out with a proof of res, we don’t need to see how much you getting, no it’s fine you can just cut it out, you can just cut it out, we just need a proof only *ukuthi* [that] okay yes you do live here, *yah* [yes]. We must see *ukuthi* [that] we must see by proof ukuthi [that] yes you do live at number 123 and confirm what, what, what because it’s very difficult for us *ukuthi* *siku* track [to track you] next thing because some of them would also like just knowing *ukuthi* [that] okay hhhhh my treatment will be completed on a certain day, I still have pills, I’m gonna keep the box I’m not gonna take it away. And we won’t get like patients taking the box without them telling us “Can I please have the box” because we would find many people ask for a permission. With the other ones probably she was trying to come back to the facility but because you gave us the wrong address we cannot go also to your place and collect it because I used to get patients who uhhh the nurse would tell them “your TB, like you are TB cured but just complete these pills that you have now” and then complete them because they don’t wanna be removed from the study yet until pills are finished. Then they would communicate and say, “I’m done with the box, uhhh I will come to you, because I’m taking my high blood medication I will come with it to the facility, then I will give it to you.” Oh, I’ve never found a patient who would leave it at different nurse, or they would always leave it with me, or they tell me *ukuthi* (that) it’s at home and you can just come and collect it. So, me coming I would send the xxx (organization name) to go and collect it then bring it back, but only if the address is correct, but sometimes we get *ukuthi* [that] uhhh this person does not live there, and it’s a patient who, who has been adhering very properly on the platform we didn’t need to do a home visit until, whereby we had to fetch the pill box, and the patient was just taking TB med….uhhh TB treatment only and then she/ he is lazy to come to the facility to give us the box, and all that, because if, I feel like if only, the only time when you are lazy its because you give us the wrong address and you leave very far, you gonna spend so much on transport coming to the clinic just to drop off pill box then going back, so they will keep it and then like yoh! And then we would send xxxx (organization name) to please go collect the box, next thing they can not find this person because this person does not leave there, and at that pacific uhhh at that specific house they said they leave in, they don’t know them, so basically maybe she was going down the road then she saw the address *ukuthi* [that] yeah *ngizoyithatha* le [I will take this one] I’m gonna say I leave in there, yeah.

I: Okay, uhhh with the box neh, were there any technical glitches with the box uhhh being reported maybe a patient opened the box but, on the platform, it shows that they didn’t open yet they claim that they opened the box?

P: Mmm yeah, uhh , yeah it goes back to knowing your patients, knowing ukuthi [that] this one I know *ukuthi* [that] this is, is one of those patients who has been bothering u [the] nurse maybe before his treatment or before her treatment, and the nurse already knows that patient [*ukuth*] that she might be lying, she didn’t open it and she is going to use the excuse of I did open it just that the platform didn’t notify then that’s when we go we do that …….khante [meanwhile] the patient did not even open the box at all, yeah because we couldn’t tell the patient *wena* [you] you are lying, then we will open it, but when we open we would see with medication, *angithi* [ right] we call neh, we call bathong sister why didn’t you open the pill box no I did maybe it didn’t recall okay fine we do it manually but when the patient comes fall out we see *ukuthi* [that] these are the four pills that the patients didn’t take for that day because uhhh the TB nurse would count if maybe they say it’s for a month its on 24th of January until the 24th of February it’s a month so you must come with empty packets but *wena* [you] have four left or six left how is that? Then we see *ukuthi* [that] oh this is the day that she didn’t so she lied basically, and we already changed manually yeah.

I: Okay, and uhhh do you think TB treatment can be improved you know using the DAT the stickers of the box, do you think it can improve TB treatment?

P: No, no I feel like with this study its quite a success straight actually because I think we had a lot of TB cured I think it was a few that were uhhh TB treatment completed lost to follow TB failure and death it was just a few but, with TB cured it was a lot like we had a lot of TB cured. So, I feel like nothing can be improved just they should also consider those ones the ones that take a lot medication for TB *yah* [yes] they should consider a very huge box for them, a reminding box also for them *ukuthi* [that] hey and also…because they do a lot of visit in clinics because they go to xxx (hospital name) they go to xxxx (clinic name) to take blood or something like that like they do a lot of things so they should be implementing a box for them also reminding them *ukuthi* [that] on this certain day you go to your clinic, on this certain day you go collect your medication and on this certain day you go see your sister for follow up and yeah.

I: Okay, and with adherence do you think uhhh the DAT helps in improving adherence?

P: Yes, it does yoh it does a lot yoh it helped a lot, a lot, a lot I mean the nurses also even nurses who were uhhh were at the facility but doing different services were like yoh ever since you guys came like TB because they would see the stats the radius very, very, very, very good compared to uhhh because stats there’s a portion where it asks *ukuthi* [that] how many lost to follow are they? Back then before we came you would find *ukuthi* [that] its ten you know its eleven but ever since it was zero, zero sister was flowing with ama zeros everywhere ever since we came, its was zero lost to follow, it was zero death it was zero also *yah* [yes] so I felt like this is helping both patient and the nurse yeah.

I: So, you are saying there was a difference uhhh before the implementation and after?

P: Yes, there was a huge difference I mean even the sister in charge told us was like yoh ever since this company came *yah* [yes] TB is going down *yah* [yes] TB is going down and also I felt like the wearing of the mask also assisted *yah* [yes] the wearing of the mask also assisted and because these patience also attended TB dell they would show them if maybe he had a cough for two weeks or more than two weeks like you feel like this is not just a regular cough and you did go to test and you still waiting for your results this is how you cough and do not like spread it you know I feel like it’s really working for both the clinic and the facility and the patient and the nurse also.

I: Okay…So now since this was still the research, right? And if it shows the success that this thing is really working uhhh and xxx (organization name) is now handing over to the DOH and the DOH is taking over now uhhh, what do you think or how can these positive changes you just highlighted they improve adherence, the success rate, patient completing treatment and be cured. How can all those positive changes be sustained in the absence of xxxx (organization name) now when the DOH is taking over, what can you say uhhh should be done by the DOH in order to, you know, to sustain these positive changes?

P: Okay uhm … I think they should… I’m not sure if this study is implemented in every clinic but I think if maybe they implement this at every clinic with with…because *yah* [yes] it was not at xxx (area name), xxxx (area name) didn’t have that I think if they implement this at every clinic it’s going to assist with the rate because I saw the rate of xxxx (clinic name) is a bit higher because I remember the nurse also would tell us *ukuthi* [that] how do you guys do this? I’m like no, because we are here, and (organisation name) is here because she thought *ukuthi* [that] it was my control facility I was doing the… with xxx (area name) I was doing hhhhh what is this…the data collecting neh xxxx (clinic name) and clinic too they both in xxx (area name) so like they would always ask *ukuthi* [that] we always see stats from xxx [facility name] the rate the lost to follow up like who does your home visits? Who goes and do the check-up I’m like no its us its xxx (organization name) and xxxx (organization name) and also xxx (organization name) so they would always ask ukuthi[that] cant these pill boxes maybe just bring five for our patients but the thing is its not like its not on the platform there’s no boxes in xxx (clinic name) and xxx (clinic name) so the nurses would also ask *ukuthi* [that] can this also be included in our clinic so I think if xxx (organization name) hands it over to the government then like it should be implemented in every clinic because they see *ukuthi* [that] the outcome is properly like its proper actually its proper for xxxx (facility name) more than if you compare . xxx [facility name] lost to follows zero, zero, xxx (clinic name) lost to follow ten or five xxx (clinic name) is ten or five so then it’s like okay why can’t this be implemented in our clinic also because i fell like if its working for xxx [facility name] it’s going to work also for this clinic so I think if maybe it becomes implemented in every clinic its going to be okay for everybody yeah the rate of TB is going to be cured for every patient.

I: Okay uhh now as I’ve just uhhh mentioned before uhhh basically it’s a continuation uhhh or it’s a follow up question from the one that I asked DOH taking over in the absence of (organisation name) what is needed hhhhh in order to sustain to make sure that this DAT continues to help patients as it does now. So, now what I want to know from you is what structures need to be put in place, what resources uhhh are needed in order to carry out this intervention?

P: Mmm what resources?

I: Yes, since you have been involved and you know and you have been, you were there using this and that and that, now what is the DOH uhhh going to need in order to carry out the job?

P: Okay, they will also need to uhhh hand out the pillboxes and also the sticker label basically this, also the tablets because they need to check the platform. So, I’m not sure with that are they going to give the nurse or nurse assistants to do that. *Yah* [yes] but I feel like you know they should do, they should carry, they shouldn’t, like when we take over, they shouldn’t change anything, they should really uhhh try to do whatever we were doing. and I also think of, it will also improve on job employment for the government also, I mean uhhh probably in other facilities there’s, there are no uhhh nurse assistants but maybe if you recruit the, the uhhh the EPWP it those ladies who are there, the guys you must teach them how to operate this and then let them work with the nurse permanently because this is, this has to be the permanent thing actually it has to.

I: Okay, and what are they going to need in terms of the resources uhhh boxes, you mentioned boxes, what else are they going to need?

P: Mmm they are must also uhhh you’ll need actually they will also need the stickers also; they will also need tablets and also need the xxxx (adherence platform) that they need there. They will also, they will also need what? Forms, you know *yah* [yes] because uhhh they need to also know *ukuthi* [that] patients are obliged to say “no” patients are, or patients should be, are willing to join like they should not force patients hence I’m saying they should take over how (organisation name), how (organisation name) is operating this already they should not change anything, but do exactly what (organisation name) has been doing yeah, continue, continuing on whatever that (organisation name) has been doing also.

I: Okay, and who should be put in place uhhh to help uhhh like preparing the boxes or the labels since you mentioned that it was some of the (organisation name) staff was doing that so, now in the absence of (organisation name) who should continue doing that?

P: The government, yeah, I think they should be the ones, that they should just take over everything yeah, should know *ukuthi* [that] where they should get the boxes, the supplying of the box, supplying of the stickers, forms, tablets like tablets should be there because without that, I mean its very pointless for them to operate the pill boxes because yoh! Its gonna be difficult basically its like giving a patient just a box just to put that medication so, yeah, I think the government should be the ones taking over and making sure *ukuthi* [that] everything is correctly by them operating everything, yeah.

I: And who should help with the technical issues, you mentioned the box where the patient, was preparing the box and it didn’t report on the platform, patient experiencing network issues, I don’t know if uhhh something can be done about that but yeah just highlighting the issues, technical issues, who should help?

P: Okay with the technical issues maybe they should, okay I don’t think they should, they should, they should, uhhh remove they should take control of everything of the (organisation name) yeah because I don’t think they will manage neh with the technical issues I think (organisation name) should stick to that also with the supplying actually so that (organisation name) should not leave the company in departments hands but uhhh the supplying should come from (organisation name), (organisation name) should be the supplier for, for the government also with the technical issue (organisation name) should be there and in terms of calling and sms should be *abo* [the] nurses, nurses should also like you know, because I saw with uhhh with TB rooms, with TB rooms in facilities they don’t have so many patients, so I feel like them maybe taking five minutes of their time maybe checking the file because you know with us neh, with me what I used to do I used to also check in the file *ukuthi* [that] when is follow date for this patient then does that correspond with the one on the Tablet neh, on the platform but if it doesn’t I change it or I call because also, we also write on the on the card on the review card neh, the thing is uhhh *angithi* *thina* our nurse was a pensioner so she would maybe uhhh switch dates on everything so would get confirmation via the file or a patient, calling a patient yeah so hence I’m saying maybe like the nurse would take five minutes of their time just to call a patient and share whatever challenges that we were facing, calling a patient writing like noting down yeah I think the nurse should be the one doing it and then calling and then also checking on the platform if the patient is taking I mean I think that one she can do it in the morning yeah because you will find *ukuthi* [that] so many patients want to take their medication very early around 7/8 yeah so *u* [the] nurse can be doing that before letting *ama* [them] patients in the after she can like maybe she done with everything, she done with her launch she can start calling patients because this what I did here, call patients *ukuthi* [that] okay in see *ukuthi* [that] you know you didn’t open your box today what is the reason she must note it down, yeah and then in terms of when a patient is being enrolled, *neh* [right?] uhm the people who are in the TB room also should also take part *yah* [yes] they should also take part, they should also know ukuth how this is working, how uhh how the, the, the, how the pills should be set inside the pillbox and what should be, which type of pills should be set in the pillbox because you would find also maybe, I remember one of the lady that worked with us, she even placed uhm flue medication in there because patient had flue. She placed flue medication in the pillbox, so the lady was busy opening it because she wanted to take flue medication then I was like no you must take them out, only TB medication supposed to be there, *yah* [yes].

I: Okay with the issues network, SMS, patients complaining about the SMS, and you trying to call patients and not reach them because the phone is on voicemail, home visit wrong addresses, so all of that uhhh I want to know, was there a system in place or were you capturing those somewhere?

P: Yes, it was also the platform under the task-

I: Mmm.

P: List, *neh* [right?] so basically *angithi* [isn’t] you call or maybe it takes you to voicemail, you hang up and then you write under the tasks list on that particular patient *ukuthi* [that] you tried calling because it helped uhhh comment bellow section, it’s the date today’s day, after it the type of hhhhh type of system that you used, it either you called, SMS or you did home visit. So, you choose and then you choose the time that you called around and then there’s a comment list, if maybe the patient did respond you comment *ukuthi* [that] the patient responded and then if maybe the patient did not respond “the patient did not respond due to maybe the phone was off or network system or something like that” even if the patient responds you note it down *ukuthi* [that] okay the patient did respond and then the patient told me that uhhh “I did take my pills” it just that maybe the system, the platform has, system either or something, *yah* [yes] maybe if you guys can please check it out form me because the patient said he did take the medication. But after that, you save it, they gonna see it, but also you go and only, only when the patient said “I did take my medication” you go and check it on the manual, you change it on the manual on the platform then it becomes green. But if the patient sis not respond you leave it red until the patient comes back until does respond your call then you ask him *ukuthi* [that] okay on the 24th uhhh I see ukuthi [that] you didn’t open your box, what was the reason, then they would tell then you can go, you can only go change it after you have heard the patient’s story.

I: Okay, and was there a common tool that was similar to this uhhh which was shared uhhh by other Health Care Workers in the TB room-

P: No.

I: Besides the one that you were using?

P: No.

I: Okay uhhh can you please describe any gaps uhhh that you know which exist in the implementation of the DAT, were there any gaps that you, you know in terms of how this uhhh intervention was being delivered? Were there things that you saw maybe that they need to be improved maybe the way uhhh with phone calls, with home visit, messages, the DATs itself, you know, whether it the box or the labels?

P: Okay uhhh I don’t think should be implemented because I feel like everything is just correct now at the moment, *neh* [right?] but also I think maybe they can improve on, on the uhhh SMS issue, they can also improve on letting uhhh letting the, the, the Research Assistants be the one doing home visit because I feel like it, it, it’s a bit difficult to open up to a person who enrolled you than a person who just came to, to, to get to know *ukuthi* [that] why didn’t you open, I mean uhhh during the enrolment, the xxxx (organization name) were not in the room, *neh* [right?] it was me and a patient and a nurse, *neh* [right?] then next thing you send somebody to come and check up on you, basically it’s like me telling another person my status or my, my, my issue, probably something like, probably the issue is very deep and you are comfortable maybe the patient is comfortable talking to you to the person who enrolled because she feels like or he feels like there’s a very good communication between you guys and he or she is open up whatever you guys are, *yah* [yes] so next thing you send somebody else and then obviously they gonna lie until the patient come and be like “yoh this is the difficult that I was facing uhhh because, because hence I said the place was very rural, *neh* [right?] hhhhh you would get a patient telling you that uhhh I didn’t have anything to eat or I couldn’t take my medication because the last time I ate was yesterday during the day so with that that’s when *wena* [you] Research Assistant involve one of the, because in the xxx (organization name) they had a Social Worker, *yah* [yes] you would involve her and talk to her *ukuthi* [that] please just go at her place and maybe you would find *ukuthi* [that] there’s nobody working even at home. So, she cannot display such matters to just a person that just literally came than a person who is busy checking up on her you know, so I feel like they should also allow the Research Assistants if the job is done in the facility to allow them to go and do home visit at some of these patients because you would get *ukuthi* [that] you calling uhhh you calling the patient he responding then next thing because you want them to take their medication, they would just open the box then close it because we don’t know what’s happening.

I: Okay, speaking of home visit neh [right?] uhhh besides the one who provides uhhh wrong addresses, now with those who, who provide uhhh the correct ones and the WBOT would go there find them where they stay-

P: Mmm.

I: But now uhhh I want to know if were there any cases of finding a patient *ukuthi* [that] okay this patient so and so stays here but they would not maybe open a door for you or they would just run away the minutes they see you by the gate, they just run away?

P: *Yah* [yes] *yah* [yes] *yah* [yes]

I: Or complain rather that “no next time don’t come; I don’t want you to come here-

P: Mmm.

I: Were there any cases?

P: [Laughing] I had uhhh we had another patient actually uhhh he chased the xxx (organization name) with the knife [laughing] he chased them with the knife and yah [yes] uhhh and when he came to the facility they were like okay what was the issue, apparently the car they came with was written xxxx (organization name) and they were wearing nurse’s outfit. So, for them is like now my neighbours are seeing me in my place obviously that means they know ukuthi [that] I’m sick or something though they don’t know the sickness but clinic people are coming at your facility. So, with that, with that address and you like okay maybe if we do home visit how abut we go casual and to maybe the sticker of the car should be removed, because I mean the neighbours know ukuthi [that] xxxx (organization name) work at the clinic maybe change the car like don’t come with the car that has a sticker of xxx (organization name) yeah because, like guys this how we gonna get chased every time, or the patient will just look at you through the window and you will knock and knock and knock, they will not open because number one you wearing nurses uniform, you are in a car that … printed (organization name) or xxx (organization name). So and they know *ukuthi* [that] I mean its very small place that place they know *ukuthi* [that] I’ve seen this nurse at xxx facility name] I wonder is this person doing in this house. So, yeah, they did get a patient who actually chased them with a knife and like “go away I don’t want you here, don’t do home visit in my place,” yeah.

I: Okay, and with the SMSs I’m wondering if uhhh where there any patients maybe who experienced the issue of unintended uhhh disclosure those who were using the labels when they were getting those SMSs maybe they say, “uhhh my partner or my girlfriend saw this SMS and that’s how they found out because I didn’t tell them.”

P: Oh yes, yes, yes, actually it was one of the guys yeah one of this guy. So, this guy also was the one who gave us the wrong address so we couldn’t, the reason why we were busy okay, he had a phone neh, the phone got stolen he told us that he was okay with us using, no he was okay with him using the girlfriends phone fine. *mara* [but] I think he didn’t tell her what he’s SMSing for what uhhh because the SMS would always remind them to take the treatment, its written treatment uhhh. Then the girl saw it and then she came to the facility alone then she was scared *ukuthi* [that] what kind of treatment is this and then uhhh she told us *ukuthi* [that] uhm because I think she even came to the TB room around 12 for a person who came in the morning because she didn’t know what treatment is this because she was busy going to all the services asking *ukuthi* [ that] guys do you know this type of patient. So, because there was this other nurse who attended uhm the (organisation name) training, she knew actually then she took her to us and then we explained *ukuthi* [that] its TB uhhh, we feel that you live like far from him because that’s what she told us but can we also test you also yeah and then we tested her the results came out negative she was okay uhh with TB yeah.

So, she also wanted to check for HIV because basically it is compulsory to test her she was negative the boyfriend also was negative but because its his treatment the girl was thinking *ukuthi* [that] uhh what kind of treatment are you taking yeah so yeah we had an issue whereby the SMS actually disclose a patients status yeah we did, yeah though she, she didn’t come fighting but she wanted to know *ukuthi* [that] what is this treatment the sms is saying like what kind of treatment is this person taking, yeah and we told him, we told her actually ahh that the next visit is this date please come with him neh, come with him so that he can be the one actually disclosing like telling you because we actually we didn’t know that you guys leave together we knew that he goes, after work he goes to and check you every day because you guys have a son together yeah, and she was like no I moved out he told me that, I should move out for three months I was thinking *ukuthi* [that] it’s because it was the first initiation like he on the first phase normally he thought maybe he is going to infect the child and the mother also and the we like no after this it’s the follow up just come with him so that he can the one disclosing then that’s when the guy disclosed actually and the girl was like no you should have told me and what not and what not yeah, but she was not mad actually just that she scared *ukuthi* [that] what type of treatment is this, yeah.

I: Okay, uhhh we have come to an end of our discussion, but before we close uhhh do you have any final thoughts, any comments, suggestions?

P: No actually.

I: Uhm or something that you just want to comment on or something I did not mention uh?

P: Mmm yeah, actually I think uhhh xxxx (organization name) neh helped us, help people yeah uhhh complete their treatment and acknowledge ukuthi [that] they are not alone in whatever they go through yeah because xxxx (organization name) was more of uhhh xxx (organization name) was more of that thing *yokuthi* [that] a friend, like if I have issues and I can not talk to my close friends that I have this person I can talk to yeah so, I feel like (organisation name) should also like consider this as an achievement *ukuthi* [that] ey guys, you guys are big up in clinics like you guys are yoh you guys are just changing people’s lives, you guys are just amazing. I’m sure like because I remember since the contract ended, one of the patient were like yoh guys we going to miss you. “I’m going to literary miss you and yoh I don’t how I’m going to cope but I know that I’m going to call just that because you were there with us, you were with us in this difficult journey and you know you didn’t even like you know *nowusafeli* *pelo* *mann* [you didn’t give up] you took, you took care of us, you, you made sure that we do take our treatment and also you also assisted us with this pill box, its busy reminding us, for the facility date for yeah.” So, I feel like big ups to xxxx (organization name) and they should continue actually they should, and also they should implement this in every facility guys because so many clinics need this literally so many clinics need this because I remember I went to this clinic in xxx (area name) I was, I was like I had flu so I was like okay why aren’t uhh its in xxx (area name) kere [I’m like] like okay why they do not implement this also in xxx (facility name) because I saw the TB line, TB room it very long, longer than the ANC (Anti natal care) , your acute, your chronic I’m like yoh like they should implement this also in like all facilities I feel like this is gonna work for them yeah it will work actually because it worked for some facilities your xxx (facility name) , your xxxx (clinic name) because I remember, I used to work in xxxx (facility name) before, I worked for xxxx (area name) and I also like the sister in charge she she was very good with us so I did go and visit her after the contract was terminated I went to see her and she was like guys you see this xxxx (area name) thing it helped us even at, I went to the TB side the sister was very very good with us also she was like ayy you guys ever since it’s a bit challenging but also I did managed, I did managed, I managed to this and do that now I’m trying to teach one the EPWP so that when ever I’m busy with the patient she can come and assist me with some yeah but, I feel like they should be implemented everywhere, because it also reduces number of lost to follows and you know it reduces so many things actually.

I: Mmm.

P: People are adhering, people are coming to their visits on the correct date not after five days yeah.

I: Thank you so much uhhh for your time, this is the end of our interview uhm the time is uhh 14: 28 pm.
