# Supplementary material for: Acceptability of Digital Adherence Technologies to support people with drug-susceptible TB in South Africa
Source: PLoS One. 2025 Sep 24;20(9):e0332103. doi: 10.1371/journal.pone.0332103 (PMC12459780; doi:10.1371/journal.pone.0332103)
Supplement: S4 File — (ZIP) [file pone.0332103.s004.zip › S4 Transcripts/HCWs and Stakeholders/IDI 8-HCW.docx]

**TRANSCRIPTION NOTATIONS**

| **Label Key** | **Meaning** |
| --- | --- |
| **I** | Start of each new utterance by the Interviewer |
| **P** | Start of each new utterance by the Participant |
| **N** | Note taker |
| **{ }** | Indicates that details were changed or pseudonyms were used to anonymise data |
| **( )** | Indicates the description provided to anonymise data |
| **XXX** | Words were omitted to anonymise data |
| **-** | Breaking into a sentence by the next speaker |
| **…** | Pause or drawn out words |
| **[ ]** | Indicates noise made, e.g. [laugh], [sigh], [pause] |
| ? | Beginning of utterance by unidentified speaker or questionable text |
| **[inaudible segment]** | Unclear section of the recording |

I: So, do we have permission to record you?

P: Yes, you have the permission to record me.

I: Okay. Date of the of the interview it’s xxxx (interview date). The location is xxx [clinic name] clinic. The language used is huh, English. The PID of the participant is xxx. The time at which the session is starting is 10:32. So, can you tell me, what’s the tittle of your current position?

P: Okay, I’m a Community Health Care Worker.

I: How long have you held this position for?

P: It’s xxxx (number of years)

I: Okay

P: Mmm.

I: Were you working in the same facility?

P: No. I’ve-okay, I started working at xxx [clinic name] then I moved to xxx [clinic name]. Now I’m currently here at xxx [clinic name] clinic.

I: Okay. How long have you worked here for?

P: For two or three years if I’m not mistaken.

I: Okay. So, when it comes to patients care and counselling, what are your roles and responsibilities?

P: Normally, when comes to counselling, *neh* (right)? We don’t have too much roles there because of who- who does the counselling most are the ones that are dealing with HIV ,unless we are doing like socially counselling, like whenever patient come to you and explain about their health, I mean their problem that are associated- like the life problem. That’s when we can say we are offering a- a counselling, but beside that, we don’t do much of a counselling.

I: Okay. So, what are your roles and responsibilities in, in the TB care?

P: Okay. Because of now, *neh* (right)? We are most from TB, but the time I was work-I was working at TB, my roles was to make sure that every patient who enters, who- who are drag- are newly diagnosed on TB. I’ll open up their file and we- we register them and then I’ll be assisting with the collection of the sputum. And also making sure that the results are in the files and also making sure I do the DOT. You’ll remember the DOT are the ones who comes daily or who comes weekly in the clinic.

I: Mmm.

P: So, that was my responsibili- the time that I was working at the TB room.

I: Okay, Alright. So, how are TB services delivered at your level huh, regarding to the Digital Adherence Technology, are you at a facility level, District, or provincial level?

P: Facility level.

I: At facility level?

P: Ehh.

I: So, now I would like to know what you know about ASCENT, right?

P: Mmm.

I: So, if you have to explain what Digital Adherence Technology is to another Health Care Worker who knows nothing about it, what would you tell them?

P: Okay, I’ll- I’d tell the pa-, that particular person that it’s more of technology communication, when you talk of community co-, I mean technology communication, we are involving cell phones, we are involving like something that has to do with technology. That, that is what I can say to that person who don’t know about the technological digital whatever.

I: Mmm.

P: Mmm.

I: And in, in relation to the digital adherence technology offered by ASCENT, what would you tell them?

P: Oh okay, I’ll tell them about- should- can I explain about the box now?

I: Yes.

P: Okay. What I would tell is that, okay this box *neh* (right)? It will help the- it helps a patient to remember the time to take the medication because they will be explain to her or him that on this time, it’s the time that they will set an alarm for you, for you to take a medication. Okay, and I’ll also tell that person that, okay it also helps with the reminding of a patient to go and collect the medication, *yah* (yes)it’s what I can tell because of here at xxx [clinic name] we are using the- the box but, okay the other information that I, I got it was when we were attending the meetings there at the ANEW hotel. Okay, I heard about the, the one that clicks that they are using for collect-, the SMS one.

I: Uhm.

P: *Yah* (yes) *leyona* (it) it one of the par-, it’s one, it’s part of the digital technology.

I: And how does that uh, sleeve one work?

P: Okay, the sleeve one work uhm, not in, I don’t have too much information about it, but one thing I know is that the patient- there’s a SMS number that the patient has to send after taking the medication. And if, if the patient is not taking the medication because of the- the cell phone numbers and what, what, they can do the follow ups.

I: Mmm.

P: *Yah* (yes), *yah* (yes) whenever a patient is taking a medication, has to send that particular sms to, to the, to that person who is monitoring the adherence of that particular person.

I: Okay

P: Mmm.

I: So, how is uh, this person monitoring the adherence of this person? You’ve mentioned the digital adherence technology from the patient perspective what they use, the sleeve and the box. What else can you tell us about the digital adherence technology?

P: What else can I tell uhm.

I: You mentioned uh, someone would be monitoring, how do they monitor?

P: How do they monitor?

I: Mmm (yes).

P: Okay, let’s say the person who’s monitoring the box, *neh* (right)? They, they capture the information on a tablet, so that information will let- will alert that person, whether the patient has taken the medication, so the person will have uhm , a calendar to see that okay on this day, the, the certain uh-this person took her medication, this person took her medication like that. So, that’s how they monitor it by, by- it will reflect whenever the box is opening, that okay today this person has opened the medication on this time, and she took her medication.

I: Okay

P: Mmm.

I: So, what’s the name of that system with the calendar on the tablet, do you know?

P: Uhm ,yoh even if I don’t-, I forgot about it [laugh].

I: Okay. So, when you check uh, on that tablet- on that calendar, how do you know that this person has missed medication, this has taken medication?

P: Okay, when the person is taking the medication it’s gonna reflects green.

I: Mmm.

P: And when the patient did not take the medication, it will reflect red. *Yah* (yes), if I’m not mistaken.

I: Okay

P: Mmm.

I: So, uhm, the red reflection is moving us to our next point of discussion. So, can you tell us, once you see that it’s red, what do you do, what happens next if it’s red?

P: Okay, the person who’s monitoring the adherence will have to call that particular person and if the person is not responding- is not doing what- then they usually send us as a community health care workers to go and do the house visit, to check whether the person is still available, what’s wrong, why is the person missing the med-, why is the person not taking the medication.

I: So, do you know what happens before uhm, the health care worker or the person monitoring even calls, what happens before they even call?

P: Do uh-I didn’t get the question.

I: So, what happens or how is the patient informed about missing doses before the health care worker even makes a phone call?

P: Yoh, I, I, I don’t know if I get the, I do understand your question.

I: Mmm.

P: But what you are saying is the, the patient, *neh* (right)? Are you talking about the patient?

I: Yes.

P: So, the patient?

I: Misses a dose-

P: Huh.

I: What’s the first thing they get?

P: I think they will get an sms, that will communicate with them to say, “hi Mrs *mang,* *mang* (what, what) you missed your, your medication and what, what from,” *yah* (yes)- from, from, *akere* (isn’t it) it’s the study. So, the people who are monitoring the study and what, what- they’ll usually send an SMS before.

I: Okay

P: *Yah* (yes).

I: So, can you tell us uhm, your role within the differentiated model of care?

P: My roles?

I: Yes, in the differentiated model of care, which is uhh, follow up actions-

P: *Yah* (yes).

I: Who take after a patient misses a dose. So, what’s your role?

P: My role, yoh, is to go and trace that particular person who missed medication, who missed the treatment.

I: Where do you trace these patients?

P: We will- normally we do tracing around Pretoria west and Marabastad, *yah* (yes) all peop-, like all the, the streets in Pretoria west, we do tracing and then Marabastad ,we also do tracing. There are other groups that do tracing at Salvokops, we are, we divided ourselves like according to the areas that we are working at.

I: Okay

P: *Yah* (yes)

I: So ,uhm ,what do you do all the time, is it just a home visit? What else do you do?

P: Okay, our- my job as a community health care workers, we go to household, okay, we do household registration if there’s like, for example, there’s a flat, we will go to that particular flat and then we will do registra- we will register everyone. Then if there’s anyone who need care, like maybe they are taking medication for TB and whatsoever, we will do follow ups, checking whether that person is taking the medication- if that person has been to clinic, if that person is okay or is there any need. We also do refer for social services if someone complains that I don’t have an ID, I don’t have what, what, we also working with-, our job is more of referral-

I: Mmm.

P: To the relevant, to the relevant helper.

I: Okay.

P: Mmm.

I: So, uhm, when you first heard about digital adherence technology, what were your expectations about-before it was implemented here at, at xxx [clinic name], what were your expectation?

P: I did not have any expectation [laugh].

I: So, how did you think it was going to, to, to change the way you do things in TB? Did you think it was going to be easy to use, did you think it was going to make it convenient, did you have any expectations?

P: Oh! Okay, when coming to this, *neh* (right)? Because of- like most of our patients, they are not technologically advanced, some of them don’t have phones, and some of them are living in the streets. So, for us whenever they said this thing is gonna be implemented, it was a little bit difficult that it was gonna exclude them because they did not have cell phones, or had no knowledge about technology.

I: Mmm.

P: Mmm (yes).

I: So, did your expectations change after you started implementing?

P: *Yah* (yes) it changed because of like- it, it covered everyone, whether having a cell phone or not having a cell phone, as long as they explained to that particular person that this is how this box work or these sleeves work.

I: Okay

P: Mmm.

I: So, do you know how many patients maybe you have or the percentage of patients with no cell phones here at xxx [clinic name]?

P: Yoh, okay in terms of percentage, I cannot tell, but there were. Okay, that time when I was working at TB room, I think maybe ten.

I: Okay. So, can you tell me about the training you received, you said you attended at Anew-

P: Mmm (yes).

I: Or on the delivery of digital adherence technology including the differentiated model of care which means the follow ups-

P: Ehh.

I: *Yah* (yes), so what were the activities at the training?

P: Okay, the activities at the traini-, okay the acti-, the activities there were first, they first told us about the consent where they explained that okay, whenever you going to put somebody in this study, that particular person have to be willing, we don’t force, you have to explain all the measures that will be taking, you have to explain every information to the patient. And again, the other activity they trained us on was the box uh, the- they told us about the box, and they told us about the sleeves, and they told us about the ones that carry the uhm, MDR medication.

I: Mmm.

P: *Yah* (yes) that’s the training that we, the activities that we did.

I: Okay, so what was your first impression about the training?

P: (……) Impression [laugh]

I: Mmm, what was your opinion about the whole training session? Did you think it was comprehensive?

P: *Yah* (yes) it was comprehensive because of like whenever you go there as a community health care worker or whenever you going to work at the clinic, you’ve got an information. So, if someone maybe- let me say they did not get it well, you can explain it to that particular person or if I don’t understand, I can also ask the ones that are going to train that, for example the ones that are working with these digital what, what. I can also ask, no, I heard this at the training, so is this true or what. You can also have conversation or you learn more from that particular person.

I: Okay.

P: *Yah* (yes)

I: So, do you think the training was enough for-, sufficient?

P: Mmm, it was enough, but I think maybe they could have done it for two or three days for, like we did it one day.

I: Mmm.

P: And some of us we are not working like every time at the cli-, at the clinic, we-okay by that time I was working at the clinic, *neh* (right)? It was more efficient, for now we are no longer like too much, too much at the clinic, we only come once, only come once at the clinic and I think maybe they, if they could have done it for five days, *yah* (yes).

I: Okay

P: That one was gonna be better.

I: So, what your uhm, your other suggestions to improve the training? You said maybe the duration must be increased from one day-

P: Mmm.

I: To five days. So, do you have other suggestions about the training? Who do you think should attend the training?

P: I think the nurse who are working at the TB room and also the community health care workers because we are the ones who go to, to the people there and I once had a patient *neh* (right)? who, who came and they registered the patient and did not understand about taking medication and opening the box because they not explain to that patient that you take your medication once a day. So, the patient did not understand because medication were big and they were-okay, they wrote three, so the patient was supposed to take three tablets all in once a day, but the patient did not understand. So, the patient was opening, opening in the morning, opening afternoon, opening late. She was opening three times a day and taking like three tablets a day. So, I think if we as a community health care workers are there because we are the ones who go to their houses to, to do the follow ups, to check their adherence and to check whether they do understand about taking the medication, *yah* (yes), I think we should be there whenever they are doing.

I: Okay

P:Mmm.

I: Okay, so how did you end up assisting a patient who was taking medication at different time intervals?

P: Uhm ,okay I did, I did explain to the patient that no, what you are doing, the- your, your, your alarm, it, it, it, it- okay, I did, I ask when it your alarm like when is your alarm set. Okay, she said it’s set at eight o’clock and then I said no, that time at eight o’clock is the time that you need to take your medication. Don’t open it or don’t what, you’ll open it on the next day because if it was like that, *neh* (right)? They could have said eight o’clock, half past one and half past six. So, now because it’s only eight o’clock it means that you must take it only at eight o’clock, don’t ever like open it. Then the patient, *yah* (yes) started understanding and then I did ask whether there was change after because they could even see that no, the patient, the patient is opening the box more frequently. So, they didn’t understand *gore* (that) it’s the patient or maybe the kids are playing with the box or what was happening.

I: Mmm.

P: Mmm (yes).

I: Okay. So, you had to counsel the patient and educate them on how they should take the medication?

P: Mmm (yes).

I: So, how else can you assist patients in terms- for them to take the medication correctly when they are using the digital adherence technology? (……) Is the-

P: I-

I: *Yah* (yes)

P: I don’t know if like it would be impossible like- let me say they have maybe, they, maybe there’s new, they are newly diagnosed, *neh* (right)? Maybe they are newly diagnosed and then they give them the tablets and what, what. Then maybe Friday someone can come, okay, they have maybe like three meetings- maybe let me say from Monday to Friday, *neh* (right)? Maybe two or three tested positive and they are newly diag-, newly, they are newly on this box. They can have a training session where they would meet and then explain to them that okay this is how you gonna take your medication because I know, I know, many people don’t, I don’t, I don’t know they are scared or what. The only time they get to understand how to take their medication, it’s when we go there, we sit with them and explain.

I: Mmm.

P: Some- I don’t know whether they scared or what because once they are told that you are diagnosed with TB, you become scared or what. I don’t know what is happening, so *yah* (yes).

I: Okay. So, you are suggesting it like a mini training session-

P: Yah (yes) maybe-

I: The whole week for newly diagnosed patients?

P: The newly diagnosed, mmm.

I: Okay, good. So, from your perspective as a health care worker uhm, can you tell me the benefits of this box and also the differentiated model of care, the follow ups, what are the benefits?

P: Okay, the benefits, *neh* (right)? For people who are staying, who are homeless.

I: Mmm.

P: Okay ,for people who are, who are homeless it helps because -I remember one day when we went to check someone. It was raining by that time, okay it’s rained and we went the other day to see this person. This person told us that you can see my things are warm- are wet, but here’s the medication, the medication was in the box. So, it, it, it protects the medication from anything. Even if like there’s fire, I don’t know about the fire because I’ve never experience someone who explain to me that no there was a fire and my medication got burned, even to get lost, you know these ones, the, the, the pyridoxin one, they, they can get just, they can, you can lose them anytime. But if they are in the box, whenever they fall, they’ll fall in the box, and you’ll know that the medication is in the box.

I: Okay. So, what are the other benefits of the digital adherence technology? You’ve mentioned uhm, it keeps the medication safe.

P: Mmm.

I: What else is a benefit?

P: Okay, it reminds the patient. It’s making, make- it keeps the adherence, it improves the adherence because whenever a patient- you’ll think oh, half past eight and then your medication is like, and when you don’t have a cell phone it’s an advantage to you because of that tab-, that box will remind you to take your medication.

I: Okay

P: Mmm.

I: And then from the perspective of the health care worker now, you’ve mentioned how it benefits the patient and how does this benefit the health care worker?

P: The health care worker uhm, okay the health care worker- it will benefits them by not being a- like distribution of medication regularly because you’ll patients saying, “no my, my medication was stolen, my medication is lost.” So, you won’t have to be writing that I gave this person medication on Monday and Tuesday told me the medication is lost, so it reduced those levels of medication being lost.

I: Okay

P: Mmm.

I: And in terms of uhm, monitoring, what can you comment on monitoring?

P: Oh, on monitoring, okay, on monitoring also it helps the health care workers to see whether the patient is really talking the medication or what and also the results will tell because you cannot say the box- it’s gonna tell all the information because of you can think that the patient is taking the medication whereas he’s not taking the medication, but using that you, you know that the person cannot open the box and leave it like that, you have to open a box and also take that tablets or medication.

I: Okay

P: *Yah* (yes) and also with the SMS one, I think that one it also help because whenever someone sends you an sms, you are aware that okay this person took the medication.

I: Mmm.

P: Mmm (yes).

I: And then uhm, the benefits of the differentiated care, the follow ups uhh, phone calls and home visits, what are the benefits of those?

P: Okay uhh, I don’t know how to, I don’t know how to explain this, but it helps, it, it, to see the adherence-

I: Mmm.

P: Improvement of adherence because you can see that this person is taking medication.

I: Okay

P: Mmm.

I: And in terms of the relationship between the patient and the health care provider-

P: Ehh.

I: Can you comment on that, that relation to the digital adherence technology?

P: The relationship how?

I: How maybe the TB nurse or you as community health care worker relate to the patient, has it changed with the introduction of the digital adherence technology?

P: Uhh, I don’t have no comments [laugh].

I: Okay. Can you tell me uhm , the challenges of uh, the differentiated model of care first, *yah* (yes) can you comment on the challenge of differentiated model of care?

P: Okay, the challenge *neh* (right) is one that I explained that the patient will open the box, open the box, open the box, so it creates a convictio- confusion to us, you don’t know whether the patient is taking medication or what is happening.

I: Mmm.

P: Mmm (yes).

I: And in terms of the follow ups, what is the challenges?

P: Normally, when it comes to follow up *neh* (right) uhm, okay for the, for the ones that are staying on the streets and then that person- we are- okay the time that I was working at the clinic, *neh* (right), I use, I, I was working at TB, now I’m still working at the clinic. The time I was working at TB I wanted to know where the person was staying, is staying so that whenever we do tracing and I’ll know where I go to because someone will say I stay at Marabastad. Marabastad is big, but whenever that person give us an address and we know it’s next to what because someone who’s staying on the streets, you cannot say this is the address number. So, but whenever we have the, the street, okay the, the next *keing* [what] the trade, is it the trademark *neh* (right)? *Yah* (yes) whenever we have the place where that person will, we will get- it was easy for us to do follow ups, but if we don’t know where the person is, just the whole of Marabastad, what, for, for, for flat Marabastad, there are many flats at Marabastad. So, that is our challenge when it comes to tracing or when it comes to do the follow ups. But whenever the, that person is adhering to clinic, so I don’t think it’s going to be a problem.

I: Okay

P: Mmm (yes).

I: And in terms of the phone calls, what are the challenges?

P: Okay, the phone calls normally, people the give wrong numbers, they do give wrong numbers.

I: And how, how can that be improved, how can we resolve that?

P: That one to resolve it, we need to call whether whenever the, we are registering you and you say “my number is this” we have to call it make sure that it’s working. And we also ask for the alternative or we al-, maybe we have three alternatives because of if you write one, what if that person is not the correct person, so there’s no way that you give us three wrong numbers.

I: Have you tried all those methods before?

P: Yes, the time we were working, *yah* (yes) that time we used to do it with xxx [colleague].

I: Did it work?

P: Yes, it worked.

I: Okay.

P: It worked, but some they come with the, without- okay, someone come say, “I don’t have a phone, or I don’t have, I don’t know anyone’s number,” then that, that was our challenge there.

I: Okay

P: Mmm.

I: And then in, in, in terms of uhm, home visits, right, according to differentiated care, we supposed to do a home visit after three or four days.

P: Mmm (yes).

I: Is it possible for that to happen here at this clinic? How often do you do the home visits if the patient is missing doses?

P: Yoh that, okay that one, *neh* (right)? Is difficult because we are not focusing on home visits only, there are sometimes where we are having the campaigns where will be giving the vitamins or where we’ll be working at the COVID. So, okay that, that time remember we were working at covid station, so it was difficult for us to do the home visit because of the patient will miss for a week then we will come maybe the other week and already it’s how many days, it's five days and when we go maybe we find the patient is not there or the patient it’s, *yah* (yes).

I: So, how can that challenge be resolved of not having staff available to go as soon as possible?

P: By taking there, by making sure that we are taking three numbers and one that, when that person missed, we call those two and then we ask them to tell that person to come to the clinic and if there’s no third, maybe they should hire or employ two who will be specifically focusing on tracing.

I: Okay

P: Mmm.

I: And Uh, in terms of the challenges with the box itself now, you’ve explained challenges with the differentiated care, the follow ups, the phone calls, and the home visits. So, what are the challenges with the use of the box itself?

P: The challenges uh, the, the box does not have any challenge.

I: Okay.

P: Because it helps keeping the medication safe and it helps improving the adherence and then *yah* [yes]. I don’t think there’s any challenge about it, unless maybe the battery, but I’ve never had anyone complaining about the battery or what so ever because- I think they did explain to them that the battery will take, they’ll, it will take six months or eight months so it takes long, it, it, *yah* (yes) and it’s not malfunctioning, it’s functioning well, from the people that I used to go to, this is what they told me, I don’t know whether they lying or what.

I: Okay. And in terms of acceptability, do you have any patients who refused to take the box for any reason?

P: *Yah* (yes), there was one who refused to take the box.

I: *Yah* (yes), what were their reasons?

P: It- okay the patient was saying like where he’s staying it’s not safe, that was the reason and then like we didn’t want, want to go deeper to what why was not safe, you can see the person was not willing to, to take this because. I remember I was working with, I forgot the name the guy-, I forgot the name of the guy. The first day- the first guy who came not- not the second one, this one is the third one *akere* [isn’t it] the first one who came, okay we did explain to him that these are the, the benefits of its, this one, and he was like no *ke* sharp [I’m all good] you can see that this person is not willing even if we try to explain, even if we try to tell that we can see many people are taking the box, for your own safety, the person was not willing.

I: Okay. And uh, do you have any report about stigma in relation to the box, have you had anything about that?

P: For now, many people did not understand about the box because they usually get it at TB room and we know that TB is, okay the TB room is where people can come and talk about these. So, people did not know about the box. Stigma, it was not that much because they don’t know what is it all about. Maybe they-some people just think- maybe it’s something, they don’t know about this box.

I: Mmm.

P: Mmm.

I: So, do you have any patients who have re-, re-, reported uhm , an incident of stigma when they are using the box in your experience of-

P: No, non, non.

I: Okay. And you mentioned that you have uh, homeless people, do you have any other group, special group of people or patients who are a challenge to support using the digital adherence technology?

P: No, many were, were like people were, were complementing it, like yoh guys this box *neh* (right)? I, I, I forgot to sta-, to set my alarm, but it reminded me that yoh girl you have to take your medication. So, I didn’t have any negative information about the box the time I was working there.

I: Okay, so can you reflect maybe on a patient and let me know their experience of the box, the patient, maybe you conducted a home visit on-

P: Mmm.

I: And Uh, let us know what the experience was?

P: The expe-, okay the patient the, *yah* (yes) [laugh]

I: Have you visited a patient-

P: Yes

I: Who is, who was using the box?

P: Yes

I: Yes, what was the experience, what was their story?

P: Pa-, okay the story it was that that I told you about the one who open the box did not understand taking medication correct until we explain. Okay she told me that oh, this box is clever. I didn’t understand why she said the box is clever because she said at eight o’clock it rings, “but I did not understand that it’s the time that I should take my medication.”

I: Mmm.

P: And I say no, the time that the box the alarm rings, you have to take your medication.

I: Okay.

P: Mmm.

I: So, for your, from your perspective as a health care worker, do you think TB treatment can be improved using this digital adherence technology?

P: Yes, I do think so.

I: How so?

P: Because of it- like it reduce these levels of people forgetting their medication and remember TB you have to ta-, whenever you said eight o’clock you must make sure that every eight o’clock you are taking your medication every day. So, that, whenever that alarm rings and then you know that it’s eight o’clock you have to take your medication. And it also remind you to go and take the medication because of you’ll remember it whenever, you be surprised that okay the light here is green light, here is orange light, here is what, what, what. And they will usually explain to you that this light means this and this light means that, okay you’ll see oh okay today I have to go to the clinic and take my medication.

I: Okay

P: Mmm and you call, and, and health care worker can monitor it because of like whenever the patient is opening the box, it will reflects on the tablet that the patient is taking, unlike the card, the card the patient can’t say oh, I’m going to the clinic today [inaudible segment]

I: Mmm.

P: Without anyone monitoring it.

I: Mmm.

P: Mmm.

I: So, can you tell me the difference in terms of TB treatment uhh, even the monitoring before this digital adherence technology, you mentioned that you were also responsible for DOT, right?

P: Ehh.

I: So, can you compare the two systems DOT and use of digital adherence technology?

P: Okay, it reduced like, some people are working, remember that you are working and then you have to come to the clinic every day, so it will lead you, to lead, it will result in losing your job because of you have to come to clinic every day which some bosses won’t tolerate. So, by having that box, you just know that it’s your turn, you take your medication and then you sit at home.

I: Okay, so can you tell me the positive changes of the differentiated model of care and the box, right? Thinking of those positive changes, what are them?

P: Okay, it reduced the, the, okay people like, being people [inaudible segment] what can I say uh, over population. People, like having more people coming here at the clinic like weekly so but, my, it reduced those kinds of things. I don’t know how to answer this question [laugh] let me not lie.

I: Okay. So, okay thinking of all the positive things you’ve mentioned about uhm, the box and the differentiated model of care, the follow ups, what should be improved at the clinic level for us to, for it to be sustained?

P: I think maybe they should, like the health promoters are the ones who give health promotion, they should tell the people about it, and they should not only- they should not uh, be for TB only, maybe it can be used to other conditions like for chronic, so that people can know about it. So, that people cannot have that stigma and say, “oh, no the box is for TB only.”

I: Mmm.

P: Everyone who’s having like, who’s taking too much medication, you know there are some people who they give them too much, too much medication, he’s taking the one for BP, he’s taking the one for, for, for sugar, he’s taking the one for, like different types of medication. So, if they can give that person that box to reduce like that person having too much medication to walk around with.

I: Ehh.

P: *Yah* (yes) that can be done.

I: Okay, so you, you mentioned Uhm, the patient uhh-okay, can you let me know the negative changes that have been brought by the box and how they can be addressed?

P: I don’t-

I: Something negative you can think of.

P: Uh, I don’t know anything negative-

I: You mentioned Uhm ,patient who was taking medication at different time intervals.

P: Ehh.

I: How can that be addressed?

P: I did say that there should be a training maybe Friday where the newly diagnosed are gonna be trained-

I: Mmm.

P: And they, okay to remember like when a health care worker is explaining to you, is, it becomes more difficult, but whenever you are in a group, whenever you walking together, you can ask oh, what did she say when it’s coming to, and then you explain no she said this and this and this. Then it will improve the knowledge.

I: Okay, so you encourage group training?

P: *Yah* [yes]

I: Not individual one?

P: Yes. And also, like maybe community health care workers they should, they should be trained about this box because of they are the ones who going to, who going door to door even the health care workers or *yah* (yes).

I: Okay. So, can you tell us uhm system levels structures that need to be improved for us to integrate the box and the differentiated care into the existing TB programme, right?

P: Mmm.

I: So, in summary I’m asking what needs to be there, what do you think needs to be there for this programme to be part of department of health in the absence of xxx (organisation name) think of us gone-

P: Mmm.

I: And what is needed for the department to continue (……) with Uh, use of the box?

P: Implementation of all levels like everyone who is taking medication they can then be given the box because this will reduce the level of people defaulting and what, what-

I: Ehh.

P: So, whenever if you are having that maybe that box it help you to remember ooh, I need to go to clinic I need to go to this and then okay I need to send an sms and then the safety of your medication and then even if like someone they can say like they hijacked me, they can’t take a box without knowing what is that, maybe they can give you box and say go and drink your medication-

I: Mmm.

P: Because of whenever someone takes the bag and-

I: Ehh.

P: *Yah* (yes) [laugh].

I: So, okay what is needed for this project to, to continue?

P: What is needed?

I: Yes, what are the resources needed required?

P: I don’t know

I: To in order to implement or to roll out or to scale up these boxes?

P: Yoh I, I don’t, I don’t know how to answer that question.

I: Okay

P: Mmm.

I: So, in order to support the patient with digital adherence technology what are the resources required?

P: Cell phone

I: Cell phone and what else? From, from the health care worker side from the clinic side what is needed?

P: I think it’s gonna be the cell phone.

I: The cell phone?

P: *Yah* (yes)

I: Okay ,so what do they use the cell phone for?

P: To keep the information of that particular person for tracing and follow ups, mmm.

I: What else?

P: The box will be needed also.

I: Yes

P: Because of the health care worker have to give the box.

I: Yes

P: Mmm.

I: And what, what happens to the box before it given to, to the patient?

P: Yoh [laugh] I forgot [laugh] Yoh no comment, I don’t know what to say.

I: Okay, you understand that the box has to be prepared first before you give the patient, for example charging?

P: Ehh.

I: Yes. So, what are your comments on preparation of boxes uhm, in the absence of uh, an organisation like xxxx (organisation name), who should be doing that?

P: Because now xxx (organisation name) is doing it, so I don’t know when it comes to the levels of government being involved or what I don’t know the procedure how it gonna be done, I don’t know.

I: Okay, uh, alright. So, uhm ,can you let us know what systems are in place to monitor the use of the differentiated care and also the, the box. Are you having a place where you record any issues with the, the technology or the, the follow ups? Let’s say you don’t find a patient uhm ,do you record that somewhere, what are systems you are using?

P: Okay, whenever, okay whenever the facility gave us uh, tra- a tracing list we go and we will write and we give it to them- the Wits are the one who are responsible for wri- for, for capturing the information that we gave. The reporting that we gave and we also, okay the time that I was doing the file I would usually write okay on this date, on this date we went, *yah* (yes), I will, I will write on the files that on this date we went to do tracing on this particular person and this is the information that we obtained from her family or from her.

I: Okay

P: Mmm.

I: So, do you use any system to capture it or it’s just manually in the file?

P: I know of manually in the file, not, not more.

I: Okay, can you tell us any gaps which currently existing with this DAT intervention (……) any short falls that are existing, any gaps, what you think can be improved?

P: Uhh, I don’t think there’s any gaps.

I: Uh, can you think of maybe the box itself, how it looks, do you have any suggestions?

P: Okay-

I: Of how it can be changed?

P: Oh, I think that it can be changed like, like inviting, like okay *akere* (isn’t it) the box it’s, it’s for, it’s a, like a square, maybe they can put lines maybe to, maybe they will implement it for other chronic users maybe they can put like one line, two-line, three lines, like that.

I: Okay

P: Mmm.

I: And then in terms of the, the platform, have you used the platform before to monitor a patient? Have you-

P: No, I’ve never used a platform.

I: Okay, have you seen maybe the TB nurse using it?

P: Yes, I’ve seen the guy, *yah* (yes).

I: Okay

P: The intern.

I: Do you have any comments on how the platform can be improved?

P: No, not really. I don’t have any.

I: Okay. So, do you have any final comments about uh, what digital, digital adherence technology uhm, as a whole from the use of the box itself and also the follow ups that come along with it, what are your final comments on that?

P: Okay, *nna* (me) I would say it came with the benefits, it impacts especially to, to the homeless people because homeless people are the ones with story, you’ll, you will, they’ll come to you and say, “no they take my medication and this, this, this, and that and then but at first the box was there. Most of them they were able to complete their medication without like coming more regularly because we will say where’s your box and then they will even, some would come with their boxes every day to refill, so it, it came with a positive change (……) *yah* [yes] it came with positive impacts, positive results, monitoring of adherence, *yah* [yes]

I: And in terms of these homeless patients uh ,have any reported that the box was stolen or missing?

P: *Yah* [yes] there was one uh, I don’t know it, I, I, I don’t remember the story quite, but there was an issue with the box.

I: Mmm.

P: *Yah* [yes]

I: So, it sounds as if there’s a lot of these homeless patients, do you know maybe a percentage or an estimate of, you know how many hhhhh homeless patients or the percentage of homeless patients you have here, TB patients you have at Folang

P: Okay, okay, for homeless I can say maybe it’s five, but the people who are staying at shelter, remember at the shelter, people have movements there, today you’ll find that your space has been taken by someone. So, that the reason we are saying they are homeless because they are living in a wall where there’s no like your, your, your, your stuff can be misplaced anytime uh, fifteen percent.

I: Fifty?

P: Fifteen not fifty

I: 15?

P: Yes, 15

I: So, you saying 15 percent of uh, the patients-

P: In the shelters and then five percent maybe for patients

I: So, about 20 percent don’t have a stable-

P: *Yah* (yes) they don’t have a stable, a stable.

I: Okay, alright. Thank you very much, we’ve reached the, the end of our interview. The time is 11:19.

P: Okay.
