# Supplementary material for: Acceptability of Digital Adherence Technologies to support people with drug-susceptible TB in South Africa
Source: PLoS One. 2025 Sep 24;20(9):e0332103. doi: 10.1371/journal.pone.0332103 (PMC12459780; doi:10.1371/journal.pone.0332103)
Supplement: S4 File — (ZIP) [file pone.0332103.s004.zip › S4 Transcripts/HCWs and Stakeholders/IDI 9- HCW.docx]

**TRANSCRIPTION NOTATIONS**

| **Label Key** | **Meaning** |
| --- | --- |
| **I** | Start of each new utterance by the Interviewer |
| **P** | Start of each new utterance by the Participant |
| **N** | Note taker |
| **{ }** | Indicates that details were changed or pseudonyms were used to anonymise data |
| **( )** | Indicates the description provided to anonymise data |
| **XXX** | Words were omitted to anonymise data |
| **-** | Breaking into a sentence by the next speaker |
| **…** | Pause or drawn out words |
| **[ ]** | Indicates noise made, e.g. [laugh], [sigh], [pause] |
| ? | Beginning of utterance by unidentified speaker or questionable text |
| **[inaudible segment]** | Unclear section of the recording |

I: Do you agree to be audio recorded?

P: Yes, I do agree.

I: Ok thank you, Mmm date of IDI xxxx (interview date), location for xxx [clinic name] language English, PID of a participant xxxx. The time at which the session started is 11:53 AM. Huh what was your title of your previous position?

P: Huh I was working as a research assistant.

I: Ok and how long did you work as a research assistant?

P: I worked for xxxx (duration).

I: Ok xxxx (number of months)?

P: xxx months, yes, xxxx months.

I: Ok and when it comes to patient care and counseling huh-

P: Yes.

I: What was or what were your roles and responsibilities when it comes to counseling patients, diagnosing and following up upon their missed doses?

P: Ok so first I was making sure that the patient clearly understands what the diagnoses is, how do you take the medication when do they take the medication, so I did all of that then.

I: Huh were you involved in counselling some of the patients that needed counseling?

P: Yes, I was.

I: Ok and how was the experience- can you share maybe if you can reflect on one patient that you counselled? How, was it? What was the problem with the patient?

P: Ok so I remember counselling this patient so like he did not understand anything about or what he was diagnosed with. So, I had to I had to clearly explain and outline everything.

I: Ok and do you think it worked?

P: Yes, it worked.

I: Ok, huh I would like you to tell me what you know about ASCENT huh let's say if you were to explain to someone who doesn’t know anything about ASCENT you know they hear the word ASCENT project or ASCENT study. Huh and they don't know what is it, what can you tell this person?

P: So let me firstly say ASCENT stands for Adherence Support, Coalition to End TB. So, what ASCENT does they patients with the digital adherence technology. They are looking for ways to improve how patients can adhere to their medication full time using technology. I think that's what ASCENT is.

I: Ok and then what is this technology that you are talking about?

P: DAT, ok, I'm talking about the smart pill box. So the smart pill box organized medication for TB patients, it sent – it alerts for refills and, and dosage. I think it sends alerts for dosage and refill, then it organizes the medication especially for TB patients.

I: And you were talking about this DAT which in your facility you were implementing the box-

P: Yes.

I: I understand that there is an App that works together with the box right?

P: Yes.

I: The xxxx (adherence platform), can you tell me a bit about this App. What is it? How does it work?

P: Ok, so the xxxx App (adherence platfom) is where we monitor the patient on a daily basis because when a patient or when a patient agrees to join the study, we then register the patient, we add his details. So, there is a calendar which we monitor everything in the morning to see that a patient has missed or he did take the medication. There is a task list there. So, the task list could show me if a patient missed yesterday, if a patient is due for sputum, if huh the box was unable to connect maybe for two months and then mmm there's a communication if a patient missed the dose. Then there's an SMS that will, that will be sent to the patient automatically.

I: Ok and then the phone calls- I mean how do you follow up on patients who have missed doses?

P: Ok so first we register a patient, we take a patient number. So, when a patient missed, then I go to the App and I dial the patient’s number.

I: Ok did you successfully reach the patients most of the times when you were calling them when they missed a dose?

P: Yes, huh I did.

I: Most of them? You never had issues with wrong cellphone numbers?

P: Mmm, Mmm [No]

I: Going to voicemail.

P: Mmm I think some they did not have a cellphone. They were using next of kin cellphone number, so it was a problem when I am trying to reach them.

I: OK and then what did you do in that situation? How did you solve-

P: In that situation then I will wait for the patient. If their return date is close, then I will wait for the patient to come to the clinic, but if the patient maybe huh like huh patient is coming next month. Then I will talk to one of the tracers to do home visits.

I: Ok huh you were not physically involved in home visit?

P: No.

I: Someone else or other teams were doing home visits?

P: We had three teams XXX [area name] what, what, what were doing the home visits us we just refer.

I: Ok and then what were some of the success or challenges?

P: Most of the patients we had were living in the shelter house and the flats .So, let's say maybe the patient moved out of the flats, then I think that was a challenge. And the patients who lived at the shelter sometimes were unreachable because they are moving from one place to another.

I: So basically, they were changing the places- Ok in the future what do you think can be done to overcome that issue of wrong addresses and all that, you know, people moving from one place to another, you know, what can be done in order to, to have access or to be able to trace these people regardless of their new addresses and all that.

P: You know what I was thinking if we maybe if we put a tracker in the DAT ( digital adherence technology) that will help or when a patient comes to the clinic regularly then they must confirm the address if the patient is still living in that address but I think DAT having a tracker it will be the best.

I: Ok please describe your role with the differentiated model of care intervention we already talked about huh task list, right?

P: We did.

I: Huh and then you said task list helps you to see patient who have missed doses-

P: Who have missed doses, sputum due for those who didn’t like and who didn’t use box for two months.

I: Ok huh and then you said you would call them-

P: Huh.

I: After you saw that they've missed doses?

P: After, after they missed one dose I called.

I: Ok and then if it's more than one doses you reach out to-

P: To the physical tracers huh, yes.

I: Ok now what I want to understand is-

P: Yes.

I: Is which one did you do more frequently between phoning patients and referring to physical tracers?

P: Phoning patients, I did that a lot.

I: Ok and then since you were working huh there in the TB room together with other healthcare workers-

P: Yes.

I: Right, how were the responsibilities and duties shared amongst everyone in the room between you and other huh-

P: Healthcare workers.

I: Healthcare workers, yes.

P: Mmm I think huh we were working together because we shared some duties like collecting sputum, counselling patients huh (.)

I: Ok so when you first heard about the DAT, the box- in your case since you said in your facility you were implementing the box, right?

P: Yes.

I: I want to know what were your expectations before you started implementing it or you started working giving this box to patients. The first time you heard about it what, what was your first impression?

P: Huh.

I: What did you think about it?

P: I found it amazing. I thought it was going to be very, very useful to the TB patients because some don't have phones to set alarms on phone. Some stay alone. So, there's no one to remind them to take medication. So, I thought the box is very convenient.

I: Ok and did your opinion change after implementing it, you said you thought it was going to be helpful .Did you find it helpful then when you were actually implementing or working with this box

P: It was very, very helpful, very, very helpful.

I: Ok huh did you receive training before you started working or implementing?

P: Yes, I did receive training.

I: Uh where were you trained?

P: Huh I was trained at huh xxxx [area]

I: Ok and who trained you?

P: Mmm, mmm huh…

I: Do you still remember?

P: I, I don't remember.

I: Ok, it's ok.

P: Huh.

I: Ok and did your opinion change after implementing it, you said you thought it was going to be helpful .Did you find it helpful then when you were actually implementing or working with this box

P: The training was good but after the training, I thought it was going to be difficult to use the box. Then when I started working with the box everything was well.

I: Ok and the training- did you think it was enough; the information that you guys’ huh- that they provided you guys with. Do you think it was sufficient?

P: Yes it was sufficient, it because huh I remembered what we were trained on at xxxx [area name] and at xxx [hotel name] so it was sufficient.

I: Ok and how long did this training take?

P: I think it took two weeks, two weeks.

I: Ok and do you think two weeks is enough or you think they should add more days?

P: No two weeks is sufficient.

I: You already said that huh you thought it was going to be difficult- a bit difficult to use the box after the training when you started working with this box you realized that no, you know.

P: Yes, it was easy very, very easy.

I: Ok huh, do you have any suggestions on how the training could be improved, that maybe these things need to be improved?

P: Mmm I think the training was fine on my side huh.

I: Ok, ok in future, who do you think should attend since this DAT is being implemented in a facility where there is you guys as research staff and there are other healthcare workers whom you are working together with. Who do you think should attend this kind of training?

P: Ok I think the community healthcare workers should attend, facility managers should also be there and the pharmacist. I think they need to attend.

I: And how long should these training last -should it be one day, a week?

P: A week I think, a week.

I: Ok and then what content huh do you think should be provided during the training, to those who will be attending this training, what should they be taught on?

P: Mmm ok, I think they should be taught about the task list and on how to initiate a patient huh and then the other thing is the support system where you make the phone calls and where you refer the patient to the physical tracers. I think that's what's the most important.

I: Ok from your perspective can you describe the benefits of the differentiated model of care , the phone calls, home visits and those messages that are being sent to patients .From your understanding what are the benefits of the differentiated model of care?

P: Ok I think mmm , I think it's easier to trace the patient sooner than waiting for the for the patient’s next visit dates because if a patient missed one dose, you call mmm how can I put it let's say a patient is supposed to come next month and then he or she missed the dose. So, I call that patient and it's easier for us, it's easier for us to know if the patient is defaulting than to wait for the next visit date.

I: Ok and talking about the box now, how then do you think the box is beneficial to, to patients?

P: So, I think the box is really helping the patient because with the box being implemented we are having less people who are lost to follow up because we have the box and it's easier to see if the patient has defaulted. So, I think the box has, has really helped patients a lot. Another thing some patients don’t use cellphones, so they don't own cell phone, they live alone so it's easier for the box to remind them to take their medication. Some don't have calendars for refills because normally they refill after one month so they have to visit the clinic and that's where the box comes in. It notifies the patient go to the clinic to take the medication and it keeps medication. So, I think the box is very helpful to the patient.

I: Ok huh when we were talking about the box before you said something about the lights reminding patients. So what I want to know now is how else does this box reminds patient?

P: Ok so the box has got three lights the first one is green, its an alarm for patients to take the medication, let's say when the patient says huh he is going to take medication around eight. Then the box is going to ring an alarm around eight, then there's going to be a green light, then there's an orange light for refill mmm when the patient is going to refill the light shows a day before let's say patient is going to the clinic huh tomorrow, today it will show that tomorrow they're supposed to go to the clinic. Then, there there's a red light, red is for the battery.

I: Ok huh now when it comes to the relationship between the healthcare workers or the nurses and patients-

P: Yes.

I: TB patients, do you think the DAT improves the relationship between the two?

P: Yes, I think so, I think their relationship has improved a lot a lot because the box requires a relationship between patient, community healthcare worker and a nurse. So, yes the relationship has improved a lot.

I: Can you maybe of a patient whom you can say after receiving a box their relationship with the healthcare worker has really improved, there were some positive changes perhaps the adherence improved, can you think of one

P: Ok I know someone. Ok so before the box was introduced this patient was not taking his medication especially on weekends, so when the box was introduced we could see that this patient is not taking his medication on weekends and it's becoming a routine now. So we talked to that patient and then we sat him down and showed him. He was happy, and he started to adhere to his medication. The relationship between the patient and the and the healthcare workers was not nice before but when we showed the patient what his fault was everything started to be ok.

I: Ok huh can you now describe the challenges huh of the differentiated model of care as we have talked about the phone calls, home visits, where there any challenges?

P: Yes there were some patients were not comfortable with getting the messages and then some don’t like phone calls when I call, “hello I am XXX [participant’s name] from xxx [clinic name]” huh they don't like that. I think those were the challenges.

I: Ok and what were some of the reasons for them not to like the phone calls?

P: Some were not disclosing their status to their partners or family. So, they wanted to keep it a secret because if maybe they're sharing a cellphone or if the message, says "don't forget to take your medication” their partner will see, then there will be a problem.

I: And why do you think they hide their status from their partners or loved ones?

P: Huh maybe-

I: What are they afraid of for not sharing their status, what could be their reason?

P: It could be that they think their partner would leave them after knowing their status huh maybe the family will panic over such a small thing.

I: Ok and then any challenges with home visits?

P: Mmm with home visits it is the change of address and the place they live, not everyone has access that place.

I: Do you know of a patient maybe who said or who didn't like huh the home visit maybe one of the tracers went to check up on this patient and he expressed his feelings about home visits?

P: No, I don’t know.

I: And then with the box, what are some of the challenges that you can think of?

P: With the box?

I: Yes.

P: Huh-

I: Are there any challenges?

P: Mmm no no but the box is small for someone who takes five tabs. They can’t fit in the box, I think that’s the only challenge with the box.

I: So, you believe they should have a much bigger size?

P: Yes, much bigger size because because let’s say they take five tabs a day. They can't fit all it all in a box. So, you have to give the patient that box and some patients once they have finished the medication in the boxes, they don't take the new packet and put them in the box. So, I think that's the challenge, so if you can get a much bigger box that will be ok.

I: Ok huh so they get the box with some medication inside and some aside?

P: And some on the side.

I: So after they finish the one inside the box, they don't refill?

P: Huh some-

I: They don’t put inside the box?

P: Some don’t refill, some do but that’s the challenge because it not going to be good on adherence.

I: Huh ok-

P: So sometimes you must call the patient just to remind them that “please when the medication is finished, you must take that one on the side and put in the box.”

I: Ok and then were there issues about the network?

P: Mmm they were but maybe, it happened once after four months but once the network is restored, then everything goes back to normal.

I: Ok what would usually happen to the xxxx (adherence platform) App and box if there is an issue with the network, how does the network actually affect the functioning of the box or the App?

P: Mmm so when there are network issues you can’t really login the App, so when you can’t really login in the App then you don’t see patients who have missed dose. So, I think that is the challenge.

I: Ok and then did you have patients who refused the box when you offered them the box and said, “no I don't need this.”

P: Yes, I did have two or three the reason they refused the box was because they are sharing spaces. So, I remember one from the xxx [ college name] who refused the box because she was sharing spaces and it would be inconvenient for others because it ringing titititi ,you see. So the only reason I had was sharing spaces.

I: Ok so they refused the box because it going to disturb others.

P: Yes.

I: Ok huh and were there any cases of stigma reported by those who are using the DAT?

P: No, I don’t remember.

I: Ok and did you have homeless people in your group who were using the DAT?

P: Yes, most of the patients were homeless.

I: The majority?

P: Yes the majority of my patients were homeless.

I: And what are some of the challenges did they have maybe with the box uh since you mentioned the issue sharing space. I understand that people who are living in the shelter normally they are sharing space.

P: So the, the challenge I had with those who are homeless some were living on the streets ,when it's raining it's, it's a problem for them, yes, and those who are living in the shelter it's very, very, very much of a problem because some they don’t like the sound of an alarm so I think that was the challenge, the sound of the alarm. I think people living in the streets when it's raining then the box -the device get water then, it will stop working, I remember changing it because it stopped working because like it rained and was damaged.

I: It was affected by the water?

P: Yes, yes.

I: Ok and then huh were you able to help this patient and maybe offer them another box?

P: Yes, huh not really a box but changed the device.

I: Huh yes, ok, ok and then it was fine.

P: They were fine.

I: Ok no one who has lost the box?

P: Mmm I did have a patient who lost the box, she was hijacked, that how she lost the box.

I: Huh ok.

P: Then I gave her another box .

I: And do you think there is available sorry there is enough staff to help with the implementation of DAT. For instance, in your clinic, do you think the staff was sufficient to help with the implementation?

P: Huh I think the stuff was sufficient because there was a nurse, then the staff.

I: Ok from your perspective.

P: Yes.

I: Can TB treatment be improved using differentiated model of care and the DAT?

P: Mmm.

I: Like adherence, can adherence be improved?

P: Yes, I think adherence can be improved because what's important is the patient adhering to medication for nine months or six months.

I: Ok and then when it comes to the work overload, does it help with work overload on healthcare workers and you guys as the research staff?

P: Yes because - I think it much easier to communicate with a patient using the DAT, than waiting for the patient to come to the clinic, I think.

I: Ok and the resources, do you think you had enough resources to work and implement the DAT. Did you feel that you had all the resources you needed to work?

P: I had all the resources, but I think space was the problem, space was the problem.

I: So there was not enough space to work?

P: There was no enough space to work on because I had to go outside to talk to the patient about the box so I think space was the problem.

I: Ok so now because after the research phase of the project- this study may be adopted by the Department of Health, so the Department of Health may take over now on the project. So, that the project will continue in the facilities, but it will no longer be the ASCENT team doing it. If staff from DOH [Department of Health] take over, what structures should they put in place in order to ensure that the interventions continue without any complications and challenges whatsoever. What do they need to put in place? What is needed for this since you've been involved and know that this is how its done, they need this. What do you think that the Department of Health needs to have in place in order to ensure that the’ the intervention of DAT continues to run smooth?

P: Huh I think first they have to have enough staff like nurses and community healthcare worker, enough boxes then charge if they run out of batteries and the room for them to work properly.

I: Ok huh and you said some of the challenges with differentiated model of care were that patients were complaining about the phone calls and-

P: Yes, the messages.

I: And the messages, did you have patients who were opening the box without taking medication?

P: Mmm no, no I didn’t have that patient.

I: Huh ok stigma, you said you don’t know any cases of stigma?

P: No, I don’t know any cases.

I: Ok and then now can you describe to me the system that needs to be in place in order to integrate the differentiated model of care together with the DAT [car hoots] in future what are systems that need to be put in place in order to have the system continuously?

P: Systems?

I: Like when it comes to preparing the box because you have to prepare the box right, before you hand them over to patients?

P: Yes.

I: You mentioned charging and all that, who should be responsible for this charging and replacing of the chargers and what not if there is an issue with the module, you mentioned the module inside the box. Who should be responsible?

P: I think I they can get someone for that process but I think if DAT is under government, they were there there's no going to be a need for ICF.

I: If sorry?

P: If the government continues with this DAT, so there won’t be a need for ICF.

I: I think they will take over the project and everything will remain as it is.

P: Mmm.

I: If I am not mistaken.

P: So they need to have someone to read the ICF to the patient. Someone to monitor the xxxx (adherence platform) application, so if we can get someone to do all these things it will be much easier.

I: You think one person can handle all of this?

P: No, two people, I think.

I: Ok, ok, ok (…) ok and then with those you, you mentioned patients who didn't like receiving phone calls and receiving those messages, automated massage, yes, were you capturing all those somewhere that ok patients complained about the phone calls or receiving the SMS, were you capturing some of the challenges that's what I am trying to say?

P: Yes ,I was capturing them on the hub under notes, I would write that patient is uncomfortable getting the messages ,then I switch off the messages. I was capturing under the notes.

I: So there is somewhere where you captured on the App?

P: Yes you can capture when you face challenges under patient profile that's where you get to write everything under the notes.

I: Ok you were the one personally capturing?

P: Yes, I was the one.

I: Ok we are almost done do you think there are any gaps in the intervention or the way the intervention is being delivered like do you think there is something that needs to be improved?

P: On the DAT?

I: Yes, in the way this intervention is delivered. Are there any gaps that you, you know of?

P: Mmm no, I think there are no gaps.

I: So you believe that the way it's being delivered is working?

P: Mmm I think it's working

I: Nothing to be to be improved whatsoever?

P: On the DAT or on the implementation of the box?

I: The implementation I mean maybe with the phone calls do you think the phone calls are enough, the SMS, what can be done maybe even the box itself-

P: On the box?

I: Yes.

P: I think maybe on the box what they can add is maybe different ringtones like the patient will choose. During initiation when registering a patient with the box they should ask whether the patient is pulmonary or extra pulmonary because on the system it's only pulmonary and some patient they are on extra pulmonary so the box will show you that this patient is done with medication whereas they are not, so if on the system there will they be an option to choose whether it's pulmonary or extra pulmonary I think that would be easy. Also putting a tracker on the box would work for homeless people because when we refer homeless people to the physical tracers they can't find them but if there's a tracker somewhere, somehow then that would be much better.

I: Ok now do you have any other comments something that you want to comment on something that maybe I didn't touch on? Something that you wish to speak on or add.

P: Ok so patients were happy about the box, they were all happy. There was this patient everyone in the house was happy you know. When the box goes tititi [beeping sound] children would call “dad, dad it’s time for medication” I think the box is very, very friendly.

I: [breath in] Ok this is the end of our interview thank you so much for your time. If you have any questions, you can contact the people at the back of the ICF, the time is 12:37 PM.
