# Supplementary material for: Acceptability of Digital Adherence Technologies to support people with drug-susceptible TB in South Africa
Source: PLoS One. 2025 Sep 24;20(9):e0332103. doi: 10.1371/journal.pone.0332103 (PMC12459780; doi:10.1371/journal.pone.0332103)
Supplement: S4 File — (ZIP) [file pone.0332103.s004.zip › S4 Transcripts/PwTB/IDI 1-PwTB.docx]

**TRANSCRIPTION NOTATIONS**

| **Label Key** | **Meaning** |
| --- | --- |
| **I** | Start of each new utterance by the Interviewer |
| **P** | Start of each new utterance by the Participant |
| **N** | Note taker |
| **{ }** | Indicates that details were changed or pseudonyms were used to anonymise data |
| **( )** | Indicates the description provided to anonymise data |
| **XXX** | Words were omitted to anonymise data |
| **-** | Breaking into a sentence by the next speaker |
| **…** | Pause or drawn out words |
| **[ ]** | Indicates noise made, e.g. [laugh], [sigh], [pause] |
| ? | Beginning of utterance by unidentified speaker or questionable text |
| **[inaudible segment]** | Unclear section of the recording |

I: Mmm before we start, do you agree that our conversation be audio recorded?

P: Mmm.

I: If your answer is yes, may you please raise your voice so that you can be heard on the audio recording.

P: Yes, I do not have a problem.

I: Ok, thank you. Today is the xxxx (interview date), we are at XXXX clinic, interview will be conducted in setswana, Patient PID: xxxx, Interview start at 10:16 am. Thank you for coming to take part in this interview, before we can start, where do you live?

P: I live in xxxxx (area name),

I: So, where you live, who do you live with?

P: I live with my younger brother; let me just say it only two of us. The rest went back home in the rural.

I: How did you find out that you have TB?

P: When I come here to take my HIV medication, the two sisters said that when I get time, I must come test for TB, and I had TB before. I had it for the second time and I also tested positive for HIV-it is an opportunistic disease, and I was admitted because of it- because I was infected with HIV.

I: When did you get TB for the first time?

P: Around xxx (year).

I: xxx (year)?

P: Mmm.

I: How long did you stay at the hospital when you were admitted?

P: Roughly, about a month but when I add, it is like two months because I was admitted to the other hospital. Then they transferred me to another hospital where I also stayed for a while.

I: For how long did you take your treatment?

P: For about 9 months.

I: And for this one, how long are you taking it for?

P: I have already passed their term.

I: Uh mmm when you find out that you have TB, you came straight to this clinic, or you were transferred from another clinic?

P: I attended this clinic.

I: So, what do you use to come from home to clinic?

P: A car.

I: Your own car?

P: Mmm.

I: So, when you came to the clinic to take your treatment, how long did you take before they explained to you about the stickers?

P: I stayed if I can-oh I started with those stickers when I came.

I: So, when you came, did they give you stickers same time?

P: Yes, they gave me and said this kind of remaining- they said they will remind me, and I will give them the number and it will no longer be in use after a certain time. So, when I saw that this person is no longer giving us the correct information because we have read these things but from this date- until to date, you can send and enter. I saw that they did not understand but then I realized that technology can be confusing sometimes.

I: Mmm can I ask that when you came to the clinic, and they showed you these stickers, who showed you?

P: It is them.

I: Them?

P: It is XXX (RA’s name).

I: Is XXX (RA’s name) a sister or an intern at the clinic? Or those with the red T-shirt.

P: The one we were with?

I: So, is the other one a sister or a nurse?

P: It seems like they work together, their doctor was XXX (doctor’s name).

I: So, how long did they take when they explained to you?

P: She did not take that long with me, and I did not believe what she was saying because I was in denial that technology can really do what she claimed, until they reached the end. It took roughly about 15 minutes to explain to me the stickers.

I: The way they explained to you, did you find it easy to use the stickers when you arrived home?

P: Yes, using them is just like clicking or clocking in of each and every SMS. If I delayed to send an SMS, the alarm on their side reports. Which is also good, and it also reminds you if you forgot time but bad to someone who do not want to be exposed.

I: So, when they explained to you about these stickers, how did you feel that these stickers will remind you to take your medication?

P: How did I feel?

I: Mmm.

P: Uh it felt like these people care to give me- they just want to know how I am coping. How am I coping with these stickers. At some point when I was beginning to use these stickers I made a mistake, I doubled- I drank medication twice and I felt that this thing is so hard but because of the overdose I realize that correct measurements matter. I thought it was just small waters, but I drank them and drank water.

I: Before you received these at the clinic, did you see them somewhere or heard about them before you received them?

P: Not at all, I saw them for the first time at the clinic.

I: So, when you received them, it was the first time you saw them?

P: Mmm.

I: So, the way they explained to you how stickers are and that you are going to SMS at this time and what was happening. Is there anything that you would like to change from the way they provided the information?

P: Pardon?

I: My question is, the way they explain to you about these stickers and then you took them home to use. Can you explain to someone the same way they have explained to you, or you could use your own way since you have observed how they work?

P: Explaining to somebody else?

I: Yes, explain to somebody else

P: Uh I can tell them that this thing is important, it like you have something as a reminder. It is importance even if in your heart, you feel you do not need them, but you just send a message. The worst part if you did not drink the treatment and you just thrown them away, so this reminder is for someone who is determined that this thing must work for them. If you are determined for this thing to work for you, then you will take your treatment and life goes on.

I: What is your experience with the stickers?

P: Experience?

I: Oh, like what is easy about using these stickers.

P: Mmm.

I: Ok, let me try to rephrase it this way. If they give a person sticker right now, by your own observation, is it going to be hard for that person to use these stickers when they arrive at home?

P: It is not difficult as it is not possible for someone else to know the problems of that person, unlike a phone ringing; phone alarm and the next thing people want to know what does it say if the alarm rings ,and you find that the person did not want to disclose. They only wanted to drink them privately.

I: So, for you, is it simple to use them?

P: Yes, for me compared to this one- when I compare, I see this one simple.

I: Since you have been using the stickers, have you encountered problems when you send an SMS, and the SMS do not go through or when you have to send an SMS and the phone is not there?

P: Mmm one time, I thought I was going to call- I mean I am going to send the SMS, then when I arrived home, I found that there was load shedding and the phone battery was depleted. So, it was like I am failing to send the SMS and the other time it was network. You find that the message went through but takes a while to report to me that the message has been sent.

I: Are you working

P: Yes, I am working

I:What time do you drink your treatment when you are working?

P: Mmm I was drinking it a couple of times; it was not this one which I drink once because there was a time I was drinking at different times and there was a time I only drank once.

I: So, what about the time you are working? What time are you drinking your medication?

P: I was drinking the other ones before I sleep and then drink the other ones in the morning. I would then package them as lunch so that I can take them during the day at work.

I: So, for you to drink them, what reminds you to drink your medication?

P: I know how many they are when they give me and they circle the return date on a card, so that when I have drank a certain amount, I can refer to the date and know that the date is near. Like if they gave me 28 pills, they count 28, so that I won’t end up making a mistakes and want to drink others first and finish them before this one.

I: So, in a day, what time do you drink your medication?

P: I nearly drank them after this one, I would just say if I drank them three times, I try to eat first.

I: So, your time to eat, do you eat at various times in a day?

P: Mmm it is not the same time, at times I do not wish to eat but then I must drink my medication and I do not drink them on an empty stomach. I just try to eat so that I drink them because I have to.

I: So, do you drink them at work?

P: Yes, even at work I drink them.

I: So, when you drink them at work, do not they affect your time of work, or you drink them only when you finished eating at work?

P: I like to drink them when I have finished eating because they are strong, and they can even cause pain if you are hungry.

I: So, do your colleagues know that you take TB treatment?

P: No, I believe only the seniors not the juniors, in fact I am asking myself what kind of TB is this because you can tell by looking at people who have it and see that they are sick but with me, a person can be shocked that I am sick because even now I am sick, but I am not coughing.

I: Since you have been using this sticker or when you started to use these stickers, have you ever been troubled that people will know that you taking TB treatment?

P: I do not really care that they know and if there is someone that I am close to. I have to tell that person like my girlfriend, I told my girlfriend and she did not take it well and I immediately told her that we need to stop kissing then and be like we are no longer connected. It proved to me that once you are exposed to this thing, you will regret disclosing that I am drinking this treatment because somewhere somebody might not accept this, and I was in denial myself. Being in denial made people even at work to say that I do not look well on some days and some would actually say, “these days you are not well”. They said, “we allow you to work but you are not ok” and that is why I took a step to go to the hospital. They said there is water in my heart, then I went for the X-ray- they said there is water in my lungs and that is when they started fighting this disease and I was supposed to join this HIV thing the following day.

I: Since you have started using these stickers, is there someone who have seen you using them?

P: Yes, the people I stay with. They used to help me understand how to text because it was a struggle for me to text a message to 4352 because you would find that I tried to send a message and it did not go through. So, my younger brother was helping me- I could not hide it from them because they are the ones who took care of me.

I: When you found out that you have TB, who is the first person you told?

P: People I am living with.

I: When you told them you were diagnosed with TB for the second time, how did they feel?

P: Even with the symptoms of TB, people cough and whatever, but I do not cough. I only cough when I’m just dry or when I just feel a small pain, then I cough. I can sit with you for an hour, or a day and you find that I have never cough. So, now it is different, and it surprises me, but I just accepted things like this and took the medication.

P: So, is there someone who has TB in your family except you?

P: Yes, in my family-it my older sister. The one I come after, she once had it including the first born. She once had it, so I just tell myself that it in the bloodline because it got me because of HIV.

I: When your older sister and the first born of the family had TB, did they take their treatment?

P: Yes, they took their treatment.

I: Did they finish their treatment?

P: Yes, they did.

I: Did not they have it for the second time

P: No.

I: Where do you put you medication? In your house, where do you put it?

P: At my house, I put it on the headboard.

I: Do you take some when you go to work?

P: There is extra medication that I take, and I keep the other medication that I pack in the car. I am no longer on the ones that I have to send a text message; I now take two and once a day. I have to separate those that I do not drink from the other ones I take before I sleep, so that they do not crush one another if I drink them close. I have to take one during the day and the other one at night.

I: So, when you are using the stickers, what is that thing that you saw as helpful when you take your treatment? When you observe, is it the information that you found about stickers, or you see that it is the reminders that you get to drink the medication?

P: I can say the reminder is helpful when it comes to drinking treatment especially if you forget but if you are just careless and you do not care whether it reminds you and it rings once, and you do not hear it. You will hear a person in denial, you must accept that this is your food of life and your daily food because when the medications start to make you complain to drink. It becomes a problem because in your mind you are complaining a lot saying this medication is a lot and when you start you will find out that they even exceed- they have been, so when you complain seven is too much. You can send your message but if I’m struggling to take my medication I will just finish and send the SMS even though I did not drink my medication. Well, you have to talk to your inner self that this medication I can take it.

I: Since you have received these stickers, have you received a remainder SMS?

P: Yes, many times when I am late to drink my medication on a usual time. I forget but they do not remind you if you drink them on time, In fact they are helpful if you have been busy and lost track of time. So, they are good if you forgot but they no longer send me those.

I: Have you find yourself being called at the clinic when you forget to take your medication or maybe they think that you forgot?

P: Mmm last time I had a problem with my phone it was damaged and I could not send them a message during that week, and they tried to find out how was it going with my medication. I then told them that I had a problem with my phone so I didn’t find a way to send you and message.

I: Oh, was there a time people from the clinic came to visit you at home?

P: I only got a visit from people who said they are from xxxx (organisation name). They are not from the clinic, and I do not know where did they get my information since they came to visit me.

I: So, when they came to visit you, how do you feel about that?

P: Uh sometimes it draws attention to you when they visit.

I: Tell me, how did you feel when these people came?

P: Sometimes it exposes you to the people who do not know your situation because those people were too many when they came and people will start making comments about sick people and say,” whoever is sick” and you see how that thing makes you feel. So, they get their way when you are sick and even if you get close to them, one day you will make a mistake, and you will get sick.

I: May I ask, are you troubled that people may know that you take TB treatment?

P: Mmm if they know, then they know. I am not troubled that much, just that I am worried about them that they do not talk things about me, and you find that they get harmed even, HIV I am not worried about them It’s just that I am agreeing that these symptoms that have condemned me I do not have them.

I: Mmm did you find out perhaps if your culture beliefs are against you taking your medication?

P: Pardon.

I: Mmm did you find out whether your culture can hinder you to take your medication or take treatment or they might take you to a traditional doctor to help you?

P: No, my culture does not hinder me. Yes, I believe in the culture but there are some things they need you to go to the clinic for because they are failing to treat those diseases.

I: Mmm according to you, if they gave the person stickers to use at home and then the person loses the phone. Will that person be able to use those stickers?

P: These stickers?

I: Yes.

P: A person lost the phone?

I: Yes.

P: If this person lost the phone- they just have to memorize the code, the same way I did. I know 4352 by heart, so I can borrow someone’s phone and report, or it will be a wrong number, one that I was using 2352.

I: According to your judgment, did the stickers help you to take your treatment daily without missing a dose?

P: To me- I can say they helped me. I just accepted that I will be taking them as food. Then I would drink them as I eat my food.

I: So, your reminder is that you take your medication after you have eaten?

P: Yes, I can be late, but I know I have taken medication even though I am late about three hours. I just now that I must take my medication and not say because I am late, I will take them tomorrow.

I: Since you have been using these stickers, what is that thing that you would say you are happy about to use the stickers?

P: Mmm the thing that I am happy about is that if I care about my life, you care too. So, it is like if I care about my life, you also care too every day and when I send an SMS to say I have taken my medication, you also respond to me to show that you care.

I: Uh at times when you get an SMS to remind you to drink your treatment, what is that thing you liked about the SMS?

P: Reminding me in case I forget.

I: Is there something you do not like about the SMS?

P: They did not irritate me when I was receiving them, but you’ll find that someone would, say, “they drawing attention to me” but they were not drawing attention to me.

I: Mmm when people from the clinic call you to ask if you took your treatment and maybe they see on their system that you did not take treatment yesterday or did not send an SMS, what is that thing you love about those calls?

P: I just love the fact that they care about me and that when I did not report to them that I failed to send an SMS and they realized it. They came to check up on me and that shows-it makes me feel they care about me.

I: How many times did people from the clinic come to visit you in your house?

P: Only once.

I: They only came once?

P: Yes.

I: So, were you excited to see them when they came to check up on you or was there something that you did not really like, or you did not like the fact that they came to visit you?

P: It’s not that I did not like it when they visit me but sometimes you get bored by someone’s behaviour; the way the person behaves bore you, not that there is something that bores me.

I: When you were diagnosed with TB, did you get counselling from the clinic?

P: I am taking medication for the second time, and I did not receive counselling. I would say I received it from XXX (RA’s name) because I did not believe that I have TB from their first half. Even now when they came to me and said, “try to test because if you once had it, it likely to come back, so it best that you test so that you know your state”. I was struggling with the sputum, but I finally got it tested and I received a call, mind you, I was not coughing by that time, and I did not I could not even produce a sputum but when I finally produced it and brought it to the clinic. They then said I had T and they called me to say the sputum that you gave us to take for tests is back and it says you have TB, but it was for the second time. So, they can tell me, but I knew because I am diagnosed with it for the second time.

I: Did you like the counselling you received?

P: I liked it; it guides me.

I: In between the interventions you received, the stickers, reminder SMS, calls and to be visited at home, which one is more important to a person who takes TB treatment?

P: The important one?

I: Yes, between SMS, calls, and home visit?

P: Mmm home visit- I do not really count it because sometimes you find that I do not have a time, I have to go to work during the day and other times I go in the morning. So, you find that someone want to do a home visit and I am not home. Therefore, the SMS is much better.

I: You find the SMS better?

P: I think between the SMS and a phone call, but I think the SMS is all right because with an SMS, you do not need to recharge airtime but with phone call, one needs to recharge before placing a call.

I: Mmm when you started for the first time- you said you had symptoms of TB when you went to test for the first time?

P: For the first time?

I: Yes.

P: I had a sharp pain, it was like I cannot sit upright because of the pain on my left side and when I arrived, they did a check-up and they told me my heart is inside water. They told me that it survived inside water, and they must drain water. So, they put that thing in my back to drain water, and that is when I started feeling less pain. Then I continued with my medication after they drained water.

I: When you were diagnosed with TB for the second time?

P: For the second time?

I: Yes.

P: For the second time, I just finished drinking my medication and I do not even have three months.

I: Did the people you live with come to test when you found out that you have TB for the second time?

P: People I live with?

I: Yes.

P: The other one came because I used to share a cigarette with him , so he came to test if he has TB because he could also get it.

I: When you were having trouble with your network, did you come to the clinic to explain to them that you are having network problems?

P: No.

I: You did not explain to them?

P: They are the ones that were concerned about me on the following day; on the following day it was like I have failed them the way they thought about it. It was like when you borrow someone something to use and then the person does not use it. Then it gets ruined, and it looks like the person does not care about the thing that was given to him to use.

I: The time you were talking about your girlfriend that you explained to her that you have TB, what was her take?

P: She no longer kisses me.

I: Did she accept that you have TB?

P: Yes, she accepted but she has resentment because it like I will infect her when I come close; it like I will infect her if I try to kiss her.

I: Did she come to test for TB?

P: She came to test after I told her to and found that she is ok but then she was afraid of kissing me.

I: Mmm how do you feel that you have TB for the second time?

P: I would just say TB is a painful, can you believe that it is something that when you have it you can go around infecting people. Worse I cannot stay with kids if I am sick and I will infect other people and that is the painful thing.

I: Mmm when you are done sending an SMS, is there an SMS that you receive?

P: Yes, they respond shortly after I sent an SMS; they respond in less than 5 minutes. I could say and it shows that my message is recorded and well received.

I: Have you ever received an SMS that reads “thank you for taking your medication” after sending an SMS?

P: Yes, that is the one.

I: When you received that SMS after sending your sticker code, how do you feel?

P: I feel that they care about me, and they want to know whether I have eaten. I can see that they care.

I: Have you told your girlfriend that you are using the stickers?

P: No.

I: You have not told her, what is your reason of not telling her?

P: She does not entertain that.

I: According to you, when you look at these stickers, is there something that they can improve or change?

P: Uh up to so far, I do not see what they can take out, it is complete the way it is.

I: It is complete. According to you, who should educate patient or people who take TB treatment about the stickers?

P: A person who should teach them?

I: Yes, a person who can explain to them that here we have such things.

P: The person who used to use stickers is the one who should educate them when they are still at home before they even arrive at the clinic. I have seen it importance when I used it and it reminded me and that is good for somebody that forgets.

I: So, when you look at the stickers, people who used them should explain to other people about them?

P: Yes, I have used them before, so I know how they work and also how they helped me. Without them what could have happened.

I: Ok, can I ask for your last perception about SMS, phone call, and home visits, how do you see them?

P: Mmm when you call me, I may not understand you because sometimes you would call me at the wrong time. Maybe I am on the road, and I might struggle to answer you on time because I am driving. So, you would call me and find that I am driving, and I wished to answer you, but the problem is that you want to speak for a long time, and I cannot while I am driving, and I might not pay attention to what you are saying because my full focus will be on the driving. With home visits, you will also find that when you pay me a visit, I am not at home. So, I see sending an SMS as a more relevant because you receive an SMS reminding you to drink things that we gave you and if I did not drink them. I just send an SMS and your system will also telling you that I have drank the medication.

I: So, what is your perception about the stickers, what can you say about them?

P: The stickers are good, it is like I am sitting next to you every time- it is like I am a person next to you when you sit on the machine, and you receive the SMS. It like when I have taken my treatment for the 115 times, the machine reports to you that 115 so far and when is the medication finishing. It will show you which medication I have taken, and you will know that the medication that we have taken so far has worked and to see that the SMS is still relevant.

I: Ok thank you my brother, the way things look, you have answered all my questions and there is nothing that is left, and I would like to thank you for taking your time to participate in this interview today and if you have questions as I explained to you earlier, you can call those numbers I have highlighted on your consent form and if you want to email them, you can do it. Thank you for your time. So, we have reached the end of this interview, the time is 11:25 AM.
