# Supplementary material for: Acceptability of Digital Adherence Technologies to support people with drug-susceptible TB in South Africa
Source: PLoS One. 2025 Sep 24;20(9):e0332103. doi: 10.1371/journal.pone.0332103 (PMC12459780; doi:10.1371/journal.pone.0332103)
Supplement: S4 File — (ZIP) [file pone.0332103.s004.zip › S4 Transcripts/PwTB/IDI 10_ PwTB.docx]

**TRANSCRIPTION NOTATIONS**

| **Label Key** | **Meaning** |
| --- | --- |
| **I** | Start of each new utterance by the Interviewer |
| **P** | Start of each new utterance by the Participant |
| **N** | Note taker |
| **{ }** | Indicates that details were changed or pseudonyms were used to anonymise data |
| **( )** | Indicates the description provided to anonymise data |
| **XXX** | Words were omitted to anonymise data |
| **-** | Breaking into a sentence by the next speaker |
| **…** | Pause or drawn out words |
| **[ ]** | Indicates noise made, e.g. [laugh], [sigh], [pause] |
| ? | Beginning of utterance by unidentified speaker or questionable text |
| **[inaudible segment]** | Unclear section of the recording |

I: Do you agree to be audio recorded?

P: Yes, I agree to be audio recorded.

I: Ok date is xxxx (interview date), PID is xxx , time is 12:04, location xxx clinic, language used Setswana. In short, could you tell me who you are and where you from?

P: I am XXX, I am from XXX at XXX here at XXX.

I: When you come to the facility do you use transportation, or you walk?

P: I walk when I come to the clinic.

I: You walk?

P: Mmm.

I: Is the distance too far for walking and it requires that you use a taxi?

P: It is a distant and to come here it means-to come here it means I have to take two taxis. I prefer walking but it is still far, cause if I come here by a taxi, it means I have to use R60 because it is R15 to station and then R15 to come here and going back like that, so I prefer walking. It is far but you can still walk.

I: So, how often do you come to the clinic?

P: At the clinic- only ones, mmm only ones.

I: So, can you take us where [cough]- how did you find out about TB- that you have TB? How did you find out?

P: I found out at the clinic that I have TB after I felt that I am not alright; I had chest pain on the side. I then I went to the TB room, and they gave me a container so that I produce a sputum. I coughed and after 3 days that is when XXX called and told me that I have to come to the clinic. When I got here, they told me that I have TB. My real problem was chest pain.

I: Chest pain?

P: Mmm.

I: So, besides chest pain, was not there any signs that would suggests that something is not well on the body?

P: It was only chest pain and tiredness but as for sweating and others-I had no sign that would suggest that I have TB. I would- even coughing, I was not coughing; it was only chest pains.

I: So, how did you feel when you were receiving your results that were positive? How were you feeling?

P: Eish, I was a bit stressed because I am taking many pills, you see, so when they told me about TB, all I could think about was that I am going to add on top of those pills. It never sat well with me; I was stressed, I do not want to lie.

I: But how is it going up to so far since you have started?

P: uh after- I was feeling all right on the second week I started treatment and even the stress was not there anymore. I felt that the pills were not causing any problems and I would just take them, and after two week the pain was gone. That is when I started to feel no pain.

I: So, do you know [paper shuffling] these labels?

P: Yes, I know them.

I: Could you tell us where do you know them from or when did you first see them?

P: I saw them for the first time at the clinic; at TB room. Cause when they gave me pills for the first time, they also gave me these stickers and registered me, and told me that I must send the SMS when I am done taking the pills and I did that.

I: So, you never took the pills- the day they registered you, it is the same day they gave you the sticker?

P: Exactly, the day I came here, and they told me that I have TB. They gave me both treatment and the stickers on the very same day and they even explained to me how they work.

I: Ok, could you tell us how do they work then?

P: These stickers-when you drank your pills you send an SMS and after you just sent the SMS, they also send you an SMS back, thanking you for taking your medication for that day. They remind you to drink your medication because when you do not send, they send you a message late around six reminding you that you have to take your pills. At least the stickers are helping us because it can remind us of and to tell the truth there are a lot of things happening and you find that you forgot to drink your medication because you were busy doing what you were busy with. When you hold your phone, it reminds you and then you remember that I did not drink my pills, let me drink them.

I: Ok, can you tell us when you came-the day you came, and they started you on treatment. Who explained to you about these stickers?

P: It was XXX (intern) who explained these stickers to me; she is the one who explained them to me, and we even completed the forms that we were filling. It was her.

I: So, is there something you would like to change from the way she explained these stickers to you, or you feel like she gave you enough information when she explained them to you?

P: According to me, the information she gave me was all right, but I would have loved if you changed where-When I came here and they gave me the pills, at least they should have asked me the time I am going to drink them. Let say I prefer 10H00, then the reminder should alert me at 10H00, you understand. There should be a reminder at 10H00 on my phone that says it is time to drink your medication.

I: Oh, so as you mention, you should have chosen the time so that it sends you an SMS-

P: -On that time, you understand?

I: I understand, so are there other things? What would you like the SMS to say, when we remind you at 10H00, if we were to edit? What would you like the SMS to say?

P: There is no problem the way it says-the way it reminds you not to forget to take your medication. I do not see any problem.

I: When it reminds you?

P: Yes, when it reminds you at 10H00. When it is 10H00 [cough] even if it is five past or what but as long it is 10H00. It is the way I think I should receive the message that says, “do not forget to drink your medication.”

I: So, you did not want maybe a ringtone or something? You just want to receive the message?

P: The ringtone is the one that is all right because I would know that this certain ringtone is special, it is not just any ringtone, and it is not a message from somewhere but a message for my medication.

I: Mmm so what would it say when you receive it? Should it be a song that you love and that will remind you in a way that you would know that it is time to drink your pills when you receive it?

P: Yes.

I: We thank you; we thank you for the information you just gave us now. So far since you have started using these stickers, would you take us through and share the experience you had, and how the journey was and how was the experience?

P: To tell the truth, it was quite easy because you find that I forgot to drink them as I am busy and obvious the phone is something that you would hold and there is no way you can spend the whole day without touching your phone. So, by the time I hold my phone I find an SMS, then I remember that I did not drink my pills, then I drink them. Some other time I drank them, but I did not send an SMS and when it reminded me, I remembered that ok, I did not send the SMS. Other times you find that I am in a hurry; I am rushing somewhere, and I just say let me eat fast and then take pills, I will send the SMS later but then I forget. However, I send it as soon as it reminds me.

I: So, the method you were using to send an SMS-

P: -It was nice; for me it was nice, and I was enjoying [laugh]- for me it was nice that I even wished that the other treatment had SMSs too so that they remind us since I am not taking TB treatment only. The way I was enjoying, I even asked myself why they are not doing the same for the other treatment so that I can remember. I was enjoying and I am going to miss that [laugh].

I: So, you did not worry when they first gave you these stickers, you did not worry that there might be a problem?

P: There were problems at the beginning, and I would say these people are giving me extra work. When I left at the clinic, I was complaining the whole way saying so much work and all the pills I am supposed drink but as time went by. I realized that these people are helping me, yes, these things are actually helping me. I would tell myself that I will not struggle but I am struggling and sometimes I forget because there are a lot of problems, so I forget but they would remind me.

I: So, you mentioned that at times you will be in a hurry and when you done eating you leave and forget to send the SMS, how often did that happen?

P: Not too aften because I stay in the house; I am always indoors, so it did not happen many times and just like now we do not have electricity in our section and there is no way I can send the SMS now. So, I will charge it later when there is electricity so that it reminds me at night, and I can send it.

I: So when you leave in a hurry and do not send the SMS. You then receive an SMS reminding you to drink your pills because it knows that you did not drink-

P: -Yes, it knows when I did not drink them.

I: “Drink your pills,” how did you feel?

P: I would be disappointed; I would have loved if it can be the same time. When XXX told me that it alerts them, I wanted it to be the same time and not different times like 8,7,6 you understand? I was disappointed- I wanted it to be the same time even if it differs by minutes but not too much. I would be disappointed if it reminds me, and I am thinking why it would remind me. I should have sent it, so that it does not remind me.

I: Was there a time you drank and even sent the SMS but still received an SMS saying that you did not drink your medication?

P: Yes, a lot of time to be exact. It started doing that around February. Yes, it started in February, even when I have sent but after 06H00 it reminds me again and that one was boring me because I have already drunk. I would tell myself that maybe it did not reach them, I did not understand. I even asked XXX because I did not understand whether the SMS reached them or what. It was stressing me because I drank but at 06H00 it would remind me again; I did not understand.

I: So, you mentioned that it was boring you, would you take us through your feelings at that time?

P: Eish just like I am telling you, I felt some type of way and I did not understand. I would tell myself that the SMS did not reach them and probably people at the clinic tell themselves that I do not drink pills. Why should it be like this and tell me that I did not drink. At that time, I wanted to send a message back and tell them that I drank but I did not know what to do or who should I send it to and tell them that I did drink my pills. I would be stressed by those things.

I: Is everything all right now?

P: Yes now that I have completed. I no longer receive the SMSs since I have completed but to be honest the stickers helped me a lot on my 6 months journey; they really helped me; I don’t want to lie. Continue with them, do not stop them. Help other people too.

I: Ok but on these stickers [paper unfolding]- what is it that you would like us to change on the stickers if we continue? You mentioned an SMS and what else?

P: I do not think there is another issue and the SMSs are all right to me. I only said at least I should receive the SMS at the same time I am supposed to be talking the pills. If I take them at 10H00, then I should receive the SMS exactly at 10H00.

I: Because it is a bit of a problem when there is nothing that reminds you before.

P: Yes, before.

I: After receiving the news that you are going to start TB treatment; did you tell anyone you are staying with?

P: My children- I stay with my kids. I told them the day I brought a sputum because I came with one of my children. I told them before that there might be a possibility that I have TB and I was with my first-born child when XXX called. I came with her to the clinic, and I was with her when I got the results, so I told her.

I: But how are they feeling, like how is it going?

P: They never stressed, they comforted me instead and said to me “Mom drink your pills you will be fine.” It better because TB is curable, unlike- I drink a lot of pills; I drink hypertension and ARV, so you see those are a lifetime. They would comfort me with TB and say, “it only just six months mom, drink and you will be alright” and I did drink, and I am alright.

I: So, what would they say if they see you drinking your medication and sending an SMS?

P: They would ask me “why are you sending an SMS after you done drinking your pills?” I tell them that it shows that I drank-they should see at the clinic that I drank, and it also helps me to remember to drink the pills.

I: Is there a time whereby you drank your pills, and you sent an SMS and sent it again, more than once a day?

P: I did that once and you know what made me do that? It is the SMS that we were talking about, it came after I drank and sent. So, when I received it after 06H00, I said to myself let me send it again and then I sent the SMS, but I did that once.

I: Ok, so since we paste these stickers on the pills, could you tell us where you keep these pill-

P: -Where did I put my pills?

I: Yes.

P: I have a box that I put my pills inside, so I would put them inside with my other pills and put in on the headboard.

I: So, it is a box?

P: Mmm it is a shoe box, I covered it nicely [laughing] I covered it and I put my pills inside.

I: Are they safe inside?

P: They are safe.

I: Have you seen this box (MERM)?

P: I saw it this other day and I even asked XXX that what is this box for. She explained to me that it is a box that reminds you to drink the pills.

I: So, since you only used the stickers, what are your thoughts on the box and the sticker [unidentified voices in the background] which one is easy to use and why do you think that?

P: To be honest [laughing] from what I heard about the box, the box is better.

I: Why do think that what is it-

P: -Because it reminds you.

I: Mmm.

P: It- they say it is loud.

I: You heard people saying it is loud?

P: Yeah, they say it is loud and obvious if it is loud, you will know that it is time for medication and even the kids inside the house would say “mama it is time” perhaps you are outside, you see.

I: It is time for the box?

P: Yeah, the box is ringing.

I: Mmm-.

P: I am not criticizing the stickers; they too are all right, but I prefer the box [coughing].

I: What is your other preference, what is it that makes you think that the box can be better than stickers?

P: My other phone- phone can break. Our phones- just like now, you remember I told you that my phone is broken. Phones break, if it falls, it no longer touches; it does not do anything, and you cannot even send your SMSs, you understand. There is no way a box can break, and it will keep on reminding every day. My phone was broken not so long ago, and I had to fix it, it was a problem to send, and it was not touching at all. When you start to touch it and you press one, it does seven. It was not touching even if you try, so that is why I say a phone is a problem for the label. I cannot send anything if my phone is broken, and I just cannot go and buy a new cell phone and replace it fast since I am not working. You must wait for SASSA (South African Social Security Agency) and SASSA takes time to process the monies before you can fix it and by the time you finally fixed it, days have passed, and you were not sending the SMS. Then people at clinic do not know whether you are drinking or not, you see.

I: Mmm I hear you. So, what are your thoughts if you are looking at the challenges that people come across when using- would you say we should give people boxes more than stickers?

P: I think the box is the best [door swing], and there are people who do not know how to use phones because as people, we are not the same and you find that some people cannot send an SMS, or they do not even have kids to whom they can say please help me. Even if they are there, you know how our kids are, they do not care, and they will be out doing their things and you are disturbing them when you tell them they should help with SMS. They get bored and they do not care since they are not the ones taking the pills. So, as longs as this box is going to remind you that it is time and not require anything.

I: Ok, I hear you. So, you think that there is not a thing that would prevent anyone from using stickers?

P That would prevent?

I: Yes.

P: There is nothing that would prevent someone from using the stickers unless that person does not have a phone. That is where you would not- how are they going to use the stickers? But it is not possible when you do not have a phone.

I: So, for you, a barrier or prevention could be when someone does not have a phone?

P: You cannot use the stickers if you do not have a phone. You will not be able to use them if you do not have a phone, how would you do it because you have to send an SMS?

I: Mmm.

P: Yes, you only need a phone to use stickers.

I: Ok, is that the only barrier?

P: To me that is the only one, I do not know about other people.

I: Yes.

P: To me, it only a phone.

I: But what helped you the most. What is it you would say helped you- these labels helped you a lot to drink your medication.

P: They helped me, you know. Most of the time it was- when I hold the pills because they paste them [clapping hands] on the pills, most time I hold the pills I see them. You see I am able to remember that I have to send an SMS, unless I am in a hurry just like I told you, but it is not often because I spend my time in the house. These stickers do help.

I: So, are there any difficulties involved in using these stickers?

P: I did not see any difficulties you know; I did not see any.

I: Mmm.

P: I did not see any difficulties with them.

I: Everything is all right.

P: Everything is all right; I did not see any difficulties concerning them.

I: You do not see any?

P: You just have to hold your phone and send an SMS, it is simple, and we are always holding phones man. You are always holding a phone if you are not busy, so you just send an SMS. Where is the difficulty in that? I do not see any difficulties.

I: Have you ever seen your adherence calendar on the tablet? That when you drink your pills it alerts on the adherence-

P: -Mmm I have never seen it.

I: You have never seen it?

P: They have not shown me.

I: They have not spoken to you about how it is going with you?

P: I do not want to lie, they have never.

I: Ok, apart from receiving SMSs that were reminding you if you forgot or when you did not send, has there ever been a time they called to check up on you?

P: XXX (intern) was calling me, she once called and checked up on me, but I was sending the SMSs. She once called and most of the time she would call just to check up on me.

I: So, how did you feel when they called you?

P: No, I would be happy, I become happy. I should be happy because they are taking care of me.

I: Mmm ok, have there been [door opening]- there are people who work at the clinic whom we call community health workers who work in the community, have they ever been to your place?

P: You know they once came, and they told me that they work with the clinic. They are from somewhere; they from XXX.

I: XXX?

P: XXX, they are the one that came but others never came.

I: Mmm they never came? When they got there-in short, could you take us through what happened?

P: No, when they got there, they explained where they are from and then [clearing throat] they told me that they know my TB status and asked for my sputum and from children I am staying. So, I told them that I- they came after a week, and I told them that I have been to the clinic with all my children and still awaiting the 6-year-old results since they injected her and told her to come back after 3 days. They collected my sputum [clearing throat] sorry. They collected my sputum and left me R150.

I: Mmm.

P: Mmm then they left.

I: So, I have not- people from the clinic never came on the second visit?

P: Mmm people from the clinic never came, people from XXX are the only ones who came.

I: So, when they got to you, was the information that they gave you enough or was a bit short somewhere?

P: No, it was enough.

I: They educate to a point where anyone could understand?

P: Yes.

I: So that they can be informed and aware?

P: Yes, they educate nicely, and you understand what they are saying.

I: So, what are your thoughts on someone who knows their status but does not drink the medication the way they are supposed. Do you think they should pay that person a visit and talk to them- could that make a difference, if you think so, why you think so?

P: They could make a difference if they can go there because they are going to sit down with them and show them the danger of not drinking the pills and then if it is someone who wants to live, they are going to listen. If you are not drinking TB treatment, it means that you want to die because it does kill, yes. So, if they can go to people who do not drink the medication and explain the way they explained to me, I believe that person who does not want to drink medication will, you see, because they explain nicely. They explain to you nicely even about the consequences of not drinking TB treatment and means you will end up going to what they call-what is that place, mmm XXX. You will stay there because you are not getting better and you are not drinking your pills correctly, yes. So, when you are drinking your pills correctly for only just 6 months, you are cured.

I: So, what are your thoughts on phone calls, visiting people or calling them reminding them to drink pills? Which one do you think could be used a lot and why do you think that? Where they call people who are on treatment and say please drink your pills or go to their house and check up on them. Which one is better and why you think that?

P: I [clearing throat]. I think all of them are all right, both home visits and phoning. They are all right, you know the more company you have and people telling you what you should do, you also get comforted because it like some sort of counselling. They explain to you so you can understand. As people, we are not the same, you find that there are people who do not see a need to drink pills. It is no use because I am going to die anyway but the more people come and talk to you and explain to you, you begin to understand TB. Then, it becomes easier to drink them.

I: I love the fact you mentioned the word “counsel”. What it is that they said to you that motivated you a lot and you decided you are going to drink your pills and complete them?

P: No, what they told me is that 6 months is nothing and you will get better if you drink your medication and if I do not drink them that means nothing but death. So, when I looked at my children, they are still too young, and I said I will not die because of TB. There is no way TB could get rid of me, let me drink my pills. Just like they are saying it is nothing, six months is nothing. So, I drink my pills correctly so that I will be cured, and I drank them. They discharged me, here we are, and I am all right.

I: Yeah, I hear you. So, since you were using the labels to drink them, I want to know how satisfied are you? What is it that you can say?

P: What can I say?

I: Are you satisfied?

P: I am satisfied.

I: Mmm.

P: I do not want to lie; I am very satisfied. I am very satisfied.

I: Do you have-

P: -and then-.

I: [clearing throat] continue.

P: It was not the first time I get TB.

I: Mmm.

P: There were no stickers the first time I got it, you understand.

I: Yes.

P: They were not there, and it was difficult. I do not want to lie it was difficult- there were times where I would go 2 days without drinking them. Sometimes I would remember late that I did not drink them but with these stickers, I do not even remember a day- I never jumped even a day. So, I saw the importance of the stickers. I drank treatment before without the stickers and it was difficult for me but on the second time stickers were there and it was quite easy.

I: When did you drink the first ones?

P: I drank the first ones in 2016, so it was for the second time now.

I: Mmm so you mentioned that you drank them without stickers before. What is it that was problematic without the stickers?

P: It is what I said before, that after 2 to 3 days, then it is when I remember that oh pills, I did not drink the pills and that time I was drinking ARV’s only. Like I did not have that understanding, I would put those ones over there and the other one over there, there were just like that. So, I do not want to lie, I would go days without taking TB medication but with luck [kids screaming in the background] I got cured. I do not know how but to be honest, I was not drinking them correctly. I would tell them even at the hospital that I forgot the pills, I was not drinking them correctly. I used to forget the TB ones, but you know that they will lash out on you, they will not just leave you like that. I told them the truth but with the stickers there is no single day I go without taking pills, I do not want to lie. There is no single day I would say I missed; there is none, not even once with these stickers because if I did not drink them, at 06H00 I receive a message on the phone. Then if I check, I did not drink and then run fast to drink my pills and send the SMS.

I: So, before when-when you were forgetting to drink your pills in 2016. Which method- how did they notice that you were not drinking your pills correctly?

P: They did not notice, how were they going to notice if I do not tell them? They would not have noticed if I did not tell the truth, If I had told them that I am drinking them every day. Who was going to tell them that I did not drink?

I: Mmm.

P: There is no way they could have known, and I am always in the house drinking, how were they going to see if I drank or not? There could have been a possibility of 6 months ending without me starting the pills and they would have not noticed, and I would tell them that I am drinking even when I am not. Then tell them that I do not know why I am not getting better because I am drinking them knowing very well that I am lying.

I: So, do you think the intervention of the stickers is it helpful the way it was implemented?

P: It is helping a lot- hundred percent. It is helping hundred percent and as for me, it helped me hundred percent even when the kids were playing games on my phone and a message comes in, they let me know and say “message mom” there is no way you could miss. Sometimes you find that did not see it and they would say “message mom” then I take the phone and check, and I find that no, it is my reminder. To be honest, stickers are helpful, and I do not want to lie, besides the case of a phone breaking but they are very helpful as long as the phone is all right.

I: I understand that you were using the stickers but what are your thoughts between them and the box.

P: Mmm.

I: SMS.

P: Mmm.

I: Phone call.

P: Mmm.

I: Even a home visit. Which one do you think it is best and why would you choose it amongst them?

P: [laughing] as for me, I speak of the stickers.

I: Mmm.

P: I do not have a problem with the stickers, problem is with a phone if it breaks. My only disappointment is with a phone, but I love them because I have an experience of a phone breaking. I prefer the stickers, you understand. Stickers are the best as long as the phone is all right. Stickers do not have a problem even if you are somewhere but with the box, I mean I will not carry the box with if going somewhere because carrying the box in the bag, you understand. At least if I am carrying a bag, I can put it in the bag and it will beep inside the bag, but how can I walk carrying the box on the street? The box will be noisy [laughing] when reminding me that I did not drink the pills or it is time, so it a no for me. With the SMS, at least everyone receives an SMS even when it says click, click [imitating notification sound] there is no one who would want to see what SMS you just received, you see. To be honest, they are fine. Only if our phones did not break but they are fine to be honest. With the box, you have to walk while carrying it and it is going to ring inside the house when you leave it behind, say you are in town. it will ring seeking your attention and you are not there.

I: So, what are your thoughts looking at how simple the SMS is? Is it simple to use the labels?

P: Mmm it is simple.

I: So-.

P: -Even when you do not want people to know of your problem, it better with the SMS because how are they going to know? It is going to remind you and you will just go to your room and drink your pills, then you come back. The box is going to make noise and they will ask a lot of questions, like what is it for? They will ask what this box is for and why is it ringing like that, but the box is fine for people who do not have a phone.

I: So, would you suggest that we take which age group, which age group should use the box and which age group should-

P: - exactly, certain groups. The box should be used by older people. Our parents do not know how to use phones, only few. Should I say which age, I do not know but older people; older than me. Our parents are the ones who can use the boxes and people in my age; people around forty-three can send the SMS. They can give boxes to people who are 50 years and older and then stickers to people who are 49 years and younger.

I: Mmm, your thoughts.

P: Mmm.

I: You find that someone prefers the stickers but cannot use them exactly the way they should.

P: P: Mmm.

I: Now, what is it you think might be better or we can bring since you think some people cannot use stickers, what is it that would be better? You just mentioned that people your age cannot use the box, the younger ones if we go by age.

P: They would not want the box? Are they struggling to use-

I: The stickers. Let us say the age group you just mentioned, the younger ones.

P: The younger ones.

I: Is not able to use-.

P: -The stickers. Phone call, you will call them.

I: So-.

P: -you will remind them.

I: We should call every day?

P: It is a lot of work [laughing]. It is a lot of work. They will tell the kids to help them. Yeah, there are kids in the house, they will help them or their partner. Not everyone in the house will struggle to do that. All of us in the house, it is impossible especially for my age, it is rare. Let me say someone who is forty-nine and younger, it is rare to find someone of that age group staying alone. If you are not staying at home, you are staying with your kids or if not your kids, then with your partner. Being alone at my age? What would have happened for you to stay alone? So, obvious people in my age can have stickers and your partner will help you or the kids. They cannot all refuse.

I: So, since you started your medication and you completed. Besides your children, is there someone from the outside whom you told about your status?

P: You know I am kind of -I do not know how to hide. I talk, I do not know how to hide it even when someone comes inside the house, and I would tell them that I am drinking TB medication. You can come in if you are not afraid but if you are afraid of TB, we can talk outside. I am that kind of a person; I do not know how to hide. There were times where you find that I fainted inside the house and the kids are at school and you come in, and you know me, how would you help me? At that time, I cannot do anything, and you are taking me to the clinic, when you get to the clinic, how would you explain that I am this kind of a person? That is why I am struggling to hide, so there is no one who does not know that I am taking hypertension, ARV, and TB. I do not how to hide.

I: Since you have already spoken to people in the house or from outside, how did you feel after you spoken to them?

P: No, I become relieved after I spoke. I was so relieved after I told them about my status. What you say about me, that is on you, as long as I told you. What you say about me, I am not getting involved, as long as I reduced the baggage. I am doing that so that even when we are chilling- because you cannot chase a person away. Am I supposed to hide myself when it is time for me to take treatment? I am not going to hide myself when it is 08H00, I drink my pills. So, I am also avoiding being asked what the pills are for because you like to ask. Yes, will you continue to ask me when I have already told you. No, you will not.

I: How are the people you have talked about your status treating you up to so far?

P: They are treating well. There has never been anyone who wanted to distance themselves from me, they are treating me nicely and everything is normal. There has never been anyone who was afraid of me or being afraid that I might infect them with TB.

I: So, what are your thoughts- your thoughts on people who are trying to share their status or disclosing to others and end being treated in a certain way. What is it that can be done to make sure people understand more about diseases?

P: No, people have to be educated, just like there should be lessons provided at the clinic so that people can know that they must not judge. You should not judge me when I tell you, my status. Instead of judging me- it just that I do not know what to say, you know. Tell me something that would comfort me if am telling you that I am taking TB treatment. You should at least say to me “no since you are taking TB treatment, stop drinking alcohol and smoking” I am just saying. I do not smoke [laughing] I am just explaining, you should be guiding me like that, you understand. “Do not sit on dust, open the windows” you should guide me and not judge me or do something like asking such questions as “how come that you have TB?” what is that no. They should not be any judgement but lessons, I do not know how or where. There should be lessons provided at the clinic maybe before we get in, educate people.

I: Your thoughts, you know that people do not come to the clinic if they are not sick or do not have any problems, so what can be done to make sure that the information you just provided reaches them?

P: So, that it reaches other people? There are people who work at the clinic who can walk around and do door to door, right?

I: Mmm.

P: Yes, I was thinking that when they come, they should explain and educate people about people who are affected by diseases and sickness. I think it will be simple.

I: You think it will be simple that way? The information should be delivered by people who works-

P: -yes, at the clinic.

I: They should go straight to the community?

P: Yes.

I: Explain to the people?

P: Yes.

I: No, we are grateful for such info, but can you tell us about your experience so far since you started treatment? You used the labels from the beginning of treatment till you completed and got discharged. What can you say?

P: About the label?

I: How are you feeling?

P: You know I love these labels. I love them you know, for me to love them it is because they helped me a lot with completing the treatment and being discharged, they helped a lot. They helped a lot, I do not know what I can say but they helped me a lot, continue giving others so that [door swing] at least they can be cured. I don’t think there is someone who can see the reminder and still ignore it like that [paper unfolding] and not drink the pills. It means that they do not want to be well, or they are ignorant.

I: Yeah.

P: When you see it-because this is a reminder mmm, it is.

I: Mmm I hear you; it seems like you really loved the labels.

P: I really loved them because they worked for me, they worked for me. I am here today because of the label; they helped.

I: Mmm but amongst them all, which one was most useful?

P: On the?

I: The label, the box-.

P: -It is these SMSs- the labels.

I: The labels?

P: They are useful.

I: So far since you started using the labels, is there any problem that you think needs to be fixed? Something that maybe you think if it could be change, it will be better.

P: Mmm it is the one I was telling you about that [clearing throat] if at least it reminds you exactly at the time you are supposed to drink, and they should ask you what time you would prefer. You give them the time and they set it automatically, I do not know how.

I: Mmm.

P: Yes, if you are drinking at 08H00, then it should remind you exactly at 08H00. If it is half past, half past you receive an SMS saying do not forget.

I: Oh so, you think so. You know the box does exactly what you just said.

P: Mmm.

I: The box does that.

P: It does?

I: Yes.

P: How does it do that?
I: We set a time on the box.

P: Mmm.

I: In time- just like you explained, it rings on the time you want.

P: Mmm.

I: And then you [inaudible segment].

P: You take them out from there?

I: Yes.

P: It is alright then [laughing] I do not know it.

I: You do not know it?

P: Oh, the pills stay in there?

I: Mmm just the way you proposed with stickers, though it does not happen with stickers.

P: Mmm

I: But it does happen with the box.

P: Oh, the pills stay in there, and then it rings, and you go there and drink the pills?

I: Mmm.

P: It is alright [laughing] but the problem is when you left it home and then it rings, do you turn it off or it does turn off?

I: [inaudible segment]

P: I mean when you are done, when you go back will it be quiet? You see when I am not around, and it rings. Will it ring until I come back and switch it off?

I: It does not ring for that long, but it will turn off.

P: Oh, ok.

I: So, with your expertise, if we changed the reminder like you said.

P: Mmm.

I: On the stickers-.

P: -on the stickers.

I: And we have the box, do you think all of them should be here helping people or we should remove one that people do not like the most?

P: They should not remove them. I think they are both working; all of them are smart.

I: So, that a person can have option to choose?

P: Yeah, to choose between them.
I: Mmm.

P: There should not remove any one of them, but box is fine for me since I do not have a phone.

I: Mmm.

P: Box would work for me. The SMSs- the sticker will also work for me if I have a phone, you see. Not all of us have phones, just like I told you that my phone was not working, and I could not send the SMS, you see, that was difficult for me. I do not know if here at the clinic, think I am drinking or not, but you also know that these things will confuse you, I am going back to the stickers.

I: Mmm.

P: Why am I going back to the stickers? With the sticker I just send when I am done drinking. I just send an SMS to the clinic, and they can see that I drank. The box will remind me, but they will not see anything at the clinic.

I: Even the box when you just drank, automatically it sends a message.

P: Oh [laughing] oh these things work the same? Oh, ok but I prefer the stickers.

I: So, you think we should keep the stickers?

P: Or because they worked for me, they worked for me. I prefer them.

I: They are ones-

P: -They worked for me.

I: They worked for you?

P: Mmm.

I: Would you recommend them to someone else?

P: To someone, yeah. I can tell them that “you know now, you are holding life, if you comply with the rules of these stickers, you will finish in 6 months” and get discharged.

I: We heard you, but before we come to an end of our interview, is there something that you would like to add or something you like to talk about?

P: I do not understand.

I: Like before I close, while we were having our conversation about the stickers and-.

P: -The box.

I: The box and everything, including SMSs and phone calls. What are your final thoughts?

P: Mmm, for me it is the stickers, I talk about them because they worked for me.

I: Mmm.

P: My thoughts, right. I recommend the stickers 100%, I wish you can give them to all the people, and I love them, and the way XXX (intern) was working. They would call and ask me whether I drank or not and I would tell them that I drank, I think it is a good thing. There will not be a day you forget to drink your medication; it is impossible. It will only happen if you are stubborn and do not want to drink your medication. They are fine though, and I love even the way they are treating us, I love it. Compared to 2016 where there was no one reminding me that you are drinking or not drinking, even when you get here and they ask if you are drinking, how would they deny it? Yes, it the SMS and I do not believe that you can send without drinking the pills, that would mean you are not alright. Just sending an SMS without taking the pills, something must be short in the mind. Immediately when you send SMS it means you drank, I think the stickers are number 1 for me.

I: They come first?

P: They come first. They come first because they let the health care worker know and will call you if you do not drink. You are always afraid that they will call you and I would get nervous that they will call even when I drank. I would get nervous saying that I do not want them to call and say I do not drink. You see that, it was like I am not taking my pills and I am taking them, but once I came to the clinic and told XXX (intern) that “XXX do not be surprised when I do not send an SMS, it is my phone; it is broken. I do not have a phone, but I am drinking my pills. [clearing throat] I am drinking because it was reminding me even when the screen was broken. I would see the message, but I could not respond, you see. I love the stickers, but I do not hate the box, just that I do not know it but at least with stickers, 100%. I recommend them that there is life.

I: There is?

P: Mmm.

I: Before we close, my last question. Your thoughts on someone who drinks medication using- who is using the stickers or the box and has to go to work in the morning. Which one between the two would be simpler to use?

P: The stickers are simple, especially for someone who is working. It is simple. To be honest, you will not go to work carrying the box. Yes, I think stickers are the best for someone who is working. Stickers are the best.

I: Mmm, we thank you for the information you just gave us today and for joining for our conversation, which was wonderful. We thank you for that and we are happy sticker helped you to drink your pills. You started with them, and you finished with them, and we thank you for completing your treatment and your courage to explain to people. We thank you for informing people and that you would recommend the stickers. I do not know if there is something you want to say before I close.

P: No, I also thank you for the stickers and the way they helped me. I was able to drink my pills from the beginning to the end without any problems because of the stickers, I thank you. Please continue to help others with the stickers, do not even cancel them because they have a big purpose, I thank you.

I: Ok, we have come to the end of our session, the end time for this interview is 12:58.
