# Supplementary material for: Acceptability of Digital Adherence Technologies to support people with drug-susceptible TB in South Africa
Source: PLoS One. 2025 Sep 24;20(9):e0332103. doi: 10.1371/journal.pone.0332103 (PMC12459780; doi:10.1371/journal.pone.0332103)
Supplement: S4 File — (ZIP) [file pone.0332103.s004.zip › S4 Transcripts/PwTB/IDI 11_PwTB.docx]

Translation Sepedi

| **Label Key** | **Meaning** |
| --- | --- |
| **I** | Start of each new utterance by the Interviewer |
| **P** | Start of each new utterance by the Participant |
| **N** | Note taker |
| **{ }** | Indicates that details were changed or pseudonyms were used to anonymise data |
| **( )** | Indicates the description provided to anonymise data |
| **XXX** | Words were omitted to anonymise data |
| **-** | Breaking into a sentence by the next speaker |
| **…** | Pause or drawn out words |
| **[ ]** | Indicates noise made, e.g. [laugh], [sigh], [pause] |
| ? | Beginning of utterance by unidentified speaker or questionable text |
| **[inaudible segment]** | Unclear section of the recording |

I: Do you ma’am consent to our interview today?

P: Yes, I consent.

I: Date xxxx (interview date), location xxx [clinic name] Clinic, PID, it’s uhm xxx and time it’s uhm 11:50 am, language used is Sepedi. Ma’am I thank you today for coming and doing the interview with us. In short, I would like you to tell me who do you live with at home?

P: At home, I’m living alone.

I: Okay, so when did you find out or how did you find out you were infected by tuberculosis?

P: I find out that I had Tuberculosis by how I breathed and coughed… I was breathing heavily and it couldn’t stop. I wanted to find out why I was coughing to a point where it hurt. I sat down and remembered that TB has been around my home. That was when I realized I should come to xxx [intern’s name]. They gave me a bottle here in xxx [clinic name], they said “take this, spit your cough-fluids in it.” I went home and spit in my cough fluids and came back and brought it here at xxx [clinic name]. They called me on the 25^th^ of October, asking me to come to the clinic on the 26^th^. I came, and when I arrived, they said I should go see xxx [nurse’s name] from room number 7, I entered and found her. When I just entered, she took out a blue file, and wrote something down, as she writes it down, I realized that here I have TB. She gave me pills, she gave me Rifafour pills… the ones with a white box. They are written Rifafour outside. She gave me those pills and said that I am supposed to drink four of them. From those pills, she gave me stickers. She said that when I drink those pills, I should send them an SMS. I had one problem because where we lived, there was xxxxx (area name) behind – Squatters, and they have illegally connected to electricity. When I made the SMS, it couldn’t go through to them [the clinic]. They sent me a message [inaudible segment caused by noise] Can we continue?

I: Yes, we can continue.

P: Alright, as they gave me the stickers so that when I drink the pills I send them the SMS, the process couldn’t be possible due to network. My message couldn’t go through to them (clinic). When they could not get the message, they sent me an SMS, and asked me to drink the pills. On some days I was lazy to SMS. I would take my medication but would not SMS because I was just tired. I saw it fit to go to xxx [clinic name] clinic, I arrived and explained to them and said “ xxx [nurse’s name] I have a problem with xxxx (squatter camp) around my area, when the lights are off the SMS cannot go through. So, I send it, it doesn’t go through – I send it, it couldn’t go through. So how should I work about it?” That was when xxx [nurse’s name] said to me “I will give you the box – I will give you the box, the box I’m giving you will clock [alerts] to you at the specific time, you will have to drink the pills – at the certain time, you have to drink the pills. The box will ring on itself, when it rings, you take the pills and drink. Immediately you open it, it will clock (alert) to me that you opened the box, and you drank the pills. Immediately when you open it, it will clock to us that you are drinking the pills.” I said “Okay, thank you” and took the box and went with it. Always truly “tlwikitlwiki, tlwikitlwiki” [imitating the box alarm] the box rings, I open it, I drink my treatment, I close the box - I drink, I close the box. But here is the problem, after 2 months, the box rings at eight, I open the box, I drink the pills, when I’m done drinking, I close my box, and I put it there. How? 10 minutes, “tlwikitlwiki, tlwikitlwiki” [imitating the box alarm] rings the box. The box costs me that – I just opened and left it there, because eight in the morning I drank the pills, 10 past eight, it rings again. So, I saw that I should open it and put it there.

I: Mmm.

P: Up until I realised that I drink well, and I’m getting used to it. I drink, I drink- I drink, the box is not giving me problems.

I: Mmm.

P: Yes

I: So, going back on the issue of symptoms, or the way you felt – signs.

P: Mmm.

I: You talked about coughing.

P: Exactly.

I: So, what other symptoms do you know of besides coughing?

P: The ones I know of? To sweat – It is to cough, to sweat and not having appetite, I was not eating alright. I wasn’t eating alright at all, but now I eat, but this sweating, doesn’t go away, up until the doctor today told me that I am getting old– the doctor explained to me that I am getting old, even at the doctors/ sister’s side they also told me that I get old. So, am yet to accept that I getting old, but now the doctor said since I cough and vomiting, they should give me the asthma medication so that at least when I feel like coughing is hard, I should spray, but not right through – he gave me a letter, and said I should go to DB to do X-ray, he wanted to find out what is the problem at that moment – because I sometimes feel like I have sores, things like that, but he explained to me that I won’t just finish the treatment at the same time and recover same time, it will get better with time, things will come around. But the hurting thing is to cough, because when I cough, I bring back the food I ate. Even the pills sometimes come back, mmm.

I: So, when you say you cough and that sometimes when you cough you cough out the intake-

P: Exactly-
I: So, besides today, have you ever explained that to a sister or the doctor about that cough?

P: Each month I come here I explain my problem of coughing and vomiting. I was told to test for COVID, I was negative. “What else am I supposed to test?” They said no, I am supposed to face the TB treatment, that is where these problems will end, but still they continue. They really continue, coughing and vomiting.

I: Okay. So, we will talk about that little later, right?

P: Okay.

I: So briefly, do you know what are these? [demonstrates something]

P: The Stickers.

I: Okay, briefly, please explain a little about them to me, how do these stickers work?

P: These stickers [tongue click] when you open, what is it, it’s 57… I can’t see clearly, you will correct me, right? It’s 579, you see it’s here, and then here are the numbers. Send a letter, best…. What is it, is it visit or what? Something like that, oh! enter these numbers with your phone, you enter them – you enter these numbers and send them, and they go through. These are the ones that were going to xxx [nurse’s name]… Exactly. Those are the ones that caused xxx [nurse’s name] to give me the box because the message couldn’t go through. That was when she wanted to know why I wasn’t taking my medication, I said “No, I do take my medication, but the problem is the network, sister.” If there is no electricity, network is not working.

I: When you said they go to xxx [nurse’s name]…the SMSs, who explained to you about these stickers.

P: It was xxx [nurse’s name] since she took the tablet and said, “Do you see, I insert your phone numbers here, when you send, they will get in here.”

I: So, during the day when you were being explained to about the stickers, briefly, what were your feelings about them?

P: I just told myself that for us- we were not around when things changed, I’ll see what I can do, it is because of this disease that I came across such things. I will just press and press, they have shown me how to operate it, I just press and press it. Sometimes they were successfully sent, and sometimes I just see that this one couldn’t go through. Network is down. Yes, I took an effort to come to the clinic, and I explained to them (HCW).

I: So, when you come to the clinic, what do you use?

P: My feet (patient walk on foot).

I: Oh, is not far from where you live?

P: Mm-mm (No).

I: When they explained to you about stickers, if you remember clearly, how long did they take to explain to you about the stickers?

P: It took her time; I spent the entire day here. I spent the entire day here and actually I don’t know how long I stayed here because when I add the time of the treatment, when they were giving me this and that. When xxx [intern name] was done giving me the pills, it forced xxx [nurse’s name]as well to come and leave me with stickers. I spent about four closes to five hours here in the clinic that day. That was the day they gave me this bag, they said it’s a bag for TB, they packed all my medication in it. Yes.

I: So, that time when they explained to you about these stickers, is there anything you think or feel that you were not informed about by any mistake, you wished they should have told you, the one you wish they should change it from the way they explained to you?

P: Mmm…I didn’t see anything wrong with how they explained it to me. I accepted the way they explained to me because they know all about things like these, where else I’m just like a child – remember I am a patient. When they told me to do this and that, I am supposed to do it the way they instructed me to. Yes.

I: Oh.

P: Yes

I: Thank you [inaudible segment], so going back a little bit, during the day they informed you about your results – when they opened you the TB file, just as you told us – So, what were your feelings about it, when they told you that you were infected by Tuberculosis?

P: I was hurt because when I thought about my home-my father died of TB in 1982. When they explained to me that my father died of TB, they said that during 82, TB didn’t have treatment, but now it has got a treatment. Then came it to my mind the fact that my father died of TB, my uncle also died of TB, that meant that I’m also meant to die of TB. That’s what came to my mind, but when I arrived at my aunt’s and explained this to her and I said, “Aunty I was told at the clinic that I have TB,” she said “No my child, you just drink your treatment and if you drink them well, you will recover,” I said, “They said it’s six months because I took test cough tests but if I took it with the X-ray, it was going to be nine months. They said mine is six months. I will drink the pills, when I drink pills well, it will reach six months and if I don’t drink the pills well, they might increase with three months to nine.” She said, “No my child! You just drink them and if you drink them right, I do trust you on taking your pills. If you drink them right, you will live. Do you hear me my child? TB recovers.”. I had the thought that my father – I don’t have a father today because of TB, I don’t have an uncle because of TB, you see, [inaudible segment] yes. But one time while I was at my home, I accepted that at the end of the day TB is disease prevalent in my home, who was supposed to be infected? The way I concluded – I comforted myself, “it’s a normal disease at my home.” I had to be infected because I am the child of the male of the family. So, it had to infect me, and I said, okay, I’ll recover, I’ll take the treatment and recover, yes.

I: So… to understand the thoughts you had when you said you will recover [inaudible segment] like when you tell me, what is that thing that motivates that you will recover-you will defeat TB and you will recover? When think you that at your home there are people that have passed, and then… I’m sorry, I’m sorry to hear that your father and uncle have passed due to the same disease [inaudible segment] but you have hope… like you have the belief that you will defeat this thing. So, share briefly with me your journey with TB. [inaudible segment]

P: Something that made me to have hope that I’ll live, I’ll recover – was that during those times around ’82, by the times of my father, there was no treatment at all, and I am lucky that I got infected in the times where there are pills. If I drink the pills, I will recover – I got infected during the good times where there are pills. Yes.

I: Oh, okay, so to go to my following question… So, ma’am because you have experience with these stickers or these labels, what was difficult or challenging with using these stickers?

P: It is the phone –honestly as for us, we are used to those phones where you press and answer. These ones where we are supposed to press somewhere to create a message, it compelled me to go ask – there’s a child next door, I would call him and say, “Come here my child.” He would come and I would say, “you guys know these things of phones, you must send these numbers with a message,” I would explain to him, explain to him, and he would say, “Oh okay, this is so easy”, he would do it and do it and then say, “You see, it went through.” So when it couldn’t go through, he would say, “There is no network, do you see, it can’t go through.” So, when it couldn’t go through, he would show me. So, after he showed me, I know I would make my way to come here at xxx [clinic name], “Good people the message couldn’t go through today due to unavailable electricity, the network is not functional. Xxx [nurse’s name] in a calm mood, said, “There is no problem, I will give you the box.” I didn’t know that box. When she says, “I will give you the box,” I said, “Okay there is no problem.” She gave me the box, she sat down and explain about it to me – she explained and explained, I packed my pills inside (box) and said to me “It’s going to ring at a particular time, you have to open and drink the pills and close it.”

I: Okay.

P: Yes.

I: So, on the issue of the next-door child (neighbour) – how many times did he sent the SMS?

P: Uhm, he did it twice, from there most of the times he would be at school, so after I drank, I was like…“oh, he showed me,” I press and press, then it goes through, and then now after it goes through you get a message , they will respond back, and said, “No, we thank you, we got the message,” Yes. They respond back there – same time, message gets in, and they say, “We thank you, you drank the medication, good.”

I: So, at the time when you were using these stickers, what was easy about using the stickers?

P: What was easy – Since they attached the stickers on the pills and showed me that you see when you drink here like – do you see at the back of those Rifafour pills? She pasted one of the stickers, and she gives you and you eat – you eat, and I finish. when I get to the second packet, you will find another sticker and so on. Eish [sighs] that problem has arrived, right? That one of burning, eish… [inaudible segment].

I: So…

P: So now I don’t know how we should work about this issue of the skin at the end? Or will it recover?

I: Are you ma’am working?

P: No, I’m not working.

I: Temporary job?

P: What?

I: Piece job, temporary job?

P: Nothing – Nothing, I am not working, I’m not doing anything. Even at that place, they gave me a letter to claim funds for TB, it has been unsuccessful. I am not working, and I have no income. The only income I receive is the R350 (social relief grant), I only eat R350s, within my house. Now since they said June – in June, my home has just been still. But some other things, you know? You will find out that it is just caused by not eating well. When you drink the medication, you must find some fruits, cabbage, some vegetables. Did you ever see things like that? Mmm, so if I would have just this maize meal [phone rings] I have something. But I just imagine, right? I just thought that maybe if I get this and that, maybe I can pick up and be alright.

I: So, is it that by the time you use these stickers to take your medication –Do you visit with the stickers or you avoid going somewhere with these stickers?

P: The stickers? I did not visit with them, something that I was using is this one. My cousin died on the 26^th^-we buried her on the 26^th of^ January, and they had (HCW) changed my medication, it was the time I was supposed to start this small one. Do you see the one that comes from this Rifafour? I was starting this one, I went with this one.

I: Oh! this box?

P: Yes, I went with this box because we left early from here indeed. We departed at 8 and eight O’clock is when I’m supposed to intake my medication. So, I realised that I must go with my box as we were travelling in a car. I went with my box. 8 O’clock exactly, I went to the car indeed, opened my box and drank. I closed and put it back in the bag, and put it.

I: So, ma’am you used the stickers and you explained to me about them. So, may you please explain to me a little bit about this box and how it works and how it reacts on you?

P: Uhm, the box has been good to me, because it would call me from the sink when I am washing the dishes, it would begin and say, “*tswiki,* *tswiki, tswiki*” (imitating the box sound) and I would know that I must take my medication, it’s time. There is no other way, and it doesn’t miss – it rings at that one time, this box. The time to drink medication is well set, number one (the best), exactly.

I: So, what reminds you to take medication other than the box?

P: Something that remind me?

I: Mmm.

P: It’s the morning – most of the time I look at the time, I would wake up exactly 6 O’clock. Exactly 6 O’clock I would wake up and say, “Oh! it’s time, it’s the morning.” I would wake up and brush teeth, wash my hands. I plug the kettle and then bath – I bath and finish. I warm my food and eat them usually around seven. I would eat for about 15 minutes, so that they could be balanced and digested, so that they would not come back. 8 O’clock the box will ring, and I would take the pills and drink, calm down, so that they could be digested. Thirty minutes – half past eight, I stand up and be busy with something. Then I would say, “*Hai* (hey), the pills are gone – they are really gone.” Aa! When I start to cough –

I: So how was it to visit or to take your medication along with the box, even if it rings?

P: I had no problem and [inaudible segment] my box – I did not have any problem at all. I saw it easy to open it during the time when I had to take the medication because if I just leave it, it will be left ringing alone in the house. So, I had to just take it and go with it.

I: Mmm.

P: Yes.

I: So, during the time they were explaining about the stickers or the box, your worries – what were your worries?

P: My worries were at the beginning because when I began with my treatment, I received stickers – my worry was that the same TB killed my people, and today I am taking the TB treatment, I am sending messages; it is all the same. I lost a father – I do not have a father today because of the very same TB. They say I am infected with this TB, but I will recover. Well, I will see. I went home and arrived. I got used to it, after a week I was then accustomed to it because they gave me a word that I will recover from TB, “You will recover, you are not the first one or the last one – lot of people are sick, and they recover.”

I: So, you talked about you telling your aunt about your situation of TB.

P: Exactly-yes

I: So, was she also aware of the TB history in your family? So, what were her feelings about it, when you told her that you have a problem with the disease of TB?

P: The way I see her, my aunt – my aunt had no problem, even the small one, she just said to me, “hey *nxa*! [tongue click] there is no problem, you will recover my child, you will recover. Don’t look at the situation of your father because during the times of your father there was no cure, now TB has a cure. You will recover if you take care of your yourself and take treatment. You will recover, you will be alright.”

I: So, when you told her about the TB disease, did you also tell her about the box? Does she know about the box, your aunt?

P: She does not, and she doesn’t even know about the stickers, she knows nothing, she is old indeed. Look, they know nothing about these things.

I: Mmm.

P: Yes. They don’t know anything. What she only wanted to see, was the pills. She wanted to see what kind of pills they were. I showed her and I said, “you, see?” She said “Drink, as long as you take your treatment – treatment is number one, you will just recover well, and go back normal self.” And I am also drinking the nightly ones, right? So those ones are risky, they tell you straight up that they are too risky because you are at the risk of getting TB and even asthma. Even when you start to cough and when you tell them that you feel this and that, they’ll ask if you have tested for TB. “No, I didn’t test for TB.” They’ll say, “go to the TB room and they will give you a bottle, you will test for TB, because as long as you drink the pills –those are friends, they go hand in hand with TB.” So that is where I left off, I said “it is all the same, it is a disease right? There is nothing I can do when it has arrived, it has arrived, I will take the treatment, if I recover, I will recover. If my time has arrived, still I will just pass on, right?”

I: So, besides the aunt and other people within the family, are there other people who are aware of your TB illness?

P: Yes, they are there.

I: If you say there are there, so that I understand, who are they?

P: Uhm…it is xxx [friend name]– it is this certain woman named xxx [friend name] and her sister named xxx [friend name], they are the ones who used to check up on me. They are those who I used to live with them. So, they come and check on me after they heard that I’m not healthy. “Hey, what is the problem?” This one called by the name of xxx [friend name], she is a traditional doctor. Her being the traditional doctor, they like to form stories around witchcraft, right? There was a time when my body had a problem, and I was coughing in front of them, I vomited, and she said “Mmm! (expression of amazement) this is witchcraft illness” I said “No, it’s not” she said, “look, this is witchcraft illness,” I said “look now, it’s not witchcraft illness, this is TB, I got infected,” “No, at the clinic, they do regard witchcraft illness as TB, they will kill you with it, this is you not listening, just leave the pills and I will give you the herbs” and then that was something they told me about at the clinic, that “now that you are sick, you are diagnosed with TB, some people will tell you that is witchcraft medicine, let me make you herbs – let me make you this and that. Do not agree to that, just focus on your treatment only.”

I: Mmm, so I am going get to that – I was writing down about the witchcraft illness. So, I’m going to ask, the following question. So, do they know about your situation of TB illness?

P: Yes.

I: And now that you are using the box – do they know about the box or the stickers?

P: They know about the box. Now a good thing is that her man is diagnosed with TB, he has the box. She even came to me and said, “I saw my friend has the box, I want to know how it works.” I said, “The box when you just open it, it rings. It rings only during your time of intaking medication. When it starts to ring, you open and you take the medication” she said, “The father of the house has also received the box, so, I told him that my friend also has the box.” With the box, when you wash your dishes there, and the time arrives, the box will ring and say, “hey go and drink the pills.”

I: Mmm, when you told xxx [friend name] and other people about the box, was it easy to tell people about the box, and that it reminds you?

P: Yes, it was very easy because we talked about it as if it was a joke, you see when you are relaxing with your friends there, “Friend look at what they gave me from the clinic” “Hey you, I thought it was a lunchbox” “No, it’s not lunchbox, my TB pills are here, when I pack them, I pack them inside here” “ How does it work?” “It rings here, do you see here, it’s a speaker, it lights up, when I just open and drink, it clocks to them, and I close”. Yes, it is just that I took it simple, had you ever seen that my dear?

I: Mmm.

P: Yes, I got used to them as my pills. Life is mine, so, it comes first, do you understand it? I should stand up for my life, I should not care about what the other people say or do. This friend of mine xxx [friend’s name] – look what the other person does for you – after seven, she came here at my home to stay with me – by the time the box rings, she said to me “my friend, don’t drink the pills.” I said, “my friend, this is the time,” she said “this is a witchcraft illness. Don’t ever drink those pills.” I left my pills, I didn’t intake them that day indeed. But after she left, something said to me "this is tour life not someone else's, take these pills and drink them". I then took them and drank. Yes, my brother, from there, that person had never set her foot at my home again – because of that, I chased her. I chased my friend away just by taking these pills, whereas she told me that they are witchcraft illness, and also by saying “it is TB, I’m going to intake the TB medication.”

I: For me to understand ma’am –

P: Yes

I: So, do you say, she never came again? –

P: She never. She doesn’t even enter my home. She doesn’t – I don’t know if I made a mistake by taking treatment because she is a traditional healer and she told me not to continue with the treatment just because this a witchcraft illness. I don’t understand what she was after. She doesn’t even enter my yard that lady. We just greet each other only on the street, “hi!” “Hi!” on the street. She was no longer that one that could enter and say, “friend I feel like I could get a tea,” then I could make a tea then drinking together. She is no longer that person. I have taken [nurse name] words when she was saying “you know what? The traditional healer could tell you that you are infected with witchcraft illness, and what, what. Come so that we could perform these and that, for you. If you could agree and not take medication, then you die in that way. Take these things [medication] then you’ll get healed.”

I: Okay. No, you could explain that thing fully when we procced.

P: [laughing]

I: But by the time you were using these stickers, had you ever sent an SMS more than once a day?

P: No – once – you drink them in the morning, right? Immediately after drinking your pills, you take your phone, “I did intake.” You remind them you had intake. Yes, once.

I: Oh

P: Ha! [laughing]

I: Uhm…When the box reminds you that this is the time of medication –

P: Yes

I: Did you ever open it more than once a day?

P: I opened it once, then I took the treatment, it repeated itself ringing again after opening. So, by that time it repeated ringing, I ignored it and it kept on ringing, it did not stop, then I concluded by saying, “a! I open it.” Then I opened it and closed it again. Exactly. I also brought this complaint here at xxx [clinic name]. I came and explained to them, and they said maybe the battery is about to die.

I: So, how often is it happening where you would find the box being opened and ring after ten minutes, then open it again and drink, then after ten minutes ring again – how often was that happening on a day or a week?

P: No, it was happening – like, on a week it might happen maybe twice a week.

I: Mmm.

P: And there was a problem, like if I could open it before eight –I could open it and take my treatment, right? –

I: Yes

P: Then closed it – exactly eight o’clock it would ring – remember I opened it before I took my medicaton and finished. Thereafter, eight o’clock it started again saying “open me.”

I: So, mama, how were your feelings, when you took your medication at eight o’clock but after ten minutes, rang again – how did you feel?

P: I was angry. The thing that was stressing me is that when it clocks (ring) on their side, they might think that I’m not drinking my medication. Do you get how it is? I was being stressed by that matter because they might think that I’m not intaking my medication – the first thing that I was doing when I enter here at xxx [clinic name], I was explaining to them that I’m being bothered by this box, I take my medication at eight in the morning, but ten minutes after closing it, it rings again. So, I started being stressed as they could assume that I’m not intaking my treatment now – so, if I don’t intake the treatment whereas the life is mine, to whom am I doing a favour to? Do you see it? I was explaining when arriving here. They said “no, as long as you’re intaking your medication, there is no problem.” And I was also worried about this person who was keeping on calling me without stopping, I should make sure that she checks what? – my urine, that it has been dyed by the pills, so that she could believe because sometimes she was saying “maybe you don’t intake them on time.” Then I said, “I take my pills, I don’t make mistake, I drink my pills.”

I: So, earlier, you said, when you were washing the dishes on the sink–

What was being released by the box?

P: It was lighting the green colour.

I: Oh.

P: Mmm

I: So, on top of that box, except the greenish of the light – what was helping you as an indication of time for intaking the medication?

P: It was ringing. It rings automatically.

I: Oh.

P: Yes, itself, hey, “tswikitswii-tswii” (tone of a box), and I don’t put my box far away. My house is RDP, it’s four room house. When I’m at the sink , it is on the couch.

I: Okay.

P: Mmm, it stays on the couch. When it started saying “twii-twii-twii” (box tone), I have already heard it. I would leave what I was doing and sit down, it is the time to give my box attention, it needs attention.

I: So, mama, you said you stay alone.

P: Yes, I live alone.

I: And are you being reminded by the box to take your medication?

P: Mmm

I: So, without this box, what else was reminding you ma’am that this is the time to intake your medication today?

P: No, I was not understanding, there was something coming on me saying “look at the time,” then I could take my phone and check the time, “Ah! I forgot to take my pills.” It is the same of these ones of the night – these ones of the night, don’t have a box, right? Exactly, I’m checking through a phone. It is just that the pills are apparently the drugs or what, I don’t know, they are apparently *nyaope* (another type of drugs) – it reminds you that this is the time. When you check the time, you find that surely, it’s the time for medication.

I: So, since you started taking your medication or using the box – is there any other day you missed to take your medication?

P: Never, it never. Even last week by the time I came here – last of last week, when I was here on Wednesday to ask them to give me the pills and to take the blood, so that I could come back to check the results – they said to me “no, we should not give you the treatment, we are only waiting for your blood results, so that you could be discharged.” I said “It’s impossible, I won’t stay without drinking my pills. Never.” As for pills, I had never. And I also don’t want that mistake.

I: So, earlier you said, when you send an SMS –

P: Yes

I: When you open a box, it reports to xxx [nurse name] and then they could recognize that you drank your medication –

P: I drank. Yes

I: So, does mama know that when she drank the medication or send an SMS, how does xxx [nurse name]recognize that mama sent an SMS today?

P: She told me that she registered me on the phone, it clocks (reports). I have been told by her, I had never seen it, as some of us doesn’t understand these things of your tablets, right? We don’t understand them.

I: Okay

P: Mmm

I: There is something that we call adherence calendar.

P: Adherence calendar? Put it in Sepedi language.

I: A calendar, it is a calendar.

P: Calendar used at home?

I: Yes, a calendar.

P: Exactly

I: And then adherence means – to adhere is to – when we give you the medication, if you are intaking it, that means you are adhering.

P: Okay

I: If you don’t take it, that means you are not adhering.

P: Okay

I: So, that calendar indicates that mama intakes her medication. On this day mama intakes, on this day mama intakes, on this day mama didn’t intake.

P: Okay

I: So, that calendar indicates that mama intakes her medication. On this day mama intakes, on this day mama intakes, on this day mama didn’t intake.

P: Okay

I: So, do you know the calendar that we’re talking about?

P: I don’t know it.

I: Okay. So, this adherence calendar is obtained on the tablet. It is the one that shows xxx [nurse name]if you took your medication or not.

P: Okay

I: Okay. So, I’m sorry to hear that you don't know the adherence calendar. So, there is something that we call differentiated care model here on our side. But briefly, it’s an SMSs, phone calls, home visits. So, you receive an SMS if you did not take your medication. If you did not take your medication – let me, say now – two hours later you get an SMS that says “take your medication” –

P: Yes, yes

I: There is the other one that if you skipped your medication for two days then we call you-

P: Yes

I: The is another one that if you skipped taking your medication for about a week, we’re coming at your side – we send the people where you are residing.

P: Mmm.

I: Do you know these things mother?

P: Yes, I know them, but they had never happened to me. As for me, I’m taking my medication. It is just that when coming for medication- I’m straight like the tail of a mouse, and I had never made a mistake for the date of visiting the clinic. I had never. Now, I was supposed to come yesterday. I had been phoned by xxx [nurse name] on Monday, and said that you’re coming on Thursday – so, I should come on Thursday and cancel that date of twenty-fifth of Wednesday – I should come today. And yesterday, she called me and said, “I’m requesting you not to come early in the morning that you were used to come when visiting for treatment. Don’t come early because you could wait for a long time, these people are coming from Joburg. So, you may leave your home at around eight, so that you can meet with those people because at that side you are just going to collect only the results.” She did not know that I wanted to see the doctor, the blood, things like those ones, she was just aware of the blood results only so that I might been discharged. Do you get me?

I: So, for my issue of an SMSs –

P: Yes

I: Have you ever received any SMS saying, “don’t forget to intake your medication?”

P: “Before twelve midnight”

I: So, had you taken your medication when you received the SMS?

P: I had taken it already. I take it in the morning, then maybe SMS could enter around six in the evening, I don’t understand what’s going on here.

I: So, according to your experience, I’m requesting you to share your feelings – you continued to receive an SMS while you had intake your medication – how was your feelings?

P: Eh! (Expression of amazement) I felt very crossed. I felt crossed to a point where – there is the other lady that I used to share with – I went to that lady, “Mama” “Mmm” “I’m sick. I’m the one who provoked them at xxx [clinic name] clinic, they are not the ones who followed me, but I’m the one who went to them. So, I receive a treatment – I intake my treatment, but every evening I receive an SMS saying, “don’t forget to intake your treatment before twelve midnight.” I also told xxx [nurse name] about this problem. I said “ xxx [nurse name]! I have a problem. I intake my treatment in the morning at eight – so, I will end up doing mistake of doubling them, by taking them twice. I intake my medication in the morning by eight, after intaking my medication – around six in the evening, there comes a message saying, “don’t forget to intake your treatment before twelve midnight.” Xxx [nurse name] responded to me by saying – but I have seen that she transfers me to school. She said to me “no, mama. It’s a message for this box. Maybe it had been used by somebody who was not well adhering.”

But I wanted to know why this message should enters in my phone. Do you get me? When I arrived at home I explained “ xxx [patient aunt’s name], I told them about the problem that I was telling you, the one-off receiving an SMSs that says so, so. And that girl responded me so. You know what, that lady laughed a lot. She said “Iyo! She was playing with your mind; she didn’t want you to go further with her. How could message for someone’s box enters on your phone?” I said I had also noticed that she is kidding me – let’s get out of things, as long as I intake my treatment. I was the one who felt pains, so I’m the one who should consider myself. The fact is to know that I intake my medication, no problem.” She then said “hey, be out of them man.”

I: Okay

P: Yes.

I: So, have you ever received a phone call, requesting you to intake your medication?

P: I had never received a phone call. I had never.

I: And then where you reside, had the people ever came?

P: People never came where I reside. They never, totally.

I: So, previously, you talked about witchcrafts illness –

P: Mmm

I: Then I said I’ll ask you about it, so now we arrived at that issue. So, [laughing]

P: You are worst, right? Maybe you will heal me with something. Mmm, let’s go [inaudible segment]

I: There are some other things that we call them barriers. So, barrier is something that could stop you to use the box –

P: Yes-

I: Or to use the sticker, or to intake your medication. So, mama, according to your experience or this matter of witchcrafts illness that you talked about, what do you think could stop a person or you to use this box?

P: The friends. You know what? If I had taken the directions of friends, I don’t know where I would be now. I don’t know if I would be still alive or dead because of my friends. They became angry because of my cough, the way I’m coughing. You heard me the way I coughed, then you have also taken care to help me to get some water to drink. Once that cough arrived, I cough repeatedly, if could not vomit, then it won’t stop –when started vomiting, she will say “you see? Do you see these things of your pills? This thing is witchcrafts illness, I just feel it that it witchcrafts illness. If I was you, I would have been out of them (pills), it is witchcrafts illness, right?” That one is a traditional healer, I believe in her, she is from ancestral school. Then I depart myself from the treatment and focus on witchcrafts illness now. Do you know what witchcrafts illness is? She needs my money. She needs money, she’s on a job that person. She went to ancestral school, so that she could work, by telling the people lies, right? So, I told myself that I commit with the one I’ve already committed to. I’m the one who decided to go to the clinic, so that they could check if this cough is a TB or not, right? Because of, TB, you can feel it even when you walk, you feel exhausted. I was exhausted, feeling that this sides are straining abnormally. And when you cough you feel that the ribs, no – here, I should go to xxx xxx[ clinic name]. And sometimes I had that thing of saying at home there is this thing of TB, do you understand it? So, how come can it attack the elders and leave the youngsters behind? It’s impossible, this is hereditary diseases at home. It goes like that. Even our new generation will have it at home. Even when I was telling my aunt (paternal one) about this issue that I’m infected with TB, she said to me, “even one of these kids was intaking the treatment, here she is, and she’s recovered.” She showed me her daughter.

I: In her home?

P: In her home. Do you understand how it is? She said “no, even this one had been infected. Weren’t you aware of it?” I said, “no, xxx [aunt’s name]had you been infected of TB?” She said “no, sister xxx [niece’s name] I was taking TB treatment here at xxx [clinic name].” I said “no, I know nothing. I’m not aware.” She said, “here she is, and she is recovered, you will recover as well.” That was the time we recovered that who, who, had been infected with what, what, so, so. Had you ever seen it? Yes.

I: So, going back to that issue of the barriers –

P: Mmm

I: What else could stop a person from using a box or to take TB treatment?

P: A person should not be stopped by another person to take the treatment. But according to my view, without the friends, no one could stop taking the treatment without getting false information from the friends. I discovered it myself. As for me, friend lied to me, she eventually got angry with me to the point where we ended up not checking on each other, the way we used to before, do you get me? And now her husband is having it, he is drinking, she doesn’t remove witchcrafts illness from him. On her husband it’s not witchcrafts illness, it’s TB –to me it’s witchcrafts illness, is it possible? [laughing]

I: So, mama. for me to get you clear or to understand you –

P: Yes

I: The person who was telling you to leave the medication, so that she could remove witchcrafts illness from you – does her person (husband) use TB medication?

P: For TB.

I: But she was telling you to leave the medication?

P: Yes, she was telling me to leave the medication. And she was also coming to me and say, “my husband is infected with TB, you know? They have given him a box as well.” She also said, “and he is coughing exactly as you do, he is vomiting as well.” I said “no, is it not witchcrafts illness? Why don’t you check him, so that you can find out if it’s witchcrafts illness or not?” I also said to her “I’m no longer taking those pills because it’s witchcrafts illness, I’m waiting to get money then I could come to you, so that you can remove it.” She didn’t even reply, she stood up and left. The way we were so close, right? – Immediately when she awakes, she could come to me “sister, xxx [patient’s name] may you please make me a tea. I don’t even have a relish at my house.”. She will come holding a small container “may you please serve me with rice, I’m craving for rice, I don’t have rice there.” She’s no longer coming now. We have fought because of this problem – she said I have witchcraft illness.

I: Iyo! I’m really sorry to hear that.

P: [laughing] That’s a person on this world.

I: Yes

P: A person on this world will make it for you.

I: Okay, for us to conclude – so, mama (mother), did you feel happy about this box? If you could tell me your feelings about this box, what could you say?

P: [laughing] As for me, I’m very glad for having this box, as you hear me now, I don’t want them to take it away from me whereas I’m still on the treatment. I should also finish this medication of mine. As for me, I like medication because it gives me life. Even though I don’t know what’s difficult to be clear, I’ll find out at the x-ray. But this box gives me life because it reminds me that hey, drink, drink [medication]. No matter how ignorant I might be – no matter if on the sink, doing something, man – by the time it started ringing, there is no other sound in my house, it is the only one with sound, do you get it? That’s why it reminds that hey, take your medication. Even that ten minutes, after eight, I hear it very quick. Just because at my side there is no TV, no radio – this is my radio. At eight, it's this one.

I: Okay

P: Yes

I: So, mama, I’m asking you to tell me your experience you had when using this box, and also about receiving an SMS reminder – the one that told you to take your medication. According to you, this thing of sending someone an SMS, when she doesn’t intake medication, and that one, of calling if she doesn’t intake medication [interrupted by unknown sound] – so, I was saying this thing of sending someone SMS, calling her, visiting her – according to you, are they the correct ways – are these ways working?

P: [coughing] They are working because of – others might not be exactly as I am, right? You will find out that the person had surely forgotten to intake the medication. When they send her an SMS, she can remember that I forgot to intake the medication indeed, then she intakes her medication.

I: So, according to your experience mama, what did you like and dislike about receiving this SMS?

P: What I didn’t like about receiving SMSs – I dislike them because I intake my medication and tell myself that dark or blue I should not make any mistake when coming to my medication. [coughing] But actually for those who don’t intake, it’s good to remind them. Yes, as for me I was intaking the medication.

I: So, now [phone rings] I’m going to wrap up, like we have reached the end, I’m about to finish. Had mama, ever received counselling?

P: To receive counselling?

I: Yes

P: No, I don’t know because I did not attend it.

I: So, counselling also involve taking to someone who doesn’t adhere to their medication, sit with her, and explain her the importance of medication – could the method work?

P: Yes, definitely [coughing]

I: So, out of all the activities I said are involved in this study –Visiting people, receiving an SMSs, and calling, and home visits. These activities – according to you, which one of these activities- which one do you think works better than all of them?

P: [sound of hand slightly beating the box as an indication of her answer] the box. A box is better than all because immediately, when you are about to forget because maybe somebody could visit after eight – so the box won’t make any mistake at its time. It’ll shaken you that man, let’s go, come to me, and touch me. I extremely prefer a box.

I: So, as mama, prefers and feels in this way about box. When you look at it, what can we do on top of it, so that it could be more improved and work better.

P: But from my side, it works very good in this way. It reminds you, right? So, for it to be better, do you want them to insert a cup of water aside? No, they won’t insert water for you, just mix your water and put it there, and when it rings, just open, take out your pills and take your water and drink. In such a way it satisfies me because it reminds you to intake medication.

I: So, by the time they were giving you this box or these stickers –

P: Mmm

I: Had you ever seen them before you recognize them here at the clinic?

P: No, I had never. I had never seen these things, I recognized them because of TB. Yes [phone rings], I said, “to go, is to see.” You will see things, you know? Seriously, I said these diseases will show you things. I started with stickers, I left them, I entered at the box. I recognize things that I was not thinking of seeing them.

I: Okay. So, here I just wrap up, so that we could finish our questions, so that we could reach the end of our conversation.

P: Yes

I: When looking at this study – including this box and these stickers – Is there anything that you think that no man, you should have done so? Is there any gap that you think we missed?

P: [coughing] no you missed nothing. [coughing]. [inaudible segment]

I: Uhm…we reached the end of our interview for today.

P: Yes

I: So, let me take this opportunity to thank you for coming and conduct this interview with us. Thanks, you for your opportunity that you have taken.

P: Yes

I: So, mama, I thank you.

P: No, I’m the one who give thanks.

I: Mmm

P: Thanks very much, but don’t expect that you can’t see me anymore. If I could find out from x-ray, that I’m not alright, I will restart the TB treatment. There is no other way, the way I feel myself, I could start it again if they could tell me to start it.

I: So, before we close this interview, like your final thoughts, your suggestions about this box and these stickers, your final one before we close this interview, what are they?

P: No, as for me I don’t see any problem. I just thank them [sound of hands slightly beating the box as an indication] the importance of this box only for reminding me, because as I’m also a human being, maybe I could forget when as I was busy doing something. Even the stickers, I don’t remember myself forgetting even a single day cause what I like about them, they had been plugged on the medication. Immediately when you open and take out your pills, here is the sticker, then you remember that I should send an SMS.

I: Okay.

P: Yes.

I: Uhm…mama, I’m thankful to hear your opinions, right?

P: Yes

I: Okay. So, but unfortunately, we have reached the end of our conversation. So, again, let me take this opportunity again to thank you for coming here to conduct this interview with us, right?

P: Yes

I: So, thank you. Time ended, it’s uh…12:53PM
