# Supplementary material for: Acceptability of Digital Adherence Technologies to support people with drug-susceptible TB in South Africa
Source: PLoS One. 2025 Sep 24;20(9):e0332103. doi: 10.1371/journal.pone.0332103 (PMC12459780; doi:10.1371/journal.pone.0332103)
Supplement: S4 File — (ZIP) [file pone.0332103.s004.zip › S4 Transcripts/PwTB/IDI 12_PwTB.docx]

**I**: Okay let’s see…. [inaudible segment]

**I**: Do you agree to be recorded for this interview?

**P**: Yes.

**I**: Okay, so [Sigh]. Date, xxxx (interview date). Location, xxx [clinic name ] Clinic. Language used, Setswana. P ID, xxx. And then the time is 11:15 am. Okay.

**I**: So, sir please explain to me, when did you start using the label or the sticker?

**P**: [Sigh]… 11^th^ of November 2021.

**I**: The 11^th^ of November-

**P**: 2021.

**I**: Last year?

**P**: Yes.

**I**: Could the 11^th^ of November be the day you started treatment?

**P**: Yes.

**I**: Okay. So, how did you feel when they explained to you about the label?

**P**: I felt proud because through the label you become updated with regards to drinking your medication.

**I**: Okay, when you sir say you become up to date, please explain fully what you mean by saying you become up to date.

**P**: [Sigh] it’s with regards to the fact that when you do not drink, it means you would not be able to send an SMS.

**I**: Okay

**P**: Yes

**I**: And then when you have drunk?

**P**: When you have drunk you send an SMS, according to our agreement in the beginning regarding this sticker.

**I**: [Sigh] Okay. So, may I please… So, before you started the treatment what encouraged you sir to come test for TB?

**P**: It is how my life standard were. I was confused, I was sweating, I had no energy, did not eat and had no cravings or no appetite for food.

[Pause]

**I**: Okay, so you sir spoke about the symptoms of TB. You spoke about sweat and appetite.

**P**: Yes.

**I**: Besides these two symptoms, were there others in the body that you sir know besides the two you have mentioned?

**P**: It’s lack of energy.

**I**: Okay [Pause] Fatigue. Okay, another?

**P**: Not eating.

**I**: Appetite, okay, thank you. So, how did you feel when they diagnosed you with TB?

**P**: I was a bit hurt but because it has cure, I did not take it too much into consideration and I told myself that I will be healed.

**I**: Okay.

**P**: You understand?

**I**: Mmm.

**I**: So, when they explained that TB has cure, what was their explanation in case you do not take your TB medication, what will happen?

**P**: Yes, when you do not take TB medication, I think one can even lose their life.

**I**: Okay, so the time they gave you this label or this technology, who explained to you Sir about this label?

**P**: A sister (nurse) explained to me whom I forget her surname at the moment, but she is on duty.

**I**: Okay, was it sister or an RA who told you about the label? An RA is an intern from xxxx (organisation name)?

**P**: [Pause] I was told by… I think they were two… No, I was told by the sister, who assisted me with my file. Yes.

**I**: So, the sister is the one who explained to you about this label?

**P**: Yes.

**I**: So, how long did the explanation about the label take?

**P**: Less than 5 min.

**I**: Okay, so even in the manner they explained to you sir, is there something you would like to change from the way it was explained?

**P**: There is nothing.

**I**: Okay [Pause] So, by the time they explained about the TB illness, please explain to me something you know about TB.

**P**: What I know about TB is that it is curable, and you need to take care and making sure you take your medication at the time you were told to take them; in the morning.

**I**: Is it that you take the medication in the morning at different times or how do you do it?

**P**: At the same hour, each day.

**I**: Okay. So, [Pause] at the time they started you on TB treatment, even when they told you about this technology or this sticker, have you ever seen this sticker elsewhere beside at the clinic?

**P**: No, I only saw it at the clinic.

**I**: Okay. In short, could you sir please explain how you use this sticker.

**P**: After drinking your medication at 8am in the morning or 9am, you SMS the number [inaudible segment].

**I**: Okay, so when you say this number, please call the number out.

**P**: Number like let me say 115 after drinking, you SMS or text it to the one on top.

**I**: Okay, so in short, at what time do you sir take your medication?

**P**: I drink it at 8 o’ Clock every evening. TB medication I drink at 9 o’ Clock only in the morning.

**I**: So, your TB medication is the one you drink at 9 in the morning?

**P**: Yes

**I**: Okay, so how do you sir feel when drinking your TB medication?

**P**: I feel much better since I have started drinking it.

**I**: Okay. When you say much better, please explain do you mean feeling well or much better?

**P**: I feel… healed in other words, or I am being healed compared to the state I was in, I do not know if you understand me in the beginning. I started off very weak-

**I**: Yes.

**P**: Yes, so now I am strong like all other people.

**I**: [Pause] Okay. So, when you say you were weak [inaudible segment]**-**

**P**: Weakness, I was weak, and I step strong now **–**

**I**: Oh, you strong?

**I**: So, when you say you were weak, explain to me in short like how would you work during the day, like on a day-to-day basis how would you work while you were weak?

**P**: I would go outside, because most of the time I would be indoors, I would sit for no longer than 20 min on the chair while I’m with other people. I would stand up because I would be tired, I would feel tired. Then I would go sleep, when I go sleep within another 10 to 15 min, I would feel a breeze or feel hot, I actually would not understand the situation I was in, you understand**-**

**I**: Mmm.

**P**: Yes

**I**: And then now that you sir is better or strong, how do you feel?

**P**: Oh, I feel happy at the moment.

**I**: So, at the time you were weak, when you go out, you would feel as if you this side and then after 10 min you are this side and then after 10 min you are sleeping, and after so min…. so, now how do you feel overall?

**P**: I do not sleep anymore; I no longer have the weakness of sleeping during the day.

**I:** Okay

**P**: Yes

[Pause]

**I**: So, my other question, when you sir say you drink your medication at 9 in the morning, is it because you are working?

**P**: I do not work.

**I**: Okay. And then, how do you take the medication if you have somewhere to go in the morning?

**P**: If I have somewhere to go, I take the medication with.

**I**: Let me say [Pause] there are things we call peace jobs**-**

**P**: Yes **–**

**I**: Yes

**P**: Okay, Jobs that we have aside. I understand you. I take my medication with.

**I**: Oh, you take your medication with?

**P**: Yes, I drink it there.

**I**: So, and then these peace jobs, how often do they come in a month

**P**: For me, I have never done them **–**

**I**: Uhm

**P**: I have never done them, I have only done them in the area I leave in, not around the community or going to other companies.

**I**: So, you worked in the house.

**P**: It’s something like gym, I would refer it to them, just to prevent me from just sitting.

**I**: Okay

**P**: Yes

**I**: So, at the time they explained to you that you have TB, at home is there someone you explained about TB and that you have been diagnosed with it.

**P**: When I came back from the clinic, but they also saw how I would sweat, the tiredness and the lack of appetite, you understand. Then when I came back from the clinic, I told them I have TB, that I am diagnosed with TB. And they also saw with the medication.

**I**: So, at the time you told them that you have TB, how was the reaction of them in the house that you have TB?

**P**: They just took it as that I must look after myself and I must spend most of the time in the bedroom, the bedroom I sleep in. They would bring me food in the bedroom.

**I**: Mmm

**I**: Okay, it’s the time that they saw you have TB?

**P:** Mmm-

**I**: And then when they saw the TB medication, how did they take it?

**P**: They were happy because they knew that eventually I will get healed and I didn’t think that death will appear, as long as I am on treatment, I will get healed.

**I**: Okay.

**P**: Mmm

**I**: So, this means you received clear explanation that with TB you can be healed.

**P**: That’s what I got.

**I**: Is there any support you receive at home to make sure that you take your TB medication?

**P**: Yes

**I**: Okay, I’m glad to hear that.

**I**: So, in short, what kind of support that you receive?

**P**: Ehhh, the support I get when I come to the clinic is that they make me porridge even before I drink my medication and again, they transport me to the clinic with a car. You understand, such things. Same as today, I was brought here with a car and they will also pick me up.

**I**: So [Inaudible segment] when you come to the clinic, what do you use?

**P**: I come with a car; they bring me with a car-

**I**: And to go back?

**P**: They come pick me up with a car.

**I**: Okay. Do you sir stay far from the clinic?

**P**: [Inaudible segment] 7km away from the… even though I do not know the distance in kilometres but I think so.

**I**: Okay

**I**: So, when you take the TB medication or when they give you the TB medication and when they explain about this sticker or label, did you have any worries about taking your TB treatment with this sticker?

**P**: No, I did not have any worries because I took it that the nurses who were assisting me knew what I will benefit at the end if only I follow what they state according to these stickers.

**I**: Okay

[Pause]

**I**: So, at home, did you give them an explanation of this sticker?

**P**: They know, I did explain to them.

**I**: Okay. So, when you explained to them about the sticker, what was their feeling?

**P**: They were happy, they were happy at the fact that I would not be able to skip my medication, or say I did not drink my medication whereas I drank it. And even where I go at the clinic, the SMS should also appear on their side.

**I**: Okay. I do not know sir, but there is something we call adherence calendar, so when you take your medication, we can see that you have taken your medication today, do you know?

**P**: [Pause] Take it and drink it or take it and drink it where I stay?

[Pause]

**I**: When you send an SMS to the tablet, we can see that patient 4015 that today he has taken their treatment, do you sir know about that calendar?

**P**: Yes, I know that calendar.

**I**: In short, would you explain to me what you know about this calendar.

**P**: What I know about this calendar is that when you make use of it, it becomes evident that you drink your medication and you drink it at the correct time.

[Pause]

**I**: Okay, besides this sticker or calendar, do you sir have any other support he gets to make sure that you have taken your medication or [inaudible segment]

**P**: It’s when the medication is finished, it gets finished.

**I**: Okay. So, when you come to collect or when you come for a refill, do you come with the medication or you leave them at home?

**P**: I come with them.

**I**: When you come with them, what does the sisters (nurses) do with them?

**P**: They take a look at them, they read them and then they would know which medication do they need to give me or which they need to refill.

[Pause]

**I**: Okay. So, because you use this label or sticker, have you ever experienced any challenges because of this label?

P: Ehhh, there was a day when these numbers did not want to send when we try to send.

I: Okay

[Pause]

I: So, you were explaining… Please explain before you say these SMS did not send…the SMS were not delivered.

P: I thought it was network but I would say when I come, I called here to tell them the SMS was not delivered and they told me that I should keep trying, you understand. I would change that number {code} and send the other and then it would go through.

**I**: So, when you send the SMSs and they do not go through, how often does it happen?

**P**: Ehhh maybe 3 times a month… [inaudible segment] not that it is an everyday thing. Or like when I am out to other places, maybe it’s the network of the place I’m at.

**I**: Okay. So, the messages that are not delivered are you still receiving them even now?

**P**: No, it’s been a while not getting them.

**I**: So, at the moment you are good?

**P**: Yes, at the moment I’m good

**I**: So, before I move on to my concern can I go back a bit. So, at the time they started you on TB treatment or when they started you on treatment, what did the clinic do to make sure that your family are prevented from TB?

**P**: They sent-I do not know whether it’s a market group at the house to come take saliva samples of those who were there in normal bottles and then after 3 weeks we received results that there is no one infected, then I carried on with taking care of myself and making sure that they are safe.

**I**: So, what were your feelings when you received news that no one in your family is infected with TB?

**P**: I was happy, I felt happy because we cannot all get sick in the family, you understand, it has to be that when one gets sick, he saves the others according to the illness they have which is infectious. I spent most of the time in my bedroom, you understand?

**I**: To make sure that others are safe from this infectious illness.

**P**: I even use my own utensils to drink water and to eat. Specific things, you understand-

**I**: Mmm

**I**: Besides immediate family, is there anyone else who you told you are taking TB medication?

**P**: It’s my wife and she knew.

**I**: She knew?

**P**: Mmm even the kids know.

**I**: Besides them, friends or anyone?

**P**: With regards to my friends the honest truth is I have not told any of them.

**I**: So, what made you not to tell your friends that you have TB?

**P**: You know friends even after the TB is cured, they fear people with TB, you understand? I even see at work they always run away; they do not say you are cured they run away when you come near them, you will just see when they walk out when you enter to greet them, they all walk out, you see?

**I**: So according to the experience you have of TB could you say it is not necessary for our communities to fear this illness?

**P**: It is feared because a lot of people still fear it… because when it enters it seems like it goes according to how strong your blood is, you can get extremely worse if you do not go to the clinic as soon as possible. I come a long way with it, I know. I was at a point whereby I was even failing to get off the bed, I weighed 37kgs, you understand? But currently I’m around 50 something… 53kgs you understand, even my voice was no more there it was so low, it takes one’s voice, it will be gone.

**I**: Okay, at home is there anyone who had TB before you?

**P**: No, there is no one.

**I**: Okay… by the time they started you on TB treatment did they explain the side effects of TB medication to you?

**P**: Yes, they told me about getting dark, to get dark like the way I am it’s TB medication [inaudible segment] it’s the ones that did this to me, it’s not my colour. You see even these brown dots are not mine, so these are the things I have encountered…. And I even had [inaudible segment] by my feet, even here, here are they, these hands have no lotion like dawn and glycerine and those things I am not fully healed, even in my face I’m a bit healed I was pitch black, you understand?

**I**: But even though you are like this you stuck to the TB medication?

**P**: Yes

**I**: Reason being?

**P**: Reason being I wanted to be healed, and the side effect that I almost forgot is the eyes, my eyes are changed, they are changed in colour, they are yellow in colour, two weeks ago I even went for liver blood tests, the results are back, I’m going to get them, they are in there, I’m sure **–**

**I**: [Inaudible segment]

**P**: They are in their book. In fact, I’m here today to fetch them.

**I**: Okay. So, did you send the SMS more than once in a day?

**P**: I only send once in a day, and when it does not go through that’s when I retry but officially we send once a day.

**I**: Okay. In short, when do you send that SMS?

**P**: You send once you drank your medication on time.

[Pause]

**I**: Is there a day where you sent an SMS without drinking your medication?

**P**: No there is no day.

**I**: Okay. Is there let me say a day whereby you did not take your medication on time from the time you started TB medication?

**P**: There isn’t.

**I**: When you say there isn’t…….

**P**: I mean I haven’t experienced it because there isn’t a day, I could say there was no electricity and couldn’t boil water, you understand, I drink at my correct time.

**I**: Okay. So, you just spoke about load shedding, so when you have load shedding how do you send the SMS?

**P**: That is when it would not be delivered.

[Pause]

**I**: So, my other question is about the difficulties, so already you have explained one which is electricity that when you have load shedding it affects the network and the SMS does not get delivered, is there other difficulties you encounter besides this one related to electricity?

**P**: No, besides the electricity one there is none, unless I do not know, but no there is nothing.

[Pause]

**I**: Okay

[Pause]

**I**: So, according to your experience when you take medication with this… you spoke about a calendar but to take medication and to take medication with this sticker, what is more helpful about taking medication with this sticker?

**P**: I do not understand.

**I**: Okay let me rephrase like this, there are those who take medication who do not have this sticker, they just take medication, and there are those who take medication with this sticker, how is it helpful to take medication with this sticker?

**P**: It is helpful in this manner, when you have this sticker wherever you go where they do not know you or where they know you work with their department, they are able to know that you are up to date, you understand? If you do not have this sticker and you say you drink your medication at 9 o’ Clock, how will they know that you drank? They wouldn’t know.

**I**: Okay. [Pause] As ASCENT, which is what we call this study, or as xxxx (organisation name) there is something we call a differentiated cared model, whereby a patient receives an SMS, so have you sir received an SMS?

**P**: From the stickers?

**I**: Mmm

**P**: Yes

**I**: Oh okay. So, what did the SMS talk about?

**P**: SMS… you know I deleted it, but I can’t remember what did it say I deleted it, but I remember it, I deleted it saying why would this SMS come through to me as I am always up to date.

**I**: So… do you know when the SMS is sent?

**P**: When you are not up to date with drinking your medication.

**I**: So, about getting an SMS, how often do you get an SMS?

**P**: It is controlled by your use with the medication and the SMSs you send.

**I**: Okay. But you according to your experience, how often do you get these SMSs?

**P**: I only got it once…. Until I came here and told them I wanted these things to be used by my girl, you understand?

**I**: I do not understand the part that they should be used by your girl **–**

**P**: Yes, so that she can update them for me because I do not use an advanced phone, maybe with a smart phone the network would be quick, you understand?

**I**: So, when you sir take your medication now, is there SMS sent by you or by your girl?

**P**: No, presently within a space of a week it has been her who has been sending the SMS however all along I have been the one sending, she started last week, that other week, yes. Now I asked her to do me that favour and send because even children step on my phone so I thought maybe it has signal problems, you see?

**I**: So, how does your daughter know that you took your medication?

**P**: No, this one is my granddaughter that I am talking about.

**I**: Okay.

**P**: Most of the time she’s by my side, after drinking my medication she starts sending, she cannot send before. She waits first will even bring me water and say uncle it’s time, because I did not get that white box which they said was an alarm, you understand?

**I**: Okay

**P**: So, we have set alarms on our phones, they even rang at 9 o’ clock while I was sitting in here.

[Pause]

**I**: Okay. So, when you say your granddaughter is the one who sends the SMSs, how old is she?

**P**: Ehhhhh… she is 33 years

**I**: Okay. So, when she is not around how do you manage to send the SMS?

**P**: Ehhh… I have a landline of that department, when she is not around, I just call TB department and ask if they received an SMS and they say “Yes we received” because these ladies are always on the road, but when she leaves she tells me she’s going out and she’s coming back, you understand? Even now she was the one who was busy calling me

**I**: Okay. So, how often does it happen that you call the TB department to ask if they received the SMS?

**P**: It is less than 3 times, yes, I haven’t called more than 3 times, I just called 2 times because where she stays, she said she’s in Mpumalanga as she said she is running a business of feeding stations, seems like its 3… 4 in fact. So, she told me where she is there is no network and the SMSs are not being delivered that’s when I took the phone and called here, you understand. Then even the date they gave me, I can’t remember last week then I told them I am going to come personally, I asked them when things are like this how do we work then they said no you not the only one, a lot of people have been calling saying the SMSs do not want to be delivered. These numbers rightfully have to circulate, when they circulate, they are only 3, it is 115, 111, and what else 777 or 515 you understand, those I remember I had.

**I**: Okay. So, this one related to the codes I will ask as time goes.

**P**: Okay

**I**: So, we have SMSs and phone calls. Have you received a phone call from the clinic?

**P**: Yes, they call me to update me about my next visit.

**I**: Okay. So, phone call related to you not drinking medication have you received it?

**P**: That I should come get my medication?

**I**: That you not taking your medication?

**P**: No, I have not received it.

**I**: Okay. So, the third we have a home visit. Have you received someone from the clinic to do a home visit where you stay?

**P:** They came once.

**I**: So, in short, what happened during that home visit?

**P**: They came within 3 weeks after I have started with my treatment, they came and did what…I was still confused at that time. All I remember is that they left 50 rands and said it is mine to buy fruits, apples and bananas. Also, to check if my feet were swollen because when you start treatment people’s feet become swollen, some even have difficulties with getting off the bed, so mine were not swollen.

**I**: Okay

[Pause]

**I**: Okay. So, is there any barrier or anything that could stop you sir from sending the SMS?

**P**:

Load sheddding could stop me from sending the SMS

[Pause]

**P**: It does not get delivered.

**I**: Besides load shedding what is another one?

**P**: I cannot remember, because I cannot say the batteries would be low, no, because every day when we sleep or when they sleep the phone are always on the charger, you understand.

**I**: So, the fact that your granddaughter is at times in Mpumalanga and at times it is network, so, at time doesn’t it disturb her to send the SMS?

**P**: Disturb her how because most of the time she’s at home, she just goes there to check up on business, you understand, and then she comes back home.

[Pause]

**I**: Okay, so going back to the SMSs. How would you feel when you receive the SMSs?

**P**: No, I would feel happy still because it meant my things are going accordingly.

**I**: And then that of receiving a phone call?

**P**: That would be reminding me?

**I:** Mmm

**P**: Even with that one I would feel alright because it showed that I am being taken care of, I am not taking care of myself alone, you understand, even other people are taking care of me.

**I**: So, in terms of satisfaction, to have this label that illustrates that you have taken your medication even these people who call you other days who care for you, how satisfied or how is your level of satisfaction to have this kind of support in all ways?

**P**: Eh it satisfies me but that satisfies me mostly is when I am not experiencing network challenges when I use this sticker because it is a daily thing, those at the clinic do not call every day.

[Pause]

**I**: Okay. So, here please tell me what you think because I told you before that we want your level of satisfaction, experience and your thoughts. So now can you in short explain what do you think of this label?

**P**: I think this label is helpful, and it’s helpful in this manner, it helps even in the clinics as they can see you drink what you have to drink even when you are where ever you at.

[Pause]

**I**: And then you said ever since you started your medication there is no day which you have skipped your medication

**P**: No there isn’t.

**I**: Okay. So, for those who are not taking medication do you think this label will help them to take their medication?

**P**: Those who are not taking medication are doing what?

**I**: This label would help them to adhere to their treatment?

**P**: Only if they do not skip their medication, because this SMS you cannot send if you have not taken your medication, if you did not drink you did not drink, you cannot send an SMS. You understand?

**I:** Mmm.

**P**: You only send when you drank your medication and by drinking it means you are looking after your medication.

**I**: Okay… So, according to the way you think, the approach or the way of using these SMSs is appropriate for patients to take TB medication?

**P**: Yes, it is appropriate.

**I**: Please explain fully.

**P**: It is appropriate because you can avoid problems related to your treatment, you drink it every day with this sticker.

**I**: So, this way of when a patient did not take their TB medication, calling them and reminding them to take their treatment is it appropriate?

**P**: No, it is not, it is not an appropriate way because you have to take care of yourself even if at the clinic… I mean when you wherever you at with your medication.

**I**: There is something we call tracing or home visit, were I say when a patient is not taking medication, we send people to go to them to check if the patient is still there or if the patient is still taking medication or not, is the home visit appropriate?

**P**: Yes, it is fine because they will even sit still in the house thinking one day… even though when they come, they call, they would not just come because it can happen that you went to the shops, they call to ask if you are available at home we will see you at 2 or at 3, hence I feel it is better that way.

**I**: So, when we conduct home visits, what could be your advices you would want to share with us so that we can follow to make sure we do not irritate or make the patient angry during the home visit?

**P**: Ehhh… I was thinking… I mean what I saw, when they come to visit us, they should not go much into our bedrooms because I saw one of the team members during the home visit sitting on my bed, you understand? Whereas I was busy watching TV, while they were asking me questions, they were even writing the way you are writing. I would have preferred it if they stood at the door… You understand? And it seems like they also look at how clean is the place, you do not stay in a shack, I do not understand there, but there I support them, that you do not stay in a dusty place, such things, you understand? And they ask if you have a fridge, a stove, such things.

**I**: So, Sir you were not pleased with the fact that when they enter your bedroom, they sat on your bed?

**P**: Yes, no there I was not happy. Like when I am sleeping, I expecting the lady to be standing there and say sir I’m here and say we work like this and we would like to know if such people are available, then I would agree with her, I would not disagree or prevent her. Then she would tell me we here for this and that, we came to hear how you feel, what’s happening, are your feet swelling, such things, you understand?

**I**: Mmm, okay.

**I**: Did you sir explain your feelings to them at the time they were in your bedroom?

**P**: No, it is not easy in that manner, it is not an easy thing that I could tell them face to face, it is not easy. I only explained to my sister that I did not like the manner in which they did things, but maybe they do not do such everywhere, you would find that maybe they were exhausted from morning and maybe she told herself that she’s resting a bit.

**I**: Mmm, Okay. So… So…regarding the SMS, phone call and home visit, to us during the study we refer to these as activities.

**P**: Mmm.

**I**: So out of these activities we have, which one do you think works with regards to making sure the patients are taking medication?

**P**: It’s… it’s the SMS.

**I**: Okay, let me explain… let me rephrase. We have that whereby when you have not taken your medication, we send you an SMS.

**P**: Mmm, okay.

**I:** I don’t know if they explained this to you.

**P**: These others I cannot remember well.

**I:** Okay.

**P**: But I will understand when you speak.

**I**: Okay let me explain to you, not this one, lets put this one aside. That one that when you do not drink your medication you get an SMS reminding you to drink your medication before the end of the day. There is that one that if you spent 3 days without drinking your medication we call you asking what is the problem please drink your medication, there is that one whereby you spend a week without drinking your medication then there is a home visit, this is where they come to your home to check if you are still alive, you are still there. So out of these three, whereby you get an SMS if you did not drink, the phone call and the home visit, which one is active, the one that works best?

**P**: Eh this one of the 5 days goes along with default, so I prefer the first one according to the way you read it.

[Pause]

**I**: Okay, so, you prefer the SMS?

**P**: Mmm

**I**: Why do you prefer the SMS?

**P**: It’s because if I take you back to the stickers it would mean you also would not be sending, you cannot send something you have not eaten, you understand, or that you did not drink.

**I**: I get you. So, out of this three again which one do you think does not work like the others?

**P**: Number 2, I can’t remember what you said it was.

**I**: Phone call

**P**: Mmm

**I**: Why do you think phone call out of this three is not working… it does not have much impact?

**P**: I think phone call is not an immediate thing, but when you SMS it is like a home visit they appear right, even though its after 5 days but an SMS reflects immediately.

**I**: Okay… [Inaudible segment]

[Pause]

**I**: So, when you do an SMS, Phone call and counselling, when you received a phone call did you receive counselling, through the cell phone?

**P**: No, counselling… through the cell phone, no I didn’t.

**I**: At the time they did a home visit did they do the counselling?

**P**: Yes, when they did a home visit yes, they’ve said something to me, regarding my illness.

**I**: Okay. So, how was the counselling or the manner they used to give this counselling how was it?

**P**: It was a manner which had questioning included, there were questions.

**I**: Okay… [inaudible segment] during our talk there is somewhere were you mentioned this box, so do you Sir know this box, because you are using the label one, but this box do you happen to know this box?

**P**: This box is an alarm box if I’m not mistaken, is it?

**I**: Yes, it is.

**P**: Yes, since I started coming here, they only told me they are finished, they will [inaudible segment] and as time goes it happened that the box does not appear and I never enquired again about it because I saw that the phone is also helpful as it also makes noise.

**I**: Okay. So, this box, was it your first time seeing it at the clinic or you saw it somewhere else before?

**P**: I saw it at the clinic.

**I**: Okay. So, I want your thoughts or opinions regarding these technologies.

**P**: Ehh… I think it is better if technology is put first and reviving for those individuals who are sick as me and for those who take treatment just like me because at the specific time you are reminded by the box to take medication, you see.

**I**: Okay. To go back a bit, where do you place your medication sir?

**P**: Ehh I have… I don’t know what to call that bag, but I have a small bag that is where my medication is stored, in a cool dry place in a small box up there, where I place my stuff and even medication for when I have flu or whatever, up there.

**I**: So, the box you place medication at, it is TB medication only placed in that box or other medication as well?

**P**: Up to so far it was only TB medication because all these other I was no longer drinking, I vomit them.

**I**: Okay… So, you place it up in the bedroom?

**P**: That box is in the kitchen.

**I**: In the kitchen at the top?

**P**: At the top.

**I**: What encouraged you to place the box on top?

**P**: They say it does not require heat or sunlight, most of the time place your things in a cool place.

**I**: So, to go back to the technology, we use the sticker as well as the box, so these technologies do you see as we go further do they improve the lives of people taking TB medication?

**P**: Yes, because when you have forgotten the box will make noise, I ever you have set it well for the appropriate medication time, you understand? The box reminds you in fact.

**I:** Mmm.

**P**: And once you have taken your medication you notify the ones in front that you have taken your medication, with this one, this sticker.

**I**: So, because you have been using this sticker or label, you have experience with this label, so what can we do to make sure we improve the way in which the label is working?

**P**: I was thinking maybe when the SMS is received on the tablet, which I do not know it will be in whose hands, maybe they could call same time and ask if it is true that you drink your medication or you are just sending for the sake of sending? It should be a question that requires a yes or no answer from me.

[Pause]

**I**: Okay, so according to the way you just spoke it means when you send the SMS you are not truly sure that the SMS is delivered.

**P**: Besides it being delivered, I can send it without drinking, you understand, it happens, I can send without drinking and it will reflect on your sending on the things you will be using…. That’s why most of the time I have people who are looking after me in my family to make sure I drink my medication at the right time. So, there is no way the SMS could be delivered without me drinking.

**I**: Okay. Besides the calling you back to make sure you took your medication, what else could be done or you can do to improve this label?

[Pause]

**P**: Ehhh. I was thinking when it’s the date you are called to improve the label, they should count your medication one by one, according to the calendar… they should know from the 1^st^ to the 15^th^ you should have taken how many tablets [inaudible segment] and count them to see whether you have been drinking your medication correctly.

**I**: Okay. So, we have reached the end of our interview. As I said it would take about 45min to an hour, so, could there be any final thoughts you have regarding these two technologies or the label one since you have experience with it?

**P**: I was thinking that they should not be stopped according to the way or manner they are utilised, they should be continued so that when people keep drinking their medication healing should be spread, because with these technologies they have motivators, some people behind them who have them, who belong to the clinic, when they are like this they are people of the clinic just like me, this box belongs to the clinic, these stickers belong to the clinic, so when you are alone you have to drink your medication when you look at these things, meaning, I take these as people.

**I**: Okay, I appreciate. And that one of the differentiated care model, that one of the SMS, phone call and home visit, what are your final thought about the model of care, the support we give you?

**P**: Ehh the support that is evident that I receive I see it as something which is important and did not interrupt me that much as the home visit was not an everyday thing, my basis are on it. Where I stay, they only came once, you understand, and the others are number 1 on me.

**I**: Okay. Sir we have reached the end of our interview, let me take this opportunity to thank you that you came and agreed to conduct this interview. So, we have reached the end, I thank you.

**P**: I thank you as well.

**I**: Let’s give thanks. So, time ended is 12:20pm
