# Supplementary material for: Acceptability of Digital Adherence Technologies to support people with drug-susceptible TB in South Africa
Source: PLoS One. 2025 Sep 24;20(9):e0332103. doi: 10.1371/journal.pone.0332103 (PMC12459780; doi:10.1371/journal.pone.0332103)
Supplement: S4 File — (ZIP) [file pone.0332103.s004.zip › S4 Transcripts/PwTB/IDI 13_PwTB.docx]

**TRANSCRIPTION NOTATIONS**

| **Label Key** | **Meaning** |
| --- | --- |
| **I** | Start of each new utterance by the Interviewer |
| **P** | Start of each new utterance by the Participant |
| **N** | Note taker |
| **{ }** | Indicates that details were changed or pseudonyms were used to anonymise data |
| **( )** | Indicates the description provided to anonymise data |
| **XXX** | Words were omitted to anonymise data |
| **-** | Breaking into a sentence by the next speaker |
| **…** | Pause or drawn out words |
| **[ ]** | Indicates noise made, e.g. [laugh], [sigh], [pause] |
| ? | Beginning of utterance by unidentified speaker or questionable text |
| **[inaudible segment]** | Unclear section of the recording |

I: I am requesting permission to record you.

P: Yes.

I: Do you agree to be recorded?

P: Yes, I agree.

I: Date xxxx (interview date), Location: xxxx (clinic name), interviewer XXX (interviewers name), language used Setswana, starting time 11:23 … Ok mama, where you are staying, who do you stay with?

P: My children took me in when I got sick, but I am now staying alone since I have gotten better.

I: So, where you are staying alone, are you able to take your medication on time?

P: I am the one who drinks on time… yes.

I: How far it is for you from here to where you staying?

P: I stay at xxxx (area name), I do not know the distance, but I stay in xxxx(area name).

I: So, how much do you pay in a taxi if you are using a taxi coming here or what are you using?

P: From xxxx (area name) to town, I pay R20 and then from town to here, I pay R17, but it is only for a single trip. I must pay again for a return.

I: So, what can you tell me about the box they gave you?

P: The box they gave me clocks, yes and I take medication at 05H00. It clocks exactly at 5 o’clock-it rings to indicate that it is time for me to take pills.

I: Who explained the box to you?

P: It was the sister when I came. I was too sick; I was on and off because the sister who was taking care of me is the one who explained to the child as I was too sick.

I: When you say you were on and off, what are you referring to?

P: The child explained that the sister said to her I must take pills at this time.

I: So, which 05H00 were you taking medication?

P: Late.

I: You mentioned that sometimes you remember and sometimes you don’t, what reminds you time to take medication?

P: Sometimes the child takes care of me when am sick, yes.

I: Ok… have you ever missed a day without taking your medication?

P: At first, when they gave me medication. I had given up thinking I will not get healed, so I would put medication under the bed when they give me.

I: Ok … how often would you do that?

P:Mmm several times but I do not know how many times exactly. I would take and sometimes I would not.

I: I am asking what made you think you will not get healed?

P: It is the way I was so sick; I was sick, I even used a wheelchair- I was weak, let me say so

I: How did you feel the time you were weak, and you could not walk?

P: I felt like I could die.

I: What changed?

P: What changed was that there is a child at home who set down with me and told me that I should not give up on my life as there are many people who takes pills and are not dying, they get healed.

I: How did you feel when they told you that you have TB?

P: At first, I had accepted that I have TB and they also counselled me; they told me to do everything as I am told at the clinic and by the doctor. I should accept.

I: You came here at the clinic with what symptoms?

P:I had TB and been taking ARV when I came to the clinic and even now am still taking them.

I: So, which signs did you have to show you are sick?

P: I was weak, but I felt no pain, just a little irritation on the left.

I: So, mama, when you say you were spitting the pills, they never saw them at home?

P: They would have not noticed if they had not pulled the bed because it is only when they pulled the bed and saw the pills and realize that this person has been throwing pills under the bed, but I was already better and drinking medication.

I: At that time, you already had the box, or it was before?

P: I had the box.

I: You had the box the time you were spitting the pills?

P: Yes, I had the box. They started by giving me the box first, so I can take medication correctly, I felt ashamed.

I: You felt like you are what?

P: I felt embarrassed.

I: Ok, because of taking medication?

P: Because I am sick, I wondered if the pills would heal me. I just did not have faith in that I will be healed.

I: You said it is a child who set you down and explained about medication?

P: It is the child of my late sister. The child was the one who was always talking to me and giving me pills.

I: Would you say the talks you had are the ones that made you to-

P: -To take medication but they did not know here at the clinic that I was spitting pills. She saw them later that this person spit pills.

I: How did you feel after the talks you had with the child

P: I realised that she is speaking the truth and said to myself let me take medication and see where I will end up.

I: So, since you are now staying alone and still using the box, what can you say about the box?

P: I feel very well now; this box helped me a lot.

I: When you say it has helped you a lot, can you explain how did the box help you?

P: I can explain by saying I was not walking; I was always lying in the bed, day and night but now I have faith that even at the auction I could score higher [laugh]

I: How does the box help you in your life?

P: It helps me because it rings at the time I am supposed to take pills. I simply know that it is time for medication.

I: Has the box ever rang and you did not open?

P: Mmm, I do not like being far from it; I look at the phone and I know that it must ring at 5 o’clock. I wait for it to ring before I open and take medication, mmm.

I: So, when you travel, do you travel with it?

P: It is always with me wherever I am; it stays in the handbag because sometimes you find that I went to visit the kids and they ask me to sleep over and I must take my medication, yes.

I: So, people do not see it when you travel with it?

P: It is in the handbag, and it does not embarrass me. I can also hold it with my hands.

I: I mean do not people ever see it? Like when it rings or when you are taking medication.

P: They do see it.

I: Do they ever ask, what is this thing?

P: Uh, uh there is no one who ever asked, even here at the clinic when I need pills, I hold it with my hands

I: Beside your family and your children, is there anyone else who ever saw this box?

P: Some other sister.

I: She did not ask any question about it?

P: Uh she did not ask anything; I was taking medication and believe she saw that this person is taking pills from the box.

I: Mmm.

P: Yes.

I: So, as you have TB, is there any person you ever told beside your children?

P: Uh I say that I have TB and I always put this thing on, so that I do not infect other people.

I: So, there are many things on this box, what do you see as the most helpful thing?

P: Everything; pills including everything they give me at the clinic even though I do not know which pills are for TB and which ones are the ARV. I have not asked which ones are for TB, I just take them all.

I: Which medication did you put inside the box?

P: These ones, the ones I get here at the clinic.

I: All the pills you get from the clinic?

P: Yes.

I: So, you take all your pills at the same time?

P: Yes, all of them, they gave me two packets and the bottle for this thing. When it is time, I take out one and I take out another one. If I must take out two, I take out and take out another two and then I drink with water.

I: Ok… is there any SMS that you ever received because of medication?

P: Am not a person who reads SMSs, I delete- I just read one and then delete them all.

I: You have never seen any that speaks of medication?

P: I have never seen them; I do not want to lie and say I have read them because maybe they were there, and I deleted them.

I: So, have they ever called you here at the clinic?

P: Yes, they called me last… yes, they called- they called me, but they did not find me; my phone was outside. They called the child to call me.

I: It was because of?

P: There were people who wanted to see me.

I: They never called because of not drinking medication?

P: Uh-huh.

I: They never called you to tell you to take medication?

P: Uh-huh they never called. I was taking my medication correctly because they gave dates for when I must come back and there is a card, we need to tick on it when we drink. I tick when I drink; I tick, yes.

I: So, you have never received an SMS or call to remind you to take your medication?

P: Uh-huh, I never received those. We registered with the child’s phone number when we registered and maybe they used to call the child.

I: When you registered, you used the child’s phone number?

P: Yes, we used the child’s phone number when we registered for the first time because she is the one who brought me here and she is the one who was helping me with medication at 05H00.

I: Have you ever missed a day without medication?

P: I missed at that time where I did not want to take pills. I personally did not want to take medication; I wanted to die.

I: You missed when you were spitting them, or you did not take them at all?

P: I was spitting them and throwing them away.

I: Oh, you meant that when you said you missed treatment.

P: Yes.

I: Oh because you were embarrassed?

P: Yes, I felt ashamed.

I: So, it was the child who explained the box to you?

P: Because the child is the one who knew I do not take pills and sometimes she would say “take pills” and I would say I do not want to.

I: Then what happens?

P: There is nothing that can happen because it means they must get inside my mouth and there was no way she would open my mouth and say drink.

I: So, is there a history of TB in your family?

P: My father had it.

I: When they diagnosed you with TB, were there not other family members scared since one had it before?

P: Uh-huh, they were not scared. They did not know what it was, they even encouraged me by saying TB can be cured, you can stay three to six months and then you will be healed.

I: I want us to talk about the box

P: Mmm

I: How much can you say the box helps

P: Sorry?

I: How much does the box help; how much does it help people?

P: [laugh] I cannot understand your Setswana; it is hard.

I: It helps… would you say the box helps?

P: Too much.

I: It helps a lot; how does this box helps?

P: It helps by keeping the pills safe and it reminds you.

I: Beside keeping the pills safe and reminding you, is there anything else the box helps with?

P: These pills that gives me life which are inside the box.

I: So, for now, what are the challenges that comes with using the box?... any problems that you encountered while using the box?

P: Explain it well.

I: What can you say prevents you or makes it hard for you to use the box?

P: There is nothing.

I: So, beside the child who brought you here at the clinic, how did the family feel about the box? What did they say?

P: Uh they felt just well, I was sitting in the bedroom the whole time, and I would tell them to put their masks on before they enter the room but they were not disgusted. They came to visit me.

I: I mean with the box; how did they feel about it?

P: Uh they did not have any problem with it.

I: So, when someone is not taking their medication, they get an SMS after a day to remind them to take their medication. Would you say that is a good way of reminding people to take their medication?

P: It is exceptionally good.

I: Now mama, for those who does not like messages, will not they feel like they are being disturbed?

P: There, I do not know because as for me, I was taking my medication day by day.

I: Then if you miss for more than 2 days-they send you SMS for missing one day but with two or three days. They must call you here at the clinic to remind you to take your medication, what can you say about calling people?

P: Uh I do not have a problem because they will be helping me to remember taking pills that gives me life if I am not taking medication.

I: When we cannot find someone on the phone, they send someone on the fourth or fifth day to come and check up on at home, what do you think about this?

P: It will be ok because they will help by speaking to them to take their medication.

I: Do you have any worries about using the box?

P: I do not have any worries with it; I have accepted.

I: So, when it rings, has it ever rung in public?

P: Mostly when it rings, it rings from home. I make sure I am home.

I: Oh, it never rung while you are around people?

P: Uh-huh.

I: So, have you ever opened your box more than once in a day? Maybe two or three time in a single day?

P: Sometimes I open twice when I am checking the pills, I check if they are enough for the days, they gave me.

I: You open to check the pills?

P: Yes, I check to see if the pills are enough.

I: What can you say helps the most on the box?

P: It is the pill inside and the alarm that rings.

I: It is the ringing alarm?

P: Mmm alarm.

I: What can you say about the volume of the alarm?

P: The volume is alright because even this young child comes to call me if it rings, and I am sitting in the dining room; he comes and say, “it is time for your pills” and he is only 7-6 years old.

I: He knows the sound of the box?

P: He heard it when it rung, and I was going there too.

I: Would you say you have support from your family?

P: I have a lot of support; it is a lot.

I: Beside going to your children’s place, have you even gone somewhere with the box and slept over?

P: Uh-huh.

I: Never went anywhere?

P: Uh-huh it is only where I was when I was sick; at the children’s place, let us say.

I: Ok, would you say it is easy to use the box?

P: Uh.

I: Would you say it is easy for people to use the box?

P: A lot easy, it is easy for this box to help you when you have accepted it in your life.

I: On everything that this box does, the alarms, keeping medication together and the lights, what can you say helps a lot about it?

P: What helps the most about it? it rings and the pills inside it.

I: You mention the pills inside the box often, did they explain how they work?

P: Yes, they explained. They only explained that these pills will help, drink them. When the sister was explaining.

I: That is how they explained?

P: Yes.

I: So, did you ever had a network problem where you are staying? Where maybe the box went a day without ringing?

P: Uh-huh we do not struggle with network.

I: From the side of our culture, is there anything that prevents you from taking your medication?

P: No.

I: Ok, from the spiritual side?

P: No, there is nothing.

I: Have you ever reached a point where you did not have a phone and they could not reach you here at the clinic?

P: Uh [cough] I think I spent 2 months; my phone was lost.

I: Was it the time you were extremely sick or before?

P: It was the time I was sick; I gave it to the child when they took me to some hospital called xxx (hospital name). Now when I was there, I gave it to the child and then the phone got lost.

I: How long have you been sick?

P: Uh?

I: How long have you been sick?

P: I think I started in the 11^th^ month, then I did not tell anyone that I am not feeling well. I only told the child in November that I am not feeling well, I am losing weight. I do not understand how I am. That is when they sent a car to come fetch me to take me to them, then I was too sick.

I: Why did you choose not to tell anyone when you started getting sick?

P: Uh I was buying *Boston,* I was taking *pain killers.*

I: Did they take you to the clinic when you got to the child’s house?

P: I got there Saturday at the child’s place; on Monday they said let us go to the clinic and then we came here at the clinic

I: When you came here, they tested you?

P: Yes, here at the clinic, they explained to me that I am sick and then tested me on my fingers. They were testing HIV, they said they cannot find it and ended up drawing blood, and that is when they got it on the blood.

I: On the box, what do you think could be modified to help people more?

P: I do not see anything that they can change because it does everything that is right.

I: If you could be asked to explain the box to someone who was newly diagnosed with TB, how would you explain it to them?

P: I would explain that the box is safe, it keeps medication, and it is safe by giving you time to take you pills.

I: So, where to you keep your box at home?

P: At home, I put it on the headboard; not inside anything, I just put it there.

I: So, how does the box affect your everyday life at home?

P: They have accepted it, there is no one who is saying this box is making noise.

I: Would you say they are positive about the box?

P: I would say they are ok, there is no one who is complaining.

I: I mean in your life since everyone has their life at home. Ever since you started having the box, is there anyhow you would say It has affected your life or daily routines?

P: Uh-huh, it did not change anything because it was the child who had it most of the time and we did not even put it inside the plastic; we just hold it like that.

I: How satisfied would you say you are with the box?

P: Am very satisfied, you know.

I: You are satisfied?

P: Yes, not that I am satisfied because I am cured but I am satisfied that they gave me this box.

I: So, on the size, what do you think about the size?

P: It is fine. It can put all the pills inside; the pills are not equal with their packaging, but they can all fit inside.

I: What about the volume?

P: Uh it is simply fine.

I: Do not want to change the colour?

P: It is white in colour.

I: Yes, it is white, would you like to change it?

P: White is fine.

I: What about the language, if ever they contact you, what language would you prefer?

P: Setswana that I can understand well.

I: When they are writing you an SMS, should it also be in Setswana?

P: Mmm.

I: Ok, you would prefer the same language.

P: Yes, I understand it well.

I: How easy is it for people from the clinic to come where you are staying?

P: Uh there is no problem.

I: I mean is it possible for them to come where you are staying?

P: At xxxx (area name)? If they want to come, there is no problem.

I: Which clinic is the closest to where you stay?

P: It is xxxx (clinic name).

I: You came here because you were at the child’s place?

P: Yes, I was at the child’s place and I love this one; it is the one that knows my sickness and where it started until where I am now

I: So, the counselling you get in the TB room, how much would you say it helps?

P: Where?

I: At the TB room where you get your medication, the talk they give you, how helpful would you say it is?

P: It helped me a lot; it helped me.

I: If you could explain it better when you say it helped you a lot?

P: I would say the help they give us; the sister was talking to me a lot; she would lecture me saying I should take my medication and I will improve. I will be like any other person who is walking, and I was not walking; I was using a wheelchair.

I: That counselling that you got, who do you think should do it to TB patients? Who is supposed to be used as counsellor to teach people about TB?

P: I see sister as the one who must teach because we get pills from her.

I: On these things I mentioned, getting SMSs, phone call and home visits, which one would you say helps the most?

P: Other people except me?

I: No, everyone including you, what can you say helps a lot?

P: Now the problem is that I do not know how other people will receive them.

I: Ok, but which one do you think would help people the most?

P: I see visiting people as the one that can help them a lot because maybe there are those who do not take medication correctly

I: You think they would say that are not taking medication correctly when they visit them?

P: There is no one who would say that but they would have seen from their file that they are not taking pills.

I: How did the box help you in coming to the clinic?... when you come to the clinic, did they ever show you your adherence on the tablet?

P: There, I did not see anything because I was sick and I do not know if they showed the child.

I: I think we have spoken enough, is there anything that you want to talk about regarding the box?

P: What I can speak of about this box is that people should accept it and drink medication inside it accordingly at the time they were given.

I: To get people to accept this box as you did, what do you think we should do in teaching them about the box?

P: You can lecture them; do not lecture only in the days you are giving the box, they should be called in sometimes to be shown the box.

I: When you say lecture, what do you mean?

P: To teach them

I: On teaching, how do we teach them?

P: Teach them that this box saves pills and keep time for you.

I: When you say we teach them, you mean like how we do when we giving them medication?

P: Like uh with medication inside, we don’t talk about it, you talk about the box, so that it does not become heavy on people. You must show them that this box we are giving you it has an alarm and this alarm will ring at the time you take your medication; It reminds you to take pills and you must take your pills.

I: You mentioned that we call people to come so we can teach them about the box, do you think people on TB treatment would agree easy to come to the clinic to be taught about the box?

P: I don’t know there because I don’t know their thinking but I see it as something that would make it easy if they are taught on how the box works as some might take it and as soon as they get home. Take out pills inside and put the pills there and the box there.

I: If mama, they do a lecture day and invite people, would you attend?

P: For someone who was sick from TB, I can teach people and I can tell them that I was sick, and I was given the box. I would also explain how did it work for me till this moment.

I: If they can ask you to explain how did they gave you the box, and how did it work, would you be able to answer just those two questions only?

P: They gave me a box, repeat the question.

I:You were sick when they first gave you the box, how did it help you? Let us say you are explaining on the lecture day, what can you explain about how the box helped you?

P: I can explain that the box helped me with time; the main thing is the time which reminds me to take pills and it helped with keeping pills safe, at one place.

I: Ok mama, I heard you, is there anything else you would like to speak about?

P: I don’t think so, what I can say is I thank the sister, the one who gives us- she is very patient and kind to people who are sick, and she is able to talk to you if you are sick and tell you that you will be cured and bring brought back to life.

I: Would you say support is important in taking treatment?

P: Support is very important.

I: Would you say the box supported you?

P: It supported me, I am repeating this a lot; it gives me time.

I: Ok, is there anything else you would like to speak about?

P: What I can say is that the child who was taking care of me and family more especially the one who works night shifts at the mines. She would come in the morning and wait with me here until I receive my medication and sometimes we would leave here around 13H00 pm, and she cooks for me when we get home. Then dish for me and I would take a nap and then the box rings. She would then wake up to give me medication. She has not slept and she works night shift.

I: We have reached the end of our interview; we thank you for your time. If you have any questions later to ask, on the paper I gave you earlier there are numbers there, call them and they will answer all your questions, thank you mama. Ending time 12:03.
