# Supplementary material for: Acceptability of Digital Adherence Technologies to support people with drug-susceptible TB in South Africa
Source: PLoS One. 2025 Sep 24;20(9):e0332103. doi: 10.1371/journal.pone.0332103 (PMC12459780; doi:10.1371/journal.pone.0332103)
Supplement: S4 File — (ZIP) [file pone.0332103.s004.zip › S4 Transcripts/PwTB/IDI 14_PwTB.docx]

I: Do you give us permission to record you?

P: Yes, yes, I do.

I: Location xxx (clinic name) , Language used Setswana, PID number xxx, Start time 08:20, Uh how far is the distance to come to the clinic and how much does it cost you?

P: Uh over kilometres or distance?

I: Yes, how far is it?

P: Around twenty something, you mean in kilometres, right?

I: Yes.

P: I never calculated.

I: How many taxis do you use?

P: Mmm bus, the leaves at, but sometimes I use local taxi if the bus is not showing up.

I: How much does a return trip cost coming to the clinic?

P: Uh it is around, coming and returning? Using a bus, it is around R15 if am using a local taxi, then it is around R25 when I double it, it makes R50.

I: R50, okay, when were you diagnosed with TB?

P: 22 November last year.

I: 22 November last year?

P: Yes.

I: How did you feel on the day they told you, you have TB?

P: I could not hear?

I: How did you feel when they told you, you have TB?

P: Ehh I didn’t have problem because I was already sick, I have accepted because I now know the cause of my sickness because I wasn’t aware of what it was. I thought it was just flu or something, but flu, why am I becoming like this? I was powerless or something like that ….

I: What symptoms did you have?

P: Symptoms, I was coughing the most and then other things I wasn’t okay. I couldn’t eat well, food uh medically it was those of coughing, but I thought it was flu at first , but they said no this is not fl. When I come here they tested me and took my sputum, and then the results came back and that is when they told me I have TB. I then started treatment.

I: Mmm, so who do you stay with at home?

P: Come again?

I: Who do you stay with at home?

P: I used to stay with my mom, my siblings, and grandchildren.

I: You do not stay with them anymore?

P: I stay with them.

I: Oh, you still staying with them?

P: Yes, since the time I found out that I have it. They asked them to be tested as well; they should submit their sputum and luckily, I brought them. They were negative, then I proceeded with treatment, uh.

I: So, about the box, did they give you this box immediately after you’ve been diagnosed?

P: When I started treatment for the first time; when I started treatment for first the time that is when they gave me this box.

I: Who explained the box to you?

P: It was xxx (intern’s name) who was just here.

I: How was the explanation?

P: Explaining how this work-

I – Mmm.

P: It alerts you at the time you are supposed to take your pills and when your medication is finished, it turns yellow or orange and then yes on average I see it been good this thing because it reminds you to take medication at the time. So, that is the main point of it that it the most important thing about the box.

I: What did you say is important about this box?

P: Uh that it helps you to remind you to take medication on time, umm it is alright even when you don’t forget time for your pills, but when they are finishing it alert you, that you must go back to the healthy facility to collect your medication.

I: I want this part that it reminds, how does it remind you?

P: Uh when it makes this siren thing.

I: It makes a siren?

P: Yes.

I: Okay and then how does it remind you when the medication is finished?

P: Uh it has lights; it shows orange. It has orange and red; it shows constant to show you it is time to collect your medication at the clinic or facility where you attend and if it has a problem, you have to report here to them again so that they can check it. At times when you opened, at first when I started, I don’t know if it was me or what and I returned it. It didn’t respond the way it was supposed to.

I: What do you mean?

P: I mean let’s say uh it has to beep at 6 o’clock in the morning, then sometimes it doesn’t make that beeping.

I: It didn’t beep?

P: Yes, but it was only one occasion or so then that was the time I experienced it. It was the time I just started medication. Only to find out after few days that it was not linked then they corrected with the system. I don’t know how but, from there it was properly functioning, working just fine.

I: So, on the day it didn’t beep did you take your medication?

P: I did take medication because I was already used to; I was used to taking medication at 6. It will ring and I also checked time so I can set an alarm, let’s say I left the box in the house and went to the toilet. A phone can remind me with an alarm before, but the box must ring first so I can take medication.

I: You have an alarm in your phone to go closer to the box?

P: Uh-huh I mean am already used to it.

I: So, from the explanation you got from the TB room, is there something you experienced you feel they should have taught you about it before?

P: Uh with the box?

I Yes.

P: Rather than the explanation I gave you?

I: No, according to education they gave you about the box at TB room. Do you feel like there is something that they should add telling people on how the box works? Something that you feel they didn’t tell you about it?

P: Uh I felt like those were the basics about the box, as for additional information uh education was put in between. I think mostly are okay, on average I understand how it works and it importance. Those are the things I was looking at mostly. It importance and what is in there.

I: Mmm so before they showed you this box in the TB room, have you seen this box somewhere before?

P: Uh-huh no, they showed it to me when I started treatment; they took it out before I went in then they explained it to me.

I: I mean before then, you have never seen it?

P: Uh-huh before that, I have never seen it. I saw it first time at that point, it was the first time when I saw it that there is such a thing and previously I had experience with people who had TB before. I had it but they were not using this system. I saw that here they are making it easier for people so that they can take medication on time, because it reminds them and when you use your own mind sometimes you can forget. So you adjust, and you get used to it that this how I should do this.

I: Okay, So what is it on the box that makes it easy to be used? Or that makes it hard to be used?

P: Uh the thing that makes it easier uh, uh … [pause].

I: Okay, let me not stress you.

P: Yes, you can see that am thinking hard about this question.

I: No, it is fine. Are you working?

P: Uh I was working before I got sick and then I stopped when I got sick. I was helping people with piece job.

I: Okay, so have you ever travelled with the box? Or left with it from home.

P: Uh to leave with it, I left with it when we had a funeral at home. I went with it to the funeral. I also I went to visit at my uncle’s and because it is distance from home, I won’t leave it back. I stayed for 2-3 days there. I took it so I can use it from that side.

I: So, how would you define the difference of using the box in that event and when you were home?

P: Uh, it was somehow different but because it is almost same principles, just that here it is at the funeral. In the room I was staying in, people in there were amazed that there is such. They even asked me, what is this thing for.

I: At your uncles, did they ask about it?

P: Uh yes, am with the family right. They asked me and then I said this box; it is pill box for medication and it is for such stuff. Then they asked me how does it work and I explained to them that it is the one that reminds me to take medication and because we were at the funeral, and it is busy we are working. They ended up being the ones to alert me that don’t forget your thing [laugh]. Don’t forget your medication or don’t go too far. Remember at the funeral we slaughter a cow, and other things.

I: How was their perception, how was their reception about the box when they saw it?

P: Uh on average it was positive.

I: It was positive?

P: Yes, it was positive, as you know other people have stigma to say. They will be asking each other why this and if this where i put cigarette. I was open about my situation; I was always telling them how I was. Most people I live with there were very supportive and encouraging me to finish treatment saying “ don’t stop taking your medication, it only 6 months.” There were encouraging me and they were positive people around me, and those I lived with.

I: So, have you ever disclosed to other people except family?

P: Mmm yes.

I: How did you feel when doing so?

P: Uh I think uh it was- because not everyone was positive about it, it only 80% of them. It felt good to disclose something like this. You become open like- I didn’t stress when I talk about it, and they were asking why are you like this and why is this like this. Some would even make assumptions that you have other diseases but, then I told them I have TB and am even taking treatment when they were asking me if I got help. They were asking, did you take any actions and that is when I told them that am even on treatment.

I: Okay, do you have any history of TB in the family?

P: Mmm, mmm, I was the first one.

I: So, have you ever opened the box maybe when it is not the time for medication? like you open but not take medication.

P: Uh I open when I want medication.

I: Only?

P: Yes, and the other time when I open it is when I put medication inside it.

I: Other than medication purpose, have you ever opened the box?

P: Uh … there was other time I was coming from here at the clinic, so there were these other pills they were inside; the ones I drink later, and I forgot to take them out. I realized that I forgot them inside, then I opened the box to take them out.

I: Okay, no problem, so where do you put it at home?

P: Please come again?

I: Where do you put it?

P: It stays in my room because I keep it away from children. If you place it just openly, kids will reach it, but my sisters can enter in my room. In there I just place it there, then I told them that these are uncle’s medication.

I: So, has there been anyone who opened the box beside you?

P: Other than me? No.

I: Okay inside, other than medication what do you keep inside?

P: like, them only.

I: Medication only?

P: Medication only, even at home they know, what are these medication for.

I: So, have you ever got an SMSs, phone call or home visit from the clinic regarding TB medication?

P: Yes, you mean from the clinic? Yes, it used-you know what was happening at the time? I thought it was a malfunctioning but yes, it was the SMS that said, “do not forget to take your treatment and such.”

I: You got the message reminding you not to forget to take your treatment?

P: Like at the time when I opened and it did not beep, then I opened again later. After 5 minutes, that is when it rang, then I thought maybe it is malfunctioning or something. Normally when it does that, is when I get the SMS.

I: So, did you ever receive a phone call?

P: Phone call concerning the box?

I: Yes, that you missed medication?

P: Uh-huh normally are just messages.

I: Did they ever come to do a home visit?

P: Normally I am the one coming here when I have an issue with the box; I tell bra xxx (intern’s name), he is the one who will sort it out.

I: So, when you receive the SMS, how do you feel about those SMSs?

P: Uh they are just fine; I don’t see any problem with them. They are doing same procedure that is being done by the box. Sometimes you get the SMS before the box rings; before taking treatment. So, it reminds me of these pills that I take outside the box. They give me those pills they work together with TB medication. So, when I get these messages, they remind me of these pills to take later on the day. I take them twice or three times a day and so forth. It was very useful, I think so.

I: Have you ever gotten worried about getting SMS?

P: Uh-huh.

I: Would you recommend that they should leave it like that?

P:Yes, I think they should leave it like that because It is technology and most people are using phones. So, sometimes you get that you went to town or somewhere and when you get the message because we always leave with our phones. So, I think it necessary that they should continue to be there, so that they can remind people that you should take medication, or that you should get closer. Let’s us say, you have left them back home and you know you must take your medication. I think they should just leave it as it is; the message must always be there because people are using their phones and we move around a lot.

I: So, if you can miss more than 2 days, they have to call you from the TB room to remind you about medication. What do you think about phone calls?

P: It is important, you mean to call... [someone walked in] [pause]…

I: I mean send you an SMS.

P: Yes,

I: Then still you continue to miss, from tomorrow they have to call. What do you think about being called to be reminded about medication?

P: Uh I think it is necessary , you see, when you talk about 2- 3 days, you see. Now you are missing treatment; someone must do something. I think it is relevant; it think it is a must.

I: Then when you miss 4- 5 days.

P: Yes.

I: Now they must come to visit you at home.

P: Yes, they must come, but also the phone call. They must still call.

I: Yes, they call you after 2 days, but if you still continue they have to come to your home.

P: They must come where you are staying, yes.

I: What would you say about doing home visits?

P: It is correct, they have to visit where we staying to see what is the matter, why are you not taking you medication correctly. Sometimes the phone call won’t be enough, you can say this, but you have missed medication, you see. You need support on top of the phone call. Yes, the home visits are needed also when you come personally. If you know a person you can inspect easy to see what is the matter, sometimes you find it is neglect; find that you have neglected yourself…[ someone walks ] [pause]

I: Alright, we can continue, we want to know what can you say is the best about the box ... uh the alarms, phone calls or the box itself. What is the best thing about the box?

P: On those things that you have mentioned?

I: Not necessarily those I have mentioned, anything that you know about the box.

P: Mmm.

I: It doesn’t have to be just one, as long as it is related to the box.

P: Those that I love, or those which I think they worked for me on the box? You see the alarm; the alarm system and the time for me to take medication. Those are the best thing about it; that is what I love the most about the box.

I: Now tell me about challenges on the box, what challenges did you come across when using the box?

P: Mmm… Challenge uh … I would say… it is reaching it, but because it is with you . I don’t see that as much of a challenge actually.

I: Would you say you did not have any challenges

P: Challenges are thing that gives you problems or it gives you tough times adapting to it? Generally, I didn't encounter anything, it made things- it was just fine.

I: Okay so, these are sort of challenges but not so much, we want to know about the barriers, where it was hard, did you ever had network issues?

P: Uh network regarding this box? There was a time where it was malfunctioning or maybe the system or something almost gave me tough time. It is just that, that almost gave me tough time, but I had already adapted. I was used to that I take medication at this time and that is where the phone was useful. I used to make a backup *mara* (the thing is) the issue was for very a short time and from there it was properly functioning .

I: Mmm okay, so culturally, traditionally has there been any barrier you uncounted regarding the use of the box?

P: Uh culturally uh, you know how our traditions are. In general when I started to disclose to my family and everybody that I live with, they were like uh what do they call it…supportive and stuff. Some were saying this western things of yours, uh but in general 80% of people were supportive, only few were concerned with their traditional things, but I stayed with the majority of people who supported me.

I: On the minority, those who were speaking about traditional things, what is that there were actually against?

P: They believe in African religion and staff, so the boxes are western things. So, they were not supportive , but very few though. I would say this thing sometimes it is created by not having information sometimes. I tried to explain to them what is this, and I could see that it is working for me. Hence I was positive that this is working for me.

I: So, I know you said you never received a phone call, you just got SMSs only. What would you say out of these 3-4 things including counselling; the talks you get in the TB room. What would you say out of these 4- what would you say it is the most effective? What do you think helps people the most? So that they can take their medication.

P: Mmm I would say the SMS and the box actually, SMSs also help. You see these two things, the box and SMS work hand in hand.

I: So, on the box, what do you feel like could be changed?

P: They change it or improve it?

I: Yeah, yeah, change it or improve it.

P: Uh I think…

I: What do you think about the size?

P: You see on the size ,the pills for whole 6 months won’t all fit inside. I think so, but at the same time it is important that we come to the clinic frequently. So that you can get counselling and whatever or come here frequently or come to the doctor and they tell you that you are adapting well with the symptoms like in a case when you taking pills, then there is time where your legs get swollen. It is necessary that I come so that when I have side effects. On the basis of improving uh the size, they can increase the size a little bit, but I think coming here at the clinic frequently is also important. Not coming here after 6 months not healed, should come after a month or two weeks.

I: And colour?

P: Oh you mean for the lights?

I: No colour for the box as it is?

P: Oh colour,

I: Volume *ya* (of)alarm?

P: Volume for the alarm, I think it is just fine, I can hear even when am at the passage. As for colour right, you can want colours now we have this cream white one, they can add colour to make it more positive. When someone has it you just see something. They should make different colours; there should be an option to other than white- cream white only. It is good ,but for attracting people more.

I: Ok, if they can ask you to teach someone who is newly diagnosed about the box, what can you tell them?

P: The Importance; the necessity of it.

I: What is it importance?

P: It keeps medication intact, and safe in the box. It is not like when you just left it there scattered or just left there and there. They are placed in one place, then the other thing is to remind them the time to take medication, yes. Those are the most important things.

I: Those are the things you can teach a newly diagnosed person?

P: Who just been diagnosed let us say.

I: You have to tell them about the box.

P: Also, the light; how they are working and also what they indicate. What they must do when it beeps, all those basic things and all the things I would tell them about.

I: Ok, we are getting to our final session, so is there-Is there anything that you feel like we did not talk about, regarding the box that you feel like I didn't ask you about? Or is there anything you would like to tell.

P: Mmm that you didn’t ask me about, regarding the box? Mmm, mmm I think we almost covered- you covered most of them in those question you asked me.

I: Okay, we come to end of our session, Thank you very much, stop time 08:56 am. Thank you.
