# Supplementary material for: Acceptability of Digital Adherence Technologies to support people with drug-susceptible TB in South Africa
Source: PLoS One. 2025 Sep 24;20(9):e0332103. doi: 10.1371/journal.pone.0332103 (PMC12459780; doi:10.1371/journal.pone.0332103)
Supplement: S4 File — (ZIP) [file pone.0332103.s004.zip › S4 Transcripts/PwTB/IDI 15_PwTB.docx]

**TRANSCRIPTION NOTATIONS**

| **Label Key** | **Meaning** |
| --- | --- |
| **I** | Start of each new utterance by the Interviewer |
| **P** | Start of each new utterance by the Participant |
| **N** | Note taker |
| **{ }** | Indicates that details were changed or pseudonyms were used to anonymise data |
| **( )** | Indicates the description provided to anonymise data |
| **XXX** | Words were omitted to anonymise data |
| **-** | Breaking into a sentence by the next speaker |
| **…** | Pause or drawn out words |
| **[ ]** | Indicates noise made, e.g. [laugh], [sigh], [pause] |
| ? | Beginning of utterance by unidentified speaker or questionable text |
| **[inaudible segment]** | Unclear section of the recording |

I: Eh thank you very much for being here with us. Eh I’m asking a permission to record our conversation

P: Ok, its fine you can record

I: Alright date xxxx (interview date) , location XXX (clinic name), language used Setswana, PID number xxx, time 13h20

So how far is the distance between your home and the clinic?

P: It’s a bit far, I’m paying R15.00 transport fare to get home from the clinic

I: May you please speak a bit louder

P: It’s a bit far, I’m paying R15.00 transport fare to get home from the clinic

I: Who do you stay with at home?

P: I’m staying with my mom, my siblings and my children

I: So when you were diagnosed with TB how did they react after they heard you have TB?

P: They understood because they know that TB catches anyone

I: If I may ask, how did you feel when you were told that you have TB?

P: I was hurt but I understood after some period

I: Mhhm

P: Eh

I: So how did you, how did you know you have TB what were the symptoms you were having?

P: I was only vomiting and lost appetite

I: Then you decided to come to the clinic?

P: Then I came to the clinic, that’s where I was told I have TB

I: Mhhm

P: Eh

I: Tell me about the box, who informed you about the box?

P: Mhhm yoh

I: Was it a nurse or research assistant?

P: It was a sister who was assisting a nurse. She’s the person who explained to me about the box

I: How long did it take for her to explain to you about the box?

P: I may say about 30 minutes

I: Mhhm

P: Explaining to me showing how it works telling me not to misuse the box

I: Mhhm

P: Telling me to handle it with care so that it cannot be damaged

I: Mhhm

So how were you feeling when she explained to you about the box?

P: I already made peace with the situation I’m in

I: Mhhm

P: I want to be cured so I can’t refuse any help

I: So do you think it’s easy to use this box

Do you find this box user friendly to use it?

P: The way this box is easy sometimes when I forgot to drink my medication it rings an alarm then I know it’s time to take my meds.

I: Mhhm

P: It’s nice to have this box because when you do not have this box you can easily forget the time to drink your medicine.

I: Mhhm, do you have any challenges using this box?

P: Nope

I: Mhhm

P: Eh

I: Do you have a job?

P: No I’m not working

I: So what time do you take your medicine?

P: 09H00 in the morning

Morning

P: Mhhm

I: So 09h00 has ever been passed while you’re not at home?

P: No I’m always at home I don’t visit other people.

I: Your afraid to go to other people, what’s the reason

P: They don’t want us near them

I: Why?

P: Because they’re afraid I’ll spread them with TB

I: Mhhm they’re afraid you’ll spread them with TB

P: Eh

I: So you don’t go anywhere?

P: I only go out when is my appointment at the clinic and when I’m going to the shops

I: Mhhm

P: Eh

I: Okay, so ever since you had this box, have you ever visited your family or take the box with you somewhere?

P: I don’t go anywhere

I: You don’t go anywhere?

P: I don’t even visit family members

I: Mhhm

P: Eh

I: Is there any effect this box has ever brought to your life?

P: No, it’s nice having this box

I: Mhhm

P: There’s no problem

I: Is there anyone who has ever asked you about this box?

P: As I leave next to the road, some do ask me about this box

I: Mhhm

P: Eh near the tavern, we call it machine sometimes I send my children go fetch my machine when its ringing

I: Ohh you call it machine

P: Yes , my children bring the machine when its ringing while I’m resting outside so some people ask why am I taking pills out of the box

I: Mhhm

P: They ask and I explain to them that its a new system

I: Mhhm

P: The same way they explained to me that we are using this box now

I: Mhhm

P: Eh

I: What is the response when you explain to them

P: They’re surprised since is a new system

I: Mhhm

P: It’s first time we use this system

I: Mhhm

P: Mhhm that’s why they’re surprised

I: How do you feel when you explain to them about this box, as it shows you’re taking medication?

P: As I already made peace that I’m sick

I: Mhhm

P: And they explained that TB is curable

I: Mhhm

P: As long as I’m taking care of myself adhering to my treatment within a matter of time I’ll be cured.

I: Mhhm

P: I feel pleased to explain to them

I: Mhhm, so is there anyone who supports you to take your medicine

P: Yes

I: Who?

P: I have support at home, most my mother and my brother

I: Mhhm

P: Yes also the father of my children they’re the people who are taking care of me

I: Mhhm

P: Eh

I: So how are they responding towards the box?

P: They accompanied me when I started the treatment

I: Mhhm

P: They already knew I’ll need their support as I’m sick

I: Mhhm

P: Yes so they know they have to support me throughout this sickness and not take it otherwise

I: Mhhm

P: Eh they must understand and make peace same as I did

I: Mhhm

P: Eh

I: So besides family members is there anyone who supports you?

P: My neighbour

I: Your neighbour?

P: Yes

[Door opens and closes]

?: Greetings

P: Greetings to you too

I: So is there anyone who has ever heard the alarm besides seeing the box?

[Door opens and closes]

I: Like hearing the alarm then ask what is ringing?

P: My children

I: Your children?

P: Yes they’re the ones that were surprised where the alarm is ringing from

I: Mhhm

P: They wanted to understand

I: Mhhm

P: Eh

I: So how are you feeling telling people about this box?

P: Because I made peace that I’m sick who ever asks me I explain the same way they explained to me at the clinic.

I: Mhhm

P: Then they understand that we are no longer getting medication on the hand, they put it in the box

I: Mhhm

P: They put it inside the box

I: Mhhm

P: Eh

I: Sister have you ever opened the box besides when the alarm ringing?

P: No I don’t open the box without any reason

I: Mhhm

P: Even my children I explained to them that this box should be opened only when is ringing

I: Mhhm

P: When I started taking treatment it used to ring over and over

I: It was ringing more than once a day?

P: Yes, it used to ring twice or triple times a day

I: Mhhm

P: Then I explained to the sister who gave me the box that it rings more than once

I: Mhhm

P: Then she told me to open it for about 30 minutes

I: Mhhm

P: Then she said maybe my children has damaged it, then I explained to her that the box is not damaged by anyone

I: Mhhm

P: Then she asked me if the battery is still okay? Then I explained to her that the battery is fine the box still beeps and flick the light the same way you explained to me

I: Mhhm

P: Then she said maybe the box has factory fault

I: The time it started to malfunction, how were you feeling about it?

P: I was afraid I thought maybe we damaged the box

I: Eh

P: But it started to function okay after a while

I: Mhhm

P: It stopped to malfunction and functioned nicely after a while

I: Mhhm

P: Mhhm

I: So where do you put this box?

P: On top of the cupboard

I: Mhhm

P: Eh

I: Since you said your children have access to the box, aren’t you afraid they can drink your medication?

P: No my first child and my second child are the only ones that have access to my box

I: Mhhm

P: I have six children, so I send only two of my eldest children to fetch the box for me.

I: Mhhm okay

P: Eh

I: So is this box helping you in anyway?

P: Children like to play with medication when is laying around, so this box helps to keep medication in one place where its not accessible to everyone

I: Mhhm

P: Yes it even helps to keep medication safe

I: Mhhm

P: Than to put medication everywhere

?: [Greetings]

P: When medication is in the box they’re safe than laying around where children can access and eat them

I: Mhhm

P: Eh

I: So what about the reminder alarm?

P: Alarm?

I: Mhhm what about the reminder alarm?

P: I can say alarm will ring continually until the box is attended

I: Mhhm

P: Eh

I: So how does it help you this alarm?

P: This alarm helps me a lot sometimes I’m alone sleeping on cold days

I: Mhhm

P: When children have gone to school and others have gone to work, I’m being reminded by this alarm that is time to take medication

I: Mhhm

P: I wake-up eat and drink my medication

I: Okay, so have you ever received an sms regarding medication?

P: SMS?

I: Eh

P: Ah yah sister sent me an sms saying I haven’t taken my medication yet I had opened my box to take medication

I: Mhhm

P: And because I have sister’s contact I call her and let her know I already took my medicine the system didn’t alert you

I: Mhhm

P: Eh

I: So you received sms explaining that you didn’t take medication while you already taken your medicine?

P: Yes while I had already taken it

I: How many times did it happen to you?

P: Maybe three to four times

I: Mhhm

P: Eh

I: How were you feeling when you received that message?

P: Yoh my mom was angry at me

I: Mhhm

P: My mother gets angry because sometimes she doesn’t see when I take my medicine and the phone number that is registered to the box is not mine

I: Mhhm

P: It’s not my phone

I: Mhhm

P: So when they receive message they show my mother telling her that the system says I’m not taking my medication

I: Mhhm

P: Eh

I: Okay, so sister have you ever received a call from clinic regarding the missed doses

P: No I only receive message’s

I: You haven’t received a call to tell you that you didn’t take your medicine?

P: I received sms’s only

I: Okay, so have you received any home visit?

P: No

I: You haven’t ?

P: Yes

I: So how will you feel if you receive a call saying you didn’t take your medication?

P: I will feel bad

I: On our work neh

P: Mhhm

I: If you miss medication you receive an sms and if you truly miss your medication what will you do? Once you miss medication you receive sms

P: Ahh

I: How do you see it?

P: Unless they say the medication that I’m taking is depleted at the clinic

I: Mhhm

P: That I can understand

I: Mhhm

P: But if I have medication at home but I’m not taking it, no!

I: Mhhm

P: I’ll never do that

I: After you miss your medication for two days we call, how do you feel about that is it right or wrong?

P: Ehh I can say is right and is also wrong because everyone is responsible for taking his/her own medication, if you don’t take your medication it simply means you want to kill yourself.

I: Mhhm

P: Eh

I: Which way is right?

P: It means when they give you medication you must drink it accordingly.

I: Mhhm

P: Know the time to take your medicine by heart

I: Mhhm

P: Eh

I: Besides alarm is there anything that reminds you to take your medication?

P: Yes they remind me

I: At home

P: Yes at home, sometimes they remind me around past eight while relaxing at home.

I: Mhhm

P: They remind me

I: What time do you drink your medication?

P: I drink at 09:00

I: At 09:00?

P: Yes

I: So they remind you at past eight?

P: Yes

I: So when they remind you at past eight, you drink your medication same time?

P: No I tell them that I’m waiting for the alarm to ring

I: Mhhm

P: When alarm rings I take my meds, and if the alarm doesn’t ring I don’t take my medicine I take my box to the clinic and let the sister know my box doesn’t ring, that’s what she told me to.

I: Mhhm

P: Eh

[Door opens, closes]

I: So how will you feel when you have a home visit to explain to you about medication? Is it right that sister from clinic visits you at home?

P: Sister already told me that there’s community health workers that visit patients at their homes.

I: Mhhm

P: I will be pleased when they check up on me at home so that I can also let them know the challenges I’m facing.

I: Mhhm so of three other things sms, phone call and home visit which one is best for patients struggling to drink their medication?

P: Message

I: Message?

P: Sms record a message and phone call record a missed call

I: Mhhm

P: Message will always be there

I: Mhhm

P: Eh message explain details

I: Mhhm so have you ever had a network challenges whereby your box skipa day without ringing an alarm?

P: No

I: It never missed a day without ringing?

P: Yes

I: So did you have any phone challenge whereby they tried calling you but calls never reached you?

P: No they always reach me on the phone.

I: Mhhm

P: Eh

I: What pleases you about this box?

P: I’m pleased about this box

I: Mhhm

P: Eh

I: Mhhm can you explain how this box helped you out to a point whereby you never missed your daily doses?

P: I can say this box is so much important

I: Mhhm

P: It’s so important to every TB patient than getting medication on a plastic bag

I: Mhhm

P: Eh

I: When you say it’s important, what makes it important?

P: It always reminds you to take your medication at the same time every day

I: Mhhm

P: At that time it rings an alarm

Mhhm

P: It reminds you its time for medication

I: Mhhm

P: Eh you must stand up and drink your medication

I: Mhhm so how did this box help you not to miss your medication?

P: Eh this box helped me out a lot because if it wasn’t for this box I would have missed some days without drinking my medication.

I: Mhhm

P: I would have taken my medication at different times

I: Mhhm

P: For instance I’m supposed to take medication at 09:00 but take it a 10:00

I: Mhhm

P: This box rings alarm at the same time every day

I: How does this box keep your medication safe?

P: My medication is safe in the box

I: Mhhm

P: Even children won’t be able to play with medication while it’s in the box

I: Mhhm

P: My children only ask what is this? Then I explain to them

I: Mhhm

P: They know only the oldest can bring me the box.

I: What do you like the most on this box? Alarm sound or lights

P: Time

I: Time?

P: Yes

I: What do you wish that can be changed on this box or to be upgraded to function more than it does?

P: Its perfect the way it is

I: Mhhm

P: Eh it’s always on time and its battery is strong. The battery can last you until you complete treatment.

I: Mhhm

P: It covers the period of your treatment.

I: What about the volume of an alarm?

P: The volume is right, is not low or too high.

I: Mhhm

P: Even when you’re sitting inside or outside the house you can easily hear the alarm

I: Mhhm

P: Eh

I: Okay so what about the size?

P: The size is normal it’s can also fit in the hand bag

I: Mhhm

P: Its not too big.

I: So which language do you prefer to communicate with?

P: Setswana

I: Setswana even on an sms?

P: Yes

I: Okay so in order to help TB patients neh

P: Mhhm

I: What do you think they should do more, like send sms everyday, call everyday or home visits how do you feel they should do to help patients on TB treatment?

P: I think sms’s are better.

I: Mhhm

P: Because calls can’t reach everyone always but sms reaches everyone

I: Mhhm

P: Eh

I: Who explained to you about this box?

P: It’s a sister who’s working with a nurse

I: Okay

P: I forgot her name

I: Name is not important, so what do you think they should do so that patients cannot refuse this box?

P: They should explain to patients before they give them boxes

I: Mhhm

P: Explain to them how it works and its advantages that will help them to complete their treatment without struggling and they should also use it wisely

I: Mhhm

P: And they also put it on the safe place

I: Mhhm

P: Because it can be broken even its battery can be damaged

I: Mhhm

P: It needs to be taken care of

I: Is there something else you think we can support TB patients with apart from the box?

P: This box is so important more than everything

I: Mhhm okay, it there any other thing you want to talk about regarding the box?

P: What I can say is that we TB patients must do everything that was told, we must not do what we are not supposed to do this and this because you are killing your self

I: This and this?

P: Like I was given medication, I’m supposed to drink it

I: Mhhm

P: If I’m given medication with medication bags without a box P: when I get home I must make sure I put it in a safe place

I: Mhhm

P: I’m not supposed to put them where children can reach them

I: Mhhm

P: I must put them far because only box is safer than papers.

I: Mhhm

P: Eh

I: Okay let’s say they just told someone that he/she had TB

P: Mhhm

I: And they say you’re supposed to explain to him/her about this box, can you explain?

P: Yes the way I was told

I: Mhhm let’s say the way you understand this box

P: Mhhm

I: As you used this box can you explain how it works?

P: First thing I will explain is that it would help you on children as they like to play with everything

I: Mhhm

P: Eh it will put your medication safe

I: Mhhm

P: So that children can’t reach them

I: Mhhm

P: And it will ring when it’s time for you to take your medication

I: Mhhm

P: When it rings you will know it’s time you were given

I: Mhhm

P: My box is ringing I must drink my medication

I: Mhhm

P: It’s my time

I: Mhhm

P: You stand up and drink your medication after you put back your box where it belongs at the safe place

I: Mhhm

P: You make sure children won’t reach it so that they won’t be able to drink your medication because it is easy to open

I: Mhhm

P: And every kid can open it even the little one

I: Mhhm

P: So when you put it on the safe place you also protect your children not catch the same disease you have by drinking your medication

I: Mhhm okay

I: Okay I think we have reached the end of our interview, is there any other thing you want to talk about? Regarding sms, phone call and home visit?

P: No I have said everything

I: Alright

I: Thank you very much for your time and for the information you provided

I: Ending time 13:50
