# Supplementary material for: Acceptability of Digital Adherence Technologies to support people with drug-susceptible TB in South Africa
Source: PLoS One. 2025 Sep 24;20(9):e0332103. doi: 10.1371/journal.pone.0332103 (PMC12459780; doi:10.1371/journal.pone.0332103)
Supplement: S4 File — (ZIP) [file pone.0332103.s004.zip › S4 Transcripts/PwTB/IDI 16_PwTB.docx]

**TRANSCRIPTION NOTATIONS**

| **Label Key** | **Meaning** |
| --- | --- |
| **I** | Start of each new utterance by the Interviewer |
| **P** | Start of each new utterance by the Participant |
| **N** | Note taker |
| **{ }** | Indicates that details were changed or pseudonyms were used to anonymise data |
| **( )** | Indicates the description provided to anonymise data |
| **XXX** | Words were omitted to anonymise data |
| **-** | Breaking into a sentence by the next speaker |
| **…** | Pause or drawn out words |
| **[ ]** | Indicates noise made, e.g. [laugh], [sigh], [pause] |
| ? | Beginning of utterance by unidentified speaker or questionable text |
| **[inaudible segment]** | Unclear section of the recording |

I: Do you agree to be audio recorded?

P: Yes.

I: Ok thank you. Date xxxx (date), location xxxx (clinic name), PID xxx, time it’s 11:25am, language used it’s Setswana. So, sister could you please in short tell me who you are?

P: Ok, my name is XXX (participant’s name), I’m 42 years old, I’m not married, I have two boys. I live with them at my place, and I stay with my mother and I’m unemployed and I have TB.

I: Ok I’m pleased to hear that. So, tell me do you stay far from the clinic? How far is the distance?

P: I don’t know the kilos, I’m not sure about the kilos bit it’s not too far.

I: So, do you walk, or you take a taxi?

P: I use a car.

I: Oh ok, I’m pleased to hear that. So, could you please tell me about [box beeping sound] this label that what do you know about this label?

P: The stickers [label]?

I: Yes, the stickers or the label.

P: Mmm this sticker I was given eeh at the clinic when I was starting TB and they told me that everytime I drink eeh treatment I have to send an sms and which is free and I have been doing that. I think that it shows that I’m drinking treatment every day.

I: Ok so, you mentioned that when you are done taking treatment you send an sms?

P: Mmm.

I: So, how do you send that sms and where do you send it to?

P: [Laughing] There’s s number that I send 42352, I don’t know where the sms it’s going but I know that the sms- I think that it’s reaching people who deal with TB patients. That can trace that are you drinking your medication, or you are not drinking but then that’s what I do every day.

I: So, the time they were explaining the label to you, could tell me who was explaining this label to you?

P: I don’t remember but then I also- now it’s XXX (Research Assistant’s name). XXX is the one who told me about the treatment- eeh the stickers. (Research Assistant’s name). XXX works here, at the clinic and on our TB section.

I: Ok, I’m pleased to hear that. So, [clearing throat] the time she told you about this sticker or this label, do you feel that there is something that she did not tell you about these stickers?

P: No, I don’t think so. I don’t know that there is something that she did not tell me but what I know is that she gave me the sticker and that it monitors my progress and I tell myself that too because they go hand in hand these things. I drink and then I sms every day [door opening/closing sound].

I: So, the day they explained this label to you, how were you feeling when they explained this box to you?

P: No, I didn’t mind because I knew that this has to do with my progress you see and then they were helping me in a way. So, I did not mind.

I: So, after you- finally you decide that now you are testing for TB, physically like how were you feeling that made you decide to test?

P: Mmm I wasn’t alright physically because I was coughing so much and then I had shortness of breath like for short distance I had shortness of breath and then I was coughing so much. So, I had to come because my mother also had TB. So, I think because I’m staying with her, I got it from here, so it was only wise that I also come and test, unfortunately when I tested that’s when I got the results that, I am positive. I have TB.

I: So, I’m sorry to hear that your mother also had TB but on the first day they told you that your results are back, you have TB. Like how did you feel, when they told your results?

P: I cried. I cried too much, and I was miserable like I thought like aah come on I survived Covid why would I have TB? I’m not even smoking, I don’t have a boyfriend that I would say maybe I kissed him and got TB, so like I was miserable, but I got to accept because I got the nicest nurse. So, like they were counselling me because I was crying, they gave me counselling and then I got to accept my situation and then even when I was starting treatment, I knew that I wanted to get well for me, for my kids, for my family so it was not a big thing but when I got the results it was very- it was not a nice feeling honestly.

I: Ok so, you mention that you were not- the time you were not feeling well, the TB symptoms you mentioned coughing and breath that you would sometimes have shortness of breath-?

P: Shortness of breath.

I: So, other than these two symptoms, are there any other TB symptoms that you know?

P: Yes, the night sweats. I remember it’s shortness of breath, coughing and then the nights sweats those were my problems.

I: Ok so, the time I was doing the consent form process I mentioned the way I would need like your experience towards this label, feeling and attitude towards the label. So, with the experience you have using this label, could you tell me some of the challenges you encountered using this label?

P: No, well SMS you [?] because we are all human. I’ll be honest, SMS at times you forget even though I don’t send it the same time every day, but I do sent it every day. I am saying that we are all human eeh we forget that we have to send things at the same time every day, but I don’t have a challenge not much challenge with the SMS.

I: So, you mentioned only one challenge that you forget, that you can take medication, but you forget to send an SMS?

P: No, I don’t forget the SMS, I forget the time. The time like I’m not sending at the same time like not the same time every day.

I: So, could you tell me that not the same time every day cause I don’t understand.

P: Ok, like I drink my medication at 10 and then mostly I send SMS before I drink medication and then at time you would find that I drank my medication before I send. I send it after you see, but then it will not be maybe I say I drink medication in the morning and then I send it in the afternoon, no aah there’s a time that I drink medication and- you see it differs maybe about 30 minutes only but it is not the same time, but it is every day.

I: Ok so, what you just- like how often does that happen in a month or a day, how aften does that happen?

P: Ai it might be 3 times, it’s not much.

I: So, could you please tell me, are you working?

P: No, I’m currently unemployed.

I: Ok, I’m sorry to hear that but a temporary job?

P: Mmm.

I: Piece job.

P: No, I’m not working. I need a job.

I: Let’s hope you will find it [laughing]. So, since you started using the box, I mean the sticker or the label. Have you ever left with the sticker, or you visit with the sticker?

P: Mmm I was a bit of a challenge. Eeh it’s a good question because in February I went to xxx (neighbouring county) , but I went for two weeks but I did- I came here and then I asked that maybe eeh they give me my medication because it was going to be finished when I am not here. So, then the sticker it was not going to work because of eeh the number was not going to work outside you see. So, I did not use it the sticker for those two weeks I wasn’t here but then medication I was not very consistent.

I: So that I understand, you took medication for two weeks, but you did not take the sticker?

P: The SMS wasn’t going to work that side because of network. Our network is not working that side.

I: Ooh ok so, to make sure that you were taking your medication in that two weeks. What is it that you were using in that two weeks period to make sure that you were taking your medication?

P: What is it that made me drink my medication? Like I’m telling you, I’m scared of dying that is motivation enough. I was very consistent with my medication.

I: [Clearing throat] so, when taking medication, you send an SMS so that the Sr could check your adherence using the platform or the tablet they are using in the TB room. So, what I would like to understand, or my question is that in those two weeks period who was taking care of your adherence?

P: No, I can- you see I’m 42 years old, I won’t say I would not say that another person should come and check if I am taking my medication hence I myself- because before I left, I saw that I would run out of my medication, I came here myself when I saw that my medication would run out and I am leaving. I’ll be out of the country so I myself came here and I asked for medication because I knew that I was travelling you see. So, I do it on my own, I have to guard myself because it’s for my own health. I don’t want to go there, so I cannot- I could not risk it.

I: So, when you say “you don’t want to go back there” to the state you once were before you started TB medication up until today. Could you tell me some of the changes that like today how are you feeling physically compared to the way you were before you started TB medication?

P: Well eeh I cannot say I’m, I’m 100% before I was diagnosed but I’m much much better in compared to the time when I did not know what’s wrong with me because the shortness of breath really I thought I was dying because I couldn’t talk like I would be blocked like I’ve never experienced such pain or should I say pain but I was not in pains, it’s like what I was feeling at that time I cannot even describe it. I thought I was- I would feel like battery running out of power [laughing] it says battery low and it’s beeping. But then compared to then and now there’s mmm I’m still not okay, I think that lungs are damaged there are times I would have shortness of breath, but I can handle them. I realized that I won’t be 100% again, I would get better as time goes by.

I: Ok so, so when you say “there are times you have shortness of breath”-.

P: Mmm.

I: Do you have them while on treatment-?

P: Yes.

I: So ever since you started treatment and then you experience the shortness of breath, have you ever consulted to Sr regarding them?

P: Yes, any problem. When I have a problem, I really do talk to eeh the nurse, I ask questions like even today I asked that high blood where do you test it cause I realized that during my sickness my heart was under pressure a bit. So, the last time I went to XXX they said “your heart is under pressure” so there are high blood pressure symptoms. So, I realized that when TB comes affects a lot of things not only the lungs.

I: I’m sorry to hear that. So, moving on to my next question. On the first they gave you these stickers or they explained them to you, what were your worries towards the stickers?

P: I did not have any worry honestly. Honestly, I did not, I accepted this like it’s part of treatment you see.

I: So, earlier on you mentioned that you stay with your two boys and your mother?

P: Mmm.

I: So, who knows about your state with the lung disease?

P: Yoo too many people, I was not hiding it.

I: And then could you tell me about their reaction to- when they knew about your status like how was their reaction when they knew about-?

P: About TB?

I: Yes.

P: The elder one because he’s 21, he was concerned that “mom where did you get it?” like the usual questions like “mom where did you find TB, you don’t even smoke?” you see. So little one doesn’t understand, he’s still too young, I did not have to explain to him that much cause he understood. So, people who are close to me like family I tell them that I, I have TB and I don’t know where I got it but they were very encouraging, everybody like “take your treatment and you’ll be alright”.

I: So, earlier on you mention that there are a lot of people who know about your status [door opening sound] sorry about that. So back to my question, other than the people you stay with, are there people from outside who know about your TB status?

P: Yes, yes. It’s family, my aunts, my cousins you see. They know about my status; I never hid it from them.

I: So, what encouraged you to disclose your TB status to-?

P: To them? Why did I feel free? I was just free, so that they don’t ask things- I felt like it’s not taboo, it’s not something that I did. So, it was very easy for me to tell them that I have this thing and I will not come to you and please don’t come to me. I don’t want you to be sick like you see because when you are sick people will be concerned and they will come to what do you call it? To support you and to show you that they are with you but then I will stop them, that guys please don’t come I have TB, I don’t want you to be sick and be like me. So, that’s how I disclosed mmm.

I: So, they know that you are diagnosed with TB disease?

P: Mmm.

I: So, the matter of stickers, do they know that you are using the stickers to monitor your TB adherence?

P: No, they don’t know. They don’t know, the only people who know about the stickers are the ones I stay with.

I: So, besides the ones you are staying with, is there someone or people who have seen you sending an SMS after you took your medication?

P: No, there’s no one because I’m drinking my medication at home since I’m not working mmm.

I: So, you mentioned that your mom had TB?

P: Mmm.

I: So, besides your mother and then now you, is there anyone who has ever been diagnosed with a lung disease in the family [door opening sound]?

P: [?] oh we are fine. [participant was responding to the unidentified individual].

I: So, just like I mentioned that your mother had TB disease, how was she feeling when you told here that mom-?

P: I also have TB? It was-, she was hurt because she felt responsible that I got it from here and she was telling me that “my child you don’t go around too much, you got it from me. Sorry I made you sick” because I was sick too much. She told me- she was very apologetic every day you see. She never felt good that she made me sick.

I: Ok so, the time they told you that you have TB, was it the same- did she complete her TB medication?

P: No, no [clearing throat]. It’s different because my mother smokes and they found it through X-RAY because her lungs are damaged [yawning]. Her lungs are damaged now, how did I get TB?

I: So, ok to my next question, is there a day whereby you sent the SMS more than once in a day?

P: No, no.

I: Ok aah earlier on- earlier on you mentioned that when you send an SMS it goes somewhere, and they could see that you took your medication?
P: Mmm.

I: So, there is something we call adherence calendar it is on the tablet, so have you ever seen your adherence calendar?

P: No, and I have never thought that I should see it.

I: Ok so you took- you sent an SMS; do you know what the Sister (nurse) uses to see that you sent an SMS, or you did not?

P: I never thought of that [talking while laughing], I don’t know.

I: So, there is what we call a adherence calendar that is on the tablet that shows us that you sent an SMS or you did not send the SMS [clearing throat] that’s what shows us.

P: So, what did you see on me, is there- did you check my adherence calendar?

I: Yes, I did before we started the interview but if you want to know there’s a Sr and an RA. They are the ones who can show you everything.

P: Mmm.

I: Yes, only after the- when we are done with our conversation or this interview-.

P: What does it say? Does it say I’m complying?

I: My question is that are you taking it?

P: Yes.

I: Are you taking the medication?

P: Huh everyday but when I say I’m afraid of dying and you are still asking me those things [laughing].

I: So, if you are taking your medication and you are sending the SMS, even the calendar will show that you are sending.

P: [Laughing].

I: So, you mentioned the way- we have the stickers [door opening sound]. So, you mentioned a support from your mother and even that she was blaming herself that you got the sickness from her-?

P: Mmm.

I: Even the stickers and the kids. Do you have any other support or any that you get other than the stickers or at home?

P: Yes, I do get it outside the house [door opening sound]. The friends, the relatives, I do get support honestly. It has been nice, and I don’t [door opening sound] like old people feel like when you TB, it is like you have some disgraceful disease as if like TB it’s HIV no. I have been the most support, I don’t want to lie. I have been- it is what made me strong [clearing throat], the support from my friend’s and my family, I don’t have any problem.

I: There is something we call- we call it differentiated care model, so differentiated care model it means that it is SMS, phone call and a home visit. So, have you ever received an SMS that was reminding you to take your medication?

P: Mmm I do get SMS but I think the time difference is the one that is not alright because I receive the SMS at night. I would prefer that I get the SMS in the morning, reminding you that don’t forget to take your medication. Aah unlike the way I receive it, I get it at night. It’s not really helping cause I get it at night and then I’m about to sleep at that time and I drank my medication a long time ago because I drink my medication at 10 in the morning.

I: Ok so, according to your answer, you would like to receive the SMS in the morning?

P: Mmm yes, yes.

I: So, when you say in the morning in terms of time-.

P: 6, 7 it will be fine, even if it can be 5 it’s fine, we woke up at that time.

I: So, that I understand, so you would like SMS- when you say morning around 5. Will it be before or after you drank-?

P: It will be before because it’ll get in at 5 in the morning and I drink medication four hours after, like 10 o’clock in the morning. So, it will be very useful if you do that.

I: So, phone call like I mentioned it’s SMS, phone call and a home visit. Have you ever received [people talking in the background] a phone call, that was reminding you that don’t forget- phone call asking if whether you drank your medication?

P: I have never received that kind of SMS or that kind of a phone call that say you haven’t drank your medication. I only receive an SMS that say “you have not drink your medication” of which it is the one that disturbs me a bit.

I: So, a home visit?

P: Ai no.

I: So, going back a bit back to- the time they told you that you have TB disease [door opening sound] so, make sure that the people you stay with inside the house they don’t have this disease.
P: Mmm.

I: So, what is it that as a clinic they did to make sure that you don’t infect the people you stay with?

P: They tested all of us, all of us tested. All of use we sent our sputum’s, my kids and they found that all of them are alright.

I: So, since you have an experience le the labels or the sticker. So, is there any barrier that could have stopped you from sending an SMS after you drink your medication?

P: No, there’s nothing that could have stopped me.

I: So, with your experience, could you tell me like how satisfied are with this label?

P: I am alright, I don’t have a problem with the SMS and the sticker, I don’t have any problems. I don’t have any problems with them.

I: But like just to check your overall satisfaction, like how satisfied are you ta have the stickers that when you are done taking your medication, you send an SMS that will show Sr that you took your medication, they check your adherence.

P: Aah it’s alright because it tracks. It tracks your compliance of how you are using your stickers and then I think it is fine I don’t have- I have nothing much to say. I don’t have any problem with them because I am complying, I am drinking the pills, I don’t have a problem.

I: So, when using this label, with your experience what is it that we can add on the label to make sure that this label is easy for you to use?

P: No, the label I don’t think there’s much you can do because it’s easy. You just send, you press only, it’s not even complicated. It depends on a person that what is it that they want to do but I think it is much easy, there’s no problem here [clearing throat].

I: To my next question I will need your perception, like how do you see the stickers. So, we were talking about the differentiated model care, which is SMS, phone call and a home visit. So, to go back to the issue of SMS, the time you received the messages late reminding you that you should your medication. Like how did you feel receiving the SMS late at night when you are asleep or you are about to sleep, knowing that you took you medication?

P: No, at first it was frustrating me a bit but then as time goes by I learnt to ignore it. I no longer have a problem with it.

I: Just so I understand your feelings when you say it was frustrating you.

P: Mmm [laughing] it was frustrating me. It was boring me.

I: And then later you managed to ignore it?

P: Mmm.

I: So, like I need you to explain to me your journey, like how did you go from being frustrated to ignoring it?

P: Is because I got the SMS that said I didn’t drink medication, I know that drank that’s why it was so easy for me to ignore it you see because I knew that the SMS meant that my SMS the one I sent did not go to the right place but I send it though and it responds that “thank you for taking your medication” but later I get the message that says I did not take medication, so that’s why it was so easy for me to ignore it.

I: So, you mentioned that you have received an SMS that was reminding you to take your medica- that you should take your medication whereas you took your medication. Have you ever spoke to the Sister (nurse) or an Research Assistant (RA) from xxx (organisation name) in the TB room, that you received an SMS that was saying you should take your medication-?

P: Mmm.

I: Whereas you drank your medication? Just so I understand-.

P: Yes, I did, at the beginning like I mentioned that it once frustrated me a bit but I had to tell them that how does your things works? I’m sending SMS and I am drinking medication, but the following day receive a message that says I did not drink. So, I was told to ignore it as long as I keep on sending and then I am keeping on drinking my medication.

I: So, to my next question, I will need your thoughts like what is it that you think about the differentiated care model. So, your feeling for a person who is not taking medication, do you think the stickers approach would work for someone who is not taking their medication?

P: A person who is not drinking medication?

I: Yes.

P: What is he/she going to do with the sticker when she/he drinking the medication?

I: Ok let me repeat that so that you understand. We have a patient who does not adhere to medication, so in order for us to monitor his/her adherence. So, the thing [door opening sound] could help him/her that he/she can adhere- so that we can monitor his/her adherence?

P: Yes, you know first of all XXX you must understand that anything a person does, they do it willingly and then even the medication they will drink it when they want. So, it is up to you that you want to send the SMS or you don’t want to send it like even medication it depends on you. So, I feel like these things they go hand in hand and whoever is monitoring my progress will see that this person is taking her medication. It’s up to a person really.

I: So, when you say, “it’s up to a person” what is it that as a clinic or as xxx (organisation name) we can do to make sure that can take their medication or can adhere to their medication?

P: First thing, a person has to make peace with their medication herself and her disease that she wants to drink her medication and she wants to get cured but then I also think the home visits could also play a role. They (health care workers) could also see how their patient is living where she stays, peoples backgrounds are not the same, so I’m thinking that the home visits they’ll be- they’ll play a role.

I: So, to stick to the issue of home visits, your thoughts. Who are the people who are qualified enough to conduct this home visits?

P: I don’t know [laughing][door opening sound] the people who work here. Those who are trained for that kind- on that department, they are the ones who should go and check the patients.

I: Ok so, when you talk about the home visits- I will talk about it after but for now to go back you mentioned that when they told you, your results on the first day [disturbed and recording was stopped]. So, you mentioned the time they gave you your results the first time it was hard to accept the results and then you got to a point whereby you cried because of your results and then it was after counselling where I say you were able to accept your situation? So, with your experience with the counselling, what can you say counselling helps you with?

P: When you have problems, when you are sharing with the second person or the third person, you hear their opinions, and you find out that you are thinking this way and then the other person bring their perception and then it makes you see that what you were thinking is not as hard as you thought it was you see. So, that’s what counselling is for it makes you- sometimes you see that the problem is huge but when you talk to a person you can you that this one I can tackle it and then I become alright, doesn’t have to be complicated.

I: So, during those counselling sessions, when you say, “you might think that the problem is huge”.

P: Mmm.

I: Whereas this thing-.

P: It’s doable-.

I: It’s doable or it’s manageable situation.

P: Mmm.

I: So, during those counselling sessions, with your experience what is it that they are supposed to talk about during those counselling sessions?

P: What can I say? They should tell you that it’s not the end of it, it’s not the end of the world. Eeh TB can be beaten and then you just have to take your medication and then that’s it XXX, and then I did that [door opening sound] I pushed where I am.

I: So, the approach of SMS, a home visit and a phone call.

P: Mmm.

I: So, out of all these three activities, which one according to you works too much to make sure that a patient is taking TB medication?

P: I think the home visit, they can come and check your medication but then I really do think that it’s up to a person you know. It depends on the person on how they want to drink their medication.

I: So, here I have this box, do you know this box?

P: Yes.

I: So, what it, can explain- what is it that you can tell me about this box?

P: I know that most of the time the box reminds you that you must drink your medication.

I: Ok and then it reminds you that it is time to drink your medication. So, I know that you don’t have much experience with the box compared to the sticker.

P: Mmm.

I: But when you see this box or when some explain the box to you, what do they say? What reminds them on this box, what is it-?

P: It has a machine, it has- it a has an alarm.

I: So, what can you tell me, what do you think about this box?

P: It [box] is alright for older people because they forget. My mother has it also.

I: Ooh.

P: Mmm.

I: So, just so I understand you when you say “it is alright for older people” it is alright in which way?

P: Because-.

I: They forget in which way?

P: It is loud. You find out that time you forgot to drink medication, it reminds you that you must drink me. It’s it loud, it is an alarm.

I: Ok so, you identified an age gap. You said older people?

P: Mmm.

I: Ok, according to you, you recommend that the box is used by people from which age, starting from which age [door opening sound]?

P: Pensioners.

I: When you say “pensioners”, they start from which-?

P: About 60.

I: And then why do you say so?

P: Because those people forget.

I: So, and then- can you recommend [door opening sound] so, the sticker when you are looking at them or from your experience, can you recommend people from which age?

P: For our age group but those whoa are still active, they are the ones who should sms because they are always on the phone. It won’t be a problem because they are used to pressing you see, unlike the pensioners [clearing throat].

I: Just so I have a clear understanding or easily understand you. So, would you agree or disagree that older people when it comes to the technology they have an issue, that they don’t understand it better than us?

P: They don’t have, they know it very well. They even know WhatsApp and certain things; they don’t have any problem.

I: So, if they don’t have a problem, why do you recommend this box to older people not the stickers?

P: Now I don’t know.

I: Ok no problem, there’s no problem but thank you-. So, according to you, your opinions regarding the stickers even if you could help me with your opinions with the box as well. So, how can we improve the sticker to be more user friendly?

P: This thing doesn’t bother, already it is user friendly. There’s nothing you could improve on here; you just send an SMS and a number then you are done. There’s nothing difficult going on here, I don’t think you know. I don’t know maybe other people can tell you, but I don’t think so.

I: So, how do you think we can improve this box?

P: I don’t use it, so I don’t know.

I: Ok I’m pleased to hear that. So, now I’m wrapping up this conversation as a whole, so I’m going to go back on the questions that I did not understand. So, since you are using this sticker and then you told me like your experience that at home your mother is using the box. So, looking at this sticker and based on your experience with the sticker, do you feel as if there’s a gap or something that is missing, that no the sticker don’t cover this like, the sticker should be doing one, two, three it would have been better?

P: I don’t have a problem with the sticker and how the sticker is used.

I: I know you are using the sticker and then you recommended the box to older people but according to you with your experience and your thoughts, between the two technologies which one do you think it is better?

P: Listen, I’m not saying we are not going to use this box, I am just saying it is good for older people that they are able to use it and for us we can use this because we are always on the phone all the time [clearing throat].

I: So, when you say- just so I wrap up the interview. So, your final thoughts about the labels, since you have experience with the labels. So, what’s the last thing you can say about this sticker?

P: It’s a good thing to remind you to drink your medication, I no longer have words, I don’t have anything to say. I don’t anything to say serious.

I: Ok I’m pleased to hear that. The sticker comes from ASCENT study, It’s ASCENT study. So, if they were here, what is it that you would tell them that the sticker helps you with- how did it help you in terms of taking you medication up until today?

P: This sticker doesn’t help me with anything, it’s just I’m sending the message that I’m drinking medication, those who gave me TB treatment see that I am drinking. Mmm but I am more motivated to drink my medication it is not- because I have to remember that sms. It is not like the box that it rings that you take medication, but sticker is not going to make noise that I did not drink your medication you see [clearing throat].

I: Ok so, with your experience with the stickers, do you think the sticker should have been implemented a way back to make sure that people are taking their medication?

P: Come again.

I: With your experience, do you think the sticker should have been implemented a way back, years back?

P: I don’t know, as things goes on things change.

I: So, your thoughts, when you are looking at this sticker, do you see it helping people in the years to come?

P: I don’t think so, I think- when time goes by things will improve mmm.

I: Ok so, I have come to the end of our interview, so but before I close the interview, what are your overall thoughts about these stickers?

P: Sticker is a good thing to remind yourself to take medication that’s all.

I: Ok, I pleased to hear your feedback 4495, but sadly I have come to the end of our today’s interview.

P: Ooh we done?

I: And then I thank you for the time you took to come and conduct this interview with us.

P: Mmm.

I: So, I thank you.

P: I’m the one who’s grateful.

I: Ok, ended time 12:25pm.
