# Supplementary material for: Acceptability of Digital Adherence Technologies to support people with drug-susceptible TB in South Africa
Source: PLoS One. 2025 Sep 24;20(9):e0332103. doi: 10.1371/journal.pone.0332103 (PMC12459780; doi:10.1371/journal.pone.0332103)
Supplement: S4 File — (ZIP) [file pone.0332103.s004.zip › S4 Transcripts/PwTB/IDI 17_PwTB.docx]

**TRANSCRIPTION NOTATIONS**

| **Label Key** | **Meaning** |
| --- | --- |
| **I** | Start of each new utterance by the Interviewer |
| **P** | Start of each new utterance by the Participant |
| **N** | Note taker |
| **{ }** | Indicates that details were changed or pseudonyms were used to anonymise data |
| **( )** | Indicates the description provided to anonymise data |
| **XXX** | Words were omitted to anonymise data |
| **-** | Breaking into a sentence by the next speaker |
| **…** | Pause or drawn out words |
| **[ ]** | Indicates noise made, e.g. [laugh], [sigh], [pause] |
| ? | Beginning of utterance by unidentified speaker or questionable text |
| **[inaudible segment]** | Unclear section of the recording |

I: I am requesting permission to record you.

P: Yes, I agree

I: Date xxxx (interview date)

P: Yes

I: Location XXXX (clinic name)

P: Yes

I: Language Setswana PID xxx start time . . . 08: 43

I: So, how far do you stay from the clinic?

P: Uhm what can I say. uhh 5 km away

I: Okay... how many taxis do you take to get here?

P: Just one.

I: How much is the fare?

P: R12

I: When did you get diagnosed with TB?

P: On the …. 10 January.

I: Which side effect did you have?

P: My nails changed color, my urine changed color, I had this thing that looked like mosquito bites, but they were not painful, they would just

I: When you ended up coming to the clinic which symptoms did you have?

P: Eh! I was coughing, even now am still coughing, I have some thing here, it attacked with E N T.

I: it attacked with?

P: ENT ear , nose, and throat, Yoh! I bought medication, alchophylics, alchophylucs yho still they are not helping, am still coughing, I have an irritation on my throat and the thing here it come to this side, the eye and nose on this side they have water and if it goes to this side the eye and the nose will have water.

I: Have you reported that at the clinic?

P: Mhhm

I: They gave you medication?

P: Yes, the sister is helping me with allergex, and I always have a cleaning cloth you see, if I can cough now yoh!

I: Since they gave you allergex, are you feeling better?

P: Yes, I was little better, u know I can drink them all the time.

I: So, mama, tell me about the box, what do you know about the box?

P: This box, they said it will monitor but am not seeing any monitoring, it rings at wrong time, send me wrong message at the wrong time, send me message at 6 pm where I take my dose at 7 am at 6 pm it reports that I didn’t take my dose and I already took my dose.

I: They said it will monitor, they said how will monitor?

P: I knew it will tell me to take my dose, but it’s not doing that it is telling me I didn’t take my dose and I have taken my dose.

I: They said it will tell you to take your dose?

P: Mhhm

I: How did they say it will tell?

P: Like, I have set an alarm on my phone it will tell, 7 o’clock it rings I take my dose.

I: So, with the box, they said it will tell you how?

P: I thought, it will tell me like phone does, but it doesn’t do so, or I didn’t hear correctly, I thought at 7 o’clock it will ring.

I: You said you receive the SMSs late?

P: Here they are, you saw them.

I: Mhhm

P: Ka 6 pm

I: What does it say?

P: I take my dose at 7 am.

I: SMS, what does it say?

P: It says I didn’t take my dose; they will send a sister to talk to me.

I: So, alarm, you said it rings at wrong time?

P: Yes, it rings at wrong time.

I: What time did you set?

P: 7 o’clock

I: What time would it ring?

P: At 6, It was supposed to ring at 7 am it rings at 6 pm

I: Alarm rings at 6 pm?

P: Mhhm

I: Okay.

P: And it tells me that I must not forget to take my medication before 12 midnight, iyhoo and that time it will be long since I have slept. Even the ARV’s I take them at 8 o’clock and I have friends who tell me, because -Generations [soapie] plays at 8 o’clock, you will hear one saying, are we not watching -Generations today? Or my child saying mama are we now watching Generations, I know what they are speaking of [laugh] mhhm.

I: Have you ever reported the box/ that it rings at wrong time?

P: Mhhm, I returned it, I showed them the messages, him xxx (intern name) said, if you don’t want it no more you can return it.

I: How long did you use it for?

P: 2 days

I: You used it for only 2 days?

P: It has to many *wara wara* don’t put it next to tv, don’t put it next to radio, I sleep with radio.

I: Did they explain to you, why you must not put it next to all those things?

P: They explained.

I: They said why must you not put it next to the tv?

P: He told me, it has what what, electric [laugh] but I understood him, he explained well.

I: Who explained to you?

P: It was xxx (intern name).

I: Is there anything you feel like they did not explain to you when they gave you the box, that you feel they should have explained?

P: Uhh uhh he told me xxx (intern name) … just that it did not work how I expected it to work, I wanted it when it is 7, it rings, then its rings when the time has already passed, it will again tell me I didn’t take my dose, I wanted it to say get up go drink medication.

I: When xxx (intern name) explain the box to you how did you feel?

O: Like I already told you, I was depressed, yho! I should have gone for counselling after everything.

I: If I may ask, what was depressing you?

P: So many things were happening in my life, and again I am diagnosed with TB, and I thought I took medication to prevent TB, they said they are for 6 months, they increased for me to 10 months, and I took them for the whole 10 months but still I still had TB.

I: When did you finish taking TB prevention medication?

P: Hai! It long, they side its not for lifetime, they help for time been.

I: When they explained the box alone, how were you feeling? Felt like it could help?

P: Mhhm, I trusted it, yrr it will ring at the wrong times, it would scare me in the house.

I: It would scare you?

P: Yes, I would here sound like there is an alarm [making alarm sounds].

I: What was scaring you?

P: Hai... I stay alone in the house and am not expecting sound that I don’t know it is from where, I didn’t get used to it, that is why I returned it. it was scaring me.

I: Before, coming to the clinic did you know anything about the box?

P: Mhhm mhhm , I was told about it here at the clinic, they taught me here in the clinic.

I: How can you describe you experience in using the box?

P: Hai I didn’t experience anything serious, it was just irritating, and when I go home, I can’t go with it. Like when my mom just passed on I went home and left the box behind.

I: Why couldn’t you leave with it?

P: I had luggage, and I traveled with public transport so I was afraid to have too much luggage.

I: Okay, would you say there is anything that makes the box easy to be used?

P: I didn’t see anything to be honest, I don’t even want to speak about it, uhh I have been taking medication without this thing eh eh that says, I am helped by my kids, I get helped by my friends … they would just show me the fingers and I would know its time.

I: So, before you had… before you had the box, what assisted you to take medication?

P: It is my phone; I have a phone that I set an alarm at 6 7 o’clock take my dose... I set an alarm just when am done taking my dose, I set alarm for the next day same time.

I: Would you say it I easy to use that method you are using to remember medication?

P: Mhhm mhhm it is easy, easy, easy and the time I will drink, if am deep sleep it wakes me up and if it rings and I don’t hear it will ring again, it rings 3 times, at o’clock, 5 past and 10 past.

I: You said you stay where?

P: Do you know Mogwase?

I: Mhhm

P: Makwejane, am sure about 5 km

I: Are you working?

P: Mhhm mhhm, I earn social grant, I have two months earning.

I: Has anyone asked you about the box?

P: Yes xxx (intern name) he asked me why I am returning, I explained to him and showed him the SMS [cough].

I: Beside people from the clinic, is there anyone else who saw you with the box at home?

P: Mhhm mhhm I stay alone

I: So, no one ever saw you with the box?

P: Mhhm mhhm.

I: So, the friends that reminds you of medication? Were they happy when you showed them the box? Have you ever told them about the box?

P: I will tell you something neh? I was afraid to tell them I have TB, but they know that I would lock the gate, they said for the first two weeks the bacteria it is still active I must not be with people, so they didn’t visit.

I: If I may ask, why were you afraid to tell them?

P: Hai I don’t know, I was sad, telling the truth, I was so sad if it was possible, I could have killed my self

I: What made you sad?

P: It is like I have all the diseases... you understand me? Am so vulnerable even if a bee passes, I will catch a disease

I: So, you mentioned that you were afraid to travel with the box?

P: I went, I traveled, oh you mean with the box?

I: Mhhm

P: I have never, I used it for 2 days only, when I open it so that it does not ring, I would open it and close medication was not inside, it is too small it cannot accommodate those pills mhhm it is too small those pills are this big.

I: Why were you not able to travel with it?

P: Luggage mhhm I was afraid to have too much luggage, and it did not make sense to me. I have alarm in my phone, it doesn’t work at all this thing this thing was not working for me.

I: You said your medication was outside the box?

P: Mhhm I put it in just 2 times only, then I open it switches off, then tomorrow it switched on and rang I returned it.

I: Why did you put medication outside the box?

P: I don’t need it, I felt like I don’t need it, serious.

I: So, is there anything that you saw been helpful about this box?

P: Mhhm for me it did not help, there was no need to keep that box.

I: Any difficulties that you can say you came across with this box?

P: Mhhm mhhm it was only the sound that was scaring me, and you can see now we have loadshedding [power cuts] , so I wonder if it is the fridge? Or what it is, when I go outside, I find that it is this box yrr.

I:So, has the box ever rang more than once in a day.

P: Mhhm mhhm, after I open it, it switches off.

I: It won’t ring again?

P: Mhhm it was giving me work having to have to open when it rang.

I: So, you have received SMS’s?

P: Yes

I: Can you describe, how you feel about the SMS’s

P: They were boring me shame, sometimes you find that am seating with people ting thing, when I check it is talking nonsense.

I: Have they ever called you from the clinic regarding missing medication?

P: I was called by xxx (intern name), I said I have taken my dose, the box is making noise at home, and I have taken my dose and I have taken my doses, I am taking my doses, I will still take my doses until I finish my treatment duration, I have beautiful kids that I love.

I: How were you feeling when xxx (intern name) called?

P: I spoke to him, he is very humble xxx (intern name), I spoke to him nicely, he asked why I didn’t take my dose, I said hai I do take my dose am just not using the box.

I: Mhhm

P: Mhhm

I: How did you feel when they said they will send people to come and visit to talk to you?

P: Hai ... I was feeling comfortable because I knew that am taking my doses and I go to fetch my medication and my sister when I come here, she speaks well with me, saying I will get healed dot worry TB its curable, you understand? I was feeling just okay she was helping a lot she was healing me.

I: How would you feel if there were sending other people coming to check upon you if you have taken

P: Something that once irritated me here at the clinic, they say I must not mix with people, so other patients were complaining who am I skipping line, the security guy stood up and said they must not be surprised when I skip the queue, I have TB [laugh] yrr yeses he was the one who irritated me the most

I: He disclosed your status to people?

P: Mhhm yeses

I: How did you feel when he did that?

P: Yrr I was angry, I told him I could sue him, I could get him arrested, he can disclose my status like that, and he did not even ask me first but xxx (intern name) intervened and spoken to him.

I: So, what can be done to the box to assist people more and effective?

P: It must ring before … or if am taking my dose at 7, before like at 5 to 7. Mhhm if it rings 7 or 5 to7 then you will know your pour water but now it is sending SMS saying you didn’t drink your dose, don’t forget to drink your medication before midnight, you saw I showed you the message what are they saying? [laugh]

I: Besides changing the alarm what else can we change that the box helps people more?

P: Mhhm I don’t know I, *nna ke Malala a laotswe.*

I: You said you are?

P: *ke Malala a laotswe*[laugh]

I: What does that mean?

P: Am alert, and I dont need to be helped, I help myself. I set the alarm myself when it rings, I take my dose then I set alarm for tomorrow same time.

I: You said the alarm, scares you, what can you say about the volume its high?

P: Mhhm mhhm, no its not loud that box, it rings just smart. I don’t need it, don’t need it *nex nex nex.*

I: I want to understand the part you said the box scares you

P: You know what is happening? If I didn’t open it, have you ever heard electric fence? It rings like electric fence.

I: Is there anything they can change so that it does not scare people?

P: Mhhm mhhm they should just leave it, it does not work, the must stop wasting money.

I: What do you like and dislike about SMS’s?

P: They should send me important messages, that I must not forget to take my dose before midnight … my last medication it is for 8 o’clock late so at 12 o’clock I will be long asleep.

I: With the phone calls, what do you like and what do dislike about them when been reminded about medication?

P: Right now, am happy, they called me yesterday xxx (intern name) saying I must not forget my appointment today, I forget a lot of things which are deep mhhm I forget a lot, so he called me I was happy, and I appreciated serious.

I: Let’s say someone is not taking their doses, then they get an SMS like that would they be happy?

P: Yes, at least, take something, take one, o ehh ehh xxx (interviewers name) are we not finishing?

I: Is here something you would like to tell us about this box as we are reaching the end of this interview?

P: Mhhm mhhm not there is nothing you know, they told me everything xxx (intern name) I understood him, just I found that thing not working for me the way I expected.

I: Is the anything you would to talk about regarding, SMS, phone call and the home visits?

P: I am happy with them, at least it shows the concerns of our nurses, mhhm and it encourage someone, and if someone is encouraging me why am I not encouraged? It gives me courage … but it almost caused me mental disorder, eish its was very painful xxx (interviewers name) and those pills are this size of my finger.

I: When you say you almost had mental disorder what do you mean?

P: Here I am still encouraged.

I: How did it almost caused you mental disorder?

P: It was painful that why me, it is the ARV and there other 2.

I: Right now, how are you feeling with this whole thing of taking medication?
P: Mhhm I have accepted

I: Have you ever received counselling from TB room.

P: I was feeling like I need it, but the sister spoke to me, telling me TB is curable I will get healed.

I: How did you feel when all that happened?

P: I was just feeling well.

I: Alright, we have reached the end of this interview, we thank you for your time and all the answers that you gave us if there were any question you would like to ask after here you can feel free gore you can ask them, and we will answer.

P: Thank you xxx (interviewers name) am fine.

I: Ending time 09:10.

**Glossary**

Loadshedding- power cuts

Nex- no
