# Supplementary material for: Acceptability of Digital Adherence Technologies to support people with drug-susceptible TB in South Africa
Source: PLoS One. 2025 Sep 24;20(9):e0332103. doi: 10.1371/journal.pone.0332103 (PMC12459780; doi:10.1371/journal.pone.0332103)
Supplement: S4 File — (ZIP) [file pone.0332103.s004.zip › S4 Transcripts/PwTB/IDI 18_PwTB.docx]

**TRANSCRIPTION NOTATIONS**

| **Label Key** | **Meaning** |
| --- | --- |
| **I** | Start of each new utterance by the Interviewer |
| **P** | Start of each new utterance by the Participant |
| **N** | Note taker |
| **{ }** | Indicates that details were changed or pseudonyms were used to anonymise data |
| **( )** | Indicates the description provided to anonymise data |
| **XXX** | Words were omitted to anonymise data |
| **-** | Breaking into a sentence by the next speaker |
| **…** | Pause or drawn out words |
| **[ ]** | Indicates noise made, e.g. [laugh], [sigh], [pause] |
| ? | Beginning of utterance by unidentified speaker or questionable text |
| **[inaudible segment]** | Unclear section of the recording |

I: Do you agree to be audio recorded?

P: Yes.

I: Date xxxxx (interview date), location: xxx (clinic name), PID:xxx, Time: 10:30 am, Language used : Setswana. Briefly tell me who are you?

P: XXX ( Participant’s name).

I: Uh mmm, who do you stay with?

P: I stay with my parents, my sister, my little brother, my son, and mom.

I: Mmm, so when did you find out that you have a lung disease or TB?

P: Mmm, last December as when I try to sleep I would wake up and experienced shortness of breath, I was sweating a lot, I couldn’t eat, and I was vomiting. I went to the doctor, the doctor gave to me a referral letter to take to hospital. At the hospital they told me to go for an X-ray, that’s the way I found out that I have TB.

I: Mmm, what do you use to arrive at the clinic?

P: I walk or they brought me with a car.

I: Mmm, okay, you have mentioned that you couldn’t eat, and you were sweating a lot, besides these two symptoms, which other symptoms are you familiar with?

P: Mmm, I used to smoke- 2017 when I smoke, I used to cough a lot in the morning when I wake up, I quitted cigarettes, and I was okay. 2018 I was staying with someone then when I wake up, I was coughing, I felt like I have a cat on my chest like the eye lash of the cat. I felt like I have something on my chest that is scrubbing me, but I didn’t take it seriously, I thought maybe It is flu things like that.

I: During 2018 yeah, when you say you had the eyelashes of the cat on your chest, did you go consult about it?

P: No, it was happening only in the morning, I was just coughing only in the morning. Then I just moved on with my life, but then I was always experiencing sinus and blocked nose.

I: When you consult at the doctor about night sweats, did you still experience the eyelashes of the cat on your chest?

P: No, I no longer experienced that, but I was coughing deeply in the morning, and when I was coughing my chest was painful.

I: Mmm, okay, so do you know this box?

P : Yes, they gave it to me, I think it was February.

I : Mmm, oh okay. So how did you feel when they explain the box to you or when they gave the box to you?

P : They explained to me that it will help me take my medication on time, it will alert me and also keep my TB medication. They also explained that it keeps a record when I don’t drink my medication; it reports to them.

I : Who explained to you about the box?

P: I forgot her name, but it was a lady, I think she was here in the morning.

I: How long was the explanation about the box?

P: It took about 15 minutes.

I: Oh, in that 15 minutes, do you feel that is there something missing that she should have said about the box?

P : Mmm, that it sends an SMS very late “don’t forget to drink your medication.”

I: Oh, okay, is there something that you would like to change about how she explained the box to you?

P: Not really, she was thorough, she was on point when explaining to me, everything that she explained to me I understood.

I: Mmm, have you ever seen the box elsewhere except here at the clinic?

P: It was my first time seeing it when they gave it to me here at the clinic.

I: How did you feel when you first found out at the hospital that you have TB?

P: The way I was so sick I took long to come to the hospital as I had experienced weight loss. I was weighing 110 kilograms when I came to the hospital, I weighed around 80 kilograms. The way I was so sick I’ve accepted to take the treatment the way it is until I finish and do everything that the doctors said I must do, including the type of food I should eat and how to take my medication thins like that.

I: So, before I move further with my questions, when you said you took long to consult with this sickness, what made you not to consult immediately when you were not feeling well?

P: Mostly when it turns like coughing, I just went to the pharmacy and bought medication like flu mixture or something or maybe sometimes I just drink Med lemon and tomorrow I feel fine, and life goes on.

I: Just to understand, is there any difference and similarities between the symptoms of flu and TB?

P: No, usually it is coughing for me, I was coughing and experienced blocked nose sinus, but now since I have started medication, I no longer have sinus and I don’t even snore the way I used to. The symptoms when I started sweating and when I woke up in the morning the sheets are wet, things like, and that is when I realized that this is something else and not flu and then I went to the doctor. Then the doctor explained to me that it might be something else and I must take the referral letter and consult at the hospital.

I: Oh, okay. So you started TB medication last year December?

P: Yes on the 15^th.^

I: So, you received the box in February?

P: Yes, early February, just after January.

I : Was there a time when you took medication without the box?

P: Yes, December and January.

I : So, now that you are using the box to take your medication and you have experience in taking your medication without the box, is there any change in your medication intake?

P: Mmm, before I was taking medication at 8:00 in the morning, but sometimes I would oversleep and wake up around 9:00 and you would find that in the house there's no one to wake me up. The box really helped alerts me everyday at 8 a.m. In the morning I wake up, warm the water which takes 2 to 3 minutes and I drink my medication.

I: The box alarms you ,but besides the box, what reminded you to take your medication?

P: The box is always besides me, so when I wake up maybe at past seven I wait for the box to alarm me or sometimes I open the box to take out medication and put it ready for consumption then when the box alarms me, I just open it and close it.

I: At the beginning you mentioned that you are staying with your parents, little brother, and your son, first day you told them you had TB, how did they feel?

P: Mmm, my mother was not there she went home, and I was left with -actually I was staying at my house, then I relocated because I stayed alone, and my wife relocated to Mhlanga so I figure out that if I stayed alone I would fail to make myself food things like that. I relocated back home so that I can get support in the morning with prepared food soft porridge things like that, because the medication that they gave me rifafour required that you drink them before you eat. So, after 30 minutes, that is when you can have your meal. I had a problem that is why I relocated back home to stay there so that I can get support and food at all times.

I: So, the day they gave you the box to take home- did you say they know the box at home?

P: All of them know the box.

I: So, when they saw the box or when you explain to them, how did they feel? Before you respond to that question, do they know that you have TB?

P: I explained to them.

I: That time you explain to them, how did they feel?

P:No, they said they will adhere to the procedure, I explained to them that in the morning we must open windows so that we can get fresh air. I came back to the clinic to ask if won’t I infect the people I live with and then they gave me containers so that they can produce their sputum. I returned them back to the clinic to be checked, but when the result came back, they found out they are all negative and we wear masks things like that.

I: So, if TB is a transmittable disease like flu when they brought their sputum and the result came back negative then you saw that you infected nobody with TB

P: Most of the time I keep my distance, whatever that I’m using they don’t use it my 2 little bottle nobody touches it and I have my own plate, teaspoon, and folks so that when I use them, I eat I use them nobody will use them just to avoid that I must not transmit TB mostly I would explain to them.

I: How did having TB change your lifestyle?

P: For the time it was difficult because there are things that you should leave because you have to take treatment, things like cooldrink, energy drink and all that. You must drink juice and avoid spicy food because it is painful when you vomit. When I cook for myself, I only pour salt, I avoid things like Chili’s, atchar and I don’t eat them until today. I never drank alcohol since I started treatment, there are certain things you must stop so you can take your treatment properly.

I: So, going back to the box, what are the challenges you experienced using it?

P: When I go to Mpumalanga I usually have to make a U- turn because I forget it or when I go sleep at my place, I forget it too in my bedroom so I have to go back home or come back early in the morning before 7:00 AM to take it. Challenges is that you forget the box at home especially when we are going because sometimes, someone comes take you quickly and when you go back you no longer go back home maybe you go sleep at your place so I have to call somebody at home to bring medication or I must go fetch it.

I: When you mention that you can forget the box, are you working?

P: I am not working but a mechanic so sometimes there are people that come to my home and take me to go and diagnose their cars things like that because now I cannot do any physical job I just diagnose and come back home.

I: So, when you say you don’t do physical jobs, where you doing them before?

P: I no longer have energy like before the medication makes me tired things that I could lift up before now they take me time to lift, and I get tired quickly.

I: So, let’s go back where you mentioned that you often go to Mpumalanga, let’s talk about the challenge that you mentioned. How often do you forget the box at your house then you have to make a U turn?

P: I visit with my luggage and a small bag where I put my medication so you will find out that I have packed my medication in the small bag then I forget it on top of my bed. I only take my luggage to the car then I forgot to take my medication then at home they call me to say you forgot your medication.

I: So, that I may understand you well ,do you forget the whole bag?

P: Sometimes it happens that when you pick your bag when you are about to take it somebody else calls you in the house you just leave what you are doing then you focus on something else then you take the bags of the kids then you go straight to the car, and you no longer check what you have left behind.

I: I want you to tell me your experience when visiting Mpumalanga with the box, how is it?

P: The box helps me because of the alarm so when I oversleep my son wakes me up and say, “daddy, daddy, the box is ringing” then I open it and take my medication things like that.

I: You mentioned that you diagnose cars, and you are a mechanic, so using this box, has it affected your work in a certain way?

P: No, the box has not affected my work it improved my work because when I oversleep 8:00 o’clock it alarms me then when I don’t open it or answer to it let’s say I’m outside I receive an SMS maybe it alarmed me 9 minutes or two and I don’t respond then I receive an SMS saying, “don't forget to take your medication” then I remember that I must go and drink my medication.

I:When they explained to you about the box, what were your worries?

P: I asked them if the battery won’t finish and they told me to bring it every time I come for refill. I must bring it and they are the ones who change the battery, but ever since they gave it to me, they haven’t changed the battery or charged it.

I: You mentioned that people you live with in your house know about the box, is there anyone that knows this box?

P: Yes, I showed it to my colleagues and my friends that I have this thing that helps me in the morning to take my medication things like that.

I: I am glad to hear that, when you showed your friends and colleagues the box ,how did they feel about it, what was their reaction towards the box?

P : No, they were happy that there is something that helps me to drink my medication because when they explained to me that if I don’t open the box first day or secondly day, on the forth they can send people to come here at home to take me to drink medication.

I: So, is there a need to tell your friends that you are on TB medication, and you are using the box?

P: The need is that how do I go about drinking my medication, how do I manage to wake up and take my treatment then say no at the clinic they gave me this then I go to the house and show it to them. When I open the box, it makes a sound and light a small green light then when you don’t open it this red one then I explained to them that it alarms me at 8 they have set it using the time I take my medication.

I: Was it easy to explain to them about the box?

P: Yes, it was easy, just a box that stores medication and it has your information on how you take your medication and what time must it alert you.

I: Is there someone who had TB in your house except you?

P: It is my uncle, but it was a long time ago, 1989, it’s only him who had the disease.

I : Since you have started using the box, have you opened it more than once?

P:Yes, sometimes you open it to take out your medication, then close it and it alarms you again after a few minutes, and you open it again and close it again, then it stops.

I: How often does that happen where you open the box and you close it, before you have to open it again?

P: Oh, maybe twice or three times a month.

I: Is there a day where you missed your medication?

P: Often times when it alarms me, I respond to it.

I: So, you mentioned that when you take your medication a box has GPS tracker, they can see that you didn’t take your medication.

P: When you take your medication you open the box when it alarms you don't get an SMS but if you don't, maybe 30 minutes before you take your medication you receive an SMS that tells you that don’t forget to drink medication.

I: For me to understand you well, when you open the box when it alarms you don’t get an SMS, but when you prolong you get an SMS.

P: If that box alarms you for a long time let’s say 10 to 15 minutes you get an SMS on your phone reading, “don't forget to drink your medication.”

I: Is there a day you took long to open the box?

P: Yes

I:How often does it happen you find out that they sent you an SMS?

P: Mostly it happens let's say maybe I go somewhere and I take time to open the box or maybe I took out the medication and left the box at home ,I will drink the medication at 08:00, but I left the box. When I come back home, I’ve found the box we sell red light.

I:How often does it happen whereby you open the box take out the medication and leave the box at home?

P:Mostly it happens when I go to visit my sibling because he stays at my house then I know that I will be coming back the following day in the morning or maybe after 8 so I’m lazy to take the whole box mainly I open the box then I take the two pills end 1 small pill then I wrap them with a tissue then I go.

I: When you come to the clinic, is there something that they show you where they can see when you have taken your medication or not maybe you've been asking yourself, how do they know that I have taken my medication or not?

P: I’m not sure about that but with the box if you don't open it more than two days they call you, if they don’t call you then they sent people to come and ask you why you don’t open the box for more than two days what is happening.

I:There is something that we call adherence calendar, have you ever seen it that on this day you drank your medication, or you didn’t drink your medication. Have you seen the adherence calendar, or have they told you about it?

P:eeh, that blue card or the green one I don’t know the adherence calendar, but everyday I tick when I’m done drinking my medication.

I: I’m sorry that they didn’t show your adherence calendar but it’s like a green card where you tick but now it’s digital it’s on the tablet. So I’m sorry you didn’t see your adherence calendar where you can see when you have taken the medication and when you did not.

P: Yes

I:Earlier you mentioned that when you leave your house to go back home you were looking for support that you will get. In terms of the box and the support you get at home, what kind of support are you talking about exactly?

P: Food you know when you are on medication you have to eat like three or four times a day and I have to eat things like porridge, soft porridge, things like that .So you would find out that I sleep so they wake me up to tell me to come and eat things like that.

I: Tell me your feelings including your experience about the support you receive ,including the box you got from the clinic, I want your feelings in these two directions?

P:The box makes me happy because even at home they know it. Even when it starts ringing, they call me, and you’ll find that sometimes I’m watching TV and I take it with me because there is medication that I take after breakfast. Then I take the box with me so I can drink the other medication at 9. They can see the box then they asked me what is happening then I explained to them so they can also understand it and next time when I leave the box at the dining room when it starts alarming, and they are near they can just take the box and give it to me in my bedroom to tell me that it’s time to drink your medication then I take the box open it and drink my medication.

I: So that I must understand you well, you take your TB medication at 8:00 AM and afterwards you mentioned that there are other medication that you are taking at 9:00 AM. So, inside the box, is there TB medication and the other medication.

P: Yes, they relate, but TB medication you drink them before breakfast then the other medication you drink them after breakfast because they require you to eat first.

I: I understand you now, so in short, explain to me how the box helped you to put your medication at the same place?

P:Yes, I used to use a toiletry bag, but it was messy inside so with the box you can pack your medication well, so you know when these ones are finished ,you drink the other ones then the box is very neat you understand me.

I: Yes, I understand .So, at the clinic there is something that is called differentiated care model and it a method where you find your SMS, the ones you were speaking about and when you don't take your medication properly, they can call you and even when you don't take your medication for a long period they come at your home. So, SMS is only for that day but phone call it’s for two days and a home visit it's more than two days or a week. So out of these three differentiated care model I told you about, which one do you know, and you have experienced?

P: The SMS.

I: The SMS that you were receiving when you haven’t taken your medication at 8:00 AM?

P:Yes, let’s say I’m outside for 30 minutes and I don’t hear the box when it’s ringing. I receive an SMS on my phone and I run to go to the house open the box then drink my medication.

I:So according to your explanation, you receive an automated SMS, but we have a reminder SMS.

P: Reminder SMS I came to the facility to explain that I received a reminder at 12:00 AM then they said they will fix it, but now I no longer receive them.

I: So that I may understand, reminder SMS you take your medication at 8:00 AM in the morning so can you tell me about your feelings when you receive an SMS at 00:00 AM?

P: I was troubled, it is confusing I was asking myself which medication are they referring to at 00:00AM.Then I came to the hospital, I met the sister then I explained that there is an error SMS and she said sorry it’s a mistake she will fix the time.

I: So, have you received a phone call where they remind you to take your medication?

P: Phone call that I have received is from the hospital, they were asking me how is my medication, how I am feeling things like that and when am I finishing my term of taking the medication.

I: So, the phone call that you received from the hospital is part of support that you get, I’m happy to hear that. So, when you using this box, here I want to experience you can take it along and travel with it or you take out medication then leave the box at home in your experience is there a barrier or something that can stop you from using the box?

P:I don’t see any barrier because right now I have given myself time to stay at home I usually don’t just go out for the duration of two days or three days. I am always at home 24/7 unless they asked me to go somewhere, I have stopped many things just so I can drink my medication properly and eat my food at proper times like 12:00 PM. I’m eating, at 7:00 PM, I’m eating things like that so it’s really up to you to give yourself time in this lifetime to say in this six months I’m going to change my lifestyle.

I: Okay, I understand, so earlier you mentioned that you are happy with the box, but you didn’t mention in full your emotions like your level of satisfaction concerning the box?

P: Honestly speaking right now it is like part of my life you understand, I don’t go somewhere and leave it behind. Mainly when I take out medication and leave the box, I would be in a rush somewhere and I would be certain that the following day in the morning I will be back home to continue but if I’m going, I take it along. Last month I was in Durban for two days and I took it so everywhere I go I take it; it was next to me in the morning, and I drank my medication things like that.

I: When you say this box it’s like part of your life, was there a period where you took medication for two months without it?

P: Yes, but that two months it was difficult because I missed the times sometimes, I overslept, and I would wake up around 9 and I have to take my medication at 8:00 AM. So, I used to have that little fear that I missed my medication by an hour. Then when I received the box, the box made things easy for me when I’m asleep it wakes me up then I take out the medication, so I’m happy that there is something that is helping me because when I’m asleep and they are asleep we both sleeping it becomes somehow so since I received the box my things are simple.

I: So, you mentioned that you overslept before they gave you the box so you having that little fear that you have missed your medication by an hour so, do you stop taking the medication for that day?

P: No, I drink my medication it is just that guilty conscience that I didn’t drink them on time.

I: According to your experience does the box simplify your medication intake as we have said before that we are conducting a study so for this box to be easy and more friendly to use where can we improve?

P: Mmm (yes), especially with the rifafour they are longer so you bend them they don’t fit and the box gets too full you can’t close it properly, there are other medications that don’t fit and you must add them all that time so I would say the size but the shape and sound it's okay.

I: So, when they can increase this size for the rifafour to fit

P: Yes ,just a little bit because you bend two lines of the rifafour for them to all fit and so that you can pack them well.

I: I’m happy to hear that suggestion even your recommendation I’m thankful for it. By the time I was asking you about the SMS, the phone calls, there is somewhere you mentioned something I’ve noted it as self discipline. You spoke about that everything is up to you since you don't have much of differentiated care where I would say they were calling you, visiting you at home to come and check up on you. So, here I’m looking for your experience like your perception or idea. I’m looking at the differentiated care model but yeah, I request your recommendation or suggestion. If a person let’s not shrink their medication, will the box be the appropriate mode to support the patient?

P: So ,you know how is the box it requires you to have it close to you if you sleep it must be close to you so that when it rings you must not you must not be far from it, You must not leave it in the kitchen or dining room knowing that you stay alone in your house because it is going to ring and ring, and ring until it lights the red light and it alerts at the hospital. If you have siblings in your house, your sister, or your mother they can bring the box to you so that you can drink your medication. So, for me it is like we are helping each other they bring a box to me so that I can drink my medication.

I: So, this mode when the patient is not drinking their medication and they sent people to check up on the patient, is it working or will it work for someone who is not taking their medication?

P: Mmm, some people just give up easily and they say they’re just tired to drink medication every day because medication it’s not nice. Honestly speaking drinking medication everyday is not nice. Sometimes I drink my medication then eat then vomit, I start to worry that I also vomited the medication but then I remember that I must not drink them again as they told us in the hospital things like that. Sometimes you would drink the soft porridge then you vomit you start asking yourself what is making me vomit then I realized that it’s the milk then I must eliminate it things like that.

I: So, when we send people to check whether when you drink medication you vomit?

P: Or you experience difficulty eating food doesn’t go through.

I: So, you have talked about a person giving up on medication due to certain reason. So, during counselling session, what should they say to the patient to make sure that the patient takes back the medication?

P: Mostly it depends on yourself ,I can come to queue on the line and another person come to the line but end up leaving saying they will see what they can do. It is their life that they put on danger when they don’t drink their medication because TB when you don’t drink your medication it becomes worse, they will take you to the hospital and admit you there for the whole year. To avoid such things, you must drink your medication and bring your sputum so that they can check whether you are still positive, or you are getting cured or what is happening about your life.

I: I’m glad to hear that, so there was automated SMS- sorry reminder SMS that you received or that you were receiving. So, according to your experience that you have with those reminder SMS, what did you like about them or and what did you not like about receiving that SMS?

P: So when I wake up in the morning let’s say I can stay and watch TV in my bed having insomnia then I receive an SMS at 12:00 AM ,then I start asking myself questions that why am I receiving the SMS that reads “don’t forget to drink your medication” at this time, which medication because my medication I drink it at 8:00 AM. So, it becomes a bit confusing to get an SMS that late hence I came to ask that lady why am I getting a late SMS then she said she is sorry she will change it on the system.

I:So, we talked about differentiated care model, so which way is more effective, the SMS, a call or home visit?

P: For me an SMS is more effective, a home visit they can come to your house and not find you there ,but an SMS talks to you because you always have your phone with you.

I: So, if the SMS is more effective, which message can you advise us to use or what can we change about the message to show that it is more effective advice us how can we write it?

P: Yes, so that I can also correspond and tell you my problems about medication and you also respond to me with an SMS, so that I don’t receive a “don’t forget to drink your medication” SMS only, but if I have a problem and I don’t want to come to the hospital I should also be able to reply. If I want to ask a question, I should be able to take my phone and reply to you as you are here at the clinic so you can go ask the doctor that we receive such an SMS from the patient, the patient is asking such a question so that the communication between us doesn't end only with a reminder SMS that reads don’t forget to drink your medication because sometimes you can receive changes in your body like getting some symptoms and allergy to avoid making trips to the clinic when I just don’t feel okay I should just send an SMS because I don’t mind coming to the clinic Monday to Friday just to ask then the nurse would just say to me it is side effects of the medication. The medication is not yet used to you are body so it is still fighting the sickness things like that.

I: So, now we are wrapping up so the way we gave the box or the way the box is helping people is there any gap?

P: I don’t see any gap the box is just fine the problem is only the size, if they can increase the size just a bit so that the medication can fit well then everything will be okay.

I: As we said that we are conducting the study according to your experience do you think that the box could have been implemented way before?

P: Honestly speaking, yes even when others start the medication it should be part of the package so that when they leave the clinic they would have received everything. After receiving counselling then they should explain the box to the patient so that when they arrive at their house they don’t struggle they can put their medication inside the box and in the morning, it will alert them to drink medication you see.

I: Mmm, According to your explanation as they mentioned this is part of the study, so when this study trial ends in coming years do you see this box helping people the way it helped you?

P: Mmm, at times we just queue on the line having our medication box and we discuss everything including the medication and the box, then we have different views. Some would say my medication is full the box is no longer ringing then I said no if they give you medication that takes about two months the best thing to do you must just only put one box and the other one put it away and when you finish the first dose then that’s when you can insert the other one don’t let them pile up to the small box. We give each other advice as we sit outside waiting to be assisted since we are not the same there are those who are smarter than the others some put all medication into the box, and it does not fit then the box no longer alarms them and it can't be closed so if it is not close it won’t ring so we advise each other as we sit outside that you must not pile up the box just put only one dose then later on you will fill up the box. The box won’t close if it is too full so you will find that a person complaints that the box no longer alarms them then when they are asked questions, they can't give answers because they don’t close the box well. The box consists of metal if you don’t close it well it won't ring at all.

I: Oh, I understand you so what are the part of the advice that you give to each other when you are outside?

P: No, we just talk about our wellbeing that how is the medication treating you, so you find that someone says for me the medication it’s like this the other one says it’s like that and the other one says it’s like this concerning our disease and we include the box also that what time are you taking the medication the other one would say at 11 but you find that at that time they are at the house so they took out the medication and left the box at home things like that because maybe they are working things like that.

I: So, let just see we have a person who is on TB medication and does not drink the medication when we send the tracing team, they don't find the person at home, When the nurse talks to the person the patient does not listen so according to your explanation that you give each other advice as you sit outside. So which approach will work to make sure that the patient is reinitiated to take back the medication properly?

P: Yeah, just like me taking TB medication and the other one refuses to drink their TB medication, I would make sure that we drink our TB medication at the same time so we can support each other drinking at the same time as teamwork you understand because they cant drink on their own. Mainly I warm my water then I mix hot and cold water then I drink my medication my kettle is always warm I always have my glass or a flask at my bedroom so that’s when it’s time to drink my medication I do just that. Most people find it difficult to drink medication I don’t know how to explain it like you get annoyed there is a point that you reach, and you see that it’s like this medication is not working ,but then that’s just a mentality of a person. I: So, unfortunately, we have reached the end of our interview even though I feel like there is more you would like to share with us.

P: Yes, I took a decision from the first day they gave me medication that I will take these pills the way they tell me. Since they gave medication, I have never had any alcohol, I love alcohol there’s no one that doesn’t love alcohol ,but then since I have never drank any alcohol. This medication they make you not to burp when you eat that thing makes you to vomit you understand so the option that they give you is that eat a green apple you see that green apple the whole packet cost about I think 50 bucks it is expensive but when you had it you can eat your food and then later on you can still eat without any challenges because when you didn’t have that apple the food just pile up in the oesophagus. That is the problem about medication that makes people to give up to not follow the procedure of drinking medication properly .

I: I’m sending the green apple, how do you eat the green apple before or after the medication?

P: You drink your medication then you eat your breakfast then you eat the apple so that the food you going to eat later the solid food like rice you can eat it. If you don’t eat the apple, you cannot eat you feel like there is something in your oesophagus so even at home, they complain that you don't eat well you feel nauseas, so the apple makes you to burp and open a channel for you to eat that is what I learned. So ,when I don’t have apples here at home, I get worried I just get angry at anyone here at home, so my mother would leave the house and go to the shop to only buy me the apples and put them in the fridge even the little children they don't eat them because they help me a lot. I’ve been at the hospital they told me I must eat green apples, salads, and fruits things like that so in my bedroom I always have a basket of fruits avocados things like that so even if I feel hungry, I just eat some fruit and I go back to sleep.

I: I didn’t know about the green apples, but that information would help us a lot as we were talking, I wish we had enough time but for today I’m going to wrap up here but before I end the interview what are your final thoughts concerning TB, the box anything regarding TB?

P: The life you live it’s yours so if you don’t take care of your life, it is only going to cost you later on the way it has cost me. This disease I saw it when it started but I thought it was something else, I thought it was flew instead of me doing what going to the clinic or hospital to consult you see only to find out the later on when it was too late for me when I was no longer coping because I went to the hospital at 3:00 AM in the morning with my water and blanket. I started getting treatment around 8:00 to 9:00 AM and I came out of hospital at 1:00 PM but during that time I had decided about my life that everything that I used to do I’m leaving it and I’m focusing on my medication. I usually meet with some of my friends they tell me that I’m scarce what is happening, I just tell them that I am home and usually I spent a lot of my time at home because once you galivant on the street you mess up your diet things like that. Medication requires you to eat but at the streets what are you going to eat Bunny chow because they said we mustn't eat such food like chips, cooldrink, But once you have friends, they buy alcohol and if it happens you buy juice then they'll tell you that you consider yourself to special and you end up drinking the alcohol so once you tell yourself let me give myself time six months is nothing you understand. I am finishing my medication. On the 9^th^ of this month actually is this week and I’m happy about it.

I: My brother let me thank you about everything that you have shared with us and some of the things I didn’t know so you gave us enlightenment and information in full that will help us achieve TB medication and how we should improve the box like you were saying about the size…

P: Even food, when you eat the wrong food it will mess you up, you end up stopping medication because of the food that is not right. You can bread in the morning, eat bread again at 12:00 PM then later in the evening you eat breath then sleep that is highly impossible at least for me they recommended soft porridge future life that is what I drink in the morning sometimes I make myself a shake, I mixed with Banana gives you energy The medication is working normally but when you eat food like bread cooldrinks it is acid it ends up depressing the pill and you wake up in the middle of the night to vomit you'll find out that the food that you are eating it is not right.

I: When you are on TB treatment you don’t change only your diet but also your lifestyle...

P: If for instance they braai meats things like Raja ( curry powder), chili's you must tell them to stop using the. I tell them when they prepare my meat to only pour salt and if I’m eating porridge, it must also have soup.

I: How do you simplify that soup?

P: So that soup is a best combination with porridge because if it’s not there you can’t eat your meal the food get stuck on your throat.

I: As I said you have a lot of information that could help us.

P: I used to come here every day and explain to them but they kept on saying it’s side effects and they told me what type of food I must stop eating.

I: All the information that you gave us we will try to change certain things, all the recommendation, we will try to implement everything that you said.
