# Supplementary material for: Acceptability of Digital Adherence Technologies to support people with drug-susceptible TB in South Africa
Source: PLoS One. 2025 Sep 24;20(9):e0332103. doi: 10.1371/journal.pone.0332103 (PMC12459780; doi:10.1371/journal.pone.0332103)
Supplement: S4 File — (ZIP) [file pone.0332103.s004.zip › S4 Transcripts/PwTB/IDI 19_PwTB.docx]

**TRANSCRIPTION NOTATIONS**

| **Label Key** | **Meaning** |
| --- | --- |
| **I** | Start of each new utterance by the Interviewer |
| **P** | Start of each new utterance by the Participant |
| **N** | Note taker |
| **{ }** | Indicates that details were changed or pseudonyms were used to anonymise data |
| **( )** | Indicates the description provided to anonymise data |
| **XXX** | Words were omitted to anonymise data |
| **-** | Breaking into a sentence by the next speaker |
| **…** | Pause or drawn out words |
| **[ ]** | Indicates noise made, e.g. [laugh], [sigh], [pause] |
| ? | Beginning of utterance by unidentified speaker or questionable text |
| **[inaudible segment]** | Unclear section of the recording |

I: We thank you for agreeing to do this interview with us, so, we are requesting permission to record you.

P: Yes, there is no stress.

I: Do you agree to be recorded?

P: Yes.

I: Uh, date of the interview xxxx (interview date), location XXX (clinic name) clinic, language used Setswana, PID number xxx, start time 13: 07, so where do you live?

P: I stay here in xxx (area name), at XXX (name of area).

In xxxx (area name)?

P: Yes.

I: Do you use a taxi to come to the clinic?

P: Uh, I walk, I don’t use a taxi.

I: How long does it take you?

P: Uh, 15 minutes.

I: Mmm.

P: From where I stay to come here.

I: How many times do you come to the clinic in a month?

P: I come once; my medication lasts me for a month.

I: Okay, so at home, who do you stay with?

P: I stay with my mother and 2 siblings.

I: So, when did they diagnose you with TB?

P: They are doing what?

I: When did they tell you, you have a TB disease?

P: They just saw me changing, I wasn’t the same xxx (patient name) they know, I started losing weight and they asked themselves what is going on. I should go and check what is wrong and that is when I came to the clinic, they took sputum, went to test it, that is when they called and told me it is TB that was making all those things.

I: How did you feel when they told you, you have TB?

P: Uh, I wasn’t feeling okay for the first day, but after a month I was okay, I got used to it, you see it is life and I won’t live well if I give in.

I: You are saying you didn’t feel, okay?

P: Ehh I felt like am not living like other people, it was my first time taking medication, I was asking myself what is going with me, but at home, they encouraged me not to stop medication because I won’t survive, I won’t even live, until I got used to it.

I: So, what do you know about this box?

P: Uh, this box helps to time remember time for medication.

I: How does it reminds you?

P: Eh because if a person is taking- it rings on the time you are supposed to take your pills, and you remember.

I: So, who explained this box to you?

P: It was explained by xxx (intern’s name), she is the one who gave me and told me it will help me with this and this.

I: Can you please explain those things she said it will help you with?

P: She told me that the box will show me when it is time to take medication, it will show me when it is time for me to come and collect medication here at the clinic.

I: So, how does it show you those things?

P: Yes, it shows me.

I: How does it show you?

P: When it is the date to come to the clinic *vandag* (today) it shows orange, when it is time to take pills, it illuminates green light and it rings and makes a sound.

I: When she was explaining to you, how long did that take?

P: She does what?

I: How long did she take to explain how this box work?

P: Am not sure, mmm *Hai* (no) around 10 minutes or 15 minutes, I didn’t stay long. She told me if there is something am not understanding, I should tell her, but I heard her and understood what she wanted to tell me at the end.

I: Would you say it is easy to understand how the box works?

P: Yes, it is easy, it is not hard.

I: Is there anything you think they should add or change when explaining this box?

P: Uh, uh, it is just fine the way it is.

I: So, when did you start seeing this box?

P: The first time I got my medication, I didn’t know anything about it. It was then I realized it existence.

I: You never saw it from anyone before?

P: Mmm, mmm, it was the first time I saw it, I was asking myself what it is for when they came with it.

I: What makes this box easy to use?

P: It does what?

I: What makes it easy for people to use this box?

P: It rings, mmm that alarm makes it understandable. It’s simple to use because it doesn’t have a lot of work, when it rings you open, you do what they said you should do when you done, you close and it will be silent.

I: Mmm, have you came across any difficulties while using this box?

P: Mmm, mmm there is nothing difficult.

I: Have you had network problems?

P: What?

I: Had network problems maybe the box not ringing for a day?

P: Yes, once, but it rang twice when it was supposed to ring once; it rang at 10:00 am and it rang again at 10:00 pm, but I opened and closed again.

I: How many times did it ring twice?

P: One time.

I: Just once?

P: Yes.

I: Are you working or attending school?

P: Am not working nor going to school.

I: What time do you take your medication?

P: I take it at 10:30am.

I: Has it ever happened that you were not home at 10:30?

P: Does what?

I: That you are not home at 10:30.

P: Uh, uh every day, uh ,uh there were days were you found that I was out, but I would come back around 11 or 11:30. I would remember ,but at home they would me that it was ringing. I would take medication when I get home.

I: Have you ever travelled with the box?

P: Mmm, mmm it stays at home, I never travelled with it.

I: So, beside reminded by family, what else reminds you to take medication?

P: It was the alarm because I put it next to me where I sleep, I can hear it even when am outside. I can hear it when it rings, so I was reminded by it.

I: Is there anyone who ever asked you about the box?

P:They asked me about it only at home.

I: So, at home when you told them about the box-

P: -They were happy because eish they knew am not used to taking medication, and with the kind of life I live, I will obviously forget. I will remember for 1 or 2 days, but at least with the box they know I will remember time for medication every day.

I: Mmm-

P: -Yes.

I: So, what was the reaction of people you live with at home to the alarm? How did they feel with this thing of alarm knowing that it is not talking to them?

P: They do not have tress; they are happy, at least it reminds me so I won’t skip dates.

I: Besides people from home, do you have anyone else who is supporting you on your TB treatment?

P: Yes, my friends are supporting me and people I spent time with at home, they tell me every time.

I: So, have you ever told them about the box?

P: Uh ,I didn’t tell them, I just told them how I was when I got diagnosed and they told me to have a long heart for them I will end up been healed and live just well.

I: You never told them you are using the box?

P: *Hai* (no) they don’t know.

I: How do you feel when you are telling people about your condition?

P: Uh, I just feel okay, I know that I will be healed one day, yes , but it hurt sometimes and would even ask myself where this disease came from because I was surprised when I started losing weight. Ehh that is one thing that hurt me the most when I think how I was at first. I thought it was flu, little did I know it a disease.

I: How long did it take you to accept that you have a TB disease?

P: It took a month, I saw after two weeks when I started changing. I started accepting that I am sick, but I never gave up, I said to myself I will finish where others are finishing.

I: Before they told you, you have TB disease, what did you know about it?

P: I didn’t know anything, I didn’t even know how long it takes to finish TB treatment, I only found out here at the clinic when they were explaining to me.

I: Who was explaining to you?

P: It was xxx (intern’s name) the box one, the one who gave me- she was the one who told me.

I: What can you say about the information that you got about TB?

P: She told me that TB-TB- I can get it from, eish from the air, she asked me if is there is anyone who has TB at home, I told her that there is no one who has it. She then told me that maybe I got it from my friends or someone whom we shared something to drink with, mmm just like that she told me how I can prevent this and that.

I: Who do you think is supposed to teach people about TB?

P: Who taught people?

I: Who must teach people.

P: I think it can be me because I have already experienced it, I already know what you must do and how to do it when you have this TB disease.

I: Okay, so do you find it easy to tell people about your disease?

P: It is not easy unless they are asking, then I will explain, but it is not easy if you are not asking. I keep quiet.

I: You have mentioned that you did not tell your friends about the box, what can you say is the reason you didn’t tell them about it?

P: They didn’t know, they only asked me when they saw me recovering, when I started gaining weight and changed in complexion. They asked what happened ,then I explained that it was this disease, you see, the mindset that I should tell them about the box wasn’t there, yes.

I: Okay, what did they say when you said that?

P: They were hurt, but they accepted that I will live with them even though am like this because eish I was short tempered at first, they saw that I no longer live well with them the way I was, they saw I was short tempered, and I was not talking. I was wondering what was going on with me, that is when they asked me questions about the matter.

I: Have you ever opened the box more than once in a day?

P: Yes.

I: What was happening?

P: I would come here to get more medication, then I would open again to put these new ones after I have opened in the morning, like today I opened in the morning when I got home and I will open to put these, but I never open countless times.

I: Beside when putting medication inside, do you ever or someone from family open?

P: Uh-huh, (no) am not sure because it stays in my room.

I: So, in your room, where do you put it?

P: Uh I put it where? On top of a drawer that is where I can hear it when it rings, I don’t hide it that I can’t hear it, I want to hear the alarm then it rings like if am sleeping and I wake up around 10:00, so that it can wake me up. I put it next to me, close to my head where I can hear it.

I: So, what do you put inside the box together with your medication,?

P: I put it with this card only (TB green card), just like the way they are, I just put them inside. I put it inside everything that I use for TB.

I: Okay, what can you say is the main thing that helps people with this box?

P: Uh, it helps to remind them remember time to drink their medication and always keeping their medication safe at all times.

I: How does it keep their medication safe; how can you explain that?

P: If you put your medication inside, it won’t get lost because it is in there, than leaving it in the kitchen drawer or somewhere and you won’t find when you are looking for it later, but if it is inside the box, you know where the box stays, and you know where to look for it.

I: Is there anything that the box helps with other than the alarm and keeping medication safe?

P: I don’t think so.

I: Uh, how important is the TB information provided at the clinic for people starting TB treatment? (…) education that they gave you here at the clinic about TB, how important would you consider it to be? (…) Okay, have you encountered any difficulties since you began using the box?

P: Uh-huh I have never.

I: Have you ever received an SMS?

P: Yes, I have received an SMS.

I: What did it say?

P: It was telling me that- eish I don’t remember well, but I remember I got an SMS.

I: Have you ever skipped a day without taking your medication?

P: Uh-huh.

I: Okay, how did you feel when you got that SMS?

P: I felt just fine because I knew what was happening, I didn’t feel anything.

I: Was the SMS talking about medication?

P: Uh, it was telling me that I will start taking treatment, eish.

I: Okay, were there any religious barriers preventing you from taking your medication? Or any faith that was preventing you from taking medication?

P: Uh-huh.

I: Do you share your cell phone with someone?

P: Uh-huh (no), I use it alone.

I: Have you ever had network problems? Maybe your box skipping a day without ringing?

P: Uh, first time it did not ring for 3 days straight, but it started ringing since then.

I: Have you ever tried to check why was it not ringing?

P: Uh, I was opening at the time they told me to take medication, but it was making a sound when I open it. Only when I open and close, but it was not giving me the alarm at the time to take medication.

I: So, at that point, what were you using to remember time to take medication?

P: I was just looking at the time on my phone straight, when it is 10 o’clock, I go to open, but after 3 days I heard it ringing.

I: Would you say it was easy for you to use your phone to remember the time?

P: I was not forgetting at the beginning ,but right now I do forget and I would hear it ringing. I was not forgetting when I was starting , I saw that I won’t get healed if I keep forgetting my pills while am this sick.

I: Okay, how satisfied are you with the box? How happy are you with the box?

P: I am really happy because it has helped me, it is the one that made me to be where I am today because every time it reminds me that it is time do this and this, am really happy, it has helped me.

I: When you say it has helped you, how?

P: It has helped me to remember the time and to keep medication safe inside that I don’t forget where I put it.

I: So, does it remind you to take your medication?

P: With the alarm and its lights display, you know it time for something just by looking at it.

I: What can we do on the box to help you to take your medication easily?

P: *Hai* (no), it is fine the way it is, it does not give problems.

I; So, what do you think about the size?

P: The size is also fine, but the earlier ones were larger (pill packets). These could not fit as it is, I had to cut them into smaller pieces. Although the earlier batch was big, the current batch fits inside.

I: Uh, what do you think about the volume?

P: With what?

I: With the volume.

P: Uh, the volume is also fine for me, I can hear it every day, I can hear it.

I: If you miss your treatment, we must send you an SMS to remind you that you have missed your treatment for that day-

P: -Yes.

I: How would you feel about receiving an SMS saying you have missed your treatment?

P: I won’t feel okay because I won’t have the thing that helps me feel well. I won’t feel well at all.

I: Would you say it is the right thing for the clinic to call someone who missed treatment?

P: Yes, it is the right thing to do.

I: If you miss more days, they call you to check up on you if you are okay, why did you miss medication. Do you see this as a good thing to call on patient for missing treatment?

P: Yes, they will be doing good by calling.

I: Last one, if they can’t find you on the phone or you keep on missing medication, they must visit you home, they are coming to check upon you, would you say it is the right thing to do?

P: Yes, they are right because they are showing that they care for us to get healed.

I: On these 3 things, which one do you think can help people with TB more?

P: They must visit at home, sometimes when they call you, you can’t answer the phone sometimes you have left it at home and then they can’t talk to you. I think home visits are better.

I: So, out of all the things that they do at the clinic, including calling people, which one do you think helps people on TB medication?

P: It the box.

I: The box?

P: Mmm.

I: Why are you saying the box?

P: Because it reminds you, this box, it tells you to do as they instructed you, like here at the clinic you can’t come every day, but it helps a lot because you live with it full time.

I: What is the main function of this box and which aspect of it is more helpful?

P: It is the alarm.

I: Beside alarm, is there anything else that you are using to remember time?

P: Mmm, mmm, it is only the alarm, am already used to it even in my mind when I hear the alarm, I remember it is time for the pills, when am not home it is then when I use cell phone, but when am at home I got used to the alarm, it is the one to give me time.

I: So, how often does it happen that the alarm rings and you are not home?

P: Its not often am usually home. I would leave around 2 o’clock ,but early morning I always hear because am always around.

I: So, according to you, what is it that you think we can improve, so that we can help people taking TB medication.

P: (…) I can’t think of anything.

I: What can be done to encourage people undergoing TB treatment to accept the medication box? What method can be used when giving the box to patients to increase acceptance?

P: I don’t know.

I: Okay, who must teach people about this box if we want to give it to someone?

P: (…) It is those who have used and experienced it.

I: You are saying it is you who experienced who can teach patients about the box?

P: Yes.

I: So, as an experienced person, what can you tell me about the box if I was a newly diagnosed person on treatment?

P: When you start with this box, I would show you the dates for your clinic visits and give you time to drink your medication and also tell you that it will also help keep medication safe.

I: You mentioned something about the date to come here?

P: Yes, it will show the orange light, when it shows orange light, it knows that the pills are finished you must come. So, it reminds you if you have forgotten date to come here.

I: What made you accept this box?

P: Uh, it was the way it was explained, I felt like it good for me, good for my treatment. That is when I accepted it.

I: When you say it is good for your treatment, what do you mean? (…) as you said it goes with your treatment, how so? (…) okay, is there anything you feel like we didn’t mention that you would like to talk about regarding the box?

P: Uh-huh (no) , there is nothing.

I: Uh, anything you would like to talk about regarding SMSs, phone call and home visits?

P: (…) Uh-huh.

I: Are you fine?

P: I am fine.

I: Okay, thank you very much we have come to the end of our interview, thank you for time, ending time 13:48.
