# Supplementary material for: Acceptability of Digital Adherence Technologies to support people with drug-susceptible TB in South Africa
Source: PLoS One. 2025 Sep 24;20(9):e0332103. doi: 10.1371/journal.pone.0332103 (PMC12459780; doi:10.1371/journal.pone.0332103)
Supplement: S4 File — (ZIP) [file pone.0332103.s004.zip › S4 Transcripts/PwTB/IDI 2-PwTB.docx]

**I**: Do you agree that we start our interview today?

**P**: Yes, I agree.

**I**: Okay. Date; xxx (interview date). Location; xxx [clinic name] . PID is xxxx. Time; 11:35am. Language used; Sesotho.

**I**: So, can *mama* (mother) tell me you stay with and where you live?

**P**: I just stay with the girl and the grandchild only.

**I**: *Mama* (mother) stays with the daughter and grandchild…We are three in the house.

**I**: Okay… So, in short, could *you* tell me what do you use to come to the clinic?
**P**: With a taxi.

**I**: How many taxis do you take?

**P**: I use only one and then when I get home and when I come here. All in all, is two taxis, because when I come here, I use a taxi and when I get back home, I also use a taxi.

**I**: Oh okay. How do you feel when you use a taxi to get to the clinic?

**P**: I do not understand.

**I**: When you come to the clinic you use a taxi.

**P**: [Inaudible segment]

**I**: Okay. So, do you *mama* (mother) know about TB?

**P**: I always hear them talk.

**I**: When they talk-

**P**: Because I do not cough-

**I**: So, when they talk what do they say about it?

**P**: About what?

**I**: The TB illness, when they talk?

**P**: I have not heard anything [Inaudible segment]

**I**: Please speak up.

[Pause]

**I**: So, how many times do you come to the clinic?

**P**: How many times do I come?

**I**: Yes

**P**: Once a month.

**I**: Oh, once a month?

**P**: Yes, they give me a date for me to come.

**I**: Okay. So, when you come in short.... [Pause]. Okay, do you perhaps know these tablets or pills?

**P**: [Pause] *Eeeii* (yes) I once taken them. I drink them. I think they once gave me, have you given me these? Yes, I once took them. [Inaudible segment] Yes, I once took them, they gave me.

**I**: So, does *mama* (mother) know about lung disease?

**P**: To cough?

**I**: To cough is one of them.

**P**: Isn’t coughing lungs? I do not know.

**I**: It’s… Coughing is also a sign or way to show that someone has lung disease or illness.

**P**: To cough, no. I just cough but the problem is the pain that is here.

**I**: Can you please tell me about the pain you feel.

**P**: It is the one causing me to be unable to walk upright.

**I**: So, the pain you feel did you perhaps tell the sister that you have the pain there?

**P**: I cannot remember. Did I tell you sister? I cannot remember. Actually, I once had stroke, then I felt this pain here. I once had stroke, I was numb on this side, but it has been years.

**I**: So, but did you tell them about the stroke you once had?

**P**: [Inaudible segment] I think I had pain

**I**: Oh okay. So, when you come to the clinic and talk to the sister, what does the sister tell you about- what illness?

**P**: She has never told me about any.

**I**: So, when a person has a lung problem, they feel like coughing, and they feel heat**-**

**P**: Yes, I used to cough and feel the heat, but now I do not.

**P**: To cough I used to cough.

**I**: Okay. So, when a person is sleeping, and they feel overwhelmed by heat can you please tell us about your experiences of being overwhelmed by the heat while you are sleeping.

**P**: Yes, I would just wake up feeling overwhelmed by the heat.

**I**: So, when you came to the clinic and explain to them about you feeling overwhelmed by the heat while you are asleep, what did they say your experiences match which illness?

**P**: They never told me. Actually, I never asked them.

**I**: Okay. To lose weight or to drop in body weight?

**P**: Yes, I was not this size, I used to be a bit of weight. I thought the weight loss was due to old age, I thought my time was up, I was ageing.

**I**: Okay. [Pause]. So, when you lost weight and being overwhelmed by the heat. How long did you stay with these symptoms?

**P**: It was long, I did not even keep track of how long did these happen. I thought it was old age, I thought I was ageing.

**I**: Okay. So, do you know about this sticker or label?

**P**: I cannot hear.

**I**: This sticker or label, do you know it?

**P**: No, I do not know it.

**I**: Is it your first time seeing it today?

**P**: Yes. I do not know it.

[Pause]

**I**: So, this sticker we place it like this on top of the pills [demonstration] do you remember?

**P**: Remember what?

**I**: That this sticker is placed on top of the pills?

**P**: Now I do not know, they only written the number of times I should take them in a day.

**I**: How many do you take in a day?

**P**: I cannot remember as to whether I was supposed to take 2 or 1, I do not know, in the morning, during the day and at night when I have to sleep.

[Pause]

**I**: How does *mama* (mother) take these?

**P**: Hey [inaudible segment] but I think I once drank them, but I do not know.

**I**: So, do you know this box?

**P**: It only rang at 12.

**I**: I cannot hear.

**P**: It rang at 12.

**I**: Okay. When it rang what did you do?

**P**: Pardon me.

**I**: When it rang at 12-

**P**: I knew I had to take my medication.

**I**: I cannot hear.

**P**: I knew I had to take my medication.

**I**: Okay. So, can you tell me in short about this box, what do you know about it.

**P**: I know nothing. All I knew was I drink my medication when it rings, I would know it is twelve.

**I**: Who explained to you about this box when you started coming to the clinic?

**P**: I now do not know. All I know when they gave is that when it rings, I drink my medication.

**I**: When you say when it rings you take your medication, please tell me where do you place your medication?

**P**: In the house, in the wardrobe drawer.

**I**: So, you were not placing the medication inside the box?

**P**: No… I would place them only when it rang knowing that I should take my medication.

**I**: So, when it rang at 12 you would take your medication-

**P**: Yes and drink it.

**I**: But you were not placing them inside the box-

**P**: No, no. I was not putting them inside the box.

**I**: Okay. So, when you take your medication when it rings, afterwards did you open the box?

**P**: When it rings, I would open and close it, knowing that it is twelve, then take my medication and drink.

**I**: So, you were reminded by the box to take your medication?

**P**: Yes, that it is time to take medication.

**I**: Okay. At home you said you stay with your daughter and grandchild?

**P**: Yes.

**I**: Besides this box, who else reminded you that it is time to take your medication?

**P**: I reminded myself, I did not forget.

**I**: So, how did you feel to have this box besides it reminding you to take your medication?

**P**: [inaudible segment] because I have to take my medication

**I**: To have something that reminds you to take your medication, how do you feel to have something that reminds you?

**P**: When it rang, I would also have a watch… and see that true it is 12.

**I**: So, who did you tell that you are taking medication?

**P**: I did not tell anyone; I just told this girl of mine.

**I**: So, when you started telling the girl that you are taking medication, how did she feel?

**P**: Whose feelings**-**

**I**: The girls’ feelings-

**P**: Ah she does not care [Laugh] she does not care.

**I**: So, when you say she does not care?

**P**: [inaudible segment] it is because she never asked me anything, she did not ask a lot of things, about how am I going.

[Pause]

**I**: So, when they gave you this box, what were the worries of taking medication with the box?

**P**: Worries?

**I**: Yes.

**P**: Mine? All I wanted was to live. I just wanted to get better.

**I**: So, when mama had this box, was there someone who told you what was this box for?
**P**: No, no one told me.

[Pause]

**I**: So, according to your experience with the box, it was to work with you to take your TB medication.

**P**: I do not understand.

**I**: It was to work aligned with taking your medication.

**P**: To take medication?

**I**: Yes.

**P**: Where? Here at the clinic?

**I**: To remind you.

**P**: Yes, yes when it rang, I knew I had to take my medication. Even when I was outside if it rang, I knew I had to take my medication.

**I**: So, it first you stated that when it rings at 12 you take your medication.

**P**: Yes, I drink them.

**I**: And then you were not putting the medication inside the box?

**P**: No, I was just putting it on the side. I had somewhere I placed them but when it rang, I would take the medication and drink, I was not putting them inside the box.

**I**: Did they perhaps tell you that the medication should be placed inside the box?

**P**: No, they did not tell me, they did not tell me anything. Was I supposed to place them inside the box?

**I**: Pardon?

**P**: Was I supposed to place the medication in the box?

**I**: We place medication inside the box.

**P**: Oh, I was just placing them aside the box. So, knowing that when it rings, I should drink. I would put them like this [demonstration] I did not put them inside the box.

**I**: Okay, I understand mama.

**P**: Is the box yours? I mean the box is it yours? Does it belong to the clinic?

**I**: It belongs to the clinic mama.

**P**: Oh.

**I**: Yes.

**P**: The truth is the box rang at 12. It would ring and I would drink my medication.

**I**: When you look at this box when it rang, what else reminded you that it is time to take your medication on the box?

**P**: [Laugh] I do not know what will I say when I answer to this. It would have a red reflection.

**I**: Pardon.

**P**: It would have a red reflection when it rings, and I would know that it is twelve and I should take my medication.

**I**: Did they explain to you about that red reflection and its purpose?

**P**: Pardon?

**I**: Did they explain to you about the red reflection?

**P**: No, they did not tell me anything. I just thought it is time for me to take my medication. The box would ring honestly, at 12 it would ring.

[Pause]

**I**: At home where you stay, do people from the clinic visit you where you stay?

**P**: They know where I stay, yes.

**I**: Did they visit you where you stay?

**P**: No, they did not come. [Pause] At times they would visit all communities.

**I**: So, when they visited the community, would you in short explain what they were doing?

**P**: They were checking up on us. They were just checking what problems we have and so forth.

**I**: So, how did you feel to have people who would come check you at your homes?
**P**: I thought it was the law of the clinic that sent them out.

[Pause]

**I**: What else was difficult about using this box?

**P**: I was always free, as long as I took my medication. [Pause] Where is the box coming from? From the clinic?

**I**: From Aurum, then we brought it here at the clinic.

**P**: Oh, it is yours?

**I**: Yes

**P**: Oh.

**I**: Was there a day you would open the box multiple times?

**P**: No, I would just open it at 12, knowing that it is my time for medication… when it rang.

**I**: Okay. [Pause] So, was there a day I would say went by without you taking your medication?

**P**: No, I drank my medication… I drank them. *Ai* (hey) but you speaking another type of Sesotho [sigh] You speaking Sotho from Free State. [inaudible segment] are you also Sotho? You are Tswana? Where did you meet? Here at Work? [Sigh] You are speaking Sotho from my home. What did you say your surname is?

**I**: I am a xxx [interviewer’s surname]

**P**: xxx [interviewer’s surname], but the xxx [interviewer’s surname] are not Sotho, my neighbours, they talk Pedi.

**I**: Mmm

**P**: You are speaking xxx[Language] from XXX[Area]. XXX[Area]. My home is in XXX[Area].

**I**: Okay.

**P**: Yes, but I no longer have parents, you can see my journey is almost over, but originally home is in XXX [Area]. [Sigh] you speaking Sotho from my hometown.

**I**: Thank you mama (mother).

**P**: [Sigh]

**I**: Thank you mama.

**P**: But the xxx [interviewer’s surname] are from XXX[Area] you are a xxx [interviewee’s surname] from where, you speak Sotho perfectly. They speak Pedi those I know, they are the xxx [interviewer’s surname], they are my neighbours.

**I**: Okay, I am pleased to hear that.

**P**: Your Sotho [Sigh] it is from my hometown you know.

**I**: Okay. So, can mama please tell me… Thank you once more mama.

**P**: Okay.

**I**: So, when people from the clinic come to visit people at home, was visiting people at home helpful?

**P**: I would not know. I do not know… because you needed to be taken care of… but it was long ago, long ago.

**I**: So, when they visit in most cases when they arrive at home and talk to you, what do they say?

**P**: They ask us how do we feel, not knowing now because it has been long, they have stopped now

**I**: So, can mama please tell me about your feeling regarding the box that is used to take medication with.

**P**: Feelings how?

**I**: How happy are you to have this box?

**P**:, I was satisfied. I did not have a problem, I had no doubts, I was satisfied. [Pause] Are you saying it comes from you?

**I**: I cannot hear?

**P**: I mean the box, where is it coming from? Is it from you not from the clinic?

**I**: It’s from the clinic.

**P**: Okay, I did not have a problem, all I wanted was to be alive and get healed.

**I**: Okay, for you since the box worked for you what can we as the clinic do to make sure that the box works at its best on your side?

**P**: I do not know what to say when it comes to that. The box at 12:00 would ring to be honest; it would ring. When it rang, I knew it was 12:00 and need to drink my medication.

**I**: I cannot hear?

**P**: Are you going to tell me about these pills? I see them being put like this.

**I**: I placed them here because I wanted you to tell me about them.

**P**: They do not have a problem, I used to drink them. The only problem is this pain.

**I**: Even now do you still feel the pain?

**P**: Yes, it is still there, now when I go to the doctor, they say it is blood.

**I**: Are you getting any help with the pain you are feeling?

**P**: What?

**I**: The pain you have, are you getting help for the pain?

**P**: [Sigh] when I come to the clinic… I even thought it was old age, but when you are old would you feel pain?

**I**: [Sigh] I do not know. So, when you are using this box, what do you like and do not like about the box?

**P**: It is not that I do not like it, I get satisfied when it tells me on time, it would indicate when it is time. It would ring at 12:00 , 12 midday not at night. Even when I have forgotten when it rang, I would know that it is time to drink my medication.

**I**: Is there a day whereby it rang several time in one day, this box?

**P**: It rang at 12:00.

**I**: Pardon

**P**: It rang at 12:00. At night it would not ring, just at 12 midday.

**I**: Okay. Did you say you have a cell phone?

**P**: No, it has broken.

**I**: Okay. So, when the clinic needs you, how do they get hold of you?

P: They have never wanted me. But I have to buy it, because I had it, but it got damaged-

I: Mmm-

P: So, I have to buy it. I will be the one looking for them due to me having health problems.

I: When they told you to come for this interview today, how did they manage to get hold of you?

**P**: They usually give me dates to come, so they gave me a date to come, they just say come on this date, so I come here by dates.

**I**: So, at home, does your daughter have a cell phone?

P: Yes, she has. But I do not know her cell phone number.

**I**: Okay, it is not a problem.

**P**: I had it and these kids damaged it, it was on the table. When you have children [Sign]

**I**: When you observe do you think this the route of calling patients who do not take their medication of great help?

**P**: Pardon

**I**: When a person is not taking their medication accordingly? The way we use of calling them to remind them to take their medication.

**P**: They only tell me when to come, just like they tell me to come on a specific date, I know I am going to get my treatment and so forth.

**I**: So, when you started taking this medication, was there perhaps where you saw this box?

**P**: No, I saw it when I was coming here, I came here for long without seeing it. I cannot even remember who gave me this box. I cannot remember.

**I**: Okay.

**P**: I cannot remember.

**I**: You spoke about seeing the red colour here.

**P**: Yes, it would ring at 12:00 and have a red reflection there and I would know I have to drink my medication.

**I**: So, when observing this box, what helped you with taking your medication?

**P**: At times I would forget and when it rings, I know it is 12:00 and I would go and drink my medication.

**I**: So, you were assisted by the box ringing?

**P**: Yes, but at times I would check the time. At times we tend to forget, and you would not know what time is it, only to find out that it is time for you to drink your medication.

**I**: Okay

**P**: At times I would check time, and it would be 12:00 and I would know it is time for medication, so it times I would forget, and when I have forgotten it would ring and that would be it is way of reminding me. You understand?

**I**: Yes, I hear you.

**P**: Yes… Is the box staying behind now? I am not leaving with it.

**I**: No, it is yours.

**P**: It is mine? So, why did they say I must come with it?

**I**: Did they say I must come with it?

**P**: [Sigh] all I knew is it is needed.

**I**: We will talk about it once we are done.

**P**: That it is needed. I thought the box is needed.

**I**: Oh okay. So, when you observe this box do you think it helps people? Without it to take TB medication?

**P**: That one I do not know. [Sigh] I honestly do not know. Because for me it rang, at 12:00, it rings. It rings and I drink my medication.

**I**: According to the manner it works for you, do you think it would work for someone using it the same way?

**P**: I do not know anyone who has it, with regards to that I do not know. Because we were a few and I thought I was the only one who has it. I do not know if others had it, I do not know. But it rang at 12:00, at 12:00 it rings. I do not know; did I take it here? Yes, I took it, it rings at 12:00 it rings, and it rings exactly on time at 12:00, which is the time I take my medication. [Pause] Oh you go around clinics? I mean you visit clinics.

**I**: Yes.

**P**: Oh, you work aligned with the clinic?

**I**: We help people who have illnesses related to lungs.

**P**: Mmm… with the Sotho that irritates me, your Sotho irritates me [Sigh]

I: [Laugh] [Sigh]-

**P**: It irritates me because I miss home [Sigh] you speak perfect Sotho; it makes me happy

**I**: Oh, I make you miss home?

**P**: Yes, my hometown.

**I**: Ah, please forgive me.

**P**: Yes, you speak my hometown language perfectly. By the way you said…. Oh, my neighbour, xxx [interviewer’s surname], and you would not say. Are the xxx [interviewer’s surname] Sotho? Be honest, the xxx [interviewer’s surname] are not Sotho, are they Pedi?

**I**: Mmm.

**P**: So, you speak Sotho while you are a xxx [interviewer’s surname]? Did you take it from the Sotho speaking people?

**I**: Yes.

**P**: It is because my neighbour is a xxx [ interviewers’ surname] and they are Pedi’s, they are from Petersburg. You are speaking my hometown Sotho, from Free State [Sigh]. Where do you stay? Where is home?

**I**: We are almost done mama.

**P**: Yes.

**I**: So, please tell me, how long have you been using this box?

**P**: Yoh, I do not know but it has been long. I have never recognised how long have I had it; all I knew was it was in the house. I honestly do not know… Ey, you speaking my hometown Sotho [Sigh]. And the lady belongs to which tribe? Is she XXX[Race]? Is she XXX[Race] or XXX[Race]?

**I**: She is XXX[Race].

**P**: [Sigh]

**I**: So, mama this box when you observe it while using it, was there something you think this box is not doing?

**P**: I was satisfied by it to be honest. I had no worries about it. I was satisfied.

**I**: Okay… So, in short can mama tell me, does people from the clinic visiting people at their home considered as being helpful?

**P**: Yes, it was helpful, I saw it being helpful. But now they no longer do it. But it was helpful.

**I**: Which approach is working best in order to make sure people are taking their medication, the phone call or the visiting was helpful?

**P**: I think coming to the clinic.

**I**: Oh, for you is for a person to come to the clinic?

**P**: Yes, coming to the clinic.

**I**: Why do you say the approach of coming to the clinic is the helpful one?

**P**: I see it being the better one because now I do not see them, they used to make visits.

**I**: So, at home is there anyone who is taking medication that is the same as this?

**P**: No, it is just me.

[Pause]

**I**: Okay. Mama, we have reached the end of our discussion today. Is there something you would like to tell me before I reach my end?

**P**: No, I have no worries, what you tell me makes me satisfied.

**I**: Okay.

**P**: I am satisfied.

**I**: I am glad to hear that. So, mama let me take this time to thank you for coming to the clinic to come have the talk we had.

**P**: Yes.

**I**: I appreciate all the answers you gave me, and all these answers are going to be used. So, I appreciate the time we have been together, I appreciate.

**I**: Time ended it is [Inaudible segment] Time ended 11:15am.

21:03
