# Supplementary material for: Acceptability of Digital Adherence Technologies to support people with drug-susceptible TB in South Africa
Source: PLoS One. 2025 Sep 24;20(9):e0332103. doi: 10.1371/journal.pone.0332103 (PMC12459780; doi:10.1371/journal.pone.0332103)
Supplement: S4 File — (ZIP) [file pone.0332103.s004.zip › S4 Transcripts/PwTB/IDI 20_ PwTB.docx]

**TRANSCRIPTION NOTATIONS**

| **Label Key** | **Meaning** |
| --- | --- |
| **I** | Start of each new utterance by the Interviewer |
| **P** | Start of each new utterance by the Participant |
| **N** | Note taker |
| **{ }** | Indicates that details were changed or pseudonyms were used to anonymise data |
| **( )** | Indicates the description provided to anonymise data |
| **XXX** | Words were omitted to anonymise data |
| **-** | Breaking into a sentence by the next speaker |
| **…** | Pause or drawn out words |
| **[ ]** | Indicates noise made, e.g. [laugh], [sigh], [pause] |
| ? | Beginning of utterance by unidentified speaker or questionable text |
| **[inaudible segment]** | Unclear section of the recording |

I: Uh brother, do you agree for us to record our interview for today?

P: Yes, I do agree.

I: Uh todays date is xxxx (interview date), the location: XXX (clinic) and the PID of a patient is xxx. The time is now 12:23 PM, the language that will be is Isizulu.

I: Uh brother could you please tell me a little bit about yourself and who you stay with?

P: Uh ok. I stay in xxxx (area in a suburb) ; xxxx (suburb) with my children and my women and we both use the box.

I: Uh so, you both use the box?

P: Yes.

I: And how did you find out that you have TB?

P: Mmm I found out that I have TB the day we to the clinic for my child who was sick at the time. So, after my daughter got tested for TB, they decided that me and my women also test too.

I: Oh, so you both -

P: - We took an X-ray test.

I: When then did you start using-

P: -The box-

I: Did you start using it right after you started taking TB medication or?

P: Yes, immediately after I started taking medication.

I: Oh ok. So, do you walk or use a taxi when coming to the clinic?

P: Mmm I walk but when I am in a hurry, I then take a taxi since it quite a distance.

I: it is a distance?

P: Yes.

I: How many taxis do you take when coming to the clinic?

P: I usually take two taxis because sometimes it difficult to find a taxi coming straight to the clinic. So, I take one from home to the nearest taxi rank then from the taxi rank to the clinic.

I: Ok. So, you said you came to the clinic because your daughter-

P: -Yes, we came for our daughter who was sick.

I: Oh, so you realized she was unwell at the time?

P: Yes, she had been complaining about chest pains for a while and we decided to visit the clinic because we suspected that she had asthma, but the results came back negative. Then she was tested for TB, and she tested positive.

I: Were you feeling sick when you were tested for TB? Did you suspect anything, or you were feeling fine?

P: Uh I was ok; I had nothing.

I: Ok. How did you feel when you found that that you have TB?

P: Uh truly speaking, I was not worried about myself but my daughter who is still young and innocent and we tried our best to take care of her more than ourselves.

I: Ok. Was she cured?

P: Yes, she survived it and she is now able to eat and has started attending school again.

I: Ok. Uh, did you know anything about TB? Did you know anything about it?

P: Mmm TB- I only knew about its existence and nothing more.

I: …Mmm you did not have information?

P: Mmm

I: Oh ok. Do you know this box brother?

P: Yes, I do know the box.

I: When did you receive the box?

P: Uh it been time though I cannot recall the exact date.

I: Who explained to you about the box?

P: Uh no one explained- I was told to use the box and look after it.

I: If I may ask, where did you get it?

P: Uh I got it here.

I: Who explained to you about it?

P: Yes, uh they told me to put my pills inside it, uh it will remind me-it beeps when I open it and it also beeps when I did not take my pill.

I: Oh… so who explained to you about the box? Was it a nurse or someone else?

P: Eish I do not remember.

I: You do not recall?

P: Yes, I do not remember.

I: All right, how long did it take them to explain to you about the box? Do you remember how long?

P: About 40 minutes.

I: 40 minutes?

P: Yes, because they took time explaining to women and I since she speaks a different language.

I: Ok. How did you feel after they explained about the box? Were you satisfied?

P: When they told me that I am supposed to keep the box in the house I asked myself what will happen if I have to go somewhere; do I take it with me or leave it at home. It was difficult at first but as the time went by, I got used to it and I told myself that I must take it with me whenever I go.

I: Oh, so you said you found out that you have TB when you and your women visited at the clinic for your child who was sick?

P: Yes.

I: Then you tested for TB?

P: Yes.

I: Have you told someone that you have TB beside your women who already knows

P: Yes, I have.

I: Who did you tell that you have TB?

P: Close neighbours, friends, and family.

I: Ok. How did they react when you told them that you have TB?

P: Uh they did not take it seriously; they joked about it, and they also told me to take care of myself because I can only be cured by adhering to my treatment.

I: Oh if you take your medication correctly?

P: Yes.

I: Ok. Uh Did you tell other people about the box? Did you show them the box that you use?

P: Uh my brother, uh my close friends. I told them that I am using this box to store my pills and it reminds me when it is time for me to take my medication and it also beeps when I did not take my medication.

I: Oh ok. How easy was it for to understand everything they explained to you about the box?

P: It was not difficult to understand everything.

I: Ok. Have you ever seen this box from someone?

P: No.

I: Mmm

P: It was the first time seeing it here.

I: How do you feel about the box since you started using it? Could you please explain to me?

P: Uh the box is ok and it easy to carry when going somewhere. It also reminds me when it is time to take medication but sometimes it beeps when I am not around, in that way it easy for me to miss it. If there could be a way for it to alert someone on their phone as it beeps so that they can quickly return home to drink their medication.

I: Oh, remind you that you have just missed your medication?

P: Yes, immediately report on the phone.

I: Ok, I hear you and we appreciate your input. Uh brother are you currently employed?

P: Uh I am not working at the moment; we lost jobs during the first wave of COVID-19.

I: How long have you been unemployed for?

P: We lost jobs following the first wave of COVID-19 as companies had to close shops.

I: Oh ok. What else do you put inside the box?

P: I put my medication.

I: What medication?

P: TB medication.

I: Ok. Where do you place your box at home?

P: Mmm I put on the rubber where it is safe and out of reach of children.

I: When the box beeps box, you open it, is that right?

P: Yes

I: Have you ever opened the box twice in a day by mistake? Have you ever opened it when you have already drank your medication?

P: Mmm… yes, I would open it when I have to refill and take out empty plastic containers.

I: Oh, by that time you have already took your medication?

P: Yes

I: Ok. So, do you open it for the second time to refill medication?

P: Yes, to refill.

I: Ok. Beside opening it to refill, have you opened it for a different reason?

P: No, I also make sure that it stays close all the time.

I: What makes it easy for you to use the box?

P: It helps by reminding me when I must take medication because I take them in the morning before breakfast. So, the box reminds me and helps me keep the time though sometimes I take medication before time to avoid missing my daily dose since it beeps when I am not home. It reminds me that I must take my medication first thing when I wake up at 06:30 AM.

I: Ok. I hear you brother saying that when you wake up you eat breakfast and then drink your medication.

P: No, I take pills on an empty stomach and sometimes I take them before time because I forget sometimes. So, when I wake up in the morning before time, I open the box and take them so that it does not beep thereafter.

I: Oh, you drink your pills before the box-

P: -I take them before it reminds me.

I: Ok. Does it beep again after you have taken your pills before time?

P: No, it does not beep.

I: Oh ok. Uh have you encountered any challenges with the box ever since you started using it.

P: I did not have problems besides the issue of malfunctioning it once had; it would just beep at midnight after I have already taken medication. It would ring in the middle of the night, and I would just open and close it.

I: What did you say was happening with box?

P: I am saying sometimes it would just ring for no reason because I would have already taken medication.

I: Oh ok. And for how long was it doing that?

P: it happened twice in a month.

I: Ok. So, do you take your pills in the morning or in the afternoon?

P: In the morning.

I: Oh, So It would ring in the morning and again in the afternoon?

P: Yes, normally it would ring in the morning when I did not open it. Now I did not understand the reason for it to ring again at midnight, so I would just open and close it so that it stop.

I: Mmm, so how did you feel when the box was ringing for the second time when you have already taken medication?

P: It disturbed me to a point where I told my women that I am having second thoughts about this box, but I told myself that it the way it supposed to work or something, however, it since then it never beeps at midnight.

PI: Did you report the malfunctioning of the box to someone at the clinic?

IP: No, I did not tell anyone here at the clinic.

PI: Mmm…ok. Did you tell anyone about the box beside your partner?

P: Yes, I have.

I: How did they react when you told them?

P: People ask me what do I put in the box and I tell them that its for my medication and then they ask what kind of pills and I tell them that its TB medication. Sometimes they ask me if I I carry it with me and is not heavy to carry around but I tell them that it not heavy at all; its portable, plus it can fit in my bag. I am able to carry it when going somewhere.

I: So, how do you feel about explaining to people when they ask about the box?

P: Uh, it was tough for me to explain to people about my TB status before, but I realized that it better for me to share so they become aware and careful especially people that I used to share a cigarette with, you know. We gave one another funny names as we joke about our conditions since they are also taking medication for other chronic diseases. One friend of my we call him VR-6 and they call me GTI, so we joke about it just like that and no one take it as offence.

I: Ok. Earlier on you mentioned that sometimes you would take the box with you when going somewhere. Did it happenthat you go somewhere in the early hours of the morning and take the box with you?

P: Yes, I would take it with me if I got a piece job somewhere.

I: Oh, so you did not mind taking it with you and that people might ask question about it?

P: No, I did not mind at all, and I never faced a situation like that before.

I: Ok. You never had issues even in the place of work?

P: No, I never had issues.

I: Ok, I hear you…Uh beside the alarm, is there any way in which you are reminded to take your medication?

P: Uh there is an SMS that I get on the phone but sometimes I would receive this SMS even when I have taken medication.

I: Oh, you were getting an SMS reminder?

P: Yes, I used to get an SMS at midnight.

I: Oh ok. How many times did you receive this SMS?

P: Yes, I got it few times.

I: Mmm

P: That is why I ended up taking my pills before time because I noticed that I do not receive a reminder when I took them before time, however, I do not receive these reminders anymore.

I: How did you feel about getting SMS reminders even when you have taken your pills?

P: To tell you the truth it did not sit well with me because I would ask myself why was it happening like that when I am taking my pills, but as the time went by, I got used to them and I was somehow motivated by these SMSs; it really show that someone out there cares about us.

I: Mmm you said you only stay with your partner and a child?

P: Uh we are four in total.

I: Oh, who is the fourth one?

P: My son; last born.

I: A child?

P: Yes.

I: So, did the last born also get tested for TB?

P: Yes, actually we all got tested for TB.

I: Ok. What did the results say?

P: He also tested positive for TB?

I: Ok. Is there a TB history in the family?

P: No, no one has ever had TB before.

I: Oh ok. What was most helpful about the box?

P: The box helped me to be disciplined especially when it come taking medication on time every day; it taught me about the importance of time when it comes to taking medication because a lot of times, we take this for granted and yet it very crucial.

I: Oh ok. Since you mentioned that you were receiving SMS reminders, I would like to find out if you also received a phone call reminding to take your medication.

P: No, I never received a phone call from the Clinic.

I: What about a home visit?
P: No, no one from the clinic has ever visited my home.

I: Oh, no one from the clinic has ever came your house?

P: No, no one has ever came ever since I started taking TB medication.

I: Oh, you mentioned getting piece jobs sometimes and taking the box with you.

P: Yes.

I: I would like to find out if the box affected your work?

P: Uh I do not want to lie, it was difficult on the first day but on the second day you get used to it and eventually become comfortable with taking your baby to work, and even friends begin to understand your situation and be the ones who reminds you all the time to take medication. I also did not care much about what people might say because I was so focused on getting better.

I: Mmm ok. Since you are familiar with the box what can you tell the next person about it and how it supposed to work?

P: Uh I would tell them to give themselves time and expect nothing, but good things though there might be some challenges but still I would tell them that its important that they give themselves time with the box and look after it because that is the only way they can see good things about the box.

I: Mmm

P: If they cannot hold onto it; they will not have something to remind them, and it will only mean a setback for them.

I: Mmm

P: So, this is how I am trying to outline the positive and benefits of the box and how am I impressed by it-

I: -Mmm-

P: - It makes my life easier.

I: Ok. Is there anything you would like to be changed from the box maybe certain features or anything that you would like us to modify?

P: Uh the box is fine; I like the way it designed; its material is of excellent quality but maybe if they can change the beeping sound and make it have different beeps for different reminders. As human beings, we are different; some people like to wake up early and do something outdoors. So, maybe if they can have a reminder that will alert you before the time and the one that will remind you after you missed medication, and both have different sounds. If they can have a feature that reminds people on their phones that they soon have to take medication or they just missed because you have someone who prefers taking their medication at 07:00 AM, so maybe have a reminder that will alert them just before 07:00; at 06:40.

I: Mmm ok. And everything else is fine the way it is?

P: Yes, everything else is fine because the most important thing is time.

I: Time?

P: Yes, because we were told not to have breakfast before taking medication and sometimes you find that one wakes up early and take off because one is trying to avoid having breakfast before taking medication and because of that one end up missing time for medication. So, if they can have like a reminder before time and in that way, it would be easy for one to immediately return home before missing the time.

I: Mmm

P: I think that could make the box even better.

I: Oh, thank for your useful information, we thank you. I hear you mentioned that if there could be a way for people to be reminded on their cellphone. What I would like to know is what about those who do not have cellphone? How do you think they can also be reminded?

P: Uh I do not have an idea and it could be a problem to them unless there is someone, they can share a phone with but still when the owner of the phone is not around, then there will not be a reminder for them. If, they can design something like a wrist belt that people can wear which has a reminder for those who do not have phones.

I: Mmm ok. Hey, I like your idea [laugh]; it might be a great idea. You also mentioned something about changing the beeping sound and alarm, right?

P: Yes, maybe if they can change that too because they all sound the same; make it sound different when opening it and when alarming.

I: Mmm

P: Yes, because when you open it, it reports to the clinic that you have just opened it. So, if they can add different sounds, making it sound different.

I: Oh, I hear you saying the box report to the clinic when opening it. Could you explain a bit what do you mean by that?

P: We get an SMS when we do not open the box which means someone at the clinic can see that the box has not been opened and they know when you are not taking medication at all because they give you a call or a visit. So, it reports to them when you open it and reports again if you did not open it and they know that you did not take your medication.

I: Mmm

P: Yes, it means they see everything on their side.

I: Mmm you are saying is that they can see when it opened, and they can also see when it not opened and-

P: - They know that you did not take medication.

I: Uh did they show you how they are able to see everything-how they see if the box has been opened or not?

P: Uh there is no way you can ask them something like that, I found out by the time I decided not open it for two days straight and that is when I received an SMS saying I have missed medication.

I: Mmm

P: Mmm

I: Oh, you are saying you did not open it on purpose[laugh]?

P: Yes, [laugh].

I: Oh, you did not open it for two days?

P: Mmm I did not open it and did not take my medication for two days because I wanted to see what is going to happen to the box and that is when I saw that they send you an SMS when you did not open the box.

I: How did feel about missing your medication for two days straight?

P: Uh I regret doing that; not taking my medication for two days just to see if the box will report to the clinic that I did not take my medication and it turned out that they actually see every action and I regret ever doing that. I risked everything especially my health because at the end of the day we want to get better.

I: Mmm then you received an SMS after two days?

P: Yes, I received an SMS.

I: At what time did you receive this SMS?

P: I got it around 12:00 PM

I: Oh, did you also receive a phone call?

P: No, I did not get a phone call.

I: You did not?

P: No.

I: Ok. Mmm so, you say you received an SMS reminder after you missed medication for two days-after you risked your life[laugh], is that what you said?

P: [laugh] Yes.

I: How did you feel about getting an SMS reminder saying you have missed your medication?

P: To tell you the truth, I laughed at myself immediately I saw the SMS and I told myself that I must drink my pills before someone from the clinic comes looking for me.

I: Oh ok. You never had home visit before?

P: No.

I: Ok. I would like to know if you received counselling as part of your treatment journey.

P: Mmm what can I say, luckily my mother is nurse and when I am visiting home, she would offer me counselling about TB and since I was a smoker, she would also encourage me to quit smoking until I am cured from TB because it not wise to smoke weed while taking TB medication. So, my mother is one person who would give me an advice and counselling.

I: Mmm so now from what your mother said to you what exactly did you find most encouraging? How did you feel about what she had to say to you?

P: Nothing she said was bad, but it showed how she cares about me, and how she wants me to get better; everything she said was true. She emphasized more on quitting smoking and avoiding things such as paint because they make it worse.

I: How did you feel when she said you must quit smoking?

P: `Even though it was something I am used to, but I told myself that I must quit because she was telling the truth though I still smoke a bit but not everyday. I tell myself everyday that I must stay away from things like that because sometimes they make life difficult.

I: Oh, so it not an everyday thing but you do it there and there?

P: Yes, once.

I: Oh, what exactly do you mean once? Is it once a day or once a month?

P: [laugh] Uh it tough to quit smoking once you started; people who have TB find it difficult to quit smoking because it already a habit and sometimes after a meal you find yourself craving for it. I usually smoke once in the afternoon or eat something like sweets just to get cravings away.

I: Ok. And how do you feel after smoking?

P: Uh it hurts us because sometimes you have a short of breath like you are suffocating or something.

I: Mmm you mentioned something about getting a phone call-an SMS reminder telling you that you forgot to take medication and a box reminding you?

P: Yes.

I: Mmm is there any of these things that you feel are just a waste of time?

P: No, they are all important.

I: Ok. Thank you for that.

P: Yes.

I: Oh, so you said the box must at least have different sounds for distinct functions

P: Yes.

I: Ok. Is there anything you want add something about which we did not talk about?

P: No, the box is perfectly fine for me but if there can be a way to accommodate those who do not have cell phones so that there is a way to remind them; something like a wrist belt that will remind them.

I: How exactly do you think this wrist belt can be like? Can you please elaborate for me?

P: Something like a watch but with a beeping sound just like the box; reminding one shortly before the time.

I: Mmm ok. [Door cranking]is there anything else that you would like to share?

P: No, that all. Some people do not have cell phones, and some lose them.

I: Ok brother I hear that you never received a phone call from the clinic as result of missing your daily doses nor home visit but what I would like to know is how would you have felt if you had been part of a follow-up phone call and a home visit?

P: Uh I would not have a problem because it shows that someone cares, on top of the SMS reminder, they check on us; it is a wonderful thing.

I: You would not mind a home visit?

P: No, not at all.

I: Ok. Thank you so much brother for your time, I think we have come to an end of our interview. If you have any queries about our study or the interview you can contact the people, I showed on the form. We have come to an end of our interview and the time is 13:07 PM.
