# Supplementary material for: Acceptability of Digital Adherence Technologies to support people with drug-susceptible TB in South Africa
Source: PLoS One. 2025 Sep 24;20(9):e0332103. doi: 10.1371/journal.pone.0332103 (PMC12459780; doi:10.1371/journal.pone.0332103)
Supplement: S4 File — (ZIP) [file pone.0332103.s004.zip › S4 Transcripts/PwTB/IDI 21_PwTB.docx]

**TRANSCRIPTION NOTATIONS**

| **Label Key** | **Meaning** |
| --- | --- |
| **I** | Start of each new utterance by the Interviewer |
| **P** | Start of each new utterance by the Participant |
| **N** | Note taker |
| **{ }** | Indicates that details were changed or pseudonyms were used to anonymise data |
| **( )** | Indicates the description provided to anonymise data |
| **XXX** | Words were omitted to anonymise data |
| **-** | Breaking into a sentence by the next speaker |
| **…** | Pause or drawn out words |
| **[ ]** | Indicates noise made, e.g. [laugh], [sigh], [pause] |
| ? | Beginning of utterance by unidentified speaker or questionable text |
| **[inaudible segment]** | Unclear section of the recording |

I: Thank you for agreeing to take part in the study, uh do you-do you give me permission to record you?

P: Yes, I agree.

I: Alright, date xxxx (interview date), location: xxxx (clinic name) language used: Setswana, PID number xxx, start time 12:14. Ehh when were you diagnosed with TB?

P:Uh , last year December.

I: You were told you last year December that you have TB disease?

P: Yes.

I: How far do you stay from the clinic.

P: It is quite a distance.

I: You need transportation?

P: Yes, I take a taxi.

I: How much does it cost you?

P: A taxi?

I: Mmm when you come to the clinic.

P: R17 +R12 how much is it? R29 *neh* (right), yes, R29 x 2.

I: So, when you come to the clinic-how often do you come to the clinic?

P: I come once a month.

I: Once a month?

P: Yes.

I: So, who do you stay with at home?

P: My girlfriend and children.

I: With children?

P:Mmm.

I: So, when you told them you are taking TB medication, how did they feels?

P: They did not believe it.

I: They couldn’t believe?

P: Yes…

I: What symptoms did you have- which symptoms did you have when you ended up coming to the clinic for the first time?

P: I lost weight; I saw that I couldn’t eat anymore and that is when I decided that I should come to the clinic.

I: So, this box, they gave you here at the clinic?

P: Yes, they gave it to me here at the clinic.

I: So, what can you tell me about the box?

P: About the box?

I:Mmm.

P: The box is helping to take medication because most of the time when you wake up, you forget that you should take medication, you see, because you can wake up and leave without taking your dose. So, when you are in the house and you have the box, it will ring when it’s time to take medication, then you eat and take medication.

I: Who explained to you about the box?

P: It was xxx (intern’s name), I forgot her surname.

I: She is the one who explained the box to you?

P: Yes.

I: How long did that take? How long did it take when they were explaining the box to you?

P: I don’t remember well, but close to an hour.

I: So, how did you feel about the information they gave you?

P:Uh, it was just fine.

I: Was it understandable? Or you felt like they could improve something so that people can understand easily?

P: *Hai (*no), it was simple the way it was explained to me; it is simple, you know, and even the information was just fine.

I: Before they gave you the box here at the clinic, have you ever see it somewhere before?

P: Uh , uh , I started seeing it here.

I: Here at the clinic?

P: Mmm.

I: So, they gave you- have you ever taken your TB medication without this box?

P: Yes, I started treatment without the box.

I: You took your medication without the box?

P: Yes.

I: So, you got it after?

P: After one month.

I: Okay, what can you say about your experience before you got the box?

P: Ehh, like you won’t take your medication the way- like when you have the box it alerts you that it is time take your pills, unlike when you have just placed them in the house and you have to know that it is 09:00 and you have to take your pills ,you see, sometimes you forget, but when you have an alarm, you know *gore* (that) it is time to take your pills.

I: Okay, so what were you using before you had the box for that first month to remember it is time to take medication?

P:Uh , I was using my phone in the morning for time, when I finished eating, I would take pills, yes.

I: You were using your phone then, have you ever missed medication?

P: The time I was using my phone?

I:Mmm.

P: Yes, a lot of times, because sometimes you can wake up late, during the day, but when you have the box and you have given them time to set it. It is better because you know that at this time you must eat and take your medication.

I: So, when you missed, like if you have missed like that, how do you catch up? When you have missed your medication.

P: like when I have missed?

I:Mmm.

P: like tomorrow only, I will drink tomorrow or later.

I: You will take them tomorrow or late?

P: Mmm.

I: It depends on what?

P: With a system of eating; it depends if I have eaten because you can’t take medication without eating.

I: Oh okay, so since you started using the box, how was your experience?

P: It is so much better , even if you haven’t eaten, you know you have to take out pills, you see.

I: So, what do you like the most about this box, that help you to take your medication easily?

P:Mmm, it is the alarm and the storage.

I: Okay, can you please explain about this alarm?

P: Alarm gives you like straight time that you must take medication, at 06:00 you know you must take out pills and put them in the pocket before you leave the yard. So, when you get to work and have eaten, after eating you take them out of the pocket ,you understand. Mmm.

I: You spoke about storage?

P: Storage, yes, it keeps pills safe, like even the kids know that they should not play around them and they cannot reach them easily, you understand.

I: so, what time do you take your medication?

P:Mmm.

I: You take your medication at what time?

P: I take them around 08:00, but the alarm is set for 06:00 because I leave the yard around 06:00, so I will eat at 08:00 at work, you see, mmm.

I: So, when you have took them with you at 06:00 when you leave and you would drink at 08:00, have you ever forgotten them at work?

P: No, I always have them, mmm I never forget them, *nex* (not really).

I: So, have you ever had any problem since you started having this box? Be it network issue?

P: Yes, the problem I faced was just one, like after taking medication, it sends a message on my phone that I did not take medication and they will contact me or what, what, mmm.

I:Mmm, how would you feel when that happens?

P: *Yoh*! (wow) I just ignore it because I know that I have taken medication.

I: So, taking TB medication using the box and you working don’t have any disturbance?

P: Uh ,uh , it is always at home.

I: You never took it at work?

P: Uh it is always at home because if I need to take my pills, I know I will take them out, put in the plastic, or packet and will drink at work.

I: So, when you are going to family gatherings, you leave it at home?

P: Yes, because most of the time I use a plastic, sachet, or these zippers.

I: So, have you ever gone for more than a day, without access to the box maybe you took your medication outside the box?

P: Take it out but do not drink?

I: Uh ,uh , just taking out.

P: I took it outside the box?

I: Yes, have you ever taken medication out of the box, then go somewhere for more than a day and the following day you don’t have the box around, like you travelled?

P: Uh , uh I have never travelled.

I: Okay, is there anyone who ever saw you using the box except the family?

P: Yes, the is this guy who saw me having this box, he is a TB patient too.

I: What did he say when he saw you having the box?

P: He asked me where I got it from, what do I use it for and I explained to him that it is for TB. He then told me he also has it, and he is using it for TB treatment.

I: He said he has what?

P: He has TB and the box too.

I: He has the box too?

P:Mmm.

I: Okay, beside that person, is there anyone who ever saw it?

P: Umm , Uh , it is only from the family

I: How did they react at home to the box? Because it has this sound thing.

P: [laugh] obviously it irritates them, you know in the morning when they are still sleeping and then it rings.

I: It was ringing.

P:Mmm.

I: How do they feel about the ringing?

P: Box is okay, because they also open it to check if the pills are moving. If they are not the same.

I: They open it?

P: Yes, when they check if am taking my pills correctly because most of the time, you will find that on weekends, you see, I would be home since it is not time for work am still asleep. I will just open the box and close so that the alarm can go off ,then sleep and will take medication later.

I:Mmm,

P: So, sometimes they check that am taking medication or what, like on weekends, because you will find that am opening and closing only that the alarm can go off then sleep and will take medication later, you see.

I: So, how often does it happen this thing of opening and closing only?

P: Yes, it is possible, you heard I said on weekends so, because I would be sleepy then it rings, I wake up and switch it off then close and sleep again. Then I will open again around 8-9 when I wake up.

I: So, at the point when you just close it, what reminds you that you closed the box without taking medication?

P: It is people at home, they will say “you closed your box. Did you take medication or what?”

I: Then you would drink at that time?

P: Yes.

I: Okay, so have you ever opened it accidentally, you opened without taking medication inside?

P: Yes, like that is why am saying I once placed it around kids and they opened it.

I: Where did you put it?

P: like you hear me when I say, I just placed it carelessly and the kids opened it, they managed to reach it and opened it. So, that is why I told you I put it far now.

I: Okay, where do you put it now?

P: I put it on top of the cardboard.

I: on top of the cardboard?

P:Mmm.

I: Okay, so does it happen that it rings and no one opens it?

P: I don’t know, I leave it at home because the last time I heard them saying it rings when am gone and I have already taken my medication. At home they said it was ringing when I asked them if it rang for long, they said they ended up opening it and close it, then it was fine.

I: So, is there anyone who knows about your TB status beside family?

P: Yes, a lot.

I: How do you feel when you tell people about it?

P: I tell them to go and check because most of the people I could see that these ones have TB, you understand, like I have referred 3 or 4 people to xxxx (clinic name), I tell them to go and check, you see.

I: Alright, you are saying you don’t have a problem about talking about your status to people?

P: Uh-huh ,uh-huh I don’t have problem.

I: So, beside seeing this box, have you told anyone about using this type of a thing?

P: Type of box?

I: Uh-huh , I mean except those who saw it, is there anyone you told that I have this type of a box?

P: Uh-huh.

I: You never talk at all.

P: Uh-huh.

I: Okay, do you have any history of TB in your family?

P: Uh-huh , am the first to experience it .

I: You said at your family, they opened the box when they wanted to check.

P: Yes, like if am taking my medication.

I: Who exactly was the one checking mostly?

P: My girlfriend.

I: She is the one checking?

P: Yes.

I: Okay, the time you said they open it, where did you put it?

P: I misplaced it, mmm.

I: So, they would open and not take anything out?

P: They don’t take anything out.

I: How often does it happen?

P:The box?

I:Mmm.

P: Because right now I put it on top of the cardboard ,you see.

I: So, the girlfriend, how often does she check?

P: Every 3 to 4 days mmm.

I: Every 3 to 4 days, she checks?

P: Because sometimes when she checks, I wouldn’t be there, mmm but there this time she found then in the pocket and she asked me if I drank my medication on that day. What is happening?

I: Was it after work or before?

P: Going where?

I: I mean when she found them in the pocket, was it before going to work or after work?

P: It was after work.

I: Meaning you didn’t take your medication that day?

P: Yes.

I: So, what time did you take them?

P: I took them the following day because the time that I did not take medication I flopped, this other guy came and bought me 6 beers, so I drank those beers and there is no way you will mix beer and pills. I then continued tomorrow.

I: If you had beers on weekends, are you not afraid to drink medication?

P: I start first by eating, then take medication, mmm.

I: Then you would drink later?

P:Mmm, later I drink, but I don’t go over the limit; I limit for myself.

I: Mmm.

P:Mmm, because you can’t mix pills with beers.

I: Okay, at work, what reminds you to drink medication?

P: I always have it in the pocket, just when I finish eating, it’s a must.

I: What reminds you?

P: I know that after eating, I have to start with them.

I: What time do you eat daily?

P:Mmm, around 08:00- 09:00, I know the latest is 9 o’clock I should have eaten.

I: Okay, have you ever received a phone call from the clinic?

P: Regarding?

I: Medication, telling you something about medication?

P: About how is medication affecting me?

I: Yes, any phone call?

P: Or you are talking about box?

I: Any phone call that you got from the clinic regarding medication or box.

P: Yes, I have received a phone call about medication, like they were asking how the medication is treating me since I have started with TB pills, if I have any allergy and stuff like that.

I: How do you feel about clinic people calling you?

P: like I do answer, mmm because I don’t know what they want.

I: But how do you feel about phone calls? Do you think it is a good thing?

P: Yes, it is good and it helps a lot.

I: So, about SMS? You mentioned that you have received the SMSs?

P: Yes, I have received SMSs about medication.

I: Saying you have missed medication?

P: Yes

I: How do you feel about those SMS?

P: I just ignore it because know I took my medication.

I: Okay, if you miss more than a day according to the study, they come to you, to come and check you at home; to check why are you not taking your medication, how would you feel about been visited at home because you didn’t take your medication?

P: It would be still alright, it helps that people come to check up on you, it helps ,you understand me.

I: So out of all these things I say we are doing based on the study, uh they send you an SMS that you have missed medication-

P:-Mmm.

I: They call you after 2 days and then visit you after maybe a week, what would you say helps people more? Which one do you see as the one that helps people who can’t take medication properly?

P: Eish I don’t know, because for me I see the box been better.

I: Even though people have the box, we still have people who struggle to adhere, so on these 3 things that we do ,which one would you say would help people more?

P: It visiting them at home; to check them if they are taking medication or what.

I: alright, so any challenges you had since you started using this box?

P: I don’t have any challenges; I am okay.

I: So, what do you find more helpful about this box

P: That I do what?

I: What is that you find more helpful about this box?

P: Ehh bra, it is taking medication; medication is important too much.

I: What is a thing that helps people more?

P: That they did on the box?

I:Mmm.

P: I would say to you it is the alarm, this alarm helps a lot.

I: What about the information you get at TB room before you started with medication?

P: Uh like I haven’t been to TB room a lot, but I have received the information, but was a lot time I started treatment.

I: What do you think about teaching people about TB medication?

P: It would be helpful and a lot you know.

I:Mmm.

P: Mmm.

I: Please elaborate a little on what do you mean when you say it would be helpful a lot.

P: I would be helpful a lot because when I had TB too, I thought it was flu, ehh I looked for help. It could really help because most people do not take it serious and they do not go to clinic. So, people go to the doctor, get medication and then go home; they never check their status. People do not check.

I: So, what method can we use, you see, which way can we use to make people accept these boxes?

P: By getting them in class and give them the research about it, like if you can introduce it too much on social media, maybe

I: We introduce what?

P: To introduce the box like if most people know it.

I:Mmm.

P:Mmm.

I: On social media?

P: Yes, like if most people can know what this box is for, and which medication is this box for and more.

I: So, let’s say uh am newly diagnosed with TB and they say teach about this box, what can you say to me?

P: [laugh] *hai* (no) this box-what can I say, but my brother I would talk about the alarm ,you see box is like a storage that helps to keep medication safe, you get me? You take them away from the kids and that will help you to take medication at the correct time, that what I would tell you.

I: So, you mentioned storage, and the alarm.

P: Yes.

I: Have they ever taught you anything about other functions of the box? Such as lights?

P: Yes, I was taught about it.

I: What do you think about those lights?

P: Those lights are fine, and they work exactly how I was taught.

I: So, this box the way it was explained to you, would you say it is easy for you to understand how it works?

P: Yes, it is easy because those lights are like robot lights, if you understand robot, you will understand what is happening.

I: Please explain little bit further around those robot lights?

P:[laugh] you know robot when it switches on, on the green it is where you can see it is time to open, you have to take out medication , then there is somewhere when the battery or if it is having an issue then it is on and shows red, you understand. Then it is on the orange then maybe it is when it has an error and you have to return it.

I: Okay, alright, so how satisfied are you with this box? How satisfied are you with this box?

P: Uh I would say 95%.

I: Can I ask you to explain- uh can you elaborate a little about your satisfaction if we divide it into categories.

P: It has helped me a lot, since I started this TB journey, it did not give me any problem, the only thing was this thing of reporting SMS and ringing the alarm when am not around and that time I know I took my medication, and my time has passed, and I went to work. Then it would ring after what and when am not there, yes.

I: Okay, (…) can we talk about missing doses, how can we improve to help so that you don’t miss any dose?

P: I think this way of SMSs, it is alright.

I: And then uh you said it would keep your medications safe?

P: It has helped me a lot because box you know it stays far from the kids there is no mistake that can happen unlike pills packages that you know kids can easily open it, but this box is good a lot.

I: is there anyhow would you say this box helped you with clinic appointments? Did it have any effects of you coming to clinic?

P: Yes, because you know when you are running out, you know the date of coming to the clinic is near.

I: You said it helps you with what?

P: When the pill is running out, you know the date to come to the clinic is near.

I: Okay, what can we do on the box that you feel like we can improve, what is something you see that we can improve on the box that it can help people more effective?

P: Uh I see nothing; it just fine, only if they increase the space.

I: What did you say?

P: I see it fine as it is.

I: What about size?

P: Yeah that is why I say, if they can increase the size a little.

I: If they could increase the box a little?

P:Mmm.

I: Okay.

P: Because at some point, they do not all fit inside.

I: And how do you keep these other pills that did not fit in the box safe?

P: I put them on top of the cardboard.

I: Okay, what about the alarm, do you feel like it effective?

P: Yes, it is fine.

I: Alarm -you feel like-

P: -It is fine.

I: Uh would you say the language we are using to communicate with the patients is okay?

P: Uh [laugh] I don’t know because you know we are different; we are not the same.

I: What about the material?

P: Material is fine.

I: I know you said you never got an SMS, phone call -I mean regarding missing medication, but do you think it is fine that they should call people to tell them that they should take medication?

P: Yes, it is good, a lot.

I: Okay, on this question it is something I asked you already, but it is now broader, everything we do, including the alarm, phone calls, what do you think has been more important that we must priorities or that can work for you more?

P: That does what?

I: What can work for you more.

P: More?

I: That can help you more, SMS, phone call-

P: -I am fine you know, am fine with the box.

I: let’s say for someone who is not adhering well, how can we help them?

P: You can help them by visiting and check up on them that they are well and if they are taking medication.

I: Okay, is there anything you see we can improve?

P: No, it is only the size.

I: So, who must teach people about TB?

P: Who can do what?

I: Who do you think should be the one to teach people about TB?

P: Who was refusing?

I: Who do you think is suitable for teaching people about TB?

P: Here at the clinic, if they can make research, and tell people that today we have TB what, what come to the clinic and learn about TB.

I: And who do you see fitting to lead this thing?

P: It is me person; the person who knows about TB, I can be the one to teach people that they should go and check their status.

I: Uh is there anything you feel like you would to talk about regarding this box that we didn’t talk about?

P: *Hai* (no) everything is fine.

I: So, anything you would like to talk about regarding phone call, SMSs, and home visits?

P: *Hai* (no) I am fine you know.

I: Okay, I think we come came to end of our session, thank you very much for participating, thank you very much, ending time 12:44.
