# Supplementary material for: Acceptability of Digital Adherence Technologies to support people with drug-susceptible TB in South Africa
Source: PLoS One. 2025 Sep 24;20(9):e0332103. doi: 10.1371/journal.pone.0332103 (PMC12459780; doi:10.1371/journal.pone.0332103)
Supplement: S4 File — (ZIP) [file pone.0332103.s004.zip › S4 Transcripts/PwTB/IDI 22_PwTB.docx]

**TRANSCRIPTION NOTATIONS**

| **Label Key** | **Meaning** |
| --- | --- |
| **I** | Start of each new utterance by the Interviewer |
| **P** | Start of each new utterance by the Participant |
| **N** | Note taker |
| **{ }** | Indicates that details were changed or pseudonyms were used to anonymise data |
| **( )** | Indicates the description provided to anonymise data |
| **XXX** | Words were omitted to anonymise data |
| **-** | Breaking into a sentence by the next speaker |
| **…** | Pause or drawn out words |
| **[ ]** | Indicates noise made, e.g. [laugh], [sigh], [pause] |
| ? | Beginning of utterance by unidentified speaker or questionable text |
| **[inaudible segment]** | Unclear section of the recording |

I: We like to thank you for agreeing to do this interview with us. May we now request permission to record you?

P: Yes, there is no problem.

I: Okay, date xxxxx (interview date), location xxx (clinic’s name), language used Setswana, PID xxx, start time 12:16, okay, when did they diagnose you with TB?

P: It was (…).

I: If you could try to remember, how long ago can that be?

P: 3 weeks-3 months-3 months back.

I: 3 months back?

P: Mmm.

I: Ever since you have been diagnosed with TB, how has it affected your life?

P: Uh, I have been good since I started taking pills and now am way better. The pills did not affect me bad and are treating me well.

I: When you say you way better, how bad were you?

P: I was weak-I was weak at the beginning, and I was not even able- I couldn’t walk, but now I can walk and that is why am saying this treatment is treating me well. I believe it to be the one that is helping not to get troubled too much.

I: Mmm.

P: Mmm.

I: Okay, do you use a taxi when you come to the clinic?

P: When I come to the clinic?

I: Mmm.

P: No, *Hai* (no).

I: Who do you stay with at home?

P: I live with my mom, father, sister and 3 siblings.

I: What symptoms did you have for you to come here at the clinic and diagnosed with TB?

P: I couldn’t eat, and I was vomiting, I couldn’t walk, I had no appetite, and I didn’t crave for anything.

I: Please tell me about the box, what do you know about this box?

P: Box- what I know about it like is that I know time, it can give me time to take medication so that I cannot be late. I like that it gives me time so well and that I don’t lose time, I know that at this time I must take medication.

I: Okay, you said it gives you time, how does it give you time?

P: I know when the alarm rings that I must take pills, unlike waiting for 09:00, then I forget and when I check time you find that it is already 10 now. So, when the alarm rings, it gives me time to take medication.

I: Mmm, so who explained to you about the box?

P: XXX (intern’s name) explained it to me.

I: How long would you say the explanation took?

P: More than 15 minutes.

I: And information that they explained to you, did you understand how the box works?

P: I understood very well.

I: So, what helped you to be able to use the box?

P: I was helped by the way it was explained, it is a simple thing you can understand it. When she told me that this box-these pills inside, when it rings you must take them, that is what helped me because I knew that when it rings, I take medication. If it hasn’t ringed yet, I do not take medication.

I: Is there you feel like you would like to change, or that you think they should tell people regarding the box before it is given to people? Or that you saw later?

P: I didn’t hear you well.

I: Did you see anything after using the box that you feel the clinic should have informed you about?

P: I know it shows green and red light, but I was surprised when it showed orange and that is what confused me.

I: So, when they explained the lights to you, what did they the green one is for?

P: Yes, the green shows that I must take medication and the red one- you find that I didn’t take medication and it rang 3 times, so if it rings 3 times.

I: It shows red light after ringing 3 times?

P: Yes, and I don’t understand what it means when it shows the red light.

I: So, you did not understand after it showed you the lights. Did you come to the clinic to ask about them?

P: No, I have never asked about them because the red light never show after some time. It shows green and I understood that it was because I missed medication.

I: So, you are saying it shows the red light if it rings 3 times. What would be the cause of it to ring 3 times?

P: Sometimes you find that I am not around, and it would ring alone three times. I can go out maybe- I have to be honest, I am a person who is using drugs, you see. So, you find that maybe I went to scrap side or someone gave me a piece job, but *mara* (but) when I come back and I missed time that is why I ended up changing time, so that they can give me time that I know I will be around. I was taking them 09:30 , but now at 07:00.

I: So, when you are out and missed medication, do you drink when you come back?

P: Yes.

I: Have you gone a day without taking treatment at all?

P: Yes, I have missed some days. The reason I missed those days is because I was not home. I have a problem that I fight with my father maybe we were fighting because I took old stuff that are not used to sell so I could buy drugs. When we were fighting I would leave with medication for a day only and would come back after two or more days so I missed those days.

I: So, do you take medication with when you leave like that?

P: Yes, I do take them sometimes.

I: And where do you keep them safe?

P: No, I just take the pack straight, they are packed in 3s ,so I just cut this 3 and take it and leave with it.

I: Then where do you keep those 3 you cut?

P: The one I cut?

I: Mmm.

P: I put them inside a plastic, then put inside the bag.

I: Okay, how often does it happen this thing that you are not around, and you don’t take medication?

P: Uh, since-maybe it happened twice, but now I have moved out, I no longer stay with them; I stay with my brother somewhere down there.

I: Have you seen this box anywhere else before you were given

P: I have never seen it.

I: Is there anyone who ever told you about it before?

P: Uh-huh(no).

I: So, what can you say about your experience using this box?

P: What?

I: Your experience, what did you realise about using this box?

P: I realised that I must take it seriously-what did it show me? It showed me the importance of time; time is the main thing.

I: Which part of the box makes it easier to use?

P: Which part does what?

I: That makes this box easy to use.

P: Eish I don’t have an answer, I don’t understand what you mean really.

I: Would you say it is easy or difficult to use this box?

P: It is easy.

I: What makes it easy to use?

P: It keeps me on track, so that I get well, unlike when I was taking pills on my own and missing time. I like that it makes sure I do not miss time.

I: What do you love most about the box?

P: About the box?

I: Mmm.

P: Uh, I love pills.

I: So, did you come across any challenges while using the box?

P: Uh, uh.

I:Has the box gone a day without ringing?

P:Uh-huh (no).

I: Ok, do you have a phone?

P: Uh-huh I don’t have a phone.

I: So, who do they send an SMS to if you missed medication?

P: To my sister.

I: So, how do your sister react to these SMSs?

P: *Hai* (no), eish she gets sad and ask why am I not taking pills, so that why I must adhere because she feels hurt.

I: How do you feel when she tells you that?

P: Sometimes you find that I have already taken medication, and they also given me medication to put inside the box , but these other pills do not fit inside the box. So, I end up taking medication that is outside the box, and not open it and that was my mistake.

I: So, have you ever taken medication outside the box?

P: Yes.

I: Can I ask, why do you take the one outside the box?

P: I would leave and take the pack outside the box , as I explained earlier on.

I: Okay, so have you ever left with the box?

P: Yes, I take it with now.

I: Where would you be going with it?

P: Sometimes you find that I got a piece job, and I must wake up around 05:00 as I would expected to clock in at 06:00 am. So, I would take the box with me so that I can medication when it ring at 07:00.

I: So, has anyone ever asked you about it when have taken it with you?

P: Yes, they have.

I: What do you say when you explain to them?

P:I explained to them that this box carries my medication and it reminds me time so that I do not forget my medication time. This box reminds me that I must take my pills.

I: Okay, so how do you feel about explaining the box to people?

P: Uh, I feel comfortable, like I feel okay and mostly its people who are drug addicts too. I give them courage go and test because you don’t know, you may find that you are sick because I also couldn’t believe when they told me I have it. I knew I have HIV when I got to the clinic and I thought my symptoms were for HIV, but they said “no they are for TB.” You find me giving them courage, I would encourage them by saying “go and check, don’t just sit and test late.”

I: Okay, how was their reaction when you told them about the box?

P: Reaction of people I explained to?

I: Mmm.

P: Some are encouraged and some don’t know what is going on with me, so they don’t take me seriously, but others are encouraged, and others already came to test.

I: So, has anyone ever complained about the box when it started ringing where you will be working?

P: No, no one has ever complained because I work outside the yard sometimes and others do not hear it because you find that it is in the bag pack and it stays close to where I work. Some other time you find that they are with me and they would ask what is that and what is inside and what are they for, then I would say, “it for TB” and they would say, “you are taking good care of yourself.”

I: Besides family and friends, is there anyone else you talked to about your disease?

P: No.

I: Do you find it easy talking to people about your TB disease?

P:About me?

I: Mmm.

P: Yes.

I: So, is there a TB history in the family?

P: No.

I: Have you ever opened the box without taking out medication?

P: To open.

I: To open without taking medication for another reason maybe.

P: Yes.

I: What would be a reason?

P: You find that sometimes I open to show someone when they are asking what is in there. I say,” there are pills” and someone would ask that how are they TB pills. You find someone showing an interest wanting to know how they are and I would open and show them how they are.

I: Yes, how often does it happen that you open without taking medication?

P: Uh, it been a while now since it happened because as I was saying that its only when people have asked to see the box. Only people am with can see me when taking medication and they would hear it when it rings.

I: So, , what else to you put inside this box beside medication?

P: Sometimes you find that I put some weed or cigarette when I don’t want them to be damaged.

I: When they gave you the box, did they tell you what can you put and what you cannot put inside it?

P: Uh, they did not explain what kind of things I mustn’t put on that day, they did not explain it.

I: Did they explain to you how it works between you and the clinic?

P: Uh-huh (no).

I: What helped you a lot with your treatment when it comes to this box?

P: That helped me a lot?

I: Yes, about the box helping you take treatment.

P: To tell the truth, it helped me a lot with time and this alarm helped me a lot when it rings.

I: Have you ever received counselling from the clinic?

P: Any counselling?

I: Mmm.

P: *Hai* (no).

I: What difficulties did you face since you started using this box?

P: Uh-huh.

I: So, do you find it easy travelling with it?

P: Yeah, I feel comfortable when I go with it, unlike when I just leave it like that.

I: Tell me about the SMS that you received.

P: SMS I received.

I: About missing medication?

P: *Hai* (no), I have never received an SMS, I only get a message maybe when they want to come-what do they call them[ referring to community health workers], they come to check on me if it happened that I missed treatment. So, they come to tell me that am wanted at the clinic. Messages I get a lot are the ones from xxx (intern’s name) when she is reminding me to take my pills. So, that is why I ended up changing the time to take medication, as I explained to them that these pills require me to eat first and staff like that. Therefore miss medication mostly because of food because you find maybe *mamazala* (mother-in-law) is still cooking porridge ,then it rings and would only take medication after an hour after I have eaten.

I: So, what do you do the time you are still waiting for porridge?

P: Mostly, I would just ignore or sometimes I open it, take out medication and keep it on myself.

I: So, you mentioned that you are using your sister’s phone.

P: Yes.

I: So, on your sister’s phone, have they ever sent an SMS saying you have missed medication?

P: Yes, they do send.

I: When does she tell you after getting that SMS?

P: Maybe after an hour, 2 hours, or 3 hours.

I: So, is your sister the one who tells you that you have missed medication?

P: Yes.

I: How would you feel when they have sent her that SMS?

P: I would explain to her that I didn’t miss medication. The issue is that I was late, I missed the time and I would have already taken it by the time they are send the SMS. Also, it means the alarm rang for too long and it must not ring 3 times because if it did, then it like that they feel you have missed medication. So, what am saying is that sometimes I miss time because I couldn’t take medication without eating first, you see how it is, yes.

I: So, have your sister ever received a call regarding your missed doses?

P: Yes, they do call her.

I: Then how would you feel that they have called her for that?

P: Eish, I feel awkward, I feel pain too because here at the clinic they think I didn’t take medication when I have, just that maybe I took it late.

I: So, here at the clinic, have they aver spoken to you over the phone call?

P: Yes.

I: What did they say?

P: They were telling me I should take medication and I told them that I have a problem that I have to eat first and they ended up giving me porridges, saying, “here are the porridges when you wake up before the alarm, you can eat so that you can take your pills” because they did not understand that sometimes I was struggling because of food. They explained to me that I can take pills even before I eat.

I: So, you are saying they have sent people to your place to come and check up on you?

P: Yes.

I: What did they say to you?

P: It is sort of like they were shouting at me saying, “these things you are doing, what are they, you have to take your medication, you see, xxx (patient’s name), you will make people get sick at home, just because you share a cup or one plate, and you are missing medication.” And that is what made me realized that sometimes you have to consider other people’s life’s, not only mine. You will find that I put people in danger.

I: You mentioned that you have moved from your home to stay with your brother?

P: Yes.

I: So, have they ever came to where you are staying now?

P: Where I stay? No, they have never.

I: So, now because you no longer staying with your sister, how do you get the communication, how do they reach you?

P: Ehh, I do not stay with them, but I do go there every day because if there is no food where am staying, I go there later to eat because I have a plate of food there and that is when I get the messages and other things.

I: So, what can you say about the method that is being used by the clinic to follow up on patient, sending the SMSs, calling patients and visiting them at home?

P: *Yona e sharp ga bad, ga e sharp* (it actually not bad, it a good initiative).

P: It is good, it is not bad, though it may not be a good thing.

I: it is not a good thing?

P: As for me I don’t see it as a good thing, they see it as a good thing because they want patient to be okay, you see, I see it as just another way of creating jobs for them or something because I know what I have to do, that is why I say for me, is it not okay, because it is something I have to do. It is something I know like now I know I have to go there and am aware that this is my life, you see.

I: So, would you say it is a good thing and they should carry on checking up on patients?

P: Uh, for me, it is not good.

I: I mean like here at the clinic, should they carry on checking up on patient; calling them, visit them at home or to send them SMSs when they have missed their doses?

P: It is a good thing, it is something that is safe for people, the clinic is doing good.

I: So, what can you say about home visit?

P: For me?

I: Yes, would you say it is the right thing that the clinic go visit people at home?

P: It is the right thing, but they must visit with the right things, not with things to remind me that they must take medication. It is a good thing that they come to check up on you, like how are the pills treating you, “we can see now you are strong, you are not like the way you were before.”

I: Okay, would you say there are barriers or challenges that prevents you from taking medication?

P: Uh-huh.

I: You have mentioned *gore* (that) you use drugs?

P: Yes.

I: They don’t disturb you with taking medication?

P: *Hai* (no), they do not disturb me physically, I don’t believe they do, but they disturb me emotionally, they do disturb me.

I: What do you mean they disturb you emotionally?

P: I mean the symptoms, like you find that they must work stronger, but they don’t work strong as they should, you see, or maybe you find that my immune system is low. I don’t know if you can understand, so I don’t know if the way I mix them is a good thing or not.

I: So, have they ever explained to you about medication and drug interaction here at the clinic?

P: Yes, one of health care [community health workers] explained to me that what am doing it is not good because now “we see you like this, but when we come back, you are different and we don’t even understand what is going with you.”

I: What do they mean when they say they see you like this and when they come back you are like this?

P: Sometimes you see them coming, they can tell me we can see that you are fine now, medication is treating you okay, but problem is these drugs, drugs are the one that maybe let me say make me lose weight, they can come like xxx (HWC name), she is the one who sees me frequently because she stays with me in the same area, she can tell me that today you are dark, “look now you are light, stop abusing drug or mixing them with medication.”

I: How do you feel when they are doing that counselling?

P: Eish to be honest, I feel hurt that someone feels that way about me, why don’t I feel sorry for myself, just that it is not simple to move away from them [referring to drugs], to leave them. There is this medicine called *Methadone,* I told them about it, and they said I can mix it with medication, you see. I am afraid to just leave it completely, just that *methadone* is very strong.

I: Where did you hear about the medicine you are speaking of?

P: I have taken it before.

I: So, how do you look when you took it?

P: Yes, I become better and at that time I didn’t know about this TB, two years back before I found out about TB I was taking this HIV (ARV) only.

I: When you say you are better, what do you mean?

P: There is a change because sometimes you find that I have stomach cramps, though it doesn’t bother much or you find that I struggle to sleep. I can sleep for 1 hour or 2 hours without experiencing intense craving. So, it does make a difference.

I: So, do you think it will be easy for HCW to come where you are staying now?

P: Yes, it will be simple.

I: Not having a phone show that you are not getting full support from the clinic, how do you feel about not getting full support that they provide here at the clinic, because they must call your sister if they want to talk to you, how do you feel about this?

P: Yes, I feel awkward, I feel hurt that an SMS has been sent to someone else and I don’t know about it, you find that they must explain something to me. They do not explain it well to her, they know if it was me they were going to give me the whole information, sometimes you find xxx (intern’s name ) calling saying, “come for check-up, They don’t disclose everything to her, they just give information only, “please tell that person to take his medication” even when I didn’t not take medication I can get an SMS saying “xxx (patients name) you didn’t take you medication, we can see that you are taking medication, how are you feeling.” You see, those must come to me straight, but now I get them from my sister, and when my sister explains , she just tell me that I should come here.

I: So, how does your sister feel about receiving calls and SMS from the clinic?

P: Uh she tries sometimes she can lend me her phone; she would say, “call xxx (intern’s name) talk to her because she can’t tell me some of the things and I must not hear them.” She can lend me her phone for 2- 3 days just that she believes if they can get me a phone, I will sell it.

I: How satisfied are you with the box?

P: I am very satisfied, am satisfied since I started using the box, I can see that am getting well.

I: Beside alarm, is there anything else this box helps with?

P: For safety of my pills, so that they are not just scattered around, I know where I will find them, I know where I have placed the box, not that when I have placed them somewhere, and when they start cleaning, they move them and they are placed somewhere, and I can’t find them.

I: So, is there anyhow the box has helped you to reduce number of days to come to the clinic?

P: Yes.

I: How?

P: It has helped me to reduce the days of coming to the clinic by giving me more pills, so when they are in there, they are safe.

I: So, is there anything that we can do on the box to help people and make the box easier to use?

P: That you can do?

I: Is there anything you would like to change to make this box easy to use?

P: Yes, there is, I just don’t know how to explain it, maybe if they can put a camera in inside and they watch to make sure that I have opened because as I was saying, I can open and take medication, but they have already called, that I didn’t take medication and when I drink them after time, they do not believe I that I took medication. They want something that will make them to be sure, they can see that this guy did take out medication.

I: So, who must see that camera?

P: It is the person who is representing me, the person who is taking care of me, the one who is looking at how my treatment is going.

I: Okay, what about the volume of the box?

P:Volume?
I: Mmm, for alarm.

P: Volume is fine, even when you are sleeping, it wake you up, I saw that with me, I have changed time to 07:00, it can wake me up straight.

I: What about the size?

P: Uh it is also fine.

I: Okay, other than putting a camera, is there anything you would like to change?

P: To change or add?

I: To change, to add or remove.

P: Uh-huh.

I: When you were receiving this SMS-

P: -Yes.

I: How did you feel about them? What do you like about SMSs and what you don’t like about SMSs?

P: I like their encouragement, that they are taking care of you, they want to make sure that you are okay.

I: So, what is it that you don’t like about SMS?

P: What I do not like is the way they keep on reminding you like you don’t know what to do.

I: About phone calls, what is that you don’t like about a phone call?

P: There is nothing I don’t like with a phone call, they call to check up on you if you are okay, I love a phone call.

I: So, when they do home visits, what is it that you don’t like?

P: What I don’t like about home visits is when they visit and start judging, they will judge you when they are supposed to help you, they judge you after they judge you only then they are trying to help you, but they have already judged you.

I: What do you like the most about home visits?

P: I love their encouragement only, the way in which they are taking care of you, they are showing you that they are with you.

I: So, on everything that I have mentioned about SMS, phone call and home visit, which one do you think helps people the most?

P: It is to visit.

I: What do you think doesn’t help much?

P: What is unnecessary, is phone call.

I: So, on visits, what do you like the most about it? Since you feel like it the one that helps the most.

P: I love that they know about a person who is taking treatment, they know a person who is on treatment the way he supposed to be, they can see and tell you that now you are okay, you coming all right, you see that a lovely thing, I love it when they visit to give me that encouragement so that you can see that you not taking pill for nothing.

I: So, on everything that we do, which would you say helped you the most?

P: On everything?

I: On these things of visiting, phone calls and SMS?

P: What helped me the most is the visits.

I: Let us be general now, what can we improve, phone call, SMS, what can we improve?

P: What you can improve is to visit a person.

I: Improve it how?

P: Like ehh you can improve maybe let’s say, grocery, some food when they come, they must say brother here is some porridge, I like porridge because I know when I ate it, I can take my pills.

I: So, when they visit and they teach you about TB, who do you think must teach about TB and the box?

P: The health care workers.

I: Are you comfortable talking about the box?

P: Yes.

I: Okay, we are reaching the end of this interview.

P: Yes.

I: For now, I want you to tell me anything that you feel like we didn’t mention that you feel like you would like to speak about regarding the box?

P: Ehh that I would like to talk about regarding the box? It is the red, what does the red light mean?

I: Before I explain about the red light, I want to understand, what do you understand about their function on the box?

P: Ehh I understand- I understand green, when it shows green it means I still have a chance that I can take medication, the orange shows me I still have a chance.

I: Chance to do what?

P: That I can still take medication, the green means I should take medication, orange means I still have a chance to take medication, so red is the one that confuses me.

I: Beside the lights, is there anything you would like to talk about?

P: *Hai* (no) because the most important thing is those lights.

I: So, do you have any comment regarding SMS, phone call and or home visits?

P: Uh-huh (non).

I: Okay, I think we have reached the end of this interview, we thank you for the information you gave us, ending time 12:58.
