# Supplementary material for: Acceptability of Digital Adherence Technologies to support people with drug-susceptible TB in South Africa
Source: PLoS One. 2025 Sep 24;20(9):e0332103. doi: 10.1371/journal.pone.0332103 (PMC12459780; doi:10.1371/journal.pone.0332103)
Supplement: S4 File — (ZIP) [file pone.0332103.s004.zip › S4 Transcripts/PwTB/IDI 23_PwTB.docx]

Translation Setswana

| **Label Key** | **Meaning** |
| --- | --- |
| **I** | Start of each new utterance by the Interviewer |
| **P** | Start of each new utterance by the Participant |
| **N** | Note taker |
| **{ }** | Indicates that details were changed or pseudonyms were used to anonymise data |
| **( )** | Indicates the description provided to anonymise data |
| **XXX** | Words were omitted to anonymise data |
| **-** | Breaking into a sentence by the next speaker |
| **…** | Pause or drawn out words |
| **[ ]** | Indicates noise made, e.g. [laugh], [sigh], [pause] |
| ? | Beginning of utterance by unidentified speaker or questionable text |
| **[inaudible segment]** | Unclear section of the recording |

I: So, do you agree that your interview be recorded?

P: Yes, there is no problem.

I: Mm…mm…okay, thank you. Uhm…. Date…16 May, location xxx [clinic name] Clinic, PID is xxx, time is 10:55AM, language used is Setswana. So, pa, may you please brief me, who do you live with where you stay?

P: Oh! where I live is my RDP (Reconstruction and Development Programme), I’m alone.

I: Oh…okay, briefly explain to me, what do you use when coming to clinic?

P: When I come here, I use – sometimes I walk, sometimes I come with a taxi.

I: So, by the time you come with a taxi, how many do you use to reach the clinic?

P: They are two.

I: Okay, no, thank you. So briefly – explain to me who are you - that who are you?

P: I’m XXX [participant’s name].

I: Okay, no, thank you. Pa, may you please explain to me a bit, what is TB illness, that when we talk about TB illness, what are we talking about?

P: Oh, TB illness is an illness that affects the lungs, it makes you to spit out the cough fluids, it weakens the body.

I: So, when you say, “TB is a lung illness,” what happened when he ended up testing for TB, what did you feel on your body?

P: Oh, I was sweating, I was spitting out the cough fluids, it affected me before and left, it came back again and again.

I: When you say it affected you, on which year, and how many times backwards?

P: Now is for the third time.

I: When you say, you were sweating, you were sweating…

P: I was sweating as if I had been streamed with water.

I: How long did it takes you sweating?

P: It’s a week.

I: So, besides sweating and coughing, what else indicates that this person might have a TB illness.

P: Uhm, at the hospital they had told me that it could go away, and it could also come back, and you would recognize it with the signs, I recognized it by coughing the fluids.

I: Okay, so, may you please explain to me, when did you start using this box?

P: I started using this box in February, it’s just that I forgot the date, that it was on which date.

I: Did you start using this box on the exact day that you started taking your pills…your TB treatment?

P: Yes, on that day they started giving me the box, I came here holding papers from xxx [hospital name].

I: xxx [hospital name]…where is xxx [hospital name]?

P: xxx [hospital name] Hospital. They gave me at xxx [hospital name], those papers. I had been given by the doctor at [hospital name], they had taken me to X-ray, When I came back from the X-ray, the doctor found out that I have TB, when I came back the doctor told me that TB is back, then I came here at xxx [clinic name] Clinic to take TB treatment. It had affected me before and now it came back again.

I: So, you say you had TB before and now it came back – so, briefly, how did you feel by the time they tell you that TB came back again?

P: I was felt sweating, I was unable to cover with the blankets and I was coughing. By the time I went to the treatment, I told them I’m coughing, then they told me to go and spit the cough fluids and they said they’ll tell me about the results. So, by the time I go back to the treatment the doctor told me to go to X-ray, that’s where I found out about TB.

I: How were your feelings when they were explaining to you that you have TB? How did you feel that TB came back again?

P: I was coughing, I was not sleeping, I was coughing the whole night.

I: So, who was briefing you about…about this box?

P: About this box, they briefed me here.

I: So, how did you feel when they briefed you about this box?

P: Uhm…I was no longer afraid cause I had it before. I knew that it could come back.

I: So, by the way they explained to you about this box, is there anything that you would like to change about the way they explained about this box?

P: Uhm…about this box, they told me to select the time to drink. I said “seven,” they said the box would ring at seven, I should intake the pills and it’s also going to ring on their side.

I: So, what could you brief me about this box – you may touch it – so, what could you explain to me about this box that how it works?

P: Oh, this box has got red light. That’s the one that shows that I should intake my pills - when this box rings, it clocks me that I should open and intake the pills.

I: So, besides ringing, what could you say about this red light, what could you explain to me about…the other lights?

P: About these other lights, no, I don’t understand them well.

I: Okay, no, I…I’m happy to hear that pa.

P: Yes

I: So, I’m requesting to move to my next question. So, here pa when I was preparing a consent form, I explained to you that I would need his experience, about this interview, and his feelings towards this box, right? So, pa, briefly, your experience when you use this box, what does this box helps with?

P: Uhm…this box helps me, not to forget the pills, yes. I always remember it, taking and opening it, thereafter, drinking my pills.

I: So, what challenges did pa meet when he using this box?

P: No, since I used this box, I’m no longer coughing.

I: So…so, briefly, are you working pa?

P: No, I’m not working.

I: What about temporary work?

P: Uhm…temporary work, when working I get dizziness. Since I started intaking the ARVs together with these pills of TB, I’m no longer having the strength.

I: So, when you say you no longer have the strength anymore this time - before you tested for this TB, even now when you are taking TB medication, how do you feel now on your body?

P: No, now if I have this TB medication, the coughing is slowing down, they said I should intake them for twelve months, thereafter they will explain to me how they reacted on me.

I: So…what do you recognise – so, by the way you say it reminds you to take your medication on time, when you look, what could you say this box makes your time of taking your medication easy or what a role is it playing on your treatment?

P: Uhm…this box is the one that shows me to intake my treatment first, it doesn’t make me to forget.

I: So, pa you said you stay alone, earlier.

P: Mm (yes)

I: So, is there anyone else who know that pa has got TB illness?

P: No, those who know are at home, I’m at the RDPs [door opening]

I: So, you say those who know are at home, [door closing] so, at home how did they feel when you tell them that you have TB?

P: Oh, they knew that I had it before and it went away, I told them it came back again.

I: Their feelings – how did they feel when you tell them?

P: Uhm…I stay with an old person there. She was born in 1950.

I: So, firstly when they saw you taking your treatment without box, it came back again, you took your medication without a box again, now it came back again, you have a box, what was their feelings when you come with box, for the third time?

P: No, they didn’t see the box until I take it out and show them, they thought it’s a watch when they heard it ringing.

I: When you took it out and show it to them, how was their feelings?

P: It’s just that I stay with only one person, I stay with my sister. Her children are married. When I tell her, she’s understanding, sometimes she is forgetting, she’s already old, she’s is born in 1951.

I: Yes. Okay, so, is there any other person besides you at home who had been infected by TB?

P: No, I just know about myself only.

I: [sound of papers] So, when you come here at the clinic for the first time, they showed you this box, had you ever seen it before?

P: No, it was for the first time I saw it here, I don’t want to lie, you won’t see this box most often from other people.

I: So, you said at home they know that you have TB?

P: No, they know, I told them that TB is back.

I: Yes

P: Yes, they know that I had it before and it went away.

I: Is there any other person besides your family, who knows that you have TB?

P: Yes, he’s also having it.

I: Uhm…how related are you with that person?

P: Uhm…he’s just a boy that I’m familiar with, he’s not one of my family.

I: But he is also having TB?

P: Yes, hey, he had been infected after.

I: Is he also using the box, or don’t you know?

P: He once told me-it’s not long time ago that he has it, but he tells me now on these months.

I: [inaudible segment] So…is there any other people who ever asked you about this box when you are on the way coming here at the clinic or when going back home?

P: No, the one I have now- there is nobody who have ever seen it.

I: Okay, so, by the time or the day they gave you this box, uhm…what were your worries or complaints towards this box?

P: Uhm…by the time they were giving me this box firstly; I was not having any worries because I knew that I had been infected earlier by TB.

I: So, since they told you that you have TB, did they ever visit you where you stay or at home?

P: Yes, when they need me, they arrive.

I: Okay, so, when they arrive – may you briefly explain to me what they’re doing when they arrived.

P: Uhm…when they arrive, they’ll only tell me that I’m needed at the hospital.

I: Okay

P: It’s just that they had been sent to tell me to come to hospital.

I: So, can you tell me about your feelings, when they visit you at home?

P: Uhm…when they arrive at my place, if they don’t find me, they call the person I uses his phone numbers that I’m needed at the hospital – here at the clinic, I would arrive and then they would explain what they need from me.

I: So, do you have phone pa?

P: No, I don’t have phone, I’m using these phone numbers. These are the phone numbers that I’m using, they are of the two people.

I: Did you say when they need you, they call those people?

P: Yes

I: Did these people ever receive the SMSs that remind you to take your TB medication?

P: Yes, these people when they need me, they usually find me.

I: So, what was your feelings when you find out that they needed you at the clinic?

P: Uhm… they go out to look for me and they find me, we don’t stay far from each other.

I: Do you ever opened this box more than once a day?

P: Yes, I open it in the morning.

I: Is there any other day where you ever opened it two or three times a day?

P: There was a day where I opened it and it wasn’t closed properly, then it rang. I went back and close it tightly; I had not packed my pills correctly.

I: So, how often does it happen that-where you found that you had incorrectly packed his pills?

P: It was happening that maybe I could pass a week closing it correctly and the other week closing it wrong, but now I’m aware of it, I always close it correctly.

I: Okay, I’m glad to hear that. But my question, I’m still going back to my question that did you ever open it in the morning and close it, then come back to open it during the day, open it now and then – even at night? Like you open it more than [inaudible segment] –

P: Yes, the other day I had opened it more than twice, it was by the time while I was no longer aware of it, I opened it in the morning and again in the day.

I: So, what happened to the point where pa reaches the stage of opening it twice a day?

P: So, it happened that I forgot to close it while I opened it, then I came back with the intention of closing it, and forgot again, on the third time that’s where I remember, and I closed it.

I: I’m glad – I’m glad to hear that. So, Pa you said when you open or when taking the medication….

P: I said I opened it when I was taking the medication, then I forgot to close it, then I came back to close it, it was late when closing it.

I: Oh, okay, I’m glad to hear that. So, when you take your medication, it clocks to them, do you know what they recognize about that you have taken your medication or not?

P: They…they recognize by the box, they said it’ll let them know if I open it and even if I don’t open.

I: Okay, does this box tells them?

P: Yes, they say it clocks on their side.

I: Okay, so, to show that you’ve drunk them - so what do think it clocks them with? Is it –

P: It clocks them that I drank or not, if I didn’t open or close it, it tells them.

I: Okay, no, I’m glad to hear that. There is something that we call adherence calendar, that indicates if you have taken or not taken your medication, it’s the one that we recognize through. Do you know about adherence calendar?

P: Is it the one that you signed earlier?

I: Mm mm(disagree) not that one. So, adherence calendar you see it on the tablet or phone that they use, have they ever shown you that adherence calendar?

P: They had never shown me.

I: So, in other words, you just know that when you take your medication, it updates them, but you don’t know know what they use to know?

P: No, that’s where I don’t know,

I: Okay, okay. So, pa you said you told them to set this box at seven o’clock?

P: They set it here to alarm at seven.

I: So, papa may you please explain to me how this box helps by ensuring that you take your treatment when it alerts you? Here I’m in need of your experience of this box, that how is it helping you to take your TB treatment?

P: No, I sometimes wake up in the morning, opening it before it rings at seven and then while I have finished drinking, that’s where it could ring.

I: So, papa do you sometimes open it before seven?

P: Mm (agrees)

I: Before it rings?

P: Mm

I: So, how often is it happening where you could open the box before the prescribed time of taking the medication?

P: Uhm…the past [inaudible segment] weeks, I was opening them, I didn’t even finish a month.

I: Oh…okay, so…you say this box remind you of taking your TB medication?

P: Mm.

I:Besides this box, is there any other way that you use, that reminds you the time of taking your TB treatment?

P: Yes, I wake up early in the morning around six waiting for seven without the box.

I: So, when you use this box, what do you say is difficult on the usage of this box?

P: It is that this box treats me well for my medication because if it wasn’t of it, my medication would be lost if they were not inside it.

I: Is there any other day or days where papa had visited somewhere, maybe visited some of his relatives at home?

P: No, I never visit with it (box), but they said when I go, I should go with it.

I: Didn’t you go with it?

P: No, since I received it in February, I didn’t go with it.

I: But did you ever go?

P: No, I didn’t visit to the point where I sleepover, I don’t sleep when I visited, I don’t visit at a far place.

I: Oh, okay, I’m thankful to hear that. There is something that we as the study people call it as Differentiated Care Model, so briefly by obtaining the SMSs, phone calls and they also come at your home, we call it home visit.

P: Mm

I: So, did you ever get a call or those people that you said we should call them when we need you, did they ever get a call asking you to take his medication?

P: The past months.

I: So, what happened when you failed to take your medication?

P: No, they phoned only to to remind me to take my medication, I was already drinking them.

I: Is there any other day where you forgot to take your TB treatment?

P: No, I don’t forget there.

I: So, papa may you please brief me from your experience that how is it having someone who could call you and remind you by the time of taking your TB medication?

P: No, having someone who could call you that you should take your medication, I realize that it helps not to forget.

I: When you say it helps you, how is it having a support or somebody who is always helping you, together with this box, ensuring that you are taking your medication?

P: No, I won’t forget this box, and the pills I won’t forget them, this is not for the first time I intake them.

I: Papa may you please tell me how it was by the time you had been infected by TB for the first and second time when you were taking your medication without a box, how was it taking the medication without a box?

P: I was waking up each morning, drinking them, I had a card to tick.

I: You had a card, that card you were [inaudible segment], how was that card working?

P: When I finished drinking medication, I had to tick them right, they had also given me a date to show that I have drunk from Monday until Sunday, I was ticking.

I: And then when you finished ticking what was happe - where do you take it to, what was happening after you finished ticking?

P: When I finished ticking the card, I was bringing it here at xxx [clinic name] Clinic, when I come on my due date.

I: Are there any other barriers or something that could disturb you to use this box and the treatment of yours?

P: No, this box doesn’t give me any problem, I drink my medication. There wasn’t any.

I: So, according to your experience papa, may you please explain to me what you were thinking when you say this box doesn’t give you any problem and then you don’t have any barrier, but do you think there could be any barrier that could disturb someone not you, that could disturb someone maybe to take his TB medication?

P: Another person how, the one that needs it?

I: The one that uses it.

P: No there is no other person who uses it. I…It’s just that I had never seen someone showing me this box.

I: I’m not sure if you understand me. Let me explain it again [inaudible segment]. There is another person that uses this box to take his TB medication, right?

P: Mm

I: According to your opinion, is there any other thing that could prevent someone who uses it, to use it?

P: No, where I stay there is nobody who is using this box, I’m alone.

I: Oh, okay, I’m glad to hear that. So briefly, please explain to me how satisfied are you with this box?

P: Uhm…I’m happy about box, it keeps my treatment and I see it for the first time, I don’t undermine it, I would hold on it until I finish with my TB treatment.

I: So, where do you place this box inside the house?

P: In the house I place this box inside the wardrobe.

I: Are you able to hear it when it is inside the wardrobe?

P: That’s where I sleep.

I: Okay. So, where do you place your pills?

P: I place my pills inside the box.

I: So, briefly, may you please explain to me how this box makes your pills safe where they stay?

P: Yes, the box prevents my pills from splitting.

I: When you say split…what do you mean?

P: It means if there was no box, they could be all over the place, so now they are safe inside the box.

I: So, pa according to your experience when using the box, may you please explain to me, what should we do as XXX[Name of the Organisation] or as clinic to ensure that this box could work – could be simple or easy, so that the box could work?

P: Uhm…this box is working well for me, even my pills fit well in here. It reminds me.

I: So, according to your opinion, what should we do to ensure that this box is simple that somebody could use it? What should we do on top of it?

P: Oh, this box reminds the patient not to forget taking his treatment with its siren.

I: Okay, so, I was asking pa about…I spoke about differentiated care which is phone calls, SMSs, and home visit, so, when you look at them, out of these three, which one papa thinks that is working too much to ensure that the person is taking his TB treatment?

P: It’s phone.

I: Okay, so, papa may you please explain bit fully when you say is phone – how simple does a phone works compared to those other ones?

P: Phone, they call those people that I’ve taken their phone numbers, they call them and tell them that they should always remind me to intake my medication, then I explain to them that no, I intake my medication.

I: So, is this method of calling a person and reminding him about the time of taking TB treatment helpful for someone who is taking TB treatment?

P: Yes, the method of calling a person is helpful.

I: So, according to your experience papa, may you please explain to me bit fully how it helps?

P: Those people that you took their phone numbers from, they call them and tell them to remind you to intake your treatment, then you tell them that no, I intake them, they said “no, we had been phoned that we should remind you not to forget intaking your medication.” I find the messages from them.

I: Papa, what do you like or dislike about the calls you are receiving that remind you about the time of taking your medication?

P: No, I like them all, there is nothing that I argue with, I find out from them, those people that I took their phone numbers.

I: Okay, what about this one of home visit, the one that they come visiting you where you stay, what do you like and what do you dislike about their thing of coming to your home to check on you?

P: No, they have never come because I don’t give them any difficulties. I find it from the phones that they did call me.

I: Papa do you know about counselling that when we talk about counselling what are we talking about?

P: No

I: Allow me to brief you about counselling.

P: Mm

I: Counselling is a conversation between the patient and his sister (nurse), they explain the importance of taking TB medication to him.

P: Mm

I: So, you recognize a counselling as a proper way to ensure that the person could intake his TB medication?

P: Uhm…even a counselling, I won’t argue with it, I listen to what they tell me.

I: When you look at this method of counselling, could it be – could it be the proper way?

P: Yes, it is also fine.

I: So, for the person who is neglecting his TB treatment, who are the right people to do counselling to ensure that the person who is neglecting his TB treatment would start intaking them?

P: It’s just that I don’t know the people who are responsible for counselling.

I: So, according to your opinion, when you look at this box, what should we do to ensure this box changes people’s lives?

P: Eish…it’s just that I don’t understand what you mean.

I: Let me…let me explain to you. This box reminds a person to intake his medication, right?

P: Mm

I: So, it works.

P: Mm

I: But for it to work easily or for us to ensure that the person is taking his medication thoroughly, what could we do on top of it, what could we add, something that you papa feels like this box is in shortage of?

P: They say if you don’t intake your medication this box would clock on their side, right? They’ll come to you, when they check them, they would find them standing on the same way, they hadn’t been drunk, that’s how they recognize them. [door opens]

I: [door closes] So, when you look at this method of counselling, phone calls and SMSs, who are – who are qualified enough or who are the right people to perform it? When they call you in this way, to whom would you prefer to talk to?

P: Oh, when they call me, they found me on the phones of those two brothers, xxx [ sibling’s name] and xxx [sibling’s name].

I: So, there are people that we call them CHW, those who are going around the street to people’s homes, who have been sent by the clinic to remind the people – they are the ones those who are going around checking if the people take their TB medication. I don’t know if you know about them.

P: Mm mm(disagree), as for me I prefer taking my TB treatment here at the clinic, I don’t want to be given at home.

I: Papa, why don’t you want to be given your treatment at home?

P: I shifted where I stayed before.

I: So, papa where do you live now?

P: I was staying at extension ten, now I shifted, I stay at the RDPs now.

I: So, papa you said – you said you had TB before.

P: Yes, now I’m infected for the third time.

I: So, according to your experience, when you look at this box, do you think this box should have been implemented sometime back?

P: Yes, we should have received them earlier, but even now it’s still working, it’s my first time to see it but I don’t undermine it.

I: According to your view, do you think the box could have changed the way you were taking your TB treatment earlier?

P: Yes, I should have obtained it earlier.

I: So, papa briefly when you look in the future or from now onwards, how does this box going to help the people?

P: Uhm…this box is still going to help a lot of people

I: Mm, okay. So, papa when you look at this box or when you look at this study of Aurum as a whole, is there any part that you feel like we didn’t perform it, the one that you feel like a shortage that should be quickly implemented?

P: No, there is no short part.

I: Okay, So, when you look at this box – you said this box has got colours that it shows

P: They are colours of the robots.

I: Plus, the sound –

P: Mm -

I: Alarm -

P: Mm

I: So, when you look at them which one do you think it works well – it works better than those others?

P: It’s an alarm.

I: Papa, may you please tell me about an alarm, why do you say is an alarm?

P: Alarm, when you didn’t open the box for taking your medication, it rings. It tells you that you didn’t take medication

I: And then what about those ones of the robots?

P: It works after you finished intaking your medication, after you closed the box.

I: So, here I’m requesting you to tell me about the overall, like your feelings as the whole about this box, like how do you feel about this box.

P: Uhm…this box, no, it treats me well, since I obtained this box, no, I don’t have any problem.

I: So, now like I just make the touch ups, like we reached the end of our interview, right? So, now I just need the questions that I could wrap-up our conversation with. They are those ones we spoke about them earlier.

P: Mm

I: So, I’m just in need of your feelings and…and your opinion that what you are thinking about it.

P: No, it doesn’t give me any problem at my side.

I: Mm

P: Mm. It treats me very well.

I: If you don’t have any problem with this box, according to your opinion, do you think is there any other person who could have the problem with this box?

P: No, if someone has got the problem with the box, I wouldn’t know because it’s unusual for someone to show you… that he takes the treatment. Still, I don’t know how I could find him.

I: Come again?

P: No, I say, still, you won’t get a person with a box. Maybe I could find him here at the hospital – here at the clinic is the place where I could find him. Still, it would be someone that I know, except that he couldn’t just show me the box.

I: So, papa, earlier when I asked him if he is working, he said that some of the pills that he is taking makes him dizzy, did you ever speak to the sister about the way some of the pills make him dizzy?

Uhm, the pills make me dizzy if I didn’t eat.

I: So, when you say they make you feel dizzy, like they make you dizzy…what is dizziness?

P: Dizziness is when you recognize darkness, you should not stand up quickly, if possible, you should rest a bit.

I: So, are you sure that understand you that to feel dizzy is to recognize darkness?

P: Mm

I: If you could – what happens when you stand up while you feel dizzy?

P: You could fall. There should be someone next to you, who would warn you not to stand up, but you will also feel that if you could stand up then you would fall.

I: Did you ever speak to sister that sometimes she feels dizzy by the pills?

P: Uhm…it’s just that I didn’t explain to her, I’m going to tell her when I come back on the thirtieth.

I: So, there are some other things that we call the side effects, so, the side effects are obtainable when you intake medication of any illness, a certain illness.

P: Mm

I: So, do you knows any side effect of taking…TB treatment?

P: No.

I: So, by the time they were starting with TB medication, did they ever explain you about some of the side effects of…of TB treatment?

P: Maybe I forgot.

I: Oh, okay, so, papa unfortunately we reached the end of our interview for today. So, our interview, uhm…I would stop it here. So, but before we end or stop it, do you have any final comments about this box, anything that he would like to talk about this box?

P: No, this box doesn’t give me any difficulties.

I: So, any final thing that you would like to speak about this TB illness?

P: It’s just that this TB illness makes me eat, I should find food.

I: Okay, so, uhm…the final thing that you could speak about Aurum, the people they came up with this box, what would it be?

P: Those people who came up with this box, they came up with an excellent idea, it doesn’t make someone to forget. Unless you just forget. Where they set its time, it’s signing.

I: If they were here maybe what would you tell them by thanking them with your mouth, what would you say?

P: Uhm…I would let them know that they helped the people…with this box, because it reminds you, it rings.

I: No, uhm…papa, thank you for taking your time to come here and perform this interview with me. So, for the time you have taken I say thank you but unfortunately, we reached the end of our conversation of today. So, papa, thank you.

P: Mm, no, you will stay fine.

I: Mm, okay. So, session ended, uhm…it’s uhm…11:45AM
