# Supplementary material for: Acceptability of Digital Adherence Technologies to support people with drug-susceptible TB in South Africa
Source: PLoS One. 2025 Sep 24;20(9):e0332103. doi: 10.1371/journal.pone.0332103 (PMC12459780; doi:10.1371/journal.pone.0332103)
Supplement: S4 File — (ZIP) [file pone.0332103.s004.zip › S4 Transcripts/PwTB/IDI 24_PwTB.docx]

**TRANSCRIPTION NOTATIONS**

| **Label Key** | **Meaning** |
| --- | --- |
| **I** | Start of each new utterance by the Interviewer |
| **P** | Start of each new utterance by the Participant |
| **N** | Note taker |
| **{ }** | Indicates that details were changed or pseudonyms were used to anonymise data |
| **( )** | Indicates the description provided to anonymise data |
| **XXX** | Words were omitted to anonymise data |
| **-** | Breaking into a sentence by the next speaker |
| **…** | Pause or drawn out words |
| **[ ]** | Indicates noise made, e.g. [laugh], [sigh], [pause] |
| ? | Beginning of utterance by unidentified speaker or questionable text |
| **[inaudible segment]** | Unclear section of the recording |

I: We thank you, for agreeing to do this interview with us, we are requesting permission to record you?

P: Yes, I agree.

I: We thank you, PID xxx, date xxxx (interview date) clinic [clinic’s name, facilitator [interviewer’s name], start time 11:15, thank you, uhh where do you stay?

P: In xxxxx (name of the area).

I: In xxxxx (name of the area), how long is the distance from the clinic?

P: Mmm when you walk, it’s about 50 minutes.

I: And then with transport?

P: 15 minutes.

I: So, which one do you use to come to the clinic?

P: I use a taxi.

I: How much is the fare?

P: R20

I: So, who do you stay with at home?

P: I stay with my mom, my child, and my grandfather.

I: So, when did you find out that you have TB disease?

P: On the 08^th^ of February.

I: Mmm and which symptoms did you have?

P: I was coughing, losing weight and sweating.

I: Mmm, okay, do you know this box?

P: Yes, I know it.

I: Who explained to you about this box?

P: It was … I forgot his name, that brother.

I: And then, how did you feel when they were explaining this box to you?

P: Uhh, I was okay, I have accepted.

I: So, how long would you say they took explaining this box to you?

P: Mmm, they took, mmm they didn’t take long.

I: Mmm.

P: Mmm.

I: So, the information they were telling you, was it easy for you to understand?

P: Yes.

I: Mmm, so is there anything else that you feel they should have told you when they were explaining this box?

P: They explained well; I heard them and I understood fully.

I: Before you saw this box at the clinic, have you seen this box before?

P: No, I haven’t seen it.

I: It was the first time seeing it here at the clinic?

P: Mmm.

I: So, if they can say explain this box to someone, what can you tell them? How can you explain it?

P: Mmm, this box helps, it reminds you to take your pills. They register you first for the box [cough], they ask you the time you going to take your pills [cough], I was taking mine at 09:00, when am home and then it reminds that take your pills and exactly at 09:00 it will ring. It rings and even the day before you come to fetch your pills, it rings again, like it helps, it reminds.

I: You mentioned that it helps?

P: Mmm.

I: If I can ask, how does it help?

P: By reminding, because at some other time you say you drink at 09:00, what if the phone loses time or you don’t have a phone, now this one rings. It rings independently, it reminds that it is 09:00, take your pills.

I: What can you tell about the ringing?

P: It rings mmm.

I: What can you say about that ringing?

P: But it is loud, yoh it rings.

I: You mentioned that it is loud?

P: Yes, it is loud.

I: That loudness, is it a good or a bad thing?

P: Mmm, at some other time it is bad, because we don’t know how is the situation where we are staying. Sometimes I stay with my sister and she is not sick, so when it starts to ring, she will say this thing of your is making noise man. Yah like the volume must be reduced a bit, “it is loud, I can hear it when am outside, this thing of yours is making noise.”

I: Okay, you mentioned that sometimes we don’t know how is the situation like where you are staying.

P: Mmm.

I: What do you mean by that, how is the situation where you are staying?

P: like the way am saying that sometimes you find that we don’t get along with one another at home, now when it start to ring, like it irritates them like you see that this thing it loud man, you see, and if you don’t open to take your pills, it will ring ,then it will ring waya, waya (forever ringing) after every 10 minutes until you open it and take out pills.

I: So, you mentioned- you said that sometimes it makes people angry at home?

P: Yes, like it is high, it is high.

I: So, have you ever found yourself in that position where someone is angry at you because the box rings loud?

P: Uhh, uhh, me, never happened.

I: Okay, you said it helps in reminding?

P: Mmm.

I: So, is there anything else that it helps you with?

P: It reminds.

I: It reminds?

P: Yeah, take your pills.

I: Beside being a reminder, is there anything else that this box helps with?

P: It makes one to take medication well because if you don’t take your pills it records at the clinic that on this day you didn’t take your pills, they have created your calendar that shows that on this day you didn’t take your pills. It makes you to take medication well because you know that at the clinic, they can tell that on this day, and there is no way you can say I did take my pills. Yeah, so it makes sure you take your pills well.

I: So, have they ever shown you your calendar when you came here at the clinic?

P: Yes

I: How was your calendar?

P: It was okay, because I only skipped a single day, someday; it was Saturday

I: Mmm.

P: Mmm.

I: So, uhh since you came here at the clinic, and told you about the TB disease, who did you tell about it?

P: Mmm, when I came here at the clinic I came with my mom, mmm then they wanted sputum. After that, they said they will call us and when they called me to come to the clinic, they told me I have TB and offered me counselling. When I get home, I told them that they say I have TB, I then started treatment.

I:So, in the family, how did they react when you came back, and told them you have TB?

P: What are they doing?

I: How did they react when you told them that you have TB disease?

P: Mmm, they were okay, that I managed to know early, and that I take my treatment. So, they were very supportive, mmm.

I: So, is it the first time you have TB?

P: Yes.

I: So, in the family, is there history of someone who had TB?

P: Mmm.

I: Their outcome, do you know them? The person who had TB, how did it end?

P: I was still young when my mom had TB.

I: Okay, so when you came with the box, how did they react?

P: Uhh they were okay, my mom said this today’s technology is smart, mmm, they were okay.

I: So, beside your family, is there anyone who is giving you support in taking your TB medication?

P: Yeah.

I: Who, are they?

P: Mmm, my baby’s father, he is the one I speak to most about many things.

I: So, what can you say is your experience with using the box?

P: Experience mmm (…) let’s skip.

I: What is it that you find ease to use on this box?

P: Skip.

I: Okay, are there any challenges you came across since you started using the box?

P: Uhh since I started using this box it has been simple for me because I can take my pills well. I know it will ring and there is no other way it will not ring.

I: So, you are saying you never had any challenges?

P: Uhh, uhh, just that the problem I had, I even ending up quitting treatment, I ended up stopping treatment because the pills they gave me. I could not even walk, I could not even pick up anything even my child. They were not good on me. I came to the clinic and told them, yoh! They said, “you will be okay ,you will be okay,” I ended up stopping treatment. Then people from the clinic came home and I ended up removing the battery so that it does not ring anymore, it does not irritate me. I could not walk, and I even asked them to change treatment for me and their response was a no because I had not yet reached the switching period, and they will tell me to come to the clinic. “We will see what we can do,” when I came, they gave me the same pills, same pills they just said you will be okay, you will be okay. I ended up not coming to the clinic not even taking pills.

I: So, you mentioned the side effect that you could not walk?

P: Yeah

I: Was there any other side effects, you had?

P: Yeah, I could not hold, just to hold, I could not.

I: So, these side affects you experienced them after how long you started taking your medication?

P: Took for 3 months

I: You took for 3 months?

P: Mmm.

I: Then that is when you had those side effects?

P: Uhh, uhh, I took medication for the first month because I started on the 8^th^ -the 22^nd^ , I could feel, I could feel man that my was not well. I could not walk properly, I could not seat down, I could not stand up as it was painful, when I had a month, I started seeing side effects, I carried on taking my pills I thought I will be okay they will get used to my body but nothing.

I: So, you have mentioned that you stopped taking your medication because of side effects, did you end up resuming medication?

P: Mmm, mmm.

I: Since you stopped that time?

P: Since I stopped. I stopped at the time, I only resumed yesterday because they came at home again yesterday, I saw that the pills they delivered, when they came, I was not home then also left containers. The pills they brought are not the same as those before, so I started taking them again.

I: So, the time you stopped taking medication, your support- what did they say- your family members and your child’s father?

P: They were complaining that I cannot stop taking medication *he,* but I told them that ever since I stopped, am okay ,am even gaining, I have gained. Am okay, am back to where I was. They said you will gain, you will be okay. I was so slim and very dark since I stopped treatment, am okay, I explained to them that you can see. I am the one who feels the pain when they were still saying you can’t stop treatment ended up saying that is up to you.

I: So, at this point when you were not taking your medication, how were you feeling?

P: Mmm, I had questions that I was asking myself that if this TB comes back stronger, what will happen to me, that when they said you will sleep on the bed with a blanket. I encouraged myself maybe it is not TB what I have, it was not TB maybe the clinic misdiagnosed me because why when I stopped treatment, I am, okay? I was worried, but at some time I would encourage myself.

I Why did you think it is not TB?

P: Because when I take treatment, I don’t get healed, it makes me sick, mmm.

I: So, have you ever received counselling about side effects on how they work?

P: They have told me that I have side effects, but they didn’t tell me about those that will happen on my body, they only told about what I would will feel sometimes, when I pee, my urine will change colour yeah, then they told me about pills, they didn’t tell me what will happen uhh, uhh.

I: Okay, are you working?

P: Am not working.

I: Since you started having the box, would you say it affected your daily life routine?

P: Uhh, it is okay.

I: How did it affect you in your life since you started having it?

P: Yes, this thing? Uhh it was just okay; I never had any issues with it, my only problem was with treatment.

I: So, have you travelled with it?

P: I did what?

I: Have you ever travelled with it, went somewhere with it?

P: Yeah.

I: If I may ask, where did you go?

P: What?

I: If I may ask, where did you go with it?

P: I went to my father’s place.

I: You went for how long?

P: Oh, I stayed for a while, I think two months [ cough] just after I got this thing, I told my mom that am going to my father’s place because things are not the same at my father’s and at my mother’s they are not the same, I said am leaving am going to father, so that I can take my treatment from my father, I stayed a bit, two months, then came back.

I: So, at your father’s , you stay with your father and who else?

P: With my sister and my other siblings.

I: How did they feel that you had TB disease?

P: Mmm, you know how they are, more especially these 18 years one’s, “uhh you have TB ,you will infect us with TB *hee, hee( hey)* you must use one cup, you must use your spoon alone,” mmm.

I: And how did you feel when they were doing so?

P: like they were boring me, they felt disgusted that (…).

I: So, about the box, how did they feel when you first came with the box?

P: *Hai* (no) they were just okay.

I: When you showed them for the first time, how was their reaction?

P: *Hai* (no) they were okay.

I: So, did you have any worries of carrying this box?

P: Mmm, mmm, I loved it.

I: Has it ever happened that someone see you with the box?

P: Yeah.

I: How did you feel about explaining what is it for?

P: Mmm, I was okay because I have already accepted, I told them this is for taking TB treatment.

I: Who was asking?

P: Mmm, my friends.

I: How was their reaction?

P: Uhh they were okay, they did not show any bad vibes.

I: Mmm (…) so, have you ever opened the box when not taking medication?

P: Yes.

I: What were you doing?

P: To be honest, I was coming from those things, I came from drinking beer then I said I do not want to mix it with medication, so I said ,today let me open a little then close so that it does not ring.

I: Okay, meaning you opened, you did not take medication?

P: Mmm.

I: So, how often does it happen?

P: It happened twice, two times.

I: And how were you feeling at that time that I did this thing?

P: *Hai (*no*)*, I was okay at that time.

I: Mmm [ cough] inside this thing,

P: Mmm.

I: Inside this box, what did you put except medication.

P: It was medication only, and the clinic care, TB green card.

I: Okay, so, where did you put your box?

P: I was putting it on top of the room divider.

I: Can I ask, why did you put it on top of the room divider?

P: So that when it rings, I could hear it.

I: So, at home when it rings, and somebody else hears it, do they call you?

P: Yeah even my child knows, maybe if am seating outside the gate, I will just hear the child saying, “*mama* (mother) boom.” They call it boom, “*mama* (mother), boom t is ringing.”

I: They call it boom?

P: Yes.

I: If I can ask, why did you decide to call it boom?

P: Because the time I was still at my father’s, I showed them when I arrived and he didn’t understand how it sounds like when it rings. Then the following day when it rang, I was outside at the neighbour’s, my cousin, it rang I was looking at the time I saw it is 9, boom, my thing is ringing when I got there I found my father not knowing what was ringing. At that time, I placed it inside the chest of drawer, at that time I can see that he is looking, I asked him what he is looking for he said there is a boom in here, what is this *tee, tee ,tee (imitating the box alarm)*? then I laughed at him. I told him and he said, |”oh I didn’t know how it sounds like,” so from then they called it boom.

I: Okay, so you mentioned that you removed the battery?

P: Mmm.

I: What was the reason for you to remove the battery?

P: I did not want it to ring anymore.

I: was it easy for you to remove the battery?

P: Yeah.

I: So, on the box, what would you say helped you a lot?

P: Mmm, it is the reminder and also that it keeps the pills safe because sometimes when you open pills, you unpin another extra pill mistakenly, if you put it inside the box, it is well stored so that you can drink it again.

I: So, what was difficult about using the box?

P: No, there was nothing difficult.

I: Mmm, so, have they ever called you here at the clinic?

P: Mmm (yes).

I: They were telling you about missing medication?

P: Mmm.

I: And then how did you feel at the time they called you at the clinic?

P: They were boring me, to be honest, they were boring because I came a lot here several times to tell them that I can’t walk, I can’t do anything. They were boring me even when they came at home, they were boring me, just the only person who was coming and understanding me was xxx (HCW NAME), mmm he was able to talk to me well, saying, “my friend don’t do that.”

I: What, is he?

P: He works here at the clinic, they are the ones to go to people’s homes and collect patients, mostly defaulters, mmm.

I:Mmmm, because you prefer xxx (HCW NAME) for home visits, what would say xxx (HCW NAME) does well, that others do not do well?

P: xxx (HCW NAME) like he can speak well, unlike some telling you that those are our pills and if you do not want them, bring them back, return them. They are our pills, but he was speaking well.

I: In future, people from the clinic, how can they improve.

P: Doing what?

I: In future, what would you say the need to improve, when they visit patients, so they don’t bore them?

P: They must talk well with people and most importantly, sometimes they just come to our place and I can do anything I like. They don’t know how am feeling and if they should come, and how are my moods. Then somebody gets there and starts shouting.

I: You mentioned that you can do anything?

P: Yeah, I can talk however I like, it is my home, so I don’t care about anyone when am at home. When am at the clinic, I must listen to all the rules no matter what it is, just so I can get my medication and leave.

I: And on the phone calls, how many would you receive per week?

P: When they call?

I: How many times do they call you in a week?

P: *ga* (once) 1.

I: once a week?

P: Mmm

I: So, on the phone call, what did they tell you?

P: “We are asking you to come to the clinic, mmm when can you come? When are you free, on this day, please come to the clinic?”

I: So, do you know this person who was calling you?

P: Tt was the brother who brought us here.

I: He is the one who called you?

P: Mmm.

I: So, have you ever received SMSs? That you have missed medication?

P: Mmm (no) mmm, SMS’s mmm, mmm. [cough]

I: Alright, is there anything that have ever prevented you to take medication? Like you mention that when you are coming from your things

P: Mmm.

I: like at that point, you don’t want to take medication because you do not want to mix it with your things.

P: Mmm

I: So, is there any other thing that has prevented you to take your medication?

P: Mmm (no), mmm.

I: so, how satisfied are you with the box?

P: Uh, the box is fine, it helps. It is fine, 100% just fine.

I: So, beside noise, is there any other thing you do not like about the box? You have mentioned that it is loud. Is there any other thing?

P: It is small.

I: It is small?

P: Mmm.

I: So, what must they do on it?

P: It must be bit long because these pills are this long, you must cut them for to all fit inside, must put others outside the box.

I: So, how is the box helping you to put medication safe?

P: How does it help me?

I: Yes, how does it help you to keep your medication safe?

P: Yeah, it helps, it is safe. You won’t say *hee* (hey) I lost my pills *hee, hee*, uhh, uhh you know when you put them inside you will find them inside unless you lose the whole box, there won’t be that you have shortage of pills because you lost them.

I: On overall satisfaction, about this box, how is it?

P: Mmm for this box?

I: Mmm including SMS’s, everything around this box?

P: *Hai* (no) it was fine, even the people who were calling they know how to speak to people, they were okay that is why when they say come on this day, when will you be able to come? I said I can come, they say okay, and I would really come.

I: So, is there anything they can do so that you can use the box easy?

P: What they can do for me is to change treatment, am okay with the box.

I: Okay, you said you never received SMS’s that you missed medication.

P: Mmm, mmm.

I: So, if truly, before they call, they send you an SMS, to say you did not take your medication, how would you feel about such a thing?

P:Uhh I was going to be like, I wasn’t going to feel okay, but I would not feel okay because I do not feel for myself.

I: Would you say the clinic should carry on using this SMS to remind people who misses medication?

P: Yeah, that one is fine, mmm.

I: What do you think can be bad about SMSs?

P: I don’t see anything that is bad because it shows that the clinic, that come to the clinic, that the problem is, on this day you didn’t come to the clinic yeah.

I: So, with the phone call, what is it that you love about phone call?

P: Mmm, sometimes they remind you, like I didn’t see its importance, I was taking medication well, I have never stopped because even a day before it reminds *gore* (that) don’t forget, it shows the orange light that tomorrow, you must go to the clinic, the red light means you must take treatment, the green light means that battery is finished.

I: You said the orange one is for saying come to the clinic?

P: Yeah.

I: The red one is for?

P: Take your pills now.

I: The red light?

P: Mmm.

I: Okay, *aow* (no) the green light?

P: It is to show that the battery is low.

I: Okay, no problem, so, what is that you don’t like about phone call?

P: I don’t see it importance, because this thing is telling me everything unless, if you have done a mistake and they don’t to come, they said let’s just call to tell you on this day you were supposed to come, it reminded you, but you did not come, passed two day still you didn’t come, let’s call her to come to hear what is the issue, mmm.

I: Would you say the clinic should carry on calling people?

P: Yeah, those who are not coming to clinic.

I: Okay, so with the home visit, what is it that you don’t like with people coming at your home?

P: That one [home visits] brings me unnecessary attention. Sometimes, my neighbor does not know I am taking treatment, and we know when the van from the clinic stops at your gate, we know the person didn’t take her medication they will say “she doesn’t drink her medications, they came for her” you see, that’s home visits.

I: How did you feel when they came like that?

P: like I said, I was okay, I was okay, they come with someone that I understand.

I: So, what is that you like about home visit?

P: *Hai* (no) there is nothing I like about it

I: So, information that they teach you about TB, what do you like or what can you say about it?

P: What am I doing?

I: The whole information they told you when you started TB treatment,

P: Mmm.

I: What do you like, what is it that you like about it.

P: There is nothing, I love it.

I: Why do feel like that?

P: Because when they take you to counselling, they say, “no, you see you are losing weight you will gain, you will be fine” but me I never got better total, I was getting worse, there is nothing I liked that they told.

I: Would you say the clinic should continue to do counselling for people starting TB treatment?

P: Yeah, we are different, counselling is fine.

I: On everything we have mentioned, counselling, phone calls, SMS, and home visits, what to you see has been the most helpful to people with TB so that they adhere well to treatment?

P: Phone call.

I: You feel like phone call can help people the most?

P: Mmm.

I: What do you see at least important?

P: Home visit, because home visit they come if I don’t want to come to the clinic I won’t come, they won’t get me into the van, it waste of petrol, when they come and ask me to come to the clinic tomorrow-come to the clinic, I will just say, just for they just go away, I would just say that I will come to the clinic tomorrow but I am not going, they will come back again, I will still agree you see home visit it is not important, phone call even they call, asking what is the matter why are you not coming to the clinic hee I was not there I was at Mpumalanga *hee hee* but I will come when they call I will come but if I want to come I will come if I don’t want to come I won’t.

I: Okay, how often does it happen that they visit you at home and you don’t go to the clinic?

P: It happened how many times?

I: Yes, how often would it happen?

P: To me?

I: Mmm.

P: Mmm, 3 times

I: Mmm, when you tell them you will come, you knew still you won’t come?

P: Yes, I knew that I won’t come.

I: The information they were telling you has it never changed your mind?

P: uhh, uhh.

I: And why did it not convince you?

P: There was nothing that they were telling me because they will still be telling me that I should come to the clinic to take treatment, come to take treatment, I tell them you can see me, they came when I couldn’t walk, I said you could see me, you can see me and we get off the taxi far before the school, I had to walk long on the pavement and at that point I could not walk properly, am okay with those pills.

I: When you tell them then you get of the taxi far and you can’t walk, how is their response?

P: Uhh they would just say you can just walk slow you will get there.

I: Is the any how you feel like the clinic supported you with your side effects?

P: What?

I: Is there anyhow you feel like the clinic supported you because you had side effects?

P: No ,there is no support I got from the clinic.

I: Mmm,

P: They kept on saying you will be okay, it is not yet used to your body or its still getting deep in your body, or it is waking old diseases you will be okay, but I was surprised this other Saturday, I came here I brought the kid so that they can check for TB and her results were going to be released on Saturday they said I should come on Saturday I came with the child, now when I wanted to seat down now the sister asked me what is wrong are you feeling any pains? I said I have pain in the legs I am struggling with my legs, I said since I started TB treatment, she said no, why it’s doing that on you, you see her too she was surprised she doesn’t know this side effects, she said this can’t be pills and I asked why she doesn’t know them, I said since I started TB treatment.

I: Okay, alright so, what do you think we can improve with everything, (…) what can they improve when it comes to uhh SMSs, phone calls, home visits and counselling, what can they improve?

P: What they can improve? That those doing home visit should also bring treatment, because they can see that you are not coming here. It will be your burden if you don’t take medication after they tried to bring you treatment and am not taking it what is my problem. Here they should improve there, they must not come empty handed, they should bring treatment.

I: So, you feel like if they can bring you treatment you can be convinced to take your TB medication?

P: If they have changed for me.

I: Okay.

P: Not the rifinah, I don’t want them rifinah, unless they try me on rifafour. Is it rifafour or what, yeah?

I: You would take medication if they have changed for to another medication?

P: Mmm.

I: And the SMSs, how can they be improved?

P: like SMSs, I don’t know because I never received it.

I: And phone call?

P: Phone call is fine.

I: And counselling, how can they be improved?

P: Me at counselling, they did not tell me these things, and even the way I was counselled you told that when you start TB treatment, you will not drink alcohol for 6 months, you can’t smoke for 6 months [cough] that for 6 months. They didn’t tell me this things here at the clinic they just gave me treatment, and counselled me saying, if you don’t drink your medication shame, you will come back being pushed on a wheel barrow, they will push you with a wheel barrow if you don’t take your treatment. This, they didn’t tell me.

I: When they gave you that kind of counselling, how did you feel?

P: They were boring me because there was nothing they were saying.

I: Mmm.

P: Mmm.

I: So, how do you think counselling should be done?

P: Who must do the counselling? The brother who brought us in here, that brother can talk to people well, he is very calm that brother.

I: Okay, so this box, who must teach the patients about how the box works?

P: Him again, he is the one who showed me the box, he even registered me, he registered to the box.

I: So, on the box, what do you think must be improved?

P: It is small.

I: Mmm, size?

P: Mmm (yes).

I: Any other thing that can be improved?

P: Uhh, uhh.

I: Beside sound, that it is loud, how do you feel about the sound?

P: I have no problem with it, am thinking for others, I don’t have a problem with it

I: Okay, material?

P: But like at the end it was starting to irritate because it rang to remind me to take pills that are not well on me, that is why I ended up taking out the battery, so it does not ring anymore, keeps quiet and just stay there.

I: So, since you removed the battery, have you connected it back?

P: Mmm (no), mmm, I just placed it safe there.

I: When they explained here at the clinic, did they mention anything about removing the battery?

P: Mmm they said when it finishes it will show I should come to the clinic, I should bring it here at the clinic they will charge it, or change it so that it can continue to ring, but they never said I should do it myself.

I: Okay, (…) I think we have reached the end of this interview now. I want you to tell me what can you say-anything about phone call, SMSs, and using the box, is there anything you would like to comment on?

P: No, am fine.

I: And then about these things we are doing to help people phone calls, SMS, and home visit, would you say we should continue with them?

P:Mmm, it is fine, it helps like now I can tell that I want them to change for me, if they can change for me, I will take my treatment because I even came to interview. There are people who must work with people and there are others who must not, can’t work with people.

I: Okay.

P: It needs someone who can speak to people (mumbling) it is like they are working to finish up, it is like they are note explaining for you to understand they just want to finish up, it does not need such people. But it does help.

I: Okay, since you had serious side effects, severe side effect, have you tried going to a private doctor or nurse?

P: Mmm(no), mmm.

I: Okay.

P: I have never.

I: So, when here at the clinic refuses to change your medication, is there anything that you tried to do?

P: Uhh, uhh I didn’t do anything, nothing.

I: Okay, thank you, I think we have reached end of our interview, we thank you for your time ad that you agreed to come here and do this interview with us if there is any question or anything you want to ask, on this copy I will give you there are numbers, you can call us anytime or even this brother who called you here. You can call him and ask him any question you feel like you have. We thank you sincerely for coming here, we thank you, end time 12:02.
