# Supplementary material for: Acceptability of Digital Adherence Technologies to support people with drug-susceptible TB in South Africa
Source: PLoS One. 2025 Sep 24;20(9):e0332103. doi: 10.1371/journal.pone.0332103 (PMC12459780; doi:10.1371/journal.pone.0332103)
Supplement: S4 File — (ZIP) [file pone.0332103.s004.zip › S4 Transcripts/PwTB/IDI 25 _PwTB.docx]

**TRANSCRIPTION NOTATIONS**

| **Label Key** | **Meaning** |
| --- | --- |
| **I** | Start of each new utterance by the Interviewer |
| **P** | Start of each new utterance by the Participant |
| **N** | Note taker |
| **{ }** | Indicates that details were changed or pseudonyms were used to anonymise data |
| **( )** | Indicates the description provided to anonymise data |
| **XXX** | Words were omitted to anonymise data |
| **-** | Breaking into a sentence by the next speaker |
| **…** | Pause or drawn out words |
| **[ ]** | Indicates noise made, e.g. [laugh], [sigh], [pause] |
| ? | Beginning of utterance by unidentified speaker or questionable text |
| **[inaudible segment]** | Unclear section of the recording |

I: Uh, sister do you agree for us to record today’s interview?

P: Yes, I do agree.

I: Ok. Today’s date is xxxx (interview date).Location: xxx (clinic name) and the patients’ PID is xxx, the language that will be used is IsiZulu and the time is now 10 :34 AM.

I: Sister, I would like to get to know you first. So, could you please tell me where you from, where do you live and who do you stay with?

P: Uh, I am a homeless person; I stay in, in, in the street near a park which is located next to the xxxxx (building near the shelter) I stay with friends that have I met in the street…I visit a shelter called xxxx (name of shelter for homeless people) to get food and to take a shower.

I: Ok. Could please elaborate further for me from what you just told me about sleeping in the park? Do you sleep in the park or near the park?

P: No. I sleep in the park; I have found a safe spot where I sleep and put my belongings.

I: Ok. So, who do you stay with in the park?

P: I stay with friends I met in the street who also don’t have a place to live. However, I don’t have a close relationship with some of them, it just that we share the same environment and that all.

I: Ok. So, when did you find out that you have TB?

P: I found out after I had been hospitalized for two months following the incident that took place on the 04^th^ of January 2020, where I started vomiting blood and collapsed. I was then taken to the hospital by an ambulance and then on the 18^th^ of January, I was started on TB treatment.

P: Uh, ok. Earlier you mentioned that by the time you found out that you have TB, you were already staying in the park. So, what I would like to know is did any of your close friends from the park get tested for TB?

P: I don’t know because I was admitted and stay in the hospital for two months. So, I wouldn’t know what happened after I was gone.

I: Uh, so when you found out that you have TB, you didn’t share your TB status to them?

P: I did tell them after I was discharged from the hospital.

I: Ok. So, you told them when you were back from the hospital?

P: Yes, simply because some of them were asking where I was for the last two months as they were not around the day I was admitted to the hospital.

I: Ok. Following your return from the hospital, didn’t some of them thought of testing for TB?

P: I only know of two who decided to get tested and the results came back negative. These two individuals are the ones who sleep close to me, and I was told by them that the results came back negative.

I: So, who gave you your TB results at the hospital?

P: I was told by a doctor from the hospital.

I: And then how did you feel when a doctor gave you the results?

P: I felt bad, but I was glade that it was early detected which means chances of being cured are high.

I: Mmm…

P: I didn’t take the news well because once you know about your TB status, you start having too many thoughts including death, but I was ok following the counselling session with a doctor. He explained everything to me; he even told me that TB is a curable disease and told me that they were going to start me on TB treatment immediately.

I: Ok. So, before the doctor explained to you about TB, did you know anything about it?

P: No, I didn’t know much about TB.

I: Ok. So, you didn’t know how TB is spread and how one does one get it?

P: No, I didn’t know.

I: Ok, I understand. So, the doctor is the one who gave you the results and explained to you about TB?

P: Yes, it’s a doctor.

I: Ok. A doctor from which Hospital?

P: A doctor from xxxxx (hospital name).

I: A doctor from xxxx (hospital name)?

P: Yes, doctor xxx {Doctor’s name}.

I: Uh, ok. Umm how then did you end up here in xxxx (clinic name)?

P: I started here in xxx (clinic name) following my discharged from the hospital. I was told from the hospital that going forward I will be receiving my TB medication from this clinic; they gave me a letter that I had to produce on my first visit here at xxxx (clinic name).

I: So, you are saying that you were told from the hospital that you will now receive your medication from this facility.

P: Yes, I was told from the hospital on the day I was discharged. They told me to produce a letter that had all the details to whomever is responsible for my TB medication here in xxxx (clinic name), in the TB room.

I: Mmm, do you know this box?

P: Yes, I do know this box.

I: Who explained to you about this box?

P: A nurse who gives me TB medication is the one who explained to me.

I: Are you saying it’s a nurse that gave you this box?

P: Yes.

P: Ok. Could you explain to me what did you understand about this box from what a nurse has told you?

P: What made me happy about being a recipient of this box is that it has a reminder which will alert me when it is time to take medication since I forget sometimes. A nurse told that this box has an alarm which can be set according to my chosen time, it will make a sound when it is time for me to take medication.

I: Ok. I hear you, what else did she say about this box?

P: She also explained to me that I should put my TB medication in the box and take from this box, and they will know whether it was opened or not on the pre-set time. It happened that I missed medication for few days and some people came looking for me. I also noticed that if you open this box before time, it won’t alert you when it’s time to take medication and I think that is one of the reasons sometimes it becomes difficult for the nurses to believe me when I tell them that I am taking my medication.

I: Ok, I understand. I would like us to go back a bit, where do you stay again?

P: I stay at the park; I move around since I don’t have a permanent place to stay.

I: Ok. Please tell me where do you place your pillbox since you stay in the streets?

P: Uh, I always put in it inside my bag that I always take with me everywhere I go. I also use the very same bag as my pillow.

I: Didn’t it happen sometimes that your box falls out of the bag?

P: No, it never happened before ever since I started taking it with me.

I: You said it was the nurse who gave you the box, is that correct?

P: Yes.

I: Please tell me how long did it take for a nurse to explain everything to you?

P: Uh, I can say that it took her maybe 20 minutes to explain to me since she also explained about TB medication.

I: Ok…have you seen this box before? Since you said it a nurse that gave you this box, have you seen it before then?

P: No, it was the first time seeing it here at the clinic.

I: Have you ever missed your daily dose since you live in the street?

P: Yes, it happened before; maybe for two days.

I: Could you explain to me the reason you missed your medication for those days?

P: I missed my daily doses for those days because I had no food and the pills that I was taking at that time were making me sick if I took them on an empty stomach; they were making me dizzy and weak. So, would not take them for that reason.

I: Ok. So, you didn’t take them for that reason?

P: Yes, but I no longer have that problem now especially with this other line of drugs that I am taking since they change me from the first line of TB drugs which were even making me hungry. The ones that I am taking now don’t make me sick even if I do take them on an empty stomach. Now I don’t skip my medication; I take them every day.

I: I would like to know how do you take your daily dose?

P: I take them once a day; I take three pills and one that is whitish in colour.

I: I hear you; I would like to find out from you if there anything you would like us to change on this box? Maybe the way it works or anything that might stand out to you? If is there anything you would like us to change at all?

P: This box? ...No there is nothing I wish to be change on this box. What I can say is that it is very important for anyone who uses this box to finish the medication inside.

I: I mean what do you wish to be change here?

P: No, I don’t want anything to be changed on the box; for me it is fine the way it is designed.

I: Earlier on you mentioned that it was your first time seeing this box from this clinic?

P: Yes.

I: Since you started using this box, are there any challenges you have encountered?

P: No, I haven’t experienced any difficulties so far except the issue of food that I mentioned earlier on.

I: Ok. So, it was only the food issue?

P: Yes, it was only the food problem but since they changed me from the other drugs, I no longer have a challenge. I can take the current ones on an empty stomach; I don’t feel sick like I used to before.

I: Ok. Are you working?

P: No, I am not working.

I: You are not working?

P: Yes.

I: Ok. Does that mean you are always at the park or in the street every day?

P: I spend a lot of time at the shelter since that is where I get food and able to shower.

I: Have you told anyone about this box ever since you, had it?

P: I told my friend after she started asking questions about it.

I: Ok. So, this friend of yours, does she also stay in the park or what?

P: Well, I could say this person is more like a girlfriend of mine. We are seeing each other.

I: Does she also stay with you in the park?

P: No, she only comes by to see me; we don’t stay together.

I: Ok. Where did you guys meet?

P: We met from the shelter; she is the one who showed me around when I first visited there.

I: Ok. What I would like to know is by the time you started attending at the shelter, had you already known about your TB status?

P: Yes, I had known about my TB status by then.

I: Ok. Does that mean you found out from the shelter?

P: No, I only found out about my TB status when I was in the hospital for two months.

I: Mmm, you spent two months in the hospital?

P: Yes, I was hospitalized for two months. I even started taking medication when I was in the hospital before the box.

I: Ok. Who did you tell again about the box?

P: I told my girlfriend; I told her after she asked me about it since she could hear it every time when it beeps, she once asked me what is it that is always making noise in my bag. Then I explained to her that I got this box from xxxxx (clinic name) ever since I started taking TB medication. I also told her that this box will remind me when it’s time for me to take my pills.

I: Ok. You mentioned that there were times when you couldn’t take your pills?

P: Yes, there was a time when I was struggling to take them.

I: What was the reason for you not to take your medication?

P: As I said, there were times when I didn’t have food and the medication, I was taking at the time was making me sick if I take them on an empty stomach. However, I don’t have that problem with the ones I am taking right now, I take my pills every day now; I don’t skip.

I: Mmm…

P: Even if the box beeps before I get to the shelter, I take my pills then and procced to the shelter to get food. I don’t experience any problems with medication I am taking now.

Ok. You said it was the nurse that explained to you about this box?

P: Yes, it was the siter/nurse.

I: What exactly did she say about the box?

P: She explained that they can monitor my daily intake; she explained that they will be able to see if the box had been open or not. So, that will give them an indication that I didn’t take my pills for that day.

I: Ok. So, the nurse explained to you that they can monitor your adherence?

P: Yes.

P: Ok. Where do you place your box?

I: I always have it in my bag.

I: Does that mean your pills are inside the box as we speak?

P: Yes, I have it with me as we speak.

I: Ok. What else do you put inside your box?

P: I only put my TB medication.

I: Ok. In what other ways does this box helps you?

P: It helps me remember when it is time to take my pills. It doesn’t miss; it beeps exactly at the time it supposed to.

I: You said you are unemployed, right?

P: Yes. I am not working.

I: And where did you say you stay?

P: I am unemployed, and I stay at the park.

I: Ok. So, you were using this box?

P: No, I am still using it.

I: Have you ever receive a call from this clinic when you have not taken your pills?

P: Yes, I have after I have missed for two days but it doesn’t happen now that I receive a call for that reason since I am taking my pills every day ever since I was started on the other line of TB drugs which doesn’t make me sick.

I: So, tell me, which pills were making you sick?

P: It’s those pills I was taking in the first two months of my treatment before they switched to the ones I am taking now.

I: Ok. Ever since you started using this box, have you ever received an SMS?

P: Yes, I have.

I: How many times have you received this SMS?

P: It happened twice; it was for those days when I have missed my pills because of the food issue.

I: Ok. Did it ever happen that you open your box more than once a day?

P: No. I only open it once a day when its time to take my pills.

I: Mmm, so you never opened it maybe before time?

P: No, I don’t open it before time now because I did it before and I realized that it then never makes a sound when it is time. This happened when I was also keeping other medication in the box such Panado, but I no longer do that.

I: Mmm, ok. I hear you. Did it happened maybe that-have you ever experience any challenge while using the box?

P: No, no challenges so far.

I: Have you ever received a call when you have missed taking your pills for two days or more?

P: No, I only received an SMS not a call. This SMS was saying “you have missed your medication”. Then it also happened that I received an SMS from this clinic which was warning me that my medication will be taken from me if I continue to not take it; it will be given to those you need it.

I: Uh, so you only received an SMS , not a phone call?

P: Yes. I think that SMS from this clinic was meant to scare me a little bit so that I continue to take my pills as I am supposed to since no one came looking for me, not even the Police.

I: Ok. So, you never had a home visit?

P: No one came, just the SMS I received.

I: Did you have an idea where was the SMS coming from?

P: [laugh]No, I didn’t know but I thought it was from people who own this box.

I: [laugh] So, one came looking for you?

P: Yes, no one came.

I: So, what is it that makes it easy to use this box?

P: I don’t wish for anything to be changed on this box; it fine the way it is. There is no other way I would want it to function.

I: How did you feel when you received an SMS reminder?

P: I don’t have an issue with those SMSs just that I was a bit shaken when I received the other one which mentioned the Police because I don’t have a good relationship with the police.

[laugh]Yoh, you never had a home visit since you started taking TB medication?

P: No.

I: So, how do you think this box can benefit someone else?

P: I think it can help anyone the same way it helped me; reminding them to take their medication on time because that is very important. So, I find it very helpful; it really helped me a lot and I wish others can benefit from it the same way I am benefiting. However, they must not mention the Police because that might scare some people.

I: Ok. Is there anything you would like to add?

P: No, for me the box is fine the way it works. I wish for other people to receive it.

I: Ok. So, you don’t have a problem with receiving phone calls, SMS reminders and home visits?

P: I don’t have a problem.

I: Mmm, ok. I think we have come to an end of our interview.

P: Ok.

I: Before we end our interview, I would like to ask you some few questions. You said you stay at the park, right?

P: Yes, I stay at the park.

I: Ok. So, what I would also like to know is how did your friends react when you tell them that you have TB?

P: It didn’t sit well with some of them because they even worn masks all the time when I am around.

I: Didn’t that make them think of testing for TB?

P: Two of them told me that they had gone for testing and the results came back negative.

I: Do you have any symptoms of TB then?

P: I had back pain, lost a lot of weight and then on the last day I vomited blood. I was then taken to the hospital and that is where they discovered that I have TB. I didn’t experience other common signs such coughing.

I: How did you feel when you told by the doctor that you have TB?

P: I was very sad you know, and I was expecting anything worse from there onwards, but I also wished myself speed recovery.

I: Did you know anything about TB then?

P: No, I didn’t know much about it.

I... Thank you so much for your time sister. Right now, the time is 11:15 AM here at xxxxxx (clinic name) .
