# Supplementary material for: Acceptability of Digital Adherence Technologies to support people with drug-susceptible TB in South Africa
Source: PLoS One. 2025 Sep 24;20(9):e0332103. doi: 10.1371/journal.pone.0332103 (PMC12459780; doi:10.1371/journal.pone.0332103)
Supplement: S4 File — (ZIP) [file pone.0332103.s004.zip › S4 Transcripts/PwTB/IDI 26_PwTB.docx]

**TRANSCRIPTION NOTATIONS**

| **Label Key** | **Meaning** |
| --- | --- |
| **I** | Start of each new utterance by the Interviewer |
| **P** | Start of each new utterance by the Participant |
| **N** | Note taker |
| **{ }** | Indicates that details were changed or pseudonyms were used to anonymised data |
| **( )** | Indicates the description provided to anonymous data |
| **XXX** | Words were omitted to anonymised data |
| **-** | Breaking into a sentence by the next speaker |
| **…** | Pause or drawn out words |
| **[ ]** | Indicates noise made, e.g. [laugh], [sigh], [pause] |
| ? | Beginning of utterance by unidentified speaker or questionable text |
| **[inaudible segment]** | Unclear section of the recording |

I:Huh LOUD, Today we are in xxx (Clinic name),Huh today’s date is the xxxxx (interview date) Huh.The time is 12:00 Huh ,the patient’s PID is xxx (.) Huh May we please audio record this interview Huh, because we need to receive all the information. Incase it happens that I forget some of the information you have shared , xxx (intern’s name) as the note taker might also forget.so that’s the reason for audio recording.May I have the permission to audio record this interview.May I please find out from you, if it’s okay that we audio record this interview or there might be a problem?

P: There is no problem, you may proceed recording.

I: LOUD ok, so may you please Huh tell me about yourself or whom do you stay with Huh how many people are you staying with?

I: At home where I currently stay, there are three of us. Two children, the other….they are two teenagers. One is doing grade 12 and the other doing grade 8. Sometimes their dad stays with us only when he is around but most often he is at work.

I: ok, so you stay with your children most of the time. (Car hooting).

P: Yes, it’s just the three of us.

I: Ok, so, when did you perhaps find out that you have TB, or when were you diagnosed. when did you find out do you still remember?

P: (Generator sound) Yes, I do remember, LOUD, I found out on February that I have TB.I.. I… I didn’t go for testing, I had no symptoms.

I: Mmm

P: I... I... I was on going to the pregnancy, so they run all the tests, that’s when I found out that I have TB, which was month end of February.

I: Yak, ok, was it here in xxxx clinic, or some other clinic?

P: It was here at the xxxxxx clinic.

I: oh ok, oh ok, and then symptoms. You mentioned that you had no symptoms when you arrived?

P: I had no symptoms that I know off. So they had explained to me on what are the symptoms .so I might be having those symptoms, so they listed the ones they know off, such as losing weight, night sweats, losing of appetite. And I had none of the above symptoms. The the the one I symptom that I had was having a headache which lasted for two weeks. A week won’t pass by without me having a headache, and they mentioned that it might have been one of the symptoms, because since I’ve started taking my medication the headache isn’t bothering me no more.

I: Oh ok ok,so when you came here, were you already taking your TB treatment.so when coming for your appointments Huh were you were you walking or perhaps you were using a local taxi or do you stay around it’s a walking distance?

P: Huh I ta- I travel by transport.

I: Yes.

P; Mmm

I: How long is the distance from where you stay?

P: It’s about 1,5km.

I: Yes, Let’s say how much time does it take?

P: Time

I: Approximately how much time does it take from where you stay to coming this side?

P: LOUD, Approximately, 30 minutes .when traveling by taxi, only when it doesn’t use the long route its 15-30.Let’s say the maximum is 30.

I: Yes oh ok, so, the box right. The box right.so you did mention to me on when you started taking your treatment. I am thinking Huh, you were diagnosed with TB, and then you started taking treatment and then you were given the box after?

P: Yes, I received the box after.

I: The box right, the box right, what can you explain about the box, on which understand about the box?

P: Huh what I understand about the box, is that it... it... it... has a reminder on what time do you prefer [Inaudible segment] it’s good for storing your medication or treatment. On the month which I was working [Inaudible segment].so its portable, because I can even put it inside my bag.so at the moment if it happens that I don’t have have a phone and its 7’o clock, I will remember that its time when its starts making beeping sounds *tititi,* I realise that it’s time for me to take my pills. And they are safe inside, because sometime before using the box, I kept my pills inside my bag, and sometimes they would get wet or they have spilled, but inside the box they are kept safe.

I: Huh ok, Huh, you’ve explained to me before Huh about the symptoms, that you found out here that you have TB ,because you came here for other certain things. And then they did check-ups .you found out that you have TB, they also explained to you about TB symptoms. And described symptoms such as losing weight, so do you recall when the Sisters were explaining to you or rather at the TB room, which symptoms do people with TB have, do you still remember?

P: I can only recall the headache symptom, they also mentioned one on having Stomach aches.

I: ok, everything is made clear. So, the box right, when did you start using it?

P: I started using the box in March.

I: March?

P: Yes, I started in March, because month end of February, I was still using…I had not received the box.

I: Huh this year in March?

P: This year.

I: oh, ok ok, LOUD, so before using the box, you started taking treatment before the box, may I say that are there any noticeable changes since you weren’t using the box versus currently when you are using the box, are you seeing any difference or change?

P: Yes, there is a difference.

I: With having the box?

P: Mmm, because sometimes I would forget taking my pills, for instance I would set an alarm on my phone and its right next to me.and tell yourself that you would return shortly after doing something and then you forget. But the box automatically [Inaudible segment] when its beeps you open it.

I: Mmm, you mentioned that the box has a reminder, so how is reminder helping you to take your pills?

P: The reminder is also helpful, because the box beeps, and it also reminds me when I have to come to the clinic whenever I see a light. (.) An orange light.

I: The orange one?

P: Yes, and then I remember that it’s my clinic date.

I: Mmm, ok, so this light, how did they explain to you that the orange light means that you have to return to the clinic?

P: Yes.

I: Then, what about the other lights?

P: The red one, the battery is about to end, they explained the battery?

I: oh ok, then who explained to you about the box, who was it here in the TB room?

P: I found Ms.xxx (intern’s name), she is the one who explained to me about the box.

I: Yes, do you perhaps know what her job title might be, is she a nurse or an intern?

P: Quite frankly I don’t know whether she is a nurse or…

I: (Laugh) ok, but she explained the box to you, handed you the box and told you how it works?

P: (Cough) Yes.

I: So when xxx (intern’s name) was explaining to you, was her explanation clear enough for you to understand that here is the box and it’s like this and that and it will help you in this manner?

P: Yes she explained to me in a meaningful manner, that the box will help me and I should quickly take my pills, the box will remind me, and that my pills are safe inside the box, she even mentioned that the box reports to them when I have drank my pills, and if there was a time when I didn’t drink my pills, they can even remind me to take my medication.

I: oh, so she mentioned that if you had not drank, they will remind you.so how you do they remind you?

P: It does show [Inaudible segment]

I: Ok, so this reminder, how do you perhaps get it, do they call or do you receive a sms, did she mention how will you receive this reminder?

P: I couldn’t hear it clearly and how it works [Inaudible segment].

I: ok, so how long was her explanation, do you remember, you can estimate how long it took her to explain everything about the box to you. How long did it take or you don’t remember exactly how long the explanation was?

P: I think she took 15 minutes when she was explaining about the box ,how it works ,what kind of features does it have, and I should put it in a safe place ,cause sometimes it won’t beep when I have put in a wrong place.

I: Ok, are you saying she explained to you on the manner in which you should put the box?

P: Yes.

I: or there is a specific place where you can put it?

P: Yes

I: ok, perhaps when she was explaining to you, did she perhaps mention which is the right place?

P: She never mentioned it, but she explained that I… (.) What can I say, I should try and put it properly.

I: ok, so where do you keep your box at home, in the bedroom, sitting room. Where do you keep it, as we are conversing with you right now? (Laugh) if I can say let’s go to your home right now, where can we find it?

P: I keep it in my bedroom, on top of the TV, because I have to drink in the morning.

I: oh ok, so you’ve placed it where it’s convenient for you to hear it?

P: Yes, It’s nearer to my head, it stays there.

I: What time in the morning do you drink your pills?

P: I drink them at 7.

I: Oh ok, so before you came here, you came here and found out that you have been infected with TB. And then you started treatment, and then time went by, and then xxx (intern’s name) gave you the box and she explained to you on how the box works, you will keep your medication inside the box.it works like this and that. Before you knew about the box, did you have knowledge about the box or have you seen it somewhere, or you it was the first time seeing it when xxx (intern’s name) gave it to you?

P: (Cough)

I: I’m sorry, I’m sorry.

P: It was my first time seeing it in xxx (intern’s name) possession. I have never seen it before.it was my first time seeing it on the day.

I: (.) ok, so since you started using the box (…..) Huh, can you describe how you’re your experience been, I mean if it’s helpful how is it helping you if it’s being helpful?

P: Its helping me, it makes the job easier, because it beeps. Obvious you open it and take your pills out and drink. (LOUD) it makes the job easier.

I: ok, so you mentioned that you are working, I heard you saying that you are working?

P: I have worked before, but we are still put on hold.

I: oh, ok so before you were put on hold, did you have the box or not?

P: I once carried the box at work, because I was leaving for work at 6:30.

I; ok.

P: so come 7 I am already at work.

I: ok, so you took your pills after 6?

P: I take them at 7.

I: ok, 7.

I: oh, so you carried it with you to make sure that you make it for 7,

I: oh, so you never drank and then left, you preferred rather taking the box.

I: So, since you carried the box to work, how was it.were there any people who asked you about it, when it beeps and so forth , may you explain to me how the situation was ,was it a situation where everyone saw the box?

I: No one was present ,because I kept the box in my classroom, I was working in a pre-school, so I kept it in my classroom, no one comes into my classroom, I was always alone. Everyone ends up at the dining hall (Cough) unless after 7 cause children all gather at the dining hall. Then after 9 that’s when they go to class, so I left it in the classroom, I enter there at 7.

I: ok, (Paper sounds) (.) so that means the box didn’t disturb you at work?

P: It didn’t disturb me.

I: (Paper sound) did it happen that you became just a bit worried that *eish*, When xxx (intern’s name) was explaining the box to you and then you took it. Perhaps when you arrived at home *eish* .did you have second thoughts that *eish* this box will cause trouble for just using it, as I was asking you if it didn’t affect you at work. Did you have any worries after taking the box, maybe I will regret taking the box?

P: (Cough) (Laugh) I had some regret at first, because I will wake people up.

I: Yes.

P: (Cough) when they are asleep.

I: Have you ever been worried about the beeping of the reminder that….

P: Yes [Inaudible segment] an alarm is an alarm, one can set it in the phone, so same applies with this one [Inaudible segment].

I: so that means you have a problem with the beeping sound of the reminder, or it’s just alright?

P: Yes.

I: It’s alright, you don’t see any problem with the reminder, which means in Aurum the reminder should always be there like it is.

P: It should always be there. Because they can even call you if it happens that you are outside, they’ll shout and say your box is beeping,

I: oh ok, so even when let’s say you’re outside but people who hear the box can call you and say *ey* your box over here.

P: Yes. They ask what this beeping sound titiitit (Laugh) is even though you won’t mention that it’s my box, but I remember that it’s my box.

I: oh so, just like you mentioned that at home when the box starts beeping everyone can hear it, that means you told everyone at home about the box, they know about it and that you are taking TB pills?

P: They know, I told them that I am taking TB pills.

I:ok, and then so (.) so about the box, when explaining , is a box I came with it from the clink which will help me in taking my pills and this and that.Huh did they have certain questions, maybe even worries and this and that?

P: They asked, how it indicates this and that.

I: ok, I mean that, did they have any complaints, as you’ve mentioned earlier about the reminder .when you said that this box will beep ….

P: (Cough)

I: Sorry.

P: They didn’t have any complaints.

I: It will beep and this and that?

P: Mmm, they didn’t.

I: (.) So have you disclosed about your status at home?

P: I haven’t disclosed to the children, I have only disclosed to my husband, my boyfriend that stays with me.

I: Ok, you have explained to him?

P: Yes, but his at work most of the time, he comes back month end.

I: And have you also explained about the box to him?

P: Mmm.

I: So, when explaining to your partner about the box, on how you got it from the clinic, they said you have TB, and you were given TB pills, how did he interpret everything?

P: He said the box makes noise (Cough) he says it’s making noise.

I: So which means the reminder is disturbing him.

I: So do the children have any problem?

P: (Laugh) so that’s why I have to quickly wake up when it beeps.so that it doesn’t beep too much. That’s why I have kept it near me, so that when it beeps I wake up.

I: ok, so by looking at how your partner is complaining about the reminder, won’t is stir any tension between the two of you, that this and that. Or he is just complaining about the box, and it’s not something that might cause further damage and not getting along between you and your partner?

P: Huh Huh, no it’s not something like that.

I: (Audio paused)

I: (Paper sound) Yes , yes so he is slightly complaining about the reminder ,it’s not something that can further cause tension tomorrow, it’s not something that would cause you to fight tomorrow ,that your box is annoying me this and that?

P: It’s not like that, he is complaining as to why I wake up, when its time because I said the box will beep. And he won’t be able to stop it, I have to be the one opening it and taking my pills

I: ok, ok, but looking at everything Huh, does he see the importance of it and how helpful it is to you, is he seeing that?

P: Yes (Inaudible segment)

I: Or is it just annoying him a bit, but he sees the importance of having the box.

P: He [referring to the husband] can see.

I: ok, ok, that’s better then. So (.) since you have started using the box, have you received what’s this, a Sms reminder?

P: [Inaudible segment] Sms reminder no (.) I have never received it.

I: Ok ok,( Paper sound) so so since you have been using the box ,can you mention maybe any challenges or any diffulties you are facing … let me start by saying are there any difficulties or any challenges you have faced with the box?

P: (.) There aren’t any, besides thinking that the box will make noise.

I: Mmm (Laugh) so infect the reminder?

P: Yes, it [referring to the box] would awaken people, and make noise when they are asleep.

I: So, there is this thing called the the Differentiatiated care model, the Differentiated care model means that Huh, You’ll receive a phone call Huh, from xxx (Intern’s name), if maybe xxx (Intern’s name) see’s …you explained to me that once you didn’t drink (referring to medication) they can see. And you mentioned that they remind you, there is a reminder. And then I asked how you are reminded. So here the Differentiated care model entails getting phone calls when they see that Huh, you didn’t drink, maybe for a day or days, have you received a phone call so far?

P: Yes, I have received a phone call Huh, *mxm* this box wasn’t functioning, it wasn’t recording, and I don’t know what was wrong with it. So sis xxx (intern’s name) called to find out what is happening with the box, and she (referring to the intern) brought a new one (referring to the box). But I was drinking my pills. It’s just that it (referring to the box) was not working, so it appeared as if –

I: You didn’t drink (referring to the medication) and you drank it, and that caused xxx (Intern’s name to call you?

P: Mmm.

I: Ok so, do you still have that problem, do you have it, or was it solved?

P: It was solved.

I: Ok so, the time you received a call from xxx (Intern’s name) or if it happens in future that you receive a call from xxx ,how do you feel about that, how do you feel by getting calls that *eish* you haven’t drank these things (referring to medication) how does that make you feel?

P: I was surprised as to why they (referring to Health workers) are calling me, I was surprised. Why are they calling me because I am from the clinic ,and I do take my pills, sometimes I even question myself and say what do they want now (Laugh)

I: (Laugh) and say *eish*, I am now regretting it seems as if they (referring to the health worker’s) are causing trouble for me. They are *tiding* me, they even call when I am in my own private places.

I: Ok

P: (Cough)

I: But, as time passes by, getting these calls …because you’ve explained to me before that your understanding of receiving calls means you are being supported it’s a reminder.so if you’ll be getting calls as time goes by. Will getting phone calls be a problem?

P: Huh, it wouldn’t be a problem.

I: Mmm ok, why if I may ask, why wouldn’t it be a problem?

P: Yes, sometimes they (referring to Health care workers) are encouraging you, they care. When they were calling me .I realised that these people care for my life. They say I didn’t drink (referring to medication) even though I did drink .which means there are those (referring to patients) who don’t drink and they follow-up on them.

I: This means that you are receiving this as a form of support, there is someone who is supporting me.

P: Yes, there is someone supporting you, they (referring to Health care workers) are not working to say that it’s your life even if you die alone.

I: So, how does that make you feel, that there is someone who is not related to you but they (referring to Health workers) are supporting you in this manner. How do you feel about that?

P: I feel good, I am okay that there are people who still support you regardless of you not being related to them. I do get support TB is not a disease that can kill you, but it’s curable when you take your treatment.

I: Ok so, you’ve explained to me that there is a reminder of the box which helps you, its reminds on time Huh, that at this specific time I should take my pills, so when explaining all this, how is the phone call helping you or how happy are you with the phone call, the box reminder and everything, how happy are you with all these things?

P: I am very happy with everything, even the reminder?

I: ( Paper sound) So regarding the Huh box right Huh,just like you have explained how it works, how the lights work Huh you mentioned the reminder is the one reminding you when its time. So is there anything maybe Huh that that what can I say. Maybe that can be added to the box so it could work better than it is currently functioning.is there anything else?

P: I think you could add a light inside for when you drink (referring to medication) at night and there is no electricity.

I:Ok, you mentioned that you are staying with your children right, and then your partner comes back sometimes.so your children- when you came to the clinic and you were diagnosed with TB, then you started treatment ,so is there a chance that your children or what can I say. Were they tested for TB?

P: Yes they tested.

I: Ok yes, were they tested here or some other clinic?

P: They tested here at the clinic.

I: So it turned out that they are negative?

P: Yes.

I: So you spoke about Huh about pills that the pills Huh got wet?

P: I said before I received the box, so I kept them in my inside my bag.

I: How did they get wet, did it rain on you?

P: Juice spilled on them.

I: Mmm, inside the bag, they got wet, then those … what did you do with them?

P: I got rid of them.

I: Oh, you never drank them, so, that means there was a day that you missed because the pills had gotten wet?

P: Mmm.

I: Ok, so did this incident occur once?

P: Yes it occurred once.

I: So, that means Huh currently the pills are safe, because you have a box if I may ask so?

P: Yes, they are alright and safe.

I: Than before, ok ok.

I: Huh, since you’ve started with TB treatment, sometimes there are home visits that are done, where there’ll be people from xxx (clinic name) visiting your home to check-up on you, have you had any home visit so far?

P: I have gotten it (Cough).

I: Ok ok,Counseling,after being told about your status that ok, you have TB and you should take TB treatment , and then counselling takes place, and then how was your experience, can you mention how was it, how satisfied were you.by the manner in which you were counselled ,did they explain about TB , treatment and everything?

P: Yes, I was satisfied, they (referring to health workers) explained in a meaningful manner.

I: Who explained to you, was it the Sister?

P: Yes, it was the Sister.

I: Ok ok (.) ok Huh, so in your opinion right, since you’re using the box, is there a (Alarm ) way that we can improve our box looking at it, is there anything we can improve overall or ?

P: I mentioned that if you could install a light for people who drink (referring to medication) in the evening and there is no electricity.

I: A light?

P: Yes, oh a globe.

I: Oh ok, a light inside the box when you open it?

P: Yes, for those who drink at night, because I drink in the morning and there’s light, some maybe drinking at night, most of the time there is no electricity only to find out that its load shedding.

I: Loashedding, That’s a beautiful suggestion, No its better that in the recorder I won’t forget anything. We will…, it’s a good suggestion it makes me happy. We will take that and always have in our thoughts , that the box should have a light , because seriously apart from drinking at night there is load shedding whenever someone needs to drink.so at least when there is a light it would be helpful wow.(Laugh) .ok ok, thank you thank you.

I: Huh so you mentioned that xxx (intern’s name) gave you the box, so do you prefer xxx (Intern’s name) to be the one giving out the box Huh (.)

P: (Cough)

I: Sorry.

I: What can I say, how can I put this, (.) Did you find xxx (Intern’s name) as the rightful person to explain to you about the box or Huh plus there is a Sister at the TB room or do you think the Sister at the TB room can also explain and give you the box, do you think the sister should be the rightful person, or xxx (Intern’s name) was alright and she explained in a meaningful manner and everything was okay?

P: Mmm xxx (Intern’s name) explained in a meaningful manner.

I: ok ok ,(Paper sounds) so we have spoken and you have explained a lot including the light Huh,is there perhaps anything you would like to leave us with, maybe … especially the ..do you remember that I explained about the differentiated care model, that the differentiated care model is when you receive phone calls, Sms reminder, if you did receive a reminder as part of the afore mentioned, in your opinion how did you feel about that, what can I say Huh, is it okay for them to be there or there should be a phone call, Sms reminder or a Home visit?

P: Phone calls and Sms’s are okay, (Laugh) Home visits-

I: What about home visits?

P: They don’t make one feel comfortable, because they attract noisy people.

I: Home visits bring unnecessary attention.

I: (Paper sound) Huh may you please elaborate on that so that I could understand or for someone else to understand.

P: People want to know why these people are here, who are they and what are they doing here.

I: Ok, who is asking you to explain this situation?

P: Neighbours.

I: Mmm ok, (.) so that makes you, what can I say, you were saying that there should be a phone call, Sms reminder in your opinion, those are fine as they are. But do you feel as though home visits are causing trouble for you?

P: It should be there but not monthly, maybe once in a while, not every month or now and then.

I: Ok, there should be there, people should get home visits but not every month, ok, because in your own opinion that will bring attention from people.

P: Yes,

I: So you’re explaining that home visits will cause people to ask why are these people here and this and that, and in turn you have to explain and you’re not comfortable explaining to people?

P: Some sicknesses are confidential, I can’t open up and say-

I: And explain to a stranger?

P: Even family.

I: Oh, it’s hard for you, ok Huh there is this thing called … as you have mentioned that xxx (Intern’s name) told you that they can see when you haven’t drank or they can see?

P: (Cough) Mmm.

I: So there is something called the Adherence calendar, that’s the one that allows them to see that today you drank or you didn’t drink tomorrow or you won’t drink tomorrow, did xxx (Intern’s name) show you this Adherence calendar?

P: Yes, she showed me.

I: Ok, where did she show you if I may ask?

P: On her Tablet.

I: Can you explain the adherence calendar, perhaps how does it look like?

P: It has shapes, shadings showing you how you’ve been drinking on past days, this container had a problem so it appeared as if I wasn’t drinking.

I: (Paper Sound) Ok so, so how does it look like where you didn’t drink?

P: It’s like... its light, I don’t remember if I saw properly, but there is a difference, this box wasn’t working not that I wasn’t drinking.

I: Ok so, so at that time the Adherence calendar appeared as if you’re not drinking and you were drinking?

P: It was the time when the box was not functioning, so maybe it appeared as if I wasn’t drinking.

I: Oh, I remember you saying something like that, when the time your box wasn’t working properly and then they said you didn’t drink when you actually drank. And I did ask if you are still encountering that problem and you said it was solved.so now the box is working fine. And your adherence calendar is good now?

P: It’s good Mmm.

I: Ok ok, Mmm, so may I just make an example that you’re taking other pills apart from the TB pills, or perhaps someone else is taking pills Huh apart from the TB ones, you take more than one medication right, so for that person or you, then you take the other medication, would it make you happy if this TB box can be used along with other medication?

P: Yes, that would make me happy.

I: Ok so you think it might do the same job?

P: It would do the same job.

I: Because its function is to remind, as you mentioned that this box is a reminder, its function for the other medication would be to do same job.

I: (Paper sounds) (.) So you mentioned that you only told your partner about your status apart from the children, so besides your partner, can I say maybe a friend, a close neighbour, family such as you’re Aunt or Grandmother, did you disclose to them that ok, I was diagnosed with TB, or it’s just only your partner?

P: I told the family? They are in xxxx (name of another province) , so so far I haven’t visited them.

I: So when disclosing to them, how did they react to that. You went to the clinic, and it was discovered that you have TB and you also have to take TB pills. How did they react when you told them?

P: They said I discovered it soon, because I also didn’t know when I found out, its better that I discovered it early. Others discover it at later stage when they are sick. And they asked me how many months the treatment is for? It is curable as long as you take treatment, you’ll be alright.

I: Ok so, that means they don’t have a problem, they are delighted that it was discovered early.

I: So ,may I ask about the support ,how much support are they giving you since you’ve started taking TB medication ,but you did mention that it’s been a while since you have visited them, ok but does it happen that you communicate with them?

P: Yes, we do communicate by phone, they ask if I am taking my pills.

I; So if may I ask specifically about the family who these people are at least two, maybe your Mum, Aunty or cousin?

P: My mum and sister.

I: Mum and your sister?

P: Yes my eldest sister.

I: So, which means they are supporting you then when they ask and support you how does that you make you?

P: I feel comfortable, I feel good and hopeful.

I: Ok, now that at home they know about you taking TB pills and using the box, will you still be able to use the box when you’re at home or there’ll be a problem?

P: Mmm.

I: You are saying that you can be able to use the box at home, ok ok.

P: (Paper sounds) so in the house you said you haven’t disclosed to the children, but they can see the box and inform you that the box is beeping?

I: Yes.

I: (.) Ok Huh, we have come to the end of our interview, we think that we have discussed everything, you gave us a lot of information that we needed, huh thank you very much, thank you for your time, thank you for agreeing and giving us your time. This information you have given us will help us a lot and will help other people. Especially with the box our aim at the end is to help people who are deserving of it, so that they can accept it as a part of their lives. Thank you for agreeing and giving us your time. Thank you very much, Huh before we close, here on the Informed consent form at the back, I did show you in case you have questions or you want to know more about the study and the interview, because it might happen that you be a part of an interview and then you start having thoughts, you understand, even if there are questions you are having. You may call them , I did explain that xxx ( Head of the Study’s name) is the senior who is above us, she is the Head of the study , and this study was approved by Human Research ethics in WITS , we also have their phone numbers ,there is also xxx ( Head of the study’s name) and her email address, so you can also call or email her, because I did mention that the study is legit by Human research of ethics right, so you can you can call these people or communicate if it happens that you have questions. Ok Huh, the time the interview ended is 12:56
