# Supplementary material for: Acceptability of Digital Adherence Technologies to support people with drug-susceptible TB in South Africa
Source: PLoS One. 2025 Sep 24;20(9):e0332103. doi: 10.1371/journal.pone.0332103 (PMC12459780; doi:10.1371/journal.pone.0332103)
Supplement: S4 File — (ZIP) [file pone.0332103.s004.zip › S4 Transcripts/PwTB/IDI 27_PwTB.docx]

**I**: We thank you ma’am for allowing us to conduct this interview with you. So, do you allow me to record you?

**P**: Yes.

**I**: Thank you. Date, xxxx (interview date). Location; xxxx (clinic name). Language used, Setswana. Pid number xxx, start time… 10:54, Okay [Sigh]. So, who do you stay with?

**P**: Pardon.

**I**: Who do you stay with at home?

**P**: [Sigh] With the husband and the kids.

**I**: Do you stay far from the clinic?

**P**: Pardon.

**I**: How far do you stay from the clinic?

**P**: In xxx (town name). We stay in (town name).

**I**: So, you travel from xxxx (town name) to the clinic?

**P**: Mmm.

**I**: So, how much does it cost you to come here for medication?

**P**: At the moment they have increased the taxi fare, we used to pay R50, so now it’s R60 for transport, single trip.

**I**: Can I ask, why did you choose this facility?

**P**: Well, I was diagnosed at xxx (hospital name) . Then for me to come to the clinic, I used the facility before during my pregnancy and their services were very good. So, I had no reason to go to another facility.

**I**: Okay.

**P**: Mmm.

**I**: So, [pause] who explained to you about the box?

**P**: It’s [sigh] xxx (intern’s name). She’s the one who explained that she’s going to give me a box for my TB treatment. Yes, she gave me a form which I read and signed as I understood. Then she gave me the box, which I used when I got home.

**I**: The process of giving you the form and giving you the box took how long?

**P**: [pause] I’m not sure, maybe about 30 minutes. 30 to 45 minutes because there were things, I did not understand which I asked, just like what if the box does not open, things like that.

**I**: So, what do you know about the box?

**P**: That it helps you take your treatment on time, it helps you when you have missed a dose, there is a message you receive that reminds you to take your medication. Even when I know I missed a dose, she can also see on her side that I missed a dose then she can call me to remind me not to forget to drink my medication. So, it is handy. You put your pills inside and you put it where you know.

**I**: So, you mentioned that it reminds you to drink your medication.

**P**: Yes, it has an alarm. When we spoke about the box with xxx (intern’s name) she asked what time do I prefer to drink my medication. I told her and she said every time when it’s the time I specified to her the alarm will go off and once the alarm rings I should know It’s time to drink medication.

**I**: Mmm.

**P**: Mmm.

**I**: You mentioned that it is handy.

**P**: Yes.

**I**: You mentioned that it reminds you to take your medication. How does it remind you?

**P**: Like you’re your medication is never scattered because you put them in there, close it and put it away. Then it is also safe for kids. When it is closed, and you put it up then the kids would not reach it easily.

**I**: [inaudible segment].

**P**: Well, me… I was… what can I say. There is excitement. You become happy for that thing; you will get excited that there is something that will wake me up to drink my medication every day. Meaning I would not forget to drink my medication, it was all excitement. Then they will keep calling you to remind you not to forget to take your medication.

**I**: How was your experience with using the box?

**P**: Okay… first month I had no problem with the box. I used it correctly for the first month, every day when it’s time for my medication it would wake me up and I would take my medication. Not that it wakes me up, but it just reminds me it’s time for medication. Then after 4 weeks I took the box with me to the clinic to get another treatment and the sister that helped me asked why are my pills untidy (I cut them into 5s). Then I explained to her that I cut them into 5s because it becomes easy for me, because I just take out 5 tablets every day. Then she told me that my method can work, she advised other patients to do the same way as I did. Like it’s easy to pack them into 5s, then I had no problem for the first month.

**I**: Mmm.

**I**: Then going forward?

**P**: After the first month the box would remind me about my treatment, just like it used to do. Then during the day, a message would say don’t forget to drink your treatment before 12 o’clock midnight. Alright, then I say I cannot take two doses of 4 in 12 hours. That is an overdose. Then I did not drink them and concluded that it is a mistake that happened there. After two days it started again, “don’t forget to drink your medication at 12.”

**I**: Mmm.

P: Now all of a sudden it would tell me that I missed two doses [sigh] and that whoever will contact you. But that time I took my medication, right. So, I spoke to xxx (intern’s name) . I went to back to her and said man this box of yours wants to kill me, now I don’t understand whether it’s the box or the people at the clinic, who want to kill me. Your box says I must drink medication twice in one day, well, I won’t. Then I said to her sister can I bring back the box, because it is giving me problems. Then she said I should bring it. I took it back to the clinic because it was giving me problems. I drink my medication, but it says I didn’t. But the funny thing is the alarm would not ring twice. No, it would ring once in the morning at the time I know it would ring. Midday the alarm of the box would not ring, then how do you say you missed a dose?

**I**: So, when you spoke to xxx (intern’s name), did you call her or she’s the one who called?

**P**: I called her. I was the one experiencing a problem, so I called her and asked her if it is possible for us to meet on that day, then she said there is no problem. It was already knockoff time, but I had to talk to her. Then we met and I explained to her that I had a problem with the box, and that the box did that and that. Even at some stage my sibling once clapped it, took it, and threw it on top of the table, because it would ring at night, at times it rang at night while the child is sleeping [making ringing sounds]. Then my sibling took it and opened it and threw it in the dining room. The child is now awake, so we kicked that box.

I: So, how much did it ring? How much did it ring?

[Pause.

**P**: I’m not sure; it took three days, right. I think it took three days ringing. Then I told her [sibling] not to close it. When I’m at [pause], like when I’m in xxxx (town name) right-

**I**: -Mmm.

P: And she would be left with the kids at home, maybe I’m at her place, when I’m there I would take out medication and drink it in the morning, then in the afternoon I’m at my place, then I return to her place when I go to sleep. Before I get to the house, she would tell me that your box has been ringing. I would say open it, don’t close it, keep it open and leave it because it was irritating, and I could not see anywhere if it had an on and off button. Hence, I told the sister that can she switch off the box, because now I could not understand if it had a system or what. “Switch off the box because it wakes the child up at night”. She should switch it off.

**I**: Mmm.

**P**: Mmm.

**I**: Where did you meet with her [intern]?

**P**: Where she stays. Like she told me where she stays then I went to her and explained that I’m having a problem.

**I**: Mmm.

P: Like I felt I could not leave it for another day because I stay with the problem, you see. So, I stay with a box that irritates me. She had to meet with me even if it was not time for her to work. So, I would fetch her where she stays then she explained to me, and I understood her. Then I told her I’m bringing back the box. Then she told me it’s fine I should bring it back. She will keep contact with her colleagues.

**I**: Mmm. So, when you leave it open like this in order for it not to irritate you when do you close it?

**P**: You know I’m not sure what time does it close. I think maybe during the day or the next day or at night once it quiet it would close. In the morning at 5am it would ring. Actually, when you have closed it, like at night, when it rings around 8pm and let’s say around 10pm it closes, it will ring tomorrow. At its time it rings, so it would inconvenience me in that it now would not say you have now missed a dose, instead it would ring twice. Then I would tell her to open it. Then we open it and close it. In the morning at 5am its alarm would ring again.

**I**: Mmm.

**I**: So, what are your worries about the box, that worries you too much about the box.

**P**: [sigh] You know when you [pause], Let me say it’s not [pause], It’s not safe. It’s not safe because you do not understand whether you supposed to drink two doses in one day or not.

**I**: When you do not understand what?

**P**: When you don’t understand what the alarm is doing [pause] let me say when you don’t understand when should it send a message if you missed a dose. You might end up thinking that these people are saying I must drink the medication again.

**I**: Mmm.

P: You understand. And then it is the same as if you don’t know who to ask or where xxx (intern) stays or when you don’t have your health care workers’ contact details, such as your doctor, to contact them when you have a problem and what you should do. So, you end up drinking the medication twice, which is an overdose, you see. And then another thing is stress. It’ stressful, like it stresses you individually to drink medication [pause] cause now having to drink it again is stressing.

[Pause]

**I**: So, you talk about [pause], you talk about your sibling-

**P**: -Yes.

I: So, are there people you have spoken to know about the box?

**P**: Like my sibling. When I got to the house with the box, I explained to her and my husband that I was told that wherever I go I should take the box with as I put my medication inside. Then I explained [inaudible segment] I even gave my sibling that first form so that she reads, you see.

**I**: Mmm.

P: They are my junior supporters right, I explained to them then they knew that even when the alarm rings and I’m maybe outside then it’s time for that woman to drink her medication, then they would call me and say, “the alarm, it’s time, come drink the medication”. So, she knew.

**I**: So, at first when you brought it home what was their perception of it?

[Pause]

**P**: It is a good thing.

**I**: Mmm.

P: It was a good thing, cause like I forget so it would be them reminding me it’s time to drink the medication. So, it was good because at least they would remind me, otherwise if they didn’t, there was someone who would remind me, the third person, which is the box, that it is time for the medication.

**I**: So, besides the sibling and the husband, is there anyone who saw you holding the box?

**P**: Yes, everyone at home. Like I would tell them when I enter. I would tell them. Everyone at home knew [laugh]-

**I**: -Mmm.

**I**: So how was their perception?

**P**: It was good. It was a good thing, you see. Hence, I would say to them this thing is surprising me because it worked correctly for a while, so I told them maybe they must take these boxes back to where they were made, you see. Because maybe something happened within the short time, maybe a factory fault. They must take the boxes back. It’s unfortunate that I finished my treatment in August. I would say they must use the box [laugh], I would put my ARVs inside it [laugh].

[Sigh]

**I**: Besides family, is there anyone else, maybe a friend or any other person who saw you holding the box?

**P**: Mmm my colleagues. I was going… I was sleeping at home having to go back to my place after work, so remember everywhere I go I take the box with, so I went with it to work.

**I**: Okay.

P: I even showed some of the Doctors, who were Dr xxx (doctor’s name), Dr xxx (doctor’s name) [inaudible segment] are you going to eat from this box? Then I told them “no, it’s my medication box”. So, they were like wow! This is good.

**I**: Mmm.

P: Yes, so even my colleagues saw it.

**I**: So, when you take it with, did you experience any challenges besides those you have mentioned already, when you take the box with?

**P**: No, it’s portable. You just put it in your bag even in a handbag it fits. You put it in and close the handbag, it’s portable. Really handy, really, handy, and really helpful. Cause even when you pack, it’s not like it’s something that is too big, it’s portable for you to place it anywhere.

**I**: Mmm.

P:But you know what my fear was? When I’m in the taxi and the alarm goes off [laugh].

**I**: Was it fear of the alarm ringing while you in the taxi?

**P**: The alarm goes off while I’m in the taxi [inaudible segment] I wonder what I would do. I would open it and apologize and tell them it’s my treatment box, it’s out of time [laugh].

**I**: Would you feel comfortable to tell them in the taxi that what’s ringing is your medication box?

**P**: Yes, I don’t have a problem. Why not? You know by so doing, if it happened, I have realised that there are plenty of people who do not know about the box, because many who see me holding the box become surprised. They would be surprised and go like TB treatment box, you understand. Then I could explain to them that it’s my TB treatment box. It helps you take medication on time; it also helps you not to default.

[Pause].

**I**: Did you disclose to people about your TB disease?

**P**: Mmm.

**I**: Was it easy?

**P**: Mmm. I didn’t have a problem. Like, at home [pause] the day I got diagnosed and also the time I had flu, flu like symptoms. The doctor tested me, they tested me using [inaudible segment] and tested positive. Then he immediately told me I’m going to start my treatment, there is no way I am going home. Then he told me that I’m going to bring in my immediate family so that they can also-

**I**: -Mmm.

P: They [family members] then tested negative. After I tested positive, I called my husband and told him that I tested positive for TB, then [pause] then he asked where did I get TB from. No, TB is airborne, I could have contracted it from Choppies or Shoprite, or wherever. But because we never in one place, we are all over, you go to the shops on weekends [inaudible segment] although we wear masks… It’s not like we wear them 100%, sometimes you take the mask out because you sneeze, and when you sneeze you also want to blow your nose. In the moment you don’t know what could happen.

**I**: Mmm.

P: Then at home I called them, my aunt, my uncle’s wife… “hey guys I tested for TB and, I’m positive.” It was the same as when I was diagnosed with COVID, I told all of them. By so doing you also helping them to go get themselves tested, so that it’s not… It’s selfishness at times not to tell people, you see. So, you help them by telling them so that if you were with them during that period, they should get tested. So, it was easy for me. I’ve got a supportive family anyway. Mmm, that is why it was not difficult.

**I**: So, what was their reaction?

[Pause]

**P**: Well, what happened, you know family will ask. They ask you say you have TB, what happened. Then I responded by saying I inhaled it. And they were like xxx (participant’s name) you inhaled TB, so you chose to inhale that TB. And I said I won’t see it guy, I am not able to see TB, but TB is there. So, you also go get tested because I’ve been spending time with you. Maybe when I was visiting you, I was already TB positive but then I did not test and did not know. That’s when they said, “you like being sick” At times things happen.

**I**: Why would they react like that and say you like being sick?

**P**: Like you know once you stay at home… I’m the only one with other sicknesses like sugar diabetes and high blood, no one has those at home, I’m the only one. You understand.

**I**: Mmm [Inaudible segment].

P: No, I was diagnosed with HIV like I think 12 years back, high blood and sugar came afterwards, I’m sure three years back. Then I had COVID with the first episode of COVID in 2019, towards the end of 2019. Then in 2021 I had TB in June, and I was highly pregnant. Was admitted, then discharged, but I was fine though. 20…22 early January I tested positive again for COVID. So even at home when I say guys, I’m sick, they’re like uh you [laugh]. But it’s okay because I always share with them. And they take me serious at home, they take me seriously. When I tell them I’m sick they take me seriously because they know there is no way xxx (participant’s name) would get sick and keep quiet. No, any time I get sick due to anything I tell them, guys I’m sick. They are supportive. Immediately you would see them come to my place and ask what am I saying. Mmm.

**I**: So, in the family do you have any history of TB?

**P**: No, it’s just me.

**I**: So besides to open the box so that it does not irritate you, is there any other reason you open the box if you not taking treatment?

**P**: No there isn’t-

**I**: -Mmm.

P: I was told I only open it when I take my medication and when I come back from the clinic and pack them.

**I**: Mmm.

P: Other than then you don’t open it, you just open when you take your medication and drink.

[Pause]

**I**: So where do you put your box?

**P**: What?

I: Like where do you store your box?

**P**: I put it on top of my dressing table and [inaudible segment]

**I**: Mmm.

I: So, it had a shelf, the top one, it stayed there, where the children can’t reach.

**I**: So inside you used to put your TB medication and which one else?

**P**: Only.

**I**: TB medication only?

**P**: Mmm.

**I**: So, let’s talk, what do you think was very helpful with the box?

**P**: Storage and time. You see I loved storage and time to drink medication. Our medication stays safe in the box…. you will find the sugar diabetes medication there too you understand. So at least storage… good storage and then time to drink medication, there was no way I could miss it. It helped me a lot with time to drink medication and storage.

**I**: Mmm.

**P**: Mmm.

**I**: So, you took your whole medication at the same time?

**P**: No, uh [pause] the doctor explained that now you taking TB medication, tablets have contraindication right, so, don’t take medication at the same time, when you have taken you TB treatment in the morning and take your ARVs at night, then you gave that chance to drink the high blood at night at a certain time, don’t drink them at the same time. And then there was this other drug which my doctor gave me; prescribed for me. He said it helps so that the tablets you take don’t clash in your body. That drug is helping because I have never had problems due to drinking too many tablets. I drink the drug only in the morning, I just forgot it name, I just drink it in the morning, then after 2 hours or 3 hours I drink my high blood and sugar diabetes medication and then at night I drink these.

**I**: So, the other medication? Cause you said the box reminded you, so what reminded you to drink the others?

**P**: I’m already used to, right. So, I don’t just forget. These TB one’s were just an addition. Yes, these I’m now used to, that I have to take high blood every day, so then TB I used to drink before 9 so that I gave the others time. Then these I drink at 9pm so I’m used to the schedule of drinking my medication. So, the addition of the TB medication, he told me, my doctor told me to start with the TB medication in the morning because you take them earlier, these you drink once you at work so start with these. Then I start with them.

**I**: So, what can you say what is your experience with your TB medication since you have brought back the box?

P: I forget time to drink medication, like I used to drink them at 5am, now without the box I don’t drink them [medication] at 5am, my time fluctuates. You will find me drinking them at 10am, the following day when I have remembered I drink them at 5am, sometimes I forget just like that (Female Participant, 38, Opted Out).

**I**: So, what can they do, something that can be changed on the box so that you can start using it again?

**P**: Uh-huh, I think they must check with the manufacturers of the box, check what could be the cause of the box to ring twice, you see.

I: Mmm.

P: Cause that’s the only thing, really, if they could check the box if it does not ring twice, or what could have happened because I realized that it has a machine in front. So, if they could check that thing, I think we could use it again and a lot of people could use it because it was handy.

**I**: When you say its handy…

**P**: So that people don’t default, like the TB default rate is very high, so for people not to default they need the box to remind them, so that they take their medication. Because you know I once… let me tell you what I once did, an experiment, right. I once said I want to see when this box rings, then I open it and not take out any medication what will it do.

**I**: Mmm.

**P**: I then opened it when it rang, I just opened and closed, I didn’t take out the medication and you know the box kept ringing.

**I**: It rang?

**P**: Yes.

**I**: After how long?

**P**: Immediately when I closed it, I closed it, when you close you tap it then I tapped. The alarm rang again. Then I laughed and asked myself how does this box know that I didn’t take out medication, so it’s quite nice to experiment a lot of things, so when I opened it for the second time and took out 5 of my tablets and closed it then it did not ring.

**I**: [laugh] okay. [Pause] So, what do you like most about the box?

**P**: Punctuality of drinking medication, cause remember medication becomes more effective when you drink it at the same time, like 12 hourly. Without jumping and making it today 12 hourly and tomorrow 14 hourly.

**I**: Mmm [pause]. So, is there anything that you feel was not added when they explained to you which you think they need to tell people about the box?

**P**: Not really. I feel like the lady who explained to me everything.

**I**: Would you recommend it for people who are on TB medication?

**P**: Yes

[Pause]

**I**: Okay.

[Pause]

**I**: Alright.

[Pause]

**I**: So, let’s talk about the SMSs, phone calls and home visits that go along with the box. How do you feel about the SMSs?

**P**: Uh-huh, to be honest they were really annoying me because I know that I have to drink my medication, why should they send an SMS? So, they were annoying cause I drank my medication. Why should they send me a message? I would snap at work and say no, you see now this box is crazy. My colleague would ask what’s wrong now xxx (participant’s name), and I would say that they say I did not drink my medication and they would say you have defaulted, and I say no I did not default, I drank my medication in the morning but see the message I just received, it says I did not drink my medication. So those SMSs really annoyed me. But for someone who does not drink their medication, it is good because it’s one of the support systems, even if someone does not visit you at home, the SMS is a support system because it alarms you that you did not take your medication, make sure before this time you drink your medication, so it’s a way of supporting you as well.

**I**: Mmm. So, have you received a phone call telling you that you have not taken your medication?

**P**: Yes.

**I**: How did you feel about that?

**P**: I was shouting, I was shouting and said I will give you 5 tablets from mine so that you drink them cause I drank mine, then she said, explained to me that she’s the one who knows that you did not drink your medication. And I was like how did she know… how did she know that I did not drink my medication, then I thought these things are advanced, probably it’s linked to a PC or something which can flick and report if you have missed a dose, that is why she can call. Then I explained to that person that I drank my medication, but the message says I didn’t. I drank them. [Inaudible segment]. I don’t know she says she looks at the screen that you didn’t drink medication, immediately oh yes, I suspected, immediately she called “Ma’am how are you, you speaking to so and so from...” yes, sister xxx (intern’s names). Uh I see that you did not drink your medication [inaudible segment] and told her to give her 4 tablets to drink because I drank mine.

**I**: Mmm.

**P**: Mmm.

**I**: So, let’s say it was not faulty, cause this one unfortunately it was for faulty reasons, if it was not faulty maybe the person did not take the medication, what can you say about the SMS and phone call?

**P**: It’s going to help them, it’s helpful because remember the treatment supporters at the moment cannot cover everybody. But with this system of this box that when you missed a dose you get a call or SMS cause other people really forget, they forget that they didn’t take their medication, so it’s helpful. Like if I could be given a box that is not faulty and go test it again, I would take it. Because this one, I honestly told her that there is something called factory faulty. Her box which was 50 for that day, you will be surprised that all 50 people are going to bring back those boxes but if I could get a new box, go try it. I’m going to try it.

**I**: Mmm.

**P**: Mmm.

[Pause]

**I**: So, about the home visit, how would you feel when they come to your house and tell you that you are not drinking your medication?

**P**: Uh-huh. I’m very welcoming. I would welcome them when they come visit me and tell me I have not taken my medication. And the best way is to explain why if I didn’t take them and, in my case, I would tell them their box is not telling the truth.

**I**: Out of these three, home visit, phone calls and SMS, what don’t you like about them?

**P**: [pause] [inaudible segment] have you noticed when you busy with something, either at home or at work, you get an SMS you attend to it. Only to find out it’s something that you’ve already done. Its irritating [pause] cause really you left something that you’ve been doing for an unnecessary message.

**I**: So, have you experienced any challenges, barriers that make it difficult for you to make use of the box. Like religious, spiritually that make it difficult to use the box.

**P**: Uh [inaudible segment] but I for one I’m an apostolic, so at church…Oh yes, I have experienced. Then they would say we going to the water, and we have to fast *neh*, I had a serious challenge because remember this medication starves you, *iyoh*! So, I did not eat, and I drank my medication, disaster. So that was my challenge, I cannot fast while I’m drinking TB treatment. So, my Pastors wife did not want to understand that. She would say no xxx (participant’s name) everybody is taking medication, and I would say, yes everybody is taking medication, but are they the same as mine? Because the one’s I drink they tell me I must eat breakfast then drink my medication then eat again and even snack, they don’t give me a chance, they want me to eat. I once took a chance and drank them; I ate before but did not eat for me to be full. You know I was vomiting the entire road. Not because of anything but because I was hungry. I was stopping the car the entire road, asking the driver to stop so that I can vomit.

**I**: Mmm.

**P**: Mmm.

**I**: So how did you overcome that challenge?

**P**: The church one?

**I**: Yes.

**P**: I didn’t fast, but I was praying.

**I**: Mmm.

**P**: Yes.

**I**: Okay.

[Pause]

**I**: So, in that case what can you say the box helped you to which extent?

**P**: Actually 90% it helped, and then I would recommend it to other people to use it because… when you use it if it’s in the right state, without any faults it becomes easy for one to use it. Its clever and helpful. It will help even with the default rate, maybe it would help the default rate to decrease.

**I**: Mmm.

**P**: Mmm.

**I**: So, what don’t you like about the box, not counting the things it did regarding the faulty mixed doses issues?

**P**: There is nothing I don’t like –

I: -Mmm.

P: I’m just okay with the box, it’s portable, so, and it’s nice you know.

**I**: So, what was their explanation regarding how the box works, if I could explain to someone who starts to take TB medication, how could I explain to them regarding how the box works?

**P**: Okay, if it’s a new person starting to take TB treatment, you first start to show them the challenges they might encounter if they don’t have the box, right. Then explain to them, here is the box and how it works, then give them a chance to tell them to tell you where they don’t understand, so that you can in service them, in order for them to know more about this box. So, you basically explain to them that when you have this box it’s going to be easy for you to take your treatment on time and it will be easy for you to recover well, because you will be taking your treatment at the same time, not skipping days, and not skipping time, you will be drinking it at the same time, every day. So, you just enlighten them about the importance of not skipping medication and drinking them at the same time.

**I**: You mentioned the challenges you might encounter if you take medication without the box, could you explain in detail what do you actually mean.

**P**: What?

**I**: You mentioned the challenges you might encounter if you take medication without the box, could you explain in detail.

**P**: When you drink them at the same time, actually the box rings at the same time, so if you… you know it’s the same as what, the same as when you take your high blood medication. When you take your high blood medication you don’t drink it at the same time, they always say “I just remembered” that time it’s 2 o’clock in the afternoon and they say they just remembered to drink their high blood medication, you see.

**I**: Mmm.

P: At times they come to the clinic and when they check their BP is high due to the fact that they have not yet taken their medication. So, TB medication… I think it’s wise if we treat them like ARV’s, remember ARV’s are taken on time. The same applies to that of TB and to all of them in fact, when you drink them at the same time it becomes easy, your recovery becomes easy.

I: Mmm have you ever missed a dose since you started not to make use of the box?

**P**: No, I haven’t missed it.

[Pause]

**I**: What do you think we can improve about the box?

**P**: [Pause] I feel like they should be double checked, the box itself is here but the programming of the box, I think it should be checked thoroughly because for those who cannot read, for those who do not have information and they are there out here. It’s going to be a problem because I didn’t go to school the next thing a message comes through saying that dad didn’t drink his medication, are you going to give him medication again? You see, so it’s really going to be a serious problem for those who do not understand. So, if the system of the box could be improved, it could be way much better. If they could double check if it’s working correctly, so that if they give it to the next person, they won’t experience challenges.

[Pause]

**I**: Okay ma’am.

[Pause]

**I**: So, who has to teach people about… who can be comfortable to teach people about the box?

**P**: [inaudible segment] I think anyone who has experience about the box, even… like xxx (intern’s name) she’s trained, right, to teach about the box, even her. But even a person who has experience about the box, who has used it, I think I could be comfortable to teach people because you would be talking about something that you know, something that you used before. Yes.

**I**: Okay.

**I**: I think we are reaching the end of the interview. So, if we could do a comparison of the satisfactions with the time you were taking the medication now and when you were taking the medication with the box, what could you say about it?

**P**: Uh… When I had the box, I would take my medication on time 100 percent, but now I know at times I do not take it at the exact time because sometimes I’m late, when I check it’s 10am and I should have taken my medication at 5am.

[Pause]

**I**: Is there anything that you feel like I did not ask about box that you would like to talk about? The box, SMS, phone call [inaudible segment]

**P**: No, not really, we have covered everything.

**I**: Mmm.

**I**: Okay, so we have reached the end of the interview, we appreciate your time, if you have any questions that you would like to ask further feel free.

**P**: Okay, I don’t have questions, I’m okay.

**I**: Ending time 11:42. Thank you.
