# Supplementary material for: Acceptability of Digital Adherence Technologies to support people with drug-susceptible TB in South Africa
Source: PLoS One. 2025 Sep 24;20(9):e0332103. doi: 10.1371/journal.pone.0332103 (PMC12459780; doi:10.1371/journal.pone.0332103)
Supplement: S4 File — (ZIP) [file pone.0332103.s004.zip › S4 Transcripts/PwTB/IDI 28_PwTB.docx]

**TRANSCRIPTION NOTATIONS**

| **Label Key** | **Meaning** |
| --- | --- |
| **I** | Start of each new utterance by the Interviewer |
| **P** | Start of each new utterance by the Participant |
| **N** | Note taker |
| **{ }** | Indicates that details were what are or pseudonyms were used to anonymise data |
| **( )** | Indicates the description provided to anonymise data |
| **XXX** | Words were omitted to anonymise data |
| **-** | Breaking into a sentence by the next speaker |
| **…** | Pause or drawn out words |
| **[ ]** | Indicates noise made, e.g. [laugh], [sigh], [pause] |
| ? | Beginning of utterance by unidentified speaker or questionable text |
| **[inaudible segment]** | Unclear section of the recording |

I: So, mama umm, do we have your permission to record you in this interview?

P: Yes.

I: Thank you for giving us the permission. So date of this interview, it’s xxxx (interview date), the Location, it’s xxx (clinic name),uh the language used for the in-depth interview session is English, the PID xxx and the interview is starting at uh, 10:22 AM. (flipping through paper) Ma’am can you tell me a bit about yourself, who do you stay with?

P: I stay with my daughter.

I: You stay with your daughter, it just the two of you. Who else do you stay with?

P: My daughter, husband and… myself in the house.

I: Okay, how many are you?

P: We are three and the daughter in the flat next door.

I: Okay, okay, so tell me when did you find out that you had TB?

P: I went to the hospital; I had lung problems and they said they think I have bronchitis or uh, I can’t remember the other name and then December was in hospital, January was in hospital, March was in hospital. Then Prof said come and see my daughter and I said they won’t let me out of the hospital until they find out what is happening. So, they take me with the sputum test from December, January and March and they find out, they tested and it’s TB.

I: Oh ,so what symptoms were you experiencing?

P: Coughing a lot and can’t breathe and something just pressing on my chest.

I: Chest pains.

P: And I cough a lot non-stop.

I: Okay, okay so tell me how did you get to the clinic today, how did you travel from home to the clinic?

P: Uh my daughter brought me because I can’t breathe, then take me to the doctor to see me. Then they have to take me in back to the hospital.

Alright, so when you were told you had TB, how did you feel?

P: I was shocked [laugh].

I: You were shocked, what made you shocked?

P: Because they all tell me TB is a terrible sickness… and then doctor said to me uh he knows its a shock for me, but for him its best now he knows what to do; how to… treat me.

I: Mmm so what make people say TB is a terrible disease?

P: I just hear from people that are -said it uh, it’s not good to have it.

I: Mmm.

P: It’s a long term of uh mediation.

I: Okay, long medication. Okay, what else do people say about TB?

P: Nothing further, I just hear from people talk about it. I don’t talk to someone who already has it.

I: Okay, okay. Alright (knock at the door] [pause].

I: Okay, so tell me what you know about this box?

P: The box I see to me, they but my tablets in and every time when the sound goes off then it’s time to take my tablets… and that’s good for me because sometimes the older you get you forget. Now when it goes *tee tee tee* [ making the alarm sound] then you know everything you take your tablets.

I: Yes.

P: So, that’s why I think it’s a good idea.

I: Yes , yes. So, who explained to you how to use the box ?

P: Sister xxx (nurse’s name) told me, from the TB clinic.

I: At this clinic? … Sister- okay the sister-

P: -The first time I used it-

I: -it was the TB nurse?

P: Yeah.

I: Okay, how long was the explanation if you can remember?

P: It wasn’t long; she just told me that she was going to give me some medicine.

I: Oh-

P: -She put it in the box, and I had to use it when the buzzer goes off

I: -Mmm-

P: -I had to drink the tablets.

I: So, can you estimate maybe how many minuets?

P: Every 09:00.

I: I mean how many minuets did she take to explain to you about the box?

P: Say about half an hour… tell me how to open-

I: Okay

P: Ys she told me how to open and close it, and I said yes, I understand. I had to drink the tablet nine o’clock uh and she said do not open it, it will tell me, and I’ll send you a massage

.

I: Okay, okay, what else did she tell you about the box?

P: That’s all that she told me.

I: Okay.

P: (Clearing throat).

I: So how did you feel her about the explanation, was it easy to understand?

P: *Ja* (yeah) it was easy.

I: So is there anything you would like to, to change about how you were informed about using the smart pill box?

P: No, I understand clearly what they said to me.

I: Okay.

P: *Ja* (yeah) clearly said.

I: Okay, have you heard about the box from anyone else besides the nurses?

P: No, only from here.

I: From here okay. So, umm when you started using the box, how-for how long had you been taking treatment before you started using the box?

P: Umm I never used it until they find out that I have TB.

I: Okay, so when you started taking TB treatment-

P: -Mmm [ inaudible segment]

I: So was there a time you took TB treatment without the box?

P: Mmm no, when I came to xxx (hospital’s name) they sent me here and when I saw sister here; she explained to me what happened. I got TB and I had to drink these tablets for uh, about six months , but they put it uh stronger-stronger and weaker.

I: Okay.

P: And then they make it stronger and stronger and stronger until September.

I: Okay.

P: So, six months treatment.

I: Okay, alright. So, uh did you start using the box the same time you started treatment?

P: *Ja* (yeah).

I: So, ever since you started using the box, what has been your experience with the box?

P: Uh I think it is very good because it reminds me every time when perhaps I forget to take it.

I: Okay, it reminds you to take it-

P: -It reminds me to take it.

I: Okay, what else do you find good about the box?

P: Uh that the tablets are not somewhere else- I put it away and they are always in the box, and I know when I take the box; medicine is inside.

I: Okay, that’s interesting-

P:-Mmm.

I: And uh do you have any difficulties or challenges you have experienced when using the box?

P: No, it’s fine with me.

I: Okay, you have never experienced any challenges whatsoever.

P: Uh-huh {No} .

I: So what do you do for a living ma’am, do you go to work?

P: I can’t work I’m 83

I: 83?

P: (laughing)

I: So umm-

P: -If someone will give me the job ,then I’ll take the job (laughing).

I: (laughing)

P: But I knit.

I: So, do you go to any places, or do you have any activities where you might need to take your box with?

P: *Ja* (yeah) when I’m playing, jukskei {South African sport} I’m starting Monday and Thursday. I’m starting at quarter to eight then I take the box with me because I know I have to drink my medicine at nine o’clock.

I: Okay, so are you planning to take it with you to other places?

P: *Ja* (yeah).

I: So, what do you think about taking the box with you to places?

P: What experience I have?

I: What do you think you’ll experience when you take the box to that area?

P: I think it’s a good because I got the box with me and I won’t take the medicine out and leave the box because if I do that I’ll forget the tablets in my pocket.

I: Okay-

P: -So now my experience is I take the box and then I know I have to drink my medicine.

I: So that you can take your medication, do you anticipate any challenges with taking your box with?

P: No.

I: You are not-

P: -No problem.

I: You are not anticipating any challenges, okay. So, uh when you were told about the box, what were your worries about having this box?

P: I didn’t worry nothing; I know is for my… uh best.

I: Okay, did anyone ask you about the box ?

P: No one knows cause my daughter knows about the box.

I: Okay… umm how did you feel about having to explain to your daughter about it?

P: No, it’s good because she was with me.

I: Okay, besides your daughter. Who else knows about the box?

P: My granddaughter and son-son in-law.

I: So how did you feel about telling them about the box?

P: I’m feeling good.

I: You felt good, umm what was their reaction when you told them about this box?

P: They said oh that’s a good idea, now you won’t forget to take your tablets at the same time.

I: Okay, okay. Do you have any history of TB in the family?

P: Uh no.

I: No, okay, is there a day you opened the box by mistake, you know, more than once. You said you take you medication at nine o’clock, is there a time- a day you opened it [the box] at 09:00 and opened it at another time on the same day which is not nine o’clock?

P: No, I only open it once a day because I’m still open minded (laughing).

I: Yes, (laughing). So if I may ask, where do you keep your box?

P: In front of my bed.

I: In front of your bed, okay. So what else do you keep in the box besides TB medication?

P: Only TB medication.

I: Okay, okay, umm so tell me what was helpful about using the box in the last three months of your treatment?

P: Uh let just say I got my medication with me; it not laying around as I put it here and not be like “oh where did I put my medicine.”

I: Yes-

P: -When I take the box, I know my medicine is in it.

I: Okay, okay, what else is helpful about the box? You said it keeps your medicine on the same place, what else do you find helpful about the box?

P: … I have nothing to say now , is just the easy way to get my medicine ready.

I: Okay, so in terms of helping you take your treatment on time. Is the box assisting with that in any way?

P: Yeah.

I: Yeah, how so?

P: It’s helpful because I know when open my box my medicine is inside.

I: Okay, how do you know when to open the box?

P: The sister told me at 09:00; they programmed it at 09:00. 09:00 the buzzer goes off, then I know I have to drink it.

I: At 09:00 the battery- the-

P: -The buzzer goes *tee tee tee* [making the box alarm sound] then I know I have to open it.

I: Oh, okay the buzzer goes off then you know. Do you find it helpful?

P: Yeah, *baie;* {means a lot in Afrikaans},

I: You mentioned that you are able to keep your medication in the same place and also the buzzer helps you take medication at 09:00. Is there anything else do you find helpful about the box? You can think of.

P: Nothing.

I: Okay, so far have you face any difficulties about using the box?

P: No difficulties.

I: Have you ever had to travel with the box?

P: *Ja* (yeah) when I got to go play jukskei, I take the box with me and even now I brought my box with me.

I: You have no issue traveling with your box?

P: Nothing.

I: Have you experienced our differentiated models of care which is the reminder SMS’s, the phone calls and the home visits should you not open the box. Have you ever received the automated SMS? To remind you to take medication should you not open the box on time?

P: No, everytime at 09:00 I open it.

I: Okay, so you have never received any SMS?

P: They, they sent me SMS, but I already drink it.

I: Okay.

P: Afterwards they just reminded me- “did you remember to drink your medication” and I said yes.

I: Okay, what made them send the SMS ,yet you already drank your medication?

P: I don’t know .

I: Had you taken the medication from the box on that day?

P: Sometime is five minutes pass-

I: -Yes -

P: -But I have already open the box then.

I: Yes.

P: and then they just reminded me “did you take your medicine” and I know I know I did.

I: So how often do you receive these reminder SMSs?

P: Uh 2 -3 times on the three months now.

I: Okay, so you -in the past 3 months you have received them how many times?

P: I’ll just check it on the[ patient searching phone] so those I received a little bite earlier they texted me and said I must drink my tablets, yes.

I: So, what makes you open it earlier than 09:00?

P: Especially when someone is there- people at the gate, I go like “ oh no, I have to open it before or after.”

I: Okay, what kind of people would be at the gate? Sorry, would you make me understand.

P: Family and friends or people that know me or people that want to know where other people stay.

I: Oh, okay so when you have visitors-

P: -*Ja* (yeah).

I: Okay.

P: About 5 or 6 times {received the reminder SMS}.

I: Okay-

P: -Always here.

I: 5 to 6 times, how did you feel about receiving these reminder SMSs?

P: I’m glad (laughing)-

I: -Why (laughing), what make you glad?

P: If I forget then they reminding me [laugh] and they be like “ hey drink your medicine” then open it at 09:00.

I: Okay, that’s interesting.

P: It was 1 or 2 times I opened it earlier, but then I bring the medicine with me… especially when I get my medicine at xxx (hospital’s name). Then I drink it at 09:00; I open it earlier, but I drink it here.

I: Okay, so how, how early do you open it?

P: My daughter leaves at 06:50, then I go with her cause there is no other vehicle. Then I open it- I open it 2 times , but then this time I was bringing the box with me to the clinic. It is better to bring the box than to open the box and bring the tablets for the nurse to see if I drank my tablets correctly.

I: The tablets, okay, when you were opening and taking the tablets. How were you remembering to take your tablets?

P: (Laugh) I take it in my bag next to my phone and then I know I had to drink it.

I: Okay, you kept it next to your phone. Have you experienced phone calls… from the TB nurse or the intern?

P: Uh-huh.

I: No or what about home visits?

P: Home?

I: Home visits in relation to your medication- TB medication.

P: No.

I: Okay, so the differentiated care or the support you have received so far is the SMS and what else?

P: That’s all.

I: That’s all?

P: Mmm.

I: So, what does the SMS read? What does it say?

P: It just say “you have to drink your tablets.”

I: Okay.

P: But I would just at 5 or 10 minutes before open it.

I: Okay, so do you think the SMS improves the relationship you have with the nurse?

P: I think so.

I: How so?

P: Umm she sent me then I know she cares for me to remember me. She remembers me even I had taken it or if I have not taken it, she reminds me.

I: Umm how does that make you feel?

P: Good because there is someone that uh care for me.

I: Okay, that is interesting; that’s really wonderful… So does the nurse, or the intern show you your adherence? How well you are taking your tablets?

P: Do they-

I: -Do they show you a calendar?

P: No.

I: Showing you how you are doing and what they see.

P: No, they didn’t say anything yet.

I: So, can you tell me any barriers or anything that can stop you or stop anyone from using the smart pill box and also the support like the SMS, the phone calls and home visits? What barriers can people experience?

P: Umm in my case because I got TB I am a little bit scared of it uh there is nothing that can keep me away from the box is my all; I use it and I take care of it.

I: Mmm.

P: Cause they take care of me.

I: Mmm.

P: (laughing)

I: Okay and also from receiving the support-

P:- *Ja* (yeah)-

P:- What else can stop you from receiving the support?

P: Nobody.

I: like the SMS.

P: Nobody can stop me.

I: So, can you think of other patients- other TB patients, what do you think can stop them from using the box?

P: I never know other people that got TB, so I can’t tell you.

I: Okay, oh you just imagining other- what could -what do you think could make other people not take the box?

P: Well then uh how do I explain in English. It may be then worse cause they don’t remember the time

I: Yes, what do you think makes them to refuse it?

P: I have got no idea why because this is a very good idea.

I: Okay, okay and what do you think can prevent people from getting the support like uh the reminder SMSs, the phone call or home visits? What do think can prevent people from getting those? What barriers can stop them?

P: Umm …. I would say if they don’t work together with the sister from the TB, if they don’t care then it won’t help.

I: Okay, so you say if they don’t care.

P: They don’t care, *Ja* (yeah).

I: Okay, what are the resources needed for them to receive that support like your SMS, your phone calls?

P: Uh.

I: What do they need?

P: …I can’t explain.

I: Okay … alright so can you tell me how satisfied are you with this box (box being put on the table).

P: My side I’m excited because that helps me not even to remember, but uh I know my medicine and I have to take it because if I don’t use it will be my fault if I get sicker. If I don’t use it ,then it my own fault because I got cause the box with me saying the time you have to open it.

I: (pages flipping) So, how does it help you- what make you satisfied with the box?

P:Beause I don’t worry because I know on time it will tell me that how to open it- when to open it.

I: Okay, umm…what would make it easier for you use the box- how would we improve the box? What would make it easier for you to use?

P: Because I got the medicine with me all the time.

I: So, looking at the way it is now, it’s like this [referring to the box presented]. Do you think of anything that can be changed to make it easier for you to use it?

P: No, the buzzer is the best. There is nothing else that I can help; the buzzer call you.

I: Okay, alright, umm what about- what do you think about the size of the box?

P: It’s big enough; it mustn’t be bigger.

I: Okay, so you are happy with the current size. Uh what are your thoughts of the colour of the box?

P: Colours doesn’t matter.

I: Alright.

P: The inside matters.

I: Yes.

P: (laughing).

I: And uh what do you think of the way you are currently being reminded?

P: Mmm I like it, *Ja* (yeah).

I: Why do you like it?

P: Because they make it easier for me to drink my medicine at a certain time.

P:

I: Mmm okay, so umm how do you feel about receiving reminder SMSs? You said you received it about 5 to 6 times.

P: *Ja* (yeah) it’s okay with me, that helps me.

I: It helps you okay, mmm is there something you dislike about it?

P: No, I like it.

I: You like it. Umm mentioned that you never received phone calls umm, how would you feel if you could receive a phone call? Let’s say you forgot to open the box and you get a phone call from the TB nurse or the intern. How would that make you feel?

P: I won’t be crossed; I would be glad that she remembered me.

I: Okay, uh and how would you feel umm about the TB staff, umm visiting your home to support you?

P: There was no one that call or come and visit me.

I: Sorry.

P: There is no one who come and visit me from the TB group, I come to the hospital.

I: So, if anyone was to visit you, how would you feel about it?

P: Uh it’s okay with me.

I: It’s okay, why do you say so?

P: I have no problem with them to come and visit me.

I: Okay, alright so can you share your experience of the counselling you received.

P: It was good.

I: Why do you say so?

P: Because I’ve gotten more info about the box.

I: Okay … What else do you like about the counselling, beside the information about the box?

P: The way you treat me and talk to me.

I: Okay, okay.

P: Makes me more open.

I: Mmm, okay.

P: The why you talk to me.

I: Okay, so of all the activities that you were part of in the study using the box- you’ve been part of in the study (box being put on the table ) and also receiving the reminder SMS for potentially receiving the phone calls or the home visit. Which one do you find the most powerful?

P: The box is the first one because its the one that always calls me (laughing)

I: (laughing)

P: They don’t speak to me, but they call me.

I: Okay, when it comes to the SMSs, the calls, and the visits. Which one do you find most powerful?

P: The call.

I: The call, why do to say so?

P: Because then I got contact with them.

I: Okay and how would that make you feel if you are contacting him with a call?

P: I’m feeling okay and I’ve got no problem with that.

I: Okay, so which activities do you find not useful?

P: [inaudible segment]

I: Which one is the least powerful one? You said the call is the most powerful one, which one is the least Powerful?

P: All of them but the phone call that tells me to drink your tablets that is okay.

I: Okay, okay, if you were to rank them in order you would say the phone call and then which one would come next?

P: …No, only the phone call.

I: What are you thought about home visits?

P: What visits?

I: The home visits; them coming to your house.

P: They can come any time, I don’t- there’s nothing umm I would not say oh no, I don’t want you there.

I: Okay, so in your opinion, how can we improve our study like everything about the project- the program the evri-med box, the SMS, the phone call, and visits? What do you think can be in proved?

P: I think that already improved, all of them at one. There is nothing more to improve.

I: Okay, do you have any suggestions on who should teach patients about the box?

P: They teach me and that’s okay, so they can tell other people the same thing, it’s okay.

I: Mmm.

P: There is nothing more they can tell you.

I: So, who are- who do you think should teach people about patients- about the box?

P: The sister.

I: The TB sister {nurse}?

P: Mmm.

I: Okay … uh so … going back to your disclosure about the box, right. You said your daughter, son in-law and grandchild know about the box. Uh does anyone else know about the box besides-

P: -No-

I: -Those three-

P: -No.

I: Why?

P: Because they that the only one in the house that can see when I use it.

I: Okay.

P: If I am going to play jukskei, then I drink my medicine and I put it back in my bag in the car ,so no one sees , but anyone that wants to know what I do. I would tell them that it a good idea to remember.

I: So, you are saying if anyone would see it and ask about the box, what would you do?

P: I would tell that a good it a good idea with all the medicine like this.

I: Mmm.

P: Cause it’s helpful especially when you getting older you forget.

I: Yes (laughing).

P: (laughing).

I: So, uh have you thought of telling someone else about the box without them asking?

P: Yes, I will…

I: Have you done it?

P: Not yet.

I: Not-

P:-Not yet, but I will.

I: But you would, do it?

P: *Ja* (yeah) if someone asks, then I will tell them…

I: That’s interesting …. (pages flipping) so are there any final thoughts you have about the support we are giving in terms of the SMS, the calls, the home visits, and the use of the box? What are your final thoughts- so what are your final comments about[knock on the door]

P: It’s excellent, that all I can say.

I: What make you say so?

P: Because uh it remembers me and that all I got, and that all I have to know.

I: Mmm.

P: Is the time they- I had to drink my medicine.

I: Right, okay, do you have any final comments about the SMSs, phone-

P: -No-

I: -Calls home, the home visits -

P: -No.

I: What are your final comments on these?

P: No, they are all good to me.

I: They are all good, why?

P: Because they are interested in me.

I: Mmm.

P: They know how I’m doing and what’s going on.

I: They know what’s going on and how does that make you feel?

P: Good.

I: Good, can you tell me more?

P: Because they are interested in me.

I: Okay.

P: If you sick umm and you’re not phoning or calling you, they are not interested in my sickness.

I: Yes, yes.

P: That, that the people that are sick - we feel better knowing that there’s other people we are talking to.

I: Yes … uh we have come to the end of our discussion, thank you very much for your participation. If you want more information about the interview we had you can also refer back to the PIS (Participant Information Sheet) we give- you signed. If you have any questions about your study participation, you can contact us; you can use the contact details provide in the consent form you signed. The time is 10:56, thank you very much.

P: Thank you.
