# Supplementary material for: Acceptability of Digital Adherence Technologies to support people with drug-susceptible TB in South Africa
Source: PLoS One. 2025 Sep 24;20(9):e0332103. doi: 10.1371/journal.pone.0332103 (PMC12459780; doi:10.1371/journal.pone.0332103)
Supplement: S4 File — (ZIP) [file pone.0332103.s004.zip › S4 Transcripts/PwTB/IDI 29_PwTB.docx]

**TRANSCRIPTION NOTATIONS**

| **Label Key** | **Meaning** |
| --- | --- |
| **I** | Start of each new utterance by the Interviewer |
| **P** | Start of each new utterance by the Participant |
| **N** | Note taker |
| **{ }** | Indicates that details were changed or pseudonyms were used to anonymise data |
| **( )** | Indicates the description provided to anonymise data |
| **XXX** | Words were omitted to anonymise data |
| **-** | Breaking into a sentence by the next speaker |
| **…** | Pause or drawn out words |
| **[ ]** | Indicates noise made, e.g. [laugh], [sigh], [pause] |
| ? | Beginning of utterance by unidentified speaker or questionable text |
| **[inaudible segment]** | Unclear section of the recording |

I: Do you agree that our interview be on audio recorder?

P: Yah (yes) I agree.

I: Ok Date xxxx (interview date), location it’s (xxxx) clinic name, PID it’s xxx, time it’s 11: 05am, language used Setswana. So sister, sister can you please tell me who you are?

P: I am XXX (patient’s name) from XXX.

I: Mmm ok, so how did you come to the clinic?

P: I came to collect my TB treatment.

I: So, when you come to the clinic do you perhaps walk or take a taxi?

P: I take a taxi cause it’s a bit far.

I: So, when you say “it’s a bit far” how many taxis to you take?

P: I take one taxi, but I take them going to and coming back, so I use take two taxis.

I: So, please tell me a bit about this box, what is it for and how does it work?

P: When I am still sleeping it notifies me that it is time to take pills. They made mine to sound an alarm at 8am, so before I go to work, I would have already taken them, the alarm would have already gone off, I drink and then I leave.

I: How did you find out that you have TB disease?

P: I saw when I was coughing, I have coughed for a long time, and I started to lose weight and then I ended up coming here so that they could check [door opening sound]. I saw by losing weight and then I was coughing for a long time and then I ended up coming here so that they could check what’s going on.

I: Ok thank you. You spoke about coughing for a long time and losing weight, so those are the symptoms or signs that shows that a person has TB disease. So, some of the symptoms, which other TB disease symptoms do you know?

P: To sweat at night when you sweat. Some I don’t remember them correctly, but I never experienced them.

I: So, who explained the box to you?

P: It’s sister XXX (intern).

I: Perhaps who is XXX (intern)?

P: Eish I don’t know if its Sr, but she works here at the clinic it’s just I don’t know of it’s Sr or what she does or social worker, I don’t know.

I: So, the time when she was explaining the box to you, so what on the box what easy for you to understand?

P: Yes because she was showing me how it’s done. She was opening and closing and told me that after a certain a time it will notify me that I must come back. Yah those are some of the things that made me understand her.

I: So, the time she explained the box to you when she was opening and closing the box, to explain to you in details and the box in full, so it might have taken her how long to explain to you about the box?

P: It might have taken her about 30 minutes, 20 to 30 or somewhere there.

I: So, by the way she explained the box to you in that 20 to 30 minutes, perhaps is there something you feel she never explained this thing about the box when she was explaining the box to me?

P: No, everything she mentioned are the things that are happening with the box, it’s like they told everything it. I understand.

I: Ok, so the time she explained in that 20 to 30 minutes, what is it that you would like to change by the way she explained the box to you?

P: Ooh, she said if maybe it notifies me and the I don’t hear it, after about 10 minutes I’ll receive a message, so I have never got that message. [Coughing] I don’t know why but I have never got it, because at times you find that I am preparing water that side and I don’t hear it ringing but I never a got a message even though the time for medication will have passed.

I: So, now I would please explain to me with the experience you have using the box to take treatment for TB . So, when using the box, so what are some of the challenges or difficulties you experienced when using the box?

P: I don’t have but when it comes to ringing eish it can even wake you up even when you don’t want to, you just must wake and then open it then drink your pills.

I: When you say “it can wake you up even when you don’t want to”, so when it’s reminding you, it reminds you in which way?

P: It rings and then it’s loud, there’s no way you can avoid it and say I am leaving it, you will just wake up because even when a person is in another room they can hear it when it rings so you just wake up. It reminded me that way.

I: What reminds you on the box that it is time to take your medication besides the alarm that it is time to take medication?

P: On it? What is it? It lights up in green. Yah, it starts first by lighting up and then it rings. I look at it that much, but it lights up and then rings.

I: Which one do you think it works better?

P: It’s when it rings.

I: So, not long ago you said you are working?

P: Mmm I am working.

I: So, when you say “yes you are working” do you perhaps work in one place or different places?

P: I work in one place, there at XXX (place of work) at the park we clean.

I: So, if you are working in one place, when you are taking your medication, how do you take your medication?

P: Right, we get on at half-past 8, so when I wake up I bath and then around 8 it notifies me then I open it then take out my pills and I drink them. Around 20 past I leave then I walk to work.

I: So, that I get you or understand, you take your medication at home?

P: I take it while I am still at home before I leave.

I: When they explained the box to you for the very first time, what are some of the worries you had?

P: They said if I don’t open, I don’t drink my medication the box will notify them, things like that. I said that “it will make it obvious” if I don’t do so and the other worry was that even at home, they will ask those kids what is it for? A lot of things because I sleep with one in the room.

I: So, when you say “make it obvious” like it will make it obvious in which way?

P: Make it obvious because it rings, those kids can hear it, and yes they it see it because where I put it it’s visible.

I: And then you spoke about “staying with one” in the room and then at home they will see it?

P: Mmm but the elders know and those young ones no, I just say it reminds me to take pills. They don’t ask too much.

I: So, at home where you stay, who do you stay with?

P: I stay with my grandmother and her grandchildren; my grandmother stays at XXX. So, I stay with my grandmother younger sister and her four grandchildren.

I: So, you spoke about “elders at home know about the box”, so how did they feel when you told them that you are using this box to take your medication?

P: Aah they don’t have stress because my grandmother was the one who sent me and said I must go to the clinic and check. Right was the one who was forcing me that I check and then when they see, when they see the box, they could see that I am taking my pills, I am sharp they are encouraged too.

I: So, according to you when they told you the first day when your results came back, they told you that no your results are positive you have a lung disease, how did you feel?

P: Mmm I never got worried that much cause eish I could see that indeed it is like that. I I accepted before I got my results that’s why I said when that Dr told me what I have I said “ok” and left it.

I: Ok, no I am pleased to hear that. And then you spoke about before you tested you were coughing a lot and lost weight? So, now your body how are you feeling compared to the way you were before you started TB treatment?

P: Ooh now, eish weight have not gained that much but I no longer cough like the way I used to cough, now I just cough before I have flu. I was doing laundry some other day but coughing that much has gone.

I: So, at work you get in at half past 8, you taking your medication at 8? So, using this box or having this box or taking TB medication, perhaps did they affect your work in any way?

P: Eish at the beginning it was tough cause we were getting in at 8, I would have to go with it and how many where they? They were four, I think. I would have to take them work so I would get bored a bit. I take them at work, get complications at work, sometimes I vomit and then they said they are changing time to half past 8 it was better because I would do everything at home before I get to work.

I: So, there was a time, a time when you started like you would take your medication at work?

P: That time when we were getting at 8, because I would leave home around 20 to 8 then get there at 8, yes I have to take them at 8. While I have it (box) in the bag.

I: Like how did you feel when thinking that you have to leave with the box, take your medication at work with it (box).

P: Aa that did not put me under pressure that much because I would guard the time, I know that when it gets to 7:59 it would start. When it says 8 and it rings once I open it same time then I take them out and drink them. It was not bad that much, even when it rings they did not hear it cause it is a big park, you could sit there and the other one sits there before we start to work.

I: So, perhaps when you are taking your medication at work with this box, is there someone who ever saw you opening it and taking your medication?

P: Mmm there is no one. [Clearing throat] There is no one because aa some people at work most of the time they get there late and then that park is big, you could find your own space alone, so no one saw me.

I: So, is there someone you told that you have TB disease?

P: Mmm my grandmother and my aunt, all the elders at home know.

I: How did you feel or how did they feel when you told them or explained to them that you this disease?

P: I don’t know but they just acted okay, there is nothing that changed. What they were happy about is that I am taking pills because my grandmother would complain and say I cough too I should go and collect pills, I mean I should check what is going on with me.

I: So, how was it telling them or explaining to them that you have lung disease?

P: Explaining to them? My grandmother would have- right she is the one who sent me, and she said when I came back said “how did you go, what did they say?” and then my aunt I told her via cell phone, it wasn’t that difficult to tell them. My aunt wasn’t difficult when I told her and my grandmother it was a bit difficult cause I told her straight (in person).

I: So, besides the people at home, in the family, is there people outside that I would you told them that you have TB disease?

P: Mmm there no one.

I: So, when you explained to them that yes you have TB disease, what is it that encouraged to tell them about this disease?

P: It’s because, eish my grandmother I think she was diagnosed with it (TB) a long time ago. So, she could see that right even I have it, even though I said I don’t tell her the truth she would see that I am lying to her and even when I am going to the clinic. Its pointless she would see that I am going to the clinic, so lying to her would not help me, so it caused me to tell them that yes, I do have TB.

I: So, when looking at the people who had TB in the family, would you say that there is someone who had TB disease in the family other than you?

P: Mmm my grandmother the one I am staying with here says she once had it and then my aunt texted me and said she has it and she found out not long ago that she has it, but we don’t stay at one place, she stays at XXX (location) at the RDP’s just I stay here at home.

I: So, the time your results came back showing that you have a lung disease or TB, so what is it to make sure that you don’t infect the people you stay with, so what is it that you did, or the clinic did to make sure that like you don’t infect the people you stay with?

P: They collected what do you call it? My sputum and when I came here they told me it (sputum) is okay, there is no way I could infect other people outside. Even when I am taking my pills, there is no way I could infect people.

I: And then how did you feel about not infecting the people you stay in the same house with?

P: Eish I was nervous a bit, I was telling myself that maybe they have it or somehow, but I saw as time goes by that they are not infected.

I: So, when you are using this box, I know you spoke about you did not encounter challenges that much but like your feelings when using this box, like what does this box help you with and then how does it help you?

P: It helps me, right sometimes maybe during the week I could say yes, I wake up because I go to work and then on weekends that’s where I say it helps me because around 8 I would still be asleep. It notifies me around 8 that I must wake up it is what it helps me with especially on weekends because I wake up late.

I: So, in terms of support, because using this box to take your medication is another way of having support Do you have any other support that encourages you to take your medication and that you use the box?

P: Mmm I do have- yes I do have it.

I: So, going back this box, is there a day whereby you opened this box more than once in a day?

P: Yes, I once opened it- eish I once opened it in the morning and then my cousin’s child opened it too, I took it from him/her, but it showed that green colour to show that it has been opened then I closed it again.

I: So, how many times would you say this box is opened more than ones in a day?

P: Aa it’s no longer happening now, I hide it from the child, there’s no way he/she could take it out again. Those other ones they don’t bother.

I: When you say you “hide it”, where do you put this box in the house?

P: Mmm I was putting it under the bed and then now I put on top on the wardrobe. I open it (wardrobe) on top then I put it there… because I was putting water on the floor, then when it rings, I pull it (box) then put those pills back in fast, close it put it back. So that’s why I put it (box) under the bed because it was simple that way [People talking in the background].

I: So, you spoke about when they explained this box to you that it notifies you and then when you taking your medication, they could see that you took your medication? So perhaps that at the clinic how do they see that- you took your medication, or you did not take, do you perhaps know what do you know that they see with what?

P: Sister XXX (intern) said that if I don’t open it, the box would- show on the tablet some dots in green if I don’t open it, telling her that I never drank my pills.

I: So, [People talking in the background] since you started using this box, is it perhaps that the day you started using this box, is it the same day you started taking your treatment?

P: Mmm treatment I stared in February, the box I think I got it in March when I was coming back again.

I: So, there is a time I would you took your medication without this box?

P: Mmm.

I: And then in that period that you started taking medication without this box, how was it taking medication without this box?

P: Aa there I could even- I could even drink maybe around 10 past 9, 10 past 10. So, since the box came, I was drinking on time, I was nervous that it would make it obvious.

I: So, in terms of taking your TB medication, so before the box there was no specific time that you were taking your medication at?

P: It was that 8 but I could take them around 9, around half past 9, around half past 8. So, since the box came right, I take them correctly at 8.

I: And then ever since you started using this box are you taking your medictaion on time?

P: Yes I drink on time.

I: So, can you share or explain to me in a bit that how the box changed how you were taking your medication before to taking your medication at the right time?

P: That time at the beginning, I did not set an alarm. I would say maybe when I was asleep, I would wake up around 9, so I remember that oh I have to drink pills then I drink. So, with the box I know that at 8am it’s going to ring, it wakes me at 8am even when I am not going anywhere, I know that at 8am it’s going to ring then I wake up and drink pills.

I: So, when you drink pills, since you started using this box. You said you were putting your box under the bed and now you are putting it (box) on the wardrobe, so where do you put your pills?

P: They (pills) stay inside the box.

I: So, when they are staying inside the box, would you say the box did help you that your pills be safe?

P: Yah, it helps me cause at the beginning eish they did not have a place, sometimes I would put them in the bag then find there are many so now they are good because I put them in here (box) all of them.

I: So, the time you did not have the box, you put them {pills} in the bag, like that. . How did you see how many you are left with and how far they are when they were the bag, and it is mixed?

P: I would- I would put them the way they pack them, I just put them under the clothes and then when I pull them, I pull them nicely.

I: So earlier you mentioned that when you don’t take your medication you will receive a message e so since you started using this box, have perhaps received a message?

P: I have never received it.

I: Ok, let me say repeat that again. There is an Automated SMS the one you receive when you did not drink- when you did not open the box, when you did not take your pills, then we the one that reminds-.

P: No I have never received the message

I: Then a phone call that reminds you that like it is time to take medication?

P: Even a call I have never received it.

I: There is a thing we call; we call it the Differentiated Care Model. So, in short it speaks that care model like care, so it is receiving SMS that I just asked you about, phone call plus a home visit which I can say they come where you stay, they are going to check up on you. Ever since you started TB medication, the third one the home visit have they ever {health care workers} come where you are staying to check that you are still taking your medication?

P: Mmm they never came.

I: So, ever since the time they explained the box to you, what are some of the barriers or things that would have stopped or could have prevented you from using the box,?

P. I would get problems when I want to travel. Just like when they say I am going somewhere maybe there is a ceremony, eish I think that I must carry it (box) too. So, that gets problematic.

I: So, since you started using the box, is there perhaps a time or period you visited somewhere or left for a ceremony?

P: Visiting yah, I sometimes visit but when I visit I leave it and then [cell phone ringing], I leave it when I am going to work.

I: So, when you are leaving with it when you are visiting, perhaps you visit for how many days?

P: Maybe I went home, like XXX, I can stay for about two weeks.

I: When you are visiting for those two weeks, so is there perhaps like in terms of taking your TB medication, is there something that changes in terms of taking your pills?

P: Mmm there is nothing. I check the date that when must I come back, it’s not like before that time I come back often, so now I can come back after a month. So, I can stay for a long period when I have left.

I: What is your reason for leaving the box behind when you visit?

P: I don’t want people to be asking me too many questions about the box. The other thing is it can be too big to fit in my luggage I won’t have space for it.

I: So, with the experience you have using this box, so your level of satisfaction or like this box, how happy or excited are you with having this box?

P: I am satisfied with it (box) because I can keep my pills inside and they are okay, it keeps them safe unlike before I would put them inside the bag. Sometimes, I would find maybe I have to leave like they say I am going to XXX, I put them in another bag and then end up mixing them etc. So, now they are okay because all of them I put them inside the box.

I: I am pleased to hear that. You just mentioned that time when they were in the bag you would put the with others and mixing them up, so this box are there other pills that you put inside the box that are not for TB?

P: Mmm I only put the TB ones I don’t mix.

I: So, by the way you explained how satisfied you are with the box, - what is it that we can do with the box like to make sure that it (box) becomes more simple or it becomes easier for you to use?

I: Aa there is nothing. I think that it is- the way it (box) is, it’s okay it is complete, it does not want anything because if you have to go to the clinic tomorrow it notifies you today that it is time. I don’t think there is anything missing, it is complete the way I see it.

I: So, when you are taking your medication, it notifies you and then you spoke about even when you are going to the clinic tomorrow it notifies you. Perhaps when you go back to collect your medication or on your refill date, it notifies you in which ways?

P: If maybe I have to go tomorrow, it’s going to light up in orange that tomorrow I have to go back and then it can- it does not go off. Even in that morning I will find it still lighting in orange and the following day when I am going to start again, it is going to notify me the way it was notifying me in green etc. It’s starting afresh.

I: You just mentioned the way it notifies you by, in two forms. By the way of an orange light and the green light, the third one can you explain to me how does it work, the other one?

P: Which colour?

I: [Clearing throat] In colour it is red.

P: The red one I reckon it is the one that tells you that it’s a date to go to the clinic if I remember correctly and then before it is orange it lights that one. It lights up 1,2,3 (Counting using fingers) yes, I think in orange.

I: Earlier on you mentioned that you never received differentiated care model or receiving SMS, phone calls [Coughing] even home visit?

P: [Throat clearing] Mmm I never received them.

I: So, but for you, please tell me your thoughts on which follow up method works better?

I: Is it SMS, phone call or visiting you at home.

P: Yah, the SMS is better, visiting I don’t understand it clearly.

I: So, when you say the way of SMS is better, what is it if you were to get an SMS that reminds you to take your medication. What is it that you will be happy about receiving an SMS and what is it you will not like about receiving an SMS?

I: You mentioned that the way of receiving SMS is the one you say it works better? So, what is it that you will like about receiving an SMS?

P: It is because it reminds you that you must drink your pills unlike if they come at home, it says that you really did something wrong, you have not been taking them for a long time or something.

I: So, you mentioned that the one of visiting you don’t understand it clearly and then you spoke about when they visit you it show as if you did something wrong?

P: Yes it means you haven’t taken pills in a long time and then people {health care workers} are watching.

I: So, for you what is it that you would not like about visiting you at home where you stay?

P: What I don’t like is that they visit me, I prefer the message. They should send a message because sometimes they can come and find out that I am not home just like now. . To me they won’t find me, I will just come to collect pills.

I: You also spoke about people perceptions?

P: Mmm [Laughing] that one was spoken by my aunt, that one of people perceptions. I said at the clinic they called me and they said I must come and then she said “go what if they will come and people end up asking what’s that car is for?” So, I never understood what she spoke about, then I thought that when she said people’s perceptions maybe it will show that truly I am going to the clinic, the car came for such things.

I: So, is there a way maybe that people talk about when people from the clinic visit you?

P: Neighbours talk they will ask themselves what is happening, I reckon they know this one is for what they are going to check up on someone plus someone lost weight maybe they are going to check up on him/her. They are telling themselves that he/she is sick and not taking medication. So, yah SMS is the better one.

I: So, besides that they talk about you, or they see someone coming at home and they talk. Do people around this community talk bad about TB or is it a disease that is being feared?

P: They are afraid of it but when you have it (TB) then the tell themselves that you have HIV because you are slimming such things, they don’t know what you are diagnosed clearly.

I: So, have you received counselling about TB disease?

P: Mmm, I got it here at the clinic when I got my results, when I got my medication. Yah so since then I never I got it again.

I: So, the time you received counselling, what is it you liked about receiving counselling?

P: Aa they told me that nothing is going to change, as long as I am drinking my pills, doing my things correctly I am going to be the way I am.

I: What is it that you would like them to talk about during the counselling sessions?

P: Mmm they must tell me when am I going to finish taking my treatment. So, they said about 3- they said it might be 3 months, it might be 6 months or what. They did not tell me straight that I am when am I going to finish it clearly, so that is what I wanted to know that when am I going to finish.

I: So, out of everything that the box does and the activities of differentiated care model. So which activity do you think we could improve on or like we could add to make sure that a person his/her medication?

P: Yah SMS because like what should I say [inaudible segment]?

I: So, all the activities differentiated care model activities that we conducted, an SMS, phone call and a home visit plus bringing the box. So, which one like out of these activities do you think like this one is perfect, it works better than all those others?

P: It’s the box, the box straight.

I: And then you spoke about, the time I asked you about what we can add. Your thoughts or according to your thoughts, what is it that we could improve on the box to make sure it improves?

P: Aa it (box) is complete how I see it, all the things that are needed on it are there. It reminds you the date you are coming back on, it reminds you to drink your pills, I don’t see anything needed on it.

I: So, this box is another part of the technology that we implemented on our study.What is it that we can improve on our study to make sure that our study becomes more effective, it improves? Where can we improve on our study?

P: I don’t know, you know.

I: Is there anywhere you saw the box before it was shown to you here?

P: I saw it (box) here at the clinic.

I: So, like this is my wrap up , so like we are closing now. So, but before I close everything, so looking at the way the study was delivered, the box, and the phone calls. Is there a gap that you feel that we did not cover maybe we should do this in a certain area?

P: I think we are perfect to be honest because itrings in the morning when you are supposed to drink your pills and then if you don’t drink them, it rings again as long as you haven’t opened it, it won’t stop. It can stop and the after 10 minutes it’s going to rings again reminding you the pills. I don’t think something is missing, I think it’s complete.

I: So what are your thoughts on the differentiated care model like the one of SMS, phone calls and a home visit. So, like what are your final thoughts or comments on the differentiated care model?

P: About?

I: The differentiated care model, the way of receiving SMS, phone calls and a home visit, what do you think about this model?

P: The SMS, visits are good maybe when they see that the person is not coming to the clinic to collect his/her medication. That is when they can visit.

I: What is it that you can tell me about the box ?

P: Mmm I think it (box) is good and then helped me too much because it keeps my pills in order, it wakes me up, I loved dearly. If it wasn’t for the box I was going to suffer, setting up alarms and then forgetting.

I: Ok, I have come to the end *neh*, so before I close. Looking at the box with the experience you have, do you think that the box should have been implemented way back?

P: Yah the box should have been given to patients long time ago, especially for older people, because some don’t even have cell phones. So, it was going to help them and then they don’t know how to set alarms like us.

I: uWhich age group are you referring to when you say “older people” like who are older people?

P: Grandmothers, grandfathers but I am happy with that it doesn’t exclude young people, it’s notification reminds you when you are young that you have to drink your pills.

I: So, you mentioned that grandfathers, you will find them struggling to set an alarm on the cell phone [---] would describe that people from this age should use this box? Like elderly people start at which age?

P: Maybe they are somewhere around 60.

I: So, looking with the experience that you have, how do you see this box helping people going forward?

P: It is because people forget to take their pills on time and the box reminds them and also the box reminds them when they are supposed to come to the clinic. That is what helps us the most about it.

I: So, like sadly I have come to the end of our conversation or our interview, so what is the last thing you would like to say about our study?

P: Ooh thank your for teaching me more about home visits, I didn’t know much about about them.

I: No, I thank you very much for everything that you said. So, your information, what you spoke about it is important for us. So, but sadly we have come to the end of our interview *neh*. Ok I thank you. Time ended its 12:00PM.

Glossary

Yah = Yes

Sharp = Alright

Via = Through

Vele = Agreeing to the statement

Eish = Uncertainty usually accompanied by thoughts/remembering

Ankere? = Verifying/ supporting a statement.

Kasi = Community/surrounding

Neh = Right?

Sr = Professional Nurse

Dr = Dr
