# Supplementary material for: Acceptability of Digital Adherence Technologies to support people with drug-susceptible TB in South Africa
Source: PLoS One. 2025 Sep 24;20(9):e0332103. doi: 10.1371/journal.pone.0332103 (PMC12459780; doi:10.1371/journal.pone.0332103)
Supplement: S4 File — (ZIP) [file pone.0332103.s004.zip › S4 Transcripts/PwTB/IDI 30_PwTB.docx]

**TRANSCRIPTION NOTATIONS**

| **Label Key** | **Meaning** |
| --- | --- |
| **I** | Start of each new utterance by the Interviewer |
| **P** | Start of each new utterance by the Participant |
| **N** | Note taker |
| **{ }** | Indicates that details were changed or pseudonyms were used to anonymise data |
| **( )** | Indicates the description provided to anonymise data |
| **XXX** | Words were omitted to anonymise data |
| **-** | Breaking into a sentence by the next speaker |
| **…** | Pause or drawn out words |
| **[ ]** | Indicates noise made, e.g. [laugh], [sigh], [pause] |
| ? | Beginning of utterance by unidentified speaker or questionable text |
| **[inaudible segment]** | Unclear section of the recording |

**I**: Do you Sir agree that we record our conversation on audio recorder?

**P**: Yes, I agree.

**I**: Okay, thank you. Date: xxxx (interview date), Location: xxxx (clinic name) , PID: it’s xxx, time: it’s 11:15am, Language used: it’s Setswana.

**I**: So, in short can you please explain to me who you stay with where you stay.

**P**: At home I just stay with the kids. My wife and I separated a long time ago, after I found out about this illness, I am just left with the kids

**I**: I am sorry to hear that Sir. So, when did you know about TB sir?

**P**: I did not know about it, I started feeling sick at work, then they took me to the doctor that is when they told me I have lung problems.

**I**: Okay, when you say you started feeling sick at work, in short can you explain further.

**P**: I was working at the butcher, working with ice. While working I started feeling pains inside, I did not have the strength. They took me to the hospital and they found that I had ice in my lungs… The problem started there.

**I**: So, I will come back to it. Where do you leave sir?

**P**: At the xxx (area name)

**I**: Here at xxxx (area name)?

**P**: [inaudible segment]

**I**: Okay, so sir when you come to the clinic which mode of transportation do you use?

**P**: I walk. I am not working, I stay home.

**I**: Oh Okay. There is no problem… The box on my side, do you Sir by any chance know this box?

**P**: Yes, I know it.

**I**: Okay. So, by the time they explained to you about this box, who explained to you about this box?

**P**: I cannot remember the sister or [inaudible segment] [background noise] because mostly when I come, I would not find the sister. They would change me and I would not know who is the first person. All I wanted was to get help and be fine.

**I**: Okay, the time they gave you an explanation about the box, how many minutes did it take?

**P**: Let’s say they took around 15 to 20 minutes, explaining everything about the box to me.

**I**: Okay. So, the time they were giving you the explanation about the box how did you feel?

**P**: I was already taking my treatment, so I was feeling alright, not losing hope.

I was just alright.

**I**: Okay, according to the way they explained to you about the box, is there something you would like to change from the way they explained about this box?

**P**: No, all I said when they told me was everything is okay [inaudible segment].

**I**: Okay… So, going back to where you said you started feeling sick at work and you were told you have ice in your lungs

**P**: Mhmm.

**I**: So, what made you to finally say now I am going to test or now I am going to the clinic to consult, what really happened to your body?

**P**: It happened that while I was leaving work going to the hospital, when they observed me, they found that my condition was bad, I had ice in my lungs, I worked for xxx years in the fridge section. I worked in the freezer section. So, I was not aware that I have this problem, they told me after the x-ray.

**I**: So, when you say you have xxx (number of years) years working where there is ice, was it your first time having a lung illness, or was it the first you were told you have ice in your lungs?

**P**: When I was at the hospital, they told me I have ice, they made an x-ray and they told me I have ice and it is packed inside.

**I**: So, for the past 14 years, you never experienced such a problem?

**P**: No.

**I**: It was for the first time?

**P**: It was my first time having this illness.

**I**: Okay. So, do you perhaps know about TB?

**P**: I only knew about it from my experience, but I hardly get sick, I keep myself busy with a lot of stuff.

**I**: Okay. So, TB is a lung disease. **–**

**P**: Mhmm.

**I**: So, at the time you were coughing a lot, what are the signs which were evident indicating that you are sick.

**P**: I was not even coughing, the only thing that was problematic was my extreme tiredness, but I was totally not coughing. [Inaudible segment].

**I**: Okay. Do you know the TB signs/ symptoms?

**P**: No, I have not seen them.

[Noise, page turning noise]

**I**: So, like I explained during the consent form that I am going to need your feelings and experience of using this box.

**P**: Mhmm.

**I**: So, can you sir please explain how this box works.

**P**: Every morning this box rings, at 6am. I take out two tablets and I drink after that I go back to my sleep again. It wakes me up.

**I**: So, when you say it alarms, in short it alarms in which way?

**P**: They asked me what time do I wake up and I said I wake up at 6am and they set it to ring every day at 6am.

**I**: So, you mentioned that it alarms for you?

**P**: Yes, it alarms

**I**: So, when it reminds you that it is time for medication it alarms, what other ways does it utilize to remind you?

**P**: When I hear the way it rings, I there and then wake up as I know it is time for me to take my medication.

**I**: So, since you have started using this box, which challenges have you encountered?

**P**: Like what? I do not understand you clearly.

**I**: Like challenges, things that… like they were challenging you from using the box? Challenges, things that would prevent you from using the box perfectly.

**P**: I’m sure they would help me with this one.

**I**: When using this box, which hardships did you encounter while using this box?

**P**: What do you mean? I do not know what to say, I honestly do not know.

**I**: So, according to the way I asked I meant which challenges did you encountered when using this box?

**P**: I did not encounter any challenges

**I**: So, the box itself you mentioned that it alarms but how has the box itself been treating you?

**P**: It has been treating me good, I even become happy.

**I**: So, when you say it has been treating you good, for me to get a better understanding, can you elaborate more on the happiness, it has been good in what manner?

**P**: It has been treating me good in terms of my medication, I also like the way it rings because when I wake up I just take out my medication from it, and then close it again.

**I**: Okay, are you sir working?

**P**: I am not working… I am on early pension… I do not have the energy to work.

**I**: Okay. So, at times do you perhaps have temporary jobs?

**P**: Yes, I do have piece jobs at times.

**I**: So, please explain the way you work around this box and your temporary work, because I know this box has its own way of affecting temporary or piece jobs.

**P**: It has not affected me.

**I**: Okay, so these piece jobs, do you perhaps have them in one area or different areas?

**P**: I do not work everywhere, I only work when they have called me to do the plumbing, to fit in the pipes and then I come back home. It is not an everyday work.

**I**: Okay. So, for me to understand, what time does this box remind you?

**P**: This box rings at 6 o’ Clock in the morning.

**I**: Oh, 6 in the morning?

**P**: Yes, in the morning.

**I**: So, when you have received a request for a piece job what time do you leave the house?

**P**: I leave at about 8am, yes around 8am.

**I**: So, at the time you leave the house you have already taken your medication?

**P**: Yes, I start with it at 6am early.

[Pause]

**I**: So, when you use this box, or when they explained to you about this box, what were your worries about this box?

**P**: I did not have any problem with this box, I have not had any problem.

**I**: Okay, is there someone I would say you have given them an explanation on this box?

**P**: No, I did not explain to anyone.

**I**: Okay. [Pause] At first you mentioned that you have kids in the house.

**P**: Mhmm.

**I**: Do they know about this box?

**P**: They do know about the box but they have never asked me anything about it.

**I**: If they know it, is there perhaps other people who know the box?

**P**: They do see it, when they ask what is the box for, I need to answer back by saying it is for TB. I need to explain to them so that they understand what is going on.

**I**: So, you just stated that when people ask, you need to explain the box is for people with TB.

**P**: No one from outside would ask me because the box stays in my bedroom, there is no way I could go wherever I go with it; it has to be in a safe place. When someone comes to me, I sit with them outside.

**I**: So, for better understand has a person from here at the clinic or someone you might have bumped into on your way to the clinic asked you about it?

**P**: No, there is no one.

[Pause]

**I**: Okay. So, do you perhaps say there was a day you probably visited a place with this box?

**P**: Yes, when I go to xxxx (town name), I take it with me.

**I**: So, at home do the children you stay with know that you have a lung illness or disease?

**P**: Yes, they know, they know.

**I**: So, at the time you explained to them that you have a lung illness, how did they feel?

**P**: It was hard for them; they did not take it well.

**I**: Okay. So, when you say the news treated them bad, they did not take it well, could you explain their emotions.

**P**: When I explained to them that I have lung illness, do not be like me, you must go to the clinic so that they can examine you.

**I**: Okay. So, at the time they started you on TB treatment what did the clinic do to make sure that the children you stay with do not get infected by TB?

**P**: Some sister came with a car at my house to check on them

**I**: Okay. So, when they came to your house with the car, to check them **[**Noise**]**

**P**: Check everyone, I would tell them people are coming here, you are not allowed to leave, they are going to check your lives.

**I**: Okay. The time you visited xxxx (town name), it is that where you visited know this box?

**P**: No, when I arrive there, I take it to the bedroom, and in the morning when it rings, I take my medication and close it again.

**I**: But to say that you Sir has lung problems do they know?

**P**: I only got the chance to explain to them now people in xxxx (town name).

**I**: So, what was their emotions when you explained to them?

**P**: They were positive and they said as long as you take your medication you will be fine; you are not the first one.

**I**: Okay. So, the time you explained to them about your medication, was it easy for you to explain to them?

**P**: What do you mean?

**I**: Was it easy for you to explain to them?

**P**: Yes, I had to explain to them so that they know what was the problem with me. Keeping a secret is not good.

**I**: So, when you say keeping a secret is not good, what would encourage a person to explain that I have this specific illness?

**P**: Be open to your emotion. Do not be afraid, tell them everything so that they know that our sibling is in this particular state. So that when I need help from them, I can call so that they can send something.

**I**: Okay. And then when you visited xxxxx (town name), you visited for how long?

**P**: Yes, for a week.

**I**: Okay. So, for you to visit xxxxx (town name) has it interfered with how you used to take your TB treatment?

**P**: I took it at the same time as I am at xxxx (town name) [Noise]

**I**: Okay. So, at home has there been anyone else who had a lung problem besides you?

**P**: No, I cannot remember. But I am the first one.

**I**: Okay. So, this box, is there a day whereby you opened it more than once in a single day?

**P**: No, I just open it once when I take out my medication and I close it again.

**I**: Okay. But my question is, have you even opened the box two or three times in a day, more than once?

**P**: I just open it once.

**I**: Okay. So, when you open it once where do you place your medication?

**P**: I put them inside the box, as they give them to me.

**I**: And then when you arrive at home, where do you place the box?

**P**: I put the box in the drawer by my bed so that when it rings, I can just take them nearby.

**I**: So, for me to understand, you say the box stay in the drawer?

**P**: No, it stays on top.

**I**: Oh, on top?

**P**: Yes, on top.

**I**: Okay. So, sir I hear the way you respond to the alarm of the box, or rather how it rings…so like we have different colours that appear here, do you know how these colours work?

**P**: As for me I just told myself I do not understand these colours. It is three different colours so I told myself I cannot understand what is going on there so I am just happy that it rings and reminds me to take my medication.

**I**: Okay.

**P**: Yes.

**I**: Okay, so for me to understand, you are only reminded by this box when it rings only?

**P**: Mhmm [inaudible segment]

**I**: Okay. Besides the box ringing there is no other way that reminds you?

**P**: No, as long as I know I have taken my medication, I am done with it. I will see it again the following day.

**I**: So, is it that the day you started taking TB medication the same day you were given the box?

**P**: The time I was sick that is the time they gave it to me.

**I**: Okay. So going back a bit you mentioned that they assessed our lungs through an x-ray.

**P**: Mhmm.

**I**: So, as it that they started you on treatment at the hospital or you started taking your treatment at the clinic?

**P**: They could not help me at the hospital, the referred me to this side and that is when in started my treatment.

**I**: Okay. So, there is no day I would say you started your medication without the box?

**P**: No, I started with the box this side.

**I**: Okay. I understand. So, do you have a cell phone?

**P**: Yes, I do have a cell phone.

**I**: Okay. Did you ever receive a message that states that you should not forget to take your medication?

**P**: Yes, I at times receive the message.

**I**: So, how many times did you receive the message?

**P**: I received the message a couple of times in line.

**I**: Okay [Pause] is there a day I would say you did not take your medication?

**P**: In that case I would have forgotten, but for as long as I sleep at home I have to start with the medication before I go do other things.

**I**: So, you understand that you have to drink your medication.

P: Yes.

**I**: But is there a day that passed without you drinking your medication?

**P**: No, I drink them. The sisters (Health Care Workers) say I do not drink my medication, I tell them I drink my medication and they said their system does not correspond to the box.

**I**: Okay. So, I am going back to the system not corresponding. The time you received the SMS, what did the SMS state?

**P**: That I must drink my medication at 12 o’ Clock then I told them that I drink my medication and they said my name reflects late on their system.

**I**: Okay. So, do you know that when you receive the SMS what would have happened?

**P**: They remind me that I must have taken my medication because I have to drink my medication.

**I**: Okay. Earlier on you said you take your medication at 6.

**P**: Yes at 6.

**I**: Is there a day I would say you forgot…

**P**: I forgot to take it.

**I**: When you say you forgot to take your medication can you explain…

**P**: I cannot understand [inaudible segment].

**I**: Okay, let me explain fully and finish up. So, you take your medication at 6,

**P**: Yes at 6.

**I**: Is there a day I would say you did not drink it at 6, maybe you took it at 7, or 8, or at 9 or maybe you drank it before 6.

**P**: To be exact I once took it at 6:30, meaning I was 30 minutes late for taking my medication. Ever since then I am always precise when it come to my medication time.

**I**: Okay… So, like besides the box reminding you to take your medication, is there perhaps something else that is in place to remind you to take your medication besides the box?

**P**: Eh… if there is no box, I would become mixed up because I am now used to being woken up by the box [laugh] it would be a problem.

**I**: Okay. Like being mixed up is interesting… so please explain what you meant being saying mixed up, because I do not understand how would be mixed up.

**P**: I would forget, the box would not be there, it would only be the medication and when I have to wake up at 6, I would be tired and I would have done a mistake myself, so it is better when it rings because you know.

**I**: Okay. So, this box assists you to take your medication everyday and on time?

**P**: Yes, on time.

**I**: So, what is another thing that you would say this box is helping with?

**P**: The box assists me with time.

**I**: So, you mentioned that at times the system does not correspond with theirs?

**P**: Yes.

**I**: So, do you know that when you did not take your medication on time or when you did not take it at all how does the sister pick that up?

**P**: That is what I want to know that how do they see that. Because I heard xxx (intern) asking if my machine rings, and I responded saying yes it rings and said on her side it shows that I do not drink my medication and I said I do drink my medication. So that is why she said I should come with the box so that she can fix that issue.

**I**: Okay. [Laugh]. So according to your answer you do not know how the sisters see when you have taken your medication and when you did not take your medication?

**P**: Yes, and that is what I want to know, how do they see.

**I**: Okay. So, there is something we call an adherence calendar. We see it on the tablet that is utilized in the TB room.

**P**: Yes.

**I**: We use it to see if whether you have taken your medication or not. So, when you have taken your medication, we see through it same applies to when you did not take your medication, we see through it.

**P**: Mhmm.

**I**: I am surprised that you never saw your adherence calendar, because its something we use it to see if you have taken your medication or not.

**I**: So, since you have never seen your adherence calendar how do you feel that you have never seen your calendar?

**P**: I have never really followed up with it to ask what is happening and all.

[Pause]

**I**: So, sir you explained that the box reminds you and also helps you take your medication on time, so this box is another way of the Clinic and Aurum to show that we support you, it is a support action that we actually do as a study to show that you are taking your treatment everyday and accordingly. So, you also mentioned that without the box you would be mixed up or messed up, so is there perhaps any other support you are getting that reminds you and encourages you to take your medication at time and accordingly?

**P**: I can feel it when I have taken it at the certain time, I feel it.

**I**: So, for me to understand you, when you say when you have taken it at the certain it you become alright, I do noy understand well. Like when you are not alright, what would have happened?

**P**: When I am not alright, I know that I did not drink my medication because I know when I have taken my medication, I become alright. I do not have a problem there [Cough].

**I**: Okay, so you mentioned that you take your treatment accordingly and on time.

**P**: Yes.

**I**: So, if you say you take it on time and accordingly, what would have happened when you say you are not alright?

**P**: It would be because they did not provide me with the required treatment, that is okay for my body.

[Pause]

**I**: Okay. So, to go back a bit, you mentioned that you know when you feel good and when you not feeling good, so in short please explain how you are currently feeling?

**P**: My body is currently okay. I am extremely okay.

**I**: Okay. So, for me to understand can you explain fully what you mean by saying you are alright?

**P**: I mean I am okay, even the pains are not there, it is like I have never been sick. I am just alright, even the headache, I am just alright at the moment. I totally do not have any problems. It is not like before.

**I**: Okay. So, when you say it is different than before, can you explain how you felt previously.

**P**: When I started being sick my mind was not even functioning well, I was sick, my body was not alright, now since I started my treatment things have changed. This life of taking medication has been treating me very well, everything seeks you to believe, if you do not believe you will not survive.

[Pause]

**I**: Okay. [Pause] So, there is something we call a differentiated cared model, let me explain what it means, a differentiated cared model is the SMS’s, phone calls and home visits.

**P**: Mhmm.

**I**: So, you mentioned that you know how to receive an SMS, do you perhaps know how to receive a phone call?

**P**: They call me.

**I**: Okay. So, they called you how many times?

**P**: Even today they called me.

**I**: When they call you what are the reasons for them to call you?

**P**: They explained that I have to come, there are people who would like to see me for an interview. When I was still busy the sister called again and I said I am on my way.

**I**: So, besides today other days…

**P**: They still call me.

**I**: When they call you what would have happened?

**P**: They remind me to come.

**I**: So, for you to understand let me be specific, have you received a call stating that you should not forget to take your medication?

**P**: Yes.

**I**: Okay. So, let me go back a bit so that I understand, you take your medication everyday at the correct time, but you also received a phone call that states you should not forget to take your medication.

**P**: Yes.

**I**: So, what had happened?

**P**: So, when they called they thought I do not take my medication, because system is not reporting to them, so I told them that I do drink my medication. Hence, I said the machine does correspond with one another.

**I**: Okay. So, a home visit, have they visited you whereby they remind you, or to check that you are taking your medication?

**P**: To come here?

**I**: I mean at the clinic have they visited you to check if you are taking your medication?
**P**: Yes, they do come and check me.

**I**: And then when they come, what do they discuss with you when they have arrived at your home?

**P**: At home?

**I**: Mhmm

**P**: We just sit with them in the veranda, they even surprise me by saying we are not allowed to sit in the veranda, I am not allowed to sit with other people. [Inaudible segment]

**I**: So, in short because you have received an SMS, phone call and home visit and you have this box. Please explain how you feel to have support from all angles, how do you feel to have support?

**P**: I just feel alright, I feel happy.

**I**: What do you mean by saying alright? Please explain.

**P**: How would I explain it.

**I**: But what were your feelings when they visited you at home, to check up on you?

**P**: They had a list of their patients who they would visit, when they come they notify you so you need to know the date they set to come to you.

**I**: Okay. When they explain about this box, what was the barrier or something that would stop you from using this box to take your medication?

**P**: Something that would stop? [Inaudible segment] until my treatment is finished.

**I**: Okay. So, but was there something that would stop you from using it?

**P**: I have to use it until I finish my treatment. I cannot just leave it in the middle, I am not yet fully recovered.

[Pause]

**I**: Okay. So, going back a bit. You mentioned that you take your medication at 6 in the morning.

**P**: Mhmm.

**I**: And then you, and you received an SMS that said you should not forget to take your medication before 12, so like what were your feeling when receiving an SMS that says you should not forget to drink your medication, whereas you have already taken your medication?

**P**: So, you see they remind me.

**I**: So, they were reminding you to take your medication

**P**: Yes, not to forget to take my medication. **– I**: Whereas you have already drank it?

**P**: Yes, whereas I have already drunk it. That is why they said the machines do not correspond correctly.

**I**: Oh okay. So, they were not corresponding well?

**P**: I think that is how they saw it, because when the machine rings it has to report to them that I am drinking. I drink at 6 and they go to work around 8 so these things do not correspond. They need to fix so that things are equal.

**I**: When you say they need to fix, did it perhaps get a chance to get fixed?

**P**: Yes, I have that hope.

**I**: Okay.

**P**: I have the hope that it must have been sorted out.

**I**: So, till today do you still get the message that reminds you?

**P**: I get it [inaudible segment] today since we are here I will ask if they have set it to be correct or not. They should not be shouted at; they should just do their jobs.

**I**: So, like how do you feel at the moment? Okay, let me [inaudible segment] now how do you feel that… okay, then you received the message because like you said the system was dysfunctional or not corresponding.

**P**: Mhmm.

**I**: So, how do you feel because like you said the matter is now fine, it is now balanced, so what are your feelings?

**P**: I just feel okay.

**I**: So, like please explain your happiness/ joy, what is your satisfaction in full? How happy are you that you are using this box?

**P**: When they gave me, I did not know its work so now that I am free, it works in my favour.

**I**: It can unfold, it works in your favour, what do you mean?

**P**: I mean I do not have pains like before, you can see I am just alright now.

**I**: So like you were using this box, what can be added to this box that would make it easy for you to use this box?

**P**: I do not know how you thought of it, I do not know what you could do.

**I**: Okay. Remember, I understand you but remember before I mentioned that every answer you give will help us to improve this box.

**P**: Mhmm.

**I**: When you look at it, because you have an experience of using it, so, like to make sure it is well equipped where can we improve it?

**P**: The box is fine as it is.

**I**: Okay. So when you say it is fine, what is… yes I understand, but what is it that you see as missing in this box, if this box had that it would work perfectly so?

**P**: [Pause] I do not see any problem; it is fine the way it is.

**I**: I am happy to hear that, there is no problem. So, now please explain how you view this box and also how you visit the ways of home visits, phone calls, SMSs, only your thoughts and perceptions on these.

**P**: I am good because they did not have to call me because the problem was the box

**I**: Okay.

**P**: Yes.

**I**: So, you told me about the alarm.

**P**: Mhmm.

**I**: That this alarm reminds you to take your medication, so this box has colours,

**P**: Mhmm.

**I**: So, please tell me, whether it is your day to go to the clinic or your medication is almost finished what reminds you that tomorrow or the following day you have to go to the clinic to fetch your medication?

**P**: They give me a date, I go there according to the date they gave me.

**I**: So, on top of this box is there something that reminds you that tomorrow you have to go to the clinic to fetch your medication?

**P**: Yes, I know on the 1^st^ I come to the clinic and then I come back home. I would never forget the date.

[Door bang]

**I**: Let me explain it in simple terms so that you can understand. You use the date to know that tomorrow you have to go to the clinic, so on top of this box there is something that I say it reminds you that tomorrow you have to fetch your medication.

**P**: I do not leave it behind, I take it with when I come to the clinic.

**I**: Okay, I am happy to hear that. I am asking for the last time.

**P**: Mhmm.

**I**: When it is time for medication the alarm reminds you.

**P**: Mhmm. Or when the light goes on, they wake me up and then I wake up and drink my medication.

**I**: So, for me to close my questions the alarm is the only thing that remind you?

**P**: Yes, it is the one that reminds me.

**I**: So, when you view this SMS method when a person does not take their medication when you send the SMS would it help?

**P**: When a person does not take their medication the SMS would help to remind them. If you do not go, it would be on you, you would be killing yourself because the life is yours.

**I**: So, you mentioned that when you do not go you are the one killing yourself, so when a person is not taking their medication when you send them an SMS, what would be the cause of them not to take their medication?

**P**: When you do not take your medication [inaudible segment] you have to do what they tell you to do.

**I**: As clinic or as Aurum what can we do to make sure that a person is taking their medication?

**P**: What do you mean?

**I**: Let me explain fully. We have this box, which reminds the patient to take their medication on time, so to fully equip this box what can we do as a clinic to make sure that the person is taking their medication, where can we help?

**P**: [Inaudible segment] so that I can carry on with life.

**I**: Please explain to me again.

**P**: You give me the medication and help me to carry on with life.

**I**: So, back you mentioned that they came home with a car to check up on you.

**P**: Mhmm.

**I**: So, have you ever received counselling?

**P**: They only came to check if I do drink my medication, after asking if I do drink my medication then they left and said they are impressed by the way I live, because many people do not drink their medication, so we do not want you to be like other people.

**I**: So, when you say they came to check up on you, what did they say when they came?

**P**: P: As an old man they came to check me and we would sit and talk. They said it is evident that you do drink your medication, they would say others do not do like this, so they encouraged me to keep on taking medication.

**I**: So, during the counselling or when they came home what would I say it is not appropriate for them to do?

**P**: We just talk, they ask me what they came for.

**I**: Okay during the counselling, when they come [door bang] when you view them which of them do you think are qualified enough to conduct, those that are qualified to conduct the counselling or visit patients at home?

**P**: [Inaudible segment] us who have TB.

**I**: Okay, so according to those activities which one do you think works best? Please explain again so that I can understand you.

**P**: I am saying when the box is working well, it would reflect on their system so they would not have to come do home visits.

**I**: Okay. For me to understand, what you are saying is they do not have to come at home.

**P**: At home.

**P**: I do not have a problem, according to me I feel that their systems should correspond [inaudible segment].

[Pause]

**I**: So, the activities I have mentioned… Because I feel you are extremely happy with the box more than anything.

**P**: Yes.

**I**: So, out of those that are left which one do you think does not work well?

**P**: The one’s you mentioned do not really give or have a problem. The only thing is that they have to correspond to the box.

**I**: So, for me to understand, when the box system corresponds, when you take your medication accurately, you do not see the need for them to do home visit.

**P**: No, I do not see the need for them to come.

**I**: I wanted to understand that part. This when you saw it at the clinic was it the first time you saw here?

**P**: Just saw it here at the clinic… You have not finished your explanation regarding the lights.

**I**: Okay. So, I am sure they explained to you about the light but I am certain because it was a while ago, you have probably forgotten their explanation about the lights. [Pause] The green light is to remind you to take your medication. The green light aligns with the sound. The middle one is the orange light and it reminds you that it is time to go to the clinic, when your medication is about to finish you will see with the orange light. It illustrates that tomorrow you must go to the clinic. The third light is the red on, it is for when the battery is about to finish.

**P**: Okay.

**I**: I am sure you did not understand.

**P**: I did not understand what was going on.

[Pause]

**I**: Okay. So, you mentioned that during a home visit the box should correspond with the system, why did you think that when they came to your home to check up on you, do you think it meant that they do not correspond with the system?

**P**: It is because it does not treat me well or rather, I did not take it well. So, for to feel free, things should be done accordingly.

**I**: When you say it did not treat you well, how did you treat you?

**P**: You see there whereby they said I do not take my medication and I am late for my medication, that did not sit well with me, because I was busy taking my medication accordingly. So, it did not sit well with me. Hence, I say it should be fixed.

**I**: So, could that and also that of the system be resolved at this stage?

**P**: I am sure the sister did it.

**I**: When you say you are sure the sister did it…

**P**: She once took the machine to check how it works.

**I**: So, like you say which are the changes that show that it is now working?

**P**: She told me that she worked on it, so that she does not have to worry because she also worries when things are not working accordingly.

**I**: So, like now we have reached the end. Now I want us to talk about things that I did not understand but it is a rap up, trying to close it up. So, when looking at this box, accordingly to your feelings do you feel that this box should have been implemented a long time ago.

**P**: Here or at Baragwanath.

**I**: Here at the clinic. This box has just been implemented to see if it works and like you mentioned it helps you as it reminds you to take your medication. So, according to how it changed the way you were going to take your medication, do you think it should have been implemented a long time ago?

**P**: Yes. According to me that is how it should have been done.

**I**: Okay. Why would you say so?

**P**: Because some have started with it in the middle of their treatment, so they would have gotten a chance to start with it like me in the beginning.

**I**: Okay. As a person who has experience with this box, how do you see this box being helpful in the near future?

**P**: It helps, more especially when you believe. I thank the person who implemented it.

**I**: Okay. So as time goes on [Door Bang] you mentioned that you would be mixed up. **P**: Mhmm.

**I**: So, accordingl to the manner it changed the way you took your medication, the manner it helps you, how do you think it would help other people?

**P**: They must take me as an example, that it can change.

**I**: Okay. So, when you look at this box, cell phone, and that at the clinic they helped you and also they came home to check up on you, that they gave you support in all angles so that you take your medication. Do you see any gap that you feel is not correctly filled? Is there a gap that needs to be filled to make sure that you get our full support?

**P**: The way it is currently I think it is okay, there is no problems.

**I**: Okay. So, unfortunately we have reached the end of our interview. I thank you.

**P**: I also thank you.

**I**: But before I close, please explain your overall satisfaction, what you think of the box and also about TB, your overall thoughts.

**P**: I believe I will get healed with this box, so that others when they are sick I can explain that they can receive the box at the clinic. I need to explain to them openly and not hide anything.

**I**: Okay. So, I am happy to hear that. Unfortunately, I have reached the end of our interview. So, I thank you 6647 for the time you took to do this interview with us today. So, I thank you for your time.

**P**: I thank the sister that side who invited me, thank you sister.

**I**: So, time ended 12:15pm.
