# Supplementary material for: Acceptability of Digital Adherence Technologies to support people with drug-susceptible TB in South Africa
Source: PLoS One. 2025 Sep 24;20(9):e0332103. doi: 10.1371/journal.pone.0332103 (PMC12459780; doi:10.1371/journal.pone.0332103)
Supplement: S4 File — (ZIP) [file pone.0332103.s004.zip › S4 Transcripts/PwTB/IDI 31_PwTB.docx]

**TRANSCRIPTION NOTATIONS**

| **Label Key** | **Meaning** |
| --- | --- |
| **I** | Start of each new utterance by the Interviewer |
| **P** | Start of each new utterance by the Participant |
| **N** | Note taker |
| **{ }** | Indicates that details were changed or pseudonyms were used to anonymise data |
| **( )** | Indicates the description provided to anonymise data |
| **XXX** | Words were omitted to anonymise data |
| **-** | Breaking into a sentence by the next speaker |
| **…** | Pause or drawn out words |
| **[ ]** | Indicates noise made, e.g. [laugh], [sigh], [pause] |
| ? | Beginning of utterance by unidentified speaker or questionable text |
| **[inaudible segment]** | Unclear section of the recording |

I: Do you agree to be audio recorded?

P: Yes.

I: Okay, thank you. Umm today's date is xxxx (interview date), we are at xxx (Clinic’s clinic), Interview conducted in Setswana, PID of a patient xxx, RA’s name xxx (interviewer’ s name). Time, we begin at 10:15 am. Okay, Sir umm may you please explain to me about yourself not using your name, that are you working? Where do you live?

P: I am not working, and I stay in xxxx (area name), should I speak loudly?

I: Yes, sir you can shift this side.

P: I am not working; I stay in xxxx (area name). I usually work piece job.

I: Who do you stay with in xxxxx (area name)?

P: My wife.

I: So, do you live in xxxxx (area name) full-time?

P: Now I live with my niece; they took me in when I was sick. I used to live in a shelter.

I: So, at the house, how many are you?

P: It is my niece and her three children.

I: Alright, umm when did you find out you have TB?

P: I found out in May-

I: -This year?

P: Yes-

I: -May, 2022?

P: Yes.

I: Where did you live when you found out you have TB?

P: Umm in xxxx (area name).

I: Okay, so when you found out you have TB, what made you to test or check if you have TB?

P: I did not come to test; I was very sick and I was losing weight very quickly within two weeks, and then I called my niece to inform her that I was sick. They came and took me to xxx (hospital name) where they found out I have TB and HIV. Then I requested a transfer at xxxx (hospital name) to come here because it is near.

I: Okay, umm from where you stay to the clinic, how much is the transport fare?

P: Transport- now I walk to the clinic from the shelter

I: So, is it not far from shelter to the clinic?

P: It is a bit far.

I: Do you always walk to the clinic?

P: Yes.

I: Is there a time you want to take a taxi to the clinic?

P: The time I felt like I needed to take a taxi, I my niece brought me here with an uber.

I: So, you don’t pay your transport fare?

P: Transport- I paid for my self today because I come from xxx (area name).

I: From xxxx (area name) to here, how much is the transport fare?

P: It is R40.

I: It is R40, from there, it is R20 even returning.

P: Yes.

I: Okay, umm when you found out you have TB, how long did it take you to receive a box?

P: Umm I took three months- it is the third month since I started using the box because I started there around 13 May till today, the 7^th^ – that means I will be three months next week or next month using the box.

I: Umm my question is when you found out you have TB ,did you receive the box same time, or you received treatment first?

P: I received treatment first at the hospital, then they gave me when I arrived here to bring the transfer letter- they gave me medication at the hospital and when I arrived here, I received a box same time when I brought the transfer from the hospital.

I: How long did you stay at the hospital?

P: I stayed two weeks in the hospital.

I: Two weeks?

P: Yes.

I: So, did they discharge you?

P: Umm they did not discharge me; I requested the transfer letter.

I: At the hospital- was it far from where you stay?

P: Yes, they discovered it afterwards. Umm they took a sputum first, then found it after two weeks and that is when they initiated me on medication.

I: Okay, since it has been a while using a box, what can you tell me about the box? What is this box and how is it operated?

P: Umm what I can explain to you about the box is that when you forget to drink medication, you work in the morning at home cleaning the yard and you will hear the alarm. You are not supposed to put it inside the bag; you must put it outside so it can report all things and tell you when to take your medication.

I: Okay.

P: I remember I was at the piece job busy working and I had forgotten to drink medication, and I heard it alarming me.

I: So, in other words when you go to the piece job you take the box with you?

P: Yes, I go with the box because I drink my medication at 09:00. So, I start taking my medication at 9 o’clock.

I: So, what time do you start at your piece job?

P: I Start at quarter past eight.

I: Quarter past eight.

P: Yes.

I: So, do you worry that you must take the box to the piece job?

P: Yes, because I am afraid to take the medication before the right time because I must take my medication at 9 o’clock straight everyday . Since having the box , I have changed the time; I was drinking my medication at half past 8 when I was still very sick, but then I saw that I’m struggling to wake up and prepare food at half past eight because it is still early.

I: Umm , umm at the shelter where you live with your niece, how long have you been staying there?

P: Now I stay with my niece in xxxxx (area name), now I’m going back to xxxxx (area name). I went back to xxxx(area name) the moment I started feeling better.

I: So, when did you start staying with your niece?

P: In April-in May.

I: In May when you found out you have TB?

P: Yes.

I: If do not mind me asking, what was the reason for them to take you from the shelter to your niece?

P: My niece said I must come and stay with them so they can look after me. My sister: their mother is the one who brought the nurse and the assistant to look after me because my wife also does piece jobs.

I: Okay, thank you sir, umm by the time you arrived at the clinic, who explained to you about the box?

P: No, they started explaining to me here.

I: Yes, I mean that when you arrived at the clinic who explained to you about the box.

P: The guy brought me.

I: According to your judgment, what do you think this guy does, is he a nurse?

P: I cannot really tell, what does he do?

I: Is it the one with a red T-shirt? {intern}

P: Yes, that is the one I:Umm, Do you still remember the day he was explaining to you about the box- how long did he take?

P:Umm, it took a little bit of time because he just told me that this thing reports to us when you forget the medication and then I took it. He can observe whether you drink medication or not, so when the box start alarming, I run so they don’t say I don’t drink medication because he told me I must drink them at the right time daily.

I: Did you find it easy to understand the way he explained to you?

P: Yes.

I: How did you feel at the time he explained to you about the box?

P: The way he explained to me about the box?

I: Yes, how did you feel the time he was explaining to you about the box, by the time he said here is the box you will put in your medication, then when you start to drink so he can monitor whether you took your medication or not.

P: I felt that I should be serious and look after this box before it reports me whether I took medication or not.

I: Umm the way he explained to you, is there anything maybe that you would like to change from the way he was explaining to you?

P: The way he explained to me; he explained to me what time do I want to take my medication, then I said 08:30 and I had to think about it for a second. When I saw him for the second time, I told him that I could no longer take medication at 08:30, he then changed the time to 9:00 o’clock as I requested. I don’t know how he changed it, but now it alarms at 09:00, not 08:30.

I: Before you saw the box here at the clinic, have you ever seen someone using it somewhere?

P: No, it was the first time seeing it.

I: Since you started using the box, how is your experience?

P: The box is okay because sometimes I forget to drink my medication because I would be at the piece job mostly, and then forget because when I’m there I concentrate on the job. So, it notifies to me that it’s time to drink medication since I put it next to me.

I: What is it that you see that makes it simple to use this box of yours?

P: Something that is simple?

I: Yes, I mean this box, what is it that makes it simple to use, umm how should I put it, what is it that is simple for you to use this box? Umm is it the alarm, or it alarms you at the right time to drink your medication?

P: Yes, that way the alarm on the box tells me that “hey you forgot to take your medication, so that you must drink your medication” and sometimes it tells me that I have 100% and when it alarms me, I drink at the right time.

I: Have you ever experienced difficulties when you did not have your phone with you or having no phone at all?

P: Yes.

I: Did you have a phone when you received this box?

P: Yes.

I: Your phone, is it not giving you any problem when receiving the message?

P: Yes, even now, I don’t receive the message of this box, oh sometimes I receive them after a while though, but not regularly, yes.

I: What does the message from the box reads?

P: The message from the box- when I forget to drink medication ,I received the message afterwards to remind me.

I: Okay, thank you sir, umm have you ever experienced any network difficulties or maybe the box alarmed you-you opened the box and took your medication, but when you arrive at the clinic, they tell you there is this day where you didn’t drink your medication?

P: No, it never happened.

I: Umm your piece job, do you change your location and work in different locations or is it a constant location?

P: It is in constant location.

I: So, when you go to the piece job you take the box?

P: I take my bag and the box because I have this TB medication and the high blood medication, so I drink them at the same time.

I: Okay, this box does not give you any trouble at work?

P: No, it doesn't give me any trouble; I put my medication inside the bag and when I arrive at work, I take my medication inside the bag.

I: When you received this box, who did you explain to first when you arrived at home?

P: That I have the box?

I: That you have the box.

P: My younger sister; the one I said she usually assists the nurse. She is the one I told that I have the box, I said “ here it is, take,” she observed it.

I: So, how was her reaction that you brought the box at home?

P: They were just puzzled that this box reports sometimes- I opened it and showed her the indicators that if I don’t drink my medication this box reports me.

I: The indicators that you are talking about, how do they look?

P: They are green like so.

I: You can take it out if you don’t have a problem.

P: (Patient opens and close the box demonstrating to the Interviewer)

I: So, have they ever explained to you about those indicators?

P: They have explained to me that the battery will get depleted, and it will become red.

I: So, is that all they have explained to you about those indicators?

P: Yes.

I: Umm when you find out while in hospital that you have TB, was it you who explained to your relatives that you have TB?

P: Yes.

I: How was their reaction when you told them that you have TB?

P: One time they came to see me and asked me that “what do the doctors say you have,” then I told them that it TB.

I: So how did they react on the news?

P: They just said no- they forced me to drink medication on time and they told me that I must not stress because treatment ends after six months and I understood.

I: So, are you troubled using this kind of a box?

P: No, I am not troubled.

I: Is there a person who has ever asked you about the box?

P: No.

I: They haven't asked you anything?

P: It’s the first time I see it.

I: I mean when you go to your piece job, then the box alarms you. Did anybody ask what is that thing that you are carrying?

P: I showed them the box.

I: So, what are they saying at your piece job?

P: They only observed it.

I: Haven’t they asked you anything?

P: No, because I told them that this box notifies me when I have to take my medication, so it a reminder.

I: Umm is there a time where you were seen by someone opening the box and taking out medication, and then the person wanted to know about the box?

P: No unless it during the day.

I: At your house- at your niece’s place, do they know that this box is working or how it functions? So, do they tell you when the box is ringing and they also remind you that it time for you to take your medication while maybe you are outside?

P: Yes.

I: When you explained to people you stay with about the box, did you find it simple to explain to them?

P: I found it very simple because it was the first time seeing it and it was their first time too.

P: Umm may I please ask, in your family, is it only you who has TB or was there anyone before you who had TB or who has TB?

I: I’m not sure, but my father was coughing, though he didn’t go for TB testing neither did he go to hospitals, but the way I see things. I think he had it.

P: Umm have you ever opened the box more than once- you know that you must open the box once when you take out your medication. So, have you found yourself opening the box more than once a day?

I: In the beginning when I showed them the box like I just did now.

I: Oh, it only that time?

P: Yes.

I: You only open it when you take out your medication?

P: I only open when I take out the medication and that’s it.

I: Besides your TB medication, is there anything else that you put inside the box?

P: No.

I: You only put medication?

P: Yes.

I: Your medication- when you took it at the hospital, were you administered by the sister(nurse)?

P: Yes.

I: Has it ever happened that you take your medication home, and it is you that drink the medication on your own.

P: Pardon.

I: I mean when you came out of the hospital.

P: The day I came out of hospital, I brought the transfer letter and then they gave me the box same time and even added TB medication inside the box.

I: My question is when you received a transfer letter, did you get out of the hospital and went home, or you came out of the hospital and came straight to the clinic?

P: I came straight to the clinic.

I: Okay, that was my question, since you have been using this box, what is it that helps you the most when using the box. Is it the alarm or the explanation you received from that boy who explained to you about the box?

P: It is mostly the alarm.

I: Have you ever experienced any problem like maybe if you have to go with it to the piece job?

P: I have never experienced any problem.

I: Since you received the box, have you ever visited somewhere?

P: No, I have never visited anywhere except being at my niece’s place.

I: Do you take it with you to your niece’s?

P: Yes, I go with it.

I: If it happens that you should perhaps go home, would you take the box with you?

P: Yes, I can go with it.

I: Do you think it will not be a problem?

P: No, it won't be a problem.

I: So, aren’t you disturbed by the fact that when you arrive at home people will ask you about it?

P: I have already explained to them at home that I have TB, so I will just show them the box. Tell them that it has my TB medication, and it reports when it’s time for me to take the medication. So, I will just tell them that they must not be surprised because it reports for me.

I: Okay, since you received a box, you said that you received an SMS that say you must drink medication?

I: Sometimes they just tell me that I must take my medication properly and they say, “thank you,” things like that.

I: Have you ever received a call from the clinic?

P: No.

I: They never called you?

P: They called me at the hospital asking where do I take my treatment.

I: Okay, concerning the box, haven't they called you?

P: They have never called me.

I: Is there a person who came to visit you at xxxx (area name) just to check how you are doing with your medication?

P: No.

I: Is there someone who came to check up on you at your niece’s place?

P: No, they have not come.

I: If it happens that when you are at your niece’s place and someone from the clinic comes to check up on you, how would you feel about that?

P: I won’t have a problem.

I: Would you have a problem if people from the clinic call just to check up on you?

P: I will not have a problem.

I: You will not have a problem?

P: Yes.

I: Are there any barriers that might prevent you from taking medication, for instance, your religion or your culture?

P: No, the thing that might prevent me is food. If it’s nearly nine and I don’t have breakfast like porridge and things like because it's by then that I prepare something to eat. So, only that can prevent me.

I: So, things that can prevent you is food before you can drink your medication. So, umm since you have started using the box till now, are you happy that you had an opportunity to drink your medication using it?

P: I am so happy.

I: When you look at this box, how did it help you to take your medication?

P: It helped me because mostly I forget as you might find that I am busy outside, so it alarms me once, then I hear the alarm and I just run to drink medication.

I: This box- what makes it easy to use? Is the volume of the alarm fine?

P: The volume of this box is not fine because the distance from here to there, you can still hear it, but once you pass there, you will never hear it ;the volume will be down.

I: So, what would you like to happen to the volume of the box?

P: They must increase the volume a bit so it is audible, but if they could increase it a bit even if you are at distance, you could hear it.

I: So ,looking at this box, do you have any suggestions to improve it?

P: The box is fine the way it is, and it is also written on the paper they gave me that the box must not stay inside the bag so you can hear the box alarm when it rings because you might not hear it inside the bag.

I: So, at your niece’s place ,where do you put the box?

P: I put it where I would be sleeping.

I: Where you would be sleeping?

P: Yes.

I: So, in xxxx (area name), where do you put the box?

P: I put it where I sleep.

I: So, when you are at your piece job?

P: I put it where I change.

I: So, where you put it at your piece job, are you able to hear it when it rings?

P: Yes.

I: The SMS that you receive, what do you like about them?

P: What I love about them is that sometimes I get distracted, and my wife just tells me the SMS is from the clinic, they say while I am just thinking that it is a phone call, but it is about the box.

I: You said they’ve never called you, right?

P: Yes.

I: They've never visited you, right?

P: Yes.

I: When you found out that you have TB, did you receive counselling?

P: No.

I: Didn’t they give you counselling?

P: Yes.

I: So, by the look of things, would you love to receive counselling?

P: No ,counselling is not necessary; I understand about TB.

I: Umm according to your perception, a person who received the box, received an SMS, received a phone call, receive a home visit. Which one among all these intervention do you think is more important to a person using a box?

P: Pardon?

I: I mean for a person using a box like you, receiving SMSs and again receiving a phone call and a home visit, among these interventions, which one do you think is important to a person that takes treatment? Is it an SMS, a phone call, or home visit?

P: It is a home visit so that they can come and tell you personally looking at you.

I: Since you have been using the box ,what is it that you see that we can fix in our project when issuing the boxes?

P: Perhaps you can fix a speaker so that when I open the box showing it to a person how it functions, it doesn’t make a sound; it must just show the lights when am showing the person how the box function. So, that it mustn’t appear that I’m opening the box many times and even the volume of the speaker must be adjusted.

I: According to your perspective, do you think that all people who have TB must be taught about the box?

P: Yes, I think it is very important.

I: So, don’t you think that since you are using the box maybe you can show people around how does the box operate and teach them since you have been using it?

P: No, I only show people who are close to me only.

I: Those close to you only?

P: Yes, those that are close to me.

I: : Umm we are coming to an end of our interview now, do have any suggestion about the box?

P: This box?

I: Yes.

P: You see if they can reduce the size of the packaging for the pill packets so that the box won’t be this big for it to be portable.

I: Do the pills fit in the box though?

P: Yes.

I: And do you have I final comment when it comes to the SMS, a phone call, and a home visit? Is there anything that you would like to mention about the SMS that you receive, are you happy to receive them, do you still want them or what?

P: I am happy to receive the SMS.

I: Would you like for a person to call you just to check up on you at home?

P: A phone call?

I: Yes.

P: No.

I: Would you like for a person from the clinic to give you a home visit?

P: Home visit is not necessary.

I: Okay, thank you sir-

P: -That person will not find me at home sometimes.

I: If you don’t mind me asking, what time do you knock off at your piece job?

P: I knock off at 3 o’clock.

I: 3pm?

P: Yes.

I: Do you go in daily, or you only go certain days?

P: Certain days.

I: Okay, thank you for answering all my questions as I have explained there are numbers of people inside the consent form, if ever you have any questions, you can contact them so that they can answer your questions.

P: Umm this thing- is there anywhere where it speaks about the box?

I: No, this consent form is about us asking you to do an interview with us and if you have a question about the box, you can ask a person that is mentioned in your ICF.

P: The time they gave me the box and the informed consent paper, so when I was at home sitting I read that informed consent and it said the box must not stay inside the bag, but now the problem is that I don’t know where did I put the paper. So I am asking if is it possible for me to receive it again.

P: Okay, I will check if ever I will be able to ask it for you, thank you for your time sir.
