# Supplementary material for: Acceptability of Digital Adherence Technologies to support people with drug-susceptible TB in South Africa
Source: PLoS One. 2025 Sep 24;20(9):e0332103. doi: 10.1371/journal.pone.0332103 (PMC12459780; doi:10.1371/journal.pone.0332103)
Supplement: S4 File — (ZIP) [file pone.0332103.s004.zip › S4 Transcripts/PwTB/IDI 32_PwTB.docx]

**TRANSCRIPTION NOTATIONS**

| **Label Key** | **Meaning** |
| --- | --- |
| **I** | Start of each new utterance by the Interviewer |
| **P** | Start of each new utterance by the Participant |
| **N** | Note taker |
| **{ }** | Indicates that details were changed or pseudonyms were used to anonymise data |
| **( )** | Indicates the description provided to anonymise data |
| **XXX** | Words were omitted to anonymise data |
| **-** | Breaking into a sentence by the next speaker |
| **…** | Pause or drawn out words |
| **[ ]** | Indicates noise made, e.g. [laugh], [sigh], [pause] |
| ? | Beginning of utterance by unidentified speaker or questionable text |
| **[inaudible segment]** | Unclear section of the recording |

I: Greetings

P: Good morning

I: Do you agree to be audio recorded?

P: Yes I agree

I: Okay thank you, today is the xxxx (interview date), we are at XXX (clinic name) we are doing the interview in Setswana , PID of the patient is xxx, interview it's done by (interviewer’s name), the time that we are starting with it is 10:03 AM. Umh daddy {referring to an elderly male participant} without the waste of time and without saying your name can you please tell me about yourself where do you live things like that?

P: I stay in (participant’s address at a shelter for homeless people) that is the address

I: When you start living at the shelter how long have you been living there?

P: It’s been xxx (number of years)

I: xxxx years?

P: Yes

I: Before you moved to the shelter where were did you stay?

P: I stayed in xxxx (Province name)

I: XXXX (Province name)?

P: Yes

I: When did you find out that you have TB?

P: It’s a while now I think I’m coming here for the third time at the clinic

I: When did they start giving you TB treatment?

P:For today?

I: Did you have TB before?

P: Yes I once had it then I got healed then I heard it for the second time I got healed so it’s for the third time that I have it.

I:When was the first time you had it? ?

P: For the first time eish I forgot

I: Were you staying at the shelter the first time you had TB?

P: I was not staying at the shelter

I: Where were you staying?

P: I was staying at xxxx (another Province)

I: For the second time when you had TB?

P: For the second time I was staying at the shelter

I: For how long had you stayed at the shelter when you had TB for the second time?

P: For the second time I think I had stayed at the shelter for about three months

I: Three months

P: Yes

I: So right now, it’s for the third time

P: Yes its for the third time

I: The third time when did you start taking treatment?

P: For the third time, it's a month now it was June though I don’t remember the date but today it makes a complete month

I: How long do you take to move from the shelter and come to the clinic?

P: About 5 minutes

I: Five minutes, do you a taxi or you walk

P: I walk, when I leave here I just jump xxx (street name) then I’ve arrived at the shelter

I: What made you to come to clinic?

P: I was sick coughing my lungs were painful and I was losing strength, I came to the clinic they showed me my X-ray and told me it doesn’t look good, I have got TB they must give me treatment.

I: Can I ask daddy when you stay at the shelter do you do piece jobs somewhere or are you working?

P: I work piece jobs

I: When you go to your piece jobs do you take the box with you?

P: Yes I take it with me because I have registered that I’m going to take my medication at 7:00 o’clock all right and I leave very early in the morning so when I arrive there I arrive around 7:00 AM so that when it alarms me I have it with me

I: At your piece job what time do you start working?

P: At 7:00 o’clock

I: If you don’t mind me asking you when you come here from (province name) was it due to work or what?

P: I was just looking for piece jobs

I: What happened for you to end up living at the shelter?

P: I didn’t have money for transport to go back home, I failed to purchase a bus ticket then I saw that I must find a place at the shelter where I could sleep when I’m going to my piece jobs.

I: Do you often go home?

P: I go home month end

I: Okay so when you go home are you able to go with a box?

P: I go with it I don’t leave the box behind every day I must take my medication in the morning, each and every day at 7:00 o'clock it alarms me so I must go with it I can't leave it behind.

I: Before you come to the clinic and they give you the box have you seen it before someone using it?

P: Not at all it was my first time seeing it here.

I: Who explained to you about the box?

P: It was the assistant of Mr XXX(nurses name) who works here

I: Do you still remember how long did the explanation take about the box?

P: Uhm it took about five minutes

I: When the assistant explained to you did you find it simple to understand?

P: I found it simple because I follow the box’s rules when it alarms me at seven I must take my medication.

I: So how did you feel when they showed you the medication box ?

P: They told us others dont take their medication they said it is better when you have a box because it reminds you hour by hour, at seven o’clock when you should take your medication it reminds you everyday during that time.

I: When you had TB for the first time what time did you take your medication?

P: Those ones-

I: When you had TB for the first time

P: Uhm I drank them at seven in the morning

I: Is there a time when you forgot to take your medication?

P: I did not forget even now i dont

I: You did not forget, did you finish the treatment?

P: Yes I finished it then they discharged me

I: Okay the second treatment did you finish it?

P: I finished it and they discharged me

I: Even then you did not forget?

P: No I did not forget

I: For the first time and second time what reminded you to takeyour medication?

P: I constantly check the time that now it is the time to take my treatment

I: The way the assistant of xxxxx (nurse’s name) explained to you about the box uhm how should I phrase it, is there anything you would like to change about how the box was explained?

P: No i dont want to change anything

I: So the way he explained to you is it fine?

P: Yes yes it is fine

I: Since you have been using the box what is simple using it?

P: The simple thing it reminds you to take medication it helps right there

I: Have you encountered problems using the box?

P: I have not encountered any problems

I: Do you have a phone?

P: No i dont have a phone

I: You dont have a phone okay, is there a time where you take your medication but when you are at the clinic they say you dont take your medication?

P: No even now they said i am 100% they checked on the phone how I take my medication

I: Okay so the 100% you are talking about have they shown you?

P: Yes yes

I: Um in your piece jobs do you work one place or they sometimes take you elsewhere?

P: I work one place

I: Umh may I go back a little bit first time you had TB you were in XXX (province name) with your family how did they take it that you have TB?

P: Umm I was coughing all right then I explained to them that I have a problem all right and I said I’m going to consult at the clinic then when I arrived they took an X-ray then when I arrived here they told me my X-ray is not good I am sick with the sickness of TB and they told me that they are going to treat me for nine months.

I: Okay when you were at XXX (province name) people you were living with did they test for TB?

P: They did not test but they are okay

I: They are okay?

P: Yes

I: When you had TB for the second time the people that you live closely to at the shelter did they test for TB?

P: they are also all right

I: Even now are they still all right?

P: They are all right

I: Are there people who ask you about the box?

P: A lot of people have never seen it

I: They never seen it, what is the reason that they did not see it?

P: When I finish taking my medication I put it in the locker

I: Is there a person who have seen you opening the box taking your medication at the shelter ?

P: There is one person who has seen me and it seems like he was also using the box but I don’t know where got it so he seems to know about it

I: Did he ask you about it?

P: Who, him?

I: Yes

P: No he just told me that when I don’t follow the rules of the box at the clinic they will show me my records because this box records every time I take the medication then it ended there

I: Um you said it for the third time you have TB?

P: Yes

I: When you evaluate the first time and the second time and even now, is using the box helping you to take your treatment?

P: Yes the box helps me

I: In your family is there anyone move once had TB?

P: Yes my younger brother had TB but unfortunately he passed away

I: Did he take treatment before he passed away?

P: Um he never took treatment he was sick he had a stroke then developed TB

I: Did you ever open the box many times in a day?

P: No I open it once when it alarms me then I take my medication I close it and leave it

I: So inside your box what do you put in?

P: Only my medication

I: Don’t you put other things?

P: No only my medication

I: So where do you put your box?

P: In the locker

I: In your locker?

P: When I come from work I put it in my locker in the evening because when I leave in the morning I take it and put it in my bag

I: At your workplace where do you put it?

P: I put it where I put my things

I: You put it where you put your things?

P: Yes

I: Okay what is it that you see that is very helpful when you use the box?

P: It is time

I: Time?

P: Yes because every time it alarms me to take my medication

I: When you go with your bag I mean the box does it give you a problem?

P: No it doesn’t give me problems

I: You have have no problems, um was it ever explained to you that when you receive this box and you have the cell phone you receive messages?

P: Yes they mentioned that when you have the phone it is just that myself I don’t have a phone, Mr XXX (nurses’s name) said we will connect through the phone but I don’t have it

I: So we have an intervention often SMS or phone call and sometimes they do home visit

P: They never visited me and I don’t have a phone SMS requires a phone

I: According to your perspective a person who does not have a phone will it hinder him to use the box?

P: Not at all even if you don’t have a phone you can still use the box

I: Have you ever encountered things that prevent you from taking your TB medication , because of the believe in your culture maybe it doesn’t allow you to take medication you must go see a traditional doctor?

P: I don’t go to traditional doctors

I: You don’t go to traditional doctors or maybe your belief in church does it prevent you?

P: They don’t prevent us to take medication at church

I: Since you have started using this box of ours have you not forgotten to take your medication?

P: No I don’t forget when I sleep I put the box near me and when it alarms me at 7:00 AM and I’m still asleep I can hear it

I: Um have you ever taken your medication a bit later after the alarm went off?

P: No I haven’t

I: When the box alarms you do you take your medication at that time?

P: Yes and when I’m finished I close the box so that it can stop flashing

I: So if I may ask you that since they gave you this box and you are using it are you happy about it?

P: Yes it really helped me a lot

I: It helped you a lot?

P: Yes

I: Umm the way the box it is right now do you think the volume is okay?

P: Yes it is okay-

I: Or they should increase the volume a little bit?

P: The volume is fine it is audible

I: Is this size fine?

P: The size of the box is okay, it is portable

I: Is the material okay the way it is?

P: It is fine it doesn't give me problems

I: I mean the material of the box is it breakable or is it hard?

P: The material is strong it won't be easily broken

I: If you had a phone would you love to receive the SMS’s that comes with a box or you wouldn't want to receive them?

P: If I had a phone I would love to receive an SMS because it is connection

I: If you had a phone would you love that the people at the clinic to call you?

P: Yes i would have loved to but right now I don’t have a phone

I: Would you have loved for people to come pay you a home visit where you are staying?

P: At the shelter?

I: Yes

P: During the week I am not available at the shelter I am at work i go early in the morning and I knock of at 5 PM in the evening then I come back on Friday evening

I: Do you stay at your piece job Monday to Friday?

P: Yes but during the day when I knock off in the evening I go back to the shelter

I: What time do you knock of at your piece job?

P: I knock off at 5 PM

I: When you knock off at 5PM what time do you arrive at the shelter?

P: I arrive at the shelter around 5:45 PM

I: Alright If the people came to visit you during the time that you are available would you like that they came to check on you or not?

P: If I was around during the week i would agree for them to come but because I am not available that is the reason we are unable to meet

I: But if they were able to come how would you feel?

P: I would be okay if they come check up on me

I: When you found out that you have TB did you receive counselling?

P: Who here at the clinic?

I:Yes did Social Workers sit down with you to counsel you

P: No they did not

I: Did they not advise you?

P: No they did not

I: When you got TB right

P: No I just arrived and they told me my X-ray it doesn’t look good

I: Would you like to receive counselling?

P: Aah counselling won’t help me because I have already started treatment and I am feeling better

I: Okay to a person taking treatment which one is important for that person to receive when using the box according to your observation, is it sms that the person receive to remind the person to take medication and also drink water and fruits, phone calls and home visits?

P: The one that is important is an sms and a phone call when you have a phone and sms they teach you the diet what type of food you should eat

I: Um since you have been using this box is there something that we can do better with this box or the project?

P: I see this box okay

I: All right by your observation must we educate people living with TB about the box?

P: Yes, it will remind them because some of them forget to take their medication when it alarms you must open it and take your medication so some of them forget sometimes they do take sometimes they don't so it will help them the most about the time to take their medication.

I: If I ask you of all things we have been talking about is there anything before we close that you, can explain what is this box, and what you see about the box that is right, what do you love about the box and what you don’t like about the box?

P: What I like about the box is that you always take medication consistently at the same time 7 o’clock you take your medication that’s what I like the most about it that you don't forget.

I: Okay so umh you said you don't have a phone right so according to your observations the SMS receiving phone calls and home visit what can you say about them?

P: Um it can be all right if you visit the patient where he lives to remind them and tell them about things that they should use even the SMS it is all right including those phone calls

I: Uh we have reached the end of our interview daddy thank you for your time that you gave to us Umm in the consent form that I explained to you earlier before we start there is phone number of people that will answer questions if you have them you can ask them but if you have a question right now you can ask it?

P: I don’t have a question

I: Okay thank you daddy interview end it 10:27 AM um can I ask a question the last question since you received this box have you ever lost it where you live at the shelter?

P: No I haven’t lost it

I: So where you put it is it safe?

P: Yes it is safe

I: do you lock your locker?

P: Yes

I: Keeps the key?

P: Myself

I: Is it only you who keeps the key okay thank you Umh the people that you stay with at shelter have they complained about the alarm of the box?

P: No my alarm is not that loud so it is only me who hears it when it alarms in the morning so it doesn't make noise

I: Our interview ends at 10:29 Am.
