# Supplementary material for: Acceptability of Digital Adherence Technologies to support people with drug-susceptible TB in South Africa
Source: PLoS One. 2025 Sep 24;20(9):e0332103. doi: 10.1371/journal.pone.0332103 (PMC12459780; doi:10.1371/journal.pone.0332103)
Supplement: S4 File — (ZIP) [file pone.0332103.s004.zip › S4 Transcripts/PwTB/IDI 33_PwTB.docx]

**TRANSCRIPTION NOTATIONS**

| **Label Key** | **Meaning** |
| --- | --- |
| **I** | Start of each new utterance by the Interviewer |
| **P** | Start of each new utterance by the Participant |
| **N** | Note taker |
| **{ }** | Indicates that details were changed or pseudonyms were used to anonymise data |
| **( )** | Indicates the description provided to anonymise data |
| **XXX** | Words were omitted to anonymise data |
| **-** | Breaking into a sentence by the next speaker |
| **…** | Pause or drawn out words |
| **[ ]** | Indicates noise made, e.g. [laugh], [sigh], [pause] |
| ? | Beginning of utterance by unidentified speaker or questionable text |
| **[inaudible segment]** | Unclear section of the recording |

I: Do you agree to be audio recorded?

P: Yes, I do agree.

I: Ok. Uh date uh: xxxx (interview date) . Location xxxx (clinic name). PID xxx. Time: 12:49PM. Language used: English. Ok. Now when did you start using the label?

P: Uhm I started this year around January.

I: Ok. When you say this year around January, can you please us elaborate for me?

P: I think it was the first week of January, after my second x-ray.

I: Oh oh oh ok. Uh before you tested for TB, what made you test TB?

P: Ok, I started coughing a lot. And it lasted about a year and a half, so I went to doctors but then they couldn’t find what’s wrong. Firstly they thought that it was flu, then uhm they gave me treatment for flu, only to find out that the cough doesn’t stop. So I tested for Corona and then the results came back negative. Then I tested again for Corona [laugh] then they came back again negative. Then I consulted again, then that’s when I tested for TB, only to find out that it was TB.

I: Mmm. You did mention coughing as one of the symptoms that you experienced, so other than coughing, what are other symptoms that you experienced?

P: Fever, loss of appetite, and also loss of weight.

I: Oh loss of weight.

P: Yes.

I: So now, in a nutshell, can you describe or explain your overall health before you started TB? How were you feeling before you started TB treatment?

P: Uh I couldn’t sleep actually. Because my body was sore, and coughing all day. The whole night, it was so difficult. So I couldn’t even cope at school because every minute I was coughing. So it was so difficult.

I: Oh ok. So before I- uh before I can go any further with this interview, can you in a nutshell just tell me, who are you? Tell me about yourself a bit.

P: Ok. [Zipping bag] Firstly, uh I’m a student. A second year student doing teaching and I also have a diploma in electrical engineering. And also, I love working with people. I love I love socialising a lot. Plus uhm I’m a media influencer.

I: Mmm. Ok. No, that- that’s good to use. So just so I have or just so that I know you like uh like how you travel to the clinic?

P: Uh I take a taxi.

I: Uh to be specific, how many? [Car hooting]

P: Uh only two.

I: And then how much do you spend on a single taxi?

P: Uhm R10 to come and also R10 to go back. So it’s like R20.

I: Oh, ok. So uh to my next section, uh, can you tell me what do you know about these Label? You can hold it.

P: Ok. Uhm. Ok uhm firstly, this Label is attached to my medication. Ok then each and every single day, I have to take my medication. After taking my medication, I have to send this code right here to this number and then I’ll get a confirmation that I took my medication. And also if I forgot to send this code, then I’ll get an sms to remind me that I have to take my medication. And then I’ll just remember “oh! I took my medication but then I forgot to send the code”. So I’ll send a code then get a confirmation and then the confirmation is just a confirmation of appreciation for taking my medication.

I: Oh ok. So prior to receiving an sms, how do you feel about receiving an sms confirming that you took your medication?

P: Ok. I feel good actually, because uhm I know that there are- there are people who care about me. Not only my family, but then also the healthcare workers, like they do care about me taking my medication, they do care about my health. So when I get a sms as a reminder, then I just remember, “ok, so they do care that I do take my medication or not”. So I have to send a code, then to show that “ok, I’m fine. I took my medication”.

I: Ok. So the day you started your TB medication, is it the same day you started using the Label?

P: No…

I: Ok. Just so I get an understanding, you took your medication before the label?

P: Yes, I did.

I: To be specific, how long did you take your medication without the label?

P: 1 month.

I: Ok, so 1 month. And then only after one month you got the labels?

P: Yes.

I: Oh ok. Thank you. Uh still on the label, who told you about the label?

P: Uh it was Sis XXX (intern’s name).

I: So briefly, how long did it take her to explain the label to you?

P: Uh, it took- I think around 30 minutes to explain everything and to assist me and to show me how it works. And also to show me what happens after I sent the code.

I: Oh ok. From the way or- judging by the way she informed you, do you feel uh is there something that she did not tell you about the label?

P: No, she told me basically everything and she showed me what to do and when to do it and also uhm she helped me with my schedule so that I know ok that I can send the sms anytime, because sometimes I’m at school. Sometimes I’m writing my exams, so she basically showed me everything and directed me towards sending the code and receiving an sms.

I: …Uhm…when the RA informed you…from the way or the way she informed you about the label, is there something you would like to change about how you were informed about the label?

P: No. Actually uhm she was pe- she was pretty thorough, so uhm I love the way she explained everything. Uhm I understood every single thing including the illustrations, so uhm I’m 100% satisfied.

I: Ok. So uh remember during the consenting form, I did mention that uh this interview basically- we are going to look- I’m going to ask you directly- get to know your experience, your level of satisfaction, your thoughts as well as your perception about the box. So now-

P: I do remember.

I: From your experience, what’s so easy to use about the Label?

P: Ok. Sending the code, obviously. That’s quite easy because uhm it’s free, first of all. And I can send it anytime. So sending the code is pretty easy. I didn’t experience any difficulties with sending the code or using the Label.

I: Oh, oh ok. Ok. So can you kindly tell me who do you stay with?

P: I stay with my mum and my two little brothers.

I: Oh ok. So can you describe to me what was your parents or your mothers’ reaction when you told her that you are- you have TB?

P: She was shocked, but then uhm she was very, very supportive. Uhm she made sure that I go for my check-ups, I go for the x-rays and I take my medication each and every single day. Uhm she kept on reminding me, monitoring me, and making sure that I eat the right food, and also drink the right stuff.

I: Oh ok. So before I move to my next question-

P: Ok.

I: In a nutshell, can you kindly tell me about TB.

P: Ok, what I know about TB is that uh i- it changes everything about your life. Uhm it changes your physical appearance and also how you feel emotionally, because firstly, you started losing weight, you start losing appetite, you start coughing every single day, and sometimes you just cough blood. So it’s- its difficult actually, it changes everything, the way you used to live and you have to take some time off people. The people that you are- used to socialise with, network with, because uhm you are scared of infecting them, so you have to take your time off for some certain time. So TB uhm is so difficult, but then what I know is that it is curable, it is treatable. If you take your medications right, the coughing will stop within a month or two months, and also the symptoms like they will lessen up and then soon you’ll be back to your old self. Being healthy and going back to the way things were before you got infected.

I: Oh. That’s wonderful to hear. But you said TB changes your life, so in a nutshell, how does it change someone else’s life?

P: Ok. Firstly, it limits your movement. Uhm you know that you are not supposed to be always in a crowded place, because like you can infect other people. And also, you have to take off yourself, make sure that the windows are open early in the morning. You have to exercise even though sometimes- Ah! Exercising, ah. Really boring. But then you have to do it in order to keep healthy. And also, you have to limit some of the food that you used to eat, especially the junk food. Then you have to stick to a healthy-diet plan, like eating vegetables and fruit, which is- ah sometimes dull. But then, ah you get be- the hold of it [laughs]. And also, you have to drink lots of water. Ok? Uhm I love cold drink and juice, but then I- ever since I started taking my treatment, then uhm I stopped on drinking cold drink and then just drank 100% juice and also water. Lots of water.

I: Oh ok. Uh earlier on, you did mention that you are a student.

P: Yes.

I: So…what time do you take you TB medication at?

P: Oh I take it 6 o clock in the morning.

I: Mmm ok….So just so I can know you, uhm uh are you working?

P: Uhm I’m part of the tutoring centre team. Uhm we tutor learners after school and also I’m in a middle of my practicals.

I: Ok. So how does uh taking tb medication affect your work schedule?

P: Actually it doesn’t. It doesn’t at all.

I: How?

P: Uhm ok, I wake up early at 5 o’ clock, and I make sure that I take my bath and prepare everything. Then 6 o’ clock, I take my medication, then after taking my medication, uh I leave for school around 7. So actually it doesn’t interrupt with anything.

I: Mmm ok. That’s- that’s wonderful to hear. So you said your mum is very supportive of you in this TB medication journey?

P: Yes, she is. Very much supportive.

I: So other than your mother, who else did you tell that you are- you have TB?

P: Uh my uncles and also, my father.

I: Oh ok. Ok. Just so I can understand their reaction, how did they feel that you are on TB medication?

P: Ok, my uncles were- were very, very supportive. Uhm they understood and- and also they checked up on me every now and then. Uhm the only person who had difficulties is my father, because uhm he’s very, very traditional. So he even suggested witchcraft, those kind of stuff. But then at the end, uhm he ended up accepting and also understanding and also being there.

I: Oh ok. So now, when you told them about the Label, what did they say or how did they feel about the Label?

P: Actually my mum thought that it was a great idea because uhm sometimes she’s working so she can’t monitor me from work. Since the label is there it can monitor and remind me if I forgot to take my medication or if I forgot to send the code. So she was very happy that there is a code to send to keep on monitoring me and to making sure that I take my medication correctly and on time.

I: So, have you ever sent an sms more than once a day?

P: Yes, I did.

I: Uh…ok. In a nutshell, can you, you know, like describe to me what led to you sending an sms more than once a day.

P: Ok it was last month. I think uhm it was about two weeks, where I sent more than one sms, because I didn’t get a response. Ok, on the first day, I did send an sms then didn’t get a response. Ok I left it, then on the second day, I got a message saying that I didn’t take my medication, whereas I sent a code to confirm that I took my medication. And then they said that a healthcare worker will contact me. Then on the second day, it happened again. I sent a code, they didn’t respond. So I had to send about three codes bef- before I get a confirmation. Then it happened again and again. I think it took about two weeks, where I sent more than one sms, so that I can get a response confirming that I took my medication.

I: So, when such events occurs, or when you experience uh these uh sending multiple times a day, have you –did you take your- you receive uh these uh difficulties whereas you took your medication?

P: Yes.

I: Ok, so how do you feel receiving an sms that you did not take your medication, whereas you indeed took your medication?

P: Ok. Firstly, uhm as a student, I do understand that sometimes like there are high volume of sms’s that the department is receiving. So uhm may the system was down. It usually happens even at school, it happens that you send an email and then you get a response after a long time. It’s because the are high volume of sms’s that are being sent at the same time.

I: Ok. So from your experience, can you describe or tell me uh what is it helpful about using the Label? How is the Label helpful?

P: Ok. Uhm as I said before, uhm the Label keeps tabs on you. It monitors uhm whether you adhere to your mediation or not. It reminds you if you forgot. And also uhm the label is free of charge, so uhm it’s totally helpful because like you are being guided, you are also being appreciated. When you appreciate someone for doing something right, like it gives then confidence like to keep on doing that thing. Because it’s the right thing to do.

I: Mmm ok. When sending an sms to- to the- to the number- when you sending the code to the number, has perhaps the RA told you how they see that you did not take your medication or you took your medication?

P: Yes, they did. Uhm they showed me uh on the tablet. Uhm there’s a bar that shows whether you took your medication or not. If you didn’t take your medication, the bar doesn’t move, but then when you take your medication, the bar moves. And then uhm it will reach a point whereby uhm you get something like a- a reward on that bar, showing that uhm you took your medication until you are finished your treatment.

I: Ok. So when you saying- when you see a bar…the bar you are talking about, do you perhaps refer to the adherence calendar?

P: Yes.

I: Tell me about the adherence calendar.

P: Ok. When you take your medication, then when you send a code and then the bar moves uhm, its- it’s quite a long bar. Ok? It moves to show that “ok, on this day, you took your medication”. And then, when you didn’t take you medication that day, uhm the colour of the bar changes on that specific day. Uhm when you took the med- when you take the medication, the bar uhm- the movement is red. If I’m correct. And then when you haven’t taken your medication, I think the bar turns green on the day that you haven’t taken your medication.

I: Mmm. Ok. So your experience, how does it feel seeing your adherence calendar? Saying like you took your medication for the whole month?

P: It feels good, because uhm actually I know that I- I did the right thing. Uhm because I- I’m not only- only doing this for the healthcare workers. Uh I’m doing it for myself, because it’s my health. If I don’t take care of it, who will take care of it? So I have to make sure that I do the right thing each and every single day until I get completely healed.

I: Ok. Earlier on, you mentioned the healthcare workers being caring and supportive.

P: Yes.

I: Besides your parents or your mother, the Label, and the healthcare workers, what any other s- uh what other uh groups support you in taking your TB medication, other than your parents, the Label, and the healthcare workers? Your support system.

P: Ok. I’ll say my friends and also uhm they teachers at school. And then like uhm they are pretty much concerned and also every day they ask me uhm “how do you feel today? Are you ok?”. Uhm “did you take your medication?”. Uhm “is there anything that you need?. So uhm I mean like they- they keep me moving. They keep me on the fighting spirit, so they give me give me confidence that “ok, I can beat this TB”. I mean like I’m stronger than this TB and I will conquer this TB.

I: You mentioned your friends and colleagues knowing about your TB status?

P: Yes.

I: Day one when you told them that you have TB, how did they take it?

P: Ok, uh my best friend was emotional, too emotional. Very sad. But then she kept on being supportive always, and even though I told her sometimes that I don’t need visitors, but then she- she would come just to check on me. And also, uhm the teachers at school, they were very, very supportive. And then uhm they send messages and also they wished me a speedy recovery and also told me if I need anything, I should not hesitate to ask or tell them.

I: Ok. Ok. Thank you. Prior to telling your friends about your TB status, why did you feel the need to tell them about your TB status?

P: Ok, uhm as you know, uhm you- you can’t go through something this hard, this big alone. I mean, you need people around you to support you, to encourage you. So I felt like they needed to know. I owed them that much. And also for them to be careful and also to alert them that TB is real. And also to let them know that tb is curable. I mean if they see a TB survivor, uhm in their closest circle- and also they know that “ok this disease is curable. This disease is treatable”. And also that that will give them power to face whatever that comes their way.

I: From your experience, what can you say is difficult about using the Label?

P: Ok. Except for the not responding to some of the sms’s, uhm there’s nothing difficult there.

I: There is something that we as ASCENT, or we as XXXX (organisation name), we call refer to as the differentiated care model.

P: Ok.

I: So this model is – it’s about receiving a reminder sms to take your medication, if you did not take your medication for the day. It’s about uh receiving a call, if you did not take your medication after two days. And then the last one is uh receiving a home visit, if you did not take your medication for a full week.

P: Ok.

I: So have you ever received an automated sms reminding you to take your medication?

P: Yes, I did.

I: So, can you describe your experience receiving the sms?

P: Ok. uh on that day, uhm I was writing my exams. So I forgot to send the code. And I think it was around 6 o clock- ya 6pm then I got an sms to remind me that I didn’t take my medication. Although I took my medication, but then only forgot to send the code. Then I sent the code, then I got a confirmation that I took my medication.

I: You’ve mentioned uh forgetting to send an sms or forgetting to send the code.

P: Yes?

I: How often does that happens?

P: It only happened once when I was writing my exam. Because I took [inaudible segment, 29:10]. So- I forgot to send the code.

I: Oh ok. Have you ever received a phone call?

P: No.

I: Has ever a home visit done to where you staying?

P: No.

I: …So- but from you experience, do you think the differentiate care model is effective?

P: Yes, it is because uhm it shows concern. Uhm when you haven’t taken your medication, they remind you. And uhm as you said also, uhm when you don’t take your medication for two days, they call you. And also they inform you about uhm when you’re about to receive a call from a healthcare worker. So you know what to do next and also it- it he- it prevents like defaulting.

I: Uh can you describe for me any barriers or something that might stop you from using the Label?

P: Uh only load shedding.

I: When you say load shedding, how often do you experience load shedding?

P: Uhm uh it’s like- yoh. It takes long before we experience load shedding. Because uhm we are only updated about the schedule, I think a day before the load shedding is about to happen. So I think maybe once. And also when it is unexpected also, but then besides load shedding, uhm there’s nothing stopping me. Because when there is load shedding, then also the network is quite low. And also sometimes the battery might be flat. So you are unable to send the code. But beside that, there are no barriers.

I: How do you see the load shedding stopping other people from taking or from using the Label?

P: Ok. Firstly, uhm the load shedding, like interrupts with the network. So that means uhm you are unable to send an sms or even call or sending email. And sometimes uhm when you are at work and then load shedding just happens, maybe your phone is flat, then you don’t get the opportunity to charge or send the code. Sometimes when you wake up in the morning, then you find out “oh there is load shedding and my phone is flat”, so there’s nothing that you can do.

I: Ok. So, remember I also mentioned that this interview will look to check how satisfied are you with the label?

P: Yes.

I: So, can you describe your level of satisfaction with the Label?

P: Ok, I’ll say I’m 90% satisfied, uhm because the service I received from- from the Label is- is quite good. Very, very good. And also uh I will prefer to like keep on using it and also encourage other people who are taking tb medication to consider using the label. Because you– it is very, very helpful. So I’m completely satisfied.

I: Mmm…So can you describe to me how the label keeps your medication safe?

P: Ok. Uhm firstly, uhm each packet of the medication, it considers its own sticker. So uhm you’ll know- ok firstly you’ll have to finish this packet first, then- then lead to the second one. So in that way, I just keep them always like uhm- what can I say? Ok, I always keep them like in order uh using the stickers. The first one here, the second one here, and then the third one here. So I’ll know that I have to finish this one first then turn to the other one.

I: So at home, where do you put your TB medication?

P: Ok, I put it in my room drawer. Because uh that’s the first place I go when I wake up.

I: Mmm ok. So, your perception about the differentiated care model, what is it that you like and what is it that you like about receiving reminder sms’s?

P: Ok. Firstly, receiving a reminder sms uhm, it shows that I- I did something wrong and then also it al- it will- it shows me that uhm the healthcare workers, they actually care about my health. So I have to take my medication to make sure that I correct the wrong that I did. And also, it is very, very helpful because uhm sometimes you go to work and then like your mind is always full of stuff and then you forget, so when you receive an sms to remind you then you would see that “oh I forgot this, I forgot that”. Then you can do it same time when you receive the sms.

I: Oh ok. So remember earlier on, I explained that the differentiated care model includes sms’s, phone calls, as well as uh a home visit?

P: Yes.

I: Oh ok. [Clears throat] Now the second one. From your experience…what is it that you would dislike about receiving a phone call and what is it again that you would like about receiving a phone call?

P: Ok. So far, I haven’t received a phone call. Only an sms to remind me to take my medication. But then also uhm I think that uhm receiving a phone call would be good, because uhm it would keep on monitoring me, and also to find out why I didn’t take my medication. And also if I took my medication and sent the code, and then the code wasn’t confirmed, then you can also fix that like with the care health worker. So I think uhm it will be good like in keeping tabs and in keeping on the safe side.

I: Mmm. Ok. So from your experience, can you say the differentiated care model is effective?

P: Yes, it is, very, very effective.

I: Oh ok. For someone who- who is not taking tb medication, how can we approach the differentiated care model?

P: Ok. Uhm Firstly, you can call and then find out why they- they are not taking their medication. And if that person doesn’t have tb, then you can also find out the- like uh- about other diseases that the person might have. And also it can also help to improve like uhm the adherence of taking medication and also being on the safe side. And if the person is taking tb medication, it will help them to always take- to adhere to their medication. And also remind them to take their medication.

I: [long pause] For someone who does not take or who does not adhere to tb medication, do you think a home visit would work?

P: Yes. Because in that way, you can talk to the person face to face and also [ringtone]-

I: For someone who does not adhere to taking medication, do uo think the home visit would work?

P: Yes I think so. Because uhm you’ll get the opportunity to speak to the person face to face. Find out their difficulties in taking their medication. And also offer a solution on what can be done for them to take their medication and also monitor uhm how like- how they live. And also help wherever you can.

I: Ok. During the home visit, what should be said during the home visit?

P: Ok. Firstly, uhm you can’t attack the person for not taking medication. But then you can listen to their problems first, offer counselling, offer guidance, encouragement and motivation. And tell them what is the importance of taking medication each and every single day. And what are the risks of not taking medication. And also offer to help and other solutions on how to make sure that they adhere to their medication.

I: [Winding sound] During the counselling sessions, who do you think will be best suited to conduct the counselling sessions?

P: Ok. I think that all care health workers like are qualified to counsel the person or the patient. Because uhm at school, there are surely being taught, or even during induction, they are being taught about psychology and uhm how to treat a patient and also how to talk to a patient. So- and they have the experience with patients, I mean like they work with patients each and every single day. So I think that they can uhm counsel the person and also advise them on what to do, guide them and also offer them encouragement and motivation.

I: …So, uh from what you said, do you think it will be appropriate to take a tb patient to one of the counselling sessions?

P: Uhm yes, I think so. Because uhm that will help them like uhm to overcome their fears and also to be open [hoot] and also to appreciate their life. And also do what is right.

I: [long pause] Ok. So again, the differentiated care model, I did mention that it’s sms, a phone call as well as a home visit. So out of all these, which one do you think is the most effective and which one do you think is the least effective?

P: Ok, uhm I think that a home visit is the most effective, because uhm you get a chance like to meet the patient and also to find out the difficulties and also encourage them and also uhm check their living standard and the kind of environment they come from and also be able to bond with them. And uhm the least effective one is the sms. Uhm because in an sms and then the patient will just uh look at it and also not take the medication, because you can’t see them if they took their medication or not. And also you can not confirm if they are taking their medication or they are just sending a code.

I: Oh oh oh oh ok. Oh all right. That’s wonderful. That’s good to hear. So we said the home visit is the most effective one.

P: Yes.

I: Ok. So when conducting these home visits, what do you think is it that we as the healthcare workers should not do during the home visits?

P: Ok. Firstly, uhm you cannot force a patient to partake in counselling or an interview. As you said, you have to get a consent first, if the patient wants to communicate with the healthcare workers or not. Because uhm they have each and every right to agree or to not agree. And also you must not ambush the person or be rude or mean to the person. Because you don’t even know what the person is going through first. You don’t know why they are not taking their medication. So you can’t just assume that they are doing it deliberately or on purpose.

I: Oh. Ok. So in your opinion based on you experience, how can we improve this study or this Label?

P: Ok. In order to improve the label, I think uhm home visits should be done to all the tb patients maybe once in a month to check if they are taking their medication right. Even though they send a code each and every day confirming that they took their medication, but then a home visit will just make sure that they are taking their medication, and also see if they are improving or not. Because some uhm- they are at home, they send a code like I said, but then they didn’t take their medication, they didn’t get better. So a home visit each and every on- like will make sure that uhm they do take their medication. Because uhm they will know that “ok, uhm the healthcare worker will be coming on this month, so I have to make sure that I take my medication, so that they- they can see that I am improving, I’m getting better, I’m doing the right thing.”.

I: The once in a month home visit will help the patient adhere to tb medication?

P: Yes. What I know most about uhm patient’s uhm, they are scared of getting caught. So if you visit them once and then you let them know, “oh next month I’ll also be coming. Then they’ll make sure they do the right thing. They will adhere to medication. Whether they like it or not, but then they will take their medication and also be healthy again. So that will not- not- I’m not saying that uh the healthcare workers like should give them fear, but then also like they are conscious or tell them “ok, I’m doing the wrong thing so I have to do the right thing otherwise I’ll get caught. So I have to take my medication because I know that the healthcare worker is coming to visit me. So they have to find me in a proper state.”

[take 5]

I: Mmm. So earlier on, you mention that you took medication for one month without the Labels?

P:Yes?

I: How was it to take uh tb mediation without the Label?

P: Oh ok, im it was quite difficult because at that time- at that time uhm I had to set an alarm each and every single day to remind me, and also do a reminder set or even set on my calendar each and every single day. So it took more time and also uhm it was difficult, because sometime I put my phone on silent and then I wouldn’t hear the alarm. So then I’ll also uhm- it made me sometimes lose the time that I used to take my medication in, meaning that uhm I had to change my routine from 6 o clock to 7 o clock sometimes to 8 o clock.

I: Oh ok. Is there a difference in taking tb medication with the Labels and without the Labels?

P: Yes, there is a difference. Because uhm they-the Label keeps on reminding. It keeps the tabs on you, but then when you don’t have uhm someone to keep the tabs on you or to send you an sms to alert you that you have not taken your medication, or when is the time to take your medication, uhm sometimes you forget like- so uhm then that’s the risky part. Because you might end up defaulting uhm and also missing uhm treatment means that uhn less chances of getting better.

I: Ok…Do you perhaps know this box?

P: Mmm, no.

I: Is this the first time seeing this box?

P: Uh I don’t remember seeing it.

I: Ok, thank you. So that- now that you are on the Label, besides the clinic or the facility, have you seen the Labels somewhere else before?

P: No.

I: And then earlier on- so now I’m trying to like wrap up the interview, ne? So uh the questions that I’m going to ask you now, they are basically on the things you said before. So I just need you to clarify for me a bit.

P: Ok, that’s fine.

I: Oh ok. So earlier on you mention that uh XXX (nurse’s name) is the one who told you about the Labels.

P: Yes, she did.

I: Oh ok. Like, judging from the way the consent went or her telling you about the box, how the process went, who do you think is most best suited to tell the patient about the Label?

P: I think the people who like specifically like work with the research teams. And also people like sister XXX (nurse’s name) because she was very, very thorough and also I understood each and every single term, even on the consent form. And also uhm she- she was not forcing me to do anything, but then uhm she explained also the benefits. Uhm and also she explained what will happen if I do not take my medication. And more like she offered me a counselling session also which is good. Then I knew that I was not alone and actually there are some people who do care.

I: Mmm ok. So in your opinion, you would say that research assistant does the consent form as well as the Label very well?

P: Yes.

I: Oh oh thank you for the feedback. So if I may ask something, is there any one in your family who has ever had tb before?

P: Yes, my grandmother, but she’s late right now.

I: Oh oh oh ok. Uh uh I’m sorry to- to- to hear that- uh that she’s no more with us. So- but what was your first reaction when they told you that you have tb, knowing that uh that there’s someone in the family who has passed away who had tb before?

P: Ok, uhm firstly uhm I understood that uhm my grandmothers’ situation, like was different, because she was staying at work and then her tb was detected late. So uhm she had to come home, but then she was seriously ill at that time, because they told her that uhm her tb was on phase 2. Ya, phase 2. And I- I was the one taking care of her. So, uhm I saw what happened and truly speaking, I was scared the first time. I thought that I was also going to die uhm from tb. But then when the nurses explained the situation to me and also told me that this is not the end of the world, tb is treatable. And uh they also told me that uhm my tb is at the earlier stage, so uhm it is- uhm it is better because uhm it’s going to be curable fast. And also I will improve fast. And I began like to gain more confidence, more self-esteem and also to fight back.

I: Ok. Uh do you think the labels or this- this sticker could have helped her in the journey of tb?

P: Yes, I think so. I definitely think so. Because uhm it would have also uhm kept monitoring her tb adherence and also letting know her employer uhm what is happening and they also keep on monitoring her.

I: Ok. Based on your experience, do you think the Labels should have been implemented a long time ago?

P: Yes, I think so. Because uhm this is the best invention ever, because uhm it- it its changing lives. It is helping people. And also uhm it makes tb be less scary for us and tb patients, and- and it also helps us like to adhere to our medication and also to improve our lifestyles and also uhm to take our treatment very well and also to get back to our feet.

I: Oh ok. And again, from your experience, do you see the Label helping people take- adhere to tb medication in the near future?

P: Yes, I do. Because uhm they will constantly get a reminder to Take their medication and also if they do not take their medication and also they can expect a- a phone call or a house visit. So uhm that will help them improve their lifestyle and also to ask questions and also to feel great about themselves. Because there are some people who are concerned about their lifestyles. And also their health. So I think it will help more people to adhere to their tb medication.

I: Oh ok. Uh my final question- my fin- my last question- so judging by the way this uh- the Label or this study was projected, uh…do you think there are any gaps that you’ve uh- like the Labels or the study does not cover? [hoots]

P: Uhm except for the home visit part, uhm I don’t think that there are gaps. Uhm on- I- I would suggest that uhm the Labels start taking initiative on the home visits. I mean like uhm there are some people like who need those home visits, who need those counselling sessions. Because uhm here at the clinic, uhm there are lots of patients with tb. So the nurses do not get enough time to counsel them thoroughly, they just only cover the basics. But then if there is uhm- there is a home visit form the Label people, like they can sit them down the whole day, talk to them for hours, and hear their thoughts. And also share their opinions, experience, and also help them to live a better and healthy life.

I: Oh ok. Oh that’s- that- that’s wonderful to hear. And then I know you gave us like a mouthful of information, but again, what are your final thoughts about the Label?

P: Ok. My finals thoughts on the Labels are that uhm the department of health should keep up the good work and also the ASCENT should keep up the good work, and keep on helping people, keep on changing lives. Because this sticker will- helps and it works and the Label helps change lives. It saves lives. And also it helps people adhere to their medication. Their treatment. And it shows care, love and appreciation to tb patients. And it also helps them fight back. I mean even though they don’t help them physically, but the emotional support counts more than the physical part, because they are there as- as motivators, encourager. And also they are there as parents, brothers, and sisters and support system when you need them.

I: Oh oh oh ok. So uh like I’m afraid uh wave reached to the uh end of our interview, ne? So like I would love if maybe we could continue and get- get more information. So- but uh thank you very much uh for stopping by uh and then we appreciate uh the time and the efforts that you took, that you came here and then uh did the interview. Uh thank you for participating in this interview. Uh uh thank you very much uh PID 36- uh 16. Uh thank you very much for coming. And then uh time session ended, it’s uh 1:51PM. Thank you.
