# Supplementary material for: Acceptability of Digital Adherence Technologies to support people with drug-susceptible TB in South Africa
Source: PLoS One. 2025 Sep 24;20(9):e0332103. doi: 10.1371/journal.pone.0332103 (PMC12459780; doi:10.1371/journal.pone.0332103)
Supplement: S4 File — (ZIP) [file pone.0332103.s004.zip › S4 Transcripts/PwTB/IDI 34_PwTB.docx]

**TRANSCRIPTION NOTATIONS**

| **Label Key** | **Meaning** |
| --- | --- |
| **I** | Start of each new utterance by the Interviewer |
| **P** | Start of each new utterance by the Participant |
| **N** | Note taker |
| **{ }** | Indicates that details were changed or pseudonyms were used to anonymise data |
| **( )** | Indicates the description provided to anonymise data |
| **XXX** | Words were omitted to anonymise data |
| **-** | Breaking into a sentence by the next speaker |
| **…** | Pause or drawn out words |
| **[ ]** | Indicates noise made, e.g. [laugh], [sigh], [pause] |
| ? | Beginning of utterance by unidentified speaker or questionable text |
| **[inaudible segment]** | Unclear section of the recording |

I: Thank you so much for agreeing to participate in the study again. Can you please allow us to audio record this interview?

P: Yes, you can do it.

I: Oh, ok. mmm…date of the interview: it’s xxxx (interview date). Location: xxxx (clinic name), xxxx district. Language uh – the session is done in English. PID number: xxx. Time of the interview: -

P: Twelve passed eleven.

I: 11:12.

P: Mmm.

I: Facilitator: xxx (Name of the Interviewer). Uh so tell me, buthi (brother) where are you staying?

P: I stay in xxxxx (suburb name)

I: Here in the area?

P: Yes.

I: Oh ok. So do you usually take a taxi when you come to the clinic or you are walking?

P: I just take a taxi.

I: You take a taxi? How many taxis are you taking?

P: Just a single taxi.

I: Ok. So who do you stay with at home?

P: I stay with my wife.

I: Mmm.

P: My wife.

I: Oh. Only?

P: Ya. The kids are not around.

I: Where are they?

P: At- at home.

I: They- they are at home?

P: Mmm.

I: Oh ok. So when did you find out that you have TB?

P: Uh I find out that- uh TB last October.

I: Mmm.

P: Mmm. October, I became sick then. I eh- I was told to go- to come here and they sent me to me to xxx (Name of the Clinic) and I was diagnosed with TB, I was diagnosed with Meningitis.

I: Mmm.

P: Mmm.

I: Oh so what were the symptoms that you were seeing?

P: Uhm the symptoms which I was seeing is uh like- I was not coughing at all, but I was- tiredness was one of the- the symptoms that I – feel tired, weak, uhm and lack of appetite. And uh ya.

I: It was that.

P: Mmm.

I: Oh ok, so uh I’ve been saying that, you know, we’ve been using this technology to support you to take medication. What do you understand about this label - this- tell me your understanding about this technology or these labels?

P: Ok. Uh the- these- these ones- uh what I understand about them is the- to- after taking the tablets, you have to sms that- that- that number- that number-that is sort of to record that you have taken your medication on that particular date and that you need to also clarify the time that you have taken your medication, because it also- what I like about also the- the- also before 6 o clock, it reminds you that you have to take the medication before 12 o clock. So that I understand this one is also very good technology because it always reminds you. Mmm.

I: Mmm. So, you are saying it’s a very good technology?

P: Ya.

I: What else is good about it?

P: Ya, what is good about it is that it always reminds you. You can’t say “I forgot, even if you forget uuuh around 6 o clock, it will tell you that you need to take your medication. And then if- if at- 12 o clock passes without taking medication, it will also tell you that you didn’t your medication and it will warn you that your health is comes first. You have to take the mediation because your health is very special.

I: Mmm.

P: Mmm.

I: Ok. So, you are mentioning that it reminds you?

P: Mmm.

I: How – how were you reminded by this technology? How were you reminded to take medication?

P: Mmm mmm It is- in fact I know that ay at 6 o clock uh uh it reminds, sends you a message on my phone, so there’s no way uh uh uh- everyone always have his phone by his side. So, uh always by that time, it reminds you before 10 o clock, before this time I must take my medication.

I: Ok.

P: Mmm.

I: So when did you start on TB treatment?

P: Uh I started as soon as October.

I: October last year?

P: Ya.

I: Ok, and when did you start on these labels?

P: On this one?

I: On this technology?

P: This technology, I- I started- I’m not sure, but- when was it? But I think around that December, the December- November then.

I: Ok.

P: Mmm.

I: So you were explaining to me how this technology works? Who told you about this? Who was explaining this whole information to you?

P: Ye- the- the- the nurse was- was supervising me was the- the one who told me how to use it.

I: Mmm.

P: It also helps- I think it also helps the nurse who’s looking after you, to see that how often do you take your mediation.

I: Ok.

P: Mmm.

I: So how do you feel that this technology also allows the nurse to follow up on you? On how you are doing?

P: Ya I thinks it’s- it’s ok, it’s fine.

I: Mmm.

P: Mmm.

I: Ok. So was it a nurse who told you about this technology or was it a- a- a- an intern, a research intern?

P: Ya, it- uh first person who- who gave me the- the first information about that was a nurse. And then she told me that the- those people will now come and then they also gave me some lesson lecture about it.

I: Ok. So you are saying that they gave you a lecture about it. Was it easy when they were lecturing how on it- on how to use it?

P: Ya, it was easy.

I: So I was still asking you, uh was it- uh was it easy to understand how to use this label when the nurse was explaining to you?

P: Ya, it was- it was easy to understand. But first I was- it- it was ?myself?, but I also find out that no, it was ok.

I: Say that again.

P: Uh ya it was- for me it was easy to understand it.

I: Ok. You- you didn’t have any-

P: I didn’t have any problem.

I: Is there anything that you would like- you would change about how you were informed about this label?

P: Mmm, uh the- the- there is nothing.

I: Mmm. Anything that you would- you can suggest on how the nurse or the intern can explain about this Label to someone who doesn’t know about it?

P: This one? Yes.

I: Ya. You said that it- it- it- it also gives them the- the knowledge to how you are taking your medication. Are you skipping? Are you not skipping? Because this one also shows that this person is not taking the medication regularly, he is skipping sometimes.

P: Mmm. You see?

I: How did you feel when the nurse was telling you about this label? How did you feel?

P: No, I- no- eh eh eh I did feel that eh this thing maybe it was myself, I thought maybe it was highlighting- it- it- it- message was going to the- the- the- the funders. You know, I thought maybe someone who is funding these eh TB- these eh TB- like to see how they- they- the- the patients they do or how they- they- they- they- they- they- they- they [inaudible mumbling] helping community. How is it eh eh affecting people. Because this one is one of the most dangerous disease. Mmm.

I: Did you feel that now we are coming with this technology to help people, because this is a- a- a very dangerous disease? How did you feel that you are feeling that we are coming with this support to you guys?

P: Uh To me, I just took it as eh one of the- as uh- as- as an advanced eh technology that was maybe ?long back?, give you tablets then you go home, just it’s- it’s up to you to drink it, whether you don’t drink it, there is no supervision. It’s fine. It’s good. It’s- it sits- it’s another way of trying to eradicate the disease.

I: Mmm. So you are mentioning something like it’s an advanced- its advanced. Tell me more about that.

P: Mmm.

I: what do you think about that?

P: Its advanced because you with your phone eh whenever- if you are- if you you registered that line with your phone- if you are- even if you get sometimes may- you are very busy doing something or you tend to forgot or you are not- doing something, or you have got some friends that you are visited you , but once the message comes, you know that “eish but although I’m still having some people here but- but I- I have- I have to take my medication. I cannot skip my medication, no matter how busy I am”.

I: Mmm.

P: Mmm.

I: All right, very good to hear all of those uh important information that you are telling us. So where else did you hear about this technology or this Label?

P: Ay, it was the first time.

I: Ok. Hearing it?

P: Mmm. It was the first time to see it.

I: Ok. You’ve never heard it- about it anywhere else except here?

P: Uh no.

I: Ok. So what aspects uh of parts of this label was easy to use for you?

P: Aspects?

I: Mmm. Or parts of- of the- of this label were easy for you to use?

P: Uh just saw it was- was an easy one because usually what you do is when you send, you know- you know- you know- you know, the- the- the- the- the- the message- once- once you send that- that single message, you just follow- you keep on sending- sending- sending- sending- sending

I: Mmm.

P: So once that batch is finished, you know tha- that-

I: Mhm.

P: Mmm.

I: So you- you are telling me that you are just sending and making sure that that batch is finished?

P: Mmm.

I: How- how- how did that feel for you to do it?

P: Uh you get used to you. You get used to it. You- you just get used to it and you know that it’s not fu-, it’s not eh eh permanent solution. It’s going to [inaudible segment, 11:22] time. As time goes- as time goes as long as you are taking your mediation, it will come to pass.

I: Mmm.

P: Mmm.

I: Ok. And tell me about your difficulties to the sending the codes as you are saying? What- what difficulties did you experience to do when you have to do that?

P: Ok, Listen maybe there- there was- maybe there was load shedding, you didn’t have electricity, you didn’t charge the phone. So you have difficulties there. Maybe the time to message you, the phone is off. Something like that.

I: Mmm. So you are mentioning that, you know, sometimes it’s the loadshedding that would eh give you challenges? What else can you tell us about the challenges when- when you have to use this technology- this- this- this electricity and what else?

P: Ah sometimes you have lost your phone, something like that.

I: How often were the challenges of electricity on you case?

P: Ya, it- it- it did have some challenges when you- was when loadshedding- you know it was electricity.

I: Mmm.

P: Mmm.

I: Ok. So what- so what would you do if there’s load shedding without electricity?

P: Eh, uhm I will just- what I will do, I’ll just take my medication or take my medication- maybe I will- I will wait for the electricity to come then I will send the- but sometimes you send then the time passed. So that’s the difficulty that you find. You can take your medication then you- you can’t send that message, you don’t have- your phone- your battery-

I: Mmm.

P: Mmm.

I: So if you are not sending the message, were you sometimes maybe getting some sms’s which maybe- what- what was happening if you did not send the message for that day?

P: Ya, it will be-you get an sms that you- would say that you didn’t take your medication.

I: Mmm.

P: That you haven’t taken your medication. But because there was not electricity, you could not send.

I: Mmm. How did that make you feel?

P: Ya, I feel bad.

I: Ok. Tell me more about that. Why would you feel bad

P:I felt bad because they will think that maybe because I didn’t send the message, I’m jeopardising my treatment but there was no way I could send the message if there was no electricity.

I: Mmm.

P: Mmm.

I: Ok. And then, do you work?

P: Ya, I work for- I- I do my work for myself.

I: Where do you work?

P: Self-employed.

I: You are self-employed.

P: Mmm.

I: Oh ok. But you- you work with people?

P: Ya.

I: You are not alone?

P: Ya. Yes.

I: What time are you- are you taking medication?

P: I’m taking medication every evening.

I: When?

P: In the evening.

I: In the evening?

P: Mmm. After meals.

I: Afternoon?

P: After meal like 8/9.

I: Oh at night?

P: Mmm.

I: Oh so you don’t take it when you are at work?

P: No.

I: So how- how- how many hours are you working? Are you working in the morning, form 8 in the morning until…?

P: Until maybe 4 or 5.

I: Ok so by the time you are taking- you are taking your medication, you are already at home?

P: Ya.

I: Oh, but have you ever maybe said something to your colleagues at work about this- this Label tech- this technology that were- we are giving you to assist your TB treatment?

P: Uh-

I: Have you ever said something?

P: Uh no.

I: Never shared?

P: I have never shared.

I: Ok. So at home, did you- at home uh have you ever said something about this technology to your wife as you said, you are staying with your wife?

P: Ya, I have said it to her.

I: Mm, what did you say to her?

P: I- I told her how- how- eh if you take this medication, you have to send a message mmm and if you don’t send the message, they will remind you.

I: What did your wife say when you were telling her about this technology or this label?

P: Ah she told me it was- it was ok, this one is fine. It’s ok.

I: Mmm. How was she- like was when- when you told her about this technology, I want to know how did she feel about it? Was she-

P: Ah no, she didn’t say anything.

I: Mmm.

P: Ya she didn’t say anything, she just said ah it’s another way of reminding- people always forget.

I: Ok. So for somebody maybe who’s working, ne? And is taking medication at work, from your opinion, from your personal opinion, what would be their- how would it be for them to take medication and send these sms’s while they are at work? In your case, it is different, you are at home already. But for someone who goes- who’s at work and they are taking medication maybe at 12 o clock, and they are at work. What are your opinions about eh them sending this message while they are at work?

P: Uhm, I think it’s almost the same way. It’s the same- eh it’s just the same.

I: Mmm.

P: Mmm. Because what is important is taking your medication. And the send the message to let- to let- to let- to make them under- aware that you are taking your mediation. Doesn’t matter- as long as you take your medication within the given time. Within that given time of your day. Because if they said take 4 tablets a day, it’s up to you when you take it then.

I: Mmm.

P: Mmm.

I: So will it be easy for someone to- to- to- to be sending these sms’s while they are at work?

P: Eh ya. I think at work, people also- they have got a break. You’ve got lunchtime, breakfast time. During that time, I think you can always do that.

I: Oh during your lunch-

P: Lunchtime. After you finish off. If you are given 30 minutes and after- maybe after your lunch you can always take it, and then you sms at the same time.

I: Oh it’s something that is doable, even if you are at work?

P: Ya.

I: Ok. So what were your worries when you were introduced to this tech- to this Label? What did you think about when they were telling about this label that- from today you are going to use this label, what- what were your worries or what were your concerns about this technology?

P: Uh to me, it doesn’t- it- it- it eh doesn’t consume data, it doesn’t consume anything. So I didn’t see any problem.

I: Ok.

P: Mmm. It doesn’t- you don’t need to have data to sms or airtime to sms, as long as your phone is on, you can sms.

I: So you mentioned earlier on that you only told your wife, you told- you told you wife that this technology, besides your wife, who else did you sort of told about- uhm you sort of informed about these technologies? Besides your wife?

P: Uh there’s nobody.

I: There’s no one?

P: Mmm.

I: Oh. What are your reasons of not sharing?

P: Uh myself, people- because first when I got sick, I- I- I- I didn’t know that I had TB, so I thought I was having maybe blood clot or other things. Because I uhm blood clots…like when you touch me, you can feel that blood is not moving, so I didn’t know this things. So when I- I- later on they told me that I have got TB.

I: Mmm. So how would you feel telling someone else now except your-

P: Even myself, I didn’t understand because I was told- me, I was thinking if someone who has got TB must be coughing. So myself I was not coughing. So, until I was told that my TB is not- is on- in- on vein, not that one like- it was- that’s when I started to understand.

I: Mmm.

P: Mmm.

I: Ok. But now can you be able to tell someone about this technology and-

P: Ya.

I: Mmm.

P: I can always tell someone.

I: Ok.

P: Mmm.

I: So what would you say to them if you are to tell them about this technology?

P: No, I will explain to them that nowadays you- if you- you have- if you are diagnosed with TB, there’s this way of taking my tablets that’s eh eh- a way of- the way they remind you by sms and then you have to send message back.

I: Ok.

P: Mmm.

I: …So uh according to what you told me that it’s- it’s you- your wife knows that you’ve got TB, right? Who else did you manage to disclose to?

P: I think maybe my sister, but she’s not staying around.

I: Your sister?

P: Mmm.

I: Ok, so did you tell your sister about-

P: Ya that I was diagnosed with TB and...

I: Did you also told your sister that you are- you are- you are using this- this technology called Label, that you get it from the clinic?

P: No, I just told her that I’m taking medication.

I: Ok. Oh you haven’t told her about this technology?

P: No no.

I: Ok. How was it when you are talking to your sister about your status, about your TB status?

P: Uhm uh uh it was just- because- I was- I find it more uh how I’m doing, I just told her that I was diagnosed with TB, but now I’m outside. She just said, “ah ok, but TB can be treated”.

I: Mmm.

P: There’s no problem with TB. TB can be treated.

I: Mhm. So when you are telling your wife about this technology the other day when you were showing her and how it works, how did she react on this technology?

P: Uh she just said ah oh its fine. She didn’t say anything much.

I: Mmm.

P: Mmm.

I: So-

P: And I just- she- she- I just- at least they are a way of reminding people that you haven’t taken your medication. It’s a way to- to remind you.

I: Mmm.

P: Mmm.

I: Any TB history in the family?

P: No.

I: No one at home who once had TB?

P: No.

I: Ok. Have you ever sent sms’s uhm more than once?

P: Ya.

I: Ok. What was the cause of you sending an sms more than once?

P: Ok, I sent the- I sent it then I saw- because– we are used to sending these sms and we maybe after 5 minutes, it will reply. “Thank you for taking your medication”. But that day it didn’t. [Inaudible segment, 23:07] So I had to resent again.

I: You thought it went?

P: Maybe I send it into a wrong number. Maybe I send it wrong.

I: Oh. You- you were thinking that maybe you sent it to the wrong number?

P: Ya.

I: Oh because you did not receive that-

P: Ya.

I: Reply?

P: Mmm.

I: Ok. When did this happen?

P: Uh-

I: Since you have started using the Label?

P: Ya.

I: When- when did it happen? Remember you said you started to use the labels- when? November?

P: Mmm.

I: November?

P: Ya.

I: Ok. From that time, like when- when- when did it happen? Did it happen around that time you- you just got the- the technology or even recently?

P: Ay it was in [inaudible word, 23:49]. Uh I think there was the- the- at that time there was problem of load shedding and I think the network was also not doing well.

I: Mhm. How often did this happen?

P: Uh maybe not- not much. Not- sometimes.

I: Ok.

P: Mmm.

I: Ok. So how did you feel when it’s doing that?

P: Uh I- no, I get sometimes annoyed. Sometimes. Maybe message is not reaching them but I just tell myself ?I’m not here to reach them?, I’m just getting annoyed that maybe it will reach at the- at the late time. Maybe around 10. Maybe then the message will reach there around 1 o clock. You see?

I: Mmm.

P: Mmm.

I: So where do you keep your- your medication at home?

P: Uh I just keep them in a- there’s a- there is a- in- in my cupboard, there’s a cupboard in there.

I: Mmm.

P: Mmm.

I: Ok. Now let’s talk about what was like, what was helpful to you to use this technology? How did it help you?

P: Ya, it helps because I’m always using this one, I always get- always remember- uh reminded- uhm I’m always reminded.

I: Mmm.

P: What was-

I: Also- also- also- it also shows me that even if I know that- eh like- like let’s say if I- today is Monday, I have up to Sunday to show that I never skipped any day.

P: Mmm.

I: It will remind that it was- at- like that, it will follow each other.

P: Mmm.

I: So eh earlier on, you mentioned that you would receive a sms that says you did not take your medication today?

P: Mmm.

I: And sometimes you can even get a call? How- how- how- have you ever- how did you feel when you were getting the sms’s that you did not take medication?

P: Mmm. Ya, you- you- you- you get embarrassed.

I: Mmm.

P: Mmm.

I: Have you received these sms’s?

P: Ya.

I: How often?

P: Uh like when- when- when I- when- when it’s like maybe I send the- I send the message maybe around like around- maybe I forgot to send the message at around 11 o clock. Once 11 o clock, I remember sometimes, I send message around 11 o clock. I took medication around 11 o clock. Maybe it will say that you didn’t take med- medication. So they send that message. But I did take my medication.

I: But you have taken?

P: Ya.

I: Ok.

P: I did. So now I try- I- I now try take at least- at least and must make it two hours before.

I: Ok.

P: Mmm.

I: So are there times where you- you for- you forget to take you medication, then-

P: Maybe I just come from work, I’m tired, I just- after your meal, just- I just sleep, then I woke up around 11 o clock. Then I take the medication, then I sent the sms maybe past 11.

I: Ok.

P: Then that side will- they will tell you tomorrow mornings message that you didn’t take your medication yesterda

I: Ok.

P: Mmm.

I: So did- did you understand why it would send you an sms to say you did not take medication?

P: I didn’t- I don’t- I didn’t understand why.

I: Mmm.

P: Because they said before 12 o clock. So me, I’ve taken the medication around 11 o clock.

I: Oh so, wena, you take it at 11 o clock, around 11?

P: Mmm.

I: But in the morning, you’ll still get an sms?

P: Mmm.

I: Saying you did not take medication?

P: Mmm.

I: How often is that happening? Is it something that happened more after or- or just-

P: Uh not more often. Sometimes. But so then I- I- I- I understand that then I- at least can you take- eh they not- not more than half past 10. Let me just take maybe 2 hours before or 1 hour 30 minutes before 12 o clock.

I: Ok. And then it stopped. You stopped getting-

P: Ya.

I: Sms’s?

P: Ya.

I: Ok. Have you received a call reminding you about taking your medication, except the sms reminder now? What about the call? Have you received a call?

P: Ya.

I: Mhm.

P: Mmm.

I: How did you feel about those calls?

P: No, I explained that I know I took my medication at this time, but I also did send a mess- a sms. Or sometimes I said, I- I took my medication but there was no electricity and my phone- my battery- my phone was very low.

I: Mmm.

P: Explain that to-

I: Ok. what about home visit? Have you received any home visits from the clinic, from the ASCENT team?

P: Uh no.

I: Never experienced the home visit?

P: Ya.

I: Ok. So how do you feel about these home visits that we are offering to patients that are struggling to take medication? What are your opinions about these home visits?

P: Ya, it’s ok. It’s fine.

I: Mmm.

P: I think they are ok.

I: Why are you saying it’s ok to do a home visit for a TB patient?

P: I think you have to- you have to sympathy- sympathize with the- the- the- the- the patient. So, sympathize with the patient.

I: Mhm.

P: Mmm.

I: Ok. Uh please describe any barriers to the use of the label and these calls that we’ve been- you’ve been getting, the- the automated sms that you have been getting. Just describe uhm the challenges for that.

P: What?

I: Please describe any challenges to the use of the label. What challenges did you experience using these Labels?

P: Eh the challenges? Uh maybe sometimes…what can I say?...ehm…sometimes maybe with like- with the numbers, these numbers, maybe you’ll find that- eh that what- what- what- what happened last time?

I: Which numbers are you talking about?

P: They are these numbers. Eh let’s say I use these numbers and then the packet is not finished- what should I do? Let’s say- I have got 2 packets ne? I take 4 tablets per day and one packet is left with 2 tablets and I have to take another 2 tablets that are in another packet, which number do I use there? Sometimes it choose to give me a problem to which number to use.

I: Mmmm. Oh so what would you do? Which number-

P: I will just send the one which I was using.

I: Oh.

P: Mmm. Then I’ll see the reply is fine.

I: Ok. So that- that happened?

P: Ya.

I: A lot?

P: Mmm.

I: Oh so you would get a reply?

P: Ya.

I: Ok.

P: And other thing that you sometimes- maybe you’ll forget you still using the same number but when you have changed the batch, that one is finished. Maybe you forget sometimes.

I: Oh have you done that like-

P: Ya.

I: To use the same number even though the batch has been changed?

P: Ya. Mmm.

I: Ok. So, were you getting the- uh the reply?

P: Ya. I would get a reply.

I: Oh ok. What else was a barrier or a challenge to use this label?

P: I think ?that’s all?.

I: Ok…Please describe how satisfied you are using this labels?

P: Uh I’m satisfied. I’m -I’m satisfied. 100%.

I: 100%?

P: Mmm.

I: That’s very interesting. Why are you satisfied 100%?

P: It helps you not to forget to take your medication and it provides a diagram for you.

I: It- it provides a diagram.

P: Yes a diagram. You can see that today I took the meds even, you can count that Monday to Friday I did take my medication.

I: You can count?

P: Mmm. You can count.

I: How do you count that?

P: The- those sms’s. Mmm.

I: Ok. So the sms’s are also helping you?

P: Mmm, ya. On my phone, I just accept the- the sms’s. just write there TB so I know it’s- because when I sent those sms’s, it will be- it will- they will line up, that-

I: Oh the sms’s are lining up in your phone?

P: Ya.

I: Mhm. OK. That sounds interesting. So it helps you to see that-

P: Ya. Just to see that.

I: Mmm.

P: Mmm.

I: So what do you do with these sms’s after receiving them in your phone? Like you are saying they are lining up, what do you do with them after you received the confirmation for today?

P: Sometimes I delete the old ones.

I: Mhm.

P: Mmm. I delete the old ones.

I: Ok. So what would make it easy for you uh to use these labels again? Like if you were to give us some suggestions to make these labels much easier, what would you say?

P: Mmm. Uh probably I’d say that you can use- maybe you just use one number. Until for a month, let’s say you come to collect medication, they just give you a number. You just sms to that number. You finish again, you come and collect another medication, they give you another number. That like that.

I: One number?

P: They just- they just give you a single number.

I: Oh.

P: Mmm.

I: Why are you saying that?

P: Uh I think eh once they give you sms a single number, you don’t need to look after those papers, you know, because sometimes you have to look for these papers.

I: Mmm.

P: Mmm.

I: Ok, so these papers, how are- how- how-

P: Did- they give you these 757?

I: Mmm.

P: They give me those 5 packets. I will lose that 57 eh continuously. That same number.

I: For the whole month?

P: For the whole month. Then you come again, then they give you another number with different-

I: Ok. So you- you feel that would make it much easier

P: Mmm.

I: Ok. What else can make it- the whole process much easier?

P: Uh I think it’s…

I: Mmm?

P: Ay it’s fine in that way.

I: Ok. Ok. So have you received maybe counselling, whether telephonically from the healthcare workers about your TB, medication? Have you received any counselling?

P: No.

I: On the first time you were here to start TB treatment, did you received counselling?

P: Ya

I: How did you feel when- when they were giving counselling?

P: Uhm I was I felt uhm uh s-

I: Mmm?

P: I felt supported.

I: Supported?

P: Mmm.

I: Mmm. What did you dislike about that counselling that you received?

P: Uh ?nothing?.

I: Everything was- was ok?

P: Ya.

I: What was the most interesting when they were giving your counselling about your TB status? Consider the fact that you are hearing it for the first time.

P: Mmm.

I: So the nurse was talking to you. What was the most interesting that kept- kept you going in- in your TB treatment?

P: Ok. I- what- they said that you- as long you take your medication regularly, within that- within that period, within that 6 months or 9 months, everything will be ok.

I: Mhm.

P: Mmm.

I: Ok. So according to the- this programme of these technologies, you get sms’s if you haven’t taken medication, we also call you, we sometimes go to your home and do a home visit. Which one do you consider the strongest or the most powerful activity in these three that I’ve mentioned? So again you get these sms’s to remind you that you did not take your medications, we call you or home visit? Which one do you consider as the most powerful activity to help a TB patient?

P: Uh I think this one of the sms.

I: The sms?

P: Ya.

I: Mmm. Can you tell me more about that? How- why are you saying it’s the most powerful one?

P: Because the one of sms is always there, everyday.

I: Mmm

P: Unlike a home visit which can only happen once.

I: Mhm.

P: You see? But this one, the sms, is always there, everyday.

I: Mmm. Ok. So please tell me ore about which did you find was the most useful- uh so you are saying it’s the sms?

P: Mhm.

I: And what else would- do you- do you say is- is the most useful in these three that I’ve mentioned?

P: Ya, it will be- I think it will be the sms and eh the one they give you a call is ok.

I: Mmm.

P: Mmm.

I: Ok. So, we are also doing sms’s, we are also doing home visits.

P: Mmm.

I: Uhm earlier on you mentioned that uh if we can maybe change this, make sure that you are using one number, then you use the same- another number on your- your second visit. What else can we do to improve uh- there’s also sms’s, there’s also home visits, there’s calling as well. I want- I want you to give me uh uh a picture, like to- to- to understand what else can we do to make sure that this problem is more effective and helpful for a patient.

P: Uh I think so far I’ve got no problem with what is happening. I’m satisfied.

I: Mmm.

P: I’m satisfied with those three.

I: Ok.

P: Mmm.

I: Ok. So in the clinic, who should teach TB patients about these technologies?

P: Mmm.

I: Who should teach TB patients about these Labels?

P: Uh I think it- the health- I think the health people-

I: Mhm.

P: And the nurse from [inaudible segment, 39:20], to the last person in the health department can always teach.

I: Ok.

P: Mmm.

I: So you are mentioning nurses, and who else?

P: Eh those who are- everyone who is in the health department.

I: Ok.

P: Mmm.

I: Why do you feel like anyone in the health department can teach about the Labels?

P: Ya, so that- to- to- to erase our illness. Mmm.

I: How- how can we erase our illness uh in- in the- in the- to- to- to people out there or in the community? How best can we erase our illness with these technologies?

P: Mmm. I think eh eh the message can be sent through radio or-

I: Radio?

P: Ya, radio or community pamphlets or newspaper or whatever. Mmm.

I: Ok. All right.

P: Because it feels like it was a disease that people- forgotten about it.

I: Mmm?

P: I mean TB, people mmm not the- they nearly forgotten about it.

I: Mmm.

P: Ay, because people- it- it used to be- it used it used- to be taken seriously, then around 90’s/80’s, but after HIV then the people started concentrating on TB, I see people they change. Now they no longer- they concentrate on Corona virus more than-

I: Mhm.

P: Other diseases.

I: Mmm.

P: Mmm. So our illness is important too. But as far as I’ve seen- I’ve visited hospital again, I can see some posters on the wall of the- the- the clinic or hospital and other things.

I: Mmm.

P: So now I can see that-

I: So- So with- with these technologies, how- how- how best can we- how best can we- can we- can we raise awareness about these technologies to people?

P: Hmm. Ah by- by maybe organising some workshops whatever.

I: Ok. All right, so I want us to talk about this box ne?

P: Mmm.

I: Can you see this box?

P: Ya.

I: Have you ever seen it before? Is it your first time seeing it?

P: First time.

I: It’s your first time seeing it?

P: Ya. No no, I don’t know if I have ever seen it. But I think it’s the first time. To me, it looks like a lunch box.

I: Ok.

P: Mmm.

I: So what else comes to your mind when you look at this box?

P: [laughs]

I: So it looks like uh a lunch box?

P: Ya.

I: Ok.

P: Mmm.

I: Would you put your medication in this box?

P: Ya.

I: Do you like it?

P: Ya.

I: For medication?

P: Ya.

I: Why do you like it for medication?

P: Ya, because it’s more closed. Closed. Can always carry your medication in it.

I: Mmm.

P: Mmm.

I: So has the nurse or the intern who works at the ASCENT research told you about this box?

P: Ya, they have told about the box but I didn’t understand what about the box.

I: Where- where did you hear about this box?

P: I don’t- there is a box uhm I forgot who told-

I: Who was telling you?

P: But- but I remember I think the last- the guys who came last time.

I: The guys who came last time?

P: Ya, they were talking about a box, but I don’t remember what they were saying about the box.

I: Ok. Mmm. So you- you don’t remember what they said about those box? Is it the nurse or the intern who was telling you about the box?

P: I think the intern, and the nurse also told me.

I: What did she say?

P: There’s a box- something like…something like this. Ah! I don’t know, something like maybe- she was talking about like- because I was telling- sometimes my phone whatever, whatever.

I: You were telling them what?

P: About the- uh I was talking about the phone.

I: Mhm. Mhm.

P: So-

I: So you were still telling me-

P: Mmm.

I: About this box. What were they saying about this box to you?

P: Ah! They were telling me about something like that can remind you.

I: Ok.

P: Mhm. Something like that.

I: That the box can remind you to take medication?

P: Mmm. Ya. It was- at that time, I was sick, so I don’t know, I don’t remember what were they saying about the box.

I: Oh you were sick on that day?

P: Mhm

I: Oh ok. Oh so they were saying it- it- it’s something that can remind you to take medication?

P: Mmm.

I: So now you have seen this box, you are- you were saying that it looks nice to keep your medication?

P: Mmm.

I: Eh if- if you were to compare it with this label, wha- what would you prefer between these two? This- using this or this for you, for taking TB medication?

P: Uh I don’t know about this one. I can’t comment on this one. I don’t know how to use it.

I: Ok.

P: Mmm.

I: All right. But is it looking good? How does it look for- for keeping TB medication?

P: Ay it’s ok. It’s fine.

I: Mhm. You like it?

P: Ya.

I: Ok. [Sighs] Uh so before we are almost done with this interview, but before we can almost conclude, are there any final thoughts you have you have about the- the differentiated care model that we gave you: that is the sms reminders, the calls, the home visit? Can you please share us your- your thoughts and your experience about that?

P: Ah the experience is very good.

I: Mhm, you are saying it’s very good?

P: It’s very good.

I: How is it very good?

P: Mmm. It’s- it’s very good. The- because it always to keep patients on check and also to see the side of other that the- the disease still needs to be fought against.

I: Mhm.

P: Mmm. Still needs to- as a need for a free environment of TB. Free TB environment.

I: Mhm.

P: Mmm.

I: So how did it feel to receive these sms’s on your phone to say ‘today you did not take your medication’ or ‘thank you for taking medication for the day’? You know, the calls, if you did not take your medication. How did you feel about that as a TB patient?

P: Mmm. Ya, that one it shows [inaudible segment, 46:28]. Shows the importance of-

I: Mhm.

P: Mmm.

I: And how did you feel when we call you and when you actually get the sms? How did you feel about that?

P: Ya, I feel happy.

I: Mhm.

P: I feel that they acknowledge that at least someone is fighting the disease.

I: Mhm. So as a TB patient, what- what’s- what’s uh other important efforts that you think we need to do to support you at home, like a healthcare workers? What would you suggest for- for us to do to support TB patients, in line with technologies? What else can we do to support?

P: Ah I think- I think it’s- its ok. What is- what- what- what you are- what you are doing is- is above always. So I thinks its fine.

I: Mmm.

P: I don’t have any additional solution.

I: Ok. Thank you so much uh for giving us all this information baba. We really appreciate. We are really closing our interview now. But before we can close this interview, any final remarks that you can tell us about the whole projects now, this ASCENT programme that we have given- we have given to you and all the technology and all the support that you been receiving, the calls, the sms? Just give us the- the- the whole overall remarks, anything that you’d like to say before we can close off?

P: Ya, I think I just- thank you for everything that you- you- you have done and I also thank the- the people who are supporting this initiative. So that’s all that’s what I can say.

I: Oh ok.

P: Mmm. I just thank you for…coming forward and fighting this disease together.

I: Mhm.

P: Mmm.

I: All right. IS that all?

P: Ya.

I: Ok. Thank you so much for your time. And we are done with this interview today and time is:…10 past 12.

P: Ya.

I: Thank you.

P: Mmm.

Glossary:

Labels: Digital adherence technology using messaged codes to monitor treatment adherence

Buthi: Brother

Wena: You

Baba: Father
