# Supplementary material for: Acceptability of Digital Adherence Technologies to support people with drug-susceptible TB in South Africa
Source: PLoS One. 2025 Sep 24;20(9):e0332103. doi: 10.1371/journal.pone.0332103 (PMC12459780; doi:10.1371/journal.pone.0332103)
Supplement: S4 File — (ZIP) [file pone.0332103.s004.zip › S4 Transcripts/PwTB/IDI 35_PwTB.docx]

**TRANSCRIPTION NOTATIONS**

| **Label Key** | **Meaning** |
| --- | --- |
| **I** | Start of each new utterance by the Interviewer |
| **P** | Start of each new utterance by the Participant |
| **N** | Note taker |
| **{ }** | Indicates that details were changed or pseudonyms were used to anonymise data |
| **( )** | Indicates the description provided to anonymise data |
| **XXX** | Words were omitted to anonymise data |
| **-** | Breaking into a sentence by the next speaker |
| **…** | Pause or drawn out words |
| **[ ]** | Indicates noise made, e.g. [laugh], [sigh], [pause] |
| ? | Beginning of utterance by unidentified speaker or questionable text |
| **[inaudible segment]** | Unclear section of the recording |

I: do you give permission to be recorded?

P: yes, I give the permission

I: alright, date uhh xxxx (interview date), location xxxx (clinic name), language used, Setswana, used PID number xxx .. start time 15:32 thank you, ,

I: Where do you stay?

P: Here, at xxxx (area name)

I: Here at xxxx (area name)?

P: xxxx (area name) yes

I: How much does it cost you to come to the clinic? Coming for medication?

P: Uhhh R15 R30 return.

I: Okay …. How do you to come to the clinic?

P: With a taxi.

I: With a taxi, okay. so, how do you know this box?

P: Uhhh box I saw it here at the clinic, when they showed me how it works.

I: Who explained?

P: Xxx (intern’s name)

I: Mhhm

P: Yes

I: How was the explanation given to you about the box?

P: Uhhh when they explained the box to me, they told me they will set an alarm, it will ring at the time I must take my medication.

I: Mhhm

P: So, they set it, it worked properly for 2 weeks then after two weeks it changed. Sometimes it would ring 30 minutes before or 30 minutes after.

I: Mhhm

P: Yes, or after taking my medication it shuts, after 5 minutes it rings again. Then I don’t understand if its network or what.

I: Mhhm….so did you understand the information they explained about the box?

P: About how the box work?

I: Mhhm

P: Yes, I understood.

I: Would you say the information it is easy for someone to process?

P: *ya dieden ya* [this thing] box?

I: Mhhm

P On how can they use the box?

I: The information they explained to you in the TB room about the box, on how it works, is it easy for someone to understand

P: Mhhm… it is simple.

I: So, before they showed it [the box] to you in the TB room had you seen it somewhere before?

P: No, I have not seen it before.

I: Mhhm ... okay what would you say ehh what you say has been your experience on using the box.

P: It was fine because there is no way you could say you have forgotten it even if you are sleeping, when it rings you wake up.

I: Mhhm

P: It was fine, it is just when it started losing time.

I: When you saying it is losing time what do you mean?

P: It does not ring at the right time, let’s say you drink your pills at 6, then it rings at half past 6 already you took you pills sometimes at half past 5 and you drink at 6 … so the problem was only that.

I: So, what would you say worked a lot for you on this box?

P: … mhhm I don’t remember because always when it rings, I would be holding my medication in my hands most of the time.

I: Mhhm

P: Yes … because I was drinking medication when I wake up and go to work before it rings, I would be awake. That is why when it started losing time, I saw that it will mislead me let me return it.

I: So, what would you say were the challenges when using the box?

P: Challenges?

I: Mhhm

P: That was the only challenge, and there is not any other thing, only this thing of losing time.

I: Ehh

P: Mhhm

I: Would you say there is anything you got out of the box except losing time?

P: Yes, I can say that, yes, yeah

I: What are the good things?

P: With the box? Uhh it [the box] was keeping the pills safe, the alarm rings at the time, ehh there is no way someone would hide and say I didn’t hear it makes noise [laugh] it makes noise, u can hear it 10 meters away from it.

I: Do you work?

P: Yes sir

I: Were you travelling with the box to work, or you would leave it behind?

P: No, I would leave it at home.

I: When you were travelling from where you stay would you leave with it?

P: Uhh I haven’t travelled since.

I: If there was a chance that you travel would you travel with it?

P: Yes, it is portable, it can travel it fits in the bag it does not have stress.

I: So, is there any person who ever saw you with the box?

P: Uhh only my family.

I: Who do you stay with at home?

P: My wife and kids only

I: So, first day when you came with the box how was their reaction?

P: [laugh] they asked me where I got the lunch box, [laugh] I said I got it from work, but when I opened to take medication, they were surprised that oh it is not a lunch box, and why it rings [laugh] they say your lunch box its crying, I say leave it its calling me to come eat the food inside.

I: What would u say about their support towards the box?

P: You know, it was okay.

I: Mhhm

P: Yoh Hhhhh it was fine, it is just that when it starts ringing, they shout your child is crying this side. [laugh] your child is crying why is the child crying, go and switch it off it is annoying. [laugh] then I would go shut it, sometimes I would already have taken my dose maybe I didn’t close it properly, then I go open it and close it, then it keeps quiet then later start to lose it and rings just once or twice then keeps quiet.

I: How do you shut it?

P: by opening and closing it.

I: Would it ring again sometimes?

P: Mhhm

I: Alright, is there anyway you would say this box affected your life in the house?

P: Mhhm mhhm No I would not say that. There has never been any complain saying maybe the box its irritating or annoying them. It is only when they are joking saying the child is crying, but it was not a problem.

I: [laugh] how would you feel when they do that?

P: When they do what?

I: When they do that, saying the child is crying?

P: [laugh] I would just take it simple because I know I already took my medication it is just I didn’t close properly.

P: It is not ringing at the correct time, I thought I would return it and they would change it, or they will check it and set it somehow.

I: So, what can they do on the box so that you can use it again?

P: Right now, I don’t know, I don’t know.

I: I mean for you, what can be changed for you to agree to use the box again.

P: Uhh… I don’t know, is it possible that they can do something to what it is?

I: Yes

P: okay I don’t know.

I: Would u recommend someone this box? That somebody uses it?

P: Mhhm (nodding to say yes)

I: and why would you recommend it?

P: No, I can use it, just only if it does not lose e time, sometimes you take your dose, then I would leave to work, leave it at home, then it rings back home, so it will disturb those at home and they won’t know what to do with it because you see time to go to work it is 6 o’clock and time for medication. Then I leave then it rings at 06:30 and when will I come back? Late around 16:30 then it rings all day there.

I: Alright

P: So, you see when someone is not going anywhere at home, yeah, they can sit with it when it starts, they switch it off if they have taken their dose, they didn’t close properly, then when it rings again, they will go and check

I: So, what, the time they were explaining about the box, what is that you liked the most?

P: That alarm.

I: Mhhm you were happy with, this alarm thing?

P: That time, that it won’t be possible to miss time yes.

I: When were you diagnosed with TB?

P: On the 4^th^ of … last month.

I How was you feeling that you have this disease

P: Ahh I took just simple, because I worked with TB patients a lot.

I: Mhhm

P: Just only problem was the side effects due to the treatment intake.

I: Which side effects did you experience?

P: You [laugh] I was vomiting having diarrhea, I could not eat, but later I would manage to eat.

I: So, what did you do when you experienced the side effects?

P: I told them here, then they gave me these other pills they said they are for vomiting yah…… you must know it was my first-time taking treatment like that or even if its not for TB only just pills [laugh] even flu medication it’s difficult for me. But this one I had a struggle, but I said I won’t die because of TB, TB is curable, people have been cured.

I: So, is there anyone you ever told except those you are staying with?

P: Yes, there is this guy where am staying, he usually comes to my place, he likes coming to my place, it rang and he asked me what is ringing, he said what is the alarm for? I said let me show you, I had already taken my dose the I fetched it I said this is what its ringing, he said why its ringing this time? I don’t know it has pills inside, when they have given you, it will be the one to remind you to take your medication time, he said that this thing is smart, there is no way you could forget[laugh] even when you are sleeping kids will come wake your u say the box it is ringing.

I: How did you feel when you explained to him about the box?

P: Uhh uh we are used to talk about such things.

I: Would you say you find it easy to talk to people about the box?

P: Yes, it is easy too much to explain to someone on how it works, it is simple.

I: How was their reaction?

P: Uhh it was okay, he was just happy, he is on high blood treatment, he was asking why it is not offered to them only us,

I: Have you ever opened the box more than once in a day?

P: It is when I open and close.

I: What would be the reason to do that

P: To switch off the alarm?

I: Did you put anything else in the box besides medication?

P: No, it was only medication.

I: Where did you put your box

P: In the wardrobe

I: In the wardrobe?

P: Yes

I: How would you say your experience was when you continued to take TB medication with that when you were using the box.

P: No according to me I have already said that that time to take medication I would be up going to work there is no way I would forget.

I: So, what is the main thing that assisted you with using the box?

P: Hhhhh firstly when I started treatment the alarm was assisting.

I: So, what would you say was difficult in using the box?

P: Nothing

I: Nothing?

P: Yes

I: Have you ever received an SMS say you missed your medication?

P:Yes, often, and even when I took medication, I would still get SMS, sometimes, I would get an SMS to say don’t forget to take your medication at 12 o’clock at night, then I don’t know if it is for me, or it is for who [laugh] or where it is coming from or where it is going

I: Have you ever missed a day without a dose?

P: Mhhm (nodding to say yes)

I: What happened, how did you miss [your dose] ?

P: That time when I started even side effects, I struggled for a week or two. When I took medication I would vomit, then I said am losing weight then I came here to explain then they gave me what they gave me, then I tried taking medication again after two days I struggled again.

I: Then what helped you?

P: Mhhm, I took long to be okay, I don’t know I think the pills I got here.

I: When you vomited after two days, what did you do, did you continue taking the pills?

P: Yes, when I took medication sometimes it comes back, mhhm then after those pills, I would follow treatment that is why I would come here and tell them that I stopped here on treatment, and this is what is left.

I: I don’t understand.

P: Let’s say I skipped 4 days, you take medication for certain duration, when that certain period ends, I would tell them add these 4 days I skipped, I owe these days add it to the new one.

I: So, the days you skipped were they consecutive?

P: No no not consecutive.

I: You would skip sometimes?

P: Yah
I: Then you came to clinic?

P: Mhhm

I: What would do when you get to the clinic?

P: I came to tell them that treatment its coming back [vomiting] , and now it is the treatment that is coming , then they would give me the other pills to stop vomiting because I was not even eating. I would take medication 1 hour or 2 hours before take medication I would still vomit then I would change take medication first then eat 1 or 2 hours later I would still vomit then sometimes I would just eat small portions after hours.

I: How would you feel on the days you skipped medication?

P: Mhhm I would feel that am weak, I was weak, I would see myself that here I am going.

I: Did you receive counselling when you received your TB medication?

P: Oh, they did give me back then when I was still struggling, they said when it starts it will do 1 2 3 but it will get used to body and when it got used there were no issue

I: When you received the SMS, that says you didn’t take medication, and those you missed medication how would you feel about them?

P: No, those one they are boring me, I wonder what they are telling me, that I missed medication even though I have taken medication and sometimes message would come before the time, then I would get SMS before when I check time it has got to take to medication.

I: and you received the SMS already?

P: [laugh] I have received the SMS, and it’s not yet time for taking medication or sometime just when I finished to take medication, I would see SMS coming in.

I: How often would you receive this faulty SMS’s?

P: Sometimes, I remember some week I received 2 in a week sometimes just 1and some weeks nothing.

I: On a month how many would you receive [SMS’s]?

P: Maybe 3

I: Mhhm on a month?

P: Mhhm

I: Have you ever received a phone call regarding your missing your you medication?

P: Yes, just once. That time I was leaving here.

I: When you leaving?

P: Here at clinic, that time I got medication for vomiting.

I: Oh okay

P: Yes

I: They called you because you missed medication?

P: Yes, I didn’t take dose in the morning , then I came to the clinic later then I got a call.

I: How would you feel about receiving a call saying that you missed your medication.

P: I know I was called by xxx (Intern name), he is the one who didn’t see me when I was inside.

I: I mean like a patient that you missed and then they remind you with an SMS again that you have missed medication how would you feel about that method?

P: No Hhhhh it was not fine because at sometimes I will have taken my dose, or you get the SMS before taking dose, then you don’t know what is going on

I: Let’s say for a patient that has missed for real and they get that SMS would they appreciate that SMS that someone is reminding me?

P: Yes, that one is straight, if you have forgotten, you have forgotten there is no way you would get angry. This is for us to live, so when you get angry it means you don’t want to live. You must take medication

I: How was your experience with receiving phone call?

P: Uhh no it was just fine. When I told him it’s not like I missed medication, I was there now I got 1 2 3, and I know I missed mediation for these reasons 1 2 3, I was here at the clinic, then at the clinic they gave me this that is why I missed.

I: Have they ever visited you at home regarding medication?

P: No

I: When someone misses medication for long period, they send health care workers so that they go and visit to check on them, what would you say with that intervention?

P: I don’t know they ever came to my place.

I: When someone taking TB treatment and they want to check on them would you recommend that?

P: If they did not get like before, things like SMS and all this, its not okay for TB patients to stay with other people in the house if they are not taking their medication, they [clinic staff] must go visit them and find out what is troubling them .

I: Have you ever had any disturbance, that have made you not to be able to take medication?

P: Nope

I: So, has the box ever went a day without ringing at all?

P: Yah

I: Ehh what would you say was the problem? Problem ya network?

P: That is what I thought, maybe it is network.

I: How did you feel that day box e salle [did not ring] at all?

P: That [laugh] that was the day I returned it.

I: Mhhm … okay one of the reasons you returned it?

P: I thought maybe it has a problem I didn’t know what was happening. I brought it saying your box it’s not working.

I: How often?

P: Two times, first time it was when I realized there is something wrong because it rang once and kept quiet, I said something is missing here the following day it was just quiet didn’t ring at all then I returned it

I: Okay, how satisfied are you with using the box?

P: Mhhm

I: How can you describe your satisfaction with using the box?

P: It is a lot, I don’t see any problem with the box just losing time, and not ringing some days, it is just fine because it does not disturb with anything, even when you take treatment you can just go with the box without having have to use a plastic [laugh]it is portable there is no one who would know what I have if they don’t know.

I: If it happens that they know what is the box for which medication would you still carry it without covering it?

P: I don’t have any problem [laugh] they have they their own treatment, why would we be afraid? TB, we know that TB is curable unlike having a lifetime disease.

I: So, when the box was working correctly, how would you say it has helped in not to miss your doses?

P: Just with time only when it rings at the time.

I: You said that it keeps your medication safe

P: Yes

I: How does it keep medication safe?

P: They don’t scatter around it stay in one place in the wardrobe

I: Is there anyway the box helped you with not coming to clinic a lot?

P: No, I don’t know there, I would come here every day, because of work.

I: See if they can explain your satisfaction in a certain manner what would you say?

P: Regarding? The box?

I: Mhhm

P: The box does work, and it helps even someone who doesn’t know when you start to explain and give them medication, with it will help them and a lot, they would never forget treatment. If they have forgotten a long as they are around it will ring, they will remember, even when they are outside or wherever they will her it and they come rushing to it to take medication.

I: So, what can be done to make the box easy for people to use?

P: Ehh I don’t know, does it use a battery?

I: Yes

P: If it has battery, they must give someone for certain period, then they bring it back to be checked, then give it back, it will be simple like that. Because I don’t think it can last for a month.

I: They didn’t tell you about battery?

P: No, they didn’t explain anything.

I: Okay I will tell you, what about the volume?

P: It is fine.
I: And then … what can be done about the size?

P: Box’s size?

I: Yes

P: No no its fine none must be changed [laugh] it is just fine

I: Do you think if they can use voice on the box to remind people about missed dose instead of SMS would be better?

P: Yes because SMS sometimes when there is no network when will you get it? You will get that the SMS was sent today, and you get tomorrow then you are left surprised that you took your medication today but your receiving SMS late and it was because of network, so if it was shouting it will be just fine[laugh].

I: You said the size has no problem.

P: I have no problem with it.

I: What about the language, color and material?

P: They are just fine.

I: On the SMS the way it is sent its easy for one to understand?

P: Ahh what can I say? For now, language is a problem. It will be better if they send the SMS in different languages.

I: What do you like and dislike about the SMS?

P: SMS you won’t know when it is sent

I: Please elaborate on that

P: When you receive an SMS that was from yesterday and you only seeing it today, after taking medication but you are receiving SMS and you know yesterday you didn’t take medication but today you took it, so you won’t know it’s for yesterday or today

I: What do you dislike about the phone call to remind people to take medication?

P: It has no problem.

I: What about the information that you got about TB when you started treatment, what do you think can be done to make it more effective?

P: The ones the nurses do? You know, we have 3 patients that are new, they should be taken into a room then explain to them about this thing, so that when someone get home, they don’t get surprised by many things [laugh]

I: With everything that go along with the box, SMS, phone call and alarms what is that you see working more to help people on TB treatment?

P: With phone call and SMS?

I: Yes

P: You see a phone call?
I: Yes

P: Let me start with the box it’s fine.

I: What do you think does not help most?

P: SMS, ahh it is not working at all.

I: SMS?

P: Mhhm

I: How can we improve? if we hand over to DOH (Department of Health) what do you see as something we should improve so that this thing works well?

P: With phone calls and alarms? With phone call we respond, and SMS is a waste of time.

I: You mentioned have newly diagnosed in a class to be taught about TB

P: Yes

I: Who must teach all these things?

P: I don’t know, anyone who is working in TB

I: Who can explain to them about the box.

P: Any trained person. Someone who is trained, is better than the nurse because the nurse is just here and receive this thing, they can explain it better.

I: Uhh we are reaching the end, is the anything you would like to share about this box you feel we didn’t talk about?

P: Uhh Uhh just the battery.

I: Battery… okay is there anything else you feel like I didn’t ask you about and you would like to say about your experience with the box?

P: No, there is nothing, box it works perfectly just the problem starts when the battery or if its network, I don’t know what it is, starts to lose time because if it was somebody else who didn’t check the time would miss medication waiting for the box to ring and it rings 30 mins later

I: Okay do you have any comment regarding SMS, phone call and home visit?

P: Mhhm uhh phone calls it is fine; it must be used as well as alarm. Home visits are also necessary, most people are dying because of TB and TB does not kill anyone who is taking medication, and some will tell you they don’t have food, or they are not working, so home visits help to check on hem.

I: We have come to end of our interview, we thank you for your information that you gave us it will help too much.

P: So many people die my brother they die from this thing of someone staying alone, and they don’t work, they don’t have food, and they must drink medication and you can’t take medication without eating, you will speed, so here the home based care must go in the field the check people., They (home based care givers) must know how many people are in the section, all the houses have home based care, so they must get the list of those patients and check up on them, and those who are at home are better because at 8 they are done with home chores they can go check on them if they are taking their medication.

I: Such patients how can the use of box assist?

P: You will get them saying they don’t have food even it rings they will just look at it.

I: Oh, the challenge is the food.

P: They want, home based care must check on these patients, and if things are like that social worker must be involved with food parcels. But if they get someone who is not responsible, they take the food parcel and they sell them to take the money and go drink alcohol with. So, it is trouble so if the person lives well and live with other people well there is no problem with the box

I: Do you anything anymore to say?

P: No

I: We thank you for the information, that you gave us. ending time 16:08
