# Supplementary material for: Acceptability of Digital Adherence Technologies to support people with drug-susceptible TB in South Africa
Source: PLoS One. 2025 Sep 24;20(9):e0332103. doi: 10.1371/journal.pone.0332103 (PMC12459780; doi:10.1371/journal.pone.0332103)
Supplement: S4 File — (ZIP) [file pone.0332103.s004.zip › S4 Transcripts/PwTB/IDI 3_PwTB.docx]

**TRANSCRIPTION NOTATIONS**

| **Label Key** | **Meaning** |
| --- | --- |
| **I** | Start of each new utterance by the Interviewer |
| **P** | Start of each new utterance by the Participant |
| **N** | Note taker |
| **{ }** | Indicates that details were changed or pseudonyms were used to anonymise data |
| **( )** | Indicates the description provided to anonymise data |
| **XXX** | Words were omitted to anonymise data |
| **-** | Breaking into a sentence by the next speaker |
| **…** | Pause or drawn out words |
| **[ ]** | Indicates noise made, e.g. [laugh], [sigh], [pause] |
| ? | Beginning of utterance by unidentified speaker or questionable text |
| **[inaudible segment]** | Unclear section of the recording |

I: Are you agreeing that this interview be audio recorded?

P: Yes, I agree.

I: Okay, date xxxx (interview date), location (xxx clinic name), xxx, time 11: 45 am, language used Sepedi (…) So, shortly papa explain where you are staying and who stay with?

P: I stay with my wife, my daughter, and grandchildren.

I: Okay, so papa please tell me when you are coming to the clinic, do walk or catch a ride?

P: Ehh, Ehh, I walk.

I: How far is the distance from home to the clinic?

P: [cough] Distance, I don’t know how to put it (…) or 20 kilo (km) ehh, it a distance.

I: Okay, papa are you aware that you have a lung disease?

P: No, I didn’t know until I came here (…)

I: I mean lung disease, TB.

P: Yes.
I: Is it the first time you hear about it?

P: I used to work at XXX [Place of work], I know about it from photos that shows that it is not flu, but TB, but I don’t know how it works.

I: So, papa you are saying you heard people talking about it when he used to work at department of health-

P: Mmm (yes).

I: Briefly, what did they say about TB disease?

P: *Nee* (right*)*, from my side. I only know about the coughing, that when someone coughs, I knew what it could mean.

I: Papa, do you know this label or sticker?

P: What colour it is? My eyes are not seeing well. Is it the red and white?

I: The one you used when you took your TB medication, and you sent SMS with your phone.

P: I Ohh I remember it now but I did not use it for long because it was giving me problems to send the SMS so I stopped. I don’t remember much about it though.

I: Okay, (…) so, this box that is used to put medication, do you know it?

P: I started seeing it when they sent it to me.

I: When they started you on this box, who explained to you about this box?

P: No, I found it there when I got home, (…) [phone sound] they told me it will clock (alarm reminder). When they brought the box, it was a time where I had ran out of pills, they asked me when they finished, I said I don’t remember, then they brought the box.

I: Who brought the box where you are staying.

P: It was the nurses, I don’t know them well, I just saw a blue thing. They come in uniform and then when they start, they said- what-it was xxx (intern name), I don’t know if it was xxx (intern name), but I think I saw xxx (intern’s name) who came.

I: So, on that day when they brought the box, how did you feel when they brought this box.

P: I was happy, I was very much happy .I knew I won’t have a problem anymore; I will know when it is time to take medication, take my pills.

I: So, are these pills meant for the disease you have?

P: Yes, I just found out, I heard them (nurses) saying they are for TB, but I didn’t know, I just get pills like this so (…).

I So, how did you feel when you discovered that you had TB?

P:Uh , I didn’t even have a problem because I didn’t even know that it was TB. I don’t know if it pains or does what . I just found myself taking medication, and I didn’t have a problem because am a person who is training (marathon runner). I didn’t understand, even when drinking my pills, I didn’t have a problem of what was affecting me [clearing throat]

I: So, according to your experience of using the box-

P: Mmm-

I: Ehh, what was one of the challenges you came cross when using the box?

P: You see this box? It was not in my heart, I was just taking my pills when it was sounding. I drink, uh, I don’t have a problem with what I was suffering from or feeling pain because of the disease, and even the way they spoke to me-the doctors didn’t express hard that I have TB, everything was smooth, and they gave me my pills-my treatment. Then I got my check-up, and I don’t have a problem with them (HCW). Even when I go to the hospital am not scared or shy to stand in queue or being in a group of TB patients, I don’t have a problem. I took it as disease and didn’t take it to heart, even if someone comes and talk about it, I wouldn’t hurt (…).

I: So, papa briefly-

P: Ehh.

I: Can you please explain how the box works?

P: *Aow* (hey), [laugh] this box is number 1(the best), that is my summary, it is number 1. Am still amazed- when they brought it, I found it to be first class. I can hear it ringing when am sitting and it is better than when I was still taking medication from plastics (pill packet). So, the box rings and I know I have to take out medication, it is alright.

I: So, can papa please explain the way the box helped him to take pills?

P: It helped me a lot, even the way I take my pills, I didn’t have any pain, I didn’t have. I would hear it ring and this disease- I don’t know how [interruption (…) *dumelang* [greetings] how it is, but as they said- even when explaining to me that I have TB, they spoke softly to me, it was not like someone talking harsh about the disease that it causes pain.

I: So, when papa say the box rings, you would take your medication?

P: Yes, boy.

I: What time did you take your pills?

P: Uh, around 9 o’clock mostly. When I hear sounds, and I realised that it is to 09:00 even when the sounds comes from somewhere, I always think it is the box. I remember that it is 09:00 or to 09:00, mmm.

I: So, at home, did you tell them you take medication? Or they were surprised when they (CHW) brought it home.

P: Yes, yes, I was already drinking pills, and they (HCW) would come to bring them in plastics, one time ehh, they came and said we will give you this box, so that you can know the time to take medication and help you. It has helped me because it was clocking (alarm reminder), and I would put the pills packet in there. I would forget to drink them sometimes but the box was the best.

I: So, when papa say he would forget pills at times, please explain here, how did you take pills without the box?

P: Uhh, I actually don’t remember anything, but the box is number 1 (the best). Sometimes I would put it somewhere and it would just ring even when am close to it. It was number 1.

I: So, on the day they brought you this box at home-

P: Mmm

I: What were your worries on the day they brought the box for first time?

P:Uhh , I didn’t have a problem, that is why I tell you that I didn’t even know TB , even when they said I have it, it didn’t sound bad. I took it like just any other disease, mmm, I took it like any disease not like AIDS as if am going to do blood test now. I would not be scared that maybe I could have a disease like AIDS, no, I would test (…). Am not scared of diseases that have no pain, mmm, the one that gives me pains would worry me like if am going for operation and they cut me. Cutting is what would be painful, but TB, I didn’t feel anything.

I: Okay, thank you. So, about this TB disease, are there any people you talked to about this disease?

P: No, it is like- I didn’t have anything. There is no one who know anything, mmm. It is only my wife who would know because she is close to me.

I: You said at home you stay with your wife-

P: My daughter-

I: Your daughter and grandchildren.

P: Yes.

I: So, do they know about the box and that you take TB medication?

P:Uhh, maybe their mother told them as for me uhh, uhh, I don’t talk about such things, saying I have TB uhh, uhh. The adults can be the one to talk about it, you, see? No, they don’t know.

I: So, shortly, can you please tell me how your wife felt about you being given the box [cough]. How did she feel when she saw you taking medication from the box, knowing you have something that gives you support to take medication?

P: *Hai* (no), from our side-maybe her-maybe she had different opinion, but for me I was accepting the box well and am thankful. If I had money, I would buy you coke. I was happy that the box will clock (alarm reminder) to me which was better that opening the plastics when drinking, you see, I didn’t have any problem.

I: So, papa you just said, no one knows [cough] that you have TB disease.

P: Mmm yes, no one knows.

I: Can you please tell me why you didn’t tell anyone about this TB disease?

P: Oh, only my wife knew, only my wife knew. She is the one I live with and others are not the people I can talk to about such things, maybe she is the one who can tell them, mmm. It not in my nature, I don’t speak about such things.

I: So, you say you do not speak about such things-

P: Yes.

I: So, as a person who lives with this disease, within our society there is stigma around TB disease-

P: Ehh, ask again.

I: As you did not tell anyone, except those living with, could it be because within the society there is still an issue of mistreatment towards a person who lives with TB?

P:Uh, for me like I said, on this TB thing; I didn’t tell anyone, and I didn’t see any problem because I had TB. There is nothing, even now there is nothing on me. Even the way I train; I am a person who loves jogging and I don’t have a problem being sick or what, uhh, uhh, nothing. I understood when they told me that I have TB and I have accepted it well. It didn’t even bother me.

I: Have you ever opened the box more than once a day?

P: Ehh, to open?

I: Yes, to open.

P: It gave me a problem at first, I struggled to open it for the first time because of the way the lid is designed ,but after one attempt, I said oh, this is how we open it. Then I was able to open it until I finished the course, I was happy to finish what I was given. I loved the box, sometimes I wished I could use it as lunch box. Am enjoying it.

I: So, when you found out you have TB disease, did people you are staying with at home come to test?

P: Yes, but for regular check-up, mmm. I once came with my child and grandchild.

I: And then, when they tested , what were the results?

P:Uh , it was children’s matters, and later that day the sisters (nurses) came by my house. I saw them talking to my daughter- it was children’s matters. They spoke but I don’t know what they were talking about, they didn’t concern me about what I have.

I: So, at home, is there anyone who had TB before?

P: Oh no, maybe uncles’ family that lives far from us, no one has had that thing at home.

I: Papa can you please tell us that at the time the nurses came to your place to test you and your grandchildren, after testing, how did you feel when they said no one at home has TB disease?

P:Uh, when they tested us- we were here- at home, mmm, mmm. They didn’t come to test, they came to check if I still had pills and if they are finished, so they would bring more. It was during the time where I couldn’t come here because I had an eye problem. I wouldn’t walk alone because I couldn’t see, I could hear sounds of cars and other things, but eyesight nothing. It is getting better now; I can see better now.

I: So, back there you said, you were happy with the box that you even wanted to make it lunchbox.

P: [laugh] Ehh, ehh, it was beautiful, the plastic thing- this plastic thing was giving me work. Some of them (pills) were open and some were still sealed, but I would end up opening and drinking from the new pill packet because I cannot find the opened ones (pills). Then with the box, it was number one (the best). So, they would be packed inside the box, then it rings and I would unclip (open the lid of the box), take out and drink. It was first class, it does not have a problem.

I: Okay, was there day you put something else in the box?

P: No, uhh, uhh it was only the pills (…) it was only the pills.

I: So, papa do you have a cell phone?

P: Yes, I do have ,but I don’t like it [phone noise], sometimes it is this marketing- my wife is the one who uses it.

I: Mmm so, is there a day papa received a call to remind him to remember take his pills?

P: No, mmm, mmm, no, I haven’t received a call. I was taking medication by myself, am actually someone who [cough] does not like shying away from my problem. Am someone who is always in the house, am able drink and come back. I don’t just go, and even if go, I don’t go far without drinking. There is never a time where it rang, and I was not around take medication, am always with them.

I: Okay, no thank you. So, is there a day you had a home visit to remind you to take medication?

P: Only xxx (intern name) came or she called, I think she called. She was asking if I had enough pills, I said uh ,they are finished. She then asked when did they finish, and my response was, uh I don’t remember, this week, I don’t remember. “Okay, they are finished,” that is when they brought the box, I was happy, I said yes, you did well.

I: When you said they are finished, what was it that was finished?

P: Oh, it was the pills.

I: What were you speaking through? Cell phone or what?

P: Mmm, mmm, no, we spoke through the phone, yes, we spoke through the phone. I think it was her, xxx (intern name), we spoke here.

I: So, you were receiving calls?

P: No, they called me just once, I had a problem- I didn’t have a phone.

I: So, when they called you, you said pills are finished?

P: Yes.

I: So, when they are finished, what would you do?

P: I would wait until I come for check-up (…) the check-up was not far; I would say it was on the following week (…) this TB thing, I didn’t understand it and I didn’t take it to heart.

I: So, shortly papa, according to your experience-

P: Yes-

I: How did you feel about being visited by people from the clinic to talk about the TB illness?

P: No, they were not coming to talk- they were coming to-they were passing. They greeted me, asked me how am I doing and if I still have pills. It was not like they were scaring me or anything, I enjoyed them (home visits). Actually, I don’t have a problem with this disease, I don’t know it (…) see if you are someone who is training. I run marathons and when you run, even if you are losing weight you think it is because you are running and I would enjoy when I was losing weight knowing I won’t be heavy. it’s not like when you are running you becoming fat anyways, you see. So, I thought I was losing weight because I run and not because of the disease, even my other brother said why you losing so much weight and I told him that I don’t know, but it was not bothering me.

I: So, when you say, you are slimming because of sports, how are you feeling now?

P: *Hai* (no), am number 1 ( feeling okay), am number 1, I don’t have a problem. Am someone who- huh- uhh, I don’t have a problem.

I: So-

P: *Askies* (excuse me*).*

I: Carry on.

P: Even the things the doctor gave me- even when my siblings come, they said *aow* (no ways) it is like you picking up weight, it is like you are fresh, things like that.

I: So, at the time you were sliming or losing weight-

P: Mmm-

I: How were you feeling on your body at the time you were losing weight?

P: Uhh, uhh, no at that time, I did not understand myself. I am a person who loves to run and when I lost weight, I thought it was because of running- you know Khoza street?

I: Sorry?

P: Khoza street that side, that side of kalafong.

I: No, I don’t know it.

P: It is the street that has steep hill, I run like nobody’s business, I would go turn at escor side, return with church street again because am coming from Lotus and you see, I cannot have a disease, it found me healthy.

I: So, papa please tell us if there was something preventing you to use the box.

P: Preventing, how?

I: Barrier, like something would prevent you from using the box-

P: *Nee* (no), I had no obstacle, I mean when they spoke to me for the first time, the box was not even there , I was using the plastic. I would drink-I was enjoying because of it (pillbox) telling me that it is time to drink. Those packets of pills were not doing anything, but the box, I loved it because it was showing- mmm, it is clocking (alarm reminder).

I: Okay, you just said your pills were staying inside the plastic?

P: Mmm.

I: According to your experience with the box, can you please explain, how the box helped you keep your medication safe.

P: Ehh, ehh, it helps too much, but the clocking (alarm reminder) is what I liked the most

I: Mmm.

P: I was enjoying too much, and some pills were - others were packed like this [demonstrating using hands] even when I open the box, I would easily take out the one on top. With the plastic ehh, those pills were just scattered and some would have fallen. Sometimes I would start a new pack because I couldn’t find the one I opened, you will find packets that are half, half. So, the box gave me a system.

I: Mmm.

P: I praised it a lot, right now I don’t know why I forgot it, I was supposed to bring it.

I: So, papa, papa, the following question is about feelings, how satisfied are you with the box? According to you, as someone who used it.

P: I like it too much, plus I didn’t know about this disease, I always used knowing am dying from something I don’t know. I would see on the card-papers and what- sputum. It is like here at the clinic or hospital they had these photos, but where I was working, we had uhh, library where these books stay, we had booked a lot of books, some days [inaudible segment], I would take the books just to read. Ehh! Like I said I am not a person of this TB, but I used it (referring to the box) serious, because I don’t know this TB disease . So, I use this box , I used it so I can heal from this disease -I don’t know. I was so happy when I had something like this box, I didn’t know it, but I listened about their treatment when they said this and that. I take this pill serious, so that I cannot be in the TB ranking.

I: So, papa for someone who is taking TB medication, do you think this box can work for them?

P: Too much, because it is the one that pushes one to take medication because it has a siren [alarm] part. It is alright, it tells you am here, because it is like a cell phone when you can’t find it you request to be called so you can locate it, now this box is first class, seriously.

I: Besides the alarm on the box, what else did you use to remind you time for medication?

P: I was placing it just next to my bed, I was placing it next to me.

I:So, papa would you hear the alarm?

P: At first, when it start ringing, I would think it just an alarm because I didn’t recognise the sound before. It would make that sounds [ making box alarm sound] until I realised- I said oh, uh , it is the box. I was not used to it, so I could not get if it was a phone alarm or what. I love it for that.

I: What is it that papa like and doesn’t like when people from the clinic come to visit where you are staying.

P: Mmm, mmm, I had nothing that I hated, I was enjoying, and I liked it. I don’t have a problem with them (HCW). It’s more like the ambulance people saw on the street, I have forgotten that the ambulance once came for me, even though others would still remember. I don’t have a problem because my smile is clean, you know when you watch sports, it makes you to feel happy, even running. Am one person who cares about myself ,I don’t care about somebody else. I think about my life.

I: Oh, am glad to hear that, so you said that xxx (intern name) or the one that works here called you one time only.

P: Something like that, asking how things are going, I said they are finished (pills) and she said she will come. She asked when were they finished, I said this week, but I don’t remember well on which day, she said okay, I will bring for you.

I: So, when they called you, what is that you liked and what is that you didn’t like when they called?

P: No, I don’t hate anything, I was enjoying everything, when they called there was never a problem, rather I didn’t feel any pain or bothered by them (HCW) calling uhh, uhh, I enjoyed the thing they called for. I said am having an eye problem but am healing now.

I: So, according to your experience, this thing of calling patients to remind them to take their TB medication and even to visit them at home and getting SMS’s. According to your experience, do they work, these activities?

P: Yes, to me it doesn’t have a problem-it was because of how I was, but I was going to come myself, but I have eye problem and now am afraid to walk on the street. It is like am a blind man, so I was afraid of such until they called, I was happy they are reducing my work. I didn’t go outside if it wasn’t for my eyes, I was going to come myself.

I: For someone who lives with a TB disease, and not taking their medication. What is it that we can do to make sure that people take their pills?

P: Ehh! People are different; we people are not the same, there are people who don’t care about their lives. What I like about my life are simple things, just to do my check-up. No, they are making a mistake these people.

I: So, for those who are making a mistake, what is it that we can do as a clinic to make sure that people take pills. Advice you can give so that they can know and make sure that they take their medication.

P: You see from my side, we are different as people, there are people living without exercise, they wake up, eat, then sleep without exercise. So, from my side, I mean from my side, I cannot fail to take my treatment, check-up, then people do this because of these things am telling you, but some people don’t care about their lives.

I: So, according to you when you look at the box, do you think this box should have been started back then?

P: Eish, hey, hey that time it was still that government, yeah, uh, black person must not- they were just giving disprin only. The disease will hit you hard, so, for now they have worked too much.

I: So, according to your experience with the box, papa do you see this box helping people take their medication?

P: Yes, yes, it is user friendly, if you can go according to it ,you will always enjoy it and love it. There are shy to fix themselves [referring to some patients in generally]. The box is enjoyable, so I take the hat off for the box.

I: So, papa according to your experience- according to this box and again, when you look, is there anything you feel like they should have done on the box.

P: Mmm, mmm, what I liked a lot is the light, when it shows the light. The light show that the box is working. It works like the charger when you have plugged it. It will show the green like- it would switch on the light to show that it is working, same as the changer. This box too shows lights.

I: In short, am left with just two questions, I had one initially, can you please tell us about this light you say the box has, can you please explain about how this light works.

P: Nee (right), the light work to show that the box is working, I didn’t pay attention if it has others on the sides. The green light show that the box is working.

I: So, this light, you are speaking of, the green one is the one to show that the box is working.

P: Yes.

I: So, according to you papa, how many lights does the box have?

P: I saw the green one, I didn’t look at all sides. I only saw the one that was visible. I didn’t see whether it shows lights on the other side. I only saw the one that was visible on the side.

I: So, papa we have reached the end of our interview.

P: Yes, but *dankie* (thank you*)* for the box, must continue, but I hear you saying will you officially offer boxes after 10 years.

I: No, for 10 years, it is the information we are talking about, we will keep it and use it for 10 years.

P: Oh*.*

I: So, according to the box papa, for our final question or our last question, can you please give us the overall about the box and about the TB pills and TB disease. Please tell me what you think about the box and TB disease.

P: This box is the one that heals, it heals when it rings, it says come on to drink. It is alright. Jokes aside, it is alright, others must not speak bad about it.

I:So, papa we have reached the end of our interview for today, I thank you for your time that you took to come here, taking your time to come and do the interview, we are done, thank you for all your answers that you gave us.

P: I thank you for calling me.

I: I thank you, but we are done with the interview, 2556.

P: Is that my number, what is it?

I: 2556

P: 2 double 5, 6.

I: Yes.

P: I used to work at health my brother.

I: Time ended 12:45 pm.
