# Supplementary material for: Acceptability of Digital Adherence Technologies to support people with drug-susceptible TB in South Africa
Source: PLoS One. 2025 Sep 24;20(9):e0332103. doi: 10.1371/journal.pone.0332103 (PMC12459780; doi:10.1371/journal.pone.0332103)
Supplement: S4 File — (ZIP) [file pone.0332103.s004.zip › S4 Transcripts/PwTB/IDI 4_PwTB.docx]

Translation Setswana

| **Label Key** | **Meaning** |
| --- | --- |
| **I** | Start of each new utterance by the Interviewer |
| **P** | Start of each new utterance by the Participant |
| **N** | Note taker |
| **{ }** | Indicates that details were changed or pseudonyms were used to anonymise data |
| **( )** | Indicates the description provided to anonymise data |
| **XXX** | Words were omitted to anonymise data |
| **-** | Breaking into a sentence by the next speaker |
| **…** | Pause or drawn out words |
| **[ ]** | Indicates noise made, e.g. [laugh], [sigh], [pause] |
| ? | Beginning of utterance by unidentified speaker or questionable text |
| **[inaudible segment]** | Unclear section of the recording |

I: Today…today’s date is xxxx (interview date). We’re at xxx (clinic name). Uhm…our conversation is going to be conducted in XXX[Language]. Uhm…PID of a patient is xxx. The time to start our conversation is 10:35. Uhm…hello pa.

P: [inaudible segment]

I: My name is xxx [interviewer’s name]. Uhm…our conversation will start by now. Uhm…I would like to ask you the first question. Uhm…my first question is – don’t say your name pa, right.

P: Yes

I: Because names – your name is not necessary in this conversation.

P: Okay.

I: Yes, may you please tell me something about yourself maybe?

P: Uhm…I’m the parent of one kid. I stay at a place – here at xxxx (area) where I have a wife and a kid. Uhm…up to so far – I love my family more than most of the things [inaudible segment], yes.

I: So, just because you explained that you stay with – you are a parent of one kid –

P: Mm

I: So, do you stay with that child and a wife?

P: Yes, I stay with them.

I: How did you know that you have TB?

P: Uhm…I have…what do we call it? Uhm…*sibari* (brother-in-law) [laughing]. I don’t know what they call it in English, it is *sibari* (brother-in-law), do you get it? M…m…my wife’s sibling, uhm…he had TB, they said it the more complicated one , that I don’t understand.

I: Yes.

P: Yes, right, he had visited us at home, right? He had visited us at home for few days, he had been invited by his sister, so that at least he could stay with us a bit. No, we stayed with him there at home. Hence, we stayed with him in the house in such a way, we were forcing him to come to clinic to test for TB. It is just that the person was coughing continuously, do you get it? His sister was always fighting him saying “no, go to the clinic to check if you don’t have TB man,” until he was admitted, uhm…I was also telling him that brother xxx [brother-in-law’s name] , just go and check man to see what’s going on, then he ended up coming and tested. When they checked him, they found out that he has got TB, they said it is a more complicated TB. They said all of us in the house should come and check if we have TB, wife, child, and I. We came here at the clinic - as for them they came the very same day that uncle came on, then they gave them the containers for all of us to spit the cough fluids inside, so that we could bring back those fluids for being tested [snot]. Uhm…when the results come back that’s where I found out that I’m infected of TB. When they came back, no, all of them in the house were not infected, I was the only one who was infected just because I was always with him most of the times, it's just that I don’t know if maybe I inhaled it or what happened there, do you get it? We found out that no, it’s only me who is infected in the house. All of them, they don’t have it. Indeed, that’s where the treatment started, right? No, they called me here at the clinic, they gave me pills together with these stickers. Indeed, I was not even aware of these stickers. The only thing that I knew was that we should drink the medication, we don’t even stick. They gave me the stickers and they told me to SMS. I said, “would these stickers work for me?” No, I took them. Uhm…indeed I used them, right? For them…when I missed, like to SMS, they would SMS me and say to me “hey, sorry, you missed to take your medication today,” do you get it? Right, then I could be able to recall “oh…eish…what should I have done?” Then that’s where I start taking my treatment. Indeed, I take my treatment seriously, just because I live with an illness my brother, do you get it?

I: Yes

P: I take chronic treatment; do you get it?

I: Okay

P: Yes, so, eish…pills are life my brother, do you get it? Life is yours , there is no other person who could live for you. You should live for yourself. So, everything, that you are doing, you do it for yourself, you don’t do it to please the other person. Life is yours; you live once, do you get it? You live for yourself. So, eish…I don’t know, for my side this thing of stickers helped me several times, it helped me a lot just because, uhm… sometimes I would forget to intake my treatment – I know that if I didn’t SMS, they could send me a message saying “hey, and then what?” Sometimes you would find that I’ve already taken them, but I told myself that I will send an SMS maybe after two minutes, after few minutes, hey, the sun is set, then late during the night that’s when I remember – the fact is that I have taken my treatment just because its my life – I don’t do favor to anyone. Even if I could do that thing…a fraud, then I do a fraud for myself, I don’t make it for somebody. So, on my side these things (stickers) and my treatment encourage me a lot. I don’t believe that there is a day where I ever missed to take my treatment, just because they always remind me, so, if you don’t SMS that’s where they could call you straight, saying “papa how, did you take your treatment? What’s going on there?” do you get it?

I: Mm

P: They always update us “what’s going on there? Do you take your medication?” sometimes they could come and check you at your house.

I: So, my brother, may you please explain to me, uhm…is this clinic your nearest one to where you stay?

P: Yes, the nearest clinic is this one.

I: So, from where you stay, what do you use to come here?

P: Uhm…right, I have a car which is mine. Most of the time I use my car, I have two cars. I have a Litra and a van, uhm…when I come here, I just go out – now I came using fourteen, I parked it outside.

P: Mm

I: Uhm…these stickers of yours, do you know – for the person who doesn’t know about these stickers, what would you say to him when explaining them?

P: Uhm…these stickers I could take them as something that reminds you about what you should do, about your medication, do you get it? So, that you will be able to take your medication – they remind you about them. When you take out a pill, you see, “oh! I should send an SMS after taking my treatment,” I should SMS, they give you their numbers, those numbers are not similar; the numbers are different. Yes, when you are done with this packet, you go to the next packet and so forth, each packet has got its own numbers.

I: Mm

P: And then I should SMS one number, one packet. So, in this way I know that after I drank my pills, I sent the SMS until that packet is finished, so, that I could move to the next packet, I know which number I should SMS again and again.

I: Okay

P: Mm

I: Uhm…by the time you came here at the clinic, when they gave you the stickers who was briefing you about them.

P: Uhm…is that sister, the small one, who do we call her? It seems like she is no longer here when I recall very well. The other one who is whitish so, who is bit thick, not this one. This one came after, they were working together, she might know her, she was the one who explained

So, was she the sister who is working here at the clinic, or was she the intern who are wearing red T-shirts?

P: Yes, it seems like she was the one (intern) because she is the one who explained how this box works, when I remember.

I: So, by the time she was explaining to you, how long did she take?

P: Uhm…it didn’t take a very long time [inaudible segment] – it didn’t take long just because she was also giving me the pills, in this way she was giving the pills to me while she was busy explaining how they work.

I: By the way she was explaining, did you understand it - so that you could understand it - did you understand it for the first time, to the point where you realised that no, I could take these stickers and go with them home and you would be able to use them?

P: Yes, she explained to me well, I was able to understand that when I’m at home, I could be able to use them just because she told me that after a packet there are the numbers, indeed I SMS the numbers of each packet, so that there would be no mistake that I could make. Indeed, when I start with packet, I start with a number. And then when I finish with that packet - when I start with another packet, I know that oh, it’s going to have its own number.

I: Mm

P: Mm

I: Uhm…by the way she explained to you, right? Could you say the way she explained to you, is there anything that maybe – she explained to you, right?

P: Mm

I: Then you went home, then you started using them. Is there anything maybe that you would like to change about how she explained to you? Just because you are now using them, you have used them for long period, uhm…when you explain them what would you say? Let me put that question in this way.

P: Uhm…when I explain this thing to somebody, uhm…right, life is yours, this thing (sticker) is going to work for you, just because it’s a reminder. It’s always like a reminder, something like that, do you get it?

I: Yes. Uhm…these stickers, before you came here at the clinic, did you ever see them or hear that there is someone whom you know using them?

P: No…indeed to be honest I was surprised because even myself – it seems like by the time TB started affecting me was the time the stickers started to be available, do you get it? [laughing]

I: [laughing]

P: Yes

I: [laughing]

P: I knew most of the people who were on TB treatment, I didn’t even see one of them having the stickers or SMSing or doing something, no. He was just taking the treatment, it didn’t matter whether he continued to take them or whether he leave them, if you collapse, that’s yours but treatment is the best, you know my brother.

I: So, what was simple on using those stickers of yours?

P: Uhm…right, to SMS –

to SMS was not difficult because we just enter these numbers to send the SMSs to once and then I just enter that code

– it’s for pills that I intake only. There is nothing difficult that I recognize or that is happening, no, there is nothing difficult there.

I: Yes, may we please wait a bit [silence]. Uhm…My brother, did you ever get any difficulties by the time you were starting to use those stickers of yours just because maybe you had no phone?

P: Uhm…Sometimes it’s difficult just because, it happened that I lost my phone and I was unable to send an SMS during that period. Eish... I was bit worried during that period.

I: Like when you lost your phone, did you ever come to the clinic and tell them that I don’t have a phone now?

P: Yes, I came “uhm…sister, no, I’m requesting to use the numbers of the one I’m living with, in the same house.”

I: Yes

P: Mm

I: Uhm…did you ever find yourself having a problem of dead phone battery, whereas you had already taken your treatment, so, you had to charge it and the electricity is not available while you were supposed to send an SMS?

P: Uhm… it’s unusual my brother because I have a car, I’m able to charge it in the car. Do you get it?

I: Yes

P: Mm

I: Did you ever get any difficulties because of network?

P: Yes, network is sometimes bothering me a lot, my brother. It’s sometimes bothering. Sometimes they could tell me that uhm…that thing of mine…my message has failed, do you get it?

I: Yes

P: Uhm…but uhm…I’m now able to go back again, I could redial that numbers, I start them afresh and when I send them, they could go through. I also get surprised sometimes when they tell me that I didn’t send in that way.

I: Mm

P: Mm

I: So…are you working?

P: Yes, my brother, I’m working.

I: Uhm…what time do you leave when going to work?

P: Uhm…most of the time, I leave the house at six.

I: Okay. At what time, do you take your treatment?

P: Uhm…most of the time I intake them around ten, at ten, uhm…is after tea, after tea it’s a time to take my treatment.

I: So, do your colleagues know that you are taking TB treatment?

P: Yes, they – yes, to be honest my colleagues know because uhm…at my work most of the time they need to know if we’re alright, or what’s happening, so that the people you are working together with, couldn’t be affected, right?

I: Yes

P: Mm, most of the time, we’re working with learners in there, I’m working at xxx (place of work), do you get it?

I: Yes

P: There are some learners in there, if you are someone having TB you should disclose, so that the other people could be aware, it’s just that we’re living with many people in there.

I: Okay

P: Mm

I: So, by the time, you found out that you are infected with TB, did you realize that you are forced – you are obligated to tell them, or had it been your choice to disclose that no, I have a TB, I tested positive and I’m taking its treatment or how?

P: Uhm…I had to disclose just because mostly uhm…I was not the first one with TB there at our side, yes, uhm…honestly maybe I’m the third person who is having TB in that section where I’m working. Two of them had also appeared, otherwise we were also aware of them that those guys have TB, and they are on their treatment, do you get it?

I: Yes

P: Until they ended with their treatment, do you get it?

I: Alright

P: Mm

I: Uhm…at your work, do you work at one place or are you supposed to go all around?

P: Uhm…I’m doing maintenance, I’m going all around.

I: Are you working like at…at – when I say at one place, it’s like a yard where you are working only, are you supposed to go outside a yard, when going to work [inaudible segment] –

P: No, the only yard where I’m working.

I: So, how is it, to use the stickers at the work?

P: Uhm…it’s easy, just because even if I forgot they (work colleagues) have to remind me that “hey, have you taken your [laughing] nyaope (type of an illicit drug) today?”

I: [laughing]

P: We know that as men we speak, we just speak the way we want, right?

I: Uhm… when you were explaining to your workmates about TB, did you ever explain to them about the stickers?

P: Yes, I explained to them, so that those people that I live with could sometimes remind me, you could sometimes find out that after taking my treatment, just the way I was telling you that sometimes I had to drink them with the intention that after five minutes I would –

I: Yes

P: Yes, so suddenly you will hear one of my work colleagues saying to me “hey, did you take that nyaope (referring to TB medication) of yours, did you SMS now?” Oh, that’s when I remember that oh, I drank my medication, but I didn’t send [the message]. Then that’s when I would send.

I: Yes, using these stickers at work doesn’t interrupt your work, right?

P: No, they don’t interrupt anywhere, they don’t bother with anything, they don’t interfere anywhere. They don’t even take a minute, to SMS is just to enter that number only, the same address, you just go[laughing] there is no work here my brother. [laughing]

I: [laughing] uhm…before you tell the people that you are using these stickers, was there any person maybe who had seen you using them, then that person asks you about those stickers?

P: Uhm…most of the time it’s when we meet here at the clinic, we meet with those ones of the boxes, then he could ask me that how this one works for me, then I would tell him that it works good for me, and he could also tell me about his box that hey this one could ring continuously, even if I didn’t take the medication [inaudible segment], you’ll find us explaining to each other.

I: Yes

P: Yes, mostly I meet them here at the clinic, those ones of the boxes.

I: The people that you don’t meet with them at the clinic, I’m asking about…like those ones you are working together with, before you explained them about these stickers, did they ever see you using them?

P: Right, uhm…you will find that they say, I like to be on the Facebook, then I would say, “no, I’m not on Facebook,” mostly sometimes only you’ll find me busy SMSing, then they would say no, you are always on Facebook, to find out that I’m not on the phone for that thing or they might think that I’m on WhatsApp, no I’m not there – I’m SMSing this thing of mine, so that I could be out of it, do you get it?

I: Yes

P: Mm

I: So, how did you feel by the time you were explaining to the people that what I’m doing is not on Facebook, I’m not on WhatsApp, these are the stickers, I found them from the clinic?

P: No, it was not difficult, it’s just that my colleagues I and are full of jokes, do you get it my brother?

I: Yes

P: P: Yes, you found that we don’t take it seriously, we take it as a joke, joke. So, most of the things are not difficult for me.

I: Alright. Uhm…these stickers, are they placed in the medication or how do they place them because I don’t know?

P: Oh! They placed them at the packets, when you open that packet, uhm…that sticker is at the bottom, when I take out a pill, sometimes they could come out with those stickers indeed. So, in that way I know that oh, I’m only going to SMS this number until I’m done with packet.

I: Alright. Uhm…the people that you stay together with in the house, do they know that you have TB when we [inaudible segment]?

P: Yes, they know. They also came and tested.

I: Uhm…the people that you stay with except those ones at work, the ones that stay with only at your place, but not those ones – let me say like your friends, people that you sometimes chill with maybe on the street, maybe like when you go out with gents, those ones that you are not working with, do they know that you have TB?

P: Yes, right most of the time – I prefer being honest because sometimes I could affect you with that thing, so, mostly, I tell you “Hey my brother” – even the cigarette, I smoke a cigarette, so mostly I tell you [laughing] and you found others not smoking my cigarette

Cause he might think that hey this person would infect me with TB, I’m being honest, I don’t want to hide for anyone, do you get it?

I: Yes

P: Mm, I prefer being open so that everybody could be aware, I don’t want to do something that tomorrow when you do it to me, I won’t feel okay with it.

I: So, when you explain to people in this way, how do they react when you observe?

P: Uhm…some they react okay, some they don’t know but when you are a human being, the life is yours, mine comes first. What the people say, eish…no, that won’t work for you. Just look at your side and be out of many things. The only thing that I’m looking at, is that they are working for me only otherwise most of the things no.

I: Yes, uhm…you say your brother-in-law was the one who came up with this TB, right?

P: Yes, yes

I: Then he came and tested, right?

P: Yes

I: Without your brother-in-law, is there any other person that had TB in your family?

P: No, it was for the first time, I recognize it with my in-law, I was [inaudible segment] on the way, no.

I: Yes

P: I didn’t [inaudible segment]

I: Uhm…I’m requesting to ask you about your brother-in-law, he came at the clinic to take the treatment, right?

P: Yes

I: Did he proceed taking his treatment?

P: Yes

I: Did he finish?

P: No, he is still on treatment even now, they sometimes take him to hospital. He sometimes visits the hospital and the clinic for his treatment.

I: Alright. Uhm…Did it happen that maybe, you take out your sticker, then finding that may be one of the stickers fall?

P: No, stickers doesn’t fall easily.

I: Are they not falling easily?

P: Yes

I: Uhm…about your pills, where do you put them?

P: Uhm…Some of them I put them in the car, I live with them in the car just because I intake them around ten at work, right?

I: Around ten?

P: Mm

I: So, your child – you said you stay with a child and the wife, right?

P: Mm

I: How old is your child?

P: He’s eighteen years old.

I: Okay, I wanted to ask that if he was still young, maybe he could try to take your stickers and try to SMS, because he would like to know where do you SMS to everyday?

P: Okay

I: Yes. So, your medication – so, during the weekend when you are not going to work, where do you put them inside the house?

P: Inside the wardrobe

I: Inside the wardrobe?

P: Mm

I: Yes, I know that you explained but I’m requesting to ask you again that…since you started using these stickers, do you think that these stickers help you, so that you will finally reach the end of your treatment?

P: Yes, these stickers do help me, just because you know... With TB you know that most of the people don’t take it seriously. You find that people lapse, and you are not supposed to lapse with your TB. Six months is nothing, just take those six months, after six months you are done, the school is out.

I: Yes.

P: Mm.

I: Eh... I’d like to ask you, so you said that when you started to take your stickers and you went with them home, how did you feel when you received your first SMS reminding you not to forget about taking your treatment?

P: Uh... right, it was alright, just because my brother I was... after I took uhm... that thing - my medication, I would SMS, after the SMS -

I: Yes.

P: They thank me – they said thank you that [laughing]

I: [laughing]

P: I take my medication, myself. But can you thank someone for [laughing]

I: [laughing].

P: You can’t thank him, he is doing it for himself, he’s not doing it for you, even though that is the case, they were able to keep up – that “thank you, here you are - you were able” [laughing].

I: Yes.

P: Do you get it? To take – just to take something that is yours.

I: So, have you found yourself forgetting to drink... your pills?

P: No, I don’t remember my brother just because I am a pill person, I live with pills, so I don’t mind.

I: You don’t mind?

P: No, no, pills are pills, they are my life.

I: Uhm... Since you were saying that there was a time where the network was giving you problems, did you ever find yourself passing a day without taking your pills on time – where you struggled with network the whole day, and you realize that you couldn’t compose the SMS that day?

P: Yes, the days are there, because they would send me a message that I missed uhm... my daily what-what the previous day and you find that I didn’t really miss it, its just that the SMSs couldn’t go through due to network issues.

I: Please describe your emotions about the phones – about the calls that you receive even though you didn’t forget – when you forgot to drink like this, maybe like... you said that you rarely forget, right?

P: Yes.

I: Like when they didn’t receive the SMS, they are still receiving them, right?

P: Yes.

I: When they didn’t receive the SMS, they -

P: They tend to call me.

I: So how do you feel receiving calls from here at clinic telling you that they did not receive the SMS the previous day, when you have already taken the pills, how did you work about that?

P: Uhm... right, you said most of the times... brother to – to be honest, usually for me there is a person who has been working with me, you know they are darling. You know this person, still being a patient, when she calls, even when she approaches you, they are not rough, they approach you sweet. You can feel yourself that there is no way you can fight with such a person, there’s nothing you can say, you must just accept and say that “I’m sorry” uhm... when I have made a mistake, you see it, right?

I: Yes.

P: Yes, because she is sweet, when she always calls – that sister, you know. Even yesterday, when she asked that... right [laughing] that I should come to the clinic, she was sweet, she was humbled, I even agreed, saying I am indeed listening.

I: [laughing].

P: [laughing].

I: Yes, I heard you saying at first saying they were checking you at home.

P: Yes, not so long, they came, right. It was those times when the SMSs couldn’t go through – they came at home, I just find out when I went back at home that people from clinic were there. “What? People from the clinic?” on my date, I came here to find out what was going on, and they explained.

I: Oh, that they are not receiving your SMSs?

P: Yes.

I: So, when they were checking at home, hasn’t there been a time when they came for checking and they found you there, like during the weekend when not working?

P: Uh... right, they only came once, they didn’t come that much -

I: They found you?

P: It is that time when they couldn’t find me, it is that once, they only came that time, then they called me and with the phone call they got me.

I: Okay.

P: Mm.

I: So, since you started to use your stickers, how is your relationship with the sister from TB going?

P: Uh... you know, it is just that my brother, working with people is hard, some people are nice and when a person is nice to you, they are nice to you, you know? So, with me, everyone I have come across here has treated me good. It is easy with my treatment, I never had to stress because of it, I found a right person, who treated me well.

I: Yes.

P: Mm.

I: You do... something they call adherence calendar?

P: Yes.

I: It shows you the days you have been drinking on, the days you have missed, do they show you yours?

P: Yes.

I: Your calendar?

P: Yes, yes, yes, they once showed now that I remember.

I: Do they show you all your dates or there and there?

P: They showed me where I sent SMSs and where I did not.

I: Okay.

P: Mm.

I: Uhm... for the who person who uses the stickers, right?

P: Yes.

I: Do you think that it will become a problem for her if she might have no phone?

P: Yes, it will be a problem but the one of the boxes, I think it is alright, when you don’t have a phone, remember they are now two it’s the box and this one [inaudible segment].

I: Yes.

P: Mm.

I: Eh... This question most of the times, even us ourselves don’t know how to properly ask it. Eh... have you ever seen yourself maybe like, sometimes uhm... your culture prohibits from drinking the pills when sick, right?

P: [laughing] uh... not at all my brother, just because uhm... right, life my bro hey, the decision is yours. Everything you do, you do it for yourself, not someone a favour, so that is what I like, the very same thing, in life I live for some, not for anyone else.

I: Yes.

P: Unless my family otherwise anyone else, no, that would be difficult for me because you must – eh... in life, eighty percent is about me.

I: Yes.

P: Ten, eh... twenty percent, I can give it to someone else.

I: Alright, during the time they visited you at home right, people from the clinic came to check on you and did not find you, these people – from home, did they have any problem that “No, these people from clinic were here, we don’t know what they wanted, they wanted you”.

P: There was no problem, it’s just that [laughing] people I live with also know that I drink the pills.

I: Yes.

P: [inaudible segment] what happens when I didn’t SMS, things like that, you will find out they will be concerned as well, that “Why isn’t dad not SMSing?”

I: Uhm... I’d like to ask that uhm... I’d like you to explain how did the stickers help you, taking your pill – taking your TB treatment?

P: Uhm... They help me that my brother, most of the times when I didn’t SMS there, it is those times I know that I drank my pills, I make certain that I drank. There is no way I can SMS without drinking the pills [laughing] then I just SMS, you, see?

I: Yes.

P: Yes, there is no way I’d SMS, how can I SMS without drinking, I must drink, then after that I’ll be on the phone [laughing] I know that -

I: [laughing].

P: I’ll throw around two to three of those numbers, they go through and hey, I took my treatment.

I: Yes.

P: So, they keep me busy, just here, do you get it?

I: Yes.

P: After drinking my pills, there is something I must eat, do you get it?

I: Yes.

P: Mm.

I: Uhm... I’d like to ask, since you took the treatment, you said that you began with taking the stickers, right?

P: Yes.

I: Yes, uhm... and when you go to clinic, how many time do you come to the clinic in a month?

P: Uhm... when it started, I started coming... each month – each end of the month I come to clinic and then when it continues, they gave me two months – pills for two months. They started with one month, remember?

I: Yes.

P: Yes, and after then they will give me in a month, after the month they will give me again, I’d say three months, now it came to the ones for – for me to take them at least for two months [inaudible segment].

I: So now, how long have you been taking the treatment?

P: Uh... now it’s roughly uh... I’m finishing soon. uhm... maybe, let me say... what is this? Five months.

I: It is the fifth month?

P: Yes.

I: Uhm... these stickers of yours, uhm... is there something that you have noticed, that no... that they can do so that the stickers become easier to use or how do you see it?

P: Uh... I see them alright, just because they also explain, you SMS... those numbers, when you are done, when you are done you input those... the ones for the pills, you input them right through, when you input those numbers, for SMS, you put then once. So, uhm... right through, you input these ones... these ones you drink with, the sticker.

I: So, this way, the one on how the stickers look like, how do you see them?

P: They are alright, there is nothing I see wrong with them, because you can see that these are stickers, they are alright, they are written, SMS this number, so and so.

I: Uhm... my question is that the time you started to receive these SMSs that reminds you when you forget to drink the pills, do these SMS – by the time they started, did you like that they reminded you?

P: Yes, I loved it, just because uhm... right, even with the people who call you to ask why you couldn’t drink [laughing], uhm... there are not the kind of people to – to fight you, they come at you beautifully, that “Hey, what’s going on, we see you didn’t send an SMS, are you alright?” That’s where you see that hey this thing, they work, they are alright because sometimes I could even pretend to have forgotten, there are people who won’t forget me, they are watching me, you see?

I: Yes.

P: Yes, they always remind me that “hey, what’s going on?”.

I: So, when your SMSs don’t go through while you are drinking your pills, you find that the problem lies with the network, when they called you, how did you feel with the calls?

P: Uh... right, that is something I have been telling you at first that when these people call you, they don’t fight you, they just don’t. She is a right person, she approaches you well, when a person approaches you that well, there is no way you – you can fight or something, you also welcome and flow well with them.

I: Uhm... they always give you the... the – the sister you are in contact with, when she encourages you to take your pills, “We see that your TB is getting better, your body is gaining, we are seeing that you are getting better.” how would you feel, when a sister encourages you in that way?

P: My brother, I – I get excited, because I will also be seeing my body building up, do you get it?

I: Yes.

P: That at least I am gaining something, I didn’t lose something, I’m gaining [laughing] something, you see at least?

I: Yes.

P: You also be alright because most of the times go to her you want this thing – your body scale, to see your weight, as it stays there or building up, do you get it?

I: Yes.

P: Mm. She will be there checking you as well, when she sees you, she encourages you that “hey, your weight is not building up” [laughing]

I: [laughing].

P: Where else you will be working hard so that it be different next time.

I: Uhm... So now, the SMSs, when you are done with the SMSs, it says thank you for...

P: For taking your medication.

I: Yes. When that SMS pops in, how do you feel?

P: [laughing] You get happy because there is a person who even though you are taking these pills for yourself, they thank you.

I: Out of all the things, the SMSs you receive, those that remind you or those that thank you for drinking your pills, being called from the clinic, or every time when you take time to send SMSs, they come check on you at home. Uhm... among all of them, uhm... do you see them as things that helps a person who is taking TB treatment?

P: Yes, sometimes they – they make you to have that thing... strength do you get it? They give you strength things like that. They don’t make you lapse; you won’t lapse. When you live around people like that, those who support you with something that you only do for yourself, do you see that...

I: Yes.

P: It’s support, there is support at least.

I: Uhm... can I ask how you feel – what do you think we can sort in the SMSs you receive and phone calls they give you – when people from clinic check up on you?

P: Eish... brother, I see these SMSs to be alright because they help since you won’t forget, the phone calls they give you – they make effort. There is someone who sees that they can fight this thing but don’t know how to fight it, there is a need for people to be helped, do you see it?

I: Yes.

P: Yes, so... I see that to be a good thing a lot, it needs to continue, do you get it?

I: So, uhm... you – what do you think about these stickers?

P: I think that brother they should continue.

I: Yes.

P: Yes. Let them continue because they help a lot, do you get it?

I: Indeed.

P: There are some people who forget themselves, so these thing... I don’t think they can forget themselves because you see that at the end you will finish.

I: Yes.

P: So, I encourage others to take them, they keep you busy as well, they are not indifferent. It is something that really keeps you busy.

I: Just as you said that you sometimes encourage others that they should also take these things. If you can stay with someone from your family or your brother – uhm... your brother in-law there at home, as he comes to the clinic, does he take the stickers as well?

P: No, no – I’m not sure because... I’m just not sure. It seems like he doesn’t get them. He goes to clinic and goes to hospital just as I said. He might be going twice a month on his dates. He comes to the clinic, after then he goes to -

I: Yes.

P: the hospital.

I: So, he is using stickers on his treatment, right?

P: Mm.

I: He’s not using the box?

P: No, more like a sticker.

I: [coughing].

P: He is not using the box when I think.

I: Yes.

P: Mm.

I: If someone from your family or work come to you, would you brief him about the stickers?

P: Yes, just like my brother in-law, it’s just that I don’t really know but if it is possible, they should also give him these stickers.

I: Yes.

P: Yes, this can suit him, the thing is that I don’t know. Why he doesn’t have them. I’ll ask but the SMSs will really suit him, I see it right for him [laughing], yes.

I: so, your last words, you see this SMSs, being called, and being visited at home by the people from the clinic, so what do you think about that?

P: Uhm... right, I see them being alright, these people push you. It’s a... you know when you are a person you need a push a little. There are some things that when you are alone, you can’t be victorious of them, do you see my brother?

I: Yes.

P: You are a person by people, at least you have a little boost. You can have strength as well, do get it? Support is the best.

I: so, when you realize, uhm…these stickers or the box as you know that there is a box as well, do you think that they should be used for TB only?

P: No, they should not only used for TB my brother, there are some other diseases that they could use it for just because most of the people – but these nowadays, I think they should use it for most of the diseases because other people – the old people should always be reminded for some of the things. You’ll find out that he is an old person, and he is no longer capable of doing things.

I: Yes

P: Yes, to be reminded, at least, do you get it? There should be something that would push you, do you see that it could also be better in that way?

I: Alright

P: Mm. There are some of the parents, they stay alone, they… [inaudible segment], do you get it?

I: Yes

P: Yes, things like this could work for them.

I: Alright

P: Mm

I: Uhm…is there any other thing that I didn’t ask you before, that you would like to share?

P: [cough sigh] Eish, I have a flue my brother.

I: Yes

P: And it’s recovering, I feel it – It’s just that it needs me to slow it down, I should throw it away. Yes, right, my brother something that I would like to share with the other people is uhm…most of the time life is only yours, do you get it? If you don’t look after yourself, you are killing yourself because life is yourself. Everything is about you. Everything that you are doing in life, you are not doing someone a favor, you are doing it for yourself. It’s you there, it’s not me, do you get it?

I: Yes

P: Mm

I: Uhm…my brother, uhm…is just that the questions that I had for you have finished, right?

P: Okay

I: I would like to thank you for the time you have taken here with us.

P: Okay

I: For answering these questions, with the truth that you gave to us. Uhm…I would also like to ask you the last question before we leave. When you look at these stickers, uhm…when they proceed, who should explain them to the people that are infected with TB when they are coming to the clinic?

P: Uhm…right, I think the person who is welcoming us at the clinic, should be the one to explain about them just because when you enter it’s a welcome, right?

I: Yes

P: Yes, they should explain you about them.

I: So, do you think that maybe the people who have finished their TB treatment using these stickers could help other people when they come here at the clinic – like now you are taking chronic medication?

P: Yes

I: Do you think that maybe you could come here at the clinic explaining to other patients about the stickers?

P: Uhm…in this way, uhm…I could be able to do something like that because, uhm…as a person you should share something that at least helps you, you should be able to share them with other people, do you get it? -

I: Yes

P: If you noticed that it could help them, as you realized its importance towards you, do you get it?

I: Yes

P: Yes. Maybe you are trying to show some other people that this thing is important, do you get it? Not that you see something but not understanding it. Indeed, you won’t understand it for the first time as I didn’t understand it for the first time that hey, we take the pills then we SMS, hey, we’ll see. Uhm…but when time proceeds, I realized that this is the right thing, even if you forget to drink, they could remind you. Do you realize that there is somebody who is taking care of you that at least

I: Yes

P: most of your things should be done in the right way, you should not be out of a way, do you get it? There is support, do you get it?

I: Yes

P: Support is the best. There are some people who are living without any support my brother, so, things like these, they need them.

I: Yes

P: Support, do you get it.

I: Uhm…no, I thank you my brother.

P: Alright

I: Uhm…in this way, I…highlighted the number phones of the people that you could contact if you have some questions, right?

P: Mm

I: Even with this interview if you have some questions regarding to it, there is no problem, you could ask them on those numbers that –

P: Yes, yes, I’m going to sit down for it, so that I could see it clearly what’s going on, I understand you, do you get it?

I: Okay. Thank you, my brother, right?

P: No, I’m the one who is thankful.

I: Uhm…the time now is 11:18AM, our conversation is already ended.
