# Supplementary material for: Acceptability of Digital Adherence Technologies to support people with drug-susceptible TB in South Africa
Source: PLoS One. 2025 Sep 24;20(9):e0332103. doi: 10.1371/journal.pone.0332103 (PMC12459780; doi:10.1371/journal.pone.0332103)
Supplement: S4 File — (ZIP) [file pone.0332103.s004.zip › S4 Transcripts/PwTB/IDI 5_PwTB.docx]

**TRANSCRIPTION NOTATIONS**

| **Label Key** | **Meaning** |
| --- | --- |
| **I** | Start of each new utterance by the Interviewer |
| **P** | Start of each new utterance by the Participant |
| **N** | Note taker |
| **{ }** | Indicates that details were changed or pseudonyms were used to anonymise data |
| **( )** | Indicates the description provided to anonymise data |
| **XXX** | Words were omitted to anonymise data |
| **-** | Breaking into a sentence by the next speaker |
| **…** | Pause or drawn out words |
| **[ ]** | Indicates noise made, e.g. [laugh], [sigh], [pause] |
| ? | Beginning of utterance by unidentified speaker or questionable text |
| **[inaudible segment]** | Unclear section of the recording |

**I**: Do you agree that the interview can be audio recorded?

**P**: Yes, I do.

**I**: Ehh, date xxx (interview date), location xxx (clinic name) clinic, Language used Setswana, PID of the participant its xxxx, date… I mean time is 10:50am. Thank you.

**P**: Alright.

**I**: So [Inaudible Segment] …So, may I ask when did you start using the label or the sticker?

**P**: On the 6^th^.

**I**: Could you please explain, the 6^th^ of which month?

**P**: The 6^th^ of September-

**I**: -This year?-

**P**: -…2021, early 2021.

**I**: Last year?

**P**: Yes, last year.

**I**: In short, please explain how you felt when they told you that you have TB?

**P**: I did not take it in a bad way, I just took it well, I just told myself I will take my treatment and be well.

**I**: Okay-

P: - Mmm.

**I**: The day you started using the label is it the same day you started using your TB treatment?

**P**: No, they started me on treatment at the hospital first.

[Pause]

**I**: Hospital first... on which date? On the 6^th^?

**P**: Mmm… let me say on the 5^th^… around the 4^th^, yes, on the 3^rd^ or the 4^th^, somewhere there. And on the 6^th,^ it’s the time they told me to come to the clinic, I started coming to the clinic on the 6^th^.

**I**: Okay. So, you started taking treatment from on the 4^th^ until the 6^th^ without the label?

**P**: Yes, and I started with the label on the 6^th^. They gave me the label.

**I**: Okay… So how did you feel the time they told you about the label or the sticker?

**P**: *Ai* (no), I understood them well, even though at that time I did not understand very well.

**I**: Pardon…

**P**: At that time, I was still a bit confused, while asking and still confused

**I**: Okay. So, when they told you about the sticker you were not feeling well?

**P**: Yes, but I was alright… But I was not feeling well.

**I**:- Okay. So, you were not feeling well or those symptoms… so in short please explain to me what you mean when you say you were not feeling well, or the symptoms you were experiencing.

**P**: Uh, you know I would just be dizzy, actually it was not long being discharged, maybe it was the medication. It was my first time taking the medication, so I think it was the medication effect.

**I**: Okay.

**P**: Mmm.

[Pause]

**I**: So, what made you end up testing for TB?

**P**: Mmm [inaudible segment] I cannot remember, it was around August… but around the end of August. I was messed up and I went to the clinic. It was on a Sunday in August, but I cannot remember the date. They took me to xxx [hospital’s name] and they tested me there at xxx [hospital’s name].

**I**: Okay.

**P**: Mmm.

**I**: When you say messed up, what do you mean, you were messed up?

**P**: I was dizzy-dizziness. Just being dizzy. Not being messed up of one being crazy or anything.

**I**: So, the day they explained about the Sticker, who explained about the sticker?

**P**: It is xxx [ RA’s name].

**I**: Who is xxx [ RA’s name]?

**P**: She’s the one who gave me the sticker, I meet her in the TB department that side… She’s the one who explained.

[Pause]

**I**: Okay… So, when they explained about this sticker… may you please explain to me how this sticker works

**P**: What they told me is that after drinking my medication I should SMS-

**I**: -SMS what?

**P**: - SMS what is it called… 135, you understand?

**I**: -Okay**-**

**P**: -Mmm.

**I**: And then how did you feel about the fact that after drinking your medication you have to SMS?

**P**: You see, you will get bored when you start, I was getting bored in the beginning. I would say there is no use because I drank my medication, you see.

**I**: Okay.

**P**: Mmm

**I**: Did they explain the importance of sending the SMS?

**P**: Yes.

**I**: So, what did they say when they explained the importance of sending an SMS?

**P**: They said so that they can see that I do not forget to take my medication…. Then after… if I do not SMS for 2 days, they would call.

[Pause]

**I**: So, the time they were explaining to you about the sticker, how long did the process of explaining about the sticker take?

**P**: I would be lying to you there my brother, it is exactly where I was dizzy, however, it took a while. Because we even filled in that form there, they even encouraged me to fill it in because I was not okay. Mmm.

**I**: So, according to the way they explained to you about the sticker or the label, is there something that you feel you want to change that you feel they did not tell you about the sticker?

**P**: No, there is nothing I want to change.

**I**: Okay. So, the time we did the consenting process, I mentioned that during this interview we are going to need your experience, level of satisfaction and your feelings.

**P**:- Mmm**-**

**I**:- So, in short like please share your experience with the label?

**P**: Can I start with the label or the tablets? I do not understand there.

**I**: About the label **-**

**P**: -Mmm.

**I**:- Yes.

**P**: I’m again taking back my brother, this is the label and actually the label is for SMS only right, that is how I understand.

**I**: So, when I say your experience with the label I mean when you use the label, the challenges or difficulties of using the label, the importance of using the label and how the label simplifies your TB intake.

[Pause]

**P**: So, I should explain? I think it is okay because it reminds you [inaudible segment] that you should SMS, it does not mean the way I understand only. Not knowing if I am explaining correctly.

**I**: Okay. So, where you stay, who do you stay with?
**P**: I stay with the child, my aunt and brother.

**I**: [Pause] Okay. By the time they started you on TB medication, did the people from the clinic come to you?

**P**: At my place?

**I**: -Mmm.

**P**:- No, they did not come.

**I**: So, the time you started treatment, to make sure the people you leave with do not get TB, what did the facility do to make sure these people are safe?

**P**: The clinic gave me bottles so that they can test their saliva (sputum), they only gave them those. You see.

[Pause]

**I**: For those days, from the 4^th^ till the 6^th^, the time you took TB medication without the sticker, who made sure you are taking your medication?

**P**: It’s my aunt.

**I**: Okay. Could you explain to me in short how is TB treatment taken?

**P**: Alright. The way I personally take it, I only take it early in the morning.

**I**: When you saying early in the morning ,what do you mean?

**P**: I do not understand you clearly. Early in the morning around 09:00, after eating, after eating I drink the medication and I take it around 09:00.

**I**: Every day or you shift?

**P**: Everyday, the problem after drinking is that I forget to SMS, then I SMS late, but I take the medication at 09:00.

**I**: Okay. So, do you work?

**P**: No.

**I**: Peace jobs?

**P**: Yes. Such as plumbing.

**I**: So, when you got the peace job ,how do you take your TB medication?

**P**: Okay let’s say maybe I leave early. Before I leave, I drink it. Let me say I leave before 09:00, around 07:00 I drink the medication.

**I**: Okay. You drink you TB medication in the morning at 09:00?

**P**: Yes, at 09:00.

**I**: When you got a peace job you take your treatment at 07:00?

**P**: Yes, because I do not know what time will I come back there, I do not take my medication with me.

**I**: Mmm.

[Pause]

**I**: When you take your TB medication home for the first day, what was their reaction at home that you taking TB medication, how was it?

**P**: They did not have a problem; they were supporting me to take it.

**I**: Okay. And then their reaction when you use the label to take your TB medication, what did they say about this label?

**P**: Ehh… you know the first time I came here for my TB medication I was with my aunt, they explained to me when she was also there, she took it well.

**I**: Okay. So, when they started you on TB treatment at the hospital and when you came here and were given the label, the day they gave you the label, what were your worries regarding the label?

**P**: No, I did not have any worries. As long as I drink my medication [inaudible segment] I did not have a problem.

[Pause]

**I**: So, who knows that you taking TB medication?

**P**: At my place.

[Pause]

**I**: Besides at home, are there other people who know about your TB status?

**P**: No… do you mean friends and so on?

**I**: Yes.

**P**: No.

**I**: Okay. When you say friends, no, in short, could you explain to me your reason for not telling them you are on TB medication.

**P**: No, I cannot just tell people that I have this illness or I’m sick [laugh], the doctor knows my condition and myself.

**I**: So, in terms of support system, who and how many are supporting you on your journey of taking TB?

**P**: People at home, my aunt and those at home

**I**: Okay [Pause]. So, you mentioned that you taking your TB medication at 09:00 and at time you tend to forget to send the SMS-

**P**: -Yes-

**I**: -So, is there a day whereby you ended up sending the SMS two times?

**P**: No, I only send once. Unless I do not know, but no I just send one.

**I**: So, when you have forgotten to send the SMS you get…

**P**:- They send me a message saying that I should not forget to take my medication, then I send or at times I do not hear it.

**I**: So, you know how to get the SMS you just mentioned, which is the reminder SMS?

**P**: Yes. I got it today.

**I**: So, how often do you get it?

**P**: I get it every day.

**I**: Even when you have taken your medication?

**P**: Yes [Pause] even if the SMS… if I have taken, I get the thank you for what SMS, right? And then if I have forgotten I receive an SMS that says, “do not forget.”

**I**: So, this one that says, “do not forget to take your medication,” how often do you get it?

**P**: If I didn’t take it in the morning, they remind me.

**I**: But how many times did they send you.

**P**: When they see I did not take the things, when they see I did not take the SMS, you understand? If I did not SMS, they send me so that I do not forget to take my medication.

**I**: Mmm

**P**: Mmm

**I**: So, when you receive an SMS that says you should not forget to take your medication, how do you feel?

**P**: I would have taken my medication, just that I would have forgotten to SMS, you understand.

**I**: -But how do you feel when you get that SMS-

**P**: -Oh, ehh I see that actually these people care about us.

**I**: When you say care, please explain.

**P**: I see that they are taking care of us more than we look after ourselves, you understand? That is why they remind us.

**I**: So, to go to the next section, there is something we call a differentiated care model, which includes the SMS we just spoke about, phone calls and home visit. So, have you received a phone call that says you should not forget to drink your TB medication?

**P**: -Mmm I have received it-

**I**: -How many times?

**P**: -Just once [Inaudible segment] I used to always SMS, but when I think clearly, I think I haven’t received it, if I remember correctly, I used to SMS, unless they remind me to come to the clinic on a particular date.

**I**: The time you said once you received a phone call, how would you feel to get the phone call?

**P**: It was during the early stages, whereby I was lazy to SMS. Mmm. I would take my medication but would not SMS. Mmm.

**I**: So, you saying you were lazy to SMS?

**P**: Or I forget.

**I**: So, now you SMS?

**P**: Mmm.

**I**: What has changed?

**P**: During that time, it was the first time starting medication, I did not like them. Actually, when you start, you do not like them until you get used to them.

**I**: Okay. When you say you did not like them, what did they do to your body-

**P**: -No, my problem was the tablets, drinking the tablets, I do not like drinking tablets, you see, so when you are used to them you realize that it is actually an easy thing and then you drink.

**I**: Okay. So, earlier on you said before you started your TB medication you were a mess.

**P**: -Yes-

**I**: -You were sick?

**P**: -I was dizzy.

**I**: -You were dizzy-

**P**: -Mmm

**I**: -So now that you are on TB medication, how do you feel?

**P**: Now I am 100 percent. I feel great.

**P**: -Mmm.

**I**: So, in other words ,you would say TB medication helps you?

**P**: Yes, it helped me. Even though these pills… during my first stages I would lose appetite a bit, but I insisted [inaudible segment]

**I**: So, when you take TB medication or TB treatment did, they tell you about the side effects of TB medication?
**P**: Uh, no, I do not remember. No, they did not tell me.

**I**: There is a home visit, did they ever do a home visit at your place?

**P**:- Have I received it?

**I**: -Did they do a home visit at your place?

[Pause]

**P**: Home visit is when people visit you.

**I**: -When people from the clinic.

**P**: -Oh people from the clinic, no they have never visited me.

**I**: Okay. [Pause] So…

**P**: -They visit under which conditions?

**I**:- Usually when you do not take your TB medication. That’s when you get a home visit.

**P**: Okay.

**I**: So, when you use this label to take your TB medication, is there a barrier or something that distracts you from taking your TB medication?

**P**: No, there is not.

**I**: Okay. You said you say with your aunt, grandchild….

**P**:- No, my child and brother

**I**: -Okay. So, is there a day you forgot to send the SMS then you asked one of them to send it on your behalf?

**P**:- No, I send it myself.

**I**: -Okay.

[Pause]

**I**: So, when you use this label, is there a day you would say you struggled to send the SMS?

**P**: No, I have not struggled, just that I forgot. [Pause] You understand.

**I**: Because it seems like you were forgetting a lot.

**P**: Ehh! Do you know tablets?

[Pause]

**I**: What caused you to forget?

**P**: I am sure it was the tablets. [Laughs] there is nothing else, it was the tablets, they will make you forget.

**I**: Okay. [Pause]. So, by the time they gave you the sticker or the label, do you by any chance recognise this box?

**P**: No, this one I do not know, I only know these.

**I**: You just know the stickers?

**P**: Mmm.

**I**: We have two technologies, which are the labels or the stickers. So, the day they gave you the sticker didn’t they introduce this box to you?

**P**: Maybe they did my brother, like I told you I was confused, I did not see anything. But this one I do remember.

**I**: Okay.

**P**: Mmm

**I**: So, is there someone at home who has taken TB medication besides you?

**P**: No, there is no one.

[Pause]

**I**: So, this label or the sticker, did you happen to see them somewhere before except here at the clinic?

**P**: No, I never saw them.

**I**: Okay. So, your level of satisfaction, could you explain to me how satisfied are you with using this label to take your TB medication?

**P**: I am good, the way I see myself as good. It has treated me well.

**I**: So, when you take this medication or when you send the SMS did, they explain to you that you have taken your TB medication?

**P**: They see with the SMS, which is why they can send me that message, right. That says I should not forget to drink my medication.

**I**: Okay. [Pause]. There is this thing we call a platform whereby it takes out your adherence calendar when you take your medication.

**P**: -Okay.

**I**: So, has the sister or the intern launched your adherence calendar?

**P**: Okay, the one that- did he showed me?

**I**: -Yes.

**P**:- Yes, she showed me.

**I**: So how did you feel when they showed you your adherence calendar?

**P**: No problem [Pause] I just took it normal.

**I**: So, could you explain to me about that adherence calendar, that when you have taken your medication, what do you see?

**P**: Yes, when you did not take your medication, it shows red and when you taken it shows green. That’s how I see it. When you have not taken it shows red and when you have taken it shows green, if I remember correctly.

**I**: Okay. And then your adherence calendar, what did it show?

**P**: I do not understand these calendars, she showed me on that thing she was holding, it is the one that shows the red and the green, mine, she showed me that.

**I**: Okay.

**P**: Mmm.

**I**: So, as we have spoken about the SMS that you received more often, the phone call you received once and the home visit, your perception, or you think or your experience-

**P**: Mmm-

**I**: -Do you think these three things work?

**P**: Yes, they do work, they work so that they can remind you. They can remind you. These SMS’s work help a lot, you understand? There is no way you would not drink your medication; you would be doing so deliberately because these people remind you. You would be doing it deliberately.

**I**: Okay.

**P**: Mmm

**I**: So, for someone who is not taking medication, based on your experience, what would, between these three would help them to adhere to their medication?

**P**: The way I view it, I think individuals can help themselves, as long they agree to take their medication then the person would be helping themselves, that’s the way I take it. Because if I do not take my medication, I would be killing myself.

**I**: Mmm

**P**: -Just to leave these SMS’s and focus on me and look after me, a person cannot be changed, they can only be changed by themselves. You need to look after yourself, that is how I did it.

**I**: Mmm.

**P**: Mmm.

**I**: So, from your experience, the approach of sending SMSs to remind someone to take their TB medication does it work?
**P**: Yes, it works. It works because you do not forget.

**I**: So, when you say it works, in a nutshell or in short could you explain how it works?

**P**: You see… you know how it works… these people remind you; they send you a message. Even if they say you are not doing it deliberately, you see the message on your phone, there is no way you are not checking the message and make excuses for not taking your medication, you would be doing it deliberately.

[Pause]

**I**: Okay.

**P**: Mmm

[Pause]

**I**: So, [Pause] is there a day you forgot to take your TB medication on time?

**P**: No, I do not forget my medication.

[Pause]

**I**: Okay. So, when you take your TB medication, what would stop you or prevent you from using this label?

**P**: Stop me how?

[Pause]

**I**: At first you said you did not like to send the SMS?

**P**: No, I forgot.

**I**: Oh, you were forgetting to send the SMS.

**P**: Yes.

**I**: Or let me rephrase. What would be the thing that would stop you from taking your TB medication?

**P**: The only things is when I have healed, and they no longer give me at the clinic. Mmm.

**I**: So, did they explain to you the importance of completing the course of TB when you started TB treatment?

**P**: Yes. Yes, they did explain. But my brother you know they explained, but I cannot tell you what they said you see.

**I**: Mmm

**P**: Mmm, but they did explain that it is important to take my TB treatment so that I can heal, they did explain. That is why I told you that during that time I was a bit dizzy. That is why I was accompanied by my aunt, they explained to her.

**I**: Okay.

**P**: Mmm

**I**: So, to go back to the activities or the differentiated cared model, so you think this home visit approach would work for someone who is not taking their TB mediation?

**P**: Yes, but what would be the persons problem because the person would be doing themselves a favour not the people coming to them, that is the problem, why would they be sitting without coming.

**I**: Okay. So, from your advice, which would be the best way to approach these models, what could be improved in order to make sure that the patient takes their TB medication?

**P**: Uh … that thing… but exactly what does the box do? Because I know this one, they send you a message if you do not SMS, right?

**I**: -Mmm.

**P**:- That you should not forget to take your medication at that specific time, before mid-night, right? So, now I think this one of sending SMS is perfect. This one of fetching people at home is costly, the SMS is better.

**I**: Okay.

**P**: Mmm.

**I**: So, according to your answer, out of these three activities, which one do you think works the most and which one do you think is least effective?
**P**: The one that works according to me is the SMS one, right? Because they remind you every day. The one of visiting, sometimes the come and you are not available, who will remind you when they do not find you at home?

**I**: Mmm

**P**: You see, so the SMS and call are the one’s that are perfect.

**I**: So, when you say the SMS… let me rephrase like this. When you have not taken your TB medication, you receive a reminder SMS.

**P**: Yes.

**I**: Yes, I talk about those SMSs you receive when you have not taken your TB medication.

**P**: Mmm or when you have not sent an SMS.

**I**:- No, when you have not taken your TB medication you receive a message, when you have not taken your TB medication for two days you receive a phone call, when you have not taken your TB medication for a week you receive a home visit.

**P**: Let me ask, so when they decide to come and do a home visit and they do not find you, so you see the simplest is the call or SMS.

**I**: So, just to… for me to understand.

**P**: -Let me say this home visit one, what if you find me deceased? Now you would be causing the people quarrels and pain, or what [Laugh] that time you came thinking you are going to see your patient not knowing the person passed on.

**I**: Okay. So, with this SMS, what is beneficial about these SMSs, about receiving a reminder SMS?

**P**: I am taking on my behalf, for me they work my brother. They make you strong and see that the people actually care about me.

**I**: Okay.

**P**: Mmm.

**I**: So, here I need your level of satisfaction. When you say these people care about you, how satisfied are you to have people who are supportive in all direction or angles, like based on your experience?

**P**: To have supportive people is great, it is nice because even the medication you take it without any trouble, you do not even get stressed. You see when you start taking medication you realize that every illness needs supportive people, when you do not have support you may fail, you understand. Now receiving support from the clinic, you regain your strength, yes.

[Pause]

**P**: That is why I say people who are not taking their medication are doing so deliberately. You understand.

**I**: Mmm.

**P**: Yes.

**I**: So, when you look at this sticker or label for it to work better, what can we change so that it can be more effective?

**P**: I do not see any problem with it. I do not see any problem with this label. I view it as being perfect, the way I worked with it. Maybe for other people it has a problem, but as for me, I do not have a problem with it.

**I**: Okay.

**P**: Mmm.

**I**: Again, during this interview, your experience, your level of satisfaction, your perception even your thoughts based on what you think about this thing, so with this study of ASCENT that came with this box as well as this label, what could you say about this study regarding helping patients to take their TB medication?

**P**: Ehh... this is simple, the only thing is for people to follow the rules. Follow the rules they were given correctly. It is simple, that it what would help them, the person should start taking responsibility for themselves, and not others taking responsibility on their behalf. You understand.

**I**: Mmm.

**P**: Mmm. Its simple, that you should look after yourself first before others could take care of you.

**I**: Okay. So, when you look at this label and the way it works, do you think this label should have been implemented a long time ago to make sure TB patients take their TB treatment?

**P**: Oh, let me ask you as well. Back then this was not available, it is only implemented recently, it is my first-time taking TB medication, so how was it back in the days?

**I**: So, what we do now, we are conducting a study which will assist in making TB patients to adhere to their treatment. So, we come with ways which will make patients to adhere to their medication.

**P**: -Okay, I understand.

**I**: -So, I would not say why it was not there, but this is what we came up with to make sure TB patients take their medication.

**P**:- Oh, they were not there?

**I**: Yes.

**P**:- Okay.

**I**: -So, but from your experience, do you think these things should have been implemented a long time ago?

**P**: Yes, according to me. They should have implemented them, people would not die due to TB, the one who would die, would be doing deliberately because they would have received an SMS. Back in the days TB was the cause of some deaths, but I ask myself what made them forget to take their tablets or the medication was not available? I want to know.

**I**: So, for someone who has experience with using this label, since now we are conducting a study, do you see this label being implemented in the near future helping people?

**P**: Yes, I think it helps people. I saw with me that it helps, it helped me. It will help other people.

**I**: So, what is your advice to someone who is not taking their TB medication, if a person is not taking their TB medication, what advice would you give them so that they take their TB medication?
**P**: Advice that I can give is for them to take their medication , even though in the beginning it is difficult because even eating becomes a problem, at times these tablets… but it’s tablets they are like that if you are not used to them but as soon as you are used to them it becomes better. But one just need to take their medication, just force taking your medication, that’s my advice to people, to take their medication, even though at times it is difficult but they should force.

**I**: When you say it is difficult to take medication in the beginning, in short, please explain to me the difficulties.

**P**: You know it’s difficult in a sense that some are big and at times you have to take about 4 tablets… you are just faced with a lot of tablets, that is what makes one to be reluctant to take the medication.

[Pause]

**I**: Okay.

**P**: It bores [inaudible segment] until you are used to them.

**I**: So, as clinic or as a study, what can we do in order to make sure that the patient does not go through that difficult process?

**P**: You know what you can do to make sure the person is… is for the person to come to the clinic everyday for medication, that’s if they fail to take their medication. However, it is not possible, one would get tired. But they would be helping the individual.

**I**: There is something we call DOT, direct observed treatment, that you just mentioned now, that the person should come and take treatment an we see that person every day. So, from your perspective, this DOT is it the best approach in order to make sure that someone takes their medication?

**P**: Yes, I think that is the best way, if the person is failing on their own. In a month they would be used to it.

**I**: For someone who is working, how can this DOT approach be implemented for them?

**P**: No, the medication is not a problem, you can go with them to work or do something, if you forget them then leave them at work then you can drink them when you are at work.

**I**: Okay.

**P**: You understand.

**I**: So, to go back to our conversation you said when you get a part time job you drink your medication at home.

**P**:- I drink them were I usually do; I drink them in the morning.

**I**: Before you go to work?

**P**: Mmm.

**I**: Based on your observations, is there a problem with taking your TB medication in front of people?

**P**: No, I do not see a problem. The way I take them before I leave is because I cannot go with them because I would be carrying my tools and, on the other, had my medication, that I cannot do. [Pause] the medication stays there so that there is no confusion.

**I**: Okay. So, again… so, now we are wrapping up, we have reached the end.

**P**: Okay.

**I**:- So, we are just talking about things we have already discussed.

**P**: -At the beginning?

**I**: Yes. So, when you take your TB medication is there someone besides that of the family who saw you taking your TB medication?

**P**: People see it, but they do not know that it is TB medication, they just see the tablets and think I am just drinking treatment, not knowing what treatment it is .

**I**: But from your experience, is there someone who saw you taking your TB medication?

**P**: A lot of them.

**I**: When you say they are a lot…

**P**: -in the yard, some would be visiting, and they would see me drinking medication.

**I**: Besides…

**P**:- One cannot be going around saying today I am drinking TB medication or tomorrow I am drinking… you, see?

[Laugh]

**I**: So, besides at home, what is the reaction of others who see you taking medication?

**P**: They are fine; they know we leave with medication. We drink medication.

**I**: So, with your experience, could you say someone taking TB treatment is being discrimination out here?

**P**: They cannot discriminate you; they do not know that you are taking TB treatment the people who know are the ones home. They cannot discriminate. And there is no way one could be discriminated for having TB.

**I**: So, when you look at this technology, here I need your overall thoughts.

**P**: Mmm.

**I**: This sticker and the box, I know you have the sticker experience and not the box, but which one do you think is more effective and works best?

**P**: Is the sticker, but the way I view it, I think the box and the sticker works the same, because the box rings if you have not taken your medication. I think these two are the same.

**I**: Okay.

**P**: Mmm. But they are not the same, what if you are far from the box? You see. This one even if you are phone, you have your phone when they send you a message.

**I:** Okay.

**P**: Mmm.

**I**: So, now since you have been using the sticker what is your overall thoughts about the sticker?

**P**:- Mmm.

**I**: -What do you think about the sticker based on your experience?

**P**: I think the sticker is good, no need to change it, if you want to change going forward, I do not know what you are going to do because the way I see the sticker works well. The sticker, not knowing which future technology will be used , but I think this one is perfect.

**I**: Okay.

**P**: Mmm.

**I**: So, the company or organisation which came with the sticker, which is ASCENT, which is the name of the study, so, what are your thought on ASCENT which came with the study?

**P**: I think for them to do this I think they were trying to decrease the workload at the clinics, for them at the clinic to decrease the calls they make to their patients. They were decreasing the workload for nurses; they were helping with TB so that help comes both ways. In that way I think their thoughts were on the right track.

**I**: So, you can say what ASCENT came with is working?

**P**:- Yes, it is working, too much. For me it is working, I’m speaking for myself here.

**I**: Okay.

**P**: -Mmm.

**I**: So, according to the way the sticker or label was brought here, are there any gaps that you think this sticker does not cover in terms of adhering to TB medication?

**P**: No, I see the sticker as perfect, I do not see any gap, this sticker is helpful.

**I**: Okay. So, my final and last question. So, what are your thoughts about the differentiated cared model or what do you think about the SMS, phone call, and home visit? What do you think about having the care and support from these activities?

**P**: The SMS my brother is fine, I am saying it is right because [inaudible segment] you see now we live in times whereby it is not good, it might happen that you are held hostage and it appears that you are the one allocated to come check on me, you will also fall into the trouble I find myself in or they can even take your equipment. With the SMS we are safe, you understand.

**I**: Okay.

**P**: This thing of doing home visits it is not good. I do not prefer it.

**I**: Okay. So, what would you recommend to people taking TB medication?
**P**: for them to drink it my brother. They should drink their medication every day until they finish the course they told them at the clinic, they will become fine. I got into that my brother I was so slim when I started ,but now the medication helped me a lot I do not want to lie.

**I**: Okay.

**P**: Mmm.

**I**: Any final thoughts you have about the sticker or the label?

**P**: The only thought is that the sticker is perfect, continue to use it.

**I**: Okay. Thank you for making it here today and managed to do this interview. In short, we have reached the end of the interview. So, I thank you for coming and we appreciate what we just had.

**P**: Thank you.

**I**: Okay, Thank you.

**I**: Time ended is 11:50 am.
