# Supplementary material for: Acceptability of Digital Adherence Technologies to support people with drug-susceptible TB in South Africa
Source: PLoS One. 2025 Sep 24;20(9):e0332103. doi: 10.1371/journal.pone.0332103 (PMC12459780; doi:10.1371/journal.pone.0332103)
Supplement: S4 File — (ZIP) [file pone.0332103.s004.zip › S4 Transcripts/PwTB/IDI 6_PwTB.docx]

**TRANSCRIPTION NOTATIONS**

| **Label Key** | **Meaning** |
| --- | --- |
| **I** | Start of each new utterance by the Interviewer |
| **P** | Start of each new utterance by the Participant |
| **N** | Note taker |
| **{ }** | Indicates that details were changed or pseudonyms were used to anonymise data |
| **( )** | Indicates the description provided to anonymise data |
| **XXX** | Words were omitted to anonymise data |
| **-** | Breaking into a sentence by the next speaker |
| **…** | Pause or drawn out words |
| **[ ]** | Indicates noise made, e.g. [laugh], [sigh], [pause] |
| ? | Beginning of utterance by unidentified speaker or questionable text |
| **[inaudible segment]** | Unclear section of the recording |

I: Ehh, mama (mother) ehh, so you don’t have a problem with recording our interview?

P: No, I don’t have a problem, are you recording now? [cough]

I: The day the interview is done is xxxx (interview date), Uh the site we are in or the clinic which we conduct the interview in is [name of the clinic] Uh then the language we will use is IsiZulu- this interview will be done in isiZulu. Uh the P-PID of the patient is xxx and then the time at the right now is 11:50 ,uh we are about to start our interview, okay .Uh, mama (mother) first, I would like to ask where do you live?

P:I stay in XXX [patient’s residing area].

I: XXX [patient’s residing area].

P: But when I come to take my treatment, I sleep here at my Aunt’s place.

I: Yes.

P: Because am trying to avoid the long queue.

I:Okay because-you are saying that it is not easy to come straight from your home to here at the clinic?

P: Coming here- I will be late, yes.

I: Okay, so you decided that it is better to come and sleep this side so that you can be early for queuing.

P: Yes, it is near.

I:Okay, at home were you stay ,who do stay with?

P: I stay with my children .

I: Okay, how many children?

P:My three children

I: Are they schooling?

P:They are not schooling ,the younger one is schooling

I: Oh, so others are done with school?

P:Yes.

I: Okay, so mama (mother) when you come here- you said-let me go back. You said you sleep this side when you come to the clinic?

P:Yes, I come from XXX [patient’s residing area] and sleep at my aunt’s place.

I: Okay, so you mean when you slept this side, you do not take a taxi to come here at clinic?

P: I take a taxi when I come to sleep at my Aunt’s and then when I come from my place to the clinic, I do not take a taxi.

I: So, you can walk the distance to here?

P:Yes, but there is a local taxi which cost R10, you can walk if you don’t have it, but when I come from XXX [patient’s residing area], I pay a taxi.

I: Taxis?

P: Yes, I take two taxis to come here.

I:How much is the tax- the local taxi?

P: A taxi from here or XXX [Name of the place which the patient residence]?

I:A taxi from XXX [ patient’s residing area] to here.

P: 23 Rands.

I: And then how much is the taxi you take from your Aunt’s to the clinic.

P: 10 Rands.

I: Okay.

P: [Clearing a throat].

I:Okay, and then mama (mother) do you still remember when did you find out that uh, you were affected by TB?

P:The date?

I: Uh yes- the year or the month.

P:Last year I was sick, on the xxxx (date) I was admitted at the hospital that’s where I found out that I had TB cause they did some tests and after the results came back saying, I am positive. So, I started taking treatment at the clinic, but that was after I came from the hospital.

I:Okay, you were sick and you were admitted and that’s how you found out that you have-

P: I have TB-

I: You have TB, but you did not know that you have TB?

P:I didn’t know but coughing right through.

I: So, the symptoms you had was coughing?

P: Yes.

I:But did you know that as you were coughing it could be TB, how long did you cough?

P:Uh, about 2 months, but when I was coughing, I was not coughing out blood .

I: So, you were only coughing?

P: Yes, and this thigh.

I:Yes ,and your back?

P: The spinal cord, yes.

I: It was painful?

P:Yes.

I:So, what can mama (mother)- did you understand, or did you know TB symptoms?

P:Yes, I knew that heating when you are sleeping [sweating] ,loss of appetite and tiredness when I am like that it could be TB or something. Yes, losing appetite ,not eating, tiredness and body heating up.

I:Mmm but the time you were coughing and having back pains, you didn’t think that it could be the symptoms of TB?

P: Yes, I didn’t know.

I: As you explained to me that you understand that sweating at night ,losing weight and coughing are the symptoms, you knew?

P: And losing appetite.

I:Mmm losing appetite, okay, and when you had these symptoms you didn’t know that you had TB?

P:Yes.

I:So, the – mama (mother) you said you started last year?

P:Yes.

I:Since you found out that you had TB ,did the children you stay with came to the clinic and tested for TB ?

P: Yes, they came and screened for TB.

I: Where did they test?

P: Here at this clinic

I: What were the results?

P: The results came back negative meaning they do not have TB.

I: And then at home, is there anyone who had TB before?

P:There is my sister XXX [ patient sister’s name] had TB, yes.

I:Okay, do you remember how she found out that she had TB?

P:My younger sister?

I:Yes.

P: Eish I don’t remember, you know .

I: Yes.

P:It has been a long time.

I:It has been a long time, okay.

P:She had a TB called MDR

I:Oh, MDR, okay. So, mama when did you started using stickers?

P:Uh, I started using stickers on my 2^nd^ month of taking treatment, I started when I was at the hospital and they transferred me to the clinic. So, I got the stickers ,XXX [intern’s name] gave me stickers.

I:Mama you are saying that you started using stickers on your second month.

P:To use the stickers ,yes.

I:After you have started taking your treatment and you were diagnosed and started your treatment.

P: I started taking my treatment and they gave me stickers, I started using stickers when I take my treatment. I use these stickers to SMS, yes.

I:And then mama (mother) when-the stickers. What can I say? Can you please explain- you said XXX [intern’s name] is the one who gave you stickers?

P:Yes.

I: And she explained to you how they-

P:Yes, how they work-

I:How do the stickers work or how do you use them. So, I want to know what you understood on XXX [intern’s name] explanation , just what you understood on how to use the stickers.

P:To me the stickers were best because I would wake up at 6 am and drink my treatment, then SMS . XXX [intern’s name] explained to me that stickers are important because when you take your treatment they clock even if you did not take your medication, they will clock. When you took your medication, they will appear in green colour and when you did not take your medication, they will appear in red colour. So ,when I am done taking my pills, I SMS the number I found in my sticker and then I will get a message which says, “thank you for taking your medication,” mmm.

I: Mama(mother) you said- let me take you a little back. You started treatment and fewer day after, you got your stickers .

P:Mmm.

I:So, the time before you came here at the clinic and XXX [intern’s name] gave you the stickers uh, is there a difference or a change in the way you take your treatment. Did you notice any difference between the time you were not using the stickers and when you were using the stickers?

P:Yes, there is a difference. The stickers were important to me because they were….they were able to remind me that today I took my pills or I didn’t take the pills ,the sticker opened my mind to take my medication, seriously, yes.

I:Okay, before you used the stickers as you explained to me-since you are using the stickers at first you told me that XXX [intern’s name] explained to you that after taking your pills you must SMS and it clocks to her showing that you did take your medication. Showing in green colour when you sent the SMS and when you did not send the SMS, It will appear red .

P:Yes.

I: Okay, so um mama (mother)- okay, as you were explaining to me that the stickers helped you to remember that there may be something you are forgetting or should do,you understand?

P:Yes.

I:So, before you used the stickers, is there a time you forget to take your pills or something like that or you were able to remember to take your pills on time?

P:Yes, that at 6am I have to wake up and take my pill.

I:Okay, so you were able to take your pills on time even before you used the stickers?

P:Yes, I was managing, but the stickers were important to me, I do not want to lie.To me they were really helping [LAUGH].

I: Uh, they helped?

P: [CLEARING THROAT]Yes, too much .

I: Even though you were managing?

P: Yes.

I: Getting the stickers helped to be easier for you to take your pills.

P: Yes, the stickers gave me the-the strength to take my pills, you see. The stickers, yes, to me, I don’t know but the stickers made me to develop the strength that I have- I have to drink my pills and SMS because if I did not drink, I don’t get the SMS which says, “thank you for taking medication,” they were important to me.

I: Oh, so you received a message after you have sent the SMS?

P:Yes, I get an SMS that say, “thank you for taking your medication.”

I: Okay, [chuckling].

P:Yes, I SMS and there is an SMS which comes back, you see, I don’t know how to explain to you, but this SMS was important to me.

I: Yes, I was about to ask that how does this SMS made you feel?

P: This SMS gave me strength, it gave me strength to drink with without missing a dose, yes.

I:Mmm.

P:Yes.

I:Uh, yes mama that good, that means the SMSs motivated you?

P: Yes.

I: Is what you are actually saying?

P:Yes, that what I was trying to say.

I:Okay.

P:Yes.

I:Okay, mama ,I hear you.

P:And when I sent the SMS and I do not get the one that say, “thank you” I would panic thinking that the SMS did reach it destiny, but sometimes it is network problem, yes. When I sent the SMS and get the thanking SMS, I felt happy because it means that they saw that I took my medication, yes.

I:Mmm.

P:To me that was important, serious [clearing throat].

I:Uh ,mama as you are saying that sometimes you send the SMS, but you thought the SMS was not sent because of the network?

P:Yes, but later the SMS with “thank you” will come through and I would be happy.

I:So, the network problem how long was it taking?

P:To send?

I: You said that sometimes you send an SMS and then don’t get the responding SMS which says, “thank you” because of network. Is it what you said?

P:Yes, it usually take 3 to 5 minutes, but I do get it, you know that phones have a problems.

I: Oh, you do get it as the day goes on, but not the same time you sent the SMS, is that’s what you are saying.

P: Yes after 2 minutes, yes or I was the one rushing for response .[laugh] [clearing throat]

I:Is there a time you never got the response message?

P: No, that never happened, when I sent SMS and never got the SMS ?

I: Yes.

P: No, that never happened.

I:Okay, so when you SMS you get the response back even if it is after a while, you get it.

P:Yes ,I will get the SMS.

I: Yes.

P: Which say, “thank you for taking your medication,” yes.

I:Okay, and then the stickers - who told you about the stickers ?

P: XXX [intern’s name] is the one who told me about the sticker .

I:Oh, XXX [intern’s name] is the one who told you. And then what’s XXX [intern name] role here at the clinic, is she a nurse?

P: What she does?

I:Uh, yes, what’s is her actual job here at the clinic?

P:Oh ,the first time I saw her at the TB room I told myself that this person- what can I say-that she motivates people about TB and to take their medication so that they can be cured even-at the time she was giving me the stickers she told me to take stickers and showed me how to use them when I finish drinking my medication and how to SMS the number on the stickers. She also [clearing throat] saved the number I must SMS to on my cell phone and that is the end, yes.

I:Mmm.

P: I told myself that she teaches about TB and how to use the stickers, with her, we were always talking about the stickers and it importance, yes.

I:Okay, it means that she explained it clear to you.

P:Yes.

I:Okay, but as she is working with the stickers, did she tell you about what is her actual job here at the clinic. Do you understand what is her job as I am told you that here at the clinic we have the sisters (nurses), the vaccine sisters and the data captures. Did she tell you the title of her job at xxx (organisation name), did you understand her job?

P:No, I understood that she works with people with TB.

I:Mmm.

P: Yes.

I: Okay, mama, the time she was explaining-as she gave you the stickers, do you remember how long she took to explain to you on how to use the stickers. Did she take 10 minutes,20 minutes, an hour, or 2 hours?

P: [clearing throat] we were siting as it was my first time, she was patient with me and I couldn’t hold my cell phone, so she was like you can give me the cell phone and I will do it for you mama showing me how to send the SMS- the code on the stickers and you will get another SMS from them after you sent the code, yes. So, we took about twenty -when I said I did not understand what is this code for, she explained until I understood this sticker thing and she was so patient with me ,yes.

I: Okay, you are saying how long was the explanation?

P: About 25 minutes, she was talking [cough] when I said I do not understand she would go back and explain to me again, telling me more about the stickers and until I fully understood.

I:Okay, I understand. You are explaining that she gave you more time to make sure you understood the sticker process and gave you a chance to ask were you do not understand to a point you fully understood.

P: Yes, until I understood how I must use the stickers.

I:Okay, so you are explaining the way she explained to you. So, was it easy to understand the explanation?

P: Yes, it was very easy to understand, and it was easy to use the stickers with the codes.

I:So, you saying you fully understood everything she explained to you. So I want to know that you are satisfied and that must it be people like XXX [intern name] who tell people about the stickers, or you suggest it must be someone else?

P: XXX [intern name] is the perfect person to tell people about the stickers.

I:Okay, mama, before they gave you the stickers here at the clinic, have you ever saw a anyone who use these stickers, or you first saw them here at the clinic?

P: It was my first time hearing about the stickers here at the clinic, I never heard about them.

I: So, mama to use-I know you explained a little about you feel about the stickers and how helpful the stickers are to you. I would like you to tell me about your experience of using these stickers.

P:Um since I started to using them-what can I say, the stickers are fine to me, I don’t know how can I explain it (cough).

I: Mmm.

P:Yes.

I: Is it easy for you to use the stickers or you had some challenges while using them.

P:I did not face any challenges ,to me it was easy to use the stickers, they really helped me.

I; You didn’t have any challenges?

P: No, I didn’t have any challenges.

I:Okay, [FLIPPING PAGES] okay, so XXX [intern name] gave you the stickers and you went home ,when you were home. Did you have second thoughts like Eish these stickers I will regret taking these sticker ,these- didn’t thoughts like that cross your mind?

P: Did I regret taking the stickers.

I:Sorry mama, you know that sometimes when you take a decision and later regret for taking that decision. In case of the sticker- you already took the stickers home ,so didn’t you regret taking them?

P:No, I didn’t, I was so happy about the stickers which I used ,I was really happy about my stickers .

I:cAt home you said you live with your children ,did you tell them about the stickers?

P:Yes, because when I was starting my TB treatment ,TB treatment is painful, so I used to ask my second born child to come help me SMS and I showed her where XXX [intern name] said I must click to SMS the code. They were the ones who helped me as they are educated more than me.

I:So, your children are the one who understood better about the stickers ?

P: Yes.

I:So, that means you told them you take-that you found out that you have TB and that you take TB treatment?

P: Did I tell them?

I: Yes .

P: Yes, they know that I have TB, that mama (mother) take TB treatment and she use stickers to send the number and get a message wish says, “thank you for taking your medication” [laugh].

I:[laugh]

P: Yeah, that made me happy, yes.

I:Mmm.

P: I used to tell them that this shows that I did drink my pills, yes, I loved this so much and it gave me strength to take my medication. I love these stickers, they made me strong and to take my medication without missing any dose .They also kill that doubt of not taking your dose for the day, I would freak sometimes when I remember that today I didn’t SMS and it didn’t clock that I took my medication to XXX [intern name] and then when I SMS I have to drink and then SMS. It will clock that I did take my pills today. I loved that.

I:Okay, and then uh, mama (mother) when you were telling them that my children I found out that I have TB ,I take TB treatment and I use stickers which I got from the clinic as you explained to me how you were told to use them. At that time when you were telling your children about your situation, how did you feel ?-that you were affected by TB ,you take TB medication, and you use stickers. How did you feel about telling your children about your health issue at that moment?

P:Um, I was okay, and they accepted that mama take TB treatment and use the stickers to SMS the code ,yes

I:Uh they accepted, and they didn’t have a problem?

P: Yes, they didn’t have any problem. They also accepted that I take TB treatment and she will be cured.

I: So when you were using stickers, were you working?

P: I am not working .

I: Okay, you were not working.

P: I totally don’t work.

I:Okay.

P:Mmm(yes).

I:Okay, [FLIPPING PAGES] so as you explained that the stickers work after you took your pills and then send the code, has it ever happened that you send/sent an SMS more than once a day?

P:I didn’t send?

I: I am saying that have you ever sent the SMS twice in a day?

P:Oh , I send it once.

I: Okay.

P: Yes.

I: You never made a mistake and send the SMS twice or something?

P: No, I never did ,I use the sticker once a day .

I:Is this how XXX [intern name] explained to you how to use the sticker.

P: Yes ,today is Tuesday, right?

I: Yes.

P: I drink the pills and when I am done ,I SMS.

I: Okay.

P:Yes.

I:Okay, okay and then your stickers-where did you put them? In your bedroom, dining room or at the kitchen?

P:In my bedroom.

I:Okay, you put them in your bedroom.

P:Yes.

I:Okay, so why did you decide to put your stickers in your bedroom ?

P: For them to be safe.

I: You thought that if you put them at kitchen they won’t be safe .

P: Yes, maybe they would throw them away and I will end up not having nothing to SMS.

I:] okay, I understand mama. So, there is what they call differentiated care model on this technology or on the sticker you use. I will explain the differentiated care model as the calls you will get- as you explained that if it didn’t clock to XXX [intern name] that you took your pills ,XXX [intern name] explained that-let’s say it has been 2 or 3 days the message didn’t clock to her ,did she tell you what will happen in this case if you missed your dose or you didn’t send the messages on these days? Did she tell you what will happen or what is going to happen?

P: Yes, when it does not clock?

I:Yes, when it didn’t clock to her?

P: It means that I did not SMS.

I:Okay.

P:I didn’t send the code.

I:Okay, mmm.

P:Yes.

I: And then did XXX [intern name] explain to you what would happen if they came across that situation, the follow ups and so on?

P:They will call asking why you didn’t send the SMS and it means you don’t take your treatment .

I: Okay, that would happen.

P:Yes.

I: As I was explaining about the differentiated care model is when you will get

P:It is okay ,do not open.

I: When you get a call saying we saw that you missed 2-3 doses that differentiated care model or you will get messages which remind your about taking your medication and there is another thing which is also part of differentiated care model which is a home visit to come check up on you and ask what is your problem or the reason you missed so many days of taking your treatment .

P:Mmm

I:So, [FLIPPING PAGES] which one did you receive since you use the stickers, a phone call or?

P:No, I never received any of those.

I:You never got-

P: I never made a mistake .[LAUGH]

I:[LAUGH]Yes.

P:Yes.

I: So, it means you never received a call because you send the SMS every day , is that what you are saying?

P:I never did.

I: So, the message that says, “thank you for taking your medication” you get it.

P:Yes

I:So ,this SMS mama-how did it made you feel. How do you feel when you get the SMS which says “don’t forget to take.”

P:[COUGH]I felt I would- this SMS reminded me that don’t forget ,it is like the one saying thank you

I:[LAUGH]

P: Which thanked me for drinking my pills, seriously I loved those.

I:Mmm.

P:Yes

I:[FLIPPING PAGES ]So, you mean that you didn’t have a problem about this SMS?

P:Yes, this SMS is like we are talking face to face.

I:Okay.

P:Mmm.

I:And mama as you are using the stickers-as people we, have beliefs which states which things we must do and not do like church or other cultural beliefs and other churches have rules like if you have tattoos or you are not allowed to do certain things when you are joining the church. So, since you are using the stickers, is there a thing which may be a barrier, culturally or spiritually which might make it difficult to use the stickers?

P: I believe in church, but it didn’t make it difficult to use the stickers as they don’t use traditional medicine.

I:Okay, so as you say it was never difficult for you to use the stickers because of your church, have you ever used the stickers at church ?

P:No, I use the stickers only at home.

I:Okay (….) okay (…) so mama as you explained to me about telling your children that mama found out that she has TB, she is taking TB treatment and she also use stickers to help her with taking the treatment and they understood. Is there someone else you told about you having TB besides the ones you live with, a friend or a family member?

P:Yes, I had a friend which I used to share my treatment journey with and encouraging her to also use the stickers so that we can be cured and live a long life, she died as she was not following-the rules yes. Though I had a power that I will- I will fight for my life, I fight for my life, yes and I take my pills with a happy heart and not with a broken soul telling myself that I will take my pills and these stickers will help me to have strength to continue to take my pills because that message which says “thank you” gave me strength.

I:[LAUGH]

P:Seriously, I would drink my pills then send the SMS and get the thank you message ,I will tell myself that they saw that I did take my treatment, yes, this gave me strength more than the watch would have.

I:[LAUGH]

P:[LAUGH]

I:So, you were also using the watch?

P:No, the sticker SMS is more important, more the watch to me. I don’t know about others.

I:Okay, you said you had a friend who was also taking the medication, but unfortunately she passed away. So, who found out first that they have TB?

P: My friend.

I:Okay, so you found out after her that you have-

P: Yes, I found out after her.

I:Okay.

P:I told her that I tested positive for TB, and she said “I also did” and we spoked about it and I said friend let’s take the treatment we will see life, so she didn’t take it serious, but I told myself I will fight for my life and I also fight to live for my kids. Taking treatment is nice and it is not a death sentence.

I:Mmm.

P:I told her about the stickers that you see these stickers will help you to drink, then you SMS [cell phone ringing], you will receive a message which thank you .

I:It is sad that you lost your friend.

P: When we were on the same journey eish, but some people (…) [CELL PHONE VIBRATING]

I:So, mama on your perspective, is there a thing you would like us to improve about the stickers?

P: Can you please repeat the question for me.

I:Is there anything you think we must change about the stickers or add to the stickers?

P:No, do not change anything, just continue giving away/giving us the stickers and do not change anything so that we can continue to SMS .

I:Okay, mama when they told you that you have TB- what’s your experience about being counselled? Are you satisfied about the way the told you that you have TB?

P:Yes .

I: You were happy about the way they counselled you?

P:Yes, they told me to take my treatment and they gave me 6 months and that’s a short time, you don’t take the treatment until you die. I was slim that time and they told me I will gain weight and I must not stop to take my medication and I saw the changes. The person who counselled me told me to take the medication and I will be fine.

I:Is there anything that didn’t sit well with you when you were counselled ?

P: I loved the way she spoked to me and the way she motivated me .

I:Okay, okay (….)[flapling pages] uh, we give you stickers ,we call you and home visit you. We are trying to provide external support .We are coming into an end-these things I have mentioned do you think they are helping, or you don’t see a need of these things?

P:These are good things because it help people to take their medication serious and these stickers are fine to me.

I: Uh, so you don’t see a problem with a person receiving a call.

P: I really loved these stickers

I:Okay, (….) uh, mama we have come to an end of our interview. Thank you for taking your time and for coming here and hear us out, thank you very much mama. the time this session ended is 12:40-12:40 pm the time we finished our interviews -12:40 pm.
