# Supplementary material for: Acceptability of Digital Adherence Technologies to support people with drug-susceptible TB in South Africa
Source: PLoS One. 2025 Sep 24;20(9):e0332103. doi: 10.1371/journal.pone.0332103 (PMC12459780; doi:10.1371/journal.pone.0332103)
Supplement: S4 File — (ZIP) [file pone.0332103.s004.zip › S4 Transcripts/PwTB/IDI 7_PwTB.docx]

**TRANSCRIPTION NOTATIONS**

| **Label Key** | **Meaning** |
| --- | --- |
| **I** | Start of each new utterance by the Interviewer |
| **P** | Start of each new utterance by the Participant |
| **N** | Note taker |
| **{ }** | Indicates that details were changed or pseudonyms were used to anonymise data |
| **( )** | Indicates the description provided to anonymise data |
| **XXX** | Words were omitted to anonymise data |
| **-** | Breaking into a sentence by the next speaker |
| **…** | Pause or drawn out words |
| **[ ]** | Indicates noise made, e.g. [laugh], [sigh], [pause] |
| ? | Beginning of utterance by unidentified speaker or questionable text |
| **[inaudible segment]** | Unclear section of the recording |

I: Uh, Sir do you agree for us to record this interview?

P: Yes, I really do agree.

I: Ok thank you.

I: Mmm today’s date is the xxxx (interview date) uh, location, uh we are here at uh xxxx Clinic. Uh and the language that will be used for this interview is English. Uh the PID of a patient uh is xxx and the time at which this session start is uh…13:05 PM.

I: Ok mmm, if I may ask Sir, where do you live?

P: I live here in [inaudible segment, 00:59] right here in XXXXX area name.

I: Ok. So how far is it from the clinic?

P: It is not that far, it’s about a kilo.

I: It’s about a kilo?

P: Yes, this location this side.

I: Ok, so it’s walking distance?

I: Yes, it’s walking distance.

I: Ok, so-

P: But sometimes I take a taxi when it’s raining.

I: Oh yes.

P: Yes, it a distance but…the one that I should have been using from that side, they are too slow.

I: Oh ok, there is a clinic from that side, but you come here because of the service that side?

P: The service that side is not good, it not right for me to attend there.

I: Ok I understand. Ok so, you usually walk but sometimes because of weather, maybe it is raining then you take a taxi?

P: If I am not too tired to walk, I take a taxi but if I am tired, I take a taxi.

I: Ok. Then how many taxis do you take if it happens that you might need to take a taxi?

P: No, it only one.

I: Just one. And how much is the taxi?

P: Mmm… R20,00

I: Ok. And then who do you stay with at home? Children, wife?

P: My fiancée.

I: Oh ok, so you are married?

P: No. Fiancée, were not married.

I: Oh fiancée [laugh]that means you are engaged to this person [laugh]?

P: Yes, I have paid *Lobolo*. We don’t call it marriage.

I: Oh yes [laugh]you know for some people one is referred to as a wife once the lobolas have been paid [laugh].

P: You see [laugh]

I: [laugh]Ok that is nice, so it only the fiancée? Ok

P: Kids are grown up; they stay there in their own houses.

I: Ok. I see.

P: Even the grandchildren don’t stay with us, we are only two. Mmm [laugh].

I: Mmm ok, I see [laugh].

P: Yes [laugh] they only come to visit when the schools are closed.

I: Ok. Mmm when were you diagnosed with TB? If not the exact month or a day? Do you still remember when were you diagnosed?

P: No, it was last year. Truly speaking I was never given my results.

I: … How?

P: I can’t say. I was told to spit on a container, and I was given a date to which to return to clinic. On that given date, I was then told to visit xxxx Hospital and take an X-ray. Mmm and I went there to take an X- ray, and no one explained what was going on with me. Again, I went back to take another X-ray Uh, then I was diagnosed. No one explained what was happening…that maybe you got this TB. No, no, no

I: So right after you gave them-

P: Then they gave me medication.

I: Ok. And then the counselling was it done?

P: No, no, no

I: So, that means the counselling was not done?

P: No, no, no. I was just checked and then from there I was given medication.

I: So, you came-

P: So, xxx (Interns name)gave me stickers.

I: Ok.

P: Uh- huh, sometimes you trust people to do their jobs properly and you can’t blame them. I don’t know who to blame because they asked for an X-ray, and I gave it to them. So, I expected them, including the doctor to tell me to what was happening. Uh so it was never explained to me what is going with you, mmm, on the following month, I was then told to visit xxx Hospital for another X-ray but when I come back with those Envelopes, again, no one ever explained to me what was going on. They {nurses/sister} just gave me medication, that is all I know.

I: Uh, Yoh I can’t even begin to imagine what you went through; no one should ever go through that, mmm it sad what you just told me. Mmm if I may ask sir, how did you feel about everything? …Not even offered counselling.

P: Uh you know you can’t question a professional because they do have many explanations to you…they know their job; they are doing their job. So, it better to shut up and let them do their job. Just follow the procedure only. If they say sit down, you sit down. When you ask them about the results, mmm they just write medication, that it.

I: Was it here at xxx (clinic name)?

P: No, right here.

I: Mmm…

P: Mmm, so I was never given my results for TB, I was just given medication I was given medication to drink for six months.

I: Mmm…

P: Mmm yes.

I: So, even when everything went that way you took your medication?

P: Yes, even when everything went that way, I still took my medication.

I: Uh so you said even after everything that has happened you will still take your medication, counselling or not you will take your medication?

I: I just said to myself; they know what they are doing…mmm better not argue, better not ask them questions. Let me just take this medication for six months.

I: … I see, mmm…so do you still, mmm remember when did you start using the labels?

P: It was last year, mmm let us count like this [patient counting]

P: It was November.

I: It was November you say?

P: No, I took them for seven months. Yes, it was October, mmm it was October.

I: Uh who did you say explained and gave you the stickers?

P: Uh xxx (Interns name)

I: Oh ok, its xxx (Interns name)

P: She explained to me and gave me the stickers; she told me that my phone will notify me to take my medication. xxx (Interns name) also explained that I will receive notification messages which are free of charge. So, I used it and it was helpful; very helpful mmm, and that is why I never skipped my medication.

I: Ok, so when she was explaining to you about the labels how long did, she take to explain to you? If you could recall? Do you still remember how long, it took her to explain to you?

P: [Cough]…She tried her level best; maybe 30 minutes[pause] I am a good listener so there is no need for her to take long with me and I have been advised before. So, I said xxx {interns name} let us continue, you have nothing to fear; nothing to worry about, mmm.

I: Yes, so when she explained, did you understand everything that she was explaining to you?

I: I do have to understand because I used those stickers; I used them till the last day. I used them for six months…the first medication that I received had stickers on them and I was never told to keep them for the period of six months, you see. Mmm after I finished the medication which had stickers, I then threw away the containers, but I continued to take my medication and my phone was reminding me.

I: Oh, so you are saying that you were never told to keep the stickers till you finish your cause of treatment.

P: No, I was never told about that part. I destroyed them and came here but I didn’t find xxx (Interns name) but my phone alarm rings, when it’s time to take medication and then I take my medication.

I: Ok. So, what you are saying is that you threw away those containers with stickers after use?

P: Yes, after finishing that period. Uh after finishing the month medication, mmm so I never knew that I must keep them, you see. No one told me to keep them for future use, mmm but my phone was very much helpful because I always receive messages. They never skipped.

I: Ok. So, you were getting SMS reminders?

P: That’s good, SMS reminders.

I: And then what did those SMS reminders say?

P: “Don’t forget to take your medication”.

I: Yes, and then how did you feel about getting those SMS reminders?

No, it helpful, it great, you know. And then in the morning they say, “thank you for taking your medication”. Mmm so, you see, something is burning inside of you.

I: Ok, ok. Mmm, you explained to me earlier on that you didn’t know that you were supposed to keep the labels after use?

P: That’s good.

I: And then when you came here in the clinic then you were told by xxx (Interns name), I believe, that you shouldn’t have thrown away the labels?

P: No, no, no I didn’t find her here, you see.

I: Ok. And then-

P: No! I never asked anyone because the person I should ask about the labels is xxx (Interns name), you see. So, I said to myself no let me not worry because I still get the SMS reminder.

I: Ok. So, mmm what I would like to find out is that after having thrown away the labels did you perhaps receive another one?

P: Yes-no, no, no I was only told you missed, you missed because you were not sending those numbers. I didn’t miss because my reminder is there for me to drink.

I: Oh, so there was a time whereby it reported to xxx (Interns name) that you have missed your medication simply because you were not sending the code after you have thrown away the stickers?

P: Yes, yes.

I: Ok, so how long, mmm was the period where you were not sending the SMS?

P: No, I was not sending the SMS-ok for me not to send the labels [patient counting months] maybe by January I was not sending.

I: Ok. are saying after a month you were no longer sending?

P: No, I was given medication for a month and was told to come back. So, I threw I away both the containers and stickers because I was never informed.

I: So, you only used them for one month?

P: Yes, I only used them for one month because I was never told. I never knew that I was supposed to reuse them until I finish the cause of medication.

I: Oh, I see for the period of five months you were not sending the SMS.

P: No, no.

I: Ok. So, what I would like to know is that were you still visiting at the clinic during this period of five months?

P: Yes, I was always coming to the clinic and my phone was always updating me, ‘drink your medication’.

I: Ok, ok. Now then did xxx (Interns name) tell you or shown perhaps that she is not receiving the SMS codes from you?

P: I did not look for her because she should have called me because they said your health worker will contact you, you see.

I: So, you are saying xxx (Interns name) never asked you anything about sending? Not sending actually because you were not sending.

P: No, no, no, no

I: She didn’t?

P: No, she didn’t. I would be telling lies now because I should have been given new ones.

I: Ok. So, one thing I would also like to know is that did she ever shown you something called an adherence calendar on her tablet which shows different colours: green and red? Has she ever shown you something like that?

P: No, no, no…she only explained to me about the stickers and told me to dial them every time, just dial them 335521, only.

I: And then who told you after that you were not supposed to throw away the stickers?

P: No, no, no, no one told me. I am just realizing it now as we are talking about it, mmm. Uh, I though those stickers were for once off since they were put outside a container. So, once medication was finished, I then thrown them away.

I: And then for the period-

P: What was important to me was the phone which kept notifying me not to forget to take medication.

I: Yes…ok so, you were getting SMS reminders and you didn’t have a problem with those SMSs you said?

P: No, no, no

I: You found them helpful, you said?

P: Yes, very much helpful.

I: Ok, ok and then before you came to the clinic, and you were given the stickers by xxx (Interns name). Mmm did you know anything about the stickers before then?

P: No. It was the first time.

I: Ok. It was the first time.

P: Yes, it was the first time and that is why I agreed to take them when I was told that they will remind me.

I: Ok. I see.

P: And the SMS was for free, you see.

I: …And then, mmm what was your experience using the stickers altogether? if you may give me an overview?

P: Using the stickers was very helpful because you get an SMS saying, “thank you for taking your medication”. So, it’s good; you feel great.

I: So, you were getting an SMS that says, “thank you for taking medication” and that made you feel great?

P: Yes, it makes you feel great [laugh]. They appreciate, you see it now. They appreciate what you are doing because they trust you to take your medication. You see, if you are not taking your medication, you are not doing anyone a favor; you are helping yourself. It for your own health.

I: Mmm so, did you have any challenges when you were using the stickers?

P: No, no

I: Did you perhaps have any concerns about the labels after you were given by xxx (Interns name)? Maybe following two to three days of use?

P: No. Thought are there but I know that research has been done, I said to myself this is a research and let me participate and comply with the rules of my diagnosis, you see.

I: I see, I see, mmm did you have anyone asking you about the labels?

P: My fiancée

I: Oh, only your fiancée.

P Because I am not that much of a mover; I stay at home most of the times. So, I only shared to my wife though xxx (Interns name) told me that I can share to anyone about labels. These stickers helped me a lot and not only for TB medication but for my other medication that I am also taking to ease the pain on my shoulder. So, when I am taking my TB medication it also reminds me to take the other medication.

I: Ok. So, what you are saying is that not only did the SMS remind you to take your TB medication, but it also helped you to take the other medication?

P: That’s good.

I: I see.

I: If I am correct, earlier on you mentioned that after receiving stickers you went home and explained to your fiancée about the sticker?

P: That’s correct. She complained saying that they should have also given her too.

I: Ok. So, your fiancée is also taking TB medication?

P: So, following my diagnosis with TB, she then decided to visit the clinic to test for TB and her results came back positive. Then, she was given the same medication.

I: Ok. so, mmm you were both taking TB medication at the same time?

P: Yes.

I…Mmm did she also manage to finish her course of treatment?

P: No, I can’t answer that.

I: Ok, ok, ok, mmm when you were using the labels were you working?

P: No, I was not working; I am a pensioner in fact.

I: Oh, you are a pensioner. Ok, ok.

P: So, when you were explaining to your fiancée about the labels how did you feel having to explain to her?

P: Uh no, my partner must know my status because we live together under the same roof, you see. I must take care of her, if I am diagnosed with TB she must also go and check because she is living with me under the same roof; we are sleeping together. Uh so, she must have that touch as she did wake up the following day and went to a clinic where she was also diagnosed with TB. As I said, I was not given my TB results. No one ever explained to me, no one explained. They should have told me; I have rights as a patient. Sometimes it seems as if you are bothering someone if you keep on asking questions and they start to look at you somehow, you see.

I: Mmm…I must say what happened was not right and no one should ever experience something like that in their lives but in that whole situation I am just happy that you never quit medication-

P: No. I took my medication from start to finish.

I: Yes, I am just happy that you continued to take your medication regardless of the situation.

P: Yes. That is what I did.

I: Mmm, besides explaining to your partner about your TB status, did you tell anyone besides her?

P: No, no, my health is for me. My partner is the only person who deserve to know as we share the same blanket, not just anyone. I can’t tell people from the shebeen that I am taking TB, no, no, no because they will start running away from you saying things like “that old timer will infect us with TB”.

I: So, the reason why you were not comfortable with sharing your TB status to anybody is because –

P: No. I take my distance because even during hard lockdown there were strict rules instructing people to leave 2 meters from one another. So, I don’t share my status with anybody, I don’t even share a cooldrink with anybody.

I: Mmm ok, if I am correct, mmm the reason you chose not to share your TB status with anybody except your partner is because you felt that you will be discriminated somehow?

P: I do accept but I didn’t think it was necessary for me to share to anybody even though I am aware that it important to share to your close friends, they must know and be careful.

I: So, you thought maybe they won’t accept-

P: Yes, you see, we are judgmental. People start assuming that you will make them sick too, just like that. People always have negative things to say.

I: Ok, mmm and then since you said you told your fiancée, uh about your TB status, how did she react?

P: No. She was just surprised because I didn’t show any symptoms of TB and she also went for a TB test the following morning and I was happy that she decided to test for TB. It was very important for her to get tested for TB since I was just diagnosed with it following the two X-ray tests, I took from the Hospital. I was never told about my results, I was just given medication. I know it my right for them to tell me about my results, but I said you know what let me just get on with it and I just went through the process.

I: Ok. So, according to your knowledge, mmm, you were not presenting with any signs or symptoms of TB until you came here, and they asked you to go for an X-ray test?

P: No, no, I came here to the clinic, and they took sputum and found noting. Then they sent me to xxxx (Hospital name) to take an X-ray test twice and when I came back to the clinic, they told me that I have TB.

I: Mmm, since we are talking about TB symptoms, are you familiar with any symptoms of TB?

P: Fever, feeling too hot and coughing.

I: Ok. So, you didn’t have any of these?

P: No. no coughing, no fever, nothing. These are some of the symptoms my wife had.

I: And your wife which one of these symptoms did she have?

P: Just coughing.

I: I see, coughing.

P: Yes, she was coughing a lot.

I: Do you still remember for how long was she coughing for?

P: She is taking medication.

I: Yes, I mean for how long was she coughing before she went to a clinic?

P: Before she went to a clinic she was coughing.

I: Ok. had she been coughing for months, weeks?

P: No, she was coughing.

I: Ok. mmm, did it happen that maybe…you sent a code more than once a day?

P: No. I was taking medication once a day.

I: Ok. So, you only sent once?

P: Yes.

I: Mmm ok, could you please give me a summary of how you found the stickers, your experience with them. What exactly did you find helpful about them?

I: As I have said, they are very helpful you see. You get an SMS that says, “don’t forget to take your medication before twelve o’clock”.

I: Ok.

I: And them mmm, there is something called a Differentiated Care Model. A differentiated care model mmm, encompasses a phone call, SMS reminder which you were receiving, and a home visit and I am happy that you did. Mmm now what I would like to know is did you receive a call from xxx (Interns name) by the time you were not sending the code, as you explained to me earlier on that you only used the stickers for one month?

P: I threw them away because I never knew that I was supposed to use them throughout.

I: Ok. Did you perhaps receive a call from xxx (Interns name) or sister in the TB room?

P: No, I don’t think so. They were supposed to give me other stickers because I never skipped my medication.

I: Ok. Did you have a home visit? Did anyone visit you at home at that time?

P: No, no, no home visit.

I: Ok. So, based on your experience with the stickers. Is there anything else that maybe you would like us to add or subtract?

P: No, for me I don’t think there is something that should be added or subtracted, mmm I [Phone rings] [pause;35:46]

I: Mmm, before we were disturbed by-

P: By my phone call?

I: [laugh]yes by the phone call. Mmm so, I was asking if is there anything that you would like us to improve on the labels.

P: As I have said for me there is nothing to be added or subtracted, they are just fine the way they are. They are user friendly.

[silent; 36:48]

I: Mmm I think we have come to an end of our interview Sir, and I want to thank you for giving us your time, and patiently waiting. Thank you so much for coming.

P: Thank you for your concern and support after everything we went through.

I: We are glad that you really-

P: Yes, thank you for checking up on us because that gives us hope knowing someone is doing their job.

I: Right now, we have come to an end of our interview and the time is 13:43 PM.
