# Supplementary material for: Acceptability of Digital Adherence Technologies to support people with drug-susceptible TB in South Africa
Source: PLoS One. 2025 Sep 24;20(9):e0332103. doi: 10.1371/journal.pone.0332103 (PMC12459780; doi:10.1371/journal.pone.0332103)
Supplement: S4 File — (ZIP) [file pone.0332103.s004.zip › S4 Transcripts/PwTB/IDI 8_PwTB.docx]

**TRANSCRIPTION NOTATIONS**

| **Label Key** | **Meaning** |
| --- | --- |
| **I** | Start of each new utterance by the Interviewer |
| **P** | Start of each new utterance by the Participant |
| **N** | Note taker |
| **{ }** | Indicates that details were changed or pseudonyms were used to anonymise data |
| **( )** | Indicates the description provided to anonymise data |
| **XXX** | Words were omitted to anonymise data |
| **-** | Breaking into a sentence by the next speaker |
| **…** | Pause or drawn out words |
| **[ ]** | Indicates noise made, e.g. [laugh], [sigh], [pause] |
| ? | Beginning of utterance by unidentified speaker or questionable text |
| **[inaudible segment]** | Unclear section of the recording |

I: Do you agree to be audio-recorded?

P: Yes, I agree to be audio-recorded.

I: Date xxxx (interview date), PID xxx, location XXX (clinic name), language used IsiZulu [Flipping paper]. Can you briefly tell me about yourself?

P: Mmm I am XXX umm (participant’s name).

I: I want to know you; can you briefly tell me who are you and where are you from?

P: I am XXX (participant’s name). I am originally from xxx (Province), xxxx (town) and we moved to xxxxx (suburb name). As you are asking me about stamps [labels], they are very important at helping with adherence because they can tell you when it’s time to take your medication. They are very important in life because when you send the code someone is able to see that you have taken your medication. These stamps [labels] are very helpful because by using them I was able to adhere to my medication at all times. I wish others who would still be using stamps [labels] to continue following the rules of the stamps so that they will be healed and educate others because when you are better, you have to advise them about the importance of the clinic, and they will be assisted.

I: Okay.

P: Mmm.

I: So, when did you find out that you have TB?

P: Uh I find out on the of xxxx (date) here at the clinic.

I: What made you go to the clinic to find out if you have TB?

P: Uh it because I was not sleeping at night, had symptoms like sweating, coughing and chest pains. It then came to my thought that I cannot be sweating every day because someone told me that daily sweating is a TB symptom. It then came to my mind that I’d better go to the clinic to see what I have. At the clinic they checked me, asked for the sputum to do TB testing and the result came back positive.

I: How did you feel when the result came back positive with TB?

P: I was feeling pain because I did not expect to be diagnosed with TB. I also felt ashamed of having TB, I was healed because I was following all the rules the nurses told me to follow. The stamps [labels] helped me, and I am able to send the SMS after taking my medication; I am back to normal. Other people should continue to do so, if someone is sick, the clinic is there to help mmm.

I: How often did you come to the clinic, were you walking or took taxis?

P: I used to come twice a month, using taxis.

I: If you estimate, how much were you spending going to the clinic?

P: I was spending R30 per day.

I: Mmm.

P: Mmm per day.

I: I heard you talking a lot about stamps [labels], can you tell me more about stamps [labels]?

P: Stamps [Labels] are very important because they tell you that every day you must take your medication with water and send a SMS when you are done, so that they can be able to see that you have taken your medication. If you did not send it, they will send it saying, “you have not taken your medication.” They are helping a lot because they remind you with a SMS that its time to take your medication. Its unlike boxes, my grandmother also had TB, but using the box. I saw that it is useful through grandmother because when it rings, she would ask me to bring it so that she can take the medication. I also know that at 11:00, it time to take my medication; l and dial the number on the stamp [label]. Stamps [labels] helped me a lot because everywhere I went, I had my medication and stamps with me.

I: So, when did you start using labels? immediately when coming to the clinic, they told you about labels? What happened?

P: Immediately on the xxxx (date) after I was given my medication, they asked if they could give me stamps or the box. I said that I will take the stamps [labels] because I will be able to use the SMS. I started my treatment on that day using labels. Labels are very important, and they helped in finishing my treatment in the past few weeks.

I: Who explained to you on how to use the stamps [labels] and how did you feel about the way it was explained to you?

P: Sister XXX (referring to an intern) explained to me on how these stamps [labels] work. I then asked if sending the SMS cost money or it free and she said it is free of charge. After taking your medication you send a message to this number then it will appear to them that you have taken your medication. I was happy that it was not a difficult thing. After taking my medication I would send the SMS and I did this until the end of my treatment.

I: Is there anything that you would like to add or change on how the nurse explained to you?

P: The way she explained how important stamps are as a reminder to take medication? Sometimes I wish stamps [labels] to be always there because when someone is healed, another one get sick. The change that I would like is for the stamp [label] to be kept so that people will end up using them. They are very important to a person with TB because when there are no stamps [labels] , they will deliberately skip medication knowing that nurses will not see. If there are stamps nurses will be able to remind the person that they did not drink the medication. When you do not have stamps, you do not heal and become more sick because you did not use the stamps as nurses could not see that you did not take your medication.

I: When sister explain to you for the first time, did you understand? Did you face any problems while using them at home?

P: I did not have a problem because the nurse told me to send using these numbers underneath. After taking my medication at home, I used 42352 and then used those underneath numbers.

I: Before having TB, had you seen these stamps from anyone else besides here at the clinic?

P:Uh, I do not want to lie; I have never seen the stamps anywhere. I started seeing stamps [labels] when the nurse explained to me on how to use them and I understood mmm.

I: Okay, I heard you talking about stamps, and you also know more about the box? What can you say or what do you understand about the box?

P: Umm the box is important because there are people who cannot use the phone. Its also important because it tells you that at the certain time, it is time to drink your treatment. The nurses ask that at what time do you like to drink your medication and you tell them. If you said at 12:00, they will set the 12:00 alarm. Then at 12:00, the box will ring reminding you that it time to take your treatment. I saw the importance of having a box with a family member who was using it. It helped a lot because she was an older person, when it rings, she would say that it time for her to drink the medication. The are very important because it tells you that “when I am ringing; it time for you to take your medication.”

I: Okay, on your opinion, which one is easy to use between the box and the stamps [labesl] and why?

P: Umm what I can say, they are both easy to use, most of the times older people are not educated and the box is more helpful to them. The box also helps the younger persons who can read but unable to send the SMS like there is someone who cannot use WhatsApp or anything in a phone. So, the box is able to help that person to continue with medication until the end of the treatment.

I: On how the labels helped you, what made it so easy to use?

P: Uh it was because the stamps [labels] did not give me the problem. When I send the message after drinking my medication, they would reply with “thank you for taking your medication” then I would make some noise because of happiness. I felt sad If they do not reply. When I am not at home and left the medication and stamps; I take my medication and use the stamp immediately when I arrive home. The stamp [label] was so easy; it did not give me any problems and I understood how it works.

I: Is there any barrier you faced when using the stamps?

P: I have never had a problem since I started using them; they were good.

I: I heard that sometimes you are not at home, how did you feel when you receive the message reminding you to take the medication?

P: I felt guilty, I did not like to be reminded to take the medication because it easy to take one pocket of medication and stamps [label] put them in the bag and go to town. When I am in town and it time for medication, I drink my medication, take the stamps [label], and send the message. And they shall not call me. But when I went out without medication, around six, they will send a message because they see that I did not drink the medication and the time has passed. So, I felt guilty because missing time for medication is not something I am used to. I usually take medication and stamps with me so that when I get where I am going, I drink my medication and send the message.

I: Were you working when you were on treatment or not?

P: Uh I was not working.

I: So, on your opinion, would there be a problem when working and on TB treatment?

P: I do not see there would be a problem because the stamp [label] and medication goes together. Its like when you taking a lunch box to work, you would eat your lunch then take the medication with your stamps [label]. I do not think stamps [labels] can be easily lost. Unlike when using a box, you would put it somewhere then when leaving for work in a hurry and forget it. But when using the stamps [labels], you take them in the bag and when it time to knock off at work, you take your bag with medication and leave.

I: So, in your opinion, the person using the box can have a problem putting it a bag when going somewhere compared to a sticker?

P: Uh it can be a problem, sometimes you can put it in the bag with your phones, then the alarm ring, and you take out the box from the bag. When the person left the place maybe forget it which is not good. With the stamps, immediately after taking your medication, you put them back in the bag. You can also forget the box after taking it out of the bag and talk to someone, without noticing you leave it there. The stamps are attached to tablets. After taking your medication you just put them back in the bag and leave.

I: Mmm so in your opinion the box is a burden?

P: Uh when I take a look at it, its a burden, so I saw that stamps [stickers] would be simple, and I understand them. On my side the box is burden because it will force me to carry it wherever I go even if I was not prepared to carry a bag. With the labels, I am able to keep the numbers I use to send a message after taking medication. Each sticker has its own number, but they are different, like this one is 135 and this one is 737. If I know that this week, I am using code 135, I keep this in my phone and use the medication in this container. I do not take them all when I am going, then when I arrive there, drink these three and send the message and it gone, simple like that.

I: I heard you talking about the box and you also have an experience with the labels. Who do you live with?

P: I live alone at night. And most of the times during the day, I am with my grandmother, my sister and her two children.

I: I shall talk about TB and everything. Who did you tell at home that you are on TB treatment?

P: My whole family, they also knew about my grandmother’s TB status because she was using the box. After receiving my result, I was given bottles to collect sputum from my family members then TB was detected. Grandmother I and then took the treatment together until she was discharged using the box. That is why I say it was working for her because even when I was not around, I was able to tell her when it rings, and she would open it and drink medication, then put it back and close it, until it ring tomorrow. She is old and always sleeping, she knows that when it ring at 11:00 that it time to take medication because I put them closer to her. As an older person the box was simple for my grandmother.

I: How did your family members feel about you and grandmother having TB?

P: Uh they were shocked and worried because they did not expect family members to be diagnosed with TB at the same time. They were shocked that maybe they might also be told that they have TB. At the clinic, they advised us that we must wear mask all the time when we are around people to prevent them from being infected with TB.

I: Besides your family, did you tell anyone maybe friends, your partner or anyone?

P: Uh I told the mother of my child. I told her that I was at the clinic, and I am diagnosed with TB and only to find out that she is an understanding person. It looked like she was once diagnosed with TB because her medication look like the one I am taking at the moment. The difference is that they did not have a sticker. I think she kept it a secret.

I: How did you feel after telling her?

P: I felt bad, but I believe that sometimes when you are sick, you tell the person you trust so that they are able to help when feeling pain. I went to see my mother and she was shocked because one of my brothers passed away because of TB. I told someone because I thought I am also going to die. I was healed after I accepted because I was told that I shall get better if I treat myself like this. I had hope because when someone encourages, you get healed, and I become happy.

I: So, umm condolences about your brothers passing, but do you think if there were labels, they would have helped your brother to take his treatment?

P: Uh I think they would have helped him. At home, they told us that he killed himself by smoking, drinking alcohol and on top of that taking TB medication. He was not supposed to smoke and drink alcohol while on TB medication. When you drinking alcohol, you urinate and urine flushes out the medication, it like you did nothing.

I: So ,in your opinion, did you think people who teach about TB are doing enough or not when you found out that you have TB? Did they elaborate on how you get infected with TB and were you satisfied?

P: Uh what I can say? Like you are working with TB, you explain to me about TB, but it not enough. I would like if there can be another technology. And you explain to me that TB is spread like this, and others explain in their own way. Like someone who was at home with a private car, they explain on how you got infected with TB. They said that you do not get infected by sharing cigarettes, but with the air in a taxi, like when someone who has is standing in front of you. TB is a killing disease you need to treat yourself like this, do this. When you combine this, you can see that sometimes they tell the truth, because if I can get the opportunity about advising people with TB. Maybe someone took a six month treatment and over one year treatment. Maybe mine was for chest, another person had one for bones. It can be nice if we can know all types of TB. Like when I was sick, I advised my friends that I am coming from a situation like this, they checked me like this. So, take care of yourselves because I would not like seeing one of you guys suffer because of TB.

I: I heard you talking about your guys. Did you tell them that you were on TB treatment and how did they feel?

P: Uh I did not tell them and when I am with them, they would say I am coughing because I have a flu. After seeing that I am healing that where I told them. One of my friends did not disclose, I saw him here at the clinic. At the township, I told him that I saw him at the clinic, and he told me that he also has TB. I sat down with him and said that I also have TB, I am from the clinic and I need you to take care of yourself. If they advise you not to eat or eat something you do as they advise you, you shall heal. It feels sad to see the person you live with sick.

I: What do you say when you advise your friends, do you see change?

P: Uh there is a change on some of them. Some people you are able to advise them saying TB does not want you to this, you need to do this and I have been there you saw me. One of them would say we saw you; we thought you were going to die. And I tell them that it depends on how you treat yourself. If something has never happened to you even if I can give you an advise you will not take me seriously. If I say TB kills, someone will say I am insane why it did not kill me. One day you get infected and not follow the rules which I followed. Then we hear that you were killed by TB, its painful because they know it kills, but if you can tell people about these labels, they shall get cured.

I: Are you talking about these labels?

P: Mmm .

I: Have you ever sent the SMS more than once in a day?

P: It happened almost a week and I called the nurse to report that she must not be surprised when she does not get my messages because I send them and there is no response. I feel guilty when they do not report within the “thank you message” within 30 minutes. Sometimes I will send four times, then I get the response. I do not feel good because they might think that I did not take my medication. The nurse told me not worry because it happens when there is a network problem but when the signal is back the nurse receives the messages. “If I have not call to remind you to take you medication, do not worry.”

I: So, you became confused when you are not receiving the response?

P: Yes, I felt a pain because I sent the message and there is no response. Sometimes I would forget to send and remember after few minutes. I get worried because they might think that I am not taking my medication, why I did not send the message. So, that is why I felt so sad.

I: Most of the times when there is a problem sending a message, what do you think was the problem?

P: Umm according to my thought, it was network, and I was not using a smart phone. I even thought maybe those small phones have the network problem. When not receiving response deep down I would be like I took my medication, sent the code, but no response. What worries me is that they have not received my message.

I: Have you ever received a call reminding you to take your medication?

P: They never called me it just that when I had a network problem, they would send a message saying I must not forget to take my medication. Sometimes I receive a message saying the Healthcare worker will call about why I did not take my medication, only to find that I did take it and sent a message. Sometimes I got confused and call the nurses that I sent the message after taking the medication, but I receive a message saying I did not. They will tell me that when the network is back, they receive all my messages.

I: Were you not worried that you take your medication and send the message, but in the morning, you receive a message?

P: Yes, it bothered me because I would send and after 45 minutes receive a message saying I have not taken my medication. And I would be like how come because I sent the message. I called the nurse, and the nurse told me that sometimes there is a problem, and I said no problem.

I: Mmm

P:Mmm.

I: Okay, when you receive these messages every day, how did you feel?

P: Uh when they tell me that sometimes they have problems?

I: Umm that you have took the medication, sent the message, but the problem is not solved?

P: I felt pain, but what worries me was that they might think that I do not take my medication whilst I am taking it. And not getting the “thank you message” after taking my medication because I think they will shout at me saying I did not take the medication. Uh that what made me feel pain.

I: Have you been shown your adherence calendar?

P: Uh the nurse showed me and thanked me for taking my medication and said that it will help me. The other sister would ask how I am doing. She would tell her that I am doing well and not missing days. That is the reason why I felt the pain when the message is not delivered.

I: What was helpful about using labels for TB treatment?

P: What was helpful is that these labels were easy to use. Each and every day I was taking my medication, and use labels and I was enjoying them. The stamps did much for me and I did not forget the labels and medication everywhere I go.

I: Okay, I heard you talking about the car, and you said you have never received a phone call. Why the car was checking you and where did it come from?

P: Uh they told us they are from the clinic, sometimes when I am here at the clinic, I would see some of them if I were here with my grandmother because sometimes we are given a same date to collect our medication. When we arrive at home, few minutes later, they also arrive saying they are from XXX [clinic name], “we are here to see grandmother” and would say that she is coming from the clinic. “We wanted to know who grandmother is and how many of you are diagnosed with TB.” Most of the times they would find me with grandmother. They also wanted to collect sputum from our contact and I told them that our contact’s sputum were collected. They told us to always wear our masks and not hold babies and not to cough in public.

I: So, you can say that home visits are helpful to patients?

P: Uh visiting patients at home?

I: Yes, is it helpful when workers from the clinic visit someone with TB at home?

P: Umm my grandmother as an older person sometimes may not be unable to take care of herself. That is why I sometimes see that it helpful if they go check people at home because some of them cannot take care of themselves. Sometimes some older person cannot walk because of TB. The nurse told me that they also deliver TB medication to elders who uses wheelchairs. It important because when they visit and find that you are not taking the medication correctly or you are not taking it. They help you to take medication. So, you must make sure to always drink your medication correctly.

I: Mmm so you are saying they were visiting your grandmother, in your opinion, was there any change when they left?

P: Uh when they have visited, after they left, my grandmother would say they are telling her different things and she is shocked because when they were told that TB kills. My grandmother was a domestic worker and that when she started smoking. It helped that she was unable to smoke because they come at any time. Grandmother had side effects from TB medication sometimes and she would say that she is not taking the medication. I would say “no ,no drink your medication so that you shall be healed.” So, it was okay.

I: Okay, in your opinion, what need to be improved on the counselling process? How was your counselling process?

P: Uh the counselling is great because they tell you about TB that it curable and how to take care of yourself. If you are doing a certain mistake, they advise you to do the correct thing. When you are at the clinic nurses check on the file how you are doing, they also work like nurses. They say when you with your family you must always wear your mask for their safety. If I know I am the one with TB, I go outside and be alone. It not because they hate me, but it for their safety because I would cough, and someone inhales bacteria. It will not be nice when that person start to get sick and we are all coughing in the same family.

I: Okay.

P:Mmm.

I: In your opinion, were you satisfied about the counselling or is there anything to be changed?

P: Uh what I can say is that coming to check on grandmother because mostly they give you some guidelines. They told grandmother not to lose hope, TB is curable. And when they are at home grandmother, I would tell them that my grandson also has TB. I hear them because when they visit, I am usually with grandmother. They tell her that TB is curable like nurses when you are at the clinic, they tell you that you are gaining weight which means you are getting better. If they say those things they encourage you, and you can see progress which means I will be cured as the time goes on.

I: So, your situation when starting and now after finishing the treatment, do you see any change? And what makes you happy when you look at yourself?

P: Umm most of the times what makes me happy is that I am getting better, there is no night coughing now. When starting the treatment, I had side effects in a way that I wished to die. I had cramps like I am having a stroke. Both grandmother I and had side effects. I was happy when I find out that I am being discharged because being sick is not nice. I believed that nothing has no end.

I: Okay.

P:Mmm.

I: Because you know them all between the sticker, the box, messages, phone calls and home visits, which one is the best and why?

P: Labels are the best for me, they encourage me as I said that I took them everywhere I go. When I have labels, I can take my medication everywhere I am. For me labels are simple than the box. If I am with my cousins, I would ask if they saw how labels are helping me. I would not be here if there were no labels; they encouraged me to take my medication. I end up sticking them to my diary so that I will be easy to remember because I sometimes forget. If it in my diary, then it easy when I enter the room to see the stamp and remember to send the message before I go somewhere. Labels were easy for me than the box, including home visits and phone calls. I used the stamps nicely and made it easy to take my medication.

I: Okay, what can be improved on the labels since you said you used it nicely and you had your own to always remember to take your medication?

P: Uh in my opinion the labels are good to use as you can see the instructions here written “everyday take your medication with water and send the message.” Here is the picture showing which number to send then it tells you if it was sent. You are able to do this, it simple.

I: Okay, mmm in your opinion, is there anything that can make not to use labels?

P: Uh some people cannot keep the phone for a long time, it get lost. Another one cannot keep the medication and labels. That person can misplace the sticker and forget. When you have the box, everyone can see it, even over the wardrobe it can be easily heard ringing. It not easy to use labels to people who cannot read or send a message. Maybe that person can ask the neighbour for help and when they are not around, a message cannot be sent in time. The box is simple when it rings, you open it, take your medication, drink it, and close it. It hard to use for other people; sticker need your understanding.

I: Okay, in your opinion you are saying the box and stamps must be presented and given as an option to choose one of them?

P: Uh it would be nice like they did the first time. They said XXX we have a box and stamps, which one would you like to use? I took a look at the labels and told the nurse that the box would be difficult for me because sometimes I visit my uncle or anywhere it can be forgotten. But labels when I have the packet of medication with me, it will not be easy to forget to send the message.

I: So, you mean in our ACSENT Project we must show you both stamp and a box not one of them?

P: Uh they would have done a great job by bringing a box and stamps because people at our age who cannot use the sticker. It would be easy if you explain that stamps work like this and the box like this. Most people say they will not be able to SMS every time, “please give me a box because I shall hear it when it rings.” And someone will say “I shall take labels because they are easy to use.” Another person will be shy when going to town and have to take the box along, it will ring when around people in town, do you understand? Another person will say labels are better, you just take out your medication in your bag, open the tap, drink your medication, and send the message. You put it back and go. In your completion it better that when someone diagnosed with TB you show the person the box and stamps to choose.

I: So, in your thought when it comes to stamps [labels], what can be done to make it easier for the people who are unable to use stamps [labels]?

P: Uh what would make it easier, lets say there is no box. It means there had to be someone in the yard at home to help that person to use stamps[labels] , it would be easy like that. If it’s my family member; I would help that person to send the message after drinking the medication.

I: Mmm…if there was a voice reminding those who cannot read , would there be a problem?

P: Uh like if there was a voice as my phone ring [ making a ringtone sound] receiving a “take your medication” message?

I: Mmm something like that.

P: It would have been difficult because some people cannot handle the phone. It would be a problem when the phone is not charged and waiting for waiting for electricity after load shedding, until tomorrow and maybe that person would have forgot by then to drink medication because what reminds him is broken. The way they did the box, because technology make things simple, but as people we are not the same. If someone chose the stamps [labels] but cannot use it, someone help that person at home because the box is difficult for that person.

I: So, what is your experience on using the stamps [labels]? Describe how satisfied you are with the use of stamps?

P: I am very satisfied because we had a sharp relationship with the stamps like you have a working relationship with someone. The stamps were like that to me until I was cured. Without the stamps I would have not gotten better. I am unable to put my medication in one place, but I put it in a safe place because I have nephews. I keep them in different places, but I know where to find them when it time for medication. With stamps everything is nice uh.

I: In overall, what can you say about stamps?

P: Uh I can say these stamps have done a lot of good things; I wish that it helps other people. I am not saying I do not like the box. As people, we cannot all be helped by one thing and maybe there is another technology coming, things change, but labels stay the same. Maybe they are not like this, they have put another technology inside. I would like that if you were thinking about inventing another technology you can come back here. I would like those technologies to help other people like stamps [labels] and boxes. But these things are made to show if someone is taking the medication, the nurses see you using the tablet if you took the medication or not, so that they shall call and say they did not receive your message. When you say you did, they shall tell you that they shall check. If your phone had a problem, it would deliver the message later on. If there were no stamps, you would take the medication and when you are at home not drink it because you know there is no way you will be seen. Boxes and labels are helpful, they helped me. I know there are other people who used the labels and got the help; they are better like me. Just taking care of yourself. You cannot go back to bad habits because you see that you are better, take your time, check yourself and if you are coughing, go to the clinic. Maybe you will be alright, maybe they might say it was hiding. Stamps [Labels] were good to me.

I: So, we are about to end our conversation, what you would like to say?

P: As you are doing this interview, I would like to encourage those who will be diagnosed with TB after us-and God has mercy to give us a second chance because that person can take care of himself. There might be someone who was taking medication with us, but not correctly, got too sick maybe ended up passing away. What you are doing is good. When you counsel me, you are encouraging me, tomorrow I shall tell people that I was once sick, but there are people like you who call us to explain about TB and ask about technologies, we are using like I am feeling good. I like your company for the good thing it is doing. Tomorrow I might be asked by someone about how were labels, how were they working for me. I shall tell the person that they are number one. I am able to tell the person that when using a stamp, you do this and this. It simple. Someone asked if the data is used or anything. I laughed because we were all sick, I told him that here it written for free. I taught him and he took the stamps [labels] . There are some men who were also using the sticker because some of them were not too sick, TB does not make us sick in a same way. Some people get the date for collecting treatment that corresponds with the workdays.

I: Mmm so in your opinion, stamps [labels] are easy and helpful especially to a working person?

P: Uh very easy for a working person and someone who is busy. It very easy for that person because he will not go around with the box. With the stamps you only take one packet and go. Labels are very better as long it is not dark; you drink them and send the SMS. Its difficult with the box because you can carry it without noticing that the battery is empty. When it time for medication and it does not ring, you forgot to take the medication, then you miss the day. You know when using the stamp [label] , you just cut the number of tablets that you need put in the bag or pocket. When you arrive at work, you drink your medication and send the message. It very easy.

I: We have reached the end of our discussion, thank you for your time. Thank you for starting the treatment using the stamps[labels] until you are being discharged, sent messages very well. And those people whom you gave advices may keep doing that. Thank you for helping us.

P: Thank you.

I: The end time for the interview is 14:37.
