# Supplementary material for: Acceptability of Digital Adherence Technologies to support people with drug-susceptible TB in South Africa
Source: PLoS One. 2025 Sep 24;20(9):e0332103. doi: 10.1371/journal.pone.0332103 (PMC12459780; doi:10.1371/journal.pone.0332103)
Supplement: S4 File — (ZIP) [file pone.0332103.s004.zip › S4 Transcripts/PwTB/IDI 9_PwTB.docx]

Translation Setswana

| **Label Key** | **Meaning** |
| --- | --- |
| **I** | Start of each new utterance by the Interviewer |
| **P** | Start of each new utterance by the Participant |
| **N** | Note taker |
| **{ }** | Indicates that details were changed or pseudonyms were used to anonymise data |
| **( )** | Indicates the description provided to anonymise data |
| **XXX** | Words were omitted to anonymise data |
| **-** | Breaking into a sentence by the next speaker |
| **…** | Pause or drawn out words |
| **[ ]** | Indicates noise made, e.g. [laugh], [sigh], [pause] |
| ? | Beginning of utterance by unidentified speaker or questionable text |
| **[inaudible segment]** | Unclear section of the recording |

P: Yes, I agree.

I: Okay, date is xxxx (interview date), PID is xxx, time is 11:40, location is xxx (clinic name) Clinic, language used is Setswana. Uhm…my brother, may you please give us a brief about yourself, who you are and where you are coming from?

P: Uhm…my name is xxxx (participant’s name), I come from xxxx (area name), Uhm…Yes, I am xxxx (participant’s name), and I am 30 years old.I came here because I am suffering form TB.

I: Mmm (yes)

P: Yes. Actually, uhm…I arrived here, on the…it seems like it was on 13th of October, and then I came here to check because I was from the work, I was not feeling fine at work, so, I decided to come here at the clinic to check what’s going on. So, they told me to test, then I tested, they told me to come back on the…which day? On the…on the 15th or 16^th^ , somewhere there, that’s when I could receive my results. Then during that period when I came back, I found that they say I have TB indeed. Then I started to intake my treatment on the eighteenth, yes on the eighteenth October that’s when I started with this treatment.

I: So…my brother when you arrived here at the clinic, how did you feel when you find your result saying you are TB positive?

P: Uhm I didn’t believe my sister; do you get me? To be honest I didn’t believe, the problem was that hey, on my mind I was not even thinking that things like that are existing thoroughly, do you see it?

I: Mmm.

P: But I said indeed, just because this is life I won’t dodge, I won’t run away, do you get me my sister?

I: Mmm.

P: I just agreed with them just because I won’t argue with them, those people won’t tell something that is not existing indeed.

I: Mmm.

P: In that way, I said no, I should do everything that they tell me to do, I will just follow, just because there at the yard, it had infected my mother-in-law – my mother had been infected by this thing, TB, do you get it? I saw her situation that she had been inside – she was also infected with HIV, lucky enough I have not infected by HIV, do you get me? I realized how dangerous it is – it made her a bedridden at the hospital for a while. So, but I told myself that there is nothing that I could do, same time I started to take whatever they were giving me.

I: Mmm.

P: Mmm.

I: So, where you are staying – what are you using when coming to the clinic or are you walking?

P: Uhm…it depends on the days, when I’m okay I use a transport and some other days, no, I walk. I mean I was staying at (area name), I found a house, then I moved to (area name). By the time I came to end, I was compelled to use a transport, I had no other plan because it’s far, I won’t travel from that side to here – coming on foot, walking? It’s impossible.

I: So, how many taxis were you using to come to the clinic, when you were there and how much did it cost you, could you estimate for us?

P: They are two indeed, it’s thirty – it’s thirty rand, fifteen, fifteen. Hey, fifteen, this means it’s sixty-rand return.

I: Oh,

P: Mmm.

I: So, how many times were you coming to clinic and how did that thing affect you?

P: Uhm…on a month I was coming – It seems like I was coming - at the beginning there, I was coming twice.

I: Mmm.

P: It was not giving me any problem because – I did like something that is going to help me, do you get me my sister?

I: Mmm.

P: No, I had no problem, it caused me to decide -

I: Mmm.

P: Mmm, so that I could receive my treatment.

I: Oh. So, does my brother recommend these things that are in front of him, the sticker, and the box?

P: Mmm, very well my sister.

I: Oh.

P: I trust them indeed.

I: What could you say about the labels, let’s talk about the labels, tell me about the labels, what do you know about labels.

P: About the labels?

I: Mmm.

P: Here on the stickers, what I could know is those things that looks like the peaches, they were reminding me to intake the treatment thoroughly.

I: Mmm.

P: Yes, I did know that if I take one pack then I should put one sticker, I take indeed knowing that when I intake I SMS, when I intake I SMS. And then the way they taught me, it clocks, it was clocking to them on their phone, they could realize that I have taken them. I didn’t use the stickers [labels] during that time because I had lost my phone, do you get me? I arrived there and told sister [TB Nurse], that’s when they gave me the box.

I: Mmm.

P: Yes, and then I ended with the box until.

I: So, when did you start with the sticker, and how long did you use it – how many days?

P: Uhm…I started with it indeed, it was on the eighteenth when I start with it. Uhm…to be honest the days of the sticker are not that much my sister. It seems like it could be around…ten, ten to fifteen, somewhere around there. That’s where my phone was lost, I have been distracted by phone, I came here to tell the sister(nurse) and she recommended me that thing, she gave me…what do they call it? A box.

I: Mmm.

P: So, I used that box until now.

I: Mmm.

P: Uhm… what I realized is that they’re treating me well definitely and you will also realize that is a sort of game, do you see? The thing is that when you drink you enjoy, you know that you should…you get me, right?

I: Mmm.

P: It doesn’t give you any difficulties, even if you sit, you know that you must…as if you are on a small game, you get me, right?

I: Mmm.

P: Mmm.

I: Mmm, sort of a bit game.

P: Mmm, It’s okay. Even your mind, it takes it away, do you get me? You don’t even recognize that you are dying or what’s happening.

I: Mmm

P: Mmm

I: So, since you used the stickers, did you ever forget to SMS?

P: Yes I forgot to send, there was a time where I strayed, during that time I had taken my treatment only, but I didn’t take these things, the sticker. I had taken the treatment only, without any sticker because I don’t remember myself missing to drink, but I just only remember when I missed SMSing.

I: Mmm. So, you didn’t find the SMS that reminds you?

P: No, it was a call if I remember well, they had called me and said – uhm…sister called me, yes, she called me and asked me if I drank, I told her that I drank, she said, “I’m now surprised because you don’t send,” and I said, “hey sister I have been interrupted, so and so.”

I: Mmm.

P: Mmm.

I: But how did you feel when you…when you drink and not SMSing?

P: Mmm, it’s just that I feel like I make a mistake by the way they taught me indeed. So, I regard them as the best people because they are the ones that are willing to help me indeed, so I felt guilty, but I console myself by saying that I drank them, right? Yes, I felt like I wronged a very serious thing.

I: So…like after you received that thing of calls together with that intervention of the SMS -

P: Mmm.

I: How did you react – what did you do about it?

P: Oh, I made sure that I plan, then I received them, these things - then I went back and sent it again, I ended up sending them.

I: Mmm.

P: Mmm. I came up with a plan, I said no, I should find this thing, a sticker so that I would be able to send.

I: Mmm. So, but for now you can only tell us about the box. Tell us only about the box, your experience about the box.

P: Mmm, yes, as for box I used it very much – it’s one that I used for a while, now, right?

I: Yes.

P: Uhm…I relied very much on the box; do you get me? And according to me I recognize it as the best, do you get me?

I: Mmm

P: Mmm

I: This one of phone, I recognize it, but it’s possible to forget a phone or doing something, so this one alerts you, regardless of what’s happening, do get me?

I: Mmm.

P: Even if it might not threaten me, even that young kid of mine, she already knows about it, when it rings you could hear her saying “hey, these are your things,” it doesn’t confuse at all. I didn’t miss since I received it.

I: Didn’t you miss?

P: No, It doesn’t easily miss because it’s ringing and it’s so louder. Regardless of where you come from or what’s happening, by nine o’clock you will hear about it.

I: Mmm

P: Yes.

I: So, by the time you arrived here at the clinic for the first time, who was explaining to you about these stickers?

P: It’s that sister, I forgot her name.

I: Mmm

P: Yes, but she is a sister.

I: Mmm

P: She is the one who gave me everything.

I: Yes

P: Mmm

I: Did she also give you these stickers?
P: She explained everything to me, she also showed me that uhm…when you didn’t drink, it shows this and this, it shows red colour, and when you drank, it shows this and that, it shows green colour, and what, what. She gave me everything.

I: When they were explaining to you about…about this thing, the person who explained you about the sticker, when you recognize is there any other thing that you would like to add there, the one that you feel like she should have told you at the beginning when she was explaining to you?

P: Uhm…about the sticker – I realized that the sticker has a problem, it doesn’t help with anything, you should think for yourself – you should remember that I should drink, it doesn’t remind, seriously. The only thing you must do – you should report that you have taken your medication, so do you recognize that this is the same as nothing if I [inaudible segment] because it doesn’t do – it doesn’t alert me, it doesn’t say that this is the time. I’m the one who should send after I drank.

I: Mmm.

P: No…the sticker – it does work but it works after I have done my things.

I: Mmm.

P: It’s the same as I do it myself.

I: I hear you that you are talking about the stickers, but it seems like you are not satisfied with them, what should we change about the stickers?

P: The matter that is needed, this thing should remind me, like maybe it can say – yes, it should speak in a certain style that papa this is the time. Not that I should do after it does – not that I should do first, then it follows me behind. This means that if I could forget – I could drink and after that going out and leaving it there and not holding my phone, not SMSing, and doing nothing.

I: Yes, so –

P: You see, this one is good because once you opened, it clocks where it should clock at the phone, right? And it helped me earlier that nine couldn’t beat me, even if it could beat me, by nine o’clock in the morning, it could awake me, then quickly I could make small amount of food then eat a bit, so could you realize that it has beaten me with only thirty minutes? I’m able to drink them around half-past. Do you recognize that it didn’t leave me very much. So, about the sticker I say maybe – I don’t know how but it should also speak. The box is speaking, and the sticker doesn’t speak.

I: Oh, you say on the sticker if it could remind you before you drink –

P: Yes [inaudible segment] -

I: After you drank then send an SMS –

P: Yes, [inaudible segment], It reminds me, if it doesn’t remind me, I could go to sleep, if I drink at nine, I will go to sleep and awake at one maybe if I was at my things yesterday. You could find that I slept around four, I awoke around twelve, it didn’t say anything, it’s here and I found it here.

I: Okay, so, my brother can you please tell us about the box? How does box work? What do you know about the box? Everything that you know about the box.

P: Yes, this box –

I: I feel like -

P: This box is excellent, you see this box is very smart, yes, it’s very smart because it’s able to speak, it’s smart because it starts you from the bottom, it reminds “hey my bro, this is the time to take medication,” you get me, after opening it clocks, right? there is no way you could make any mistake for it unless you just open it and then close it without taking your treatment. And I don’t believe that there could be a person who can do such a thing, unless if the person is selfish, you know?

I: Yes

P: After it rang you open it, what I realize is that the box is completed, it rings loudly, it’s fine, and it’s also strong cause its battery doesn’t flattening, [inaudible segment], yes it won’t confuse you with anything. I don’t think it should be extended with anything, it’s okay in this way.

I: Okay, so, do you think is easy to use it?

P: Mmm (Yes), it’s very easy. So, you get used to it, even now I’m still get used of it***,*** do you get it? Even this time, when it’s no longer there, I just said “hey I finished that thing.”

I: Mmm.

P: I get used of it; I got used of it, even that child of mine, you get him saying “that thing of yours is no longer here, we’re no longer [inaudible segment],” do you get it? And there was no possibility for me to miss, even if I’m sleeping, I’m lying, the kid could come and say, “here is that thing of yours.” No, this box doesn’t confuse, it’s totally still.

I: It’s still, right?

P: Mmm.

I: Is there any problem that you observed about this box?

P***:*** No, they told me about the batteries, what, what, but since, they didn’t do anything. When I brought it back, I’m sure that it was still lighting and strong.

I: Okay

P: It had never confused me with anything.

I: Is there any other day where you did open your box more than ones?

P: Yes, I did open it more than ones.

I: Mmm

P: Mmm

I: What was happening?

P: But not several times.

I: Mmm

P: Two – three times, I opened it for the first time, you could notice that during that period nine was already beaten, then I was strayed because I was not awake. When I awake, I should eat first, do you get it? I won’t drink without eating, so, I took it and opened it, I left it opened so that I could eat a bit [inaudible segment], do you get me? So, that kid closed it, then it clocked again, in that way it caused me to open it again.

I: For drinking the medication?

P: Yes, I couldn’t drink them with empty stomach.

I: Mmm

P: So, I opened it and left it there opened, because I knew that if I could close it, it is going to clock.

I: Mmm

P: Mmm, so, she closed it by mistake.

I: Oh

P: Mmm

I: How many times did it happen that it rings while you were sleeping whereas you should awake and eat first?

P: Uhm…but it was not several times my sister.

I: Mmm

P: Maybe around – sometimes I was eating first then drink them after, right?

I: Mmm

P: Maybe around…three, three or four times.

I: Okay, so, since you changed to the box, did you ever receive an SMS that remind you to intake the treatment?

P: No. Now since I had lost my phone – you know, my sister, I, and the phone to be honest - I have a certain style, do you get me? Since I lost a phone, I don’t know, I use a certain small phone, and I gave them the numbers of that girl of mine.

I: Okay

P: I don’t know at her side; I will ask her.

I: Okay

P: Mmm

I: Didn’t you have a phone?

P: No, I’m using a certain small phone, I, and phone are not friends, do you get me?

I: So…by the time you were not having the phone, were you not worried that maybe they might need to speak to you or maybe to give you a message?

P: I gave them someone’s phone numbers. Even for them to get me now, they got me because of the other person, they didn’t get me straight.

I: Mmm

P: They tell her straight, then she tells me, there is no way she could miss, do you see? She’s the mother of my kid.

I: Okay

P: Yes; if she didn’t tell me that shows that they didn’t call.

I: So…uhm…may you please brief us, who do you live with and where you stay? [inaudible segment]

P: I stay alone in the shack, I live in a shack but by the time I started, I was staying straight at the yard with my mother-in-law, together with my two siblings, one of them is in grade twelve now.

I: Okay

P: Mmm

I: So, did you ever – did you tell them you are taking TB medication?

P: Yes, I did tell them.

I: So, after you telling them, how did they feel about that matter?

P: Yes, to me they are very supportive, do you get me?

I: Yes

P: Yes, my family is very supportive, and they don’t confuse me with anything, they just told me to take the medication, do you get me? They advised me to take the treatment – “do whatever they told you to do in a right way, drink them until you finish the treatment.” They told me straight that it doesn’t kill, it kills only if you are playing, you should do things in a right way. They had never been disgusted at me, they hadn’t reacted in a strange way at me, yes.

I: By the support that they gave to you, do you realize that it changed your life somehow?

P: Yes, a lot, it changed my life a lot, to be honest when I look at this treatment, I think its availability has done a great job for me, I’m referring to this illness, do you get me? I was smoking a lot, and now smoking is no longer my thing – after I received this treatment, after they explained to me, I said eish…cigarette because I know – so, let me stop it for a while. Do you see, even now it’s difficult for me to go back to smoke, do you get me? They changed – they made me to change a lot of things and I get used to this style, yes.

I: Okay, so, without those people in the house, is there any other people, maybe like your friends or any other person that you told?

P: Yes, uhm…I told all my friends, do you get it?

I: Mmm.

P: Yes. “Hey guys, I have this and this, so as for me, cigarette, so and so.” If I’m drinking beer we don’t share anymore, do you get me?

I: Mmm

P: They didn’t react negatively, but you know how a person is, he might on other side [inaudible segment] straight, after that pretend to you, but as for them I didn’t see anyone reacting in a strange way, no, they reacted only good and supportive. When you are admiring to smoke, they could say to you, “no, my brother, you should not smoke, you know this and that,” yes, they didn’t [inaudible segment].

I: So, how did you feel about the support that you had been given by your friends and those people around you?

P: Eh! (Expression of amazement), I felt very good, very good, because when you come - by the time when I came to clinic, like that period when I was staying here, I was walking, and they were sometimes accompanying me. They realized that hey, this man has got no plan, if one of them is available – just because the job was waiting a bit because of…you get me, right? If one of them didn’t go to work, he could accompany me, coming with me here at the clinic, staying with me inside the clinic as if he’s sick but whereas he is not, he just accompanied me only. So, that thing made me to realize that there is no difference, what I’m doing is not a flop, let me continue by taking my treatment, then be okay, do you get me? There were people around me who were supportive to me, do you get me?

I: Mmm.

P: Mmm.

I: Okay, so, by the time when you were with these stickers before they change them to the box, were you having some worries?

P: Some worries, how, my sister?

I: Like this - were you not having any problem when thinking about them, maybe saying eish this thing looks like is going to be a flop to me?

P: No, no, I took them, exactly the way they explained to me; indeed, do you get me? I didn’t [inaudible segment] my sister – just the way the explained to me, that’s how I reacted towards them. I realised that they don’t have any problem, they don’t have any problem, right? I realized that they are pushing something you see, that’s shows that their work is progressing at their side, do you get me? By their words that they are coughing out towards us, doesn’t mean that [inaudible segment], so, that’s to indicate that at their side the work is progressing indeed, do you get me? So, you will end up saying, there is no problem, let me just do it because they told me to do, let me just comfort them by doing this thing of theirs, so that their work could carry on.

I: Mmm

P: I might drink them, but not sending them, I might just drink without sending, so, it’s not compulsory, right? That’s just an indication that indicates that their work is progressing, what they are telling us understandable.

I: According to your statement, I heard you saying that your mother-in-law was infected with TB, which year was that?

P: Uhm, now is 2022-2021, [inaudible segment], it seems like it was around…twelve, thirteen, fourteen, somewhere there.

I: Mmm

P: Mmm

I: So, according to you when you realize, if a person is taking a treatment without this intervention of sticker and box –

P: Mmm

I: Is it difficult or easy?

P: P: To be honest it’s difficult to take medication without box or sticker, do you get me? I like them, they remind you, do you get me? It seems like someone is in front of you, they’re acting as if they’re slightly forcing you, I won’t say straight [inaudible segment] if you are alone, you just say – but the pills are obviously tiring indeed. Yes, they are good, they are needed, they are pushing, they make a person to recall, to recognize what’s happening. When they’re available, it seems like there is somebody next to you who keeps on pressing you if you don’t drink them – maybe if you didn’t take them, what would you say if something happens to you during the night, what could you say? There is no way, you could realize that you are playing. By the time she was drinking them – but there was no way she couldn’t take them, the flop came here – It came because she realized it after a while, do you get me, she kept on taking ARV medication whereas she was having TB in the meantime. She was admitted for a while at the hospital. No, but I prefer these things to be available. Yes, they’re helping.

I: Stickers and the box?

P: Yes, honestly both of them.

I: Mmm, so, did you ever see your adherence calendar, the one with green colour and [inaudible segment] –

P: Yes, I see it, yes.

I: What do you say about it?

P: [inaudible segment] when I look at it, I was not satisfied. Do you get me?

I: Yes

P: But it was during the time when I was drinking without sending.

I: Okay.

P: Do you get me? Yes, the redness that I have seen is slightly higher. It’s too much. The greenness was not much.

I: So, did you see it before drinking – before – by the time you were using the labels, were you having a lot of redness?

P: Yes. By the time I was using the labels, yes.

I: So, since you used the box, did you ever see it and how is it?

P: Uhm…during the box, I didn’t check it well, but surely, it’s a total, it has got no problem.

I: It has got no problem?

P: Yes, during the box I drank. Honestly, I don’t remember missing -

I: During the box? –

P: I don’t remember me, missing, at all, even with – unless I missed to send, I don’t remember missing to intake the pills, do you get me?

I: Mmm

P: Mmm

I: So, what could you say helps a lot by using the box?

P: By using the box, what helps a lot? You know the matters is created by the person; you could do it but if you don’t like, you don’t like but this thing… this alarm. Alarm is the best, it is noisy, but you end up getting used of it indeed. It is very enjoyable. But now, you as a person if you don’t want to drink, you won’t drink. As for it, it helps a lot because when you stay there you can see it, on other side the stores your pills safely, do you get me? It locked them inside, it is respectable, even a child could be afraid of opening it or doing something. Yes [inaudible segment] box.

I: So, since you used this box, didn’t you reach any challenge where the child opens the box or maybe –

P: I have it – I had it but not for a long time, we moved it on top – so, they said it should be stored in a cool place, right? On that space that’s where my child xxx (participant daughter’s name) had been able to reach. So, when we moved it to the top of the fridge, she was no longer able to reach it. She didn’t bother me for a very long time, yes.

I: But can you say she opened it, and she opened how many times?

P: xxx (participant daughter’s name) What I know, maybe three, three or four. Maybe I didn’t notice her somewhere, but she didn’t open it many times where I noticed her, no.

I: So, since you used this box, you didn’t receive any SMS because you say you had no phone, right?

P: Yes, no, she should have told me, this person – but I don’t know, according to the phones, she was the one who was answering all my calls, she should have told me, there is no way she couldn’t tell me, plus she was more concerned by this matter of TB, she should have told me.

I: Mmm

P: Because I’m sure that those SMSs were telling her that I’m not taking the medication, right? She could be worried that my brother, they say you are not taking your medication, yes.

I: So, do you think that there could be something that could prevent someone to use these stickers or the box?

P: Yes, it’s a lot of things that could prevent a person on his life. There is a lot of things. There is a lot of things because now this thing changes you – you come along getting used of your lifestyle, hey there is a lot. First step – the first three months or two, you count them with weeks –that first six months, uhm…if you were getting used of reacting in other way, maybe you get used that during Friday, Saturday, Sunday – during the week; you go to work, you go and return, you know that you travel with it in the morning. Uhm… if you used to do your things during the weekends, you will feel it. You will go to your places and come back realizing you have been passed by time – these things of stickers help you, what if they were not there – what do they call it? If the box was not there, when you are sleeping, you are sleeping, you will find that you are really resting, when it is there, it is able to alert and remind you. For smoking, you must stop it. Someone might say, “I am smoking, I drink pills and smoke. I realized that most of them they end up elapsing, that people they get extremely hurt because they started them then they ended leaving them, so most of them they got hurt indeed.

I: Mmm

P: Mmm, most of them got hurt. There was another one who died, one of my friends who stayed there at Extension4, he’s dead because of this thing. He took long time before he got tested, doing all his things. So, I know that it’s killing.

I: So, according to your opinion, right?

P: Mmm

I: For the people who had been prevented to intake these pills, what should be done, so that they could be able to get help?

P: Eish…my sister, according to me a person is a person, that’s why I say it could depend on slight plan [cough sigh]. Eish…you couldn’t push a person, even though you gave him a box, so, he’s gone indeed, I should defend myself on other side, you couldn’t force me to intake them. So, how could you deal with this kind of a thing? Could you say he should come and intake them at the clinic? He won’t come, he will dodge. Would you come and force him to drink? Will you always be able to come and knock for him? Maybe it could be the best option. If you don’t get him, still, do you realize that is a waste of petrol? That’s a flop, yes. If you follow him, that means you must follow him thoroughly indeed, in that way he doesn’t have any other plan, or you should lock him there. There is a certain hospital in Johannesburg, right? Where is it, is in Joburg? That one of [inaudible segment], if you stay there, you will tell them the truth. Yes, unless you lock them there. People doesn’t listen, they listen when they feel pain.

I: Mmm

P: But as the people we won’t react the same.

I: So, since you start with this treatment, did the people who works – from the clinic, ever come to your yard?

P: Mmm

I: Mmm

P: Yes, they came. It’s just that they came –

I: Yes, could you explain a bit –

P: They came twice.

I: Mmm

P: So, it’s just that when they come there, they said they are there to re-collect the cough fluids of the other people who stay in the yard.

I: Mmm

P: Mmm, they came, then I came back because I was not available – when they came, I was not available, then they came back again, that’s where they found me. They spoke to me, then they took my cough fluids again, and they also took them from the other people who stay in the house and all their details. They said they wanted to check if they were not also infected. Mm, yes, they came.

I: What – what did they say? When they arrived at you ,what did they say, what did they say they are coming from, what did they tell you?

P: Hey, they told me that they are coming from here at the clinic indeed. Yes, they said “we’re coming from the clinic, we’re in need of this kind of a person,” then I said “yes, it’s me.”

I: Mmm

P: They said, “do you have this kind of illness?” I said “yes” [door cranking] they said they want…to check the people that I’m living with, and one of them had been infected before, what, what, and so forth. They asked me how many they are, then I counted them all. I asked them how they (family members) get involved. They said no, we’re just in need of their particulars, so that we could be able to check if nothing happened to them. But they had also checked me again.

I: Mmm.

P: Yes, just like that.

I: Didn’t they talk to you about that disease, did they teach you a bit or did they give you counselling?

P: Eh! I stayed with them a while in the car. They explained to me. Yes, they did explain to me about what kind of illness this is one, the importance of intaking the medication, things like those ones. No, they spoke to me.

I: Okay

P: Yes, they gave me [inaudible segment], they gave a bit advice.

I: So, how do you feel about the way they briefed you? Is there anything that shorts?

P: Uhm… I feel – it’s okay, it’s just that it indicates that there are some other people…they care, do you get me? A lot, do you get me? They take all the efforts for – according to me, if somebody disagree, no, that shows he enjoys being in that way, so, I could leave him in that way, do you get me? So, I recognized that some other people give a lot of care, they are caring very much, do you get me? They take an effort to come and tell us that what is what, and so forth, do you get me? I recognize very well that they gave me the strength to do – these many people – I can’t count them, nurses from the clinic, those who were dealing with me there, those ones – yes, they are the ones who encourage me that why should I confuse myself, let me just intake them, do you get me? Let me just say, I don’t have no doubts, maybe I might think in other way, saying no, what is TB, because when I came here, I was – honestly, I was not having more strength anymore, but I was not very much weak.

I: Mmm.

P: Hey, I could just say – maybe I could say no man, it’s just a TB – what is TB actually? I felt it when I take the medication, then that’s where I realized that a person was seriously sick. Even that game – the game that I’m playing now, I realized that the person was seriously sick, but I was not recognizable, right? It won’t show, I was not recognizable at all. So, I felt lifting [inaudible segment], then I recognize that I get hurt.

I: So, those people that came at your home, the way they explained to you, according to your view, do you think that they could make the difference if it might happen that they should consult the people that doesn’t take the treatment?

P: Yes, they could but I’m still sticking on the issue of saying that if you want to listen you could listen but if you don’t want, you won’t listen.

I: Mmm

P: As for them, they are talking the correct things, right?

I: Mmm

P: If I’m stubborn, I’m stubborn. As for them they should carry on trying to help these people.

I: Mmm

P: Those who win – those who win, they will [inaudible segment] all. They could consult them all but is not all of them who could follow them. Those two or those three that they helped –

I: Mmm

P: [inaudible segment] it’s okay, that’s the effort because a person is a person my sister.

I: Okay. So, are you satisfied by using the stickers?

P: Yes, a lot, but I’m still complaining that its problem is that, no, you should stand for yourself. The box – but the box just because –it’s a slight old age – do you get me – that’s why m others might say it’s a slight old age, do you get me?

I: Mmm

P: They want the people on the phones, do you get me? So, phone is another thing.

I: So…okay, between phone and box, may you please explain to me, which one satisfied you a lot and why do you say so? May you please give me few reasons.

P: The box, it helped me for a very long time.

I: Mmm

P: If – even now I’m finished, you see it, right? It’s a box, yes.

I: Oh! So, the only thing that gave you the strength about the box, was the reminder?

P: Honestly, I need to be reminded.

I: Mmm

P: It’s portable, it’s safe, it’s okay.

I: Mmm

P: Mmm

I: So, according to your view, nothing should be changed?

P: According to my view?

I: Yes, what could you say? What is short?

P: No, it shorts nothing, the pills are sustainable, right?

I: Mmm

P: It’s okay, nice.

I: Are you satisfied about it?

P: Yes, it’s okay, yes. The pills are sustainable, maybe I should say the size, so that the pills could not be well sustained, right?

I: Mmm

P: Yes, it’s okay.

I: Okay –

I: Doesn’t it have any problem?

P: No

I: Nothing that you could wish to change?

P: There is nothing wrong. I was able to come back realizing that tomorrow I won’t be able to waka up, then I could take it and put it so –

I: Mm

P: Yes, but it could awaken me very well.

I: Okay. So, the way it was, wasn’t it – there is nothing that –

P: No

I: Do you have this thing – is it perfect the way it –

P: Hundred percent (100%), and it doesn’t short anything my sister.

I: So, at the beginning when you were starting – before your phone got lost, didn’t – how did you feel about the SMSs that you were receiving?

P: The one that I received, how? The one that they were sending to me.

I: Yes, while you did drink but not SMSed.

P: Mm

I: [inaudible segment]

P: I felt like I was the one who wronged, right?

I: Mm

P: Because I was the one who was indeed wrong. Because at the beginning when they issued me this thing, they told me. I agreed, right?

I: Mmm

P: I agreed to all those things, so, when they sent me, I was the one who wrong. There was no way I should be angry, why should I be angry because I’m the one who should SMSed, but I failed SMSing.

I: Mmm

P: Yes, I had to take the blame.

I: Okay

P: Yes

I: And wasn’t it hard for you when they were calling you – problem when they were calling you? Whereas you drank, you know very well that you drank but then you failed to SMS, then they call you.

P: As you know that there should be a bit of that thing indeed, there should be that thing of saying eish…do you see these people now, and I’ve already drunk, do you get me?

I: Mmm

P: But at the end you’ll end up agreed, recognizing that this is the same, what are you complaining about because they tell you the truth, they want to confirm if you drank. Honestly, you could be bit worried sometimes, “hey, these people are abusing us now, I’ve taken my pills, and now they keep on calling me.”

I: Yes, so, your feelings when they come at you –how did you feel initially when you heard that people from clinic are here at your yard?

P: When I saw them, I thought they are the CIDs, I didn’t think they are from here at the clinic, do you get me?

I: Mmm

P: I did ask myself that what’s happened now?

I: Mm

P: [cough sigh] they just arrived and said “no, we’re here just to know what’s happening in here.” So, but I got out of it, saying hey indeed they [inaudible segment], then they asked us to do this and that and so forth. Yes, indeed, I entered their car but as for my feelings did never…I was relieved after I realized that they were people from the clinic.

I: Mmm

P: I don’t have any flop with the people from the clinic [inaudible segment].

I: Don’t you have any flop with them?

P: No, I don’t have any flop with something that helps me. You couldn’t force me, and you can’t push me, and you can’t do me anything. I come by myself, I’m not afraid it because I know that this is the same. Yes.

I: So, according to your observation, is it difficult for someone who doesn’t have any support and someone who’ve the supports, when he intakes the medication?

P: Yes, it’s difficult.

I: Mm

P: Honestly, it’s difficult because there are some days where you need someone just to remind you that today is the day, go and fetch your treatment.

I: Mm

P: But if he is there, at least [inaudible segment] or someone else, do you get me? “Hey man, the treatment, right? There is nothing inside the box, it rang, and we saw that there is nothing inside.” He pushes you but if you are alone you take it with that mind of being alone.

I: Yes

P: You could just say “no man, I will go and fetch them, I’ll fetch them man.” What I know is that you should not jump (avoid) when taking this treatment. You made them to be familiar with your body, so you should intake them until you finish.

P: So, what could you just say to someone who doesn’t have phone whereas he uses the box? Do you think it could help him a lot or it could change his life, or do you think that could just be the same?

P: Whereas he is alone in that way? Are you referring to someone who doesn’t have any support?

I: Yes, whereas he’s alone. Someone who intakes the pills but not having a phone –

P: Mm

I: Having nothing that we could say reminds him, like a phone.

P: Mm

I: But he intakes the pills and uses the box. Do you think it…it could change his life, or do you think it’s the same?

P: Eish! Sister I’m still repeating on that one again. People are not the same. This is an effort, you have taken a serious effort, do get me? If he is a person, it should change him, do you see? He should settle his things, so that his things could be okay.

I: Mm

P: But if he’s stubborn, he’s stubborn. Now if you can look at this nowadays’ age – if you look at an old age, yes, it’s the one that I know that if you gave papa something like this, you should know that he’s going to take it seriously [inaudible segment].So, this age of ours, hey, if he doesn’t want something, he doesn’t, he could just say “ how! This is the same, these people are getting familiar of me in a wrong way,” and then throw them [inaudible segment]. But then according to me, the best thing, right? By myside it helped me a lot, do you get me my sister? I won’t argue with it, it helped me, it helped me very much.

I: Okay

P: Speaking for somebody, even now my answer could be yes, it could help another person, but only if he agrees.

I: Okay, so, according to your view, do you think if someone is familiar to his lifestyle, and they give him the pills without any reminder –

P: Yes

I: Without a phone, without anything that could remind her, could he reach any problem maybe?

P: Very serious one. He could find a problem, actually he won’t drink them. He’ll be gone. Now they told me here to select a time, right – what time would you drink them, and you should follow that selected time. Now, nothing, nobody, it’s just the pills only, I put them there. You just entered and intake them at seven, at nine [inaudible segment], the following day you walk away then you come back and intake them at that time you arrived. So, they could confuse you. That’s why I say this thing at least has got the dignity, do you get me? It’s not just a box like that, it has got the dignity that you could be able to…eish, this thing, yes. Just like that, no, it doesn’t.

I: So, do you think that having this box could be very helpful to most of the people, especially those who have got no phones – those who don’t have things that could remind them?

P: Yes, I agree, it could be a lot of help. It has already started with me; do you see that it helped me also? Yes, I agree, it could help the people.

I: So, according to your view, do you think it could proceed being used or given to the people.

P: Yes, let it proceed.

I: Mm

P: Yes, let it proceed, it’s still going to help the people, I’m sure. People just pretend to be stubborn; they are not stubborn; they are not stubborn. Yes, it’s going to help them.

I: Okay. So…so far, right? Let’s say on this sticker – label, right?

P: Mm

I: The box, right?

P: Mm

I: Let’s say SMS, let’s say call.

P: Mm

I: Let’s also say home visit, where people from the clinic come and check on you at home.

P: Mm

I: Which one do you recognize that hey, yes, this one work more than the others when coming to a reminder that remind you to intake the pills?

P: Mm

I: Mm

P: For me, at my side?

I: Yes, for your side.

P: It’s just that for my side, I used the box –

I: Mm

P: It’s obvious that I could say the box, but when we go back to the stickers, I could say now, you are always available here, do you get me? So, next time I won’t be here, do you get me? Some could prefer the stickers, so that he could just finish and then he could be able to do his things in his own way. This one of phone, I don’t get it clear, that one of calling me. So, “for pills, yes, it’s okay, it’s off,” that’s me when I’m alone with the pills. It’s just that even if I don’t drink you won’t recognize, right? Actually, those two, yes, I [inaudible segment]. Those ones of the phones go together with the one of the stickers. Yes. So, for me, no, uhm…I –

I: Are you using the box?

P: Mmm

I: Is it working good for you?

P: Yes, it worked and I’ve finished, my sister.

I: Mmm

P: Mmm

I: Okay

P: Mmm

I: So, in this study that we’re doing, this one of [inaudible segment], do you think there should be the tools that we should enclose since we brought the sticker and the box?

P: Eish…tools that you could add is to plan, so that the sticker could also have a voice.

I: Mmm. Should there be a voice on the sticker?

P: Yes, it ought to speak [inaudible segment]

I: Mmm

P: Yes, it ought to speak plus as the people we’re not the same. If it was me, I should stick with it forever, do you get me? It should also speak.

I: Mm

P: Mmm

I: So…I hear you saying it must speak. So, would you be able to help us a bit – what should we do, so that it could speak maybe? What are we supposed to do, how could we do it?

P: Eish…Just because you see that it is just a sticker, there is no way it could speak, how could it speak?

I: Mmm

P: Eish, no, but there is no way the sticker could speak. Actually, it is just that the speaker goes together with a phone. You have the people who works with the calls, right? You could call me every day, I drink them every day, right? You can call me every day at nine, “hello!” yes.

On my side once you call me then you remind me, right? Once you drop, then I prove to you by SMSing the sticker.

I: Okay. So, you say someone from the clinic –

P: Mmm

I: Should call you?

P: Yes, “now is the time papa.” There are some of the people that you call, right?

I: Mmm

P: Yes, “now is the time, my brother.” “Oh, it’s the time.” [inaudible segment] then I prove you after drinking, by –

I: There after you send an SMS?

P: Mmm

I: Do you mean that a person should be your alarm?

P: Mmm

I: He should call.

P: Mmm

I: Then you could say “hello” then he should say “hi, now is the time to drink.” –

P: This is the time. Yes.

I: Then you drink, there after you send an SMS every day.

P: Mmm

I: So…in this way I understand you now. So, in this way, do you mean someone should first call you, then you –

P: Then you give the reward with this thing, an SMS.

I: Okay

P: Mmm

I: Mmm! Okay, no, I understand you. But up to so far, since you use the box – I hear, you spoke about an alarm, could you also speak about the other thing, uhm…that you noted about the box?

P: What looks like this thing my sister? [laughing] this box is just nice, it doesn’t have anything, it doesn’t have any mixed up.

I: Mmm

P: I just recognize it as nice [inaudible segment], it’s an opinion, nothing else.

I: Mm

P: This is the box that when I look at it, I see something that helped me. It’s now capable of talking, my sister, right?

I: Yes, it’s capable of talking.

P: Yes, as for me I don’t believe I could win without it.

I: Okay.

P: Do you get me? Maybe after – because during that period I was fighting, going there and there. I was going there and there whereas I’m drinking the pills. It took me a while, so that I could stay still and do my things in a right way.

I: When you say you were going there and there, do you mean like…you were coming back home, or were you going out then come back?

P: Oh! It’s obvious, there is no way I couldn’t come back, but it’s just that when I went and arrived, you could find that I come back at a certain time. If I was not having the box, I could had been beaten by the time of intaking the pills. So, if it’s available, by the time when I say, no, then it’s able to awake me. There after I could intake the pills, then going back again to things that I was doing before.

I: So, on…on your journey of using the box, did you ever go with it when visiting somewhere?

P: Yes, I did go with it.

I: Mmm

P: I went with it few days ago, I’m sure at the beginning there.

I: Mmm

P: Yes, it seems like I took around three days. Yes, I went with it.

I: Okay

P: Mmm

I: So, what did people say when they saw you using the box, what did they say about it?

P: Uhm…one of them told me that he was using it.

I: Mmm

P: Some people-as you know people, someone could say “hey…”and another one says, “do this,” do you get me?

I: Mmm

P: Only one told me that “hey, I know exactly what infected you”. I said, “what’s it?” He said, “I had also used this box.”

I: Yes –

P: But he was an adult, that one, yes.

I: So, what about people that you met saying they know. What would you say to them?

P: They know…?

I: That this box is for what.

P: Uhm…the one that told me straight is the only one, he said he was using it, it’s not a while since he finished. The other one is the man who [inaudible segment] down there. By the time he saw it – but during that period I was taking it back, do you get me? I was holding it, going with it then he said, “hey man, that thing that you are holding…” yes, he said “hey man, I’m also on top of that thing.” I told him that as for me, I’ve already finished, I just take it back. He said “hey, men!” So, do you see that – is this box really made for TB only? Or is it also made for the other pills?

I: Yes, here – since we start with this study, we’re making it for TB only.

P: Okay. Don’t you see – this means that the man I was talking to- He also had TB, that man.

I: Mmm

P: Because he said that he was having this box.

I: So, according to your observation, those people that were responding, except those ones, could you say – do you think when the people see you holding it, could they mistreat you?

P: Yes, in a bad way. Yes, some are reacting very snags indeed, do you see it? They don’t want to see – especially when they say TB, *iyooh*!(wow). Even where I was working, when I got out there, When I was telling them, they chased me – I don’t know how they got me out there – I don’t know what happened.

I: Mmm

P: Mmm. You would be afraid of talking in other places.

I: Mmm

P: There are some other times where you find yourself locked, feeling like “hey, actually what’s happening, what kind of person am I, being infected with such a snags thing?” When your heart cool down, you just say, “you know what’s actually happening, there is no flop.” Some other people are very snags, you could just realize that this person doesn’t want to see himself next to me, he’s playing far away from you.

I: Mmm

P: Yes, hey, they are there indeed. But as for me, I don’t care about them, but they are there.

I: So, how do you feel, when the people realized that you have TB treatment on your side, then mistreat you? How are your feelings?

P: Yes, sometimes, it’s bit painful, right? If you are not patient, you would kill yourself. The painful thing is that you ask yourself what do these people think? This doesn’t mean that I infected myself. I’m not even aware how it infects me, I don’t know [interruption caused by unknown sound], I was thinking that maybe they could tell me about what, what only, then give me – now they tell me about TB. “TB! And how did it infect me, this thing? I…this thing?” You find someone acting disgusted on you as if - when you act strange on them, they end up seeing that this man doesn’t get involved, this is the same, right?

I: So, by the way people treat others when they recognized that they are sick, do you think they should be taught, could there be any change?

P: Mmm, eish…yes, they should be taught, do you get me? [inaudible segment]. Starting from the Boers there. Those Boers are the ones – I came here and then they gave me letter to give them. It seems like that letter were talking that – what’s that? After what? It’s two weeks.

I: Mm

P: What’s that? It’s two weeks, so that I should drink the pills whereas I’m still at home, then from there I could go back to work.

I: Mm

P: [Inaudible segment] so that it won’t affect the others. Eh! Now they disagree as if, I don’t know what’s happening, you know? You know now that thing hurt me extremely – when I got out of there I said “eh! Jerr…in this way?” But I kept quiet, I ended saying “it doesn’t matter, it doesn’t have any flop.”

I: So, according to you do you think the way people are being treated, they could end up keeping quiet, without talking –

P: Mm –

I: That they are infected with TB? –

P: Yes, they’ll keep quiet –

I: And will it end up being a problem?

P: Mm, most are keeping quiet, clearly just like that. I’m sure that even now there are those who kept quiet. No, they are going to keep quiet. The other one could drink them, there after fighting to fit in again if he was preferring to go out with other people. He could drink them hiding himself aside, there after acting so – when I observe, they are not supposed be balanced in such a way. They should not be taken here and there; you should drink them indeed.

I: So, according to you, what should we do to bring this message of TB to the people, so that they should know more. What should we do?

P: Eish…let me say internet and phones – we could use these phones.

I: Mm

P: It’s just that they’ll just read and get out of it, these people. We should teach them bit by bit, then they’ll end up understanding. Bit by bit, my sister. You will slightly understand.

I: Okay

P: These small teams, one day at the locations. When you arrived there, finding them in groups, you should explain to them. They have a serious negative attitude. When they are trying to be negative, you must stand still and explain to them.

I: So, according to your view, on these teams who should explain to those groups? Should it be someone from the clinic or someone who had been infected by TB or…? –

P: Yes, it’s someone who had been infected.

I: Mm

P: Yes, someone who had been infected, he’s the one who could work this thing, do you get me?

I: Yes

P: It should be someone who could be unshaken. You should talk whatever you want to talk indeed.

I: Mm

P: It needs such a person, the one who knows how this pain is, so that if you react in this way, it could be in this way.

I: Mm

P: You should explain to them. In a small group of people, at least two people should understood, even if they might pretend as if they didn’t understand, but two people will understand.

I: Mmm

P: Yes, they are going to understand clearly.

I: No, I heard you. So…maybe before we close, could you just tell me like…your experience before we close. Your final about the stickers…and the box, what could you say? What do you want to say?

P: Uhm…the last thing that I would like to speak, thankful, do you get me my sister? It’s to give thanks, do you get me? –

I: Mm

P: These stickers and these things, together with people from the clinic. I also give thanks to the minds that they have – they enclosed by the stickers, by all these things, do you get me? Because these things had this thing that when I see it, also – these things also helped me, when I see them, I took them and put them in front of me, that’s the one that made me to intake the pills.

I: Mmm

P: But you should carry on with them.

I: Mmm

P: Yes, carry on with them. They would drink them, the pills, I’m sure because I had also been able to drink them. Yes, carry on with it but about that sticker you should plan for it, or you should give it to the youngsters, it seems like they are also in need of the stickers indeed. Yes.

I: So, according to you, everything could proceed, the way I understand you –

P: Yes, everything should proceed –

I: Then, someone should have a choice, he should say –

P: Yes, we make them exactly so –

I: I’ll be capable of box or sticker –

P: Yes

I: Is it what you say?

P: Yes. According to me –the box is for older people including grandmothers and grandfathers, when it rings it reminds them to take medication

I: Oh! So –

P: I go together with adults, old age. Yes, this one would be for youngsters, the ones with phones [inaudible segment].

I: So, you said the youngsters, so, could you highlight us with the age group when you say sticker should be for youth, which age group should we give?

P: No, until thirty.
I: Until thirty?

P: Mm

I: And they –

P: They –

I: Those who use the box…age?

P: It’s just that after thirty – after…thirty.

I: Mm

P: [unknown sound] you should ask them if they want it or how – you would explain to them, right?

I: Mm

P: Mm

I: Now…now how are we going to do it? So, the way you talk about the age group, what should we do if someone is less than thirty, but he doesn’t have any phone?

P: Uhm –

I: Let me say –

P: One who doesn’t have phone is the same as me. That doesn’t mean that he doesn’t have any phone.

I: Mm

P: It’s just that he doesn’t like to use phone.

I: Mm

P: You give him this one. This is a steer. This one is going to frighten him, my sister, do you get me?

I: Mm

P: According to me, this one is the best, do you get me? This one definitely beat this other one, they are not going with the same number. This one is down.

I: Is this one slightly down?

P: Yes, I could give it around seventy-eight – seventy percent (70%). Hundred percent (100%) this side, here on the box.

I: On the box? Do you rate hundred at the box?

P: Yes

I: Then do you rate seventy at the stickers?

P: Mm. The stickers could be lost. Do you see [inaudible segment] They respect the box; do you get me?

I: Yes

P: Even if it stays here, they know that it belongs to whom.

I: Okay

P: Mm

I: So, according to you, you think when the kids look at the box, they don’t touch it?

P: They know, you tell them for the first time, right? –

I: Mm

P: “Hey, this is my pills, right?”

I: Mm

P: In this way then he knows very well. So, you see sticker – you also see that it’s just a paper. Could you tell him that this is my sticker? You should always hide them.

I: Mm

P: Then you end up forgetting, plus these pills make you to forget. You find out that you forgot where you put them, do you see?

I: Mm

P: So, this one, there is no way you could forget it, you won’t forget it.

I: Okay

P: Mm

I: Mm! Okay, no, I heard you. Today I heard you and I would like to thank you for the conversation that we had, together with the information that you gave us.

P: Mm

I: I also thank you for an affiliation that you had with us on using the sticker and the box, by drinking your medication and get healed. I salute you, carry on reacting like that.

P: Sure, my sister.

I: Yes, I thank you very much for your affiliation and your help. When we’re in need of your help, please proceed on reacting like that even on the following days.

P: Mm

I: Yes, we’ve reached the end of our interview. Now the time is 12:35

P: Yes, I’m the one who is thankful my sister.

I: Sure.
