# Supplementary material for: Acceptability of Digital Adherence Technologies to support people with drug-susceptible TB in South Africa
Source: PLoS One. 2025 Sep 24;20(9):e0332103. doi: 10.1371/journal.pone.0332103 (PMC12459780; doi:10.1371/journal.pone.0332103)
Supplement: Attachment — Submitted filename: Responses to reviewers.docx [file pone.0332103.s005.docx]

PLOS ONE
1265 Battery Street, Suite 200
San Francisco, CA 94111
United States

8 August 2025

Dear Editors in chief

We would like to thank the editor and reviewers for the very valuable comments made. They have certainly enabled us to strengthen the quality of our paper and increase its relevance. In response to the valuable suggestions, we have incorporated publications on qualitative research to support our methodology. We have also included references of the papers to briefly describe what Direct Observed Therapy is. We have also included studies which showed high adherence among people with TB who used the pill box.

In the present letter, we have a table showing the comments raised and our responses. Both clean and marked-up copies of the revised manuscript are attached.

Regards,

Tanyaradzwa Dube, corresponding author.

**Responses regarding data availability**

| Comment | Response |
| --- | --- |
| All PLOS journals now require all data underlying the findings described in their manuscript to be freely available to other researchers, either 1. In a public repository, 2. Within the manuscript itself, or 3. Uploaded as supplementary information. | The data is available within the manuscript presented as quotes and deidentified transcripts are uploaded in supplementary files. |
| We note that this data set consists of interview transcripts. Can you please confirm that all participants gave consent for interview transcript to be published? | Yes, the participants consented to having their de identified data shared in published journals.  The transcripts have been checked to remove any identifying information as recommended. |

**Responses to the Editors Comments**

| **Comment** | **Revision** |
| --- | --- |
| All PLOS journals now require all data underlying the findings described in their manuscript to be freely available to other researchers, either 1. In a public repository, 2. Within the manuscript itself, or 3. Uploaded as supplementary information.  This policy applies to all data except where public deposition would breach compliance with the protocol approved by your research ethics board. If your data cannot be made publicly available for ethical or legal reasons (e.g., public availability would compromise patient privacy), please explain your reasons on resubmission and your exemption request will be escalated for approval. | Transcripts will be provided as supplementary information. |
| In the abstract, write 'and/or' instead of 'and or' (line 28) | Done (line 29) |
| Proofread spacing around references (see line 110 for example) and also remove full-stops for titles and sub-headings | Full stops removed on subheadings lines |
| Elaborate more on what was done to manage the pre-existing relationships between data collectors and interviewees mentioned in lines134-5. Did data collectors reflect on this in field notes? Was this discussed during the consent process. Consider revisiting this in the limitations section as well. | To manage the relationship, the interviewers encouraged the HCWs to feel free to share their honest opinions and assured them that their responses were confidential. During interviews, the interviewers emphasized neutrality and created a respectful, non-judgmental space to mitigate social desirability bias and support candid engagement. (line 143-146)  Referenced  Added the following in the **Limitations:**  The existing relationship between the HCWs and interviewers might have increased social desirability. (line 502-505) |
| The idea of sampling to reach data saturation was mentioned in line 140. However, this was not addressed in terms of how the final sample was decided. Clarify if data analysis was happening concurrently with data collection so that sampling ended once saturation was met or if other factors influenced the final sample. The term saturation in the analysis section (line 166) seemed to imply a different concept of saturation, involving the three coders rather than something that informed sampling. | Saturation during data collection was achieved through regular debriefing discussions with the data collectors and interviews were stopped when no new information emerged. (line 150-153)  References |
| Clarify the language(s) of interviews and who was involved in the translation and transcription process (a service or authors) in lines 152-53 | Interviews were conducted in the dominant languages spoken in the study areas the participants preferred (Setswana, isiZulu, Sesotho or English) based on each participant’s preferred language. line (163-164)  Transcriptions and translations were done by SK and other trained research staff fluent in the study languages. SK, TD and FM checked the accuracy of the transcripts against digital recordings. line (166-168) |
| Specify a reference for the qualitative analysis (if grounded in a particular form of thematic analysis) on line 165 | Thematic analysis referenced (line 179)  21. Braun V, Clarke V. Using thematic analysis in psychology. Qual Res Psychol. 2006;3(2):77–101.    22. Saunders CH, Sierpe A, Von Plessen C, Kennedy AM, Leviton LC, Bernstein SL, et al. Practical thematic analysis: a guide for multidisciplinary health services research teams engaging in qualitative analysis. BMJ. 2023; |
| Move up the UTAUT reference to line 168. | Reference moved to line 182 |

Responses to Reviewer 1

| Comment | Revision |
| --- | --- |
| Abstract  Recommendation to add a sentence or two about the number of people with TB globally and/or in South Africa. | Done  TB cases in South Africa added (line 23) |
| Intro  -You need a sentence or two to say something about TB in South Africa (see below for line 110).  -line 60: spell out health care workers  -Briefly say what DOT is | TB incidence reported (line 57-59)  Healthcare workers spelt out (line 61-62)  DOT is a monitoring strategy which can be facility-based, whereby patients visit a health facility daily to have a HCW observe their medication intake; or community-based, where a HCW or trained treatment supporter supervises the patient's treatment at home  (line 63-67)  Reference  Maher D, Mikulencak M. What is DOTS? What is DOTS? A Guide to Understanding the WHO-recommended TB Control Strategy Known as DOTS. |
| Methods -line 110” “In 2022 the TB incidence in South Africa was 280K.” This belongs in the introduction.  -line 111: the districts were Tshwane and Bojanala? I would just say that.  -line 111: “conducted at public health clinics providing care”. I would change to something like “conducted at public health clinics where care for TB was offered…” The former sounds like the clinics themselves were providing care.  -Data collection: what is the definition of “good adherence” vs “poorer adherence”. This needs to be stated somewhere in methods.  -Data collection: line 114 you say intended to conduct 20 interviews which makes one think that you did not do this many interviews? If you did do this number of interviews, be direct and just say we conducted x interviews. If not, you can say you intended to do so many, but only ended up doing x because of whatever reason. It’s also confusing that in the findings section the numbers are different as you say 37 PWTB and 35 HCW were approached. Clarification would be helpful. | TB incidence moved to introduction (line 58-59)  Tshwane and Bojanala districts mentioned (line 98)  Changed to “conducted at public health clinics where care for TB was offered…” as suggested (line 118)  Good adherence was defined as 90% or more adherence and bad adherence was less than 90% adherence. (line 125-127)  Deleted the confusing numbers. We now have only mentioned the number approached and the number we finally interviewed under findings. (line 194) |
| Findings -Line 180: 37 PWTB, 35 HCW + stakeholders were approached. Be consistent. If you are going to split out HCW and stakeholders in other sections of the paper please do that throughout. Table 1 also lumps HCW & stakeholders together. You need to decide if you want to treat these groups as separate or lump them together.  -Line 180: The issue above also makes it difficult to follow the math for the number of participants. 2 people declined from each group so 37-2=35 and 35-2=33 but then somehow, we end up with 26 HCWs which does not add up. | HCWs and stakeholders combined (line 128)  Agreed. I had separated HCWs from stakeholders (26 HCWs and 7 stakeholders=33). I have now merged the two to make it easier to read. (line194-196) |

Response to Reviewer 2

| Comment |  |
| --- | --- |
| It is good to dig into approaches increase patient’s adherence to long treatments for killer diseases like Tuberculosis. However, these technologies were or how much successful they were, it is paramount to share experiences so other researchers can develop more or find alternative approaches. | Cluster randomized trials conducted in South Africa and China found improvement in adherence among PWTB who used the smart pill box (line 77-79)  Referenced |
